# Supplementary material for: Host association and intracellularity evolved multiple times independently in the Rickettsiales
Source: Nat Commun. 2024 Feb 6;15:1093. doi: 10.1038/s41467-024-45351-7 (PMC10847448; doi:10.1038/s41467-024-45351-7)
Supplement: Supplementary file 1 — Supplementary Information [file 41467_2024_45351_MOESM1_ESM.pdf]

Supplementary Information

Host association and intracellularity evolved multiple times independently in the *Rickettsiales*

Michele Castelli[1], Tiago Nardi[1], Leandro Gammuto[2], Greta Bellinzona[1], Elena Sabaneyeva[3], Alexey Potekhin[4,5], Valentina Serra[2], Giulio Petroni[2]*, Davide Sassera[1,6]*

1. Department of Biology and Biotechnology, University of Pavia, Italy
2. Department of Biology, University of Pisa, Italy
3. Department of Cytology and Histology, Saint Petersburg State University, Russia
4. Department of Microbiology, Saint Petersburg State University, Russia
5. Research Department for Limnology, University of Innsbruck, Mondsee, Austria
6. IRCCS Policlinico San Matteo, Pavia, Italy

corresponding authors: Giulio Petroni and Davide Sassera: giulio.petroni@unipi.it, davide.sassera@unipv.it

This file contains the following:

Supplementary Note 1. Taxonomic descriptions of novel species and families discovered in this study.

Supplementary Note 2. General comparative description of metabolic and functional features.

Supplementary Note 3. Detailed description of the genome sequencing and assembly procedures (with embedded Supplementary Figures 1-17 and Supplementary Tables 1-8)

Supplementary Note 4. Detailed description of the phylogenomic analyses.

Supplementary Note 5. Detailed description of the gene content analyses.

Supplementary Figures 1-32. (Supplementary Figures 1-17 are embedded within Supplementary Note 3; Supplementary Figures 18-32 are individually and consecutively presented at the end of this file, with each respective legend preceding the corresponding Supplementary Figure)

Supplementary Note 1: taxonomic descriptions

Description of "*Candidatus* Vederia obscura" gen. nov. sp. nov.

"Candidatus Ve.de'ri.a ob.scu'ra". (Ve.de'ri.a: N.L. fem. n., light as a feather, due to the small genome size, from the Dutch veder, and resembling a character's name in George Lucas' Star Wars saga; ob.scu'ra. L. fem. adj. obscura, hidden, unseen, as the bacterium was not directly observed). This bacterium was found in association with the ciliate *Plagiopyla frontata* strain IBS-3, and belongs to the family "*Candidatus* Midichloriaceae". Uncultured so far. Basis of the assignment, genome sequence.

Description of "*Candidatus* Diomedesia obscura" gen. nov. sp. nov.

"*Candidatus* Di.o.me.de'si.a ob.scu'ra". (Di.o.me.de'si.a: N.L. fem. n., dedicated to Diomedes, in reference to mythological Thracian king Diomedes, owner of mares stolen by Heracles in one of his labours; ob.scu'ra. L. fem. adj. obscura, hidden, unseen, as the bacterium was not directly observed). The genome sequence of this bacterium was obtained from an environmental water sample collected from Black Sea, next to Varna, Bulgaria. Uncultured so far. Basis of the assignment, genome sequence (GCA_016780625.1).

Description of "*Candidatus* Diomedesiaceae" fam. nov.

"*Candidatus* Diomedesiaceae" (Di.o.me.de.si.a'ce.ae, N.L. fem. n. "*Candidatus* Diomedesia" type genus of the family; suff. -aceae ending to denote a family; N.L. fem. pl. n. "*Candidatus* Diomedesiaceae" the family of genus "*Candidatus* Diomedesia").

The family "*Candidatus* Diomedesiaceae" is defined based on phylogenetic analyses of concatenated orthologous genes of uncultured representatives from various marine environments.

The family belongs to the order *Rickettsiales*, and currently contains one genus, "*Candidatus* Diomedesia".

Description of "*Candidatus* Jistubacter obscurus" gen. nov. sp. nov.

"*Candidatus* Jis.tu.bac'ter ob.scu'rus". (Jis.tu.bac'ter: N.L. Jistu, the Native American mythological trickster rabbit, a light-hearted character prone to humorously inappropriate behaviour; N. L. masc. n. bacter, rod; N. L. masc. n. Jistubacter, Jistu rod [also intended as an ironic compliment to one of the authors]; ob.scu'rus. L. adj. obscurus, hidden, unseen, as the bacterium was not directly observed). The genome sequence of this bacterium was obtained from environmental freshwater sample collected from the Chattahoochee river, USA. Uncultured so far. Basis of the assignment, genome sequence (GCA_010031735.1).

Description of "Candidatus Jistubacteraceae" fam. nov.

"*Candidatus* Jistubacteraceae" (Jis.tu.bac.te.ra'ce.ae, N.L. fem. n. "*Candidatus* Jistubacter" type genus of the family; suff. -aceae ending to denote a family; N.L. fem. pl. n. "*Candidatus* Jistubacteraceae" the family of genus "*Candidatus* Jistubacter").

The family "*Candidatus* Jistubacteraceae" is defined based on phylogenetic analyses of concatenated orthologous genes of uncultured representatives from various freshwater and terrestrial environments. The family belongs to the order *Rickettsiales*, and currently contains one genus, "*Candidatus* Jistubacter".

Description of "*Candidatus* Arkhamia obscura" gen. nov. sp. nov.

"*Candidatus* Ar.kha'mi.a ob.scu'ra". (Ar.kha'mi.a: N.L. fem. n., dedicated to Arkham, fictional town in Massachussets created by Howard Phillips Lovecraft; ob.scu'ra. L. fem. adj. obscura, hidden, unseen, as the bacterium was not directly observed). The genome sequence of this

bacterium was found in an environmental soil sample from Massachussets, USA. Basis of the assignment, genome sequence (GCA_013288575.1).

Description of “Candidatus Arkhamiaceae” fam. nov.

“*Candidatus* Arkhamiaceae” (Ar.kha.mi.a'ce.ae, N.L. fem. n. “*Candidatus* Arkhamia” type genus of the family; suff. -aceae ending to denote a family; N.L. fem. pl. n. “*Candidatus* Arkhamiaceae” the family of genus “*Candidatus* Arkhamia”).

The family “*Candidatus* Arkhamiaceae” is defined based on phylogenetic analyses of concatenated orthologous genes of uncultured representatives from freshwater and terrestrial environments. The family belongs to the order *Rickettsiales*, and currently contains one genus, “*Candidatus* Arkhamia”.

Supplementary Note 2: detailed comparative description of additional metabolic and functional features of *Rickettsiales*

A number of relevant traits/functions for the interaction with eukaryotic cells were selected for detailed analyses, described in the main text, in particular biosynthesis and uptake of metabolic precursors (i.e. amino acids and nucleotides), secretion/adhesion/motility apparatuses and putative effector molecules. A general overview of gene content variation and evolution in *Rickettsiales*, with a special focus on general "family-level" trends, as well as on the single newly characterised genomes, is presented below.

Housekeeping functions and DNA repair

Central housekeeping functions (DNA replication. cell division, general transcription, translation, chaperones) are conserved in all families and their representatives. The main DNA repair systems, namely mismatch repair (rare in *Gamibacteraceae* and *Deianiraeaceae*), nucleotide-excision repair, single- and double-strand break homologous repair/recombination, are present and overall common in all families. The most notable peculiarities in the novel genomes include the lack of nucleotide-excision and single-strand break repair systems in *Lyticum* and *Vederia*, and the split into two ORFs of *mutS* in *Bandiella*, possibly non-functional. Moreover, as previously observed in other unrelated *Rickettsiales* symbionts of ciliates [1-3], only the 5'-3' exonuclease domain of DNA polymerase I is present in *Fokinia cryptica* and *Lyticum*.

Carbohydrate and energy metabolism

The repertoire for carbohydrate and energy metabolism is overall rich in *Rickettsiales*, but quite variable between and within families (especially in crown families). The "canonical" repertoire composed by central reversible core of gluconeogenesis and glycolysis (linking fructose 1-6-bisphosphate and phosphoenolpyruvate) plus some gluconeogenesis-specific enzymes (malate dehydrogenase, fructose 1-6 bisphosphatase, phosphoenolpyruvate synthase), alongside with non-oxidative pentose-phosphate pathway, pyruvate dehydrogenase, Krebs cycle, electron transport chain (including NADH-quinone oxidoreductase, qor type NADPH-quinone oxidoreductase, alternatively followed by ubiquinol-cytochrome c oxidoreductase+cytochrome c oxidase, and/or by the bd-cytochrome ubiquinol terminal oxidase), and ATP synthase are present in all families.

The lineage-specific and possible convergent streamlining of the glycolysis/gluconeogenesis is quite frequent, up to retaining only the initial steps (leading to phosphoenolpyruvate), as in many "classical" *Rickettsiaceae* (namely the monophyletic lineage comprising all characterised organisms, thereby with the exclusion of "basal *Rickettsiaceae*", as previously defined [3]), including the novel genomes obtained in this study, but also occurring in other families, e.g. among *Midichloriaceae, Vederia* has the same subpathway, plus triose-phosphate isomerase. Pentose-phosphate pathway is missing in *Cyrtobacter*, and composed only by ribose-5-phosphate isomerase in *F. cryptica* and *Lyticum*. Moreover, genes belonging to the oxidative branch were found in basal *Rickettsiales*, in particular in *Mitibacteracae*.

Noteworthy is the case of cytochrome c oxidase, of which two distinct alternative forms are available, Cco or cbb3, the latter considered advantageous in anaerobic conditions [4]. Basal *Rickettsiales* present both forms, while in each crown family in general just one alternative is present, namely Cco in *Rickettsiaceae*, *Anaplasmataceae*, *Diomedesiaceae*, and cbb3 in *Midichloriaceae*, *Gamibacteraceae*, and neither in most *Deianiraceaeae* (lacking also ubiquinol-cytochrome c oxidoreductase, thus likely exploiting only their bd alternative oxidase). Such a pattern is clearly not consistent with phylogenetic relationships.

Multiple, probably independent, instances of combined reduction of Krebs cycle and electron transport chain were found. Besides some previously known cases [1, 3, 5], among the newly sequenced *Midichloriaceae*, *Bandiella* lacks the bd oxidase, conversely *Cyrtobacter* lacks c reductase and oxidase, while both *F. cryptica* and *Lyticum* miss the (almost) entire Krebs cycle and possess only NADH-quinone and bd terminal oxidases. The latter two organisms probably exploit NADH from other sources for ATP synthesis [1], or only in case of *Lyticum*, the succinyl-CoA synthase. *Vederia* and the *Midichloriaceae* symbiont of *Reticulomyxa* are fully devoid of Krebs and electron transport, and possibly take ATP directly from their hosts or exploit alternative paths, such as a peculiar protein homologous to the glycolytic enzyme pyruvate kinase in *Vederia*.
As previously observed [6], glyoxylate cycle is present in basal *Rickettsiales*, in particular *Mitibacteraceae*. Additionally, the phosphoenolpyruvate carboxylase, which forms oxaloacetate, thus potentially fuelling both Krebs and glyoxylate cycle, was found in basal *Rickettsiales* and in few crown *Rickettsiales* that are early diverging in the respective families, e.g. *Jidaibacter* and the *Rickettsiaceae* endosymbiont of *Amblyomma* Ac37b, while the nitrate reductase, which may enable the use of this compound as terminal electron acceptor [7], was found in *Mitibacteraceae*, *Jistubacteraceae*, and *Gamibacteraceae*.

The main biosynthetic pathways overall mostly follow comparable presence/absence patterns among *Rickettsiales*. They are present in all families, and experienced multiple, probably independent, reductions or even full losses in single organisms/lineages in crown *Rickettsiales* families, as a possible consequence of the ability to obtain intermediates or final products directly from their hosts.

<u>Lipids and membranes</u>

The biosynthesis of lipids and phospholipids is well conserved among *Rickettsiales*, with exceptions including the pathway holes (lack of plsX and plsY) in *Rickettsia* [8] and many classical *Rickettsiaceae* (including the newly sequenced genomes), but not in basal *Rickettsiaceae*. Concerning isoprenoid precursor biosynthesis, a previously proposed evolutionary scenario appears confirmed [3], proposing an ancestral and widespread methylerythritol pathway, and a replacement by a horizontally-acquired mevalonate pathway in few representatives of *Deianiraeaceae* and classical *Rickettsiaceae*, which include *Trichorickettsia* and the two symbionts of Apicomplexa [9, 10], possibly indicating some link with Alveolata hosts.

Peptidoglycan biosynthesis likely experienced an independent reduction in different *Rickettsiales* sublineages for transpeptidase and transglycosylase activities, in particular *mrcB* gene is absent in *Deianiraeaceae*, *Anaplasmatacae* and many *Rickettsiaceae* (except basal members). Different further steps of streamlining can be observed in lineages such as *Orientia* [11] and multiple *Anaplasmataceae*, up to the condition of *Ehrlichia*, lacking multiple genes related to such activities (e.g. *fstI*, *mrdA*, *mrcA*). Lipopolysaccharide (LPS) biosynthesis is globally absent in *Anaplasmataceae*, and in some members of other crown families, such as *Orientia* and *Fokinia solitaria* [1, 11], as well as *F. cryptica* and *Cyrtobacter*, while *Lyticum* is equipped with most LPS biosynthetic steps, with the exclusion of those producing deoxy-manno-octulosonate.

An interesting pattern is found for the synthesis of N-acetylglucosamine, precursor of both peptidoglycan and LPS in the classical *Rickettsiaceae.* The full pathway from fructose was present in the ancestor, with the last two steps catalysed by distinct polypeptides, due to a split of *glmU* gene, a situation conserved in *Megaira polyxenophila* and *Tisiphia*. Then, most of the pathway was lost independently in *Rickettsia* [8] and *Megaira venefica*, both retaining only the N-terminus of *glmU*, catalysing the very last step.

The synthesis of polyhydroxyalkanoate (PHA) granules as storage compounds, present in all four basal families, is quite common in *Rickettsiaceae*, and found also in several *Midichloriaceae* (including all the novel genomes except *Bandiella*) and in few *Anaplasmataceae* from aquatic environments such as *Xenolissoclinum*.

Cofactors/vitamins

Biosynthetic pathways for cofactors/vitamins are quite common in *Rickettsiales*, differently from those for aminoacids and nucleotides (see below), though some lineages present reduction or complete absence of the pathways for one or more cofactors, with some degree of consistence with the lack of enzymes requiring them. Very common in all families are the synthesis of lipoate, ubiquinone (notable the exception of *Vederia*), and NADP from NAD (with *Lyticum* among few exceptions). Other quite frequent pathways are those for folate and heme (the latter absent in *Cyrtobacter*, *F. cryptica*, *Vederia* and endosymbiont of *Reticulomyxa*, consistently with the lack of cytochrome c reductase and oxidase).

The case of biotin is noteworthy, also considering a beneficial role hypothesised for this vitamin in some invertebrate hosts of *Rickettsiales*, and the multiple previous reports of putative horizontal-gene-transfer events involving genes of this biosynthetic pathway [12-14]. The presence/absence of this pathway in *Rickettsiales* somehow inversely correlates with the one of the bioY biotin transporter, conversely present in representatives of genus *Wolbachia*, the *Midichloriaceae* endosymbiont of *Reticulomyxa*, as well as a group of closely related *Rickettsiaceae* (*Rickettsia*, *Tisiphia*, *Trichorickettsia*, *Megaira*, and symbiont of *Cardiosporidium cionae*). Some exceptions exist, e.g. *Trichorickettsia* and *Rickettsia* endosymbiont of *Ixodes scapularis* have both, while many other classical *Rickettsiaceae* have neither (e.g. *Orientia*, *Sarmatiella*, the latter possibly

representing a recent loss of the biosynthesis [3]). Similarly, a single and short gene (*bioA*) is a possible sign of recent loss of the pathway also in *Bandiella*.

NAD synthesis is quite rare in *Rickettsiaceae* (exceptions include *Sarmatiella* and *Megaira venefica*), while in *Midichloriacae*, if present, it is represented only by the final two steps (among the novel genomes only in the *Reticulomyxa* endosymbiont). Similarly, FAD synthesis is quite rare among *Rickettsiaceae*, being found only in the basal representatives (such as endosymbionts of *Stachyamoeba* and of *Amblyomma* Ac37b), while in the novel *Midichloriaceae* genomes, except for *F. cryptica*, at least the last step is present. For what concerns pantothenate and CoA, the pathway is overall present in all families at variable levels of completeness, starting from the minimal final steps (coaDE), found in most representatives (among the novel genomes, the exception is *Lyticum*, while the richest is *F. cryptica*, from the bifunctional dfp on), up to including also the initial steps from 3-methyl-2-oxobutanoate, common only in the basal families and in *Gamibacteraceae*. Pyridoxal phosphate synthesis is quite rare in crown families, with earlier steps of the pathway being more common in *Rickettsiaceae* and *Midichloriaceae*, while differences can be seen through the order for the final steps, with most *Rickettsiales* (including *Anaplasmataceae*) displaying pdxJ and pdxH, while *Midichloriaceae* display mostly the alternative pdxT and pdxS, as well as the salvage pathway. The latter pathway is the only one present in *F. cryptica*, while *Vederia* is fully devoid of pathways for pyridoxal production/recycling. The presence of biosynthetic pathways for thiamine follows a comparable pattern, with initial steps (thiH) only present in *Mitibacteraceae*, successive steps found also in the other basal families, *Anaplasmataceae* and DDG clade (*Gamibacteraceae* and *Diomedesiaceae* in particular), and the final steps (thiD, thiE and thiL) rather common in all families, though rare in classical *Rickettsiaceae*. This pathway is uncommon in the novel genomes, and only its final step is present solely in *Vederia* and *Bandiella*. Additional cofactor biosynthetic pathways, such as molybdopterin and cobalamin are quite rare in *Rickettsiales*

and found mostly/exclusively in basal families, namely both cofactors in *Mitibacteraceae*, and the former also in *Gamibacteraceae*.

Additional functions

Multiple *Rickettsiales* bear signs of the presence of different kinds of mobile elements, including plasmids (among which identified plasmidic contigs in published [5, 15] and in novel genomes, or plasmidic-like genes such as parA and toxin-antitoxins in other assemblies including e.g. *Vederia*, *Cyrtobacter* and *M. venefica*), phages (e.g. *Cyrtobacter*, the two *Megaira*, *Vederia*, and *Trichorickettsia*, the latter consistent with previous observations [16]), transposons (e.g. *Cyrtobacter*, the two *Megaira*, *Bandiella*), and retrotransposons (e.g *Bandiella* and *M. polyxenophila*).

Another noteworthy feature is the presence of cytoskeletal bactofilin proteins, such as the one previously described in *Deianiraea* [2], in members of the three DDG-clade families, as well as in the basal *Rickettsiales*.

Interestingly, some detoxification and inorganic nutrient uptake proteins, previously found only in basal *Rickettsiales* [6], were also found in some crown lineages. Namely, KatG catalase/peroxidase and arsenic resistance mechanisms (efflux pump and/or reductase) were found in several *Gamibacteraceae* and even in *Diomedesiacae*, whereas ammonium transporters were found in *Diomedesiaceae*, *Gamibacteracaeae*, and a basal *Rickettsiaceae* MAG.

Supplementary Note 3: genome assembly procedures

In this work, the novel genome sequences of 9 *Rickettsiales* bacteria were assembled. This was obtained starting from 7 ciliate strains/populations (with *Euplotes woodruffi* NDG2 harbouring 2 different *Rickettsiales* bacteria) and from one sample derived from a previous study [17] on the foraminiferan *Reticulomyxa filosa*.

Sample origin and maintenance

Among, the seven ciliate strains/populations employed in this study, five were obtained and described in previous studies [16, 18-23]. The origin and laboratory maintenance of the other two strains is reported below.

The population of *Plagiopyla frontata* IBS-3 was obtained from a brackish water sample collected in May 2018 from the Baltic Sea in Inkoo (Finland), following a previously described protocol for the laboratory propagation of anaerobic ciliates [24].

The monoclonal strain *Euplotes woodruffi* NDG2 was isolated from a water sample collected on 15th March 2013 at Naval Dockyard Gate in Visakhapatnam (Andhra Pradesh, India). It was maintained in the laboratory by regular feeding (every 1-2 weeks) with *Dunaliella tertiolecta*, grown in artificial water at 5‰ salinity as previously described (e.g. [23]).

Sample preparation and Illumina sequencing

Four ciliates (*Paramecium biaurelia* US_Bl 11III1, *Paramecium nephridiatum* Sr 2-6, *Euplotes woodruffi* NDG2, *Plagiopyla frontata* IBS-3) were subjected to whole-genome amplification (WGA) with the REPLI-g Single Cell Kit (Qiagen), starting from a limited number of cells (see Supplementary Data 1), and following the protocol for live cells (as in [3]). This procedure allows to obtain a sufficient amount of DNA for Illumina sequencing even for samples (*Plagiopyla*

frontata IBS-3) that are not easily propagated in the laboratory, as well as to minimise the presence of food bacteria contamination thanks to the manual washing procedure, which in any case would not be amenable for larger amounts of host cells. For the sample *Euplotes harpa* BOD18,which was not available as live material, WGA was performed from a DNA extract obtained in a previous study [23], following the dedicated protocol of the Repli-G kit.

For the sample *Paramecium biaurelia* USBL-36I1, 1.2 liters of laboratory culture (ca. 1,500-2,000 *Paramecium* cells/ml) were processed and DNA was extracted for sequencing using a modified CTAB protocol, as previously described [1].

For sample *Paramecium multimicronucleatum* Kr154-4, 400 ml of laboratory culture (ca. 600 *Paramecium* cells/ml) were process and DNA extraction was performed following a phenol-chloroform protocol [25].

Each of the seven DNA samples from ciliates was processed through a Nextera XT library, and sequenced by Admera Health (South Plainfield, NJ, USA) on a Illumina HiSeq X machine producing 2x150 bp paired-end reads (Supplementary Data 1).

The reads from the *Reticulomyxa filosa* sample [17], were kindly provided by Gernot Gloeckner and Marco Groth (University of Koeln, Germany).

Sample preparation and Oxford Nanopore sequencing

In order to perform Nanopore sequencing, a large enough amount of DNA extract was obtained from *Euplotes woodruffii* NDG2 as follows. A total of 3 liters of NDG2 culture were obtained (~ 300,000 *Euplotes* cells). The culture was passed through a sieve with 20 µm-sized pores, so that cells were retained by the filter, resulting concentrated in the final 12 ml of unfiltered liquid, which was then centrifuged at 5000 g for 5 min. The pellet was resuspended in 2 ml of distilled water, and centrifuged again. The final pellet was fixed in ethanol and stored until DNA extraction, performed

with the NucleoSpin™ Tissue Kit (Macherey-Nagel™). The DNA extract was processed through a SQK-LSK109 ligation-sequencing library, following the manufacturer's instructions. Afterwards, library was loaded onto a FLO-MIN106 flowcell and sequenced on a Minion device using the MinKNOW software 18.12.9, for 72 total hours.

Basecalling was then performed with the guppy 5.0.11 software by Oxford Nanopore. Then, reads were processed with Porechop 0.2.4 [26] with default options. Quality of the reads was assessed with NanoPlot 1.23.0 [27]. The following steps are described in the detailed report on the selective assembly of the sample NDG2 below.

Selective assembly of the *Rickettsiales* bacterial genomes from shotgun sequencing of their host samples

In this section a general account of the procedures is presented. Subsequently, a detailed report for each sample is available.

For each sample, after a quality assessment with FastQC [28], the reads were assembled using SPAdes 3.6 [29] with default settings (except for the *Reticulomyxa filosa* sample, which was assembled with Unicycler 0.4.8 [30]), obtaining a "preliminary assembly".

Then, a multi-step procedure was applied, in order to select only those contigs belonging to the symbiont of interest and discard those belonging to the host and to additional organisms present in the sample (e.g. residual food, additional associated bacteria), as described previously (e.g. [2]).

For this purpose, the blobology pipeline was applied [31]. Briefly, the contigs of the "preliminary assembly" were classified according to their length, GC% content, sequencing coverage based on reads mapped with Bowtie2 [32], and NCBI taxonomy of the best megablast hit on NCBI nucleotide. Results were visualised by the R package ggplot2 [33]. Accordingly, a set of contigs was selected, considering also ribosomal RNA genes identified with barrnap [34], using default options for *Bacteria*. Thus, reads mapping on the selected contigs were reassembled separately with

SPAdes. Results were manually revised prior and after reassembly, by examining blastp results on NCBI nr protein database after annotation with Prokka [35], as well as taking advantage of Bandage [36] for visualisation of assembly graphs to identify connections between contigs. The "contigs.fasta", "contigs.fastg", "scaffolds.fasta", and "scaffolds.fastg" SPAdes output files were considered, and, unless specified, reported results refer to the "contigs" files. Only for the sample *E. woodruffi* NDG2, for which Nanopore reads were produced, re-assembly was directly performed as hybrid (Illumina+Nanopore) with Unicycler 0.4.8-beta (see details below).

Moreover, an additional round of screening was performed in order to account for potential sequences belonging to *Rickettsiales* symbiont(s) in contigs falling outside the selected sets of contigs, especially considering possible "hybrid" contigs between host and symbiont, which may otherwise have been discarded in case of best hit on host sequences. In detail, all the respectively annotated ORFs [37] where queried on NCBI nr protein database with DIAMOND [38], applying an e-value threshold of 1e-5. Contigs with at least one ORF with a best hit on *Bacteria* were inspected manually (by their blast and DIAMOND hits), and, in case, further contigs selections and/or refinements of the previous ones were made, followed by re-assemblies and manual revision, as described above.

Genome closing by PCR and Sanger sequencing

After selecting the *Rickettsiales* genome sequences from SPAdes assemblies, two samples (*P. multimicronucleatum* 12 and *P. biaurelia* US_Bl 11III1) were selected, based on assembly quality, for molecular biology based finishing. PCR primers were designed next to the ends of larger contigs, and PCR reactions were performed in order to test their connections and join them into larger contigs/scaffolds. PCRs were performed with TaKaRa Ex Taq and reagents (Takara Bio, Japan), following manufacturer's instructions (each primer with final concentration 0.5 µM). Successful PCR results were confirmed by bidirectional Sanger sequencing (after purification with

the EuroGOLD Cycle-Pure kit - EuroClone, Italy) performed by GATC Biotech (Germany) with amplification primers and/or internal primers (see sections below for details on each sample) .

***Paramecium multimicronucleatum* Kr154-4 hosting "*Ca.* Trichorickettsia mobilis"**

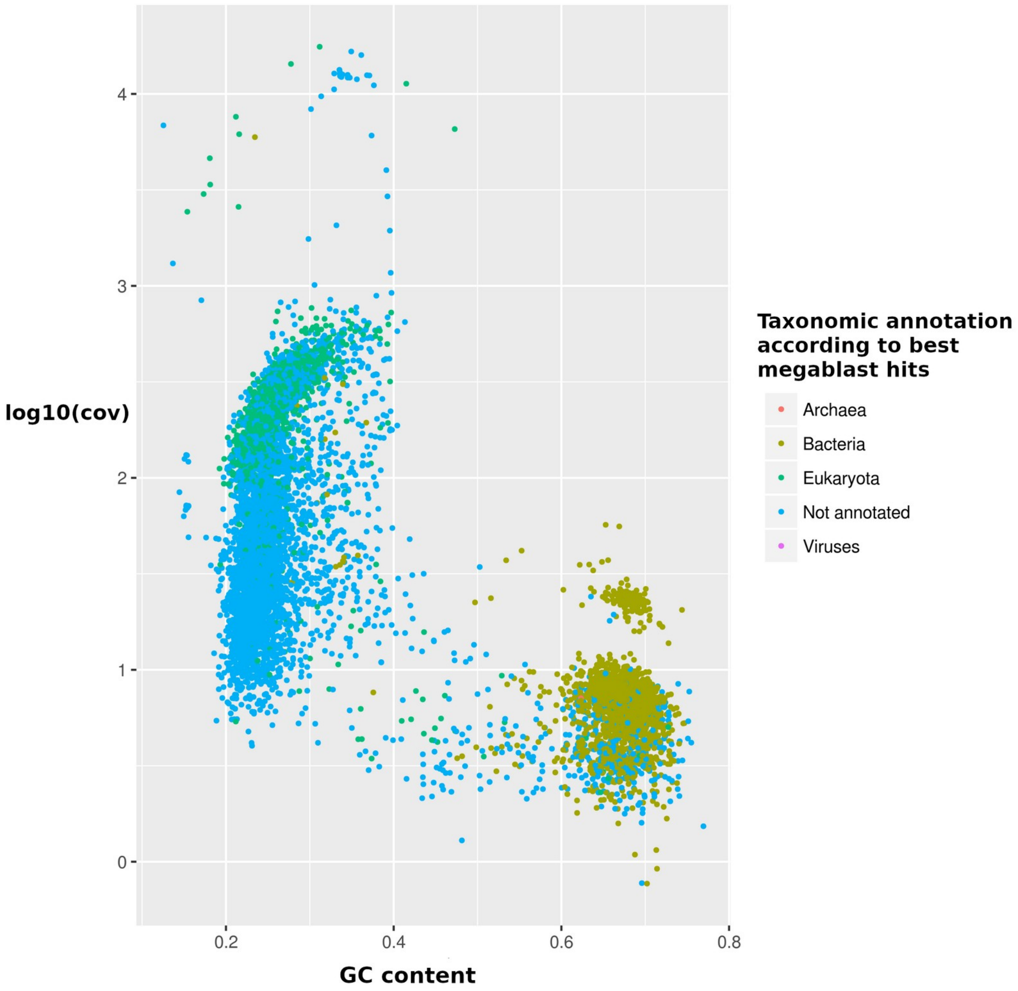

Supplementary Figure 1 Plot of the preliminary assembly contigs according to their GC content and $\log_{10}$ of sequencing coverage, coloured according to the respective best megablast hit. Only contigs with length higher than or equal to 1000 bp are shown for viewers' clarity.

In total, 24 full-length or partial rRNA genes were identified in the "preliminary assembly" (Supplementary Figure 1; Supplementary Data 4):

- 13 complete or partial rRNA genes had best megablast hits on nuclear or mitochondrial *Paramecium* spp., and were thus assigned to the host. The respective contigs ranged from 18.59 to 17607.88 in sequencing coverage
- 3 rRNA genes (16S, 23S and 5S) were identified as belonging to the symbiont, based on their best megablast hits on "*Ca*. Trichorickettsia" (16S) or *Rickettsia* (23S), or for being in the same contig as the 23S (5S). The respective contigs ranged from 36.57 to 38.55 in sequencing coverage, and ~0.34 GC content
- 8 remaining complete or partial rRNA gene sequences had megablast hits on other organisms. The respective contigs ranged from 3.85 to 356.00 sequencing coverage, having generally a higher GC content than the those assigned to "*Ca*. Trichorickettsia" (>0.44), with just one exception (0.26 GC)

Taking into account the plot and the rRNA gene features, we designed a "selection from preliminary assembly" for "*Ca*. Trichorickettsia", including all contigs with $\log_{10}$ of coverage lower than 2.5 and GC content between 0.25 and 0.4, with the exclusion of those having best megablast hit on members of order Peniculida (to with the *Paramecium* host belongs).

The reads mapping to "selection from preliminary assembly" were extracted and reassembled separately with SPAdes default settings: "final SPAdes assembly" (10,563,744 bp, 11700 contigs, N50=2,955 bp; L50=570). In this assembly, a selection was made of contigs belonging to "*Ca*. Trichorickettsia (14 contigs, 1,468,577 bp; N50=1,094,380 bp; L50=1; Supplementary Figure 2).

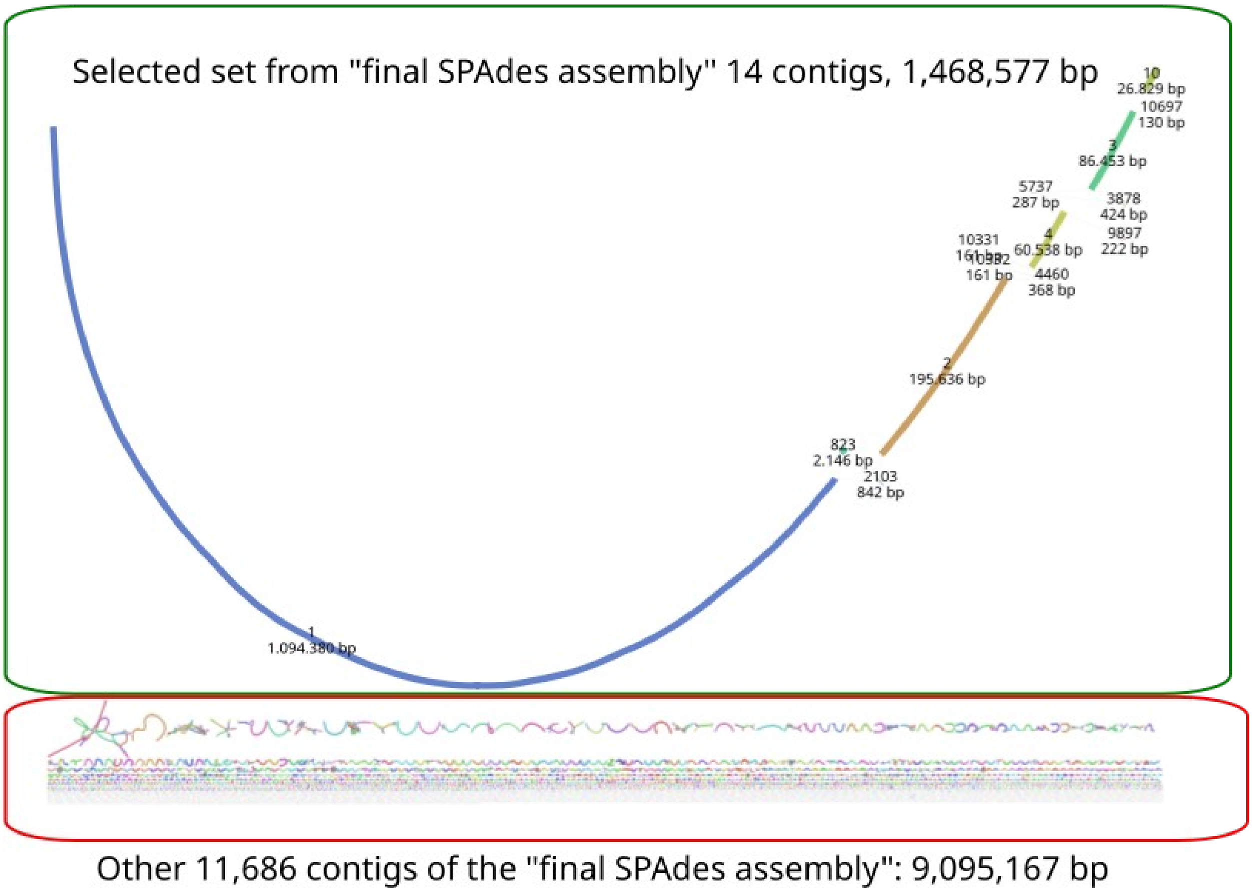

Supplementary Figure 2 Visual representation of the 11700 contigs of the "final SPAdes assembly" and the relative connections in the assembly graph, obtained with the Bandage software. The green square highlights the selected set of contigs. The red square highlights the remaining contigs of the assembly.

Then, primers were designed next to the ends of the 6 larger contigs in the selected set, and PCR reactions were performed in order to identify/confirm junctions among those contigs. The combinations of primers used for amplification and sequencing, primer sequences, and the reaction protocols used are reported below (Supplementary Table 1, 2, 3, 4).

Supplementary Table 1: primer combinations for closing "*Ca.* Trichorickettsia mobilis" genome

Reaction	Amplification primer 1	Amplification primer 2	Sequencing primer 1	Sequencing primer 2	Protocol
1	Tricho_10Fa	Tricho_3Ra	Tricho_10Fb	Tricho_3Rb	A
2	Tricho_3F	Tricho_4R	Tricho_3F	Tricho_4R	A

3	Tricho_4Fa	Tricho_2Ra	Tricho_4Fb	Tricho_2Rb	A
4	Tricho_2F	Tricho_823F	Tricho_2F	Tricho_823F	B
5	Tricho_823Ra	Tricho_1Fa	Tricho_823Rb	Tricho_1Fb	A
6	Tricho_1Ra	Tricho_10R	Tricho_1Rb	Tricho_10R	A

Supplementary Table 2: primer sequences for closing "*Ca*. Trichorickettsia mobilis" genome

**Primer**	**Sequence (F: forward; R: reverse)**
Tricho_1Fa	5'-TTGGCAGAAGCACTAATTGAG-3'
Tricho_1Fb	5'-TAACTGGTAATGATCTCCACG-3'
Tricho_1Ra	5'-AGCGAAGCAGTGACATTGG-3'
Tricho_1Rb	5'-TACAGTGACCACAGTTCGG-3'
Tricho_2F	5'-CCCTCATATCTAGGTTGAGC-3'
Tricho_2Ra	5'-TGATAACATACGAGTAGCAGC-3'
Tricho_2Rb	5'-TTAACGCAGGCACTACTCC-3'
Tricho_3F	5'-TCAACGCTCTTTCCTGTGG-3'
Tricho_3Ra	5'-AACCTGCCGTGAGGTGC-3'
Tricho_3Rb	5'-ATCAGTAGCAGCGCCTAC-3'
Tricho_4Fa	5'-AACAGCAGCATGCACTGAG-3'
Tricho_4Fb	5'-CTACCCTCATAGGGTATCC-3'
Tricho_4R	5'-CGACTCAAGGCTCGTGG-3'
Tricho_10Fa	5'-CCAGAGTCCGTTAGAATAGG-3'
Tricho_10Fb	5'-GCCCCAGGCCTTAACATC-3'
Tricho_10R	5'-GTAATTGGAGCCTTAGAGTTC-3'
Tricho_823F	5'-CTTGATTAGGTCTTGCTTCC-3'
Tricho_823Ra	5'-ACTTATCTCGATAGGCTGAAC-3'
Tricho_823Rb	5'-CTGACCTCCTCTATCAAGC-3'

Supplementary Table 3: PCR protocol A (standard) for closing "*Ca*. Trichorickettsia mobilis" genome

94°C	3 min	
94°C	30 s	40 cycles
50°C	30 s	
72°C	4 min	
72°C	10 min	

Supplementary Table 4: PCR protocol B (touchdown) for closing "*Ca*. Trichorickettsia mobilis" genome

94°C	3 min	
94°C<br>56°C<br>72°C	30 s<br>30 s<br>4 min	5 cycles
94°C<br>53°C<br>72°C	30 s<br>30 s<br>4 min	10 cycles
94°C<br>50°C<br>72°C	30 s<br>30 s<br>4 min	30 cycles
72°C	10 min	

Thanks to these PCR experiments, it was possible to close the "*Ca*. Trichorickettsia mobilis" genome into a single circular scaffold (1,470,091 bp).

In addition, the DIAMOND hit-based inspection of contigs of the "preliminary assembly" outside the "selection from preliminary assembly" evidenced the presence of putative bacterial/*Rickettsia*-like sequences in higher coverage contigs, which were assigned to putative "*Ca*. Trichorickettsia" plasmid(s). Thus, we designed an additional "selection for plasmids" from the "preliminary assembly", including all contigs with $\log_{10}$ of coverage higher than 1.5.

The reads mapping to "selection for plasmids" were extracted and reassembled separately with SPAdes (based on different attempts, the option -k 21,33,55,77,99 was chosen): "plasmid re-assembly" (39,539,931 bp, 4182 contigs, N50=82,014 bp; L50=128). In this assembly, at least 3 plasmids were identified. Two plasmids were assembled in single circular contigs, 57,766 bp and 17,134 bp long, respectively. The other plasmidic sequences were assembled into 69 contigs (457,770 bp), and may even constitute one or more additional plasmids: "additional plasmid(s)" (Supplementary Figure 3).

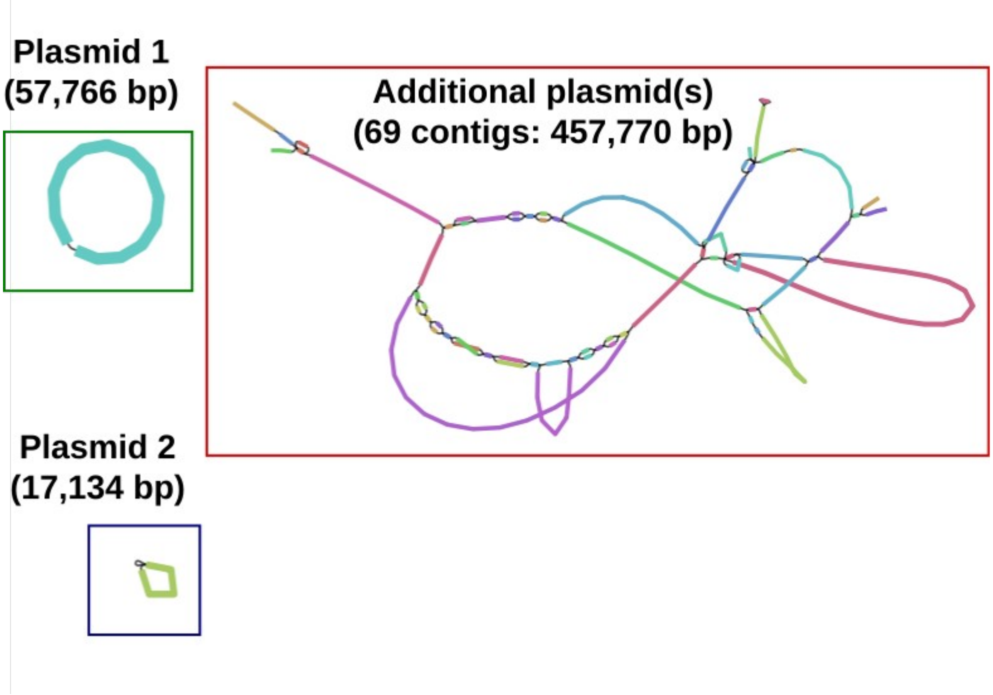

Supplementary Figure 3 Visual representation of the selected 71 contigs of the “plasmid re-assembly” and the relative connections in the assembly graph, obtained with the Bandage software. The green square highlights the plasmid 1, the blue square highlights plasmid 2, and the red square highlights the “additional plasmid(s)”

Finally, the assembly of “additional plasmid(s)” was refined by removing all contigs <5000bp containing no validated ORF (i.e. with no hits on NCBI nr or with hits only on hypothetical proteins, transposases, phages). Thus, the “final assembly” of the “additional plasmid(s)” included 36 contigs (420,184 bp; N50=28,079 bp; L50=26).

In total, the assembly of “*Ca*. Trichorickettsia”, including chromosome and all plasmids, consisted of 39 contigs (1,965,805 bp).

***Paramecium nephridiatum* Sr 2-6 hosting “*Ca.* Megaira venefica”**

The “preliminary assembly” was constituted by 21,901 contigs (21,515,176 bp; N50 = 2,009 bp; L50 = 2817)

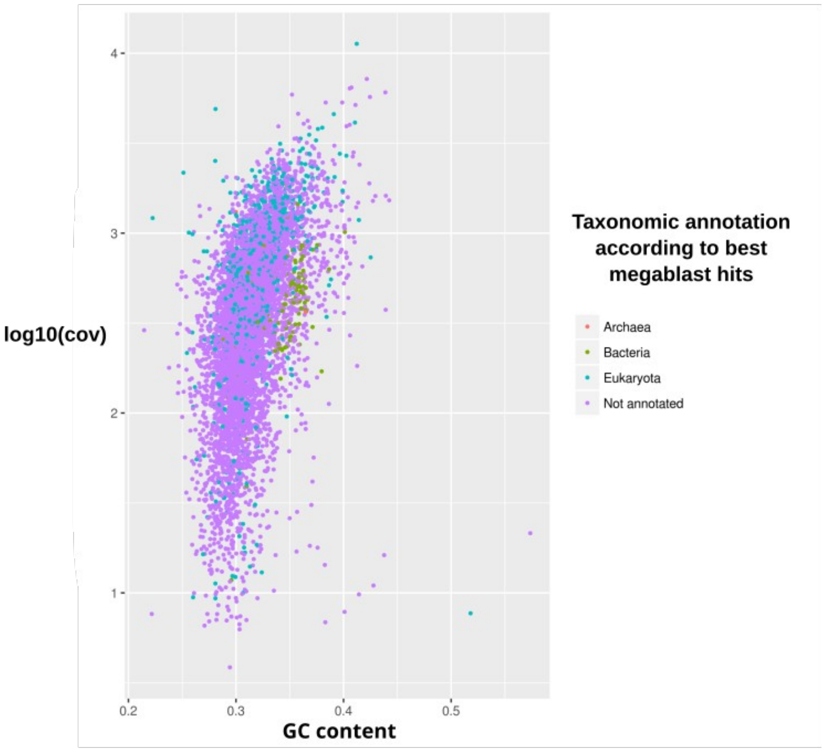

Supplementary Figure 4 Plot of the preliminary contigs of the sample Sr 2-6 according to their GC content and log_{10} of sequencing coverage, coloured according to the respective best megablast hit. Only contigs with length higher than or equal to 1000 bp are shown for viewers' clarity.

In total, 36 full-length or partial rRNA genes were identified in the "preliminary assembly" (Suppelementary Figure 4; Supplementary Data 4):

- 34 complete rRNA genes had best megablast hits on nuclear *Paramecium* sequences, and were thus assigned to the host. The respective contigs displayed 7.28 and 11285.11 sequencing coverage
- 2 rRNA gene sequences (16S and 5S) were identified as belonging to "*Ca*. Megaira venefica", based on their respective megablast hits. Namely the 16S rRNA gene had high identity (>99%) with "*Ca*. Megaira spp.", while 5S rRNA gene had no "informative" hit (low identity hits ~80% with several other proteobacterial sequences), but its full-length contig had best hit on *Rickettsia japonica*. The two respective contigs displayed 595.70 and 569.53 sequencing coverage, and ~0.36 GC content.

As done for the other samples, we selected contigs based on sequencing coverage, GC content, and best megablast hits. Then, we reassembled the reads mapping on these contigs. However, no significant improvement of the assembly of "*Ca*. Megaira venefica" genome was observed (not shown). Therefore, selection of "*Ca*. Megaira venefica" sequences was performed directly on the "preliminary assembly", counting 628 contigs (2,047,029 bp).

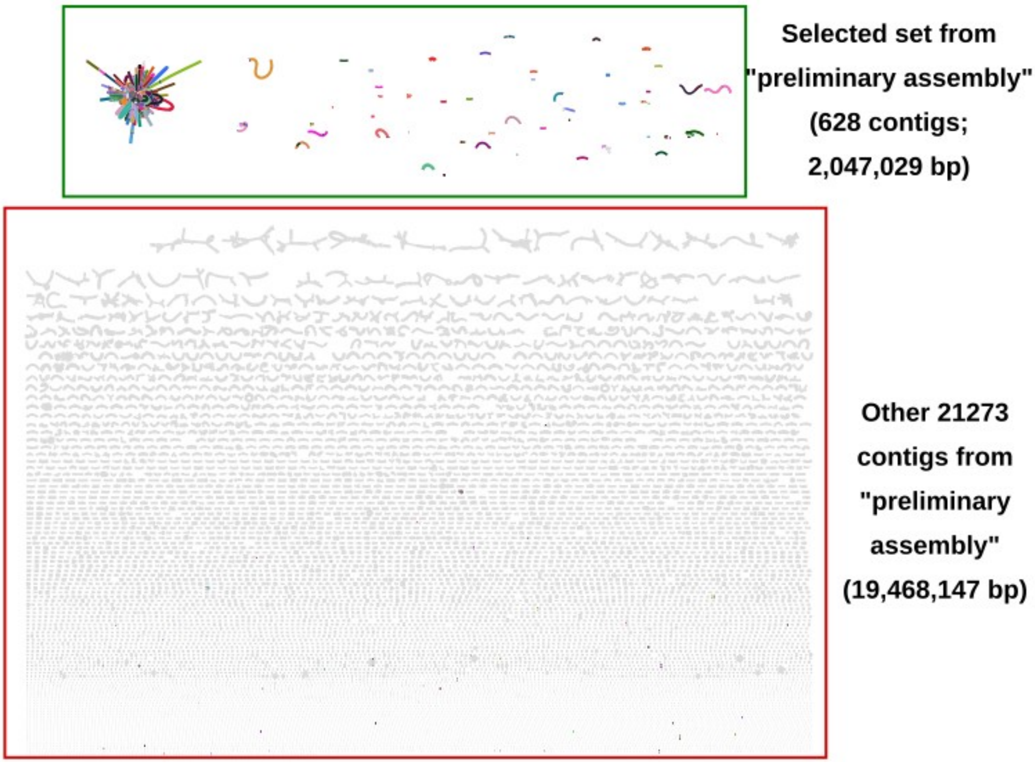

Supplementary Figure 5 Visual representation of the 21,901 contigs of the "preliminary assembly" and the relative connections in the assembly graph, obtained with the Bandage software. The green square highlights the selected set of contigs, the red square highlights the remaining contigs of the assembly.

The relatively high fragmentation degree and the presence of several small contigs in the selection from "preliminary assembly" were interpreted as due to the presence of many repeated elements in the genome of "*Ca*. Megaira venefica", considering the high connection degree (Supplementary Figure 5) and the presence of multiple transposase genes. Accordingly, and accounting both for the careful manual revision applied and for the comparative BUSCO results with other *Rickettsiales*

(Supplementary Data 2), we concluded that this assembly likely contains the whole genome sequence (in terms of gene sequences) of "*Ca*. Megaira venefica".

Finally, the assembly was refined by removing all contigs <3000bp containing no validated ORF (i.e. with no hits on NCBI nr or with hits only on hypothetical proteins, transposases, phages). Thus, the "final assembly" of "*Ca*. Megaira venefica" counted 281 contigs (1,862,567 bp; N50=10,544 bp; L50=58).

Euplotes woodruffi NDG2 hosting "*Ca.* Megaira polyxenophila" and "*Ca.* Bandiella woodruffii"

The "preliminary assembly" was constituted by 26,682 contigs (15,101,154 bp; N50 = 772 bp; L50 = 1633)

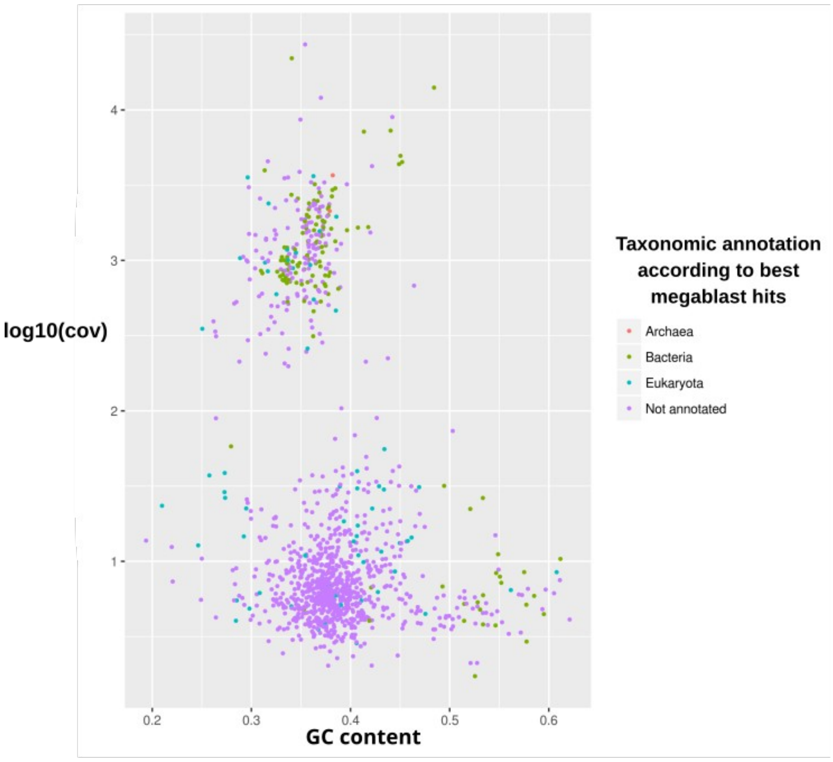

Supplementary Figure 6 Plot of the preliminary contigs of the sample NDG2 according to their GC content and log_{10} of sequencing coverage, coloured according to the respective best megablast hit. Only contigs with length higher than or equal to 1000 bp are shown for viewers' clarity.

In total, 17 full-length or partial rRNA genes were identified in the "preliminary assembly" (Supplementary Figure 6; Supplementary Data 4):

- 3 partial rRNA genes had best megablast hits on nuclear *Euplotes* sequences, and were thus assigned to the host. The two respective contigs displayed quite lower sequencing coverage (31.12 and 37.25)
- 3 rRNA genes (5S, 16S, 23S) formed a putative operon on a single contig. Based on megablast hits, they were identified as belonging to *Polynucleobacter necessarius*, a betaproteobacterial obligate symbiont of many *Euplotes spp.*, including *E. woodruffi* (e.g. [39]). This contig displayed 4363.69 sequencing coverage
- 3 rRNA genes (5S, 16S, 23S) were identified as belonging to a "*Ca*. Bandiella woodruffii" symbiont, based on their respective best megablast hits on "*Ca*. Midichloriaceae" bacteria. The precise species assignment was due to the 99.70% identity of the 16S rRNA gene with the type "*Ca*. Bandiella woodruffii" endosymbiont of *E. woodruffi* Sq1 [39], though not representing the best hit due to the relatively short length of this sequence. The two respective contigs displayed 648.22 and 1664.01 sequencing coverage
- 3 rRNA gene sequences (16S, 23S and 5S) were identified as belonging to a "*Ca*. Megaira polyxenophila" symbiont, based on their respective megablast hits, including for the 16S rRNA gene hits with high identity (>99%) on "*Ca*. Megaira polyxenophila" sequences (including symbiont of *Carteria cerasiformis* [40]), and for the 23S rRNA hits on *Rickettsia japonica*. The two respective contigs displayed 899.13 and 914.37 sequencing coverage
- 5 remaining complete or partial rRNA gene sequences had megablast hits on other organisms. The respective contigs had comparably much lower coverage (<26.42) than the ones assigned to the two *Rickettsiales* bacteria

Taking into account the plot and the rRNA gene features, we designed a “selection from preliminary assembly” targeting the two *Rickettsiales*, including all contigs with log_{10} of coverage higher than 1.5, and excluding those with best megablast hit on *Polynucleobacter*.

The reads mapping to this “selection from preliminary assembly” were extracted. In parallel, the corresponding Nanopore reads were extracted by performing a blastn search of the total reads as queries againts the contigs of the same “selection from preliminary assembly” (default megablast options) and keeping all reads with hits. The hybrid assembly of these two sets of reads “Unicycler hybrid re-assembly” included 195 contigs (3,240,079 bp, N50=503,502 bp; L50=2). In this assembly, two selection were made for sequences belonging respectively to “*Ca*. Megaira polyxenophila” (28 contigs, 1,995,794 bp), and to “*Ca*. Bandiella woodruffii” (163 contigs; 1,205,980 bp; Supplementary Figure 7). Each selection contained multiple putative plasmids (at least 9 for “*Ca*. Megaira”, 2 for “*Ca*. Bandiella”), which were assigned to each organism based on the connection in the Illumina assembly graphs, as well as on the sequence similarity of the annotated genes among them and with published sequences (e.g. published plasmidic sequences in *Rickettsia* for sequences assigned to “*Ca*. Megaira”).

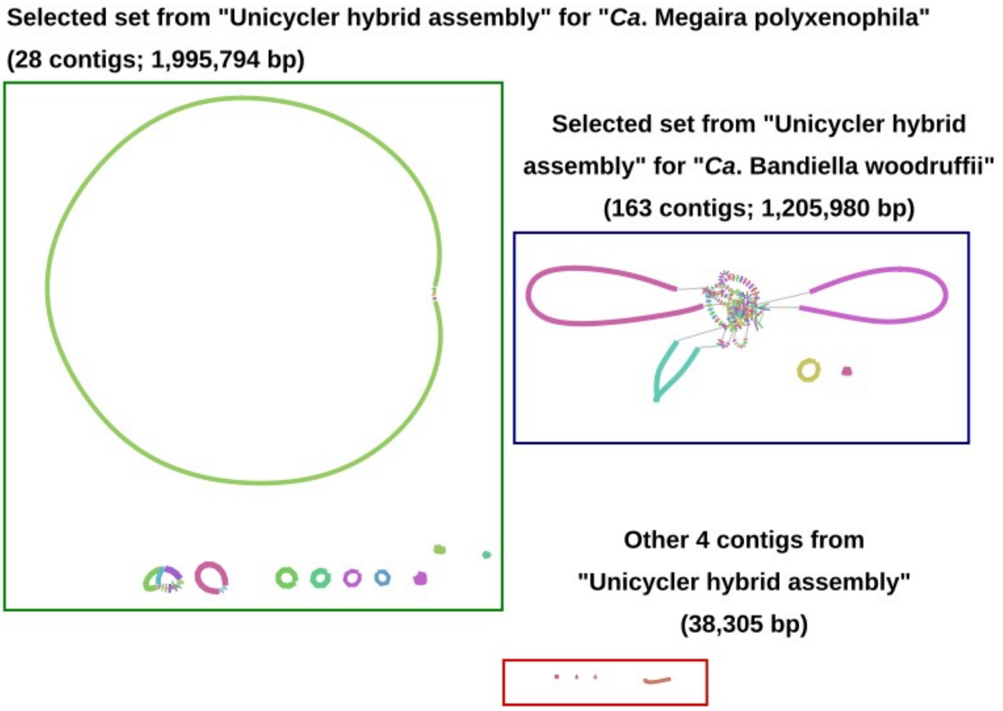

Supplementary Figure 7 Visual representation of the 195 contigs of the "Unicycler hybrid assembly" and the relative connections in the assembly graph, obtained with the Bandage software. The green square highlights the selected set of contigs for "*Ca*. Megaira polyxenophila", the blue square highlights the selected set of contigs for "*Ca*. Bandiella woodruffii", and red square highlights the remaining contigs of the assembly.

For what concerns the putative "*Ca*. Megaira polyxenophila" and "*Ca*. Bandiella woodruffii" chromosomes, a further manual step was performed in order to improve the assembly. Namely, all selected Nanopore reads were employed as queries in a blastn search (default megablast options) against the contig ends (1000 bp, only for contigs larger than 1000 bp), and those with hits were manually inspected, in order to identify potentially bridging reads between contigs. Accordingly, it was possible to close each genome into a single circular contig ("*Ca*. Megaira polyxenophila": 1,508,753 bp; "*Ca*. Bandiella woodruffii": 1,129,747 bp). Moreover, the assembly of plasmids was further refined by removing all contigs ≤1000bp containing no validated ORF (i.e. with no hits on NCBI nr or with hits only on hypothetical proteins or transposases). Thus, the two "final assemblies", including chromosome and plasmids, counted respectively 14 contigs (1,994,314 bp) for "*Ca*. Megaira polyxenophila", and 3 contigs (1,225,299 bp) for "*Ca*. Bandiella woodruffii" (see Supplementary Data 1 for details on the length of each plasmid).

***Euplotes harpa* BOD18 hosting "*Ca*. Cyrtobacter comes"**

The "preliminary assembly" was constituted by 135,782 contigs (99,489,754 bp; N50 = 1,566 bp; L50 = 16270)

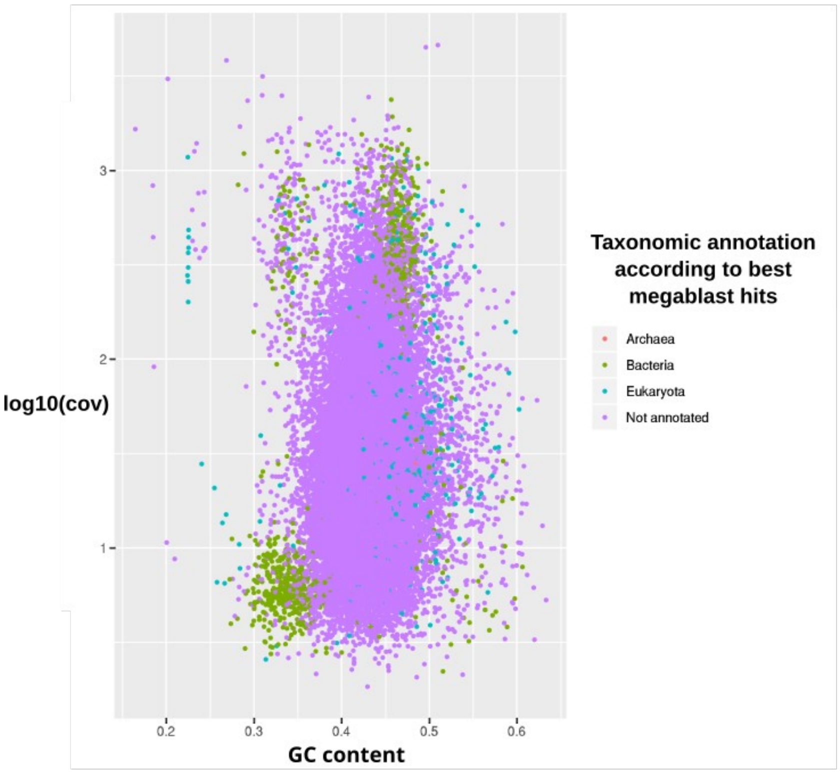

Supplementary Figure 8 Plot of the preliminary contigs of the sample BOD18 according to their GC content and log_{10} of sequencing coverage, coloured according to the respective best megablast hit. Only contigs with length higher than or equal to 1000 bp are shown for viewers' clarity.

In total, 15 full-length or partial rRNA genes were identified in the "preliminary assembly" (Supplementary Figure 8; Supplementary Data 4):

- 2 complete rRNA genes had best megablast hits on nuclear *Euplotes* sequences, and were thus assigned to the host. The respective contigs displayed 50.00 and 61.05 sequencing coverage
- one 16 rRNA gene sequence was identified as belonging to "*Ca*. Cyrtobacter comes", based on its best megablast hit on this organism with 100% identity. The respective contig displayed 668.69 sequencing coverage, and 0.36 GC content
- one complete and one partial rRNA gene sequences were identified based on their megablast hits as belonging to the *Polynucleobacter necessarius* (*Betaproteobacteria*) symbiont previously characterised in the BOD18 strain (Vannini et al. 2010). The respective contigs

had relatively comparable coverage (330.76 and 557.69) to the “*Ca*. Cyrtobacter” one, but significantly higher GC content (0.48 and 0.52).

- 10 remaining complete or partial rRNA gene sequences had megablast hits on other organisms (9) or no hit (1). The respective contigs had a clearly lower coverage (<37.57) than the one assigned to “*Ca*. Cyrtobacter”

Taking into account the plot and the rRNA gene features, we designed a “selection from preliminary assembly” targeting “*Ca*. Cyrtobacter”, including all contigs with $\log_{10}$ of coverage higher then 1.5. The reads mapping to this “selection from preliminary assembly” were extracted and reassembled separately with SPAdes (based on different attempts, the option -k 21,33,55,77,99 was chosen). This “SPAdes re-assembly” included 22442 contigs (36,602,319 bp, N50=3,630 bp; L50=2747). In this assembly, a selection was made of sequences belonging to “*Ca*. Cyrtobacter” (689 contigs, 1,524,096 bp; Supplementary Figure 9).

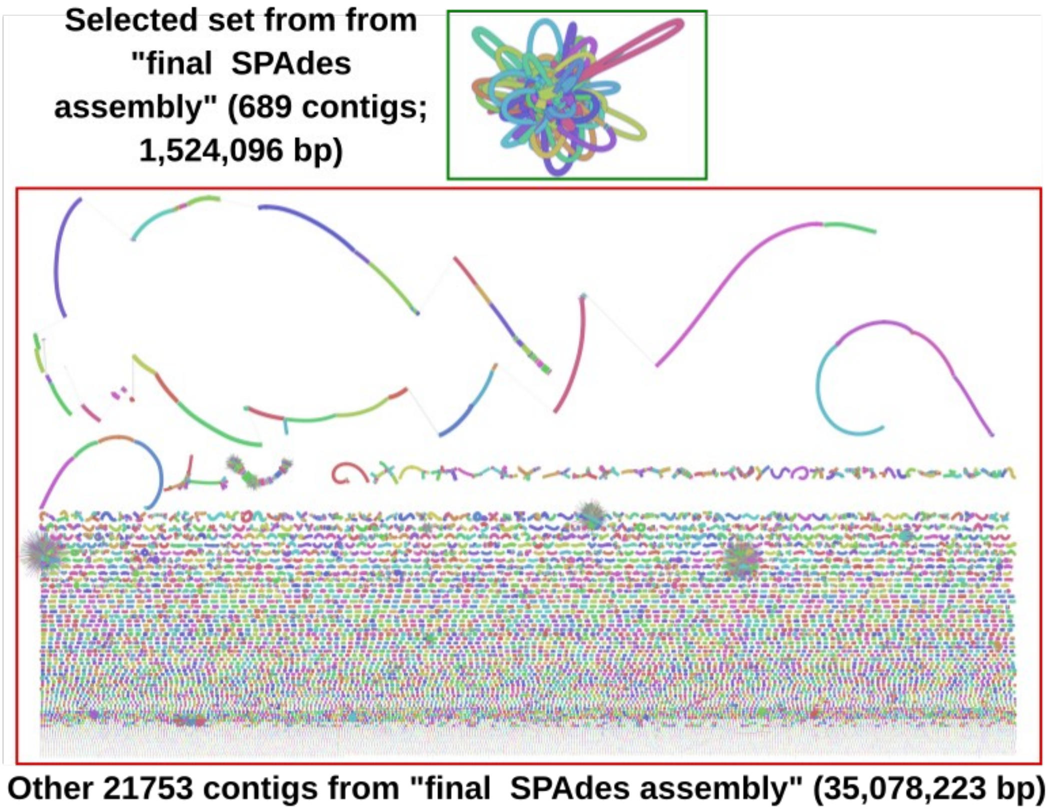

Supplementary Figure 9 Visual representation of the 22442 contigs of the “final SPAdes assembly” and the relative connections in the assembly graph, obtained with the Bandage software. The green square highlights the selected set of contigs, the red square highlights the remaining contigs of the assembly.

The relatively high fragmentation degree and the presence of several small contigs in “final SPAdes assembly” were interpreted as due to the presence of many repeated elements in the genome of “*Ca*. Cyrtobacter”, considering the high level of connection between the contigs (Supplementary Figure 9) and the presence of multiple transposase genes (data not shown). Accordingly, and accounting both for the careful manual revision applied and for the comparative BUSCO results with other *Rickettsiales* (Supplementary Data 2):, we concluded that this assembly likely contains the whole genome sequence (in terms of gene sequences) of “*Ca*. Cyrtobacter comes”.

Finally, the assembly was refined by removing all contigs <2000bp containing no validated ORF (i.e. with no hits on NCBI nr or with hits only on hypothetical proteins or transposases). Thus, the “final assembly” of the “*Ca*. Cyrtobacter comes” counted 141 contigs (1,308,703 bp; N50=14,445 bp; L50=28).

***Paramecium biaurelia* US_Bl 11III1 hosting “*Ca*. Fokinia cryptica”**

The “preliminary assembly” was constituted by 115,057 contigs (136,752,753 bp; N50 = 10,679 bp; L50 = 2209)

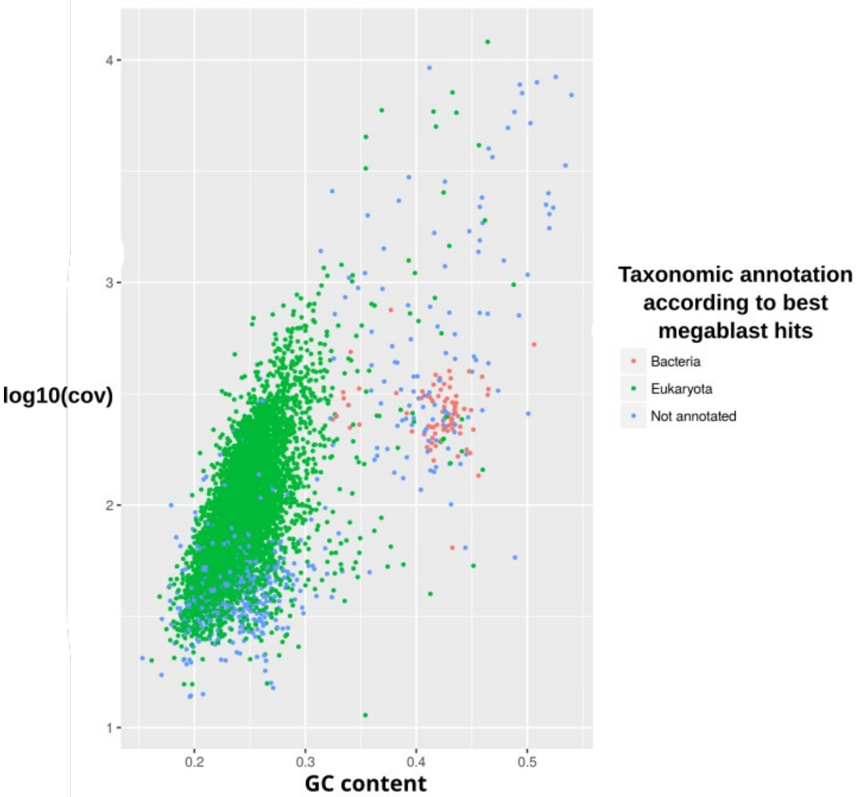

Supplementary Figure 10 Plot of the preliminary contigs of the sample US_Bl 11III1 according to their GC content and $\log_{10}$ of sequencing coverage, coloured according to the respective best megablast hit. Only contigs with length higher than or equal to 1000 bp are shown for viewers' clarity.

In total, 31 full-length or partial rRNA genes were identified in the "preliminary assembly" (Supplementary Figure 10; Supplementary Data 4):

- 25 complete or partial rRNA genes had best megablast hits on nuclear or mitochondrial *Paramecium* spp., and were thus assigned to the host (in one case, the partial gene sequence had no hit and the assignment was based on the megablast hit of the whole respective contig). The respective contigs ranged from 6.34 to 5944.93 in sequencing coverage
- 3 rRNA gene sequences (16S, 23S, 5S) were identified as belonging to "*Ca*. Fokinia cryptica", based on their best megablast hits on "*Ca*. Fokinia spp.". The two respective contigs displayed 281.37 and 301.99 sequencing coverage, and ~0.34 GC content
- 3 rRNA genes (5S, 16S, 23S) formed a putative operon on a single contig. Based on megablast hits, they were identified as belonging to the "*Ca*. Bealeia paramacronuclearis"

(*Holosporales*) symbiont previously characterised in the US_Bl 11III1 strain [21]. The contig had comparable coverage (312.91) to the "*Ca*. Fokinia" ones, but significantly higher GC content (0.46).

Taking into account the plot and the rRNA gene features, we designed a "selection from preliminary assembly" for "*Ca.* Fokinia cryptica" , including all contigs with $\log_{10}$ of coverage higher than 1.6, and GC content higher than 0.3, with the exception of those having best megablast hit on members of order Peniculida (to with the *Paramecium* host belongs).

The reads mapping to this "selection from preliminary assembly" were extracted and reassembled separately with SPAdes (based on different attempts, the option -k 21,33,55,77,99,127 was chosen). This "SPAdes re-assembly" included 840 contigs (2,952,405 bp, N50=23,802 bp; L50=25). In this assembly, a selection was made of sequences belonging to "*Ca.* Fokinia cryptica" (15 contigs, 812,007 bp; Supplementary Figure 11).

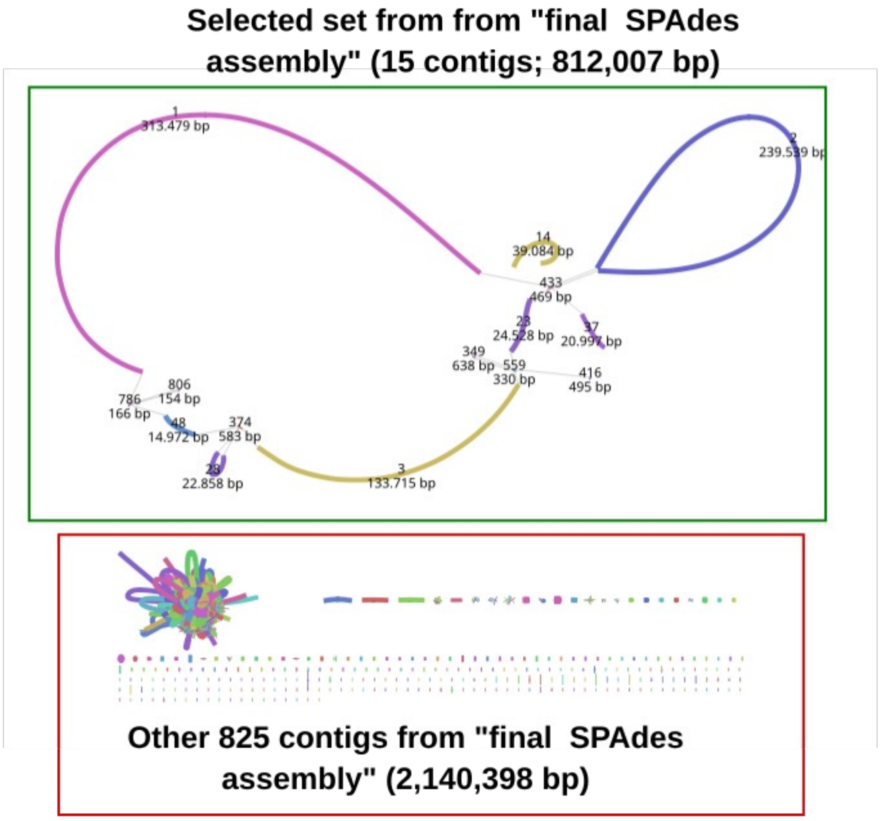

Supplementary Figure 11 Visual representation of the 7695 contigs of the "final SPAdes assembly" and the relative connections in the assembly graph, obtained with the Bandage software. The green square highlights the selected set of contigs, the red square highlights the remaining contigs of the assembly.

Then, primers were designed next to the ends of the 8 larger contigs in the selected set, and PCR reactions were performed in order to identify/confirm junctions among those contigs. The combinations of primers used for amplification and sequencing, primer sequences, and the reaction protocols used are reported below (Supplementary Table 5, 6, 7, 8).

Supplementary Table 5: primer combinations for closing "*Ca*. Fokinia cryptica" genome

Reaction	Amplification primer 1	Amplification primer 2	Sequencing primer 1	Sequencing primer 2	Protocol
1	Fcry_1F	Fcry_48R_a	Fcry_1F	Fcry_48R_b	B
2	Fcry_48F_a	Fcry_28F_a	Fcry_48F_b	Fcry_28F_b	A
3	Fcry_28R_a	Fcry_3F_a	Fcry_28R_b	Fcry_3F_b	B
4	Fcry_3R	Fcry_23F	Fcry_3R	Fcry_23F	A
5	Fcry_23R_a	Fcry_14R_a	Fcry_23R_b	Fcry_14R_b	A
6	Fcry_37F_a	Fcry_2F_a	Fcry_37F_b	Fcry_2F_b	B
7	Fcry_2R_a	Fcry_1R	Fcry_2R_b	Fcry_1R	B

Supplementary Table 6: primer sequences for closing "*Ca*. Fokinia cryptica" genome

Primer	Sequence (F: forward; R: reverse)
Fcry_1F	5'-GACACATCAACCGGAACG-3'
Fcry_48R_a	5'-GTTATACATCAGAGAGAAGCAC-3'
Fcry_48R_b	5'-TGATATGTTACGAGAGTGTGC-3'
Fcry_48F_a	5'-TCTTTAGACTACCACAGAGTG-3'
Fcry_48F_b	5'-AGCAGTTTGCTCAGCCTC-3'
Fcry_28F_a	5'-GGTATCTTACGGGTCAAGC-3'
Fcry_28F_b	5'-ATTGTGAAGCCGTAGTTGTAG-3'
Fcry_28R_a	5'-ACATTATGATACGCTCAACGC-3'
Fcry_28R_b	5'-TGGAGCATACGGAGTGAG-3'
Fcry_3F_a	5'-AGATGTGCCAAGGATTCGC-3'
Fcry_3F_b	5'-CCATTTCCGCCTTATTACCC-3'
Fcry_3R_b	5'-TTATGACGAACGATACAACTAG-3'
Fcry_23F	5'-CGATGTACGTTGTTCTCCC-3'
Fcry_23R_a	5'-GAAATGGGAAGGCAGTAGC-3'

Fcry_23R_b	5'-AGAAGTACTAGGAGCCCAC-3'
Fcry_14R_a	5'-CATGAAAGACGTGTAACGGC-3'
Fcry_14R_b	5'-GTTACTATAATGCCGCGAGG-3'
Fcry_14F_a	5'-ATGTTAAGAGCAGCATCGTAC-3'
Fcry_14F_b	5'-AAGAGGAACAAGAGACGAAC-3'
Fcry_37R_a	5'-GTAGATGTATGGTGGCTTGC-3'
Fcry_37R_b	5'-TTGGAGCAGTGAAGGGATG-3'
Fcry_37F_a	5'-AACATCGCTATTGCCGGTG-3'
Fcry_37F_b	5'-GAGTTACCACAATACCGATG-3'
Fcry_2F_a	5'-AGGTGGTGACTCCTGTTG-3'
Fcry_2F_b	5'-CTAAATAGCAGACGCGGTAG-3'
Fcry_2R_a	5'-TCGCATAACACTGCATTACC-3'
Fcry_2R_b	5'-ATCAAGTAGTGAAGCATCAGG-3'
Fcry_1R	5'-CACAACAGAAGGAATCGCG-3'

Supplementary Table 7: PCR protocol A (standard) for closing "*Ca*. Fokinia cryptica" genome

94°C	3 min	
94°C<br>50°C<br>72°C	30 s<br>30 s<br>4 min	40 cycles
72°C	10 min	

Supplementary Table 8: PCR protocol B (touchdown) for closing "*Ca*. Fokinia cryptica" genome

94°C	3 min	
94°C<br>56°C<br>72°C	30 s<br>30 s<br>4 min	5 cycles
94°C<br>53°C<br>72°C	30 s<br>30 s<br>4 min	10 cycles
94°C<br>50°C<br>72°C	30 s<br>30 s<br>4 min	30 cycles
72°C	10 min	

Thanks to such PCR experiments, it was possible to close the "*Ca*. Fokinia cryptica" genome into a single scaffold (815,586 bp).

Paramecium biaurelia* USBL-36I1 hosting *Lyticum linuosum

The "preliminary assembly" was constituted by 115,057 contigs (136,752,753 bp; N50 = 10,679 bp; L50 = 2209)

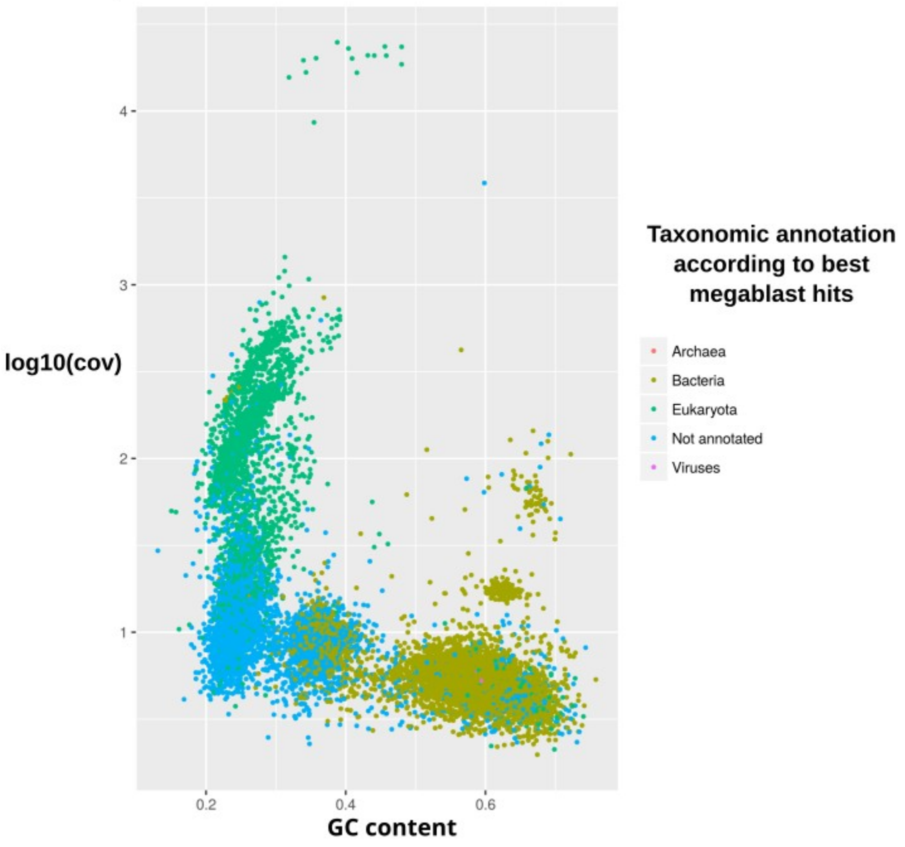

Supplementary Figure 12 Plot of the preliminary contigs of the sample USBL-36I1 according to their GC content and log_{10} of sequencing coverage, coloured according to the respective best megablast hit. Only contigs with length higher than or equal to 1000 bp are shown for viewers' clarity.

In total, 52 full-length or partial rRNA genes were identified in the "preliminary assembly" (Supplementary Figure 12; Supplementary Data 4):

- 27 complete or partial rRNA genes had best megablast hits on nuclear or mitochondrial *Paramecium* spp., and were thus assigned to the host. The respective contigs ranged from 71.83 to 20140.69 in sequencing coverage
- 2 rRNA gene sequences (16S, 23S) were identified as belonging to *Lyticum*, based on their best megablast hits on *L. sinuosum* (16S), and on the "*Ca*. Midichloriaceae" endosymbiont of *Acanthamoeba* sp. UWC8 (23S). The respective contigs displayed 258.45 and 843.75 sequencing coverage, and 0.37 and 0.25 GC content
- 23 remaining complete or partial rRNA gene sequences had megablast hits on other organisms (22) or no hit (1). The respective contigs had comparably lower coverage and/or clearly higher GC content (≥0.42) than those with *Lyticum* genes

Taking into account the plot and the rRNA gene features, we designed a "selection from preliminary assembly" for *Lyticum,* including all those contigs with $\log_{10}$ of coverage between 2 and 3, and GC content lower than 0.4.

The reads mapping to this "selection from preliminary assembly" were extracted and reassembled separately with SPAdes (based on different attempts, the option -k 21,33,55,77,99 was chosen). This "SPAdes re-assembly" included 7695 scaffolds (59,286,337 bp, N50=41,667 bp; L50=396). In this assembly, a selection was made of sequences belonging to *Lyticum* (30 scaffolds, 1,038,633 bp; Supplementary Figure 13).

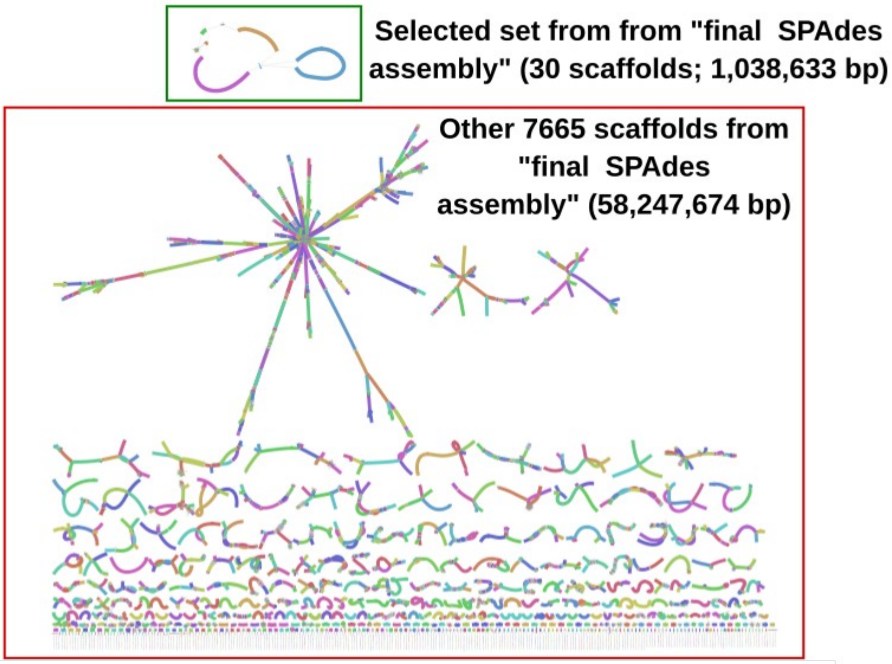

Supplementary Figure 13 Visual representation of the 7695 scaffolds of the “final SPAdes assembly” and the relative connections in the assembly graph, obtained with the Bandage software. The green square highlights the selected set of scaffolds, the red square highlights the remaining scaffolds of the assembly.

Finally, the assembly was refined by removing all scaffolds <5000 bp containing no validated ORF (i.e. with no hits on NCBI nr or with hits only on hypothetical proteins). Thus, the “final assembly” of *Lyticum* counted 6 scaffolds (1,018,517 bp; N50=336,044 bp; L50=2).

Plagiopyla frontata IBS-3 hosting the novel “_Ca_. Midichloriaceae” bacterium “_Ca_. Vederia obscura”

The “preliminary assembly” counted 49,376 contigs (66,833,832 bp; N50 = 33,240 bp; L50 = 443).

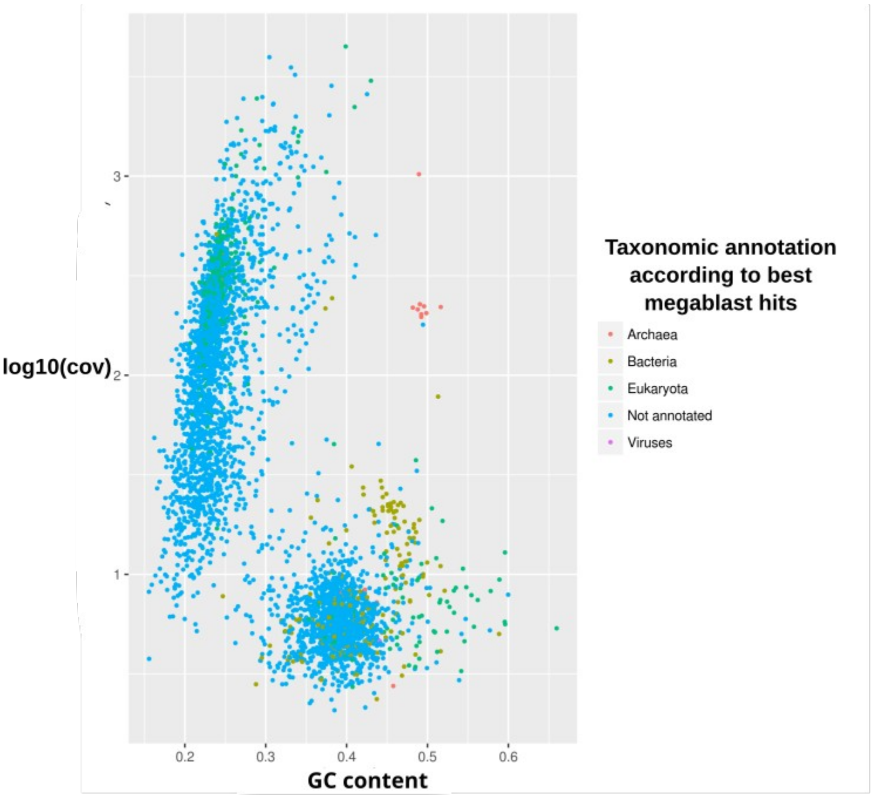

Supplementary Figure 14 Plot of the preliminary contigs of the sample IBS-3 according to their GC content and log_{10} of sequencing coverage, coloured according to the respective best megablast hit. Only contigs with length higher than or equal to 1000 bp are shown for viewers' clarity.

In total, 21 full-length or partial rRNA genes were identified in the "preliminary assembly" (Supplementary Figure 14; Supplementary Data 4):

- 1 partial LSU rRNA gene had best megablast hit on the ciliate *Cryptocaryon irritans*. This sequence was assigned to the host, considering the absence of LSU rRNA genes of *Plagiopyla* spp. on NCBI nucleotide. The respective contig had 3014.34 sequencing coverage
- 3 rRNA genes (5S, 16S, 23S) formed a putative operon on a single contig. They had best hits on *Methanocorpospulum* spp., and were thus assigned to a putative archaeal symbiont of *Plagiopyla*. The contig displayed 1022.82 sequencing coverage and 0.49 GC content
- 2 rRNA genes (16S, 23S) were identified as belonging to a novel "*Ca*. Midichloriaceae" bacterium from a new genus, based on their best megablast hits (with <90% identity) on the

"*Ca*. Midichloriaceae" endosymbiont of *Acanthamoeba* sp. UWC8. The respective contigs displayed 243.72 and 216.54 sequencing coverage, and 0.38 and 0.37 GC content

- 15 remaining complete or partial rRNA gene sequences had megablast hits on other organisms. The respective contigs had comparably lower coverage (≤78.10) and higher GC (>0.40) than the ones assigned to the "*Ca*. Midichloriaceae" bacterium

Taking into account the plot and the rRNA gene features, we designed a "selection from preliminary assembly" for this "*Ca.* Midichloriaceae" bacterium (from now on, "*Ca.* Vederia obscura"), including all contigs with $\log_{10}$ of coverage higher than 1.5, excluding those having best megablast hits on ciliate (Ciliophora) sequences.

The reads mapping to "selection from preliminary assembly" were extracted and reassembled separately with SPAdes (based on different attempts, the option -k 21,33,55,77,99 was chosen). This "SPAdes re-assembly" included 3875 contigs (39,068,512 bp, N50=55,917 bp; L50=188). In this assembly, a selection was made of contigs belonging to *"Ca.* Vederia obscura" (35 contigs, 894,612 bp; Supplementary Figure 15).

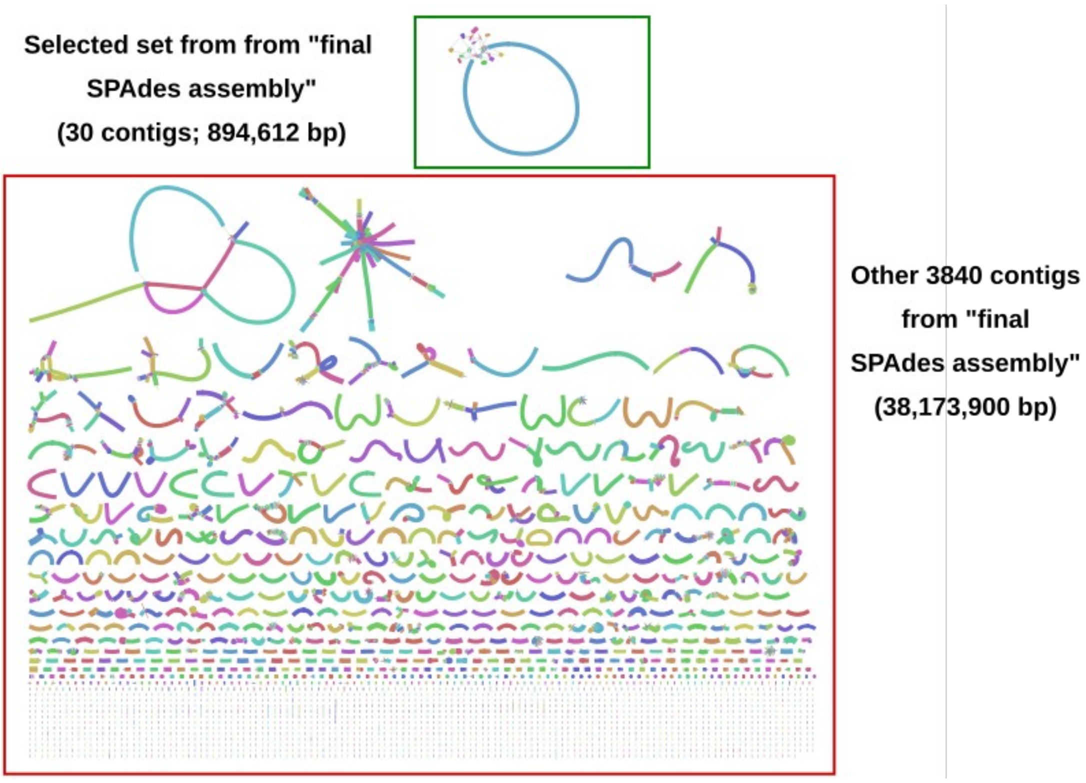

Supplementary Figure 15 Visual representation of the 3875 contigs of the "final SPAdes assembly" and the relative connections in the assembly graph, obtained with the Bandage software. The green square highlights the selected set of contigs, the red square highlights the remaining contigs of the assembly.

Finally, the assembly was refined by removing all contigs <5000 bp containing no validated ORF (i.e. with no hits on NCBI nr or with hits only on hypothetical proteins. Thus, the "final assembly" of "*Ca*. Vederia obscura" counted 19 contigs (886,222 bp; N50=785,721 bp; L50=1).

Reticulomyxa filosa hosting a novel "*Ca*. Midichloriaceae" bacterium

The "preliminary assembly" counted 25064 contigs (85,838,763 bp; N50 = 5,657 bp; L50 = 4263).

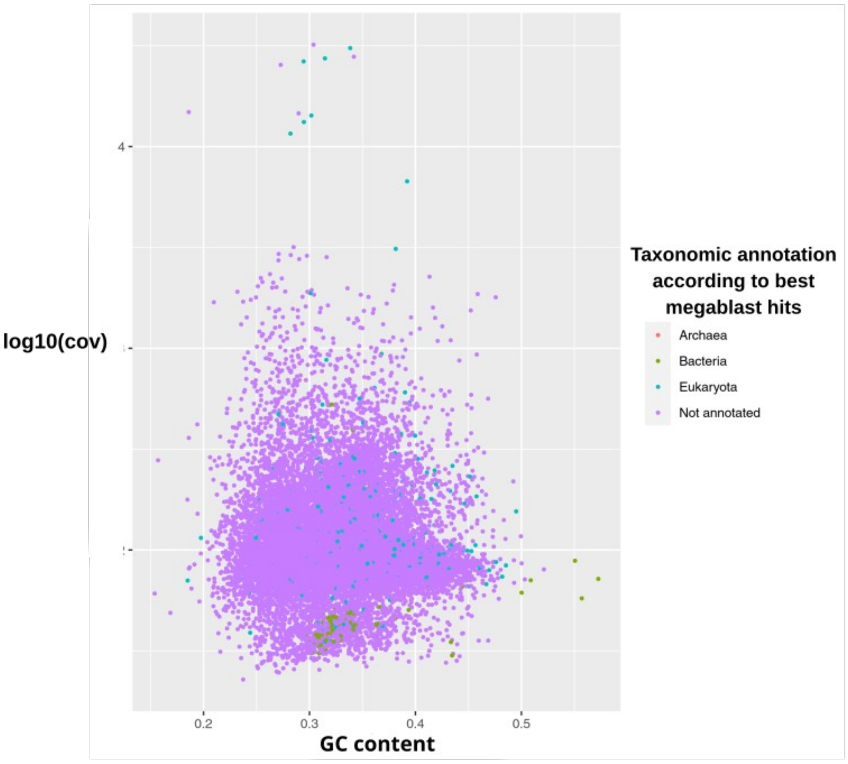

Supplementary Figure 16 Plot of the preliminary contigs of the sample *Reticulomyxa filosa* according to their GC content and $\log_{10}$ of sequencing coverage, coloured according to the respective best megablast hit. Only contigs with length higher than or equal to 1000 bp are shown for viewers' clarity.

In total, 11 full-length or partial rRNA genes were identified in the "preliminary assembly" (Supplementary Figure 16; Supplementary Data 4):

- 3 rRNA genes (16S, 23S, 5S) were identified as belonging to a novel "*Ca*. Midichloriaceae" bacterium from a new genus, based on their best megablast hits (with <94% identity) on the "*Ca*. Midichloriaceae" endosymbiont of *Acanthamoeba* sp. UWC8 and on "*Candidatus* Fokinia solitaria". The respective contigs displayed 89.09 and 101.35 sequencing coverage, and 0.26 and 0.34 GC content
- 8 remaining complete or partial rRNA gene sequences had megablast hits on other organisms (7) or no hit (1). The respective contigs had comparable coverage (78.23-133.15) and GC (0.23-0.31) with the ones assigned to the "*Ca*. Midichloriaceae" bacterium

Taking into account the plot and the rRNA gene features, we designed a "selection from preliminary assembly" for this "*Ca.* Midichloriaceae" bacterium, including all contigs with $\log_{10}$ of coverage lower than 1.5. Moreover, the careful inspection of the DIAMOND hits of contigs with higher coverage revealed the presence of 61 additional contigs putatively belonging to the "*Ca.* Midichloriaceae" bacterium, which were thus individually added to the "selection from preliminary assembly".

The reads mapping to such "selection from preliminary assembly" were extracted and reassembled separately with SPAdes (based on different attempts, the option -k 21,33,55,63 was chosen). This "SPAdes re-assembly" included 14367 contigs (33,864,241 bp, N50=6,105 bp; L50=1629). In this assembly, a selection was made of contigs belonging to the *"Ca. Midichloriaceae"* bacterium (498 contigs, 1,378,145 bp; Supplementary Figure 17).

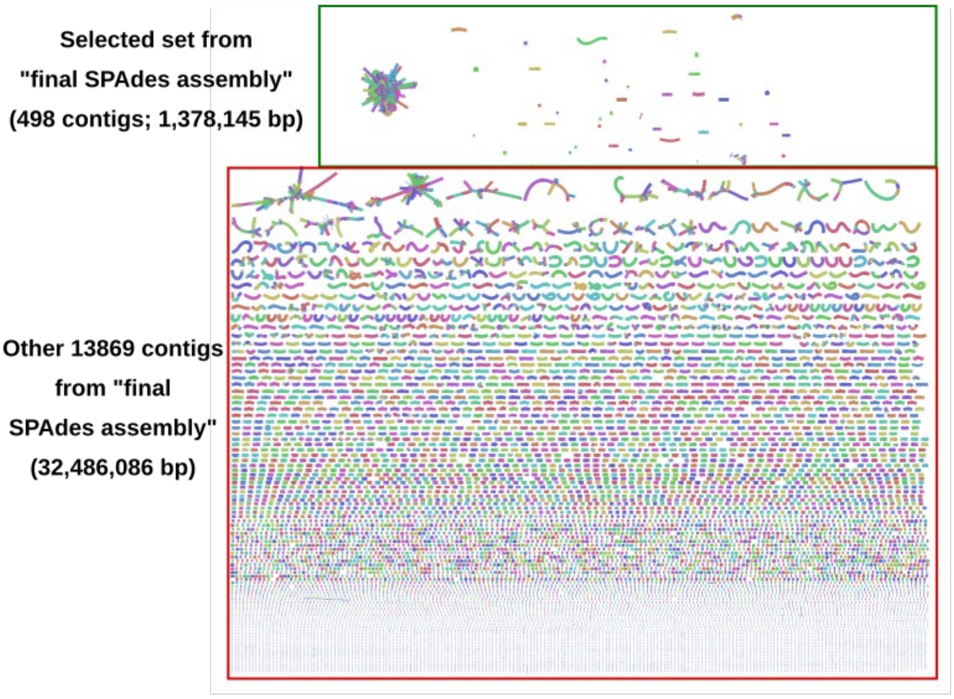

Supplementary Figure 17 Visual representation of the 14367 contigs of the "final SPAdes assembly" and the relative connections in the assembly graph, obtained with the Bandage software. The green square highlights the selected set of contigs, the red square highlights the remaining contigs of the assembly.

Finally, the assembly was refined by removing all contigs <1000 bp containing no validated ORF (i.e. with no hits on NCBI nr or with hits only on hypothetical proteins or transposases) Thus, the "final assembly" of the "*Ca*. Midichloriaceae bacterium" counted 242 contigs (1,305,893 bp; N50=7,721 bp; L50=53).

Supplementary Note 4: Phylogenomics methods

Phylogenomic analyses were aimed to get a comprehensive view on the evolution and diversity of *Rickettsiales*. To do so, we collected a representative dataset of *Rickettsiales*, including at least one representative per genus. We considered all available genomes and MAGs (metagenome-assembled genomes) belonging to "core" described *Rickettsiales* lineages as of February 2021 (i.e. the four families *Rickettsiaceae*, *Anaplasmataceae*, "*Ca*. Midichloriaceae", "*Ca*. Deianiraeaceae"), and looked also for additional *Rickettsiales*-related organisms, namely any lineage forming a supported monophyletic group with *Rickettsiales* with the exclusion of other (alpha)proteobacterial orders. GenBank assemblies for the phylogenomics were downloaded from NCBI ftp (ftp.ncbi.nlm.nih.gov/genomes/all/GCA) initially in February 2021, and later updated in July 2021 (see below for details).

To reach such objective, the final species dataset for the phylogenomic analyses was built with a progressive refining procedure, described in detail below. The main steps were the following:

1. Collection of the initial dataset including *Rickettsiales* genomes, putative *Rickettsiales* MAGs, other *Alphaproteobacteria* and outgroup organisms (February 2021)
2. Expansion of the set of putative *Rickettsiales* MAGs, and 1st filtering by assembly quality
3. Selection of orthologs for phylogenomics from *Rickettsiales* genomes, *Alphaproteobacteria*, and outgroup
4. Phylogeny-based identification of main MAG clades, and 2nd filtering by phylogenetic relatedness
5. Phylogeny-based tests for the affiliation of each MAG clade to *Rickettsiales*
6. Creation of the final dataset composed by *Rickettsiales* genomes, confirmed *Rickettsiales* MAGs, other *Alphaproteobacteria,* and outgroup organisms, including newly available assemblies (July 2021)

7. Phylogenomic analyses on the final dataset to reconstruct the inner relationships among *Rickettsiales*

Step 1: Collection of the initial dataset including *Rickettsiales* genomes, putative *Rickettsiales* MAGs, other *Alphaproteobacteria* and outgroup organisms (February 2021)

The sequences of other 89 representative non-*Rickettsiales Alphaproteobacteria* (including *Magnetococcia sensu* Parks et al. 2018 [41]) as well as 8 *Gammaproteobacteria* and *Betaproteobacteria* as outgroup were downloaded, taking inspiration from the selection by Muñoz-Gómez and co-authors [42].

All assemblies assigned by NCBI taxid to *Rickettsiales* (as of February 2021) were downloaded in the latest version, excluding anomalous assemblies. This set was manually edited to remove "*Ca*. Hepatobacter penaei" (GCA_000742475.1), actually belonging to *Holosporales*, and conversely to add the endosymbiont of *Acanthamoeba* UWC8 [43], which, though labelled as member of *Holosporales*, is actually a close relative of the "*Ca*. Midichloriaceae" bacterium "*Ca*. Jidaibacter acanthamoeba" UWC36 [44], thus ending up with 1679 assemblies. Out of those, 1164 were obtained in a large-scale study screening *Wolbachia* sequences from host genome sequencing projects [45]. We considered those *Wolbachia* assemblies as phylogenetically redundant for the divergence scale of the present phylogenomic dataset, and thus they were excluded from our analyses. The other assemblies were inspected in detail, and 401 of them, which were found to be derived from genome sequencing studies (in particular, though not exclusively, those labelled as described species or described genera), were treated as "*Rickettsiales* genome assemblies", while the other 114 were treated as "putative *Rickettsiales* MAGs". A manual selection was applied to the 401 "*Rickettsiales* genome assemblies", to obtain a set accounting for at least one representative for

each described genus, and in few cases (highly represented/diversified genera, e.g. *Rickettsia*, *Wolbachia*) more than one, resulting in 35 "selected *Rickettsiales* genomes".

Step 2: Expansion of the set of putative *Rickettsiales* MAGs and 1st filtering by assembly quality

The set of "putative *Rickettsiales* MAGs" was expanded in two ways. On one side, aiming to account for other deep-branching alphaproteobacterial MAGs (which might be potentially related to *Rickettsiales*), those identified in a previous study [46], i.e. "MarineAlpha", were downloaded. On the other side, in order to collect other potentially overlooked (e.g. due to mislabelling) lineages related to *Rickettsiales* or to "MarineAlpha", the respective taxonomy of all previously identified assemblies was collected from gtdb r95 [47], and all other additional MAGs belonging to the same lineages (as well as closely related lineages forming monophyletic clades in the gtdb reference tree) were downloaded as well and added up to the previously selected ones, ending up with 294 total "extended putative *Rickettsiales* MAGs". The quality of the extended MAG set was assessed with BUSCO 5.0.0 [48] with a search on 219 proteobacterial orthologs ("proteobacteria_odb10") (Supplementary Data 2). Based on a comparison with the "selected *Rickettsiales* genomes", MAGs were retained only if having ≥50% single-copy orthologs and <5% duplicated orthologs, thereby discarding potentially poor quality/contaminated assemblies, obtaining 219 "BUSCO-filtered putative *Rickettsiales* MAGs".

Step 3: Selection of orthologs for phylogenomics from *Rickettsiales* genomes, *Alphaproteobacteria*, and outgroup

For all "non-MAG genomes", whenever available, protein sequences were taken directly from the NCBI annotation included in the assembly. In all other cases, i.e. non-annotated genomes (including newly obtained ones) and MAGs, genome sequences were annotated with Prokka 1.10 [35].

For all assemblies (including *Rickettsiales* genomes downloaded from NCBI and newly sequenced ones, MAGs, *Alphaproteobacteria* and outgroup) the eggNOG v5 assignments [49] were then predicted on the annotated proteins with eggnog-mapper 2.0.6 [50] with a taxonomic scope on *Alphaproteobacteria* (options: --tax_scope 28211 --go_evidence non-electronic --target_orthologs all --seed_ortholog_evalue 0.001 --seed_ortholog_score 60 --query_cover 20 --subject_cover 50). Taking into account all assemblies except MAGs, proteins were grouped based on the assigned orthogroup, and each resulting orthogroup was aligned with MAFFT 7.475 L-INS-i [51] and trimmed with BMGE 1.12 [52] with BLOSUM30 matrix. Single gene phylogenies were then performed with FastTree 2.1 [53], and individually examined to remove paralogs and short/poorly aligned sequences. After this "polishing" step, orthogroups were kept only if present in single copy in at least 85% of the genomes of the "*Rickettsiales* genome set for phylogeny" (which, taking together "selected *Rickettsiales* genomes" and newly sequenced genomes, totals 44 organisms), in at least 85% of other *Alphaproteobacteria*, and in at least 50% of outgroup. Thus, by this procedure 179 orthogroups were selected, which were employed for all following phylogenetic analyses (Supplementary Data 5).

Step 4: Phylogeny-based identification of main MAG clades, and 2nd filtering of MAGs by phylogenetic relatedness

The genes assigned to the 179 selected orthogroups in the "BUSCO-filtered putative *Rickettsiales* MAGs" were added to those of the previous selection of organisms, each orthogroup was re-aligned and trimmed with MAFFT and BMGE, and single-gene phylogenies were inferred as described above. Each tree was manually inspected, for removing paralogs and short/poorly aligned sequences derived from the MAGs, ending up with the final sequence selection for those MAGs (Supplementary Data 5). By this procedures 8 MAG were found to harbour <10

alphaproteobacterial orthogroups. Via online blastp searches on NCBI nr, it was found that (a significant part of) their proteome had much higher identities with non-alphaproteobacterial sequences, and were thus deemed as taxonomically mislabelled and removed from successive analyses, leaving 211 "BUSCO+eggNOG-filtered putative *Rickettsiales* MAGs" (Supplementary Data 5).

Then, a first phylogenomic tree was inferred, with the aim to get a general view on the phylogenetic diversity of included MAGs, and on their relationships with *Rickettsiales* and other *Alphaproteobacteria*. Thus, the 179 orthogroup sequences of all the "BUSCO+eggNOG-filtered putative *Rickettsiales* MAGs" were used, together with a "Genome assembly set for MAG classification", namely the other *Alphaproteobacteria*, outgroup organisms and, to speed up phylogenetic computations, a small and phylogenetically-balanced selection of 13 *Rickettsiales* genome assemblies ("*Ca*. Deianiraea vastatrix" for "*Ca*. Deianiraeaceae" and 4 for each other family). Each orthogroup was *de novo* aligned with MAFFT, and trimmed with BMGE, as described above, and all orthogroups were concatenated using AMAS [54]. Then, in order to avoid artefacts due to compositional heterogeneity in the datasets (in particular potential "erroneous" phylogenetic proximity of MAG lineages to "core" *Rickettsiales* due GC/AT biases in the respective genomes), the approach by Muñoz-Gómez and co-authors [42] was applied, and the concatenated alignment was trimmed removing 10%, 20%, 30%, 40% or 50% of most heterogeneous sites. For each of these six alignments (the original and the five trimmed ones), maximum likelihood phylogenetic trees were inferred with IQ-TREE 1.6.12 [55] employing ModelFinder [56] for model selection, performing 1000 ultrafast bootstraps [57] and SH-aLRT with 1000 replicates.

In all the resulting trees, 35 MAGs were found consistently branching within "core" *Rickettsiales* with full support, and thus were directly treated as "confirmed *Rickettsiales* MAGs" (Supplementary Figure 29). Other 2 MAGs were found consistently branching with high support within other alphaproteobacterial lineages (respectively, *Holosporales* and *Rhodospirillales*), and

thus were considered as non-*Rickettsiales* and directly discarded. The other 174 MAGs were grouped into 13 groups (named A,B,C,D,E,F,G,H,I,J,K,L,M), based on monophyly observed across the obtained trees, with the aim to verify the position of each group with a dedicated phylogenomic dataset (see below, Step 5). Moreover, in order to speed up all the following analyses, phylogenetically-redundant MAGs (both within the 13 groups and within the already "confirmed *Rickettsiales* MAGs") were removed as follows. Clusters of MAGs were defined based on supported monophyly and reciprocal AAI (Average Amino acid Identity) >0.85 [58]; Supplementary Figure 30), and for each cluster only the single MAG with best BUSCO scores (in terms of higher single-copy orthologs, or, secondarily, lower duplicated orthologs) (Supplementary Data 2) was kept, thus resulting in 21 "confirmed *Rickettsiales* MAGs" and collectively 106 MAGs in the 13 groups.

Step 5: Phylogeny-based tests for the affiliation of each MAG clade to *Rickettsiales*

The MAGs belonging to each of the 13 previously identified groups were tested separately for the potential relatedness to "core" *Rickettsiales*, with a separate dataset. This was done in order to remove phylogenetic "noise" and potential artefacts caused by the contemporary addition of multiple deep-branching and fast-evolving lineages, and to get the "cleanest" phylogenetic position for the members of each group (such an approach has been previously utilized for similar problematic datasets, e.g. [59]). Accordingly, for each group, the 179 ortholog sequences of the respective organisms were *de novo* aligned with those of the "Genome assembly set for MAG classification" using MAFFT, trimmed with BMGE, and all ortholgs were concatenated with AMAS, as described above. Each concatenated alignment was trimmed removing 10%, 20%, 30%, 40% or 50% of most heterogeneous sites, as described above. For each resulting alignment,

maximum likelihood phylogeny was inferred with IQ-TREE and the LG+C60+F+R6 model, performing 1000 ultrafast bootstraps and SH-aLRT with 1000 replicates (Supplementary Figure 31). All the resulting trees were inspected, allowing the classification of 14 MAGs belonging to groups F, G, and H as additional, deep-branching "confirmed *Rickettsiales* MAGs", based on strongly supported monophyly with "core" *Rickettsiales* on the original concatenated alignment and on all the five alignments with removal of heterogeneous sites. On the other side, none of the MAGs from the other 10 groups was found as monophyletic with *Rickettsiales* with high support in the respective trees, and were thus deemed as non-*Rickettsiales*, and not considered for the following analyses.

Step 6: Creation of the final dataset composed by *Rickettsiales* genomes, confirmed *Rickettsiales* MAGs, other *Alphaproteobacteria,* and outgroup organisms, including newly available assemblies (July 2021)

Then, the assembly and MAG selection was updated according to the latest NCBI version (July 2021), employing the same selection criteria as described above. Namely, the newly published genome of "*Ca*. Echinorickettsia raffii" [60] was directly added to the "*Rickettsiales* genome set for phylogeny", while 100 "new putative *Rickettsiales* MAGs" were processed as described above and 95 of them passed the BUSCO-based filtering by assembly quality (Supplementary Data 2). In order to test the evolutionary relatedness of these novel MAGs to *Rickettsiales*, similarly to what described above, after assignment of their genes to the 179 orthogroups, the respective sequences were newly aligned together with those of the "Genome assembly set for MAG classification" and of the previously identified deep-branching *Rickettiales* lineages (now treated as ascertained *Rickettsiales*, similarly to the "core" lineages). Aligned genes were trimmed and concatenated, and the concatenated alignment was trimmed to remove more compositionally heterogeneous sites, as

described above. For each resulting concatenated alignment, maximum-likelihood phylogeny was inferred with IQ-TREE employing ModelFinder for model selection, as described above (Supplementary Figure 32). In all the resulting trees, 77 MAGs were found consistently branching within "core" or "deep-branching" *Rickettsiales* with full support, and thus were directly added to the previously identified "confirmed *Rickettsiales* MAGs". The other 18 MAGs were found consistently and with high support branching within other alphaproteobacterial lineages (respectively, 1 *Sphingomonadales*, 2 *Rhizobiales*, and 15 *Holosporales*), and thus were considered as non-*Rickettsiales* and discarded. No MAG fell outside previously identified lineages, so no additional phylogeny-based test was required.

In order to remove phylogenetically redundant genomes, among all "confirmed *Rickettsiales* MAGs", including those derived from the July 2021, updated clusters were again defined based on monophyly and reciprocal AAI >0.85 (Supplementary Figure 30) As described above, for each cluster just the MAG with the best BUSCO scores was kept (Supplementary Data 2).

Moreover, few MAGs were found to be closely related to some representatives "*Rickettsiales* genome set for phylogeny". Thus, based on the monophyly and AAI criteria described above, among those the additional "phylogenetically-redundant" MAGs were removed (Supplementary Figure 30).

Therefore, the final "*Rickettsiales* selection for phylogeny" comprised 45 genome assemblies and 68 MAGs, and, together with the 89 other representative *Alphaproteobacteria* and 8 outgroup organisms, formed the "final species selection for phylogeny" (210 total organisms).

Step 7: Phylogenomic analyses on the final dataset to reconstruct the inner relationships among *Rickettsiales*

The gene sequences of the 179 orthogroups for the 210 taxa of the final "*Rickettsiales* selection for phylogeny" were aligned, trimmed, and concatenated as described above. The phylogeny on this final dataset was aimed to reconstruct accurately the inner relationships among core and deep-branching *Rickettsiales* organisms, and at the same time, the relationship of *Rickettsiales* with other *Alphaproteobacteria*, building up the basis for the following gene-content analyses.

Accordingly, we inferred phylogenies on the full "untreated" alignment, as well as after removing 10%, 20%, 30%, 40% or 50% of most heterogeneous sites as described above, with IQ-TREE and the same options as above (LG+C60+F+R6 model, 1000 ultrafast bootstraps and SH-aLRT with 1000 replicates) (Fig. 1; Supplementary Figure 18).

Supplementary Note 5: gene content analyses

Annotation

The newly obtained genomes were all annotated with Prokka 1.10 [35], using the --rfam option. Afterwards, annotation of the genomes of ciliate symbionts was manually curated by a detailed inspection of blastp hits on NCBI nr and on *Rickettsiales* proteins as described previously [2].

Creation of a set of "homology groups" for gene content comparisons

In order to perform gene content comparisons and reconstruct its variation along the inferred species tree, a set of "homology groups" was obtained for the 210 total organisms in the final phylogeny, starting from the previously obtained eggNOGs (see Supplementary Note 4).

The eggNOG [49] database is organised by taxonomy, namely a dedicated set of eggNOG is identified for each lineage from the domain to the order/family level (domain, phylum, class, order, family), and eggNOGs are hierarchically linked. For example 2TSRZ@28211 (DNA sliding clamp in *Alphaproteobacteria* – NCBI taxid 28211) is linked to corresponding eggNOGs in higher taxonomic ranks, such as 1MVD9@1224 (same function in *Proteobacteria* – NCBI taxid 1224) and COG0592@2 (same function in *Bacteria* – NCBI taxid 2). Such hierarchical organisation allows to take advantage of lineage-specific annotations, such as labelling of lineage-specific paralogs, and at the same time gives the possibility to compare eggNOGs identified in different lineages.
In this work, we aimed to obtained "clustered" homology groups of homologous genes, to be inspected and analysed later (see below) for their evolutionary history across *Rickettsiales*, including potential duplication, loss and transfer events, leading to paralogies as well as xenologies,

For the dataset of this study, the raw eggNOG assignments were not directly suitable for the intended approach. First, we considered that any pre-computed orthologs, tailored on already described organisms and sublineages, are inherently not fully suitable to discriminate ortholog/paralog (or xenolog) on novel lineages such as the multiple ones herein treated.. Some eggNOG assignments may also be "impractical" under the desired perspective of homologous groups described above. Extreme observed examples are thaose of some ribosomal protein genes in "*Candidatus* Aquarickettsia rohweri" [61], namely S10 (RST68169.1), L3 (RST68170.1), L10 (RST68925.1), L14 (RST68179.1), L24 (RST68180.1), which have their best hits in eggNOGs assigned to lineages as distant as Metazoa (3C1KH@33208, 3BT1X@33208, 3C01C@33208), *Acyclobacillaceae* (*Firmicutes*) (278I9@186823), and *Sphingomonadales* (2K3XB@204457). While these results might be seen as suggestive of horizontal gene transfer, multiple other reasons are plausible, and it is not possible to discriminate among those a priori, For example, there could be a mis-assignment as a consequence of the high sequence evolution rates in *Rickettsiales*, which may result in extremely high sequence divergence with respect to any other organism (including other *Rickettsiales*), so that hits can be almost equivalently distant from closely related and unrelated lineages [2], even for housekeeping genes. Alternatively, taxonomically mislabelled reference sequences in the eggNOG database could mislead the assignment as well. Contamination of the *Rickettsiales* assemblies is in principle another possibility, which seems overall rather unlikely/rare, as we included in our dataset only closed high-quality genomes, or quality-filtered MAGs. Therefore, aiming at reconstructing the largest "meaningful" homology groups starting from eggNOG assignments, and at the same time retaining as much lineage specific information as possible from the eggNOG database, we designed the following two-step "telescopic" approach, taking advantage of the hierarchical structure of the eggNOG database:

- We started from the eggNOG taxonomic path leading to *Rickettsiales* (root; *Bacteria*; *Proteobacteria*; *Alphaproteobacteria*; *Rickettsiales*), and we considered the "eggNOG OGs"

matches for each gene. When a gene was assigned to an eggNOG belonging to a taxon outside such path, it was moved "upwards" to the respectively proximate higher rank. Such passage was applied recursively until a taxon along the *Rickettsiales* path was met. This allowed also the "connection" of eggNOGs in the non-*Rickettsiales* organisms of the dataset. For example, if a gene was assigned to the 2JQAW@204441 eggNOG (DNA clamp in *Rhodospirillales* – NCBI taxid 204441), it was labelled as the corresponding alphaproteobacterial eggNOG 2TSRZ@28211

- The second step was intended to "connect" the information between different eggNOGs, Namely, whenever appropriate,taxonomically-linked eggNOGs were joined at the respectively highest taxonomic rank. In detail, for each eggNOGs assigned at the "root" level after the previous step, all genes assigned to eggNOGs of lower taxonomic ranks (*Bacteria*; *Proteobacteria*; *Alphaproteobacteria*; *Rickettsiales*) that were linked by the eggNOG hierarchy to the given root eggNOG were moved "upwards" to such root eggNOG. For example, if a gene was assigned to the root DNA clamp COG0592@1, any other gene assigned to the linked 2TSRZ@28211 (*Alphaproteobacteria*) or 1MVD9@1224 (*Proteobacteria*) was moved "upwards" to the COG0592@1. This passage was performed progressively for remaining eggNOGs of lower ranks. It was thus possible to "merge" genes into the desired larger and meaningful "homology groups", while still keeping as much as possible lineage-specific refined annotation.

Applying such "telescopic" approach, a total of 444,226 genes were assigned to 20,041 "homology groups". Only 4009 of such "homology groups" were present in at least one member of *Rickettsiales* ("core" or "deep-branching"), and only 2990 of those were present in 4 or more organisms of the total dataset, and were thus considered for following gene content and phylogenetic analyses.

We thus carefully manually inspected the presence/absence and copy numbers of "homology groups" in the *Rickettsiales*, in comparison with the other *Alphaproteobacteria*, to get a comprehensive overview of their functional repertoire. This allowed us to get some preliminary evolutionary insight, and select traits and functions for further in-depth and more refined analyses, as described in the following sections.

Phylogenetic analyses on biosynthetic pathways for amino acids and nucleotides

We aimed at phylogenetic inferences for the genes involved biosynthetic pathways for amino acids and nucleotides. To this purpose, reference biosynthetic pathways were obtained from the Biocyc database [62]. In order to get more robust and reliable phylogenetic inferences, we aimed to concatenate together sequences of the proteins involved in the same pathway, thus obtaining more phylogenetically informative datasets. For the same purpose, pathways sharing common reactions (e.g. purines, pyrimidines, branched-chain amino acids, aromatic amino acids) were considered together (Supplementary Data 6). Under similar assumptions, single (or two) gene pathways, namely those for glycine, aspartate, asparagine, glutamate, glutamine, and alanine, were not considered phylogenetically informative enough, and were thus not employed in the analyses.

For each gene involved in a chosen pathway performed a blastp search with the selected Biocyc reference sequence as query against the protein sequences from all the 210 selected taxa as database, and manually inspecting the results, in order to selected the corresponding "homology groups". Then, the composition of each group was manually refined, by visual inspection of the respective alignment and of a maximum-likelihood tree obtained with IQ-TREE 1.6.12 [55] employing ModelFinder [56] for model selection, and performing 1000 ultrafast bootstraps [57] and SH-aLRT with 1000 replicates. In particular, short and/or poorly aligned sequences and distant

paralogs were manually removed, especially among non-*Rickettsiales* organisms, and, in some cases (e.g. biosynthetic genes for histidine) single domains from alternative fusions of multi-domain proteins were selected and analysed separately. NCBI conserved domain search [63] was also used to identify such distant paralogs, based on distinct domain annotation. Each manually refined orthogroup was then re-aligned with MAFFT 7.475 L-INS-i and trimmed with BMGE 1.12 with the BLOSUM30 matrix. Phylogeny on each single manually refined was performed as above (Supplementary Figure 25).

Then, based on inspection of such "final" single-gene phylogenies, suspected non-orthologs were removed, and orthogroups belonging to the same pathway (or same group of related pathways) were concatenated together using AMAS [54].

Then, in order to account for compositional heterogeneity (see Supplementary Note 4), each of the concatenated alignments was processed for removing 10%, 20%, 30%, 40% or 50% of the most compositionally heterogeneous sites, following the approach by Muñoz-Gómez and co-authors (2019). On each resulting alignment, phylogeny was inferred with IQ-TREE and the LG+C60+F+R6 model, performing 1000 ultrafast bootstraps and SH-aLRT with 1000 replicates (Supplementary Figure 26).

Identification of amino acid transporters

Amino acids transporters were identified from the TCDB database [64]. The transporter families in TCDB were queried by keywords such as "amino acid(s)" and the name of each of the twenty proteinogenic amino acids. The single database entries (i.e. proteins) belonging to each member of the retrieved families were individually examined, in particular information on the kind of substrate (if known) were collected, and those members with ascertained substrate(s) that did not include

amino acids were discarded, ending up with a list of trusted and substrate-labelled entries of putative amino acid transporters from the TCDB database. We then performed a blastp search with all the *Rickettsiales* proteins of our dataset as queries against the full TCDB. Then, for each *Rickettsiales* genome, only the proteins having a best significant hit (e-value threshold of 1e-5) on each trusted entry were selected, and counted (Supplementary Figure 28).

Identification and phylogenetic analysis of the tlc nucleotide translocasaes

Analyses on nucleotide transporters were focused on the tlc nucleotide translocase transporters, common in *Rickettsiales* and in other host-associated lineages [65]. Starting from the eggNOG classification, a blastp-based inspection using the protein sequences from the paper by Major and co-authors [65] as queries led to the identification of the corresponding homology group assigned to tlc translocases (COG3202@1), which allowed to count the number of genes present for each organism of our dataset. Then, the phylogenetic dataset of tlc translocases by Major and co-authors (2017) was downloaded. Considering the large size of this dataset, a subset was selected, corresponding to a clade of sequences consisting only of the nucleotide transport protein domain and belonging to multiple bacteria (including *Chlamydiae* and several *Proteobacteria* such as *Rickettsiales* and *Holosporales*), as well as to eukaryotes such as Microsporidia, Stramenopiles, and Viridiplantae. The selected sequences from the published dataset and those identified in our dataset (COG3202@1) were all aligned together with MAFFT 7.475 L-INS-i and trimmed with BMGE 1.12 with the BLOSUM30 matrix, and phylogeny was inferred as in [65], namely with IQ-TREE with the LG+C60 model, performing 1000 ultrafast bootstraps and SH-aLRT with 1000 replicates (Supplementary Figure 27).

Identification of genes involved in the interaction with host cells

For getting information on the presence and multiplicity of genes involved in multiple features of the interaction of *Rickettsiales* with host cells, such as secretion systems, putative toxins and other secreted effectors, flagella, adhesion and invasion molecules, the VFDB core reference database was employed [66] (downloaded on 6th May 2022). The VFDB is organised in order to have a separate focus on single pathogens, which implies a significant redundancy of entries of orthologous genes (e.g. flagellar genes) identified in each pathogen. This organisation would not be suitable for the analyses of our large dataset composed almost exclusively by non-model bacteria. Therefore, orthologs were identified within the database with the following approach. Sequences were divided by organism (i.e. single strain) and by VFC (VF Classes). Then, within each selected VFC ("Adherence", "Biofilm", "Effector delivery system", "Exotoxin", "Exoenzyme", "Invasion", "Motility") orthogroups were identified with OrthoFinder 2.5.4 [67], employing blastp as sequence search program. The composition of orthogroups produced by OrthoFinder was then manually inspected and curated. Then, all proteins of our dataset of *Rickettsiales* were employed as queries in a blastp search against the full VFDB core database. Similarly to the previous TCDB blastp search, the whole VFDB set of sequences was employed as database in order to minimise both false negatives and false positive hits. Then, for each *Rickettsiales* genome, proteins were "assigned" to each curated orthogroup as follows: a protein was counted if displaying a significant (evalue 1e-5) best hit on any sequence belonging to the orthogroup, and the total number of proteins having such hits were summed over each orthogroup (Supplementary Figure 20, 21, 22, 23).

Supplementary Figure 18: phylogenomic trees of the final dataset. These include the "untreated" and the "compositionally trimmed" alignments (namely, with the 10%, 20%, 30%, 40%, or 50% most compositionally heterogeneous sites removed). On each branch, support values by SH-aLRT with 1000 replicates and by 1000 ultrafast bootstraps are reported.

original alignment

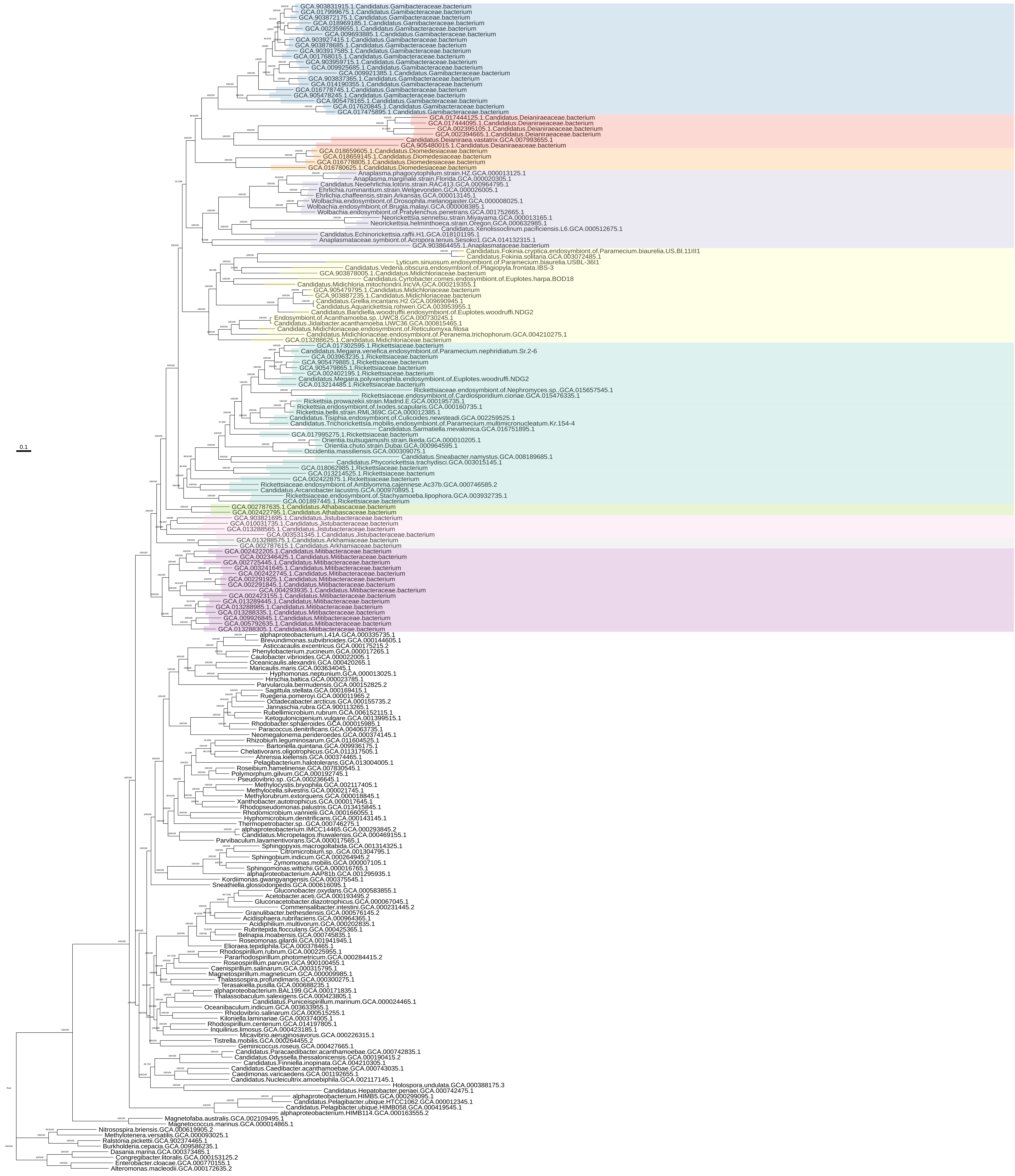

10% most compositionally heterogeneous sites removed

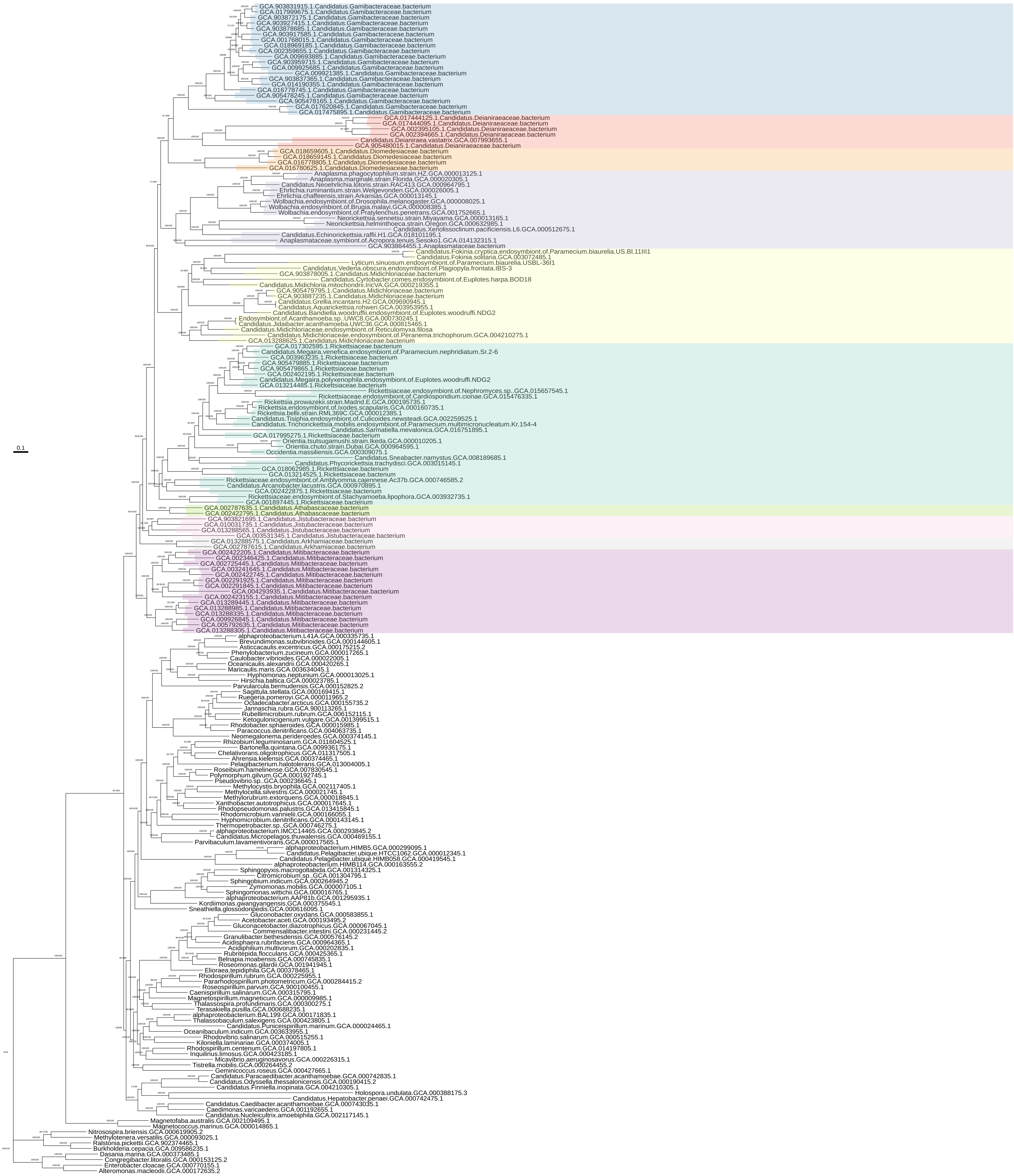

20% most compositionally heterogeneous sites removed

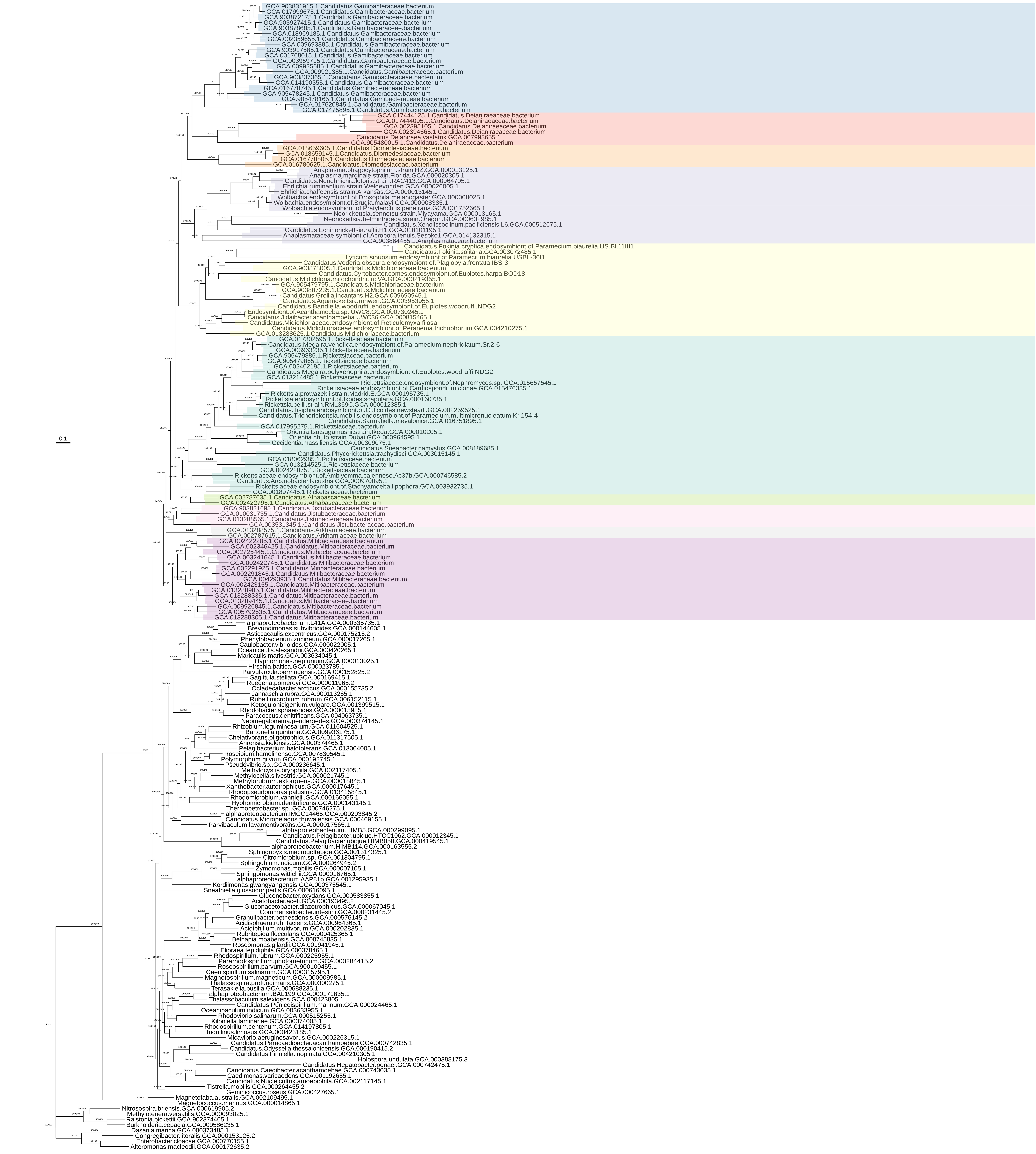

30% most compositionally heterogeneous sites removed

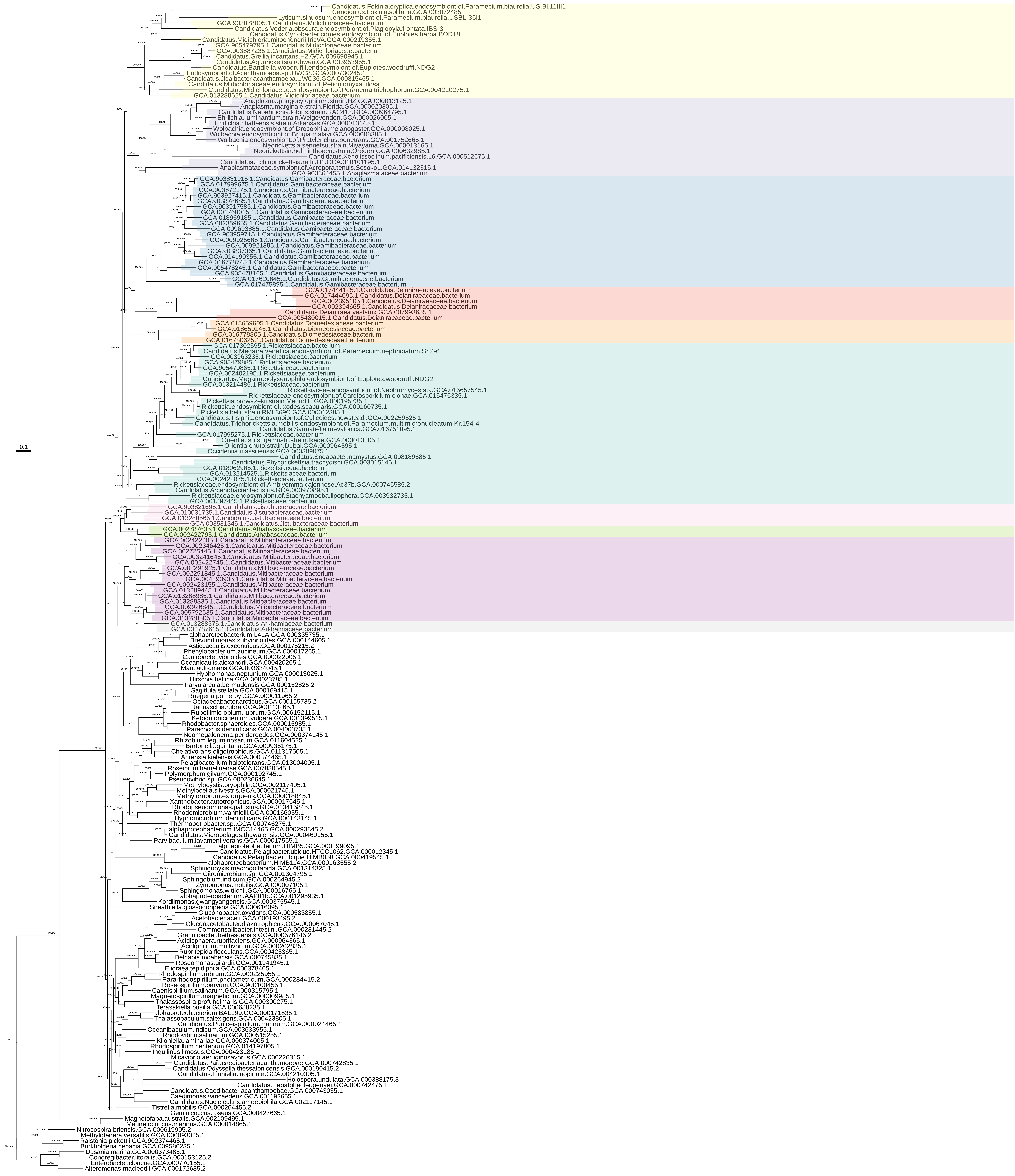

40% most compositionally heterogeneous sites removed

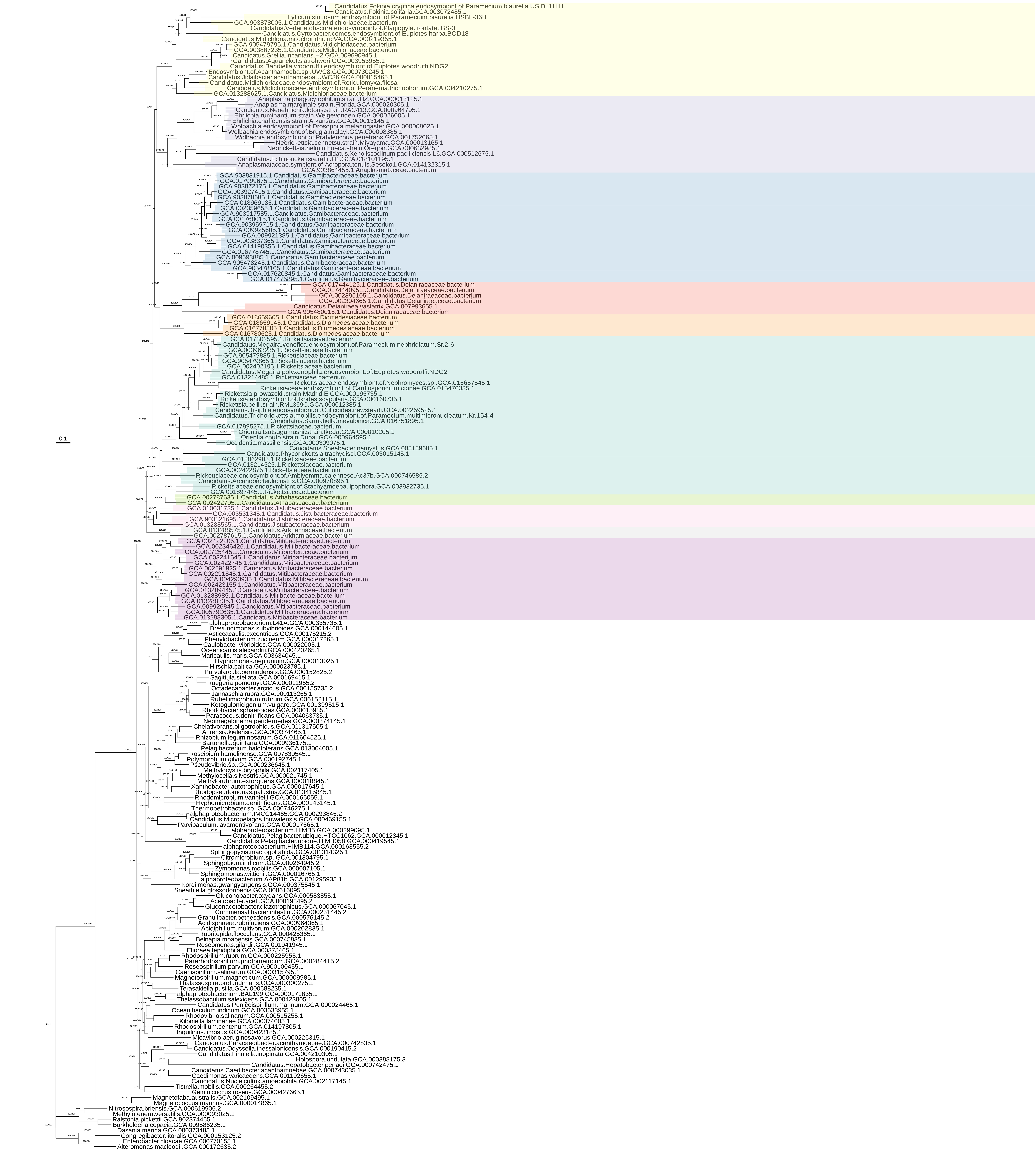

50% most compositionally heterogeneous sites removed

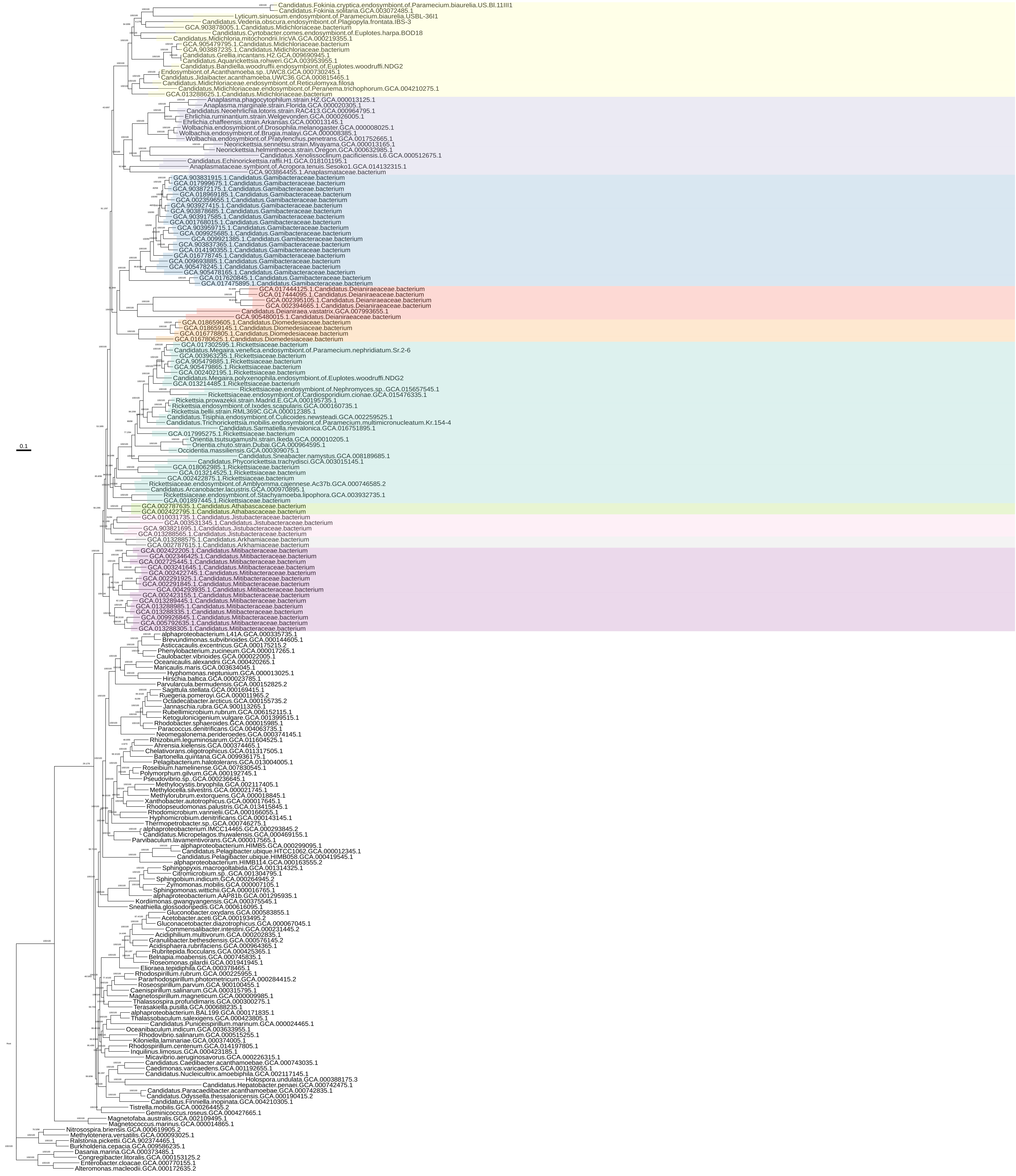

Supplementary Figure 19: copy numbers of "homology groups" in the analysed *Rickettsiales*.

Each column represents total values, or subdivided by functional category.

	Total	A	B	C	D	E	F	G	H	I	J	K	L	M	N	O	P	Q	S	T	U	V	W	Z
Candidatus_Midichloria_mitochondrii_IricVA_GCA_000219355.1	706	0	0	69	19	34	25	26	40	25	122	23	68	59	27	38	23	4	36	12	38	10	0	0
Candidatus_Cyrtobacter_comes_endosymbiont_of_Euplotes_harpa_BOD18	681	0	0	61	21	32	26	19	33	28	119	34	71	40	24	34	21	3	34	9	42	11	0	0
Lyticum_sinuosum_endosymbiont_of_Paramecium_biaurelia_USBL-36I1	554	0	0	44	17	26	25	19	33	27	113	16	38	46	26	30	13	5	22	9	40	8	0	0
Candidatus_Fokinia_solitaria_GCA_003072485.1	518	0	0	49	13	16	25	19	27	27	112	18	57	29	6	25	16	4	22	10	35	9	0	0
Candidatus_Fokinia_cryptica_endosymbiont_of_Paramecium_biaurelia_US_Bl_11III1	514	0	0	46	15	16	26	20	28	28	112	17	51	31	6	26	15	3	20	10	34	9	0	0
GCA_903878005.1_Candidatus_Midichloriaceae_bacterium	738	0	0	60	19	49	25	26	47	28	106	34	66	53	31	36	21	8	48	27	35	17	0	0
Candidatus_Vederia_obscura_endosymbiont_of_Plagiopyla_frontata_IBS-3	566	0	0	26	23	27	24	18	17	28	112	17	50	53	29	28	10	5	27	6	41	11	0	0
Candidatus_Bandiella_woodruffii_endosymbiont_of_Euplotes_woodruffi_NDG2	969	0	0	67	27	33	29	19	40	26	128	26	264	59	5	37	22	4	38	12	32	9	0	0
GCA_905479795.1_Candidatus_Midichloriaceae_bacterium	769	0	0	67	19	37	30	28	40	21	117	30	75	52	28	41	27	11	54	13	39	12	0	0
GCA_903887235.1_Candidatus_Midichloriaceae_bacterium	959	0	0	73	30	48	33	30	51	28	127	41	119	64	34	50	29	11	66	20	52	17	0	0
Candidatus_Grellia_incantans_H2_GCA_009690945.1	982	0	0	75	34	61	33	30	63	27	131	35	113	62	33	54	35	11	77	18	50	20	0	0
Candidatus_Aquarickettsia_rohweri_GCA_003953955.1	884	0	0	72	20	44	30	29	53	26	132	33	82	63	29	48	26	8	54	19	44	15	0	0
Candidatus_Jidaibacter_acanthamoeba_UWC36_GCA_008815465.1	1145	0	0	76	30	54	28	34	47	33	138	62	122	92	44	48	29	12	121	26	73	22	0	0
Endosymbiont_of_Acanthamoeba_sp._UWC8_GCA_007730245.1	1033	0	0	78	26	49	33	34	50	36	139	42	88	89	44	53	26	7	113	27	60	18	0	0
Candidatus_Midichloriaceae_endosymbiont_of_Reticulomyxa_filosa	897	0	0	73	31	44	28	29	38	35	134	32	110	85	11	43	37	7	58	18	42	22	0	0
Candidatus_Midichloriaceae_endosymbiont_of_Peranema_trichophorum_GCA_004210275.1	766	0	0	31	23	55	25	18	37	30	128	22	85	80	9	33	18	8	58	17	52	9	0	0
GCA_013288625.1_Candidatus_Midichloriaceae_bacterium	784	0	0	50	28	44	27	32	32	27	87	42	56	97	11	41	37	10	70	18	44	29	0	0
Anaplasma_phagocytophilum_strain_HZ_GCA_000013125.1	737	0	0	80	22	25	47	27	63	26	137	24	71	18	7	47	35	6	38	11	41	4	0	0
Anaplasma_marginale_strain_Florida_GCA_000020305.1	718	0	0	73	21	34	42	26	58	27	126	21	63	30	6	45	33	6	42	11	40	4	0	0
Candidatus_Neoehrlichia_lotoris_strain_RAC413_GCA_000964795.1	711	0	0	70	21	36	41	25	55	27	123	23	63	33	6	43	33	7	42	9	38	4	0	0
Ehrlichia_ruminantium_strain_Welgevonden_GCA_000026005.1	685	0	0	70	17	37	40	24	54	26	123	23	65	18	5	43	34	7	39	9	36	4	0	0
Ehrlichia_chaffeensis_strain_Arkansas_GCA_000013145.1	707	0	0	71	17	38	43	24	56	27	127	22	69	18	6	47	35	7	39	9	37	4	0	0
Wolbachia_endosymbiont_of_Brugia_malayi_GCA_000008385.1	632	0	0	68	20	29	42	20	33	26	120	14	61	31	4	37	32	7	26	7	36	5	0	0
Wolbachia_endosymbiont_of_Drosophila_melanogaster_GCA_000008025.1	814	0	0	76	27	32	44	24	40	27	121	31	98	55	8	46	35	7	57	11	40	9	0	0
Wolbachia_endosymbiont_of_Pratylenchus_penetrans_GCA_001752665.1	610	0	0	66	18	33	38	19	31	27	113	14	59	23	6	39	31	7	29	5	36	7	0	0
Neorickettsia_sennetsu_strain_Miyayama_GCA_000013165.1	620	0	0	67	12	23	39	21	51	25	119	21	54	23	5	37	28	5	28	9	39	5	0	0
Neorickettsia_helminthoeca_strain_Oregon_GCA_000632985.1	623	0	0	67	15	28	41	22	53	25	113	20	49	32	4	35	28	4	27	9	40	5	0	0
Candidatus_Xenolissoclinum_pacificiensis_L6_GCA_000512675.1	525	0	0	54	14	16	32	18	29	25	91	15	54	28	22	27	24	4	21	5	35	9	0	0
Candidatus_Echinorickettsia_raffii_H1_GCA_018101195.1	743	0	0	71	20	43	45	24	53	24	122	24	64	41	23	42	41	6	42	8	41	7	0	0
Anaplasmataceae_symbiont_of_Acropora_tenuis_Sesoko1_GCA_014132315.1	593	0	0	57	12	31	37	22	32	27	107	17	58	18	8	36	29	2	42	13	36	8	0	0
GCA_903864455.1_Anaplasmataceae_bacterium	580	0	0	61	15	33	37	19	43	26	101	17	52	35	4	35	22	4	23	7	35	15	0	0
Candidatus_Deianiraea_vastatrix_GCA_007993655.1	704	0	0	64	17	76	24	24	39	28	110	23	75	70	5	28	17	4	32	9	34	21	0	0
GCA_017444125.1_Candidatus_Deianiraeaceae_bacterium	462	0	0	26	12	35	18	9	13	23	108	20	47	46	6	18	14	4	18	6	29	6	0	0
GCA_017444095.1_Candidatus_Deianiraeaceae_bacterium	467	1	0	24	14	31	19	4	17	19	113	19	61	29	7	22	10	4	20	6	31	11	0	0
GCA_002395105.1_Candidatus_Deianiraeaceae_bacterium	402	0	0	22	10	21	13	5	13	19	103	15	49	33	7	20	7	3	21	5	26	6	0	0
GCA_002394665.1_Candidatus_Deianiraeaceae_bacterium	528	1	0	28	13	58	23	11	12	23	114	20	55	48	8	23	9	4	27	9	33	10	0	0
GCA_905480015.1_Candidatus_Deianiraeaceae_bacterium	754	0	0	69	15	88	46	21	59	28	105	16	82	60	11	29	23	3	42	8	37	16	0	0
GCA_018969185.1_Candidatus_Gambibacteraceae_bacterium	1037	0	0	70	24	106	46	33	66	38	134	34	107	82	9	54	50	6	71	18	50	27	0	0
GCA_002359655.1_Candidatus_Gambibacteraceae_bacterium	1214	1	0	80	29	115	55	40	71	39	141	38	135	87	10	61	57	10	121	28	56	27	0	0
GCA_009693885.1_Candidatus_Gambibacteraceae_bacterium	603	0	0	51	14	72	32	14	41	24	88	17	43	40	10	28	38	7	34	9	40	5	0	0
GCA_903872175.1_Candidatus_Gambibacteraceae_bacterium	1280	0	0	90	32	115	59	49	78	39	144	41	148	92	13	63	61	9	129	22	63	39	0	0
GCA_903831915.1_Candidatus_Gambibacteraceae_bacterium	1109	0	0	87	28	115	53	33	75	38	143	42	99	83	11	62	60	8	77	21	51	24	0	0
GCA_017999675.1_Candidatus_Gambibacteraceae_bacterium	1014	0	0	85	24	104	52	33	79	35	133	30	89	74	10	51	60	4	75	16	45	15	0	0
GCA_903927415.1_Candidatus_Gambibacteraceae_bacterium	1065	1	0	81	27	110	52	40	79	32	135	35	90	85	11	50	53	8	86	17	48	20	0	0
GCA_903878685.1_Candidatus_Gambibacteraceae_bacterium	1108	1	0	87	32	106	52	37	81	35	140	43	101	87	10	50	56	6	93	20	51	19	1	0
GCA_903917585.1_Candidatus_Gambibacteraceae_bacterium	950	2	0	76	22	103	47	31	67	29	130	24	77	74	10	50	48	7	60	19	43	26	0	0
GCA_001768015.1_Candidatus_Gambibacteraceae_bacterium	1029	0	0	85	30	110	49	30	78	34	138	34	93	76	9	55	57	5	58	13	46	25	0	0
GCA_903837365.1_Candidatus_Gambibacteraceae_bacterium	843	0	0	68	19	93	49	32	59	31	119	24	65	65	7	47	43	4	46	14	42	16	0	0
GCA_014190355.1_Candidatus_Gambibacteraceae_bacterium	975	1	0	75	23	110	53	33	72	34	137	26	80	76	9	50	51	5	62	13	48	19	0	0
GCA_903959715.1_Candidatus_Gambibacteraceae_bacterium	918	0	0	74	29	100	49	28	66	34	125	29	76	69	10	39	41	3	56	16	48	25	0	0
GCA_009925685.1_Candidatus_Gambibacteraceae_bacterium	933	0	0	75	22	108	49	33	68	36	125	30	82	72	10	44	50	5	50	14	43	14	0	0
GCA_009921385.1_Candidatus_Gambibacteraceae_bacterium	879	0	0	76	21	99	54	29	68	33	137	21	68	64	10	40	41	7	45	11	44	20	0	0
GCA_016778745.1_Candidatus_Gambibacteraceae_bacterium	829	0	0	69	16	83	41	29	61	33	125	20	76	66	10	42	47	6	45	13	38	15	0	0
GCA_905478245.1_Candidatus_Gambibacteraceae_bacterium	936	0	0	73	24	97	51	31	66	31	131	30	79	70	10	51	48	5	55	12	44	20	0	0
GCA_905478165.1_Candidatus_Gambibacteraceae_bacterium	852	0	0	72	21	98	49	32	66	32	129	22	75	77	9	46	36	5	41	7	40	13	0	0
GCA_017620845.1_Candidatus_Gambibacteraceae_bacterium	602	0	0	23	15	21	27	24	20	20	118	23	78	68	10	27	12	10	34	7	42	19	0	0
GCA_017475895.1_Candidatus_Gambibacteraceae_bacterium	587	1	0	32	11	23	25	20	21	22	122	22	71	61	10	27	8	10	35	8	39	18	0	0
GCA_016780625.1_Candidatus_Diomedesiaceae_bacterium	740	0	0	67	19	83	42	24	59	27	102	14	64	49	7	40	47	3	41	10	35	7	0	0
GCA_018659605.1_Candidatus_Diomedesiaceae_bacterium	851	1	0	69	19	97	44	26	61	29	116	28	71	63	10	38	48	4	54	19	43	14	0	0
GCA_018659145.1_Candidatus_Diomedesiaceae_bacterium	1067	1	0	80	29	112	49	39	76	35	132	35	93	80	12	53	54	9	74	24	50	25	0	0
GCA_016778805.1_Candidatus_Diomedesiaceae_bacterium	865	1	0	71	19	97	46	32	62	29	111	25	74	68	12	48	49	3	57	14	42	12	0	0
Rickettsia_prowazekii_strain_Madrid_E_GCA_000195735.1	733	1	0	79	25	29	23	18	28	29	130	21	65	63	5	42	32	5	58	13	45	11	0	0
Rickettsia_endosymbiont_of_Ixodes_scapularis_GCA_000160735.1	1187	1	0	78	31	45	25	23	38	22	126	53	200	88	4	42	41	10	159	28	54	22	0	0
Rickettsia_bellii_strain_RML369C_GCA_000012385.1	1074	1	0	85	29	43	24	31	34	31	138	51	120	97	10	44	44	8	134	25	58	35	0	0
Candidatus_Tisiphia_endosymbiont_of_Culicoides_newsteadi_GCA_002259525.1	907	0	0	76	29	29	20	25	34	24	132	54	95	86	3	45	37	9	79	28	51	25	0	0
Candidatus_Trichorickettsia_mobilis_endosymbiont_of_Paramecium_multimicronucleatum_Kr_154-4	1207	0	0	91	38	63	33	39	41	37	143	54	128	102	34	58	53	14	130	34	59	30	0	0
Candidatus_Megaira_polyxenophila_endosymbiont_of_Euplotes_woodruffi_NDG2	1206	1	0	86	40	53	23	33	34	34	142	77	166	105	11	56	52	17	124	41	66	28	0	0
GCA_017302595.1_Rickettsiaceae_bacterium	918	0	0	83	26	46	22	33	33	34	140	33	76	71	30	47	47	5	82	27	49	18	0	0
Candidatus_Megaira_venefica_endosymbiont_of_Paramecium_nephridiatum_Sr_2-6	1117	0	0	91	36	50	27	38	30	33	147	53	123	93	33	58	46	12	116	34	56	26	0	0
GCA_003963235.1_Rickettsiaceae_bacterium	809	0	0	71	23	43	16	33	28	25	121	31	92	67	26	43	32	6	67	25	43	13	0	0
GCA_905479885.1_Rickettsiaceae_bacterium	851	0	0	84	26	38	22	30	32	28	110	30	76	75	30	49	36	8	79	25	50	17	0	0
GCA_905479865.1_Rickettsiaceae_bacterium	871	0	0	81	31	43	19	30	34	26	133	31	87	71	6	49	37	6	76	23	52	21	0	0
GCA_002402195.1_Rickettsiaceae_bacterium	955	0	0	78	31	46	26	26	38	23	129	47	106	78	29	49	36	9	83	28	39	24	0	0
GCA_013214485.1_Rickettsiaceae_bacterium	1029	0	0	91	34	54	25	33	47	37	138	37	95	97	27	51	39	12	104	23	55	23	0	0
Rickettsiaceae_endosymbiont_of_Nephromyces_sp._GCA_015657545.1	588	0	0	66	20	34	15	11	17	23	117	22	61	33	24	38	21	5	31	5	39	6	0	0
Rickettsiaceae_endosymbiont_of_Cardiosporidium_cionae_GCA_015476335.1	702	0	0	72	28	38	18	17	20	26	119	22	67	49	26	45	29	7	51	12	47	7	0	3
Candidatus_Sarmatiella_mevalonica_GCA_016751895.1	688	0	0	48	24	27	18	14	26	32	123	19	81	48	23	29	23	4	34	13	44	15	0	0
GCA_017995275.1_Rickettsiaceae_bacterium	646	0	0	60	24	32	19	21	21	26	117	12	61	62	4	36	25	7	52	14	33	15	0	0
Orientia_tsutsugamushi_strain_Ikeda_GCA_000010205.1	1263	1	0	66	22	40	18	13	24	18	121	77	221	65	4	38	28	9	239	66	88	10	0	0
Orientia_chuto_strain_Dubai_GCA_000964595.1	651	1	0	64	22	27	17	13	20	18	125	29	69	31	4	39	28	7	65	15	55	7	0	0
Occidentia_massiliensis_GCA_000309075.1	840	0	0	86	27	56	24	27	34	25	132	28	86	77	6	50	32	5	63	17	48	17	0	0
Candidatus_Sneabacter_namystus_GCA_008189685.1	438	0	0	24	19	23	13	6	8	15	112	25	55	20	21	28	9	1	26	10	22	8	0	0
Candidatus_Phycorickettsia_trachydisci_GCA_003015145.1	737	0	0	60	26	41	22	20	19	30	123	25	85	68	6	34	26	9	42	8	47	22	0	0
GCA_018062985.1_Rickettsiaceae_bacterium	553	0	0	44	13	28	18	27	35	21	98	14	49	50	6	32	29	1	33	11	27	12	0	0
GCA_013214525.1_Rickettsiaceae_bacterium	805	1	0	35	21	56	35	31	36	28	130	28	91	78	5	33	32	8	58	17	49	25	0	0
GCA_002422875.1_Rickettsiaceae_bacterium	738	0	0	64	22	30	25	29	37	29	120	29	69	57	15	35	35	5	62	23	38	11	0	0
Rickettsiaceae_endosymbiont_of_Amblyomma_cajennese_Ac37b_GCA_007746585.2	1213	0	0	86	26	48	52	42	58	39	137	40	85	86	47	56	54	8	102	38	64	15	0	0
Candidatus_Arcanobacter_lacustris_GCA_009970895.1	530	0	0	30	20	22	12	18	17	23	73	25	55	34	31	23	18	4	37	23	26	8	0	0
Rickettsiaceae_endosymbiont_of_Stachyamoeba_lipophora_GCA_003932735.1	987	0	0	81	23	55	35	39	53	36	141	32	84	80	42	52	46	9	70	30	51	22	0	0
GCA_001897445.1_Rickettsiaceae_bacterium	1054	0	0	84	30	51	36	39	51	40	137	38	89	85	53	52	55	8	86	46	52	27	0	0
GCA_002787635.1_Candidatus_Athabascaceae_bacterium	1452	0	0	102	28	126	64	42	82	47	143	50	122	100	72	76	76	20	139	62	83	21	4	0
GCA_002422795.1_Candidatus_Athabascaceae_bacterium	1456	0	0	93	31	116	50	42	86	50	145	65	123	94	30	88	88	21	155	52	67	25	0	0
GCA_013288565.1_Candidatus_Jistubacteraceae_bacterium	955	0	1	63	22	98	31	29	62	41	101	35	65	91	39	64	51	19	70	25	40	11	0	0
GCA_903821695.1_Candidatus_Jistubacteraceae_bacterium	1443	0	0	101	32	116	60	47	85	48	139	52	123	111	61	74	88	17	163	46	62	23	0	0
GCA_010031735.1_Candidatus_Jistubacteraceae_bacterium	1414	0	0	95	25	123	55	40	91	45	143	48	116	109	70	87	78	22	139	59	65	23	0	0
GCA_003531345.1_Candidatus_Jistubacteraceae_bacterium	890	0	0	78	19	87	39	25	47	32	105	23	62	75	57	48	41	10	69	38	47	11	0	0
GCA_013288575.1_Candidatus_Arkhamiaceae_bacterium	662	0	0	55	14	65	32	20	44	28	78	24	52	54	34	39	22	6	51	20	27	11	0	1
GCA_002787615.1_Candidatus_Arkhamiaceae_bacterium	1027	0	0	83	18	91	45	33	71	35	115	28	75	76	52	52	49	15	94	39	62	7	0	0
GCA_013289445.1_Candidatus_Mitibacteraceae_bacterium	859	0	1	58	23	83	36	35	44	32	95	18	46	71	32	61	42	16	72	29	47	13	0	1
GCA_013288985.1_Candidatus_Mitibacteraceae_bacterium	845	1	0	65	17	76	41	25	43	33	94	23	53	56	32	50	47	15	77	33	47	11	0	0
GCA_013288335.1_Candidatus_Mitibacteraceae_bacterium	971	1	1	59	23	80	42	38	55	31	102	29	69	79	39	50	43	18	84	45	58	15	0	2
GCA_013288305.1_Candidatus_Mitibacteraceae_bacterium	752	0	0	65	20	60	25	18	48	31	81	28	40	54	40	34	41	9	54	32	35	9	0	0
GCA_009926845.1_Candidatus_Mitibacteraceae_bacterium	1273	0	0	96	30	113	47	36	77	46	138	49	86	89	62	68	68	11	128	53	63	15	0	0
GCA_005792635.1_Candidatus_Mitibacteraceae_bacterium	1442	0	0	90	35	117	55	48	99	48	142	77	105	87	77	68	68	13	133	60	73	15	0	0
GCA_003241645.1_Candidatus_Mitibacteraceae_bacterium	1380	0	0	91	33	119	57	49	77	49	139	45	108	100	52	82	73	19	142	49	79	24	0	0
GCA_002422745.1_Candidatus_Mitibacteraceae_bacterium	1184	0	0	55	29	109	53	36	74	36	120	40	87	90	65	66	52	16	123	51	74	15	0	0
GCA_002725445.1_Candidatus_Mitibacteraceae_bacterium	1117	1	0	76	23	102	42	45	55	31	121	36	81	79	45	56	67	12	120	53	60	15	0	0
GCA_002422205.1_Candidatus_Mitibacteraceae_bacterium	1491	0	0	95	40	128	57	49	80	44	148	51	108	105	79	90	84	13	146	70	80	23	0	1
GCA_002346425.1_Candidatus_Mitibacteraceae_bacterium	1458	0	0	92	36	122	57	63	77	46	145	53	116	101	74	77	69	16	154	67	65	21	0	0
GCA_002423155.1_Candidatus_Mitibacteraceae_bacterium	1407	0	0	93	42	118	54	43	76	41	144	67	111	87	80	68	63	10	138	63	66	25	0	0
GCA_004293935.1_Candidatus_Mitibacteraceae_bacterium	1128	0	0	79	26	113	49	38	63	38	126	35	96	82	54	58	45	14	97	41	62	18	1	0
GCA_002291925.1_Candidatus_Mitibacteraceae_bacterium	1466	1	0	94	34	135	57	62	78	49	154	43	111	104	71	82	63	21	131	72	75	21	1	2
GCA_002291845.1_Candidatus_Mitibacteraceae_bacterium	1262	0	0	81	30	108	50	55	80	44	132	38	99	91	69	74	56	19	103	56	63	23	0	0

Supplementary Figure 20: components of type IV and type VI secretion systems in the *Rickettsiales*. Heat-map showing the numbers of orthologs identified for each component of type IV and type VI secretion systems in the *Rickettsiales* of the final dataset, ordered by their phylogenetic relationships (Supplementary Figure 18), with families highlighted by different colours on the left. The bar plots on the right indicate the percentage of proteobacterial BUSCO single-copy orthogroups identified in each assembly.

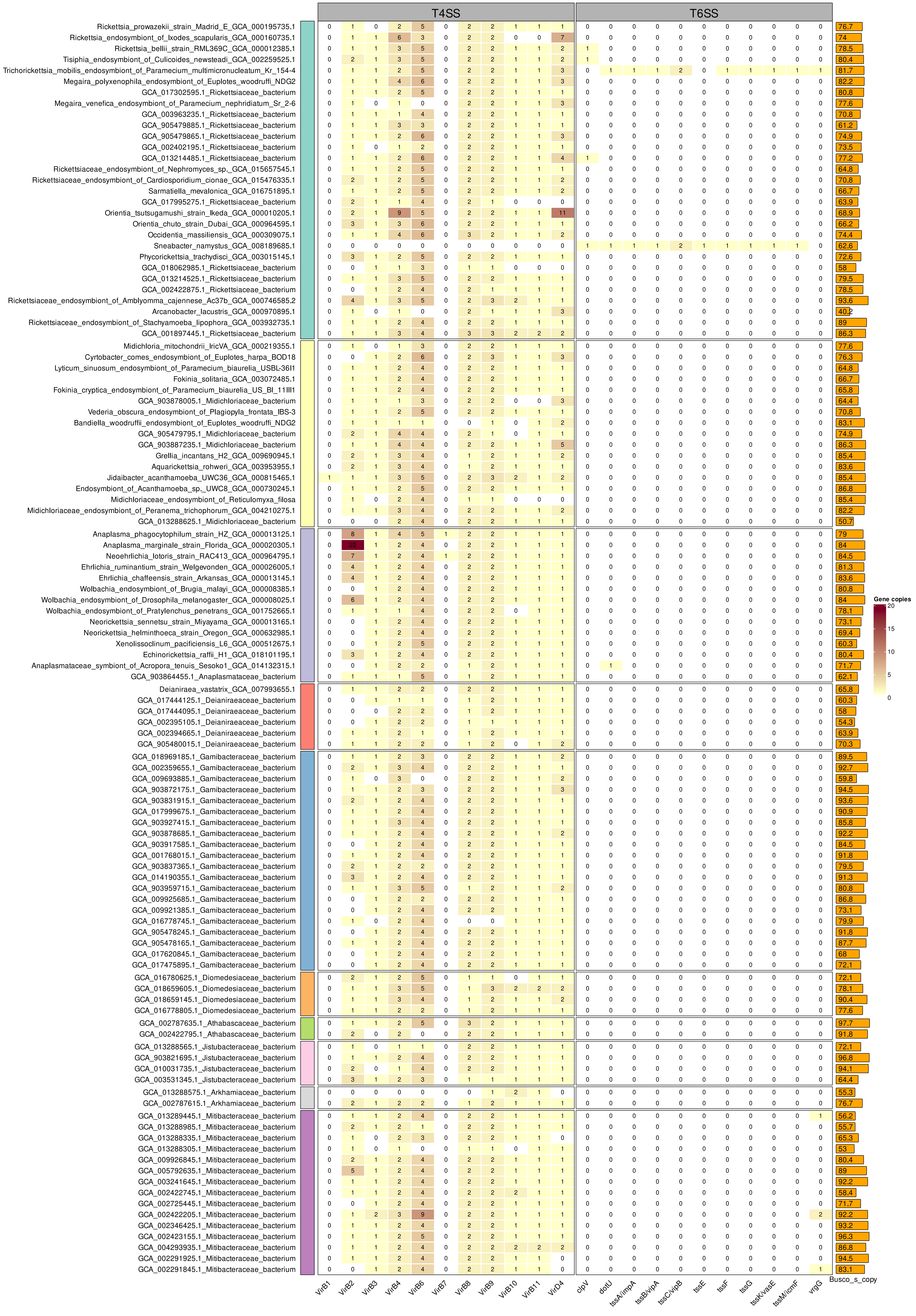
T4SS
T6SS
Rickettsia_prowazekii_strain_Madrid_E_GCA_000195735.1
Rickettsia_endosymbiont_of_Ixodes_scapularis_GCA_000160735.1
Rickettsia_bellii_strain_RML369C_GCA_000012385.1
Tisiphia_endosymbiont_of_Culicoides_newsteadi_GCA_002259525.1
Trichorickettsia_mobilis_endosymbiont_of_Paramecium_multimicronucleatum_Kr_154-4
Megaira_polyxenophila_endosymbiont_of_Euplotes_woodruffi_NDG2
GCA_017302595.1_Rickettsiaceae_bacterium
Megaira_venefica_endosymbiont_of_Paramecium_nephridiatum_Sr_2-6
GCA_003963235.1_Rickettsiaceae_bacterium
GCA_905479885.1_Rickettsiaceae_bacterium
GCA_905479865.1_Rickettsiaceae_bacterium
GCA_002402195.1_Rickettsiaceae_bacterium
GCA_013214485.1_Rickettsiaceae_bacterium
Rickettsiaceae_endosymbiont_of_Nephromyces_sp._GCA_015657545.1
Rickettsiaceae_endosymbiont_of_Cardiosporidium_cionae_GCA_015476335.1
Sarmatiella_mevalonica_GCA_016751895.1
GCA_017995275.1_Rickettsiaceae_bacterium
Orientia_tsutsugamushi_strain_Ikeda_GCA_000010205.1
Orientia_chuto_strain_Dubai_GCA_000964595.1
Occidentia_massiliensis_GCA_000309075.1
Sneabacter_namystus_GCA_008189685.1
Phycorickettsia_trachydisci_GCA_003015145.1
GCA_018062985.1_Rickettsiaceae_bacterium
GCA_013214525.1_Rickettsiaceae_bacterium
GCA_002422875.1_Rickettsiaceae_bacterium
Rickettsiaceae_endosymbiont_of_Amblyomma_cajennense_Ac37b_GCA_000746585.2
Arcanobacter_lacustris_GCA_000970895.1
Rickettsiaceae_endosymbiont_of_Stachyamoeba_lipophora_GCA_003932735.1
GCA_001897445.1_Rickettsiaceae_bacterium
Midichloria_mitochondrii_IricVA_GCA_000219355.1
Cyrtobacter_comes_endosymbiont_of_Euplotes_harpa_BOD18
Lyticum_sinuosum_endosymbiont_of_Paramecium_biaurelia_USBL-36I1
Fokinia_solitaria_GCA_003072485.1
Fokinia_cryptica_endosymbiont_of_Paramecium_biaurelia_US_Bl_11III1
GCA_903878005.1_Midichloriaceae_bacterium
Vederia_obscura_endosymbiont_of_Plagiopyla_frontata_IBS-3
Bandiella_woodruffii_endosymbiont_of_Euplotes_woodruffi_NDG2
GCA_905479795.1_Midichloriaceae_bacterium
GCA_903887235.1_Midichloriaceae_bacterium
Grellia_incantans_H2_GCA_009690945.1
Aquarickettsia_rohweri_GCA_003953955.1
Jidaibacter_acanthamoeba_UWC36_GCA_000815465.1
Endosymbiont_of_Acanthamoeba_sp._UWC8_GCA_000730245.1
Midichloriaceae_endosymbiont_of_Reticulomyxa_filosa
Midichloriaceae_endosymbiont_of_Peranema_trichophorum_GCA_004210275.1
GCA_013288625.1_Midichloriaceae_bacterium
Anaplasma_phagocytophilum_strain_HZ_GCA_000013125.1
Anaplasma_marginale_strain_Florida_GCA_000020305.1
Neoehrlichia_lotoris_strain_RAC413_GCA_000964795.1
Ehrlichia_ruminantium_strain_Welgevonden_GCA_000026005.1
Ehrlichia_chaffeensis_strain_Arkansas_GCA_000013145.1
Wolbachia_endosymbiont_of_Brugia_malayi_GCA_000008385.1
Wolbachia_endosymbiont_of_Drosophila_melanogaster_GCA_000008025.1
Wolbachia_endosymbiont_of_Pratylenchus_penetrans_GCA_001752665.1
Neorickettsia_sennetsu_strain_Miyayama_GCA_000013165.1
Neorickettsia_helminthoeca_strain_Oregon_GCA_000632985.1
Xenolissoclinum_pacificiensis_L6_GCA_000512675.1
Echinorickettsia_raffii_H1_GCA_018101195.1
Anaplasmataceae_symbiont_of_Acropora_tenuis_Sesoko1_GCA_014132315.1
GCA_903864455.1_Anaplasmataceae_bacterium
Deianiraea_vastatrix_GCA_007993655.1
GCA_017444125.1_Deianiraeaceae_bacterium
GCA_017444095.1_Deianiraeaceae_bacterium
GCA_002395105.1_Deianiraeaceae_bacterium
GCA_002394665.1_Deianiraeaceae_bacterium
GCA_905480015.1_Deianiraeaceae_bacterium
GCA_018969185.1_Gamibacteraceae_bacterium
GCA_002359655.1_Gamibacteraceae_bacterium
GCA_009693885.1_Gamibacteraceae_bacterium
GCA_903872175.1_Gamibacteraceae_bacterium
GCA_903831915.1_Gamibacteraceae_bacterium
GCA_017999675.1_Gamibacteraceae_bacterium
GCA_903927415.1_Gamibacteraceae_bacterium
GCA_903878685.1_Gamibacteraceae_bacterium
GCA_903917585.1_Gamibacteraceae_bacterium
GCA_001768015.1_Gamibacteraceae_bacterium
GCA_903837365.1_Gamibacteraceae_bacterium
GCA_014190355.1_Gamibacteraceae_bacterium
GCA_903959715.1_Gamibacteraceae_bacterium
GCA_009925685.1_Gamibacteraceae_bacterium
GCA_009921385.1_Gamibacteraceae_bacterium
GCA_016778745.1_Gamibacteraceae_bacterium
GCA_905478245.1_Gamibacteraceae_bacterium
GCA_905478165.1_Gamibacteraceae_bacterium
GCA_017620845.1_Gamibacteraceae_bacterium
GCA_017475895.1_Gamibacteraceae_bacterium
GCA_016780625.1_Diomedesiaceae_bacterium
GCA_018659605.1_Diomedesiaceae_bacterium
GCA_018659145.1_Diomedesiaceae_bacterium
GCA_016778805.1_Diomedesiaceae_bacterium
GCA_002787635.1_Athabascaceae_bacterium
GCA_002422795.1_Athabascaceae_bacterium
GCA_013288565.1_Jistubacteraceae_bacterium
GCA_903821695.1_Jistubacteraceae_bacterium
GCA_010031735.1_Jistubacteraceae_bacterium
GCA_003531345.1_Jistubacteraceae_bacterium
GCA_013288575.1_Arkhamiaceae_bacterium
GCA_002787615.1_Arkhamiaceae_bacterium
GCA_013289445.1_Mitibacteraceae_bacterium
GCA_013288985.1_Mitibacteraceae_bacterium
GCA_013288335.1_Mitibacteraceae_bacterium
GCA_013288305.1_Mitibacteraceae_bacterium
GCA_009926845.1_Mitibacteraceae_bacterium
GCA_005792635.1_Mitibacteraceae_bacterium
GCA_003241645.1_Mitibacteraceae_bacterium
GCA_002422745.1_Mitibacteraceae_bacterium
GCA_002725445.1_Mitibacteraceae_bacterium
GCA_002422205.1_Mitibacteraceae_bacterium
GCA_002346425.1_Mitibacteraceae_bacterium
GCA_002423155.1_Mitibacteraceae_bacterium
GCA_004293935.1_Mitibacteraceae_bacterium
GCA_002291925.1_Mitibacteraceae_bacterium
GCA_002291845.1_Mitibacteraceae_bacterium
VirB1
VirB2
VirB3
VirB4
VirB6
VirB7
VirB8
VirB9
VirB10
VirB11
VirD4
clpV
dotU
tssA/impA
tssB/vipA
tssC/vipB
tssE
tssF
tssG
tssK/vasE
tssM/icmF
vrgG
Busco_s_copy
Gene copies
20
15
10
5
0

Supplementary Figure 21: putative effector protein in the *Rickettsiales*. Heat-map showing the numbers of orthologs identified for each putative effector protein in the *Rickettsiales* of the final dataset, ordered by their phylogenetic relationships (Supplementary Figure 18), with families highlighted by different colours on the left. The bar plots on the right indicate the percentage of proteobacterial BUSCO single-copy orthogroups identified in each assembly.

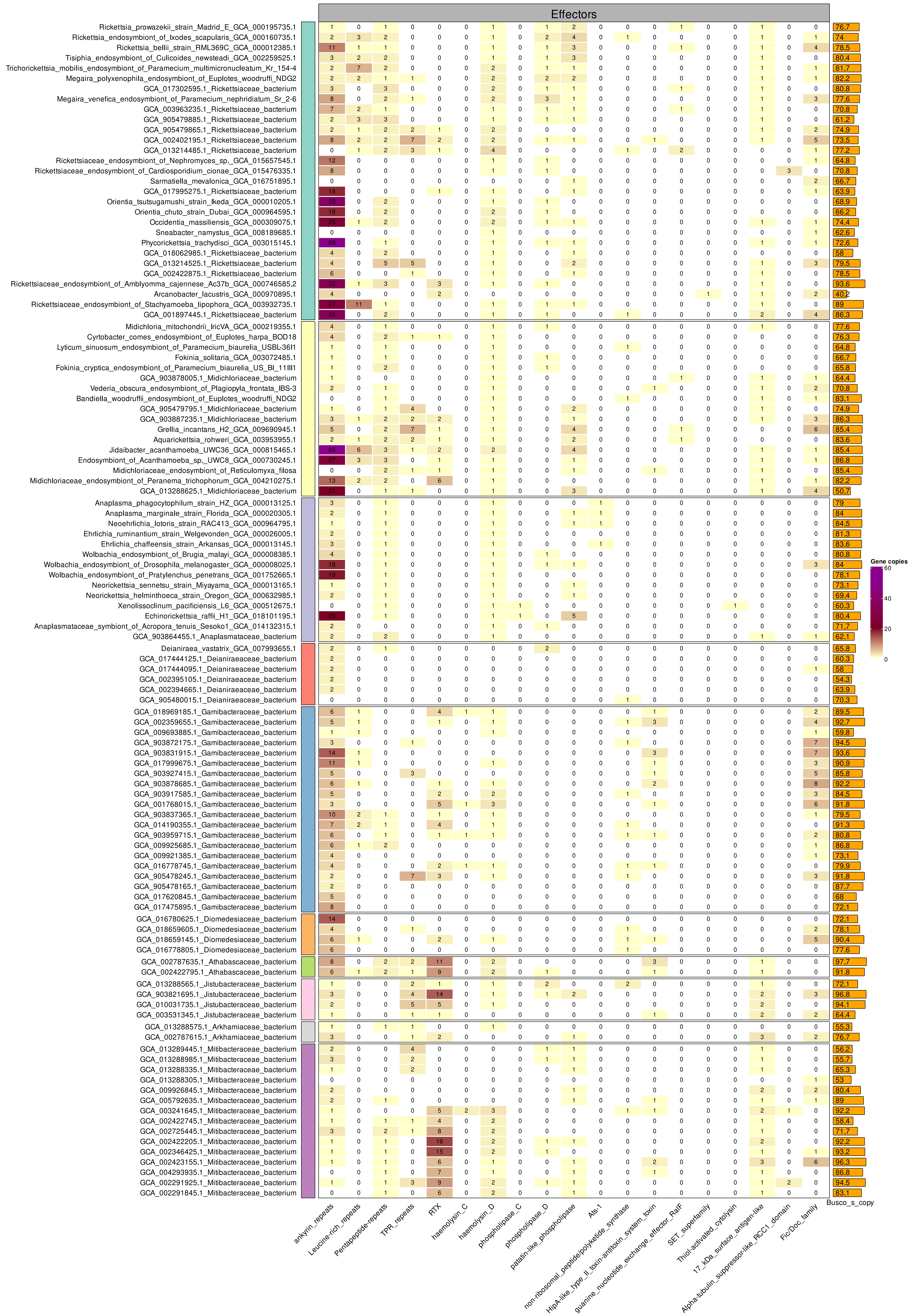
Effectors
Rickettsia_prowazekii_strain_Madrid_E_GCA_000195735.1
Rickettsia_endosymbiont_of_Ixodes_scapularis_GCA_000160735.1
Rickettsia_bellii_strain_RML369C_GCA_000012385.1
Tisiphia_endosymbiont_of_Culicoides_newsteadi_GCA_002259525.1
Trichorickettsia_mobilis_endosymbiont_of_Paramecium_multimicronucleatum_Kr_154-4
Megaira_polyxenophila_endosymbiont_of_Euplotes_woodruffi_NDG2
GCA_017302595.1_Rickettsiaceae_bacterium
Megaira_venefica_endosymbiont_of_Paramecium_nephridiatum_Sr_2-6
GCA_003963235.1_Rickettsiaceae_bacterium
GCA_905479885.1_Rickettsiaceae_bacterium
GCA_905479865.1_Rickettsiaceae_bacterium
GCA_002402195.1_Rickettsiaceae_bacterium
GCA_013214485.1_Rickettsiaceae_bacterium
Rickettsiaceae_endosymbiont_of_Nephromyces_sp._GCA_015657545.1
Rickettsiaceae_endosymbiont_of_Cardiosporidium_cionae_GCA_015476335.1
Sarmatiella_mevalonica_GCA_016751895.1
GCA_017995275.1_Rickettsiaceae_bacterium
Orientia_tsutsugamushi_strain_Ikeda_GCA_000010205.1
Orientia_chuto_strain_Dubai_GCA_000964595.1
Occidentia_massiliensis_GCA_000309075.1
Sneabacter_namystus_GCA_008189685.1
Phycorickettsia_trachydisci_GCA_003015145.1
GCA_018062985.1_Rickettsiaceae_bacterium
GCA_013214525.1_Rickettsiaceae_bacterium
GCA_002422875.1_Rickettsiaceae_bacterium
Rickettsiaceae_endosymbiont_of_Amblyomma_cajennese_Ac37b_GCA_000746585.2
Arcanobacter_lacustris_GCA_000970895.1
Rickettsiaceae_endosymbiont_of_Stachyamoeba_lipophora_GCA_003932735.1
GCA_001897445.1_Rickettsiaceae_bacterium
Midichloria_mitochondrii_IricVA_GCA_000219355.1
Cyrtobacter_comes_endosymbiont_of_Euplotes_harpa_BOD18
Lyticum_sinuosum_endosymbiont_of_Paramecium_biaurelia_USBL-36I1
Fokinia_solitaria_GCA_003072485.1
Fokinia_cryptica_endosymbiont_of_Paramecium_biaurelia_US_Bl_11III1
GCA_903878005.1_Midichloriaceae_bacterium
Vederia_obscura_endosymbiont_of_Plagiopyla_frontata_IBS-3
Bandiella_woodruffii_endosymbiont_of_Euplotes_woodruffi_NDG2
GCA_905479795.1_Midichloriaceae_bacterium
GCA_903887235.1_Midichloriaceae_bacterium
Grellia_incantans_H2_GCA_009690945.1
Aquarickettsia_rohweri_GCA_003953955.1
Jidaibacter_acanthamoeba_UWC36_GCA_000815465.1
Endosymbiont_of_Acanthamoeba_sp._UWC8_GCA_000730245.1
Midichloriaceae_endosymbiont_of_Reticulomyxa_filosa
Midichloriaceae_endosymbiont_of_Peranema_trichophorum_GCA_004210275.1
GCA_013288625.1_Midichloriaceae_bacterium
Anaplasma_phagocytophilum_strain_HZ_GCA_000013125.1
Anaplasma_marginale_strain_Florida_GCA_000020305.1
Neoehrlichia_lotoris_strain_RAC413_GCA_000964795.1
Ehrlichia_ruminantium_strain_Welgevonden_GCA_000026005.1
Ehrlichia_chaffeensis_strain_Arkansas_GCA_000013145.1
Wolbachia_endosymbiont_of_Brugia_malayi_GCA_000008385.1
Wolbachia_endosymbiont_of_Drosophila_melanogaster_GCA_000008025.1
Wolbachia_endosymbiont_of_Pratylenchus_penetrans_GCA_001752665.1
Neorickettsia_sennetsu_strain_Miyayama_GCA_000013165.1
Neorickettsia_helminthoeca_strain_Oregon_GCA_000632985.1
Xenolissoclinum_pacificiensis_L6_GCA_000512675.1
Echinorickettsia_raffii_H1_GCA_018101195.1
Anaplasmataceae_symbiont_of_Acropora_tenuis_Sesoko1_GCA_014132315.1
GCA_903864455.1_Anaplasmataceae_bacterium
Deianiraea_vastatrix_GCA_007993665.1
GCA_017444125.1_Deianiraeaceae_bacterium
GCA_017444095.1_Deianiraeaceae_bacterium
GCA_002395105.1_Deianiraeaceae_bacterium
GCA_002394665.1_Deianiraeaceae_bacterium
GCA_905480015.1_Deianiraeaceae_bacterium
GCA_018969185.1_Gamibacteraceae_bacterium
GCA_002359655.1_Gamibacteraceae_bacterium
GCA_009693885.1_Gamibacteraceae_bacterium
GCA_903872175.1_Gamibacteraceae_bacterium
GCA_903831915.1_Gamibacteraceae_bacterium
GCA_017999675.1_Gamibacteraceae_bacterium
GCA_903927415.1_Gamibacteraceae_bacterium
GCA_903878685.1_Gamibacteraceae_bacterium
GCA_903917585.1_Gamibacteraceae_bacterium
GCA_001768015.1_Gamibacteraceae_bacterium
GCA_903837365.1_Gamibacteraceae_bacterium
GCA_014190355.1_Gamibacteraceae_bacterium
GCA_903959715.1_Gamibacteraceae_bacterium
GCA_009925685.1_Gamibacteraceae_bacterium
GCA_009921385.1_Gamibacteraceae_bacterium
GCA_016778745.1_Gamibacteraceae_bacterium
GCA_905478245.1_Gamibacteraceae_bacterium
GCA_905478165.1_Gamibacteraceae_bacterium
GCA_017620845.1_Gamibacteraceae_bacterium
GCA_017475895.1_Gamibacteraceae_bacterium
GCA_016780625.1_Diomedesiaceae_bacterium
GCA_018659605.1_Diomedesiaceae_bacterium
GCA_018659145.1_Diomedesiaceae_bacterium
GCA_016778805.1_Diomedesiaceae_bacterium
GCA_002787635.1_Athabascaceae_bacterium
GCA_002422795.1_Athabascaceae_bacterium
GCA_013288565.1_Jistubacteraceae_bacterium
GCA_903821695.1_Jistubacteraceae_bacterium
GCA_010031735.1_Jistubacteraceae_bacterium
GCA_003531345.1_Jistubacteraceae_bacterium
GCA_013288575.1_Arkhamiaceae_bacterium
GCA_002787615.1_Arkhamiaceae_bacterium
GCA_013289445.1_Mitibacteraceae_bacterium
GCA_013288985.1_Mitibacteraceae_bacterium
GCA_013288335.1_Mitibacteraceae_bacterium
GCA_013288305.1_Mitibacteraceae_bacterium
GCA_009926845.1_Mitibacteraceae_bacterium
GCA_005792635.1_Mitibacteraceae_bacterium
GCA_003241645.1_Mitibacteraceae_bacterium
GCA_002422745.1_Mitibacteraceae_bacterium
GCA_002725445.1_Mitibacteraceae_bacterium
GCA_002422205.1_Mitibacteraceae_bacterium
GCA_002346425.1_Mitibacteraceae_bacterium
GCA_002423155.1_Mitibacteraceae_bacterium
GCA_004293935.1_Mitibacteraceae_bacterium
GCA_002291925.1_Mitibacteraceae_bacterium
GCA_002291845.1_Mitibacteraceae_bacterium
ankyrin_repeats
Leucine-rich_repeats
Pentapeptide-repeats
TPR_repeats
RTX
haemolysin_C
haemolysin_D
phospholipase_C
phospholipase_D
patatin-like_phospholipase
Ats-1
non-ribosomal_peptide/polyketide_synthase
HipA-like_type_II_toxin-antitoxin_system_toxin
guanine_nucleotide_exchange_effector_RalF
SET_superfamily
Thiol-activated_cytolysin
17_kDa_surface_antigen-like
Alpha-tubulin_suppressor-like_RCC1_domain
Fic/Doc_family
Busco_s_copy
Gene copies
60
40
20
0

Supplementary Figure 22: component of flagellum and chemotaxis apparatuses in the *Rickettsiales*. Heat-map showing the numbers of orthologs identified for each component of flagellum and chemotaxis apparatuses in the *Rickettsiales* of the final dataset, ordered by their phylogenetic relationships (Supplementary Figure 18), with families highlighted by different colours on the left. The bar plots on the right indicate the percentage of proteobacterial BUSCO single-copy orthogroups identified in each assembly.

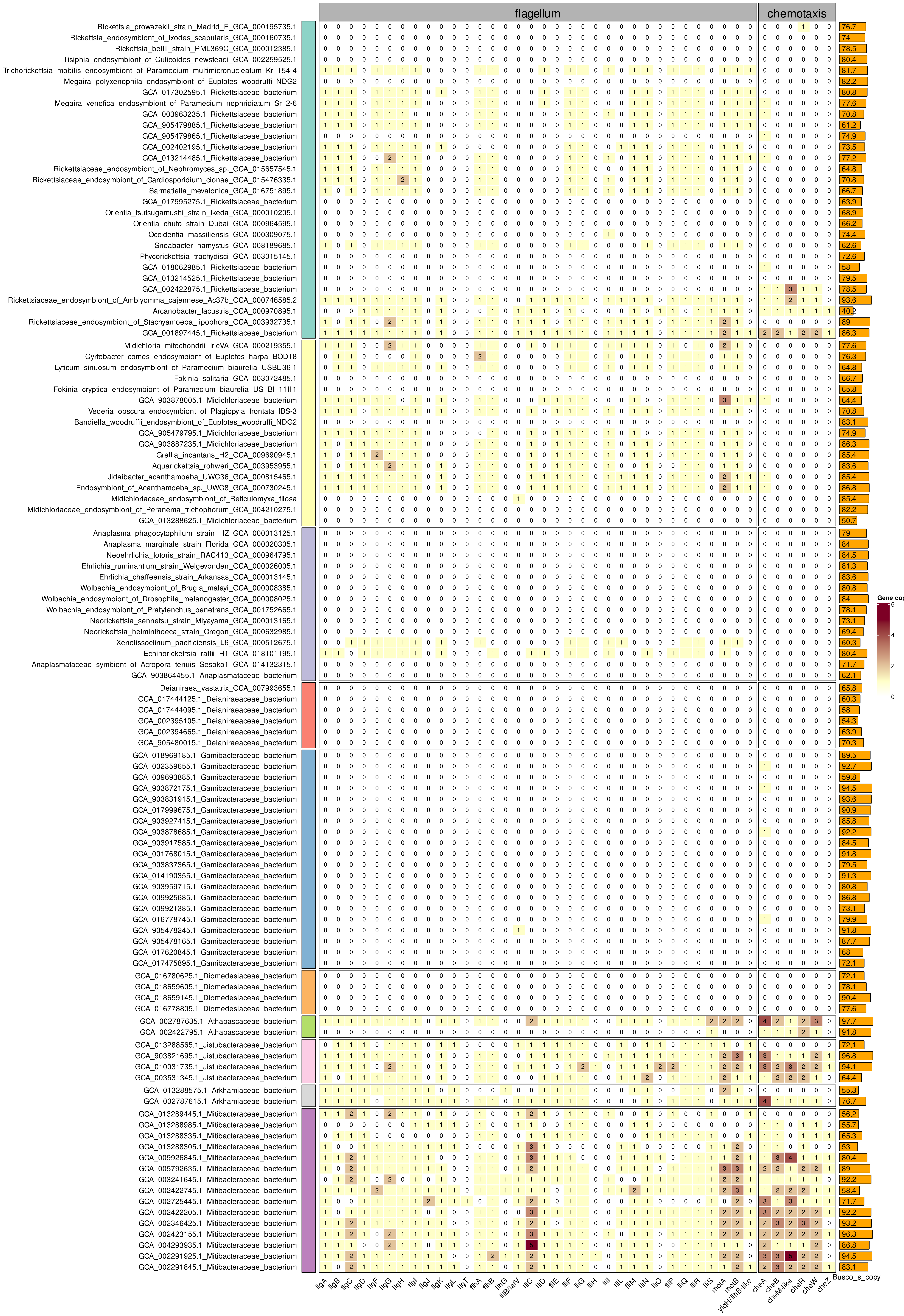
flagellum
chemotaxis
Rickettsia_prowazekii_strain_Madrid_E_GCA_000195735.1
Rickettsia_endosymbiont_of_Ixodes_scapularis_GCA_000160735.1
Rickettsia_bellii_strain_RML369C_GCA_000012385.1
Tisiphia_endosymbiont_of_Culicoides_newsteadi_GCA_002259525.1
Trichorickettsia_mobilis_endosymbiont_of_Paramecium_multimicronucleatum_Kr_154-4
Megaira_polyxenophila_endosymbiont_of_Euplotes_woodruffi_NDG2
GCA_017302595.1_Rickettsiaceae_bacterium
Megaira_venefica_endosymbiont_of_Paramecium_nephridiatum_Sr_2-6
GCA_003963235.1_Rickettsiaceae_bacterium
GCA_905479885.1_Rickettsiaceae_bacterium
GCA_905479865.1_Rickettsiaceae_bacterium
GCA_002402195.1_Rickettsiaceae_bacterium
GCA_013214485.1_Rickettsiaceae_bacterium
Rickettsiaceae_endosymbiont_of_Nephromyces_sp._GCA_015657545.1
Rickettsiaceae_endosymbiont_of_Cardiosporidium_cionae_GCA_015476335.1
Sarmatiella_mevalonica_GCA_016751895.1
GCA_017995275.1_Rickettsiaceae_bacterium
Orientia_tsutsugamushi_strain_Ikeda_GCA_000010205.1
Orientia_chuto_strain_Dubai_GCA_000964595.1
Occidentia_massiliensis_GCA_000309075.1
Sneabacter_namystus_GCA_008189685.1
Phycorickettsia_trachydisci_GCA_003015145.1
GCA_018062985.1_Rickettsiaceae_bacterium
GCA_013214525.1_Rickettsiaceae_bacterium
GCA_002422875.1_Rickettsiaceae_bacterium
Rickettsiaceae_endosymbiont_of_Amblyomma_cajennese_Ac37b_GCA_007746585.2
Arcanobacter_lacustris_GCA_000970895.1
Rickettsiaceae_endosymbiont_of_Stachyamoeba_lipophora_GCA_003932735.1
GCA_001897445.1_Rickettsiaceae_bacterium
Midichloria_mitochondrii_IricVA_GCA_000219355.1
Cyrtobacter_comes_endosymbiont_of_Euplotes_harpa_BOD18
Lyticum_sinuosum_endosymbiont_of_Paramecium_biaurelia_USBL-36I1
Fokinia_solitaria_GCA_003072485.1
Fokinia_cryptica_endosymbiont_of_Paramecium_biaurelia_US_Bl_11III1
GCA_903878005.1_Midichloriaceae_bacterium
Vederia_obscura_endosymbiont_of_Plagiopyla_frontata_IBS-3
Bandiella_woodruffii_endosymbiont_of_Euplotes_woodruffi_NDG2
GCA_905479795.1_Midichloriaceae_bacterium
GCA_903887235.1_Midichloriaceae_bacterium
Grellia_incantans_H2_GCA_009690945.1
Aquarickettsia_rohweri_GCA_003953955.1
Jidaibacter_acanthamoeba_UWC36_GCA_000815465.1
Endosymbiont_of_Acanthamoeba_sp._UWC8_GCA_000730245.1
Midichloriaceae_endosymbiont_of_Reticulomyxa_filosa
Midichloriaceae_endosymbiont_of_Peranema_trichophorum_GCA_004210275.1
GCA_013288625.1_Midichloriaceae_bacterium
Anaplasma_phagocytophilum_strain_HZ_GCA_000013125.1
Anaplasma_marginale_strain_Florida_GCA_000020305.1
Neoehrlichia_lotoris_strain_RAC413_GCA_000964795.1
Ehrlichia_ruminantium_strain_Welgevonden_GCA_000026005.1
Ehrlichia_chaffeensis_strain_Arkansas_GCA_000013145.1
Wolbachia_endosymbiont_of_Brugia_malayi_GCA_000008385.1
Wolbachia_endosymbiont_of_Drosophila_melanogaster_GCA_000008025.1
Wolbachia_endosymbiont_of_Pratylenchus_penetrans_GCA_001752665.1
Neorickettsia_sennetsu_strain_Miyayama_GCA_000013165.1
Neorickettsia_helminthoeca_strain_Oregon_GCA_000632985.1
Xenolissoclinum_pacificiensis_L6_GCA_000512675.1
Echinorickettsia_raffii_H1_GCA_018101195.1
Anaplasmataceae_symbiont_of_Acropora_tenuis_Sesoko1_GCA_014132315.1
GCA_903864455.1_Anaplasmataceae_bacterium
Deianiraea_vastatrix_GCA_007993655.1
GCA_017444125.1_Deianiraeaceae_bacterium
GCA_017444095.1_Deianiraeaceae_bacterium
GCA_002395105.1_Deianiraeaceae_bacterium
GCA_002394665.1_Deianiraeaceae_bacterium
GCA_905480015.1_Deianiraeaceae_bacterium
GCA_018969185.1_Gamibacteraceae_bacterium
GCA_002359655.1_Gamibacteraceae_bacterium
GCA_009693885.1_Gamibacteraceae_bacterium
GCA_903872175.1_Gamibacteraceae_bacterium
GCA_903831915.1_Gamibacteraceae_bacterium
GCA_017999675.1_Gamibacteraceae_bacterium
GCA_903927415.1_Gamibacteraceae_bacterium
GCA_903878685.1_Gamibacteraceae_bacterium
GCA_903917585.1_Gamibacteraceae_bacterium
GCA_001768015.1_Gamibacteraceae_bacterium
GCA_903837365.1_Gamibacteraceae_bacterium
GCA_014190355.1_Gamibacteraceae_bacterium
GCA_903959715.1_Gamibacteraceae_bacterium
GCA_009925685.1_Gamibacteraceae_bacterium
GCA_009921385.1_Gamibacteraceae_bacterium
GCA_016778745.1_Gamibacteraceae_bacterium
GCA_905478245.1_Gamibacteraceae_bacterium
GCA_905478165.1_Gamibacteraceae_bacterium
GCA_017620845.1_Gamibacteraceae_bacterium
GCA_017475895.1_Gamibacteraceae_bacterium
GCA_016780625.1_Diomedesiaceae_bacterium
GCA_018659605.1_Diomedesiaceae_bacterium
GCA_018659145.1_Diomedesiaceae_bacterium
GCA_016778805.1_Diomedesiaceae_bacterium
GCA_002787635.1_Athabascaceae_bacterium
GCA_002422795.1_Athabascaceae_bacterium
GCA_013288565.1_Jistubacteraceae_bacterium
GCA_903821695.1_Jistubacteraceae_bacterium
GCA_010031735.1_Jistubacteraceae_bacterium
GCA_003531345.1_Jistubacteraceae_bacterium
GCA_013288575.1_Arkhamiaceae_bacterium
GCA_002787615.1_Arkhamiaceae_bacterium
GCA_013289445.1_Mitibacteraceae_bacterium
GCA_013288985.1_Mitibacteraceae_bacterium
GCA_013288335.1_Mitibacteraceae_bacterium
GCA_013288305.1_Mitibacteraceae_bacterium
GCA_009926845.1_Mitibacteraceae_bacterium
GCA_005792635.1_Mitibacteraceae_bacterium
GCA_003241645.1_Mitibacteraceae_bacterium
GCA_002422745.1_Mitibacteraceae_bacterium
GCA_002725445.1_Mitibacteraceae_bacterium
GCA_002422205.1_Mitibacteraceae_bacterium
GCA_002346425.1_Mitibacteraceae_bacterium
GCA_002423155.1_Mitibacteraceae_bacterium
GCA_004293935.1_Mitibacteraceae_bacterium
GCA_002291925.1_Mitibacteraceae_bacterium
GCA_002291845.1_Mitibacteraceae_bacterium
flgA
flgB
flgC
flgD
flgF
flgG
flgH
flgI
flgJ
flgK
flgL
flgT
flhA
flhB
flhG
fliB/latV
fliC
fliD
fliE
fliF
fliG
fliH
fliI
fliL
fliM
fliN
fliO
fliP
fliQ
fliR
fliS
motA
motB
ylqH/flhB-like
cheA
cheB
cheM-like
cheR
cheW
cheZ
Busco_s_copy
Gene copies
6
4
2
0

Supplementary Figure 23: possible proteins proteins involved in the interaction with host cells in the *Rickettsiales*. Heat-map showing the numbers of orthologs identified for each component of multiple proteins possibly involved in the interaction with host cells in the *Rickettsiales* of the final dataset. Genes are ordered by functional groups (namely, components of type IV pilus/type II secretion, tight adherence pilus, fimbriae, curli, as well as exoproteins homologous to the FhaBC two-partner secretion system, previously characterised effectors in some *Rickettsiales* bacteria, and further other proteins). Organisms are ordered by their phylogenetic relationships (Supplementary Figure 18), with families highlighted by different colours on the left. The bar plots on the right indicate the percentage of proteobacterial BUSCO single-copy orthogroups identified in each assembly.

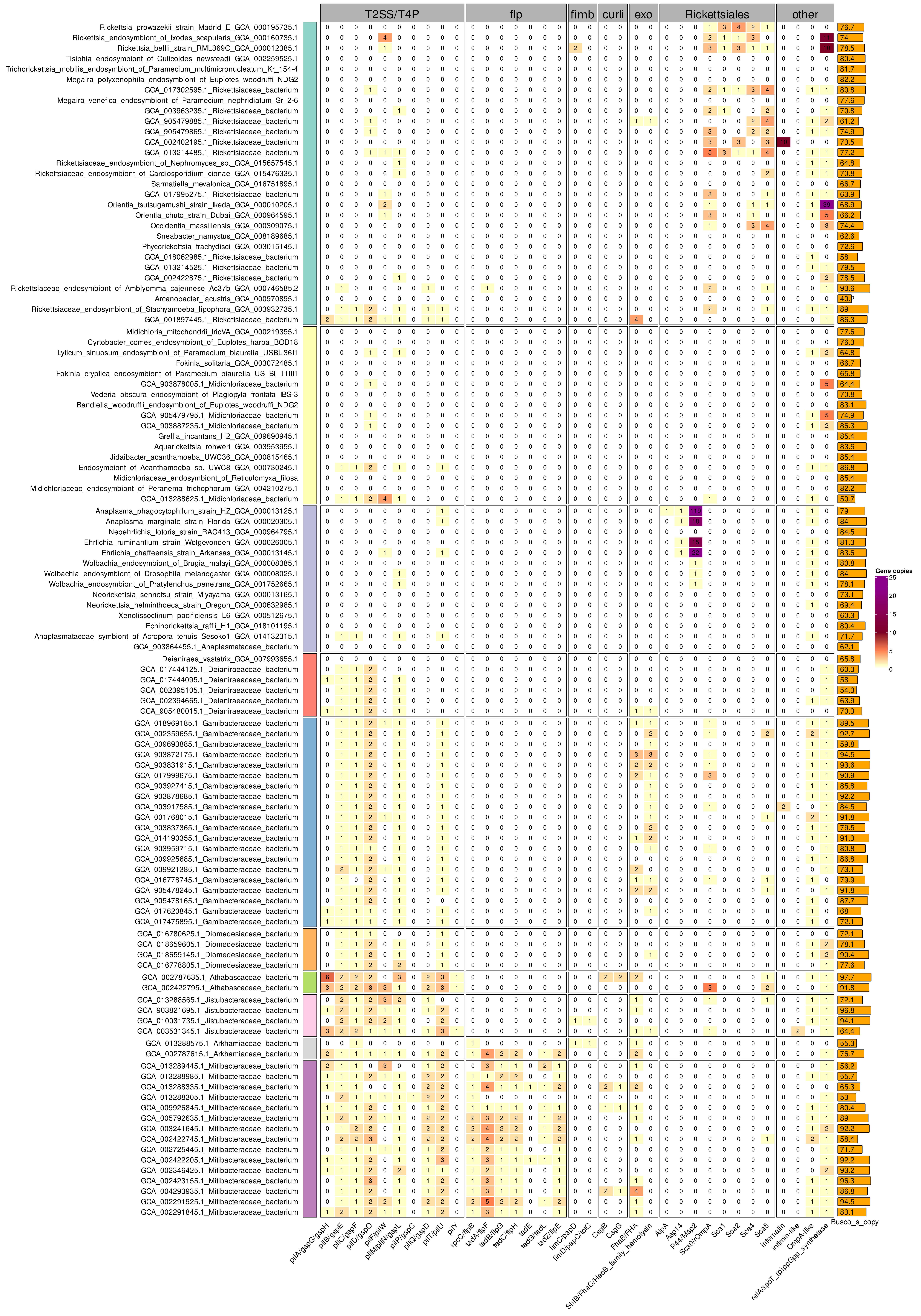

T2SS/T4P
flp
fimb
curli
exo
Rickettsiales
other
Rickettsia_prowazekii_strain_Madrid_E_GCA_000195735.1
Rickettsia_endosymbiont_of_Ixodes_scapularis_GCA_000160735.1
Rickettsia_bellii_strain_RML369C_GCA_000012385.1
Tisiphia_endosymbiont_of_Culicoides_newsteadi_GCA_002259525.1
Trichorickettsia_mobilis_endosymbiont_of_Paramecium_multimicronucleatum_Kr_154-4
Megaira_polyxenophila_endosymbiont_of_Euplotes_woodruffi_NDG2
GCA_017302595.1_Rickettsiaceae_bacterium
Megaira_venefica_endosymbiont_of_Paramecium_nephridiatum_Sr_2-6
GCA_003963235.1_Rickettsiaceae_bacterium
GCA_905479885.1_Rickettsiaceae_bacterium
GCA_905479865.1_Rickettsiaceae_bacterium
GCA_002402195.1_Rickettsiaceae_bacterium
GCA_013214485.1_Rickettsiaceae_bacterium
Rickettsiaceae_endosymbiont_of_Nephromyces_sp._GCA_015657545.1
Rickettsiaceae_endosymbiont_of_Cardiosporidium_cionae_GCA_015476335.1
Sarmatiella_mevalonica_GCA_016751895.1
GCA_017995275.1_Rickettsiaceae_bacterium
Orientia_tsutsugamushi_strain_Ikeda_GCA_000010205.1
Orientia_chuto_strain_Dubai_GCA_000964595.1
Occidentia_massiliensis_GCA_000309075.1
Sneabacter_namystus_GCA_008189685.1
Phycorickettsia_trachydisci_GCA_003015145.1
GCA_018062985.1_Rickettsiaceae_bacterium
GCA_013214525.1_Rickettsiaceae_bacterium
GCA_002422875.1_Rickettsiaceae_bacterium
Rickettsiaceae_endosymbiont_of_Amblyomma_cajennese_Ac37b_GCA_000746585.2
Arcanobacter_lacustris_GCA_000970895.1
Rickettsiaceae_endosymbiont_of_Stachyamoeba_lipophora_GCA_003932735.1
GCA_001897445.1_Rickettsiaceae_bacterium
Midichloria_mitochondrii_IricVA_GCA_000219355.1
Cyrtobacter_comes_endosymbiont_of_Euplotes_harpa_BOD18
Lyticum_sinuosum_endosymbiont_of_Paramecium_biaurelia_USBL-36I1
Fokinia_solitaria_GCA_003072485.1
Fokinia_cryptica_endosymbiont_of_Paramecium_biaurelia_US_Bl_11III1
GCA_903878005.1_Midichloriaceae_bacterium
Vederia_obscura_endosymbiont_of_Plagiopyla_frontata_IBS-3
Bandiella_woodruffii_endosymbiont_of_Euplotes_woodruffi_NDG2
GCA_905479795.1_Midichloriaceae_bacterium
GCA_903887235.1_Midichloriaceae_bacterium
Grellia_incantans_H2_GCA_009690945.1
Aquarickettsia_rohweri_GCA_003953955.1
Jidaibacter_acanthamoeba_UWC36_GCA_000815465.1
Endosymbiont_of_Acanthamoeba_sp._UWC8_GCA_000730245.1
Midichloriaceae_endosymbiont_of_Reticulomyxa_filosa
Midichloriaceae_endosymbiont_of_Peranema_trichophorum_GCA_004210275.1
GCA_013288625.1_Midichloriaceae_bacterium
Anaplasma_phagocytophilum_strain_HZ_GCA_000013125.1
Anaplasma_marginale_strain_Florida_GCA_000020305.1
Neoehrlichia_lotoris_strain_RAC413_GCA_000964795.1
Ehrlichia_ruminantium_strain_Welgevonden_GCA_000026005.1
Ehrlichia_chaffeensis_strain_Arkansas_GCA_000013145.1
Wolbachia_endosymbiont_of_Brugia_malayi_GCA_000008385.1
Wolbachia_endosymbiont_of_Drosophila_melanogaster_GCA_000008025.1
Wolbachia_endosymbiont_of_Pratylenchus_penetrans_GCA_001752665.1
Neorickettsia_sennetsu_strain_Miyayama_GCA_000013165.1
Neorickettsia_helminthoeca_strain_Oregon_GCA_000632985.1
Xenolissoclinum_pacificiensis_L6_GCA_000512675.1
Echinorickettsia_raffii_H1_GCA_018101195.1
Anaplasmataceae_symbiont_of_Acropora_tenuis_Sesoko1_GCA_014132315.1
GCA_903864455.1_Anaplasmataceae_bacterium
Deianiraea_vastatrix_GCA_007993655.1
GCA_017444125.1_Deianiraeaceae_bacterium
GCA_017444095.1_Deianiraeaceae_bacterium
GCA_002395105.1_Deianiraeaceae_bacterium
GCA_002394665.1_Deianiraeaceae_bacterium
GCA_905480015.1_Deianiraeaceae_bacterium
GCA_018969185.1_Gamibacteraceae_bacterium
GCA_002359655.1_Gamibacteraceae_bacterium
GCA_009693885.1_Gamibacteraceae_bacterium
GCA_903872175.1_Gamibacteraceae_bacterium
GCA_903831915.1_Gamibacteraceae_bacterium
GCA_017999675.1_Gamibacteraceae_bacterium
GCA_903927415.1_Gamibacteraceae_bacterium
GCA_903878685.1_Gamibacteraceae_bacterium
GCA_903917585.1_Gamibacteraceae_bacterium
GCA_001768015.1_Gamibacteraceae_bacterium
GCA_903837365.1_Gamibacteraceae_bacterium
GCA_014190355.1_Gamibacteraceae_bacterium
GCA_903959715.1_Gamibacteraceae_bacterium
GCA_009925685.1_Gamibacteraceae_bacterium
GCA_009921385.1_Gamibacteraceae_bacterium
GCA_016778745.1_Gamibacteraceae_bacterium
GCA_905478245.1_Gamibacteraceae_bacterium
GCA_905478165.1_Gamibacteraceae_bacterium
GCA_017620845.1_Gamibacteraceae_bacterium
GCA_017475895.1_Gamibacteraceae_bacterium
GCA_016780625.1_Diomedesiaceae_bacterium
GCA_018659605.1_Diomedesiaceae_bacterium
GCA_018659145.1_Diomedesiaceae_bacterium
GCA_016778805.1_Diomedesiaceae_bacterium
GCA_002787635.1_Athabascaceae_bacterium
GCA_002422795.1_Athabascaceae_bacterium
GCA_013288565.1_Jistubacteraceae_bacterium
GCA_903821695.1_Jistubacteraceae_bacterium
GCA_010031735.1_Jistubacteraceae_bacterium
GCA_003531345.1_Jistubacteraceae_bacterium
GCA_013288575.1_Arkhamiaceae_bacterium
GCA_002787615.1_Arkhamiaceae_bacterium
GCA_013289445.1_Mitibacteraceae_bacterium
GCA_013288985.1_Mitibacteraceae_bacterium
GCA_013288335.1_Mitibacteraceae_bacterium
GCA_013288305.1_Mitibacteraceae_bacterium
GCA_009926845.1_Mitibacteraceae_bacterium
GCA_005792635.1_Mitibacteraceae_bacterium
GCA_003241645.1_Mitibacteraceae_bacterium
GCA_002422745.1_Mitibacteraceae_bacterium
GCA_002725445.1_Mitibacteraceae_bacterium
GCA_002422205.1_Mitibacteraceae_bacterium
GCA_002346425.1_Mitibacteraceae_bacterium
GCA_002423155.1_Mitibacteraceae_bacterium
GCA_004293935.1_Mitibacteraceae_bacterium
GCA_002291925.1_Mitibacteraceae_bacterium
GCA_002291845.1_Mitibacteraceae_bacterium
pilA/gspG/gspH
pilB/gspE
pilC/gspF
pilD/gspO
pilF/pilW
pilM/pilN/gspL
pilP/gspC
pilQ/gspD
pilT/pilU
pilY
rpcC/flpB
tadA/flpF
tadB/flpG
tadC/flpH
tadE
tadG/tadL
tadZ/flpE
fimC/papD
fimD/papC/fcfC
CsgB
CsgG
ShlB/FhaC/HecB_family_hemolysin
FhaB/FHA
AlpA
Asp14
P44/Msp2
Sca0/rOmpA
Sca1
Sca2
Sca4
Sca5
internalin
intimin-like
OmpA-like
relA/spoT_(p)ppGpp_synthetase
Busco_s_copy
Gene copies
25
20
15
10
5
0

Supplementary Figure 24: biosynthetic pathways and transporters for nucleotides. Heat-map showing the presence and abundance of biosynthetic pathways of nucleotides (purines and pyrimidines; blue) and their tlc transporters (red) in *Rickettsiales*. For biosynthesis, the proportion of the total genes of the pathway is shown (Supplementary Data 7), while for transporters, the number of genes is reported. Organisms are ordered by their phylogenetic relationships (Supplementary Figure 18), with families highlighted by different colours on the right. The bar plots on the right indicate the percentage of proteobacterial BUSCO single-copy orthogroups identified in each assembly.

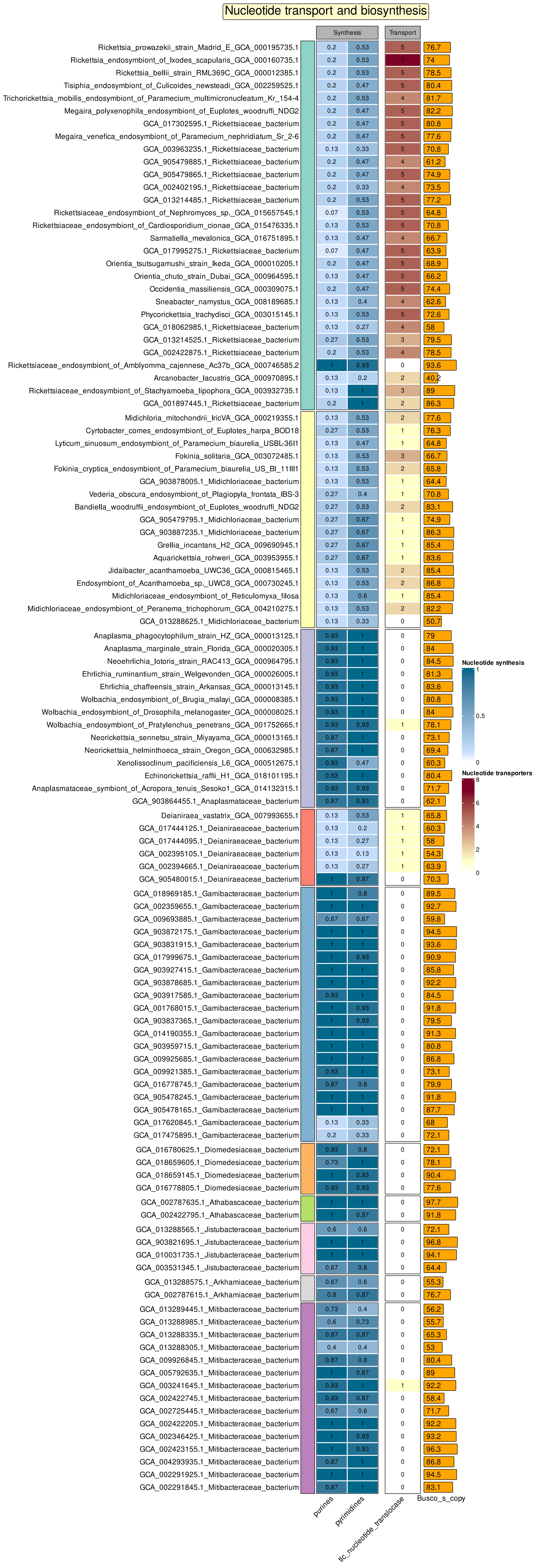

Nucleotide transport and biosynthesis
Synthesis
Transport
Rickettsia_prowazekii_strain_Madrid_E_GCA_000195735.1 0.2 0.53 5 76.7
Rickettsia_endosymbiont_of_Ixodes_scapularis_GCA_000160735.1 0.2 0.53 7 74
Rickettsia_bellii_strain_RML369C_GCA_000012385.1 0.2 0.53 5 78.5
Tisiphia_endosymbiont_of_Culicoides_newsteadi_GCA_002259525.1 0.2 0.47 5 80.4
Trichorickettsia_mobilis_endosymbiont_of_Paramecium_multimicronucleatum_Kr_154-4 0.2 0.53 4 81.7
Megaira_polyxenophila_endosymbiont_of_Euplotes_woodruffi_NDG2 0.2 0.47 5 82.2
GCA_017302595.1_Rickettsiaceae_bacterium 0.2 0.47 5 80.8
Megaira_venefica_endosymbiont_of_Paramecium_nephridiatum_Sr_2-6 0.2 0.47 5 77.6
GCA_003963235.1_Rickettsiaceae_bacterium 0.13 0.33 5 70.8
GCA_905479885.1_Rickettsiaceae_bacterium 0.2 0.47 4 61.2
GCA_905479865.1_Rickettsiaceae_bacterium 0.2 0.47 5 74.9
GCA_002402195.1_Rickettsiaceae_bacterium 0.2 0.33 4 73.5
GCA_013214485.1_Rickettsiaceae_bacterium 0.2 0.53 5 77.2
Rickettsiaceae_endosymbiont_of_Nephromyces_sp._GCA_015657545.1 0.07 0.53 5 64.8
Rickettsiaceae_endosymbiont_of_Cardiosporidium_cionae_GCA_015476335.1 0.13 0.53 5 70.8
Sarmatiella_mevalonica_GCA_016751895.1 0.13 0.47 4 66.7
GCA_017995275.1_Rickettsiaceae_bacterium 0.07 0.47 5 63.9
Orientia_tsutsugamushi_strain_Ikeda_GCA_000010205.1 0.2 0.47 5 68.9
Orientia_chuto_strain_Dubai_GCA_000964595.1 0.13 0.47 5 66.2
Occidentia_massiliensis_GCA_000309075.1 0.2 0.47 5 74.4
Sneabacter_namystus_GCA_008189685.1 0.13 0.4 4 62.6
Phycorickettsia_trachydisci_GCA_003015145.1 0.13 0.53 5 72.6
GCA_018062985.1_Rickettsiaceae_bacterium 0.13 0.27 4 58
GCA_013214525.1_Rickettsiaceae_bacterium 0.27 0.53 3 79.5
GCA_002422875.1_Rickettsiaceae_bacterium 0.2 0.53 4 78.5
Rickettsiaceae_endosymbiont_of_Amblyomma_cajennese_Ac37b_GCA_000746585.2 1 0.93 0 93.6
Arcanobacter_lacustris_GCA_000970895.1 0.13 0.2 2 40.2
Rickettsiaceae_endosymbiont_of_Stachyamoeba_lipophora_GCA_003932735.1 0.13 1 3 89
GCA_001897445.1_Rickettsiaceae_bacterium 0.2 1 2 86.3
Midichloria_mitochondrii_IricVA_GCA_000219355.1 0.13 0.53 2 77.6
Cyrtobacter_comes_endosymbiont_of_Euplotes_harpa_BOD18 0.27 0.53 1 76.3
Lyticum_sinuosum_endosymbiont_of_Paramecium_biaurelia_USBL-36I1 0.13 0.47 1 64.8
Fokinia_solitaria_GCA_003072485.1 0.13 0.53 3 66.7
Fokinia_cryptica_endosymbiont_of_Paramecium_biaurelia_US_Bl_11III1 0.13 0.53 2 65.8
GCA_903878005.1_Midichloriaceae_bacterium 0.13 0.53 1 64.4
Vederia_obscura_endosymbiont_of_Plagiopyla_frontata_IBS-3 0.27 0.4 1 70.8
Bandiella_woodruffii_endosymbiont_of_Euplotes_woodruffi_NDG2 0.27 0.53 2 83.1
GCA_905479795.1_Midichloriaceae_bacterium 0.27 0.67 1 74.9
GCA_903887235.1_Midichloriaceae_bacterium 0.27 0.67 1 86.3
Grellia_incantans_H2_GCA_009690945.1 0.27 0.67 1 85.4
Aquarickettsia_rohweri_GCA_003953955.1 0.27 0.67 1 83.6
Jidaibacter_acanthamoeba_UWC36_GCA_000815465.1 0.13 0.53 2 85.4
Endosymbiont_of_Acanthamoeba_sp._UWC8_GCA_000730245.1 0.13 0.53 2 86.8
Midichloriaceae_endosymbiont_of_Reticulomyxa_filosa 0.13 0.6 1 85.4
Midichloriaceae_endosymbiont_of_Peranema_trichophorum_GCA_004210275.1 0.13 0.53 2 82.2
GCA_013288625.1_Midichloriaceae_bacterium 0.13 0.33 0 50.7
Anaplasma_phagocytophilum_strain_HZ_GCA_000013125.1 0.93 1 0 79
Anaplasma_marginale_strain_Florida_GCA_000020305.1 0.93 1 0 84
Neoehrlichia_lotoris_strain_RAC413_GCA_000964795.1 0.93 1 0 84.5
Ehrlichia_ruminantium_strain_Welgevonden_GCA_000026005.1 0.93 1 0 81.3
Ehrlichia_chaffeensis_strain_Arkansas_GCA_000013145.1 0.93 1 0 83.6
Wolbachia_endosymbiont_of_Brugia_malayi_GCA_000008385.1 0.93 1 0 80.8
Wolbachia_endosymbiont_of_Drosophila_melanogaster_GCA_000008025.1 0.93 1 0 84
Wolbachia_endosymbiont_of_Pratylenchus_penetrans_GCA_001752665.1 0.93 0.93 1 78.1
Neorickettsia_sennetsu_strain_Miyayama_GCA_000013165.1 0.87 1 0 73.1
Neorickettsia_helminthoeca_strain_Oregon_GCA_000632985.1 0.87 1 0 69.4
Xenolissoclinum_pacificiensis_L6_GCA_000512675.1 0.93 0.47 0 60.3
Echinorickettsia_raffii_H1_GCA_018101195.1 0.93 1 0 80.4
Anaplasmataceae_symbiont_of_Acropora_tenuis_Sesoko1_GCA_014132315.1 0.93 0.93 0 71.7
GCA_903864455.1_Anaplasmataceae_bacterium 0.87 0.93 0 62.1
Deianiraea_vastatrix_GCA_007993655.1 0.13 0.53 1 65.8
GCA_017444125.1_Deianiraeaceae_bacterium 0.13 0.2 1 60.3
GCA_017444095.1_Deianiraeaceae_bacterium 0.13 0.27 1 58
GCA_002395105.1_Deianiraeaceae_bacterium 0.13 0.13 1 54.3
GCA_002394665.1_Deianiraeaceae_bacterium 0.13 0.27 1 63.9
GCA_905480015.1_Deianiraeaceae_bacterium 1 0.87 0 70.3
GCA_018969185.1_Gamibacteraceae_bacterium 1 0.8 0 89.5
GCA_002359655.1_Gamibacteraceae_bacterium 1 1 0 92.7
GCA_009693885.1_Gamibacteraceae_bacterium 0.67 0.67 0 59.8
GCA_903872175.1_Gamibacteraceae_bacterium 1 1 0 94.5
GCA_903831915.1_Gamibacteraceae_bacterium 1 1 0 93.6
GCA_017999675.1_Gamibacteraceae_bacterium 1 0.93 0 90.9
GCA_903927415.1_Gamibacteraceae_bacterium 1 1 0 85.8
GCA_903878685.1_Gamibacteraceae_bacterium 1 1 0 92.2
GCA_903917585.1_Gamibacteraceae_bacterium 0.93 1 0 84.5
GCA_001768015.1_Gamibacteraceae_bacterium 1 0.93 0 91.8
GCA_903837365.1_Gamibacteraceae_bacterium 1 0.93 0 79.5
GCA_014190355.1_Gamibacteraceae_bacterium 1 1 0 91.3
GCA_903959715.1_Gamibacteraceae_bacterium 1 1 0 80.8
GCA_009925685.1_Gamibacteraceae_bacterium 1 1 0 86.8
GCA_009921385.1_Gamibacteraceae_bacterium 0.93 1 0 73.1
GCA_016778745.1_Gamibacteraceae_bacterium 0.87 0.8 0 79.9
GCA_905478245.1_Gamibacteraceae_bacterium 1 1 0 91.8
GCA_905478165.1_Gamibacteraceae_bacterium 1 1 0 87.7
GCA_017620845.1_Gamibacteraceae_bacterium 0.13 0.33 0 68
GCA_017475895.1_Gamibacteraceae_bacterium 0.2 0.33 0 72.1
GCA_016780625.1_Diomedesiaceae_bacterium 0.93 0.8 0 72.1
GCA_018659605.1_Diomedesiaceae_bacterium 0.73 1 0 78.1
GCA_018659145.1_Diomedesiaceae_bacterium 1 0.93 0 90.4
GCA_016778805.1_Diomedesiaceae_bacterium 0.93 0.93 0 77.6
GCA_002787635.1_Athabascaceae_bacterium 1 1 0 97.7
GCA_002422795.1_Athabascaceae_bacterium 1 0.87 0 91.8
GCA_013288565.1_Jistubacteraceae_bacterium 0.6 0.6 0 72.1
GCA_903821695.1_Jistubacteraceae_bacterium 1 1 0 96.8
GCA_010031735.1_Jistubacteraceae_bacterium 1 1 0 94.1
GCA_003531345.1_Jistubacteraceae_bacterium 0.67 0.8 0 64.4
GCA_013288575.1_Arkhamiaceae_bacterium 0.67 0.6 0 55.3
GCA_002787615.1_Arkhamiaceae_bacterium 0.8 0.87 0 76.7
GCA_013289445.1_Mitibacteraceae_bacterium 0.73 0.4 0 56.2
GCA_013288985.1_Mitibacteraceae_bacterium 0.6 0.73 0 55.7
GCA_013288335.1_Mitibacteraceae_bacterium 0.87 0.87 0 65.3
GCA_013288305.1_Mitibacteraceae_bacterium 0.4 0.4 0 53
GCA_009926845.1_Mitibacteraceae_bacterium 0.87 0.8 0 80.4
GCA_005792635.1_Mitibacteraceae_bacterium 1 0.87 0 89
GCA_003241645.1_Mitibacteraceae_bacterium 0.93 1 1 92.2
GCA_002422745.1_Mitibacteraceae_bacterium 0.93 0.87 0 58.4
GCA_002725445.1_Mitibacteraceae_bacterium 0.67 0.6 0 71.7
GCA_002422205.1_Mitibacteraceae_bacterium 1 1 0 92.2
GCA_002346425.1_Mitibacteraceae_bacterium 1 0.93 0 93.2
GCA_002423155.1_Mitibacteraceae_bacterium 1 0.93 0 96.3
GCA_004293935.1_Mitibacteraceae_bacterium 0.87 1 0 86.8
GCA_002291925.1_Mitibacteraceae_bacterium 1 1 0 94.5
GCA_002291845.1_Mitibacteraceae_bacterium 0.87 1 0 83.1
purines
pyrimidines
tlc_nucleotide_translocase
Busco_s_copy
Nucleotide synthesis
1
0.5
0
Nucleotide transporters
8
6
4
2
0

Supplementary Figure 25: single-gene phylogenies for nucleotide and amino acid biosynthesis. Phylogenetic trees of the singles genes of biosynthetic pathways for nucleotides and amino acids (Supplementary Data 7). On each tree, the members of each *Rickettsiales* family are highlighted by different background colours. On each branch, support values by SH-aLRT with 1000 replicates and by 1000 ultrafast bootstraps are reported. The tree scales stand for estimated sequence divergences.

pyrimidine/01_carA_1MUB9@1224

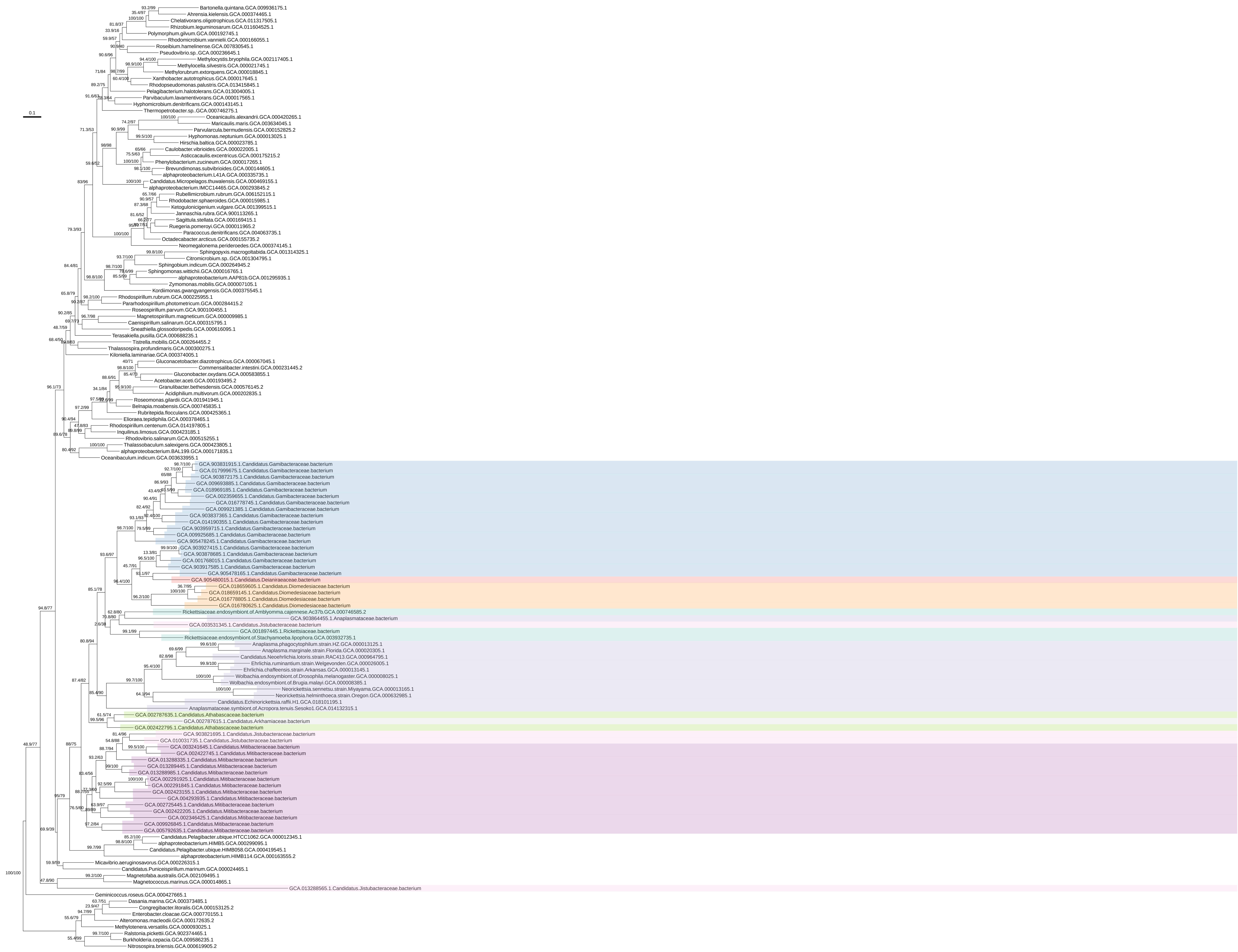

02_carB_COG0458@1

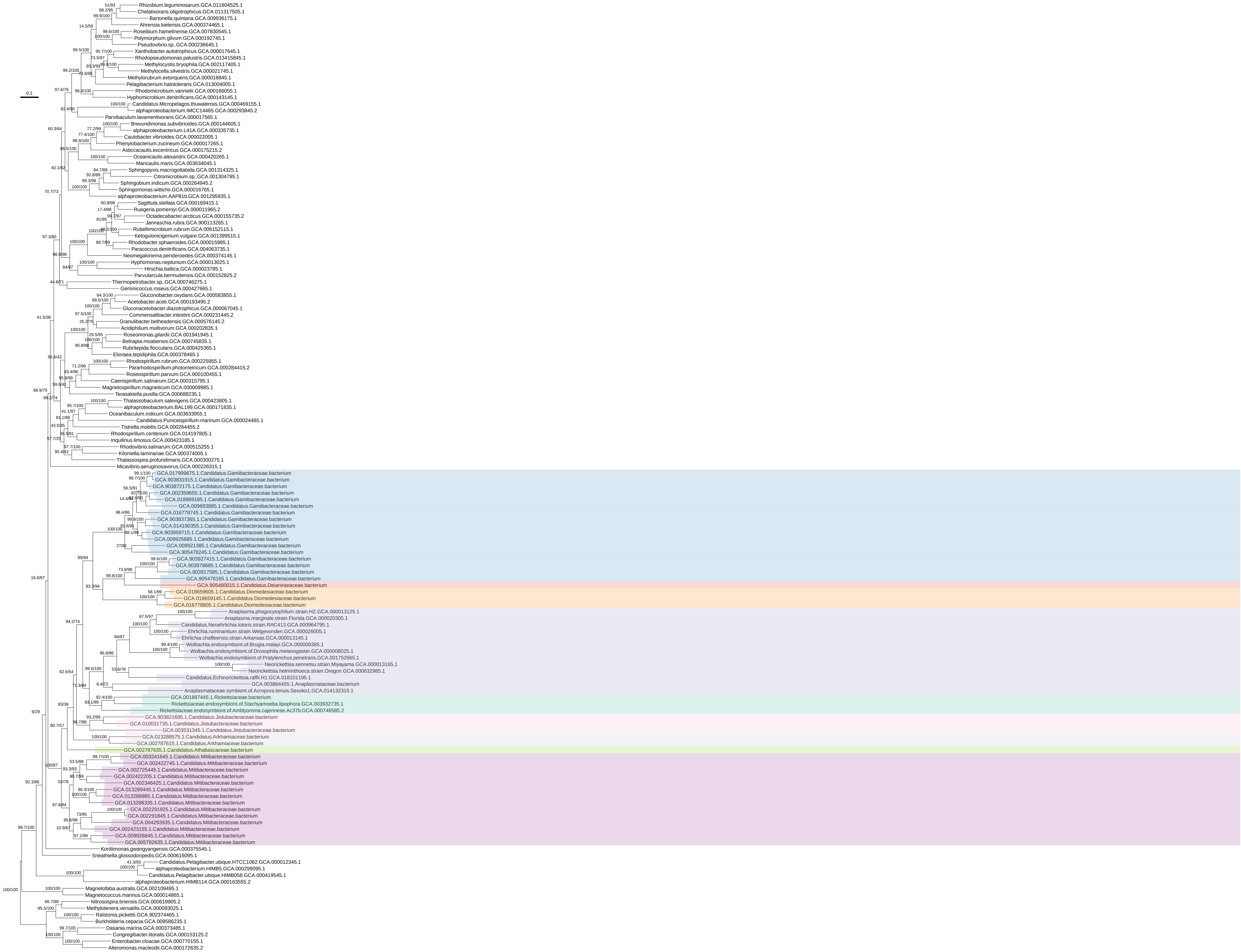

pyrimidine/03_pyrB_COG0540@1

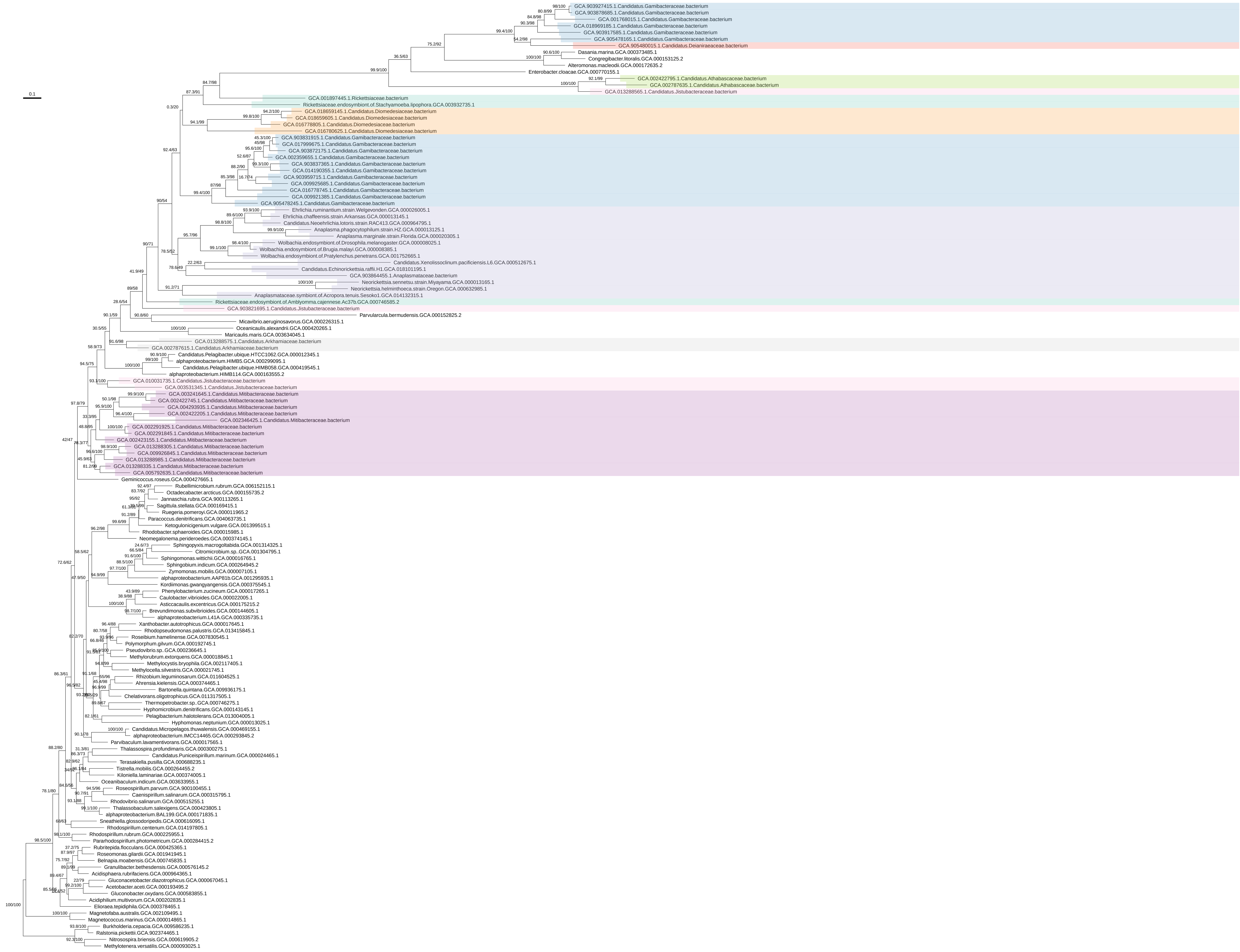

pyrimidine/04_pyrC_COG0044@1

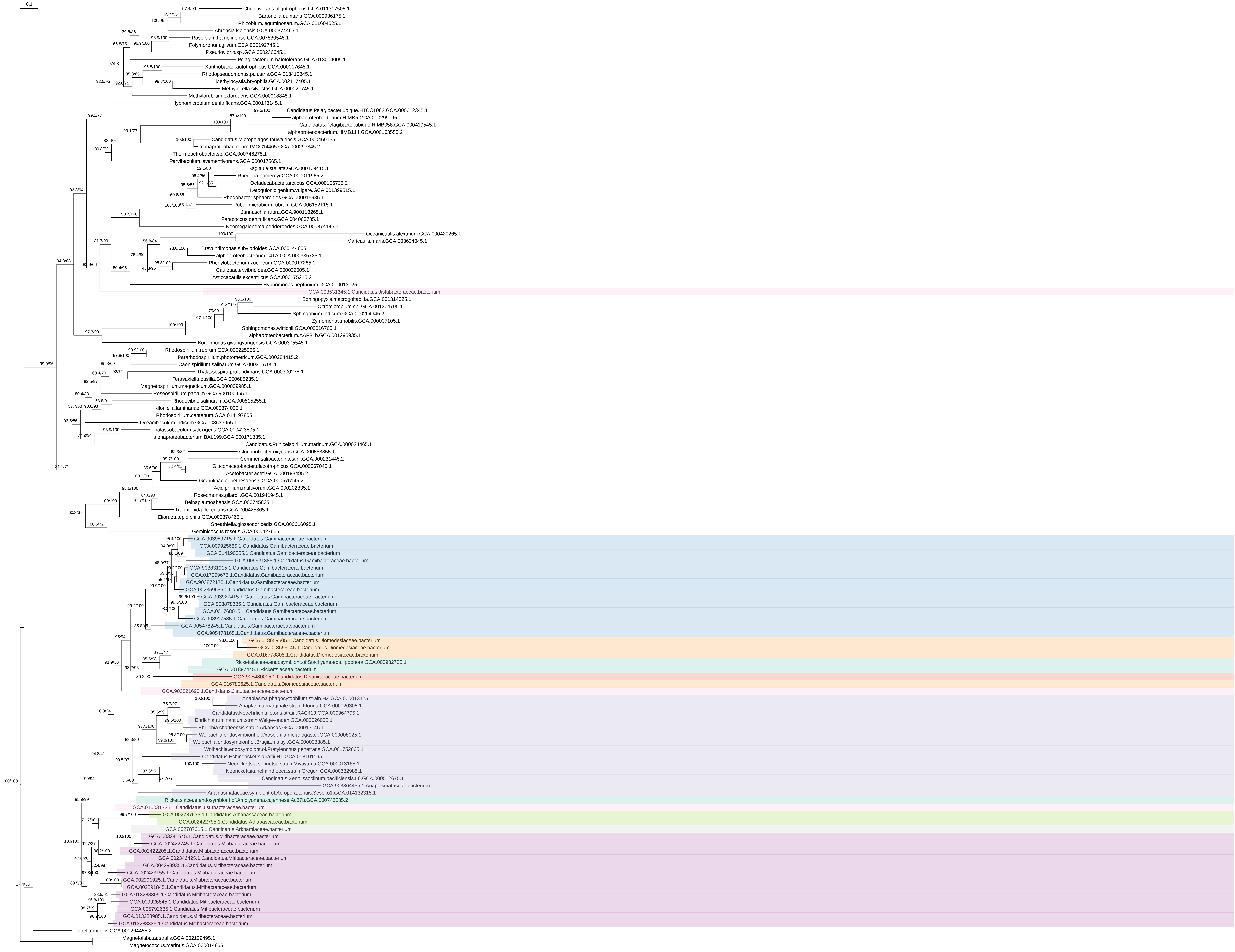

pyrimidine/05_pyrD_COG0167@1

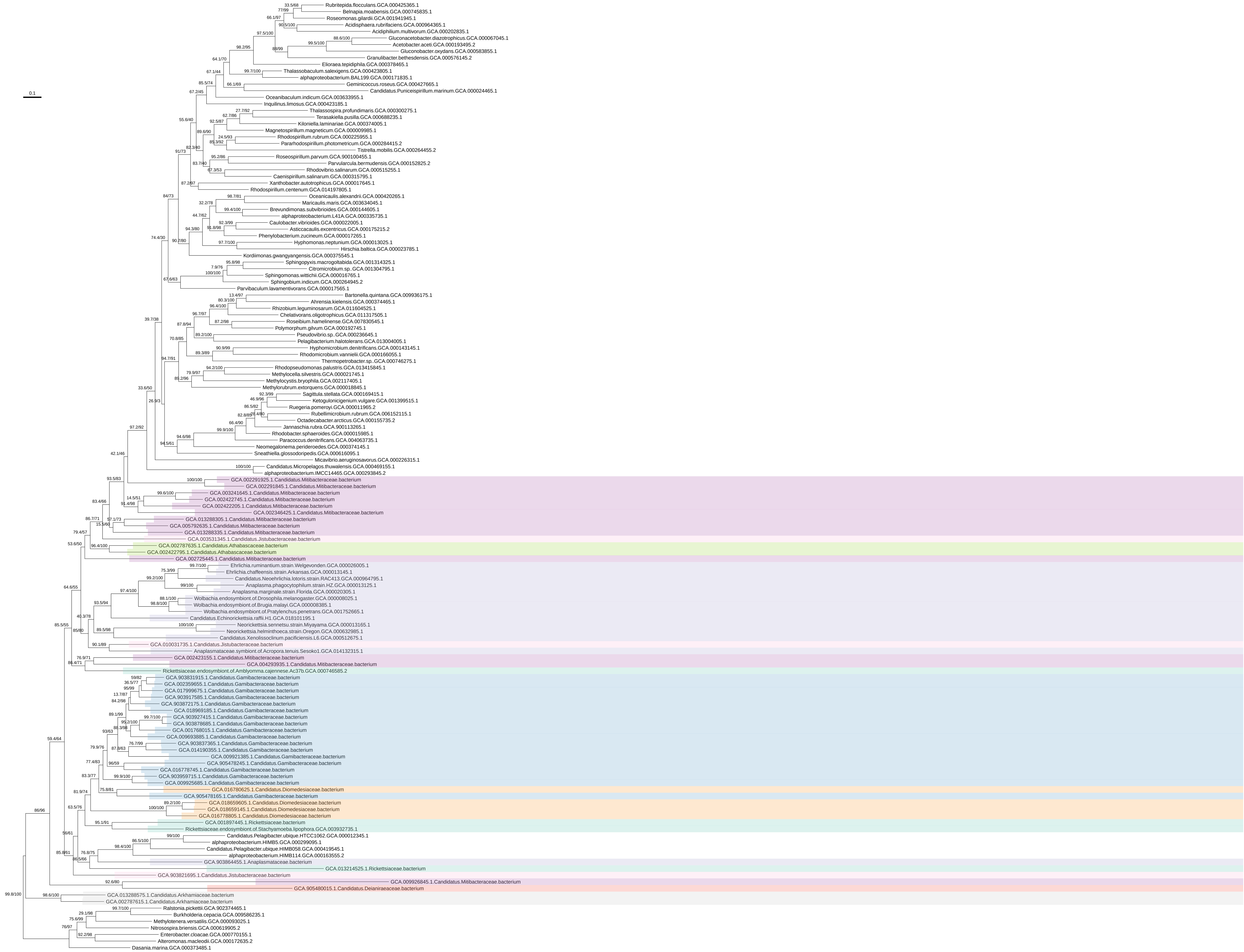

pyrimidine/06_pyrE_COG0461@1

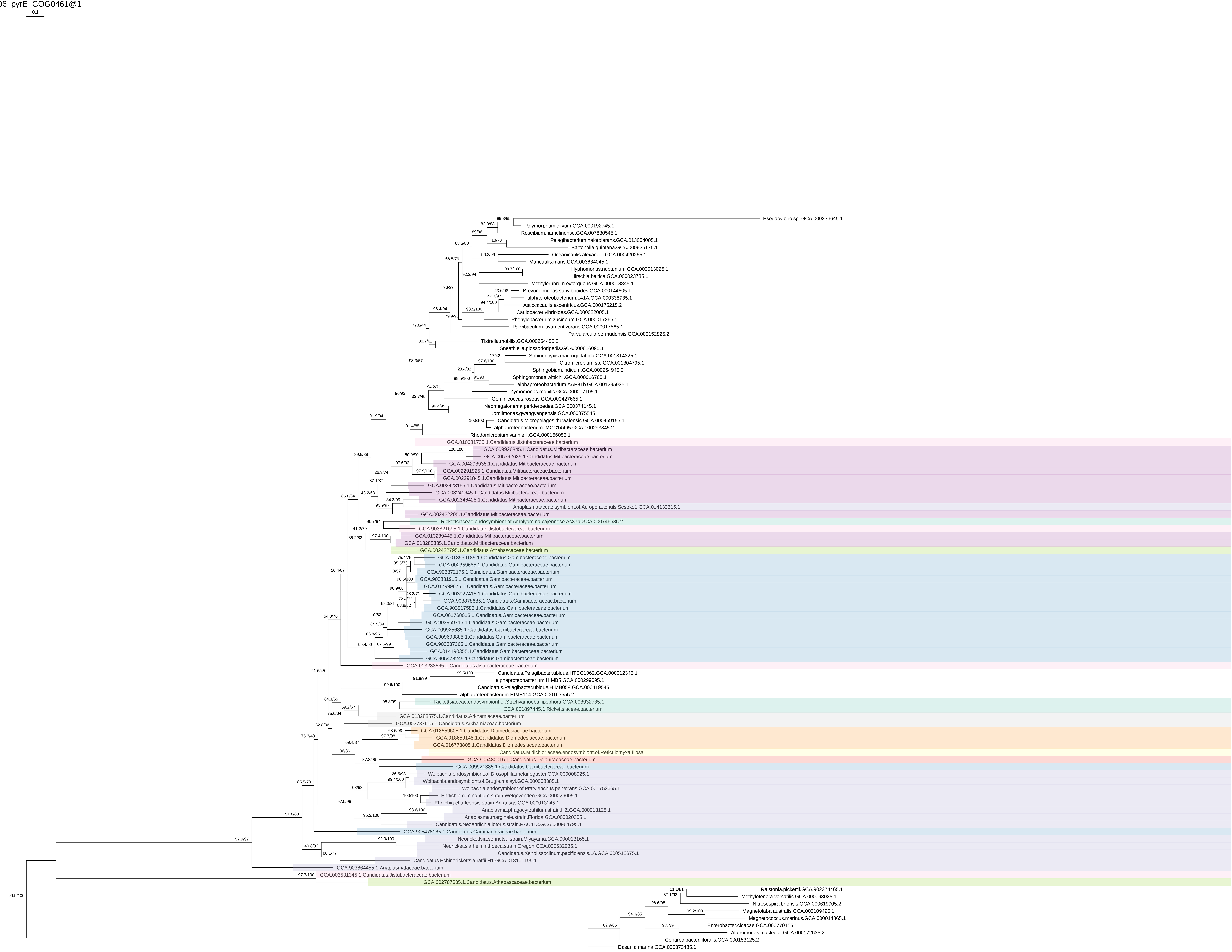

pyrimidine/07_pyrF_1MW2C@1224

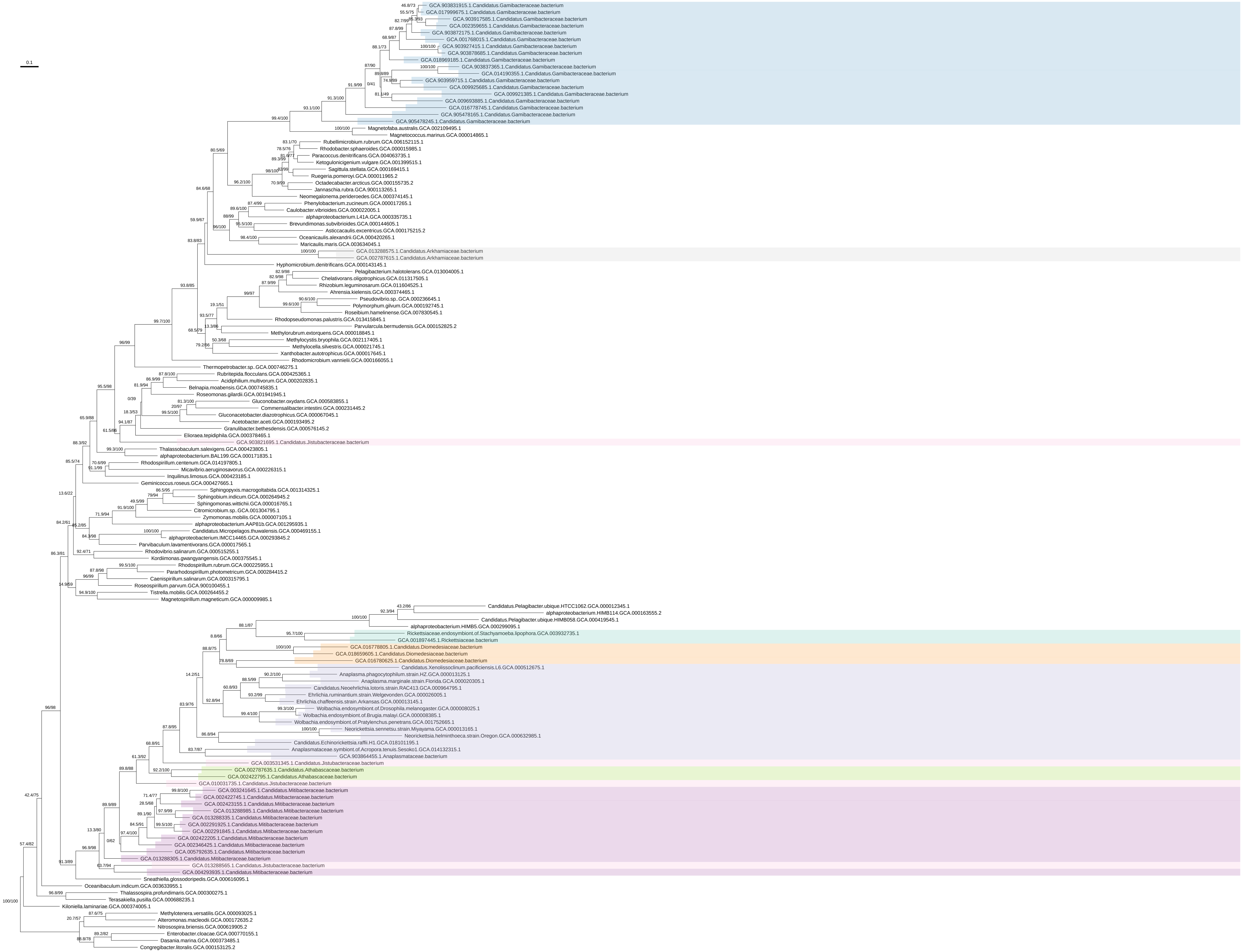

pyrimidine/08_pyrH_COG0528@2

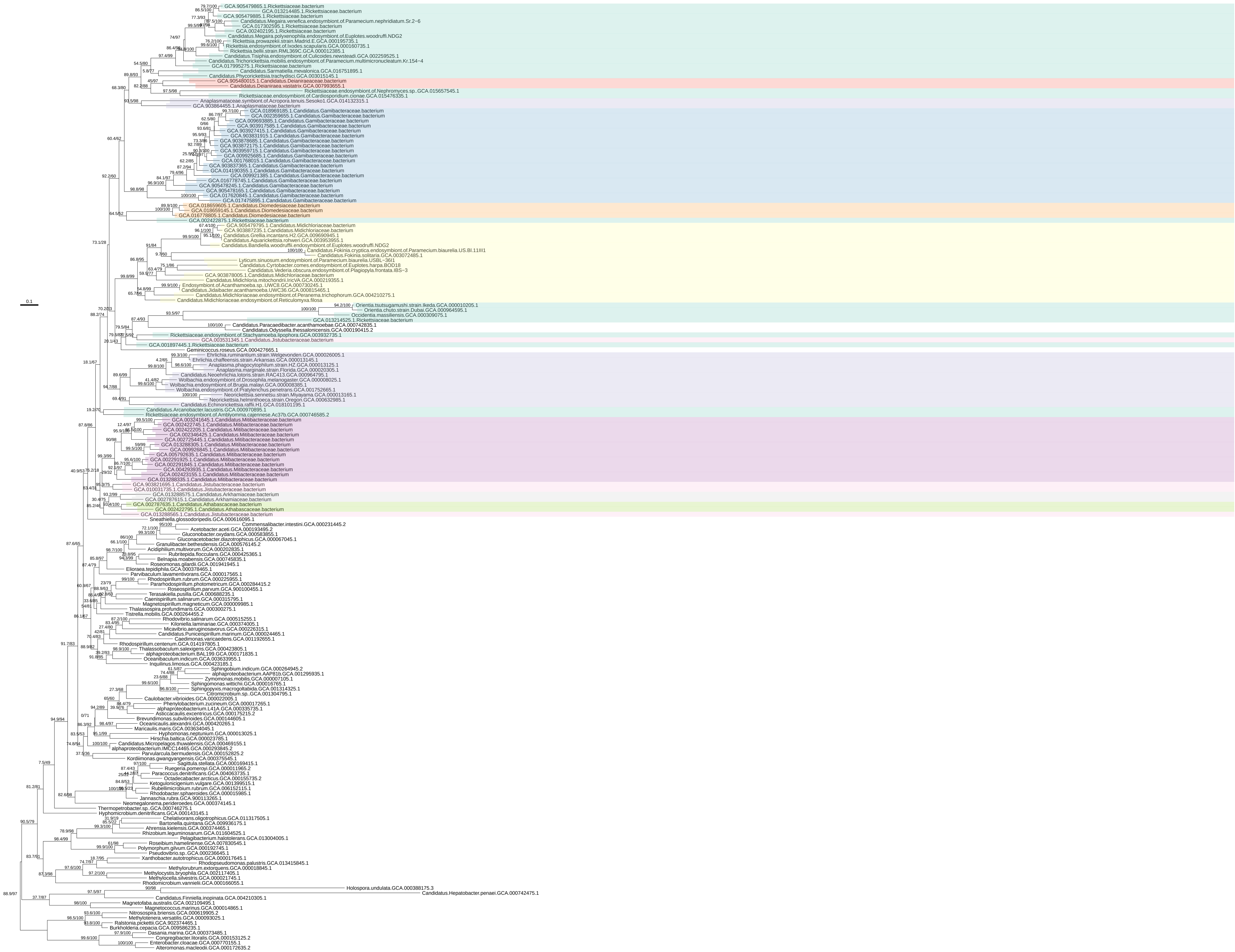

pyrimidine/09_ndk_COG0105@1

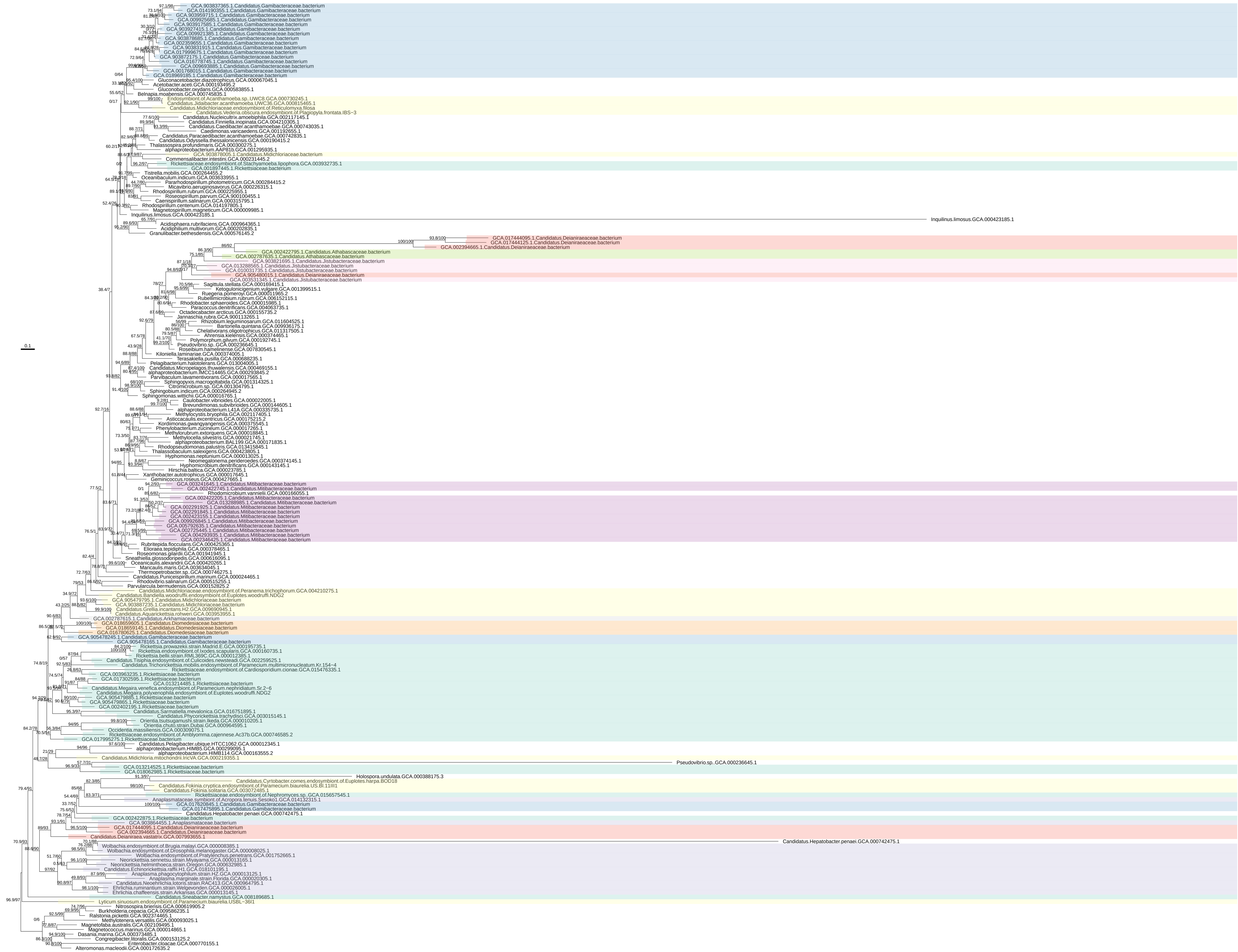

pyrimidine/10_pyrG_COG0504@2

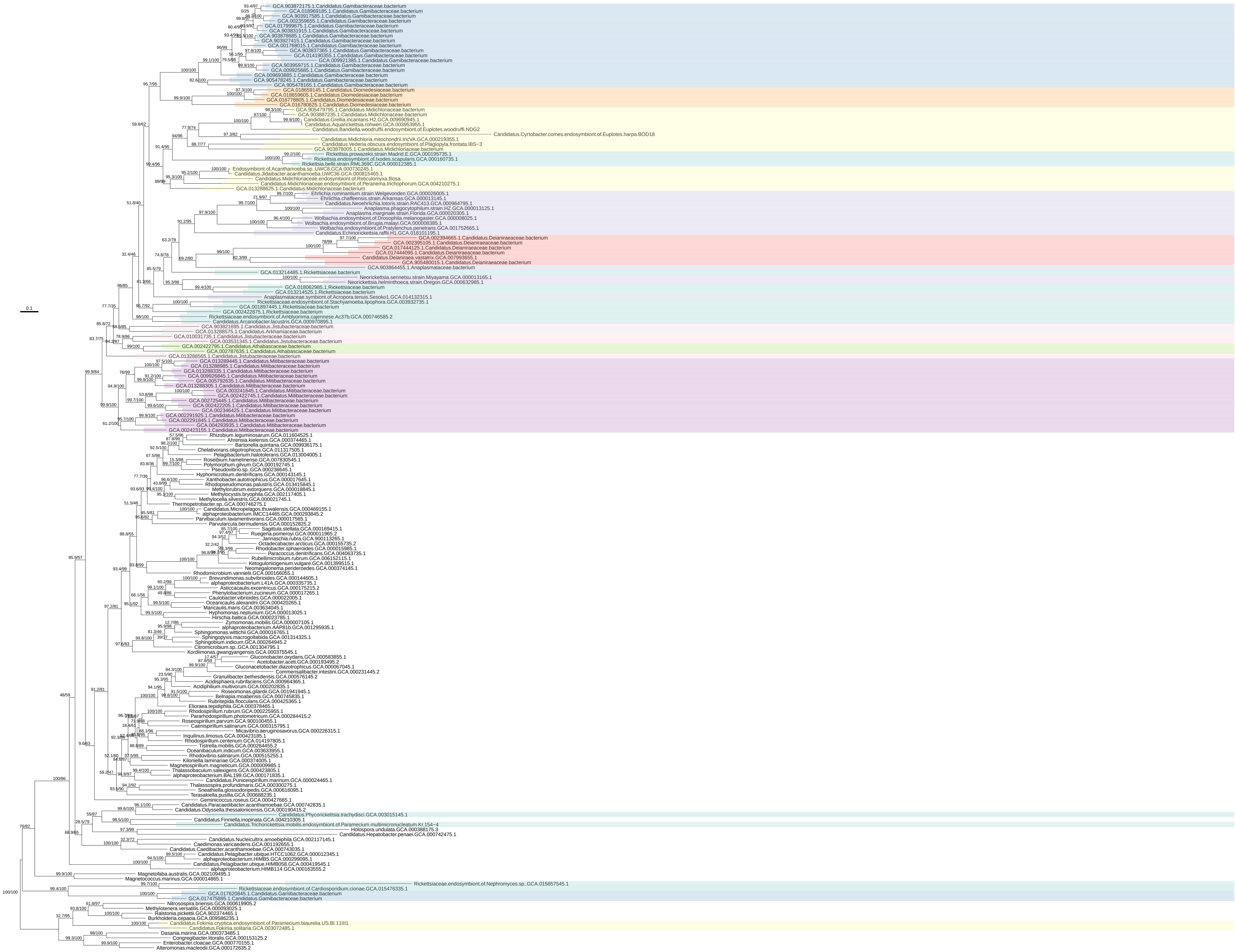

pyrimidine/11_nrdA_COG0209@1

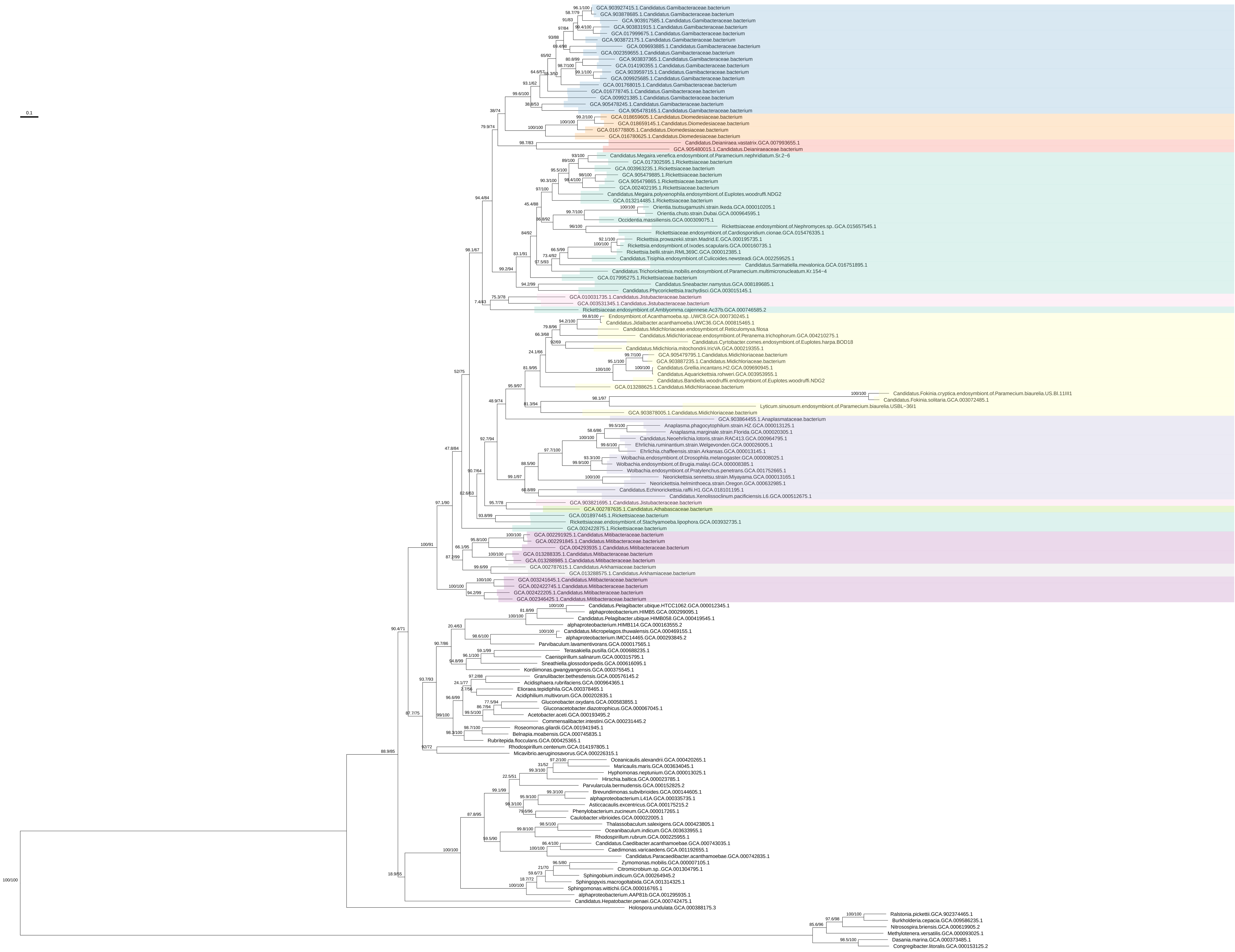

pyrimidine/12_nrdB_COG0208@2

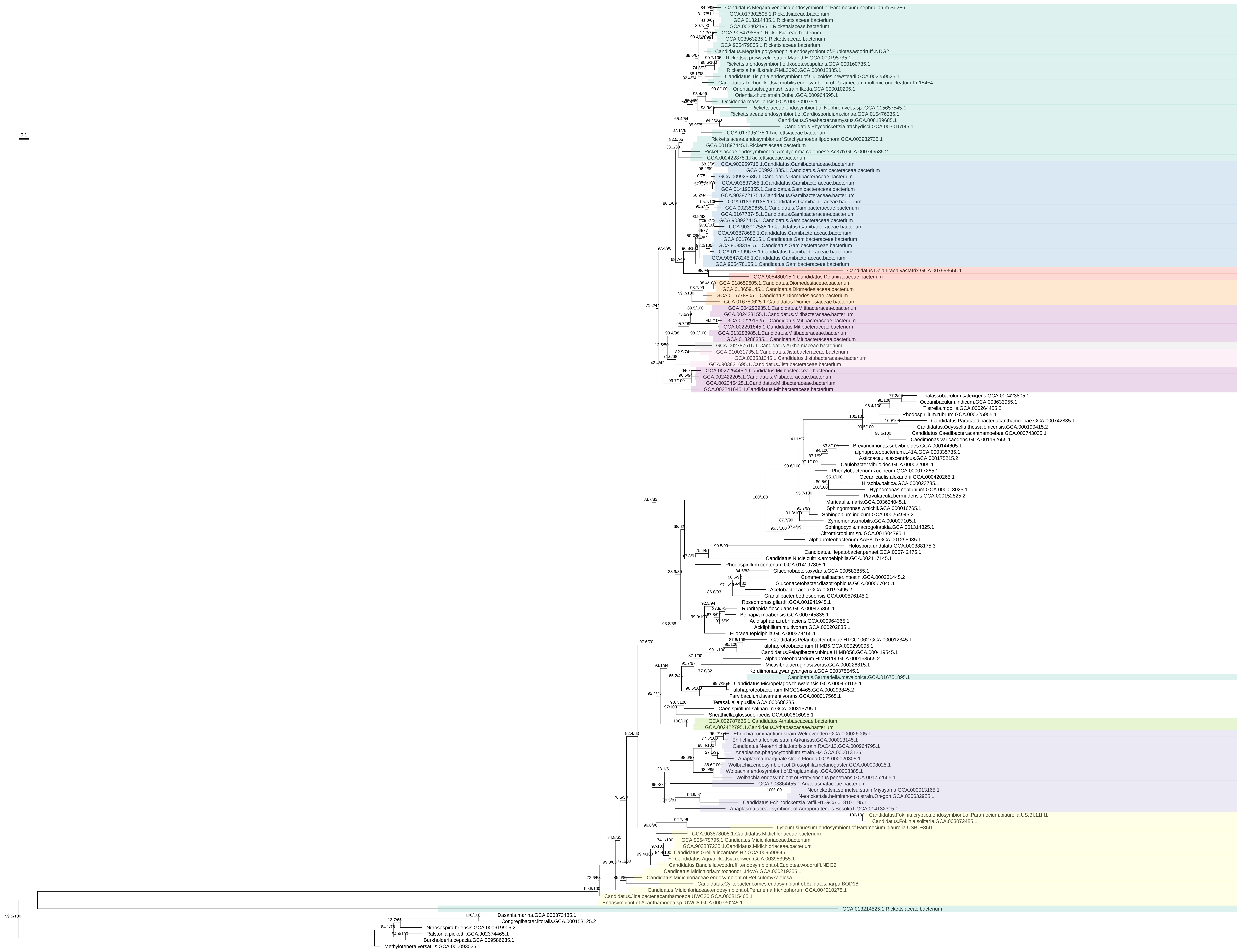

pyrimidine/13_dut_COG0756@1

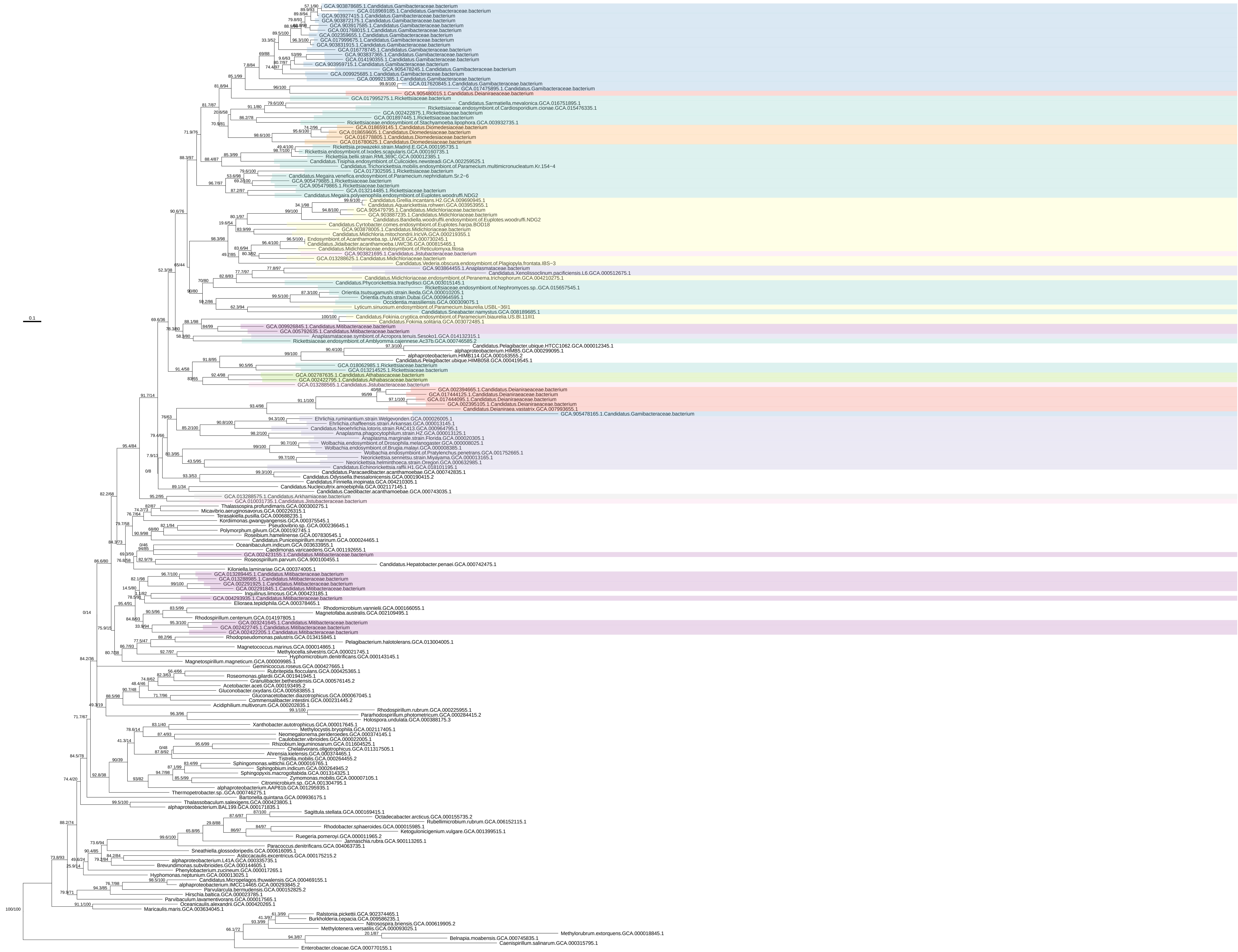

pyrimidine/14_thiX_COG1351@2

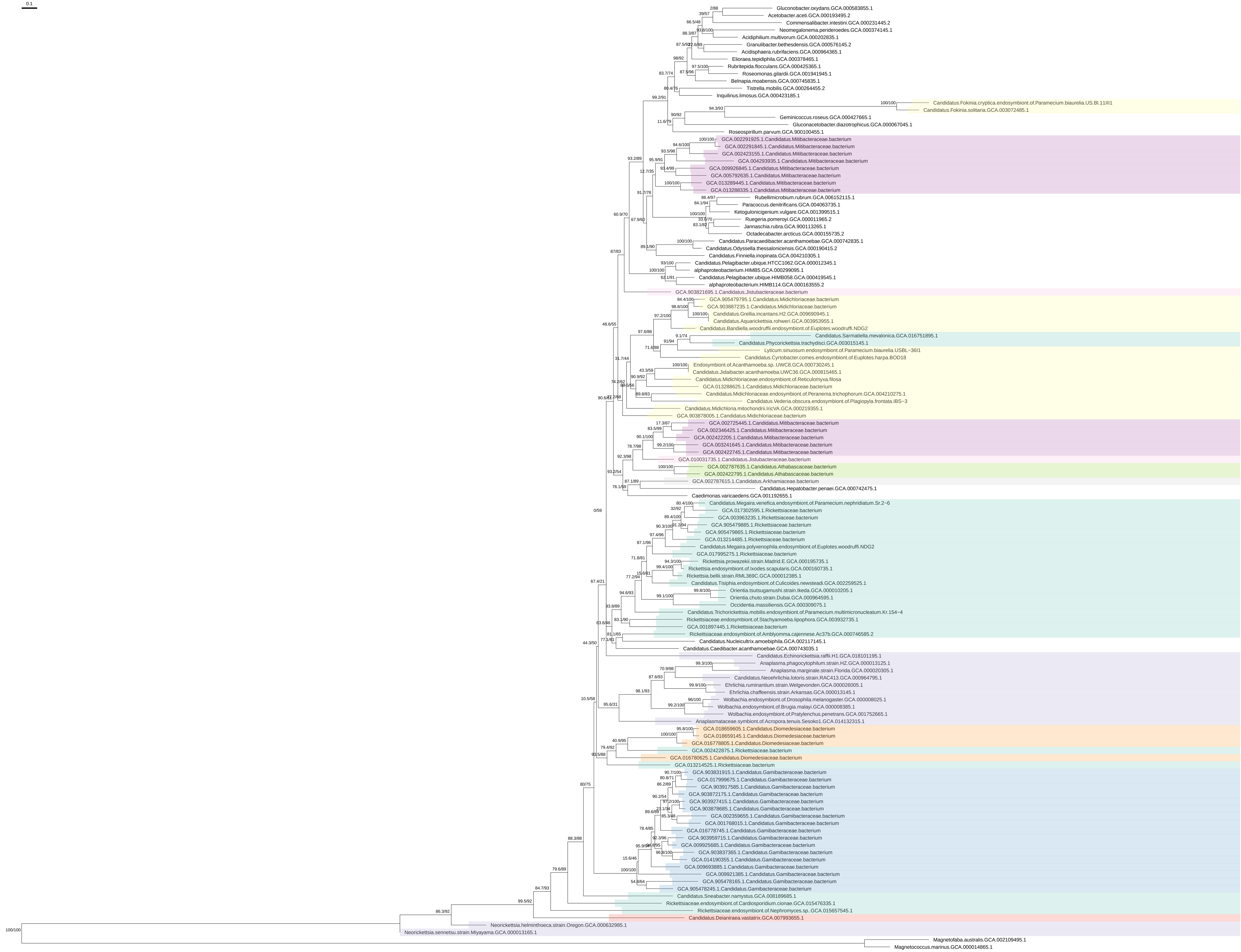

pyrimidine/15_tmk_COG0125@2

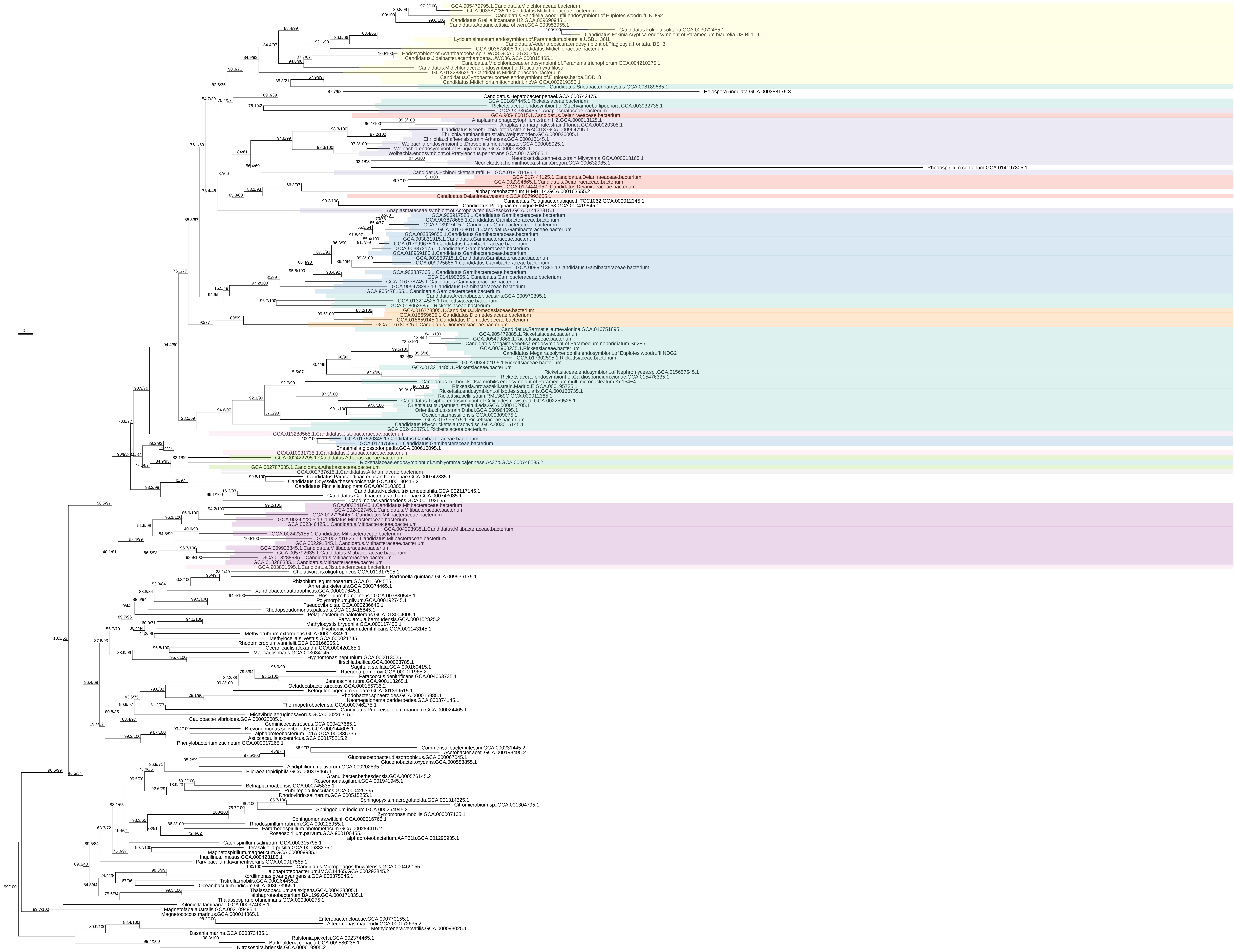

purine/01_purF_1MU0V@1224

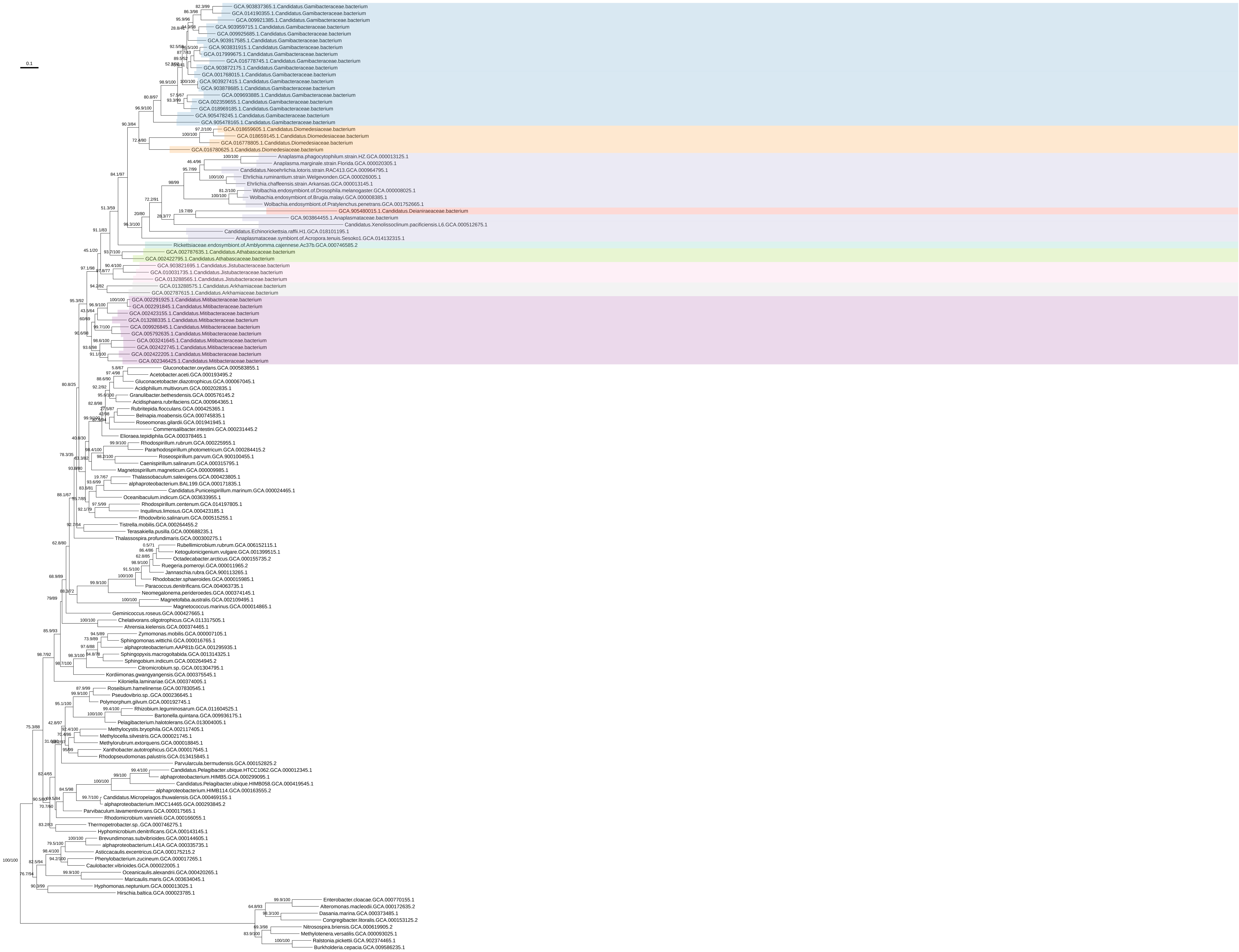

purine/02_purD_COG0151@2

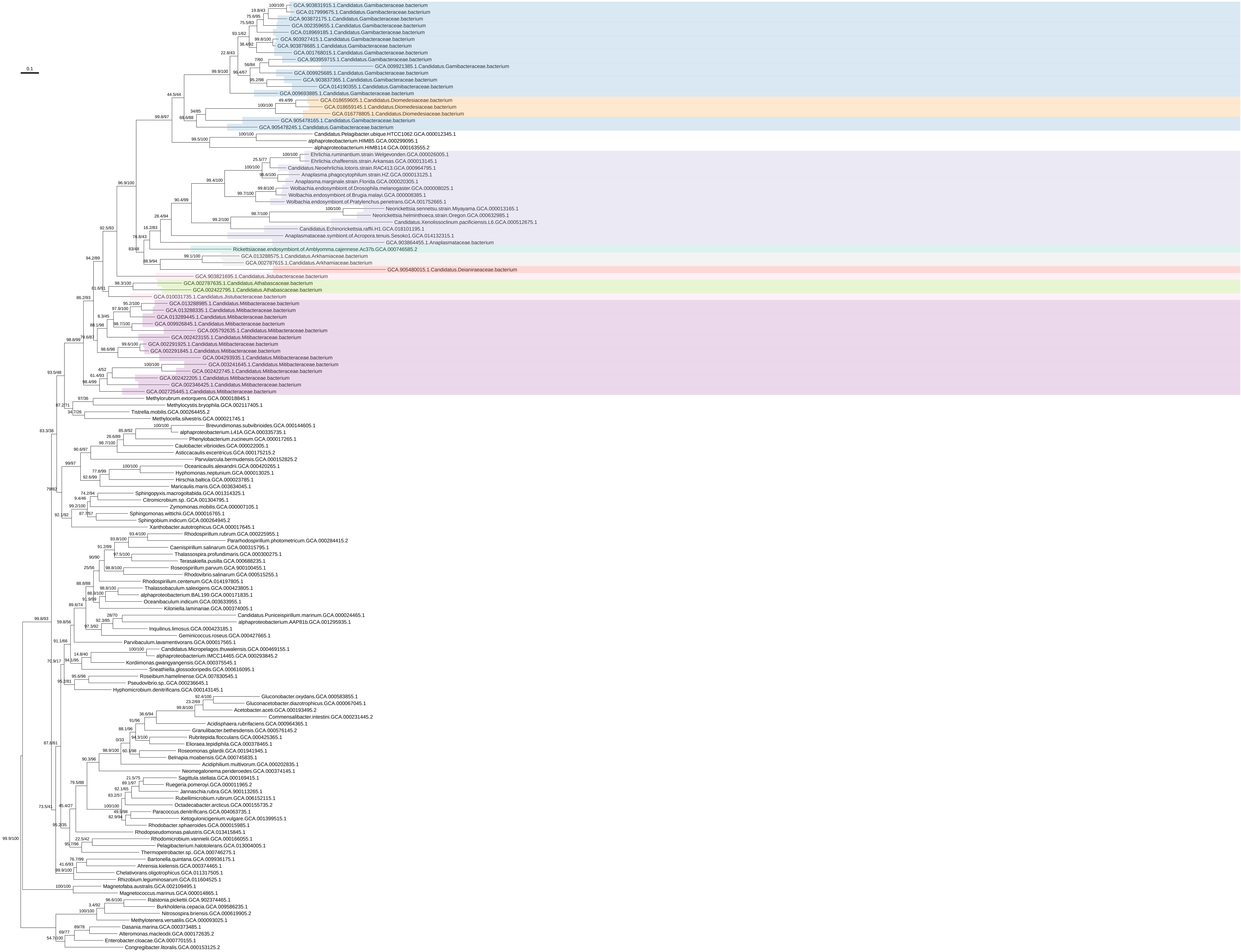

purine/03_purN_COG0299@1

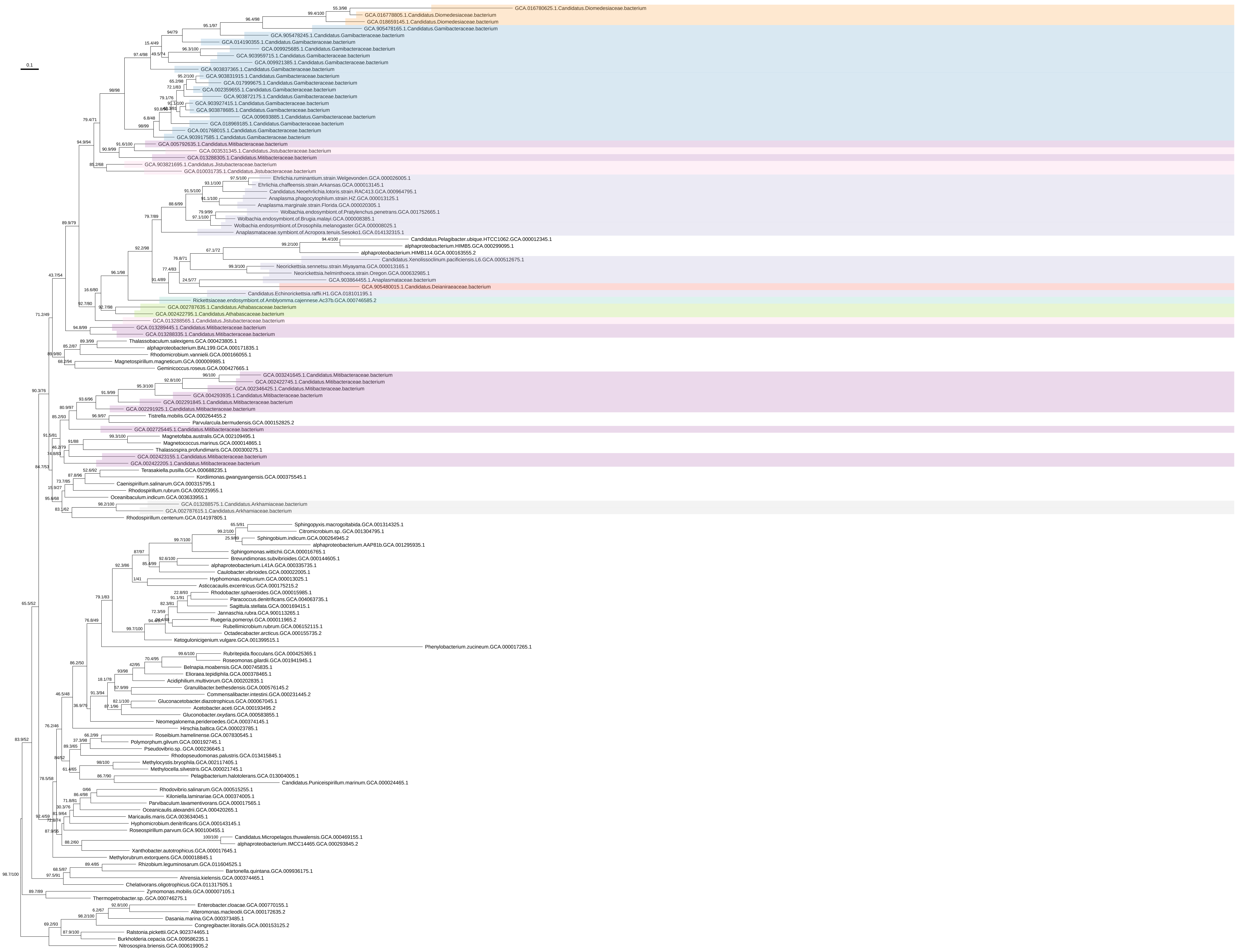

purine/04_purL_COG0046@2

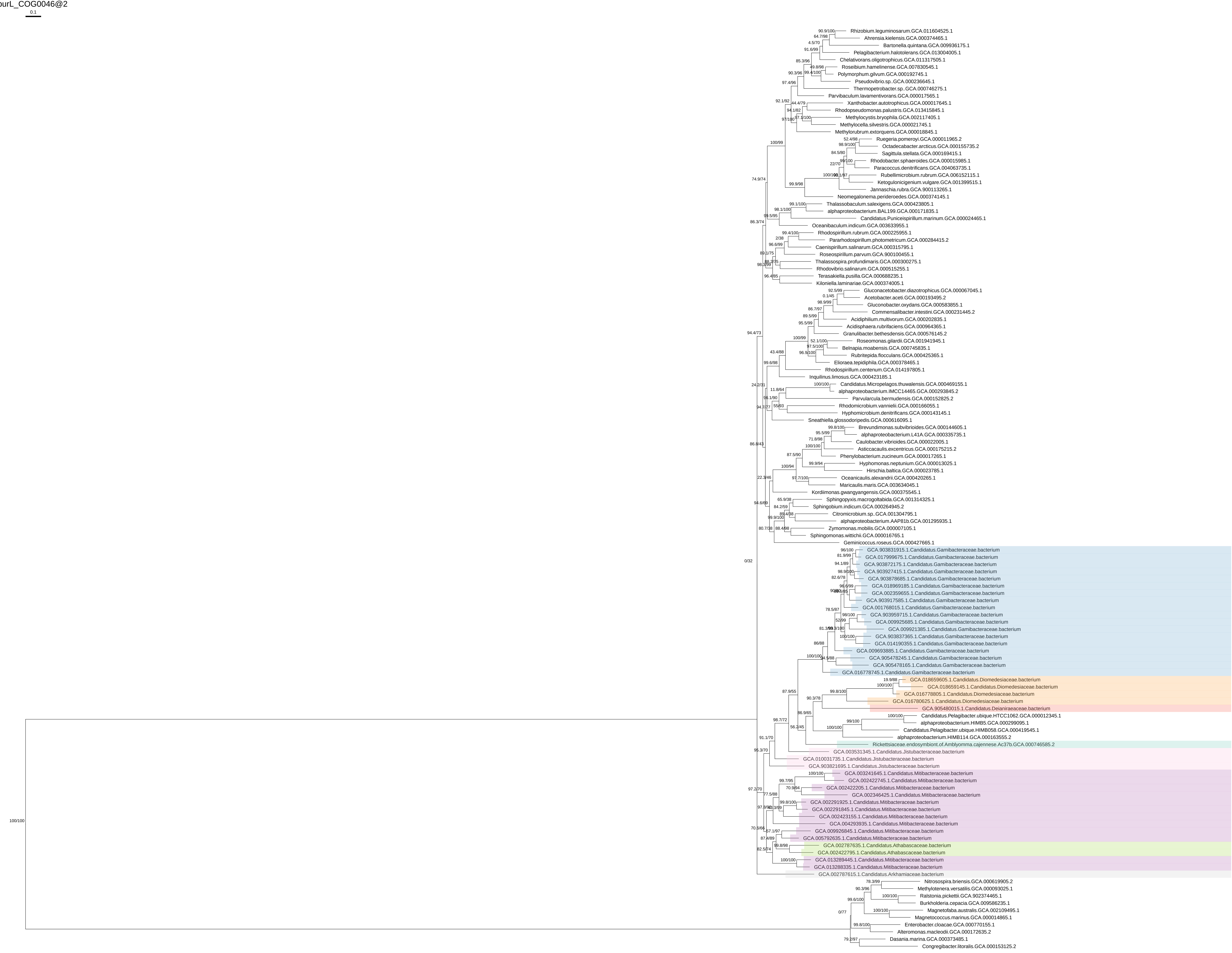

purine/05_purM_COG0150@2

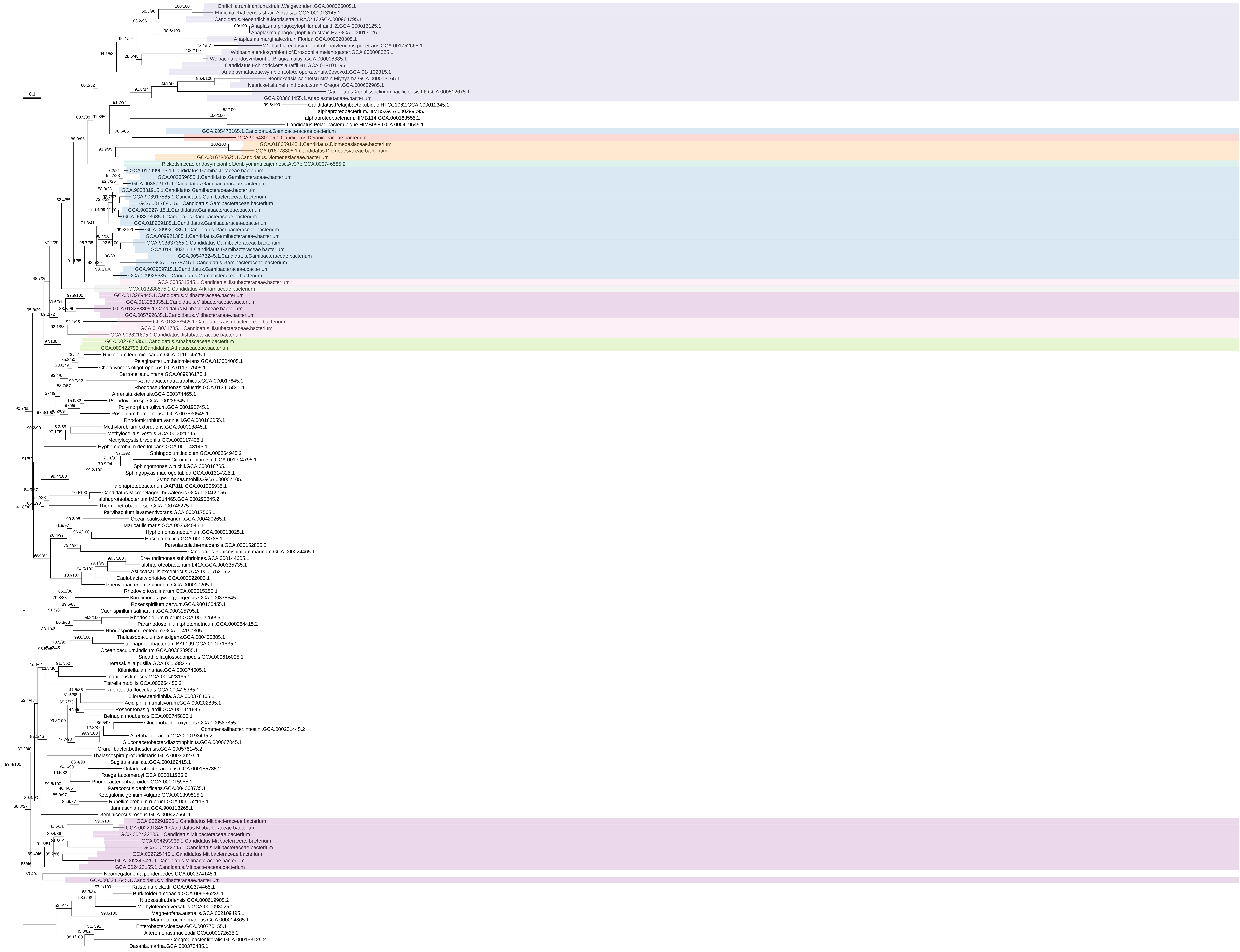

purine/06_purK_1MU70@1224

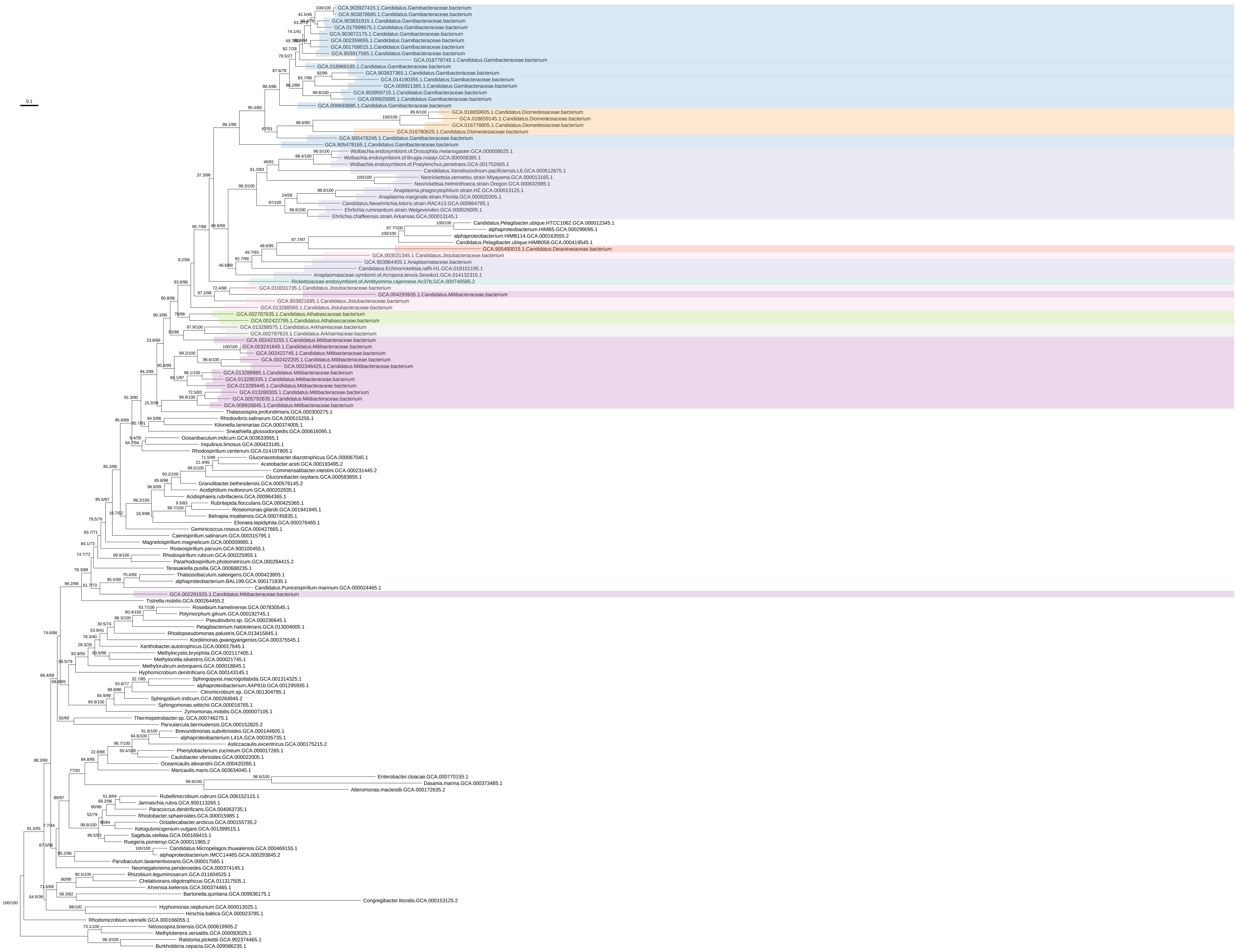

purine/07_purE_COG0041@2

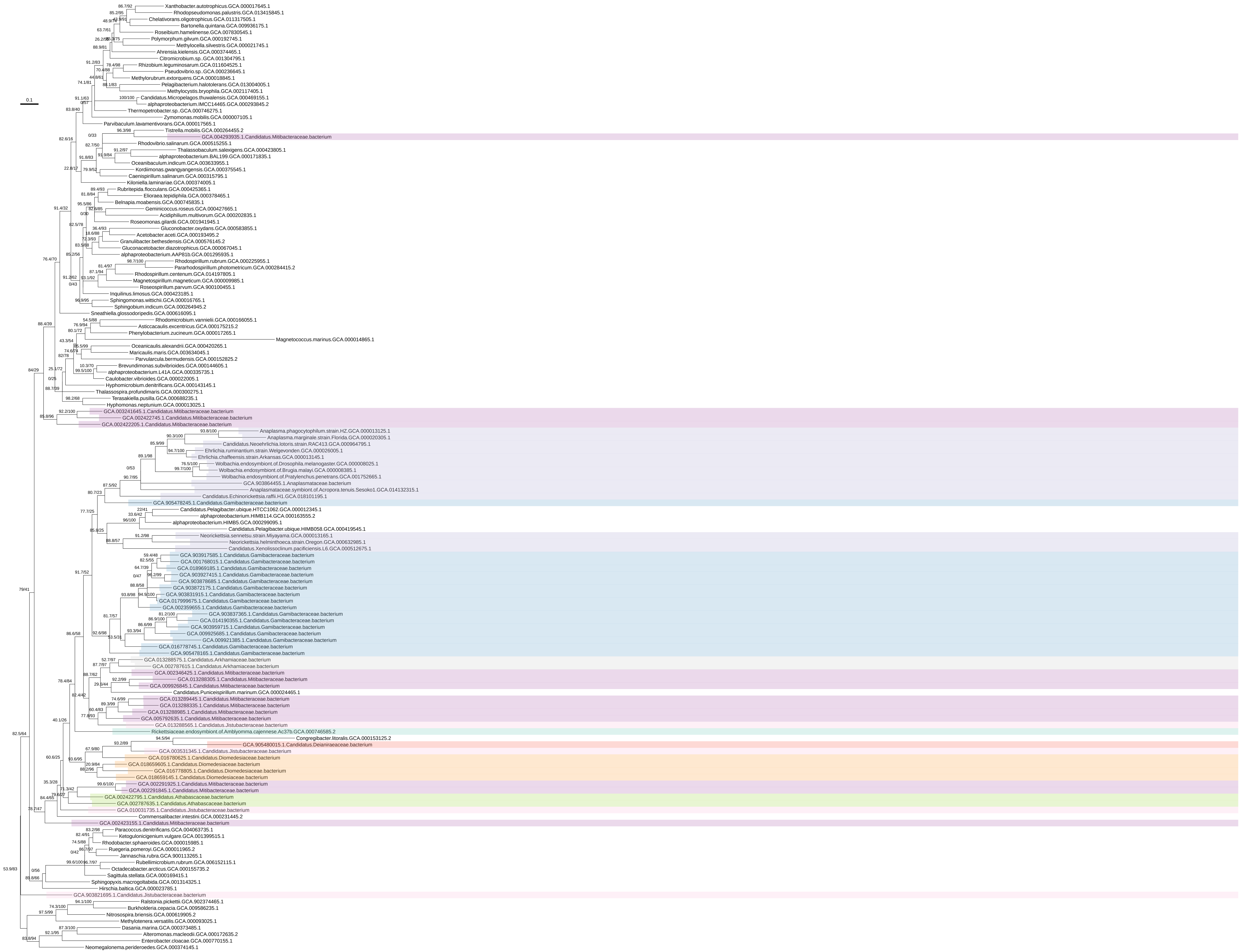

purine/08_purC_COG0152@1

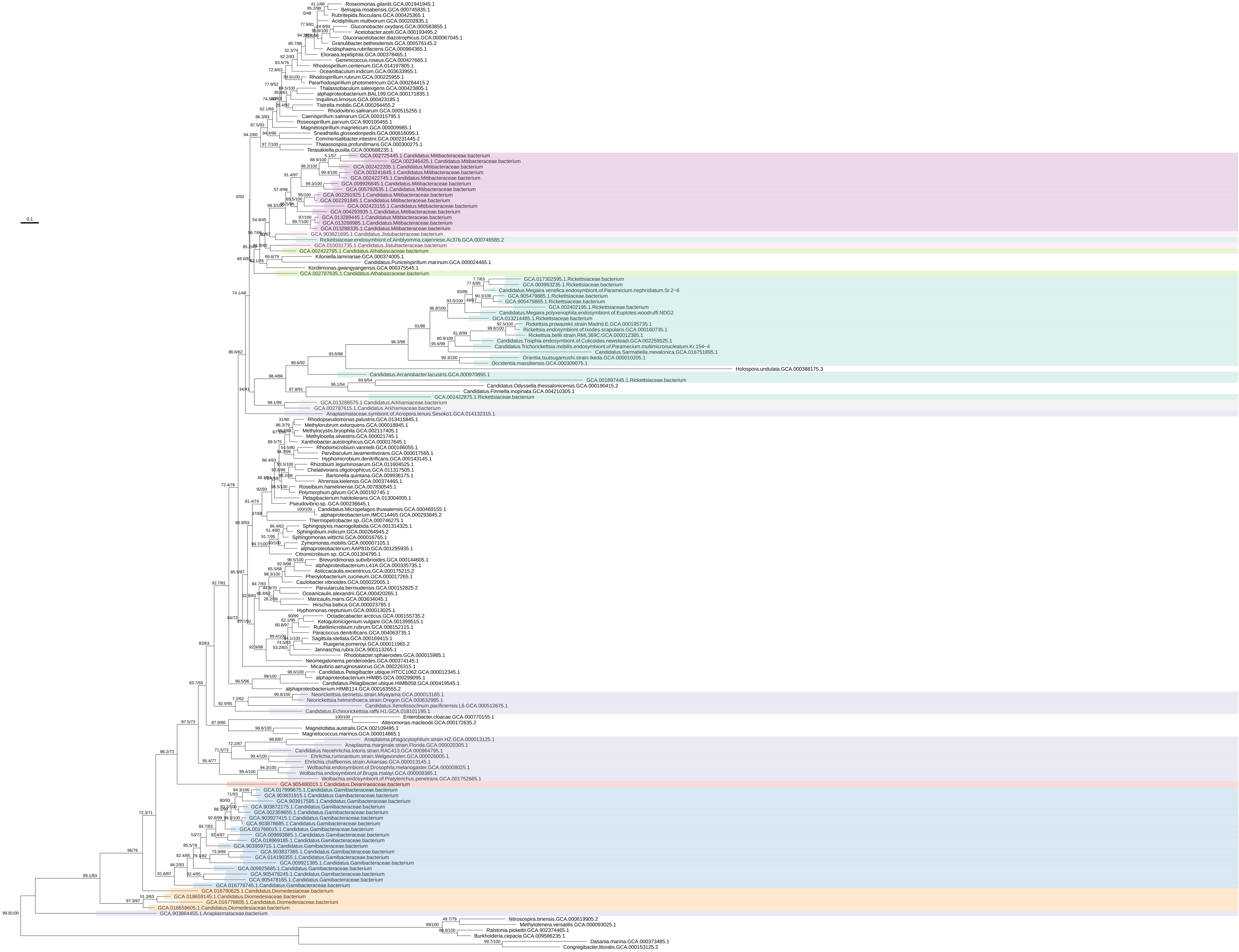

purine/09_purB_COG0015@1

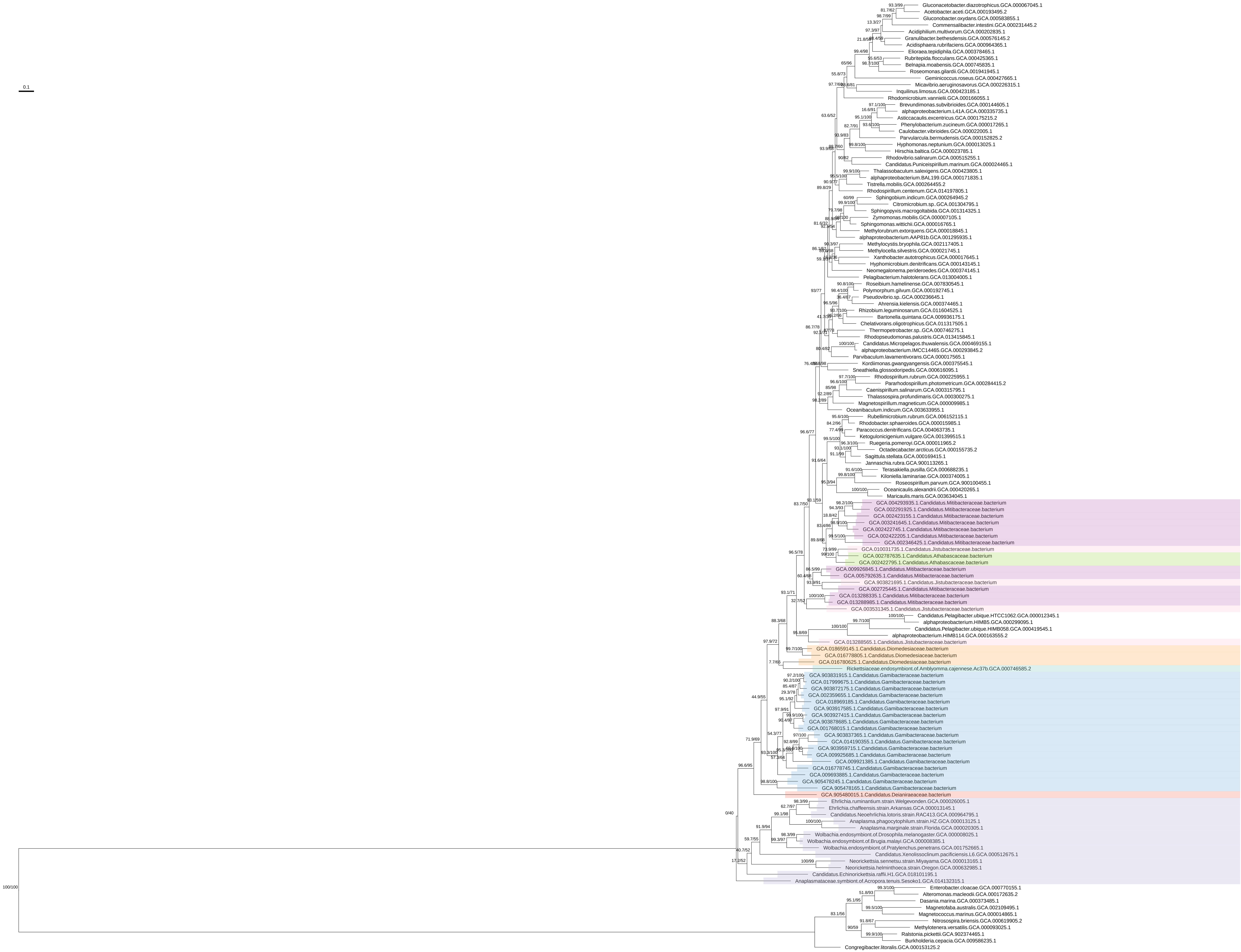

purine/10_purH_COG0138@2

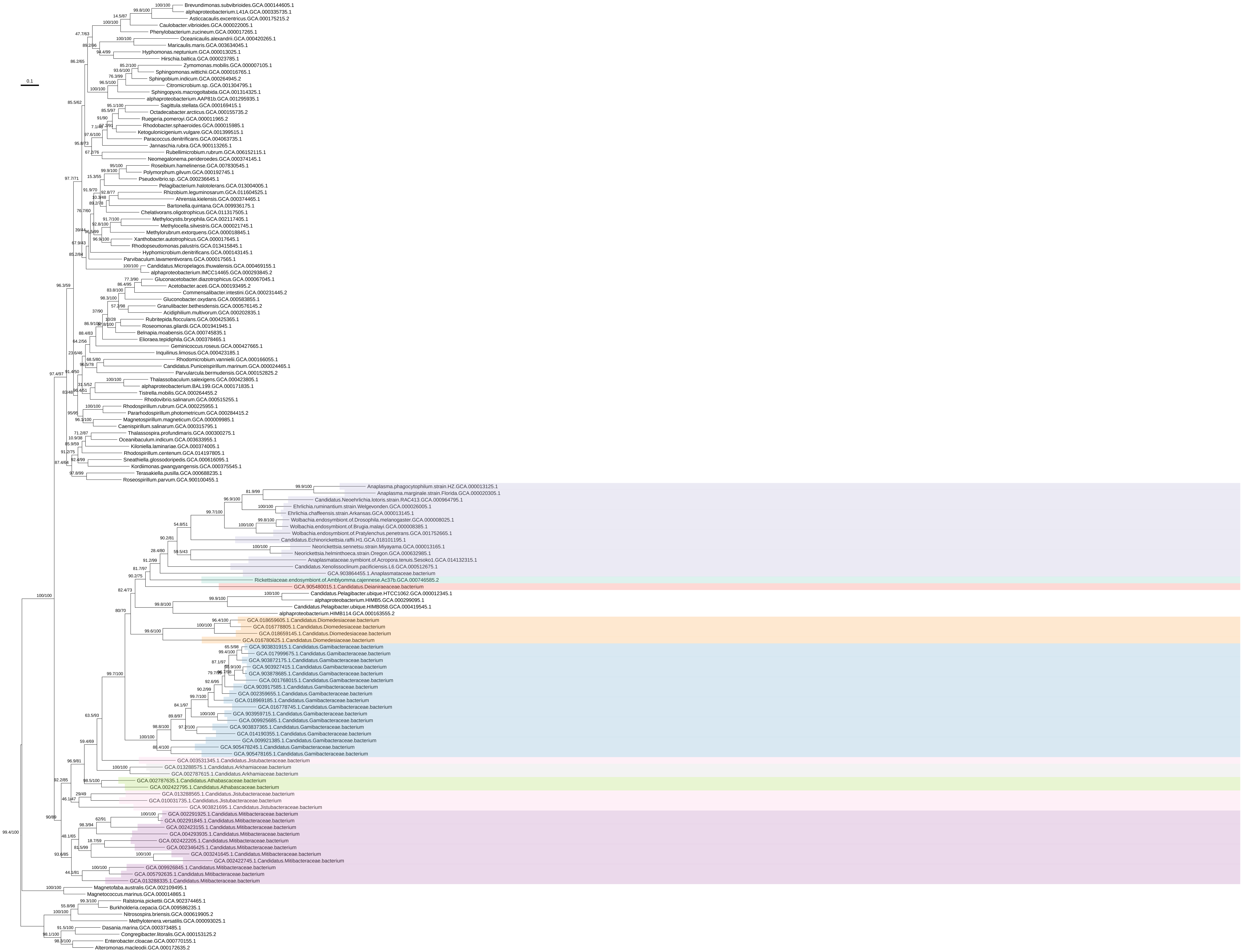

purine/11_guaB_COG0516@1

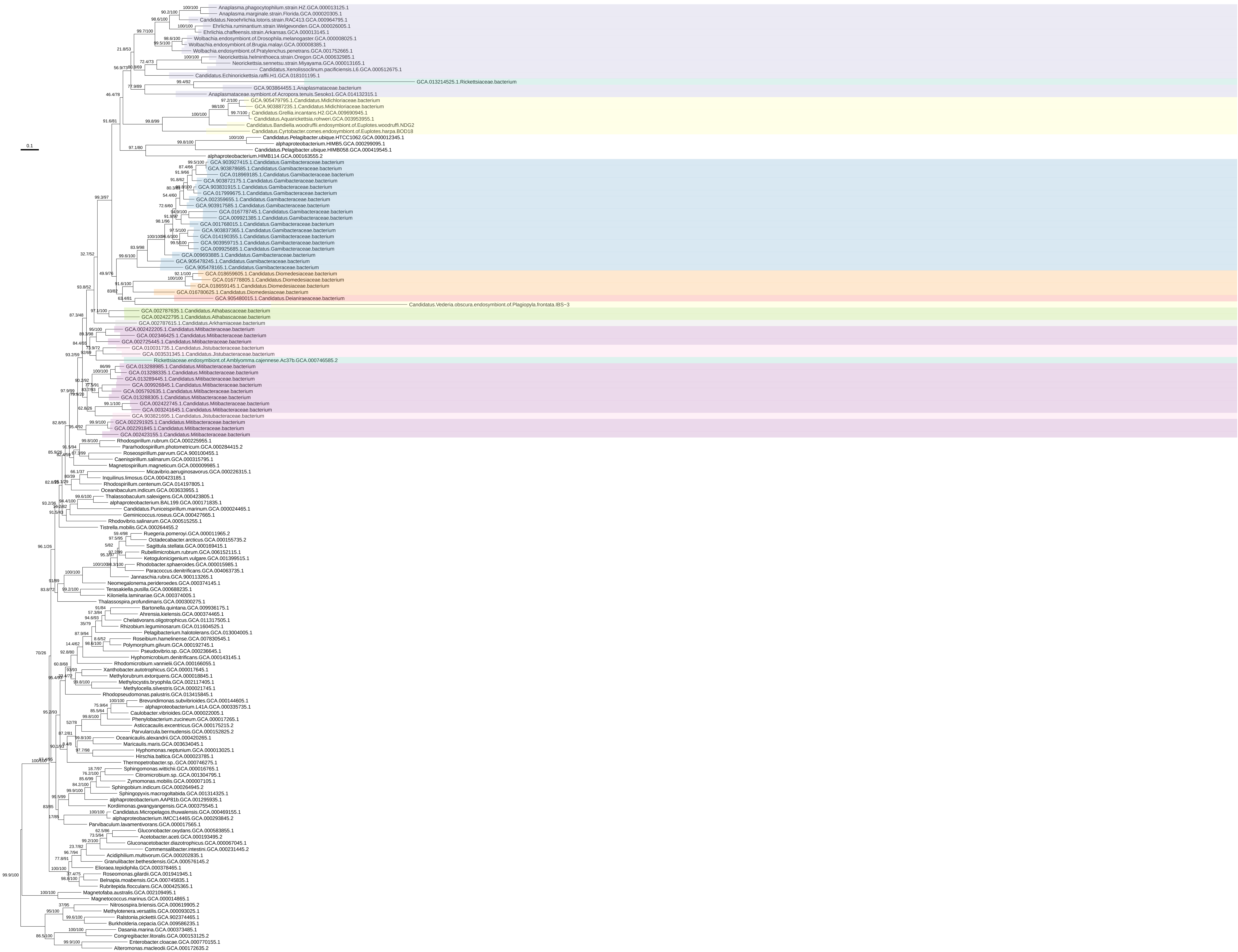

purine/12_guaA_COG0519@1

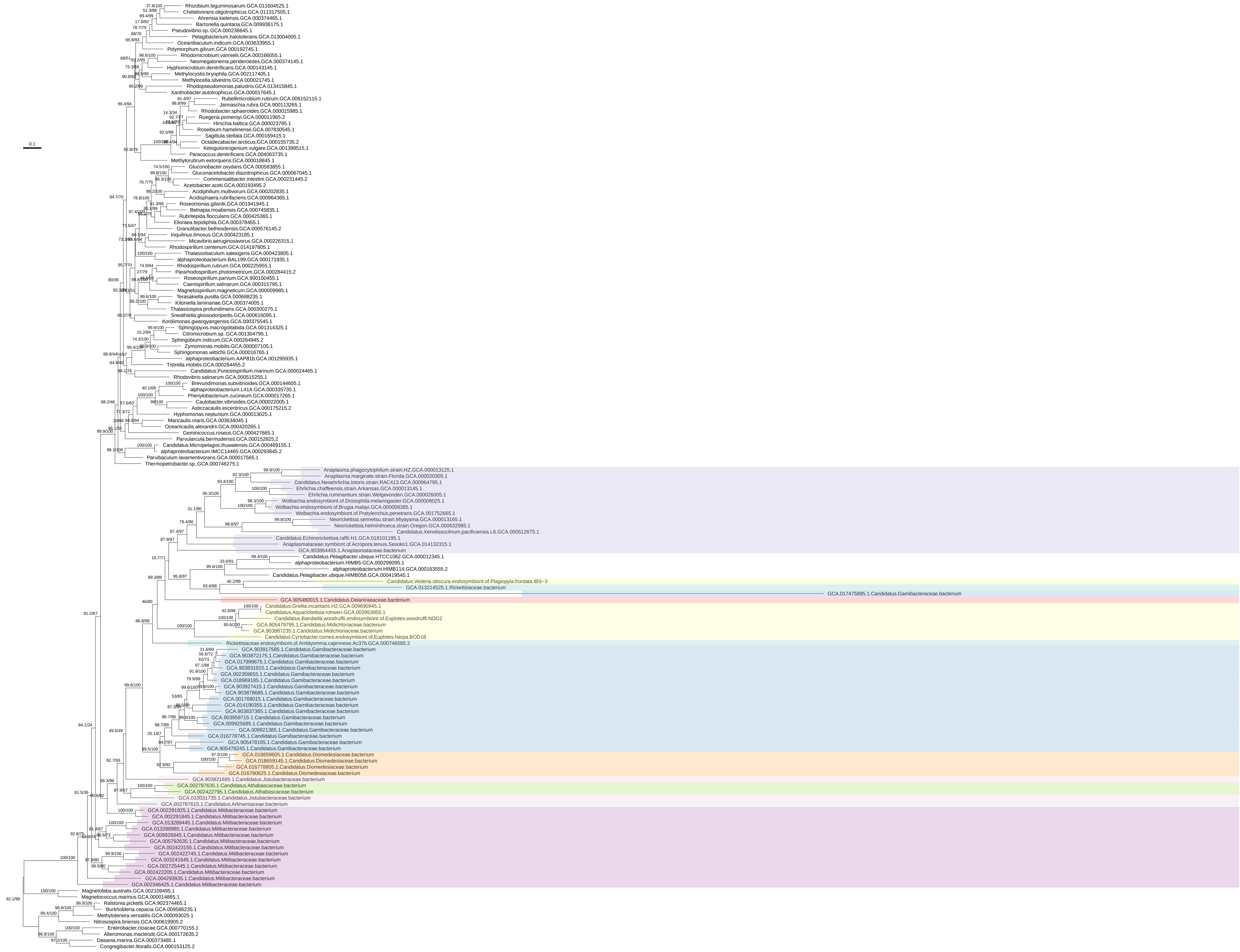

purine/13_gmk_COG0194@1

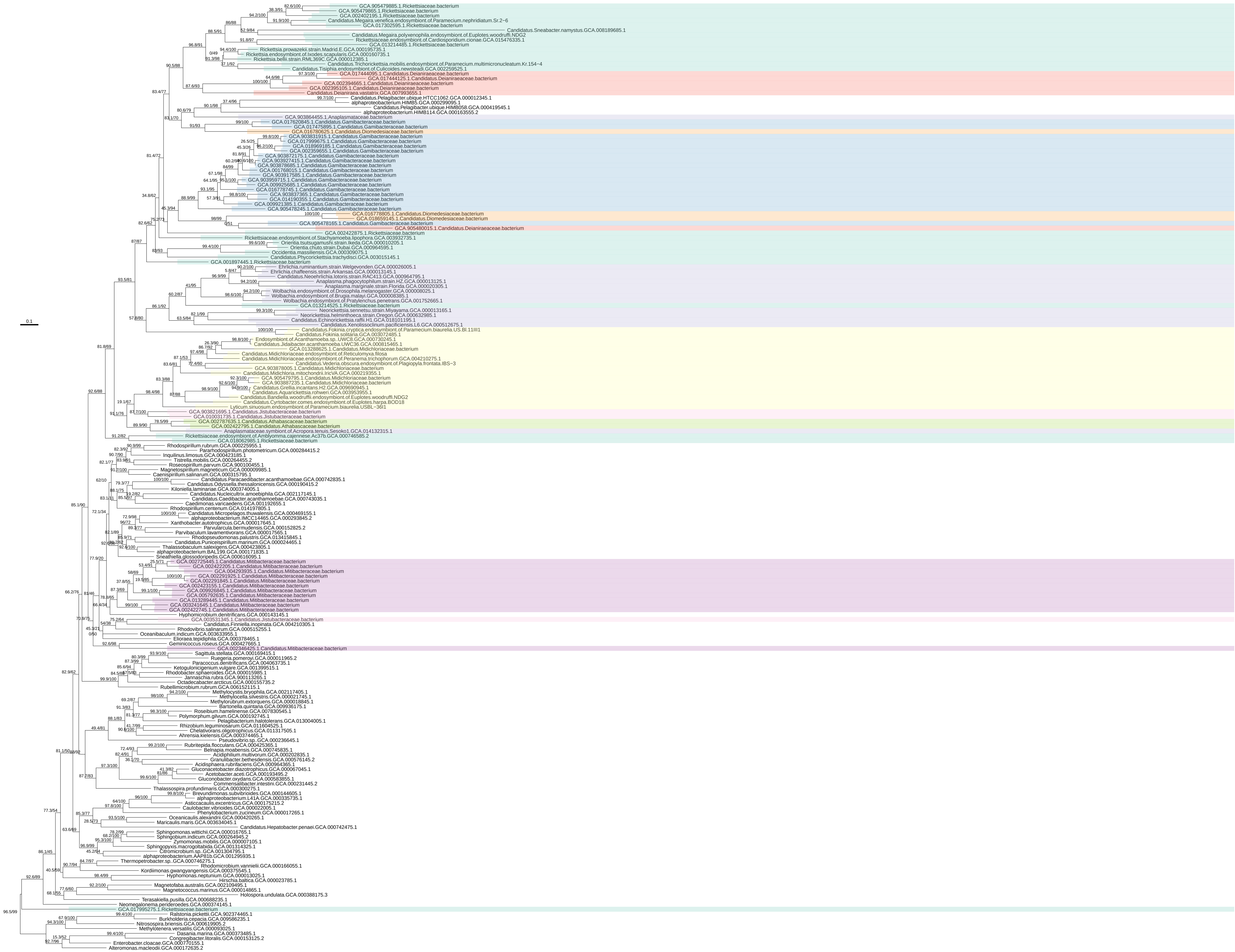

purine/14_purA_COG0104@2

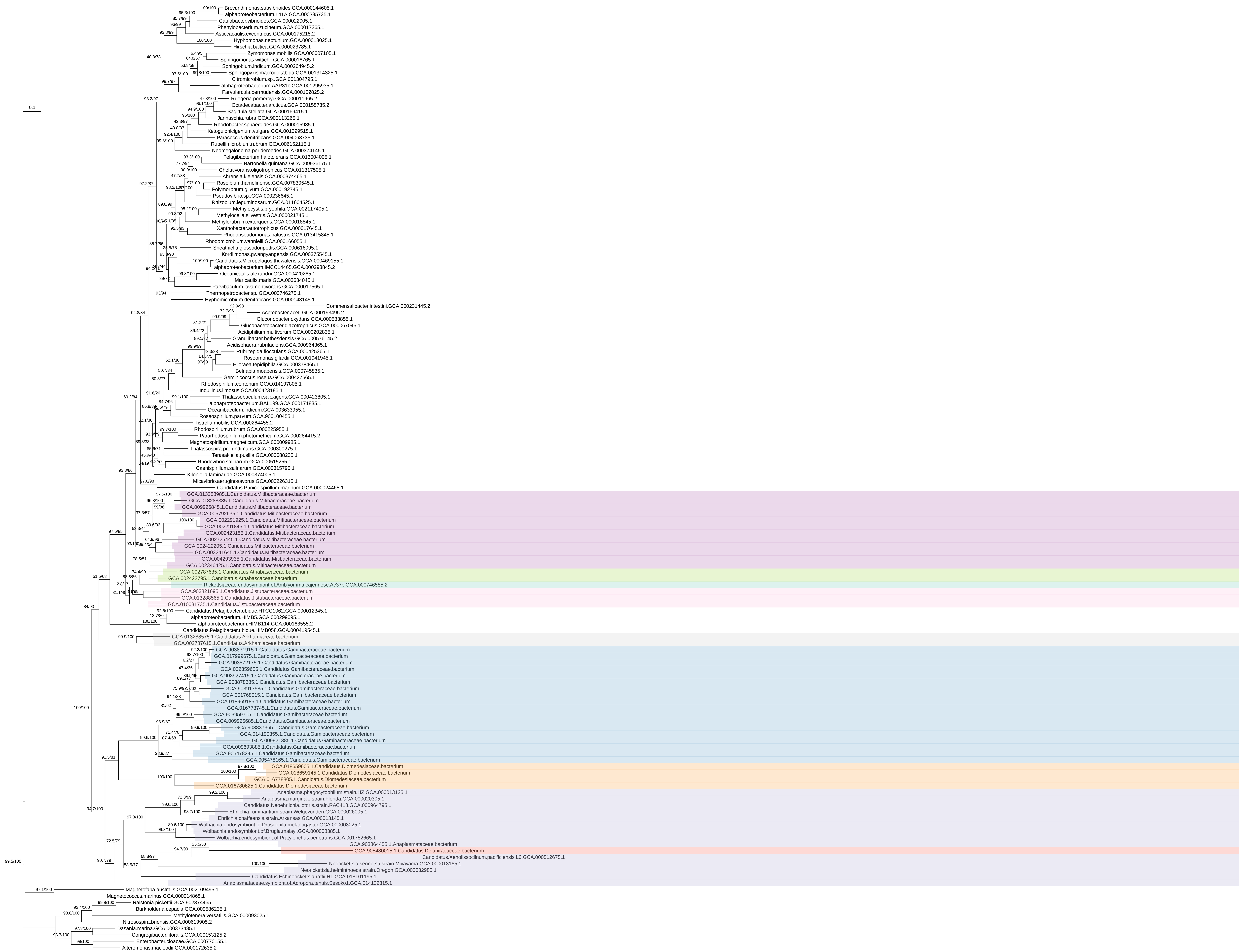

purine/15_adk_COG0563@1

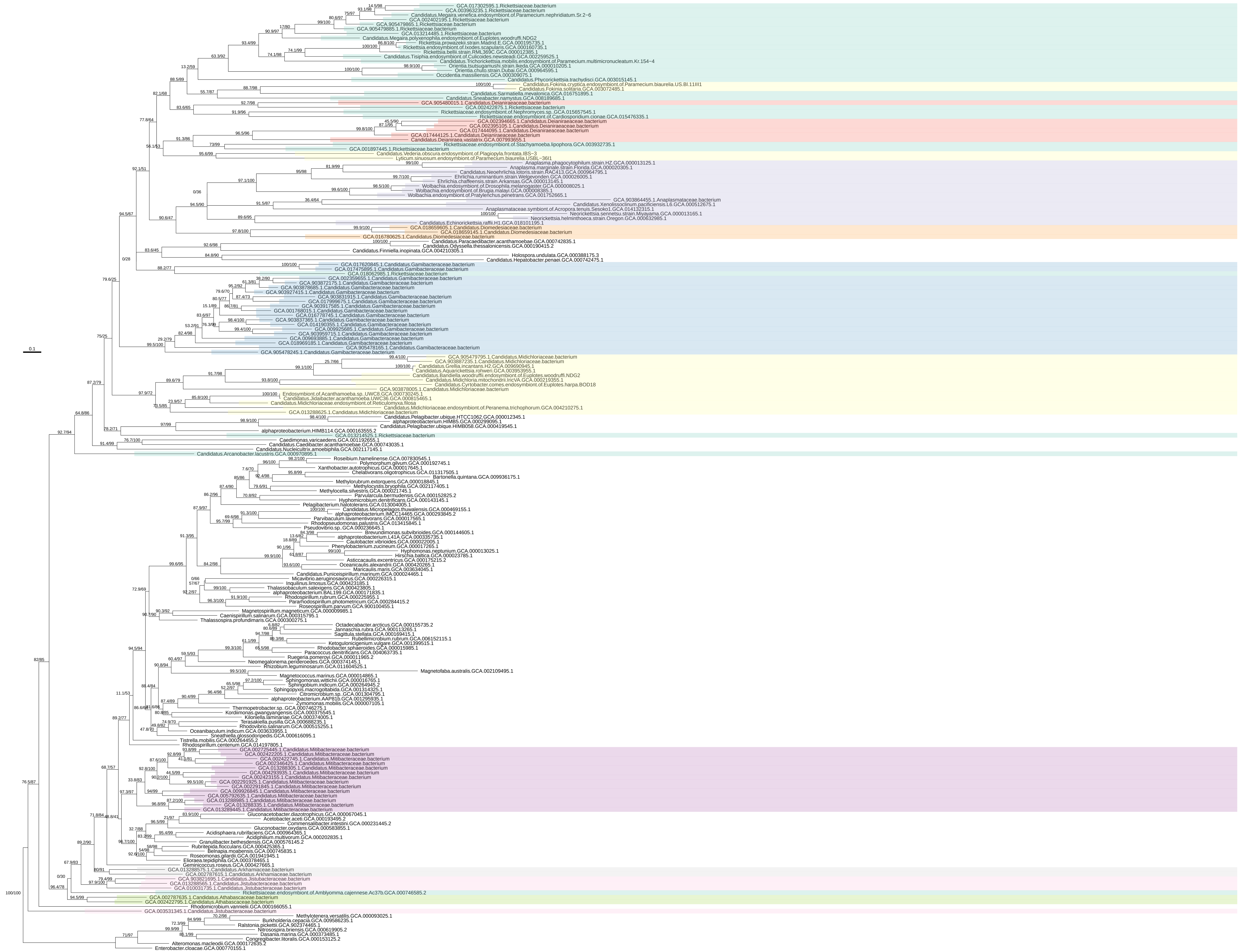

Arg/01_argJ_COG1364@2

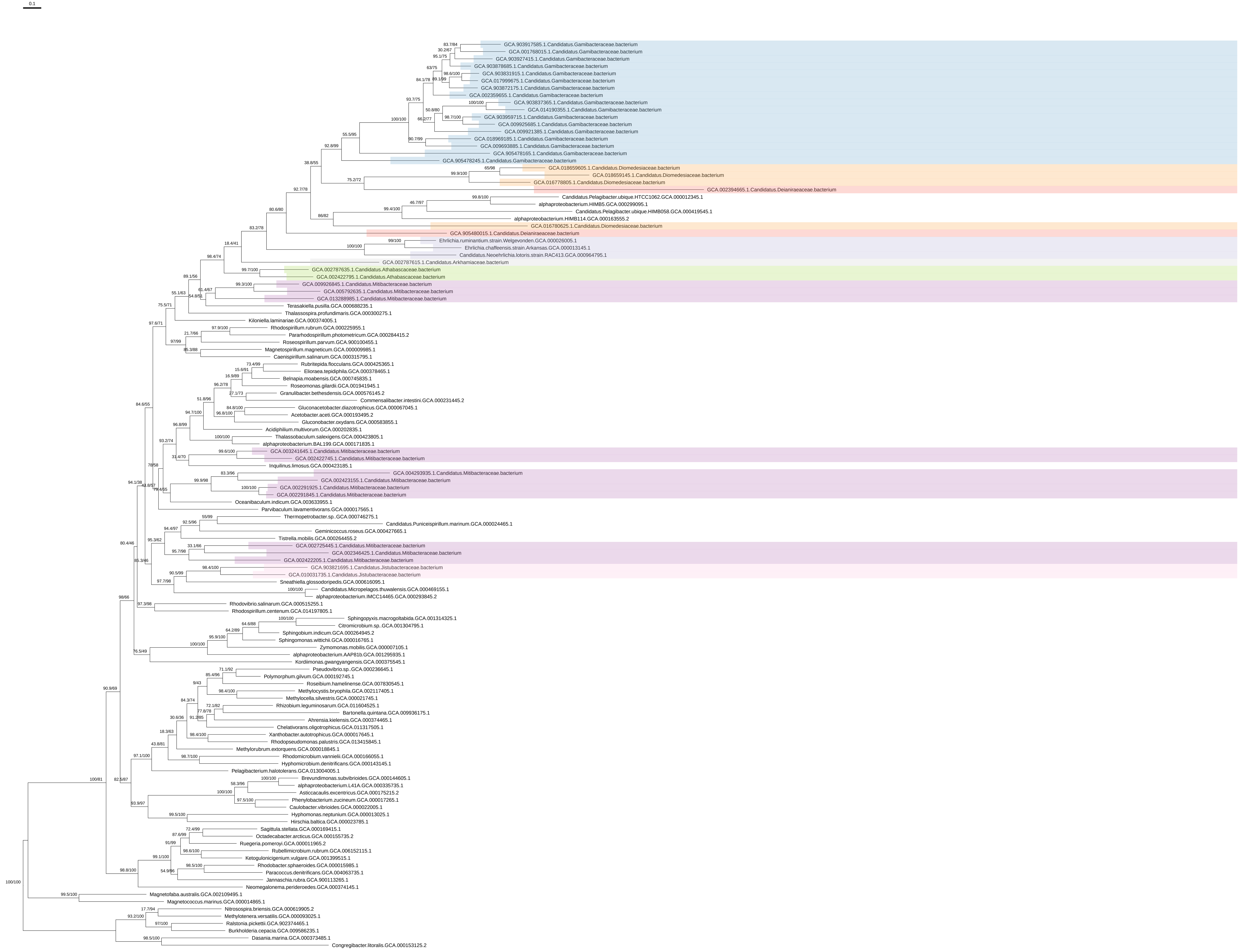

Arg/02_argB_COG0548@2

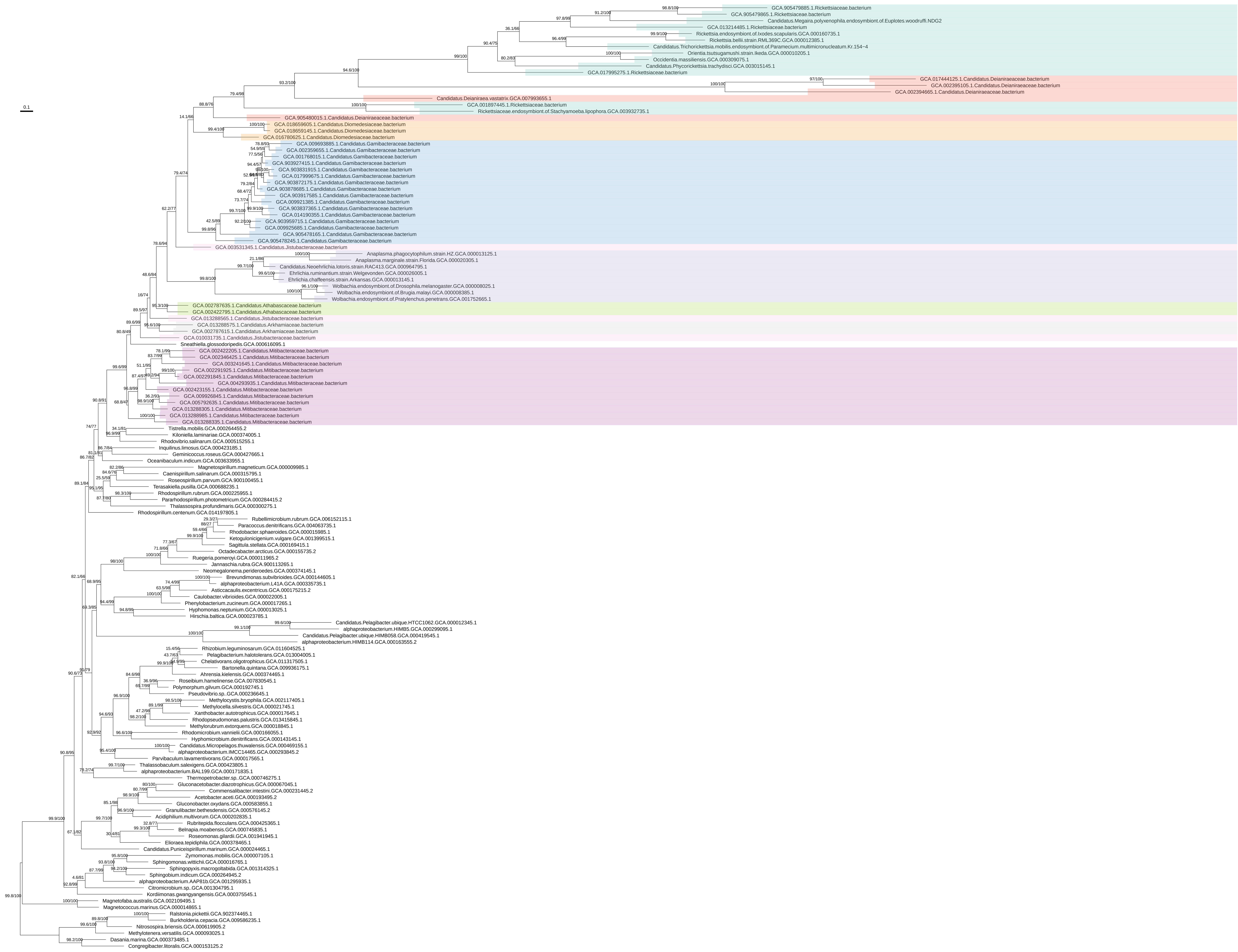

Arg/03_argC_COG0002@1

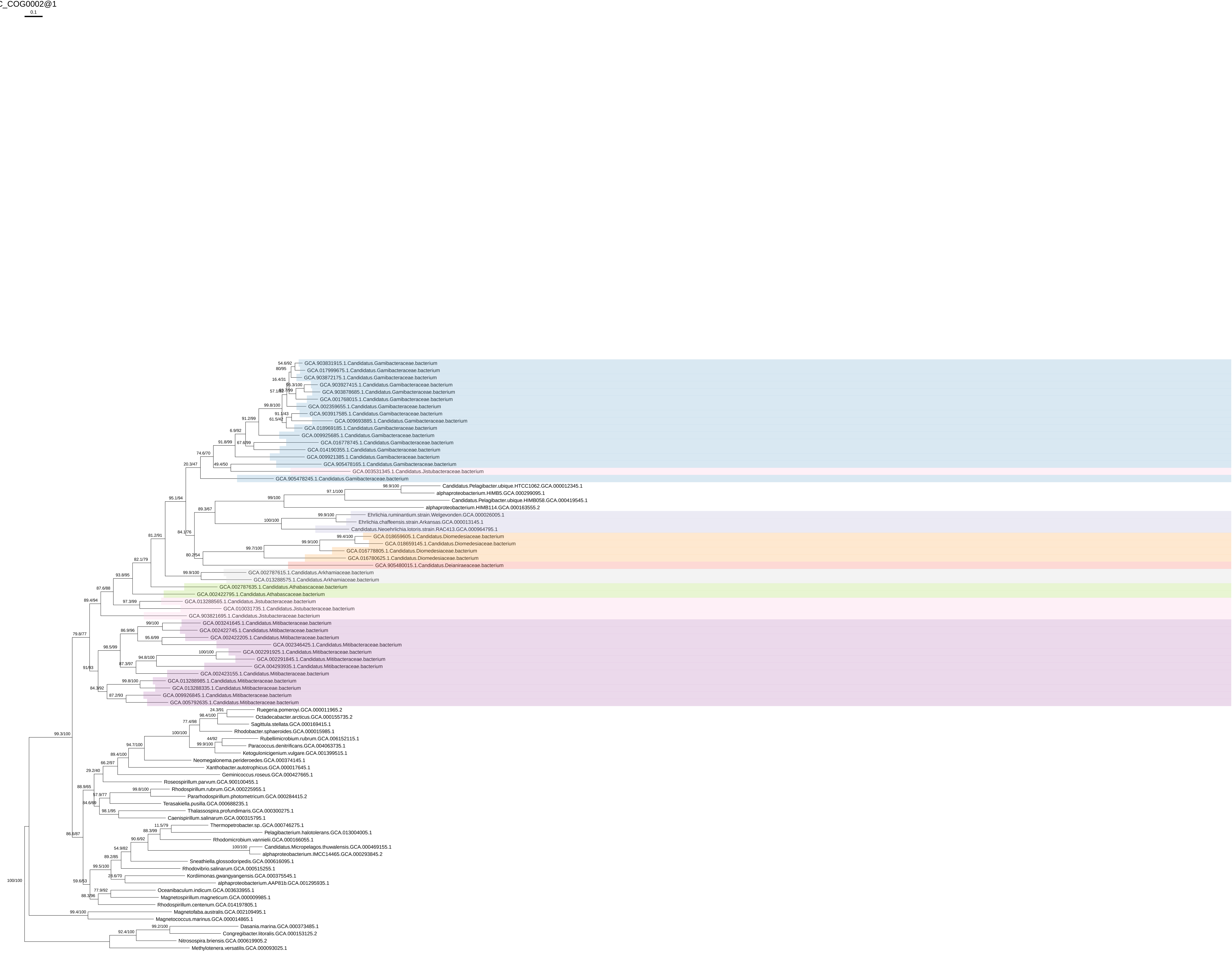

Arg/04_argD_COG4992@1

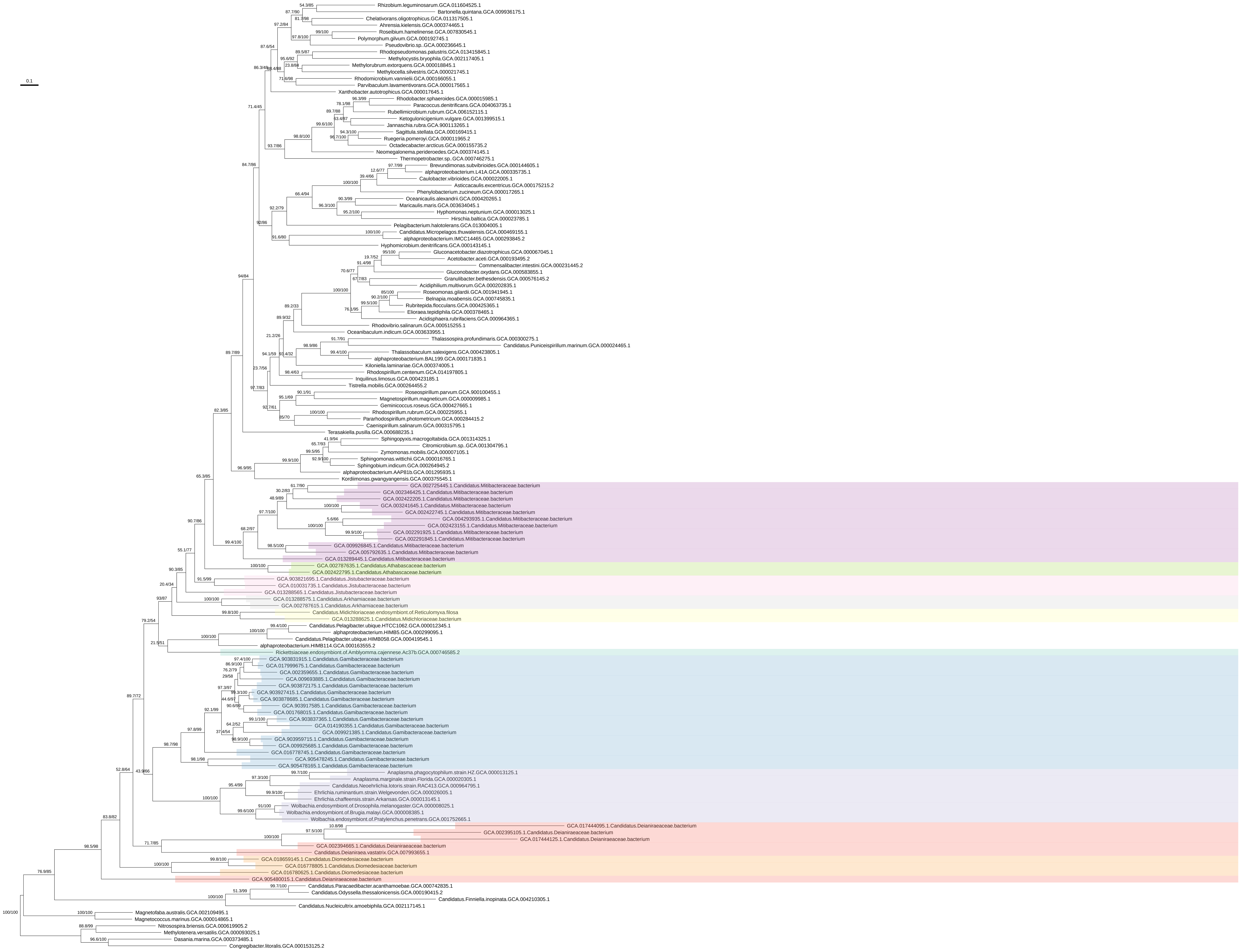

Arg/05_argF_COG0078@2

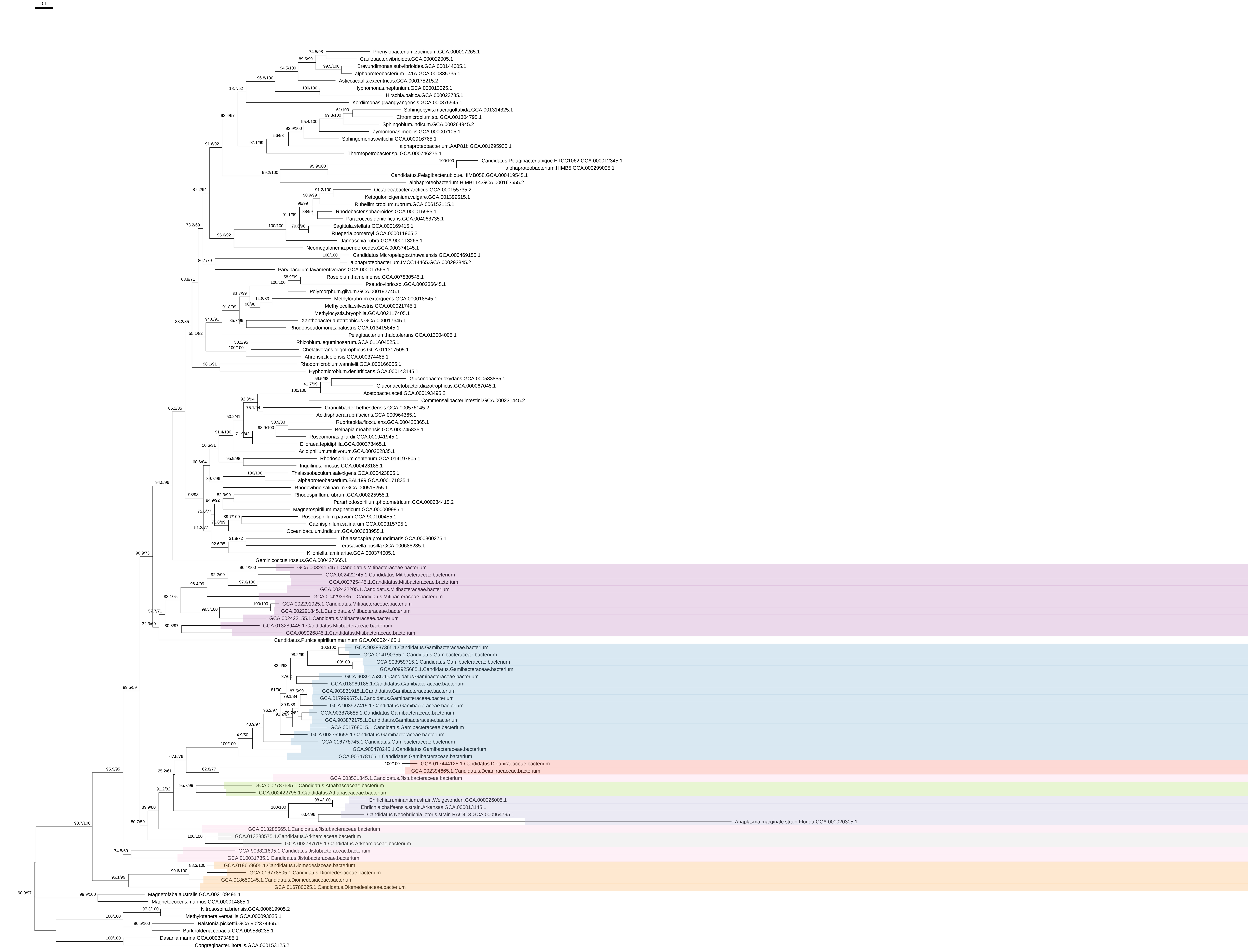

Arg/06_argG_COG0137@1

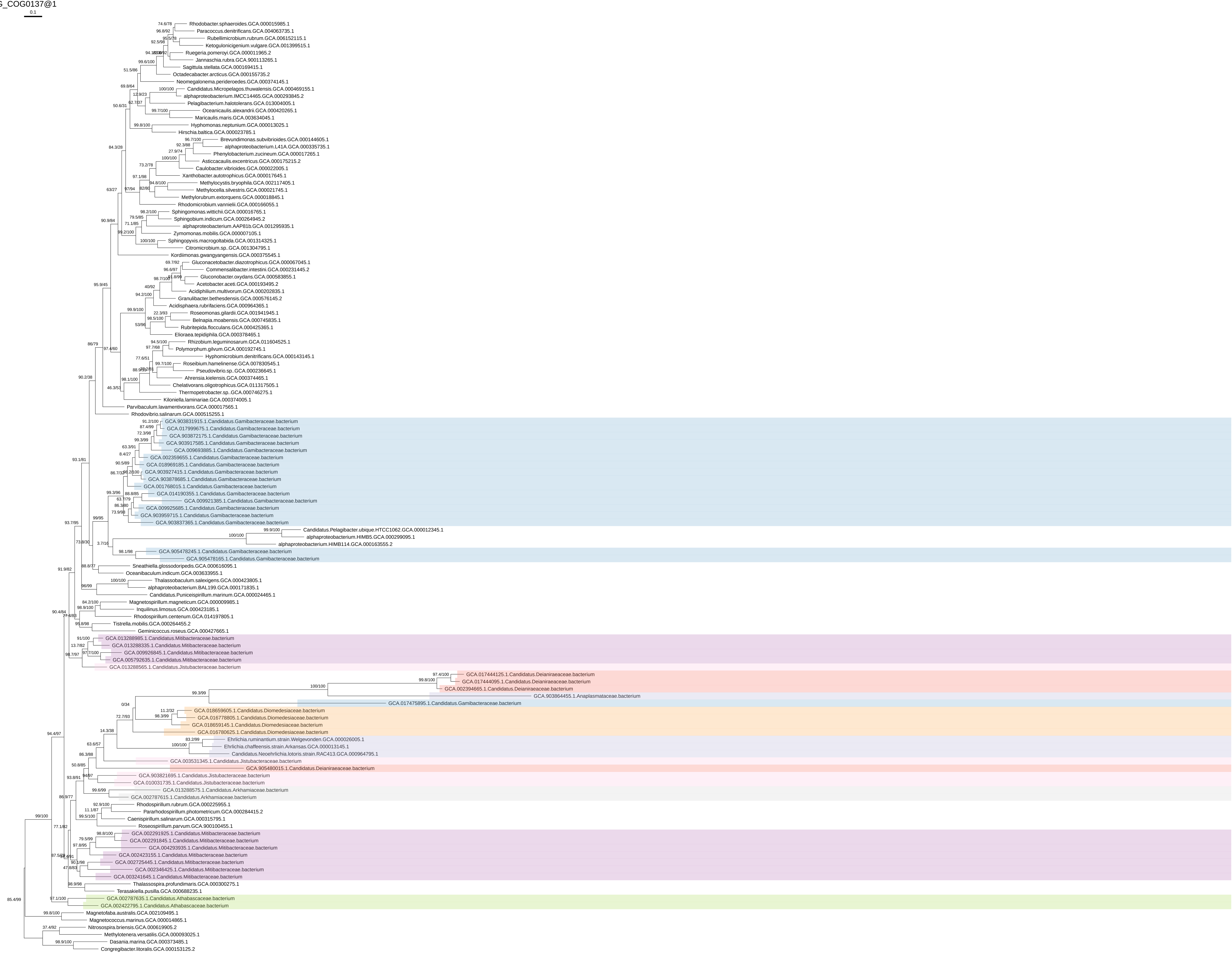

Arg/07_argH_COG0165@2

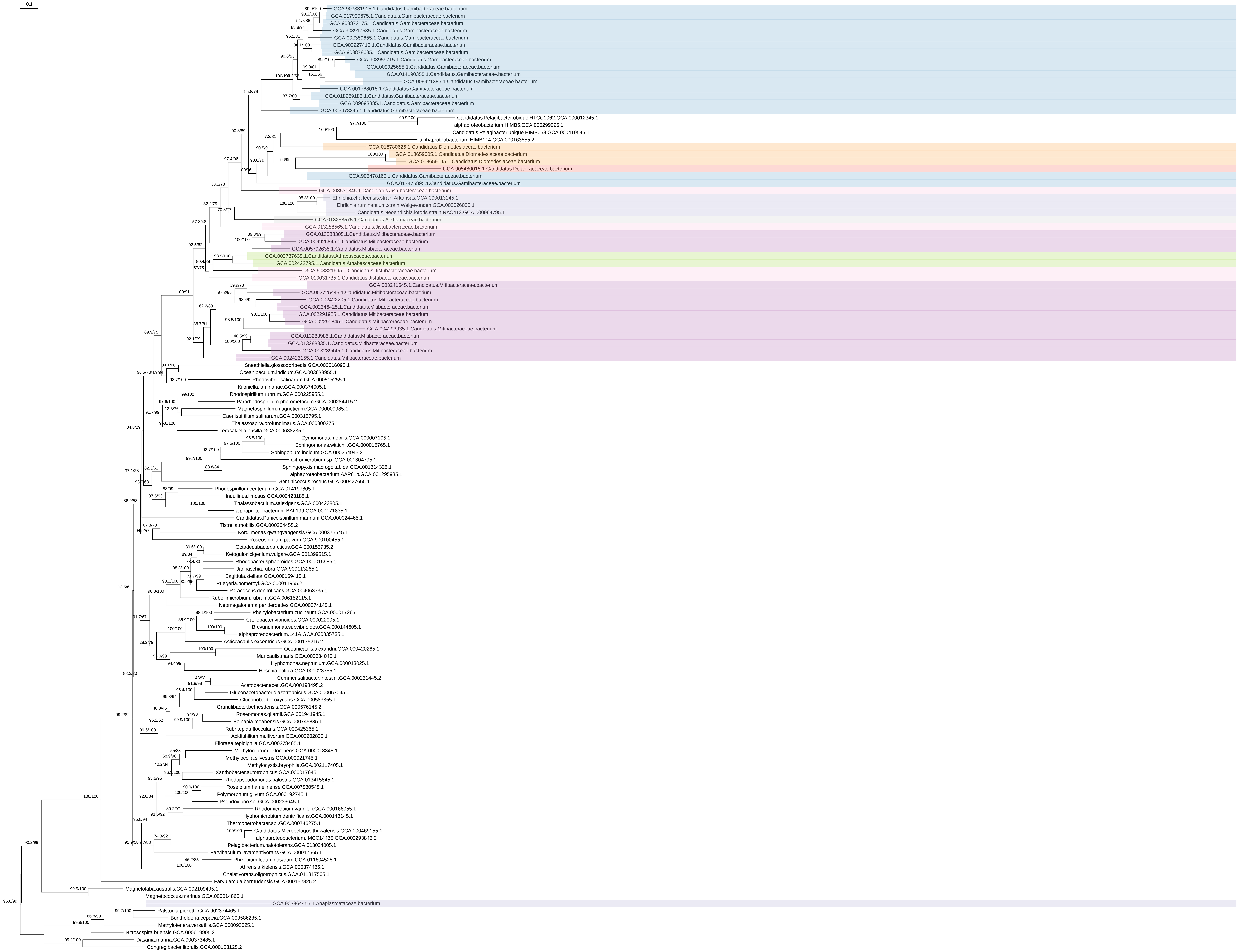

Ser/01_serA_COG0111@2

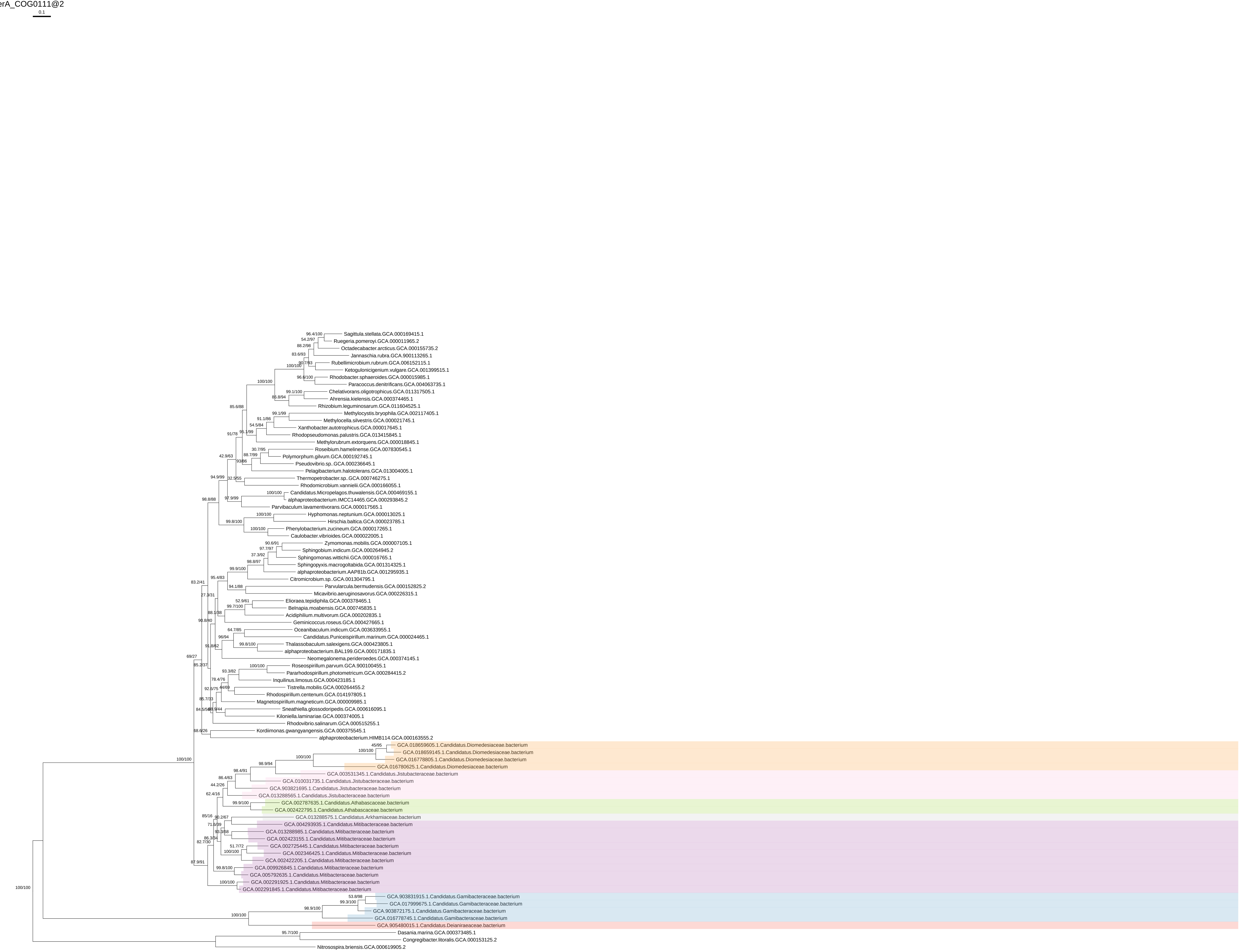

Ser/02_serC_COG1932@1

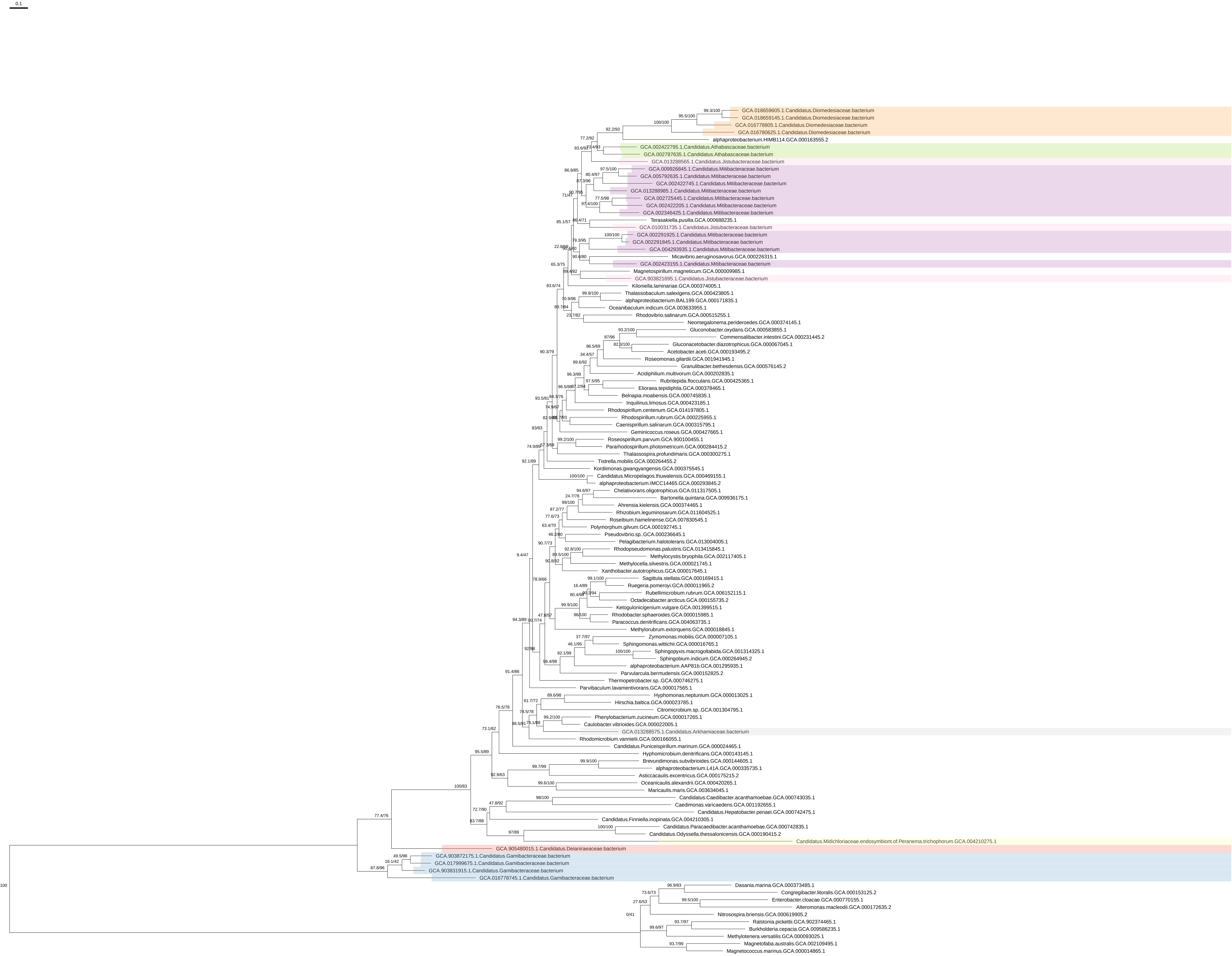

Ser/03_serB_COG0560@2

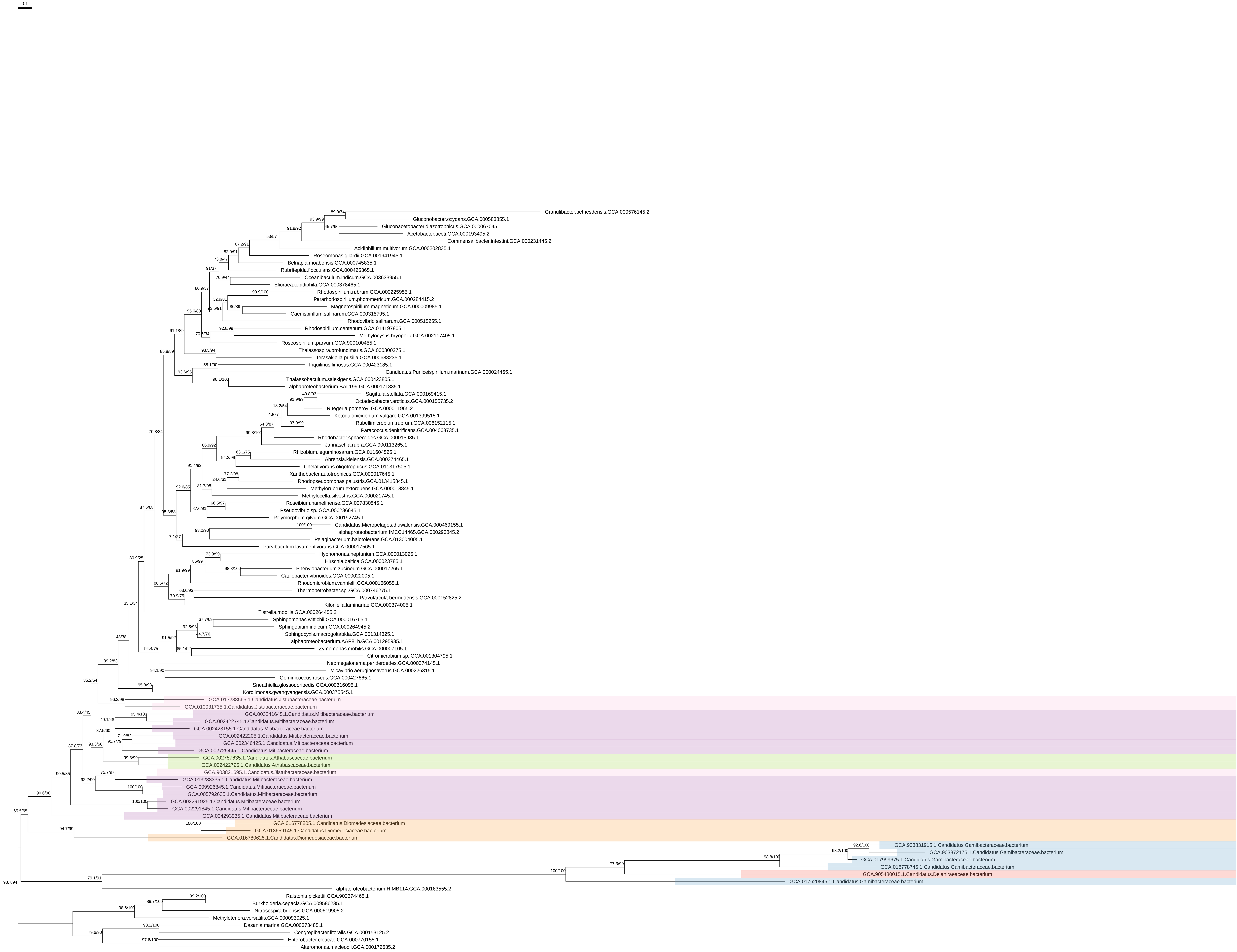

Pro/01_proB_1MUBG@1224

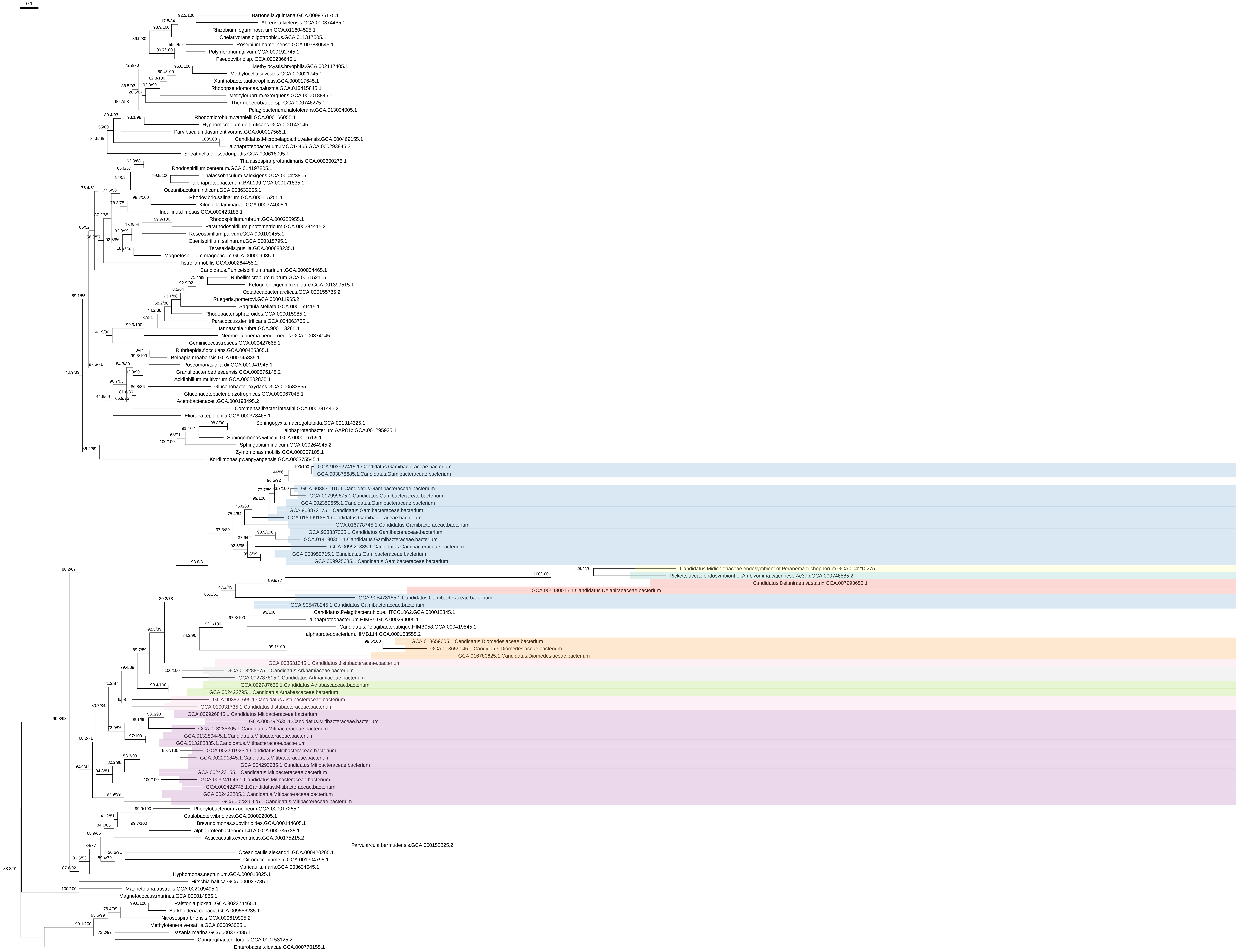

Pro/02_proA_COG0014@2

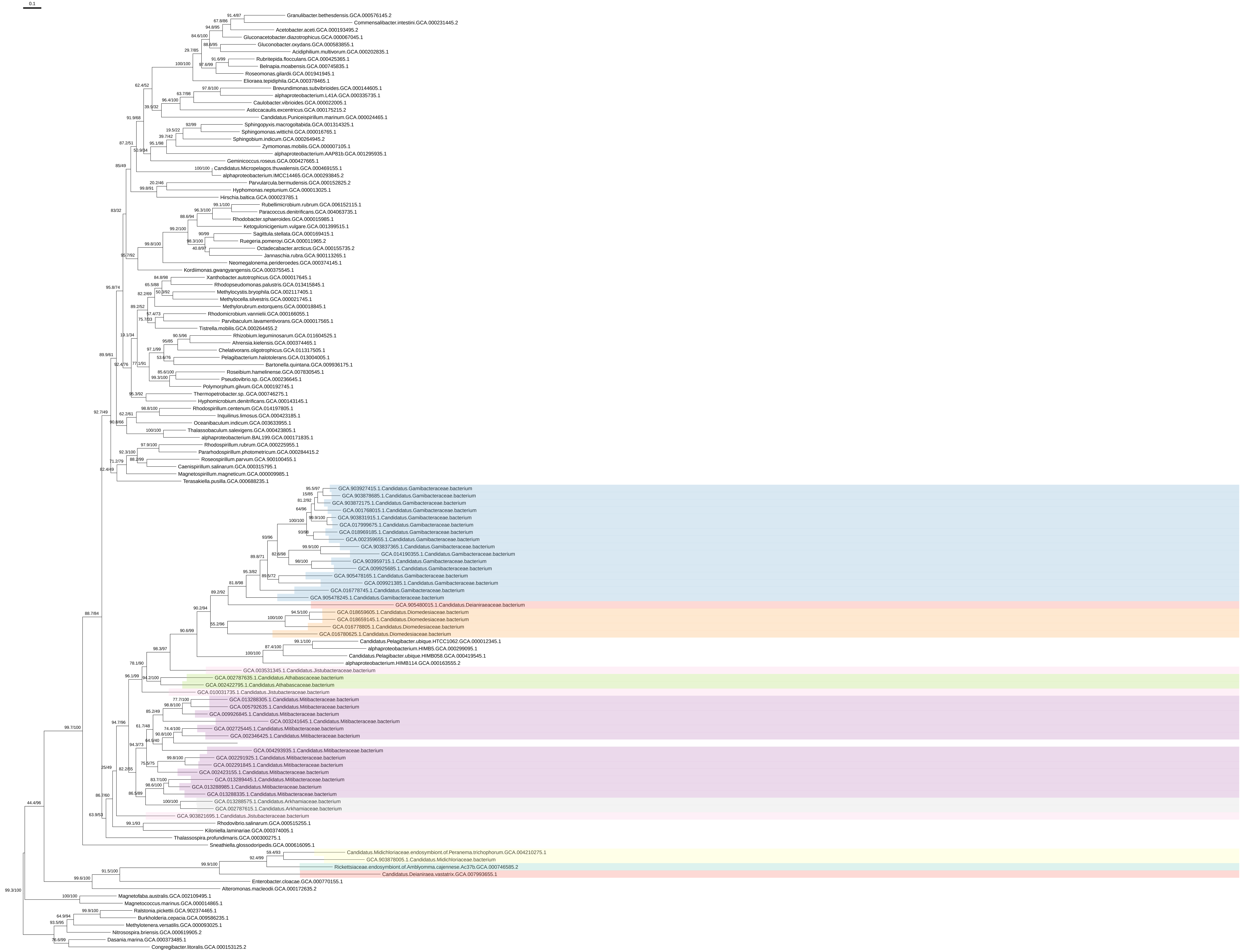

Pro/03_COG0345@2

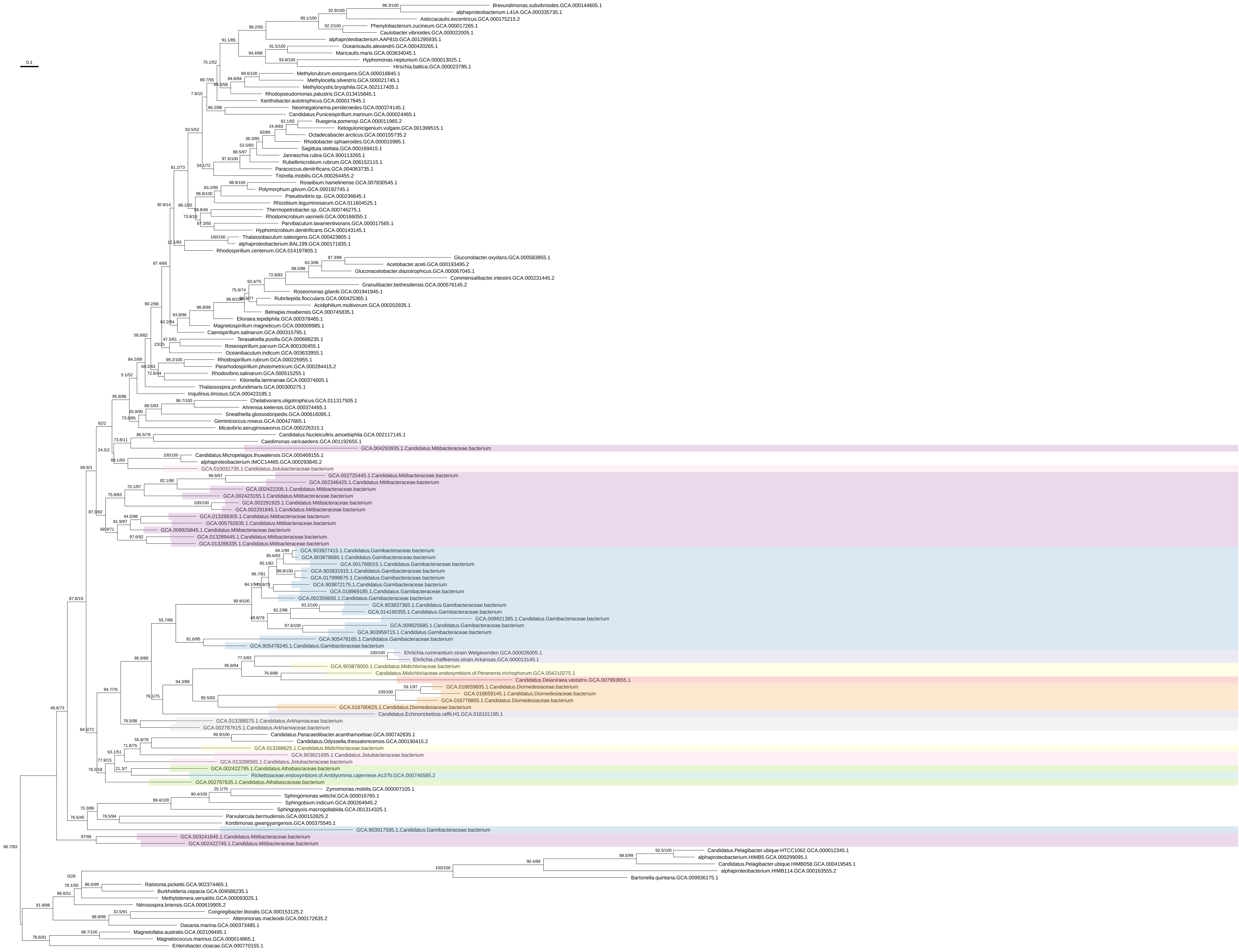

His/01_hisG_COG0040@2

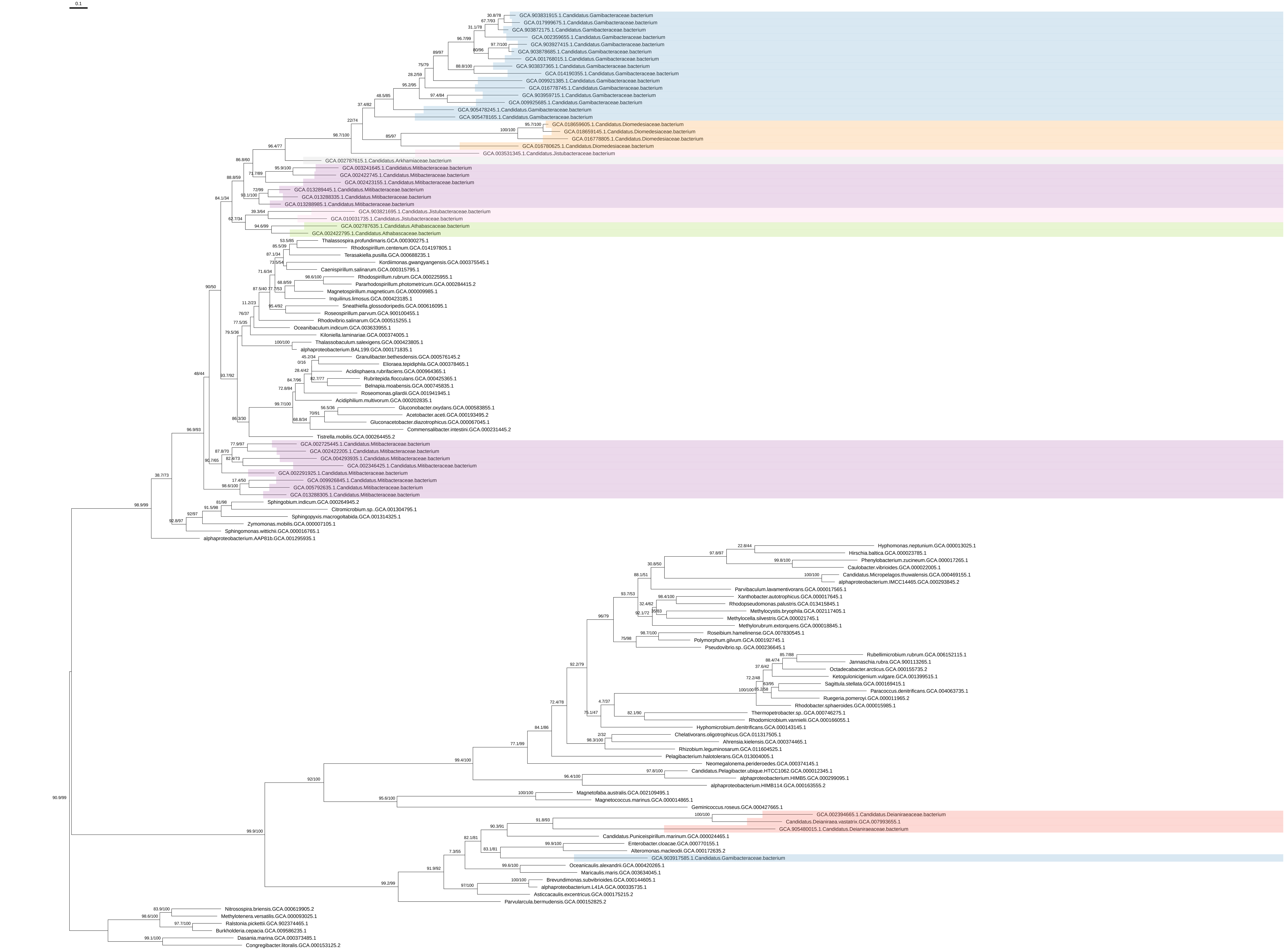

His/02_hisI_COG0139@1

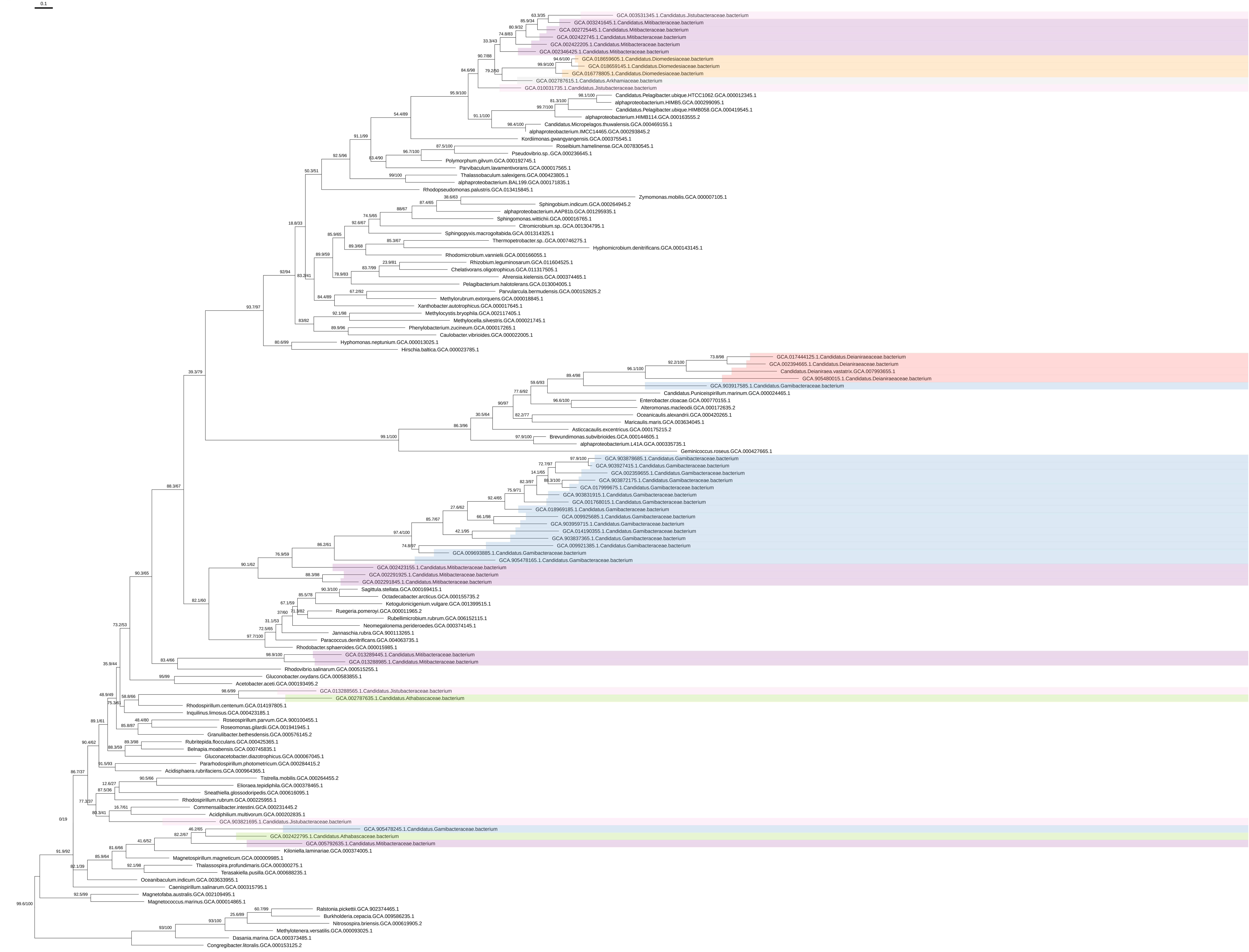

His/03_hisA_COG0106@2

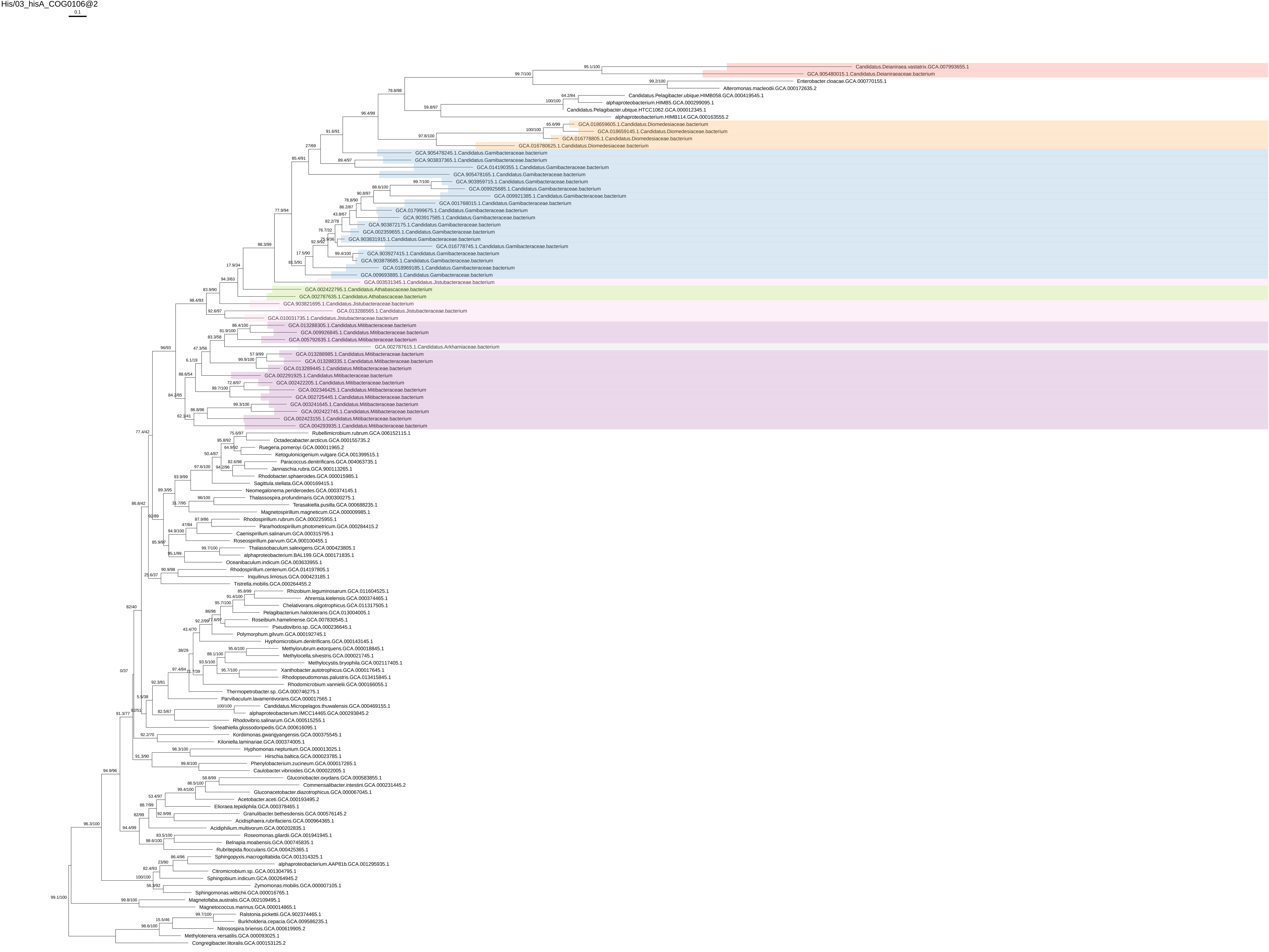

His/04_hisF_COG0107@2

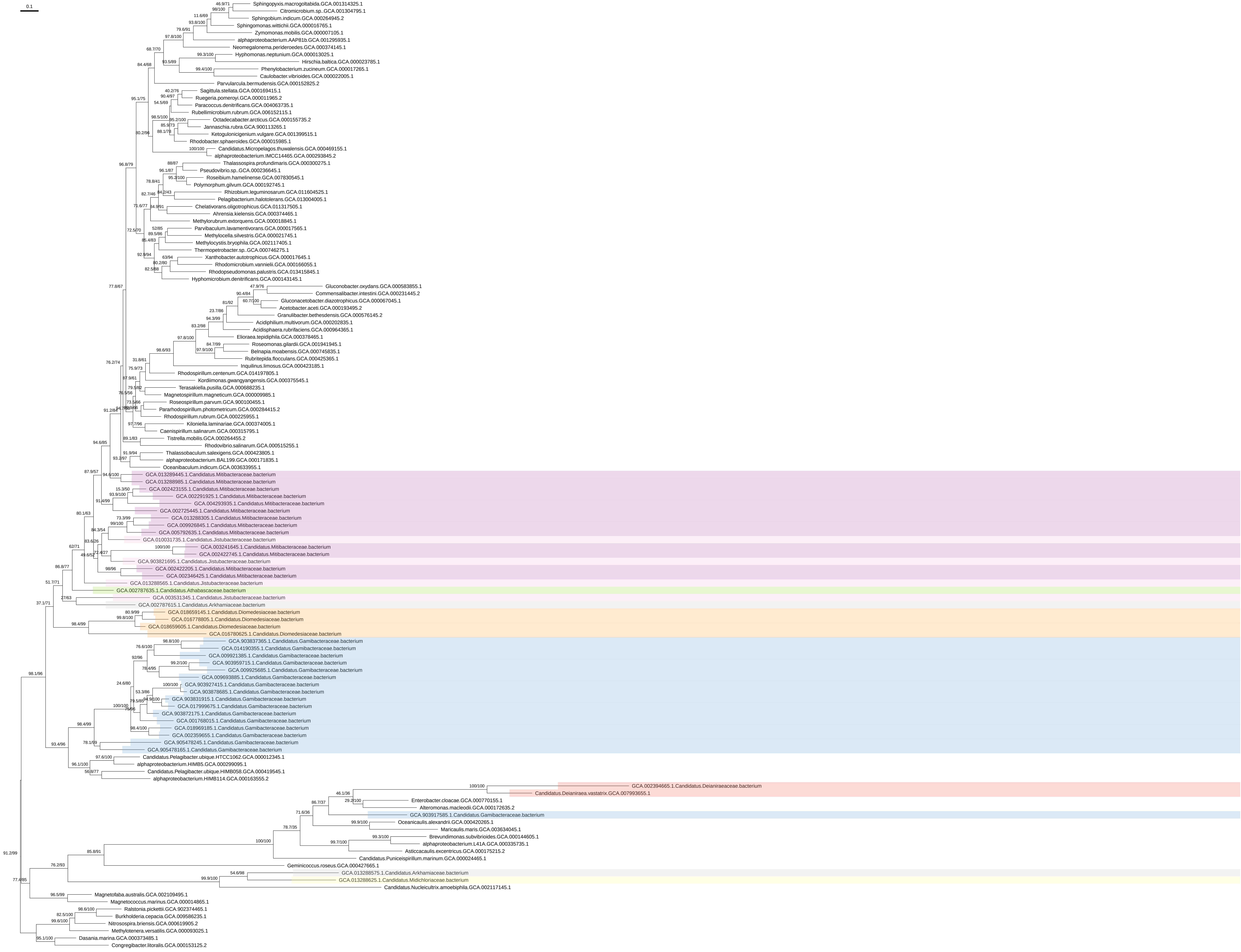

His/05_hisH_COG0118@2

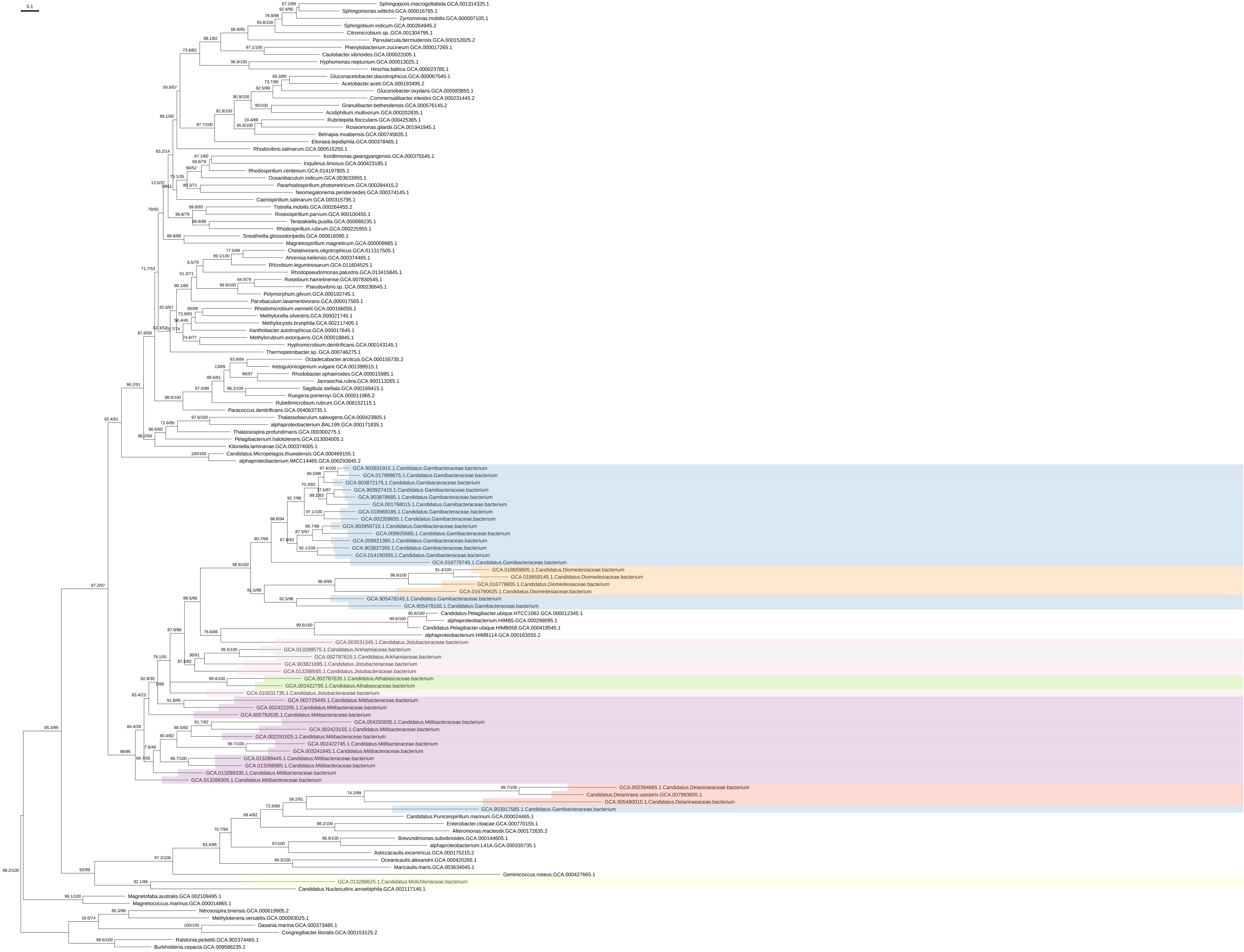

His/06_hisB_1MWBS@1224

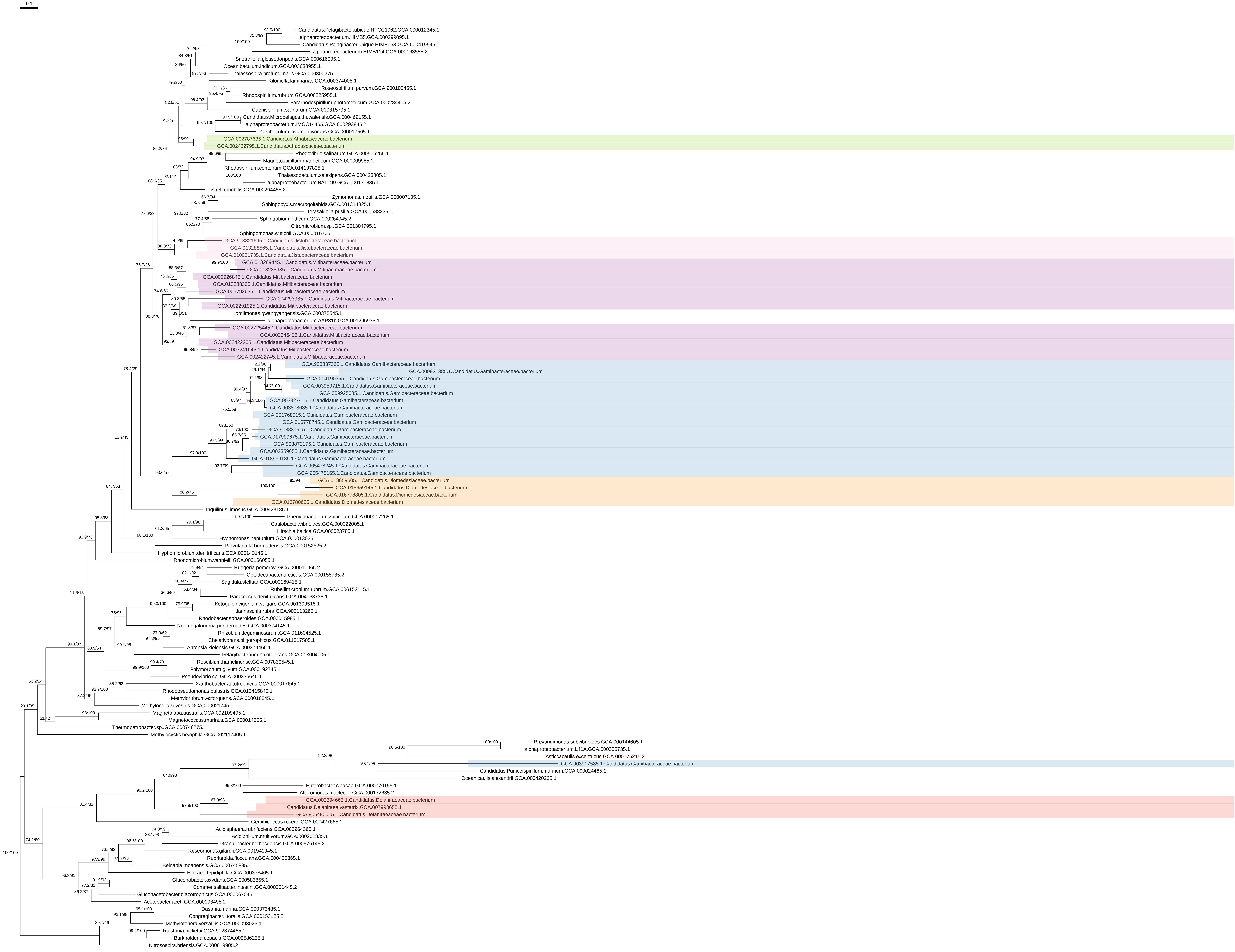

His/07_hisC_COG0079@2

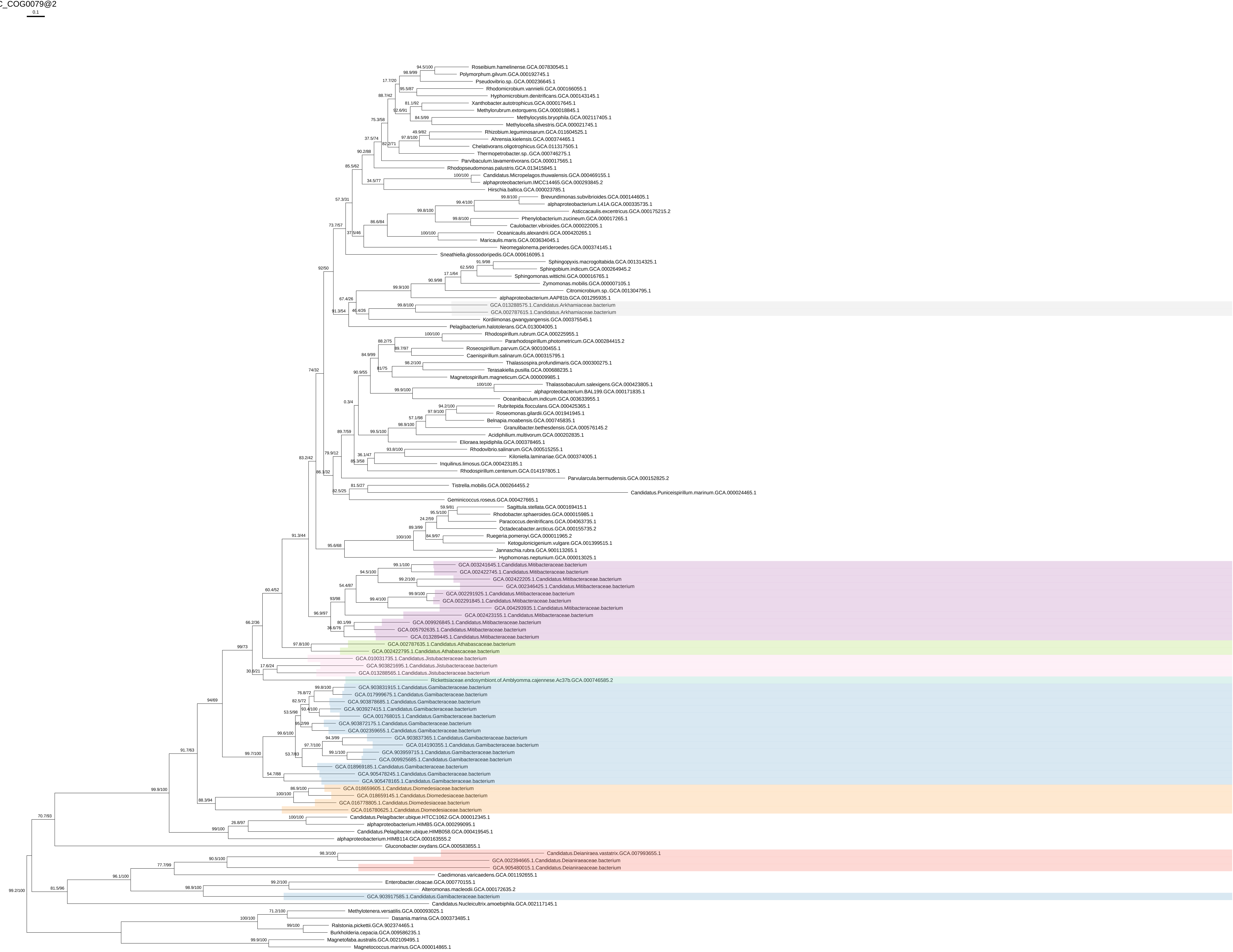

His/08_hisN_hisB_COG0241@2

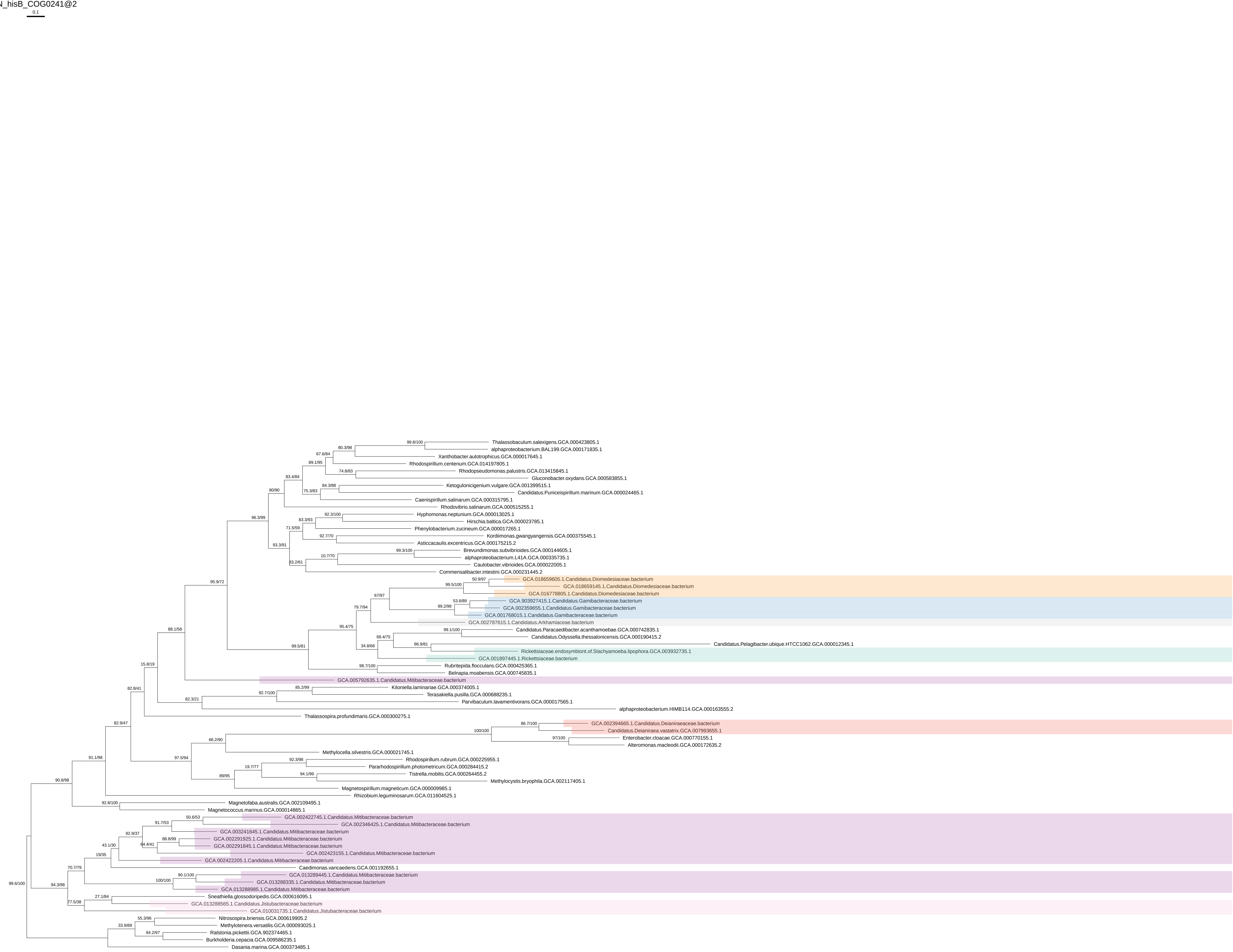

His/09_hisD_COG0141

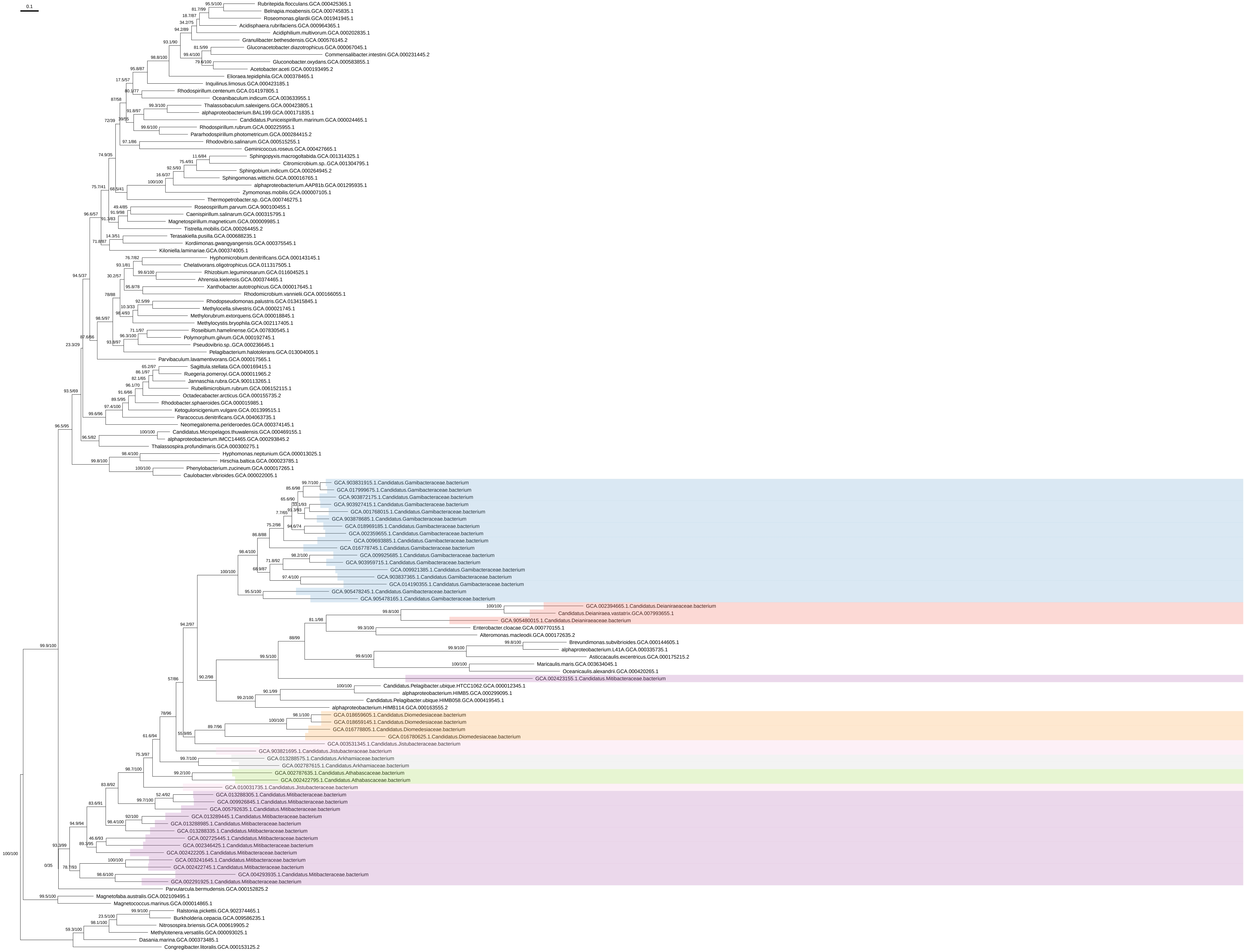

Cys/01_cysN_COG2895@2

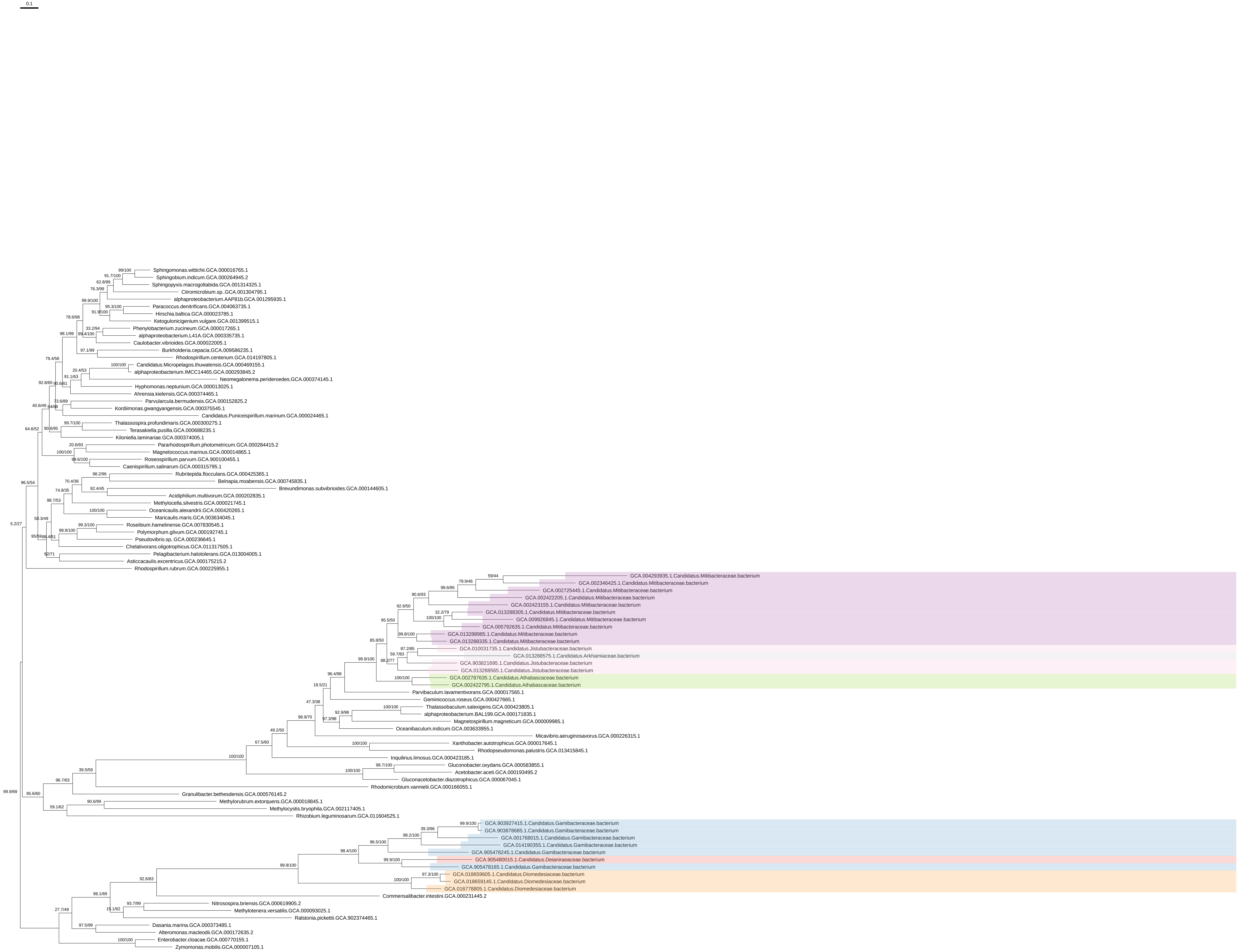

Cys/02_cysD_COG0175@2

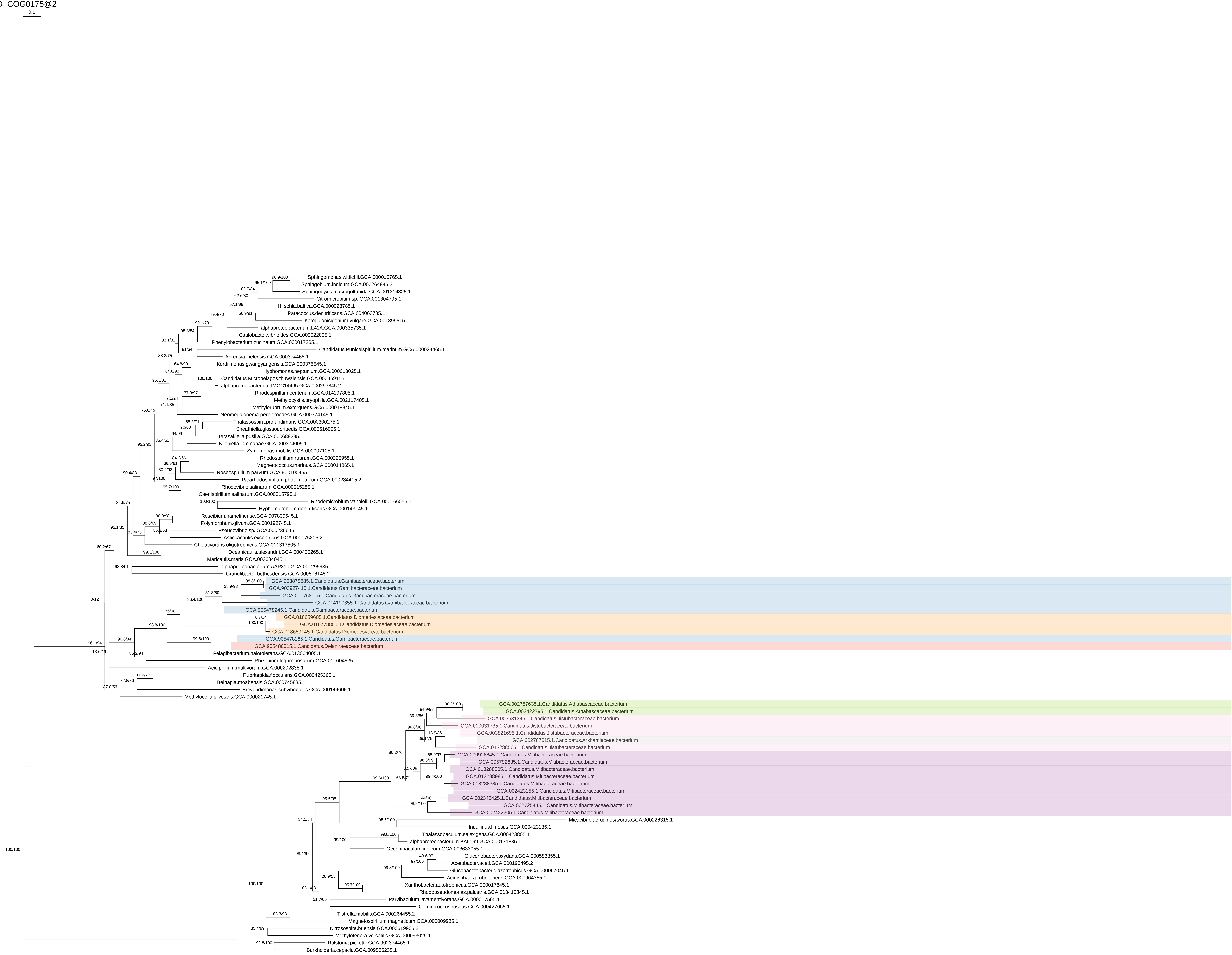

Cys/03_cysC_COG0529@2

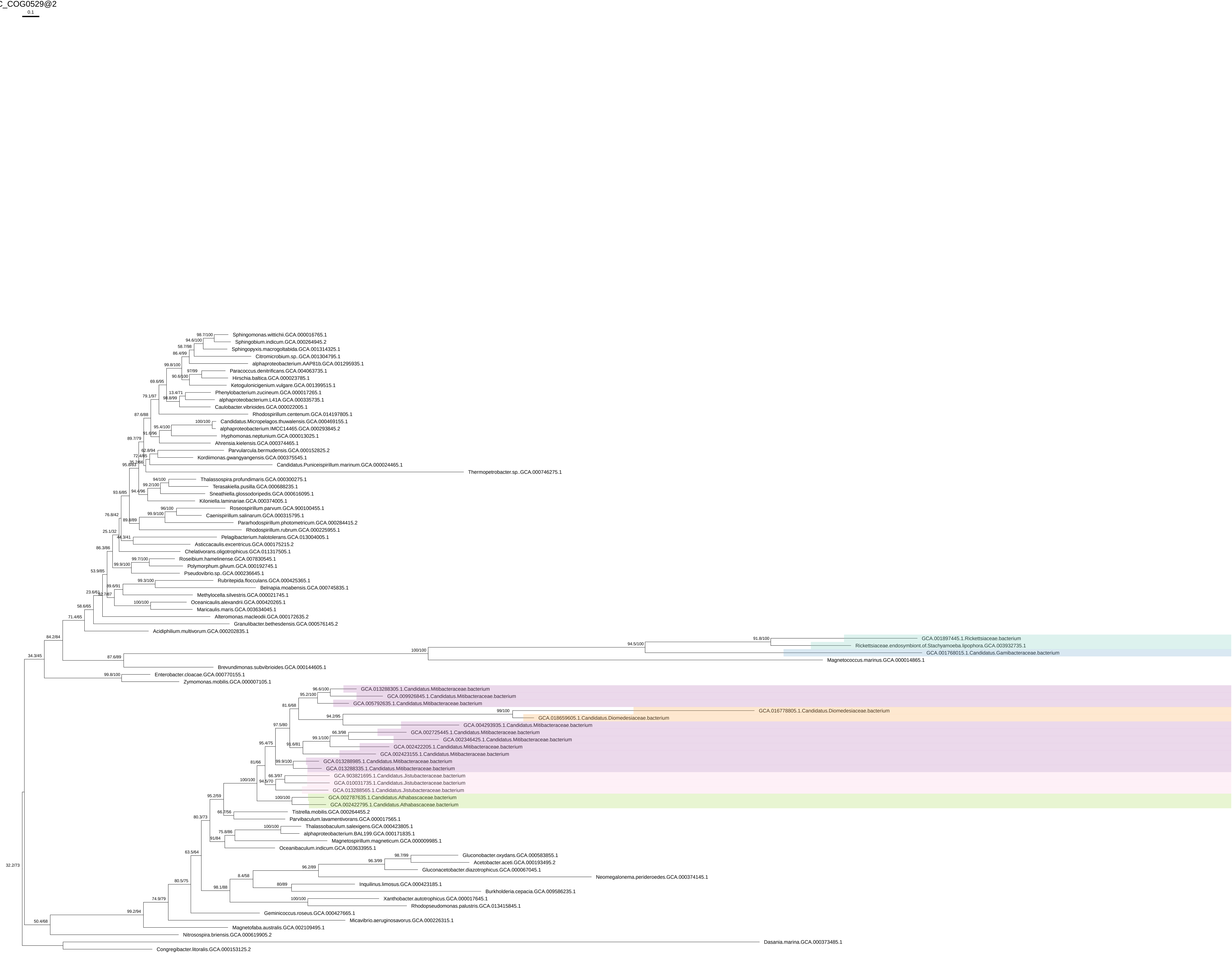

Cys/04_cysH_COG0175@2

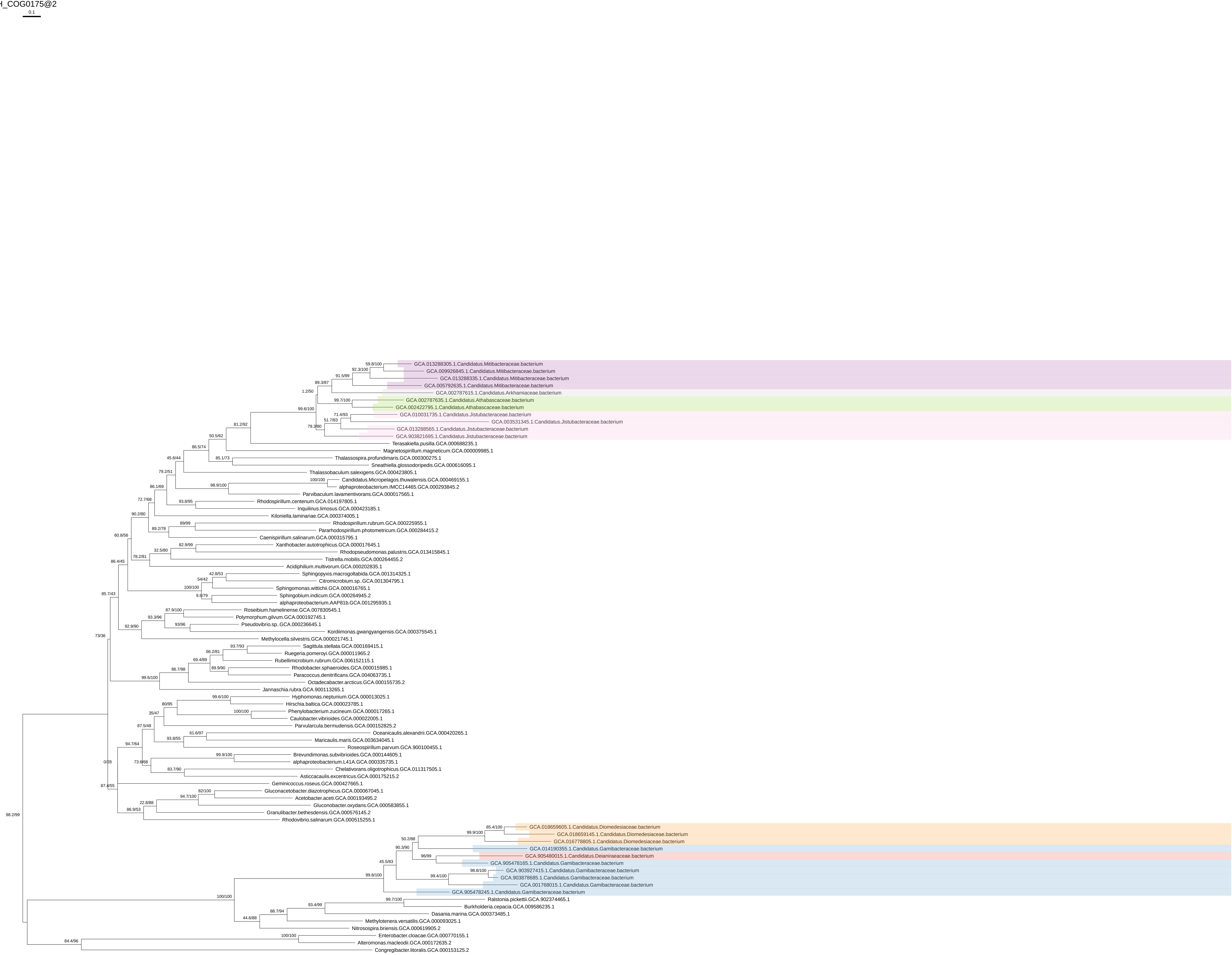

Cys/05_cysJ_COG0369@2

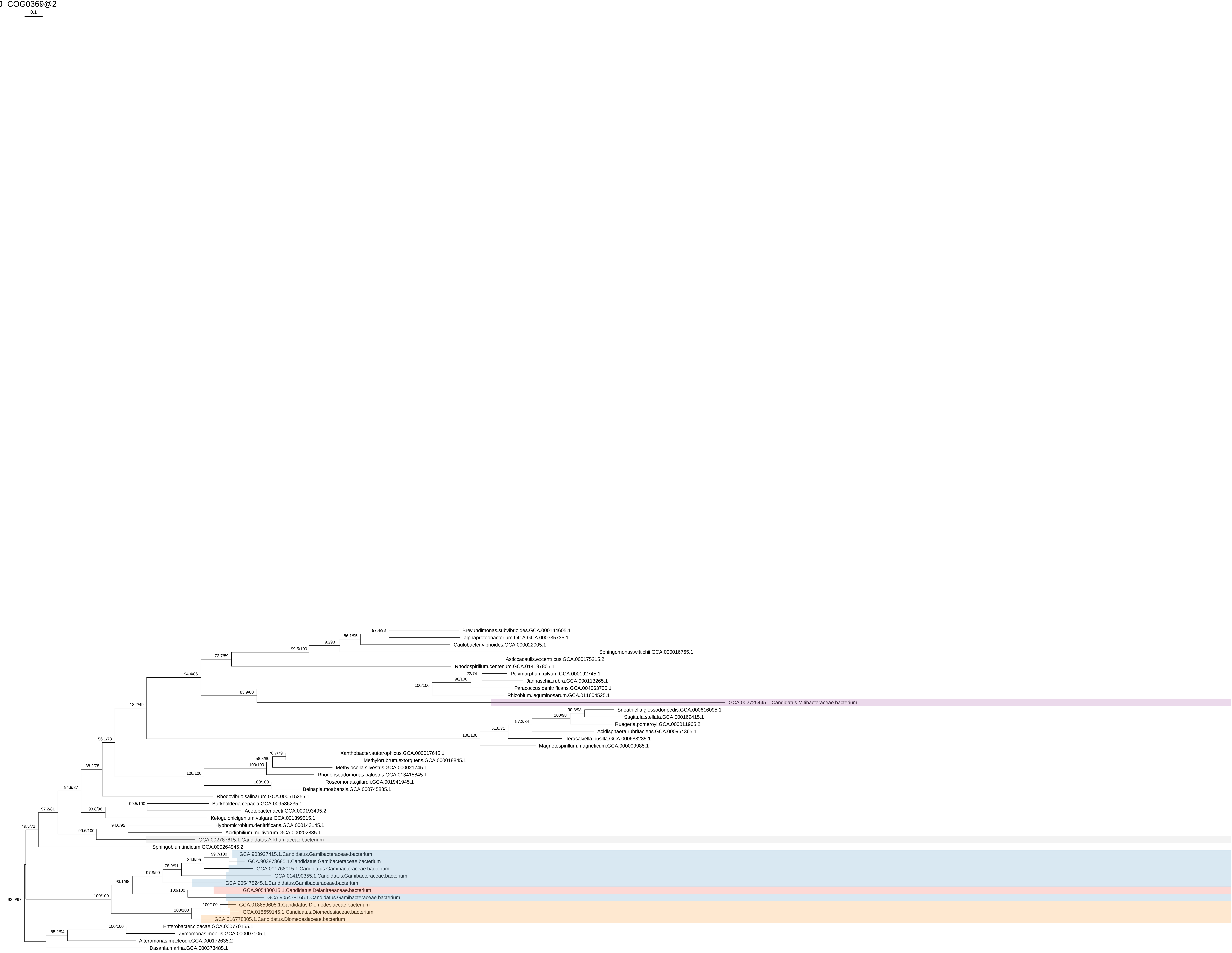

Cys/06_cysI_COG0155@2

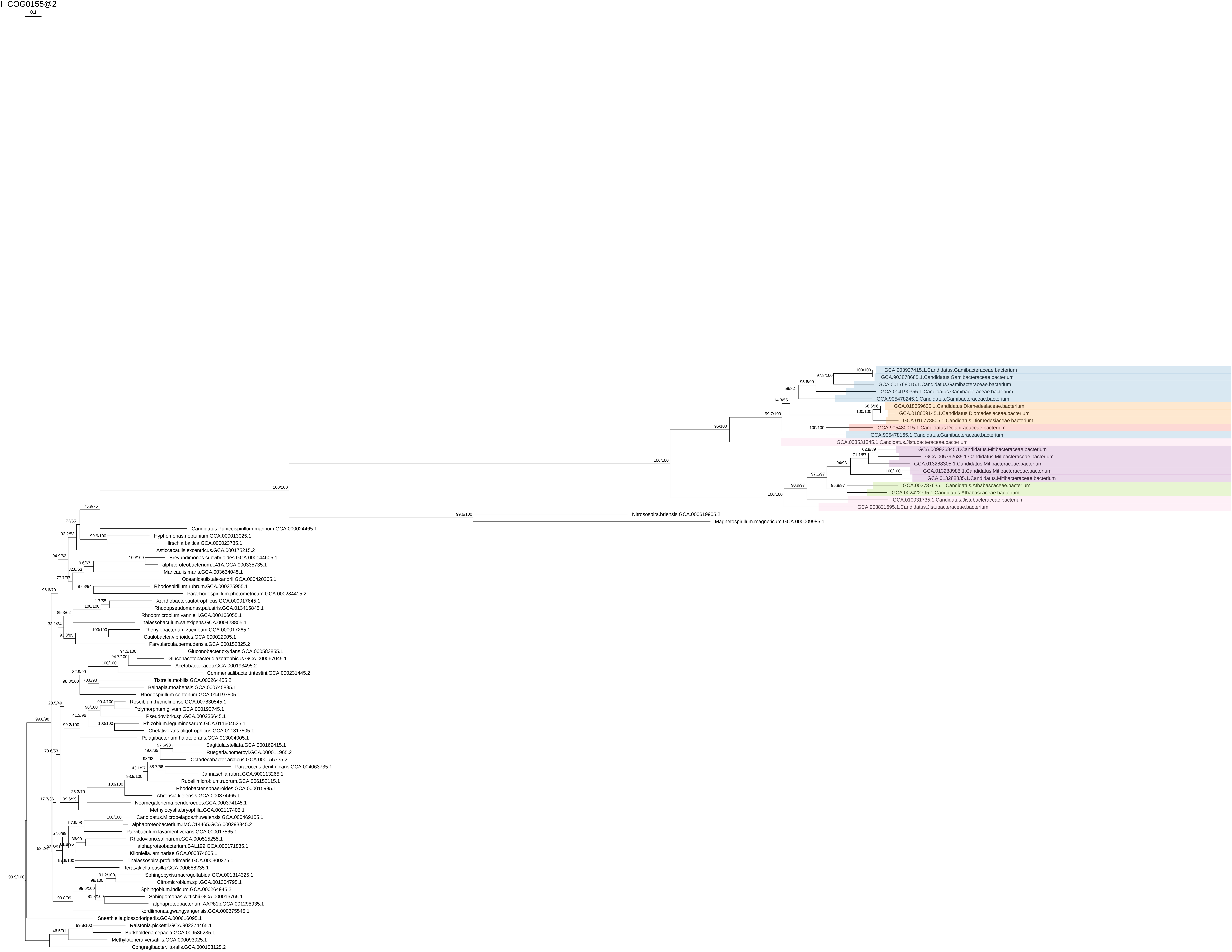

Cys/07_cysE_COG1045@1

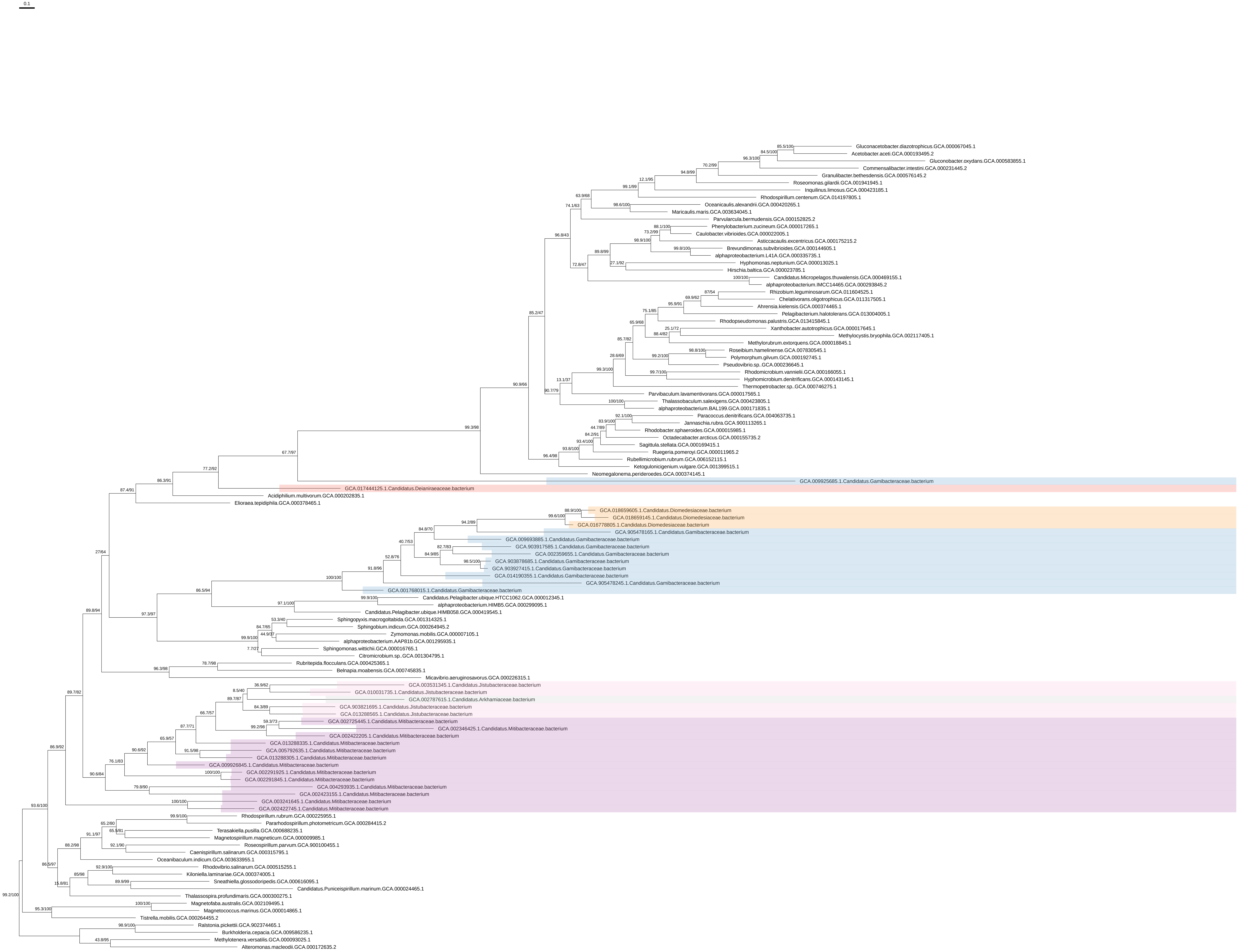

Cys/08_cysKM_COG0031@1

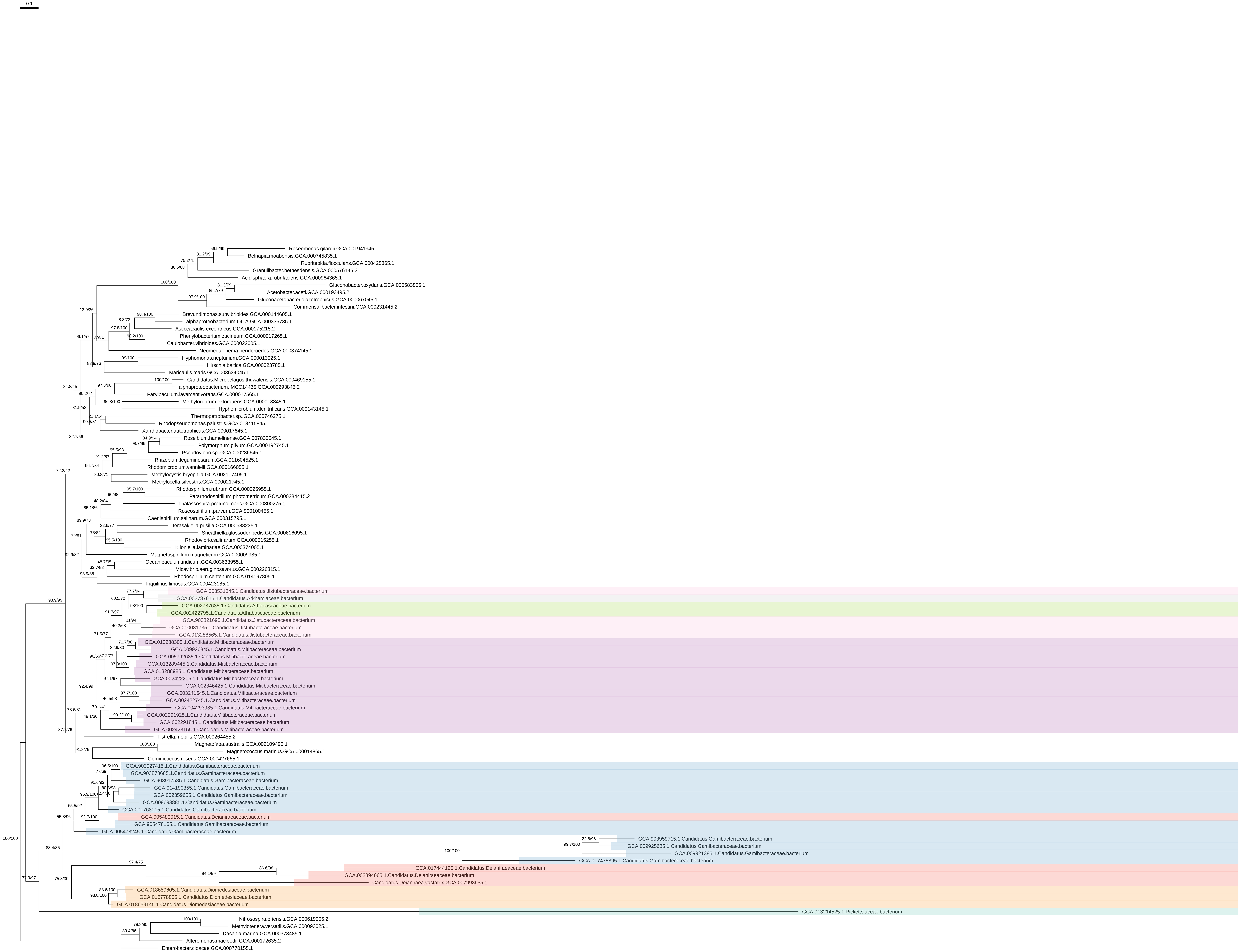

Ile_Val_Leu/01_ilvA_COG1171@2

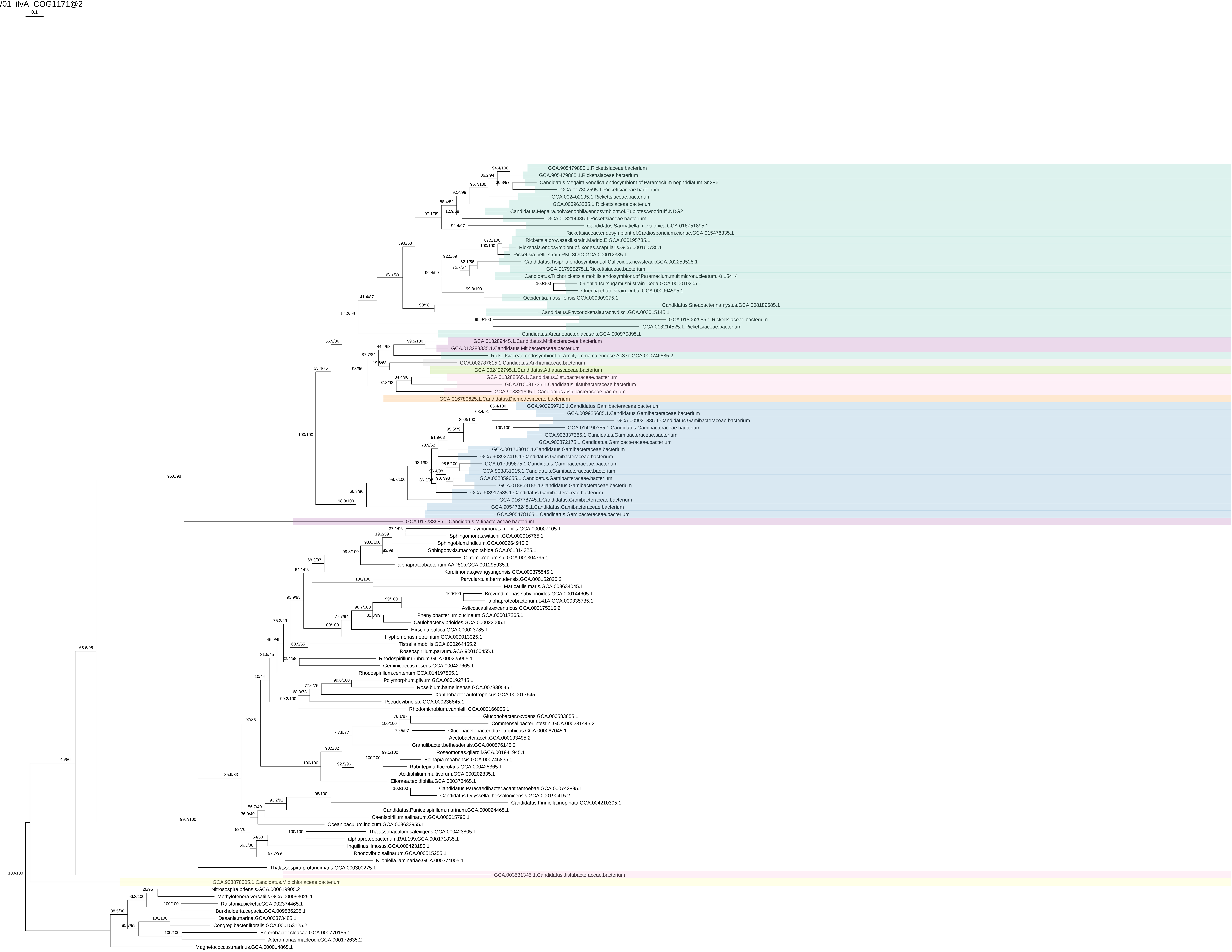

Ile_Val_Leu/02_ilvIB_COG0028@2

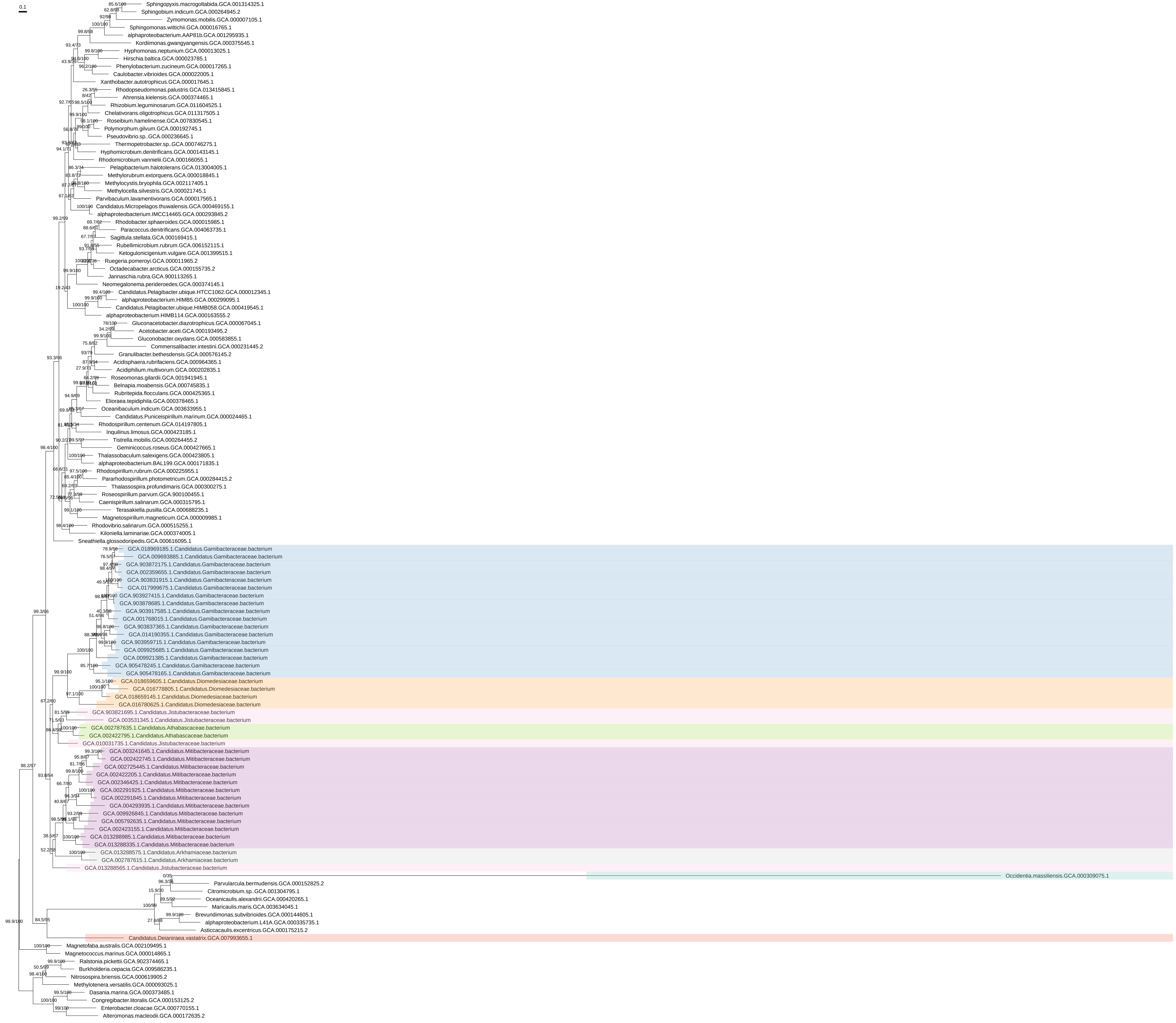

Ile_Val_Leu/03_ilvH_1RAGN@1224

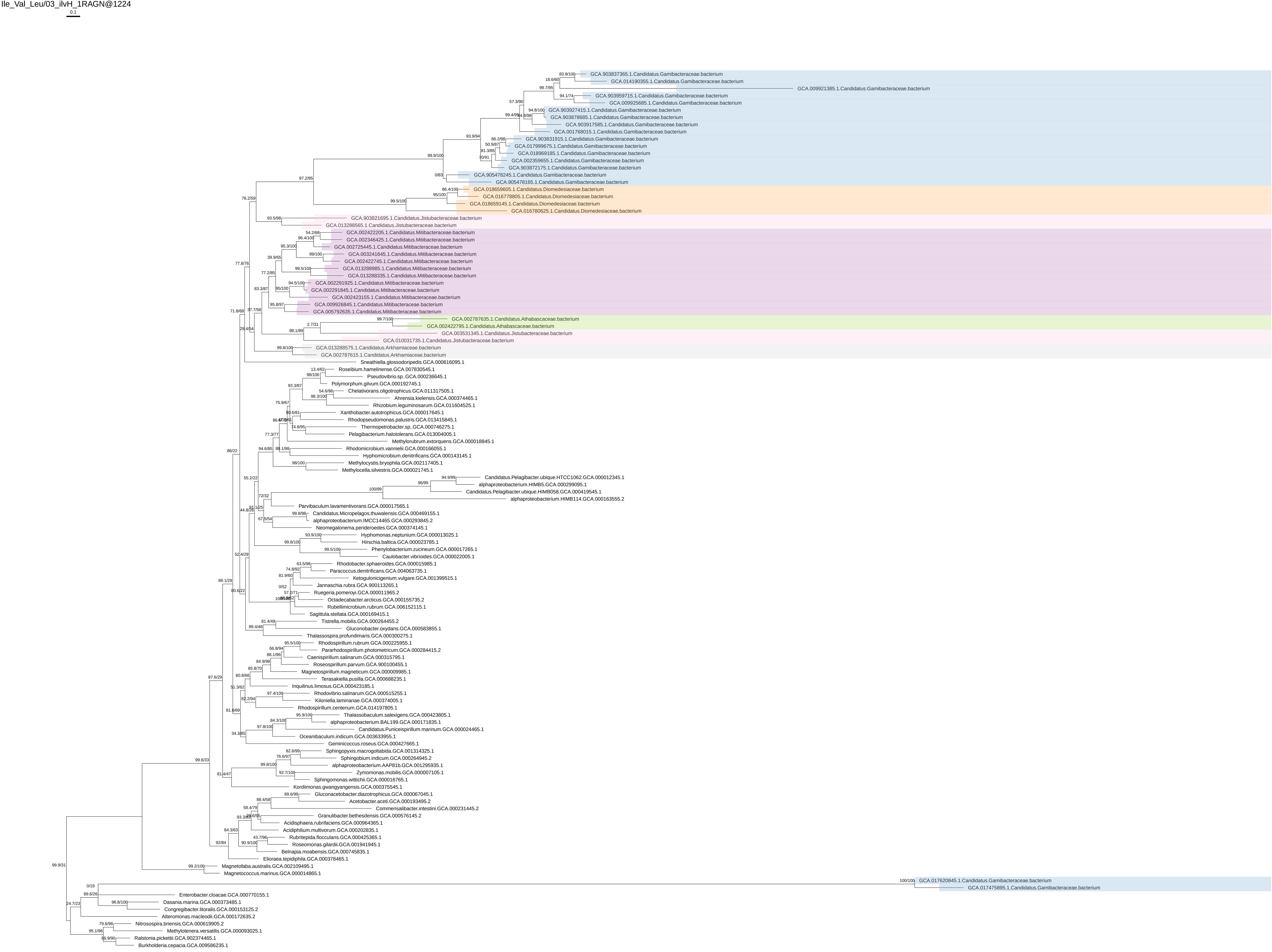

Ile_Val_Leu/04_ilvC_COG0059@2

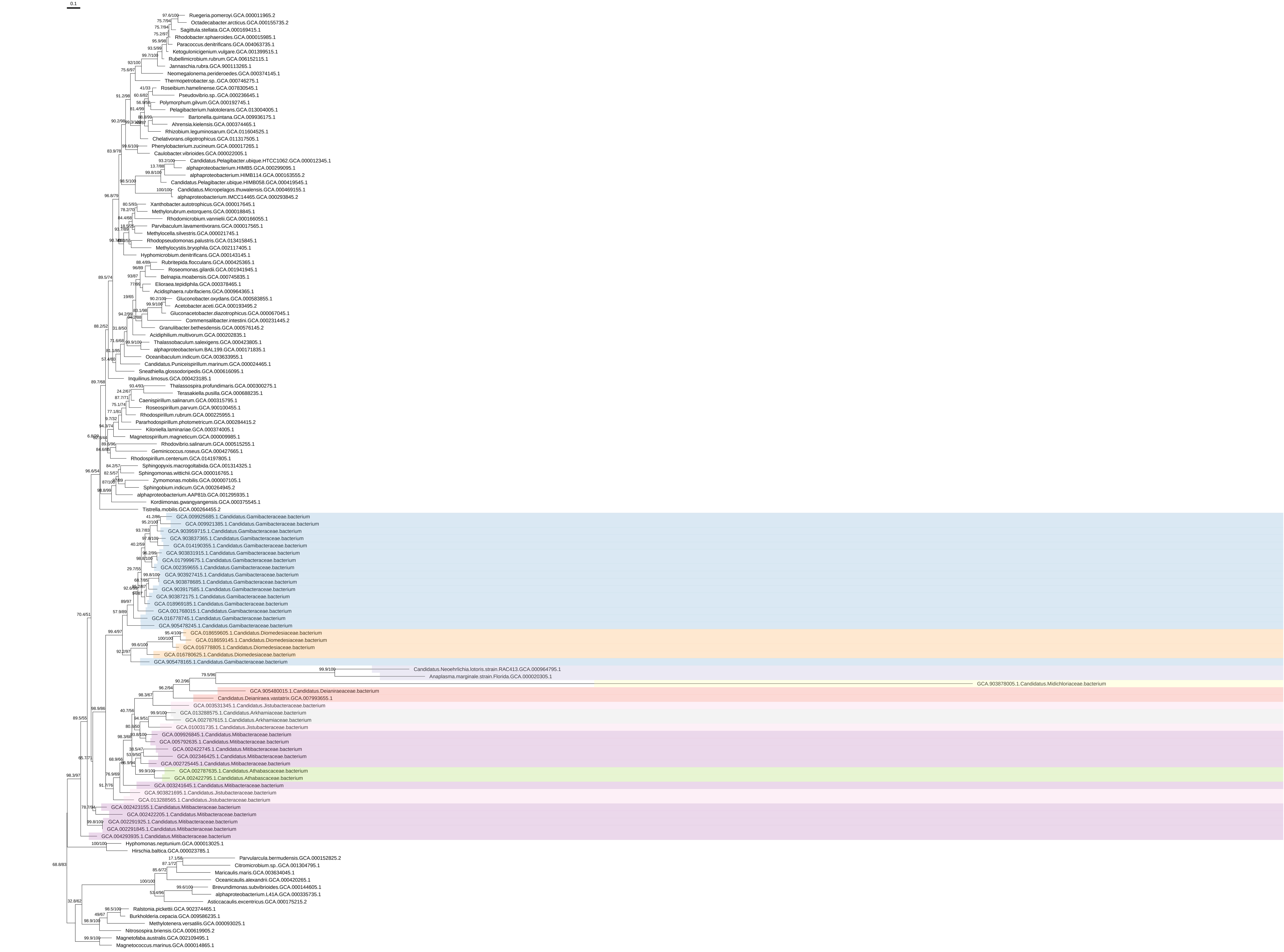

Ile_Val_Leu/05_ilvD_COG0129@2

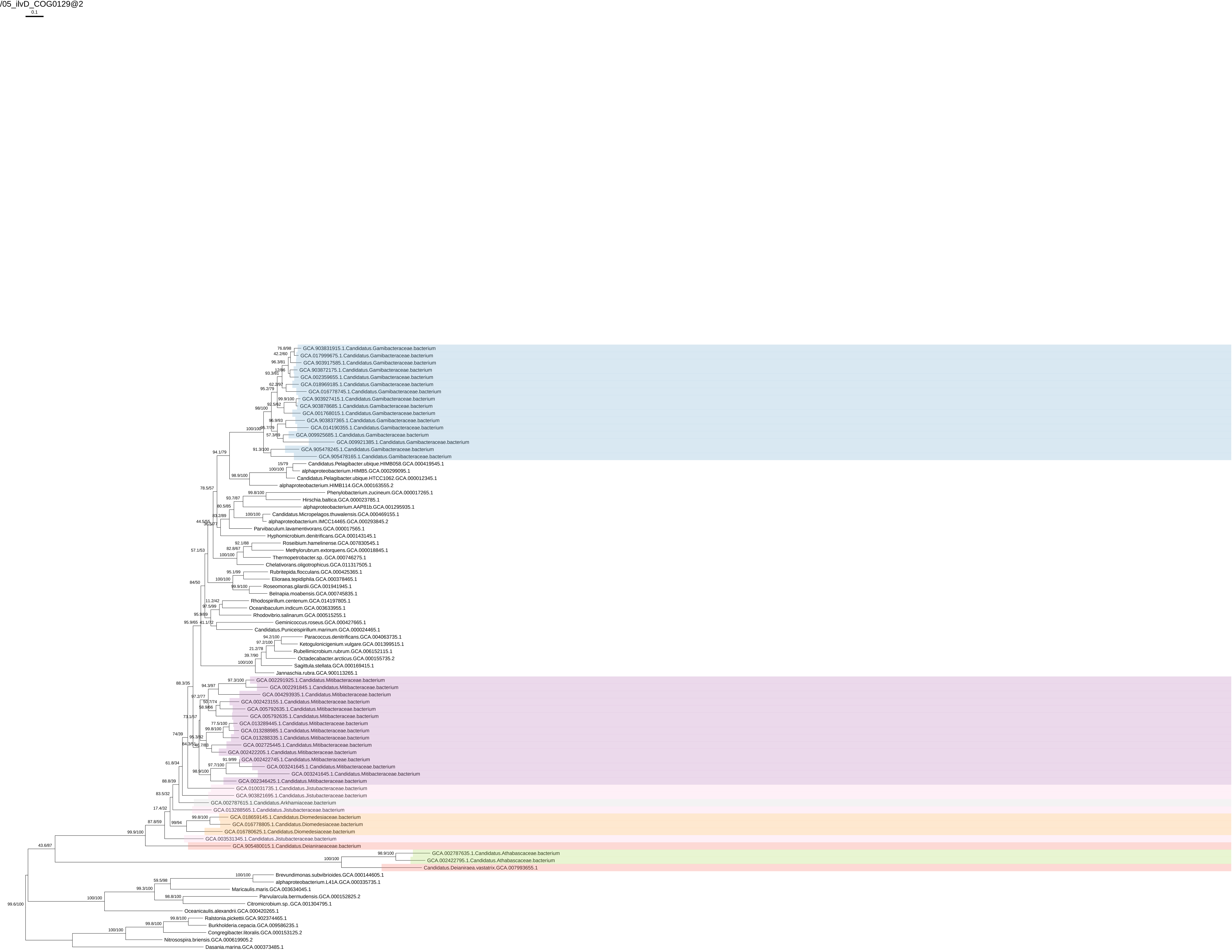

Ile_Val_Leu/06_cimA_COG0119@2

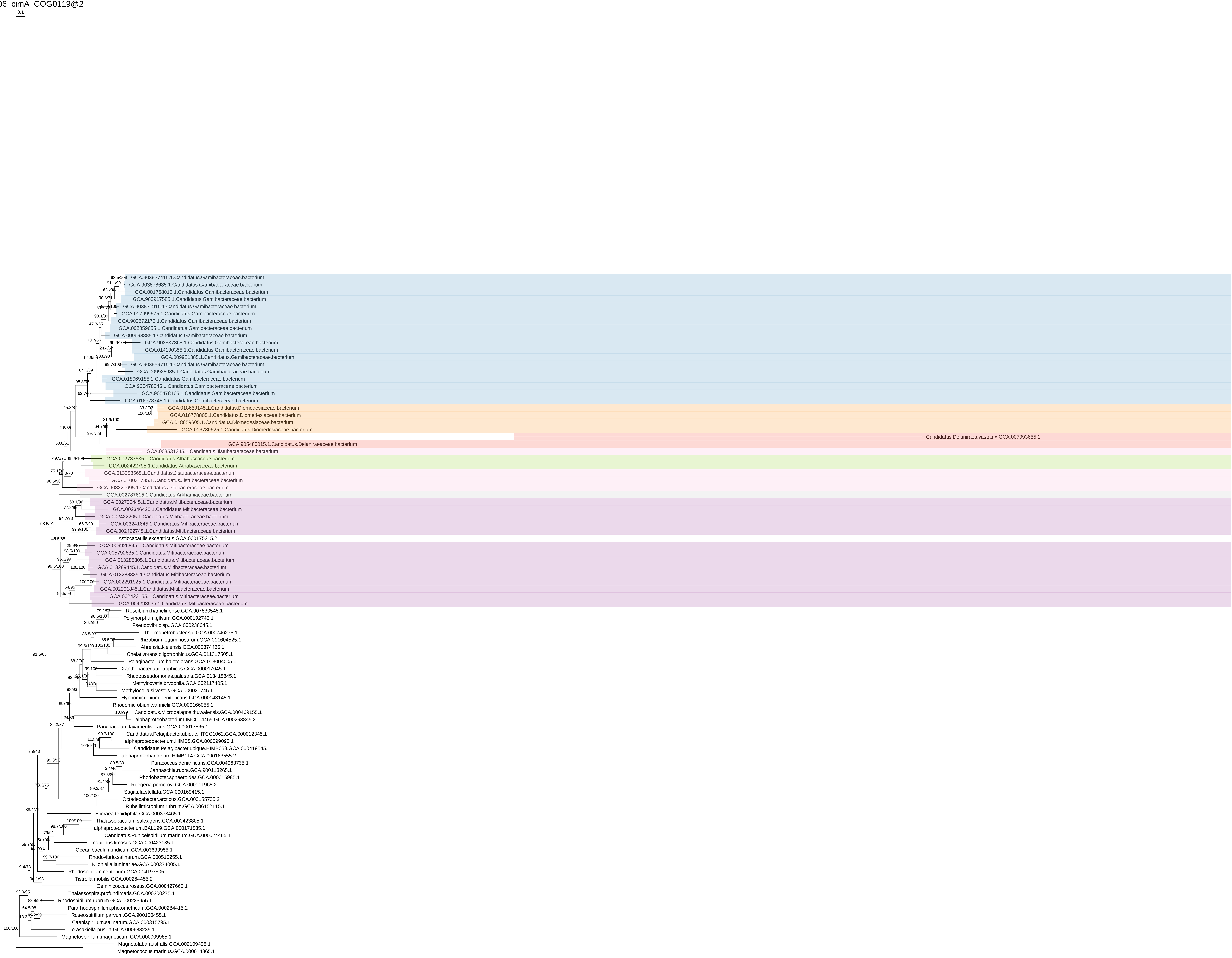

Ile_Val_Leu/07_leuA_COG0119@2

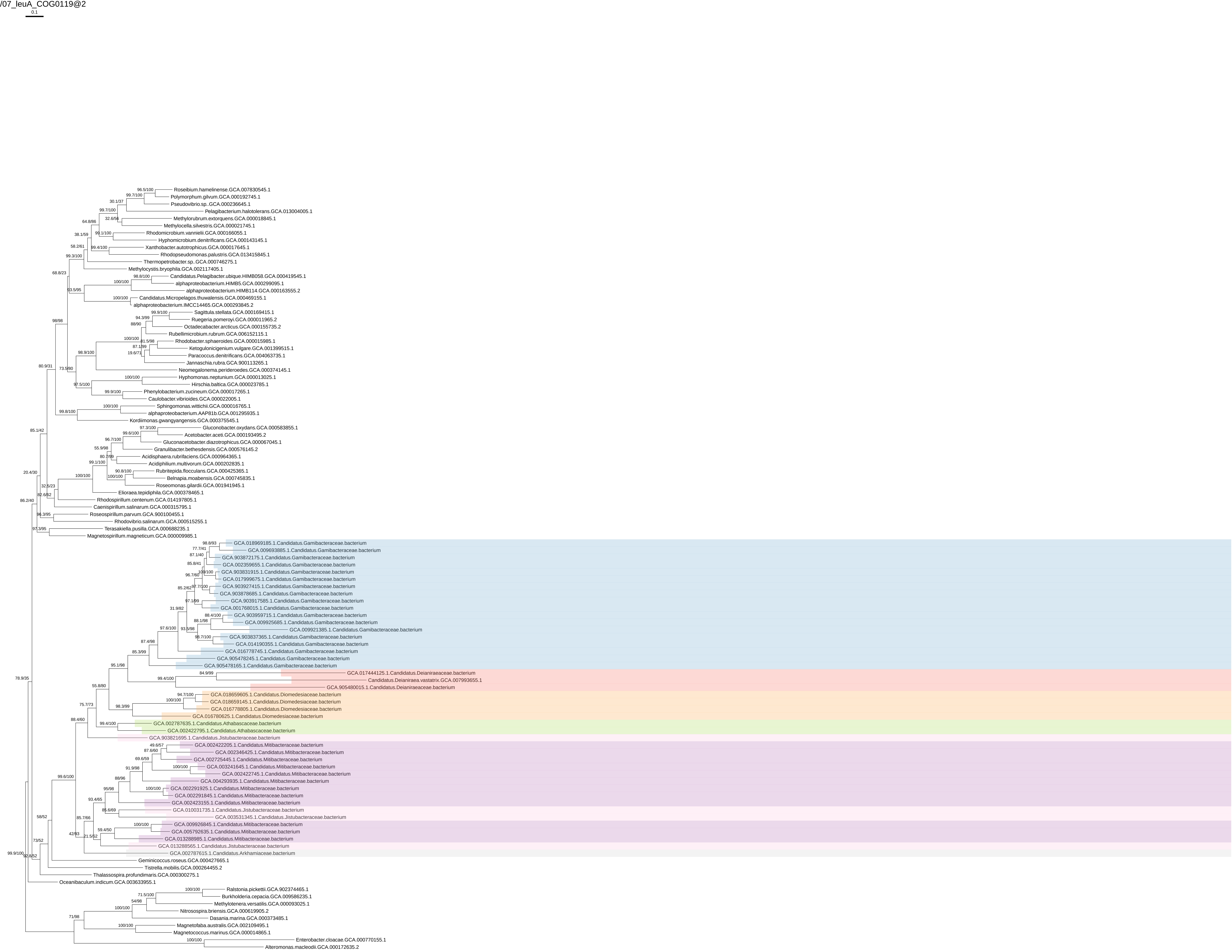

Ile_Val_Leu/08_leuC_COG0065@2

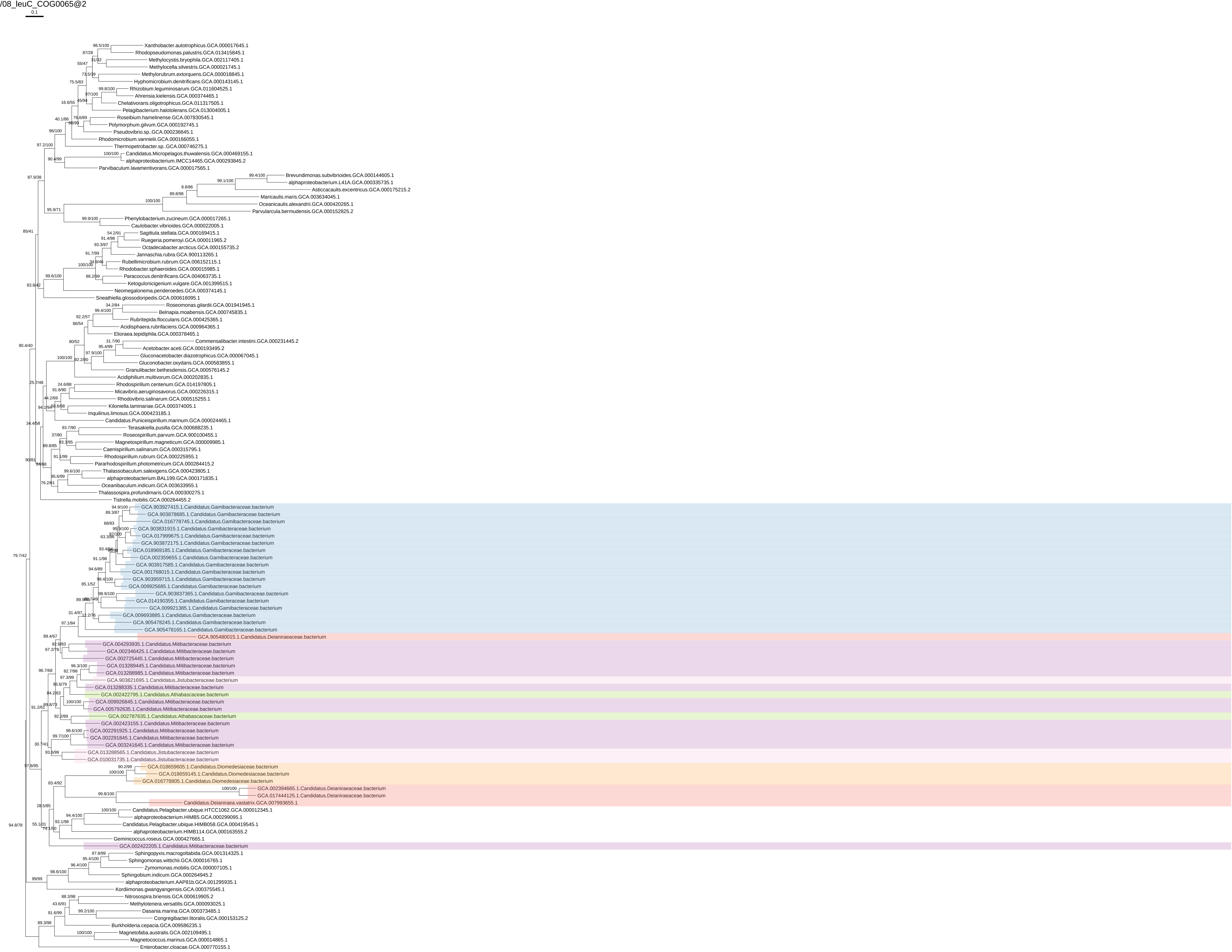

Ile_Val_Leu/09_leuD_COG0066@2

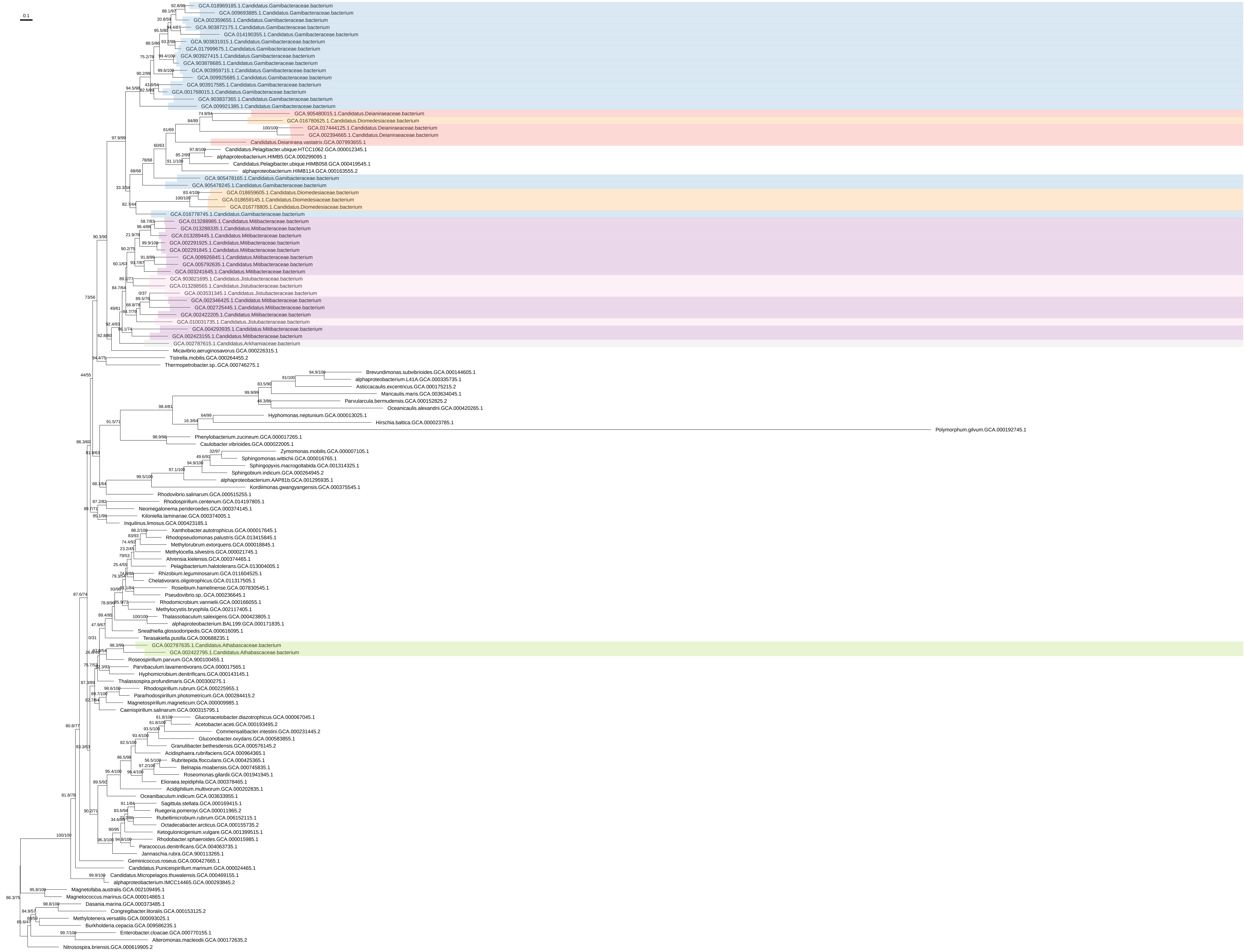

Ile_Val_Leu/10_leuB_COG0473@2

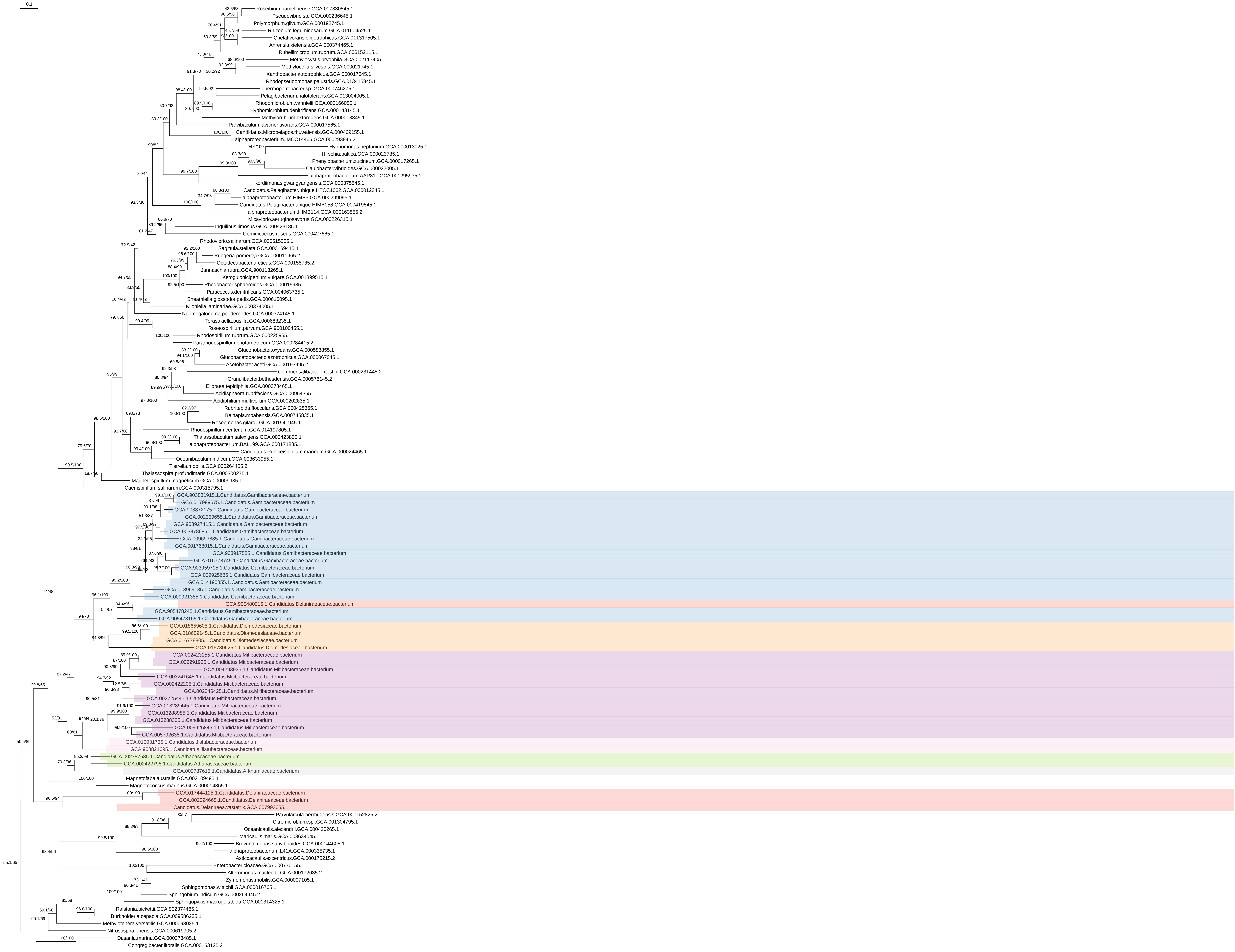

Trp_Tyr_Phe/01_aroF_COG3200@1

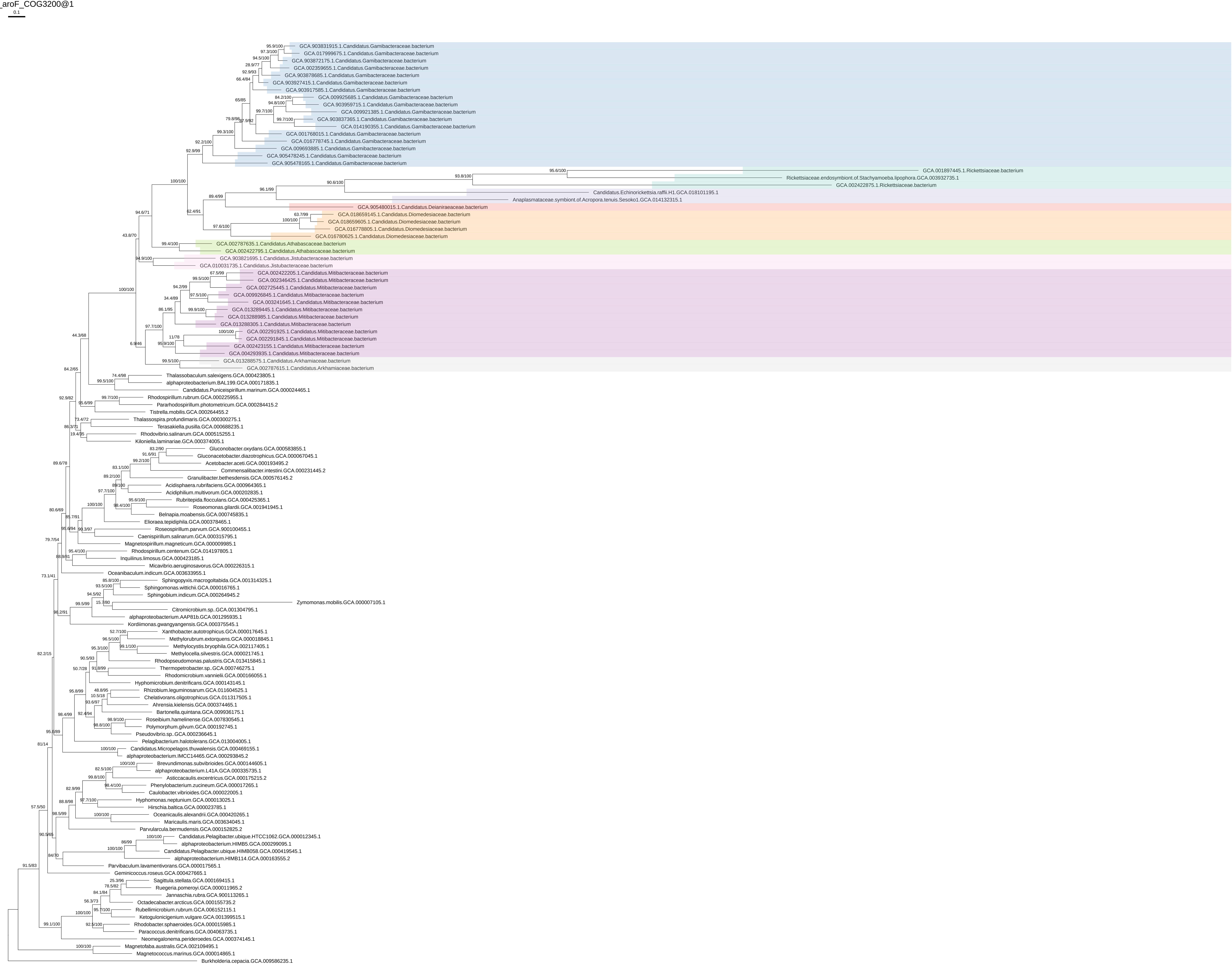

Trp_Tyr_Phe/02_aroB_COG0337@1

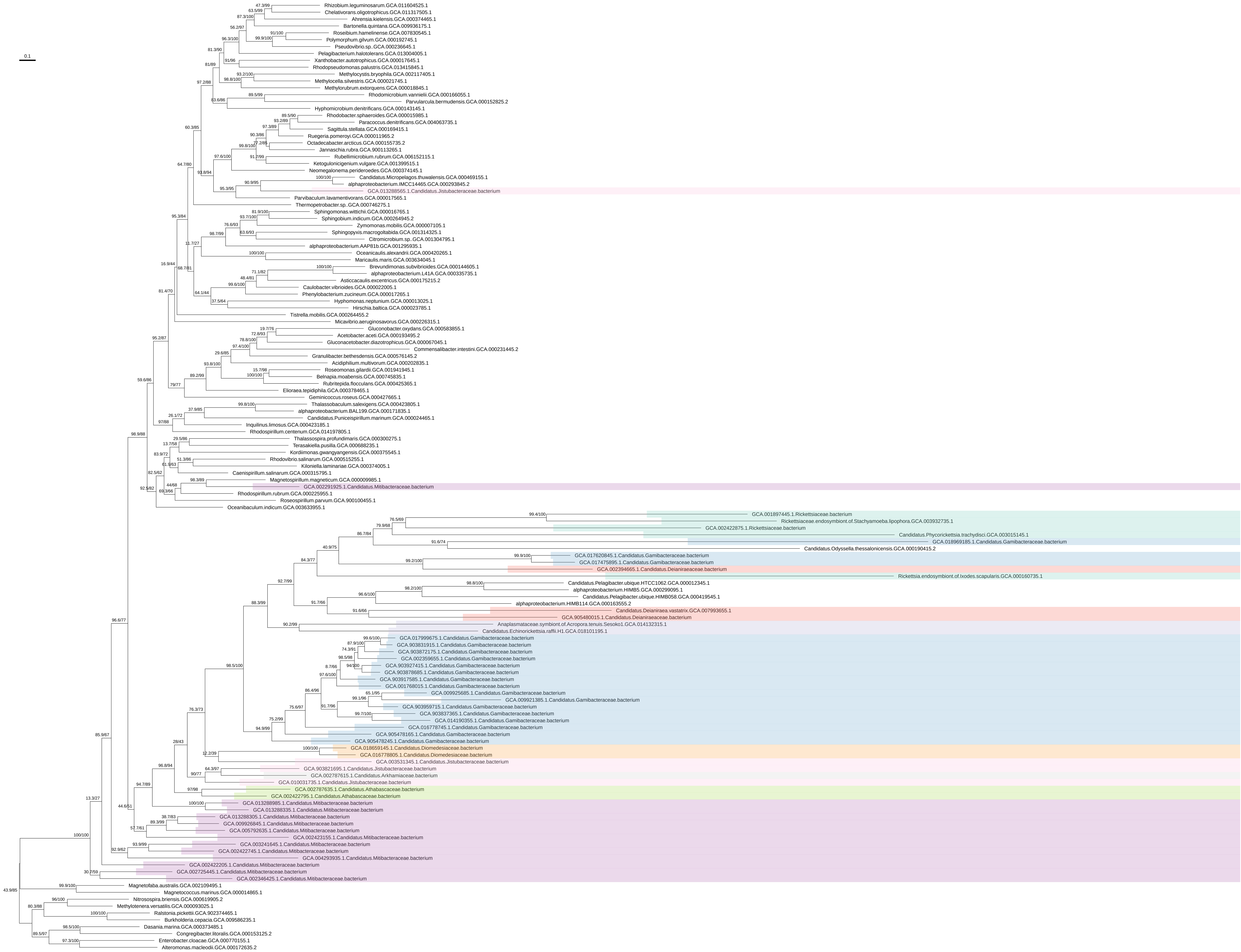

Trp_Tyr_Phe/03_aroQ_COG0757@1

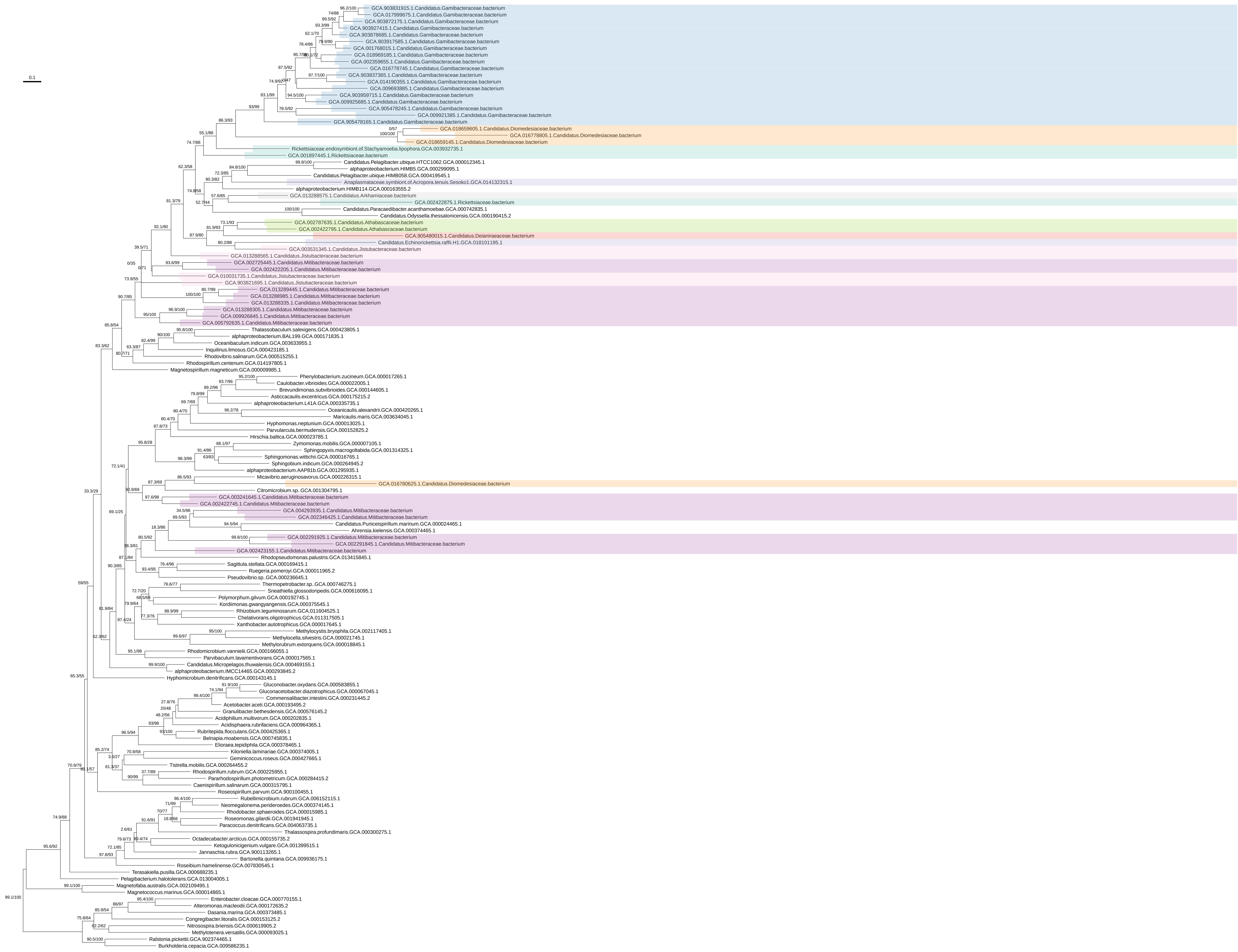

Trp_Tyr_Phe/04_aroE_COG0169@1

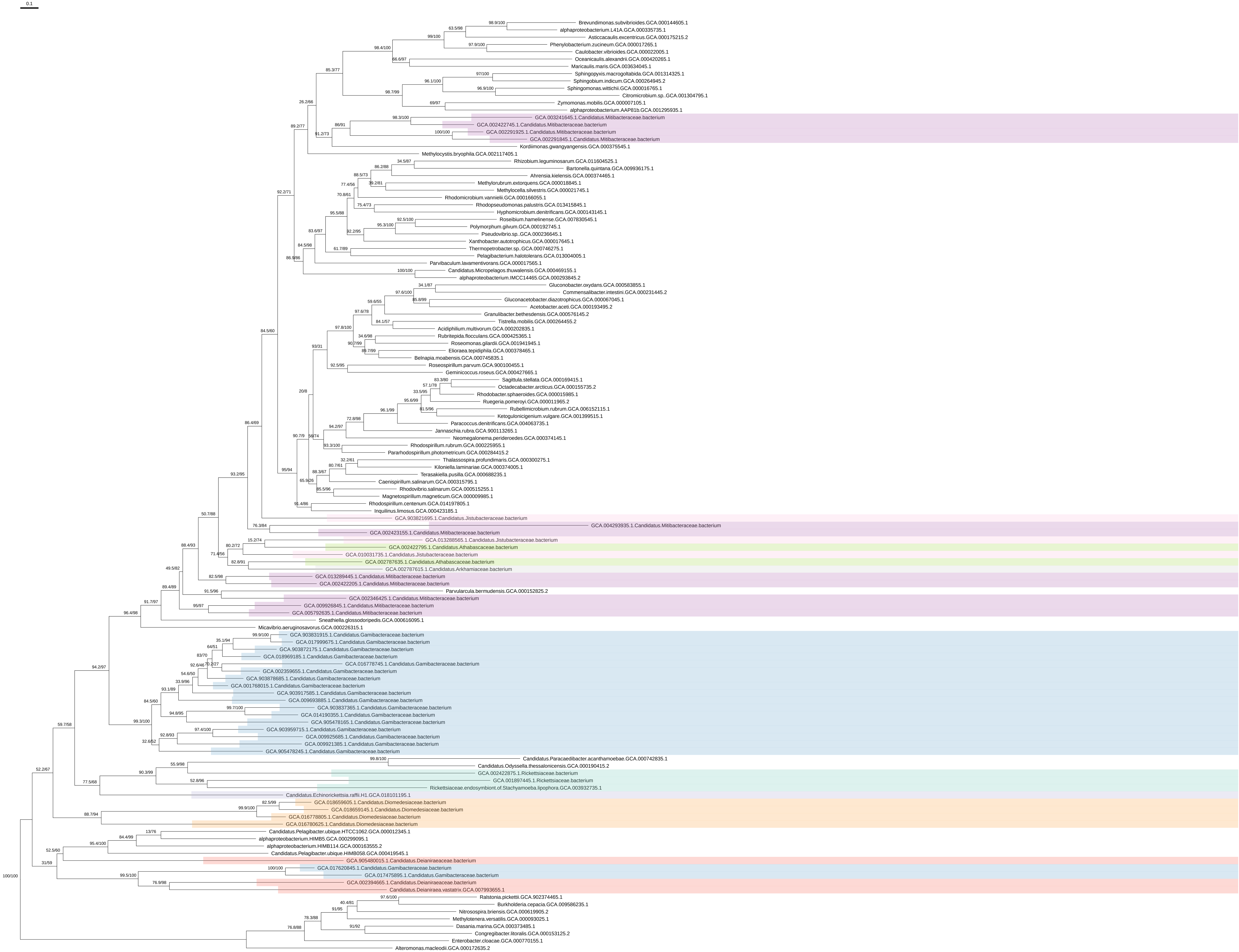

Trp_Tyr_Phe/05_aroK_COG0703@1

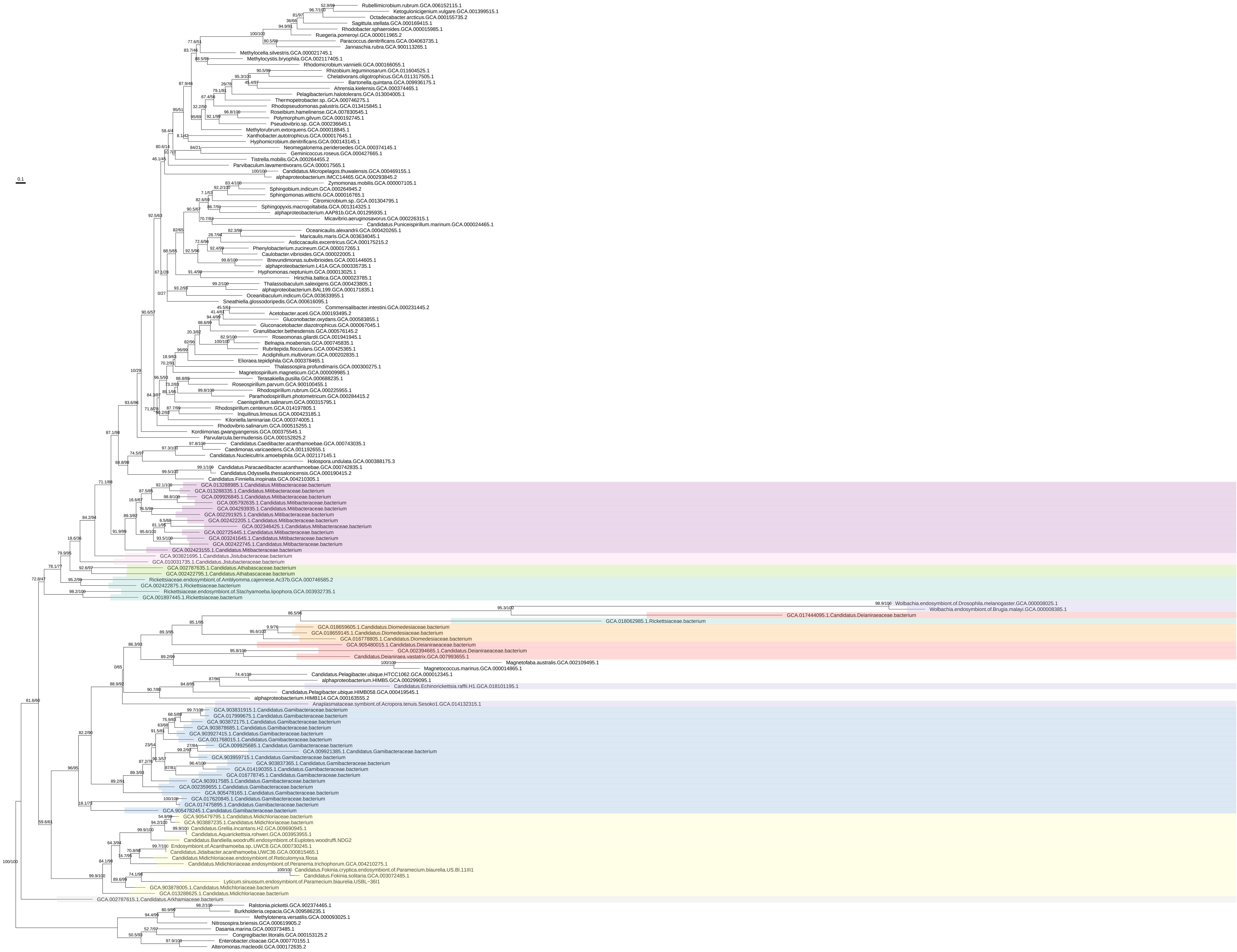

Trp_Tyr_Phe/06_aroA_COG0128@2

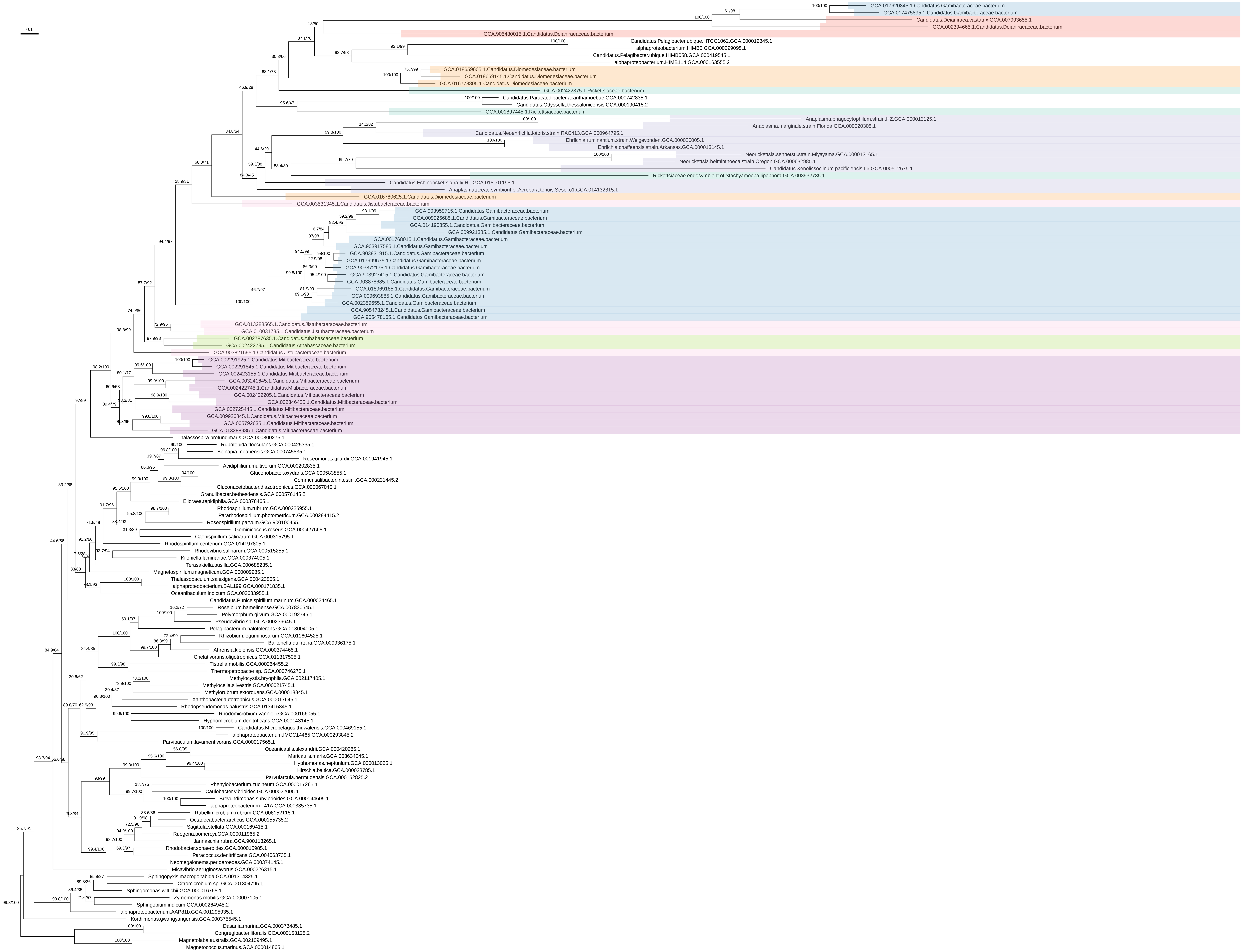

Trp_Tyr_Phe/07_aroC_COG0082@2

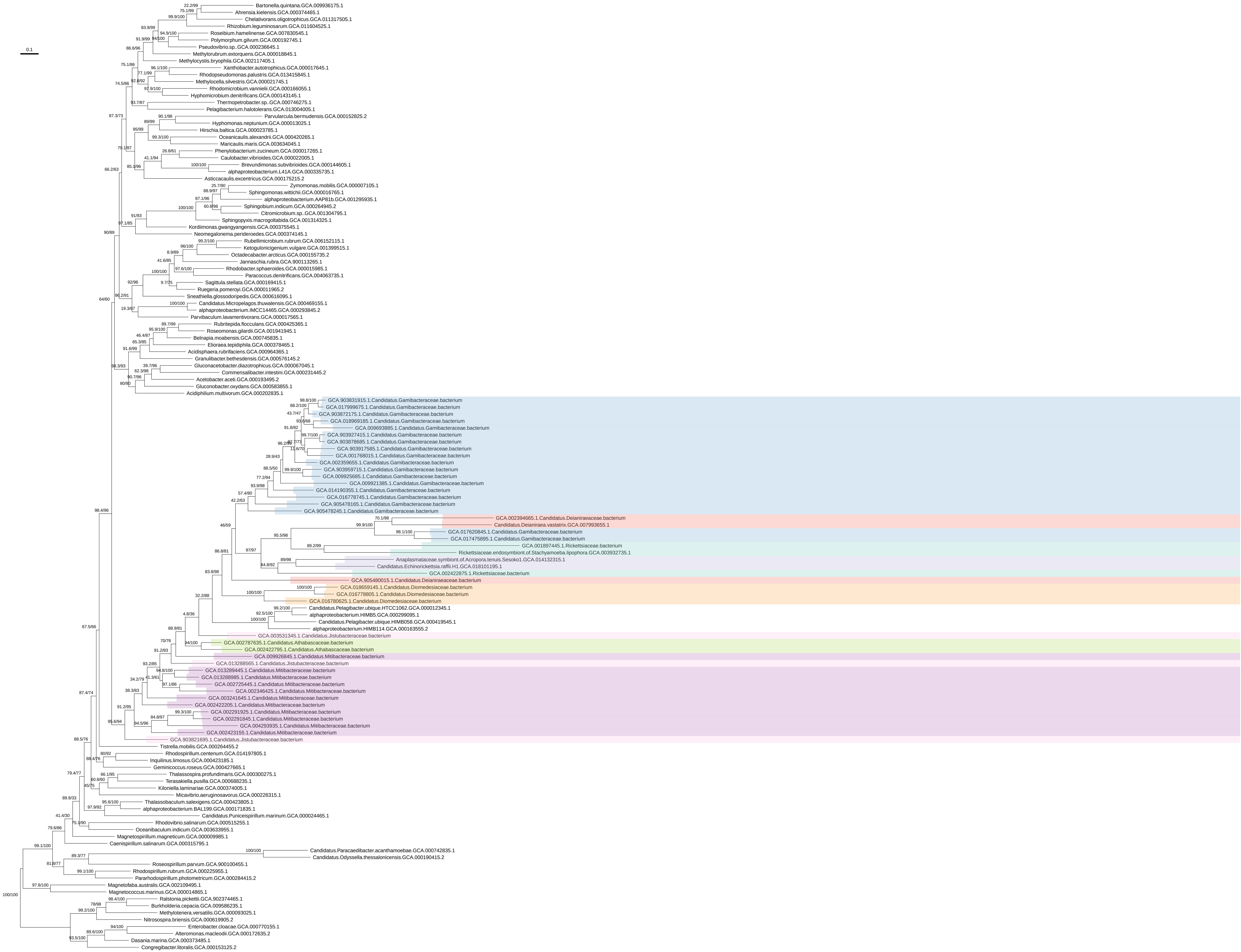

Trp_Tyr_Phe/08_trpE_COG0147@2

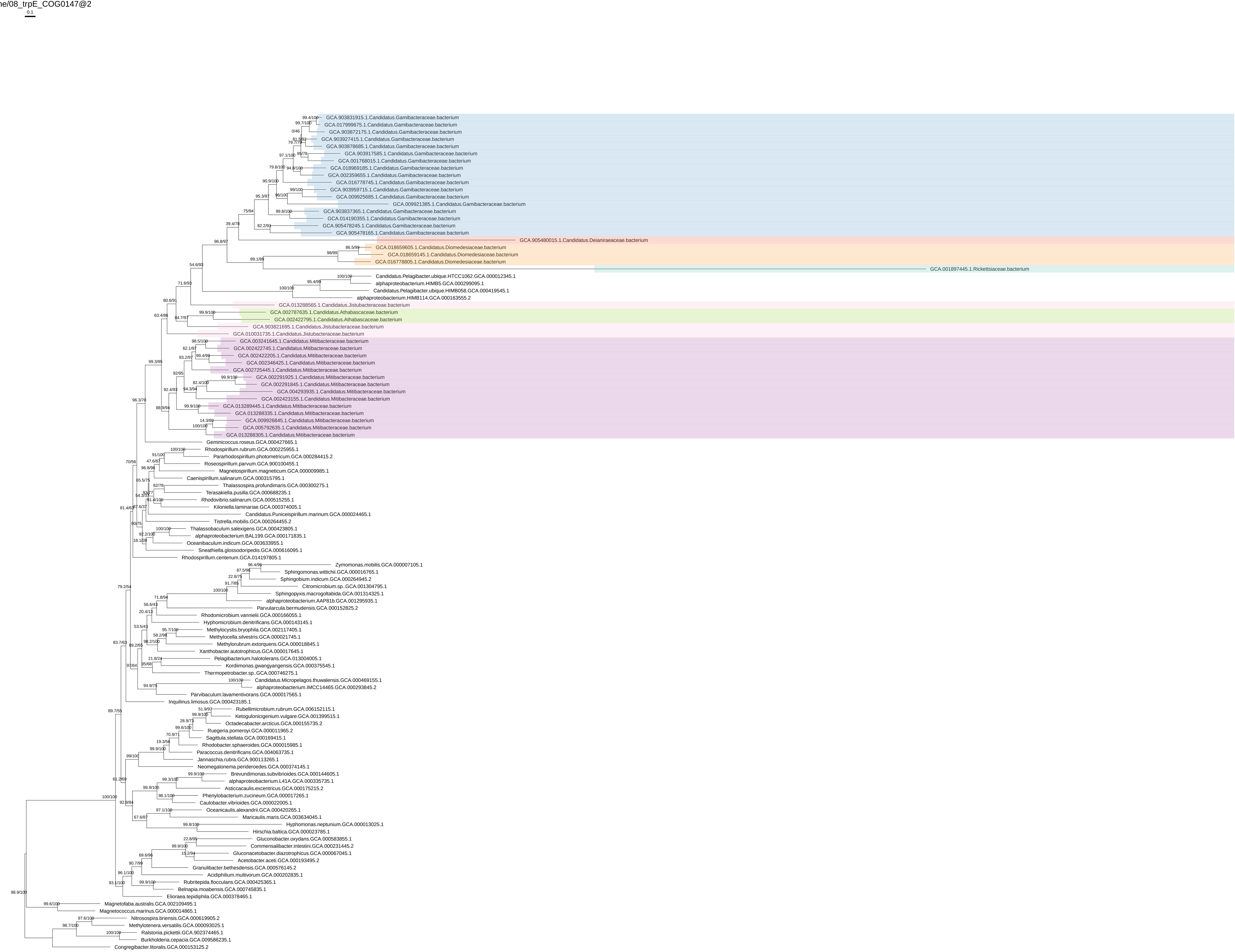

Trp_Tyr_Phe/09_trpD_COG0547@2

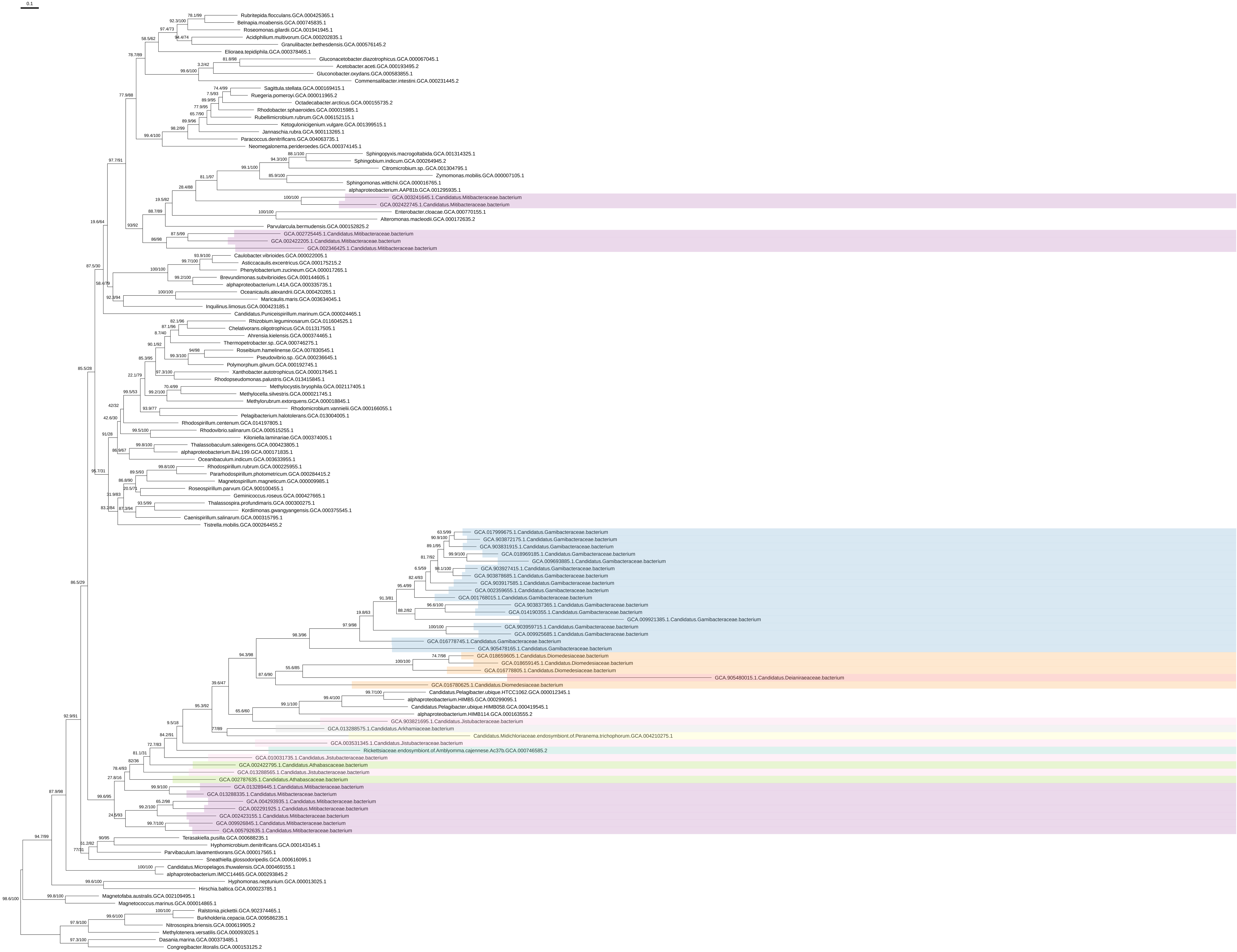

Trp_Tyr_Phe/10_trpF_COG0135@2

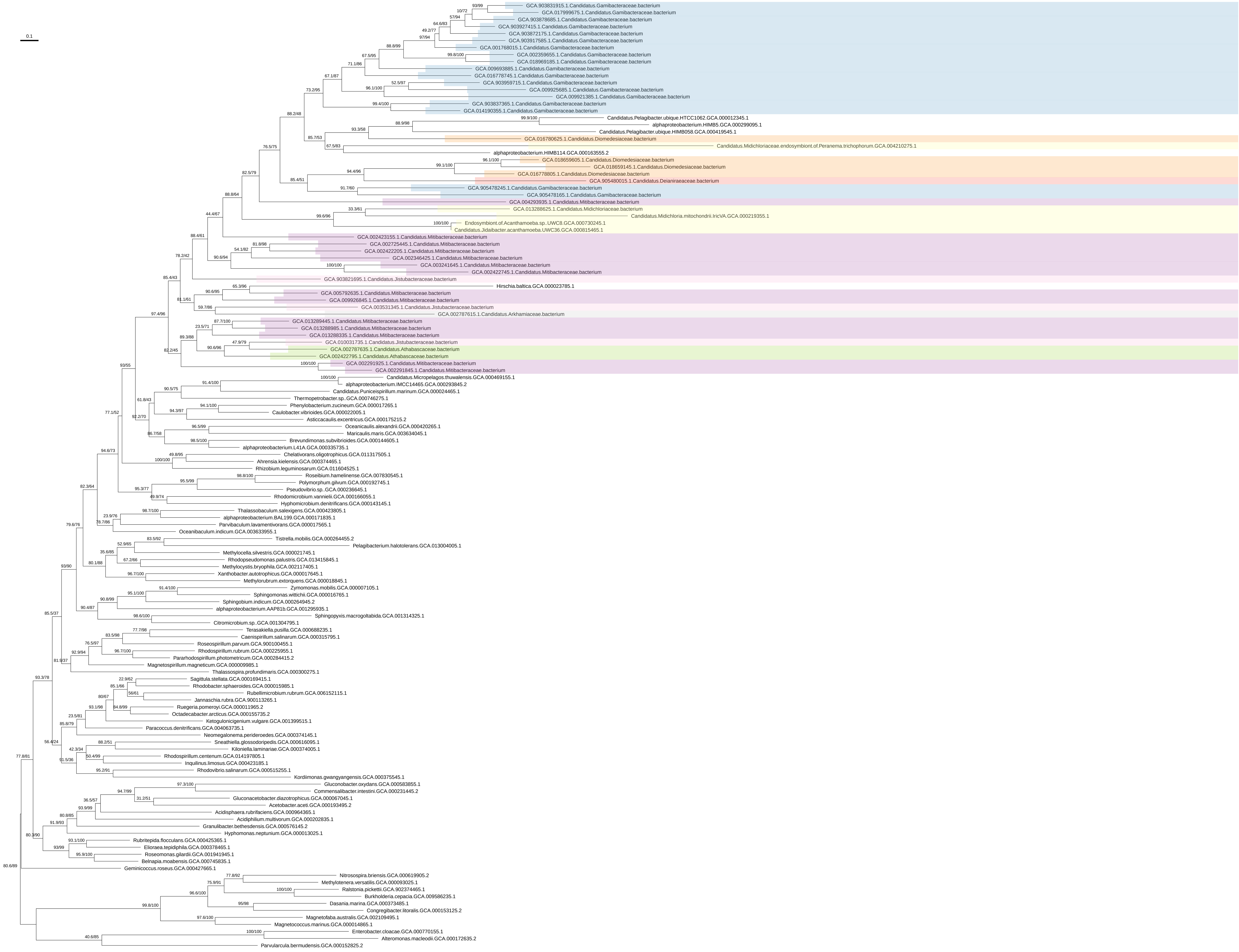

Trp_Tyr_Phe/11_trpC_1MW5K@1224

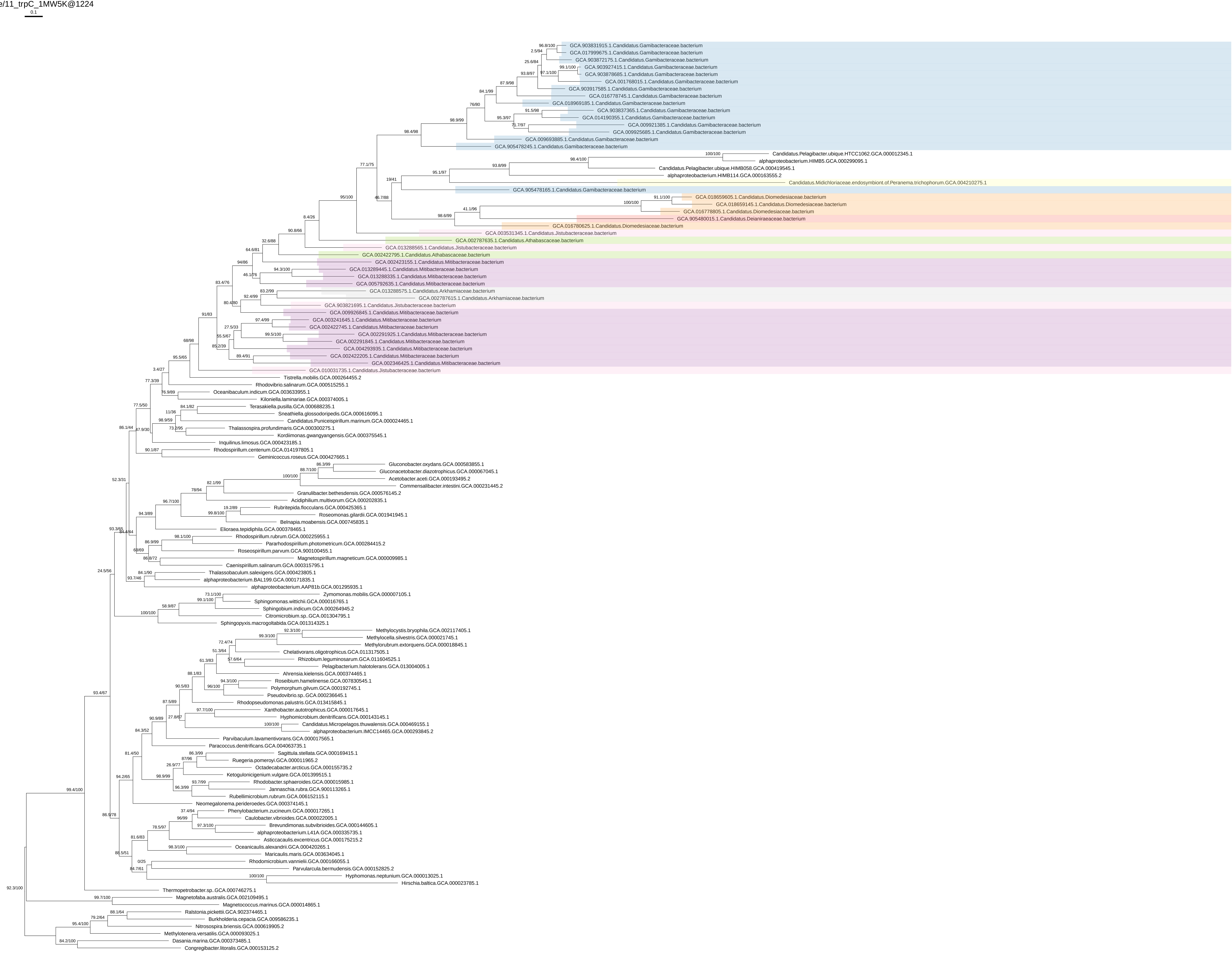

Trp_Tyr_Phe/12_trpA_COG0159@2

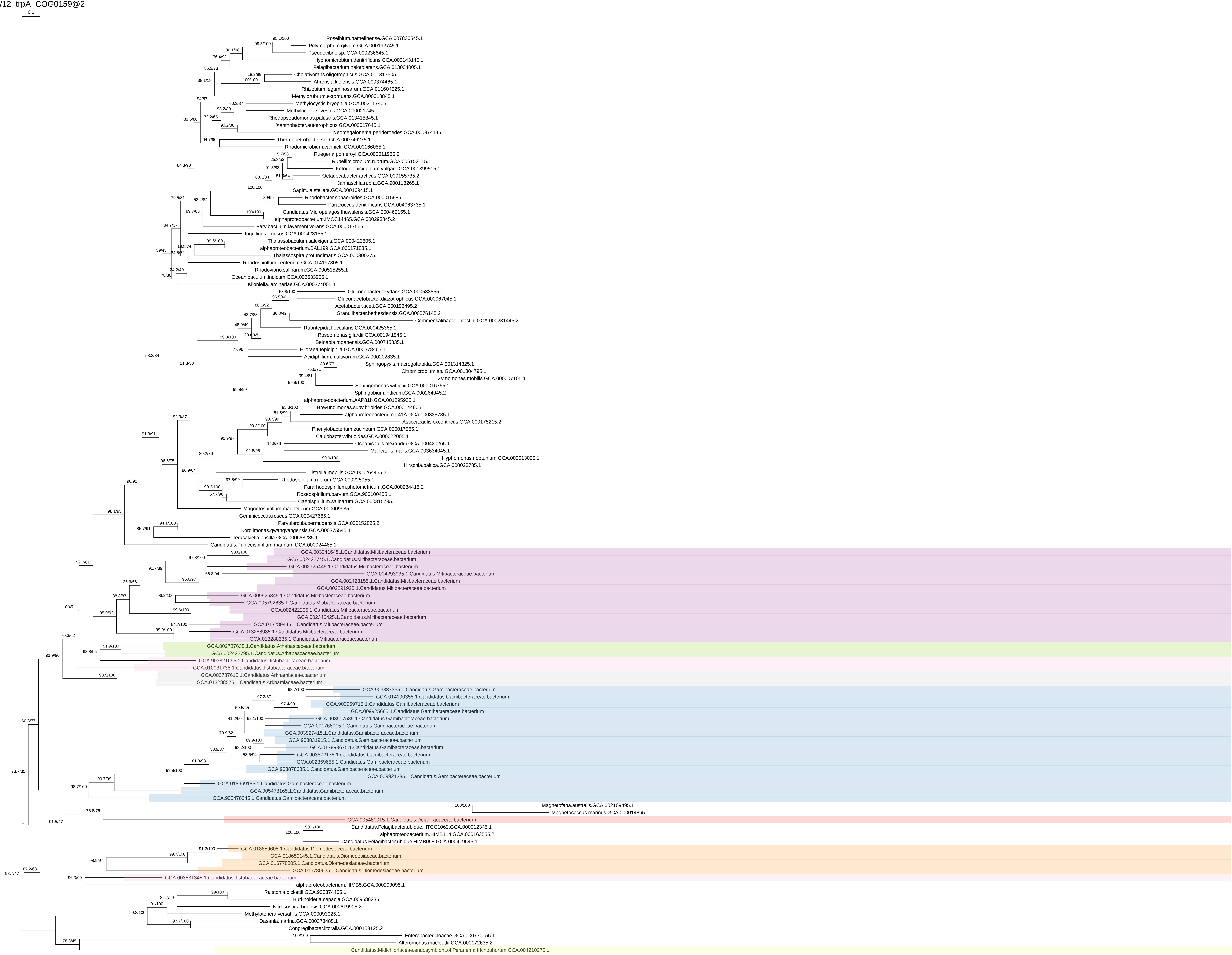

Trp_Tyr_Phe/13_trpB_COG0133@2

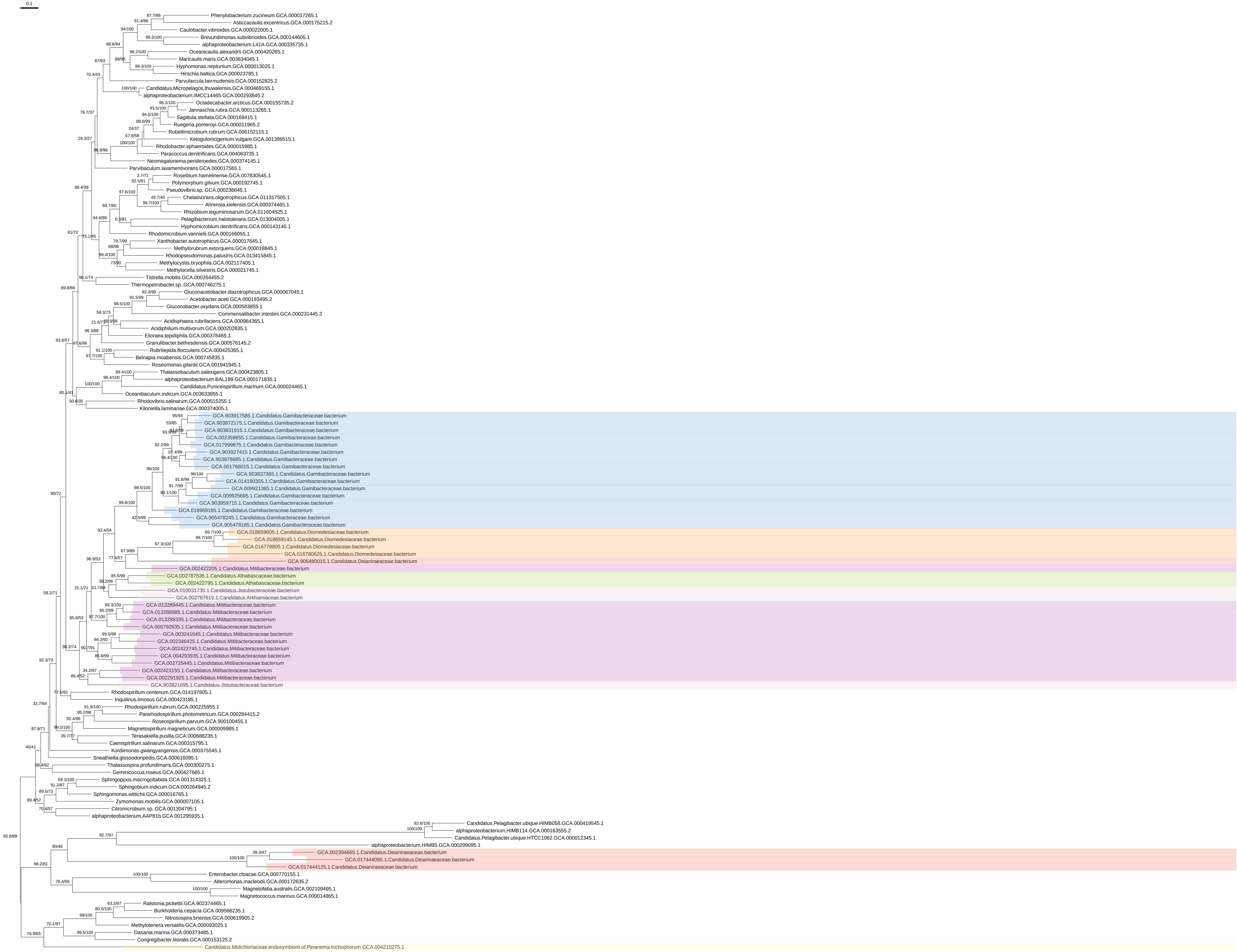

Trp_Tyr_Phe/14_pheA_COG0077@2

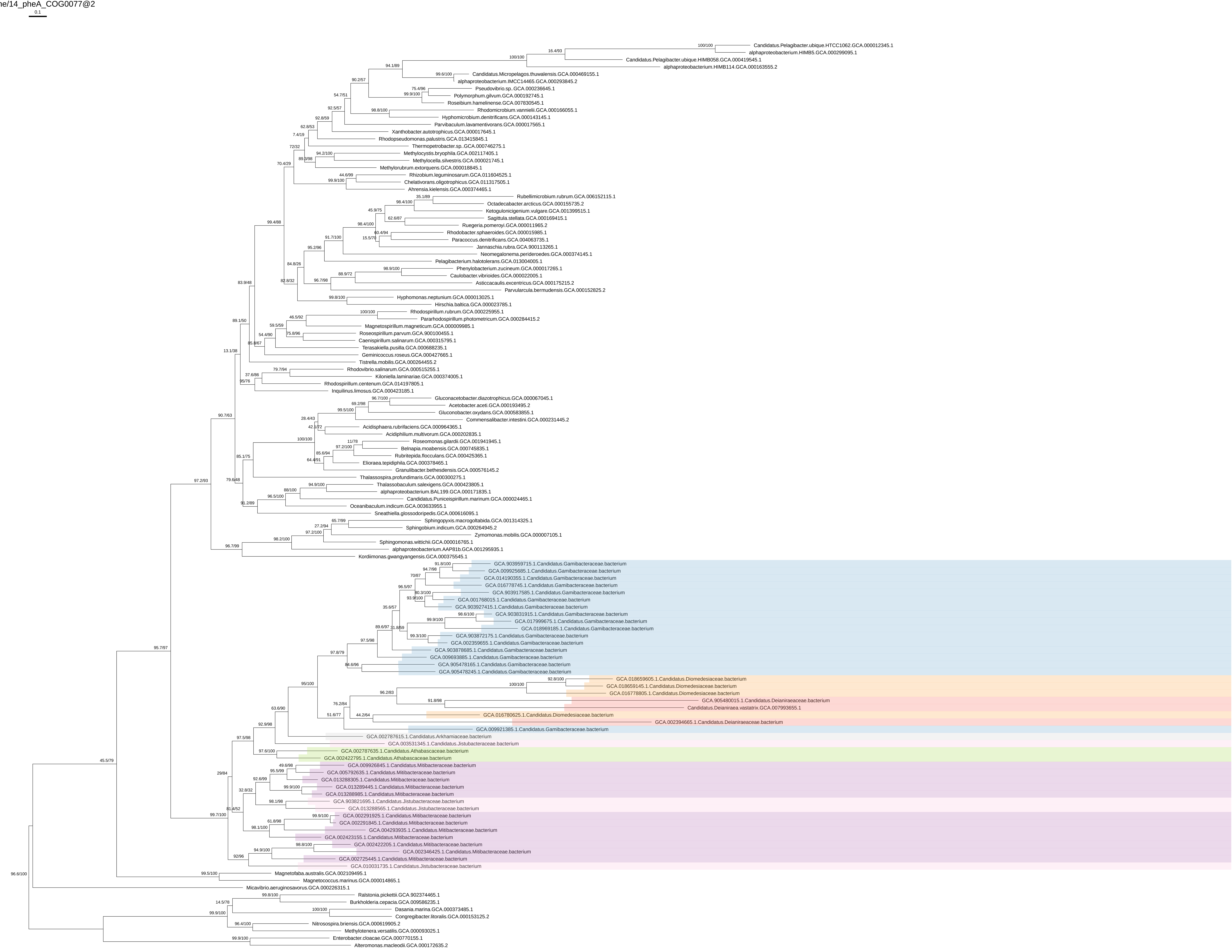

Trp_Tyr_Phe/15_tyrA_COG0287@2

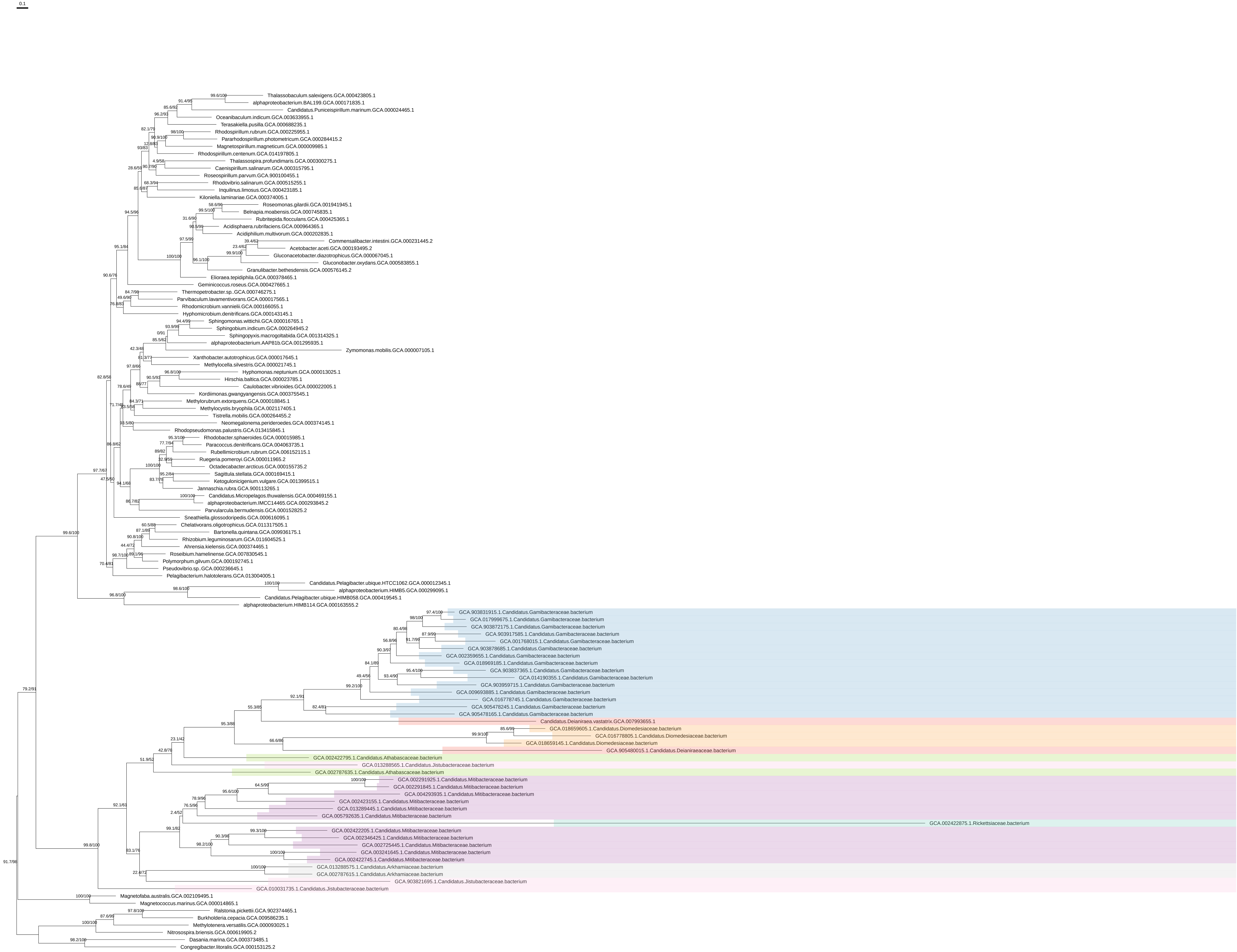

Lys_Thr_Met/01_lysC_thrA_COG0527@1

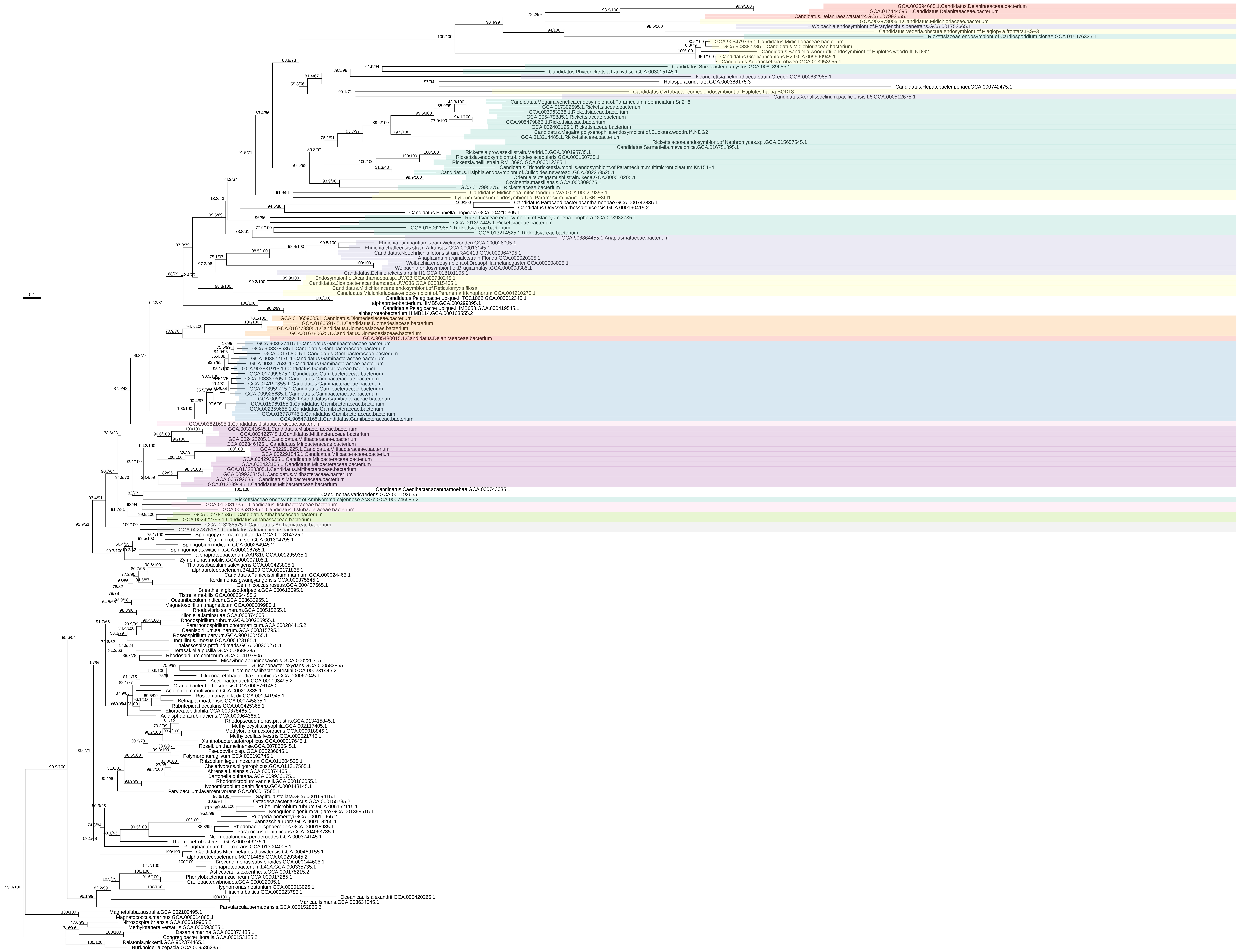

Lys_Thr_Met/02_asd_COG0136@2

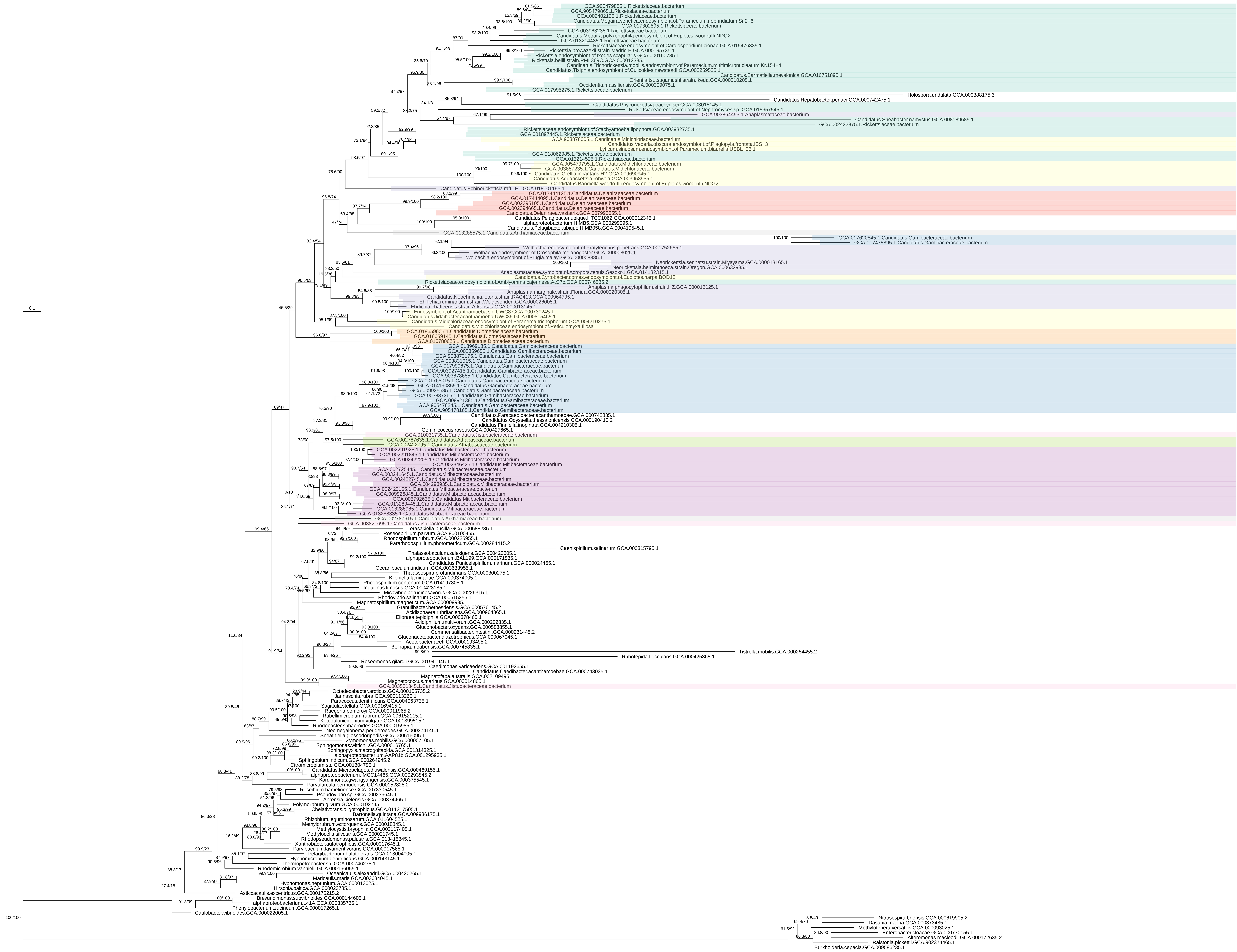

Lys_Thr_Met/03_thrA_COG0460@2

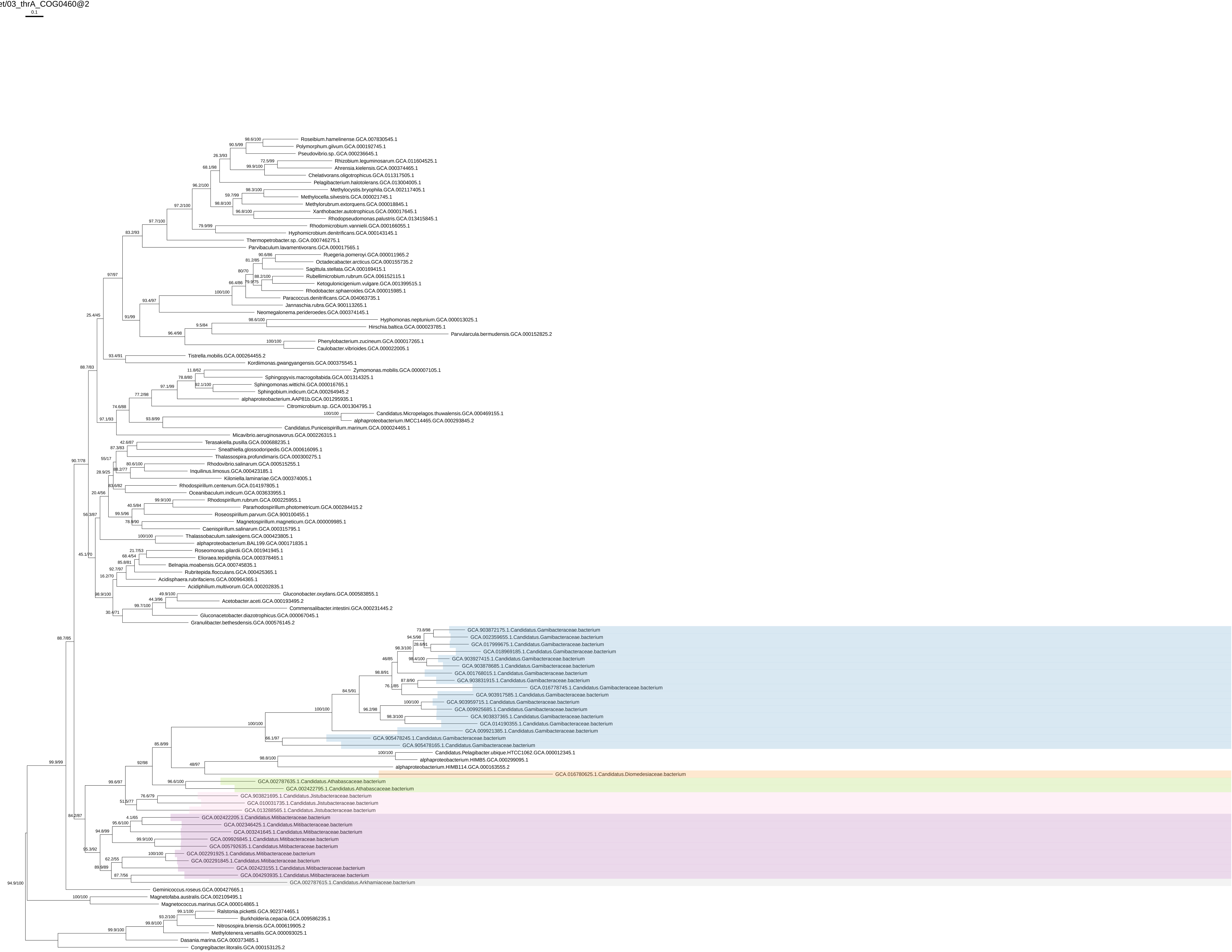

Lys_Thr_Met/04_thrB_COG2334@2

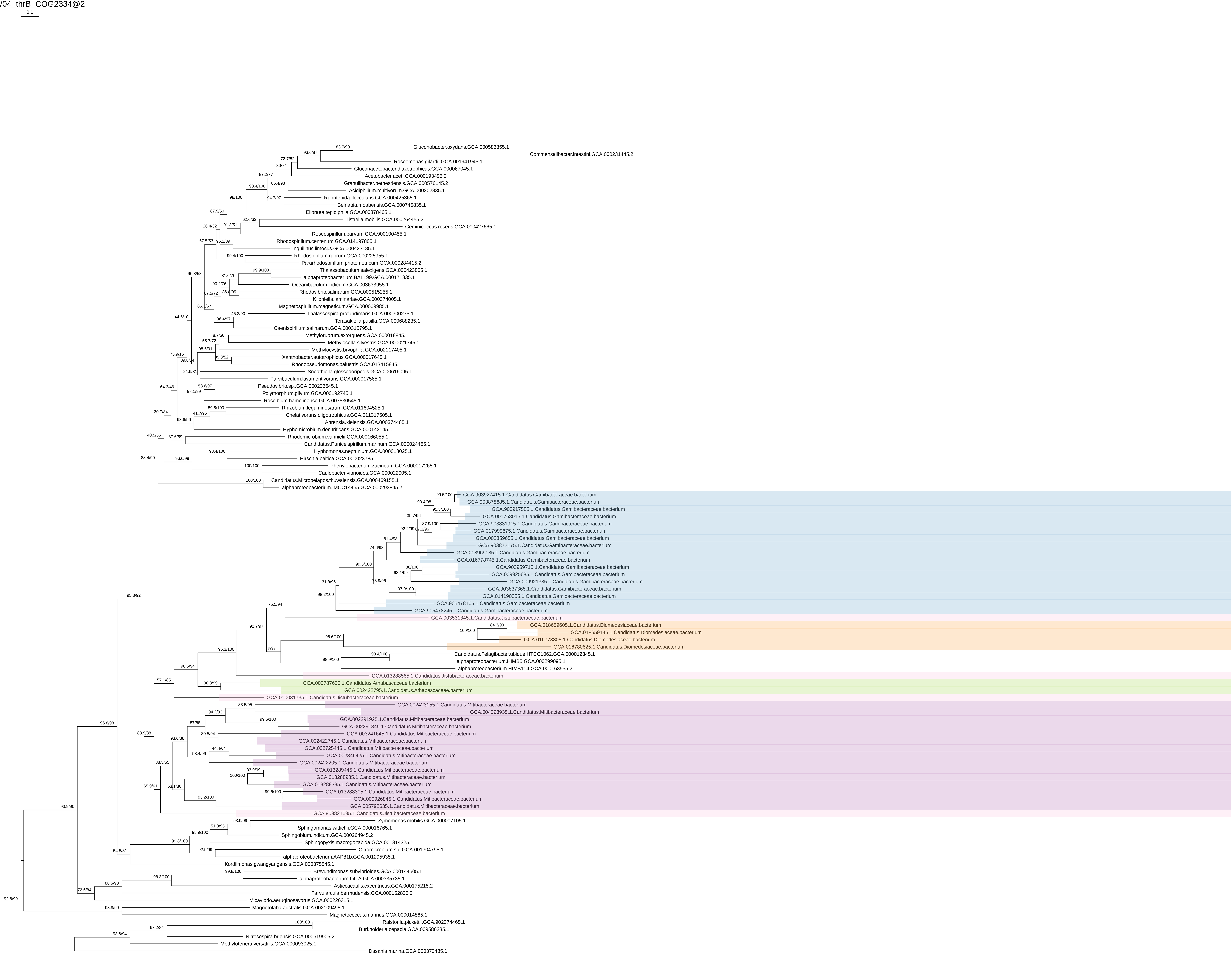

Lys_Thr_Met/05_thrC_COG0498@1

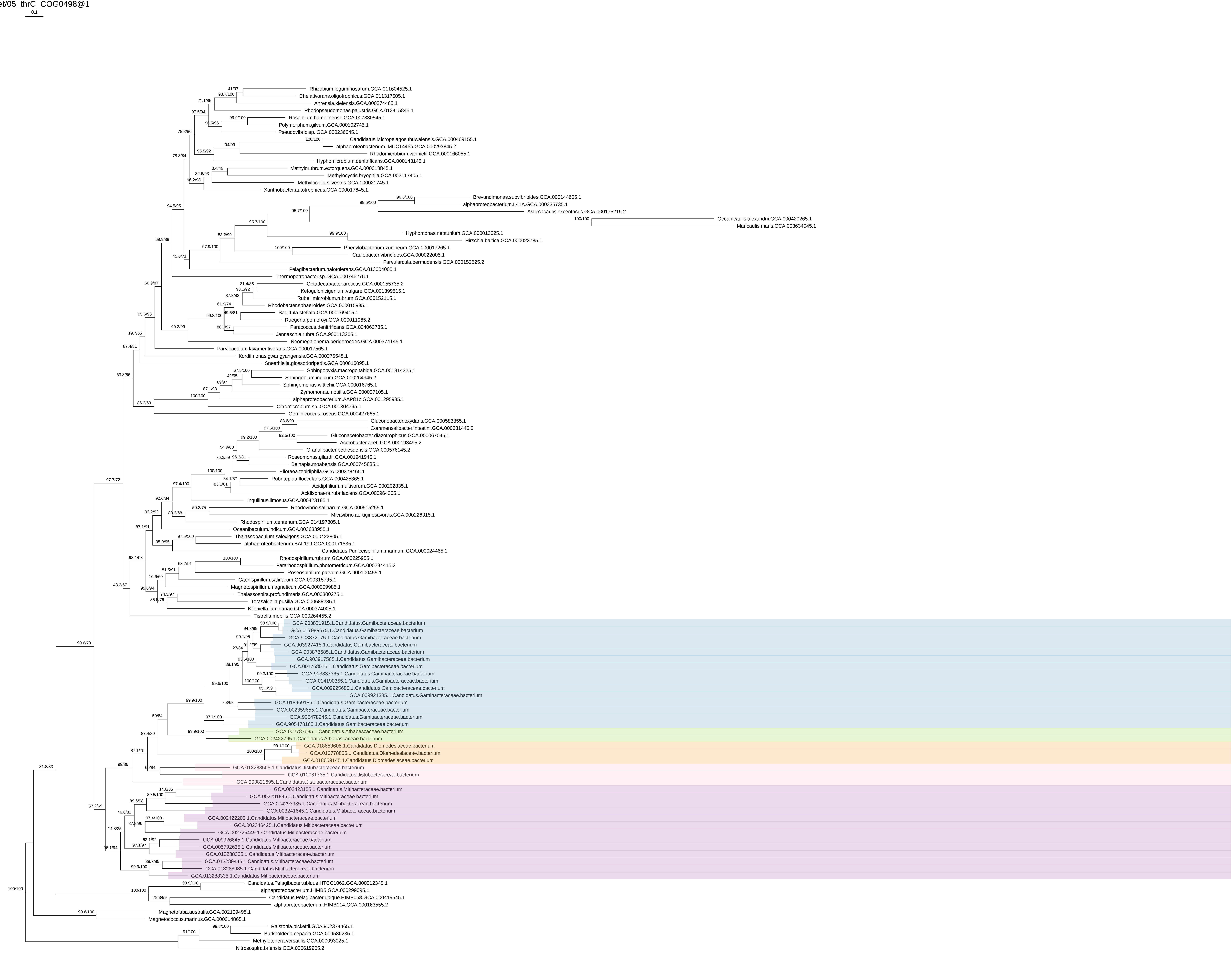

Lys_Thr_Met/06_dapA_COG0329@1

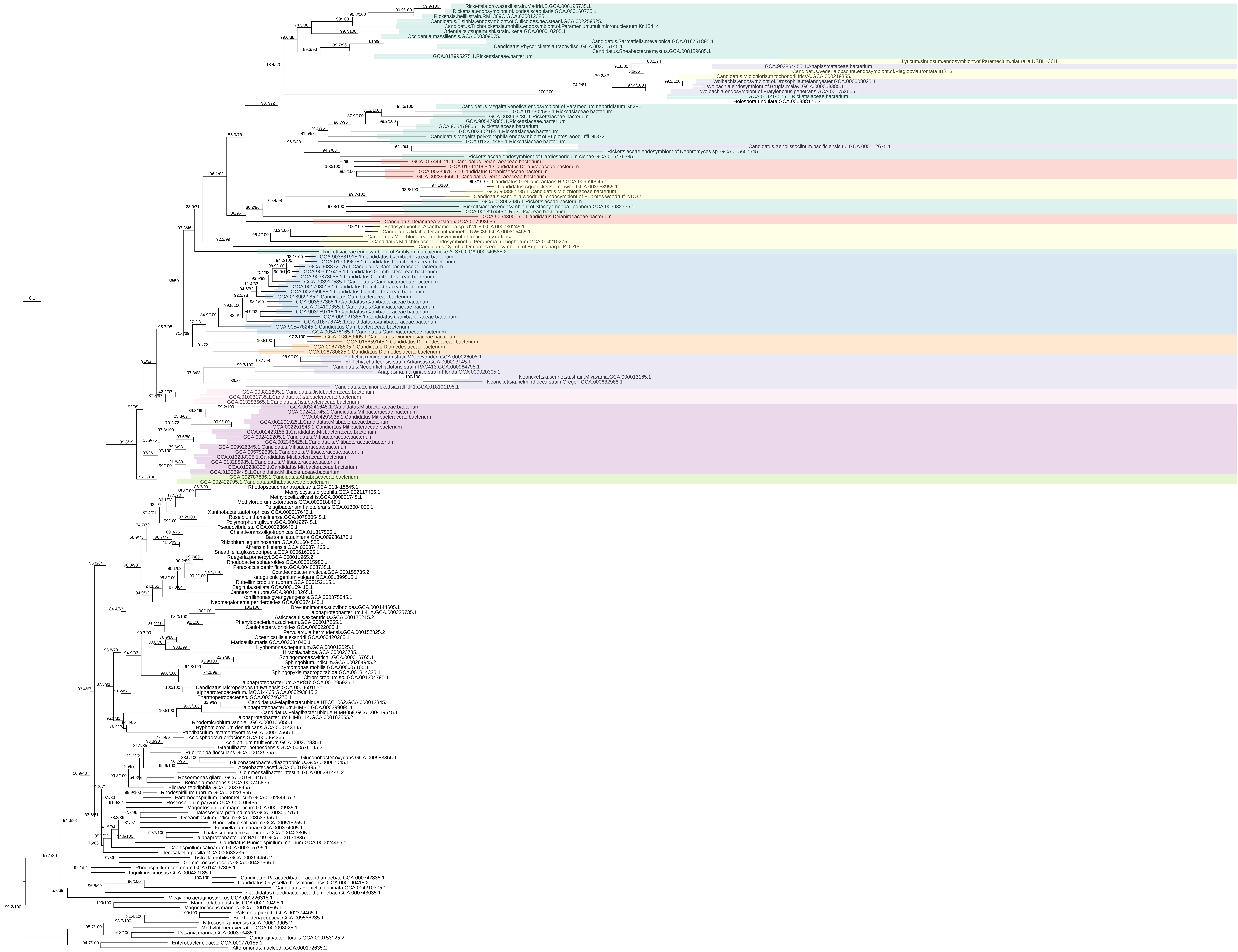

Lys_Thr_Met/07_dapB_COG0289@2

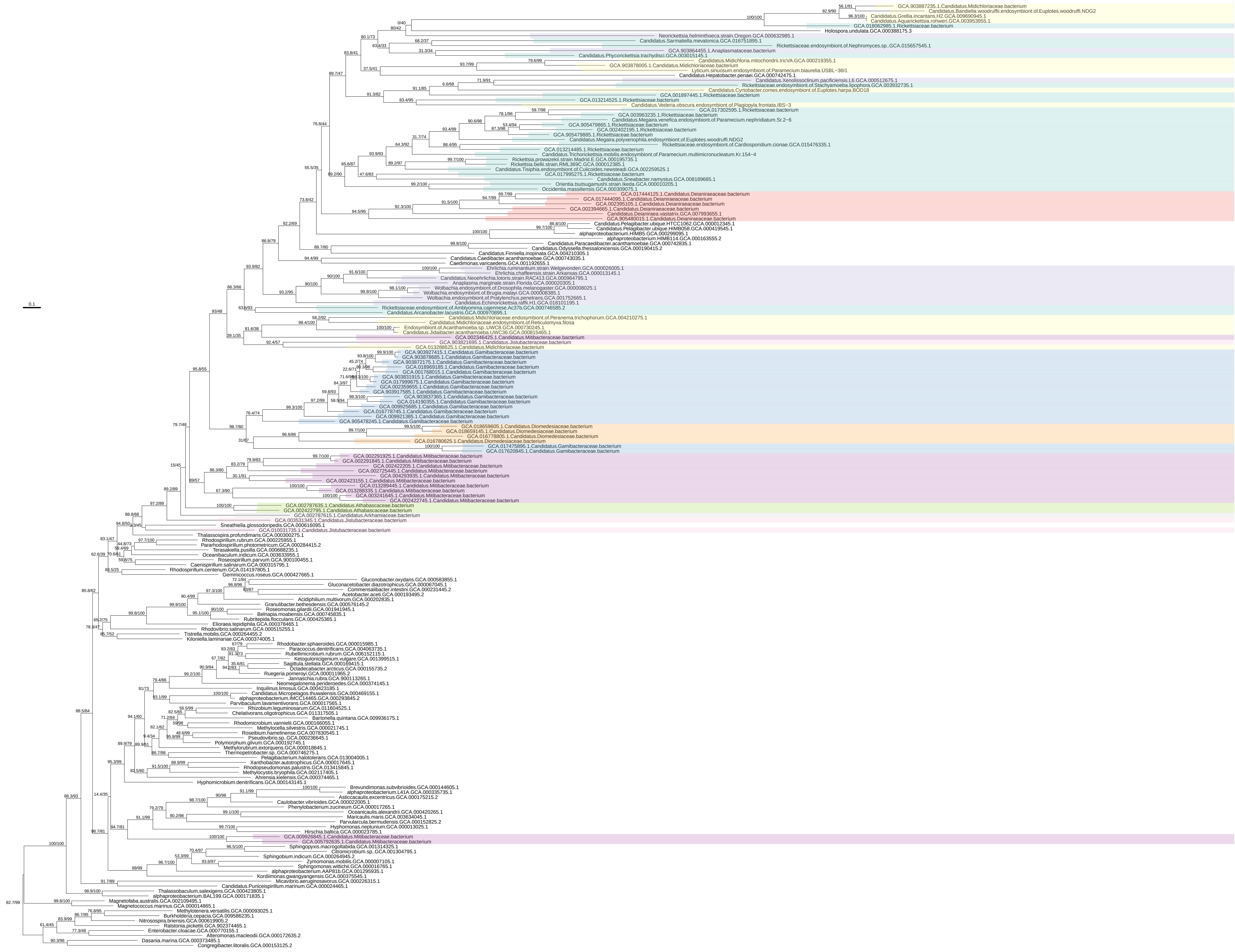

Lys_Thr_Met/08_dapD_1MU0Y@1224

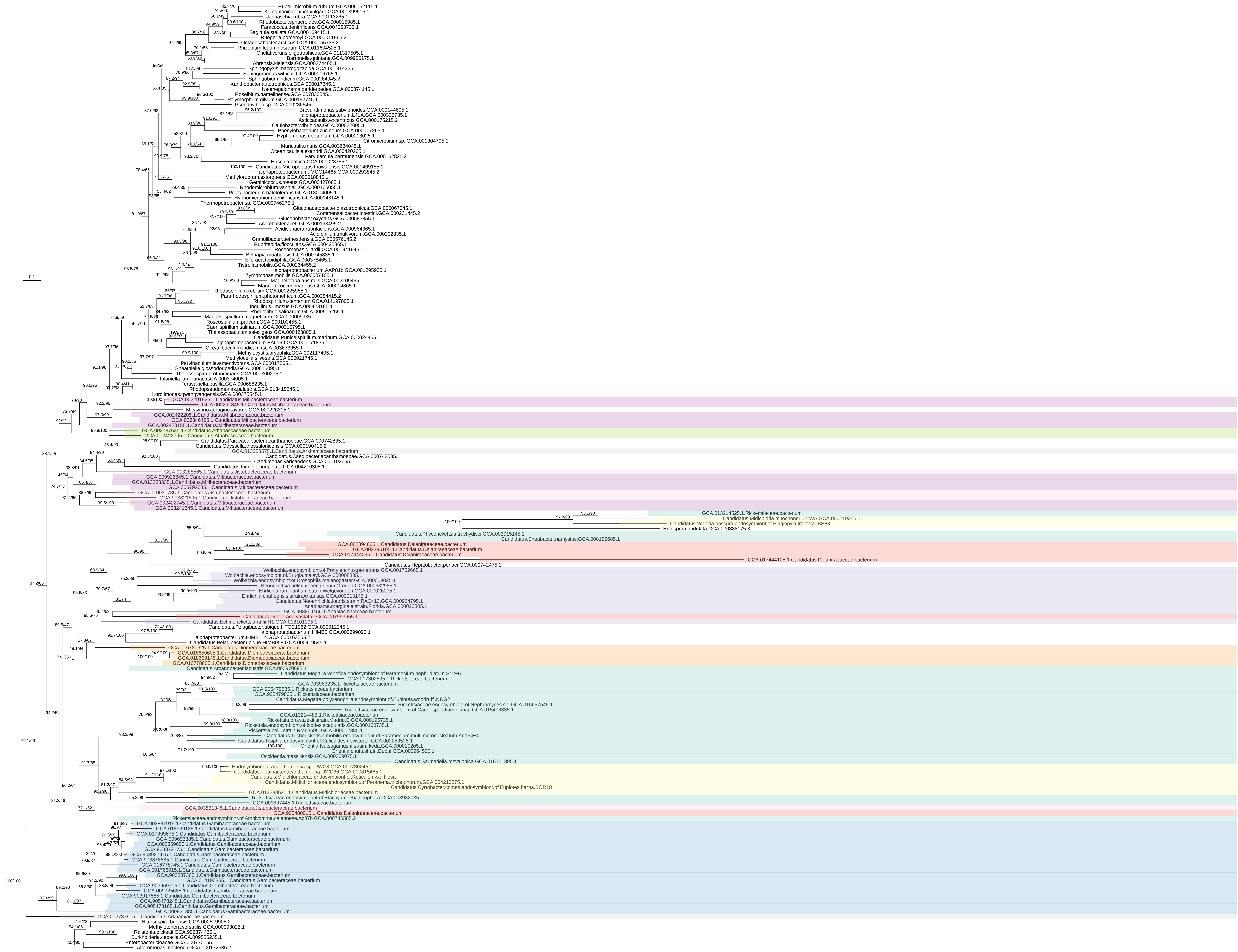

Lys_Thr_Met/09_dapE_COG0624@1

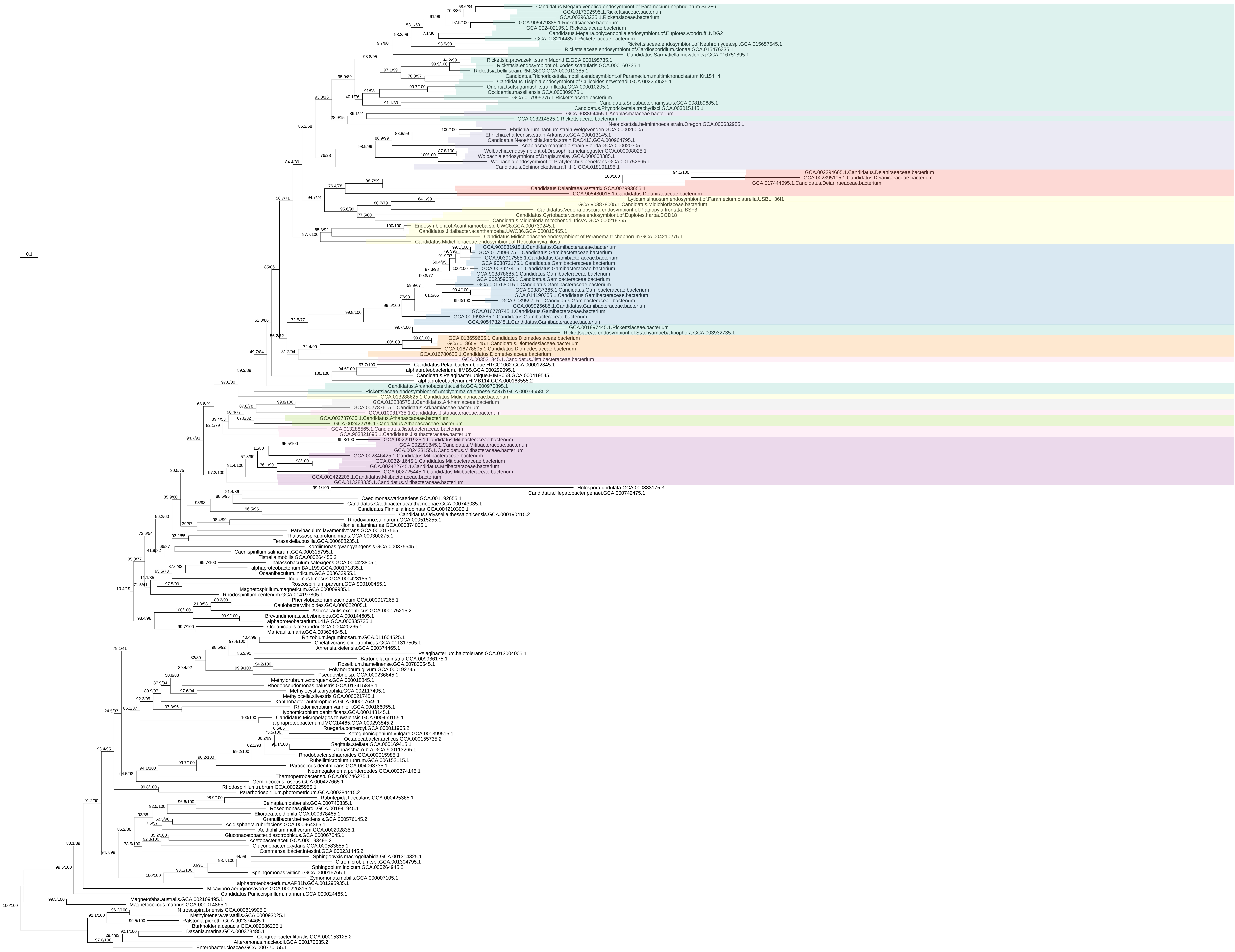

Lys_Thr_Met/10_dapF_COG0253@1

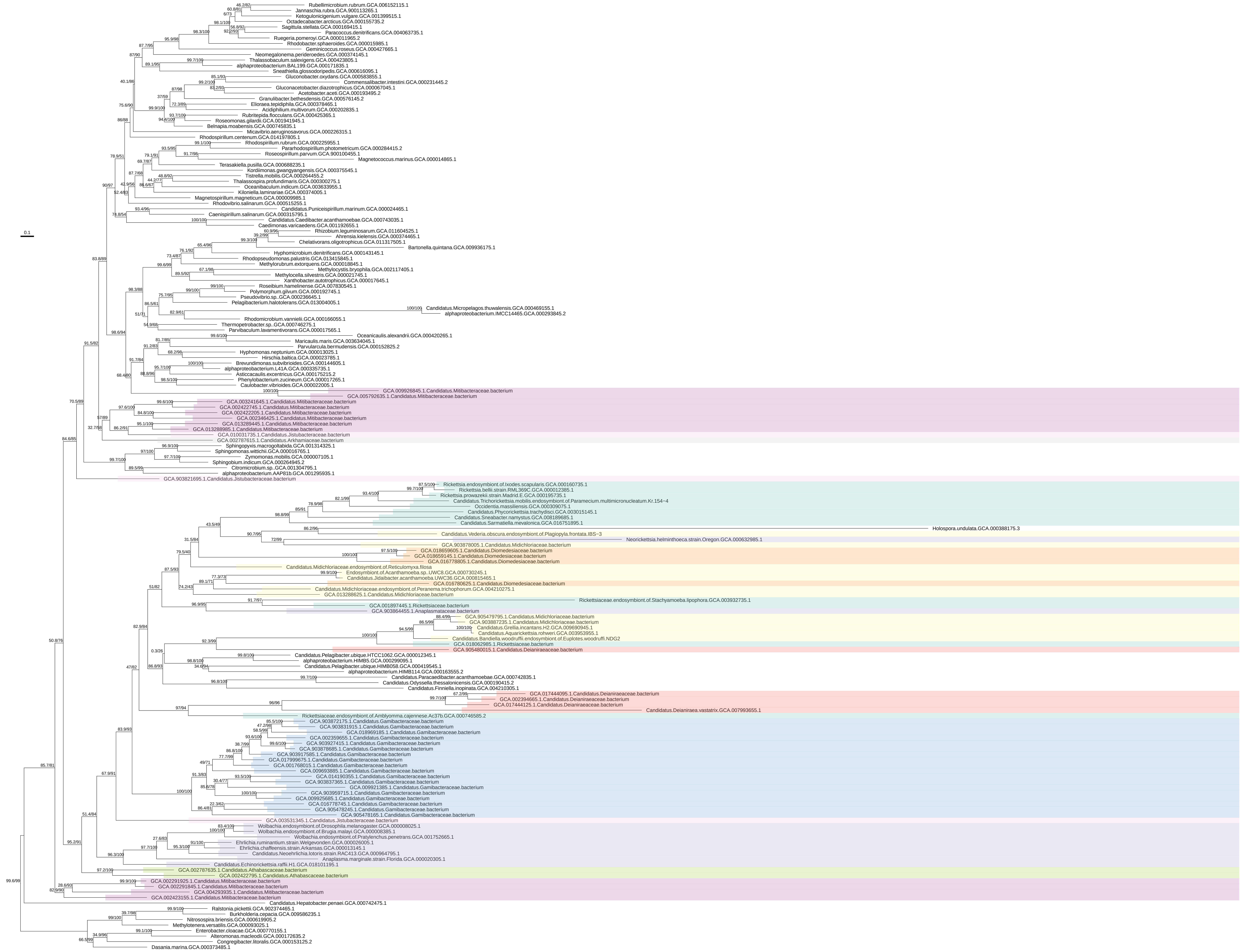

Lys_Thr_Met/11_lysA_COG0019@1

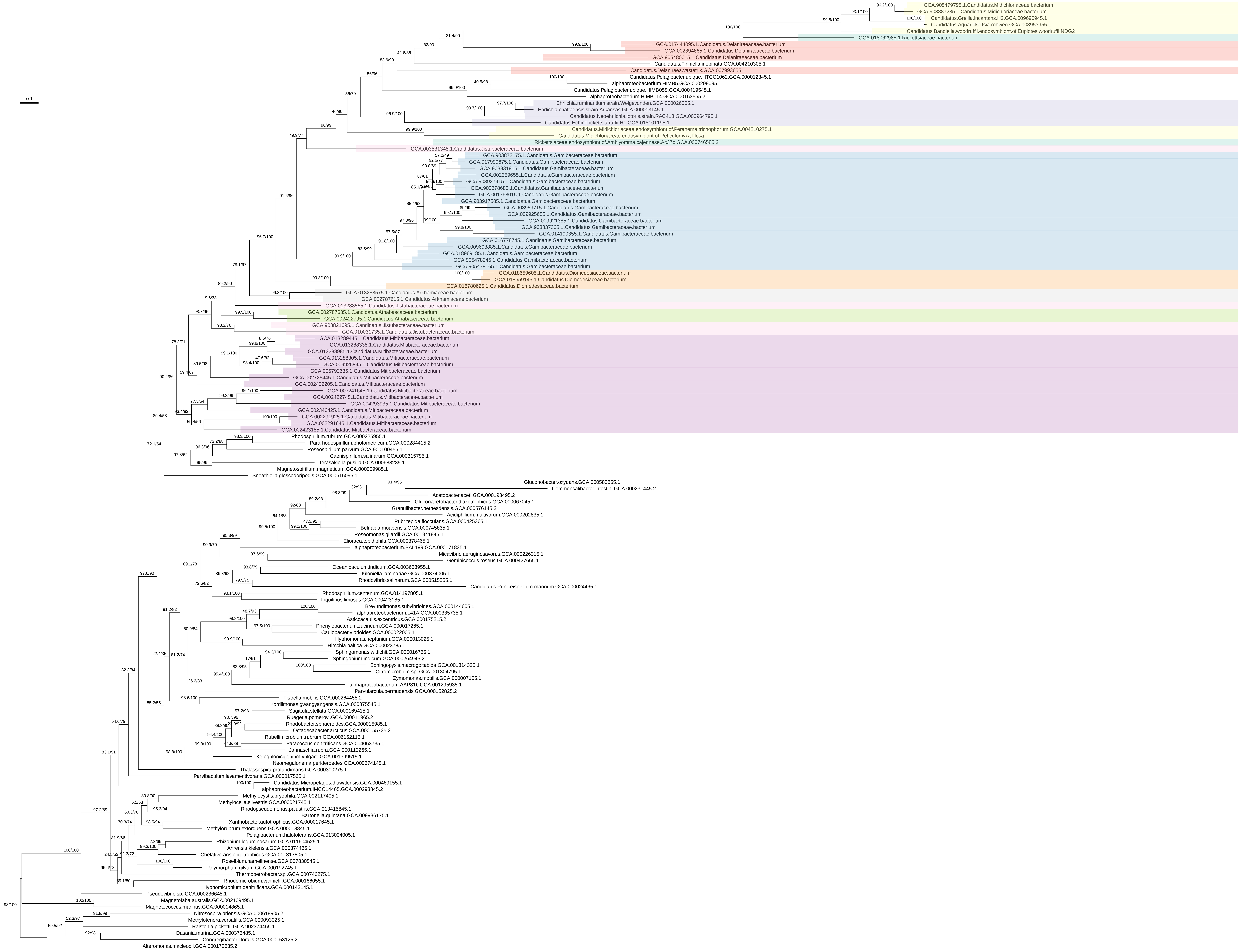

Lys_Thr_Met/12_metXA_COG2021@2

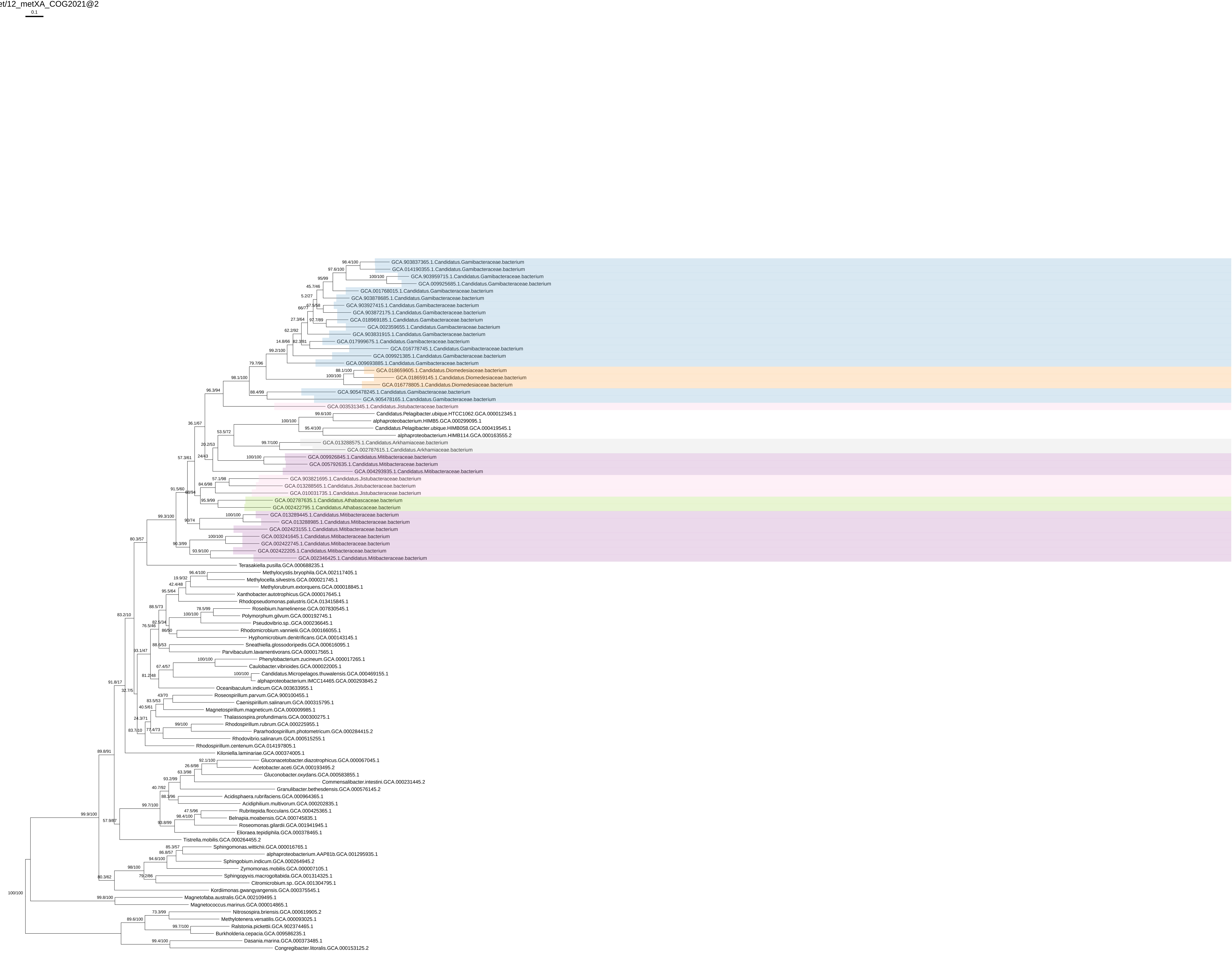

Lys_Thr_Met/13_metC_COG0626@1

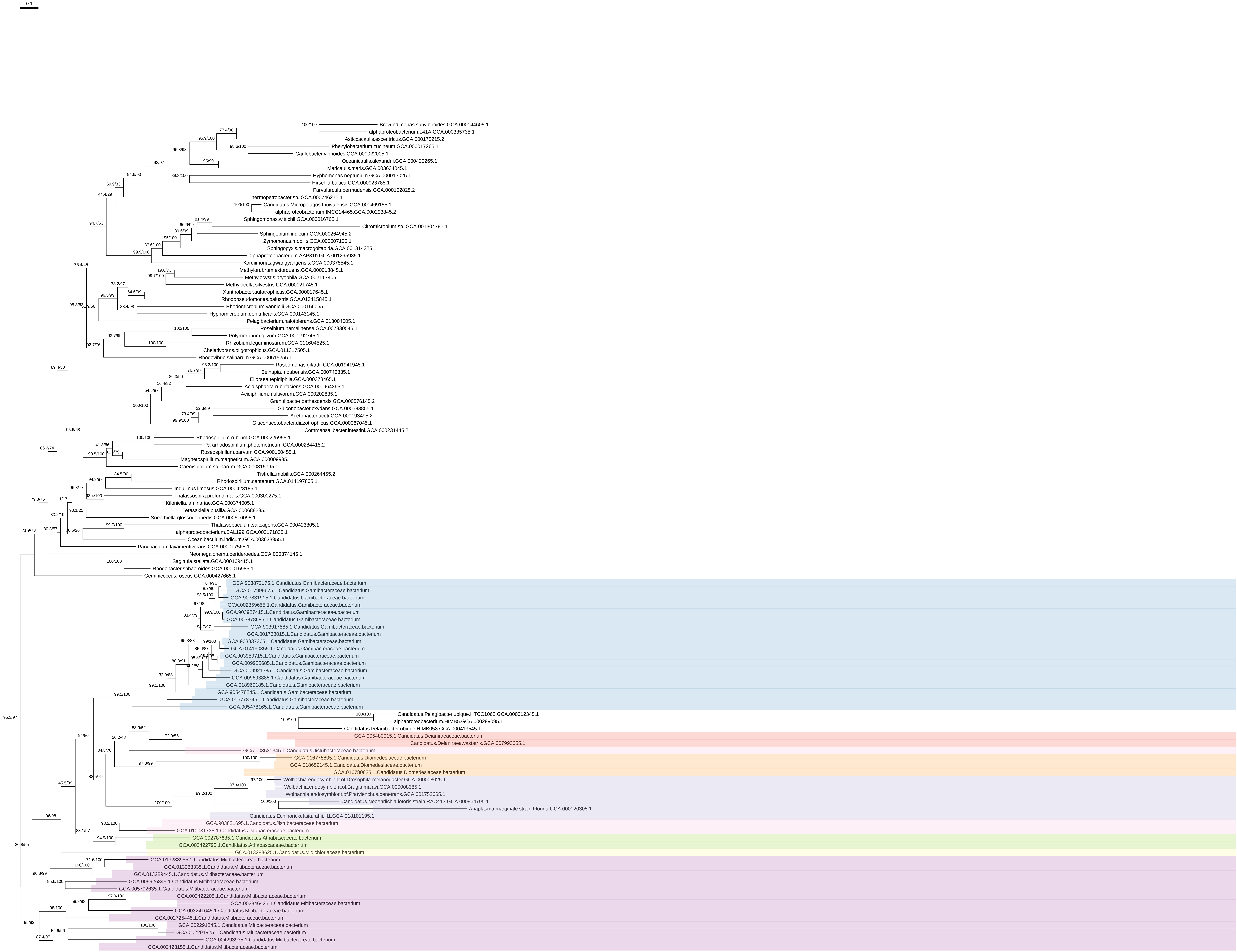

Lys_Thr_Met/14_metE_COG0620@2

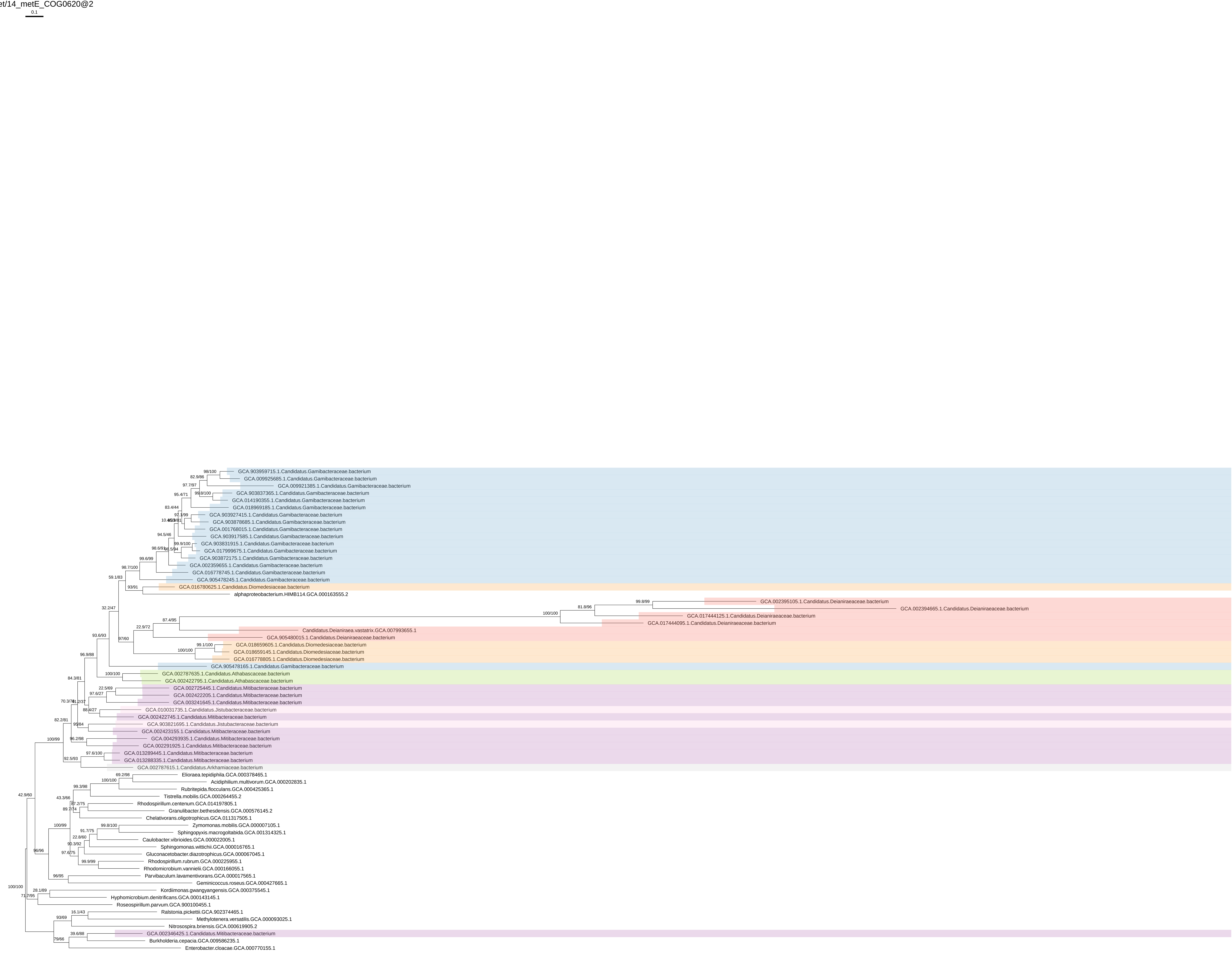

Lys_Thr_Met/15_metZ_COG0626@1

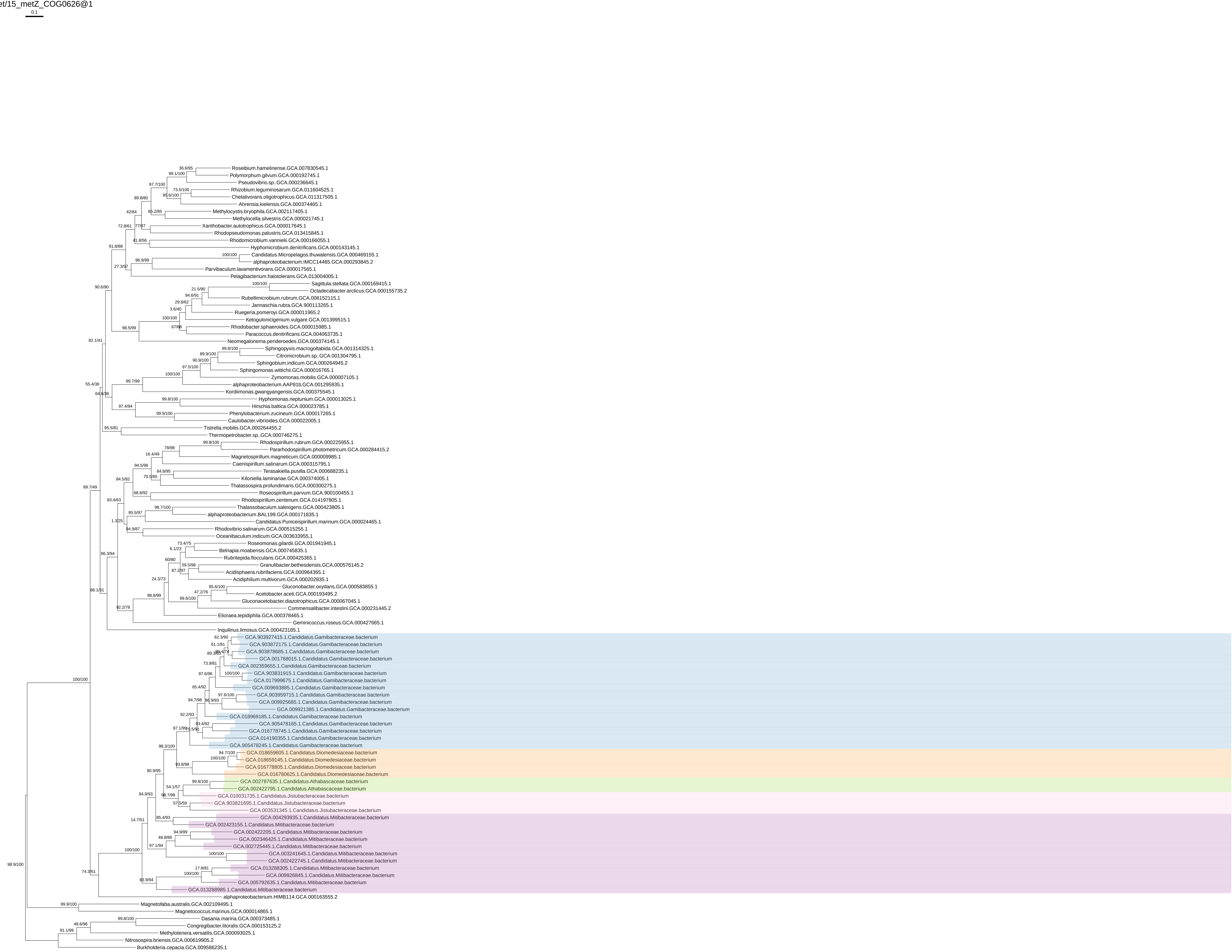

Lys_Thr_Met/16_metX_1MVSY@1224_1QYBJ@1224

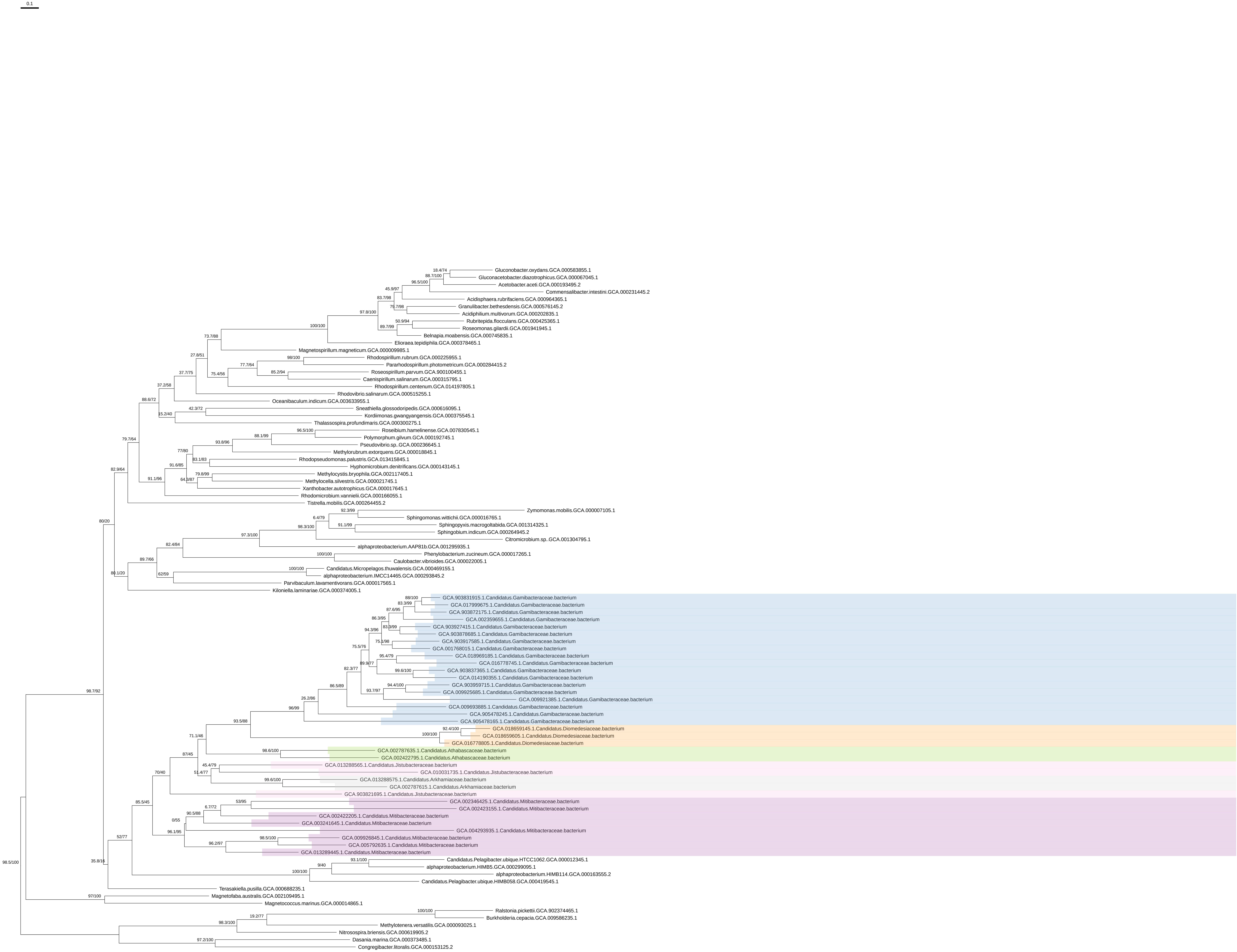

Supplementary Figure 26: concatenated phylogenies for nucleotide and amino acid biosynthesis. Phylogenetic trees of the concatenated genes of biosynthetic pathways for nucleotides and amino acids. For each pathway, the phylogenies are presented for untrimmed alignment and for the alignment with the 10%, 20%, 30%, 40%, or 50% most compositionally heterogeneous sites removed. On each tree, the members of each *Rickettsiales* family are highlighted by different background colours. On each branch, support values by SH-aLRT with 1000 replicates and by 1000 ultrafast bootstraps are reported. The tree scales stand for estimated sequence divergences.

Purine

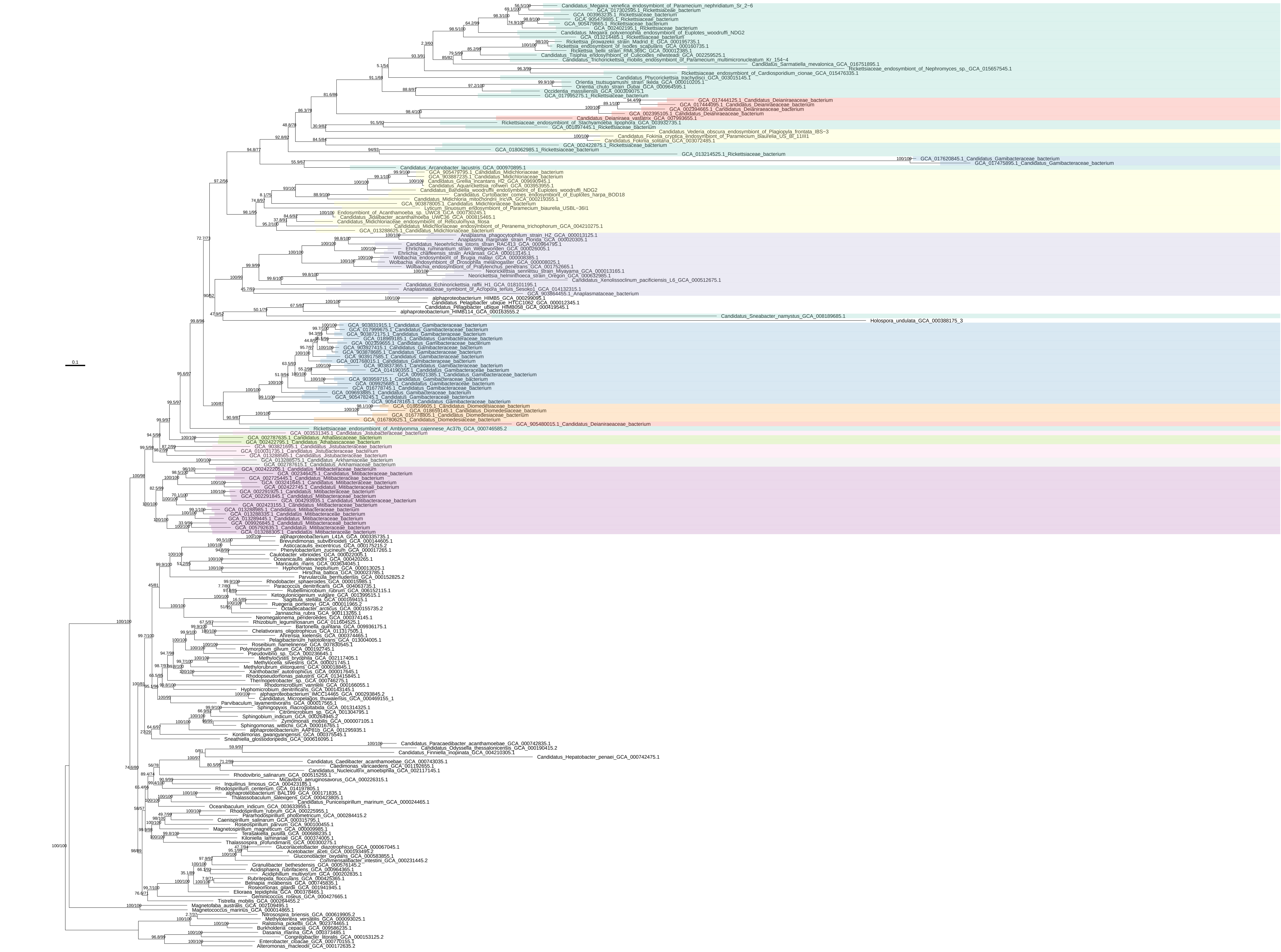

0.1
Candidatus_Megaira_venefica_endosymbiont_of_Paramecium_nephridiatum_Sr_2-6
GCA_017302595.1_Rickettsiaceae_bacterium
GCA_003963235.1_Rickettsiaceae_bacterium
GCA_905479885.1_Rickettsiaceae_bacterium
GCA_905479865.1_Rickettsiaceae_bacterium
GCA_002402195.1_Rickettsiaceae_bacterium
Candidatus_Megaira_polyxenophila_endosymbiont_of_Euplotes_woodruffi_NDG2
GCA_013214485.1_Rickettsiaceae_bacterium
Rickettsia_prowazekii_strain_Madrid_E_GCA_000195735.1
Rickettsia_endosymbiont_of_Ixodes_scapularis_GCA_000160735.1
Rickettsia_bellii_strain_RML369C_GCA_000012385.1
Candidatus_Tisiphia_endosymbiont_of_Culicoides_newsteadi_GCA_002259525.1
Candidatus_Trichorickettsia_mobilis_endosymbiont_of_Paramecium_multimicronucleatum_Kr_154-4
Candidatus_Sarmatiella_mevalonica_GCA_016751895.1
Rickettsiaceae_endosymbiont_of_Nephromyces_sp._GCA_015657545.1
Rickettsiaceae_endosymbiont_of_Cardiosporidium_cionae_GCA_015476335.1
Candidatus_Phycorickettsia_trachydisci_GCA_003015145.1
Orientia_tsutsugamushi_strain_Ikeda_GCA_000010205.1
Orientia_chuto_strain_Dubai_GCA_000964595.1
Occidentia_massiliensis_GCA_000309075.1
GCA_017995275.1_Rickettsiaceae_bacterium
GCA_017444125.1_Candidatus_Deianiraeaceae_bacterium
GCA_017444095.1_Candidatus_Deianiraeaceae_bacterium
GCA_002394665.1_Candidatus_Deianiraeaceae_bacterium
GCA_002395105.1_Candidatus_Deianiraeaceae_bacterium
Candidatus_Deianiraea_vastatrix_GCA_007993655.1
Rickettsiaceae_endosymbiont_of_Stachyamoeba_lipophora_GCA_003932735.1
GCA_001897445.1_Rickettsiaceae_bacterium
Candidatus_Vederia_obscura_endosymbiont_of_Plagiopyla_frontata_IBS-3
Candidatus_Fokinia_cryptica_endosymbiont_of_Paramecium_biaurelia_US_Bl_11III1
Candidatus_Fokinia_solitaria_GCA_003072485.1
GCA_002422875.1_Rickettsiaceae_bacterium
GCA_018062985.1_Rickettsiaceae_bacterium
GCA_013214525.1_Rickettsiaceae_bacterium
GCA_017620845.1_Candidatus_Gamibacteraceae_bacterium
GCA_017475895.1_Candidatus_Gamibacteraceae_bacterium
Candidatus_Arcanobacter_lacustris_GCA_000970895.1
GCA_905479795.1_Candidatus_Midichloriaceae_bacterium
GCA_903887235.1_Candidatus_Midichloriaceae_bacterium
Candidatus_Grellia_incantans_H2_GCA_009690945.1
Candidatus_Aquarickettsia_rohweri_GCA_003953955.1
Candidatus_Bandiella_woodruffii_endosymbiont_of_Euplotes_woodruffi_NDG2
Candidatus_Cyrtobacter_comes_endosymbiont_of_Euplotes_harpa_BOD18
Candidatus_Midichloria_mitochondrii_IricVA_GCA_000219355.1
GCA_903878005.1_Candidatus_Midichloriaceae_bacterium
Lyticum_sinuosum_endosymbiont_of_Paramecium_biaurelia_USBL-36I1
Endosymbiont_of_Acanthamoeba_sp._UWC8_GCA_000730245.1
Candidatus_Jidaibacter_acanthamoeba_UWC36_GCA_000815465.1
Candidatus_Midichloriaceae_endosymbiont_of_Reticulomyxa_filosa
Candidatus_Midichloriaceae_endosymbiont_of_Peranema_trichophorum_GCA_004210275.1
GCA_013288625.1_Candidatus_Midichloriaceae_bacterium
Anaplasma_phagocytophilum_strain_HZ_GCA_000013125.1
Anaplasma_marginale_strain_Florida_GCA_000020305.1
Candidatus_Neoehrlichia_lotoris_strain_RAC413_GCA_000964795.1
Ehrlichia_ruminantium_strain_Welgevonden_GCA_000026005.1
Ehrlichia_chaffeensis_strain_Arkansas_GCA_000013145.1
Wolbachia_endosymbiont_of_Brugia_malayi_GCA_000008385.1
Wolbachia_endosymbiont_of_Drosophila_melanogaster_GCA_000008025.1
Wolbachia_endosymbiont_of_Pratylenchus_penetrans_GCA_001752665.1
Neorickettsia_sennetsu_strain_Miyayama_GCA_000013165.1
Neorickettsia_helminthoeca_strain_Oregon_GCA_000632985.1
Candidatus_Xenolissoclinum_pacificiensis_L6_GCA_000512675.1
Candidatus_Echinorickettsia_raffii_H1_GCA_018101195.1
Anaplasmataceae_symbiont_of_Acropora_tenuis_Sesoko1_GCA_014132315.1
GCA_903864455.1_Anaplasmataceae_bacterium
alphaproteobacterium_HIMB5_GCA_000299095.1
Candidatus_Pelagibacter_ubique_HTCC1062_GCA_000012345.1
Candidatus_Pelagibacter_ubique_HIMB058_GCA_000419545.1
alphaproteobacterium_HIMB114_GCA_000163555.2
Candidatus_Sneabacter_namystus_GCA_008189685.1
Holospora_undulata_GCA_000388175_3
GCA_903831915.1_Candidatus_Gamibacteraceae_bacterium
GCA_017999675.1_Candidatus_Gamibacteraceae_bacterium
GCA_903872175.1_Candidatus_Gamibacteraceae_bacterium
GCA_018969185.1_Candidatus_Gamibacteraceae_bacterium
GCA_002359655.1_Candidatus_Gamibacteraceae_bacterium
GCA_903927415.1_Candidatus_Gamibacteraceae_bacterium
GCA_903878685.1_Candidatus_Gamibacteraceae_bacterium
GCA_903917585.1_Candidatus_Gamibacteraceae_bacterium
GCA_001768015.1_Candidatus_Gamibacteraceae_bacterium
GCA_903837365.1_Candidatus_Gamibacteraceae_bacterium
GCA_014190355.1_Candidatus_Gamibacteraceae_bacterium
GCA_009921385.1_Candidatus_Gamibacteraceae_bacterium
GCA_903959715.1_Candidatus_Gamibacteraceae_bacterium
GCA_009925685.1_Candidatus_Gamibacteraceae_bacterium
GCA_016778745.1_Candidatus_Gamibacteraceae_bacterium
GCA_009693885.1_Candidatus_Gamibacteraceae_bacterium
GCA_905478245.1_Candidatus_Gamibacteraceae_bacterium
GCA_905478165.1_Candidatus_Gamibacteraceae_bacterium
GCA_018659605.1_Candidatus_Diomedesiaceae_bacterium
GCA_018659145.1_Candidatus_Diomedesiaceae_bacterium
GCA_016778805.1_Candidatus_Diomedesiaceae_bacterium
GCA_016780625.1_Candidatus_Diomedesiaceae_bacterium
GCA_905480015.1_Candidatus_Deianiraeaceae_bacterium
Rickettsiaceae_endosymbiont_of_Amblyomma_cajennese_Ac37b_GCA_000746585.2
GCA_003531345.1_Candidatus_Jistubacteraceae_bacterium
GCA_002787635.1_Candidatus_Athabascaceae_bacterium
GCA_002422795.1_Candidatus_Athabascaceae_bacterium
GCA_903821695.1_Candidatus_Jistubacteraceae_bacterium
GCA_010031735.1_Candidatus_Jistubacteraceae_bacterium
GCA_013288565.1_Candidatus_Jistubacteraceae_bacterium
GCA_013288575.1_Candidatus_Arkhamiaceae_bacterium
GCA_002787615.1_Candidatus_Arkhamiaceae_bacterium
GCA_002422205.1_Candidatus_Mitibacteraceae_bacterium
GCA_002346425.1_Candidatus_Mitibacteraceae_bacterium
GCA_002725445.1_Candidatus_Mitibacteraceae_bacterium
GCA_003241645.1_Candidatus_Mitibacteraceae_bacterium
GCA_002422745.1_Candidatus_Mitibacteraceae_bacterium
GCA_002291925.1_Candidatus_Mitibacteraceae_bacterium
GCA_002291845.1_Candidatus_Mitibacteraceae_bacterium
GCA_004293935.1_Candidatus_Mitibacteraceae_bacterium
GCA_002423155.1_Candidatus_Mitibacteraceae_bacterium
GCA_013288985.1_Candidatus_Mitibacteraceae_bacterium
GCA_013288335.1_Candidatus_Mitibacteraceae_bacterium
GCA_013289445.1_Candidatus_Mitibacteraceae_bacterium
GCA_009926845.1_Candidatus_Mitibacteraceae_bacterium
GCA_005792635.1_Candidatus_Mitibacteraceae_bacterium
GCA_013288305.1_Candidatus_Mitibacteraceae_bacterium
alphaproteobacterium_L41A_GCA_000335735.1
Brevundimonas_subvibrioides_GCA_000144605.1
Asticcacaulis_excentricus_GCA_000175215.2
Phenylobacterium_zucineum_GCA_000017265.1
Caulobacter_vibrioides_GCA_000022005.1
Oceanicaulis_alexandrii_GCA_000420265.1
Maricaulis_maris_GCA_003634045.1
Hyphomonas_neptunium_GCA_000013025.1
Hirschia_baltica_GCA_000023785.1
Parvularcula_bermudensis_GCA_000152825.2
Rhodobacter_sphaeroides_GCA_000015985.1
Paracoccus_denitrificans_GCA_004063735.1
Rubellimicrobium_rubrum_GCA_006152115.1
Ketogulonicigenium_vulgare_GCA_001399515.1
Sagittula_stellata_GCA_000169415.1
Ruegeria_pomeroyi_GCA_000011965.2
Octadecabacter_arcticus_GCA_000155735.2
Jannaschia_rubra_GCA_900113265.1
Neomegalonema_perideroedes_GCA_000374145.1
Rhizobium_leguminosarum_GCA_011604525.1
Bartonella_quintana_GCA_009936175.1
Chelativorans_oligotrophicus_GCA_011317505.1
Ahrensia_kielensis_GCA_000374465.1
Pelagibacterium_halotolerans_GCA_013004005.1
Roseibium_hamelinense_GCA_007830545.1
Polymorphum_gilvum_GCA_000192745.1
Pseudovibrio_sp._GCA_000236645.1
Methylocystis_bryophila_GCA_002117405.1
Methylocella_silvestris_GCA_000021745.1
Methylorubrum_extorquens_GCA_000018845.1
Xanthobacter_autotrophicus_GCA_000017645.1
Rhodopseudomonas_palustris_GCA_013415845.1
Thermopetrobacter_sp._GCA_000746275.1
Rhodomicrobium_vannielii_GCA_000166055.1
Hyphomicrobium_denitrificans_GCA_000143145.1
alphaproteobacterium_IMCC14465_GCA_000293845.2
Candidatus_Micropelagos_thuwalensis_GCA_000469155_1
Parvibaculum_lavamentivorans_GCA_000017565.1
Sphingopyxis_macrogoltabida_GCA_001314325.1
Citromicrobium_sp._GCA_001304795.1
Sphingobium_indicum_GCA_000264945.2
Zymomonas_mobilis_GCA_000007105.1
Sphingomonas_wittichii_GCA_000016765.1
alphaproteobacterium_AAP81b_GCA_001295935.1
Kordiimonas_gwangyangensis_GCA_000375545.1
Sneathiella_glossodoripedis_GCA_000616095.1
Candidatus_Paracaedibacter_acanthamoebae_GCA_000742835.1
Candidatus_Odyssella_thessalonicensis_GCA_000190415.2
Candidatus_Finniella_inopinata_GCA_004210305.1
Candidatus_Hepatobacter_penaei_GCA_000742475.1
Candidatus_Caedibacter_acanthamoebae_GCA_000743035.1
Caedimonas_varicaedens_GCA_001192655.1
Candidatus_Nucleicultrix_amoebiphila_GCA_002117145.1
Rhodovibrio_salinarum_GCA_000515255.1
Micavibrio_aeruginosavorus_GCA_000226315.1
Inquilinus_limosus_GCA_000423185.1
Rhodospirillum_centenum_GCA_014197805.1
alphaproteobacterium_BAL199_GCA_000171835.1
Thalassobaculum_salexigens_GCA_000423805.1
Candidatus_Puniceispirillum_marinum_GCA_000024465.1
Oceanibaculum_indicum_GCA_003633955.1
Rhodospirillum_rubrum_GCA_000225955.1
Pararhodospirillum_photometricum_GCA_000284415.2
Caenispirillum_salinarum_GCA_000315795.1
Roseospirillum_parvum_GCA_900100455.1
Magnetospirillum_magneticum_GCA_000009985.1
Terasakiella_pusilla_GCA_000688235.1
Kiloniella_laminariae_GCA_000374005.1
Thalassospira_profundimaris_GCA_000300275.1
Gluconacetobacter_diazotrophicus_GCA_000067045.1
Acetobacter_aceti_GCA_000193495.2
Gluconobacter_oxydans_GCA_000583855.1
Commensalibacter_intestini_GCA_000231445.2
Granulibacter_bethesdensis_GCA_000576145.2
Acidisphaera_rubrifaciens_GCA_000964365.1
Acidiphilium_multivorum_GCA_000202835.1
Rubritepida_flocculans_GCA_000425365.1
Belnapia_moabensis_GCA_000745835.1
Roseomonas_gilardii_GCA_001941945.1
Elioraea_tepidiphila_GCA_000378465.1
Geminicoccus_roseus_GCA_000427665.1
Tistrella_mobilis_GCA_000264455.2
Magnetofaba_australis_GCA_002109495.1
Magnetococcus_marinus_GCA_000014865.1
Nitrosospira_briensis_GCA_000619905.2
Methyloteriera_versatilis_GCA_000093025.1
Ralstonia_pickettii_GCA_902374465.1
Burkholderia_cepacia_GCA_009586235.1
Dasania_marina_GCA_000373485.1
Congregibacter_litoralis_GCA_000153125.2
Enterobacter_cloacae_GCA_000770155.1
Alteromonas_macleodii_GCA_000172635.2

Purine_z_0.10

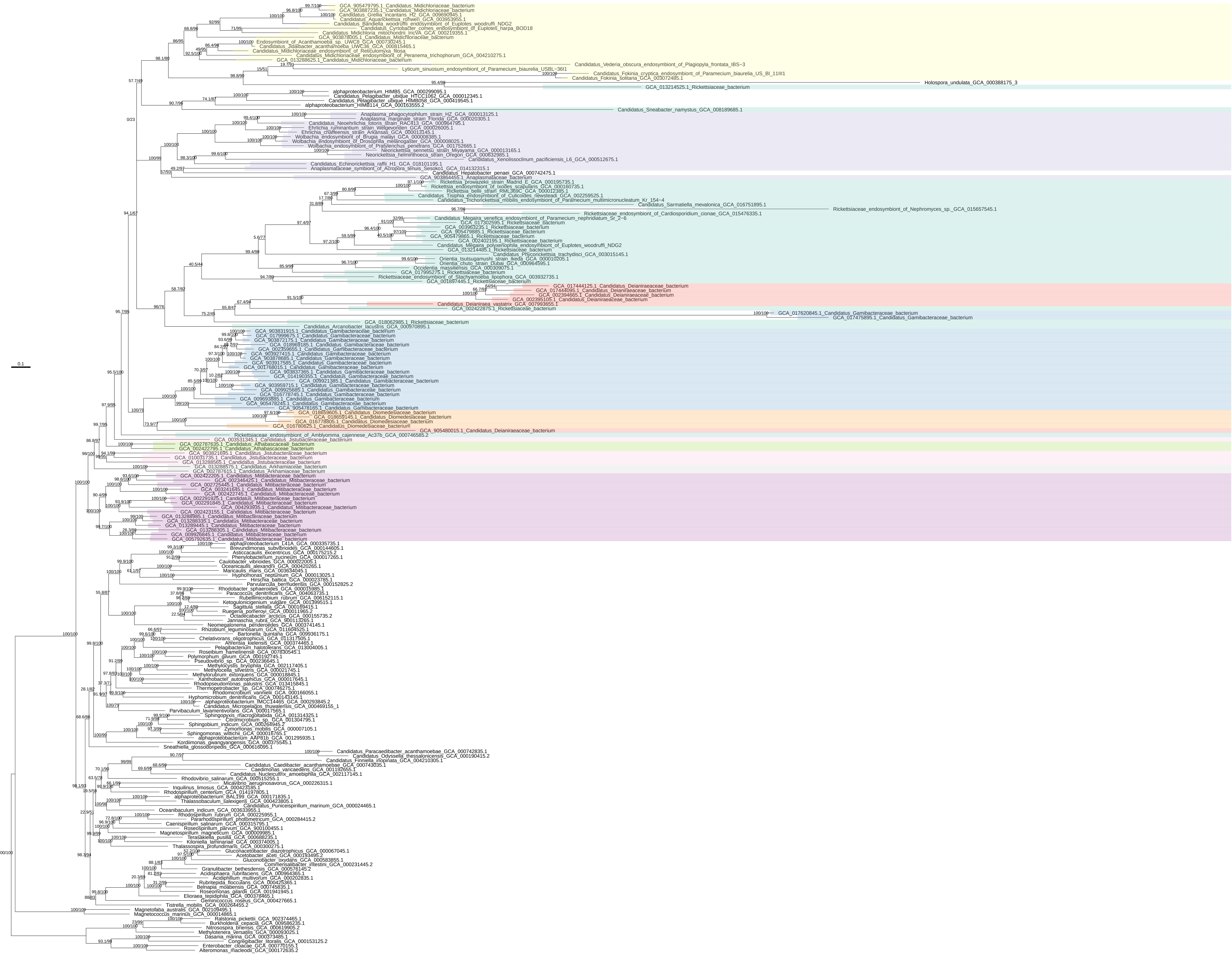

0.1
GCA_905479795.1_Candidatus_Midichloriaceae_bacterium
GCA_903887235.1_Candidatus_Midichloriaceae_bacterium
Candidatus_Grellia_incantans_H2_GCA_009690945.1
Candidatus_Aquarickettsia_rohweri_GCA_003953955.1
Candidatus_Bandiella_woodruffii_endosymbiont_of_Euplotes_woodruffi_NDG2
Candidatus_Cyrtobacter_comes_endosymbiont_of_Euplotes_harpa_BOD18
Candidatus_Midichloria_mitochondrii_IricVA_GCA_000219355.1
GCA_903878005.1_Candidatus_Midichloriaceae_bacterium
Endosymbiont_of_Acanthamoeba_sp._UWC8_GCA_000730245.1
Candidatus_Jidaibacter_acanthamoeba_UWC36_GCA_000815465.1
Candidatus_Midichloriaceae_endosymbiont_of_Reticulomyxa_filosa
Candidatus_Midichloriaceae_endosymbiont_of_Peranema_trichophorum_GCA_004210275.1
GCA_013288625.1_Candidatus_Midichloriaceae_bacterium
Candidatus_Vederia_obscura_endosymbiont_of_Plagiopyla_frontata_IBS-3
Lyticum_sinuosum_endosymbiont_of_Paramecium_biaurelia_USBL-36I1
Candidatus_Fokinia_cryptica_endosymbiont_of_Paramecium_biaurelia_US_BI_11III1
Candidatus_Fokinia_solitaria_GCA_003072485.1
Holospora_undulata_GCA_000388175_3
GCA_013214525.1_Rickettsiaceae_bacterium
alphaproteobacterium_HIMB5_GCA_000299095.1
Candidatus_Pelagibacter_ubique_HTCC1062_GCA_000012345.1
Candidatus_Pelagibacter_ubique_HIMB058_GCA_000419545.1
alphaproteobacterium_HIMB114_GCA_000163555.2
Candidatus_Sneabacter_namystus_GCA_008189685.1
Anaplasma_phagocytophilum_strain_HZ_GCA_000013125.1
Anaplasma_marginale_strain_Florida_GCA_000020305.1
Candidatus_Neoehrlichia_lotoris_strain_RAC413_GCA_000964795.1
Ehrlichia_ruminantium_strain_Welgevonden_GCA_000026005.1
Ehrlichia_chaffeensis_strain_Arkansas_GCA_000013145.1
Wolbachia_endosymbiont_of_Brugia_malayi_GCA_000008385.1
Wolbachia_endosymbiont_of_Drosophila_melanogaster_GCA_000008025.1
Wolbachia_endosymbiont_of_Pratylenchus_penetrans_GCA_001752665.1
Neorickettsia_sennetsu_strain_Miyayama_GCA_000013165.1
Neorickettsia_helminthoeca_strain_Oregon_GCA_000632985.1
Candidatus_Xenolissoclinum_pacificiensis_L6_GCA_000512675.1
Candidatus_Echinorickettsia_raffii_H1_GCA_018101195.1
Anaplasmataceae_symbiont_of_Acropora_tenuis_Sesoko1_GCA_014132315.1
Candidatus_Hepatobacter_penaei_GCA_000742475.1
GCA_903864455.1_Anaplasmataceae_bacterium
Rickettsia_prowazekii_strain_Madrid_E_GCA_000195735.1
Rickettsia_endosymbiont_of_Ixodes_scapularis_GCA_000160735.1
Rickettsia_bellii_strain_RML369C_GCA_000012385.1
Candidatus_Tisiphia_endosymbiont_of_Culicoides_newsteadi_GCA_002259525.1
Candidatus_Trichorickettsia_mobilis_endosymbiont_of_Paramecium_multimicronucleatum_Kr_154-4
Candidatus_Sarmatiella_mevalonica_GCA_016751895.1
Rickettsiaceae_endosymbiont_of_Nephromyces_sp._GCA_015657545.1
Rickettsiaceae_endosymbiont_of_Cardiosporidium_cionae_GCA_015476335.1
Candidatus_Megaira_venefica_endosymbiont_of_Paramecium_nephridiatum_Sr_2-6
GCA_017302595.1_Rickettsiaceae_bacterium
GCA_003963235.1_Rickettsiaceae_bacterium
GCA_905479885.1_Rickettsiaceae_bacterium
GCA_905479865.1_Rickettsiaceae_bacterium
GCA_002402195.1_Rickettsiaceae_bacterium
Candidatus_Megaira_polyxenophila_endosymbiont_of_Euplotes_woodruffi_NDG2
GCA_013214485.1_Rickettsiaceae_bacterium
Candidatus_Phycorickettsia_trachydisci_GCA_003015145.1
Orientia_tsutsugamushi_strain_Ikeda_GCA_000010205.1
Orientia_chuto_strain_Dubai_GCA_000964595.1
Occidentia_massiliensis_GCA_000309075.1
GCA_017995275.1_Rickettsiaceae_bacterium
Rickettsiaceae_endosymbiont_of_Stachyamoeba_lipophora_GCA_003932735.1
GCA_001897445.1_Rickettsiaceae_bacterium
GCA_017444125.1_Candidatus_Deianiraeaceae_bacterium
GCA_017444095.1_Candidatus_Deianiraeaceae_bacterium
GCA_002394665.1_Candidatus_Deianiraeaceae_bacterium
GCA_002395105.1_Candidatus_Deianiraeaceae_bacterium
Candidatus_Deianiraea_vastatrix_GCA_007993655.1
GCA_002422875.1_Rickettsiaceae_bacterium
GCA_017620845.1_Candidatus_Gamibacteraceae_bacterium
GCA_017475895.1_Candidatus_Gamibacteraceae_bacterium
GCA_018062985.1_Rickettsiaceae_bacterium
Candidatus_Arcanobacter_lacustris_GCA_000970895.1
GCA_903831915.1_Candidatus_Gamibacteraceae_bacterium
GCA_017999675.1_Candidatus_Gamibacteraceae_bacterium
GCA_903872175.1_Candidatus_Gamibacteraceae_bacterium
GCA_018969185.1_Candidatus_Gamibacteraceae_bacterium
GCA_002359655.1_Candidatus_Gamibacteraceae_bacterium
GCA_903927415.1_Candidatus_Gamibacteraceae_bacterium
GCA_903878685.1_Candidatus_Gamibacteraceae_bacterium
GCA_903917585.1_Candidatus_Gamibacteraceae_bacterium
GCA_001768015.1_Candidatus_Gamibacteraceae_bacterium
GCA_903837365.1_Candidatus_Gamibacteraceae_bacterium
GCA_014190355.1_Candidatus_Gamibacteraceae_bacterium
GCA_009921385.1_Candidatus_Gamibacteraceae_bacterium
GCA_903959715.1_Candidatus_Gamibacteraceae_bacterium
GCA_009925685.1_Candidatus_Gamibacteraceae_bacterium
GCA_016778745.1_Candidatus_Gamibacteraceae_bacterium
GCA_009693885.1_Candidatus_Gamibacteraceae_bacterium
GCA_905478245.1_Candidatus_Gamibacteraceae_bacterium
GCA_905478165.1_Candidatus_Gamibacteraceae_bacterium
GCA_018659605.1_Candidatus_Diomedesiaceae_bacterium
GCA_018659145.1_Candidatus_Diomedesiaceae_bacterium
GCA_016778805.1_Candidatus_Diomedesiaceae_bacterium
GCA_016780625.1_Candidatus_Diomedesiaceae_bacterium
GCA_905480015.1_Candidatus_Deianiraeaceae_bacterium
Rickettsiaceae_endosymbiont_of_Amblyomma_cajennense_Ac37b_GCA_000746585.2
GCA_003531345.1_Candidatus_Jistubacteraceae_bacterium
GCA_002787635.1_Candidatus_Athabascaceae_bacterium
GCA_002422795.1_Candidatus_Athabascaceae_bacterium
GCA_903821695.1_Candidatus_Jistubacteraceae_bacterium
GCA_010031735.1_Candidatus_Jistubacteraceae_bacterium
GCA_013288565.1_Candidatus_Jistubacteraceae_bacterium
GCA_013288575.1_Candidatus_Arkhamiaceae_bacterium
GCA_002787615.1_Candidatus_Arkhamiaceae_bacterium
GCA_002422205.1_Candidatus_Mitibacteraceae_bacterium
GCA_002346425.1_Candidatus_Mitibacteraceae_bacterium
GCA_002725445.1_Candidatus_Mitibacteraceae_bacterium
GCA_003241645.1_Candidatus_Mitibacteraceae_bacterium
GCA_002422745.1_Candidatus_Mitibacteraceae_bacterium
GCA_002291925.1_Candidatus_Mitibacteraceae_bacterium
GCA_002291845.1_Candidatus_Mitibacteraceae_bacterium
GCA_004293935.1_Candidatus_Mitibacteraceae_bacterium
GCA_002423155.1_Candidatus_Mitibacteraceae_bacterium
GCA_013288985.1_Candidatus_Mitibacteraceae_bacterium
GCA_013288335.1_Candidatus_Mitibacteraceae_bacterium
GCA_013289445.1_Candidatus_Mitibacteraceae_bacterium
GCA_013288305.1_Candidatus_Mitibacteraceae_bacterium
GCA_009926845.1_Candidatus_Mitibacteraceae_bacterium
GCA_005792635.1_Candidatus_Mitibacteraceae_bacterium
alphaproteobacterium_L41A_GCA_000335735.1
Brevundimonas_subvibrioides_GCA_000144605.1
Asticcacaulis_excentricus_GCA_000175215.2
Phenylobacterium_zucineum_GCA_000017265.1
Caulobacter_vibrioides_GCA_000022005.1
Oceanicaulis_alexandrii_GCA_000420265.1
Maricaulis_maris_GCA_003634045.1
Hyphomonas_neptunium_GCA_000013025.1
Hirschia_baltica_GCA_000023785.1
Parvularcula_bermudensis_GCA_000152825.2
Rhodobacter_sphaeroides_GCA_000015985.1
Paracoccus_denitrificans_GCA_004063735.1
Rubellimicrobium_rubrum_GCA_006152115.1
Ketogulonicigenium_vulgare_GCA_001399515.1
Sagittula_stellata_GCA_000169415.1
Ruegeria_pomeroyi_GCA_000011965.2
Octadecabacter_arcticus_GCA_000155735.2
Jannaschia_rubra_GCA_900113265.1
Neomegalonema_perideroedes_GCA_000374145.1
Rhizobium_leguminosarum_GCA_011604525.1
Bartonella_quintana_GCA_009936175.1
Chelativorans_oligotrophicus_GCA_011317505.1
Ahrensia_kielensis_GCA_000374465.1
Pelagibacterium_halotolerans_GCA_013004005.1
Roseibium_hamelinense_GCA_007830545.1
Polymorphum_gilvum_GCA_000192745.1
Pseudovibrio_sp._GCA_000236645.1
Methylocystis_bryophila_GCA_002117405.1
Methylocella_silvestris_GCA_000021745.1
Methylorubrum_extorquens_GCA_000018845.1
Xanthobacter_autotrophicus_GCA_000017645.1
Rhodopseudomonas_palustris_GCA_013415845.1
Thermopetrobacter_sp._GCA_000746275.1
Rhodomicrobium_vannielii_GCA_000166055.1
Hyphomicrobium_denitrificans_GCA_000143145.1
alphaproteobacterium_IMCC14465_GCA_000293845.2
Candidatus_Micropelagos_thuwalensis_GCA_000469155_1
Parvibaculum_lavamentivorans_GCA_000017565.1
Sphingopyxis_macrogoltabida_GCA_001314325.1
Citromicrobium_sp._GCA_001304795.1
Sphingobium_indicum_GCA_000264945.2
Zymomonas_mobilis_GCA_000007105.1
Sphingomonas_wittichii_GCA_000016765.1
alphaproteobacterium_AAP81b_GCA_001295935.1
Kordiimonas_gwangyangensis_GCA_000375545.1
Sneathiella_glossodoripedis_GCA_000616095.1
Candidatus_Paracaedibacter_acanthamoebae_GCA_000742835.1
Candidatus_Odyssella_thessalonicensis_GCA_000190415.2
Candidatus_Finniella_inopinata_GCA_004210305.1
Candidatus_Caedibacter_acanthamoebae_GCA_000743035.1
Caedimonas_varicaedens_GCA_001192655.1
Candidatus_Nucleicultrix_amoebiphila_GCA_002117145.1
Rhodovibrio_salinarum_GCA_000515255.1
Micavibrio_aeruginosavorus_GCA_000226315.1
Inquilinus_limosus_GCA_000423185.1
Rhodospirillum_centenum_GCA_014197805.1
alphaproteobacterium_BAL199_GCA_000171835.1
Thalassobaculum_salexigens_GCA_000423805.1
Candidatus_Puniceispirillum_marinum_GCA_000024465.1
Oceanibaculum_indicum_GCA_003633955.1
Rhodospirillum_rubrum_GCA_000225955.1
Pararhodospirillum_photometricum_GCA_000284415.2
Caenispirillum_salinarum_GCA_000315795.1
Roseospirillum_parvum_GCA_900100455.1
Magnetospirillum_magneticum_GCA_000009985.1
Terasakiella_pusilla_GCA_000688235.1
Kiloniella_laminariae_GCA_000374005.1
Thalassospira_profundimaris_GCA_000300275.1
Gluconacetobacter_diazotrophicus_GCA_000067045.1
Acetobacter_aceti_GCA_000193495.2
Gluconobacter_oxydans_GCA_000583855.1
Commensalibacter_intestini_GCA_000231445.2
Granulibacter_bethesdensis_GCA_000576145.2
Acidisphaera_rubrifaciens_GCA_000964365.1
Acidiphilium_multivorum_GCA_000202835.1
Rubritepida_flocculans_GCA_000425365.1
Belnapia_moabensis_GCA_000745835.1
Roseomonas_gilardii_GCA_001941945.1
Elioraea_tepidiphila_GCA_000378465.1
Geminicoccus_roseus_GCA_000427665.1
Tistrella_mobilis_GCA_000264455.2
Magnetofaba_australis_GCA_002109495.1
Magnetococcus_marinus_GCA_000014865.1
Ralstonia_pickettii_GCA_902374465.1
Burkholderia_cepacia_GCA_009586235.1
Nitrosospira_briensis_GCA_000619905.2
Methylotenera_versatilis_GCA_000093025.1
Dasania_marina_GCA_000373485.1
Congregibacter_litoralis_GCA_000153125.2
Enterobacter_cloacae_GCA_000770155.1
Alteromonas_macleodii_GCA_000172635.2

Purine_z_0.20

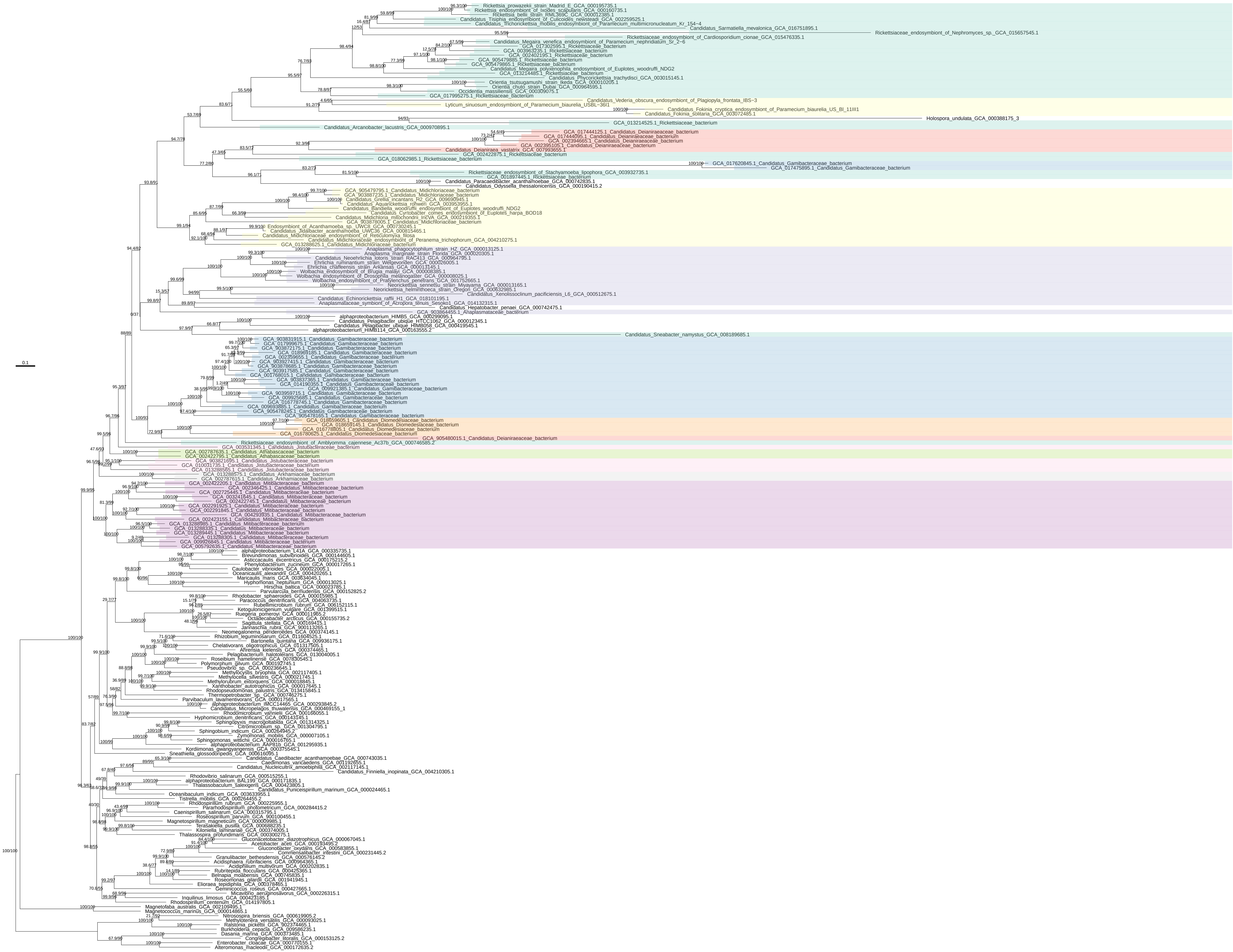
Rickettsia_prowazekii_strain_Madrid_E_GCA_000195735.1
Rickettsia_endosymbiont_of_Ixodes_scapularis_GCA_000160735.1
Rickettsia_bellii_strain_RML369C_GCA_000012385.1
Candidatus_Tisiphia_endosymbiont_of_Culicoides_newsteadi_GCA_002259525.1
Candidatus_Trichorickettsia_mobilis_endosymbiont_of_Paramecium_multimicronucleatum_Kr_154-4
Candidatus_Sarmatiella_mevalonica_GCA_016751895.1
Rickettsiaceae_endosymbiont_of_Nephromyces_sp._GCA_015657545.1
Rickettsiaceae_endosymbiont_of_Cardiosporidium_cionae_GCA_015476335.1
Candidatus_Megaira_venefica_endosymbiont_of_Paramecium_nephridiatum_Sr_2-6
GCA_017302595.1_Rickettsiaceae_bacterium
GCA_003963235.1_Rickettsiaceae_bacterium
GCA_002402195.1_Rickettsiaceae_bacterium
GCA_905479885.1_Rickettsiaceae_bacterium
GCA_905479865.1_Rickettsiaceae_bacterium
Candidatus_Megaira_polyxenophila_endosymbiont_of_Euplotes_woodruffi_NDG2
GCA_013214485.1_Rickettsiaceae_bacterium
Candidatus_Phycorickettsia_trachydisci_GCA_003015145.1
Orientia_tsutsugamushi_strain_Ikeda_GCA_000010205.1
Orientia_chuto_strain_Dubai_GCA_000964595.1
Occidentia_massiliensis_GCA_000309075.1
GCA_017995275.1_Rickettsiaceae_bacterium
Candidatus_Vederia_obscura_endosymbiont_of_Plagiopyla_frontata_IBS-3
Lyticum_sinuosum_endosymbiont_of_Paramecium_biaurelia_USBL-36I1
Candidatus_Fokinia_cryptica_endosymbiont_of_Paramecium_biaurelia_US_Bl_11III1
Candidatus_Fokinia_solitaria_GCA_003072485.1
Holospora_undulata_GCA_000388175_3
GCA_013214525.1_Rickettsiaceae_bacterium
Candidatus_Arcanobacter_lacustris_GCA_000970895.1
GCA_017444125.1_Candidatus_Deianiraeaceae_bacterium
GCA_017444095.1_Candidatus_Deianiraeaceae_bacterium
GCA_002394665.1_Candidatus_Deianiraeaceae_bacterium
GCA_002395105.1_Candidatus_Deianiraeaceae_bacterium
Candidatus_Deianiraea_vastatrix_GCA_007993655.1
GCA_002422875.1_Rickettsiaceae_bacterium
GCA_018062985.1_Rickettsiaceae_bacterium
GCA_017620845.1_Candidatus_Gamibacteraceae_bacterium
GCA_017475895.1_Candidatus_Gamibacteraceae_bacterium
Rickettsiaceae_endosymbiont_of_Stachyamoeba_lipophora_GCA_003932735.1
GCA_001897445.1_Rickettsiaceae_bacterium
Candidatus_Paracaedibacter_acanthamoebae_GCA_000742835.1
Candidatus_Odyssella_thessalonicensis_GCA_000190415.2
GCA_905479795.1_Candidatus_Midichloriaceae_bacterium
GCA_903887235.1_Candidatus_Midichloriaceae_bacterium
Candidatus_Grellia_incantans_R2_GCA_009690945.1
Candidatus_Aquarickettsia_rohweri_GCA_003953955.1
Candidatus_Bandiella_woodruffii_endosymbiont_of_Euplotes_woodruffi_NDG2
Candidatus_Cyrtobacter_comes_endosymbiont_of_Euplotes_harpa_BOD18
Candidatus_Midichloria_mitochondrii_IricVA_GCA_000219355.1
GCA_903878005.1_Candidatus_Midichloriaceae_bacterium
Endosymbiont_of_Acanthamoeba_sp._UWC8_GCA_000730245.1
Candidatus_Jidaibacter_acanthamoeba_UWC36_GCA_000815465.1
Candidatus_Midichloriaceae_endosymbiont_of_Reticulomyxa_filosa
Candidatus_Midichloriaceae_endosymbiont_of_Peranema_trichophorum_GCA_004210275.1
GCA_013288625.1_Candidatus_Midichloriaceae_bacterium
Anaplasma_phagocytophilum_strain_HZ_GCA_000013125.1
Anaplasma_marginale_strain_Florida_GCA_000020305.1
Candidatus_Neoehrlichia_lotoris_strain_RAC413_GCA_000964795.1
Ehrlichia_ruminantium_strain_Welgevonden_GCA_000026005.1
Ehrlichia_chaffeensis_strain_Arkansas_GCA_000013145.1
Wolbachia_endosymbiont_of_Brugia_malayi_GCA_000008385.1
Wolbachia_endosymbiont_of_Drosophila_melanogaster_GCA_000008025.1
Wolbachia_endosymbiont_of_Pratylenchus_penetrans_GCA_001752665.1
Neorickettsia_sennetsu_strain_Miyayama_GCA_000013165.1
Neorickettsia_helminthoeca_strain_Oregon_GCA_000632985.1
Candidatus_Xenolissoclinum_pacificiensis_L6_GCA_000512675.1
Candidatus_Echinorickettsia_raffii_H1_GCA_018101195.1
Anaplasmataceae_symbiont_of_Acropora_tenuis_Sesoko1_GCA_014132315.1
Candidatus_Hepatobacter_penaei_GCA_000742475.1
GCA_903864455.1_Anaplasmataceae_bacterium
alphaproteobacterium_HIMB5_GCA_000299095.1
Candidatus_Pelagibacter_ubique_HTCC1062_GCA_000012345.1
Candidatus_Pelagibacter_ubique_HIMB058_GCA_000419545.1
alphaproteobacterium_HIMB114_GCA_000163555.2
Candidatus_Sneabacter_namystus_GCA_008189685.1
0.1
GCA_903831915.1_Candidatus_Gamibacteraceae_bacterium
GCA_017999675.1_Candidatus_Gamibacteraceae_bacterium
GCA_903872175.1_Candidatus_Gamibacteraceae_bacterium
GCA_018969185.1_Candidatus_Gamibacteraceae_bacterium
GCA_002359655.1_Candidatus_Gamibacteraceae_bacterium
GCA_903927415.1_Candidatus_Gamibacteraceae_bacterium
GCA_903878685.1_Candidatus_Gamibacteraceae_bacterium
GCA_903917585.1_Candidatus_Gamibacteraceae_bacterium
GCA_001768015.1_Candidatus_Gamibacteraceae_bacterium
GCA_903837365.1_Candidatus_Gamibacteraceae_bacterium
GCA_014190355.1_Candidatus_Gamibacteraceae_bacterium
GCA_009921385.1_Candidatus_Gamibacteraceae_bacterium
GCA_903959715.1_Candidatus_Gamibacteraceae_bacterium
GCA_009925685.1_Candidatus_Gamibacteraceae_bacterium
GCA_016778745.1_Candidatus_Gamibacteraceae_bacterium
GCA_009693885.1_Candidatus_Gamibacteraceae_bacterium
GCA_905478245.1_Candidatus_Gamibacteraceae_bacterium
GCA_905478165.1_Candidatus_Gamibacteraceae_bacterium
GCA_018659605.1_Candidatus_Diomedesiaceae_bacterium
GCA_018659145.1_Candidatus_Diomedesiaceae_bacterium
GCA_016778805.1_Candidatus_Diomedesiaceae_bacterium
GCA_016780625.1_Candidatus_Diomedesiaceae_bacterium
GCA_905480015.1_Candidatus_Deianiraeaceae_bacterium
Rickettsiaceae_endosymbiont_of_Amblyomma_cajennese_Ac37b_GCA_000746585.2
GCA_003531345.1_Candidatus_Jistubacteraceae_bacterium
GCA_002787635.1_Candidatus_Athabascaceae_bacterium
GCA_002422795.1_Candidatus_Athabascaceae_bacterium
GCA_903821695.1_Candidatus_Jistubacteraceae_bacterium
GCA_010031735.1_Candidatus_Jistubacteraceae_bacterium
GCA_013288555.1_Candidatus_Jistubacteraceae_bacterium
GCA_013288575.1_Candidatus_Arkhamiaceae_bacterium
GCA_002787615.1_Candidatus_Arkhamiaceae_bacterium
GCA_002422205.1_Candidatus_Mitibacteraceae_bacterium
GCA_002346425.1_Candidatus_Mitibacteraceae_bacterium
GCA_002725445.1_Candidatus_Mitibacteraceae_bacterium
GCA_003241645.1_Candidatus_Mitibacteraceae_bacterium
GCA_002422745.1_Candidatus_Mitibacteraceae_bacterium
GCA_002291925.1_Candidatus_Mitibacteraceae_bacterium
GCA_002291845.1_Candidatus_Mitibacteraceae_bacterium
GCA_004293935.1_Candidatus_Mitibacteraceae_bacterium
GCA_002423155.1_Candidatus_Mitibacteraceae_bacterium
GCA_013288985.1_Candidatus_Mitibacteraceae_bacterium
GCA_013288335.1_Candidatus_Mitibacteraceae_bacterium
GCA_013289445.1_Candidatus_Mitibacteraceae_bacterium
GCA_013288305.1_Candidatus_Mitibacteraceae_bacterium
GCA_009926845.1_Candidatus_Mitibacteraceae_bacterium
GCA_005792635.1_Candidatus_Mitibacteraceae_bacterium
alphaproteobacterium_L41A_GCA_000335735.1
Brevundimonas_subvibrioides_GCA_000144605.1
Asticcacaulis_excentricus_GCA_000175215.2
Phenylobacterium_zucineum_GCA_000017265.1
Caulobacter_vibrioides_GCA_000022005.1
Oceanicaulis_alexandrii_GCA_000420265.1
Maricaulis_maris_GCA_003634045.1
Hyphomonas_neptunium_GCA_000013025.1
Hirschia_baltica_GCA_000023785.1
Parvularcula_bermudensis_GCA_000152825.2
Rhodobacter_sphaeroides_GCA_000015985.1
Paracoccus_denitrificans_GCA_004063735.1
Rubellimicrobium_rubrum_GCA_006152115.1
Ketogulonicigenium_vulgare_GCA_001399515.1
Ruegeria_pomeroyi_GCA_000011965.2
Octadecabacter_arcticus_GCA_000155735.2
Sagittula_stellata_GCA_000169415.1
Jannaschia_rubra_GCA_900113265.1
Neomegalonema_perideroedes_GCA_000374145.1
Rhizobium_leguminosarum_GCA_011604525.1
Bartonella_quintana_GCA_009936175.1
Chelativorans_oligotrophicus_GCA_011317505.1
Ahrensia_kielensis_GCA_000374465.1
Pelagibacterium_halotolerans_GCA_013004005.1
Roseibium_hamelinense_GCA_007830545.1
Polymorphum_gilvum_GCA_000192745.1
Pseudovibrio_sp._GCA_000236645.1
Methylocystis_bryophila_GCA_002117405.1
Methylocella_silvestris_GCA_000021745.1
Methylorubrum_extorquens_GCA_000018845.1
Xanthobacter_autotrophicus_GCA_000017645.1
Rhodopseudomonas_palustris_GCA_013415845.1
Thermopetrobacter_sp._GCA_000746275.1
Parvibaculum_lavamentivorans_GCA_000017565.1
alphaproteobacterium_IMCC14465_GCA_000293845.2
Candidatus_Micropelagos_thuwalensis_GCA_000469155_1
Rhodomicrobium_vannielii_GCA_000166055.1
Hyphomicrobium_denitrificans_GCA_000143145.1
Sphingopyxis_macrogoltabida_GCA_001314325.1
Citromicrobium_sp._GCA_001304795.1
Sphingobium_indicum_GCA_000264945.2
Zymomonas_mobilis_GCA_000007105.1
Sphingomonas_wittichii_GCA_000016765.1
alphaproteobacterium_AAP81b_GCA_001295935.1
Kordiimonas_gwangyangensis_GCA_000375545.1
Sneathiella_glossodoripedis_GCA_000616095.1
Candidatus_Caedibacter_acanthamoebae_GCA_000743035.1
Caedimonas_varicaedens_GCA_001192655.1
Candidatus_Nucleicultrix_amoebiphila_GCA_002117145.1
Candidatus_Finniella_inopinata_GCA_004210305.1
Rhodovibrio_salinarum_GCA_000515255.1
alphaproteobacterium_BAL199_GCA_000171835.1
Thalassobaculum_salexigens_GCA_000423805.1
Candidatus_Puniceispirillum_marinum_GCA_000024465.1
Oceanibaculum_indicum_GCA_003633955.1
Tistrella_mobilis_GCA_000264455.2
Rhodospirillum_rubrum_GCA_000225955.1
Pararhodospirillum_photometricum_GCA_000284415.2
Caenispirillum_salinarum_GCA_000315795.1
Roseospirillum_parvum_GCA_900100455.1
Magnetospirillum_magneticum_GCA_000009985.1
Terasakiella_pusilla_GCA_000688235.1
Kiloniella_laminariae_GCA_000374005.1
Thalassospira_profundimaris_GCA_000300275.1
Gluconacetobacter_diazotrophicus_GCA_000067045.1
Acetobacter_aceti_GCA_001193495.2
Gluconobacter_oxydans_GCA_000583855.1
Commensalibacter_intestini_GCA_000231445.2
Granulibacter_bethesdensis_GCA_000576145.2
Acidisphaera_rubrifaciens_GCA_000964365.1
Acidiphilium_multivorum_GCA_000202835.1
Rubritepida_flocculans_GCA_000425365.1
Belnapia_moabensis_GCA_000745835.1
Roseomonas_gilardii_GCA_001941945.1
Elioraea_tepidiphila_GCA_000378465.1
Geminicoccus_roseus_GCA_000427665.1
Micavibrio_aeruginosavorus_GCA_000226315.1
Inquilinus_limosus_GCA_000423185.1
Rhodospirillum_centenum_GCA_014197805.1
Magnetofaba_australis_GCA_002109495.1
Magnetococcus_marinus_GCA_000014865.1
Nitrosospira_briensis_GCA_000619905.2
Methylotenera_versatilis_GCA_000093025.1
Ralstonia_pickettii_GCA_902374465.1
Burkholderia_cepacia_GCA_009586235.1
Dasania_marina_GCA_000373485.1
Congregibacter_litoralis_GCA_000153125.2
Enterobacter_cloacae_GCA_000770155.1
Alteromonas_macleodii_GCA_000172635.2

Purine_z_0.30

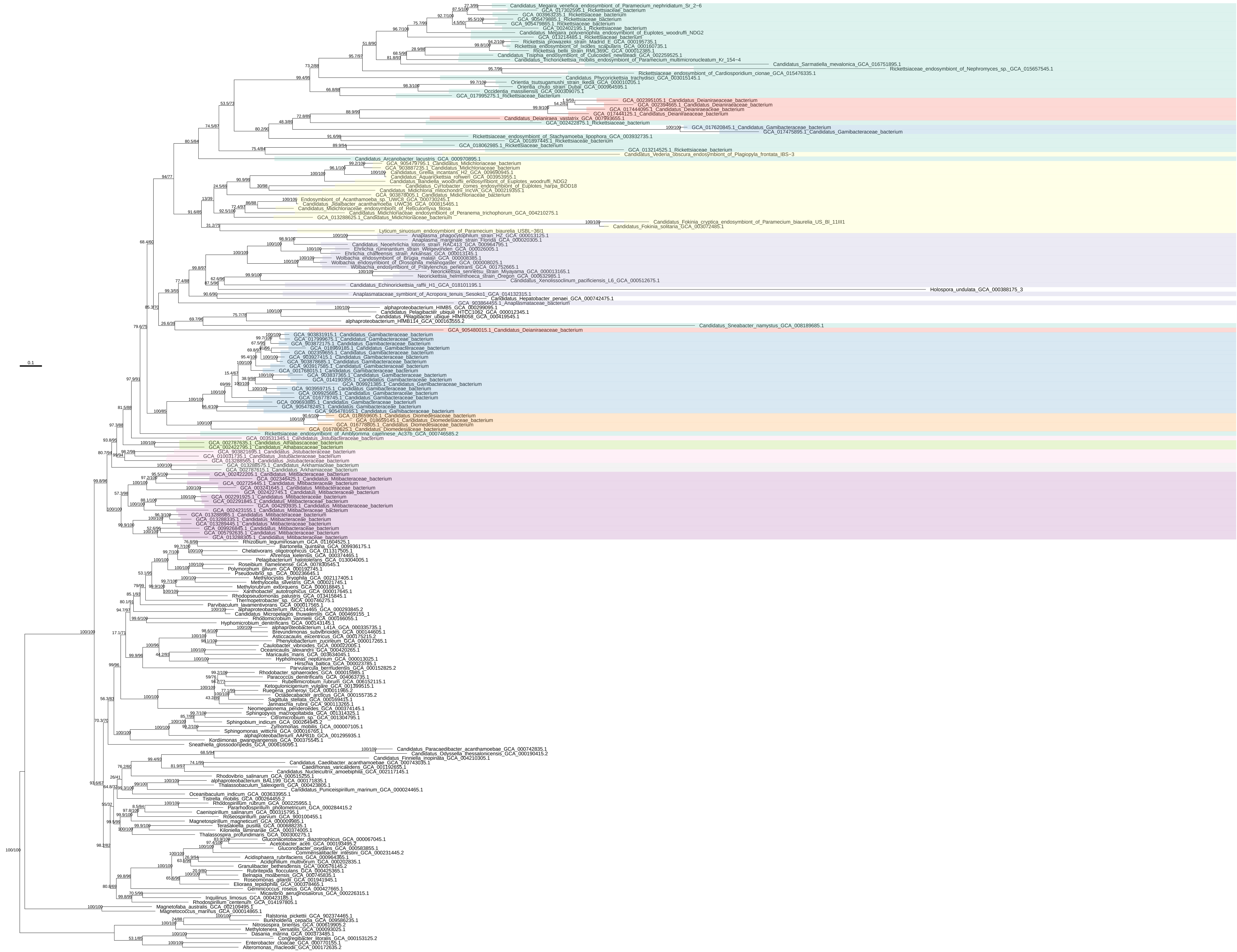
0.1
Candidatus_Megaira_venefica_endosymbiont_of_Paramecium_nephridiatum_Sr_2-6
GCA_017302595.1_Rickettsiaceae_bacterium
GCA_003963235.1_Rickettsiaceae_bacterium
GCA_905479885.1_Rickettsiaceae_bacterium
GCA_905479865.1_Rickettsiaceae_bacterium
GCA_002402195.1_Rickettsiaceae_bacterium
Candidatus_Megaira_polyxenophila_endosymbiont_of_Euplotes_woodruffi_NDG2
GCA_013214485.1_Rickettsiaceae_bacterium
Rickettsia_prowazekii_strain_Madrid_E_GCA_000195735.1
Rickettsia_endosymbiont_of_Ixodes_scapularis_GCA_000160735.1
Rickettsia_bellii_strain_RML369C_GCA_000012385.1
Candidatus_Tisiphia_endosymbiont_of_Culicoides_newsteadi_GCA_002259525.1
Candidatus_Trichorickettsia_mobilis_endosymbiont_of_Paramecium_multimicronucleatum_Kr_154-4
Candidatus_Sarmatiella_mevalonica_GCA_016751895.1
Rickettsiaceae_endosymbiont_of_Nephromyces_sp._GCA_015657545.1
Rickettsiaceae_endosymbiont_of_Cardiosporidium_cionae_GCA_015476335.1
Candidatus_Phycorickettsia_trachydisci_GCA_003015145.1
Orientia_tsutsugamushi_strain_Ikeda_GCA_000010205.1
Orientia_chuto_strain_Dubai_GCA_000964595.1
Occidentia_massiliensis_GCA_000309075.1
GCA_017995275.1_Rickettsiaceae_bacterium
GCA_002395105.1_Candidatus_Deianiraeaceae_bacterium
GCA_002394665.1_Candidatus_Deianiraeaceae_bacterium
GCA_017444095.1_Candidatus_Deianiraeaceae_bacterium
GCA_017444125.1_Candidatus_Deianiraeaceae_bacterium
Candidatus_Deianiraea_vastatrix_GCA_007993655.1
GCA_002422875.1_Rickettsiaceae_bacterium
GCA_017620845.1_Candidatus_Gamibacteraceae_bacterium
GCA_017475895.1_Candidatus_Gamibacteraceae_bacterium
Rickettsiaceae_endosymbiont_of_Stachyamoeba_lipophora_GCA_003932735.1
GCA_001897445.1_Rickettsiaceae_bacterium
GCA_018062985.1_Rickettsiaceae_bacterium
GCA_013214525.1_Rickettsiaceae_bacterium
Candidatus_Vederia_obscura_endosymbiont_of_Plagiopyla_frontata_IBS-3
Candidatus_Arcanobacter_lacustris_GCA_000970895.1
GCA_905479795.1_Candidatus_Midichloriaceae_bacterium
GCA_903887235.1_Candidatus_Midichloriaceae_bacterium
Candidatus_Grellia_incantans_H2_GCA_009690945.1
Candidatus_Aquarickettsia_rohweri_GCA_003953955.1
Candidatus_Bandiella_woodruffii_endosymbiont_of_Euplotes_woodruffi_NDG2
Candidatus_Cyrtobacter_comes_endosymbiont_of_Euplotes_harpa_BOD18
Candidatus_Midichloria_mitochondrii_IricVA_GCA_000219355.1
GCA_903878005.1_Candidatus_Midichloriaceae_bacterium
Endosymbiont_of_Acanthamoeba_sp._UWC8_GCA_000730245.1
Candidatus_Jidaibacter_acanthamoeba_UWC36_GCA_000815465.1
Candidatus_Midichloriaceae_endosymbiont_of_Reticulomyxa_filosa
Candidatus_Midichloriaceae_endosymbiont_of_Peranema_trichophorum_GCA_004210275.1
GCA_013288625.1_Candidatus_Midichloriaceae_bacterium
Candidatus_Fokinia_cryptica_endosymbiont_of_Paramecium_biaurelia_US_Bl_11III1
Candidatus_Fokinia_solitaria_GCA_003072485.1
Lyticum_sinuosum_endosymbiont_of_Paramecium_biaurelia_USBL-36I1
Anaplasma_phagocytophilum_strain_HZ_GCA_000013125.1
Anaplasma_marginale_strain_Florida_GCA_000020305.1
Candidatus_Neoehrlichia_lotoris_strain_RAC413_GCA_000964795.1
Ehrlichia_ruminantium_strain_Welgevonden_GCA_000026005.1
Ehrlichia_chaffeensis_strain_Arkansas_GCA_000013145.1
Wolbachia_endosymbiont_of_Brugia_malayi_GCA_000008385.1
Wolbachia_endosymbiont_of_Drosophila_melanogaster_GCA_000008025.1
Wolbachia_endosymbiont_of_Pratylenchus_penetrans_GCA_001752665.1
Neorickettsia_sennetsu_strain_Miyayama_GCA_000013165.1
Neorickettsia_helminthoeca_strain_Oregon_GCA_000632985.1
Candidatus_Xenolissoclinum_pacificiensis_L6_GCA_000512675.1
Candidatus_Echinorickettsia_raffii_H1_GCA_018101195.1
Holospora_undulata_GCA_000388175_3
Anaplasmataceae_symbiont_of_Acropora_tenuis_Sesoko1_GCA_014132315.1
Candidatus_Hepatobacter_penaei_GCA_000742475.1
GCA_903864455.1_Anaplasmataceae_bacterium
alphaproteobacterium_HIMB5_GCA_000299095.1
Candidatus_Pelagibacter_ubique_HTCC1062_GCA_000012345.1
Candidatus_Pelagibacter_ubique_HIMB058_GCA_000419545.1
alphaproteobacterium_HIMB114_GCA_000163555.2
Candidatus_Sneabacter_namystus_GCA_008189685.1
GCA_905480015.1_Candidatus_Deianiraeaceae_bacterium
GCA_903831915.1_Candidatus_Gamibacteraceae_bacterium
GCA_017999675.1_Candidatus_Gamibacteraceae_bacterium
GCA_903872175.1_Candidatus_Gamibacteraceae_bacterium
GCA_018969185.1_Candidatus_Gamibacteraceae_bacterium
GCA_002359655.1_Candidatus_Gamibacteraceae_bacterium
GCA_903927415.1_Candidatus_Gamibacteraceae_bacterium
GCA_903878685.1_Candidatus_Gamibacteraceae_bacterium
GCA_903917585.1_Candidatus_Gamibacteraceae_bacterium
GCA_001768015.1_Candidatus_Gamibacteraceae_bacterium
GCA_903837365.1_Candidatus_Gamibacteraceae_bacterium
GCA_014190355.1_Candidatus_Gamibacteraceae_bacterium
GCA_009921385.1_Candidatus_Gamibacteraceae_bacterium
GCA_903959715.1_Candidatus_Gamibacteraceae_bacterium
GCA_009925685.1_Candidatus_Gamibacteraceae_bacterium
GCA_016778745.1_Candidatus_Gamibacteraceae_bacterium
GCA_009693885.1_Candidatus_Gamibacteraceae_bacterium
GCA_905478245.1_Candidatus_Gamibacteraceae_bacterium
GCA_905478165.1_Candidatus_Gamibacteraceae_bacterium
GCA_018659605.1_Candidatus_Diomedesiaceae_bacterium
GCA_018659145.1_Candidatus_Diomedesiaceae_bacterium
GCA_016778805.1_Candidatus_Diomedesiaceae_bacterium
GCA_016780625.1_Candidatus_Diomedesiaceae_bacterium
Rickettsiaceae_endosymbiont_of_Amblyomma_cajennense_Ac37b_GCA_000746585.2
GCA_003531345.1_Candidatus_Jistubacteraceae_bacterium
GCA_002787635.1_Candidatus_Athabascaceae_bacterium
GCA_002422795.1_Candidatus_Athabascaceae_bacterium
GCA_903821695.1_Candidatus_Jistubacteraceae_bacterium
GCA_010031735.1_Candidatus_Jistubacteraceae_bacterium
GCA_013288565.1_Candidatus_Jistubacteraceae_bacterium
GCA_013288575.1_Candidatus_Arkhamiaceae_bacterium
GCA_002787615.1_Candidatus_Arkhamiaceae_bacterium
GCA_002422205.1_Candidatus_Mitibacteraceae_bacterium
GCA_002346425.1_Candidatus_Mitibacteraceae_bacterium
GCA_002725445.1_Candidatus_Mitibacteraceae_bacterium
GCA_003241645.1_Candidatus_Mitibacteraceae_bacterium
GCA_002422745.1_Candidatus_Mitibacteraceae_bacterium
GCA_002291925.1_Candidatus_Mitibacteraceae_bacterium
GCA_002291845.1_Candidatus_Mitibacteraceae_bacterium
GCA_004293935.1_Candidatus_Mitibacteraceae_bacterium
GCA_002423155.1_Candidatus_Mitibacteraceae_bacterium
GCA_013288985.1_Candidatus_Mitibacteraceae_bacterium
GCA_013288335.1_Candidatus_Mitibacteraceae_bacterium
GCA_013289445.1_Candidatus_Mitibacteraceae_bacterium
GCA_009926845.1_Candidatus_Mitibacteraceae_bacterium
GCA_005792635.1_Candidatus_Mitibacteraceae_bacterium
GCA_013288305.1_Candidatus_Mitibacteraceae_bacterium
Rhizobium_leguminosarum_GCA_011604525.1
Bartonella_quintana_GCA_009936175.1
Chelativorans_oligotrophicus_GCA_011317505.1
Ahrensia_kielensis_GCA_000374465.1
Pelagibacterium_halotolerans_GCA_013004005.1
Roseibium_hamelinense_GCA_007830545.1
Polymorphum_gilvum_GCA_000192745.1
Pseudovibrio_sp._GCA_000236645.1
Methylocystis_bryophila_GCA_002117405.1
Methylocella_silvestris_GCA_000021745.1
Methylorubrum_extorquens_GCA_000018845.1
Xanthobacter_autotrophicus_GCA_000017645.1
Rhodopseudomonas_palustris_GCA_013415845.1
Thermopetrobacter_sp._GCA_000746275.1
Parvibaculum_lavamentivorans_GCA_000017565.1
alphaproteobacterium_IMCC14465_GCA_000293845.2
Candidatus_Micropelagos_thuwalensis_GCA_000469155.1
Rhodomicrobium_vannielii_GCA_000166055.1
Hyphomicrobium_denitrificans_GCA_000143145.1
alphaproteobacterium_L41A_GCA_000335735.1
Brevundimonas_subvibrioides_GCA_000144605.1
Asticcacaulis_excentricus_GCA_000175215.2
Phenylobacterium_zucineum_GCA_000017265.1
Caulobacter_vibrioides_GCA_000022005.1
Oceanicaulis_alexandrii_GCA_000420265.1
Maricaulis_maris_GCA_003634045.1
Hyphomonas_neptunium_GCA_000013025.1
Hirschia_baltica_GCA_000023785.1
Parvularcula_bermudensis_GCA_000152825.2
Rhodobacter_sphaeroides_GCA_000015985.1
Paracoccus_denitrificans_GCA_004063735.1
Rubellimicrobium_rubrum_GCA_006152115.1
Ketogulonicigenium_vulgare_GCA_001399515.1
Ruegeria_pomeroyi_GCA_000011965.2
Octadecabacter_arcticus_GCA_000155735.2
Sagittula_stellata_GCA_000169415.1
Jannaschia_rubra_GCA_900113265.1
Neomegalonema_perideroedes_GCA_000374145.1
Sphingopyxis_macrogoltabida_GCA_001314325.1
Citromicrobium_sp._GCA_001304795.1
Sphingobium_indicum_GCA_000264945.2
Zymomonas_mobilis_GCA_000007105.1
Sphingomonas_wittichii_GCA_000016765.1
alphaproteobacterium_AAP81b_GCA_001295935.1
Kordiimonas_gwangyangensis_GCA_000375545.1
Sneathiella_glossodoripedis_GCA_000616095.1
Candidatus_Paracaedibacter_acanthamoebae_GCA_000742835.1
Candidatus_Odyssella_thessalonicensis_GCA_000190415.2
Candidatus_Finniella_inopinata_GCA_004210305.1
Candidatus_Caedibacter_acanthamoebae_GCA_000743035.1
Caedimonas_varicaedens_GCA_001192655.1
Candidatus_Nucleicultrix_amoebiphila_GCA_002117145.1
Rhodovibrio_salinarum_GCA_000515255.1
alphaproteobacterium_BAL199_GCA_000171835.1
Thalassobaculum_salexigens_GCA_000423805.1
Candidatus_Puniceispirillum_marinum_GCA_000024465.1
Oceanibaculum_indicum_GCA_003633955.1
Tistrella_mobilis_GCA_000264455.2
Rhodospirillum_rubrum_GCA_000225955.1
Pararhodospirillum_photometricum_GCA_000284415.2
Caenispirillum_salinarum_GCA_000315795.1
Roseospirillum_parvum_GCA_900100455.1
Magnetospirillum_magneticum_GCA_000009985.1
Terasakiella_pusilla_GCA_000688235.1
Kiloniella_laminariae_GCA_000374005.1
Thalassospira_profundimaris_GCA_000300275.1
Gluconacetobacter_diazotrophicus_GCA_000067045.1
Acetobacter_aceti_GCA_000193495.2
Gluconobacter_oxydans_GCA_000583855.1
Commensalibacter_intestini_GCA_000231445.2
Acidisphaera_rubrifaciens_GCA_000964365.1
Acidiphilium_multivorum_GCA_000202835.1
Granulibacter_bethesdensis_GCA_000576145.2
Rubritepida_flocculans_GCA_000425365.1
Belnapia_moabensis_GCA_000745835.1
Roseomonas_gilardii_GCA_001941945.1
Elioraea_tepidiphila_GCA_000378465.1
Geminicoccus_roseus_GCA_000427665.1
Micavibrio_aeruginosavorus_GCA_000226315.1
Inquilinus_limosus_GCA_000423185.1
Rhodospirillum_centenum_GCA_014197805.1
Magnetofaba_australis_GCA_002109495.1
Magnetococcus_marinus_GCA_000014865.1
Ralstonia_pickettii_GCA_902374465.1
Burkholderia_cepacia_GCA_009586235.1
Nitrosospira_briensis_GCA_000619905.2
Methylotenera_versatilis_GCA_000093025.1
Dasania_marina_GCA_000373485.1
Congregibacter_litoralis_GCA_000153125.2
Enterobacter_cloacae_GCA_000770155.1
Alteromonas_macleodii_GCA_000172635.2

Purine_z_0.40

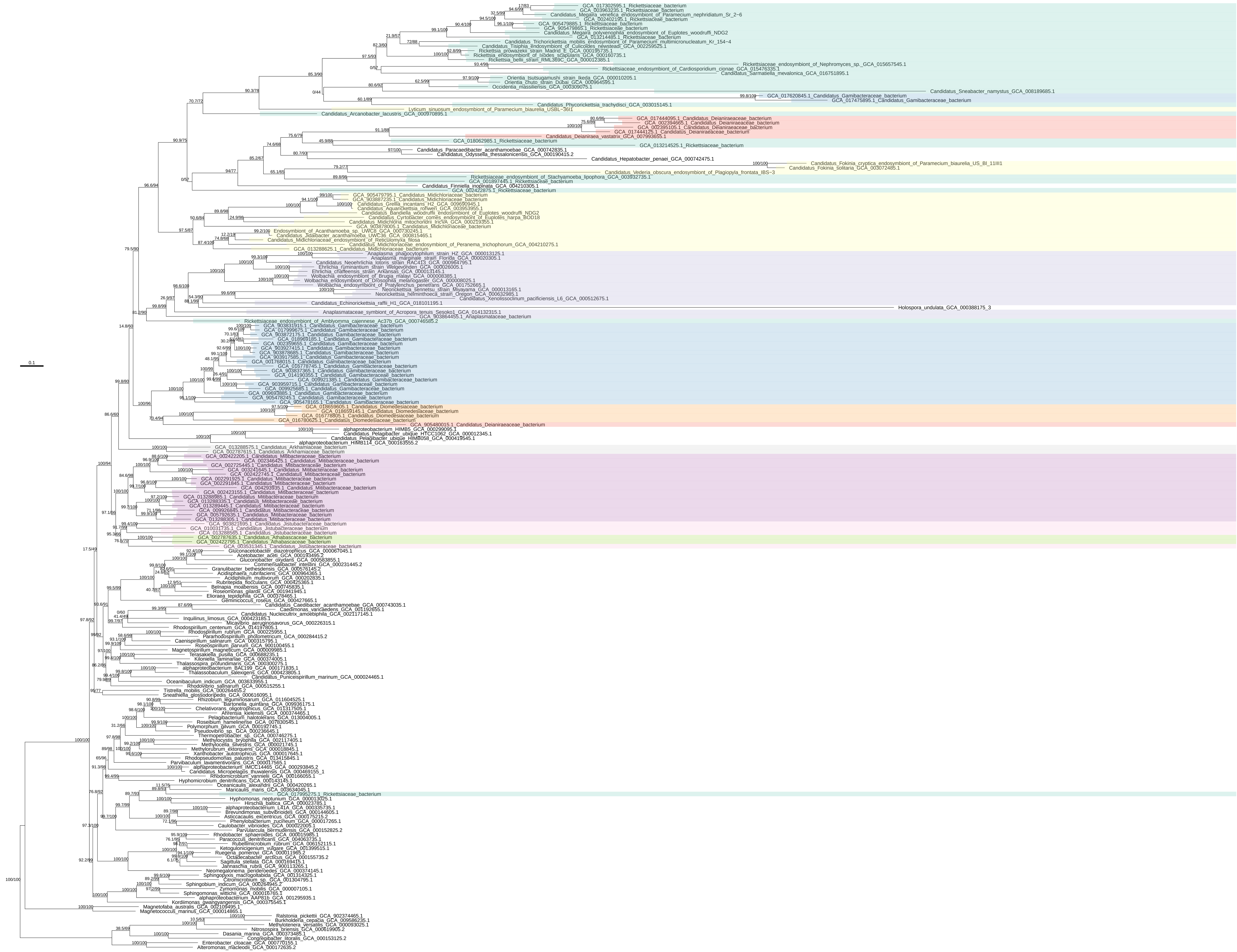

GCA_017302595.1_Rickettsiaceae_bacterium
GCA_003963235.1_Rickettsiaceae_bacterium
Candidatus_Megaira_venefica_endosymbiont_of_Paramecium_nephridiatum_Sr_2-6
GCA_002402195.1_Rickettsiaceae_bacterium
GCA_905479885.1_Rickettsiaceae_bacterium
GCA_905479865.1_Rickettsiaceae_bacterium
Candidatus_Megaira_polyxenophila_endosymbiont_of_Euplotes_woodruffi_NDG2
GCA_013214485.1_Rickettsiaceae_bacterium
Candidatus_Trichorickettsia_mobilis_endosymbiont_of_Paramecium_multimicronucleatum_Kr_154-4
Candidatus_Tisiphia_endosymbiont_of_Culicoides_newsteadi_GCA_002259525.1
Rickettsia_prowazekii_strain_Madrid_E_GCA_000195735.1
Rickettsia_endosymbiont_of_Ixodes_scapularis_GCA_000160735.1
Rickettsia_bellii_strain_RML369C_GCA_000012385.1
Rickettsiaceae_endosymbiont_of_Nephromyces_sp._GCA_015657545.1
Rickettsiaceae_endosymbiont_of_Cardiosporidium_cionae_GCA_015476335.1
Candidatus_Sarmatiella_mevalonica_GCA_016751895.1
Orientia_tsutsugamushi_strain_Ikeda_GCA_000010205.1
Orientia_chuto_strain_Dubai_GCA_000964595.1
Occidentia_massiliensis_GCA_003309075.1
Candidatus_Sneabacter_namystus_GCA_008189685.1
GCA_017620845.1_Candidatus_Gamibacteraceae_bacterium
GCA_017475895.1_Candidatus_Gamibacteraceae_bacterium
Candidatus_Phycorickettsia_trachydisci_GCA_003015145.1
Lyticum_sinuosum_endosymbiont_of_Paramecium_biaurelia_USBL-36I1
Candidatus_Arcanobacter_lacustris_GCA_000970895.1
GCA_017444095.1_Candidatus_Deianiraeaceae_bacterium
GCA_002394665.1_Candidatus_Deianiraeaceae_bacterium
GCA_002395105.1_Candidatus_Deianiraeaceae_bacterium
GCA_017444125.1_Candidatus_Deianiraeaceae_bacterium
Candidatus_Deianiraea_vastatrix_GCA_007993655.1
GCA_018062985.1_Rickettsiaceae_bacterium
GCA_013214525.1_Rickettsiaceae_bacterium
Candidatus_Paracaedibacter_acanthamoebae_GCA_000742835.1
Candidatus_Odyssella_thessalonicensis_GCA_000190415.2
Candidatus_Hepatobacter_penaei_GCA_000742475.1
Candidatus_Fokinia_cryptica_endosymbiont_of_Paramecium_biaurelia_US_Bl_11III1
Candidatus_Fokinia_solitaria_GCA_003072485.1
Candidatus_Vederia_obscura_endosymbiont_of_Plagiopyla_frontata_IBS-3
Rickettsiaceae_endosymbiont_of_Stachyamoeba_lipophora_GCA_003932735.1
GCA_001897445.1_Rickettsiaceae_bacterium
Candidatus_Finniella_inopinata_GCA_004210305.1
GCA_002422875.1_Rickettsiaceae_bacterium
GCA_905479795.1_Candidatus_Midichloriaceae_bacterium
GCA_903887235.1_Candidatus_Midichloriaceae_bacterium
Candidatus_Grellia_incantans_H2_GCA_009690945.1
Candidatus_Aquarickettsia_rohweri_GCA_003953955.1
Candidatus_Bandiella_woodruffii_endosymbiont_of_Euplotes_woodruffi_NDG2
Candidatus_Cyrtobacter_comes_endosymbiont_of_Euplotes_harpa_BOD18
Candidatus_Midichloria_mitochondrii_IricVA_GCA_000219355.1
GCA_903878005.1_Candidatus_Midichloriaceae_bacterium
Endosymbiont_of_Acanthamoeba_sp._UWC8_GCA_000730245.1
Candidatus_Jidaibacter_acanthamoeba_UWC36_GCA_000815465.1
Candidatus_Midichloriaceae_endosymbiont_of_Reticulomyxa_filosa
Candidatus_Midichloriaceae_endosymbiont_of_Peranema_trichophorum_GCA_004210275.1
GCA_013288625.1_Candidatus_Midichloriaceae_bacterium
Anaplasma_phagocytophilum_strain_HZ_GCA_000013125.1
Anaplasma_marginale_strain_Florida_GCA_000020305.1
Candidatus_Neoehrlichia_lotoris_strain_RAC413_GCA_000964795.1
Ehrlichia_ruminantium_strain_Welgevonden_GCA_000026005.1
Ehrlichia_chaffeensis_strain_Arkansas_GCA_000013145.1
Wolbachia_endosymbiont_of_Brugia_malayi_GCA_000008385.1
Wolbachia_endosymbiont_of_Drosophila_melanogaster_GCA_000008025.1
Wolbachia_endosymbiont_of_Pratylenchus_penetrans_GCA_001752665.1
Neorickettsia_sennetsu_strain_Miyayama_GCA_000013165.1
Neorickettsia_helminthoeca_strain_Oregon_GCA_000632985.1
Candidatus_Xenolissoclinum_pacificiensis_L6_GCA_000512675.1
Candidatus_Echinorickettsia_raffii_H1_GCA_018101195.1
Holospora_undulata_GCA_000388175_3
Anaplasmataceae_symbiont_of_Acropora_tenuis_Sesoko1_GCA_014132315.1
GCA_903864455.1_Anaplasmataceae_bacterium
Rickettsiaceae_endosymbiont_of_Amblyomma_cajennese_Ac37b_GCA_000746585.2
GCA_903831915.1_Candidatus_Gamibacteraceae_bacterium
GCA_017999675.1_Candidatus_Gamibacteraceae_bacterium
GCA_903872175.1_Candidatus_Gamibacteraceae_bacterium
GCA_018969185.1_Candidatus_Gamibacteraceae_bacterium
GCA_002359655.1_Candidatus_Gamibacteraceae_bacterium
GCA_903927415.1_Candidatus_Gamibacteraceae_bacterium
GCA_903878685.1_Candidatus_Gamibacteraceae_bacterium
GCA_903917585.1_Candidatus_Gamibacteraceae_bacterium
GCA_001768015.1_Candidatus_Gamibacteraceae_bacterium
GCA_016778745.1_Candidatus_Gamibacteraceae_bacterium
GCA_903837365.1_Candidatus_Gamibacteraceae_bacterium
GCA_014190355.1_Candidatus_Gamibacteraceae_bacterium
GCA_009921385.1_Candidatus_Gamibacteraceae_bacterium
GCA_903959715.1_Candidatus_Gamibacteraceae_bacterium
GCA_009925685.1_Candidatus_Gamibacteraceae_bacterium
GCA_009693885.1_Candidatus_Gamibacteraceae_bacterium
GCA_905478245.1_Candidatus_Gamibacteraceae_bacterium
GCA_905478165.1_Candidatus_Gamibacteraceae_bacterium
GCA_018659605.1_Candidatus_Diomedesiaceae_bacterium
GCA_018659145.1_Candidatus_Diomedesiaceae_bacterium
GCA_016778805.1_Candidatus_Diomedesiaceae_bacterium
GCA_016780625.1_Candidatus_Diomedesiaceae_bacterium
GCA_905480015.1_Candidatus_Deianiraeaceae_bacterium
alphaproteobacterium_HIMB5_GCA_000299095.1
Candidatus_Pelagibacter_ubique_HTCC1062_GCA_000012345.1
Candidatus_Pelagibacter_ubique_HIMB058_GCA_000419545.1
alphaproteobacterium_HIMB114_GCA_000163555.2
GCA_013288575.1_Candidatus_Arkhamiaceae_bacterium
GCA_002787615.1_Candidatus_Arkhamiaceae_bacterium
GCA_002422205.1_Candidatus_Mitibacteraceae_bacterium
GCA_002346425.1_Candidatus_Mitibacteraceae_bacterium
GCA_002725445.1_Candidatus_Mitibacteraceae_bacterium
GCA_003241645.1_Candidatus_Mitibacteraceae_bacterium
GCA_002422745.1_Candidatus_Mitibacteraceae_bacterium
GCA_002291925.1_Candidatus_Mitibacteraceae_bacterium
GCA_002291845.1_Candidatus_Mitibacteraceae_bacterium
GCA_004293935.1_Candidatus_Mitibacteraceae_bacterium
GCA_002423155.1_Candidatus_Mitibacteraceae_bacterium
GCA_013288985.1_Candidatus_Mitibacteraceae_bacterium
GCA_013288335.1_Candidatus_Mitibacteraceae_bacterium
GCA_013289445.1_Candidatus_Mitibacteraceae_bacterium
GCA_009926845.1_Candidatus_Mitibacteraceae_bacterium
GCA_005792635.1_Candidatus_Mitibacteraceae_bacterium
GCA_013288305.1_Candidatus_Mitibacteraceae_bacterium
GCA_903821695.1_Candidatus_Jistubacteraceae_bacterium
GCA_010031735.1_Candidatus_Jistubacteraceae_bacterium
GCA_013288565.1_Candidatus_Jistubacteraceae_bacterium
GCA_002787635.1_Candidatus_Athabascaceae_bacterium
GCA_002422795.1_Candidatus_Athabascaceae_bacterium
GCA_003531345.1_Candidatus_Jistubacteraceae_bacterium
Gluconacetobacter_diazotrophicus_GCA_000067045.1
Acetobacter_aceti_GCA_001193495.2
Gluconobacter_oxydans_GCA_000583855.1
Commensalibacter_intestini_GCA_000231445.2
Granulibacter_bethesdensis_GCA_000576145.2
Acidisphaera_rubrifaciens_GCA_000964365.1
Acidiphilium_multivorum_GCA_000202835.1
Rubritepida_flocculans_GCA_000425365.1
Belnapia_moabensis_GCA_000745835.1
Roseomonas_gilardii_GCA_001941945.1
Elioraea_tepidiphila_GCA_000378465.1
Geminicoccus_roseus_GCA_000427665.1
Candidatus_Caedibacter_acanthamoebae_GCA_000743035.1
Caedimonas_varicaedens_GCA_001192655.1
Candidatus_Nucleicultrix_amoebiphila_GCA_002117145.1
Inquilinus_limosus_GCA_000423185.1
Micavibrio_aeruginosavorus_GCA_000226315.1
Rhodospirillum_centenum_GCA_014197805.1
Rhodospirillum_rubrum_GCA_000225955.1
Pararhodospirillum_photometricum_GCA_000284415.2
Caenispirillum_salinarum_GCA_000315795.1
Roseospirillum_parvum_GCA_900100455.1
Magnetospirillum_magneticum_GCA_000009985.1
Terasakiella_pusilla_GCA_000688235.1
Kiloniella_laminariae_GCA_000374005.1
Thalassospira_profundimaris_GCA_000300275.1
alphaproteobacterium_BAL199_GCA_000171835.1
Thalassobaculum_salexigens_GCA_000423805.1
Candidatus_Puniceispirillum_marinum_GCA_000024465.1
Oceanibaculum_indicum_GCA_003633955.1
Rhodovibrio_salinarum_GCA_000515255.1
Tistrella_mobilis_GCA_000264455.2
Sneathiella_glossodoripedis_GCA_000616095.1
Rhizobium_leguminosarum_GCA_011604525.1
Bartonella_quintana_GCA_009936175.1
Chelativorans_oligotrophicus_GCA_011317505.1
Ahrensia_kielensis_GCA_000374465.1
Pelagibacterium_halotolerans_GCA_013004005.1
Roseibium_hamelinense_GCA_007830545.1
Polymorphum_gilvum_GCA_000192745.1
Pseudovibrio_sp._GCA_000236645.1
Thermopetrobacter_sp._GCA_000746275.1
Methylocystis_bryophila_GCA_002117405.1
Methylocella_silvestris_GCA_000021745.1
Methylorubrum_extorquens_GCA_000018845.1
Xanthobacter_autotrophicus_GCA_000017645.1
Rhodopseudomonas_palustris_GCA_013415845.1
Parvibaculum_lavamentivorans_GCA_000017565.1
alphaproteobacterium_IMCC14465_GCA_000293845.2
Candidatus_Micropelagos_thuwalensis_GCA_000469155.1
Rhodomicrobium_vannielii_GCA_000166055.1
Hyphomicrobium_denitrificans_GCA_000143145.1
Oceanicaulis_alexandrii_GCA_000420265.1
Maricaulis_maris_GCA_003634045.1
GCA_017995275.1_Rickettsiaceae_bacterium
Hyphomonas_neptunium_GCA_000013025.1
Hirschia_baltica_GCA_000023785.1
alphaproteobacterium_L41A_GCA_000335735.1
Brevundimonas_subvibrioides_GCA_000144605.1
Asticcacaulis_excentricus_GCA_000175215.2
Phenylobacterium_zucineum_GCA_000017265.1
Caulobacter_vibrioides_GCA_000022005.1
Parvularcula_bermudensis_GCA_000152825.2
Rhodobacter_sphaeroides_GCA_000015985.1
Paracoccus_denitrificans_GCA_004063735.1
Rubellimicrobium_rubrum_GCA_006152115.1
Ketogulonicigenium_vulgare_GCA_001399515.1
Ruegeria_pomeroyi_GCA_000011965.2
Octadecabacter_arcticus_GCA_000155735.2
Sagittula_stellata_GCA_000169415.1
Jannaschia_rubra_GCA_900113265.1
Neomegalonema_perideroedes_GCA_000374145.1
Sphingopyxis_macrogoltabida_GCA_001314325.1
Citromicrobium_sp._GCA_001304795.1
Sphingobium_indicum_GCA_000264945.2
Zymomonas_mobilis_GCA_000007105.1
Sphingomonas_wittichii_GCA_000016765.1
alphaproteobacterium_AAP81b_GCA_001295935.1
Kordiimonas_gwangyangensis_GCA_000375545.1
Magnetofaba_australis_GCA_002109495.1
Magnetococcus_marinus_GCA_000014865.1
Ralstonia_pickettii_GCA_902374465.1
Burkholderia_cepacia_GCA_009586235.1
Methylotenera_versatilis_GCA_000093025.1
Nitrosospira_briensis_GCA_000619905.2
Dasania_marina_GCA_000373485.1
Congregibacter_litoralis_GCA_000153125.2
Enterobacter_cloacae_GCA_000770155.1
Alteromonas_macleodii_GCA_000172635.2
0.1

Purine_z_0.50

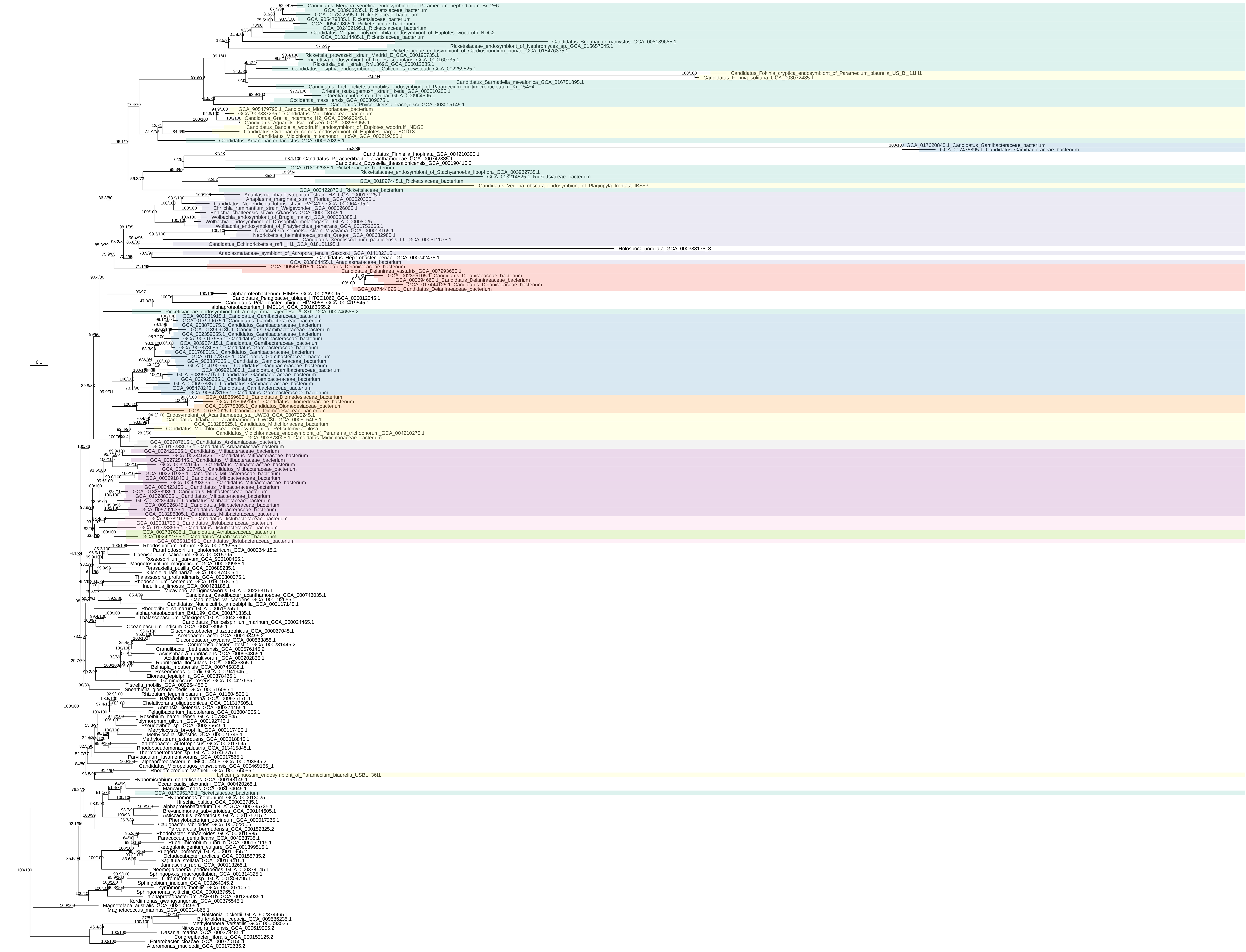

Candidatus_Megaira_venefica_endosymbiont_of_Paramecium_nephridiatum_Sr_2-6
GCA_003963235.1_Rickettsiaceae_bacterium
GCA_017302595.1_Rickettsiaceae_bacterium
GCA_905479885.1_Rickettsiaceae_bacterium
GCA_905479865.1_Rickettsiaceae_bacterium
GCA_002402195.1_Rickettsiaceae_bacterium
Candidatus_Megaira_polyxenophila_endosymbiont_of_Euplotes_woodruffi_NDG2
GCA_013214485.1_Rickettsiaceae_bacterium
Candidatus_Sneabacter_namystus_GCA_008189685.1
Rickettsiaceae_endosymbiont_of_Nephromyces_sp._GCA_015657545.1
Rickettsiaceae_endosymbiont_of_Cardiosporidium_cionae_GCA_015476335.1
Rickettsia_prowazekii_strain_Madrid_E_GCA_000195735.1
Rickettsia_endosymbiont_of_Ixodes_scapularis_GCA_000160735.1
Rickettsia_bellii_strain_RML369C_GCA_000012385.1
Candidatus_Tisiphia_endosymbiont_of_Culicoides_newsteadi_GCA_002259525.1
Candidatus_Fokinia_cryptica_endosymbiont_of_Paramecium_biaurelia_US_Bl_11III1
Candidatus_Fokinia_solitaria_GCA_003072485.1
Candidatus_Sarmatiella_mevalonica_GCA_016751895.1
Candidatus_Trichorickettsia_mobilis_endosymbiont_of_Paramecium_multimicronucleatum_Kr_154-4
Orientia_tsutsugamushi_strain_Ikeda_GCA_000010205.1
Orientia_chuto_strain_Dubai_GCA_000964595.1
Occidentia_massiliensis_GCA_000309075.1
Candidatus_Phycorickettsia_trachydisci_GCA_003015145.1
GCA_905479795.1_Candidatus_Midichloriaceae_bacterium
GCA_903887235.1_Candidatus_Midichloriaceae_bacterium
Candidatus_Grellia_incantans_H2_GCA_009690945.1
Candidatus_Aquarickettsia_rohweri_GCA_003953955.1
Candidatus_Bandiella_woodruffii_endosymbiont_of_Euplotes_woodruffi_NDG2
Candidatus_Cyrtobacter_comes_endosymbiont_of_Euplotes_harpa_BOD18
Candidatus_Midichloria_mitochondrii_IricVA_GCA_000219355.1
Candidatus_Arcanobacter_lacustris_GCA_000970895.1
GCA_017620845.1_Candidatus_Gamibacteraceae_bacterium
GCA_017475895.1_Candidatus_Gamibacteraceae_bacterium
Candidatus_Finniella_inopinata_GCA_004210305.1
Candidatus_Paracaedibacter_acanthamoebae_GCA_000742835.1
Candidatus_Odyssella_thessalonicensis_GCA_000190415.2
GCA_018062985.1_Rickettsiaceae_bacterium
Rickettsiaceae_endosymbiont_of_Stachyamoeba_lipophora_GCA_003932735.1
GCA_013214525.1_Rickettsiaceae_bacterium
GCA_001897445.1_Rickettsiaceae_bacterium
Candidatus_Vederia_obscura_endosymbiont_of_Plagiopyla_frontata_IBS-3
GCA_002422875.1_Rickettsiaceae_bacterium
Anaplasma_phagocytophilum_strain_HZ_GCA_000013125.1
Anaplasma_marginale_strain_Florida_GCA_000020305.1
Candidatus_Neoehrlichia_lotoris_strain_RAC413_GCA_000964795.1
Ehrlichia_ruminantium_strain_Welgevonden_GCA_000026005.1
Ehrlichia_chaffeensis_strain_Arkansas_GCA_000013145.1
Wolbachia_endosymbiont_of_Brugia_malayi_GCA_000008385.1
Wolbachia_endosymbiont_of_Drosophila_melanogaster_GCA_000008025.1
Wolbachia_endosymbiont_of_Pratylenchus_penetrans_GCA_001752665.1
Neorickettsia_sennetsu_strain_Miyayama_GCA_000013165.1
Neorickettsia_helminthoeca_strain_Oregon_GCA_000632985.1
Candidatus_Xenolissoclinum_pacificiensis_L6_GCA_000512675.1
Candidatus_Echinorickettsia_raffii_H1_GCA_018101195.1
Holospora_undulata_GCA_000388175_3
Anaplasmataceae_symbiont_of_Acropora_tenuis_Sesoko1_GCA_014132315.1
Candidatus_Hepatobacter_penaei_GCA_000742475.1
GCA_903864455.1_Anaplasmataceae_bacterium
GCA_905480015.1_Candidatus_Deianiraeaceae_bacterium
Candidatus_Deianiraea_vastatrix_GCA_007993655.1
GCA_002395105.1_Candidatus_Deianiraeaceae_bacterium
GCA_002394665.1_Candidatus_Deianiraeaceae_bacterium
GCA_017444125.1_Candidatus_Deianiraeaceae_bacterium
GCA_017444095.1_Candidatus_Deianiraeaceae_bacterium
alphaproteobacterium_HIMB5_GCA_000299095.1
Candidatus_Pelagibacter_ubique_HTCC1062_GCA_000012345.1
Candidatus_Pelagibacter_ubique_HIMB058_GCA_000419545.1
alphaproteobacterium_HIMB114_GCA_000163555.2
Rickettsiaceae_endosymbiont_of_Amblyomma_cajennese_Ac37b_GCA_000746585.2
GCA_903831915.1_Candidatus_Gamibacteraceae_bacterium
GCA_017999675.1_Candidatus_Gamibacteraceae_bacterium
GCA_903872175.1_Candidatus_Gamibacteraceae_bacterium
GCA_018969185.1_Candidatus_Gamibacteraceae_bacterium
GCA_002359655.1_Candidatus_Gamibacteraceae_bacterium
GCA_903917585.1_Candidatus_Gamibacteraceae_bacterium
GCA_903927415.1_Candidatus_Gamibacteraceae_bacterium
GCA_903878685.1_Candidatus_Gamibacteraceae_bacterium
GCA_001768015.1_Candidatus_Gamibacteraceae_bacterium
GCA_016778745.1_Candidatus_Gamibacteraceae_bacterium
GCA_903837365.1_Candidatus_Gamibacteraceae_bacterium
GCA_014190155.1_Candidatus_Gamibacteraceae_bacterium
GCA_009921385.1_Candidatus_Gamibacteraceae_bacterium
GCA_903959715.1_Candidatus_Gamibacteraceae_bacterium
GCA_009925685.1_Candidatus_Gamibacteraceae_bacterium
GCA_009693885.1_Candidatus_Gamibacteraceae_bacterium
GCA_905478245.1_Candidatus_Gamibacteraceae_bacterium
GCA_905478165.1_Candidatus_Gamibacteraceae_bacterium
GCA_018659605.1_Candidatus_Diomedesiaceae_bacterium
GCA_018659145.1_Candidatus_Diomedesiaceae_bacterium
GCA_016778805.1_Candidatus_Diomedesiaceae_bacterium
GCA_016780625.1_Candidatus_Diomedesiaceae_bacterium
Endosymbiont_of_Acanthamoeba_sp._UWC8_GCA_000730245.1
Candidatus_Jidaibacter_acanthamoeba_UWC36_GCA_000815465.1
GCA_013288625.1_Candidatus_Midichloriaceae_bacterium
Candidatus_Midichloriaceae_endosymbiont_of_Reticulomyxa_filosa
Candidatus_Midichloriaceae_endosymbiont_of_Peranema_trichophorum_GCA_004210275.1
GCA_903878005.1_Candidatus_Midichloriaceae_bacterium
GCA_002787615.1_Candidatus_Arkhamiaceae_bacterium
GCA_013288575.1_Candidatus_Arkhamiaceae_bacterium
GCA_002422205.1_Candidatus_Mitibacteraceae_bacterium
GCA_002346425.1_Candidatus_Mitibacteraceae_bacterium
GCA_002725445.1_Candidatus_Mitibacteraceae_bacterium
GCA_003241645.1_Candidatus_Mitibacteraceae_bacterium
GCA_002422745.1_Candidatus_Mitibacteraceae_bacterium
GCA_002291925.1_Candidatus_Mitibacteraceae_bacterium
GCA_002291845.1_Candidatus_Mitibacteraceae_bacterium
GCA_004293935.1_Candidatus_Mitibacteraceae_bacterium
GCA_002423155.1_Candidatus_Mitibacteraceae_bacterium
GCA_013288985.1_Candidatus_Mitibacteraceae_bacterium
GCA_013288335.1_Candidatus_Mitibacteraceae_bacterium
GCA_013289445.1_Candidatus_Mitibacteraceae_bacterium
GCA_009926845.1_Candidatus_Mitibacteraceae_bacterium
GCA_005792635.1_Candidatus_Mitibacteraceae_bacterium
GCA_013288305.1_Candidatus_Mitibacteraceae_bacterium
GCA_903821695.1_Candidatus_Jistubacteraceae_bacterium
GCA_010031735.1_Candidatus_Jistubacteraceae_bacterium
GCA_013288565.1_Candidatus_Jistubacteraceae_bacterium
GCA_002787635.1_Candidatus_Athabascaceae_bacterium
GCA_002422795.1_Candidatus_Athabascaceae_bacterium
GCA_003531345.1_Candidatus_Jistubacteraceae_bacterium
Rhodospirillum_rubrum_GCA_000225955.1
Pararhodospirillum_photometricum_GCA_000284415.2
Caenispirillum_salinarum_GCA_000315795.1
Roseospirillum_parvum_GCA_900100455.1
Magnetospirillum_magneticum_GCA_000009985.1
Terasakiella_pusilla_GCA_000688235.1
Kiloniella_laminariae_GCA_000374005.1
Thalassospira_profundimaris_GCA_000300275.1
Rhodospirillum_centenum_GCA_014197805.1
Inquilinus_limosus_GCA_000423185.1
Micavibrio_aeruginosavorus_GCA_000226315.1
Candidatus_Caedibacter_acanthamoebae_GCA_000743035.1
Caedimonas_varicaedens_GCA_001192655.1
Candidatus_Nucleicultrix_amoebiphila_GCA_002117145.1
Rhodovibrio_salinarum_GCA_000515255.1
alphaproteobacterium_BAL199_GCA_000171835.1
Thalassobaculum_salexigens_GCA_000423805.1
Candidatus_Puniceispirillum_marinum_GCA_000024465.1
Oceanibaculum_indicum_GCA_003633955.1
Gluconacetobacter_diazotrophicus_GCA_000067045.1
Acetobacter_aceti_GCA_000193495.2
Gluconobacter_oxydans_GCA_000583855.1
Commensalibacter_intestini_GCA_000231445.2
Granulibacter_bethesdensis_GCA_000576145.2
Acidisphaera_rubrifaciens_GCA_000964365.1
Acidiphilium_multivorum_GCA_000202835.1
Rubritepida_flocculans_GCA_000425365.1
Belnapia_moabensis_GCA_000745835.1
Roseomonas_gilardii_GCA_001941945.1
Elioraea_tepidiphila_GCA_000378465.1
Geminicoccus_roseus_GCA_000427665.1
Tistrella_mobilis_GCA_000264455.2
Sneathiella_glossodoripedis_GCA_000616095.1
Rhizobium_leguminosarum_GCA_011604525.1
Bartonella_quintana_GCA_009936175.1
Chelativorans_oligotrophicus_GCA_011317505.1
Ahrensia_kielensis_GCA_000374465.1
Pelagibacterium_halotolerans_GCA_013004005.1
Roseibium_hamelinense_GCA_007830545.1
Polymorphum_gilvum_GCA_000192745.1
Pseudovibrio_sp._GCA_000236645.1
Methylocystis_bryophila_GCA_002117405.1
Methylocella_silvestris_GCA_000021745.1
Methylorubrum_extorquens_GCA_000018845.1
Xanthobacter_autotrophicus_GCA_000017645.1
Rhodopseudomonas_palustris_GCA_013415845.1
Thermopetrobacter_sp._GCA_000746275.1
Parvibaculum_lavamentivorans_GCA_000017565.1
alphaproteobacterium_IMCC14465_GCA_000293845.2
Candidatus_Micropelagos_thuwalensis_GCA_000469155_1
Rhodomicrobium_vannielii_GCA_000166055.1
Lyticum_sinuosum_endosymbiont_of_Paramecium_biaurelia_USBL-36I1
Hyphomicrobium_denitrificans_GCA_000143145.1
Oceanicaulis_alexandrii_GCA_000420265.1
Maricaulis_maris_GCA_003634045.1
GCA_017995275.1_Rickettsiaceae_bacterium
Hyphomonas_neptunium_GCA_000013025.1
Hirschia_baltica_GCA_000023785.1
alphaproteobacterium_L41A_GCA_000335735.1
Brevundimonas_subvibrioides_GCA_000144605.1
Asticcacaulis_excentricus_GCA_000175215.2
Phenylobacterium_zucineum_GCA_000017265.1
Caulobacter_vibrioides_GCA_000022005.1
Parvularcula_bermudensis_GCA_000152825.2
Rhodobacter_sphaeroides_GCA_000015985.1
Paracoccus_denitrificans_GCA_004063735.1
Rubellimicrobium_rubrum_GCA_006152115.1
Ketogulonicigenium_vulgare_GCA_001399515.1
Ruegeria_pomeroyi_GCA_000011965.2
Octadecabacter_arcticus_GCA_000155735.2
Sagittula_stellata_GCA_000169415.1
Jannaschia_rubra_GCA_900113265.1
Neomegalonema_perideroedes_GCA_000374145.1
Sphingopyxis_macrogoltabida_GCA_001314325.1
Citromicrobium_sp._GCA_001304795.1
Sphingobium_indicum_GCA_000264945.2
Zymomonas_mobilis_GCA_000007105.1
Sphingomonas_wittichii_GCA_000016765.1
alphaproteobacterium_AAP81b_GCA_001295935.1
Kordiimonas_gwangyangensis_GCA_000375545.1
Magnetofaba_australis_GCA_002109495.1
Magnetococcus_marinus_GCA_000014865.1
Ralstonia_pickettii_GCA_902374465.1
Burkholderia_cepacia_GCA_009586235.1
Methylotenera_versatilis_GCA_000093025.1
Nitrosospira_briensis_GCA_000619905.2
Dasania_marina_GCA_000373485.1
Congregibacter_litoralis_GCA_000153125.2
Enterobacter_cloacae_GCA_000770155.1
Alteromonas_macleodii_GCA_000172635.2
0.1

Pirimidine

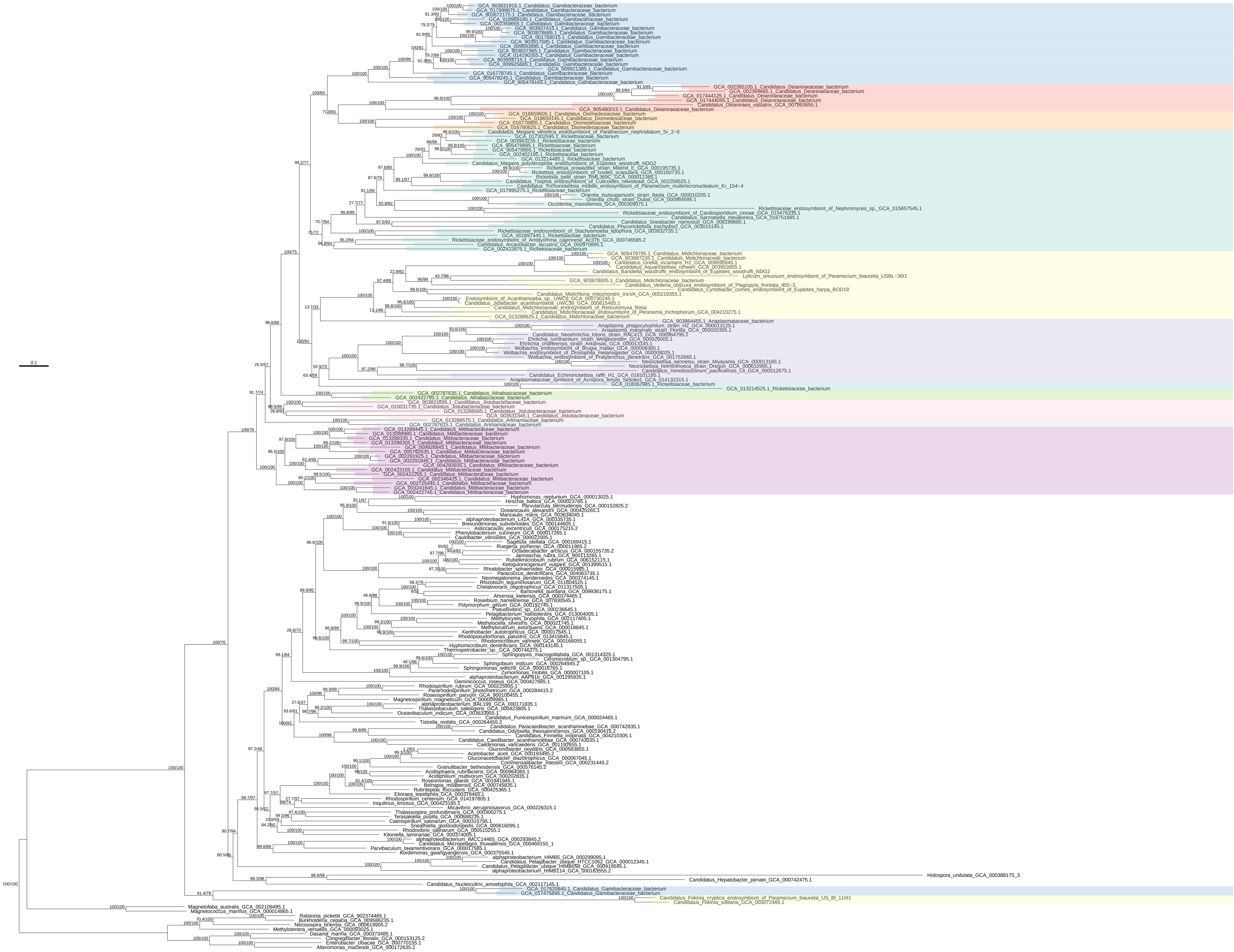
0.1
GCA_903831915.1_Candidatus_Gamibacteraceae_bacterium
GCA_017999675.1_Candidatus_Gamibacteraceae_bacterium
GCA_903872175.1_Candidatus_Gamibacteraceae_bacterium
GCA_018969185.1_Candidatus_Gamibacteraceae_bacterium
GCA_002359655.1_Candidatus_Gamibacteraceae_bacterium
GCA_903927415.1_Candidatus_Gamibacteraceae_bacterium
GCA_903878685.1_Candidatus_Gamibacteraceae_bacterium
GCA_001768015.1_Candidatus_Gamibacteraceae_bacterium
GCA_903917585.1_Candidatus_Gamibacteraceae_bacterium
GCA_009693885.1_Candidatus_Gamibacteraceae_bacterium
GCA_903837365.1_Candidatus_Gamibacteraceae_bacterium
GCA_014190355.1_Candidatus_Gamibacteraceae_bacterium
GCA_903959715.1_Candidatus_Gamibacteraceae_bacterium
GCA_009925685.1_Candidatus_Gamibacteraceae_bacterium
GCA_009921385.1_Candidatus_Gamibacteraceae_bacterium
GCA_016778745.1_Candidatus_Gamibacteraceae_bacterium
GCA_905478245.1_Candidatus_Gamibacteraceae_bacterium
GCA_905478165.1_Candidatus_Gamibacteraceae_bacterium
GCA_002395105.1_Candidatus_Deianiraeaceae_bacterium
GCA_002394665.1_Candidatus_Deianiraeaceae_bacterium
GCA_017444125.1_Candidatus_Deianiraeaceae_bacterium
GCA_017444095.1_Candidatus_Deianiraeaceae_bacterium
Candidatus_Deianiraea_vastatrix_GCA_007993655.1
GCA_905480015.1_Candidatus_Deianiraeaceae_bacterium
GCA_018659605.1_Candidatus_Diomedesiaceae_bacterium
GCA_018659145.1_Candidatus_Diomedesiaceae_bacterium
GCA_016778805.1_Candidatus_Diomedesiaceae_bacterium
GCA_016780625.1_Candidatus_Diomedesiaceae_bacterium
Candidatus_Megaira_venefica_endosymbiont_of_Paramecium_nephridiatum_Sr_2-6
GCA_017302595.1_Rickettsiaceae_bacterium
GCA_003963235.1_Rickettsiaceae_bacterium
GCA_905479885.1_Rickettsiaceae_bacterium
GCA_905479865.1_Rickettsiaceae_bacterium
GCA_002402195.1_Rickettsiaceae_bacterium
GCA_013214485.1_Rickettsiaceae_bacterium
Candidatus_Megaira_polyxenophila_endosymbiont_of_Euplotes_woodruffi_NDG2
Rickettsia_prowazekii_strain_Madrid_E_GCA_000195735.1
Rickettsia_endosymbiont_of_Ixodes_scapularis_GCA_000160735.1
Rickettsia_bellii_strain_RML369C_GCA_000012385.1
Candidatus_Tisiphia_endosymbiont_of_Culicoides_newsteadi_GCA_002259525.1
Candidatus_Trichorickettsia_mobilis_endosymbiont_of_Paramecium_multimicronucleatum_Kr_154-4
GCA_017995275.1_Rickettsiaceae_bacterium
Orientia_tsutsugamushi_strain_Ikeda_GCA_000010205.1
Orientia_chuto_strain_Dubai_GCA_000964595.1
Occidentia_massiliensis_GCA_000309075.1
Rickettsiaceae_endosymbiont_of_Nephromyces_sp._GCA_015657545.1
Rickettsiaceae_endosymbiont_of_Cardiosporidium_cionae_GCA_015476335.1
Candidatus_Sarmatiella_mevalonica_GCA_016751895.1
Candidatus_Sneabacter_namystus_GCA_008189685.1
Candidatus_Phycorickettsia_trachydisci_GCA_003015145.1
Rickettsiaceae_endosymbiont_of_Stachyamoeba_lipophora_GCA_003932735.1
GCA_001897445.1_Rickettsiaceae_bacterium
Rickettsiaceae_endosymbiont_of_Amblyomma_cajennense_Ac37b_GCA_000746585.2
Candidatus_Arcanobacter_lacustris_GCA_000970895.1
GCA_002422875.1_Rickettsiaceae_bacterium
GCA_905479795.1_Candidatus_Midichloriaceae_bacterium
GCA_903887235.1_Candidatus_Midichloriaceae_bacterium
Candidatus_Grellia_incantans_H2_GCA_009690945.1
Candidatus_Aquarickettsia_rohweri_GCA_003953955.1
Candidatus_Bandiella_woodruffii_endosymbiont_of_Euplotes_woodruffi_NDG2
Lyticum_sinuosum_endosymbiont_of_Paramecium_biaurelia_USBL-36I1
GCA_903878005.1_Candidatus_Midichloriaceae_bacterium
Candidatus_Vederia_obscura_endosymbiont_of_Plagiopyla_frontata_IBS-3
Candidatus_Cyrtobacter_comes_endosymbiont_of_Euplotes_harpa_BOD18
Candidatus_Midichloria_mitochondrii_IricVA_GCA_000219355.1
Endosymbiont_of_Acanthamoeba_sp._UWC8_GCA_000730245.1
Candidatus_Jidaibacter_acanthamoeba_UWC36_GCA_000815465.1
Candidatus_Midichloriaceae_endosymbiont_of_Reticulomyxa_filosa
Candidatus_Midichloriaceae_endosymbiont_of_Peranema_trichophorum_GCA_004210275.1
GCA_013288625.1_Candidatus_Midichloriaceae_bacterium
GCA_903864455.1_Anaplasmataceae_bacterium
Anaplasma_phagocytophilum_strain_HZ_GCA_000013125.1
Anaplasma_marginale_strain_Florida_GCA_000020305.1
Candidatus_Neoehrlichia_lotoris_strain_RAC413_GCA_000964795.1
Ehrlichia_ruminantium_strain_Welgevonden_GCA_000026005.1
Ehrlichia_chaffeensis_strain_Arkansas_GCA_000013145.1
Wolbachia_endosymbiont_of_Brugia_malayi_GCA_000008385.1
Wolbachia_endosymbiont_of_Drosophila_melanogaster_GCA_000008025.1
Wolbachia_endosymbiont_of_Pratylenchus_penetrans_GCA_001752665.1
Neorickettsia_sennetsu_strain_Miyayama_GCA_000013165.1
Neorickettsia_helminthoeca_strain_Oregon_GCA_000632985.1
Candidatus_Xenolissoclinum_pacificiensis_L6_GCA_000512675.1
Candidatus_Echinorickettsia_raffii_H1_GCA_018101195.1
Anaplasmataceae_symbiont_of_Acropora_tenuis_Sesoko1_GCA_014132315.1
GCA_018062985.1_Rickettsiaceae_bacterium
GCA_013214525.1_Rickettsiaceae_bacterium
GCA_002787635.1_Candidatus_Athabascaceae_bacterium
GCA_002422795.1_Candidatus_Athabascaceae_bacterium
GCA_903821695.1_Candidatus_Jistubacteraceae_bacterium
GCA_010031735.1_Candidatus_Jistubacteraceae_bacterium
GCA_013288565.1_Candidatus_Jistubacteraceae_bacterium
GCA_003531345.1_Candidatus_Jistubacteraceae_bacterium
GCA_013288575.1_Candidatus_Arkhamiaceae_bacterium
GCA_002787615.1_Candidatus_Arkhamiaceae_bacterium
GCA_013289445.1_Candidatus_Mitibacteraceae_bacterium
GCA_013288985.1_Candidatus_Mitibacteraceae_bacterium
GCA_013288335.1_Candidatus_Mitibacteraceae_bacterium
GCA_013288305.1_Candidatus_Mitibacteraceae_bacterium
GCA_009926845.1_Candidatus_Mitibacteraceae_bacterium
GCA_005792635.1_Candidatus_Mitibacteraceae_bacterium
GCA_002291925.1_Candidatus_Mitibacteraceae_bacterium
GCA_002291845.1_Candidatus_Mitibacteraceae_bacterium
GCA_004293935.1_Candidatus_Mitibacteraceae_bacterium
GCA_002423155.1_Candidatus_Mitibacteraceae_bacterium
GCA_002422205.1_Candidatus_Mitibacteraceae_bacterium
GCA_002346425.1_Candidatus_Mitibacteraceae_bacterium
GCA_002725445.1_Candidatus_Mitibacteraceae_bacterium
GCA_003241645.1_Candidatus_Mitibacteraceae_bacterium
GCA_002422745.1_Candidatus_Mitibacteraceae_bacterium
Hyphomonas_neptunium_GCA_000013025.1
Hirschia_baltica_GCA_000023785.1
Parvularcula_bermudensis_GCA_000152825.2
Oceanicaulis_alexandrii_GCA_000420265.1
Maricaulis_maris_GCA_003634045.1
alphaproteobacterium_L41A_GCA_000335735.1
Brevundimonas_subvibrioides_GCA_000144605.1
Asticcacaulis_excentricus_GCA_000175215.2
Phenylobacterium_zucineum_GCA_000017265.1
Caulobacter_vibrioides_GCA_000022005.1
Sagittula_stellata_GCA_000169415.1
Ruegeria_pomeroyi_GCA_000011965.2
Octadecabacter_arcticus_GCA_000155735.2
Jannaschia_rubra_GCA_900113265.1
Rubellimicrobium_rubrum_GCA_006152115.1
Ketogulonicigenium_vulgare_GCA_001399515.1
Rhodobacter_sphaeroides_GCA_000015985.1
Paracoccus_denitrificans_GCA_004063735.1
Neomegalonema_perideroedes_GCA_000374145.1
Rhizobium_leguminosarum_GCA_011604525.1
Chelativorans_oligotrophicus_GCA_011317505.1
Bartonella_quintana_GCA_009936175.1
Ahrensia_kielensis_GCA_000374465.1
Roseibium_hamelinense_GCA_007830545.1
Polymorphum_gilvum_GCA_000192745.1
Pseudovibrio_sp._GCA_000236645.1
Pelagibacterium_halotolerans_GCA_013004005.1
Methylocystis_bryophila_GCA_002117405.1
Methylocella_silvestris_GCA_000021745.1
Methylorubrum_extorquens_GCA_000018845.1
Xanthobacter_autotrophicus_GCA_000017645.1
Rhodopseudomonas_palustris_GCA_013415845.1
Rhodomicrobium_vannielii_GCA_000166055.1
Hyphomicrobium_denitrificans_GCA_000143145.1
Thermopetrobacter_sp._GCA_000746275.1
Sphingopyxis_macrogoltabida_GCA_001314325.1
Citromicrobium_sp._GCA_001304795.1
Sphingobium_indicum_GCA_000264945.2
Sphingomonas_wittichii_GCA_000016765.1
Zymomonas_mobilis_GCA_000007105.1
alphaproteobacterium_AAP81b_GCA_001295935.1
Geminicoccus_roseus_GCA_000427665.1
Rhodospirillum_rubrum_GCA_000225955.1
Pararhodospirillum_photometricum_GCA_000284415.2
Roseospirillum_parvum_GCA_900100455.1
Magnetospirillum_magneticum_GCA_000009985.1
alphaproteobacterium_BAL199_GCA_000171835.1
Thalassobaculum_salexigens_GCA_000423805.1
Oceanibaculum_indicum_GCA_003633955.1
Candidatus_Puniceispirillum_marinum_GCA_000024465.1
Tistrella_mobilis_GCA_000264455.2
Candidatus_Paracaedibacter_acanthamoebae_GCA_000742835.1
Candidatus_Odyssella_thessalonicensis_GCA_000190415.2
Candidatus_Finniella_inopinata_GCA_004210305.1
Candidatus_Caedibacter_acanthamoebae_GCA_000743035.1
Caedimonas_varicaedens_GCA_001192655.1
Gluconobacter_oxydans_GCA_000583855.1
Acetobacter_aceti_GCA_000193495.2
Gluconacetobacter_diazotrophicus_GCA_000067045.1
Commensalibacter_intestini_GCA_000231445.2
Granulibacter_bethesdensis_GCA_000576145.2
Acidisphaera_rubrifaciens_GCA_000964365.1
Acidiphilium_multivorum_GCA_000202835.1
Roseomonas_gilardii_GCA_001941945.1
Belnapia_moabensis_GCA_000745835.1
Rubritepida_flocculans_GCA_000425365.1
Elioraea_tepidiphila_GCA_000378465.1
Rhodospirillum_centenum_GCA_014197805.1
Inquilinus_limosus_GCA_000423185.1
Micavibrio_aeruginosavorus_GCA_000226315.1
Thalassospira_profundimaris_GCA_000300275.1
Terasakiella_pusilla_GCA_000688235.1
Caenispirillum_salinarum_GCA_000315795.1
Sneathiella_glossodoripedis_GCA_000616095.1
Rhodovibrio_salinarum_GCA_000515255.1
Kiloniella_laminariae_GCA_000374005.1
alphaproteobacterium_IMCC14465_GCA_000293845.2
Candidatus_Micropelagos_thuwalensis_GCA_000469155.1
Parvibaculum_lavamentivorans_GCA_000017565.1
Kordiimonas_gwangyangensis_GCA_000375545.1
alphaproteobacterium_HIMB5_GCA_000299095.1
Candidatus_Pelagibacter_ubique_HTCC1062_GCA_000012345.1
Candidatus_Pelagibacter_ubique_HIMB058_GCA_000419545.1
alphaproteobacterium_HIMB114_GCA_000163555.2
Holospora_undulata_GCA_000388175_3
Candidatus_Hepatobacter_penaei_GCA_000742475.1
Candidatus_Nucleicultrix_amoebiphila_GCA_002117145.1
GCA_017620845.1_Candidatus_Gamibacteraceae_bacterium
GCA_017475895.1_Candidatus_Gamibacteraceae_bacterium
Candidatus_Fokinia_cryptica_endosymbiont_of_Paramecium_biaurelia_US_Bl_11III1
Candidatus_Fokinia_solitaria_GCA_003072485.1
Magnetofaba_australis_GCA_002109495.1
Magnetococcus_marinus_GCA_000014865.1
Ralstonia_pickettii_GCA_902374465.1
Burkholderia_cepacia_GCA_009586235.1
Nitrosospira_briensis_GCA_000619905.2
Methylotenera_versatilis_GCA_000093025.1
Dasania_marina_GCA_000373485.1
Congregibacter_litoralis_GCA_000153125.2
Enterobacter_cloacae_GCA_000770155.1
Alteromonas_macleodii_GCA_000172635.2

Pirimidine_z_0.10

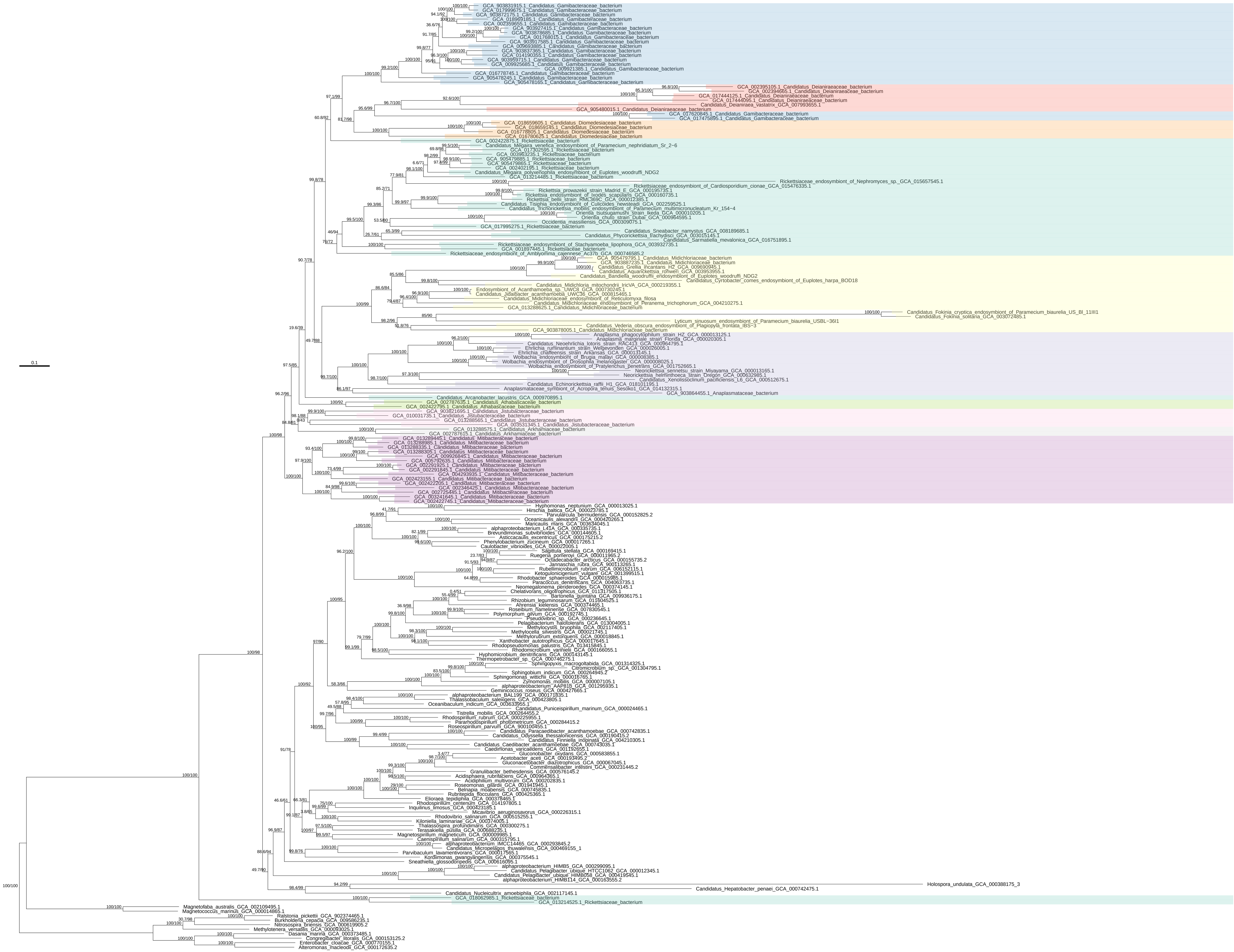

Pirimidine_z_0.20

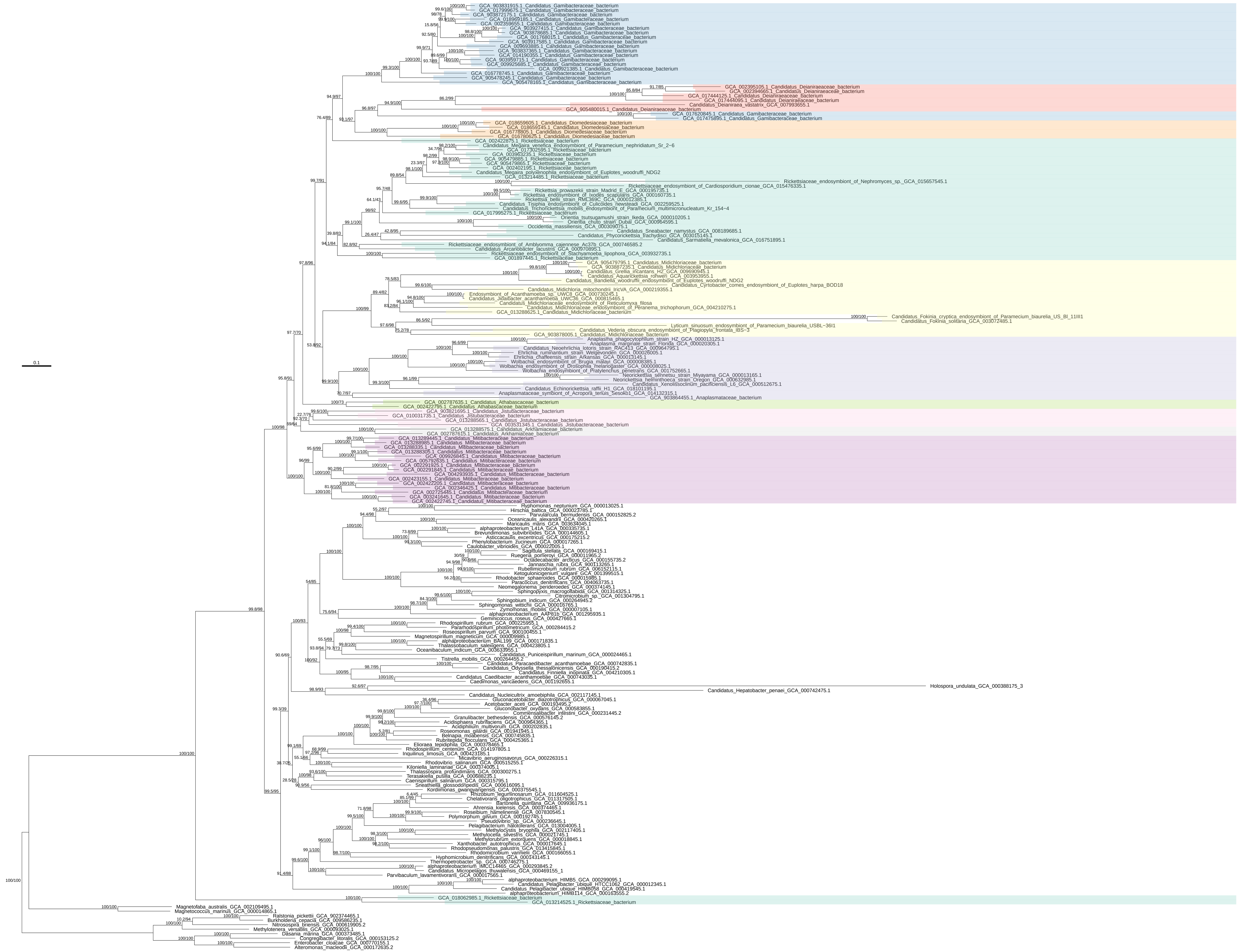

Pirimidine_z_0.30

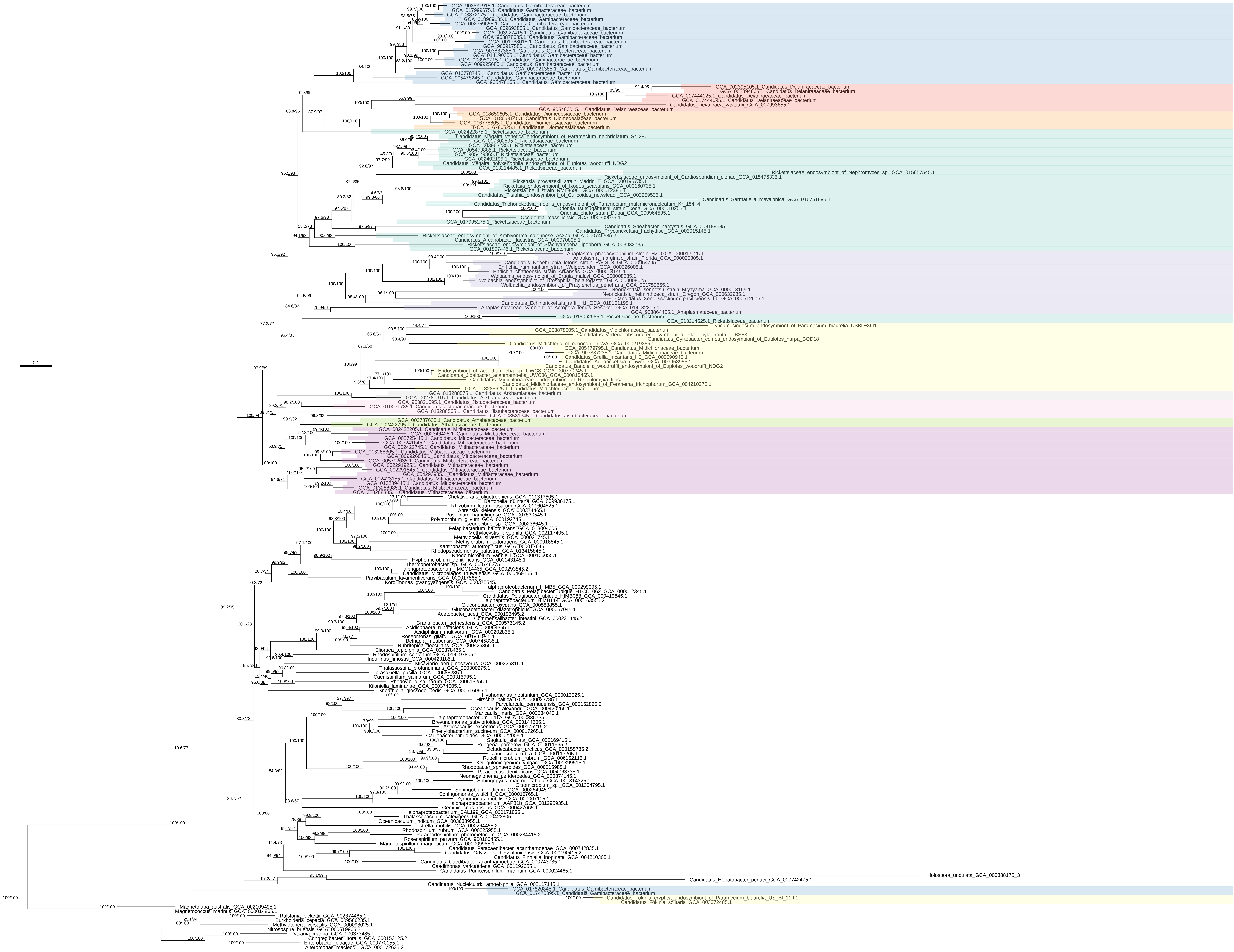

Pirimidine_z_0.40

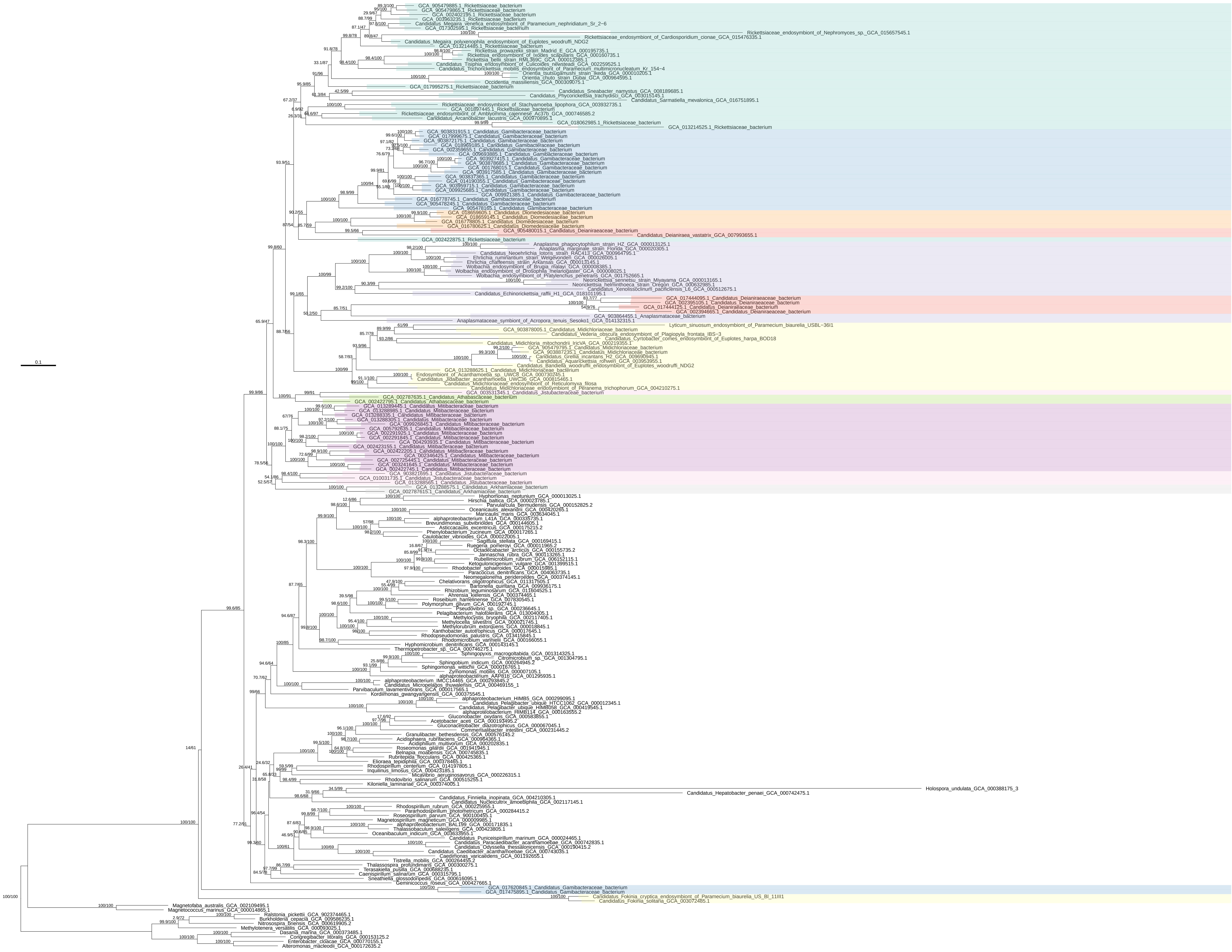

Pirimidine_z_0.50

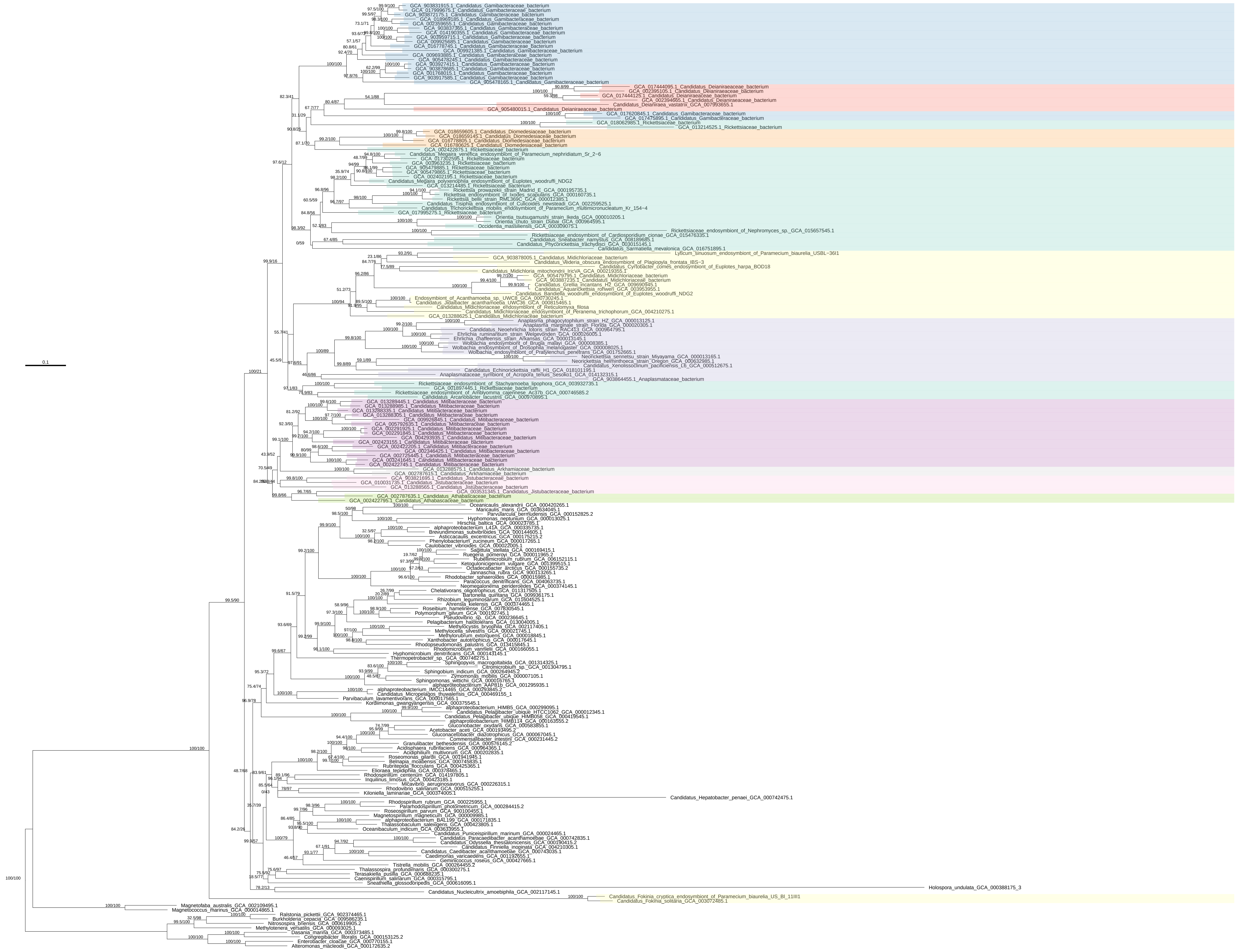

Arg

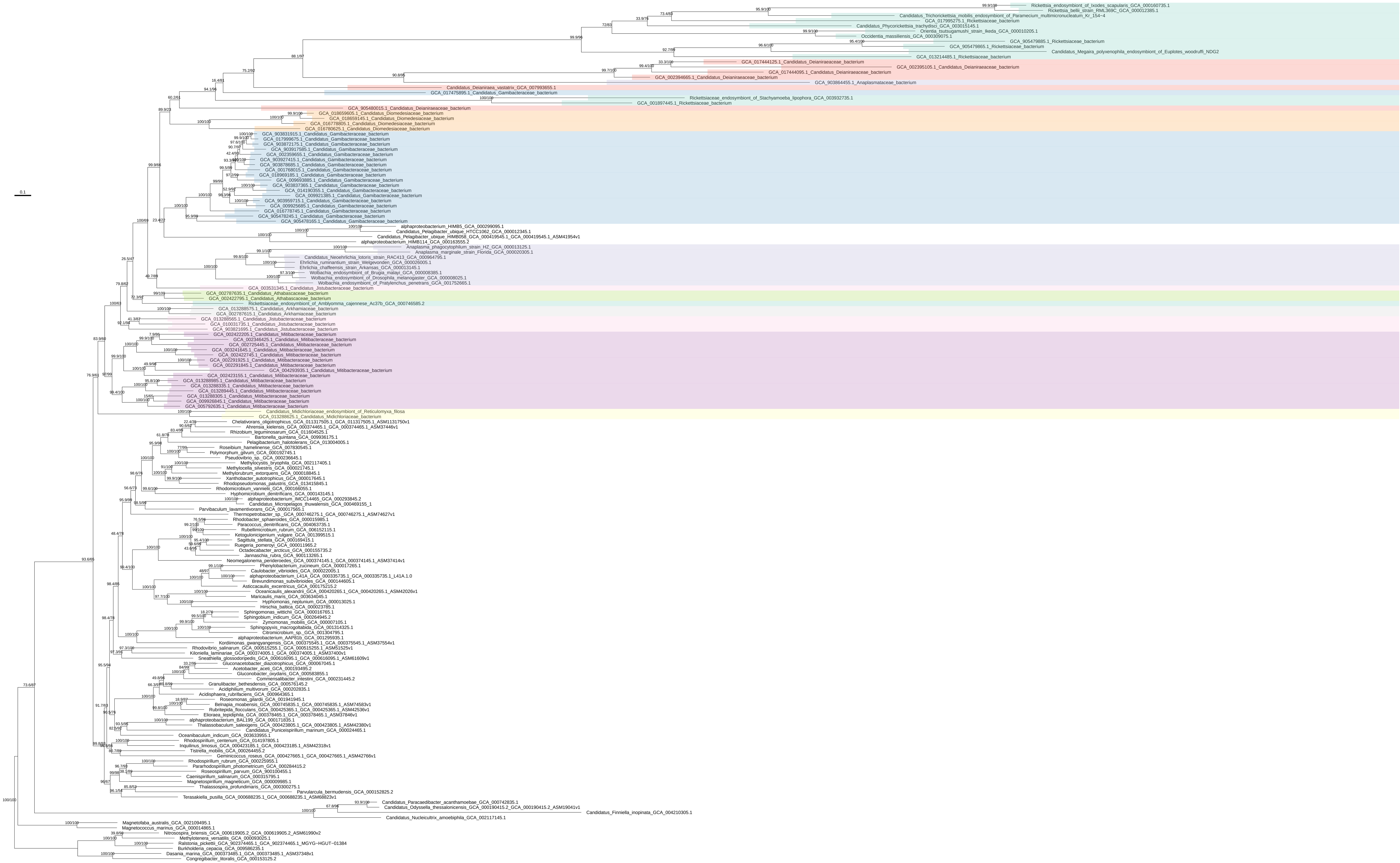

0.1
Rickettsia_endosymbiont_of_Ixodes_scapularis_GCA_000160735.1
Rickettsia_bellii_strain_RML369C_GCA_000012385.1
Candidatus_Trichorickettsia_mobilis_endosymbiont_of_Paramecium_multimicronucleatum_Kr_154-4
GCA_017995275.1_Rickettsiaceae_bacterium
Candidatus_Phycorickettsia_trachydisci_GCA_003015145.1
Orientia_tsutsugamushi_strain_Ikeda_GCA_000010205.1
Occidentia_massiliensis_GCA_000309075.1
GCA_905479885.1_Rickettsiaceae_bacterium
GCA_905479865.1_Rickettsiaceae_bacterium
Candidatus_Megaira_polyxenophila_endosymbiont_of_Euplotes_woodruffi_NDG2
GCA_013214485.1_Rickettsiaceae_bacterium
GCA_017444125.1_Candidatus_Deianiraeaceae_bacterium
GCA_002395105.1_Candidatus_Deianiraeaceae_bacterium
GCA_017444095.1_Candidatus_Deianiraeaceae_bacterium
GCA_002394665.1_Candidatus_Deianiraeaceae_bacterium
GCA_903864455.1_Anaplasmataceae_bacterium
Candidatus_Deianiraea_vastatrix_GCA_007993655.1
GCA_017475895.1_Candidatus_Gamibacteraceae_bacterium
Rickettsiaceae_endosymbiont_of_Stachyamoeba_lipophora_GCA_003932735.1
GCA_001897445.1_Rickettsiaceae_bacterium
GCA_905480015.1_Candidatus_Deianiraeaceae_bacterium
GCA_018659605.1_Candidatus_Diomedesiaceae_bacterium
GCA_018659145.1_Candidatus_Diomedesiaceae_bacterium
GCA_016778805.1_Candidatus_Diomedesiaceae_bacterium
GCA_016780625.1_Candidatus_Diomedesiaceae_bacterium
GCA_903831915.1_Candidatus_Gamibacteraceae_bacterium
GCA_017999675.1_Candidatus_Gamibacteraceae_bacterium
GCA_903872175.1_Candidatus_Gamibacteraceae_bacterium
GCA_903917585.1_Candidatus_Gamibacteraceae_bacterium
GCA_002359655.1_Candidatus_Gamibacteraceae_bacterium
GCA_903927415.1_Candidatus_Gamibacteraceae_bacterium
GCA_903878685.1_Candidatus_Gamibacteraceae_bacterium
GCA_001768015.1_Candidatus_Gamibacteraceae_bacterium
GCA_018969185.1_Candidatus_Gamibacteraceae_bacterium
GCA_009693885.1_Candidatus_Gamibacteraceae_bacterium
GCA_903837365.1_Candidatus_Gamibacteraceae_bacterium
GCA_014190355.1_Candidatus_Gamibacteraceae_bacterium
GCA_009921385.1_Candidatus_Gamibacteraceae_bacterium
GCA_903959715.1_Candidatus_Gamibacteraceae_bacterium
GCA_009925685.1_Candidatus_Gamibacteraceae_bacterium
GCA_016778745.1_Candidatus_Gamibacteraceae_bacterium
GCA_905478245.1_Candidatus_Gamibacteraceae_bacterium
GCA_905478165.1_Candidatus_Gamibacteraceae_bacterium
alphaproteobacterium_HIMB5_GCA_000299095.1
Candidatus_Pelagibacter_ubique_HTCC1062_GCA_000012345.1
Candidatus_Pelagibacter_ubique_HIMB058_GCA_000419545.1_GCA_000419545.1_ASM41954v1
alphaproteobacterium_HIMB114_GCA_000163555.2
Anaplasma_phagocytophilum_strain_HZ_GCA_000013125.1
Anaplasma_marginale_strain_Florida_GCA_000020305.1
Candidatus_Neoehrlichia_lotoris_strain_RAC413_GCA_000964795.1
Ehrlichia_ruminantium_strain_Welgevonden_GCA_000026005.1
Ehrlichia_chaffeensis_strain_Arkansas_GCA_000013145.1
Wolbachia_endosymbiont_of_Brugia_malayi_GCA_000008385.1
Wolbachia_endosymbiont_of_Drosophila_melanogaster_GCA_000008025.1
Wolbachia_endosymbiont_of_Pratylenchus_penetrans_GCA_001752665.1
GCA_003531345.1_Candidatus_Jistubacteraceae_bacterium
GCA_002787635.1_Candidatus_Athabascaceae_bacterium
GCA_002422795.1_Candidatus_Athabascaceae_bacterium
Rickettsiaceae_endosymbiont_of_Amblyomma_cajennese_Ac37b_GCA_000746585.2
GCA_013288575.1_Candidatus_Arkhamiaceae_bacterium
GCA_002787615.1_Candidatus_Arkhamiaceae_bacterium
GCA_013288565.1_Candidatus_Jistubacteraceae_bacterium
GCA_010031735.1_Candidatus_Jistubacteraceae_bacterium
GCA_903821695.1_Candidatus_Jistubacteraceae_bacterium
GCA_002422205.1_Candidatus_Mitibacteraceae_bacterium
GCA_002346425.1_Candidatus_Mitibacteraceae_bacterium
GCA_002725445.1_Candidatus_Mitibacteraceae_bacterium
GCA_003241645.1_Candidatus_Mitibacteraceae_bacterium
GCA_002422745.1_Candidatus_Mitibacteraceae_bacterium
GCA_002291925.1_Candidatus_Mitibacteraceae_bacterium
GCA_002291845.1_Candidatus_Mitibacteraceae_bacterium
GCA_004293935.1_Candidatus_Mitibacteraceae_bacterium
GCA_002423155.1_Candidatus_Mitibacteraceae_bacterium
GCA_013288985.1_Candidatus_Mitibacteraceae_bacterium
GCA_013288335.1_Candidatus_Mitibacteraceae_bacterium
GCA_013289445.1_Candidatus_Mitibacteraceae_bacterium
GCA_013288305.1_Candidatus_Mitibacteraceae_bacterium
GCA_009926845.1_Candidatus_Mitibacteraceae_bacterium
GCA_005792635.1_Candidatus_Mitibacteraceae_bacterium
Candidatus_Midichloriaceae_endosymbiont_of_Reticulomyxa_filosa
GCA_013288625.1_Candidatus_Midichloriaceae_bacterium
Chelativorans_oligotrophicus_GCA_011317505.1_GCA_011317505.1_ASM1131750v1
Ahrensia_kielensis_GCA_000374465.1_GCA_000374465.1_ASM37446v1
Rhizobium_leguminosarum_GCA_011604525.1
Bartonella_quintana_GCA_009936175.1
Pelagibacterium_halotolerans_GCA_013004005.1
Roseibium_hamelinense_GCA_007830545.1
Polymorphum_gilvum_GCA_000192745.1
Pseudovibrio_sp._GCA_000236645.1
Methylocystis_bryophila_GCA_002117405.1
Methylocella_silvestris_GCA_000021745.1
Methylorubrum_extorquens_GCA_000018845.1
Xanthobacter_autotrophicus_GCA_000017645.1
Rhodopseudomonas_palustris_GCA_013415845.1
Rhodomicrobium_vannielii_GCA_000166055.1
Hyphomicrobium_denitrificans_GCA_000143145.1
alphaproteobacterium_IMCC14465_GCA_000293845.2
Candidatus_Micropelagos_thuwalensis_GCA_000469155_1
Parvibaculum_lavamentivorans_GCA_000017565.1
Thermopetrobacter_sp._GCA_000746275.1_GCA_000746275.1_ASM74627v1
Rhodobacter_sphaeroides_GCA_000015985.1
Paracoccus_denitrificans_GCA_004063735.1
Rubellimicrobium_rubrum_GCA_006152115.1
Ketogulonicigenium_vulgare_GCA_001399515.1
Sagittula_stellata_GCA_000169415.1
Ruegeria_pomeroyi_GCA_000011965.2
Octadecabacter_arcticus_GCA_000155735.2
Jannaschia_rubra_GCA_900113265.1
Neomegalonema_perideroedes_GCA_000374145.1_GCA_000374145.1_ASM37414v1
Phenylobacterium_zucineum_GCA_000017265.1
Caulobacter_vibrioides_GCA_000022005.1
alphaproteobacterium_L41A_GCA_000335735.1_GCA_000335735.1_L41A.1.0
Brevundimonas_subvibrioides_GCA_000144605.1
Asticcacaulis_excentricus_GCA_000175215.2
Oceanicaulis_alexandrii_GCA_000420265.1_GCA_000420265.1_ASM42026v1
Maricaulis_maris_GCA_003634045.1
Hyphomonas_neptunium_GCA_000013025.1
Hirschia_baltica_GCA_000023785.1
Sphingomonas_wittichii_GCA_000016765.1
Sphingobium_indicum_GCA_000264945.2
Zymomonas_mobilis_GCA_000007105.1
Sphingopyxis_macrogoltabida_GCA_001314325.1
Citromicrobium_sp._GCA_001304795.1
alphaproteobacterium_AAP81b_GCA_001295935.1
Kordiimonas_gwangyangensis_GCA_000375545.1_GCA_000375545.1_ASM37554v1
Rhodovibrio_salinarum_GCA_000515255.1_GCA_000515255.1_ASM51525v1
Kiloniella_laminariae_GCA_000374005.1_GCA_000374005.1_ASM37400v1
Sneathiella_glossodoripedis_GCA_000616095.1_GCA_000616095.1_ASM61609v1
Gluconacetobacter_diazotrophicus_GCA_000067045.1
Acetobacter_aceti_GCA_000193495.2
Gluconobacter_oxydans_GCA_000583855.1
Commensalibacter_intestini_GCA_000231445.2
Granulibacter_bethesdensis_GCA_000576145.2
Acidiphilium_multivorum_GCA_000202835.1
Acidisphaera_rubrifaciens_GCA_000964365.1
Roseomonas_gilardii_GCA_001941945.1
Belnapia_moabensis_GCA_000745835.1_GCA_000745835.1_ASM74583v1
Rubritepida_flocculans_GCA_000425365.1_GCA_000425365.1_ASM42536v1
Elioraea_tepidiphila_GCA_000378465.1_GCA_000378465.1_ASM37846v1
alphaproteobacterium_BAL199_GCA_000171835.1
Thalassobaculum_salexigens_GCA_000423805.1_GCA_000423805.1_ASM42380v1
Candidatus_Puniceispirillum_marinum_GCA_000024465.1
Oceanibaculum_indicum_GCA_003633955.1
Rhodospirillum_centenum_GCA_014197805.1
Inquilinus_limosus_GCA_000423185.1_GCA_000423185.1_ASM42318v1
Tistrella_mobilis_GCA_000264455.2
Geminicoccus_roseus_GCA_000427665.1_GCA_000427665.1_ASM42766v1
Rhodospirillum_rubrum_GCA_000225955.1
Pararhodospirillum_photometricum_GCA_000284415.2
Roseospirillum_parvum_GCA_900100455.1
Caenispirillum_salinarum_GCA_000315795.1
Magnetospirillum_magneticum_GCA_000009985.1
Thalassospira_profundimaris_GCA_000300275.1
Parvularcula_bermudensis_GCA_000152825.2
Terasakiella_pusilla_GCA_000688235.1_GCA_000688235.1_ASM68823v1
Candidatus_Paracaedibacter_acanthamoebae_GCA_000742835.1
Candidatus_Odyssella_thessalonicensis_GCA_000190415.2_GCA_000190415.2_ASM19041v1
Candidatus_Finniella_inopinata_GCA_004210305.1
Candidatus_Nucleicultrix_amoebiphila_GCA_002117145.1
Magnetofaba_australis_GCA_002109495.1
Magnetococcus_marinus_GCA_000014865.1
Nitrosospira_briensis_GCA_000619905.2_GCA_000619905.2_ASM61990v2
Methylotenera_versatilis_GCA_000093025.1
Ralstonia_pickettii_GCA_902374465.1_GCA_902374465.1_MGYG-HGUT-01384
Burkholderia_cepacia_GCA_009586235.1
Dasania_marina_GCA_000373485.1_GCA_000373485.1_ASM37348v1
Congregibacter_litoralis_GCA_000153125.2

Arg_z_0.10

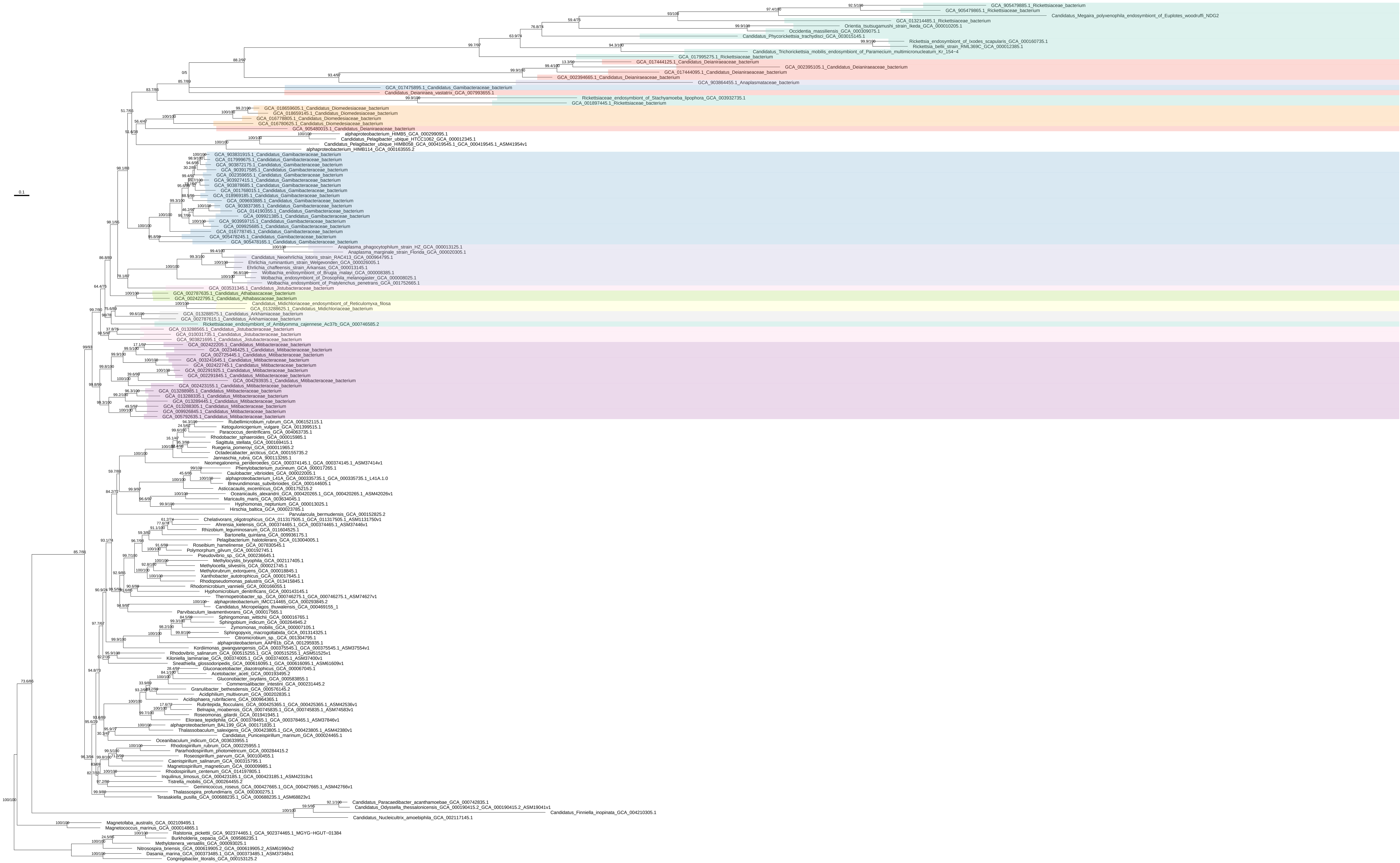
0.1
GCA_905479885.1_Rickettsiaceae_bacterium
GCA_905479865.1_Rickettsiaceae_bacterium
Candidatus_Megaira_polyxenophila_endosymbiont_of_Euplotes_woodruffi_NDG2
GCA_013214485.1_Rickettsiaceae_bacterium
Orientia_tsutsugamushi_strain_Ikeda_GCA_000010205.1
Occidentia_massiliensis_GCA_000309075.1
Candidatus_Phycorickettsia_trachydisci_GCA_003015145.1
Rickettsia_endosymbiont_of_Ixodes_scapularis_GCA_000160735.1
Rickettsia_bellii_strain_RML369C_GCA_000012385.1
Candidatus_Trichorickettsia_mobilis_endosymbiont_of_Paramecium_multimicronucleatum_Kr_154-4
GCA_017995275.1_Rickettsiaceae_bacterium
GCA_017444125.1_Candidatus_Deianiraeaceae_bacterium
GCA_002395105.1_Candidatus_Deianiraeaceae_bacterium
GCA_017444095.1_Candidatus_Deianiraeaceae_bacterium
GCA_002394665.1_Candidatus_Deianiraeaceae_bacterium
GCA_903864455.1_Anaplasmataceae_bacterium
GCA_017475895.1_Candidatus_Gamibacteraceae_bacterium
Candidatus_Deianiraea_vastatrix_GCA_007993655.1
Rickettsiaceae_endosymbiont_of_Stachyamoeba_lipophora_GCA_003932735.1
GCA_001897445.1_Rickettsiaceae_bacterium
GCA_018659605.1_Candidatus_Diomedesiaceae_bacterium
GCA_018659145.1_Candidatus_Diomedesiaceae_bacterium
GCA_016778805.1_Candidatus_Diomedesiaceae_bacterium
GCA_016780625.1_Candidatus_Diomedesiaceae_bacterium
GCA_905480015.1_Candidatus_Deianiraeaceae_bacterium
alphaproteobacterium_HIMB5_GCA_000299095.1
Candidatus_Pelagibacter_ubique_HTCC1062_GCA_000012345.1
Candidatus_Pelagibacter_ubique_HIMB058_GCA_000419545.1_GCA_000419545.1_ASM41954v1
alphaproteobacterium_HIMB114_GCA_000163555.2
GCA_903831915.1_Candidatus_Gamibacteraceae_bacterium
GCA_017999675.1_Candidatus_Gamibacteraceae_bacterium
GCA_903872175.1_Candidatus_Gamibacteraceae_bacterium
GCA_903917585.1_Candidatus_Gamibacteraceae_bacterium
GCA_002359655.1_Candidatus_Gamibacteraceae_bacterium
GCA_903927415.1_Candidatus_Gamibacteraceae_bacterium
GCA_903878685.1_Candidatus_Gamibacteraceae_bacterium
GCA_001768015.1_Candidatus_Gamibacteraceae_bacterium
GCA_018969185.1_Candidatus_Gamibacteraceae_bacterium
GCA_009693885.1_Candidatus_Gamibacteraceae_bacterium
GCA_903837365.1_Candidatus_Gamibacteraceae_bacterium
GCA_014190355.1_Candidatus_Gamibacteraceae_bacterium
GCA_009921385.1_Candidatus_Gamibacteraceae_bacterium
GCA_903959715.1_Candidatus_Gamibacteraceae_bacterium
GCA_009925685.1_Candidatus_Gamibacteraceae_bacterium
GCA_016778745.1_Candidatus_Gamibacteraceae_bacterium
GCA_905478245.1_Candidatus_Gamibacteraceae_bacterium
GCA_905478165.1_Candidatus_Gamibacteraceae_bacterium
Anaplasma_phagocytophilum_strain_HZ_GCA_000013125.1
Anaplasma_marginale_strain_Florida_GCA_000020305.1
Candidatus_Neoehrlichia_lotoris_strain_RAC413_GCA_000964795.1
Ehrlichia_ruminantium_strain_Welgevonden_GCA_000026005.1
Ehrlichia_chaffeensis_strain_Arkansas_GCA_000013145.1
Wolbachia_endosymbiont_of_Brugia_malayi_GCA_000008385.1
Wolbachia_endosymbiont_of_Drosophila_melanogaster_GCA_000008025.1
Wolbachia_endosymbiont_of_Pratylenchus_penetrans_GCA_001752665.1
GCA_003531345.1_Candidatus_Jistubacteraceae_bacterium
GCA_002787635.1_Candidatus_Athabascaceae_bacterium
GCA_002422795.1_Candidatus_Athabascaceae_bacterium
Candidatus_Midichloriaceae_endosymbiont_of_Reticulomyxa_filosa
GCA_013288625.1_Candidatus_Midichloriaceae_bacterium
GCA_013288575.1_Candidatus_Arkhamiaceae_bacterium
GCA_002787615.1_Candidatus_Arkhamiaceae_bacterium
Rickettsiaceae_endosymbiont_of_Amblyomma_cajennese_Ac37b_GCA_000746585.2
GCA_013288565.1_Candidatus_Jistubacteraceae_bacterium
GCA_010031735.1_Candidatus_Jistubacteraceae_bacterium
GCA_903821695.1_Candidatus_Jistubacteraceae_bacterium
GCA_002422205.1_Candidatus_Mitibacteraceae_bacterium
GCA_002346425.1_Candidatus_Mitibacteraceae_bacterium
GCA_002725445.1_Candidatus_Mitibacteraceae_bacterium
GCA_003241645.1_Candidatus_Mitibacteraceae_bacterium
GCA_002422745.1_Candidatus_Mitibacteraceae_bacterium
GCA_002291925.1_Candidatus_Mitibacteraceae_bacterium
GCA_002291845.1_Candidatus_Mitibacteraceae_bacterium
GCA_004293935.1_Candidatus_Mitibacteraceae_bacterium
GCA_002423155.1_Candidatus_Mitibacteraceae_bacterium
GCA_013288985.1_Candidatus_Mitibacteraceae_bacterium
GCA_013288335.1_Candidatus_Mitibacteraceae_bacterium
GCA_013289445.1_Candidatus_Mitibacteraceae_bacterium
GCA_013288305.1_Candidatus_Mitibacteraceae_bacterium
GCA_009926845.1_Candidatus_Mitibacteraceae_bacterium
GCA_005792635.1_Candidatus_Mitibacteraceae_bacterium
Rubellimicrobium_rubrum_GCA_006152115.1
Ketogulonicigenium_vulgare_GCA_001399515.1
Paracoccus_denitrificans_GCA_004063735.1
Rhodobacter_sphaeroides_GCA_000015985.1
Sagittula_stellata_GCA_000169415.1
Ruegeria_pomeroyi_GCA_000011965.2
Octadecabacter_arcticus_GCA_000155735.2
Jannaschia_rubra_GCA_900113265.1
Neomegalonema_perideroedes_GCA_000374145.1_GCA_000374145.1_ASM37414v1
Phenylobacterium_zucineum_GCA_000017265.1
Caulobacter_vibrioides_GCA_000022005.1
alphaproteobacterium_L41A_GCA_000335735.1_GCA_000335735.1_L41A.1.0
Brevundimonas_subvibrioides_GCA_000144605.1
Asticcacaulis_excentricus_GCA_000175215.2
Oceanicaulis_alexandrii_GCA_000420265.1_GCA_000420265.1_ASM42026v1
Maricaulis_maris_GCA_003634045.1
Hyphomonas_neptunium_GCA_000013025.1
Hirschia_baltica_GCA_000023785.1
Parvularcula_bermudensis_GCA_000152825.2
Chelativorans_oligotrophicus_GCA_011317505.1_GCA_011317505.1_ASM1131750v1
Ahrensia_kielensis_GCA_000374465.1_GCA_000374465.1_ASM37446v1
Rhizobium_leguminosarum_GCA_011604525.1
Bartonella_quintana_GCA_009936175.1
Pelagibacterium_halotolerans_GCA_013004005.1
Roseibium_hamelinense_GCA_007830545.1
Polymorphum_gilvum_GCA_000192745.1
Pseudovibrio_sp._GCA_000236645.1
Methylocystis_bryophila_GCA_002117405.1
Methylocella_silvestris_GCA_000021745.1
Methylorubrum_extorquens_GCA_000018845.1
Xanthobacter_autotrophicus_GCA_000017645.1
Rhodopseudomonas_palustris_GCA_013415845.1
Rhodomicrobium_vannielii_GCA_000166055.1
Hyphomicrobium_denitrificans_GCA_000143145.1
Thermopetrobacter_sp._GCA_000746275.1_GCA_000746275.1_ASM74627v1
alphaproteobacterium_IMCC14465_GCA_000293845.2
Candidatus_Micropelagos_thuwalensis_GCA_000469155_1
Parvibaculum_lavamentivorans_GCA_000017565.1
Sphingomonas_wittichii_GCA_000016765.1
Sphingobium_indicum_GCA_000264945.2
Zymomonas_mobilis_GCA_000007105.1
Sphingopyxis_macrogoltabida_GCA_001314325.1
Citromicrobium_sp._GCA_001304795.1
alphaproteobacterium_AAP81b_GCA_001295935.1
Kordiimonas_gwangyangensis_GCA_000375545.1_GCA_000375545.1_ASM37554v1
Rhodovibrio_salinarum_GCA_000515255.1_GCA_000515255.1_ASM51525v1
Kiloniella_laminariae_GCA_000374005.1_GCA_000374005.1_ASM37400v1
Sneathiella_glossodoripedis_GCA_000616095.1_GCA_000616095.1_ASM61609v1
Gluconacetobacter_diazotrophicus_GCA_000067045.1
Acetobacter_aceti_GCA_001193495.2
Gluconobacter_oxydans_GCA_000583855.1
Commensalibacter_intestini_GCA_000231445.2
Granulibacter_bethesdensis_GCA_000576145.2
Acidiphilium_multivorum_GCA_000202835.1
Acidisphaera_rubrifaciens_GCA_000964365.1
Rubritepida_flocculans_GCA_000425365.1_GCA_000425365.1_ASM42536v1
Belnapia_moabensis_GCA_000745835.1_GCA_000745835.1_ASM74583v1
Roseomonas_gilardii_GCA_001941945.1
Elioraea_tepidiphila_GCA_000378465.1_GCA_000378465.1_ASM37846v1
alphaproteobacterium_BAL199_GCA_000171835.1
Thalassobaculum_salexigens_GCA_000423805.1_GCA_000423805.1_ASM42380v1
Candidatus_Puniceispirillum_marinum_GCA_000024465.1
Oceanibaculum_indicum_GCA_003633955.1
Rhodospirillum_rubrum_GCA_000225955.1
Pararhodospirillum_photometricum_GCA_000284415.2
Roseospirillum_parvum_GCA_900100455.1
Caenispirillum_salinarum_GCA_000315795.1
Magnetospirillum_magneticum_GCA_000009985.1
Rhodospirillum_centenum_GCA_014197805.1
Inquilinus_limosus_GCA_000423185.1_GCA_000423185.1_ASM42318v1
Tistrella_mobilis_GCA_000264455.2
Geminicoccus_roseus_GCA_000427665.1_GCA_000427665.1_ASM42766v1
Thalassospira_profundimaris_GCA_000300275.1
Terasakiella_pusilla_GCA_000688235.1_GCA_000688235.1_ASM68823v1
Candidatus_Paracaedibacter_acanthamoebae_GCA_000742835.1
Candidatus_Odyssella_thessalonicensis_GCA_000190415.2_GCA_000190415.2_ASM19041v1
Candidatus_Finniella_inopinata_GCA_004210305.1
Candidatus_Nucleicultrix_amoebiphila_GCA_002117145.1
Magnetofaba_australis_GCA_002109495.1
Magnetococcus_marinus_GCA_000014865.1
Ralstonia_pickettii_GCA_902374465.1_GCA_902374465.1_MGYG-HGUT-01384
Burkholderia_cepacia_GCA_009586235.1
Methylotenera_versatilis_GCA_000093025.1
Nitrosospira_briensis_GCA_000619905.2_GCA_000619905.2_ASM61990v2
Dasania_marina_GCA_000373485.1_GCA_000373485.1_ASM37348v1
Congregibacter_litoralis_GCA_000153125.2

Arg_z_0.20

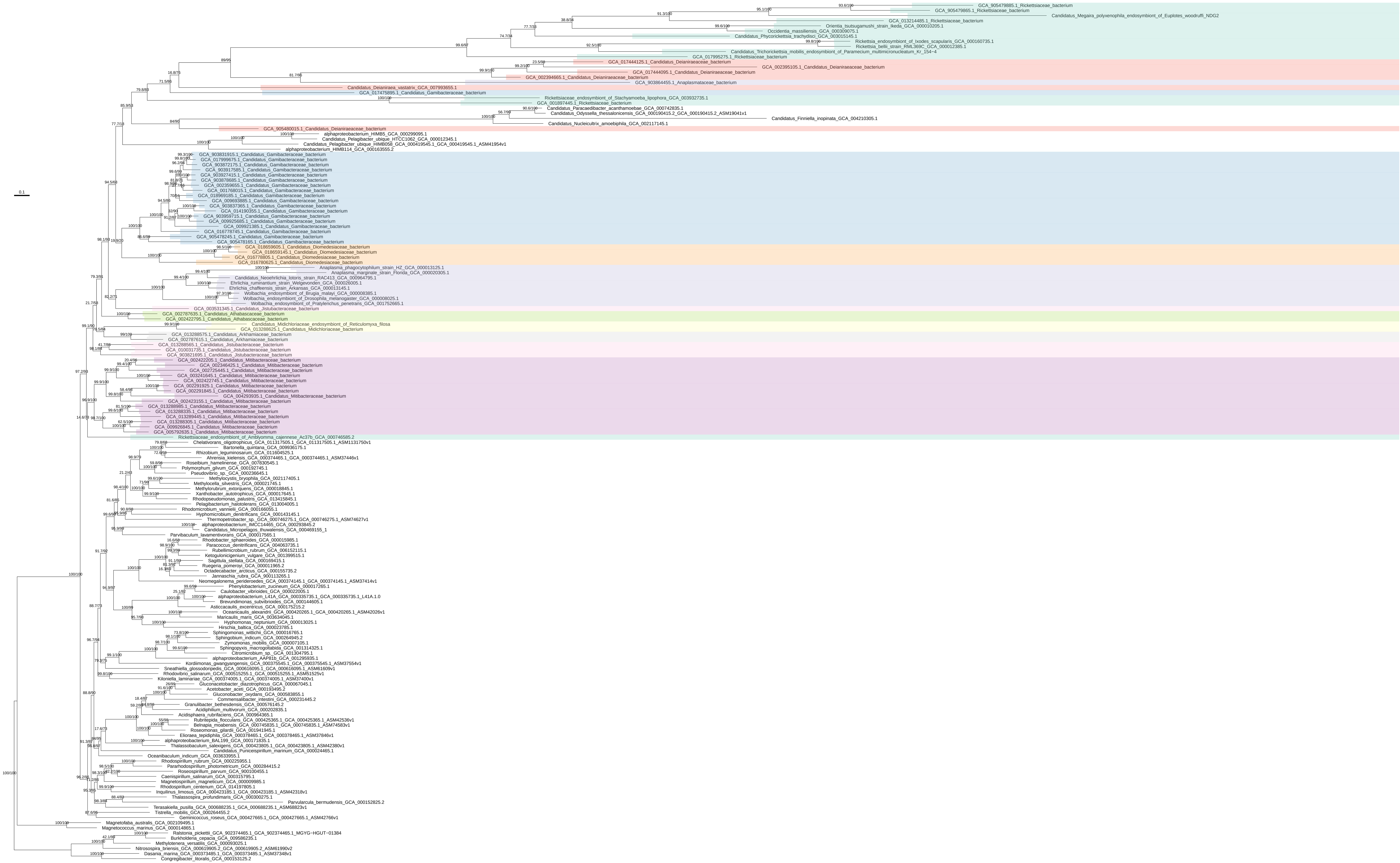

0.1
GCA_905479885.1_Rickettsiaceae_bacterium
GCA_905479865.1_Rickettsiaceae_bacterium
Candidatus_Megaira_polyxenophila_endosymbiont_of_Euplotes_woodruffi_NDG2
GCA_013214485.1_Rickettsiaceae_bacterium
Orientia_tsutsugamushi_strain_Ikeda_GCA_000010205.1
Occidentia_massiliensis_GCA_000309075.1
Candidatus_Phycorickettsia_trachydisci_GCA_003015145.1
Rickettsia_endosymbiont_of_Ixodes_scapularis_GCA_000160735.1
Rickettsia_bellii_strain_RML369C_GCA_000012385.1
Candidatus_Trichorickettsia_mobilis_endosymbiont_of_Paramecium_multimicronucleatum_Kr_154-4
GCA_017995275.1_Rickettsiaceae_bacterium
GCA_017444125.1_Candidatus_Deianiraeaceae_bacterium
GCA_002395105.1_Candidatus_Deianiraeaceae_bacterium
GCA_017444095.1_Candidatus_Deianiraeaceae_bacterium
GCA_002394665.1_Candidatus_Deianiraeaceae_bacterium
GCA_903864455.1_Anaplasmataceae_bacterium
Candidatus_Deianiraea_vastatrix_GCA_007993655.1
GCA_017475895.1_Candidatus_Gamibacteraceae_bacterium
Rickettsiaceae_endosymbiont_of_Stachyamoeba_lipophora_GCA_003932735.1
GCA_001897445.1_Rickettsiaceae_bacterium
Candidatus_Paracaedibacter_acanthamoebae_GCA_000742835.1
Candidatus_Odyssella_thessalonicensis_GCA_000190415.2_GCA_000190415.2_ASM19041v1
Candidatus_Finniella_inopinata_GCA_004210305.1
Candidatus_Nucleicultrix_amoebiphila_GCA_002117145.1
GCA_905480015.1_Candidatus_Deianiraeaceae_bacterium
alphaproteobacterium_HIMB5_GCA_000299095.1
Candidatus_Pelagibacter_ubique_HTCC1062_GCA_000012345.1
Candidatus_Pelagibacter_ubique_HIMB058_GCA_000419545.1_GCA_000419545.1_ASM41954v1
alphaproteobacterium_HIMB114_GCA_000163555.2
GCA_903831915.1_Candidatus_Gamibacteraceae_bacterium
GCA_017999675.1_Candidatus_Gamibacteraceae_bacterium
GCA_903872175.1_Candidatus_Gamibacteraceae_bacterium
GCA_903917585.1_Candidatus_Gamibacteraceae_bacterium
GCA_903927415.1_Candidatus_Gamibacteraceae_bacterium
GCA_903878685.1_Candidatus_Gamibacteraceae_bacterium
GCA_002359655.1_Candidatus_Gamibacteraceae_bacterium
GCA_001768015.1_Candidatus_Gamibacteraceae_bacterium
GCA_018969185.1_Candidatus_Gamibacteraceae_bacterium
GCA_009693885.1_Candidatus_Gamibacteraceae_bacterium
GCA_903837365.1_Candidatus_Gamibacteraceae_bacterium
GCA_014190355.1_Candidatus_Gamibacteraceae_bacterium
GCA_903959715.1_Candidatus_Gamibacteraceae_bacterium
GCA_009925685.1_Candidatus_Gamibacteraceae_bacterium
GCA_009921385.1_Candidatus_Gamibacteraceae_bacterium
GCA_016778745.1_Candidatus_Gamibacteraceae_bacterium
GCA_905478245.1_Candidatus_Gamibacteraceae_bacterium
GCA_905478165.1_Candidatus_Gamibacteraceae_bacterium
GCA_018659605.1_Candidatus_Diomedesiaceae_bacterium
GCA_018659145.1_Candidatus_Diomedesiaceae_bacterium
GCA_016778805.1_Candidatus_Diomedesiaceae_bacterium
GCA_016780625.1_Candidatus_Diomedesiaceae_bacterium
Anaplasma_phagocytophilum_strain_HZ_GCA_000013125.1
Anaplasma_marginale_strain_Florida_GCA_000020305.1
Candidatus_Neoehrlichia_lotoris_strain_RAC413_GCA_000964795.1
Ehrlichia_ruminantium_strain_Welgevonden_GCA_000026005.1
Ehrlichia_chaffeensis_strain_Arkansas_GCA_000013145.1
Wolbachia_endosymbiont_of_Brugia_malayi_GCA_000008385.1
Wolbachia_endosymbiont_of_Drosophila_melanogaster_GCA_000008025.1
Wolbachia_endosymbiont_of_Pratylenchus_penetrans_GCA_001752665.1
GCA_003531345.1_Candidatus_Jistubacteraceae_bacterium
GCA_002787635.1_Candidatus_Athabascaceae_bacterium
GCA_002422795.1_Candidatus_Athabascaceae_bacterium
Candidatus_Midichloriaceae_endosymbiont_of_Reticulomyxa_filosa
GCA_013288625.1_Candidatus_Midichloriaceae_bacterium
GCA_013288575.1_Candidatus_Arkhamiaceae_bacterium
GCA_002787615.1_Candidatus_Arkhamiaceae_bacterium
GCA_013288565.1_Candidatus_Jistubacteraceae_bacterium
GCA_010031735.1_Candidatus_Jistubacteraceae_bacterium
GCA_903821695.1_Candidatus_Jistubacteraceae_bacterium
GCA_002422205.1_Candidatus_Mitibacteraceae_bacterium
GCA_002346425.1_Candidatus_Mitibacteraceae_bacterium
GCA_002725445.1_Candidatus_Mitibacteraceae_bacterium
GCA_003241645.1_Candidatus_Mitibacteraceae_bacterium
GCA_002422745.1_Candidatus_Mitibacteraceae_bacterium
GCA_002291925.1_Candidatus_Mitibacteraceae_bacterium
GCA_002291845.1_Candidatus_Mitibacteraceae_bacterium
GCA_004293935.1_Candidatus_Mitibacteraceae_bacterium
GCA_002423155.1_Candidatus_Mitibacteraceae_bacterium
GCA_013288985.1_Candidatus_Mitibacteraceae_bacterium
GCA_013288335.1_Candidatus_Mitibacteraceae_bacterium
GCA_013289445.1_Candidatus_Mitibacteraceae_bacterium
GCA_013288305.1_Candidatus_Mitibacteraceae_bacterium
GCA_009926845.1_Candidatus_Mitibacteraceae_bacterium
GCA_005792635.1_Candidatus_Mitibacteraceae_bacterium
Rickettsiaceae_endosymbiont_of_Amblyomma_cajennese_Ac37b_GCA_000746585.2
Chelativorans_oligotrophicus_GCA_011317505.1_GCA_011317505.1_ASM1131750v1
Bartonella_quintana_GCA_009936175.1
Rhizobium_leguminosarum_GCA_011604525.1
Ahrensia_kielensis_GCA_000374465.1_GCA_000374465.1_ASM37446v1
Roseibium_hamelinense_GCA_007830545.1
Polymorphum_gilvum_GCA_000192745.1
Pseudovibrio_sp._GCA_000236645.1
Methylocystis_bryophila_GCA_002117405.1
Methylocella_silvestris_GCA_000021745.1
Methylorubrum_extorquens_GCA_000018845.1
Xanthobacter_autotrophicus_GCA_000017645.1
Rhodopseudomonas_palustris_GCA_013415845.1
Pelagibacterium_halotolerans_GCA_013004005.1
Rhodomicrobium_vannielii_GCA_000166055.1
Hyphomicrobium_denitrificans_GCA_000143145.1
Thermopetrobacter_sp._GCA_000746275.1_GCA_000746275.1_ASM74627v1
alphaproteobacterium_IMCC14465_GCA_000293845.2
Candidatus_Micropelagos_thuwalensis_GCA_000469155_1
Parvibaculum_lavamentivorans_GCA_000017565.1
Rhodobacter_sphaeroides_GCA_000015985.1
Paracoccus_denitrificans_GCA_004063735.1
Rubellimicrobium_rubrum_GCA_006152115.1
Ketogulonicigenium_vulgare_GCA_001399515.1
Sagittula_stellata_GCA_000169415.1
Ruegeria_pomeroyi_GCA_000011965.2
Octadecabacter_arcticus_GCA_000155735.2
Jannaschia_rubra_GCA_900113265.1
Neomegalonema_perideroedes_GCA_000374145.1_GCA_000374145.1_ASM37414v1
Phenylobacterium_zucineum_GCA_000017265.1
Caulobacter_vibrioides_GCA_000022005.1
alphaproteobacterium_L41A_GCA_000335735.1_GCA_000335735.1_L41A.1.0
Brevundimonas_subvibrioides_GCA_000144605.1
Asticcacaulis_excentricus_GCA_000175215.2
Oceanicaulis_alexandrii_GCA_000420265.1_GCA_000420265.1_ASM42026v1
Maricaulis_maris_GCA_003634045.1
Hyphomonas_neptunium_GCA_000013025.1
Hirschia_baltica_GCA_000023785.1
Sphingomonas_wittichii_GCA_000016765.1
Sphingobium_indicum_GCA_000264945.2
Zymomonas_mobilis_GCA_000007105.1
Sphingopyxis_macrogoltabida_GCA_001314325.1
Citromicrobium_sp._GCA_001304795.1
alphaproteobacterium_AAP81b_GCA_001295935.1
Kordiimonas_gwangyangensis_GCA_000375545.1_GCA_000375545.1_ASM37554v1
Sneathiella_glossodoripedis_GCA_000616095.1_GCA_000616095.1_ASM61609v1
Rhodovibrio_salinarum_GCA_000515255.1_GCA_000515255.1_ASM51525v1
Kiloniella_laminariae_GCA_000374005.1_GCA_000374005.1_ASM37400v1
Gluconacetobacter_diazotrophicus_GCA_000067045.1
Acetobacter_aceti_GCA_000193495.2
Gluconobacter_oxydans_GCA_000583855.1
Commensalibacter_intestini_GCA_000231445.2
Granulibacter_bethesdensis_GCA_000576145.2
Acidiphilium_multivorum_GCA_000202835.1
Acidisphaera_rubrifaciens_GCA_000964365.1
Rubritepida_flocculans_GCA_000425365.1_GCA_000425365.1_ASM42536v1
Belnapia_moabensis_GCA_000745835.1_GCA_000745835.1_ASM74583v1
Roseomonas_gilardii_GCA_001941945.1
Elioraea_tepidiphila_GCA_000378465.1_GCA_000378465.1_ASM37846v1
alphaproteobacterium_BAL199_GCA_000171835.1
Thalassobaculum_salexigens_GCA_000423805.1_GCA_000423805.1_ASM42380v1
Candidatus_Puniceispirillum_marinum_GCA_000024465.1
Oceanibaculum_indicum_GCA_003633955.1
Rhodospirillum_rubrum_GCA_000225955.1
Pararhodospirillum_photometricum_GCA_000284415.2
Roseospirillum_parvum_GCA_900100455.1
Caenispirillum_salinarum_GCA_000315795.1
Magnetospirillum_magneticum_GCA_000009985.1
Rhodospirillum_centenum_GCA_014197805.1
Inquilinus_limosus_GCA_000423185.1_GCA_000423185.1_ASM42318v1
Thalassospira_profundimaris_GCA_000300275.1
Parvularcula_bermudensis_GCA_000152825.2
Terasakiella_pusilla_GCA_000688235.1_GCA_000688235.1_ASM68823v1
Tistrella_mobilis_GCA_000264455.2
Geminicoccus_roseus_GCA_000427665.1_GCA_000427665.1_ASM42766v1
Magnetofaba_australis_GCA_002109495.1
Magnetococcus_marinus_GCA_000014865.1
Ralstonia_pickettii_GCA_902374465.1_GCA_902374465.1_MGYG-HGUT-01384
Burkholderia_cepacia_GCA_009586235.1
Methylotenera_versatilis_GCA_000093025.1
Nitrosospira_briensis_GCA_000619905.2_GCA_000619905.2_ASM61990v2
Dasania_marina_GCA_000373485.1_GCA_000373485.1_ASM37348v1
Congregibacter_litoralis_GCA_000153125.2

Arg_z_0.30

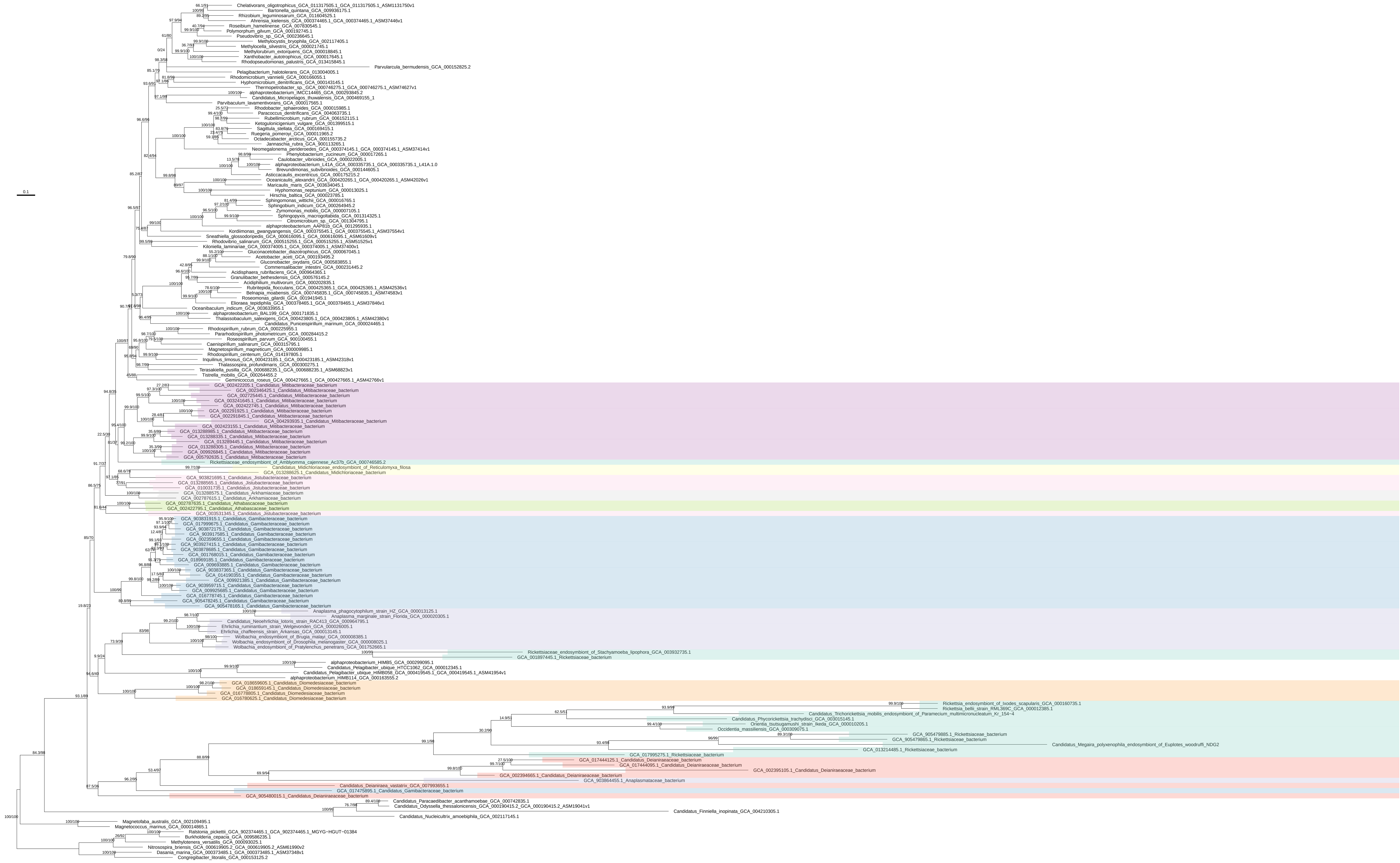
0.1
Chelativorans_oligotrophicus_GCA_011317505.1_GCA_011317505.1_ASM1131750v1
Bartonella_quintana_GCA_009936175.1
Rhizobium_leguminosarum_GCA_011604525.1
Ahrensia_kielensis_GCA_000374465.1_GCA_000374465.1_ASM37446v1
Roseibium_hamelinense_GCA_007830545.1
Polymorphum_gilvum_GCA_000192745.1
Pseudovibrio_sp._GCA_000236645.1
Methylocystis_bryophila_GCA_002117405.1
Methylocella_silvestris_GCA_000021745.1
Methylorubrum_extorquens_GCA_000018845.1
Xanthobacter_autotrophicus_GCA_000017645.1
Rhodopseudomonas_palustris_GCA_013415845.1
Parvularcula_bermudensis_GCA_000152825.2
Pelagibacterium_halotolerans_GCA_013004005.1
Rhodomicrobium_vannielii_GCA_000166055.1
Hyphomicrobium_denitrificans_GCA_000143145.1
Thermopetrobacter_sp._GCA_000746275.1_GCA_000746275.1_ASM74627v1
alphaproteobacterium_IMCC14465_GCA_000293845.2
Candidatus_Micropelagos_thuwalensis_GCA_000469155_1
Parvibaculum_lavamentivorans_GCA_000017565.1
Rhodobacter_sphaeroides_GCA_000015985.1
Paracoccus_denitrificans_GCA_004063735.1
Rubellimicrobium_rubrum_GCA_006152115.1
Ketogulonicigenium_vulgare_GCA_001399515.1
Sagittula_stellata_GCA_000169415.1
Ruegeria_pomeroyi_GCA_000011965.2
Octadecabacter_arcticus_GCA_000155735.2
Jannaschia_rubra_GCA_900113265.1
Neomegalonema_perideroedes_GCA_000374145.1_GCA_000374145.1_ASM37414v1
Phenylobacterium_zucineum_GCA_000017265.1
Caulobacter_vibrioides_GCA_000022005.1
alphaproteobacterium_L41A_GCA_000335735.1_GCA_000335735.1_L41A.1.0
Brevundimonas_subvibrioides_GCA_000144605.1
Asticcacaulis_excentricus_GCA_000175215.2
Oceanicaulis_alexandrii_GCA_000420265.1_GCA_000420265.1_ASM42026v1
Maricaulis_maris_GCA_003634045.1
Hyphomonas_neptunium_GCA_000013025.1
Hirschia_baltica_GCA_000023785.1
Sphingomonas_wittichii_GCA_000016765.1
Sphingobium_indicum_GCA_000264945.2
Zymomonas_mobilis_GCA_000007105.1
Sphingopyxis_macrogoltabida_GCA_001314325.1
Citromicrobium_sp._GCA_001304795.1
alphaproteobacterium_AAP81b_GCA_001295935.1
Kordiimonas_gwangyangensis_GCA_000375545.1_GCA_000375545.1_ASM37554v1
Sneathiella_glossodoripedis_GCA_000616095.1_GCA_000616095.1_ASM61609v1
Rhodovibrio_salinarum_GCA_000515255.1_GCA_000515255.1_ASM51525v1
Kiloniella_laminariae_GCA_000374005.1_GCA_000374005.1_ASM37400v1
Gluconacetobacter_diazotrophicus_GCA_000067045.1
Acetobacter_aceti_GCA_000193495.2
Gluconobacter_oxydans_GCA_000583855.1
Commensalibacter_intestini_GCA_000231445.2
Acidisphaera_rubrifaciens_GCA_000964365.1
Granulibacter_bethesdensis_GCA_000576145.2
Acidiphilium_multivorum_GCA_000202835.1
Rubritepida_flocculans_GCA_000425365.1_GCA_000425365.1_ASM42536v1
Belnapia_moabensis_GCA_000745835.1_GCA_000745835.1_ASM74583v1
Roseomonas_gilardii_GCA_001941945.1
Elioraea_tepidiphila_GCA_000378465.1_GCA_000378465.1_ASM37846v1
Oceanibaculum_indicum_GCA_003633955.1
alphaproteobacterium_BAL199_GCA_000171835.1
Thalassobaculum_salexigens_GCA_000423805.1_GCA_000423805.1_ASM42380v1
Candidatus_Puniceispirillum_marinum_GCA_000024465.1
Rhodospirillum_rubrum_GCA_000225955.1
Pararhodospirillum_photometricum_GCA_000284415.2
Roseospirillum_parvum_GCA_900100455.1
Caenispirillum_salinarum_GCA_000315795.1
Magnetospirillum_magneticum_GCA_000009985.1
Rhodospirillum_centenum_GCA_014197805.1
Inquilinus_limosus_GCA_000423185.1_GCA_000423185.1_ASM42318v1
Thalassospira_profundimaris_GCA_000300275.1
Terasakiella_pusilla_GCA_000688235.1_GCA_000688235.1_ASM68823v1
Tistrella_mobilis_GCA_000264455.2
Geminicoccus_roseus_GCA_000427665.1_GCA_000427665.1_ASM42766v1
GCA_002422205.1_Candidatus_Mitibacteraceae_bacterium
GCA_002346425.1_Candidatus_Mitibacteraceae_bacterium
GCA_002725445.1_Candidatus_Mitibacteraceae_bacterium
GCA_003241645.1_Candidatus_Mitibacteraceae_bacterium
GCA_002422745.1_Candidatus_Mitibacteraceae_bacterium
GCA_002291925.1_Candidatus_Mitibacteraceae_bacterium
GCA_002291845.1_Candidatus_Mitibacteraceae_bacterium
GCA_004293935.1_Candidatus_Mitibacteraceae_bacterium
GCA_002423155.1_Candidatus_Mitibacteraceae_bacterium
GCA_013288985.1_Candidatus_Mitibacteraceae_bacterium
GCA_013288335.1_Candidatus_Mitibacteraceae_bacterium
GCA_013289445.1_Candidatus_Mitibacteraceae_bacterium
GCA_013288305.1_Candidatus_Mitibacteraceae_bacterium
GCA_009926845.1_Candidatus_Mitibacteraceae_bacterium
GCA_005792635.1_Candidatus_Mitibacteraceae_bacterium
Rickettsiaceae_endosymbiont_of_Amblyomma_cajennense_Ac37b_GCA_000746585.2
Candidatus_Midichloriaceae_endosymbiont_of_Reticulomyxa_filosa
GCA_013288625.1_Candidatus_Midichloriaceae_bacterium
GCA_903821695.1_Candidatus_Jistubacteraceae_bacterium
GCA_013288565.1_Candidatus_Jistubacteraceae_bacterium
GCA_010031735.1_Candidatus_Jistubacteraceae_bacterium
GCA_013288575.1_Candidatus_Arkhamiaceae_bacterium
GCA_002787615.1_Candidatus_Arkhamiaceae_bacterium
GCA_002787635.1_Candidatus_Athabascaceae_bacterium
GCA_002422795.1_Candidatus_Athabascaceae_bacterium
GCA_003531345.1_Candidatus_Jistubacteraceae_bacterium
GCA_903831915.1_Candidatus_Gamibacteraceae_bacterium
GCA_017999675.1_Candidatus_Gamibacteraceae_bacterium
GCA_903872175.1_Candidatus_Gamibacteraceae_bacterium
GCA_903917585.1_Candidatus_Gamibacteraceae_bacterium
GCA_002359655.1_Candidatus_Gamibacteraceae_bacterium
GCA_903927415.1_Candidatus_Gamibacteraceae_bacterium
GCA_903878685.1_Candidatus_Gamibacteraceae_bacterium
GCA_001768015.1_Candidatus_Gamibacteraceae_bacterium
GCA_018969185.1_Candidatus_Gamibacteraceae_bacterium
GCA_009693885.1_Candidatus_Gamibacteraceae_bacterium
GCA_903837365.1_Candidatus_Gamibacteraceae_bacterium
GCA_014190355.1_Candidatus_Gamibacteraceae_bacterium
GCA_009921385.1_Candidatus_Gamibacteraceae_bacterium
GCA_903959715.1_Candidatus_Gamibacteraceae_bacterium
GCA_009925685.1_Candidatus_Gamibacteraceae_bacterium
GCA_016778745.1_Candidatus_Gamibacteraceae_bacterium
GCA_905478245.1_Candidatus_Gamibacteraceae_bacterium
GCA_905478165.1_Candidatus_Gamibacteraceae_bacterium
Anaplasma_phagocytophilum_strain_HZ_GCA_000013125.1
Anaplasma_marginale_strain_Florida_GCA_000020305.1
Candidatus_Neoehrlichia_lotoris_strain_RAC413_GCA_000964795.1
Ehrlichia_ruminantium_strain_Welgevonden_GCA_000026005.1
Ehrlichia_chaffeensis_strain_Arkansas_GCA_000013145.1
Wolbachia_endosymbiont_of_Brugia_malayi_GCA_000008385.1
Wolbachia_endosymbiont_of_Drosophila_melanogaster_GCA_000008025.1
Wolbachia_endosymbiont_of_Pratylenchus_penetrans_GCA_001752665.1
Rickettsiaceae_endosymbiont_of_Stachyamoeba_lipophora_GCA_003932735.1
GCA_001897445.1_Rickettsiaceae_bacterium
alphaproteobacterium_HIMB5_GCA_000299095.1
Candidatus_Pelagibacter_ubique_HTCC1062_GCA_000012345.1
Candidatus_Pelagibacter_ubique_HIMB058_GCA_000419545.1_GCA_000419545.1_ASM41954v1
alphaproteobacterium_HIMB114_GCA_000163555.2
GCA_018659605.1_Candidatus_Diomedesiaceae_bacterium
GCA_018659145.1_Candidatus_Diomedesiaceae_bacterium
GCA_016778805.1_Candidatus_Diomedesiaceae_bacterium
GCA_016780625.1_Candidatus_Diomedesiaceae_bacterium
Rickettsia_endosymbiont_of_Ixodes_scapularis_GCA_000160735.1
Rickettsia_bellii_strain_RML369C_GCA_000012385.1
Candidatus_Trichorickettsia_mobilis_endosymbiont_of_Paramecium_multimicronucleatum_Kr_154-4
Candidatus_Phycorickettsia_trachydisci_GCA_003015145.1
Orientia_tsutsugamushi_strain_Ikeda_GCA_000010205.1
Occidentia_massiliensis_GCA_000309075.1
GCA_905479885.1_Rickettsiaceae_bacterium
GCA_905479865.1_Rickettsiaceae_bacterium
Candidatus_Megaira_polyxenophila_endosymbiont_of_Euplotes_woodruffi_NDG2
GCA_017995275.1_Rickettsiaceae_bacterium
GCA_013214485.1_Rickettsiaceae_bacterium
GCA_017444125.1_Candidatus_Deianiraeaceae_bacterium
GCA_017444095.1_Candidatus_Deianiraeaceae_bacterium
GCA_002395105.1_Candidatus_Deianiraeaceae_bacterium
GCA_002394665.1_Candidatus_Deianiraeaceae_bacterium
GCA_903864455.1_Anaplasmataceae_bacterium
Candidatus_Deianiraea_vastatrix_GCA_007993655.1
GCA_017475895.1_Candidatus_Gamibacteraceae_bacterium
GCA_905480015.1_Candidatus_Deianiraeaceae_bacterium
Candidatus_Paracaedibacter_acanthamoebae_GCA_000742835.1
Candidatus_Odyssella_thessalonicensis_GCA_000190415.2_GCA_000190415.2_ASM19041v1
Candidatus_Finniella_inopinata_GCA_004210305.1
Candidatus_Nucleicultrix_amoebiphila_GCA_002117145.1
Magnetofaba_australis_GCA_002109495.1
Magnetococcus_marinus_GCA_000014865.1
Ralstonia_pickettii_GCA_902374465.1_GCA_902374465.1_MGYG-HGUT-01384
Burkholderia_cepacia_GCA_009586235.1
Methylotenera_versatilis_GCA_000093025.1
Nitrosospira_briensis_GCA_000619905.2_GCA_000619905.2_ASM61990v2
Dasania_marina_GCA_000373485.1_GCA_000373485.1_ASM37348v1
Congregibacter_litoralis_GCA_000153125.2

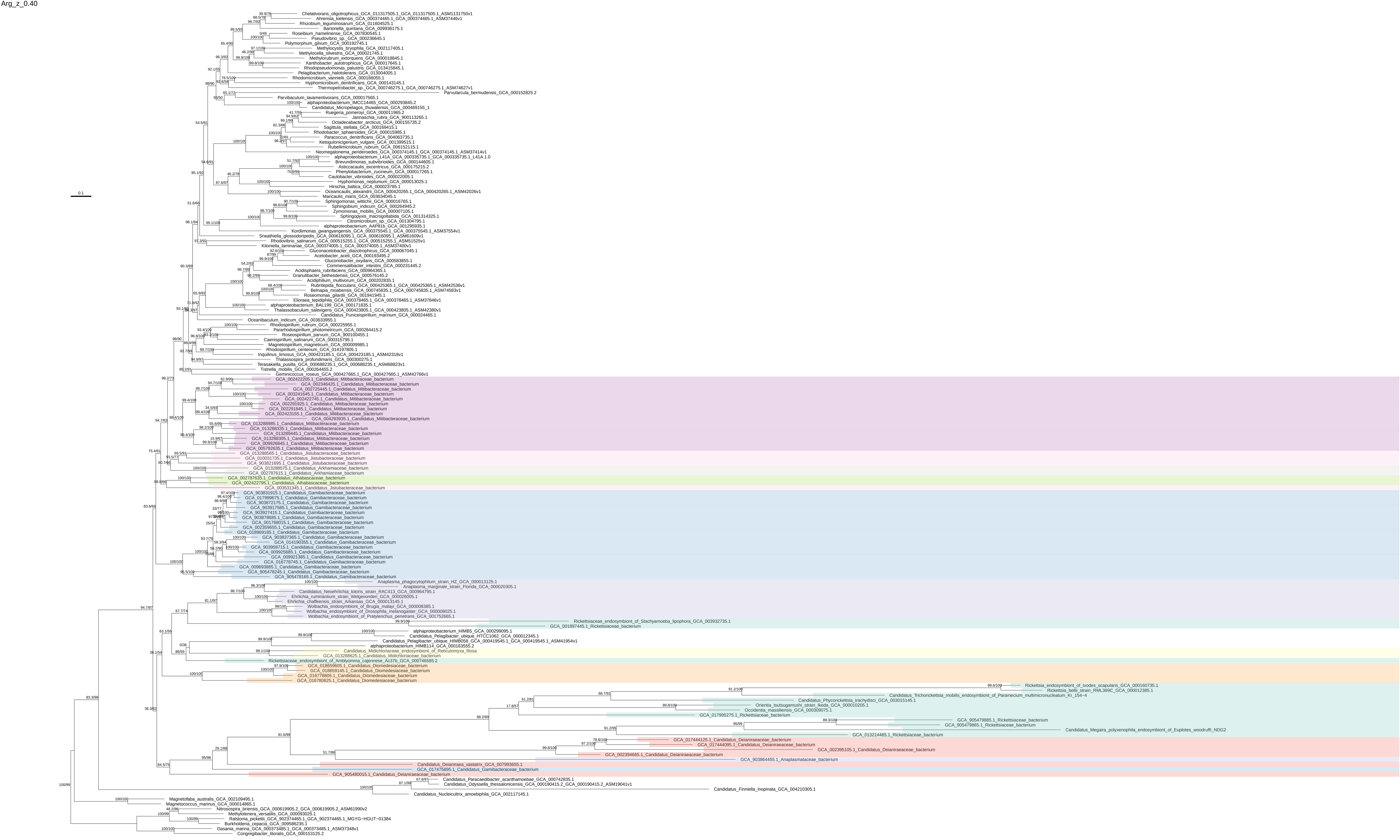

Arg_z_0.50

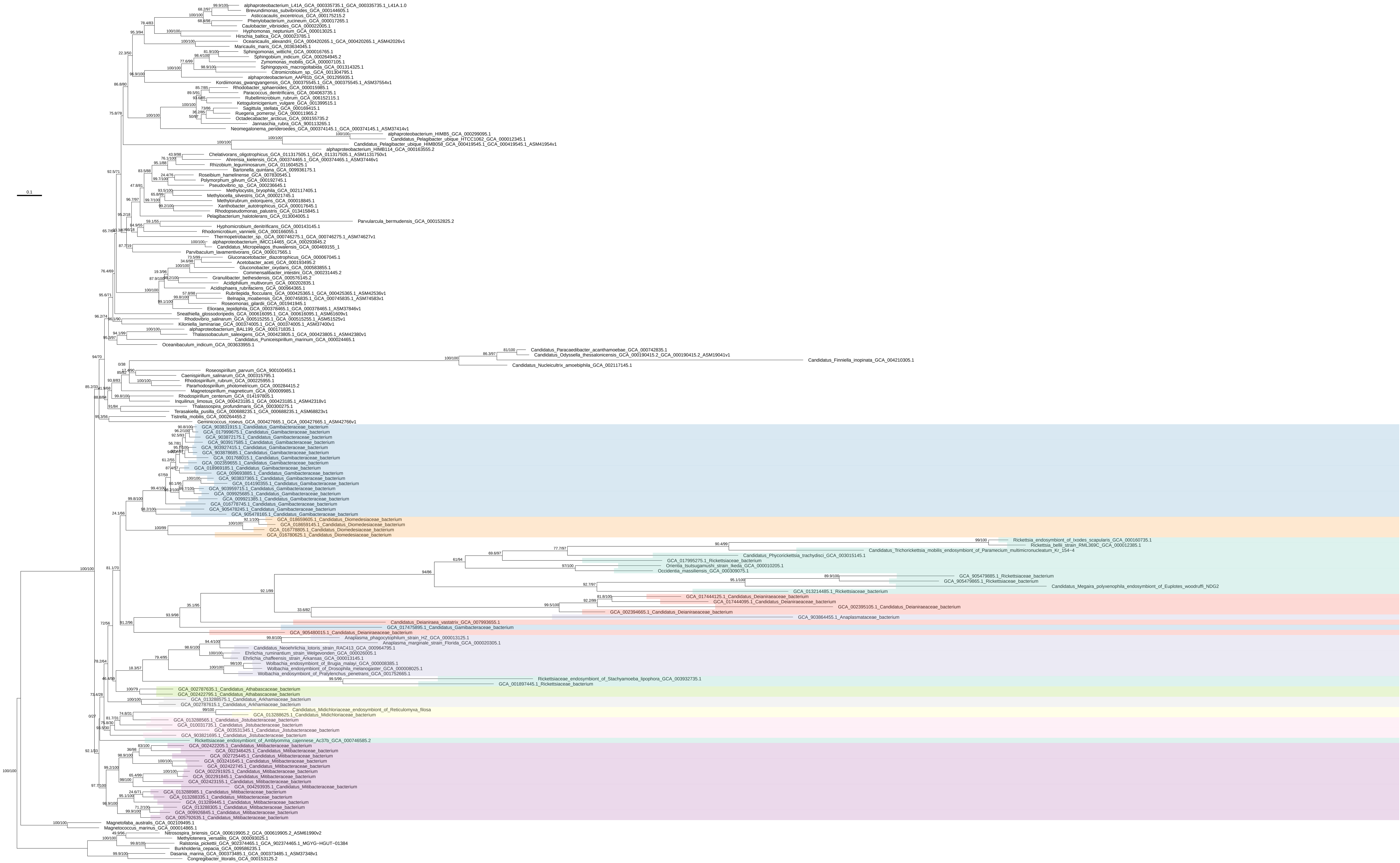

0.1
alphaproteobacterium_L41A_GCA_000335735.1_GCA_000335735.1_L41A.1.0
Brevundimonas_subvibrioides_GCA_000144605.1
Asticcacaulis_excentricus_GCA_000175215.2
Phenylobacterium_zucineum_GCA_000017265.1
Caulobacter_vibrioides_GCA_000022005.1
Hyphomonas_neptunium_GCA_000013025.1
Hirschia_baltica_GCA_000023785.1
Oceanicaulis_alexandrii_GCA_000420265.1_GCA_000420265.1_ASM42026v1
Maricaulis_maris_GCA_003634045.1
Sphingomonas_wittichii_GCA_000016765.1
Sphingobium_indicum_GCA_000264945.2
Zymomonas_mobilis_GCA_000007105.1
Sphingopyxis_macrogoltabida_GCA_001314325.1
Citromicrobium_sp._GCA_001304795.1
alphaproteobacterium_AAP81b_GCA_001295935.1
Kordiimonas_gwangyangensis_GCA_000375545.1_GCA_000375545.1_ASM37554v1
Rhodobacter_sphaeroides_GCA_000015985.1
Paracoccus_denitrificans_GCA_004063735.1
Rubellimicrobium_rubrum_GCA_006152115.1
Ketogulonicigenium_vulgare_GCA_001399515.1
Sagittula_stellata_GCA_000169415.1
Ruegeria_pomeroyi_GCA_000011965.2
Octadecabacter_arcticus_GCA_000155735.2
Jannaschia_rubra_GCA_900113265.1
Neomegalonema_perideroedes_GCA_000374145.1_GCA_000374145.1_ASM37414v1
alphaproteobacterium_HIMB5_GCA_000299095.1
Candidatus_Pelagibacter_ubique_HTCC1062_GCA_000012345.1
Candidatus_Pelagibacter_ubique_HIMB058_GCA_000419545.1_GCA_000419545.1_ASM41954v1
alphaproteobacterium_HIMB114_GCA_000163555.2
Chelativorans_oligotrophicus_GCA_011317505.1_GCA_011317505.1_ASM1131750v1
Ahrensia_kielensis_GCA_000374465.1_GCA_000374465.1_ASM37446v1
Rhizobium_leguminosarum_GCA_011604525.1
Bartonella_quintana_GCA_009936175.1
Roseibium_hamelinense_GCA_007830545.1
Polymorphum_gilvum_GCA_000192745.1
Pseudovibrio_sp._GCA_000236645.1
Methylocystis_bryophila_GCA_002117405.1
Methylocella_silvestris_GCA_000021745.1
Methylorubrum_extorquens_GCA_000018845.1
Xanthobacter_autotrophicus_GCA_000017645.1
Rhodopseudomonas_palustris_GCA_013415845.1
Pelagibacterium_halotolerans_GCA_013004005.1
Parvularcula_bermudensis_GCA_000152825.2
Hyphomicrobium_denitrificans_GCA_000143145.1
Rhodomicrobium_vannielii_GCA_000166055.1
Thermopetrobacter_sp._GCA_000746275.1_GCA_000746275.1_ASM74627v1
alphaproteobacterium_IMCC14465_GCA_000293845.2
Candidatus_Micropelagos_thuwalensis_GCA_000469155_1
Parvibaculum_lavamentivorans_GCA_000017565.1
Gluconacetobacter_diazotrophicus_GCA_000067045.1
Acetobacter_aceti_GCA_001193495.2
Gluconobacter_oxydans_GCA_000583855.1
Commensalibacter_intestini_GCA_000231445.2
Granulibacter_bethesdensis_GCA_000576145.2
Acidiphilium_multivorum_GCA_000202835.1
Acidisphaera_rubrifaciens_GCA_000964365.1
Rubritepida_flocculans_GCA_000425365.1_GCA_000425365.1_ASM42536v1
Belnapia_moabensis_GCA_000745835.1_GCA_000745835.1_ASM74583v1
Roseomonas_gilardii_GCA_001941945.1
Elioraea_tepidiphila_GCA_000378465.1_GCA_000378465.1_ASM37846v1
Sneathiella_glossodoripedis_GCA_000616095.1_GCA_000616095.1_ASM61609v1
Rhodovibrio_salinarum_GCA_000515255.1_GCA_000515255.1_ASM51525v1
Kiloniella_laminariae_GCA_000374005.1_GCA_000374005.1_ASM37400v1
alphaproteobacterium_BAL199_GCA_000171835.1
Thalassobaculum_salexigens_GCA_000423805.1_GCA_000423805.1_ASM42380v1
Candidatus_Puniceispirillum_marinum_GCA_000024465.1
Oceanibaculum_indicum_GCA_003633955.1
Candidatus_Paracaedibacter_acanthamoebae_GCA_000742835.1
Candidatus_Odyssella_thessalonicensis_GCA_000190415.2_GCA_000190415.2_ASM19041v1
Candidatus_Finniella_inopinata_GCA_004210305.1
Candidatus_Nucleicultrix_amoebiphila_GCA_002117145.1
Roseospirillum_parvum_GCA_900100455.1
Caenispirillum_salinarum_GCA_000315795.1
Rhodospirillum_rubrum_GCA_000225955.1
Pararhodospirillum_photometricum_GCA_000284415.2
Magnetospirillum_magneticum_GCA_000009985.1
Rhodospirillum_centenum_GCA_014197805.1
Inquilinus_limosus_GCA_000423185.1_GCA_000423185.1_ASM42318v1
Thalassospira_profundimaris_GCA_000300275.1
Terasakiella_pusilla_GCA_000688235.1_GCA_000688235.1_ASM68823v1
Tistrella_mobilis_GCA_000264455.2
Geminicoccus_roseus_GCA_000427665.1_GCA_000427665.1_ASM42766v1
GCA_903831915.1_Candidatus_Gamibacteraceae_bacterium
GCA_017999675.1_Candidatus_Gamibacteraceae_bacterium
GCA_903872175.1_Candidatus_Gamibacteraceae_bacterium
GCA_903917585.1_Candidatus_Gamibacteraceae_bacterium
GCA_903927415.1_Candidatus_Gamibacteraceae_bacterium
GCA_903878685.1_Candidatus_Gamibacteraceae_bacterium
GCA_001768015.1_Candidatus_Gamibacteraceae_bacterium
GCA_002359655.1_Candidatus_Gamibacteraceae_bacterium
GCA_018969185.1_Candidatus_Gamibacteraceae_bacterium
GCA_009693885.1_Candidatus_Gamibacteraceae_bacterium
GCA_903837365.1_Candidatus_Gamibacteraceae_bacterium
GCA_014190355.1_Candidatus_Gamibacteraceae_bacterium
GCA_903959715.1_Candidatus_Gamibacteraceae_bacterium
GCA_009925685.1_Candidatus_Gamibacteraceae_bacterium
GCA_009921385.1_Candidatus_Gamibacteraceae_bacterium
GCA_016778745.1_Candidatus_Gamibacteraceae_bacterium
GCA_905478245.1_Candidatus_Gamibacteraceae_bacterium
GCA_905478165.1_Candidatus_Gamibacteraceae_bacterium
GCA_018659605.1_Candidatus_Diomedesiaceae_bacterium
GCA_018659145.1_Candidatus_Diomedesiaceae_bacterium
GCA_016778805.1_Candidatus_Diomedesiaceae_bacterium
GCA_016780625.1_Candidatus_Diomedesiaceae_bacterium
Rickettsia_endosymbiont_of_Ixodes_scapularis_GCA_000160735.1
Rickettsia_bellii_strain_RML369C_GCA_000012385.1
Candidatus_Trichorickettsia_mobilis_endosymbiont_of_Paramecium_multimicronucleatum_Kr_154−4
Candidatus_Phycorickettsia_trachydisci_GCA_003015145.1
GCA_017995275.1_Rickettsiaceae_bacterium
Orientia_tsutsugamushi_strain_Ikeda_GCA_000010205.1
Occidentia_massiliensis_GCA_000309075.1
GCA_905479885.1_Rickettsiaceae_bacterium
GCA_905479865.1_Rickettsiaceae_bacterium
Candidatus_Megaira_polyxenophila_endosymbiont_of_Euplotes_woodruffi_NDG2
GCA_013214485.1_Rickettsiaceae_bacterium
GCA_017444125.1_Candidatus_Deianiraeaceae_bacterium
GCA_017444095.1_Candidatus_Deianiraeaceae_bacterium
GCA_002395105.1_Candidatus_Deianiraeaceae_bacterium
GCA_002394665.1_Candidatus_Deianiraeaceae_bacterium
GCA_903864455.1_Anaplasmataceae_bacterium
Candidatus_Deianiraea_vastatrix_GCA_007993655.1
GCA_017475895.1_Candidatus_Gamibacteraceae_bacterium
GCA_905480015.1_Candidatus_Deianiraeaceae_bacterium
Anaplasma_phagocytophilum_strain_HZ_GCA_000013125.1
Anaplasma_marginale_strain_Florida_GCA_000020305.1
Candidatus_Neoehrlichia_lotoris_strain_RAC413_GCA_000964795.1
Ehrlichia_ruminantium_strain_Welgevonden_GCA_000026005.1
Ehrlichia_chaffeensis_strain_Arkansas_GCA_000013145.1
Wolbachia_endosymbiont_of_Brugia_malayi_GCA_000008385.1
Wolbachia_endosymbiont_of_Drosophila_melanogaster_GCA_000008025.1
Wolbachia_endosymbiont_of_Pratylenchus_penetrans_GCA_001752665.1
Rickettsiaceae_endosymbiont_of_Stachyamoeba_lipophora_GCA_003932735.1
GCA_001897445.1_Rickettsiaceae_bacterium
GCA_002787635.1_Candidatus_Athabascaceae_bacterium
GCA_002422795.1_Candidatus_Athabascaceae_bacterium
GCA_013288575.1_Candidatus_Arkhamiaceae_bacterium
GCA_002787615.1_Candidatus_Arkhamiaceae_bacterium
Candidatus_Midichloriaceae_endosymbiont_of_Reticulomyxa_filosa
GCA_013288625.1_Candidatus_Midichloriaceae_bacterium
GCA_013288565.1_Candidatus_Jistubacteraceae_bacterium
GCA_010031735.1_Candidatus_Jistubacteraceae_bacterium
GCA_003531345.1_Candidatus_Jistubacteraceae_bacterium
GCA_903821695.1_Candidatus_Jistubacteraceae_bacterium
Rickettsiaceae_endosymbiont_of_Amblyomma_cajennese_Ac37b_GCA_000746585.2
GCA_002422205.1_Candidatus_Mitibacteraceae_bacterium
GCA_002346425.1_Candidatus_Mitibacteraceae_bacterium
GCA_002725445.1_Candidatus_Mitibacteraceae_bacterium
GCA_003241645.1_Candidatus_Mitibacteraceae_bacterium
GCA_002422745.1_Candidatus_Mitibacteraceae_bacterium
GCA_002291925.1_Candidatus_Mitibacteraceae_bacterium
GCA_002291845.1_Candidatus_Mitibacteraceae_bacterium
GCA_002423155.1_Candidatus_Mitibacteraceae_bacterium
GCA_004293935.1_Candidatus_Mitibacteraceae_bacterium
GCA_013288985.1_Candidatus_Mitibacteraceae_bacterium
GCA_013288335.1_Candidatus_Mitibacteraceae_bacterium
GCA_013289445.1_Candidatus_Mitibacteraceae_bacterium
GCA_013288305.1_Candidatus_Mitibacteraceae_bacterium
GCA_009926845.1_Candidatus_Mitibacteraceae_bacterium
GCA_005792635.1_Candidatus_Mitibacteraceae_bacterium
Magnetofaba_australis_GCA_002109495.1
Magnetococcus_marinus_GCA_000014865.1
Nitrosospira_briensis_GCA_000619905.2_GCA_000619905.2_ASM61990v2
Methylotenera_versatilis_GCA_000093025.1
Ralstonia_pickettii_GCA_902374465.1_GCA_902374465.1_MGYG−HGUT−01384
Burkholderia_cepacia_GCA_009586235.1
Dasania_marina_GCA_000373485.1_GCA_000373485.1_ASM37348v1
Congregibacter_litoralis_GCA_000153125.2

Histidine

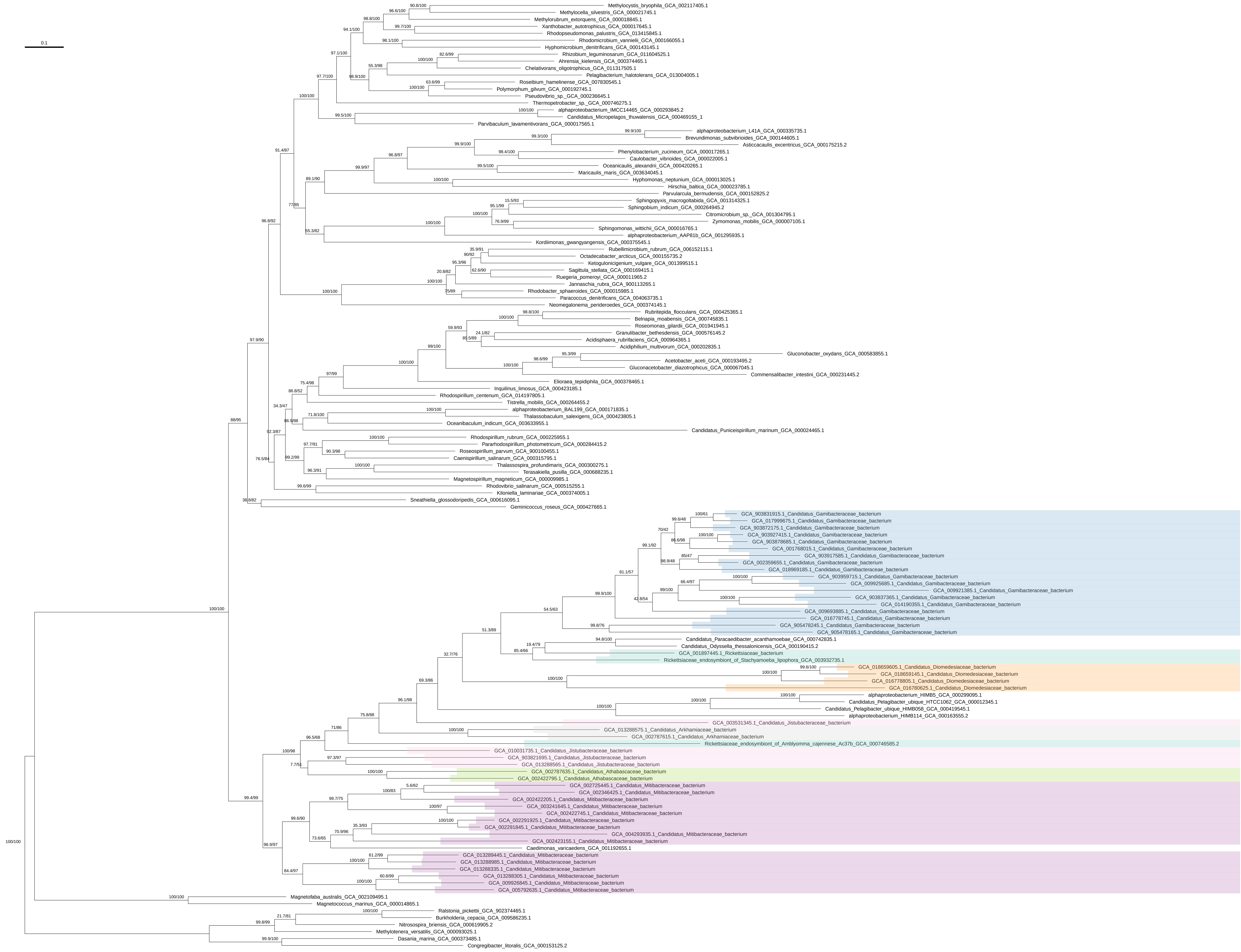

0.1
Methylocystis_bryophila_GCA_002117405.1
Methylocella_silvestris_GCA_000021745.1
Methylorubrum_extorquens_GCA_000018845.1
Xanthobacter_autotrophicus_GCA_000017645.1
Rhodopseudomonas_palustris_GCA_013415845.1
Rhodomicrobium_vannielii_GCA_000166055.1
Hyphomicrobium_denitrificans_GCA_000143145.1
Rhizobium_leguminosarum_GCA_011604525.1
Ahrensia_kielensis_GCA_000374465.1
Chelativorans_oligotrophicus_GCA_011317505.1
Pelagibacterium_halotolerans_GCA_013004005.1
Roseibium_hamelinense_GCA_007830545.1
Polymorphum_gilvum_GCA_000192745.1
Pseudovibrio_sp._GCA_000236645.1
Thermopetrobacter_sp._GCA_000746275.1
alphaproteobacterium_IMCC14465_GCA_000293845.2
Candidatus_Micropelagos_thuwalensis_GCA_000469155_1
Parvibaculum_lavamentivorans_GCA_000017565.1
alphaproteobacterium_L41A_GCA_000335735.1
Brevundimonas_subvibrioides_GCA_000144605.1
Asticcacaulis_excentricus_GCA_000175215.2
Phenylobacterium_zucineum_GCA_000017265.1
Caulobacter_vibrioides_GCA_000022005.1
Oceanicaulis_alexandrii_GCA_000420265.1
Maricaulis_maris_GCA_003634045.1
Hyphomonas_neptunium_GCA_000013025.1
Hirschia_baltica_GCA_000023785.1
Parvularcula_bermudensis_GCA_000152825.2
Sphingopyxis_macrogoltabida_GCA_001314325.1
Sphingobium_indicum_GCA_000264945.2
Citromicrobium_sp._GCA_001304795.1
Zymomonas_mobilis_GCA_000007105.1
Sphingomonas_wittichii_GCA_000016765.1
alphaproteobacterium_AAP81b_GCA_001295935.1
Kordiimonas_gwangyangensis_GCA_000375545.1
Rubellimicrobium_rubrum_GCA_006152115.1
Octadecabacter_arcticus_GCA_000155735.2
Ketogulonicigenium_vulgare_GCA_001399515.1
Sagittula_stellata_GCA_000169415.1
Ruegeria_pomeroyi_GCA_000011965.2
Jannaschia_rubra_GCA_900113265.1
Rhodobacter_sphaeroides_GCA_000015985.1
Paracoccus_denitrificans_GCA_004063735.1
Neomegalonema_perideroedes_GCA_000374145.1
Rubritepida_flocculans_GCA_000425365.1
Belnapia_moabensis_GCA_000745835.1
Roseomonas_gilardii_GCA_001941945.1
Granulibacter_bethesdensis_GCA_000576145.2
Acidisphaera_rubrifaciens_GCA_000964365.1
Acidiphilium_multivorum_GCA_000202835.1
Gluconobacter_oxydans_GCA_000583855.1
Acetobacter_aceti_GCA_000193495.2
Gluconacetobacter_diazotrophicus_GCA_000067045.1
Commensalibacter_intestini_GCA_000231445.2
Elioraea_tepidiphila_GCA_000378465.1
Inquilinus_limosus_GCA_000423185.1
Rhodospirillum_centenum_GCA_014197805.1
Tistrella_mobilis_GCA_000264455.2
alphaproteobacterium_BAL199_GCA_000171835.1
Thalassobaculum_salexigens_GCA_000423805.1
Oceanibaculum_indicum_GCA_003633955.1
Candidatus_Puniceispirillum_marinum_GCA_000024465.1
Rhodospirillum_rubrum_GCA_000225955.1
Pararhodospirillum_photometricum_GCA_000284415.2
Roseospirillum_parvum_GCA_900100455.1
Caenispirillum_salinarum_GCA_000315795.1
Thalassospira_profundimaris_GCA_000300275.1
Terasakiella_pusilla_GCA_000688235.1
Magnetospirillum_magneticum_GCA_000009985.1
Rhodovibrio_salinarum_GCA_000515255.1
Kiloniella_laminariae_GCA_000374005.1
Sneathiella_glossodoripedis_GCA_000616095.1
Geminicoccus_roseus_GCA_000427665.1
GCA_903831915.1_Candidatus_Gamibacteraceae_bacterium
GCA_017999675.1_Candidatus_Gamibacteraceae_bacterium
GCA_903872175.1_Candidatus_Gamibacteraceae_bacterium
GCA_903927415.1_Candidatus_Gamibacteraceae_bacterium
GCA_903878685.1_Candidatus_Gamibacteraceae_bacterium
GCA_001768015.1_Candidatus_Gamibacteraceae_bacterium
GCA_903917585.1_Candidatus_Gamibacteraceae_bacterium
GCA_002359655.1_Candidatus_Gamibacteraceae_bacterium
GCA_018969185.1_Candidatus_Gamibacteraceae_bacterium
GCA_903959715.1_Candidatus_Gamibacteraceae_bacterium
GCA_009925685.1_Candidatus_Gamibacteraceae_bacterium
GCA_009921385.1_Candidatus_Gamibacteraceae_bacterium
GCA_903837365.1_Candidatus_Gamibacteraceae_bacterium
GCA_014190355.1_Candidatus_Gamibacteraceae_bacterium
GCA_009693885.1_Candidatus_Gamibacteraceae_bacterium
GCA_016778745.1_Candidatus_Gamibacteraceae_bacterium
GCA_905478245.1_Candidatus_Gamibacteraceae_bacterium
GCA_905478165.1_Candidatus_Gamibacteraceae_bacterium
Candidatus_Paracaedibacter_acanthamoebae_GCA_000742835.1
Candidatus_Odyssella_thessalonicensis_GCA_000190415.2
GCA_001897445.1_Rickettsiaceae_bacterium
Rickettsiaceae_endosymbiont_of_Stachyamoeba_lipophora_GCA_003932735.1
GCA_018659605.1_Candidatus_Diomedesiaceae_bacterium
GCA_018659145.1_Candidatus_Diomedesiaceae_bacterium
GCA_016778805.1_Candidatus_Diomedesiaceae_bacterium
GCA_016780625.1_Candidatus_Diomedesiaceae_bacterium
alphaproteobacterium_HIMB5_GCA_000299095.1
Candidatus_Pelagibacter_ubique_HTCC1062_GCA_000012345.1
Candidatus_Pelagibacter_ubique_HIMB058_GCA_000419545.1
alphaproteobacterium_HIMB114_GCA_000163555.2
GCA_003531345.1_Candidatus_Jistubacteraceae_bacterium
GCA_013288575.1_Candidatus_Arkhamiaceae_bacterium
GCA_002787615.1_Candidatus_Arkhamiaceae_bacterium
Rickettsiaceae_endosymbiont_of_Amblyomma_cajennese_Ac37b_GCA_000746585.2
GCA_010031735.1_Candidatus_Jistubacteraceae_bacterium
GCA_903821695.1_Candidatus_Jistubacteraceae_bacterium
GCA_013288565.1_Candidatus_Jistubacteraceae_bacterium
GCA_002787635.1_Candidatus_Athabascaceae_bacterium
GCA_002422795.1_Candidatus_Athabascaceae_bacterium
GCA_002725445.1_Candidatus_Mitibacteraceae_bacterium
GCA_002346425.1_Candidatus_Mitibacteraceae_bacterium
GCA_002422205.1_Candidatus_Mitibacteraceae_bacterium
GCA_003241645.1_Candidatus_Mitibacteraceae_bacterium
GCA_002422745.1_Candidatus_Mitibacteraceae_bacterium
GCA_002291925.1_Candidatus_Mitibacteraceae_bacterium
GCA_002291845.1_Candidatus_Mitibacteraceae_bacterium
GCA_004293935.1_Candidatus_Mitibacteraceae_bacterium
GCA_002423155.1_Candidatus_Mitibacteraceae_bacterium
Caedimonas_varicaedens_GCA_001192655.1
GCA_013289445.1_Candidatus_Mitibacteraceae_bacterium
GCA_013288985.1_Candidatus_Mitibacteraceae_bacterium
GCA_013288335.1_Candidatus_Mitibacteraceae_bacterium
GCA_013288305.1_Candidatus_Mitibacteraceae_bacterium
GCA_009926845.1_Candidatus_Mitibacteraceae_bacterium
GCA_005792635.1_Candidatus_Mitibacteraceae_bacterium
Magnetofaba_australis_GCA_002109495.1
Magnetococcus_marinus_GCA_000014865.1
Ralstonia_pickettii_GCA_902374465.1
Burkholderia_cepacia_GCA_009586235.1
Nitrosospira_briensis_GCA_000619905.2
Methylotenera_versatilis_GCA_000093025.1
Dasania_marina_GCA_000373485.1
Congregibacter_litoralis_GCA_000153125.2

Histidine_z_0.10

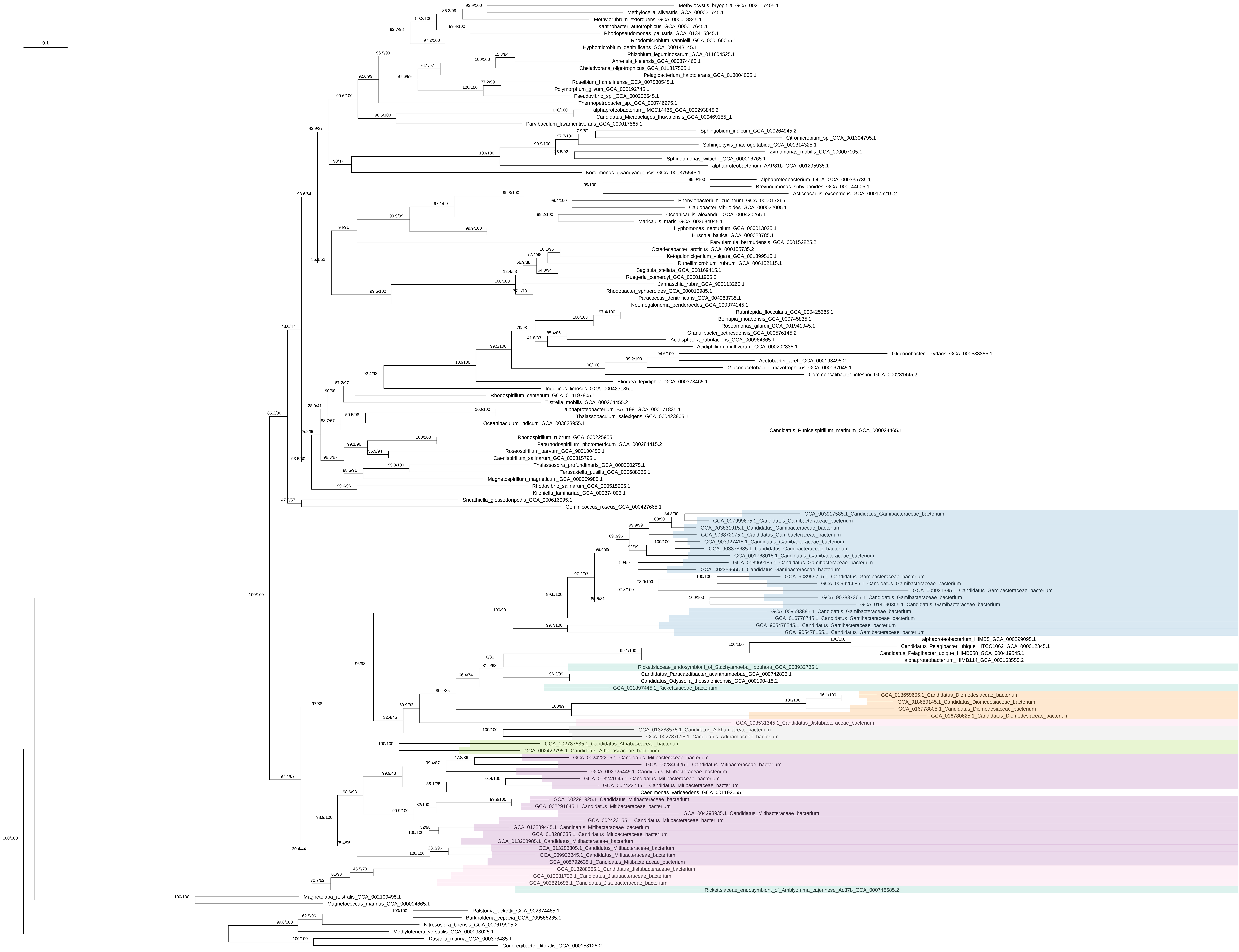

Histidine_z_0.20

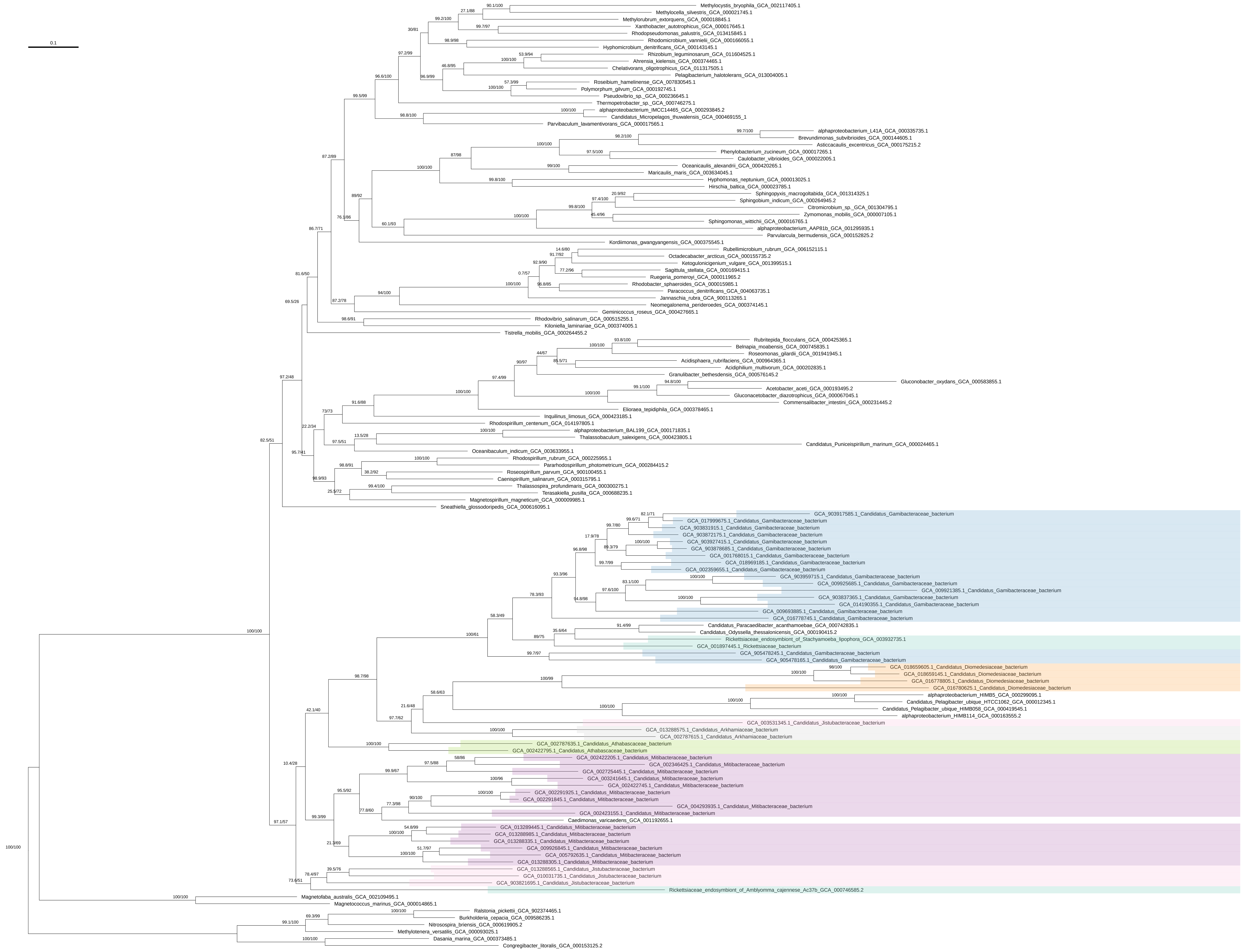

Histidine_z_0.30

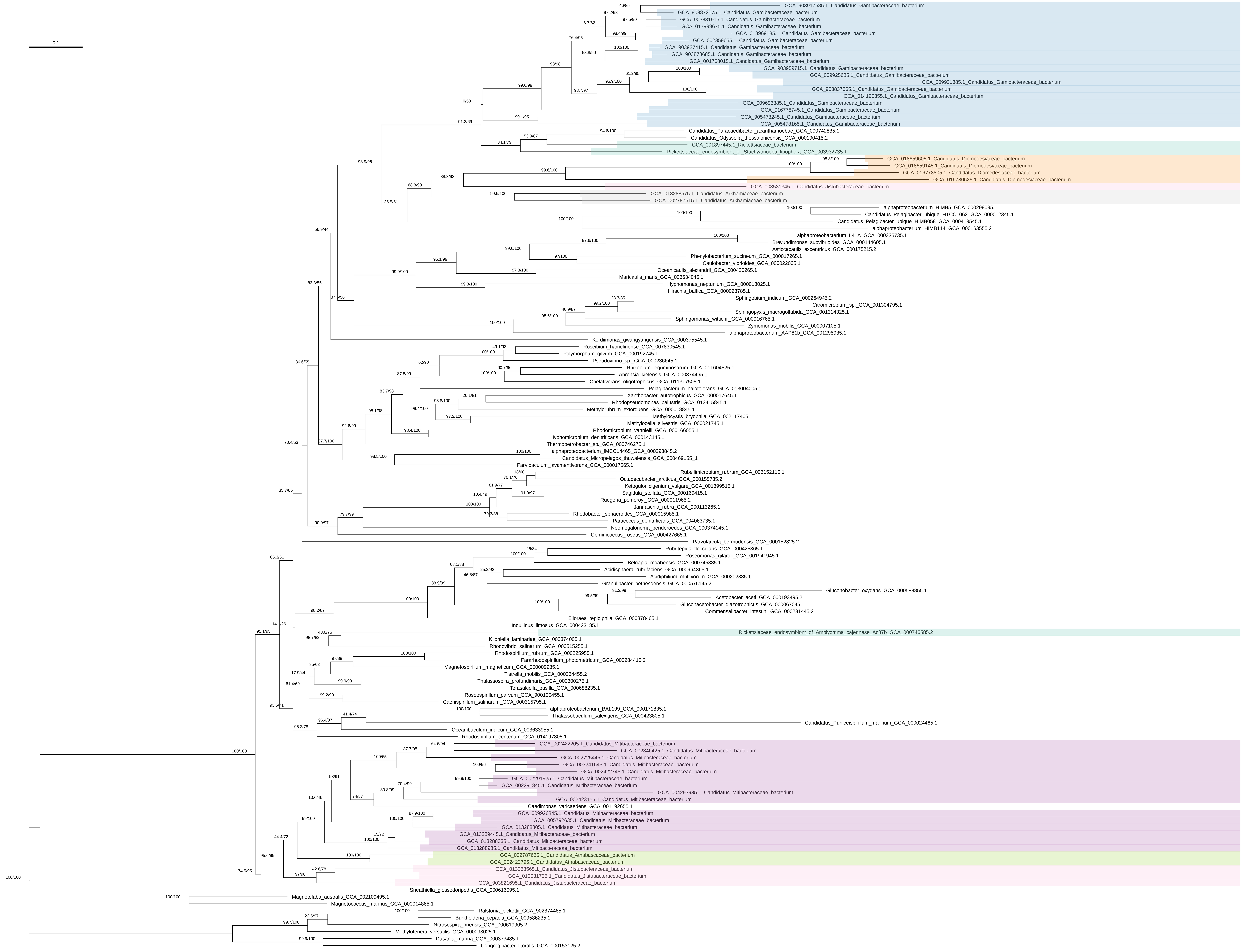

Histidine_z_0.40

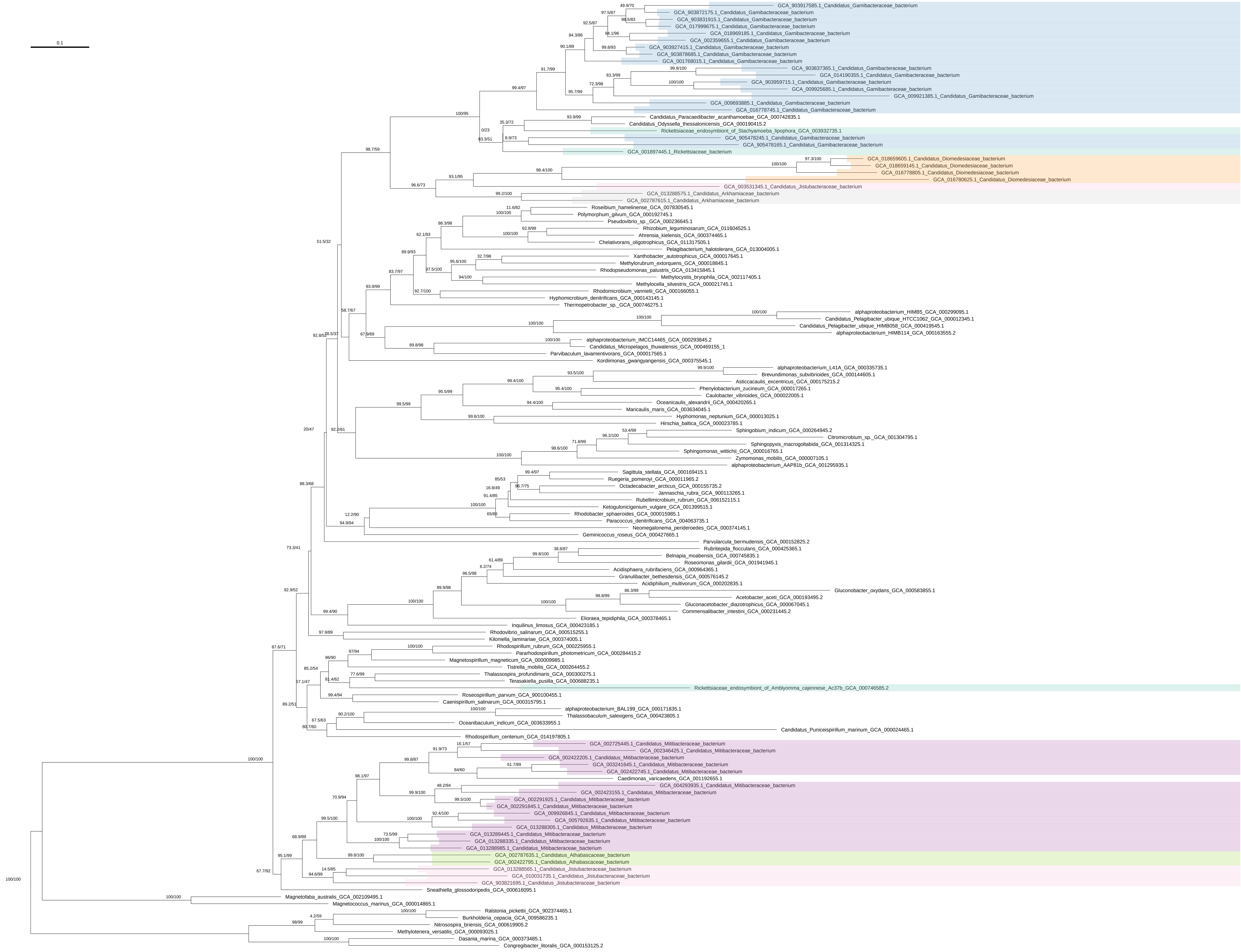

Histidine_z_0.50

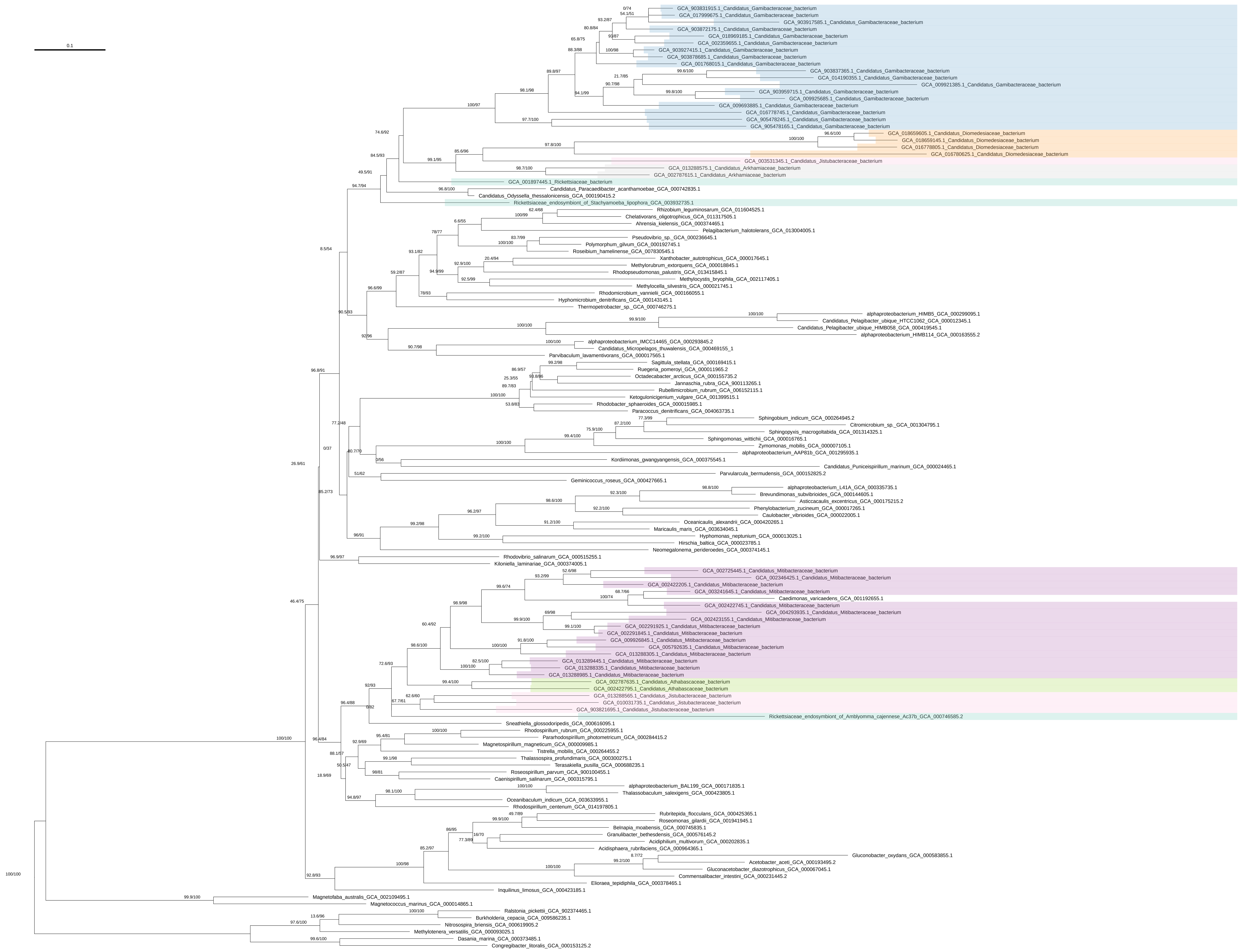

Ile_Val_Leu

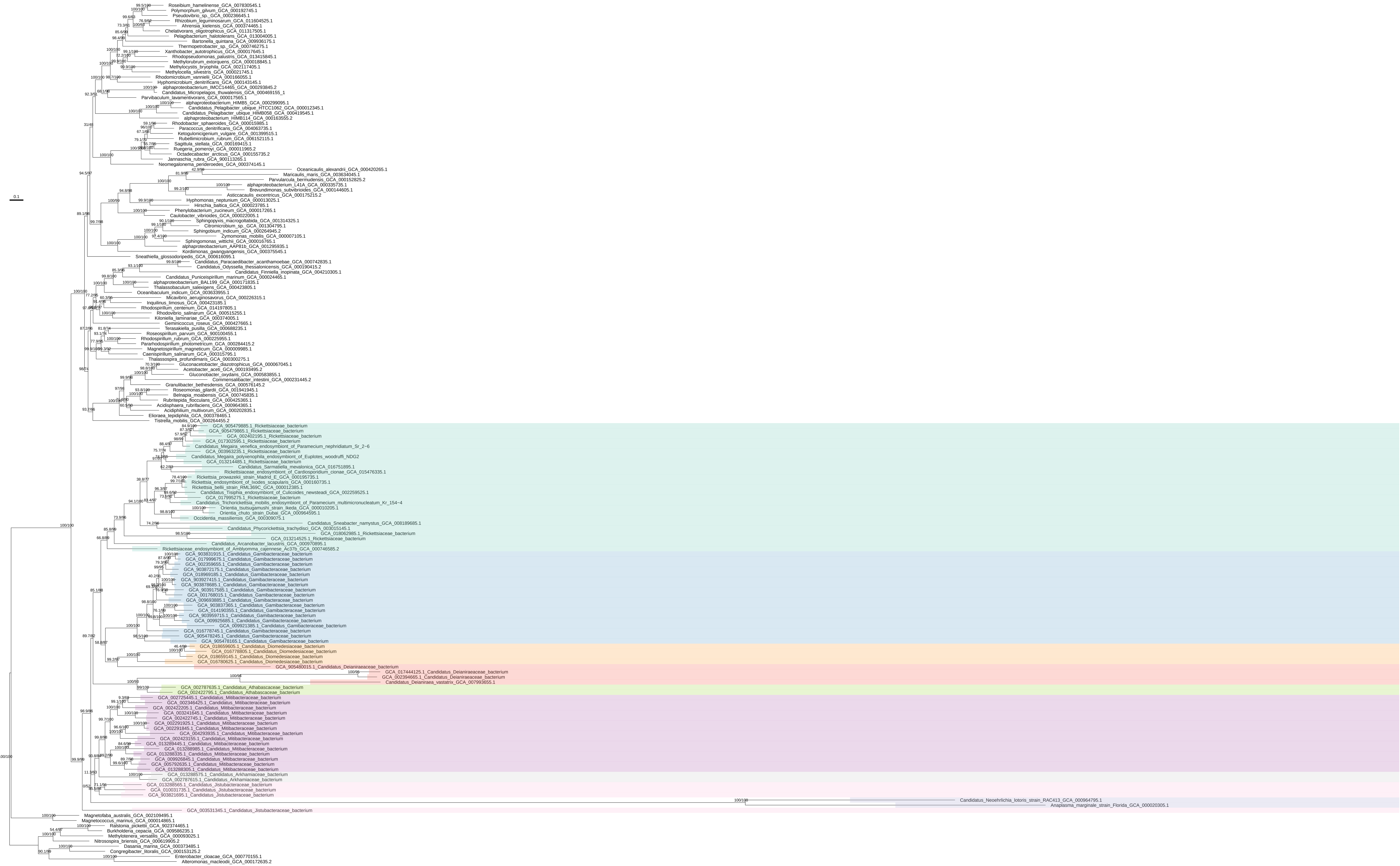

0.1
Roseibium_hamelinense_GCA_007830545.1
Polymorphum_gilvum_GCA_000192745.1
Pseudovibrio_sp._GCA_000236645.1
Rhizobium_leguminosarum_GCA_011604525.1
Ahrensia_kielensis_GCA_000374465.1
Chelativorans_oligotrophicus_GCA_011317505.1
Pelagibacterium_halotolerans_GCA_013004005.1
Bartonella_quintana_GCA_009936175.1
Thermopetrobacter_sp._GCA_000746275.1
Xanthobacter_autotrophicus_GCA_000017645.1
Rhodopseudomonas_palustris_GCA_013415845.1
Methylorubrum_extorquens_GCA_000018845.1
Methylocystis_bryophila_GCA_002117405.1
Methylocella_silvestris_GCA_000021745.1
Rhodomicrobium_vannielii_GCA_000166055.1
Hyphomicrobium_denitrificans_GCA_000143145.1
alphaproteobacterium_IMCC14465_GCA_000293845.2
Candidatus_Micropelagos_thuwalensis_GCA_000469155_1
Parvibaculum_lavamentivorans_GCA_000017565.1
alphaproteobacterium_HIMB5_GCA_000299095.1
Candidatus_Pelagibacter_ubique_HTCC1062_GCA_000012345.1
Candidatus_Pelagibacter_ubique_HIMB058_GCA_000419545.1
alphaproteobacterium_HIMB114_GCA_000163555.2
Rhodobacter_sphaeroides_GCA_000015985.1
Paracoccus_denitrificans_GCA_004063735.1
Ketogulonicigenium_vulgare_GCA_001399515.1
Rubellimicrobium_rubrum_GCA_006152115.1
Sagittula_stellata_GCA_000169415.1
Ruegeria_pomeroyi_GCA_000011965.2
Octadecabacter_arcticus_GCA_000155735.2
Jannaschia_rubra_GCA_900113265.1
Neomegalonema_perideroedes_GCA_000374145.1
Oceanicaulis_alexandrii_GCA_000420265.1
Maricaulis_maris_GCA_003634045.1
Parvularcula_bermudensis_GCA_000152825.2
alphaproteobacterium_L41A_GCA_000335735.1
Brevundimonas_subvibrioides_GCA_000144605.1
Asticcacaulis_excentricus_GCA_000175215.2
Hyphomonas_neptunium_GCA_000013025.1
Hirschia_baltica_GCA_000023785.1
Phenylobacterium_zucineum_GCA_000017265.1
Caulobacter_vibrioides_GCA_000022005.1
Sphingopyxis_macrogoltabida_GCA_001314325.1
Citromicrobium_sp._GCA_001304795.1
Sphingobium_indicum_GCA_000264945.2
Zymomonas_mobilis_GCA_000007105.1
Sphingomonas_wittichii_GCA_000016765.1
alphaproteobacterium_AAP81b_GCA_001295935.1
Kordiimonas_gwangyangensis_GCA_000375545.1
Sneathiella_glossodoripedis_GCA_000616095.1
Candidatus_Paracaedibacter_acanthamoebae_GCA_000742835.1
Candidatus_Odyssella_thessalonicensis_GCA_000190415.2
Candidatus_Finniella_inopinata_GCA_004210305.1
Candidatus_Puniceispirillum_marinum_GCA_000024465.1
alphaproteobacterium_BAL199_GCA_000171835.1
Thalassobaculum_salexigens_GCA_000423805.1
Oceanibaculum_indicum_GCA_003633955.1
Micavibrio_aeruginosavorus_GCA_000226315.1
Inquilinus_limosus_GCA_000423185.1
Rhodospirillum_centenum_GCA_014197805.1
Rhodovibrio_salinarum_GCA_000515255.1
Kiloniella_laminariae_GCA_000374005.1
Geminicoccus_roseus_GCA_000427665.1
Terasakiella_pusilla_GCA_000688235.1
Roseospirillum_parvum_GCA_900100455.1
Rhodospirillum_rubrum_GCA_000225955.1
Pararhodospirillum_photometricum_GCA_000284415.2
Magnetospirillum_magneticum_GCA_000009985.1
Caenispirillum_salinarum_GCA_000315795.1
Thalassospira_profundimaris_GCA_000300275.1
Gluconacetobacter_diazotrophicus_GCA_000067045.1
Acetobacter_aceti_GCA_000193495.2
Gluconobacter_oxydans_GCA_000583855.1
Commensalibacter_intestini_GCA_000231445.2
Granulibacter_bethesdensis_GCA_000576145.2
Roseomonas_gilardii_GCA_001941945.1
Belnapia_moabensis_GCA_000745835.1
Rubritepida_flocculans_GCA_000425365.1
Acidisphaera_rubrifaciens_GCA_000964365.1
Acidiphilium_multivorum_GCA_000202835.1
Elioraea_tepidiphila_GCA_000378465.1
Tistrella_mobilis_GCA_000264455.2
GCA_905479885.1_Rickettsiaceae_bacterium
GCA_905479865.1_Rickettsiaceae_bacterium
GCA_002402195.1_Rickettsiaceae_bacterium
GCA_017302595.1_Rickettsiaceae_bacterium
Candidatus_Megaira_venefica_endosymbiont_of_Paramecium_nephridiatum_Sr_2-6
GCA_003963235.1_Rickettsiaceae_bacterium
Candidatus_Megaira_polyxenophila_endosymbiont_of_Euplotes_woodruffi_NDG2
GCA_013214485.1_Rickettsiaceae_bacterium
Candidatus_Sarmatiella_mevalonica_GCA_016751895.1
Rickettsiaceae_endosymbiont_of_Cardiosporidium_cionae_GCA_015476335.1
Rickettsia_prowazekii_strain_Madrid_E_GCA_000195735.1
Rickettsia_endosymbiont_of_Ixodes_scapularis_GCA_000160735.1
Rickettsia_bellii_strain_RML369C_GCA_000012385.1
Candidatus_Tisiphia_endosymbiont_of_Culicoides_newsteadi_GCA_002259525.1
GCA_017995275.1_Rickettsiaceae_bacterium
Candidatus_Trichorickettsia_mobilis_endosymbiont_of_Paramecium_multimicronucleatum_Kr_154-4
Orientia_tsutsugamushi_strain_Ikeda_GCA_000010205.1
Orientia_chuto_strain_Dubai_GCA_000964595.1
Occidentia_massiliensis_GCA_000309075.1
Candidatus_Sneabacter_namystus_GCA_008189685.1
Candidatus_Phycorickettsia_trachydisci_GCA_003015145.1
GCA_018062985.1_Rickettsiaceae_bacterium
GCA_013214525.1_Rickettsiaceae_bacterium
Candidatus_Arcanobacter_lacustris_GCA_000970895.1
Rickettsiaceae_endosymbiont_of_Amblyomma_cajennense_Ac37b_GCA_000746585.2
GCA_903831915.1_Candidatus_Gamibacteraceae_bacterium
GCA_017999675.1_Candidatus_Gamibacteraceae_bacterium
GCA_002359655.1_Candidatus_Gamibacteraceae_bacterium
GCA_903872175.1_Candidatus_Gamibacteraceae_bacterium
GCA_018969185.1_Candidatus_Gamibacteraceae_bacterium
GCA_903927415.1_Candidatus_Gamibacteraceae_bacterium
GCA_903878685.1_Candidatus_Gamibacteraceae_bacterium
GCA_903917585.1_Candidatus_Gamibacteraceae_bacterium
GCA_001768015.1_Candidatus_Gamibacteraceae_bacterium
GCA_009693885.1_Candidatus_Gamibacteraceae_bacterium
GCA_903837365.1_Candidatus_Gamibacteraceae_bacterium
GCA_014190355.1_Candidatus_Gamibacteraceae_bacterium
GCA_903959715.1_Candidatus_Gamibacteraceae_bacterium
GCA_009925685.1_Candidatus_Gamibacteraceae_bacterium
GCA_009921385.1_Candidatus_Gamibacteraceae_bacterium
GCA_016778745.1_Candidatus_Gamibacteraceae_bacterium
GCA_905478245.1_Candidatus_Gamibacteraceae_bacterium
GCA_905478165.1_Candidatus_Gamibacteraceae_bacterium
GCA_018659605.1_Candidatus_Diomedesiaceae_bacterium
GCA_016778805.1_Candidatus_Diomedesiaceae_bacterium
GCA_018659145.1_Candidatus_Diomedesiaceae_bacterium
GCA_016780625.1_Candidatus_Diomedesiaceae_bacterium
GCA_905480015.1_Candidatus_Deianiraeaceae_bacterium
GCA_017444125.1_Candidatus_Deianiraeaceae_bacterium
GCA_002394665.1_Candidatus_Deianiraeaceae_bacterium
Candidatus_Deianiraea_vastatrix_GCA_007993655.1
GCA_002787635.1_Candidatus_Athabascaceae_bacterium
GCA_002422795.1_Candidatus_Athabascaceae_bacterium
GCA_002725445.1_Candidatus_Mitibacteraceae_bacterium
GCA_002346425.1_Candidatus_Mitibacteraceae_bacterium
GCA_002422205.1_Candidatus_Mitibacteraceae_bacterium
GCA_003241645.1_Candidatus_Mitibacteraceae_bacterium
GCA_002422745.1_Candidatus_Mitibacteraceae_bacterium
GCA_002291925.1_Candidatus_Mitibacteraceae_bacterium
GCA_002291845.1_Candidatus_Mitibacteraceae_bacterium
GCA_004293935.1_Candidatus_Mitibacteraceae_bacterium
GCA_002423155.1_Candidatus_Mitibacteraceae_bacterium
GCA_013289445.1_Candidatus_Mitibacteraceae_bacterium
GCA_013288985.1_Candidatus_Mitibacteraceae_bacterium
GCA_013288335.1_Candidatus_Mitibacteraceae_bacterium
GCA_009926845.1_Candidatus_Mitibacteraceae_bacterium
GCA_005792635.1_Candidatus_Mitibacteraceae_bacterium
GCA_013288305.1_Candidatus_Mitibacteraceae_bacterium
GCA_013288575.1_Candidatus_Arkhamiaceae_bacterium
GCA_002787615.1_Candidatus_Arkhamiaceae_bacterium
GCA_013288565.1_Candidatus_Jistubacteraceae_bacterium
GCA_010031735.1_Candidatus_Jistubacteraceae_bacterium
GCA_903821695.1_Candidatus_Jistubacteraceae_bacterium
Candidatus_Neoehrlichia_lotoris_strain_RAC413_GCA_000964795.1
Anaplasma_marginale_strain_Florida_GCA_000020305.1
GCA_003531345.1_Candidatus_Jistubacteraceae_bacterium
Magnetofaba_australis_GCA_002109495.1
Magnetococcus_marinus_GCA_000014865.1
Ralstonia_pickettii_GCA_902374465.1
Burkholderia_cepacia_GCA_009586235.1
Methylotenera_versatilis_GCA_000093025.1
Nitrosospira_briensis_GCA_000619905.2
Dasania_marina_GCA_000373485.1
Congregibacter_litoralis_GCA_000153125.2
Enterobacter_cloacae_GCA_000770155.1
Alteromonas_macleodii_GCA_000172635.2

Ile_Val_Leu_z_0.10

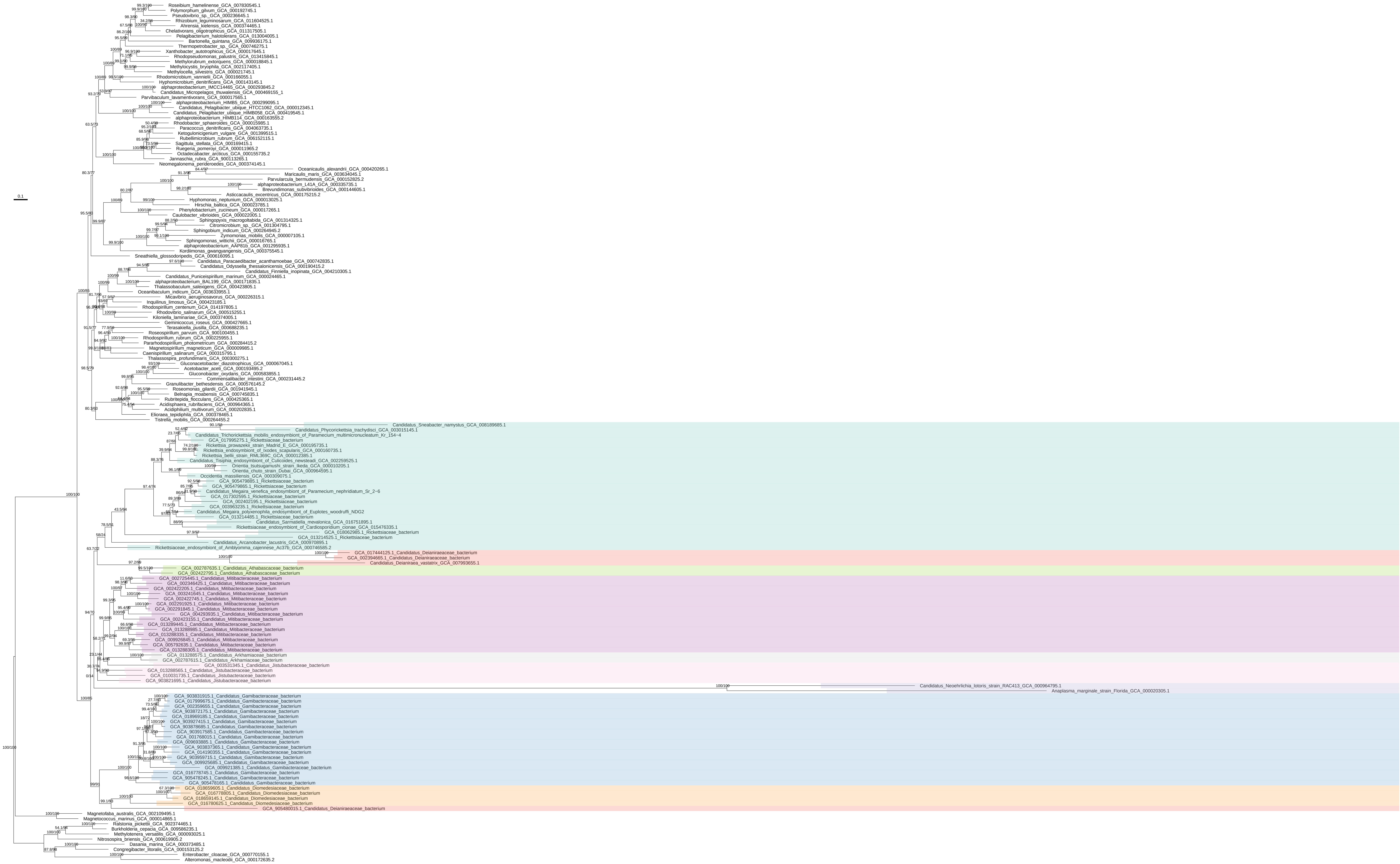

Ile_Val_Leu_z_0.20

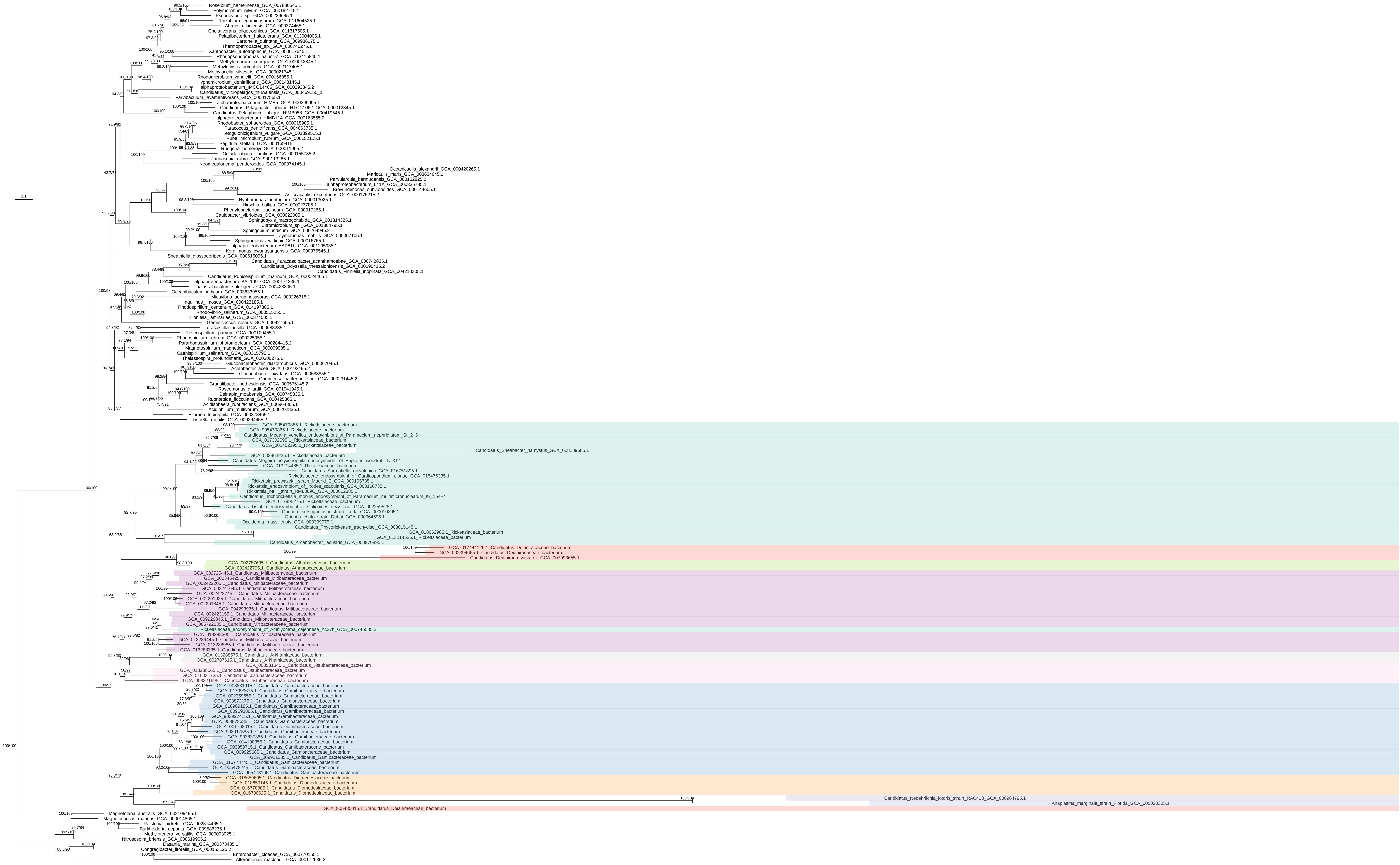

Ile_Val_Leu_z_0.30

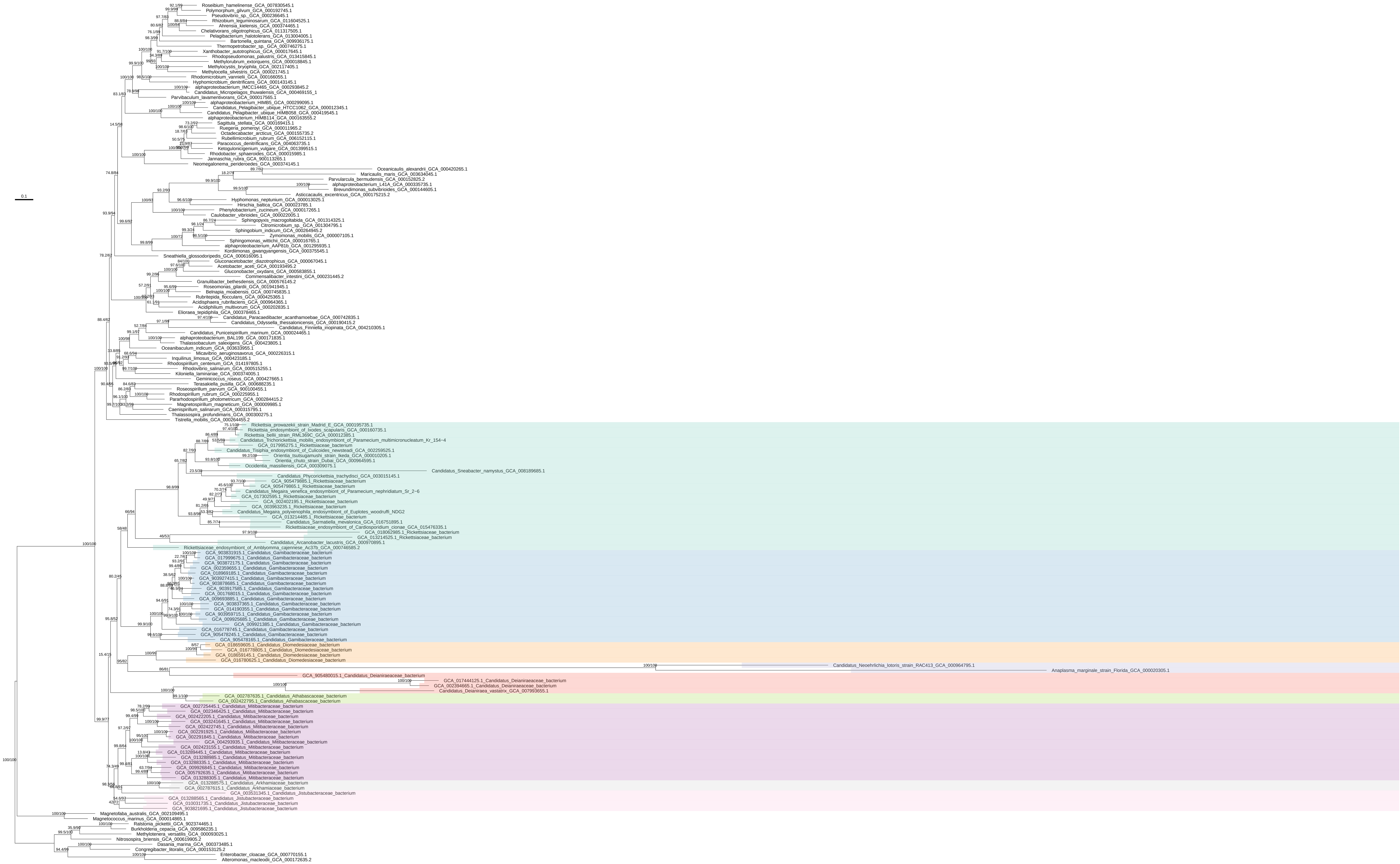

Ile_Val_Leu_z_0.40

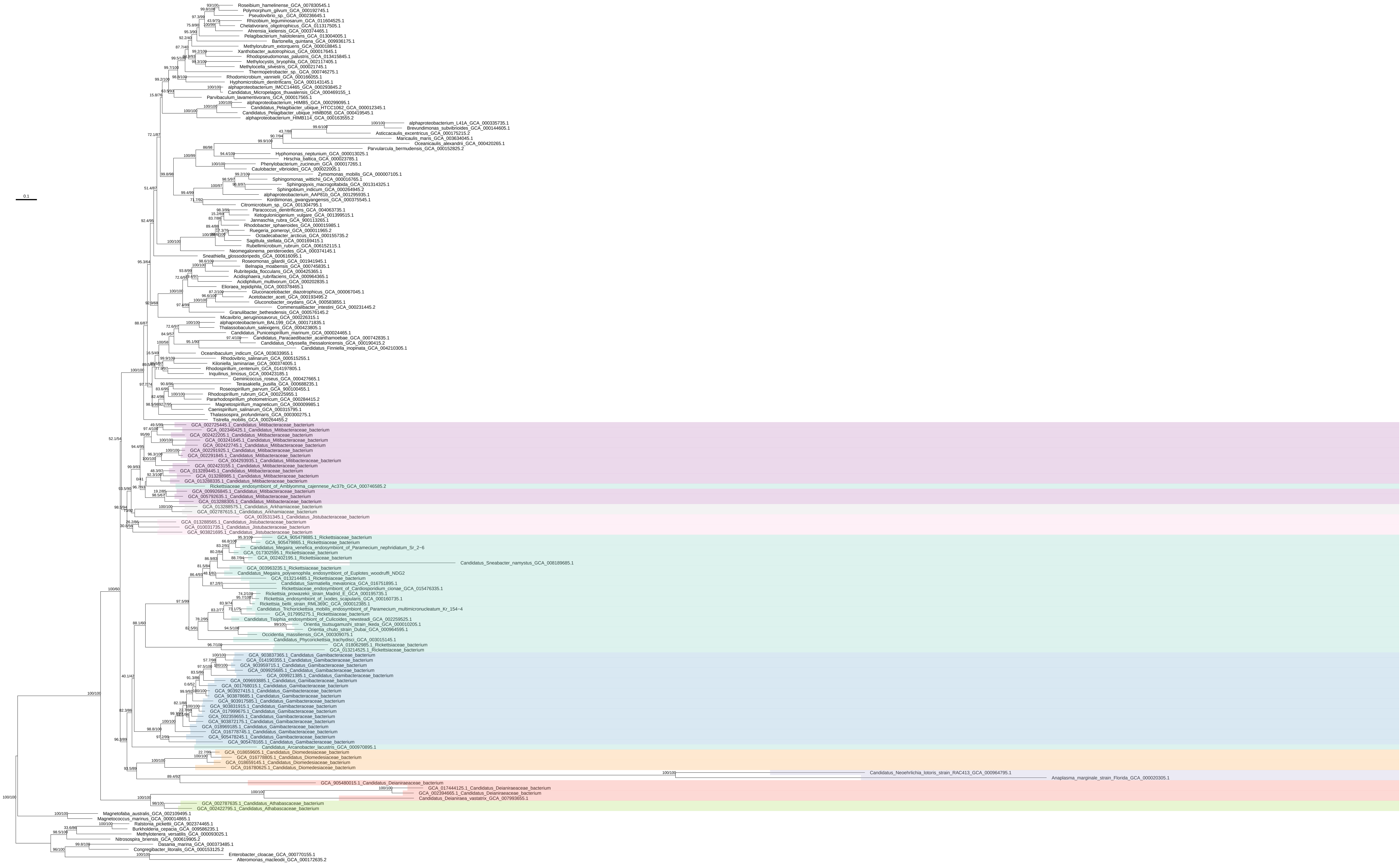

Ile_Val_Leu_z_0.50

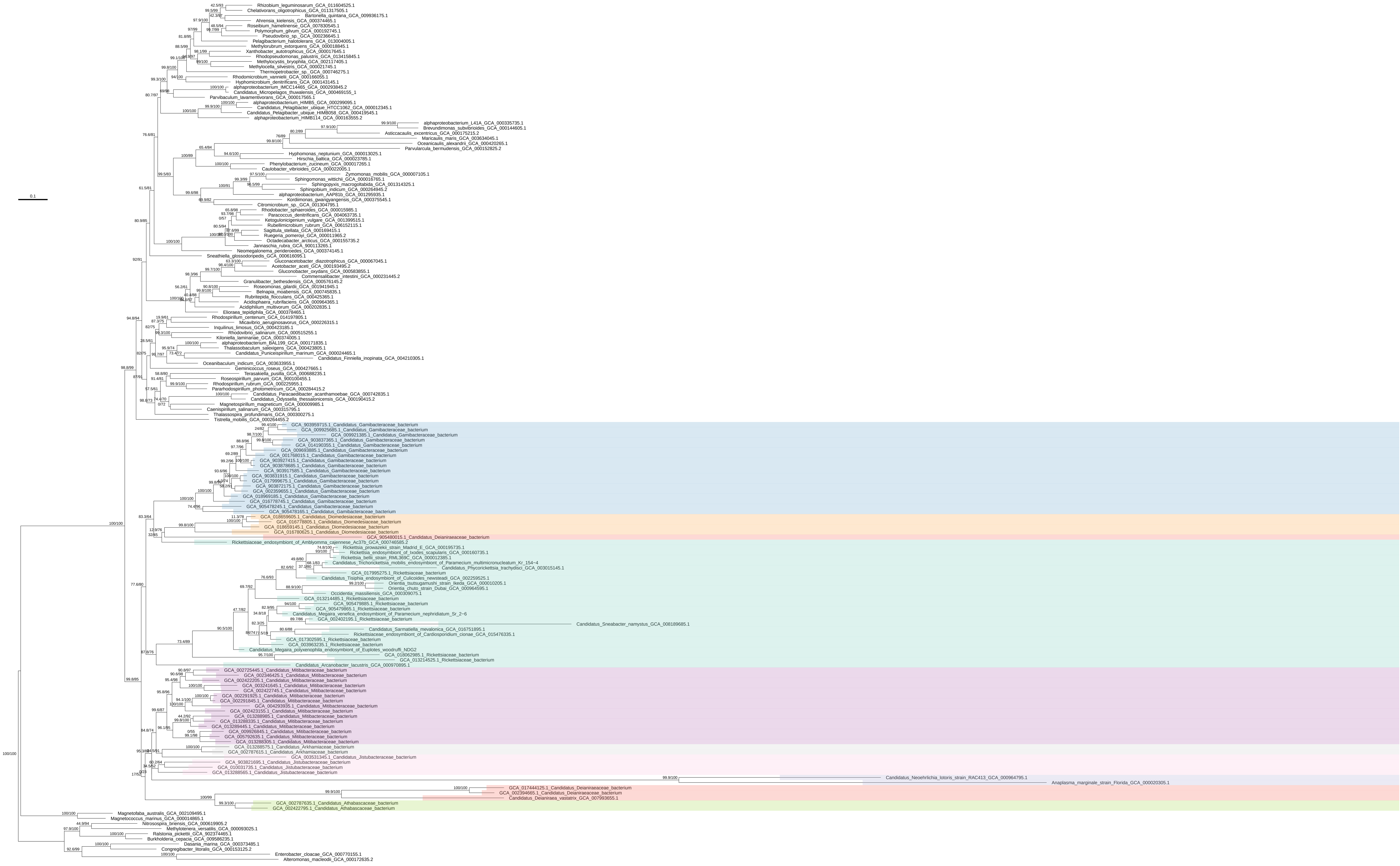

Lys_Thr_Met

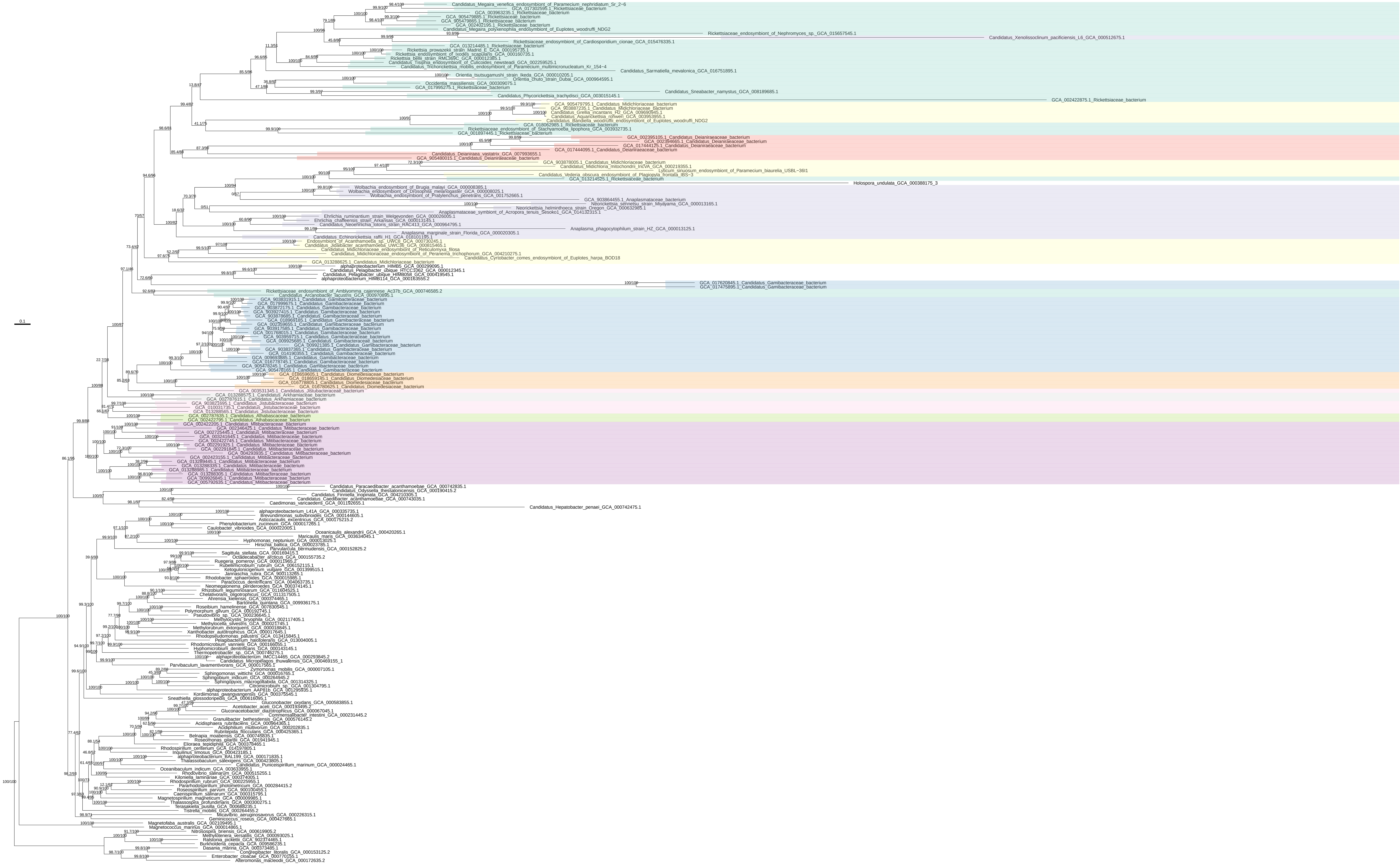

Lys_Thr_Met_z_0.10

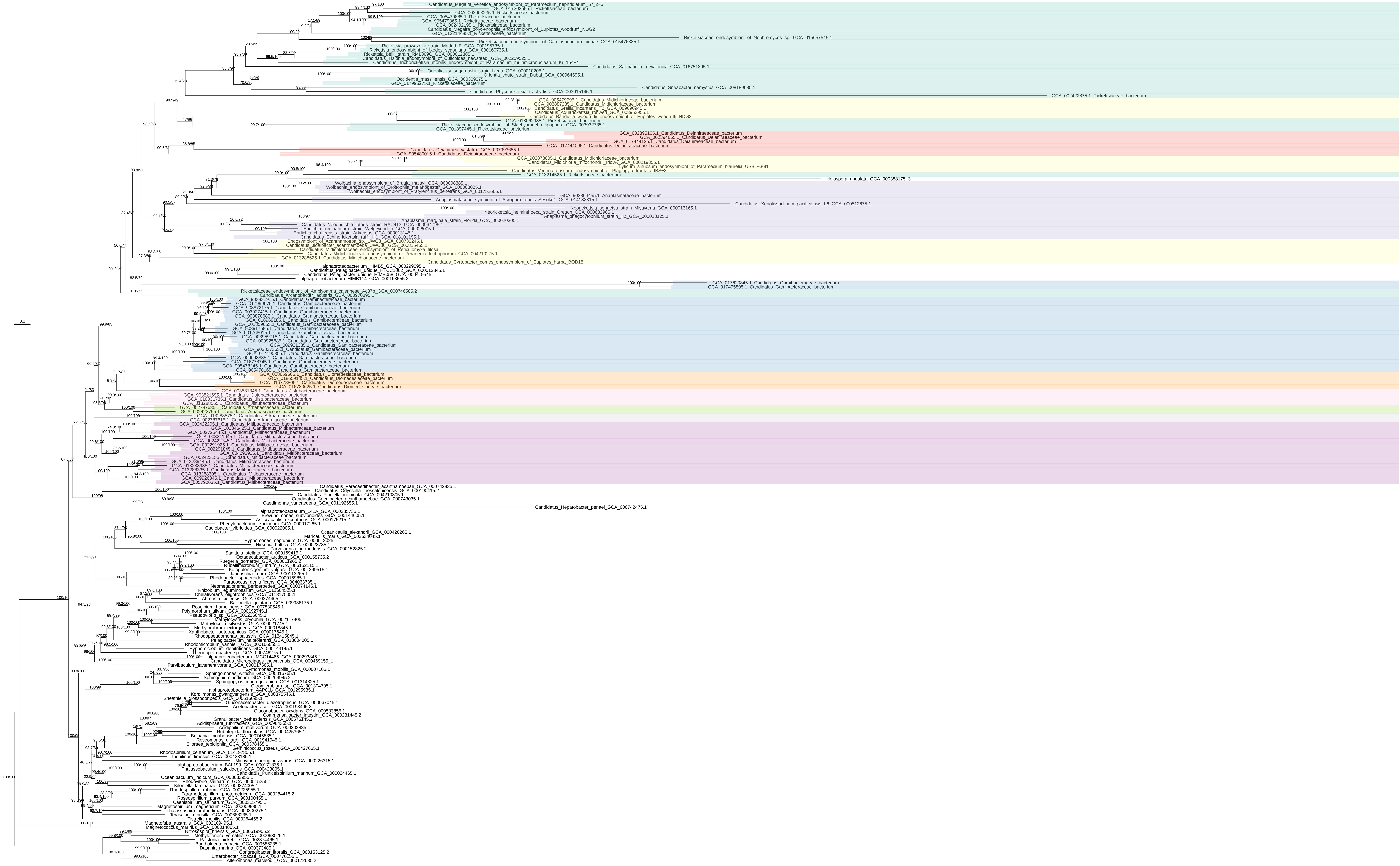

Lys_Thr_Met_z_0.20

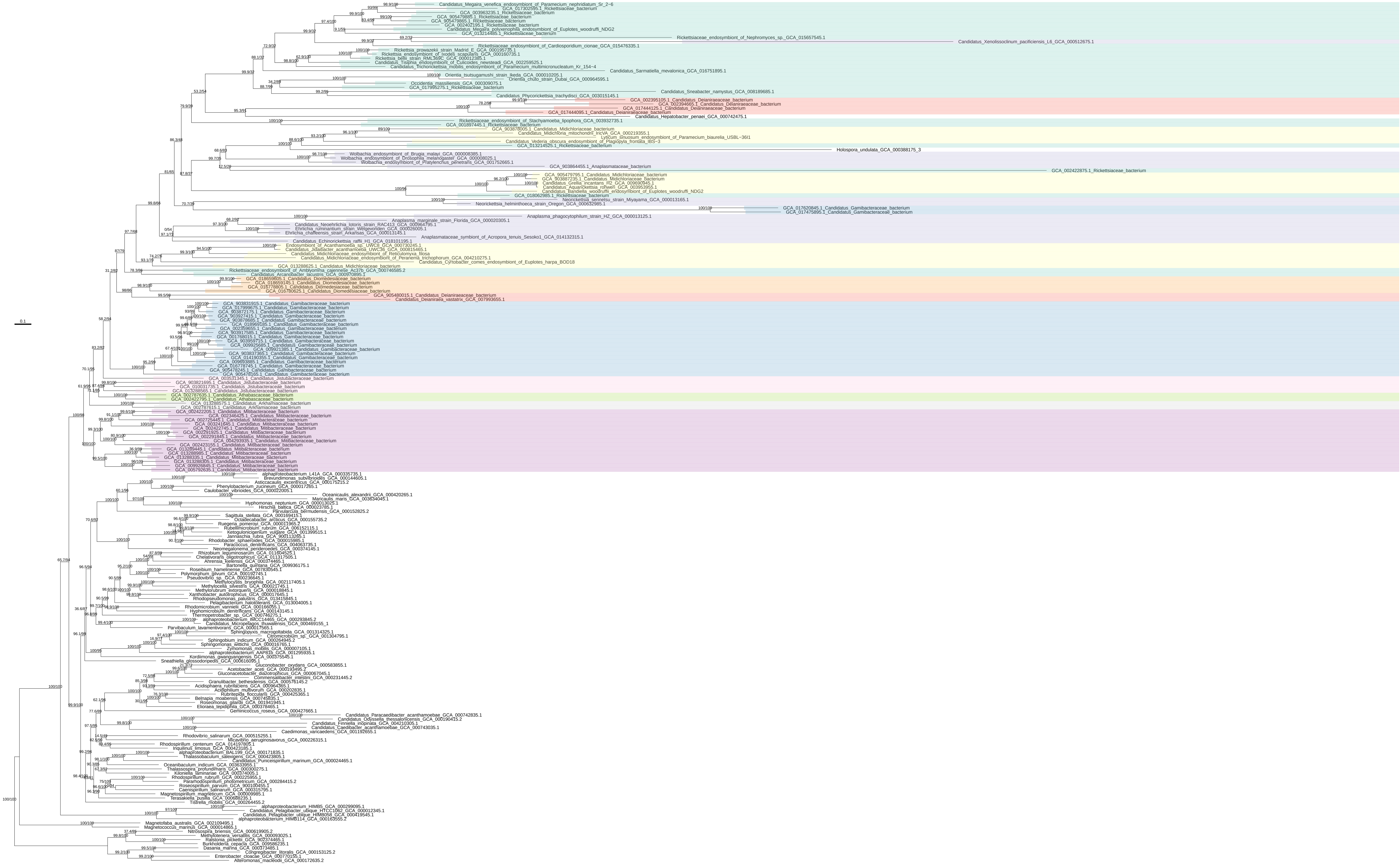

Lys_Thr_Met_z_0.30

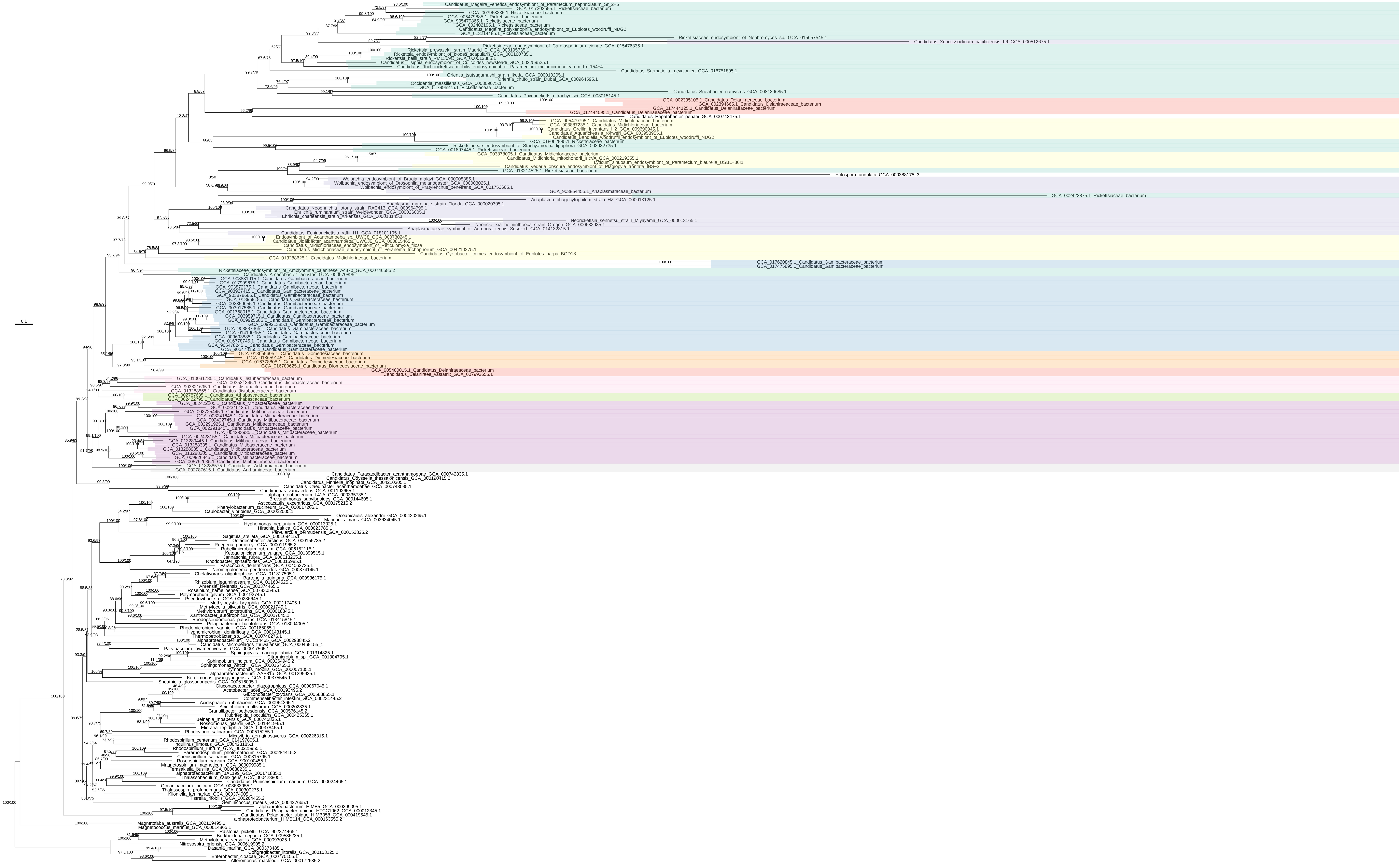

Lys_Thr_Met_z_0.40

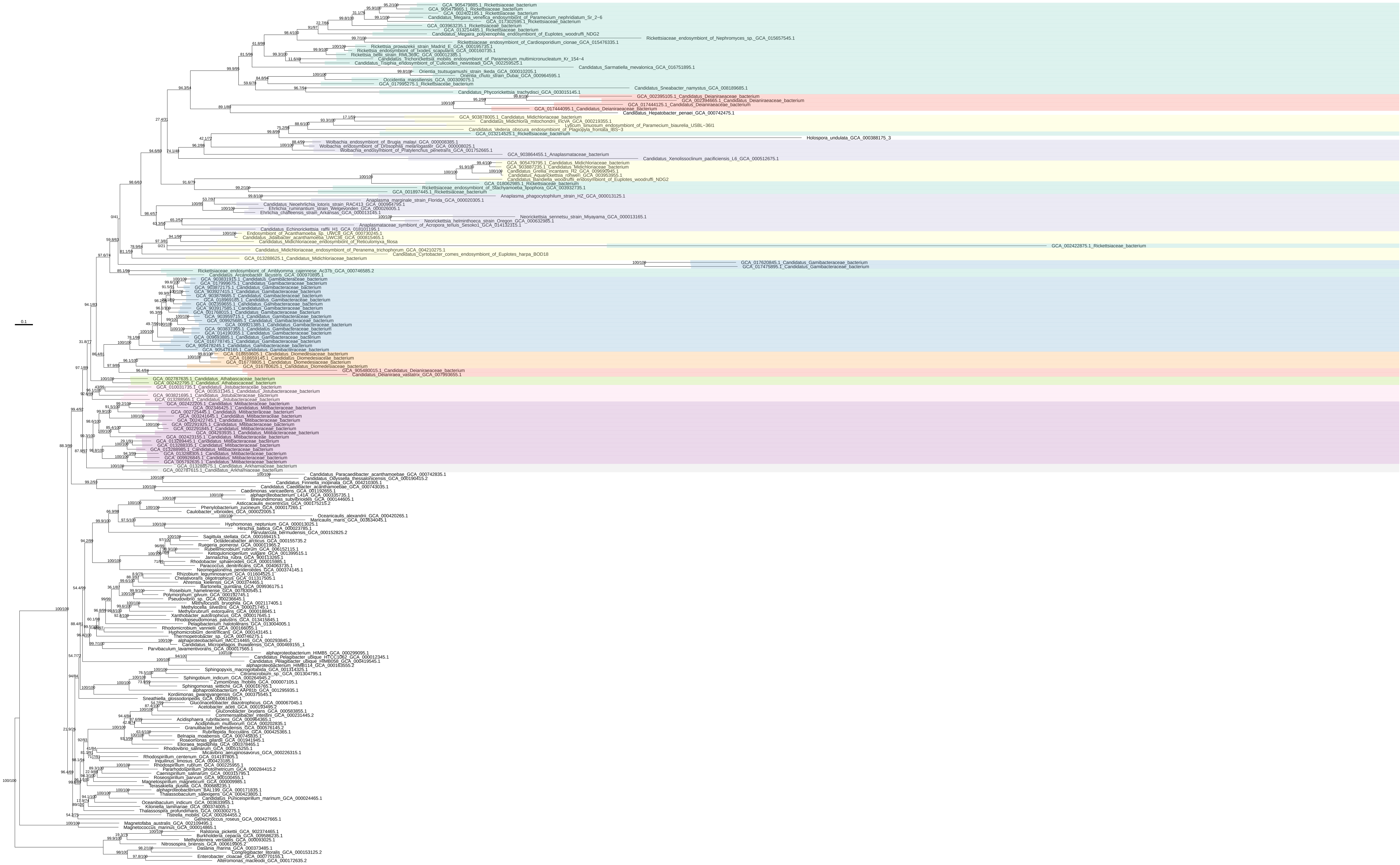

Lys_Thr_Met_z_0.50

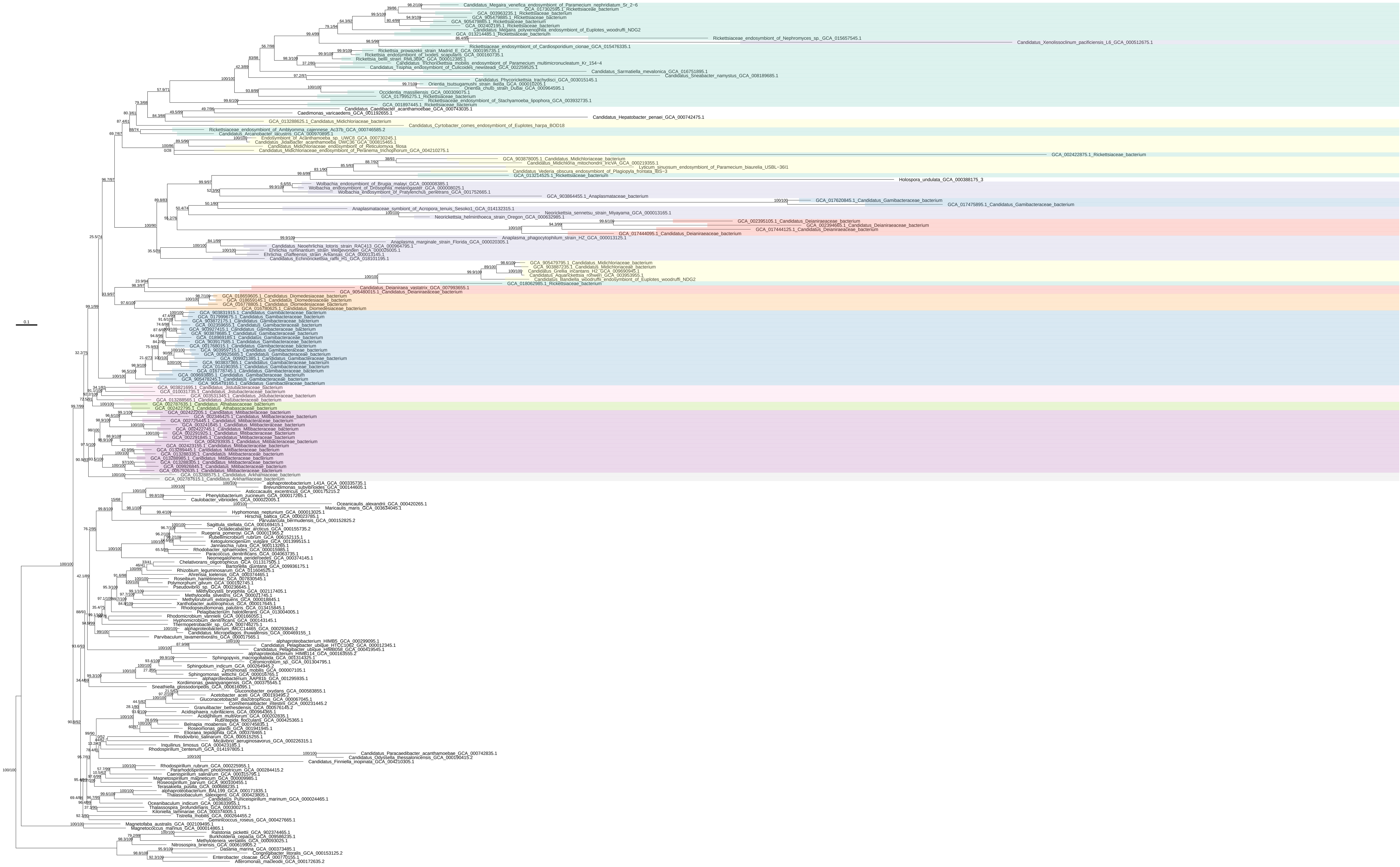

Pro

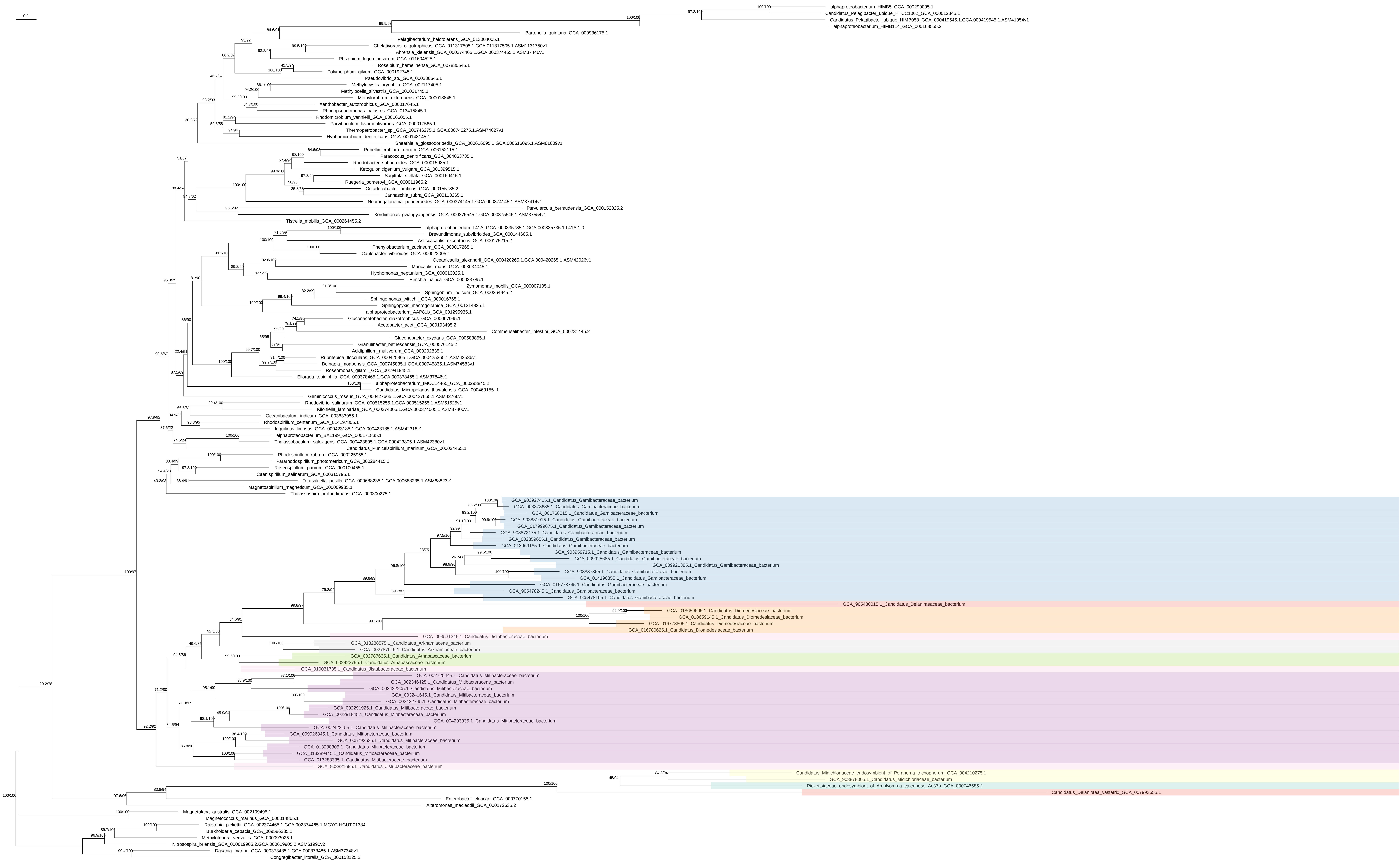
0.1
alphaproteobacterium_HIMB5_GCA_000299095.1
Candidatus_Pelagibacter_ubique_HTCC1062_GCA_000012345.1
Candidatus_Pelagibacter_ubique_HIMB058_GCA_000419545.1.GCA.000419545.1.ASM41954v1
alphaproteobacterium_HIMB114_GCA_000163555.2
Bartonella_quintana_GCA_009936175.1
Pelagibacterium_halotolerans_GCA_013004005.1
Chelativorans_oligotrophicus_GCA_011317505.1.GCA.011317505.1.ASM1131750v1
Ahrensia_kielensis_GCA_000374465.1.GCA.000374465.1.ASM37446v1
Rhizobium_leguminosarum_GCA_011604525.1
Roseibium_hamelinense_GCA_007830545.1
Polymorphum_gilvum_GCA_000192745.1
Pseudovibrio_sp._GCA_000236645.1
Methylocystis_bryophila_GCA_002117405.1
Methylocella_silvestris_GCA_000021745.1
Methylorubrum_extorquens_GCA_000018845.1
Xanthobacter_autotrophicus_GCA_000017645.1
Rhodopseudomonas_palustris_GCA_013415845.1
Rhodomicrobium_vannielii_GCA_000166055.1
Parvibaculum_lavamentivorans_GCA_000017565.1
Thermopetrobacter_sp._GCA_000746275.1.GCA.000746275.1.ASM74627v1
Hyphomicrobium_denitrificans_GCA_000143145.1
Sneathiella_glossodoripedis_GCA_000616095.1.GCA.000616095.1.ASM61609v1
Rubellimicrobium_rubrum_GCA_006152115.1
Paracoccus_denitrificans_GCA_004063735.1
Rhodobacter_sphaeroides_GCA_000015985.1
Ketogulonicigenium_vulgare_GCA_001399515.1
Sagittula_stellata_GCA_000169415.1
Ruegeria_pomeroyi_GCA_000011965.2
Octadecabacter_arcticus_GCA_000155735.2
Jannaschia_rubra_GCA_900113265.1
Neomegalonema_perideroedes_GCA_000374145.1.GCA.000374145.1.ASM37414v1
Parvularcula_bermudensis_GCA_000152825.2
Kordiimonas_gwangyangensis_GCA_000375545.1.GCA.000375545.1.ASM37554v1
Tistrella_mobilis_GCA_000264455.2
alphaproteobacterium_L41A_GCA_000335735.1.GCA.000335735.1.L41A.1.0
Brevundimonas_subvibrioides_GCA_000144605.1
Asticcacaulis_excentricus_GCA_000175215.2
Phenylobacterium_zucineum_GCA_000017265.1
Caulobacter_vibrioides_GCA_000022005.1
Oceanicaulis_alexandrii_GCA_000420265.1.GCA.000420265.1.ASM42026v1
Maricaulis_maris_GCA_003634045.1
Hyphomonas_neptunium_GCA_000013025.1
Hirschia_baltica_GCA_000023785.1
Zymomonas_mobilis_GCA_000007105.1
Sphingobium_indicum_GCA_000264945.2
Sphingomonas_wittichii_GCA_000016765.1
Sphingopyxis_macrogoltabida_GCA_001314325.1
alphaproteobacterium_AAP81b_GCA_001295935.1
Gluconacetobacter_diazotrophicus_GCA_000067045.1
Acetobacter_aceti_GCA_000193495.2
Commensalibacter_intestini_GCA_000231445.2
Gluconobacter_oxydans_GCA_000583855.1
Granulibacter_bethesdensis_GCA_000576145.2
Acidiphilium_multivorum_GCA_000202835.1
Rubritepida_flocculans_GCA_000425365.1.GCA.000425365.1.ASM42536v1
Belnapia_moabensis_GCA_000745835.1.GCA.000745835.1.ASM74583v1
Roseomonas_gilardii_GCA_001941945.1
Elioraea_tepidiphila_GCA_000378465.1.GCA.000378465.1.ASM37846v1
alphaproteobacterium_IMCC14465_GCA_000293845.2
Candidatus_Micropelagos_thuwalensis_GCA_000469155_1
Geminicoccus_roseus_GCA_000427665.1.GCA.000427665.1.ASM42766v1
Rhodovibrio_salinarum_GCA_000515255.1.GCA.000515255.1.ASM51525v1
Kiloniella_laminariae_GCA_000374005.1.GCA.000374005.1.ASM37400v1
Oceanibaculum_indicum_GCA_003633955.1
Rhodospirillum_centenum_GCA_014197805.1
Inquilinus_limosus_GCA_000423185.1.GCA.000423185.1.ASM42318v1
alphaproteobacterium_BAL199_GCA_000171835.1
Thalassobaculum_salexigens_GCA_000423805.1.GCA.000423805.1.ASM42380v1
Candidatus_Puniceispirillum_marinum_GCA_000024465.1
Rhodospirillum_rubrum_GCA_000225955.1
Pararhodospirillum_photometricum_GCA_000284415.2
Roseospirillum_parvum_GCA_900100455.1
Caenispirillum_salinarum_GCA_000315795.1
Terasakiella_pusilla_GCA_000688235.1.GCA.000688235.1.ASM68823v1
Magnetospirillum_magneticum_GCA_000009985.1
Thalassospira_profundimaris_GCA_000300275.1
GCA_903927415.1_Candidatus_Gamibacteraceae_bacterium
GCA_903878685.1_Candidatus_Gamibacteraceae_bacterium
GCA_001768015.1_Candidatus_Gamibacteraceae_bacterium
GCA_903831915.1_Candidatus_Gamibacteraceae_bacterium
GCA_017999675.1_Candidatus_Gamibacteraceae_bacterium
GCA_903872175.1_Candidatus_Gamibacteraceae_bacterium
GCA_002359655.1_Candidatus_Gamibacteraceae_bacterium
GCA_018969185.1_Candidatus_Gamibacteraceae_bacterium
GCA_903959715.1_Candidatus_Gamibacteraceae_bacterium
GCA_009925685.1_Candidatus_Gamibacteraceae_bacterium
GCA_009921385.1_Candidatus_Gamibacteraceae_bacterium
GCA_903837365.1_Candidatus_Gamibacteraceae_bacterium
GCA_014190355.1_Candidatus_Gamibacteraceae_bacterium
GCA_016778745.1_Candidatus_Gamibacteraceae_bacterium
GCA_905478245.1_Candidatus_Gamibacteraceae_bacterium
GCA_905478165.1_Candidatus_Gamibacteraceae_bacterium
GCA_905480015.1_Candidatus_Deianiraeaceae_bacterium
GCA_018659605.1_Candidatus_Diomedesiaceae_bacterium
GCA_018659145.1_Candidatus_Diomedesiaceae_bacterium
GCA_016778805.1_Candidatus_Diomedesiaceae_bacterium
GCA_016780625.1_Candidatus_Diomedesiaceae_bacterium
GCA_003531345.1_Candidatus_Jistubacteraceae_bacterium
GCA_013288575.1_Candidatus_Arkhamiaceae_bacterium
GCA_002787615.1_Candidatus_Arkhamiaceae_bacterium
GCA_002787635.1_Candidatus_Athabascaceae_bacterium
GCA_002422795.1_Candidatus_Athabascaceae_bacterium
GCA_010031735.1_Candidatus_Jistubacteraceae_bacterium
GCA_002725445.1_Candidatus_Mitibacteraceae_bacterium
GCA_002346425.1_Candidatus_Mitibacteraceae_bacterium
GCA_002422205.1_Candidatus_Mitibacteraceae_bacterium
GCA_003241645.1_Candidatus_Mitibacteraceae_bacterium
GCA_002422745.1_Candidatus_Mitibacteraceae_bacterium
GCA_002291925.1_Candidatus_Mitibacteraceae_bacterium
GCA_002291845.1_Candidatus_Mitibacteraceae_bacterium
GCA_004293935.1_Candidatus_Mitibacteraceae_bacterium
GCA_002423155.1_Candidatus_Mitibacteraceae_bacterium
GCA_009926845.1_Candidatus_Mitibacteraceae_bacterium
GCA_005792635.1_Candidatus_Mitibacteraceae_bacterium
GCA_013288305.1_Candidatus_Mitibacteraceae_bacterium
GCA_013289445.1_Candidatus_Mitibacteraceae_bacterium
GCA_013288335.1_Candidatus_Mitibacteraceae_bacterium
GCA_903821695.1_Candidatus_Jistubacteraceae_bacterium
Candidatus_Midichloriaceae_endosymbiont_of_Peranema_trichophorum_GCA_004210275.1
GCA_903878005.1_Candidatus_Midichloriaceae_bacterium
Rickettsiaceae_endosymbiont_of_Amblyomma_cajennese_Ac37b_GCA_000746585.2
Candidatus_Deianiraea_vastatrix_GCA_007993655.1
Enterobacter_cloacae_GCA_000770155.1
Alteromonas_macleodii_GCA_000172635.2
Magnetofaba_australis_GCA_002109495.1
Magnetococcus_marinus_GCA_000014865.1
Ralstonia_pickettii_GCA_902374465.1.GCA.902374465.1.MGYG.HGUT.01384
Burkholderia_cepacia_GCA_009586235.1
Methylotenera_versatilis_GCA_000093025.1
Nitrosospira_briensis_GCA_000619905.2.GCA.000619905.2.ASM61990v2
Dasania_marina_GCA_000373485.1.GCA.000373485.1.ASM37348v1
Congregibacter_litoralis_GCA_000153125.2

Pro_z_0.10

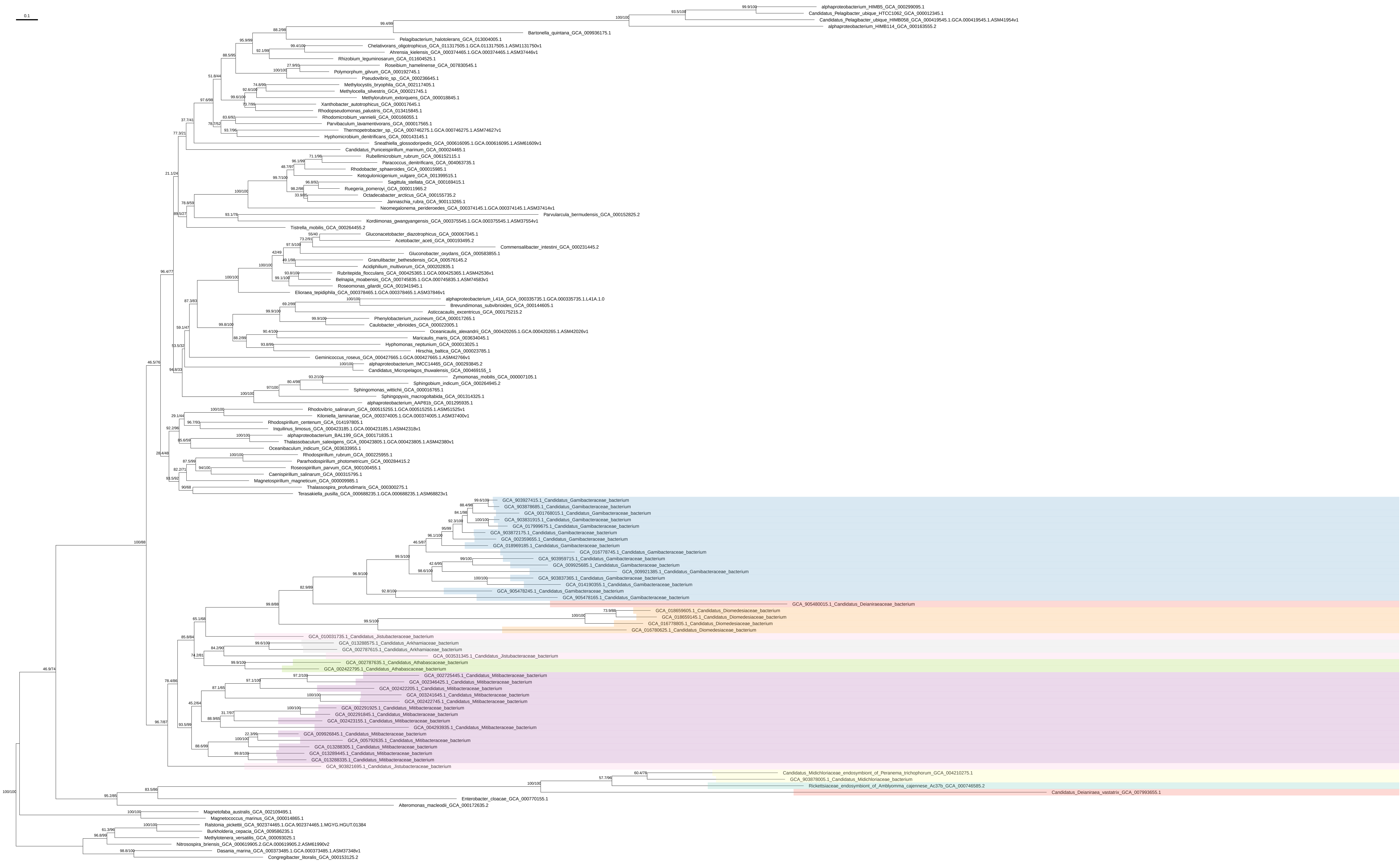

0.1
alphaproteobacterium_HIMB5_GCA_000299095.1
Candidatus_Pelagibacter_ubique_HTCC1062_GCA_000012345.1
Candidatus_Pelagibacter_ubique_HIMB058_GCA_000419545.1.GCA.000419545.1.ASM41954v1
alphaproteobacterium_HIMB114_GCA_000163555.2
Bartonella_quintana_GCA_009936175.1
Pelagibacterium_halotolerans_GCA_013004005.1
Chelativorans_oligotrophicus_GCA_011317505.1.GCA.011317505.1.ASM1131750v1
Ahrensia_kielensis_GCA_000374465.1.GCA.000374465.1.ASM37446v1
Rhizobium_leguminosarum_GCA_011604525.1
Roseibium_hamelinense_GCA_007830545.1
Polymorphum_gilvum_GCA_000192745.1
Pseudovibrio_sp._GCA_000236645.1
Methylocystis_bryophila_GCA_002117405.1
Methylocella_silvestris_GCA_000021745.1
Methylorubrum_extorquens_GCA_000018845.1
Xanthobacter_autotrophicus_GCA_000017645.1
Rhodopseudomonas_palustris_GCA_013415845.1
Rhodomicrobium_vannielii_GCA_000166055.1
Parvibaculum_lavamentivorans_GCA_000017565.1
Thermopetrobacter_sp._GCA_000746275.1.GCA.000746275.1.ASM74627v1
Hyphomicrobium_denitrificans_GCA_000143145.1
Sneathiella_glossodoripedis_GCA_000616095.1.GCA.000616095.1.ASM61609v1
Candidatus_Puniceispirillum_marinum_GCA_000024465.1
Rubellimicrobium_rubrum_GCA_006152115.1
Paracoccus_denitrificans_GCA_004063735.1
Rhodobacter_sphaeroides_GCA_000015985.1
Ketogulonicigenium_vulgare_GCA_001399515.1
Sagittula_stellata_GCA_000169415.1
Ruegeria_pomeroyi_GCA_000011965.2
Octadecabacter_arcticus_GCA_000155735.2
Jannaschia_rubra_GCA_900113265.1
Neomegalonema_perideroedes_GCA_000374145.1.GCA.000374145.1.ASM37414v1
Parvularcula_bermudensis_GCA_000152825.2
Kordiimonas_gwangyangensis_GCA_000375545.1.GCA.000375545.1.ASM37554v1
Tistrella_mobilis_GCA_000264455.2
Gluconacetobacter_diazotrophicus_GCA_000067045.1
Acetobacter_aceti_GCA_000193495.2
Commensalibacter_intestini_GCA_000231445.2
Gluconobacter_oxydans_GCA_000583855.1
Granulibacter_bethesdensis_GCA_000576145.2
Acidiphilium_multivorum_GCA_000202835.1
Rubritepida_flocculans_GCA_000425365.1.GCA.000425365.1.ASM42536v1
Belnapia_moabensis_GCA_000745835.1.GCA.000745835.1.ASM74583v1
Roseomonas_gilardii_GCA_001941945.1
Elioraea_tepidiphila_GCA_000378465.1.GCA.000378465.1.ASM37846v1
alphaproteobacterium_L41A_GCA_000335735.1.GCA.000335735.1.L41A.1.0
Brevundimonas_subvibrioides_GCA_000144605.1
Asticcacaulis_excentricus_GCA_000175215.2
Phenylobacterium_zucineum_GCA_000017265.1
Caulobacter_vibrioides_GCA_000022005.1
Oceanicaulis_alexandrii_GCA_000420265.1.GCA.000420265.1.ASM42026v1
Maricaulis_maris_GCA_003634045.1
Hyphomonas_neptunium_GCA_000013025.1
Hirschia_baltica_GCA_000023785.1
Geminicoccus_roseus_GCA_000427665.1.GCA.000427665.1.ASM42766v1
alphaproteobacterium_IMCC14465_GCA_000293845.2
Candidatus_Micropelagos_thuwalensis_GCA_000469155_1
Zymomonas_mobilis_GCA_000007105.1
Sphingobium_indicum_GCA_000264945.2
Sphingomonas_wittichii_GCA_000016765.1
Sphingopyxis_macrogoltabida_GCA_001314325.1
alphaproteobacterium_AAP81b_GCA_001295935.1
Rhodovibrio_salinarum_GCA_000515255.1.GCA.000515255.1.ASM51525v1
Kiloniella_laminariae_GCA_000374005.1.GCA.000374005.1.ASM37400v1
Rhodospirillum_centenum_GCA_014197805.1
Inquilinus_limosus_GCA_000423185.1.GCA.000423185.1.ASM42318v1
alphaproteobacterium_BAL199_GCA_000171835.1
Thalassobaculum_salexigens_GCA_000423805.1.GCA.000423805.1.ASM42380v1
Oceanibaculum_indicum_GCA_003633955.1
Rhodospirillum_rubrum_GCA_000225955.1
Pararhodospirillum_photometricum_GCA_000284415.2
Roseospirillum_parvum_GCA_900100455.1
Caenispirillum_salinarum_GCA_000315795.1
Magnetospirillum_magneticum_GCA_000009985.1
Thalassospira_profundimaris_GCA_000300275.1
Terasakiella_pusilla_GCA_000688235.1.GCA.000688235.1.ASM68823v1
GCA_903927415.1_Candidatus_Gamibacteraceae_bacterium
GCA_903878685.1_Candidatus_Gamibacteraceae_bacterium
GCA_001768015.1_Candidatus_Gamibacteraceae_bacterium
GCA_903831915.1_Candidatus_Gamibacteraceae_bacterium
GCA_017999675.1_Candidatus_Gamibacteraceae_bacterium
GCA_903872175.1_Candidatus_Gamibacteraceae_bacterium
GCA_002359655.1_Candidatus_Gamibacteraceae_bacterium
GCA_018969185.1_Candidatus_Gamibacteraceae_bacterium
GCA_016778745.1_Candidatus_Gamibacteraceae_bacterium
GCA_903959715.1_Candidatus_Gamibacteraceae_bacterium
GCA_009925685.1_Candidatus_Gamibacteraceae_bacterium
GCA_009921385.1_Candidatus_Gamibacteraceae_bacterium
GCA_903837365.1_Candidatus_Gamibacteraceae_bacterium
GCA_014190355.1_Candidatus_Gamibacteraceae_bacterium
GCA_905478245.1_Candidatus_Gamibacteraceae_bacterium
GCA_905478165.1_Candidatus_Gamibacteraceae_bacterium
GCA_905480015.1_Candidatus_Deianiraeaceae_bacterium
GCA_018659605.1_Candidatus_Diomedesiaceae_bacterium
GCA_018659145.1_Candidatus_Diomedesiaceae_bacterium
GCA_016778805.1_Candidatus_Diomedesiaceae_bacterium
GCA_016780625.1_Candidatus_Diomedesiaceae_bacterium
GCA_010031735.1_Candidatus_Jistubacteraceae_bacterium
GCA_013288575.1_Candidatus_Arkhamiaceae_bacterium
GCA_002787615.1_Candidatus_Arkhamiaceae_bacterium
GCA_003531345.1_Candidatus_Jistubacteraceae_bacterium
GCA_002787635.1_Candidatus_Athabascaceae_bacterium
GCA_002422795.1_Candidatus_Athabascaceae_bacterium
GCA_002725445.1_Candidatus_Mitibacteraceae_bacterium
GCA_002346425.1_Candidatus_Mitibacteraceae_bacterium
GCA_002422205.1_Candidatus_Mitibacteraceae_bacterium
GCA_003241645.1_Candidatus_Mitibacteraceae_bacterium
GCA_002422745.1_Candidatus_Mitibacteraceae_bacterium
GCA_002291925.1_Candidatus_Mitibacteraceae_bacterium
GCA_002291845.1_Candidatus_Mitibacteraceae_bacterium
GCA_002423155.1_Candidatus_Mitibacteraceae_bacterium
GCA_004293935.1_Candidatus_Mitibacteraceae_bacterium
GCA_009926845.1_Candidatus_Mitibacteraceae_bacterium
GCA_005792635.1_Candidatus_Mitibacteraceae_bacterium
GCA_013288305.1_Candidatus_Mitibacteraceae_bacterium
GCA_013289445.1_Candidatus_Mitibacteraceae_bacterium
GCA_013288335.1_Candidatus_Mitibacteraceae_bacterium
GCA_903821695.1_Candidatus_Jistubacteraceae_bacterium
Candidatus_Midichloriaceae_endosymbiont_of_Peranema_trichophorum_GCA_004210275.1
GCA_903878005.1_Candidatus_Midichloriaceae_bacterium
Rickettsiaceae_endosymbiont_of_Amblyomma_cajennense_Ac37b_GCA_000746585.2
Candidatus_Deianiraea_vastatrix_GCA_007993655.1
Enterobacter_cloacae_GCA_000770155.1
Alteromonas_macleodii_GCA_000172635.2
Magnetofaba_australis_GCA_002109495.1
Magnetococcus_marinus_GCA_000014865.1
Ralstonia_pickettii_GCA_902374465.1.GCA.902374465.1.MGYG.HGUT.01384
Burkholderia_cepacia_GCA_009586235.1
Methylotenera_versatilis_GCA_000093025.1
Nitrosospira_briensis_GCA_000619905.2.GCA.000619905.2.ASM61990v2
Dasania_marina_GCA_000373485.1.GCA.000373485.1.ASM37348v1
Congregibacter_litoralis_GCA_000153125.2

Pro_z_0.20

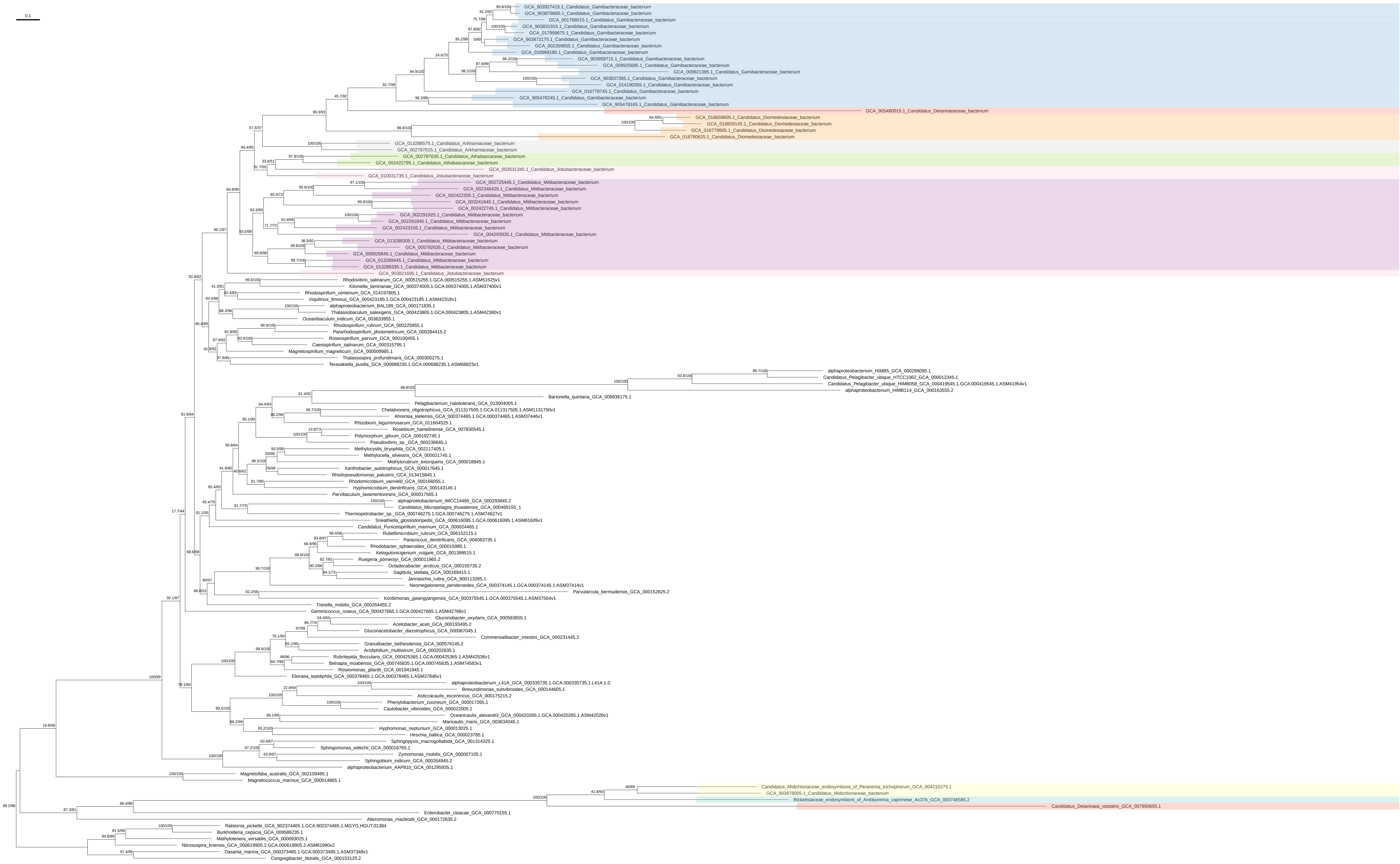
0.1
GCA_903927415.1_Candidatus_Gamibacteraceae_bacterium
GCA_903878685.1_Candidatus_Gamibacteraceae_bacterium
GCA_001768015.1_Candidatus_Gamibacteraceae_bacterium
GCA_903831915.1_Candidatus_Gamibacteraceae_bacterium
GCA_017999675.1_Candidatus_Gamibacteraceae_bacterium
GCA_903872175.1_Candidatus_Gamibacteraceae_bacterium
GCA_002359655.1_Candidatus_Gamibacteraceae_bacterium
GCA_018969185.1_Candidatus_Gamibacteraceae_bacterium
GCA_903959715.1_Candidatus_Gamibacteraceae_bacterium
GCA_009925685.1_Candidatus_Gamibacteraceae_bacterium
GCA_009921385.1_Candidatus_Gamibacteraceae_bacterium
GCA_903837365.1_Candidatus_Gamibacteraceae_bacterium
GCA_014190355.1_Candidatus_Gamibacteraceae_bacterium
GCA_016778745.1_Candidatus_Gamibacteraceae_bacterium
GCA_905478245.1_Candidatus_Gamibacteraceae_bacterium
GCA_905478165.1_Candidatus_Gamibacteraceae_bacterium
GCA_905480015.1_Candidatus_Deianiraeaceae_bacterium
GCA_018659605.1_Candidatus_Diomedesiaceae_bacterium
GCA_018659145.1_Candidatus_Diomedesiaceae_bacterium
GCA_016778805.1_Candidatus_Diomedesiaceae_bacterium
GCA_016780625.1_Candidatus_Diomedesiaceae_bacterium
GCA_013288575.1_Candidatus_Arkhamiaceae_bacterium
GCA_002787615.1_Candidatus_Arkhamiaceae_bacterium
GCA_002787635.1_Candidatus_Athabascaceae_bacterium
GCA_002422795.1_Candidatus_Athabascaceae_bacterium
GCA_003531345.1_Candidatus_Jistubacteraceae_bacterium
GCA_010031735.1_Candidatus_Jistubacteraceae_bacterium
GCA_002725445.1_Candidatus_Mitibacteraceae_bacterium
GCA_002346425.1_Candidatus_Mitibacteraceae_bacterium
GCA_002422205.1_Candidatus_Mitibacteraceae_bacterium
GCA_003241645.1_Candidatus_Mitibacteraceae_bacterium
GCA_002422745.1_Candidatus_Mitibacteraceae_bacterium
GCA_002291925.1_Candidatus_Mitibacteraceae_bacterium
GCA_002291845.1_Candidatus_Mitibacteraceae_bacterium
GCA_002423155.1_Candidatus_Mitibacteraceae_bacterium
GCA_004293935.1_Candidatus_Mitibacteraceae_bacterium
GCA_013288305.1_Candidatus_Mitibacteraceae_bacterium
GCA_005792635.1_Candidatus_Mitibacteraceae_bacterium
GCA_009926845.1_Candidatus_Mitibacteraceae_bacterium
GCA_013289445.1_Candidatus_Mitibacteraceae_bacterium
GCA_013288335.1_Candidatus_Mitibacteraceae_bacterium
GCA_903821695.1_Candidatus_Jistubacteraceae_bacterium
Rhodovibrio_salinarum_GCA_000515255.1.GCA.000515255.1.ASM51525v1
Kiloniella_laminariae_GCA_000374005.1.GCA.000374005.1.ASM37400v1
Rhodospirillum_centenum_GCA_014197805.1
Inquilinus_limosus_GCA_000423185.1.GCA.000423185.1.ASM42318v1
alphaproteobacterium_BAL199_GCA_000171835.1
Thalassobaculum_salexigens_GCA_000423805.1.GCA.000423805.1.ASM42380v1
Oceanibaculum_indicum_GCA_003633955.1
Rhodospirillum_rubrum_GCA_000225955.1
Pararhodospirillum_photometricum_GCA_000284415.2
Roseospirillum_parvum_GCA_900100455.1
Caenispirillum_salinarum_GCA_000315795.1
Magnetospirillum_magneticum_GCA_000009985.1
Thalassospira_profundimaris_GCA_000300275.1
Terasakiella_pusilla_GCA_000688235.1.GCA.000688235.1.ASM68823v1
alphaproteobacterium_HIMB5_GCA_000299095.1
Candidatus_Pelagibacter_ubique_HTCC1062_GCA_000012345.1
Candidatus_Pelagibacter_ubique_HIMB058_GCA_000419545.1.GCA.000419545.1.ASM41954v1
alphaproteobacterium_HIMB114_GCA_000163555.2
Bartonella_quintana_GCA_009936175.1
Pelagibacterium_halotolerans_GCA_013004005.1
Chelativorans_oligotrophicus_GCA_011317505.1.GCA.011317505.1.ASM1131750v1
Ahrensia_kielensis_GCA_000374465.1.GCA.000374465.1.ASM37446v1
Rhizobium_leguminosarum_GCA_011604525.1
Roseibium_hamelinense_GCA_007830545.1
Polymorphum_gilvum_GCA_000192745.1
Pseudovibrio_sp._GCA_000236645.1
Methylocystis_bryophila_GCA_002117405.1
Methylocella_silvestris_GCA_000021745.1
Methylorubrum_extorquens_GCA_000018845.1
Xanthobacter_autotrophicus_GCA_000017645.1
Rhodopseudomonas_palustris_GCA_013415845.1
Rhodomicrobium_vannielii_GCA_000166055.1
Hyphomicrobium_denitrificans_GCA_000143145.1
Parvibaculum_lavamentivorans_GCA_000017565.1
alphaproteobacterium_IMCC14465_GCA_000293845.2
Candidatus_Micropelagos_thuwalensis_GCA_000469155_1
Thermopetrobacter_sp._GCA_000746275.1.GCA.000746275.1.ASM74627v1
Sneathiella_glossodoripedis_GCA_000616095.1.GCA.000616095.1.ASM61609v1
Candidatus_Puniceispirillum_marinum_GCA_000024465.1
Rubellimicrobium_rubrum_GCA_006152115.1
Paracoccus_denitrificans_GCA_004063735.1
Rhodobacter_sphaeroides_GCA_000015985.1
Ketogulonicigenium_vulgare_GCA_001399515.1
Ruegeria_pomeroyi_GCA_000011965.2
Octadecabacter_arcticus_GCA_000155735.2
Sagittula_stellata_GCA_000169415.1
Jannaschia_rubra_GCA_900113265.1
Neomegalonema_perideroedes_GCA_000374145.1.GCA.000374145.1.ASM37414v1
Parvularcula_bermudensis_GCA_000152825.2
Kordiimonas_gwangyangensis_GCA_000375545.1.GCA.000375545.1.ASM37554v1
Tistrella_mobilis_GCA_000264455.2
Geminicoccus_roseus_GCA_000427665.1.GCA.000427665.1.ASM42766v1
Gluconobacter_oxydans_GCA_000583855.1
Acetobacter_aceti_GCA_000193495.2
Gluconacetobacter_diazotrophicus_GCA_000067045.1
Commensalibacter_intestini_GCA_000231445.2
Granulibacter_bethesdensis_GCA_000576145.2
Acidiphilium_multivorum_GCA_000202835.1
Rubritepida_flocculans_GCA_000425365.1.GCA.000425365.1.ASM42536v1
Belnapia_moabensis_GCA_000745835.1.GCA.000745835.1.ASM74583v1
Roseomonas_gilardii_GCA_001941945.1
Elioraea_tepidiphila_GCA_000378465.1.GCA.000378465.1.ASM37846v1
alphaproteobacterium_L41A_GCA_000335735.1.GCA.000335735.1.L41A.1.0
Brevundimonas_subvibrioides_GCA_000144605.1
Asticcacaulis_excentricus_GCA_000175215.2
Phenylobacterium_zucineum_GCA_000017265.1
Caulobacter_vibrioides_GCA_000022005.1
Oceanicaulis_alexandrii_GCA_000420265.1.GCA.000420265.1.ASM42026v1
Maricaulis_maris_GCA_003634045.1
Hyphomonas_neptunium_GCA_000013025.1
Hirschia_baltica_GCA_000023785.1
Sphingopyxis_macrogoltabida_GCA_001314325.1
Sphingomonas_wittichii_GCA_000016765.1
Zymomonas_mobilis_GCA_000007105.1
Sphingobium_indicum_GCA_000264945.2
alphaproteobacterium_AAP81b_GCA_001295935.1
Magnetofaba_australis_GCA_002109495.1
Magnetococcus_marinus_GCA_000014865.1
Candidatus_Midichloriaceae_endosymbiont_of_Peranema_trichophorum_GCA_004210275.1
GCA_903878005.1_Candidatus_Midichloriaceae_bacterium
Rickettsiaceae_endosymbiont_of_Amblyomma_cajennese_Ac37b_GCA_000746585.2
Candidatus_Deianiraea_vastatrix_GCA_007993655.1
Enterobacter_cloacae_GCA_000770155.1
Alteromonas_macleodii_GCA_000172635.2
Ralstonia_pickettii_GCA_902374465.1.GCA.902374465.1.MGYG.HGUT.01384
Burkholderia_cepacia_GCA_009586235.1
Methylotenera_versatilis_GCA_000093025.1
Nitrosospira_briensis_GCA_000619905.2.GCA.000619905.2.ASM61990v2
Dasania_marina_GCA_000373485.1.GCA.000373485.1.ASM37348v1
Congregibacter_litoralis_GCA_000153125.2

Pro_z_0.30

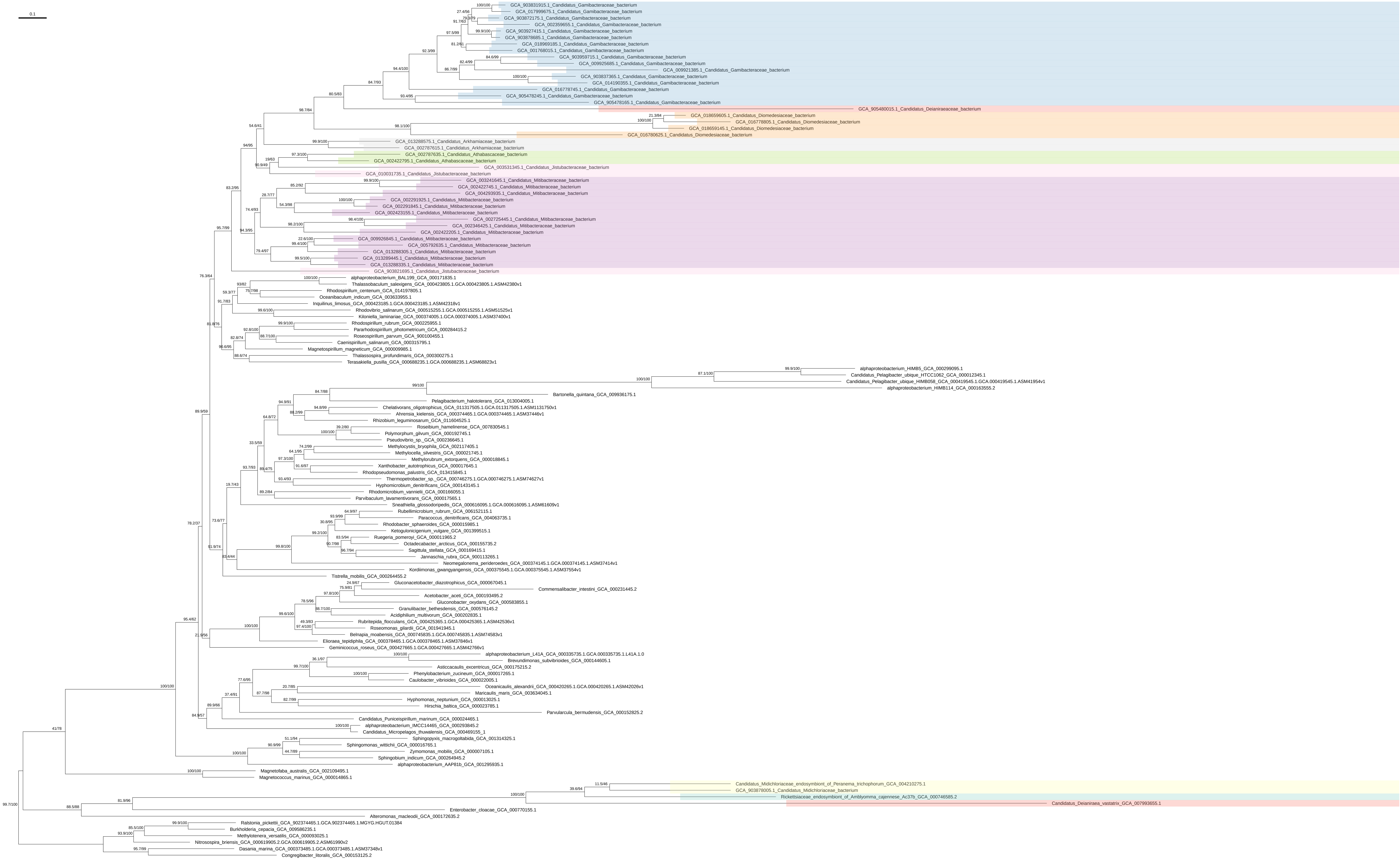

0.1
GCA_903831915.1_Candidatus_Gamibacteraceae_bacterium
GCA_017999675.1_Candidatus_Gamibacteraceae_bacterium
GCA_903872175.1_Candidatus_Gamibacteraceae_bacterium
GCA_002359655.1_Candidatus_Gamibacteraceae_bacterium
GCA_903927415.1_Candidatus_Gamibacteraceae_bacterium
GCA_903878685.1_Candidatus_Gamibacteraceae_bacterium
GCA_018969185.1_Candidatus_Gamibacteraceae_bacterium
GCA_001768015.1_Candidatus_Gamibacteraceae_bacterium
GCA_903959715.1_Candidatus_Gamibacteraceae_bacterium
GCA_009925685.1_Candidatus_Gamibacteraceae_bacterium
GCA_009921385.1_Candidatus_Gamibacteraceae_bacterium
GCA_903837365.1_Candidatus_Gamibacteraceae_bacterium
GCA_014190355.1_Candidatus_Gamibacteraceae_bacterium
GCA_016778745.1_Candidatus_Gamibacteraceae_bacterium
GCA_905478245.1_Candidatus_Gamibacteraceae_bacterium
GCA_905478165.1_Candidatus_Gamibacteraceae_bacterium
GCA_905480015.1_Candidatus_Deianiraeaceae_bacterium
GCA_018659605.1_Candidatus_Diomedesiaceae_bacterium
GCA_016778805.1_Candidatus_Diomedesiaceae_bacterium
GCA_018659145.1_Candidatus_Diomedesiaceae_bacterium
GCA_016780625.1_Candidatus_Diomedesiaceae_bacterium
GCA_013288575.1_Candidatus_Arkhamiaceae_bacterium
GCA_002787615.1_Candidatus_Arkhamiaceae_bacterium
GCA_002787635.1_Candidatus_Athabascaceae_bacterium
GCA_002422795.1_Candidatus_Athabascaceae_bacterium
GCA_003531345.1_Candidatus_Jistubacteraceae_bacterium
GCA_010031735.1_Candidatus_Jistubacteraceae_bacterium
GCA_003241645.1_Candidatus_Mitibacteraceae_bacterium
GCA_002422745.1_Candidatus_Mitibacteraceae_bacterium
GCA_004293935.1_Candidatus_Mitibacteraceae_bacterium
GCA_002291925.1_Candidatus_Mitibacteraceae_bacterium
GCA_002291845.1_Candidatus_Mitibacteraceae_bacterium
GCA_002423155.1_Candidatus_Mitibacteraceae_bacterium
GCA_002725445.1_Candidatus_Mitibacteraceae_bacterium
GCA_002346425.1_Candidatus_Mitibacteraceae_bacterium
GCA_002422205.1_Candidatus_Mitibacteraceae_bacterium
GCA_009926845.1_Candidatus_Mitibacteraceae_bacterium
GCA_005792635.1_Candidatus_Mitibacteraceae_bacterium
GCA_013288305.1_Candidatus_Mitibacteraceae_bacterium
GCA_013289445.1_Candidatus_Mitibacteraceae_bacterium
GCA_013288335.1_Candidatus_Mitibacteraceae_bacterium
GCA_903821695.1_Candidatus_Jistubacteraceae_bacterium
alphaproteobacterium_BAL199_GCA_000171835.1
Thalassobaculum_salexigens_GCA_000423805.1.GCA.000423805.1.ASM42380v1
Rhodospirillum_centenum_GCA_014197805.1
Oceanibaculum_indicum_GCA_003633955.1
Inquilinus_limosus_GCA_000423185.1.GCA.000423185.1.ASM42318v1
Rhodovibrio_salinarum_GCA_000515255.1.GCA.000515255.1.ASM51525v1
Kiloniella_laminariae_GCA_000374005.1.GCA.000374005.1.ASM37400v1
Rhodospirillum_rubrum_GCA_000225955.1
Pararhodospirillum_photometricum_GCA_000284415.2
Roseospirillum_parvum_GCA_900100455.1
Caenispirillum_salinarum_GCA_000315795.1
Magnetospirillum_magneticum_GCA_000009985.1
Thalassospira_profundimaris_GCA_000300275.1
Terasakiella_pusilla_GCA_000688235.1.GCA.000688235.1.ASM68823v1
alphaproteobacterium_HIMB5_GCA_000299095.1
Candidatus_Pelagibacter_ubique_HTCC1062_GCA_000012345.1
Candidatus_Pelagibacter_ubique_HIMB058_GCA_000419545.1.GCA.000419545.1.ASM41954v1
alphaproteobacterium_HIMB114_GCA_000163555.2
Bartonella_quintana_GCA_009936175.1
Pelagibacterium_halotolerans_GCA_013004005.1
Chelativorans_oligotrophicus_GCA_011317505.1.GCA.011317505.1.ASM1131750v1
Ahrensia_kielensis_GCA_000374465.1.GCA.000374465.1.ASM37446v1
Rhizobium_leguminosarum_GCA_011604525.1
Roseibium_hamelinense_GCA_007830545.1
Polymorphum_gilvum_GCA_000192745.1
Pseudovibrio_sp._GCA_000236645.1
Methylocystis_bryophila_GCA_002117405.1
Methylocella_silvestris_GCA_000021745.1
Methylorubrum_extorquens_GCA_000018845.1
Xanthobacter_autotrophicus_GCA_000017645.1
Rhodopseudomonas_palustris_GCA_013415845.1
Thermopetrobacter_sp._GCA_000746275.1.GCA.000746275.1.ASM74627v1
Hyphomicrobium_denitrificans_GCA_000143145.1
Rhodomicrobium_vannielii_GCA_000166055.1
Parvibaculum_lavamentivorans_GCA_000017565.1
Sneathiella_glossodoripedis_GCA_000616095.1.GCA.000616095.1.ASM61609v1
Rubellimicrobium_rubrum_GCA_006152115.1
Paracoccus_denitrificans_GCA_004063735.1
Rhodobacter_sphaeroides_GCA_000015985.1
Ketogulonicigenium_vulgare_GCA_001399515.1
Ruegeria_pomeroyi_GCA_000011965.2
Octadecabacter_arcticus_GCA_000155735.2
Sagittula_stellata_GCA_000169415.1
Jannaschia_rubra_GCA_900113265.1
Neomegalonema_perideroedes_GCA_000374145.1.GCA.000374145.1.ASM37414v1
Kordiimonas_gwangyangensis_GCA_000375545.1.GCA.000375545.1.ASM37554v1
Tistrella_mobilis_GCA_000264455.2
Gluconacetobacter_diazotrophicus_GCA_000067045.1
Commensalibacter_intestini_GCA_000231445.2
Acetobacter_aceti_GCA_000193495.2
Gluconobacter_oxydans_GCA_000583855.1
Granulibacter_bethesdensis_GCA_000576145.2
Acidiphilium_multivorum_GCA_000202835.1
Rubritepida_flocculans_GCA_000425365.1.GCA.000425365.1.ASM42536v1
Roseomonas_gilardii_GCA_001941945.1
Belnapia_moabensis_GCA_000745835.1.GCA.000745835.1.ASM74583v1
Elioraea_tepidiphila_GCA_000378465.1.GCA.000378465.1.ASM37846v1
Geminicoccus_roseus_GCA_000427665.1.GCA.000427665.1.ASM42766v1
alphaproteobacterium_L41A_GCA_000335735.1.GCA.000335735.1.L41A.1.0
Brevundimonas_subvibrioides_GCA_000144605.1
Asticcacaulis_excentricus_GCA_000175215.2
Phenylobacterium_zucineum_GCA_000017265.1
Caulobacter_vibrioides_GCA_000022005.1
Oceanicaulis_alexandrii_GCA_000420265.1.GCA.000420265.1.ASM42026v1
Maricaulis_maris_GCA_003634045.1
Hyphomonas_neptunium_GCA_000013025.1
Hirschia_baltica_GCA_000023785.1
Parvularcula_bermudensis_GCA_000152825.2
Candidatus_Puniceispirillum_marinum_GCA_000024465.1
alphaproteobacterium_IMCC14465_GCA_000293845.2
Candidatus_Micropelagos_thuwalensis_GCA_000469155_1
Sphingopyxis_macrogoltabida_GCA_001314325.1
Sphingomonas_wittichii_GCA_000016765.1
Zymomonas_mobilis_GCA_000007105.1
Sphingobium_indicum_GCA_000264945.2
alphaproteobacterium_AAP81b_GCA_001295935.1
Magnetofaba_australis_GCA_002109495.1
Magnetococcus_marinus_GCA_000014865.1
Candidatus_Midichloriaceae_endosymbiont_of_Peranema_trichophorum_GCA_004210275.1
GCA_903878005.1_Candidatus_Midichloriaceae_bacterium
Rickettsiaceae_endosymbiont_of_Amblyomma_cajennense_Ac37b_GCA_000746585.2
Candidatus_Deianiraea_vastatrix_GCA_007993655.1
Enterobacter_cloacae_GCA_000770155.1
Alteromonas_macleodii_GCA_000172635.2
Ralstonia_pickettii_GCA_902374465.1.GCA.902374465.1.MGYG.HGUT.01384
Burkholderia_cepacia_GCA_009586235.1
Methylotenera_versatilis_GCA_000093025.1
Nitrosospira_briensis_GCA_000619905.2.GCA.000619905.2.ASM61990v2
Dasania_marina_GCA_000373485.1.GCA.000373485.1.ASM37348v1
Congregibacter_litoralis_GCA_000153125.2

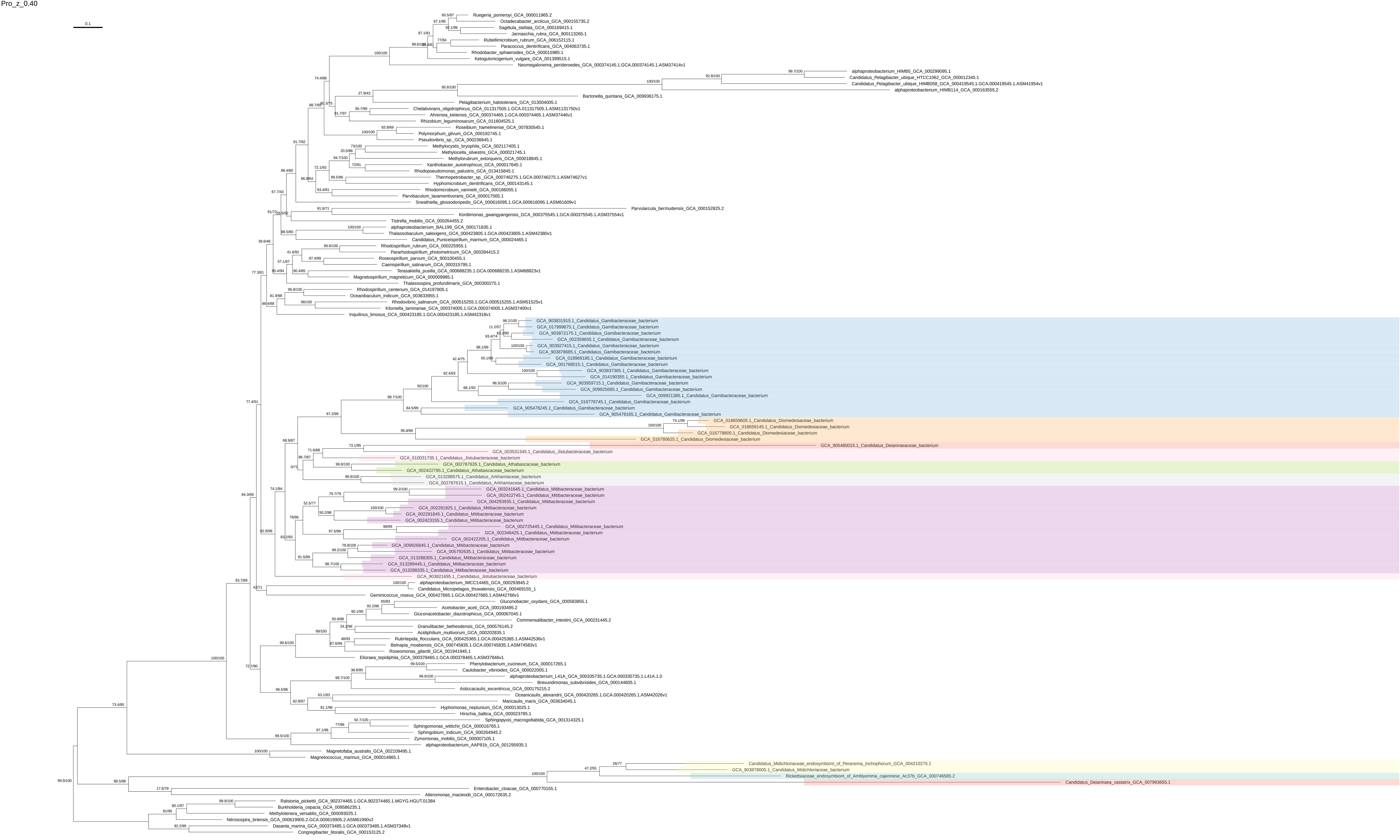
Pro_z_0.40
0.1
Ruegeria_pomeroyi_GCA_000011965.2
Octadecabacter_arcticus_GCA_000155735.2
Sagittula_stellata_GCA_000169415.1
Jannaschia_rubra_GCA_900113265.1
Rubellimicrobium_rubrum_GCA_006152115.1
Paracoccus_denitrificans_GCA_004063735.1
Rhodobacter_sphaeroides_GCA_000015985.1
Ketogulonicigenium_vulgare_GCA_001399515.1
Neomegalonema_perideroedes_GCA_000374145.1.GCA.000374145.1.ASM37414v1
alphaproteobacterium_HIMB5_GCA_000299095.1
Candidatus_Pelagibacter_ubique_HTCC1062_GCA_000012345.1
Candidatus_Pelagibacter_ubique_HIMB058_GCA_000419545.1.GCA.000419545.1.ASM41954v1
alphaproteobacterium_HIMB114_GCA_000163555.2
Bartonella_quintana_GCA_009936175.1
Pelagibacterium_halotolerans_GCA_013004005.1
Chelativorans_oligotrophicus_GCA_011317505.1.GCA.011317505.1.ASM1131750v1
Ahrensia_kielensis_GCA_000374465.1.GCA.000374465.1.ASM37446v1
Rhizobium_leguminosarum_GCA_011604525.1
Roseibium_hamelinense_GCA_007830545.1
Polymorphum_gilvum_GCA_000192745.1
Pseudovibrio_sp._GCA_000236645.1
Methylocystis_bryophila_GCA_002117405.1
Methylocella_silvestris_GCA_000021745.1
Methylorubrum_extorquens_GCA_000018845.1
Xanthobacter_autotrophicus_GCA_000017645.1
Rhodopseudomonas_palustris_GCA_013415845.1
Thermopetrobacter_sp._GCA_000746275.1.GCA.000746275.1.ASM74627v1
Hyphomicrobium_denitrificans_GCA_000143145.1
Rhodomicrobium_vannielii_GCA_000166055.1
Parvibaculum_lavamentivorans_GCA_000017565.1
Sneathiella_glossodoripedis_GCA_000616095.1.GCA.000616095.1.ASM61609v1
Parvularcula_bermudensis_GCA_000152825.2
Kordiimonas_gwangyangensis_GCA_000375545.1.GCA.000375545.1.ASM37554v1
Tistrella_mobilis_GCA_000264455.2
alphaproteobacterium_BAL199_GCA_000171835.1
Thalassobaculum_salexigens_GCA_000423805.1.GCA.000423805.1.ASM42380v1
Candidatus_Puniceispirillum_marinum_GCA_000024465.1
Rhodospirillum_rubrum_GCA_000225955.1
Pararhodospirillum_photometricum_GCA_000284415.2
Roseospirillum_parvum_GCA_900100455.1
Caenispirillum_salinarum_GCA_000315795.1
Terasakiella_pusilla_GCA_000688235.1.GCA.000688235.1.ASM68823v1
Magnetospirillum_magneticum_GCA_000009985.1
Thalassospira_profundimaris_GCA_000300275.1
Rhodospirillum_centenum_GCA_014197805.1
Oceanibaculum_indicum_GCA_003633955.1
Rhodovibrio_salinarum_GCA_000515255.1.GCA.000515255.1.ASM51525v1
Kiloniella_laminariae_GCA_000374005.1.GCA.000374005.1.ASM37400v1
Inquilinus_limosus_GCA_000423185.1.GCA.000423185.1.ASM42318v1
GCA_903831915.1_Candidatus_Gamibacteraceae_bacterium
GCA_017999675.1_Candidatus_Gamibacteraceae_bacterium
GCA_903872175.1_Candidatus_Gamibacteraceae_bacterium
GCA_002359655.1_Candidatus_Gamibacteraceae_bacterium
GCA_903927415.1_Candidatus_Gamibacteraceae_bacterium
GCA_903878685.1_Candidatus_Gamibacteraceae_bacterium
GCA_018969185.1_Candidatus_Gamibacteraceae_bacterium
GCA_001768015.1_Candidatus_Gamibacteraceae_bacterium
GCA_903837365.1_Candidatus_Gamibacteraceae_bacterium
GCA_014190355.1_Candidatus_Gamibacteraceae_bacterium
GCA_903959715.1_Candidatus_Gamibacteraceae_bacterium
GCA_009925685.1_Candidatus_Gamibacteraceae_bacterium
GCA_009921385.1_Candidatus_Gamibacteraceae_bacterium
GCA_016778745.1_Candidatus_Gamibacteraceae_bacterium
GCA_905478245.1_Candidatus_Gamibacteraceae_bacterium
GCA_905478165.1_Candidatus_Gamibacteraceae_bacterium
GCA_018659605.1_Candidatus_Diomedesiaceae_bacterium
GCA_018659145.1_Candidatus_Diomedesiaceae_bacterium
GCA_016778805.1_Candidatus_Diomedesiaceae_bacterium
GCA_016780625.1_Candidatus_Diomedesiaceae_bacterium
GCA_905480015.1_Candidatus_Deianiraeaceae_bacterium
GCA_003531345.1_Candidatus_Jistubacteraceae_bacterium
GCA_010031735.1_Candidatus_Jistubacteraceae_bacterium
GCA_002787635.1_Candidatus_Athabascaceae_bacterium
GCA_002422795.1_Candidatus_Athabascaceae_bacterium
GCA_013288575.1_Candidatus_Arkhamiaceae_bacterium
GCA_002787615.1_Candidatus_Arkhamiaceae_bacterium
GCA_003241645.1_Candidatus_Mitibacteraceae_bacterium
GCA_002422745.1_Candidatus_Mitibacteraceae_bacterium
GCA_004293935.1_Candidatus_Mitibacteraceae_bacterium
GCA_002291925.1_Candidatus_Mitibacteraceae_bacterium
GCA_002291845.1_Candidatus_Mitibacteraceae_bacterium
GCA_002423155.1_Candidatus_Mitibacteraceae_bacterium
GCA_002725445.1_Candidatus_Mitibacteraceae_bacterium
GCA_002346425.1_Candidatus_Mitibacteraceae_bacterium
GCA_002422205.1_Candidatus_Mitibacteraceae_bacterium
GCA_009926845.1_Candidatus_Mitibacteraceae_bacterium
GCA_005792635.1_Candidatus_Mitibacteraceae_bacterium
GCA_013288305.1_Candidatus_Mitibacteraceae_bacterium
GCA_013289445.1_Candidatus_Mitibacteraceae_bacterium
GCA_013288335.1_Candidatus_Mitibacteraceae_bacterium
GCA_903821695.1_Candidatus_Jistubacteraceae_bacterium
alphaproteobacterium_IMCC14465_GCA_000293845.2
Candidatus_Micropelagos_thuwalensis_GCA_000469155_1
Geminicoccus_roseus_GCA_000427665.1.GCA.000427665.1.ASM42766v1
Gluconobacter_oxydans_GCA_000583855.1
Acetobacter_aceti_GCA_000193495.2
Gluconacetobacter_diazotrophicus_GCA_000067045.1
Commensalibacter_intestini_GCA_000231445.2
Granulibacter_bethesdensis_GCA_000576145.2
Acidiphilium_multivorum_GCA_000202835.1
Rubritepida_flocculans_GCA_000425365.1.GCA.000425365.1.ASM42536v1
Belnapia_moabensis_GCA_000745835.1.GCA.000745835.1.ASM74583v1
Roseomonas_gilardii_GCA_001941945.1
Elioraea_tepidiphila_GCA_000378465.1.GCA.000378465.1.ASM37846v1
Phenylobacterium_zucineum_GCA_000017265.1
Caulobacter_vibrioides_GCA_000022005.1
alphaproteobacterium_L41A_GCA_000335735.1.GCA.000335735.1.L41A.1.0
Brevundimonas_subvibrioides_GCA_000144605.1
Asticcacaulis_excentricus_GCA_000175215.2
Oceanicaulis_alexandrii_GCA_000420265.1.GCA.000420265.1.ASM42026v1
Maricaulis_maris_GCA_003634045.1
Hyphomonas_neptunium_GCA_000013025.1
Hirschia_baltica_GCA_000023785.1
Sphingopyxis_macrogoltabida_GCA_001314325.1
Sphingomonas_wittichii_GCA_000016765.1
Sphingobium_indicum_GCA_000264945.2
Zymomonas_mobilis_GCA_000007105.1
alphaproteobacterium_AAP81b_GCA_001295935.1
Magnetofaba_australis_GCA_002109495.1
Magnetococcus_marinus_GCA_000014865.1
Candidatus_Midichloriaceae_endosymbiont_of_Peranema_trichophorum_GCA_004210275.1
GCA_903878005.1_Candidatus_Midichloriaceae_bacterium
Rickettsiaceae_endosymbiont_of_Amblyomma_cajennese_Ac37b_GCA_000746585.2
Candidatus_Deianiraea_vastatrix_GCA_007993655.1
Enterobacter_cloacae_GCA_000770155.1
Alteromonas_macleodii_GCA_000172635.2
Ralstonia_pickettii_GCA_902374465.1.GCA.902374465.1.MGYG.HGUT.01384
Burkholderia_cepacia_GCA_009586235.1
Methylotenera_versatilis_GCA_000093025.1
Nitrosospira_briensis_GCA_000619905.2.GCA.000619905.2.ASM61990v2
Dasania_marina_GCA_000373485.1.GCA.000373485.1.ASM37348v1
Congregibacter_litoralis_GCA_000153125.2

Pro_z_0.50

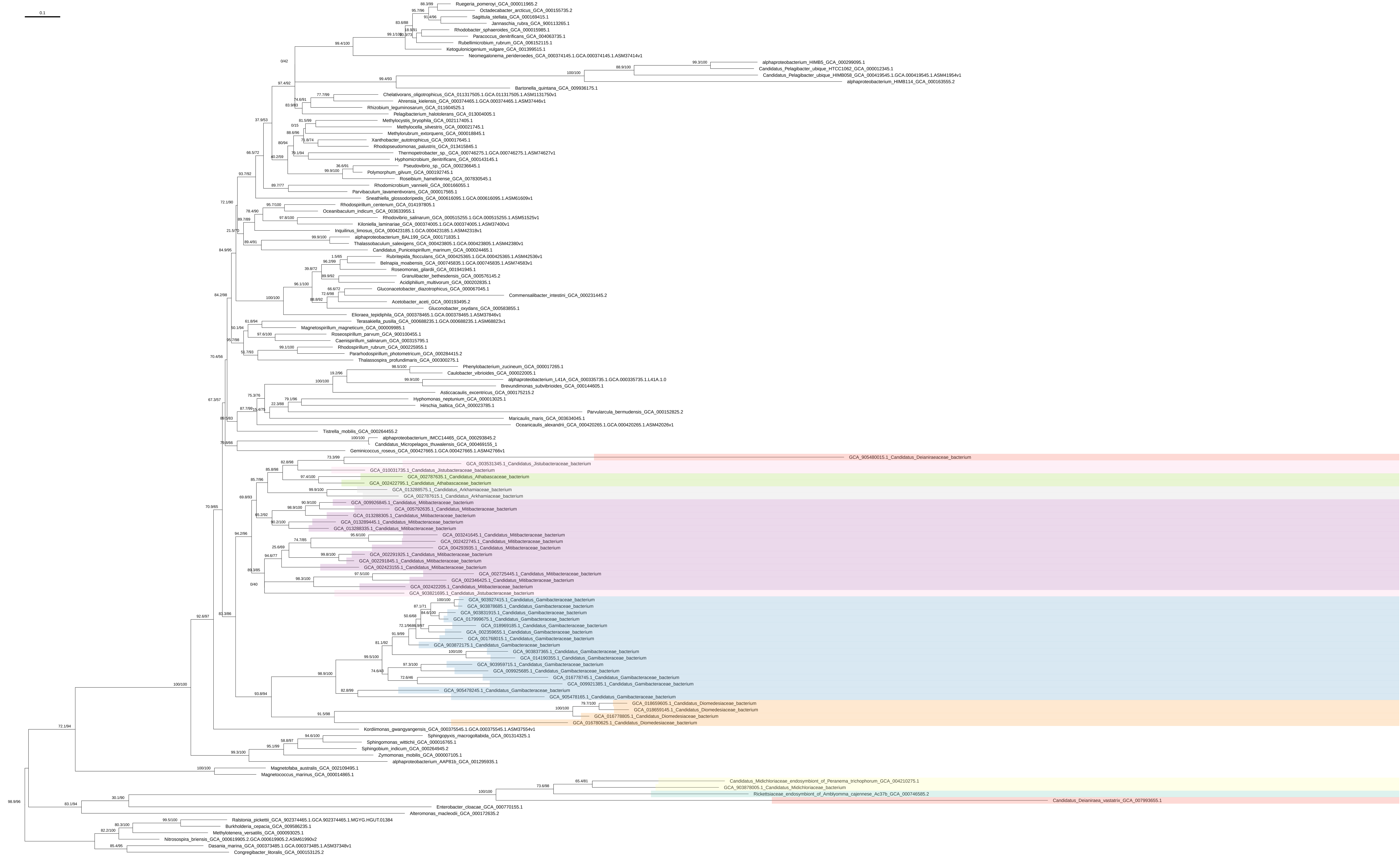
0.1
Ruegeria_pomeroyi_GCA_000011965.2
Octadecabacter_arcticus_GCA_000155735.2
Sagittula_stellata_GCA_000169415.1
Jannaschia_rubra_GCA_900113265.1
Rhodobacter_sphaeroides_GCA_000015985.1
Paracoccus_denitrificans_GCA_004063735.1
Rubellimicrobium_rubrum_GCA_006152115.1
Ketogulonicigenium_vulgare_GCA_001399515.1
Neomegalonema_perideroedes_GCA_000374145.1.GCA.000374145.1.ASM37414v1
alphaproteobacterium_HIMB5_GCA_000299095.1
Candidatus_Pelagibacter_ubique_HTCC1062_GCA_000012345.1
Candidatus_Pelagibacter_ubique_HIMB058_GCA_000419545.1.GCA.000419545.1.ASM41954v1
alphaproteobacterium_HIMB114_GCA_000163555.2
Bartonella_quintana_GCA_009936175.1
Chelativorans_oligotrophicus_GCA_011317505.1.GCA.011317505.1.ASM1131750v1
Ahrensia_kielensis_GCA_000374465.1.GCA.000374465.1.ASM37446v1
Rhizobium_leguminosarum_GCA_011604525.1
Pelagibacterium_halotolerans_GCA_013004005.1
Methylocystis_bryophila_GCA_002117405.1
Methylocella_silvestris_GCA_000021745.1
Methylorubrum_extorquens_GCA_000018845.1
Xanthobacter_autotrophicus_GCA_000017645.1
Rhodopseudomonas_palustris_GCA_013415845.1
Thermopetrobacter_sp._GCA_000746275.1.GCA.000746275.1.ASM74627v1
Hyphomicrobium_denitrificans_GCA_000143145.1
Pseudovibrio_sp._GCA_000236645.1
Polymorphum_gilvum_GCA_000192745.1
Roseibium_hamelinense_GCA_007830545.1
Rhodomicrobium_vannielii_GCA_000166055.1
Parvibaculum_lavamentivorans_GCA_000017565.1
Sneathiella_glossodoripedis_GCA_000616095.1.GCA.000616095.1.ASM61609v1
Rhodospirillum_centenum_GCA_014197805.1
Oceanibaculum_indicum_GCA_003633955.1
Rhodovibrio_salinarum_GCA_000515255.1.GCA.000515255.1.ASM51525v1
Kiloniella_laminariae_GCA_000374005.1.GCA.000374005.1.ASM37400v1
Inquilinus_limosus_GCA_000423185.1.GCA.000423185.1.ASM42318v1
alphaproteobacterium_BAL199_GCA_000171835.1
Thalassobaculum_salexigens_GCA_000423805.1.GCA.000423805.1.ASM42380v1
Candidatus_Puniceispirillum_marinum_GCA_000024465.1
Rubritepida_flocculans_GCA_000425365.1.GCA.000425365.1.ASM42536v1
Belnapia_moabensis_GCA_000745835.1.GCA.000745835.1.ASM74583v1
Roseomonas_gilardii_GCA_001941945.1
Granulibacter_bethesdensis_GCA_000576145.2
Acidiphilium_multivorum_GCA_000202835.1
Gluconacetobacter_diazotrophicus_GCA_000067045.1
Commensalibacter_intestini_GCA_000231445.2
Acetobacter_aceti_GCA_000193495.2
Gluconobacter_oxydans_GCA_000583855.1
Elioraea_tepidiphila_GCA_000378465.1.GCA.000378465.1.ASM37846v1
Terasakiella_pusilla_GCA_000688235.1.GCA.000688235.1.ASM68823v1
Magnetospirillum_magneticum_GCA_000009985.1
Roseospirillum_parvum_GCA_900100455.1
Caenispirillum_salinarum_GCA_000315795.1
Rhodospirillum_rubrum_GCA_000225955.1
Pararhodospirillum_photometricum_GCA_000284415.2
Thalassospira_profundimaris_GCA_000300275.1
Phenylobacterium_zucineum_GCA_000017265.1
Caulobacter_vibrioides_GCA_000022005.1
alphaproteobacterium_L41A_GCA_000335735.1.GCA.000335735.1.L41A.1.0
Brevundimonas_subvibrioides_GCA_000144605.1
Asticcacaulis_excentricus_GCA_000175215.2
Hyphomonas_neptunium_GCA_000013025.1
Hirschia_baltica_GCA_000023785.1
Parvularcula_bermudensis_GCA_000152825.2
Maricaulis_maris_GCA_003634045.1
Oceanicaulis_alexandrii_GCA_000420265.1.GCA.000420265.1.ASM42026v1
Tistrella_mobilis_GCA_000264455.2
alphaproteobacterium_IMCC14465_GCA_000293845.2
Candidatus_Micropelagos_thuwalensis_GCA_000469155_1
Geminicoccus_roseus_GCA_000427665.1.GCA.000427665.1.ASM42766v1
GCA_905480015.1_Candidatus_Deianiraeaceae_bacterium
GCA_003531345.1_Candidatus_Jistubacteraceae_bacterium
GCA_010031735.1_Candidatus_Jistubacteraceae_bacterium
GCA_002787635.1_Candidatus_Athabascaceae_bacterium
GCA_002422795.1_Candidatus_Athabascaceae_bacterium
GCA_013288575.1_Candidatus_Arkhamiaceae_bacterium
GCA_002787615.1_Candidatus_Arkhamiaceae_bacterium
GCA_009926845.1_Candidatus_Mitibacteraceae_bacterium
GCA_005792635.1_Candidatus_Mitibacteraceae_bacterium
GCA_013288305.1_Candidatus_Mitibacteraceae_bacterium
GCA_013289445.1_Candidatus_Mitibacteraceae_bacterium
GCA_013288335.1_Candidatus_Mitibacteraceae_bacterium
GCA_003241645.1_Candidatus_Mitibacteraceae_bacterium
GCA_002422745.1_Candidatus_Mitibacteraceae_bacterium
GCA_004293935.1_Candidatus_Mitibacteraceae_bacterium
GCA_002291925.1_Candidatus_Mitibacteraceae_bacterium
GCA_002291845.1_Candidatus_Mitibacteraceae_bacterium
GCA_002423155.1_Candidatus_Mitibacteraceae_bacterium
GCA_002725445.1_Candidatus_Mitibacteraceae_bacterium
GCA_002346425.1_Candidatus_Mitibacteraceae_bacterium
GCA_002422205.1_Candidatus_Mitibacteraceae_bacterium
GCA_903821695.1_Candidatus_Jistubacteraceae_bacterium
GCA_903927415.1_Candidatus_Gamibacteraceae_bacterium
GCA_903878685.1_Candidatus_Gamibacteraceae_bacterium
GCA_903831915.1_Candidatus_Gamibacteraceae_bacterium
GCA_017999675.1_Candidatus_Gamibacteraceae_bacterium
GCA_018969185.1_Candidatus_Gamibacteraceae_bacterium
GCA_002359655.1_Candidatus_Gamibacteraceae_bacterium
GCA_001768015.1_Candidatus_Gamibacteraceae_bacterium
GCA_903872175.1_Candidatus_Gamibacteraceae_bacterium
GCA_903837365.1_Candidatus_Gamibacteraceae_bacterium
GCA_014190355.1_Candidatus_Gamibacteraceae_bacterium
GCA_903959715.1_Candidatus_Gamibacteraceae_bacterium
GCA_009925685.1_Candidatus_Gamibacteraceae_bacterium
GCA_016778745.1_Candidatus_Gamibacteraceae_bacterium
GCA_009921385.1_Candidatus_Gamibacteraceae_bacterium
GCA_905478245.1_Candidatus_Gamibacteraceae_bacterium
GCA_905478165.1_Candidatus_Gamibacteraceae_bacterium
GCA_018659605.1_Candidatus_Diomedesiaceae_bacterium
GCA_018659145.1_Candidatus_Diomedesiaceae_bacterium
GCA_016778805.1_Candidatus_Diomedesiaceae_bacterium
GCA_016780625.1_Candidatus_Diomedesiaceae_bacterium
Kordiimonas_gwangyangensis_GCA_000375545.1.GCA.000375545.1.ASM37554v1
Sphingopyxis_macrogoltabida_GCA_001314325.1
Sphingomonas_wittichii_GCA_000016765.1
Sphingobium_indicum_GCA_000264945.2
Zymomonas_mobilis_GCA_000007105.1
alphaproteobacterium_AAP81b_GCA_001295935.1
Magnetofaba_australis_GCA_002109495.1
Magnetococcus_marinus_GCA_000014865.1
Candidatus_Midichloriaceae_endosymbiont_of_Peranema_trichophorum_GCA_004210275.1
GCA_903878005.1_Candidatus_Midichloriaceae_bacterium
Rickettsiaceae_endosymbiont_of_Amblyomma_cajennese_Ac37b_GCA_000746585.2
Candidatus_Deianiraea_vastatrix_GCA_007993655.1
Enterobacter_cloacae_GCA_000770155.1
Alteromonas_macleodii_GCA_000172635.2
Ralstonia_pickettii_GCA_902374465.1.GCA.902374465.1.MGYG.HGUT.01384
Burkholderia_cepacia_GCA_009586235.1
Methylotenera_versatilis_GCA_000093025.1
Nitrosospira_briensis_GCA_000619905.2.GCA.000619905.2.ASM61990v2
Dasania_marina_GCA_000373485.1.GCA.000373485.1.ASM37348v1
Congregibacter_litoralis_GCA_000153125.2

Serine

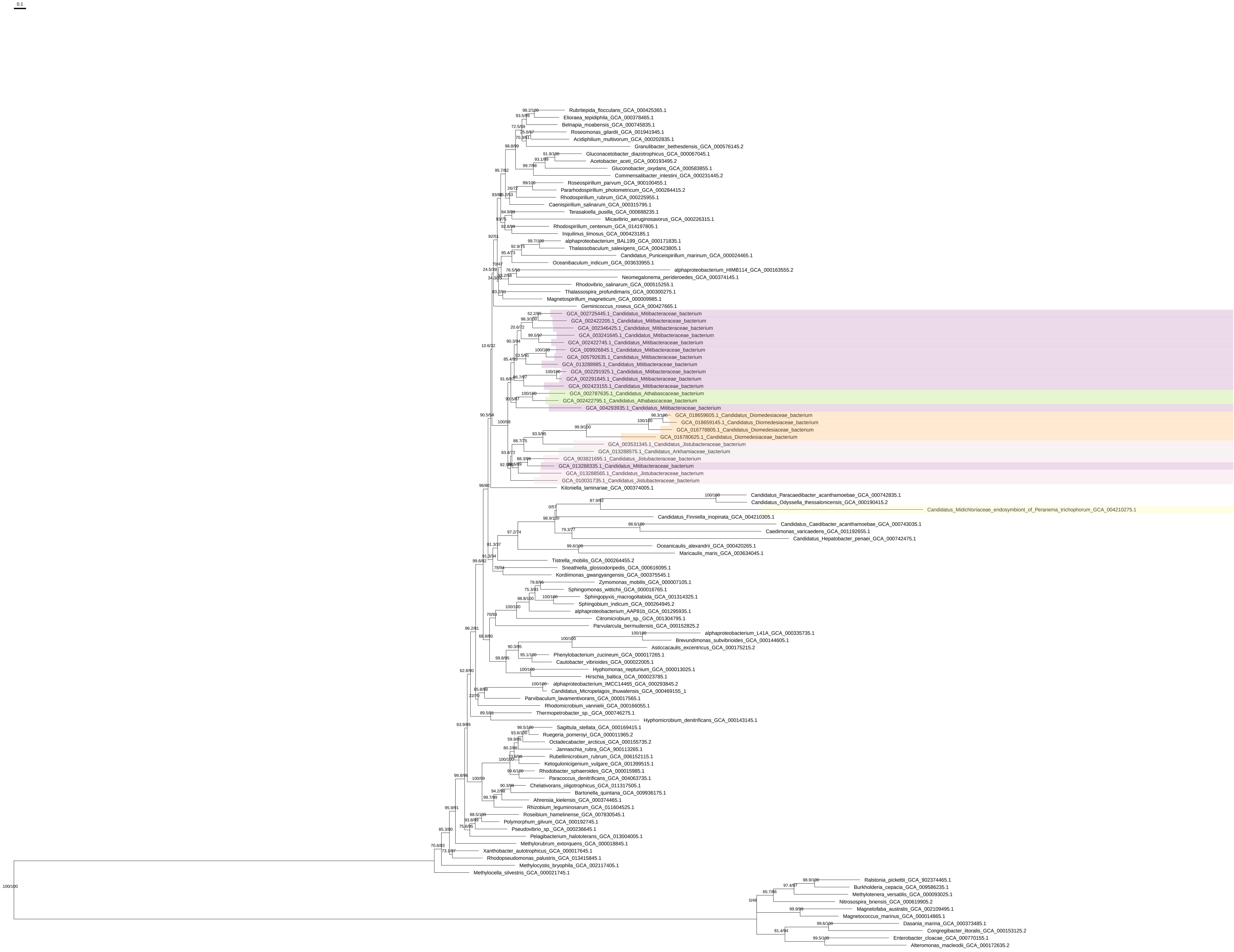
0.1
Rubritepida_flocculans_GCA_000425365.1
Elioraea_tepidiphila_GCA_000378465.1
Belnapia_moabensis_GCA_000745835.1
Roseomonas_gilardii_GCA_001941945.1
Acidiphilium_multivorum_GCA_000202835.1
Granulibacter_bethesdensis_GCA_000576145.2
Gluconacetobacter_diazotrophicus_GCA_000067045.1
Acetobacter_aceti_GCA_000193495.2
Gluconobacter_oxydans_GCA_000583855.1
Commensalibacter_intestini_GCA_000231445.2
Roseospirillum_parvum_GCA_900100455.1
Pararhodospirillum_photometricum_GCA_000284415.2
Rhodospirillum_rubrum_GCA_000225955.1
Caenispirillum_salinarum_GCA_000315795.1
Terasakiella_pusilla_GCA_000688235.1
Micavibrio_aeruginosavorus_GCA_000226315.1
Rhodospirillum_centenum_GCA_014197805.1
Inquilinus_limosus_GCA_000423185.1
alphaproteobacterium_BAL199_GCA_000171835.1
Thalassobaculum_salexigens_GCA_000423805.1
Candidatus_Puniceispirillum_marinum_GCA_000024465.1
Oceanibaculum_indicum_GCA_003633955.1
alphaproteobacterium_HIMB114_GCA_000163555.2
Neomegalonema_perideroedes_GCA_000374145.1
Rhodovibrio_salinarum_GCA_000515255.1
Thalassospira_profundimaris_GCA_000300275.1
Magnetospirillum_magneticum_GCA_000009985.1
Geminicoccus_roseus_GCA_000427665.1
GCA_002725445.1_Candidatus_Mitibacteraceae_bacterium
GCA_002422205.1_Candidatus_Mitibacteraceae_bacterium
GCA_002346425.1_Candidatus_Mitibacteraceae_bacterium
GCA_003241645.1_Candidatus_Mitibacteraceae_bacterium
GCA_002422745.1_Candidatus_Mitibacteraceae_bacterium
GCA_009926845.1_Candidatus_Mitibacteraceae_bacterium
GCA_005792635.1_Candidatus_Mitibacteraceae_bacterium
GCA_013288985.1_Candidatus_Mitibacteraceae_bacterium
GCA_002291925.1_Candidatus_Mitibacteraceae_bacterium
GCA_002291845.1_Candidatus_Mitibacteraceae_bacterium
GCA_002423155.1_Candidatus_Mitibacteraceae_bacterium
GCA_002787635.1_Candidatus_Athabascaceae_bacterium
GCA_002422795.1_Candidatus_Athabascaceae_bacterium
GCA_004293935.1_Candidatus_Mitibacteraceae_bacterium
GCA_018659605.1_Candidatus_Diomedesiaceae_bacterium
GCA_018659145.1_Candidatus_Diomedesiaceae_bacterium
GCA_016778805.1_Candidatus_Diomedesiaceae_bacterium
GCA_016780625.1_Candidatus_Diomedesiaceae_bacterium
GCA_003531345.1_Candidatus_Jistubacteraceae_bacterium
GCA_013288575.1_Candidatus_Arkhamiaceae_bacterium
GCA_903821695.1_Candidatus_Jistubacteraceae_bacterium
GCA_013288335.1_Candidatus_Mitibacteraceae_bacterium
GCA_013288565.1_Candidatus_Jistubacteraceae_bacterium
GCA_010031735.1_Candidatus_Jistubacteraceae_bacterium
Kiloniella_laminariae_GCA_000374005.1
Candidatus_Paracaedibacter_acanthamoebae_GCA_000742835.1
Candidatus_Odyssella_thessalonicensis_GCA_000190415.2
Candidatus_Midichloriaceae_endosymbiont_of_Peranema_trichophorum_GCA_004210275.1
Candidatus_Finniella_inopinata_GCA_004210305.1
Candidatus_Caedibacter_acanthamoebae_GCA_000743035.1
Caedimonas_varicaedens_GCA_001192655.1
Candidatus_Hepatobacter_penaei_GCA_000742475.1
Oceanicaulis_alexandrii_GCA_000420265.1
Maricaulis_maris_GCA_003634045.1
Tistrella_mobilis_GCA_000264455.2
Sneathiella_glossodoripedis_GCA_000616095.1
Kordiimonas_gwangyangensis_GCA_000375545.1
Zymomonas_mobilis_GCA_000007105.1
Sphingomonas_wittichii_GCA_000016765.1
Sphingopyxis_macrogoltabida_GCA_001314325.1
Sphingobium_indicum_GCA_000264945.2
alphaproteobacterium_AAP81b_GCA_001295935.1
Citromicrobium_sp._GCA_001304795.1
Parvularcula_bermudensis_GCA_000152825.2
alphaproteobacterium_L41A_GCA_000335735.1
Brevundimonas_subvibrioides_GCA_000144605.1
Asticcacaulis_excentricus_GCA_000175215.2
Phenylobacterium_zucineum_GCA_000017265.1
Caulobacter_vibrioides_GCA_000022005.1
Hyphomonas_neptunium_GCA_000013025.1
Hirschia_baltica_GCA_000023785.1
alphaproteobacterium_IMCC14465_GCA_000293845.2
Candidatus_Micropelagos_thuwalensis_GCA_000469155_1
Parvibaculum_lavamentivorans_GCA_000017565.1
Rhodomicrobium_vannielii_GCA_000166055.1
Thermopetrobacter_sp._GCA_000746275.1
Hyphomicrobium_denitrificans_GCA_000143145.1
Sagittula_stellata_GCA_000169415.1
Ruegeria_pomeroyi_GCA_000011965.2
Octadecabacter_arcticus_GCA_000155735.2
Jannaschia_rubra_GCA_900113265.1
Rubellimicrobium_rubrum_GCA_006152115.1
Ketogulonicigenium_vulgare_GCA_001399515.1
Rhodobacter_sphaeroides_GCA_000015985.1
Paracoccus_denitrificans_GCA_004063735.1
Chelativorans_oligotrophicus_GCA_011317505.1
Bartonella_quintana_GCA_009936175.1
Ahrensia_kielensis_GCA_000374465.1
Rhizobium_leguminosarum_GCA_011604525.1
Roseibium_hamelinense_GCA_007830545.1
Polymorphum_gilvum_GCA_000192745.1
Pseudovibrio_sp._GCA_000236645.1
Pelagibacterium_halotolerans_GCA_013004005.1
Methylorubrum_extorquens_GCA_000018845.1
Xanthobacter_autotrophicus_GCA_000017645.1
Rhodopseudomonas_palustris_GCA_013415845.1
Methylocystis_bryophila_GCA_002117405.1
Methylocella_silvestris_GCA_000021745.1
Ralstonia_pickettii_GCA_902374465.1
Burkholderia_cepacia_GCA_009586235.1
Methylotenera_versatilis_GCA_000093025.1
Nitrosospira_briensis_GCA_000619905.2
Magnetofaba_australis_GCA_002109495.1
Magnetococcus_marinus_GCA_000014865.1
Dasania_marina_GCA_000373485.1
Congregibacter_litoralis_GCA_000153125.2
Enterobacter_cloacae_GCA_000770155.1
Alteromonas_macleodii_GCA_000172635.2

Serine_z_0.10

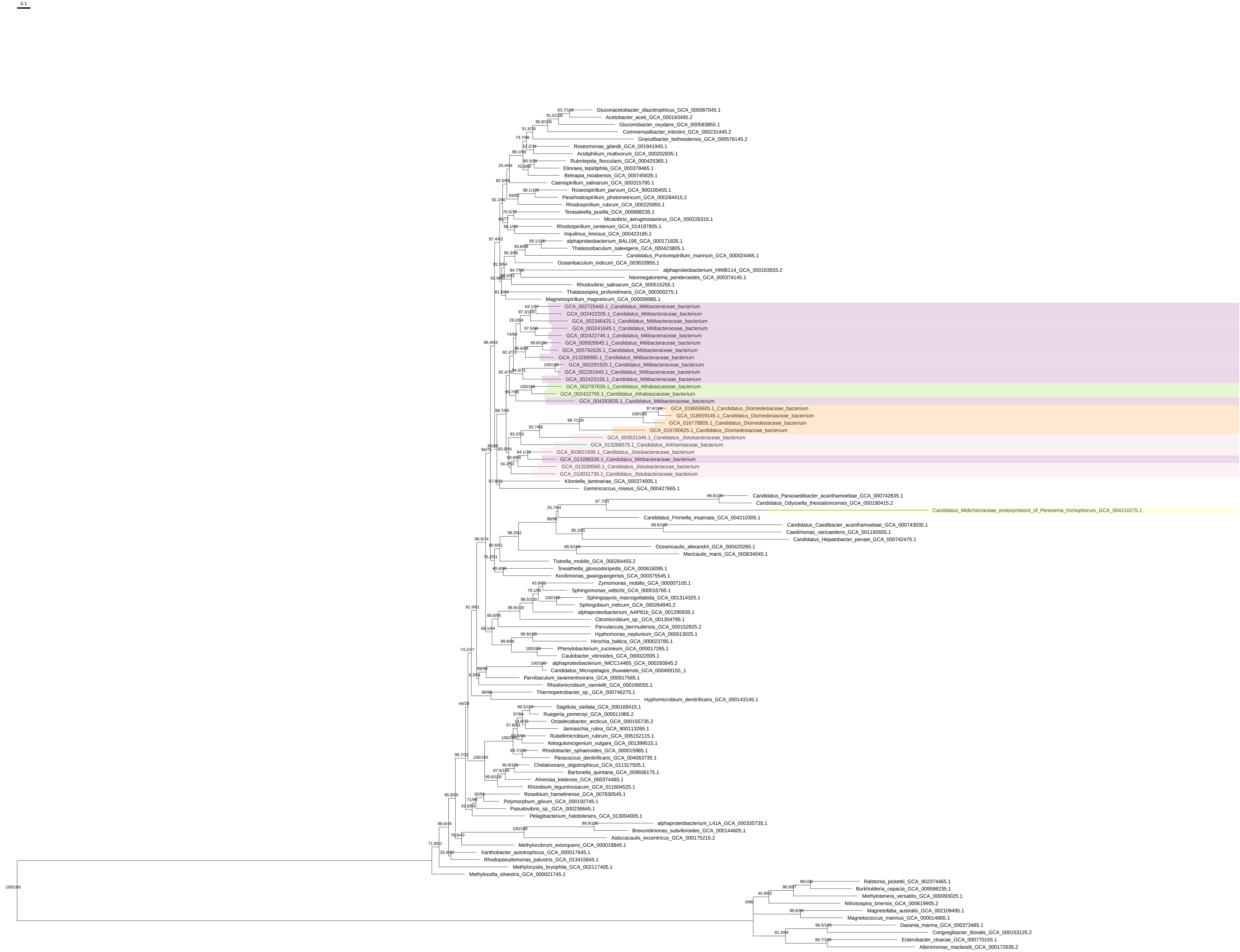
0.1
Gluconacetobacter_diazotrophicus_GCA_000067045.1
Acetobacter_aceti_GCA_000193495.2
Gluconobacter_oxydans_GCA_000583855.1
Commensalibacter_intestini_GCA_000231445.2
Granulibacter_bethesdensis_GCA_000576145.2
Roseomonas_gilardii_GCA_001941945.1
Acidiphilium_multivorum_GCA_000202835.1
Rubritepida_flocculans_GCA_000425365.1
Elioraea_tepidiphila_GCA_000378465.1
Belnapia_moabensis_GCA_000745835.1
Caenispirillum_salinarum_GCA_000315795.1
Roseospirillum_parvum_GCA_900100455.1
Pararhodospirillum_photometricum_GCA_000284415.2
Rhodospirillum_rubrum_GCA_000225955.1
Terasakiella_pusilla_GCA_000688235.1
Micavibrio_aeruginosavorus_GCA_000226315.1
Rhodospirillum_centenum_GCA_014197805.1
Inquilinus_limosus_GCA_000423185.1
alphaproteobacterium_BAL199_GCA_000171835.1
Thalassobaculum_salexigens_GCA_000423805.1
Candidatus_Puniceispirillum_marinum_GCA_000024465.1
Oceanibaculum_indicum_GCA_003633955.1
alphaproteobacterium_HIMB114_GCA_000163555.2
Neomegalonema_perideroedes_GCA_000374145.1
Rhodovibrio_salinarum_GCA_000515255.1
Thalassospira_profundimaris_GCA_000300275.1
Magnetospirillum_magneticum_GCA_000009985.1
GCA_002725445.1_Candidatus_Mitibacteraceae_bacterium
GCA_002422205.1_Candidatus_Mitibacteraceae_bacterium
GCA_002346425.1_Candidatus_Mitibacteraceae_bacterium
GCA_003241645.1_Candidatus_Mitibacteraceae_bacterium
GCA_002422745.1_Candidatus_Mitibacteraceae_bacterium
GCA_009926845.1_Candidatus_Mitibacteraceae_bacterium
GCA_005792635.1_Candidatus_Mitibacteraceae_bacterium
GCA_013288985.1_Candidatus_Mitibacteraceae_bacterium
GCA_002291925.1_Candidatus_Mitibacteraceae_bacterium
GCA_002291845.1_Candidatus_Mitibacteraceae_bacterium
GCA_002423155.1_Candidatus_Mitibacteraceae_bacterium
GCA_002787635.1_Candidatus_Athabascaceae_bacterium
GCA_002422795.1_Candidatus_Athabascaceae_bacterium
GCA_004293935.1_Candidatus_Mitibacteraceae_bacterium
GCA_018659605.1_Candidatus_Diomedesiaceae_bacterium
GCA_018659145.1_Candidatus_Diomedesiaceae_bacterium
GCA_016778805.1_Candidatus_Diomedesiaceae_bacterium
GCA_016780625.1_Candidatus_Diomedesiaceae_bacterium
GCA_003531345.1_Candidatus_Jistubacteraceae_bacterium
GCA_013288575.1_Candidatus_Arkhamiaceae_bacterium
GCA_903821695.1_Candidatus_Jistubacteraceae_bacterium
GCA_013288335.1_Candidatus_Mitibacteraceae_bacterium
GCA_013288565.1_Candidatus_Jistubacteraceae_bacterium
GCA_010031735.1_Candidatus_Jistubacteraceae_bacterium
Kiloniella_laminariae_GCA_000374005.1
Geminicoccus_roseus_GCA_000427665.1
Candidatus_Paracaedibacter_acanthamoebae_GCA_000742835.1
Candidatus_Odyssella_thessalonicensis_GCA_000190415.2
Candidatus_Midichloriaceae_endosymbiont_of_Peranema_trichophorum_GCA_004210275.1
Candidatus_Finniella_inopinata_GCA_004210305.1
Candidatus_Caedibacter_acanthamoebae_GCA_000743035.1
Caedimonas_varicaedens_GCA_001192655.1
Candidatus_Hepatobacter_penaei_GCA_000742475.1
Oceanicaulis_alexandrii_GCA_000420265.1
Maricaulis_maris_GCA_003634045.1
Tistrella_mobilis_GCA_000264455.2
Sneathiella_glossodoripedis_GCA_000616095.1
Kordiimonas_gwangyangensis_GCA_000375545.1
Zymomonas_mobilis_GCA_000007105.1
Sphingomonas_wittichii_GCA_000016765.1
Sphingopyxis_macrogoltabida_GCA_001314325.1
Sphingobium_indicum_GCA_000264945.2
alphaproteobacterium_AAP81b_GCA_001295935.1
Citromicrobium_sp._GCA_001304795.1
Parvularcula_bermudensis_GCA_000152825.2
Hyphomonas_neptunium_GCA_000013025.1
Hirschia_baltica_GCA_000023785.1
Phenylobacterium_zucineum_GCA_000017265.1
Caulobacter_vibrioides_GCA_000022005.1
alphaproteobacterium_IMCC14465_GCA_000293845.2
Candidatus_Micropelagos_thuwalensis_GCA_000469155_1
Parvibaculum_lavamentivorans_GCA_000017565.1
Rhodomicrobium_vannielii_GCA_000166055.1
Thermopetrobacter_sp._GCA_000746275.1
Hyphomicrobium_denitrificans_GCA_000143145.1
Sagittula_stellata_GCA_000169415.1
Ruegeria_pomeroyi_GCA_000011965.2
Octadecabacter_arcticus_GCA_000155735.2
Jannaschia_rubra_GCA_900113265.1
Rubellimicrobium_rubrum_GCA_006152115.1
Ketogulonicigenium_vulgare_GCA_001399515.1
Rhodobacter_sphaeroides_GCA_000015985.1
Paracoccus_denitrificans_GCA_004063735.1
Chelativorans_oligotrophicus_GCA_011317505.1
Bartonella_quintana_GCA_009936175.1
Ahrensia_kielensis_GCA_000374465.1
Rhizobium_leguminosarum_GCA_011604525.1
Roseibium_hamelinense_GCA_007830545.1
Polymorphum_gilvum_GCA_000192745.1
Pseudovibrio_sp._GCA_000236645.1
Pelagibacterium_halotolerans_GCA_013004005.1
alphaproteobacterium_L41A_GCA_000335735.1
Brevundimonas_subvibrioides_GCA_000144605.1
Asticcacaulis_excentricus_GCA_000175215.2
Methylorubrum_extorquens_GCA_000018845.1
Xanthobacter_autotrophicus_GCA_000017645.1
Rhodopseudomonas_palustris_GCA_013415845.1
Methylocystis_bryophila_GCA_002117405.1
Methylocella_silvestris_GCA_000021745.1
Ralstonia_pickettii_GCA_902374465.1
Burkholderia_cepacia_GCA_009586235.1
Methylotenera_versatilis_GCA_000093025.1
Nitrosospira_briensis_GCA_000619905.2
Magnetofaba_australis_GCA_002109495.1
Magnetococcus_marinus_GCA_000014865.1
Dasania_marina_GCA_000373485.1
Congregibacter_litoralis_GCA_000153125.2
Enterobacter_cloacae_GCA_000770155.1
Alteromonas_macleodii_GCA_000172635.2

Serine_z_0.20

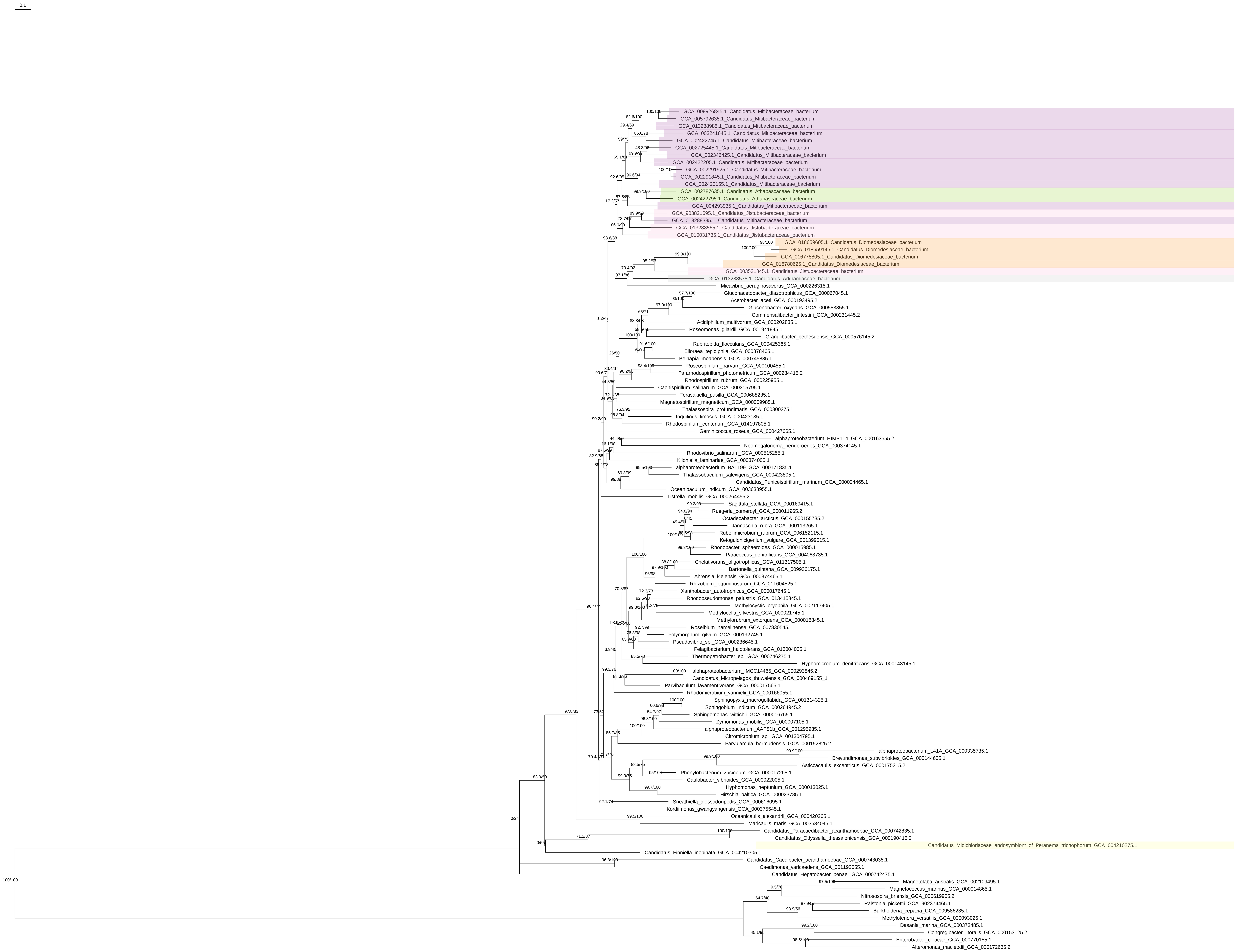
0.1
GCA_009926845.1_Candidatus_Mitibacteraceae_bacterium
GCA_005792635.1_Candidatus_Mitibacteraceae_bacterium
GCA_013288985.1_Candidatus_Mitibacteraceae_bacterium
GCA_003241645.1_Candidatus_Mitibacteraceae_bacterium
GCA_002422745.1_Candidatus_Mitibacteraceae_bacterium
GCA_002725445.1_Candidatus_Mitibacteraceae_bacterium
GCA_002346425.1_Candidatus_Mitibacteraceae_bacterium
GCA_002422205.1_Candidatus_Mitibacteraceae_bacterium
GCA_002291925.1_Candidatus_Mitibacteraceae_bacterium
GCA_002291845.1_Candidatus_Mitibacteraceae_bacterium
GCA_002423155.1_Candidatus_Mitibacteraceae_bacterium
GCA_002787635.1_Candidatus_Athabascaceae_bacterium
GCA_002422795.1_Candidatus_Athabascaceae_bacterium
GCA_004293935.1_Candidatus_Mitibacteraceae_bacterium
GCA_903821695.1_Candidatus_Jistubacteraceae_bacterium
GCA_013288335.1_Candidatus_Mitibacteraceae_bacterium
GCA_013288565.1_Candidatus_Jistubacteraceae_bacterium
GCA_010031735.1_Candidatus_Jistubacteraceae_bacterium
GCA_018659605.1_Candidatus_Diomedesiaceae_bacterium
GCA_018659145.1_Candidatus_Diomedesiaceae_bacterium
GCA_016778805.1_Candidatus_Diomedesiaceae_bacterium
GCA_016780625.1_Candidatus_Diomedesiaceae_bacterium
GCA_003531345.1_Candidatus_Jistubacteraceae_bacterium
GCA_013288575.1_Candidatus_Arkhamiaceae_bacterium
Micavibrio_aeruginosavorus_GCA_000226315.1
Gluconacetobacter_diazotrophicus_GCA_000067045.1
Acetobacter_aceti_GCA_000193495.2
Gluconobacter_oxydans_GCA_000583855.1
Commensalibacter_intestini_GCA_000231445.2
Acidiphilium_multivorum_GCA_000202835.1
Roseomonas_gilardii_GCA_001941945.1
Granulibacter_bethesdensis_GCA_000576145.2
Rubritepida_flocculans_GCA_000425365.1
Elioraea_tepidiphila_GCA_000378465.1
Belnapia_moabensis_GCA_000745835.1
Roseospirillum_parvum_GCA_900100455.1
Pararhodospirillum_photometricum_GCA_000284415.2
Rhodospirillum_rubrum_GCA_000225955.1
Caenispirillum_salinarum_GCA_000315795.1
Terasakiella_pusilla_GCA_000688235.1
Magnetospirillum_magneticum_GCA_000009985.1
Thalassospira_profundimaris_GCA_000300275.1
Inquilinus_limosus_GCA_000423185.1
Rhodospirillum_centenum_GCA_014197805.1
Geminicoccus_roseus_GCA_000427665.1
alphaproteobacterium_HIMB114_GCA_000163555.2
Neomegalonema_perideroedes_GCA_000374145.1
Rhodovibrio_salinarum_GCA_000515255.1
Kiloniella_laminariae_GCA_000374005.1
alphaproteobacterium_BAL199_GCA_000171835.1
Thalassobaculum_salexigens_GCA_000423805.1
Candidatus_Puniceispirillum_marinum_GCA_000024465.1
Oceanibaculum_indicum_GCA_003633955.1
Tistrella_mobilis_GCA_000264455.2
Sagittula_stellata_GCA_000169415.1
Ruegeria_pomeroyi_GCA_000011965.2
Octadecabacter_arcticus_GCA_000155735.2
Jannaschia_rubra_GCA_900113265.1
Rubellimicrobium_rubrum_GCA_006152115.1
Ketogulonicigenium_vulgare_GCA_001399515.1
Rhodobacter_sphaeroides_GCA_000015985.1
Paracoccus_denitrificans_GCA_004063735.1
Chelativorans_oligotrophicus_GCA_011317505.1
Bartonella_quintana_GCA_009936175.1
Ahrensia_kielensis_GCA_000374465.1
Rhizobium_leguminosarum_GCA_011604525.1
Xanthobacter_autotrophicus_GCA_000017645.1
Rhodopseudomonas_palustris_GCA_013415845.1
Methylocystis_bryophila_GCA_002117405.1
Methylocella_silvestris_GCA_000021745.1
Methylorubrum_extorquens_GCA_000018845.1
Roseibium_hamelinense_GCA_007830545.1
Polymorphum_gilvum_GCA_000192745.1
Pseudovibrio_sp._GCA_000236645.1
Pelagibacterium_halotolerans_GCA_013004005.1
Thermopetrobacter_sp._GCA_000746275.1
Hyphomicrobium_denitrificans_GCA_000143145.1
alphaproteobacterium_IMCC14465_GCA_000293845.2
Candidatus_Micropelagos_thuwalensis_GCA_000469155.1
Parvibaculum_lavamentivorans_GCA_000017565.1
Rhodomicrobium_vannielii_GCA_000166055.1
Sphingopyxis_macrogoltabida_GCA_001314325.1
Sphingobium_indicum_GCA_000264945.2
Sphingomonas_wittichii_GCA_000016765.1
Zymomonas_mobilis_GCA_000007105.1
alphaproteobacterium_AAP81b_GCA_001295935.1
Citromicrobium_sp._GCA_001304795.1
Parvularcula_bermudensis_GCA_000152825.2
alphaproteobacterium_L41A_GCA_000335735.1
Brevundimonas_subvibrioides_GCA_000144605.1
Asticcacaulis_excentricus_GCA_000175215.2
Phenylobacterium_zucineum_GCA_000017265.1
Caulobacter_vibrioides_GCA_000022005.1
Hyphomonas_neptunium_GCA_000013025.1
Hirschia_baltica_GCA_000023785.1
Sneathiella_glossodoripedis_GCA_000616095.1
Kordiimonas_gwangyangensis_GCA_000375545.1
Oceanicaulis_alexandrii_GCA_000420265.1
Maricaulis_maris_GCA_003634045.1
Candidatus_Paracaedibacter_acanthamoebae_GCA_000742835.1
Candidatus_Odyssella_thessalonicensis_GCA_000190415.2
Candidatus_Midichloriaceae_endosymbiont_of_Peranema_trichophorum_GCA_004210275.1
Candidatus_Finniella_inopinata_GCA_004210305.1
Candidatus_Caedibacter_acanthamoebae_GCA_000743035.1
Caedimonas_varicaedens_GCA_001192655.1
Candidatus_Hepatobacter_penaei_GCA_000742475.1
Magnetofaba_australis_GCA_002109495.1
Magnetococcus_marinus_GCA_000014865.1
Nitrosospira_briensis_GCA_000619905.2
Ralstonia_pickettii_GCA_902374465.1
Burkholderia_cepacia_GCA_009586235.1
Methylotenera_versatilis_GCA_000093025.1
Dasania_marina_GCA_000373485.1
Congregibacter_litoralis_GCA_000153125.2
Enterobacter_cloacae_GCA_000770155.1
Alteromonas_macleodii_GCA_000172635.2

Serine_z_0.30

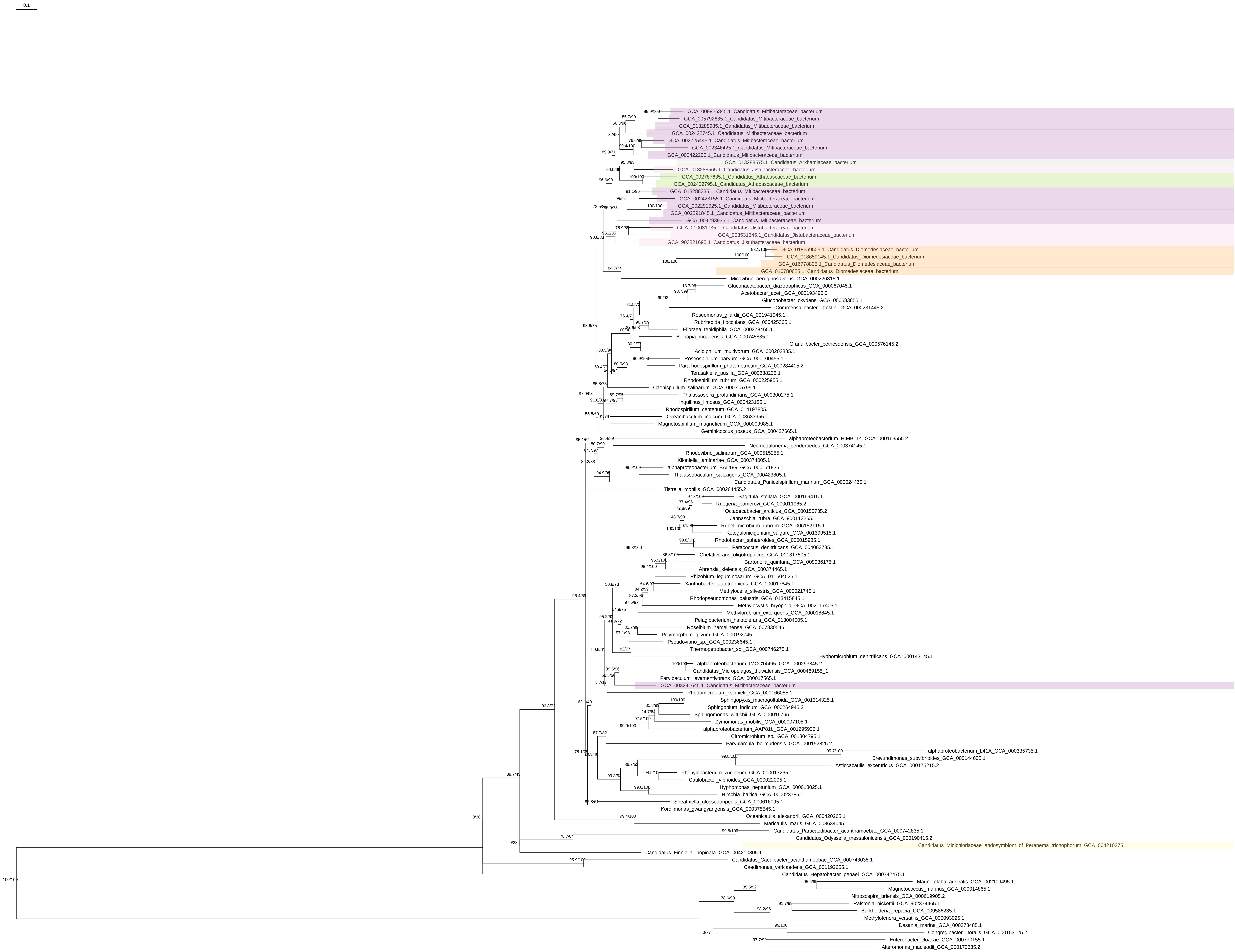

0.1
GCA_009926845.1_Candidatus_Mitibacteraceae_bacterium
GCA_005792635.1_Candidatus_Mitibacteraceae_bacterium
GCA_013288985.1_Candidatus_Mitibacteraceae_bacterium
GCA_002422745.1_Candidatus_Mitibacteraceae_bacterium
GCA_002725445.1_Candidatus_Mitibacteraceae_bacterium
GCA_002346425.1_Candidatus_Mitibacteraceae_bacterium
GCA_002422205.1_Candidatus_Mitibacteraceae_bacterium
GCA_013288575.1_Candidatus_Arkhamiaceae_bacterium
GCA_013288565.1_Candidatus_Jistubacteraceae_bacterium
GCA_002787635.1_Candidatus_Athabascaceae_bacterium
GCA_002422795.1_Candidatus_Athabascaceae_bacterium
GCA_013288335.1_Candidatus_Mitibacteraceae_bacterium
GCA_002423155.1_Candidatus_Mitibacteraceae_bacterium
GCA_002291925.1_Candidatus_Mitibacteraceae_bacterium
GCA_002291845.1_Candidatus_Mitibacteraceae_bacterium
GCA_004293935.1_Candidatus_Mitibacteraceae_bacterium
GCA_010031735.1_Candidatus_Jistubacteraceae_bacterium
GCA_003531345.1_Candidatus_Jistubacteraceae_bacterium
GCA_903821695.1_Candidatus_Jistubacteraceae_bacterium
GCA_018659605.1_Candidatus_Diomedesiaceae_bacterium
GCA_018659145.1_Candidatus_Diomedesiaceae_bacterium
GCA_016778805.1_Candidatus_Diomedesiaceae_bacterium
GCA_016780625.1_Candidatus_Diomedesiaceae_bacterium
Micavibrio_aeruginosavorus_GCA_000226315.1
Gluconacetobacter_diazotrophicus_GCA_000067045.1
Acetobacter_aceti_GCA_000193495.2
Gluconobacter_oxydans_GCA_000583855.1
Commensalibacter_intestini_GCA_000231445.2
Roseomonas_gilardii_GCA_001941945.1
Rubritepida_flocculans_GCA_000425365.1
Elioraea_tepidiphila_GCA_000378465.1
Belnapia_moabensis_GCA_000745835.1
Granulibacter_bethesdensis_GCA_000576145.2
Acidiphilium_multivorum_GCA_000202835.1
Roseospirillum_parvum_GCA_900100455.1
Pararhodospirillum_photometricum_GCA_000284415.2
Terasakiella_pusilla_GCA_000688235.1
Rhodospirillum_rubrum_GCA_000225955.1
Caenispirillum_salinarum_GCA_000315795.1
Thalassospira_profundimaris_GCA_000300275.1
Inquilinus_limosus_GCA_000423185.1
Rhodospirillum_centenum_GCA_014197805.1
Oceanibaculum_indicum_GCA_003633955.1
Magnetospirillum_magneticum_GCA_000009985.1
Geminicoccus_roseus_GCA_000427665.1
alphaproteobacterium_HIMB114_GCA_000163555.2
Neomegalonema_perideroedes_GCA_000374145.1
Rhodovibrio_salinarum_GCA_000515255.1
Kiloniella_laminariae_GCA_000374005.1
alphaproteobacterium_BAL199_GCA_000171835.1
Thalassobaculum_salexigens_GCA_000423805.1
Candidatus_Puniceispirillum_marinum_GCA_000024465.1
Tistrella_mobilis_GCA_000264455.2
Sagittula_stellata_GCA_000169415.1
Ruegeria_pomeroyi_GCA_000011965.2
Octadecabacter_arcticus_GCA_000155735.2
Jannaschia_rubra_GCA_900113265.1
Rubellimicrobium_rubrum_GCA_006152115.1
Ketogulonicigenium_vulgare_GCA_001399515.1
Rhodobacter_sphaeroides_GCA_000015985.1
Paracoccus_denitrificans_GCA_004063735.1
Chelativorans_oligotrophicus_GCA_011317505.1
Bartonella_quintana_GCA_009936175.1
Ahrensia_kielensis_GCA_000374465.1
Rhizobium_leguminosarum_GCA_011604525.1
Xanthobacter_autotrophicus_GCA_000017645.1
Methylocella_silvestris_GCA_000021745.1
Rhodopseudomonas_palustris_GCA_013415845.1
Methylocystis_bryophila_GCA_002117405.1
Methylorubrum_extorquens_GCA_000018845.1
Pelagibacterium_halotolerans_GCA_013004005.1
Roseibium_hamelinense_GCA_007830545.1
Polymorphum_gilvum_GCA_000192745.1
Pseudovibrio_sp._GCA_000236645.1
Thermopetrobacter_sp._GCA_000746275.1
Hyphomicrobium_denitrificans_GCA_000143145.1
alphaproteobacterium_IMCC14465_GCA_000293845.2
Candidatus_Micropelagos_thuwalensis_GCA_000469155_1
Parvibaculum_lavamentivorans_GCA_000017565.1
GCA_003241645.1_Candidatus_Mitibacteraceae_bacterium
Rhodomicrobium_vannielii_GCA_000166055.1
Sphingopyxis_macrogoltabida_GCA_001314325.1
Sphingobium_indicum_GCA_000264945.2
Sphingomonas_wittichii_GCA_000016765.1
Zymomonas_mobilis_GCA_000007105.1
alphaproteobacterium_AAP81b_GCA_001295935.1
Citromicrobium_sp._GCA_001304795.1
Parvularcula_bermudensis_GCA_000152825.2
alphaproteobacterium_L41A_GCA_000335735.1
Brevundimonas_subvibrioides_GCA_000144605.1
Asticcacaulis_excentricus_GCA_000175215.2
Phenylobacterium_zucineum_GCA_000017265.1
Caulobacter_vibrioides_GCA_000022005.1
Hyphomonas_neptunium_GCA_000013025.1
Hirschia_baltica_GCA_000023785.1
Sneathiella_glossodoripedis_GCA_000616095.1
Kordiimonas_gwangyangensis_GCA_000375545.1
Oceanicaulis_alexandrii_GCA_000420265.1
Maricaulis_maris_GCA_003634045.1
Candidatus_Paracaedibacter_acanthamoebae_GCA_000742835.1
Candidatus_Odyssella_thessalonicensis_GCA_000190415.2
Candidatus_Midichloriaceae_endosymbiont_of_Peranema_trichophorum_GCA_004210275.1
Candidatus_Finniella_inopinata_GCA_004210305.1
Candidatus_Caedibacter_acanthamoebae_GCA_000743035.1
Caedimonas_varicaedens_GCA_001192655.1
Candidatus_Hepatobacter_penaei_GCA_000742475.1
Magnetofaba_australis_GCA_002109495.1
Magnetococcus_marinus_GCA_000014865.1
Nitrosospira_briensis_GCA_000619905.2
Ralstonia_pickettii_GCA_902374465.1
Burkholderia_cepacia_GCA_009586235.1
Methylotenera_versatilis_GCA_000093025.1
Dasania_marina_GCA_000373485.1
Congregibacter_litoralis_GCA_000153125.2
Enterobacter_cloacae_GCA_000770155.1
Alteromonas_macleodii_GCA_000172635.2

Serine_z_0.40

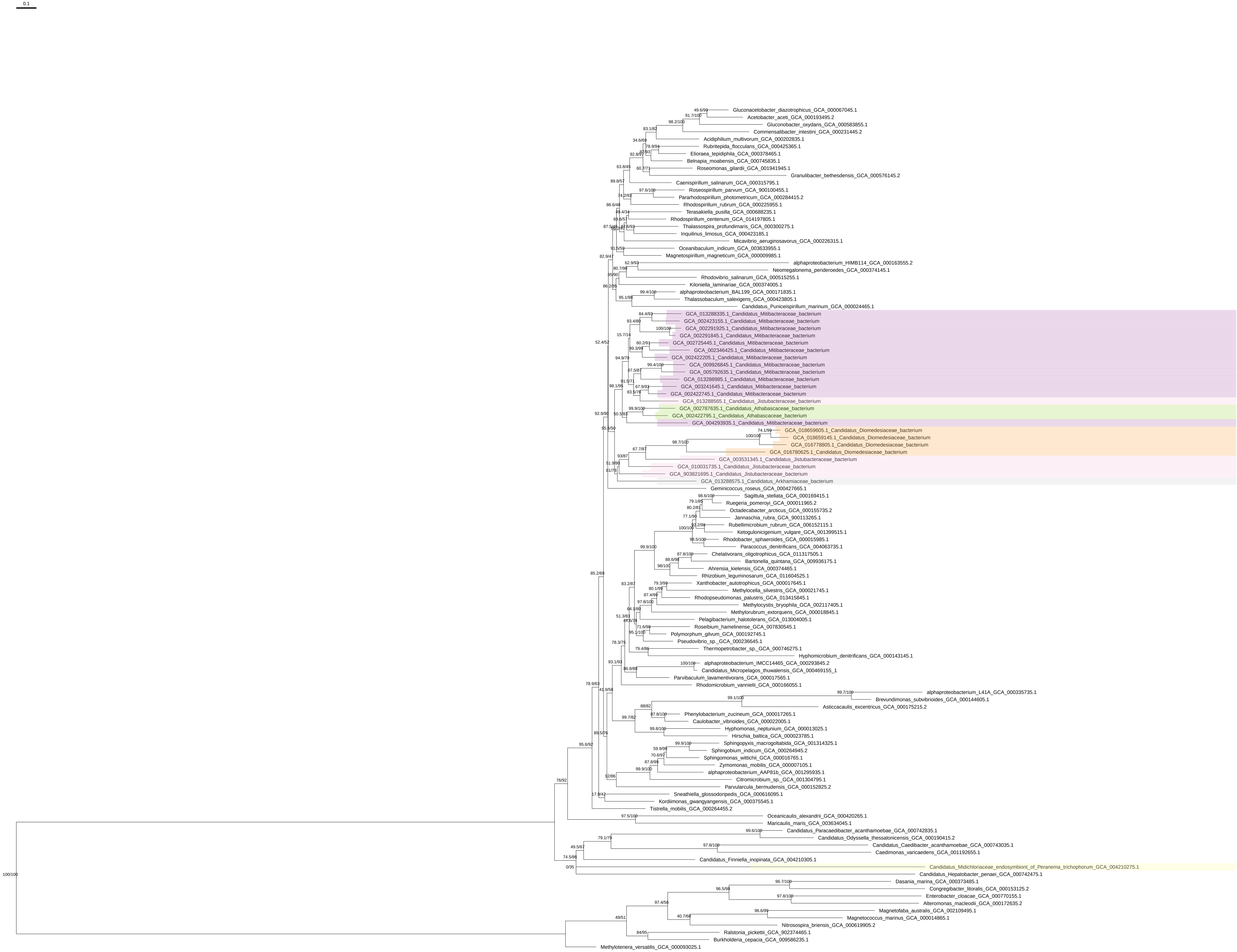

0.1
Gluconacetobacter_diazotrophicus_GCA_000067045.1
Acetobacter_aceti_GCA_000193495.2
Gluconobacter_oxydans_GCA_000583855.1
Commensalibacter_intestini_GCA_000231445.2
Acidiphilium_multivorum_GCA_000202835.1
Rubritepida_flocculans_GCA_000425365.1
Elioraea_tepidiphila_GCA_000378465.1
Belnapia_moabensis_GCA_000745835.1
Roseomonas_gilardii_GCA_001941945.1
Granulibacter_bethesdensis_GCA_000576145.2
Caenispirillum_salinarum_GCA_000315795.1
Roseospirillum_parvum_GCA_900100455.1
Pararhodospirillum_photometricum_GCA_000284415.2
Rhodospirillum_rubrum_GCA_000225955.1
Terasakiella_pusilla_GCA_000688235.1
Rhodospirillum_centenum_GCA_014197805.1
Thalassospira_profundimaris_GCA_000300275.1
Inquilinus_limosus_GCA_000423185.1
Micavibrio_aeruginosavorus_GCA_000226315.1
Oceanibaculum_indicum_GCA_003633955.1
Magnetospirillum_magneticum_GCA_000009985.1
alphaproteobacterium_HIMB114_GCA_000163555.2
Neomegalonema_perideroedes_GCA_000374145.1
Rhodovibrio_salinarum_GCA_000515255.1
Kiloniella_laminariae_GCA_000374005.1
alphaproteobacterium_BAL199_GCA_000171835.1
Thalassobaculum_salexigens_GCA_000423805.1
Candidatus_Puniceispirillum_marinum_GCA_000024465.1
GCA_013288335.1_Candidatus_Mitibacteraceae_bacterium
GCA_002423155.1_Candidatus_Mitibacteraceae_bacterium
GCA_002291925.1_Candidatus_Mitibacteraceae_bacterium
GCA_002291845.1_Candidatus_Mitibacteraceae_bacterium
GCA_002725445.1_Candidatus_Mitibacteraceae_bacterium
GCA_002346425.1_Candidatus_Mitibacteraceae_bacterium
GCA_002422205.1_Candidatus_Mitibacteraceae_bacterium
GCA_009926845.1_Candidatus_Mitibacteraceae_bacterium
GCA_005792635.1_Candidatus_Mitibacteraceae_bacterium
GCA_013288985.1_Candidatus_Mitibacteraceae_bacterium
GCA_003241645.1_Candidatus_Mitibacteraceae_bacterium
GCA_002422745.1_Candidatus_Mitibacteraceae_bacterium
GCA_013288565.1_Candidatus_Jistubacteraceae_bacterium
GCA_002787635.1_Candidatus_Athabascaceae_bacterium
GCA_002422795.1_Candidatus_Athabascaceae_bacterium
GCA_004293935.1_Candidatus_Mitibacteraceae_bacterium
GCA_018659605.1_Candidatus_Diomedesiaceae_bacterium
GCA_018659145.1_Candidatus_Diomedesiaceae_bacterium
GCA_016778805.1_Candidatus_Diomedesiaceae_bacterium
GCA_016780625.1_Candidatus_Diomedesiaceae_bacterium
GCA_003531345.1_Candidatus_Jistubacteraceae_bacterium
GCA_010031735.1_Candidatus_Jistubacteraceae_bacterium
GCA_903821695.1_Candidatus_Jistubacteraceae_bacterium
GCA_013288575.1_Candidatus_Arkhamiaceae_bacterium
Geminicoccus_roseus_GCA_000427665.1
Sagittula_stellata_GCA_000169415.1
Ruegeria_pomeroyi_GCA_000011965.2
Octadecabacter_arcticus_GCA_000155735.2
Jannaschia_rubra_GCA_900113265.1
Rubellimicrobium_rubrum_GCA_006152115.1
Ketogulonicigenium_vulgare_GCA_001399515.1
Rhodobacter_sphaeroides_GCA_000015985.1
Paracoccus_denitrificans_GCA_004063735.1
Chelativorans_oligotrophicus_GCA_011317505.1
Bartonella_quintana_GCA_009936175.1
Ahrensia_kielensis_GCA_000374465.1
Rhizobium_leguminosarum_GCA_011604525.1
Xanthobacter_autotrophicus_GCA_000017645.1
Methylocella_silvestris_GCA_000021745.1
Rhodopseudomonas_palustris_GCA_013415845.1
Methylocystis_bryophila_GCA_002117405.1
Methylorubrum_extorquens_GCA_000018845.1
Pelagibacterium_halotolerans_GCA_013004005.1
Roseibium_hamelinense_GCA_007830545.1
Polymorphum_gilvum_GCA_000192745.1
Pseudovibrio_sp._GCA_000236645.1
Thermopetrobacter_sp._GCA_000746275.1
Hyphomicrobium_denitrificans_GCA_000143145.1
alphaproteobacterium_IMCC14465_GCA_000293845.2
Candidatus_Micropelagos_thuwalensis_GCA_000469155_1
Parvibaculum_lavamentivorans_GCA_000017565.1
Rhodomicrobium_vannielii_GCA_000166055.1
alphaproteobacterium_L41A_GCA_000335735.1
Brevundimonas_subvibrioides_GCA_000144605.1
Asticcacaulis_excentricus_GCA_000175215.2
Phenylobacterium_zucineum_GCA_000017265.1
Caulobacter_vibrioides_GCA_000022005.1
Hyphomonas_neptunium_GCA_000013025.1
Hirschia_baltica_GCA_000023785.1
Sphingopyxis_macrogoltabida_GCA_001314325.1
Sphingobium_indicum_GCA_000264945.2
Sphingomonas_wittichii_GCA_000016765.1
Zymomonas_mobilis_GCA_000007105.1
alphaproteobacterium_AAP81b_GCA_001295935.1
Citromicrobium_sp._GCA_001304795.1
Parvularcula_bermudensis_GCA_000152825.2
Sneathiella_glossodoripedis_GCA_000616095.1
Kordiimonas_gwangyangensis_GCA_000375545.1
Tistrella_mobilis_GCA_000264455.2
Oceanicaulis_alexandrii_GCA_000420265.1
Maricaulis_maris_GCA_003634045.1
Candidatus_Paracaedibacter_acanthamoebae_GCA_000742835.1
Candidatus_Odyssella_thessalonicensis_GCA_000190415.2
Candidatus_Caedibacter_acanthamoebae_GCA_000743035.1
Caedimonas_varicaedens_GCA_001192655.1
Candidatus_Finniella_inopinata_GCA_004210305.1
Candidatus_Midichloriaceae_endosymbiont_of_Peranema_trichophorum_GCA_004210275.1
Candidatus_Hepatobacter_penaei_GCA_000742475.1
Dasania_marina_GCA_000373485.1
Congregibacter_litoralis_GCA_000153125.2
Enterobacter_cloacae_GCA_000770155.1
Alteromonas_macleodii_GCA_000172635.2
Magnetofaba_australis_GCA_002109495.1
Magnetococcus_marinus_GCA_000014865.1
Nitrosospira_briensis_GCA_000619905.2
Ralstonia_pickettii_GCA_902374465.1
Burkholderia_cepacia_GCA_009586235.1
Methylotenera_versatilis_GCA_000093025.1

Serine_z_0.50

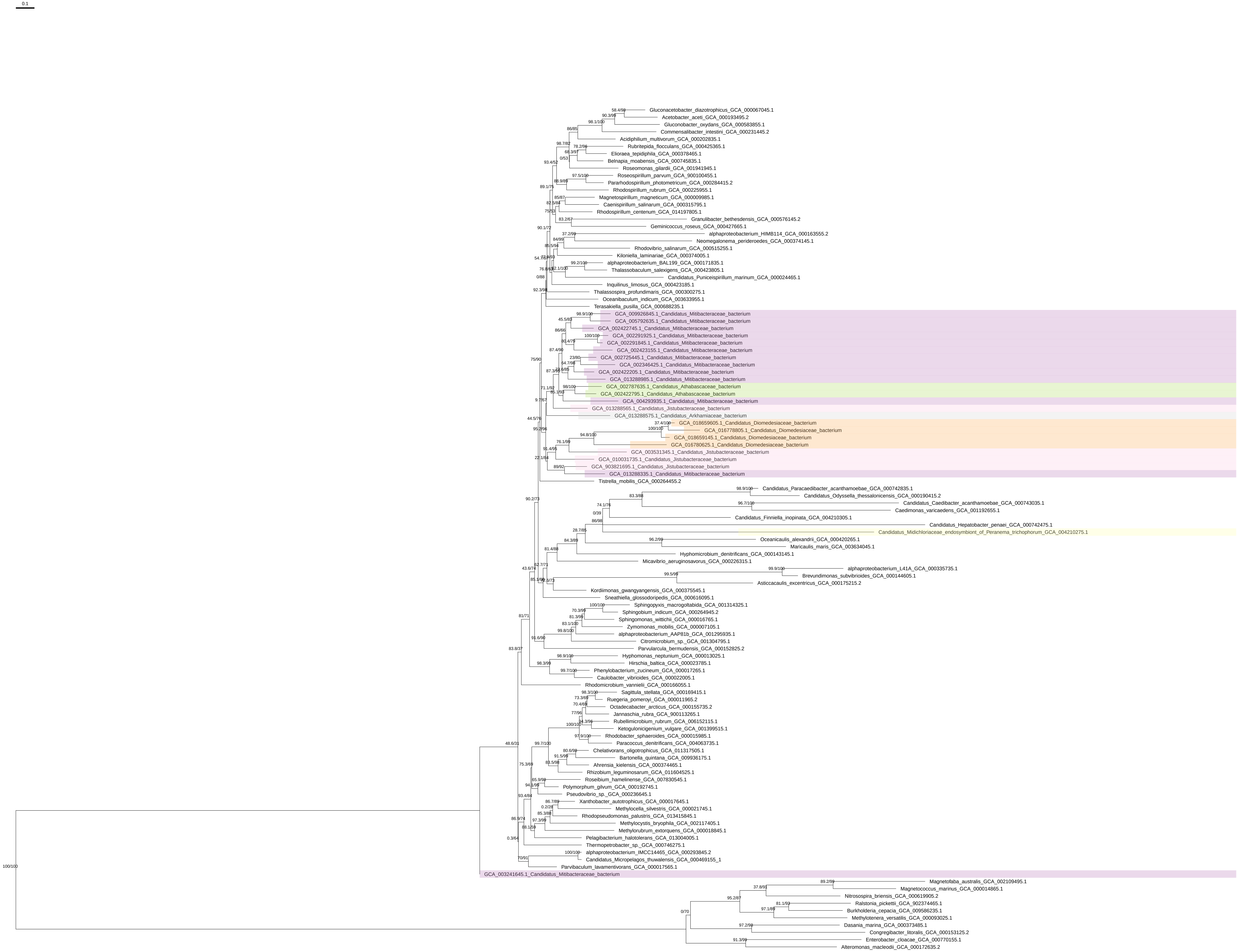
0.1
Gluconacetobacter_diazotrophicus_GCA_000067045.1
Acetobacter_aceti_GCA_000193495.2
Gluconobacter_oxydans_GCA_000583855.1
Commensalibacter_intestini_GCA_000231445.2
Acidiphilium_multivorum_GCA_000202835.1
Rubritepida_flocculans_GCA_000425365.1
Elioraea_tepidiphila_GCA_000378465.1
Belnapia_moabensis_GCA_000745835.1
Roseomonas_gilardii_GCA_001941945.1
Roseospirillum_parvum_GCA_900100455.1
Pararhodospirillum_photometricum_GCA_000284415.2
Rhodospirillum_rubrum_GCA_000225955.1
Magnetospirillum_magneticum_GCA_000009985.1
Caenispirillum_salinarum_GCA_000315795.1
Rhodospirillum_centenum_GCA_014197805.1
Granulibacter_bethesdensis_GCA_000576145.2
Geminicoccus_roseus_GCA_000427665.1
alphaproteobacterium_HIMB114_GCA_000163555.2
Neomegalonema_perideroedes_GCA_000374145.1
Rhodovibrio_salinarum_GCA_000515255.1
Kiloniella_laminariae_GCA_000374005.1
alphaproteobacterium_BAL199_GCA_000171835.1
Thalassobaculum_salexigens_GCA_000423805.1
Candidatus_Puniceispirillum_marinum_GCA_000024465.1
Inquilinus_limosus_GCA_000423185.1
Thalassospira_profundimaris_GCA_000300275.1
Oceanibaculum_indicum_GCA_003633955.1
Terasakiella_pusilla_GCA_000688235.1
GCA_009926845.1_Candidatus_Mitibacteraceae_bacterium
GCA_005792635.1_Candidatus_Mitibacteraceae_bacterium
GCA_002422745.1_Candidatus_Mitibacteraceae_bacterium
GCA_002291925.1_Candidatus_Mitibacteraceae_bacterium
GCA_002291845.1_Candidatus_Mitibacteraceae_bacterium
GCA_002423155.1_Candidatus_Mitibacteraceae_bacterium
GCA_002725445.1_Candidatus_Mitibacteraceae_bacterium
GCA_002346425.1_Candidatus_Mitibacteraceae_bacterium
GCA_002422205.1_Candidatus_Mitibacteraceae_bacterium
GCA_013288985.1_Candidatus_Mitibacteraceae_bacterium
GCA_002787635.1_Candidatus_Athabascaceae_bacterium
GCA_002422795.1_Candidatus_Athabascaceae_bacterium
GCA_004293935.1_Candidatus_Mitibacteraceae_bacterium
GCA_013288565.1_Candidatus_Jistubacteraceae_bacterium
GCA_013288575.1_Candidatus_Arkhamiaceae_bacterium
GCA_018659605.1_Candidatus_Diomedesiaceae_bacterium
GCA_016778805.1_Candidatus_Diomedesiaceae_bacterium
GCA_018659145.1_Candidatus_Diomedesiaceae_bacterium
GCA_016780625.1_Candidatus_Diomedesiaceae_bacterium
GCA_003531345.1_Candidatus_Jistubacteraceae_bacterium
GCA_010031735.1_Candidatus_Jistubacteraceae_bacterium
GCA_903821695.1_Candidatus_Jistubacteraceae_bacterium
GCA_013288335.1_Candidatus_Mitibacteraceae_bacterium
Tistrella_mobilis_GCA_000264455.2
Candidatus_Paracaedibacter_acanthamoebae_GCA_000742835.1
Candidatus_Odyssella_thessalonicensis_GCA_000190415.2
Candidatus_Caedibacter_acanthamoebae_GCA_000743035.1
Caedimonas_varicaedens_GCA_001192655.1
Candidatus_Finniella_inopinata_GCA_004210305.1
Candidatus_Hepatobacter_penaei_GCA_000742475.1
Candidatus_Midichloriaceae_endosymbiont_of_Peranema_trichophorum_GCA_004210275.1
Oceanicaulis_alexandrii_GCA_000420265.1
Maricaulis_maris_GCA_003634045.1
Hyphomicrobium_denitrificans_GCA_001143145.1
Micavibrio_aeruginosavorus_GCA_000226315.1
alphaproteobacterium_L41A_GCA_000335735.1
Brevundimonas_subvibrioides_GCA_000144605.1
Asticcacaulis_excentricus_GCA_000175215.2
Kordiimonas_gwangyangensis_GCA_000375545.1
Sneathiella_glossodoripedis_GCA_000616095.1
Sphingopyxis_macrogoltabida_GCA_001314325.1
Sphingobium_indicum_GCA_000264945.2
Sphingomonas_wittichii_GCA_000016765.1
Zymomonas_mobilis_GCA_000007105.1
alphaproteobacterium_AAP81b_GCA_001295935.1
Citromicrobium_sp._GCA_001304795.1
Parvularcula_bermudensis_GCA_000152825.2
Hyphomonas_neptunium_GCA_000013025.1
Hirschia_baltica_GCA_000023785.1
Phenylobacterium_zucineum_GCA_000017265.1
Caulobacter_vibrioides_GCA_000022005.1
Rhodomicrobium_vannielii_GCA_000166055.1
Sagittula_stellata_GCA_000169415.1
Ruegeria_pomeroyi_GCA_000011965.2
Octadecabacter_arcticus_GCA_000155735.2
Jannaschia_rubra_GCA_900113265.1
Rubellimicrobium_rubrum_GCA_006152115.1
Ketogulonicigenium_vulgare_GCA_001399515.1
Rhodobacter_sphaeroides_GCA_000015985.1
Paracoccus_denitrificans_GCA_004063735.1
Chelativorans_oligotrophicus_GCA_011317505.1
Bartonella_quintana_GCA_009936175.1
Ahrensia_kielensis_GCA_000374465.1
Rhizobium_leguminosarum_GCA_011604525.1
Roseibium_hamelinense_GCA_007830545.1
Polymorphum_gilvum_GCA_000192745.1
Pseudovibrio_sp._GCA_000236645.1
Xanthobacter_autotrophicus_GCA_000017645.1
Methylocella_silvestris_GCA_000021745.1
Rhodopseudomonas_palustris_GCA_013415845.1
Methylocystis_bryophila_GCA_002117405.1
Methylorubrum_extorquens_GCA_000018845.1
Pelagibacterium_halotolerans_GCA_013004005.1
Thermopetrobacter_sp._GCA_000746275.1
alphaproteobacterium_IMCC14465_GCA_000293845.2
Candidatus_Micropelagos_thuwalensis_GCA_000469155_1
Parvibaculum_lavamentivorans_GCA_000017565.1
GCA_003241645.1_Candidatus_Mitibacteraceae_bacterium
Magnetofaba_australis_GCA_002109495.1
Magnetococcus_marinus_GCA_000014865.1
Nitrosospira_briensis_GCA_000619905.2
Ralstonia_pickettii_GCA_902374465.1
Burkholderia_cepacia_GCA_009586235.1
Methylotenera_versatilis_GCA_000093025.1
Dasania_marina_GCA_000373485.1
Congregibacter_litoralis_GCA_000153125.2
Enterobacter_cloacae_GCA_000770155.1
Alteromonas_macleodii_GCA_000172635.2

Trp_Tyr_Phe

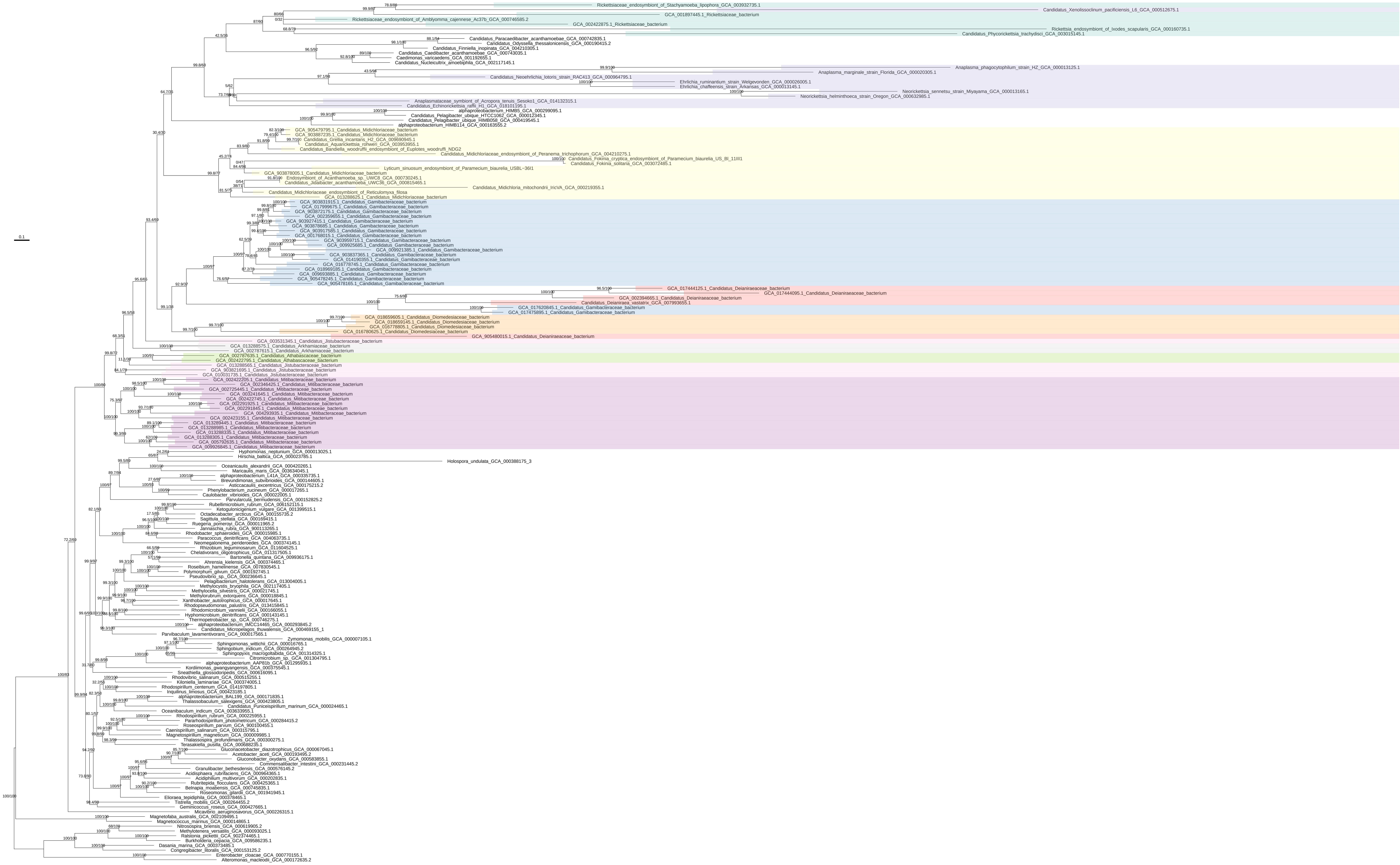
0.1
Rickettsiaceae_endosymbiont_of_Stachyamoeba_lipophora_GCA_003932735.1
Candidatus_Xenolissoclinum_pacificiensis_L6_GCA_000512675.1
GCA_001897445.1_Rickettsiaceae_bacterium
Rickettsiaceae_endosymbiont_of_Amblyomma_cajennese_Ac37b_GCA_000746585.2
GCA_002422875.1_Rickettsiaceae_bacterium
Rickettsia_endosymbiont_of_Ixodes_scapularis_GCA_000160735.1
Candidatus_Phycorickettsia_trachydisci_GCA_003015145.1
Candidatus_Paracaedibacter_acanthamoebae_GCA_000742835.1
Candidatus_Odyssella_thessalonicensis_GCA_000190415.2
Candidatus_Finniella_inopinata_GCA_004210305.1
Candidatus_Caedibacter_acanthamoebae_GCA_000743035.1
Caedimonas_varicaedens_GCA_001192655.1
Candidatus_Nucleicultrix_amoebiphila_GCA_002117145.1
Anaplasma_phagocytophilum_strain_HZ_GCA_000013125.1
Anaplasma_marginale_strain_Florida_GCA_000020305.1
Candidatus_Neoehrlichia_lotoris_strain_RAC413_GCA_000964795.1
Ehrlichia_ruminantium_strain_Welgevonden_GCA_000026005.1
Ehrlichia_chaffeensis_strain_Arkansas_GCA_000013145.1
Neorickettsia_sennetsu_strain_Miyayama_GCA_000013165.1
Neorickettsia_helminthoeca_strain_Oregon_GCA_000632985.1
Anaplasmataceae_symbiont_of_Acropora_tenuis_Sesoko1_GCA_014132315.1
Candidatus_Echinorickettsia_raffii_H1_GCA_018101195.1
alphaproteobacterium_HIMB5_GCA_000299095.1
Candidatus_Pelagibacter_ubique_HTCC1062_GCA_000012345.1
Candidatus_Pelagibacter_ubique_HIMB058_GCA_000419545.1
alphaproteobacterium_HIMB114_GCA_000163555.2
GCA_905479795.1_Candidatus_Midichloriaceae_bacterium
GCA_903887235.1_Candidatus_Midichloriaceae_bacterium
Candidatus_Grellia_incantans_H2_GCA_009690945.1
Candidatus_Aquarickettsia_rohweri_GCA_003953955.1
Candidatus_Bandiella_woodruffii_endosymbiont_of_Euplotes_woodruffi_NDG2
Candidatus_Midichloriaceae_endosymbiont_of_Peranema_trichophorum_GCA_004210275.1
Candidatus_Fokinia_cryptica_endosymbiont_of_Paramecium_biaurelia_US_Bl_11III1
Candidatus_Fokinia_solitaria_GCA_003072485.1
Lyticum_sinuosum_endosymbiont_of_Paramecium_biaurelia_USBL-36I1
GCA_903878005.1_Candidatus_Midichloriaceae_bacterium
Endosymbiont_of_Acanthamoeba_sp._UWC8_GCA_000730245.1
Candidatus_Jidaibacter_acanthamoeba_UWC36_GCA_000815465.1
Candidatus_Midichloria_mitochondrii_IricVA_GCA_000219355.1
Candidatus_Midichloriaceae_endosymbiont_of_Reticulomyxa_filosa
GCA_013288625.1_Candidatus_Midichloriaceae_bacterium
GCA_903831915.1_Candidatus_Gamibacteraceae_bacterium
GCA_017999675.1_Candidatus_Gamibacteraceae_bacterium
GCA_903872175.1_Candidatus_Gamibacteraceae_bacterium
GCA_002359655.1_Candidatus_Gamibacteraceae_bacterium
GCA_903927415.1_Candidatus_Gamibacteraceae_bacterium
GCA_903878685.1_Candidatus_Gamibacteraceae_bacterium
GCA_903917585.1_Candidatus_Gamibacteraceae_bacterium
GCA_001768015.1_Candidatus_Gamibacteraceae_bacterium
GCA_903959715.1_Candidatus_Gamibacteraceae_bacterium
GCA_009925685.1_Candidatus_Gamibacteraceae_bacterium
GCA_009921385.1_Candidatus_Gamibacteraceae_bacterium
GCA_903837365.1_Candidatus_Gamibacteraceae_bacterium
GCA_014190355.1_Candidatus_Gamibacteraceae_bacterium
GCA_016778745.1_Candidatus_Gamibacteraceae_bacterium
GCA_018969185.1_Candidatus_Gamibacteraceae_bacterium
GCA_009693885.1_Candidatus_Gamibacteraceae_bacterium
GCA_905478245.1_Candidatus_Gamibacteraceae_bacterium
GCA_905478165.1_Candidatus_Gamibacteraceae_bacterium
GCA_017444125.1_Candidatus_Deianiraeaceae_bacterium
GCA_017444095.1_Candidatus_Deianiraeaceae_bacterium
GCA_002394665.1_Candidatus_Deianiraeaceae_bacterium
Candidatus_Deianiraea_vastatrix_GCA_007993655.1
GCA_017620845.1_Candidatus_Gamibacteraceae_bacterium
GCA_017475895.1_Candidatus_Gamibacteraceae_bacterium
GCA_018659605.1_Candidatus_Diomedesiaceae_bacterium
GCA_018659145.1_Candidatus_Diomedesiaceae_bacterium
GCA_016778805.1_Candidatus_Diomedesiaceae_bacterium
GCA_016780625.1_Candidatus_Diomedesiaceae_bacterium
GCA_905480015.1_Candidatus_Deianiraeaceae_bacterium
GCA_003531345.1_Candidatus_Jistubacteraceae_bacterium
GCA_013288575.1_Candidatus_Arkhamiaceae_bacterium
GCA_002787615.1_Candidatus_Arkhamiaceae_bacterium
GCA_002787635.1_Candidatus_Athabascaceae_bacterium
GCA_002422795.1_Candidatus_Athabascaceae_bacterium
GCA_013288565.1_Candidatus_Jistubacteraceae_bacterium
GCA_903821695.1_Candidatus_Jistubacteraceae_bacterium
GCA_010031735.1_Candidatus_Jistubacteraceae_bacterium
GCA_002422205.1_Candidatus_Mitibacteraceae_bacterium
GCA_002346425.1_Candidatus_Mitibacteraceae_bacterium
GCA_002725445.1_Candidatus_Mitibacteraceae_bacterium
GCA_003241645.1_Candidatus_Mitibacteraceae_bacterium
GCA_002422745.1_Candidatus_Mitibacteraceae_bacterium
GCA_002291925.1_Candidatus_Mitibacteraceae_bacterium
GCA_002291845.1_Candidatus_Mitibacteraceae_bacterium
GCA_004293935.1_Candidatus_Mitibacteraceae_bacterium
GCA_002423155.1_Candidatus_Mitibacteraceae_bacterium
GCA_013289445.1_Candidatus_Mitibacteraceae_bacterium
GCA_013288985.1_Candidatus_Mitibacteraceae_bacterium
GCA_013288335.1_Candidatus_Mitibacteraceae_bacterium
GCA_013288305.1_Candidatus_Mitibacteraceae_bacterium
GCA_005792635.1_Candidatus_Mitibacteraceae_bacterium
GCA_009926845.1_Candidatus_Mitibacteraceae_bacterium
Hyphomonas_neptunium_GCA_000013025.1
Hirschia_baltica_GCA_000023785.1
Holospora_undulata_GCA_000388175_3
Oceanicaulis_alexandrii_GCA_000420265.1
Maricaulis_maris_GCA_003634045.1
alphaproteobacterium_L41A_GCA_000335735.1
Brevundimonas_subvibrioides_GCA_000144605.1
Asticcacaulis_excentricus_GCA_000175215.2
Phenylobacterium_zucineum_GCA_000017265.1
Caulobacter_vibrioides_GCA_000022005.1
Parvularcula_bermudensis_GCA_000152825.2
Rubellimicrobium_rubrum_GCA_006152115.1
Ketogulonicigenium_vulgare_GCA_001399515.1
Octadecabacter_arcticus_GCA_000155735.2
Sagittula_stellata_GCA_000169415.1
Ruegeria_pomeroyi_GCA_000011965.2
Jannaschia_rubra_GCA_900113265.1
Rhodobacter_sphaeroides_GCA_000015985.1
Paracoccus_denitrificans_GCA_004063735.1
Neomegalonema_perideroedes_GCA_000374145.1
Rhizobium_leguminosarum_GCA_011604525.1
Chelativorans_oligotrophicus_GCA_011317505.1
Bartonella_quintana_GCA_009936175.1
Ahrensia_kielensis_GCA_000374465.1
Roseibium_hamelinense_GCA_007830545.1
Polymorphum_gilvum_GCA_000192745.1
Pseudovibrio_sp._GCA_000236645.1
Pelagibacterium_halotolerans_GCA_013004005.1
Methylocystis_bryophila_GCA_002117405.1
Methylocella_silvestris_GCA_000021745.1
Methylorubrum_extorquens_GCA_000018845.1
Xanthobacter_autotrophicus_GCA_000017645.1
Rhodopseudomonas_palustris_GCA_013415845.1
Rhodomicrobium_vannielii_GCA_000166055.1
Hyphomicrobium_denitrificans_GCA_000143145.1
Thermopetrobacter_sp._GCA_000746275.1
alphaproteobacterium_IMCC14465_GCA_000293845.2
Candidatus_Micropelagos_thuwalensis_GCA_000469155_1
Parvibaculum_lavamentivorans_GCA_000017565.1
Zymomonas_mobilis_GCA_000007105.1
Sphingomonas_wittichii_GCA_000016765.1
Sphingobium_indicum_GCA_000264945.2
Sphingopyxis_macrogoltabida_GCA_001314325.1
Citromicrobium_sp._GCA_001304795.1
alphaproteobacterium_AAP81b_GCA_001295935.1
Kordiimonas_gwangyangensis_GCA_000375545.1
Sneathiella_glossodoripedis_GCA_000616095.1
Rhodovibrio_salinarum_GCA_000515255.1
Kiloniella_laminariae_GCA_000374005.1
Rhodospirillum_centenum_GCA_014197805.1
Inquilinus_limosus_GCA_000423185.1
alphaproteobacterium_BAL199_GCA_000171835.1
Thalassobaculum_salexigens_GCA_000423805.1
Candidatus_Puniceispirillum_marinum_GCA_000024465.1
Oceanibaculum_indicum_GCA_003633955.1
Rhodospirillum_rubrum_GCA_000225955.1
Pararhodospirillum_photometricum_GCA_000284415.2
Roseospirillum_parvum_GCA_900100455.1
Caenispirillum_salinarum_GCA_000315795.1
Magnetospirillum_magneticum_GCA_000009985.1
Thalassospira_profundimaris_GCA_000300275.1
Terasakiella_pusilla_GCA_000688235.1
Gluconacetobacter_diazotrophicus_GCA_000067045.1
Acetobacter_aceti_GCA_000193495.2
Gluconobacter_oxydans_GCA_000583855.1
Commensalibacter_intestini_GCA_000231445.2
Granulibacter_bethesdensis_GCA_000576145.2
Acidisphaera_rubrifaciens_GCA_000964365.1
Acidiphilium_multivorum_GCA_000202835.1
Rubritepida_flocculans_GCA_000425365.1
Belnapia_moabensis_GCA_000745835.1
Roseomonas_gilardii_GCA_001941945.1
Elioraea_tepidiphila_GCA_000378465.1
Tistrella_mobilis_GCA_000264455.2
Geminicoccus_roseus_GCA_000427665.1
Micavibrio_aeruginosavorus_GCA_000226315.1
Magnetofaba_australis_GCA_002109495.1
Magnetococcus_marinus_GCA_000014865.1
Nitrosospira_briensis_GCA_000619905.2
Methylotenera_versatilis_GCA_000093025.1
Ralstonia_pickettii_GCA_002374465.1
Burkholderia_cepacia_GCA_009586235.1
Dasania_marina_GCA_000373485.1
Congregibacter_litoralis_GCA_000153125.2
Enterobacter_cloacae_GCA_000770155.1
Alteromonas_macleodii_GCA_000172635.2

Trp_Tyr_Phe_z_0.10

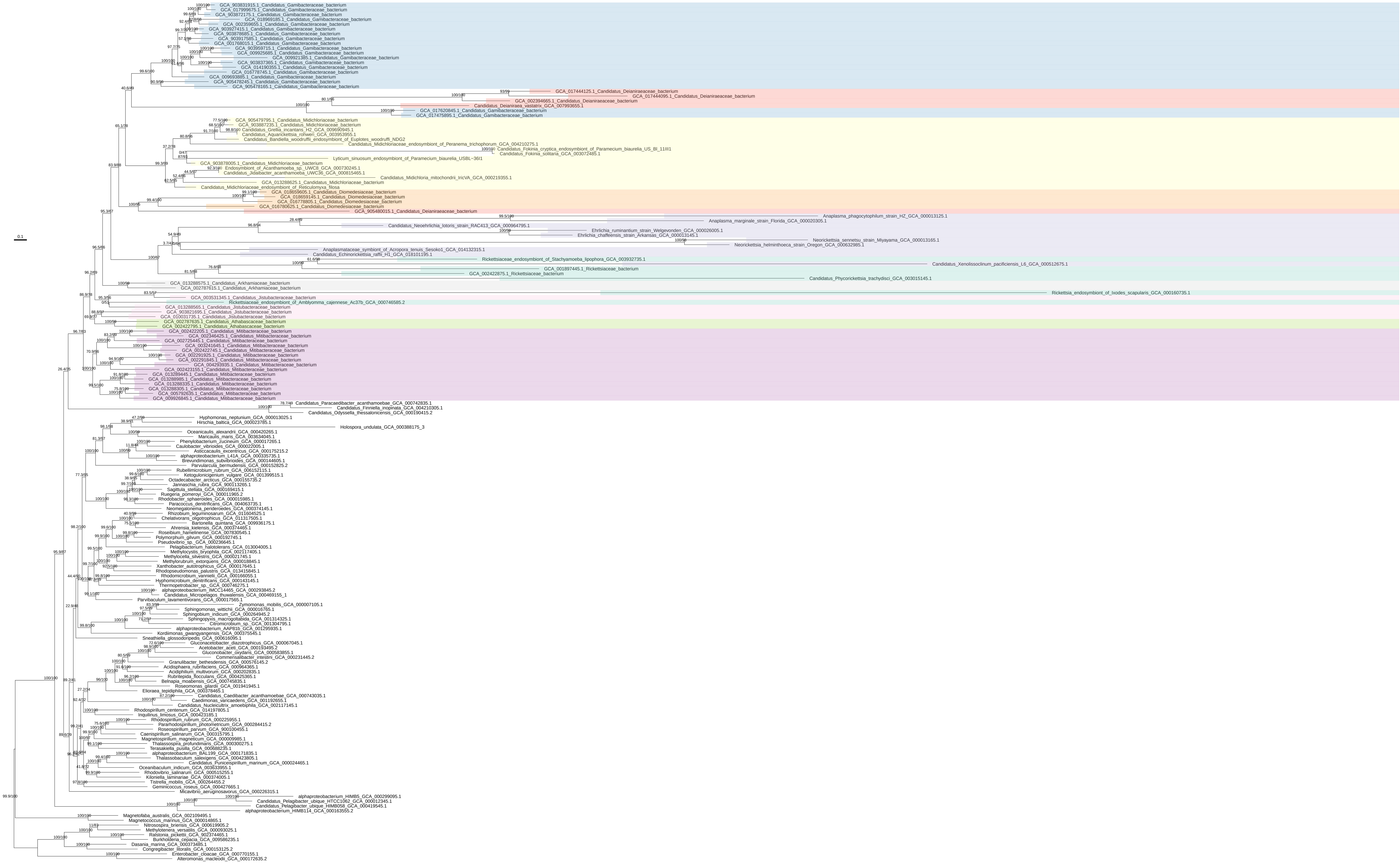

Trp_Tyr_Phe_z_0.20

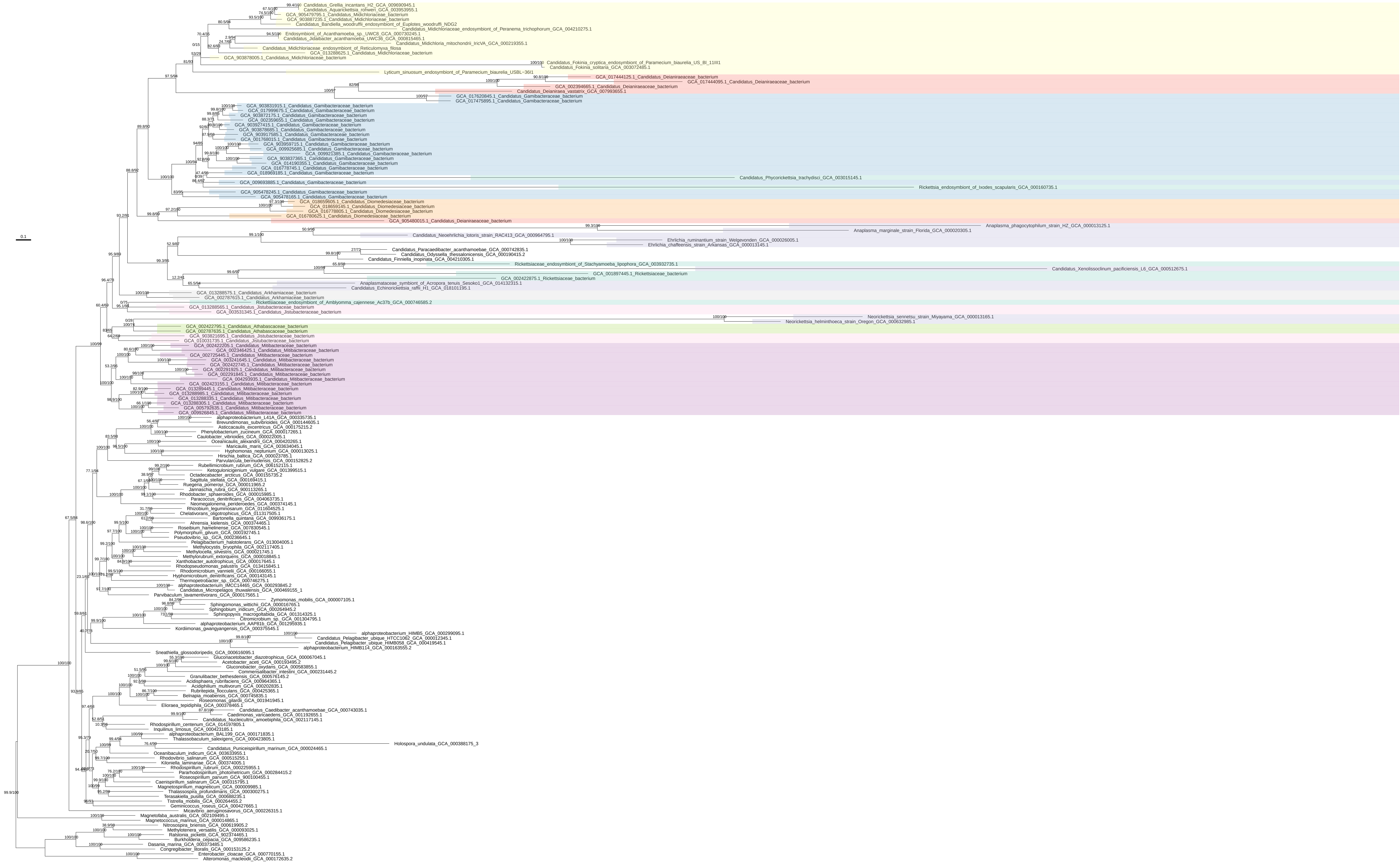

Trp_Tyr_Phe_z_0.30

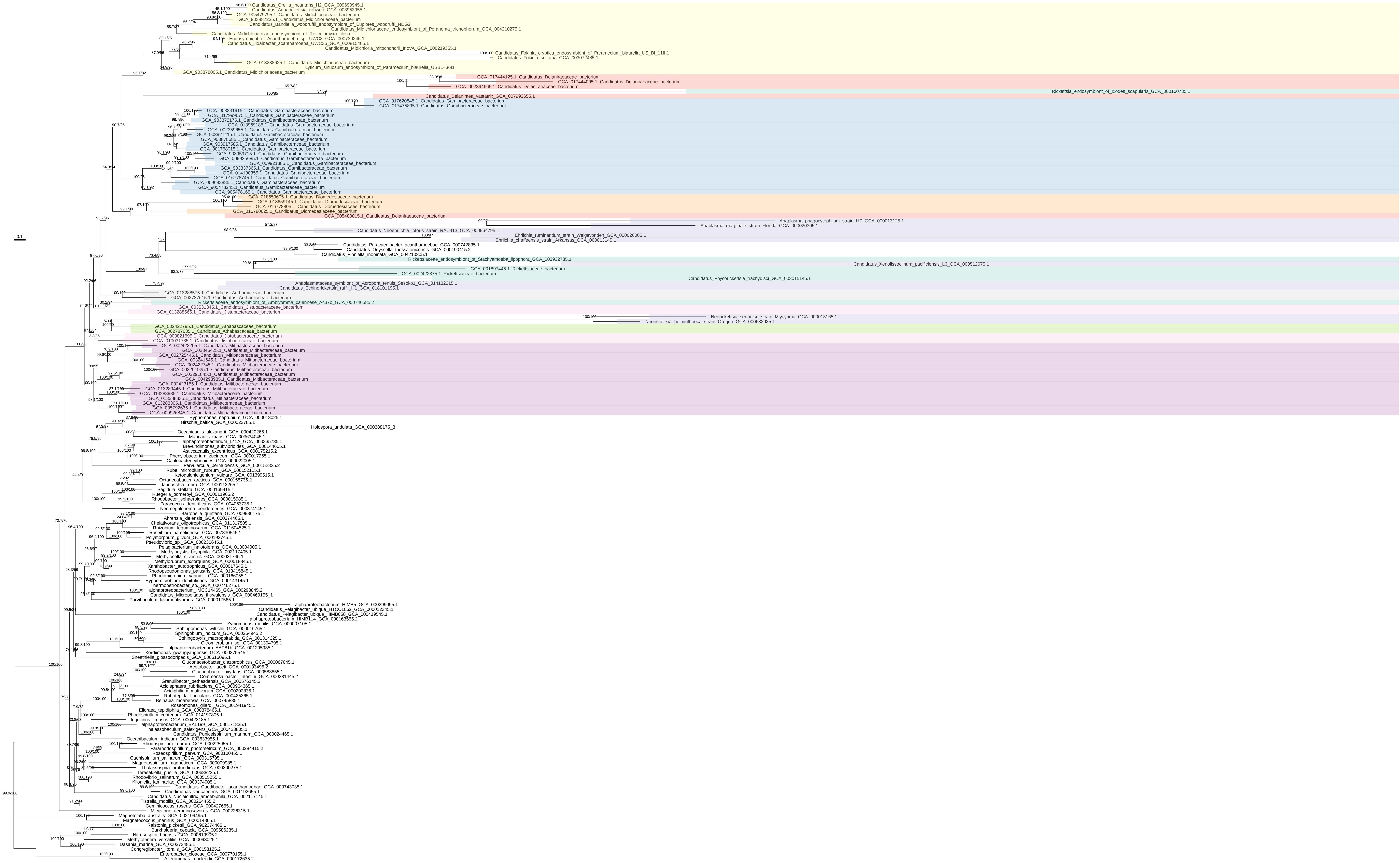

Trp_Tyr_Phe_z_0.40

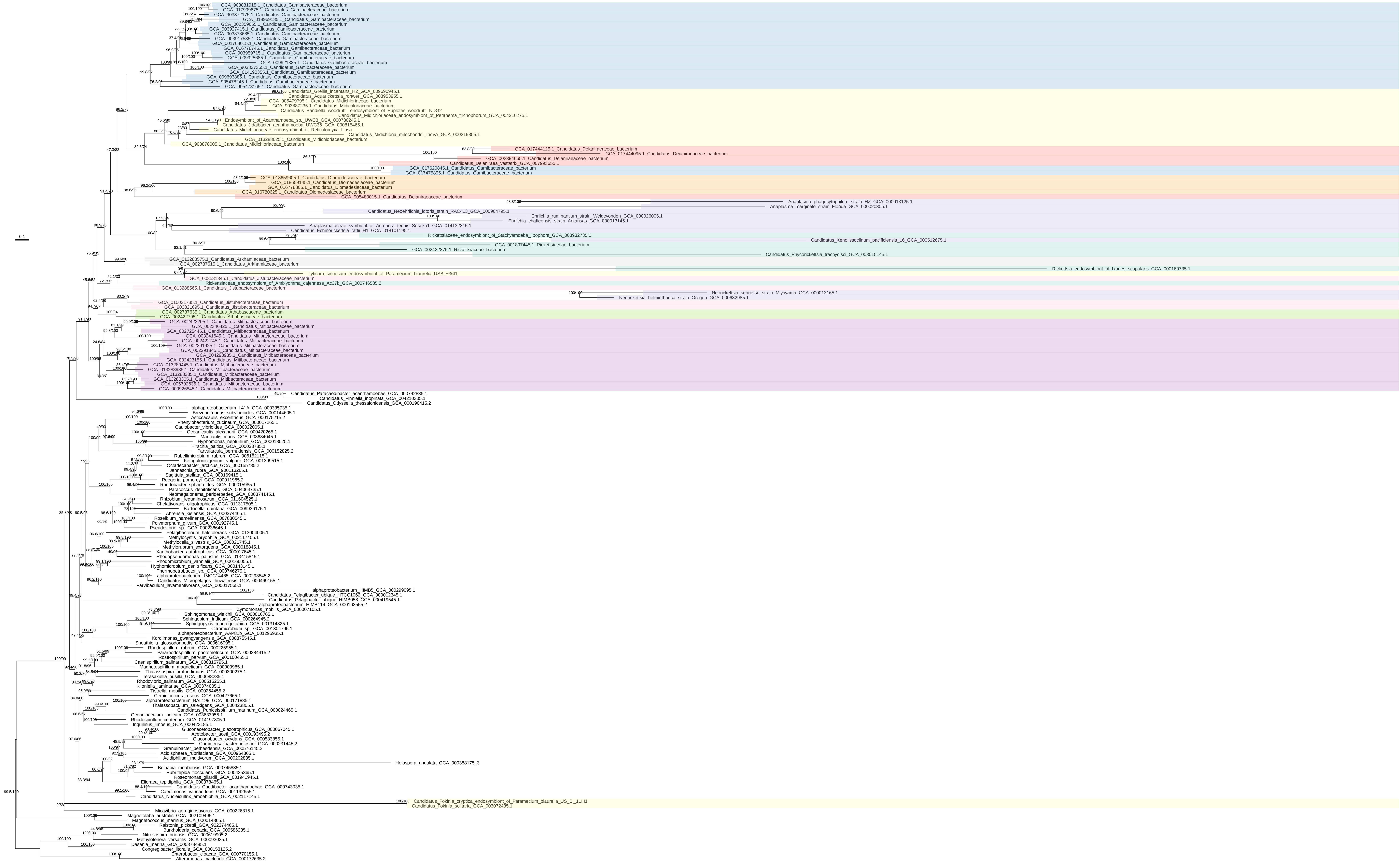

Trp_Tyr_Phe_z_0.50

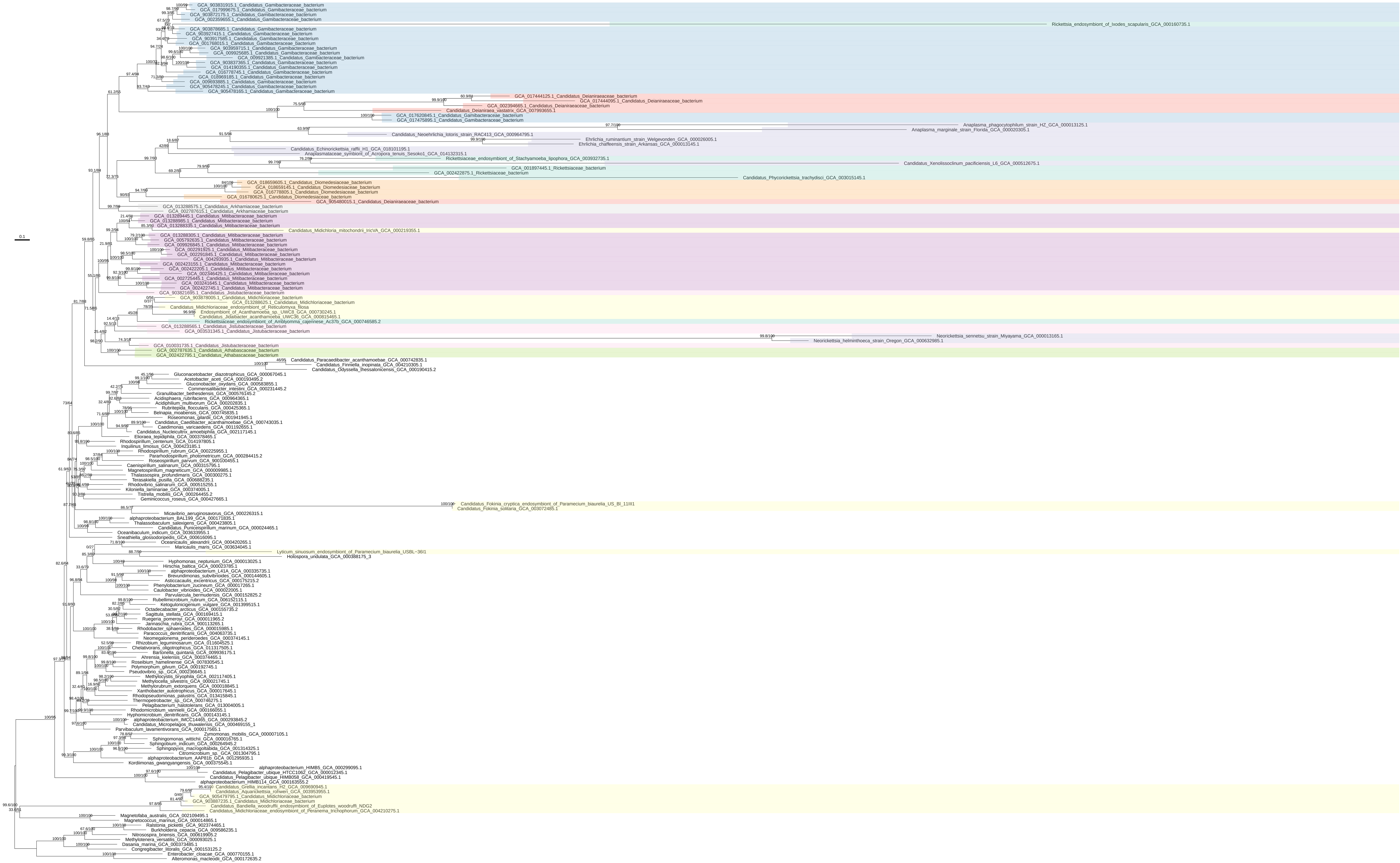

Supplementary Figure 27: phylogenetic tree of the tlc gene family. The members of each *Rickettsiales* family from the dataset of this study are highlighted by different background colours. On each branch, support values by SH-aLRT with 1000 replicates and by 1000 ultrafast bootstraps are reported. The tree scale stands for estimated sequence divergence. The branches on which the ten maximal number of predicted events of HGT with some *Rickettsiales* as recipients are coloured in red.

ADP/ATP translocase

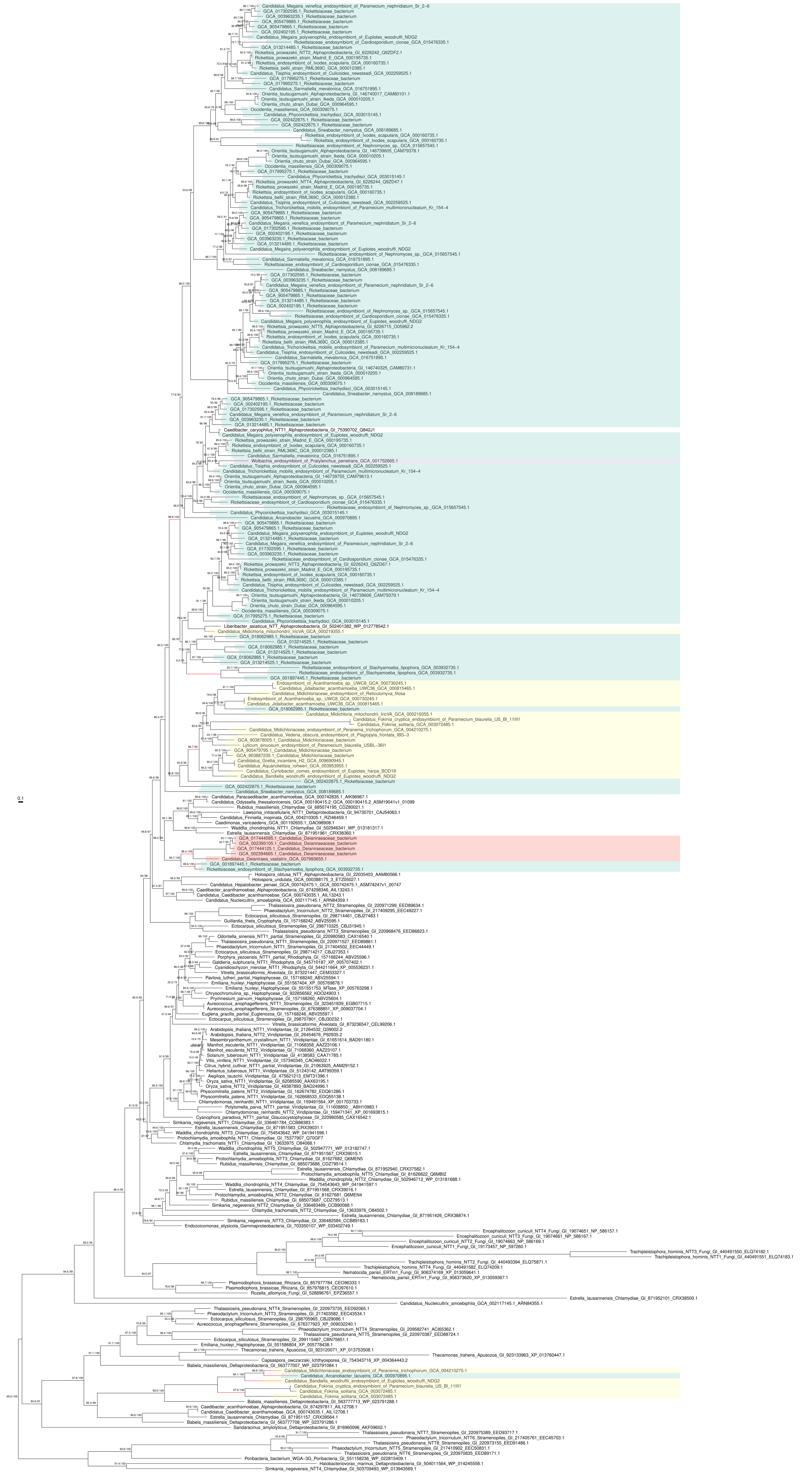

Supplementary Figure 28: biosynthetic pathways and transporters for amino acids. Heat-map showing the presence and abundance of biosynthetic pathways of amino acids (grouped by to their mutually shared enzymatic steps according to BioCyc; blue) and their respective transporters (red) in *Rickettsiales*. Transporters are divided into "characterised" and "uncharacterised" hits, based on whether the corresponding TCDB entry have an experimentally characterised specificity for the given amino acids, or are representatives of a transporter family including amino acid transporters, but their own specificity was not ascertained. For biosynthesis, the proportion of the total genes of the pathway is shown (Supplementary Data 7), while for transporters, the number of genes is reported. Organisms are ordered by their phylogenetic relationships (Supplementary Figure 18), with families highlighted by different colours on the right. The bar plots on the right indicate the percentage of proteobacterial BUSCO single-copy orthogroups identified in each assembly.

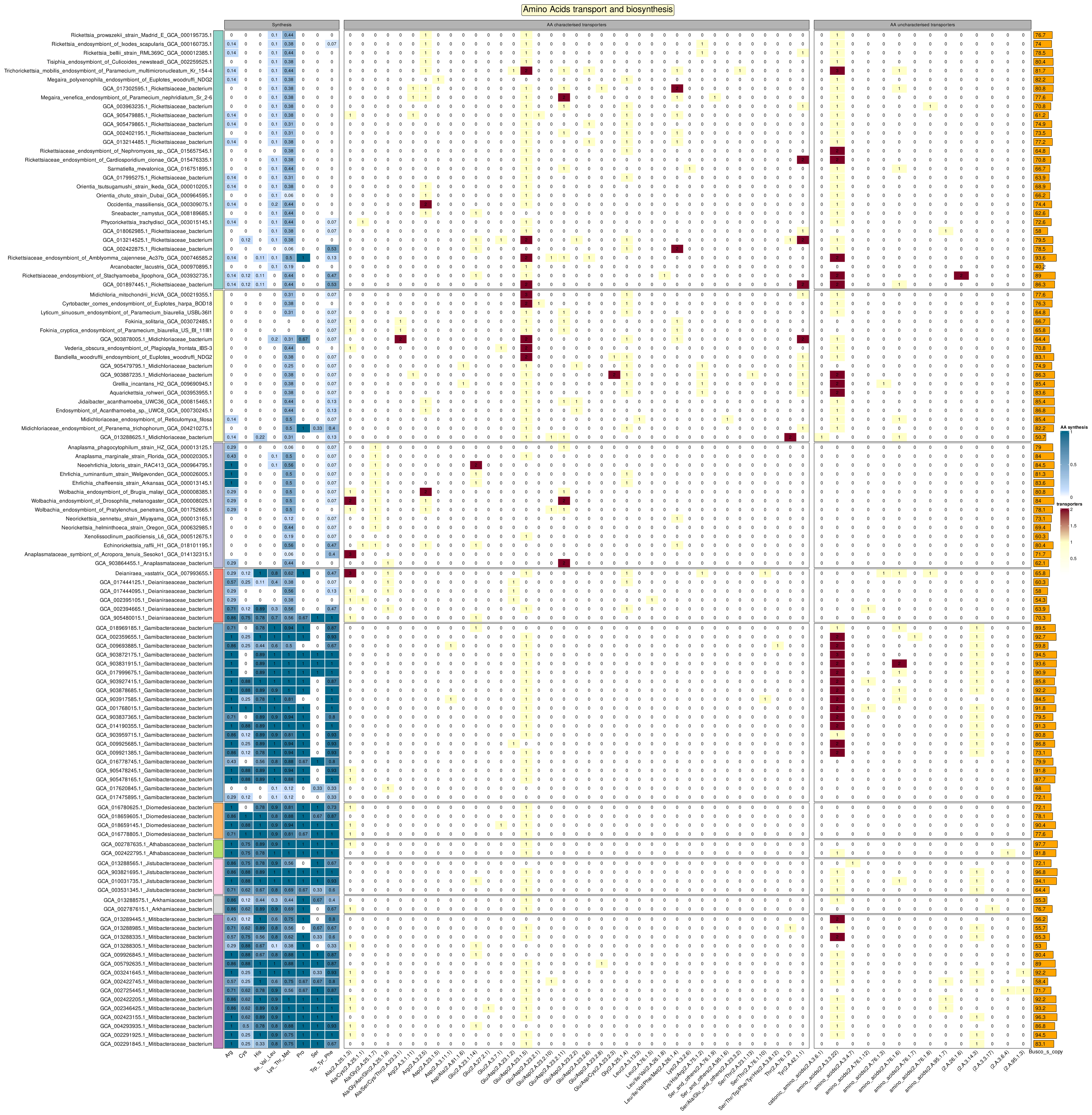

Amino Acids transport and biosynthesis
Synthesis
AA characterised transporters
AA uncharacterised transporters
Rickettsia_prowazekii_strain_Madrid_E_GCA_000195735.1
Rickettsia_endosymbiont_of_Ixodes_scapularis_GCA_000160735.1
Rickettsia_bellii_strain_RML369C_GCA_000012385.1
Tisiphia_endosymbiont_of_Culicoides_newsteadi_GCA_002259525.1
Trichorickettsia_mobilis_endosymbiont_of_Paramecium_multimicronucleatum_Kr_154-4
Megaira_polyxenophila_endosymbiont_of_Euplotes_woodruffi_NDG2
GCA_017302595.1_Rickettsiaceae_bacterium
Megaira_venefica_endosymbiont_of_Paramecium_nephridiatum_Sr_2-6
GCA_003963235.1_Rickettsiaceae_bacterium
GCA_905479885.1_Rickettsiaceae_bacterium
GCA_905479865.1_Rickettsiaceae_bacterium
GCA_002402195.1_Rickettsiaceae_bacterium
GCA_013214485.1_Rickettsiaceae_bacterium
Rickettsiaceae_endosymbiont_of_Nephromyces_sp._GCA_015657545.1
Rickettsiaceae_endosymbiont_of_Cardiosporidium_cionae_GCA_015476335.1
Sarmatiella_mevalonica_GCA_016751895.1
GCA_017995275.1_Rickettsiaceae_bacterium
Orientia_tsutsugamushi_strain_Ikeda_GCA_000010205.1
Orientia_chuto_strain_Dubai_GCA_000964595.1
Occidentia_massiliensis_GCA_000309075.1
Sneabacter_namystus_GCA_008189685.1
Phycorickettsia_trachydisci_GCA_003015145.1
GCA_018062985.1_Rickettsiaceae_bacterium
GCA_013214525.1_Rickettsiaceae_bacterium
GCA_002422875.1_Rickettsiaceae_bacterium
Rickettsiaceae_endosymbiont_of_Amblyomma_cajennese_Ac37b_GCA_007746585.2
Arcanobacter_lacustris_GCA_000970895.1
Rickettsiaceae_endosymbiont_of_Stachyamoeba_lipophora_GCA_003932735.1
GCA_001897445.1_Rickettsiaceae_bacterium
Midichloria_mitochondrii_IricVA_GCA_000219355.1
Cyrtobacter_comes_endosymbiont_of_Euplotes_harpa_BOD18
Lyticum_sinuosum_endosymbiont_of_Paramecium_biaurelia_USBL-36I1
Fokinia_solitaria_GCA_003072485.1
Fokinia_cryptica_endosymbiont_of_Paramecium_biaurelia_US_Bl_11III1
GCA_903878005.1_Midichloriaceae_bacterium
Vederia_obscura_endosymbiont_of_Plagiopyla_frontata_IBS-3
Bandiella_woodruffii_endosymbiont_of_Euplotes_woodruffi_NDG2
GCA_905479795.1_Midichloriaceae_bacterium
GCA_903887235.1_Midichloriaceae_bacterium
Grellia_incantans_H2_GCA_009690945.1
Aquarickettsia_rohweri_GCA_003953955.1
Jidaibacter_acanthamoeba_UWC36_GCA_000815465.1
Endosymbiont_of_Acanthamoeba_sp._UWC8_GCA_000730245.1
Midichloriaceae_endosymbiont_of_Reticulomyxa_filosa
Midichloriaceae_endosymbiont_of_Peranema_trichophorum_GCA_004210275.1
GCA_013288625.1_Midichloriaceae_bacterium
Anaplasma_phagocytophilum_strain_HZ_GCA_000013125.1
Anaplasma_marginale_strain_Florida_GCA_000020305.1
Neoehrlichia_lotoris_strain_RAC413_GCA_000964795.1
Ehrlichia_ruminantium_strain_Welgevonden_GCA_000026005.1
Ehrlichia_chaffeensis_strain_Arkansas_GCA_000013145.1
Wolbachia_endosymbiont_of_Brugia_malayi_GCA_000008385.1
Wolbachia_endosymbiont_of_Drosophila_melanogaster_GCA_000008025.1
Wolbachia_endosymbiont_of_Pratylenchus_penetrans_GCA_001752665.1
Neorickettsia_sennetsu_strain_Miyayama_GCA_000013165.1
Neorickettsia_helminthoeca_strain_Oregon_GCA_000632985.1
Xenolissoclinum_pacificiensis_L6_GCA_000512675.1
Echinorickettsia_raffii_H1_GCA_018101195.1
Anaplasmataceae_symbiont_of_Acropora_tenuis_Sesoko1_GCA_014132315.1
GCA_903864455.1_Anaplasmataceae_bacterium
Deianiraea_vastatrix_GCA_007993655.1
GCA_017444125.1_Deianiraeaceae_bacterium
GCA_017444095.1_Deianiraeaceae_bacterium
GCA_002395105.1_Deianiraeaceae_bacterium
GCA_002394665.1_Deianiraeaceae_bacterium
GCA_905480015.1_Deianiraeaceae_bacterium
GCA_018969185.1_Gamibacteraceae_bacterium
GCA_002359655.1_Gamibacteraceae_bacterium
GCA_009693885.1_Gamibacteraceae_bacterium
GCA_903872175.1_Gamibacteraceae_bacterium
GCA_903831915.1_Gamibacteraceae_bacterium
GCA_017999675.1_Gamibacteraceae_bacterium
GCA_903927415.1_Gamibacteraceae_bacterium
GCA_903878685.1_Gamibacteraceae_bacterium
GCA_903917585.1_Gamibacteraceae_bacterium
GCA_001768015.1_Gamibacteraceae_bacterium
GCA_903837365.1_Gamibacteraceae_bacterium
GCA_014190355.1_Gamibacteraceae_bacterium
GCA_903959715.1_Gamibacteraceae_bacterium
GCA_009925685.1_Gamibacteraceae_bacterium
GCA_009921385.1_Gamibacteraceae_bacterium
GCA_016778745.1_Gamibacteraceae_bacterium
GCA_905478245.1_Gamibacteraceae_bacterium
GCA_905478165.1_Gamibacteraceae_bacterium
GCA_017620845.1_Gamibacteraceae_bacterium
GCA_017475895.1_Gamibacteraceae_bacterium
GCA_016780625.1_Diomedesiaceae_bacterium
GCA_018659605.1_Diomedesiaceae_bacterium
GCA_018659145.1_Diomedesiaceae_bacterium
GCA_016778805.1_Diomedesiaceae_bacterium
GCA_002787635.1_Athabascaceae_bacterium
GCA_002422795.1_Athabascaceae_bacterium
GCA_013288565.1_Jistubacteraceae_bacterium
GCA_903821695.1_Jistubacteraceae_bacterium
GCA_010031735.1_Jistubacteraceae_bacterium
GCA_003531345.1_Jistubacteraceae_bacterium
GCA_013288575.1_Arkhamiaceae_bacterium
GCA_002787615.1_Arkhamiaceae_bacterium
GCA_013289445.1_Mitibacteraceae_bacterium
GCA_013288985.1_Mitibacteraceae_bacterium
GCA_013288335.1_Mitibacteraceae_bacterium
GCA_013288305.1_Mitibacteraceae_bacterium
GCA_009926845.1_Mitibacteraceae_bacterium
GCA_005792635.1_Mitibacteraceae_bacterium
GCA_003241645.1_Mitibacteraceae_bacterium
GCA_002422745.1_Mitibacteraceae_bacterium
GCA_002725445.1_Mitibacteraceae_bacterium
GCA_002422205.1_Mitibacteraceae_bacterium
GCA_002346425.1_Mitibacteraceae_bacterium
GCA_002423155.1_Mitibacteraceae_bacterium
GCA_004293935.1_Mitibacteraceae_bacterium
GCA_002291925.1_Mitibacteraceae_bacterium
GCA_002291845.1_Mitibacteraceae_bacterium
Arg
Cys
His
Ile_Val_Leu
Lys_Thr_Met
Pro
Ser
Trp_Tyr_Phe
Ala(2.A.25.1.3)
Ala/Cys(2.A.25.1.1)
Ala/Gly(2.A.25.1.7)
Ala/Gly/Asn/Gln(2.A.25.1.9)
Ala/Ser/Cys/Thr(2.A.23.3.1)
Arg(2.A.3.1.11)
Arg(2.A.3.2.5)
Asp(2.A.3.1.5)
Asp(2.A.3.11.1)
Asp/Ala(2.A.81.1.6)
Glu(2.A.23.1.14)
Glu(2.A.27.2.1)
Glu/Asp(2.A.3.7.1)
Glu/Asp(2.A.23.1.2)
Glu/Asp(2.A.23.1.5)
Glu/Asp(2.A.23.2.1)
Glu/Asp(2.A.23.2.10)
Glu/Asp(2.A.23.2.11)
Glu/Asp(2.A.23.2.2)
Glu/Asp(2.A.23.2.6)
Glu/Asp(2.A.23.2.8)
Glu/Asp/Cys(2.A.23.2.3)
Gly(2.A.25.1.4)
Leu(2.A.3.13.3)
Leu(2.A.76.1.5)
Leu/Ile/Val(2.A.26.1.8)
Leu/Ile/Val/Phe/Met(2.A.26.1.4)
Lys(2.A.3.2.6)
Lys/His/Arg(2.A.75.1.2)
Ser_and_others(2.A.76.1.9)
Ser_and_others(2.A.95.1.6)
Ser/Ala/Glu_and_others(2.A.23.3.2)
Ser/Thr(2.A.3.1.13)
Ser/Thr(2.A.76.1.10)
Ser/Thr/Trp/Phe/Tyr/His(2.A.3.8.12)
Thr(2.A.76.1.2)
Tyr(2.A.42.1.1)
cationic_amino_acids(2.A.3.6.1)
amino_acids(2.A.3.3.22)
amino_acids(2.A.3.4.7)
amino_acids(2.A.76.1.12)
amino_acids(2.A.76.1.3)
amino_acids(2.A.76.1.6)
amino_acids(2.A.76.1.7)
amino_acids(2.A.76.1.8)
amino_acids(2.A.95.1.7)
(2.A.26.1.6)
(2.A.3.14.3)
(2.A.3.3.17)
(2.A.3.6.4)
(2.A.95.1.3)
Busco_s_copy
AA synthesis
1
0.5
0
transporters
2
1.5
1
0.5

Supplementary Figure 29: “step 4” phylogenomic trees. Phylogenomic trees of *Rickettsiales* and other *Alphaproteobacteria*, for testing the affiliation to *Rickettsiales* of 211 “BUSCO+eggNOG-filtered putative *Rickettsiales* MAGs” (Step 4 in Supplementary Note 4). The phylogeny on the untrimmed concatenated gene alignment, as well as on alignments with the 10%, 20%, 30%, 40%, or 50% most compositionally heterogeneous sites removed are consecutively presented in each panel of this figure. In each tree, the ascertained “core” *Rickettsiales* MAGs are highlighted in green, the ascertained non-*Rickettsiales* MAGs in gray and italics, and the members of each of 13 additional groups (labelled from A to M) in a different colour. On each branch, support values by SH-aLRT with 1000 replicates and by 1000 ultrafast bootstraps are reported. The tree scales stand for estimated sequence divergence.

original concatenated alignment

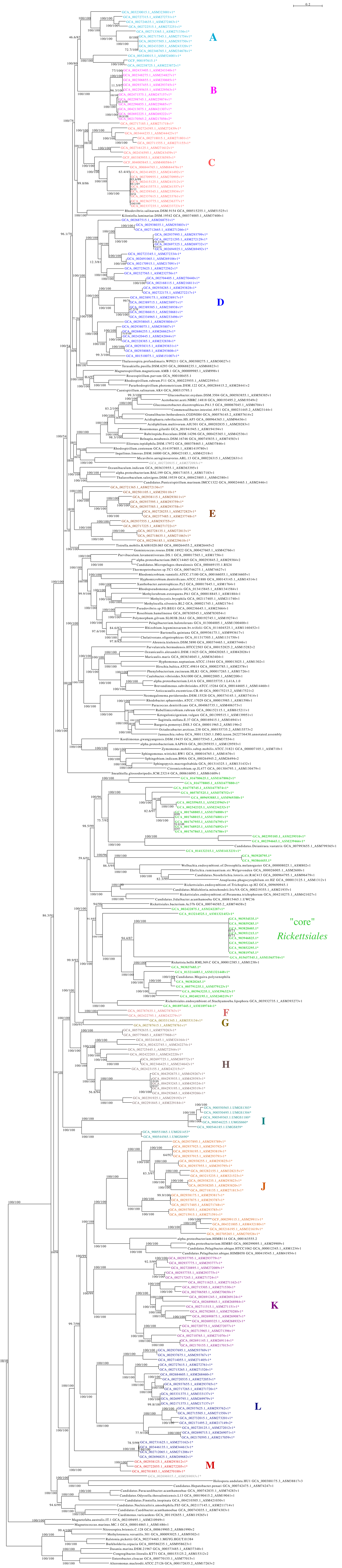

alignment with 10% most heterogeneous sites removed

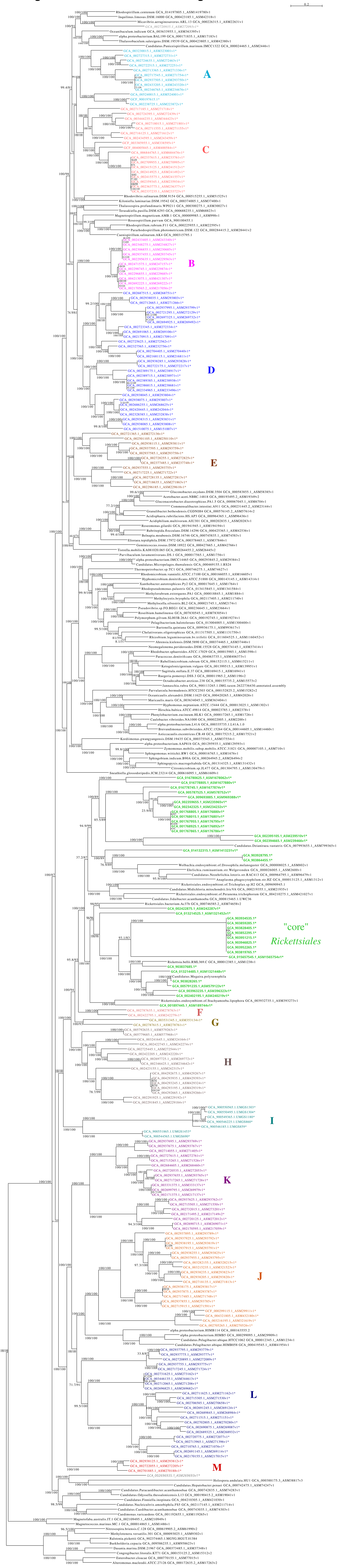

alignment with 20% most heterogeneous sites removed

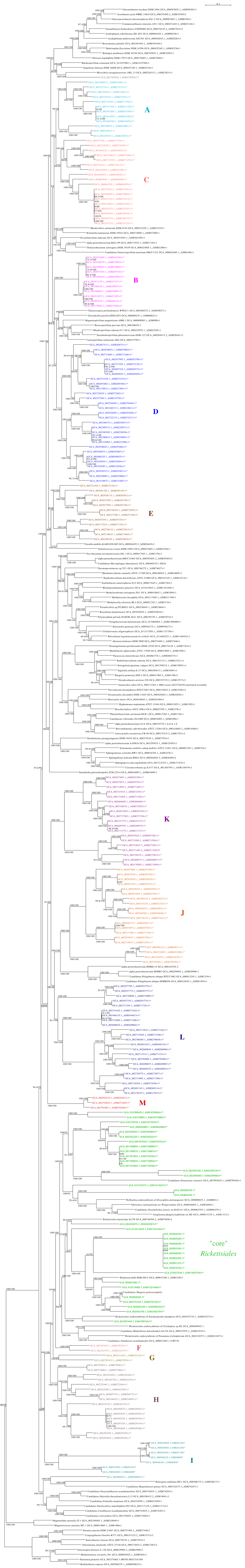

alignment with 30% most heterogeneous sites removed

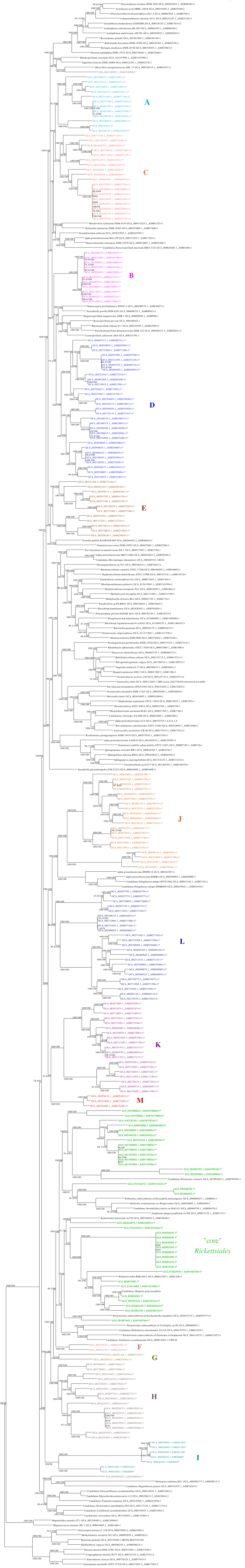

alignment with 40% most heterogeneous sites removed

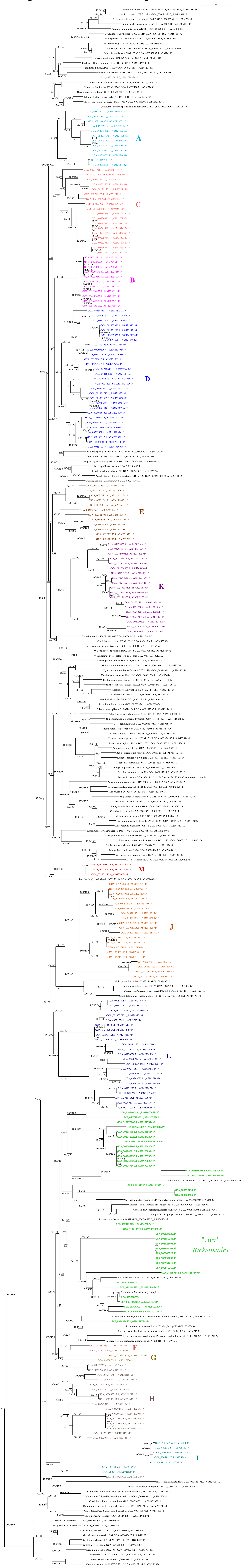

alignment with 50% most heterogeneous sites removed

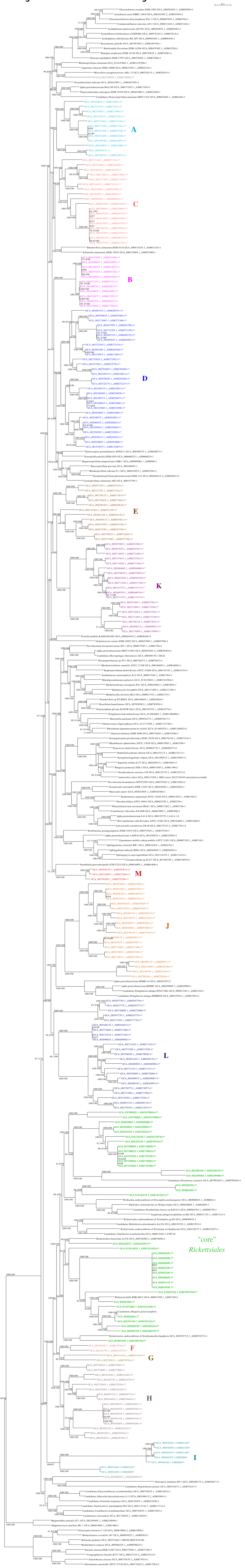

Supplementary Figure 30: identity values. AAI (Average Amino acid Identity) values within each examined clade of MAGs and *Rickettsiales* genomes. Specifically, in the order, “core *Rickettsiales*”, groups A-M, five combinations of “new putative *Rickettsiales* MAGs” with “BUSCO+eggNOG-filtered putative *Rickettsiales* MAGs”, and finally a combination of *Rickettsiales* genomes and closely related MAGs.

	GCA_002422875.1_ASM242287v1	GCA_001897445.1_ASM189744v1	GCA_013214525.1_ASM1321452v1	GCA_014132315.1_ASM1413231v1	GCA_015657545.1_ASM1565754v1	GCA_016778805.1_ASM1677880v1	GCA_016780625.1_ASM1678062v1	GCA_002394665.1_ASM239466v1	GCA_002395105.1_ASM239510v1	GCA_903928795.1_freshwater_MAG_---_Kir	GCA_903864455.1_freshwater_MAG_---_Kir	GCA_002402195.1_ASM240219v1	GCA_003963235.1_ASM396323v1	GCA_013214485.1_ASM1321448v1	GCA_903837685.1_freshwater_MAG_---_Alli	GCA_005791235.1_ASM579123v1	GCA_903828265.1_freshwater_MAG_---_Erk	GCA_009693885.1_ASM969388v1	GCA_005787525.1_ASM578752v1	GCA_016778745.1_ASM1677874v1	GCA_002359655.1_ASM235965v1	GCA_002342325.1_ASM234232v1	GCA_001767865.1_ASM176786v1	GCA_001768015.1_ASM176801v1	GCA_001767955.1_ASM176795v1	GCA_001768925.1_ASM176892v1	GCA_001768805.1_ASM176880v1	GCA_903951215.1_freshwater_MAG_---_C-	GCA_903828405.1_freshwater_MAG_---_C2	GCA_903952265.1_freshwater_MAG_---_C-	GCA_903946825.1_freshwater_MAG_---_C-	GCA_903819765.1_freshwater_MAG_---_C3	GCA_903852295.1_freshwater_MAG_---_C4	GCA_903859285.1_freshwater_MAG_---_G1	GCA_903934535.1_freshwater_MAG_---_G-
GCA_002422875.1_ASM242287v1	100	45	43	43	42	43	44	38	39	39	39	44	45	45	46	46	47	43	43	44	44	44	44	44	44	44	43	42	42	42	42	42	42	42	42
GCA_001897445.1_ASM189744v1	45	100	42	43	41	42	44	38	38	39	39	43	44	43	44	44	44	42	43	43	44	44	44	44	44	44	42	41	42	42	41	41	41	41	41
GCA_013214525.1_ASM1321452v1	43	42	100	41	41	41	42	37	38	38	38	42	43	42	43	43	44	41	41	42	41	42	42	42	42	42	41	41	42	42	41	41	41	41	41
GCA_014132315.1_ASM1413231v1	43	43	41	100	40	42	44	37	37	40	40	42	42	42	42	42	43	42	42	43	43	43	43	43	43	43	42	40	40	41	40	40	40	40	40
GCA_015657545.1_ASM1565754v1	42	41	41	40	100	40	41	36	37	38	38	45	45	45	45	45	46	39	40	40	41	41	40	41	41	40	40	42	42	42	41	42	42	42	42
GCA_016778805.1_ASM1677880v1	43	42	41	42	40	100	50	37	38	39	39	41	42	41	42	42	42	44	45	45	45	45	45	45	45	45	44	39	40	40	39	39	39	39	40
GCA_016780625.1_ASM1678062v1	44	44	42	44	41	50	100	39	39	40	40	42	43	43	43	43	44	44	46	46	46	46	45	46	46	45	44	40	41	41	40	40	40	40	40
GCA_002394665.1_ASM239466v1	38	38	37	37	36	37	39	100	56	37	36	37	37	37	38	37	38	37	38	38	38	38	39	39	39	38	38	37	37	37	37	37	37	37	37
GCA_002395105.1_ASM239510v1	39	38	38	37	37	38	39	56	100	37	37	37	38	38	38	38	38	36	38	38	38	38	38	38	38	38	37	37	37	37	37	37	37	37	37
GCA_903928795.1_freshwater_MAG	39	39	38	40	38	39	40	37	37	100	99	38	39	39	38	39	39	38	39	39	39	39	39	39	39	39	39	38	38	38	38	38	38	38	38
GCA_903864455.1_freshwater_MAG	39	39	38	40	38	39	40	36	37	99	100	38	39	39	38	39	39	38	39	39	39	39	39	40	40	39	39	38	38	38	38	38	38	38	38
GCA_002402195.1_ASM240219v1	44	43	42	42	45	41	42	37	37	38	38	100	60	55	52	58	58	41	41	42	42	42	42	42	42	42	41	44	44	44	43	44	43	43	43
GCA_003963235.1_ASM396323v1	45	44	43	42	45	42	43	37	38	39	39	60	100	56	53	59	58	41	42	42	43	43	43	43	43	43	42	44	44	44	43	44	44	43	43
GCA_013214485.1_ASM1321448v1	45	43	42	42	45	41	43	37	38	39	39	55	56	100	54	57	57	41	42	43	42	43	43	43	43	43	42	44	44	44	44	44	44	44	44
GCA_903837685.1_freshwater_MAG	46	44	43	42	45	42	43	38	38	38	38	52	53	54	100	54	55	42	42	43	43	43	43	43	43	43	41	44	44	44	43	44	44	44	44
GCA_005791235.1_ASM579123v1	46	44	43	42	45	42	43	37	38	39	39	58	59	57	54	100	87	42	42	42	43	43	43	43	43	43	42	44	44	44	43	44	44	44	44
GCA_903828265.1_freshwater_MAG	47	44	44	43	46	42	44	38	38	39	39	58	58	57	55	87	100	42	43	43	43	43	43	43	43	43	42	45	45	45	44	45	45	45	45
GCA_009693885.1_ASM969388v1	43	42	41	42	39	44	44	37	36	38	38	41	41	41	42	42	42	100	58	57	63	63	62	63	63	62	59	38	39	39	38	38	38	38	38
GCA_005787525.1_ASM578752v1	43	43	41	42	40	45	46	38	38	39	39	41	42	42	42	42	43	58	100	57	61	61	62	62	62	62	60	39	39	40	39	39	39	39	39
GCA_016778745.1_ASM1677874v1	44	43	42	43	40	45	46	38	38	39	39	42	42	43	43	42	43	57	57	100	60	60	60	61	61	60	58	40	40	40	39	40	40	40	40
GCA_002359655.1_ASM235965v1	44	44	41	43	41	45	46	38	38	39	39	42	43	42	43	43	43	63	61	60	100	100	67	67	67	67	65	41	41	41	40	41	41	41	41
GCA_002342325.1_ASM234232v1	44	44	42	43	41	45	46	38	38	39	39	42	43	43	43	43	43	63	61	60	100	100	67	67	67	67	65	41	41	41	40	41	40	41	41
GCA_001767865.1_ASM176786v1	44	44	42	43	40	45	45	39	38	39	39	42	43	43	43	43	43	62	62	60	67	67	100	100	100	100	99	40	40	41	40	40	40	40	41
GCA_001768015.1_ASM176801v1	44	44	42	43	41	45	46	39	38	39	40	42	43	43	43	43	43	63	62	61	67	67	100	100	100	100	99	41	40	41	41	41	41	40	41
GCA_001767955.1_ASM176795v1	44	44	42	43	41	45	46	39	38	39	40	42	43	43	43	43	43	63	62	61	67	67	100	100	100	100	99	40	41	41	40	40	40	40	41
GCA_001768925.1_ASM176892v1	44	44	42	43	40	45	45	38	38	39	39	42	43	43	43	43	43	62	62	60	67	67	100	100	100	100	99	40	40	41	40	40	40	40	40
GCA_001768805.1_ASM176880v1	43	42	41	42	40	44	44	38	37	39	39	41	42	42	41	42	42	59	60	58	65	65	99	99	99	99	100	39	40	40	39	39	39	39	40
GCA_903951215.1_freshwater_MAG	42	41	41	40	42	39	40	37	37	38	38	44	44	44	44	44	45	38	39	40	41	41	40	41	40	40	39	100	100	100	100	100	100	100	100
GCA_903828405.1_freshwater_MAG	42	42	42	40	42	40	41	37	37	38	38	44	44	44	44	44	45	39	39	40	41	41	40	40	41	40	40	100	100	100	100	100	100	100	100
GCA_903952265.1_freshwater_MAG	42	42	42	41	42	40	41	37	37	38	38	44	44	44	44	44	45	39	40	40	41	41	41	41	41	41	40	100	100	100	100	100	100	100	100
GCA_903946825.1_freshwater_MAG	42	41	41	40	41	39	40	37	37	38	38	43	43	44	43	43	44	38	39	39	40	40	40	41	40	40	39	100	100	100	100	100	100	100	100
GCA_903819765.1_freshwater_MAG	42	41	41	40	42	39	40	37	37	38	38	44	44	44	44	44	45	38	39	40	41	41	40	41	40	40	39	100	100	100	100	100	100	100	100
GCA_903852295.1_freshwater_MAG	42	41	41	40	42	39	40	37	37	38	38	43	44	44	44	44	45	38	39	40	41	40	40	41	40	40	39	100	100	100	100	100	100	100	100
GCA_903859285.1_freshwater_MAG	42	41	41	40	42	39	40	37	37	38	38	43	43	44	44	44	45	38	39	40	41	41	40	40	40	40	39	100	100	100	100	100	100	100	100
GCA_903934535.1_freshwater_MAG	42	41	41	40	42	40	40	37	37	38	38	43	43	44	44	44	45	38	39	40	41	41	41	41	41	40	40	100	100	100	100	100	100	100	100

GCA_002433205.1_ASM	GCA_002937505.1_ASM	GCA_002717545.1_ASM	GCA_002346765.1_ASM	GCA_002713365.1_ASM	GCA_002724635.1_ASM	GCA_002722515.1_ASM	GCA_002727315.1_ASM	GCA_003230015.1_ASM	GCA_005240015.1_ASM	GCF_900197615.1_Rhod	GCA_002238725.1_ASM	
100	100	100	98	70	61	61	60	51	47	49	49	GCA_002433205.1_ASM243320v1
100	100	100	99	70	61	61	61	51	47	49	50	GCA_002937505.1_ASM293750v1
100	100	100	98	70	60	60	61	50	46	49	49	GCA_002717545.1_ASM271754v1
98	99	98	100	67	58	57	58	47	44	47	47	GCA_002346765.1_ASM234676v1
70	70	70	67	100	61	61	61	51	47	49	49	GCA_002713365.1_ASM271336v1
61	61	60	58	61	100	81	76	54	49	52	52	GCA_002724635.1_ASM272463v1
61	61	60	57	61	81	100	75	53	48	51	51	GCA_002722515.1_ASM272251v1
60	61	61	58	61	76	75	100	53	49	52	52	GCA_002727315.1_ASM272731v1
51	51	50	47	51	54	53	53	100	48	50	50	GCA_003230015.1_ASM323001v1
47	47	46	44	47	49	48	49	48	100	54	54	GCA_005240015.1_ASM524001v1
49	49	49	47	49	52	51	52	50	54	100	90	GCF_900197615.1_Rhodospirillacea
49	50	49	47	49	52	51	52	50	54	90	100	GCA_002238725.1_ASM223872v1

	GCA_002170565.2_AS	GCA_002692225.1_AS	GCA_004213075.1_AS	GCA_002471575.1_AS	GCA_002298745.1_AS	GCA_002296855.1_AS	GCA_002348275.1_AS	GCA_002306855.1_AS	GCA_002937455.1_AS	GCA_002295635.1_AS	GCA_002433405.1_AS
GCA_002170565.2_ASM217056v2	100	100	99	95	95	95	77	78	77	77	76
GCA_002692225.1_ASM269222v1	100	100	99	96	95	95	77	77	77	77	76
GCA_004213075.1_ASM421307v1	99	99	100	96	96	96	79	79	79	79	78
GCA_002471575.1_ASM247157v1	95	96	96	100	100	100	79	79	79	79	78
GCA_002298745.1_ASM229874v1	95	95	96	100	100	100	79	79	79	79	78
GCA_002296855.1_ASM229685v1	95	95	96	100	100	100	79	79	79	79	78
GCA_002348275.1_ASM234827v1	77	77	79	79	79	79	100	100	100	100	100
GCA_002306855.1_ASM230685v1	78	77	79	79	79	79	100	100	100	100	99
GCA_002937455.1_ASM293745v1	77	77	79	79	79	79	100	100	100	100	100
GCA_002295635.1_ASM229563v1	77	77	79	79	79	79	100	100	100	100	99
GCA_002433405.1_ASM243340v1	76	76	78	78	78	78	100	99	100	99	100

	GCA_006844765.1_ASM684476v1	GCF_004005845.1_ASM400584v1	GCF_003385955.1_ASM338595v1	GCA_002716125.1_ASM271612v1	GCA_002434595.1_ASM243459v1	GCA_002717185.1_ASM271718v1	GCA_003444235.1_ASM344423v1	GCA_002724395.1_ASM272439v1	GCA_002718015.1_ASM271801v1	GCA_002711555.1_ASM271155v1	GCA_002359345.1_ASM235934v1	GCA_002414925.1_ASM241492v1	GCA_002337235.1_ASM233723v1	GCA_002363775.1_ASM236377v1	GCA_002337615.1_ASM233761v1	GCA_002415125.1_ASM241512v1	GCA_002709955.1_ASM270995v1	GCA_002415575.1_ASM241557v1
GCA_006844765.1_ASM684476v1	100	56	54	51	48	50	49	50	47	49	58	58	58	58	57	58	57	57
GCF_004005845.1_ASM400584v1	56	100	55	53	51	52	50	51	50	51	56	56	56	56	56	56	56	56
GCF_003385955.1_ASM338595v1	54	55	100	52	50	51	49	51	49	50	55	55	55	55	55	55	55	54
GCA_002716125.1_ASM271612v1	51	53	52	100	75	60	55	55	52	53	52	51	51	51	51	51	51	51
GCA_002434595.1_ASM243459v1	48	51	50	75	100	58	54	53	49	51	50	50	50	49	49	49	50	49
GCA_002717185.1_ASM271718v1	50	52	51	60	58	100	53	54	51	53	51	51	51	51	51	51	51	50
GCA_003444235.1_ASM344423v1	49	50	49	55	54	53	100	72	66	69	49	49	49	49	49	49	49	49
GCA_002724395.1_ASM272439v1	50	51	51	55	53	54	72	100	67	68	51	51	50	50	51	50	51	50
GCA_002718015.1_ASM271801v1	47	50	49	52	49	51	66	67	100	87	49	49	49	49	49	48	49	48
GCA_002711555.1_ASM271155v1	49	51	50	53	51	53	69	68	87	100	50	50	50	50	50	49	50	49
GCA_002359345.1_ASM235934v1	58	56	55	52	50	51	49	51	49	50	100	100	100	100	100	100	100	100
GCA_002414925.1_ASM241492v1	58	56	55	51	50	51	49	51	49	50	100	100	100	100	100	100	100	100
GCA_002337235.1_ASM233723v1	58	56	55	51	50	51	49	50	49	50	100	100	100	100	100	100	100	100
GCA_002363775.1_ASM236377v1	58	56	55	51	49	51	49	50	49	50	100	100	100	100	100	100	100	100
GCA_002337615.1_ASM233761v1	57	56	55	51	49	51	49	51	49	50	100	100	100	100	100	100	100	99
GCA_002415125.1_ASM241512v1	58	56	55	51	49	51	49	50	48	49	100	100	100	100	100	100	100	100
GCA_002709955.1_ASM270995v1	57	56	55	51	50	51	49	51	49	50	100	100	100	100	100	100	100	100
GCA_002415575.1_ASM241557v1	57	56	54	51	49	50	49	50	48	49	100	100	100	100	99	100	100	100

	GCA_002938315.1_ASM293831v1	GCA_002938085.1_ASM293808v1	GCA_001510075.1_ASM151007v1	GCA_002938045.1_ASM293804v1	GCA_002686255.1_ASM268625v1	GCA_002328385.1_ASM232838v1	GCA_002420445.1_ASM242044v1	GCA_002938075.1_ASM293807v1	GCA_002938285.1_ASM293828v1	GCA_002722175.1_ASM272217v1	GCA_002334965.1_ASM233496v1	GCA_002389385.1_ASM238938v1	GCA_002386815.1_ASM238681v1	GCA_002389715.1_ASM238971v1	GCA_002389175.1_ASM238917v1	GCA_002327565.1_ASM232756v1	GCA_002725625.1_ASM272562v1	GCA_002687515.1_ASM268751v1	GCA_002938035.1_ASM293803v1	GCA_002712665.1_ASM271266v1	GCA_002691065.1_ASM269106v1	GCA_002170915.1_ASM217091v1	GCA_002723345.1_ASM272334v1	GCA_002168115.1_ASM216811v1	GCA_002704405.1_ASM270440v1	GCA_002697325.1_ASM269732v1	GCA_002694925.1_ASM269492v1	GCA_002937995.1_ASM293799v1	GCA_002721295.1_ASM272129v1
GCA_002938315.1_ASM293831v1	100	99	70	62	71	72	71	71	54	54	57	57	57	57	56	56	55	55	54	52	52	52	54	51	51	49	49	51	50
GCA_002938085.1_ASM293808v1	99	100	68	60	70	71	71	70	53	54	57	57	57	56	56	56	54	55	53	52	51	50	54	51	51	48	49	50	49
GCA_001510075.1_ASM151007v1	70	68	100	60	71	71	71	70	54	54	57	57	57	57	56	55	53	54	52	50	49	49	52	49	49	47	47	49	48
GCA_002938045.1_ASM293804v1	62	60	60	100	63	63	63	62	53	55	56	56	56	56	56	54	53	53	52	49	49	49	52	49	49	45	47	49	47
GCA_002686255.1_ASM268625v1	71	70	71	63	100	99	99	85	54	54	57	57	57	57	56	56	54	55	54	53	51	50	54	50	50	48	49	51	50
GCA_002328385.1_ASM232838v1	72	71	71	63	99	100	99	85	54	54	58	57	58	57	57	56	54	56	54	52	51	51	54	51	51	48	49	51	50
GCA_002420445.1_ASM242044v1	71	71	71	63	99	99	100	85	54	54	57	57	57	57	57	56	54	56	54	52	51	51	54	50	51	48	49	51	50
GCA_002938075.1_ASM293807v1	71	70	70	62	85	85	85	100	54	55	58	57	57	57	57	55	54	54	53	51	51	50	53	50	50	47	48	50	49
GCA_002938285.1_ASM293828v1	54	53	54	53	54	54	54	54	100	99	53	53	53	52	52	49	48	48	49	47	45	45	48	48	47	45	46	47	47
GCA_002722175.1_ASM272217v1	54	54	54	55	54	54	54	55	99	100	53	53	54	52	52	49	48	49	49	48	47	46	49	48	48	46	46	48	47
GCA_002334965.1_ASM233496v1	57	57	57	56	57	58	57	58	53	53	100	99	99	99	69	51	50	50	51	49	49	48	51	48	48	46	47	48	48
GCA_002389385.1_ASM238938v1	57	57	57	56	57	57	57	57	53	53	99	100	99	99	69	51	50	50	51	48	49	48	51	48	48	45	47	48	47
GCA_002386815.1_ASM238681v1	57	57	57	56	57	58	57	57	53	54	99	99	100	99	69	51	50	50	51	49	49	48	51	48	48	46	47	48	47
GCA_002389715.1_ASM238971v1	57	56	57	56	57	57	57	57	52	52	99	99	99	100	68	51	49	50	50	49	49	48	51	48	48	45	46	48	47
GCA_002389175.1_ASM238917v1	56	56	56	56	56	57	57	57	52	52	69	69	69	68	100	51	49	50	50	48	48	48	50	48	48	46	47	47	47
GCA_002327565.1_ASM232756v1	56	56	55	54	56	56	56	55	49	49	51	51	51	51	51	100	67	64	61	60	60	60	62	63	62	56	56	57	56
GCA_002725625.1_ASM272562v1	55	54	53	53	54	54	54	54	48	48	50	50	50	49	49	67	100	62	59	57	58	57	59	57	57	53	54	55	54
GCA_002687515.1_ASM268751v1	55	55	54	53	55	56	56	54	48	49	50	50	50	50	50	64	62	100	59	57	58	57	59	55	54	54	54	54	54
GCA_002938035.1_ASM293803v1	54	53	52	52	54	54	54	53	49	49	51	51	51	50	50	61	59	59	100	93	62	62	65	57	57	60	60	61	61
GCA_002712665.1_ASM271266v1	52	52	50	49	53	52	52	51	47	48	49	48	49	49	48	60	57	57	93	100	61	61	63	55	55	58	59	60	59
GCA_002691065.1_ASM269106v1	52	51	49	49	51	51	51	51	45	47	49	49	49	49	48	60	58	58	62	61	100	100	84	56	56	57	57	57	56
GCA_002170915.1_ASM217091v1	52	50	49	49	50	51	51	50	45	46	48	48	48	48	48	60	57	57	62	61	100	100	83	56	56	57	57	57	56
GCA_002723345.1_ASM272334v1	54	54	52	52	54	54	54	53	48	49	51	51	51	51	50	62	59	59	65	63	84	83	100	57	57	59	59	59	59
GCA_002168115.1_ASM216811v1	51	51	49	49	50	51	50	50	48	48	48	48	48	48	48	63	57	55	57	55	56	56	57	100	100	55	56	56	55
GCA_002704405.1_ASM270440v1	51	51	49	49	50	51	51	50	47	48	48	48	48	48	48	62	57	54	57	55	56	56	57	100	100	55	55	56	55
GCA_002697325.1_ASM269732v1	49	48	47	45	48	48	48	47	45	46	46	45	46	45	46	56	53	54	60	58	57	57	59	55	55	100	95	94	92
GCA_002694925.1_ASM269492v1	49	49	47	47	49	49	49	48	46	46	47	47	47	46	47	56	54	54	60	59	57	57	59	56	55	95	100	95	94
GCA_002937995.1_ASM293799v1	51	50	49	49	51	51	51	50	47	48	48	48	48	48	47	57	55	54	61	60	57	57	59	56	56	94	95	100	96
GCA_002721295.1_ASM272129v1	50	49	48	47	50	50	50	49	47	47	48	47	47	47	47	56	54	54	61	59	56	56	59	55	55	92	94	96	100

	GCA_002728135.1_ASM	GCA_002718635.1_ASM	GCA_002296185.1_ASM	GCA_002717225.1_ASM	GCA_002937555.1_ASM	GCA_002937585.1_ASM	GCA_002937595.1_ASM	GCA_002938115.1_ASM	GCA_002377485.1_ASM	GCA_002728255.1_ASM	GCA_002501105.1_ASM	GCA_002721365.1_ASM
GCA_002728135.1_ASM272813v1	100	99	66	56	55	49	49	49	47	48	48	49
GCA_002718635.1_ASM271863v1	99	100	64	54	54	47	47	47	46	46	47	48
GCA_002296185.1_ASM229618v1	66	64	100	56	55	49	49	48	47	48	49	49
GCA_002717225.1_ASM271722v1	56	54	56	100	74	51	51	50	48	49	51	51
GCA_002937555.1_ASM293755v1	55	54	55	74	100	52	52	51	48	50	52	53
GCA_002937585.1_ASM293758v1	49	47	49	51	52	100	100	78	62	63	58	55
GCA_002937595.1_ASM293759v1	49	47	49	51	52	100	100	78	62	63	58	56
GCA_002938115.1_ASM293811v1	49	47	48	50	51	78	78	100	61	63	57	55
GCA_002377485.1_ASM237748v1	47	46	47	48	48	62	62	61	100	99	53	51
GCA_002728255.1_ASM272825v1	48	46	48	49	50	63	63	63	99	100	55	52
GCA_002501105.1_ASM250110v1	48	47	49	51	52	58	58	57	53	55	100	56
GCA_002721365.1_ASM272136v1	49	48	49	51	53	55	56	55	51	52	56	100

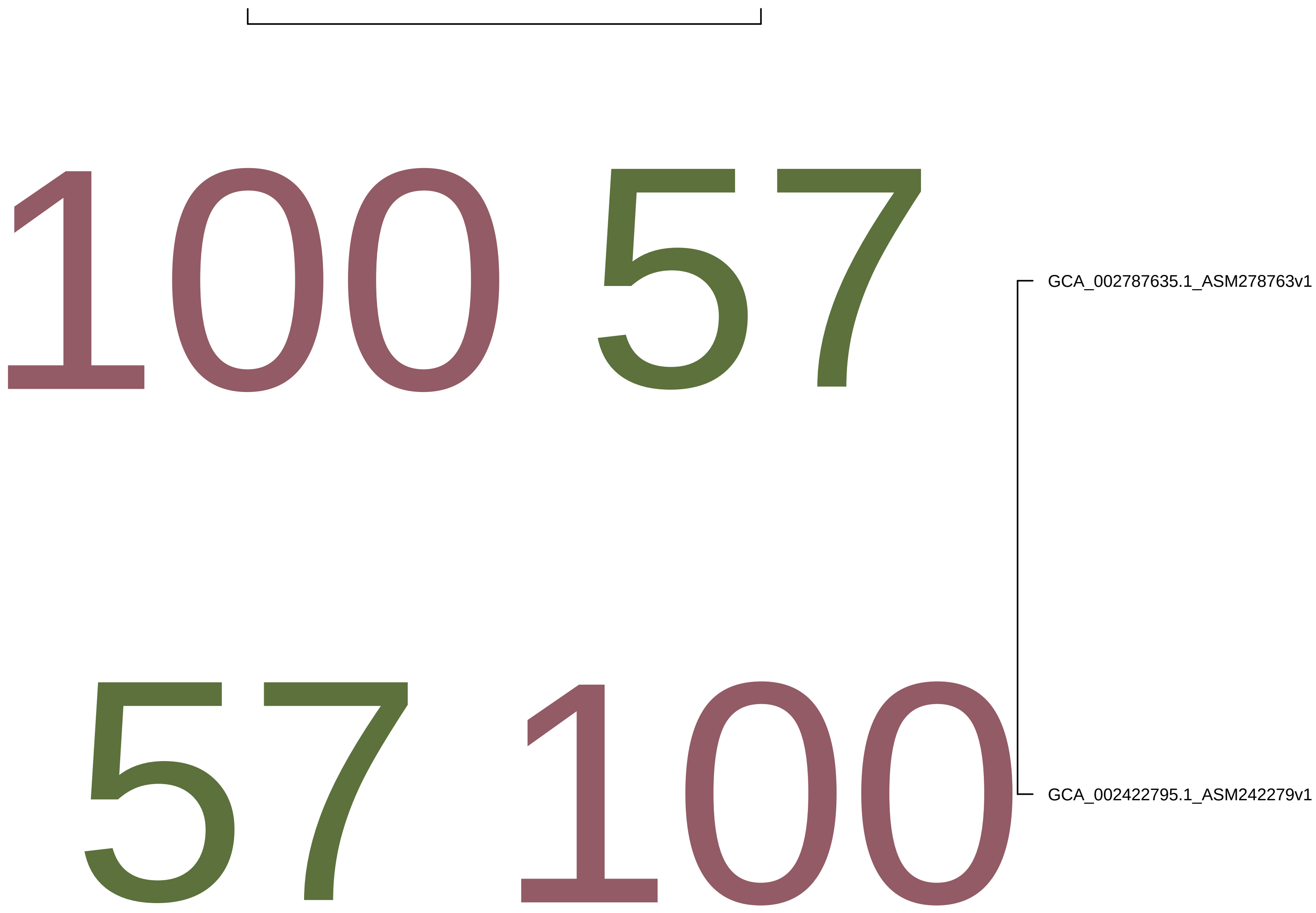
100
57
57
100
GCA_002787635.1_ASM278763v1
GCA_002422795.1_ASM242279v1

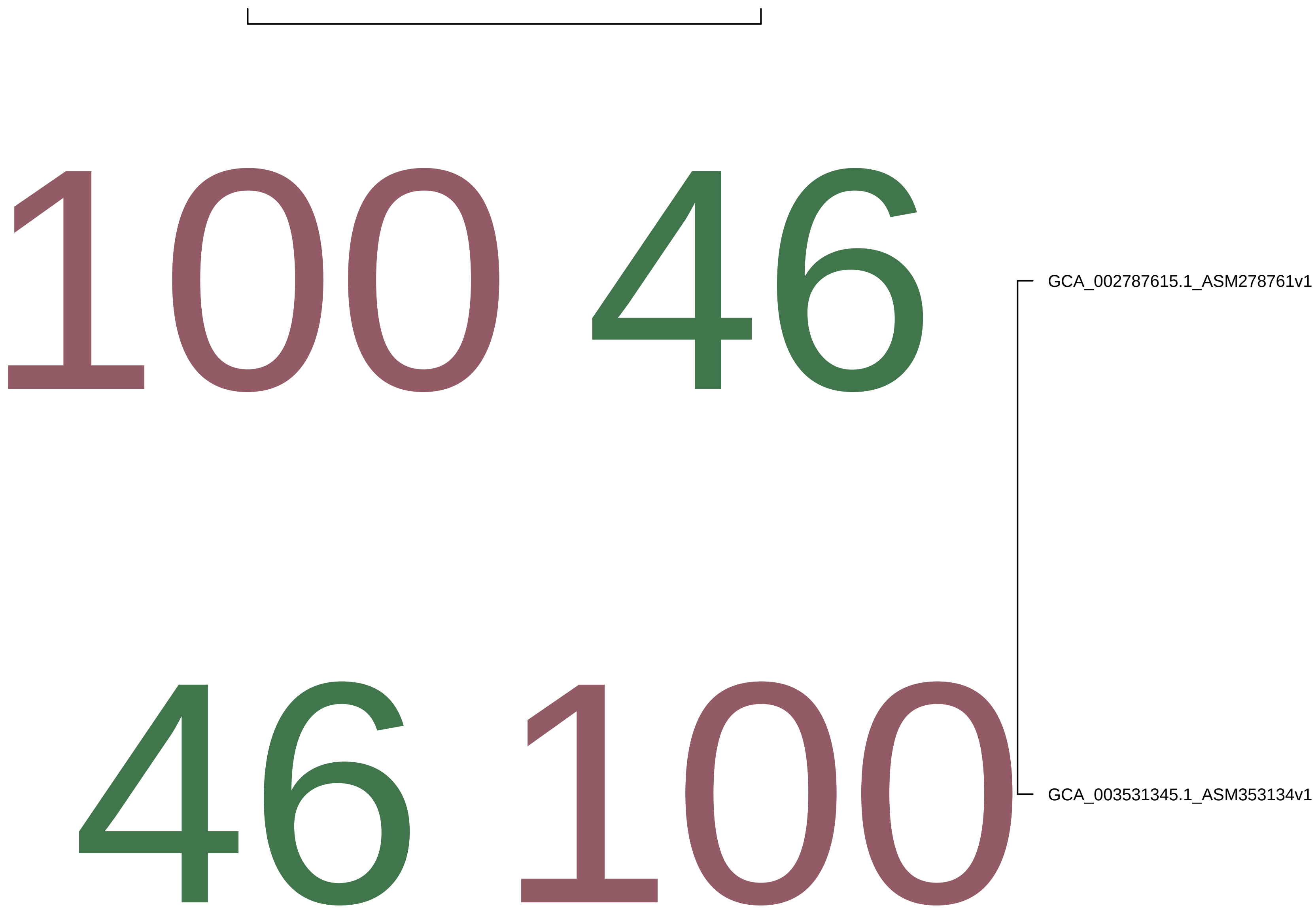
100 100 46
46 100 100
GCA_002787615.1_ASM278761v1
GCA_003531345.1_ASM353134v1

GCA_002422205.1_ASM242220v	GCA_002725445.1_ASM272544v	GCA_002423155.1_ASM242315v	GCA_003241645.1_ASM324164v	GCA_002422745.1_ASM242274v	GCA_002291845.1_ASM229184v	GCA_002291925.1_ASM229192v	GCA_002697725.1_ASM269772v	GCA_002346425.1_ASM234642v	GCA_005792635.1_ASM579263v	GCA_005779685.1_ASM577968v	GCA_004293935.1_ASM429393v	GCA_004292665.1_ASM429266v	GCA_004293195.1_ASM429319v	GCA_004293245.1_ASM429324v	GCA_004292675.1_ASM429267v	
100	58	53	55	54	53	53	53	57	53	51	50	50	50	51	50	GCA_002422205.1_ASM242220v1
58	100	51	54	53	51	52	52	55	50	49	49	50	49	49	49	GCA_002725445.1_ASM272544v1
53	51	100	51	50	54	54	47	50	53	52	51	51	51	50	50	GCA_002423155.1_ASM242315v1
55	54	51	100	62	52	52	50	52	51	50	49	49	49	50	49	GCA_003241645.1_ASM324164v1
54	53	50	62	100	51	51	48	51	49	49	47	47	47	47	47	GCA_002422745.1_ASM242274v1
53	51	54	52	51	100	74	48	51	51	50	51	52	51	51	51	GCA_002291845.1_ASM229184v1
53	52	54	52	51	74	100	48	51	51	50	51	52	51	51	52	GCA_002291925.1_ASM229192v1
53	52	47	50	48	48	48	100	94	46	45	46	45	45	45	46	GCA_002697725.1_ASM269772v1
57	55	50	52	51	51	51	94	100	50	49	49	49	49	49	49	GCA_002346425.1_ASM234642v1
53	50	53	51	49	51	51	46	50	100	100	49	48	48	49	48	GCA_005792635.1_ASM579263v1
51	49	52	50	49	50	50	45	49	100	100	46	46	46	46	46	GCA_005779685.1_ASM577968v1
50	49	51	49	47	51	51	46	49	49	46	100	100	99	99	94	GCA_004293935.1_ASM429393v1
50	50	51	49	47	52	52	45	49	48	46	100	100	99	98	93	GCA_004292665.1_ASM429266v1
50	49	51	49	47	51	51	45	49	48	46	99	99	100	98	93	GCA_004293195.1_ASM429319v1
51	49	50	50	47	51	51	45	49	49	46	99	98	98	100	93	GCA_004293245.1_ASM429324v1
50	49	50	49	47	51	52	46	49	48	46	94	93	93	93	100	GCA_004292675.1_ASM429267v1

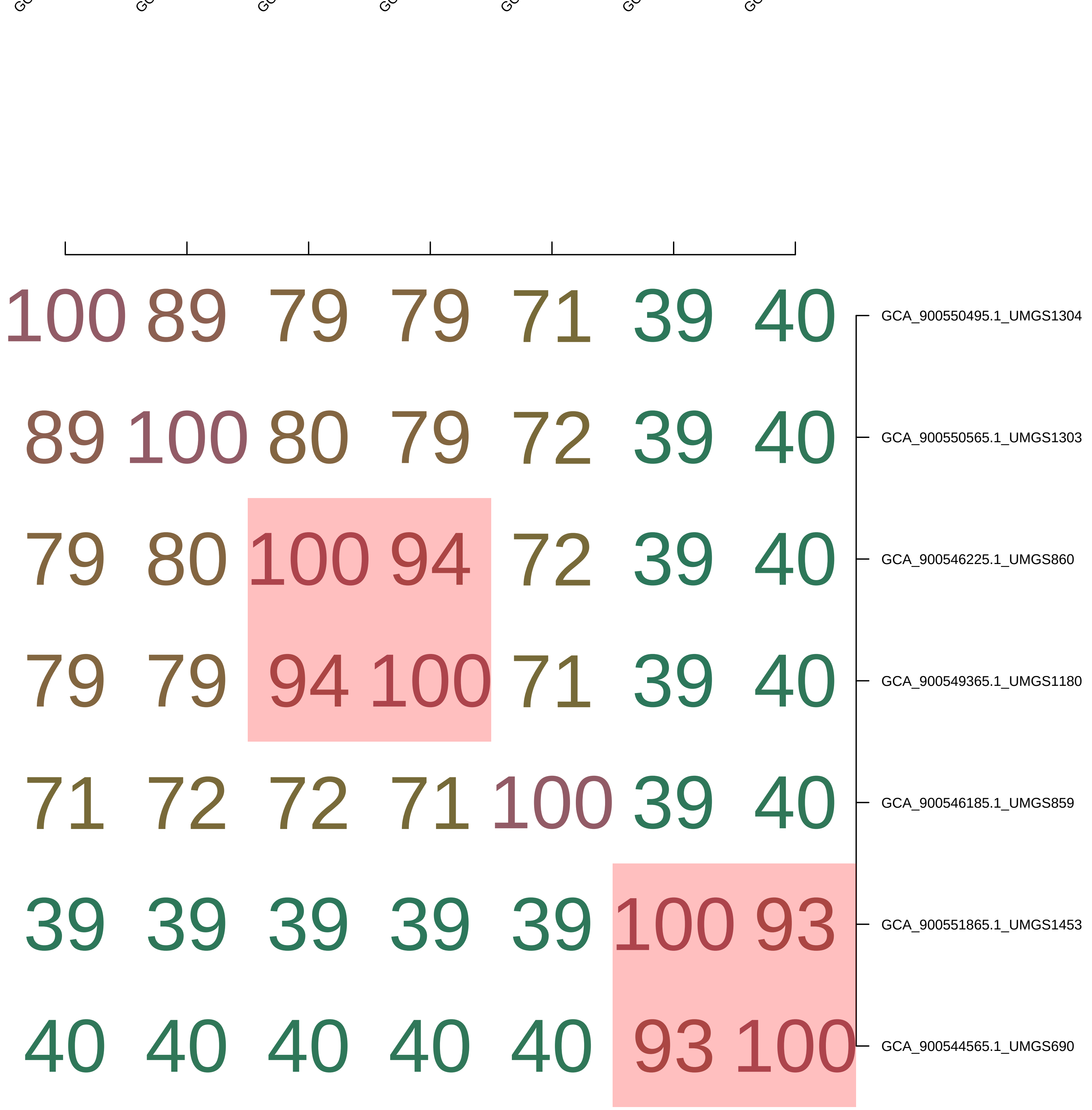
100 89 79 79 71 39 40
GCA_900550495.1_UMGS1304
89 100 80 79 72 39 40
GCA_900550565.1_UMGS1303
79 80 100 94 72 39 40
GCA_900546225.1_UMGS860
79 79 94 100 71 39 40
GCA_900549365.1_UMGS1180
71 72 72 71 100 39 40
GCA_900546185.1_UMGS859
39 39 39 39 39 100 93
GCA_900551865.1_UMGS1453
40 40 40 40 40 93 100
GCA_900544565.1_UMGS690

	GCA_002938205.1_ASM293820v1	GCA_002938235.1_ASM293823v1	GCA_002718135.1_ASM271813v1	GCA_002937895.1_ASM293789v1	GCA_003282155.1_ASM328215v1	GCA_003215235.1_ASM321523v1	GCA_002938255.1_ASM293825v1	GCA_002937955.1_ASM293795v1	GCA_002937925.1_ASM293792v1	GCA_002938195.1_ASM293819v1	GCA_002937915.1_ASM293791v1	GCA_002717485.1_ASM271748v1	GCA_002938175.1_ASM293817v1	GCA_002937875.1_ASM293787v1	GCA_002937855.1_ASM293785v1	GCA_002715915.1_ASM271591v1	GCA_004321805.1_ASM432180v1	GCF_000299115.1_ASM29911v1	GCA_003216195.1_ASM321619v1	GCA_002705265.1_ASM270526v1
GCA_002938205.1_ASM293820v1	100	100	66	62	66	65	64	64	64	64	63	57	57	57	58	58	46	46	46	46
GCA_002938235.1_ASM293823v1	100	100	66	62	65	65	64	64	64	64	63	57	56	56	58	58	45	47	46	46
GCA_002718135.1_ASM271813v1	66	66	100	57	60	60	57	58	58	59	59	52	51	51	54	53	43	44	44	44
GCA_002937895.1_ASM293789v1	62	62	57	100	59	59	61	61	63	63	63	59	58	58	60	60	46	46	46	46
GCA_003282155.1_ASM328215v1	66	65	60	59	100	81	61	61	60	61	60	55	55	54	56	55	46	47	47	46
GCA_003215235.1_ASM321523v1	65	65	60	59	81	100	60	60	60	61	59	55	54	53	55	54	45	46	47	46
GCA_002938255.1_ASM293825v1	64	64	57	61	61	60	100	98	65	65	63	57	55	55	57	56	45	46	45	46
GCA_002937955.1_ASM293795v1	64	64	58	61	61	60	98	100	64	65	64	56	55	55	57	55	45	45	45	45
GCA_002937925.1_ASM293792v1	64	64	58	63	60	60	65	64	100	99	98	57	56	56	58	57	44	45	45	45
GCA_002938195.1_ASM293819v1	64	64	59	63	61	61	65	65	99	100	99	57	57	57	58	57	46	46	45	45
GCA_002937915.1_ASM293791v1	63	63	59	63	60	59	63	64	98	99	100	57	55	55	58	57	44	45	45	45
GCA_002717485.1_ASM271748v1	57	57	52	59	55	55	57	56	57	57	57	100	99	99	66	65	45	46	45	45
GCA_002938175.1_ASM293817v1	57	56	51	58	55	54	55	55	56	57	55	99	100	98	66	65	44	45	44	44
GCA_002937875.1_ASM293787v1	57	56	51	58	54	53	55	55	56	57	55	99	98	100	65	65	44	45	44	44
GCA_002937855.1_ASM293785v1	58	58	54	60	56	55	57	57	58	58	58	66	66	65	100	99	46	47	46	47
GCA_002715915.1_ASM271591v1	58	58	53	60	55	54	56	55	57	57	57	65	65	65	99	100	45	46	46	45
GCA_004321805.1_ASM432180v1	46	45	43	46	46	45	45	45	44	46	44	45	44	44	46	45	100	77	73	62
GCF_000299115.1_ASM29911v1	46	47	44	46	47	46	46	45	45	46	45	46	45	45	47	46	77	100	73	63
GCA_003216195.1_ASM321619v1	46	46	44	46	47	47	45	45	45	45	45	45	44	44	46	46	73	73	100	62
GCA_002705265.1_ASM270526v1	46	46	44	46	46	46	46	45	45	45	45	45	44	44	47	45	62	63	62	100

	GCA_002717265.1_ASM271726v1	GCA_002937655.1_ASM293765v1	GCA_002720535.1_ASM272053v1	GCA_002171375.1_ASM217137v1	GCA_002699795.1_ASM269979v1	GCA_003331375.1_ASM333137v1	GCA_002684605.1_ASM268460v1	GCA_002715265.1_ASM271526v1	GCA_002727615.1_ASM272761v1	GCA_002937695.1_ASM293769v1	GCA_002937675.1_ASM293767v1	GCA_002714055.1_ASM271405v1	GCA_002690715.1_ASM269071v1	GCA_002170595.1_ASM217059v1	GCA_002720125.1_ASM272012v1	GCA_002171495.2_ASM217149v2	GCA_002732015.1_ASM273201v1	GCA_002715505.1_ASM271550v1	GCA_002937625.1_ASM293762v1
GCA_002717265.1_ASM271726v1	100	99	81	78	80	79	71	66	66	61	61	57	47	48	47	47	48	48	48
GCA_002937655.1_ASM293765v1	99	100	81	80	81	81	71	67	66	61	62	60	48	48	47	48	49	48	49
GCA_002720535.1_ASM272053v1	81	81	100	81	82	81	71	67	67	61	61	59	48	48	47	48	48	48	48
GCA_002171375.1_ASM217137v1	78	80	81	100	100	97	72	67	67	61	62	59	48	48	47	47	48	48	48
GCA_002699795.1_ASM269979v1	80	81	82	100	100	99	72	67	67	62	62	61	49	49	47	48	49	49	49
GCA_003331375.1_ASM333137v1	79	81	81	97	99	100	72	67	67	61	62	58	48	48	47	47	48	48	48
GCA_002684605.1_ASM268460v1	71	71	71	72	72	72	100	65	65	61	61	58	47	47	46	47	47	48	48
GCA_002715265.1_ASM271526v1	66	67	67	67	67	67	65	100	97	61	62	59	48	48	47	48	49	49	49
GCA_002727615.1_ASM272761v1	66	66	67	67	67	67	65	97	100	61	61	58	47	48	47	47	48	48	48
GCA_002937695.1_ASM293769v1	61	61	61	61	62	61	61	61	61	100	99	74	47	48	47	48	48	48	48
GCA_002937675.1_ASM293767v1	61	62	61	62	62	62	61	62	61	99	100	74	48	48	47	48	48	48	49
GCA_002714055.1_ASM271405v1	57	60	59	59	61	58	58	59	58	74	74	100	45	47	44	46	46	47	47
GCA_002690715.1_ASM269071v1	47	48	48	48	49	48	47	48	47	47	48	45	100	99	59	59	60	60	60
GCA_002170595.1_ASM217059v1	48	48	48	48	49	48	47	48	48	48	48	47	99	100	59	60	60	60	60
GCA_002720125.1_ASM272012v1	47	47	47	47	47	47	46	47	47	47	47	44	59	59	100	59	60	60	60
GCA_002171495.2_ASM217149v2	47	48	48	47	48	47	47	48	47	48	48	46	59	60	59	100	82	73	73
GCA_002732015.1_ASM273201v1	48	49	48	48	49	48	47	49	48	48	48	46	60	60	60	82	100	74	74
GCA_002715505.1_ASM271550v1	48	48	48	48	49	48	48	49	48	48	48	47	60	60	60	73	74	100	100
GCA_002937625.1_ASM293762v1	48	49	48	48	49	48	48	49	48	48	49	47	60	60	60	73	74	100	100

	GCA_002937755.1_ASM293775v1	GCA_002717245.1_ASM271724v1	GCA_002720895.1_ASM272089v1	GCA_002937775.1_ASM293777v1	GCA_002937795.1_ASM293779v1	GCA_003446135.1_ASM344613v1	GCA_002712065.1_ASM271206v1	GCA_002731625.1_ASM273162v1	GCA_002696825.1_ASM269682v1	GCA_002691145.1_ASM269114v1	GCA_002170155.1_ASM217015v1	GCA_002710765.1_ASM271076v1	GCA_002713965.1_ASM271396v1	GCA_002720775.1_ASM272077v1	GCA_002702805.1_ASM270280v1	GCA_002711515.1_ASM271151v1	GCA_002691245.1_ASM269124v1	GCA_002689845.1_ASM268984v1	GCA_002711625.1_ASM271162v1	GCA_002690875.1_ASM269087v1	GCA_002689325.1_ASM268932v1	GCA_002715305.1_ASM271530v1	GCA_002706585.1_ASM270658v1
GCA_002937755.1_ASM293775v1	100	99	75	77	77	47	48	47	48	47	45	44	45	44	45	45	46	45	45	45	46	43	43
GCA_002717245.1_ASM271724v1	99	100	76	77	77	47	47	47	48	46	45	45	46	44	45	45	46	45	45	45	46	43	43
GCA_002720895.1_ASM272089v1	75	76	100	74	71	46	47	46	47	46	44	45	45	43	44	43	45	45	44	44	45	43	42
GCA_002937775.1_ASM293777v1	77	77	74	100	97	47	47	46	48	46	46	45	45	43	44	45	46	46	44	45	46	42	44
GCA_002937795.1_ASM293779v1	77	77	71	97	100	46	47	46	47	46	44	44	45	44	44	44	46	45	44	44	45	42	43
GCA_003446135.1_ASM344613v1	47	47	46	47	46	100	97	95	94	46	44	44	45	43	44	44	45	45	44	44	45	42	42
GCA_002712065.1_ASM271206v1	48	47	47	47	47	97	100	97	95	46	45	45	46	45	45	45	46	46	45	45	46	43	43
GCA_002731625.1_ASM273162v1	47	47	46	46	46	95	97	100	95	46	45	45	45	44	44	44	45	45	44	45	46	42	43
GCA_002696825.1_ASM269682v1	48	48	47	48	47	94	95	95	100	46	45	45	46	44	45	45	45	46	45	45	46	43	43
GCA_002691145.1_ASM269114v1	47	46	46	46	46	46	46	46	46	100	100	59	59	55	56	57	56	56	57	55	57	55	53
GCA_002170155.1_ASM217015v1	45	45	44	46	44	44	45	45	45	100	100	57	57	53	54	55	55	55	55	55	56	53	53
GCA_002710765.1_ASM271076v1	44	45	45	45	44	44	45	45	45	59	57	100	57	54	54	54	55	55	54	54	56	53	51
GCA_002713965.1_ASM271396v1	45	46	45	45	45	45	46	45	46	59	57	57	100	85	55	55	56	55	56	54	56	54	52
GCA_002720775.1_ASM272077v1	44	44	43	43	44	43	45	44	44	55	53	54	85	100	52	53	53	53	53	51	54	49	49
GCA_002702805.1_ASM270280v1	45	45	44	44	44	44	45	44	45	56	54	54	55	52	100	63	60	60	59	64	65	58	55
GCA_002711515.1_ASM271151v1	45	45	43	45	44	44	45	44	45	57	55	54	55	53	63	100	62	60	60	62	65	57	58
GCA_002691245.1_ASM269124v1	46	46	45	46	46	45	46	45	45	56	55	55	56	53	60	62	100	63	60	60	62	60	57
GCA_002689845.1_ASM268984v1	45	45	45	46	45	45	46	45	46	56	55	55	55	53	60	60	63	100	59	60	61	58	57
GCA_002711625.1_ASM271162v1	45	45	44	44	44	44	45	44	45	57	55	54	56	53	59	60	60	59	100	58	60	64	59
GCA_002690875.1_ASM269087v1	45	45	44	45	44	44	45	45	45	55	55	54	54	51	64	62	60	60	58	100	86	58	56
GCA_002689325.1_ASM268932v1	46	46	45	46	45	45	46	46	46	57	56	56	56	54	65	65	62	61	60	86	100	59	59
GCA_002715305.1_ASM271530v1	43	43	43	42	42	42	43	42	43	55	53	53	54	49	58	57	60	58	64	58	59	100	71
GCA_002706585.1_ASM270658v1	43	43	42	44	43	42	43	43	43	53	53	51	52	49	55	58	57	57	59	56	59	71	100

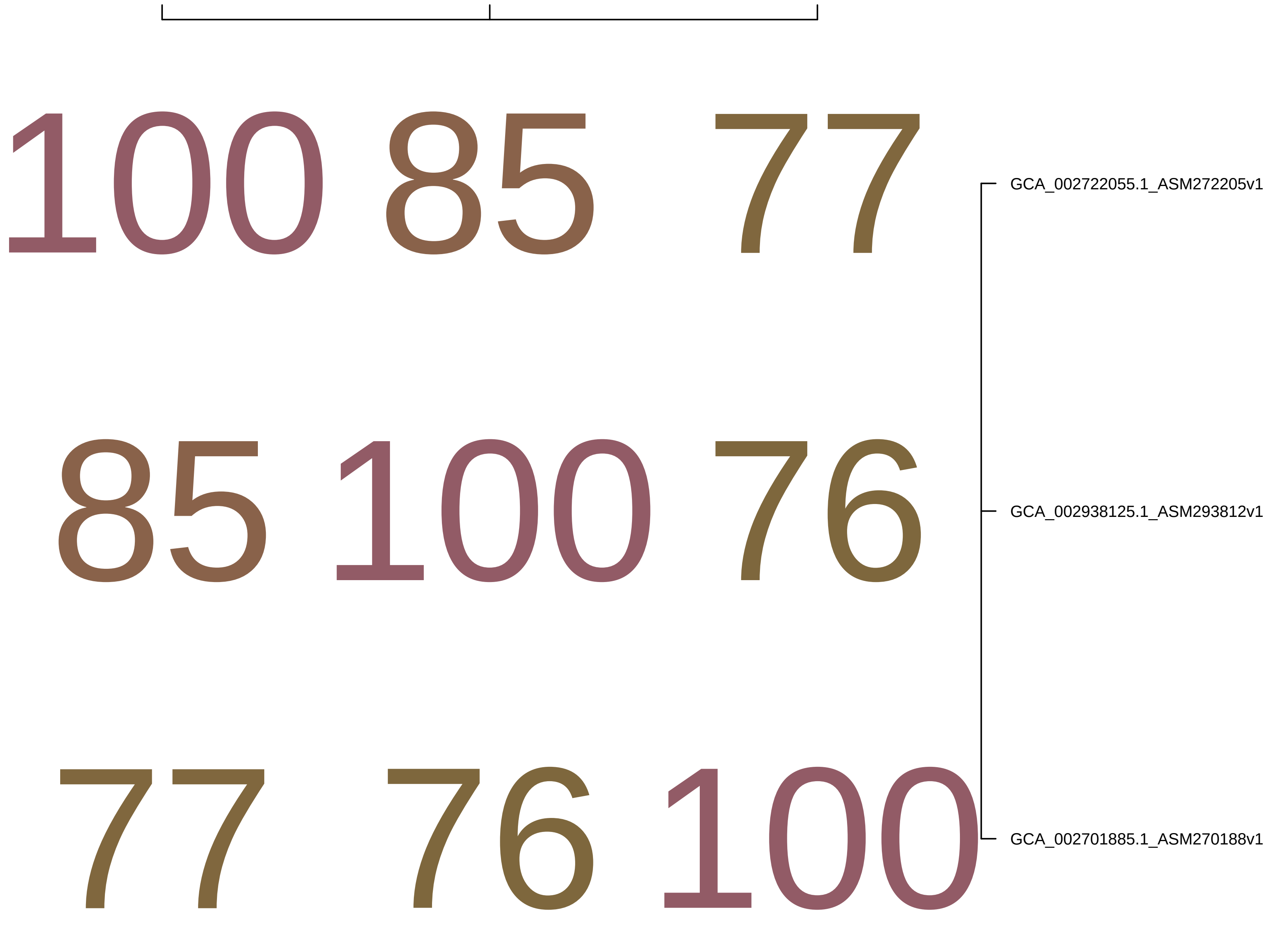
100 85 77
85 100 76
77 76 100
GCA_002722055.1_ASM272205v1
GCA_002938125.1_ASM293812v1
GCA_002701885.1_ASM270188v1

	GCA_905478245.1_0.2_20180403_Bin_MAX	GCA_905478165.1_0.2_20180403_Bin_25_s	GCA_016780625.1_ASM1678062v1	GCA_905480015.1_10_20180529_Bin_6_1	GCA_018659605.1_ASM1865960v1	GCA_018659145.1_ASM1865914v1	GCA_016778805.1_ASM1677880v1	GCA_017620845.1_ASM1762084v1	GCA_017475895.1_ASM1747589v1	GCA_017444095.1_ASM1744409v1	GCA_017444125.1_ASM1744412v1	GCA_002395105.1_ASM239510v1	GCA_002394665.1_ASM239466v1	GCA_014190355.1_ASM1419035v1	GCA_903837365.1_freshwater_MAG_---_L	GCA_009921385.1_ASM992138v1	GCA_903959715.1_freshwater_MAG_---_L	GCA_005787525.1_ASM578752v1	GCA_009925685.1_ASM992568v1	GCA_009924125.1_ASM992412v1	GCA_002359655.1_ASM235965v1	GCA_018969185.1_ASM1896918v1	GCA_009693885.1_ASM969388v1	GCA_903917585.1_freshwater_MAG_---_L	GCA_001768015.1_ASM176801v1	GCA_903927415.1_freshwater_MAG_---_L	GCA_903878685.1_freshwater_MAG_---_L	GCA_017999675.1_ASM1799967v1	GCA_903831915.1_freshwater_MAG_---_K	GCA_903872175.1_freshwater_MAG_---_L	GCA_016778745.1_ASM1677874v1
GCA_905478245.1_0.2_20180403_B	100	54	47	42	46	46	46	43	43	38	38	39	38	53	53	51	53	53	53	54	55	55	53	54	55	55	55	54	54	54	54
GCA_905478165.1_0.2_20180403_B	54	100	46	42	45	46	46	42	42	38	38	38	38	50	50	49	50	50	51	51	51	51	50	51	51	52	52	51	51	51	51
GCA_016780625.1_ASM1678062v1	47	46	100	42	49	50	50	41	40	38	38	39	39	46	45	45	46	46	46	46	46	46	44	46	46	46	46	45	45	45	46
GCA_905480015.1_10_20180529_B	42	42	42	100	41	41	41	37	38	37	37	37	38	41	41	41	41	41	41	41	41	41	40	42	41	42	41	41	41	41	41
GCA_018659605.1_ASM1865960v1	46	45	49	41	100	76	67	39	39	37	38	38	38	44	44	44	44	44	44	44	44	45	43	44	45	44	44	44	44	44	44
GCA_018659145.1_ASM1865914v1	46	46	50	41	76	100	66	39	40	37	38	38	38	44	44	44	44	44	44	44	44	45	44	45	45	45	45	45	44	44	45
GCA_016778805.1_ASM1677880v1	46	46	50	41	67	66	100	40	40	37	38	38	37	45	44	44	45	45	45	45	45	45	44	45	45	45	45	45	44	44	45
GCA_017620845.1_ASM1762084v1	43	42	41	37	39	39	40	100	69	38	38	38	38	42	42	42	42	42	42	42	42	43	41	42	42	42	43	42	42	42	42
GCA_017475895.1_ASM1747589v1	43	42	40	38	39	40	40	69	100	39	38	38	38	42	42	41	42	42	42	42	42	43	41	42	43	42	42	42	42	42	42
GCA_017444095.1_ASM1744409v1	38	38	38	37	37	37	37	38	39	100	55	54	54	38	38	38	38	37	38	37	37	38	37	38	38	38	38	38	37	38	38
GCA_017444125.1_ASM1744412v1	38	38	38	37	38	38	38	38	38	55	100	53	55	38	38	38	38	37	38	38	38	38	37	38	38	38	38	38	38	38	38
GCA_002395105.1_ASM239510v1	39	38	39	37	38	38	38	38	38	54	53	100	56	38	38	38	38	38	39	38	38	38	36	38	38	38	38	38	38	38	38
GCA_002394665.1_ASM239466v1	38	38	39	38	38	38	37	38	38	54	55	56	100	39	38	38	38	38	39	38	38	39	37	39	39	38	38	39	39	39	38
GCA_014190355.1_ASM1419035v1	53	50	46	41	44	44	45	42	42	38	38	38	39	100	70	57	61	61	61	59	60	59	58	60	62	61	61	60	60	60	57
GCA_903837365.1_freshwater_MAG	53	50	45	41	44	44	44	42	42	38	38	38	38	70	100	57	62	62	61	60	61	60	58	62	63	62	62	61	61	62	57
GCA_009921385.1_ASM992138v1	51	49	45	41	44	44	44	42	41	38	38	38	38	57	57	100	58	58	58	56	56	56	54	56	57	57	57	56	57	56	54
GCA_903959715.1_freshwater_MAG	53	50	46	41	44	44	45	42	42	38	38	38	38	61	62	58	100	86	72	61	61	60	58	62	62	62	62	62	62	62	58
GCA_005787525.1_ASM578752v1	53	50	46	41	44	44	45	42	42	37	37	38	38	61	62	58	86	100	72	61	61	60	58	61	62	62	62	62	62	62	57
GCA_009925685.1_ASM992568v1	53	51	46	41	44	44	45	42	42	38	38	39	39	61	61	58	72	72	100	60	60	60	57	61	62	62	61	61	61	61	57
GCA_009924125.1_ASM992412v1	54	51	46	41	44	44	45	42	42	37	38	38	38	59	60	56	61	61	60	100	100	68	63	67	67	70	70	69	70	71	60
GCA_002359655.1_ASM235965v1	55	51	46	41	44	44	45	42	42	37	38	38	38	60	61	56	61	61	60	100	100	68	63	67	67	70	70	69	70	71	60
GCA_018969185.1_ASM1896918v1	55	51	46	41	45	45	45	43	43	38	38	38	39	59	60	56	60	60	60	68	68	100	62	65	65	67	67	66	67	67	60
GCA_009693885.1_ASM969388v1	53	50	44	40	43	44	44	41	41	37	37	36	37	58	58	54	58	58	57	63	63	62	100	62	63	64	64	63	64	63	57
GCA_903917585.1_freshwater_MAG	54	51	46	42	44	45	45	42	42	38	38	38	39	60	62	56	62	61	61	67	67	65	62	100	69	70	70	68	69	68	60
GCA_001768015.1_ASM176801v1	55	51	46	41	45	45	45	42	43	38	38	38	39	62	63	57	62	62	62	67	67	65	63	69	100	71	71	69	69	69	61
GCA_903927415.1_freshwater_MAG	55	52	46	42	44	45	45	42	42	38	38	38	38	61	62	57	62	62	62	70	70	67	64	70	71	100	81	72	72	73	61
GCA_903878685.1_freshwater_MAG	55	52	46	41	44	45	45	43	42	38	38	38	38	61	62	57	62	62	61	70	70	67	64	70	71	81	100	71	72	72	61
GCA_017999675.1_ASM1799967v1	54	51	45	41	44	45	45	42	42	38	38	38	39	60	61	56	62	62	61	69	69	66	63	68	69	72	71	100	82	74	61
GCA_903831915.1_freshwater_MAG	54	51	45	41	44	44	44	42	42	37	38	38	39	60	61	57	62	62	61	70	70	67	64	69	69	72	72	82	100	74	61
GCA_903872175.1_freshwater_MAG	54	51	45	41	44	44	44	42	42	38	38	38	39	60	62	56	62	62	61	71	71	67	63	68	69	73	72	74	74	100	62
GCA_016778745.1_ASM1677874v1	54	51	46	41	44	45	45	42	42	38	38	38	38	57	57	54	58	57	57	60	60	60	57	60	61	61	61	61	61	62	100

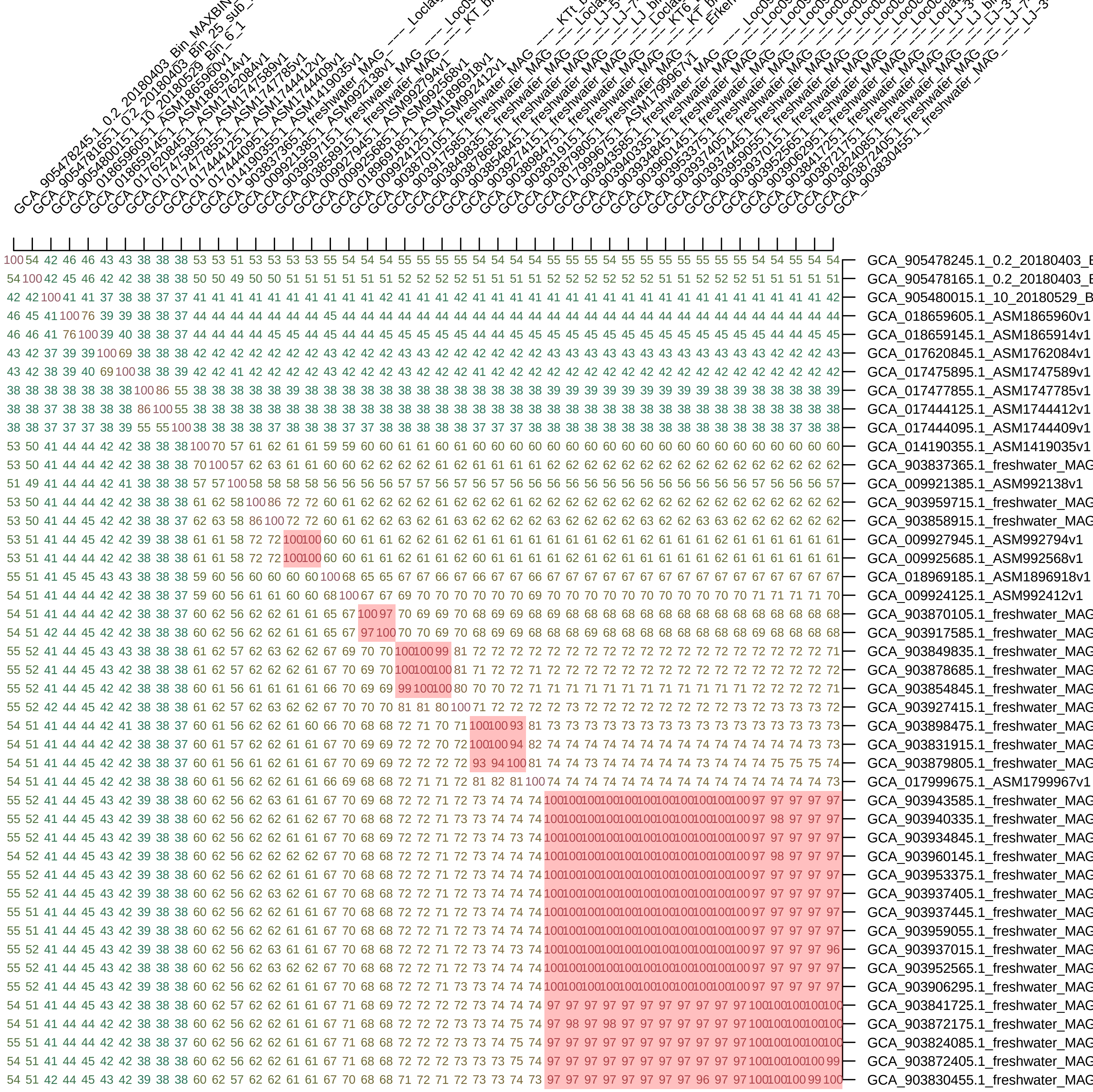
GCA_905478245.1_0.2_20180403_B
GCA_905478165.1_0.2_20180403_B
GCA_905480015.1_10_20180529_B
GCA_018659605.1_ASM1865960v1
GCA_018659145.1_ASM1865914v1
GCA_017620845.1_ASM1762084v1
GCA_017475895.1_ASM1747589v1
GCA_017477855.1_ASM1747785v1
GCA_017444125.1_ASM1744412v1
GCA_017444095.1_ASM1744409v1
GCA_014190355.1_ASM1419035v1
GCA_903837365.1_freshwater_MAG
GCA_009921385.1_ASM992138v1
GCA_903959715.1_freshwater_MAG
GCA_903858915.1_freshwater_MAG
GCA_009927945.1_ASM992794v1
GCA_009925685.1_ASM992568v1
GCA_018969185.1_ASM1896918v1
GCA_009924125.1_ASM992412v1
GCA_903870105.1_freshwater_MAG
GCA_903917585.1_freshwater_MAG
GCA_903849835.1_freshwater_MAG
GCA_903878685.1_freshwater_MAG
GCA_903854845.1_freshwater_MAG
GCA_903927415.1_freshwater_MAG
GCA_903898475.1_freshwater_MAG
GCA_903831915.1_freshwater_MAG
GCA_903879805.1_freshwater_MAG
GCA_017999675.1_ASM1799967v1
GCA_903943585.1_freshwater_MAG
GCA_903940335.1_freshwater_MAG
GCA_903934845.1_freshwater_MAG
GCA_903960145.1_freshwater_MAG
GCA_903953375.1_freshwater_MAG
GCA_903937405.1_freshwater_MAG
GCA_903937445.1_freshwater_MAG
GCA_903959055.1_freshwater_MAG
GCA_903937015.1_freshwater_MAG
GCA_903952565.1_freshwater_MAG
GCA_903906295.1_freshwater_MAG
GCA_903841725.1_freshwater_MAG
GCA_903872175.1_freshwater_MAG
GCA_903824085.1_freshwater_MAG
GCA_903872405.1_freshwater_MAG
GCA_903830455.1_freshwater_MAG

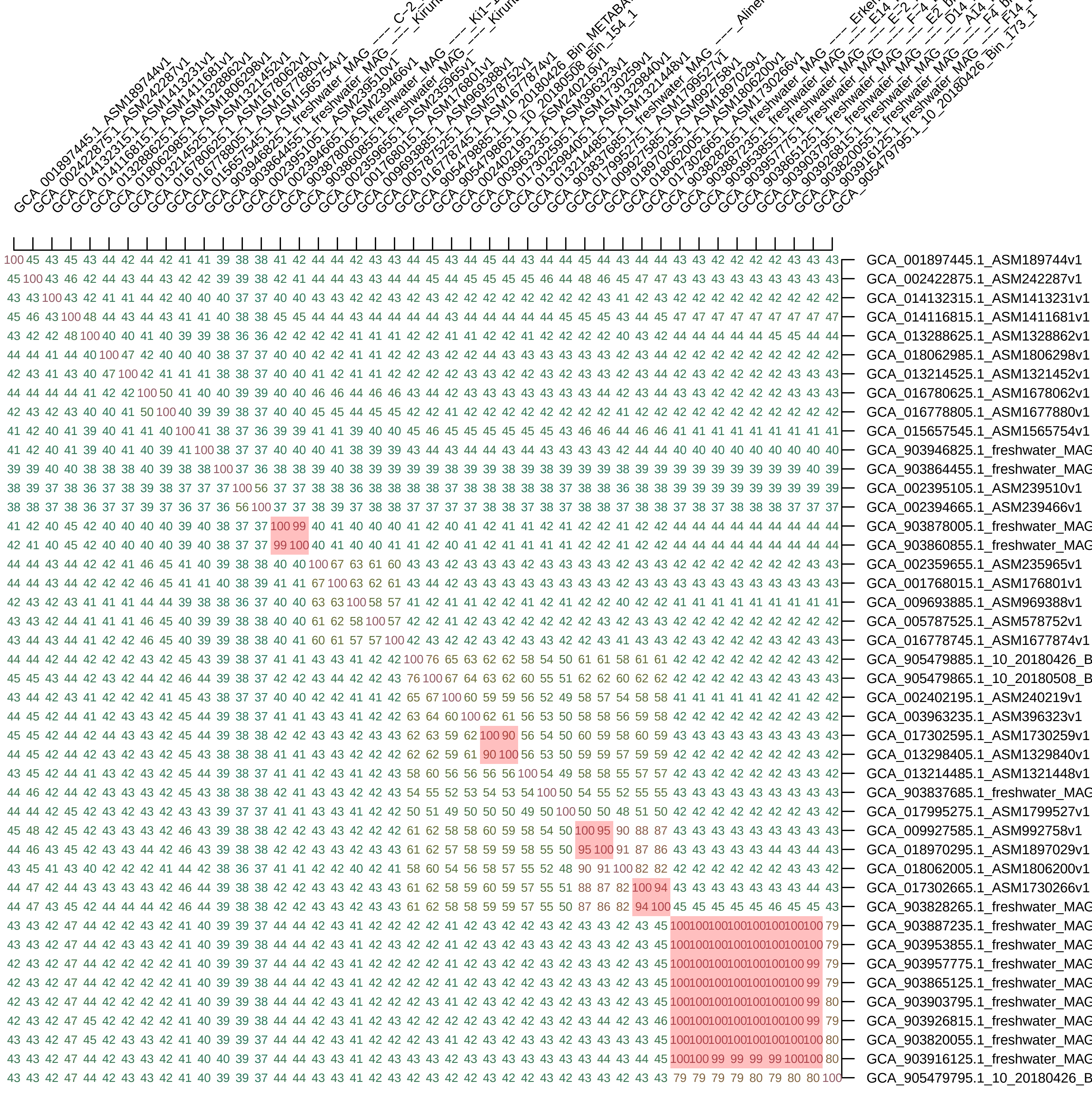
GCA_001897445.1_ASM189744v1
GCA_002422875.1_ASM242287v1
GCA_014132315.1_ASM1413231v1
GCA_014116815.1_ASM1411681v1
GCA_013288625.1_ASM1328862v1
GCA_018062985.1_ASM1806298v1
GCA_013214525.1_ASM1321452v1
GCA_016780625.1_ASM1678062v1
GCA_016778805.1_ASM1677880v1
GCA_015657545.1_ASM1565754v1
GCA_903946825.1_freshwater_MAG
GCA_903864455.1_freshwater_MAG
GCA_002395105.1_ASM239510v1
GCA_002394665.1_ASM239466v1
GCA_903878005.1_freshwater_MAG
GCA_903860855.1_freshwater_MAG
GCA_002359655.1_ASM235965v1
GCA_001768015.1_ASM176801v1
GCA_009693885.1_ASM969388v1
GCA_005787525.1_ASM578752v1
GCA_016778745.1_ASM1677874v1
GCA_905479885.1_10_20180426_B
GCA_905479865.1_10_20180508_B
GCA_002402195.1_ASM240219v1
GCA_003963235.1_ASM396323v1
GCA_017302595.1_ASM1730259v1
GCA_013298405.1_ASM1329840v1
GCA_013214485.1_ASM1321448v1
GCA_903837685.1_freshwater_MAG
GCA_017995275.1_ASM1799527v1
GCA_009927585.1_ASM992758v1
GCA_018970295.1_ASM1897029v1
GCA_018062005.1_ASM1806200v1
GCA_017302665.1_ASM1730266v1
GCA_903828265.1_freshwater_MAG
GCA_903887235.1_freshwater_MAG
GCA_903953855.1_freshwater_MAG
GCA_903957775.1_freshwater_MAG
GCA_903865125.1_freshwater_MAG
GCA_903903795.1_freshwater_MAG
GCA_903926815.1_freshwater_MAG
GCA_903820055.1_freshwater_MAG
GCA_903916125.1_freshwater_MAG
GCA_905479795.1_10_20180426_B

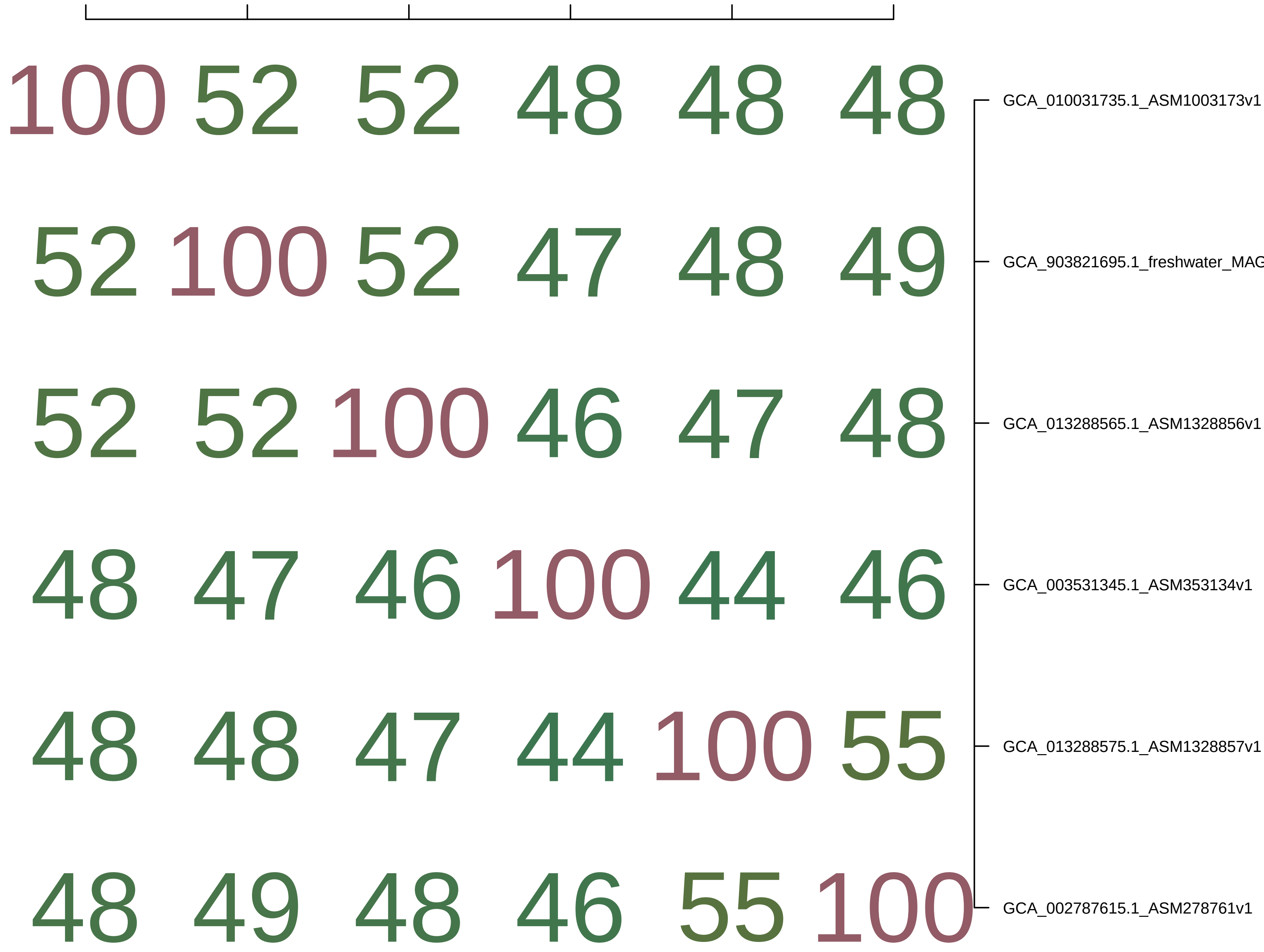

100 52 52 48 48 48
52 100 52 47 48 49
52 52 100 46 47 48
48 47 46 100 44 46
48 48 47 44 100 55
48 49 48 46 55 100
GCA_010031735.1_ASM1003173v1
GCA_903821695.1_freshwater_MAG
GCA_013288565.1_ASM1328856v1
GCA_003531345.1_ASM353134v1
GCA_013288575.1_ASM1328857v1
GCA_002787615.1_ASM278761v1

GCA_005792635.1_ASM57926	GCA_009926845.1_ASM99268	GCA_013288305.1_ASM13288	GCA_013288335.1_ASM13288	GCA_013288985.1_ASM13288	GCA_013289445.1_ASM13289	GCA_002423155.1_ASM24231	GCA_004293935.1_ASM42939	GCA_002422205.1_ASM24222	GCA_002725445.1_ASM27254	GCA_002346425.1_ASM23464	GCA_002422745.1_ASM24227	GCA_003241645.1_ASM32416	GCA_002291925.1_ASM22919	GCA_002291845.1_ASM22918	
100	62	60	52	52	51	53	49	53	50	50	50	51	51	51	GCA_005792635.1_ASM579263v1
62	100	60	52	52	51	52	49	53	50	49	50	52	52	52	GCA_009926845.1_ASM992684v1
60	60	100	51	52	51	51	48	51	49	50	48	50	51	50	GCA_013288305.1_ASM1328830v1
52	52	51	100	61	60	51	47	50	49	48	48	49	50	49	GCA_013288335.1_ASM1328833v1
52	52	52	61	100	60	51	47	51	49	49	49	50	51	50	GCA_013288985.1_ASM1328898v1
51	51	51	60	60	100	50	47	51	49	48	48	50	50	49	GCA_013289445.1_ASM1328944v1
53	52	51	51	51	50	100	51	53	51	50	50	51	54	54	GCA_002423155.1_ASM242315v1
49	49	48	47	47	47	51	100	50	49	49	47	49	52	51	GCA_004293935.1_ASM429393v1
53	53	51	50	51	51	53	50	100	58	57	54	55	53	53	GCA_002422205.1_ASM242220v1
50	50	49	49	49	49	51	49	58	100	55	53	54	52	51	GCA_002725445.1_ASM272544v1
50	49	50	48	49	48	50	49	57	55	100	51	52	51	51	GCA_002346425.1_ASM234642v1
50	50	48	48	49	48	50	47	54	53	51	100	62	51	51	GCA_002422745.1_ASM242274v1
51	52	50	49	50	50	51	49	55	54	52	62	100	52	52	GCA_003241645.1_ASM324164v1
51	52	51	50	51	50	54	52	53	52	51	51	52	100	74	GCA_002291925.1_ASM229192v1
51	52	50	49	50	49	54	51	53	51	51	51	52	74	100	GCA_002291845.1_ASM229184v1

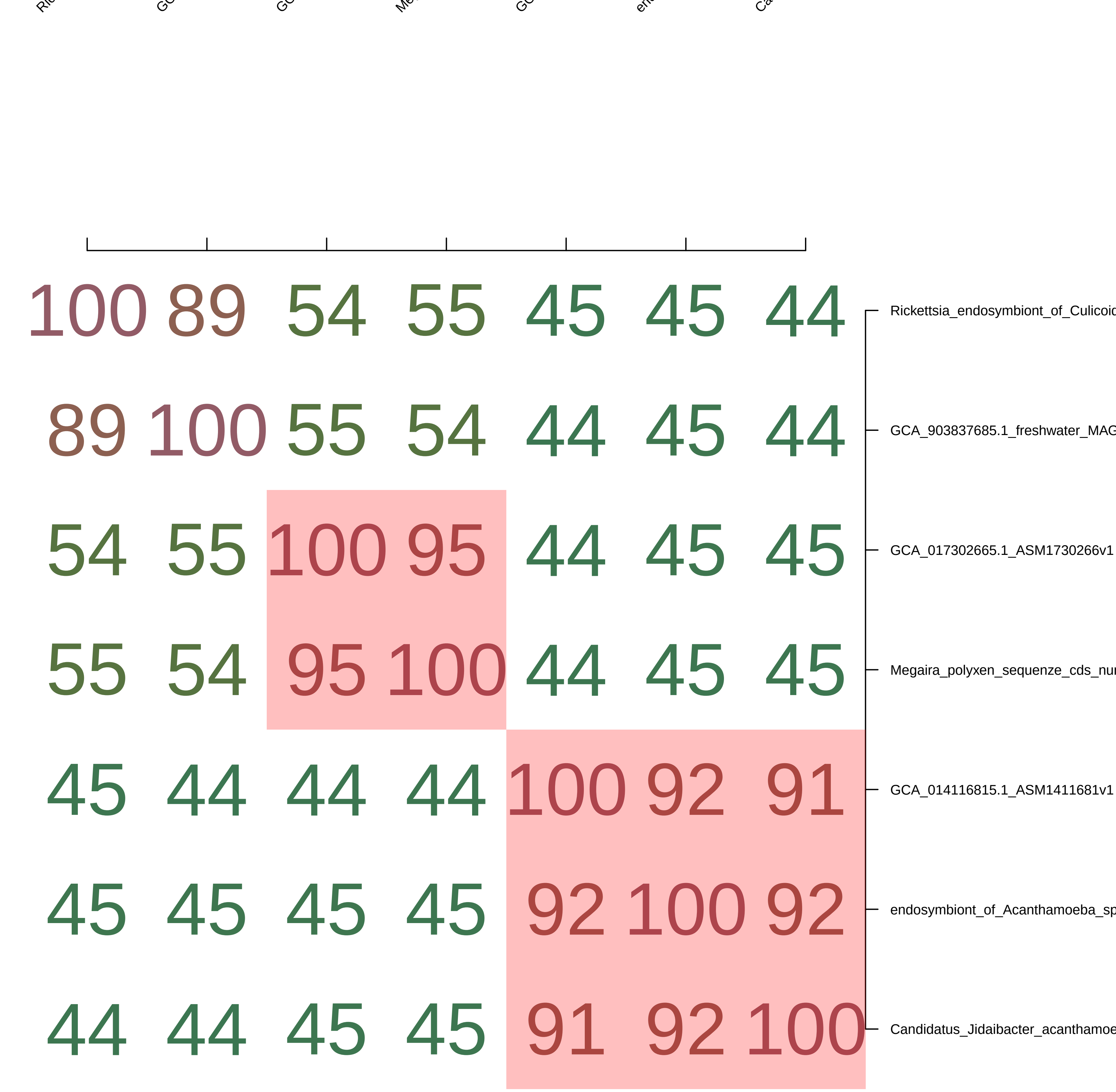

100 89 54 55 45 45 44
89 100 55 54 44 45 44
54 55 100 95 44 45 45
55 54 95 100 44 45 45
45 44 44 44 100 92 91
45 45 45 45 92 100 92
44 44 45 45 91 92 100
Rickettsia_endosymbiont_of_Culicoid
GCA_903837685.1_freshwater_MAG
GCA_017302665.1_ASM1730266v1
Megaira_polyxen_sequenze_cds_nu
GCA_014116815.1_ASM1411681v1
endosymbiont_of_Acanthamoeba_sp
Candidatus_Jidaibacter_acanthamoe

Supplementary Figure 31: "step 5" phylogenomic trees. Phylogenomic trees of *Rickettsiales* and other *Alphaproteobacteria*, for testing the affiliation to *Rickettsiales* of the members of each previously identified MAG groups (Groups A-M; Step 5 in Supplementary Note 4). For each clade, the phylogenies on the respective untrimmed concatenated gene alignment, as well as on alignments with the 10%, 20%, 30%, 40%, or 50% most compositionally heterogeneous sites removed, are reported in order. In each tree, the investigated MAGs are highlighted in blue. On each branch, support values by SH-aLRT with 1000 replicates and by 1000 ultrafast bootstraps are reported.

Group A

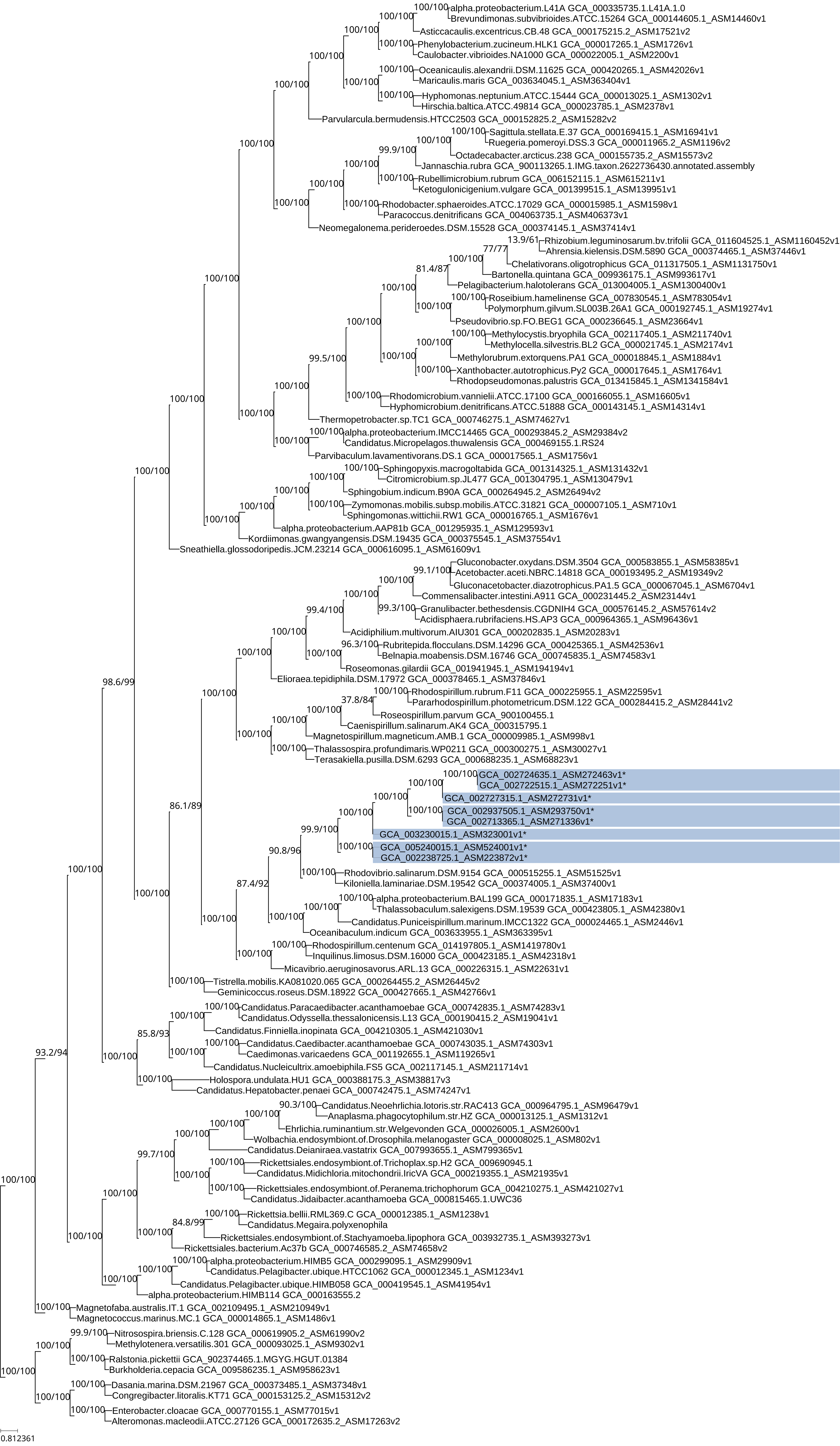

100/100
alpha.proteobacterium.L41A GCA_000335735.1.L41A.1.0
100/100
Brevundimonas.subvibrioides.ATCC.15264 GCA_000144605.1_ASM14460v1
100/100
Asticcacaulis.excentricus.CB.48 GCA_000175215.2_ASM17521v2
100/100
Phenylobacterium.zucineum.HLK1 GCA_000017265.1_ASM1726v1
100/100
Caulobacter.vibrioides.NA1000 GCA_000022005.1_ASM2200v1
100/100
Oceanicaulis.alexandrii.DSM.11625 GCA_000420265.1_ASM42026v1
100/100
Maricaulis.maris GCA_003634045.1_ASM363404v1
100/100
Hyphomonas.neptunium.ATCC.15444 GCA_000013025.1_ASM1302v1
Hirschia.baltica.ATCC.49814 GCA_000023785.1_ASM2378v1
Parvularcula.bermudensis.HTCC2503 GCA_000152825.2_ASM15282v2
100/100
100/100
Sagittula.stellata.E.37 GCA_000169415.1_ASM16941v1
100/100
Ruegeria.pomeroyi.DSS.3 GCA_000011965.2_ASM1196v2
99.9/100
Octadecabacter.arcticus.238 GCA_000155735.2_ASM15573v2
Jannaschia.rubra GCA_900113265.1.IMG.taxon.2622736430.annotated.assembly
100/100
100/100
Rubellimicrobium.rubrum GCA_006152115.1_ASM615211v1
Ketogulonicigenium.vulgare GCA_001399515.1_ASM139951v1
100/100
100/100
Rhodobacter.sphaeroides.ATCC.17029 GCA_000015985.1_ASM1598v1
Paracoccus.denitrificans GCA_004063735.1_ASM406373v1
Neomegalonema.perideroedes.DSM.15528 GCA_000374145.1_ASM37414v1
13.9/61
Rhizobium.leguminosarum.bv.trifolii GCA_011604525.1_ASM1160452v1
77/77
Ahrensia.kielensis.DSM.5890 GCA_000374465.1_ASM37446v1
100/100
Chelativorans.oligotrophicus GCA_011317505.1_ASM1131750v1
81.4/87
Bartonella.quintana GCA_009936175.1_ASM993617v1
100/100
Pelagibacterium.halotolerans GCA_013004005.1_ASM1300400v1
100/100
Roseibium.hamelinense GCA_007830545.1_ASM783054v1
100/100
Polymorphum.gilvum.SL003B.26A1 GCA_000192745.1_ASM19274v1
100/100
Pseudovibrio.sp.FO.BEG1 GCA_000236645.1_ASM23664v1
100/100
Methylocystis.bryophila GCA_002117405.1_ASM211740v1
100/100
Methylocella.silvestris.BL2 GCA_000021745.1_ASM2174v1
100/100
Methylorubrum.extorquens.PA1 GCA_000018845.1_ASM1884v1
99.5/100
100/100
Xanthobacter.autotrophicus.Py2 GCA_000017645.1_ASM1764v1
Rhodopseudomonas.palustris GCA_013415845.1_ASM1341584v1
100/100
100/100
Rhodomicrobium.vannielii.ATCC.17100 GCA_000166055.1_ASM16605v1
100/100
Hyphomicrobium.denitrificans.ATCC.51888 GCA_000143145.1_ASM14314v1
100/100
Thermopetrobacter.sp.TC1 GCA_000746275.1_ASM74627v1
100/100
alpha.proteobacterium.IMCC14465 GCA_000293845.2_ASM29384v2
100/100
Candidatus.Micropelagos.thuwalensis GCA_000469155.1.RS24
Parvibaculum.lavamentivorans.DS.1 GCA_000017565.1_ASM1756v1
100/100
100/100
Sphingopyxis.macrogoltabida GCA_001314325.1_ASM131432v1
Citromicrobium.sp.JL477 GCA_001304795.1_ASM130479v1
100/100
Sphingobium.indicum.B90A GCA_000264945.2_ASM26494v2
100/100
Zymomonas.mobilis.subsp.mobilis.ATCC.31821 GCA_000007105.1_ASM710v1
100/100
Sphingomonas.wittichii.RW1 GCA_000016765.1_ASM1676v1
100/100
alpha.proteobacterium.AAP81b GCA_001295935.1_ASM129593v1
Kordiimonas.gwangyangensis.DSM.19435 GCA_000375545.1_ASM37554v1
Sneathiella.glossodoripedis.JCM.23214 GCA_000616095.1_ASM61609v1
99.1/100
Gluconobacter.oxydans.DSM.3504 GCA_000583855.1_ASM58385v1
100/100
Acetobacter.aceti.NBRC.14818 GCA_000193495.2_ASM19349v2
Gluconacetobacter.diazotrophicus.PA1.5 GCA_000067045.1_ASM6704v1
100/100
Commensalibacter.intestini.A911 GCA_000231445.2_ASM23144v1
99.4/100
99.3/100
Granulibacter.bethesdensis.CGDNIH4 GCA_000576145.2_ASM57614v2
Acidisphaera.rubrifaciens.HS.AP3 GCA_000964365.1_ASM96436v1
100/100
Acidiphilium.multivorum.AIU301 GCA_000202835.1_ASM20283v1
96.3/100
Rubritepida.flocculans.DSM.14296 GCA_000425365.1_ASM42536v1
100/100
100/100
Belnapia.moabensis.DSM.16746 GCA_000745835.1_ASM74583v1
Roseomonas.gilardii GCA_001941945.1_ASM194194v1
Elioraea.tepidiphila.DSM.17972 GCA_000378465.1_ASM37846v1
100/100
100/100
Rhodospirillum.rubrum.F11 GCA_000225955.1_ASM22595v1
37.8/84
Pararhodospirillum.photometricum.DSM.122 GCA_000284415.2_ASM28441v2
100/100
Roseospirillum.parvum GCA_900100455.1
100/100
Caenispirillum.salinarum.AK4 GCA_000315795.1
100/100
Magnetospirillum.magneticum.AMB.1 GCA_000009985.1_ASM998v1
100/100
Thalassospira.profundimaris.WP0211 GCA_000300275.1_ASM30027v1
Terasakiella.pusilla.DSM.6293 GCA_000688235.1_ASM68823v1
100/100
GCA_002724635.1_ASM272463v1*
100/100
GCA_002722515.1_ASM272251v1*
100/100
GCA_002727315.1_ASM272731v1*
100/100
GCA_002937505.1_ASM293750v1*
100/100
GCA_002713365.1_ASM271336v1*
99.9/100
GCA_003230015.1_ASM323001v1*
100/100
GCA_005240015.1_ASM524001v1*
GCA_002238725.1_ASM223872v1*
86.1/89
90.8/96
100/100
Rhodovibrio.salinarum.DSM.9154 GCA_000515255.1_ASM51525v1
87.4/92
Kiloniella.laminariae.DSM.19542 GCA_000374005.1_ASM37400v1
98.6/99
100/100
alpha.proteobacterium.BAL199 GCA_000171835.1_ASM17183v1
100/100
100/100
Thalassobaculum.salexigens.DSM.19539 GCA_000423805.1_ASM42380v1
Candidatus.Puniceispirillum.marinum.IMCC1322 GCA_000024465.1_ASM2446v1
100/100
Oceanibaculum.indicum GCA_003633955.1_ASM363395v1
100/100
100/100
Rhodospirillum.centenum GCA_014197805.1_ASM1419780v1
Inquilinus.limosus.DSM.16000 GCA_000423185.1_ASM42318v1
Micavibrio.aeruginosavorus.ARL.13 GCA_000226315.1_ASM22631v1
100/100
Tistrella.mobilis.KA081020.065 GCA_000264455.2_ASM26445v2
Geminicoccus.roseus.DSM.18922 GCA_000427665.1_ASM42766v1
100/100
Candidatus.Paracaedibacter.acanthamoebae GCA_000742835.1_ASM74283v1
100/100
Candidatus.Odyssella.thessalonicensis.L13 GCA_000190415.2_ASM19041v1
85.8/93
Candidatus.Finniella.inopinata GCA_004210305.1_ASM421030v1
100/100
Candidatus.Caedibacter.acanthamoebae GCA_000743035.1_ASM74303v1
100/100
100/100
Caedimonas.varicaedens GCA_001192655.1_ASM119265v1
Candidatus.Nucleicultrix.amoebiphila.FS5 GCA_002117145.1_ASM211714v1
93.2/94
100/100
Holospora.undulata.HU1 GCA_000388175.3_ASM38817v3
Candidatus.Hepatobacter.penaei GCA_000742475.1_ASM74247v1
90.3/100
Candidatus.Neoehrlichia.lotoris.str.RAC413 GCA_000964795.1_ASM96479v1
100/100
Anaplasma.phagocytophilum.str.HZ GCA_000013125.1_ASM1312v1
100/100
Ehrlichia.ruminantium.str.Welgevonden GCA_000026005.1_ASM2600v1
100/100
Wolbachia.endosymbiont.of.Drosophila.melanogaster GCA_000008025.1_ASM802v1
Candidatus.Deianiraea.vastatrix GCA_007993655.1_ASM799365v1
99.7/100
100/100
Rickettsiales.endosymbiont.of.Trichoplax.sp.H2 GCA_009690945.1
100/100
Candidatus.Midichloria.mitochondrii.IricVA GCA_000219355.1_ASM21935v1
100/100
Rickettsiales.endosymbiont.of.Peranema.trichophorum GCA_004210275.1_ASM421027v1
100/100
Candidatus.Jidaibacter.acanthamoeba GCA_000815465.1.UWC36
100/100
Rickettsia.bellii.RML369.C GCA_000012385.1_ASM1238v1
84.8/99
Candidatus.Megaira.polyxenophila
100/100
100/100
Rickettsiales.endosymbiont.of.Stachyamoeba.lipophora GCA_003932735.1_ASM393273v1
Rickettsiales.bacterium.Ac37b GCA_000746585.2_ASM74658v2
100/100
alpha.proteobacterium.HIMB5 GCA_000299095.1_ASM29909v1
100/100
Candidatus.Pelagibacter.ubique.HTCC1062 GCA_000012345.1_ASM1234v1
100/100
Candidatus.Pelagibacter.ubique.HIMB058 GCA_000419545.1_ASM41954v1
alpha.proteobacterium.HIMB114 GCA_000163555.2
100/100
Magnetofaba.australis.IT.1 GCA_002109495.1_ASM210949v1
Magnetococcus.marinus.MC.1 GCA_000014865.1_ASM1486v1
99.9/100
Nitrosospira.briensis.C.128 GCA_000619905.2_ASM61990v2
100/100
Methylotenera.versatilis.301 GCA_000093025.1_ASM9302v1
100/100
Ralstonia.pickettii GCA_902374465.1.MGYG.HGUT.01384
Burkholderia.cepacia GCA_009586235.1_ASM958623v1
100/100
100/100
Dasania.marina.DSM.21967 GCA_000373485.1_ASM37348v1
100/100
Congregibacter.litoralis.KT71 GCA_000153125.2_ASM15312v2
100/100
Enterobacter.cloacae GCA_000770155.1_ASM77015v1
Alteromonas.macleodii.ATCC.27126 GCA_000172635.2_ASM17263v2
0.812361

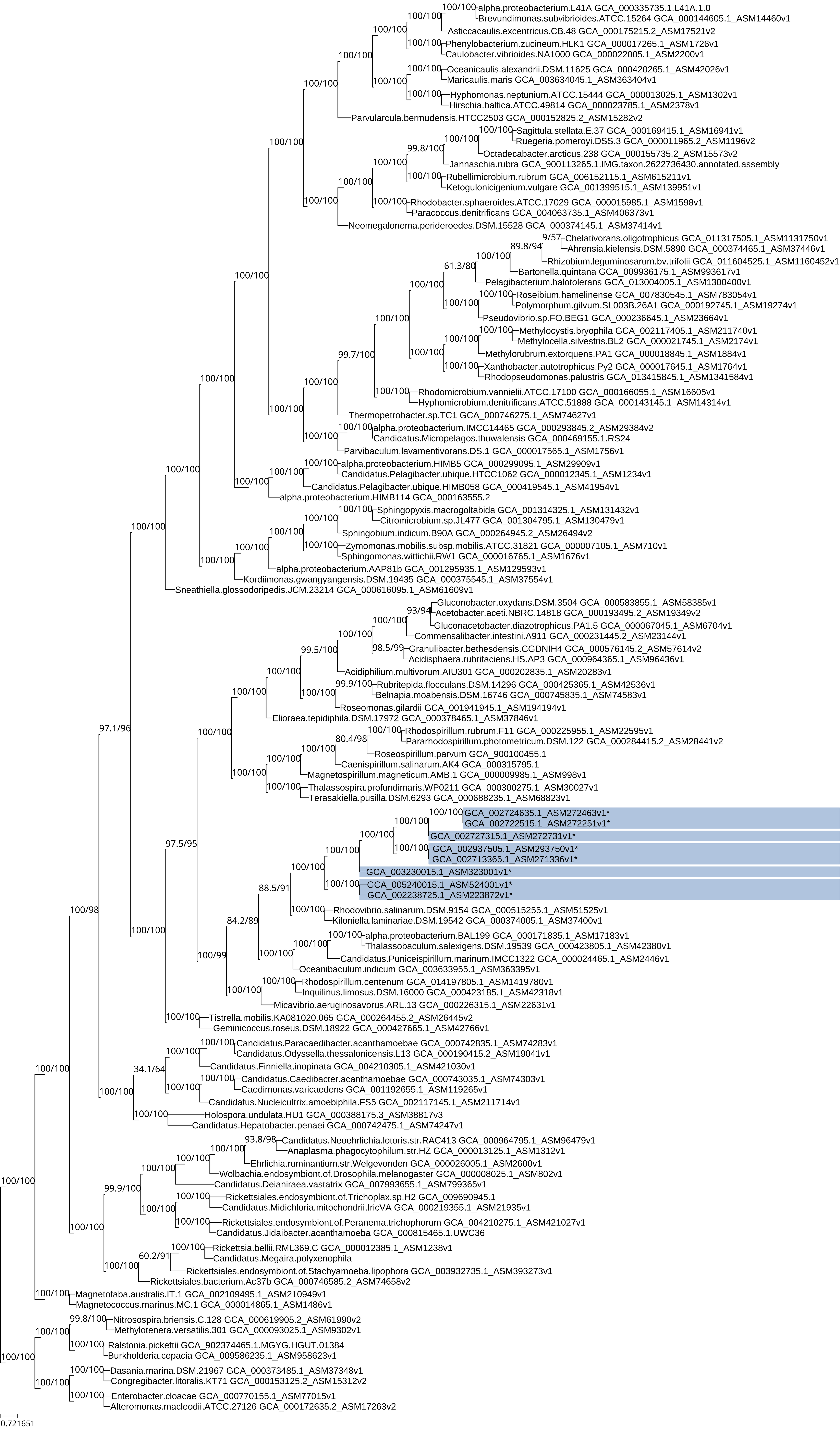
alpha.proteobacterium.L41A GCA_000335735.1.L41A.1.0
Brevundimonas.subvibrioides.ATCC.15264 GCA_000144605.1_ASM14460v1
Asticcacaulis.excentricus.CB.48 GCA_000175215.2_ASM17521v2
Phenylobacterium.zucineum.HLK1 GCA_000017265.1_ASM1726v1
Caulobacter.vibrioides.NA1000 GCA_000022005.1_ASM2200v1
Oceanicaulis.alexandrii.DSM.11625 GCA_000420265.1_ASM42026v1
Maricaulis.maris GCA_003634045.1_ASM363404v1
Hyphomonas.neptunium.ATCC.15444 GCA_000013025.1_ASM1302v1
Hirschia.baltica.ATCC.49814 GCA_000023785.1_ASM2378v1
Parvularcula.bermudensis.HTCC2503 GCA_000152825.2_ASM15282v2
Sagittula.stellata.E.37 GCA_000169415.1_ASM16941v1
Ruegeria.pomeroyi.DSS.3 GCA_000011965.2_ASM1196v2
Octadecabacter.arcticus.238 GCA_000155735.2_ASM15573v2
Jannaschia.rubra GCA_900113265.1.IMG.taxon.2622736430.annotated.assembly
Rubellimicrobium.rubrum GCA_006152115.1_ASM615211v1
Ketogulonicigenium.vulgare GCA_001399515.1_ASM139951v1
Rhodobacter.sphaeroides.ATCC.17029 GCA_000015985.1_ASM1598v1
Paracoccus.denitrificans GCA_004063735.1_ASM406373v1
Neomegalonema.perideroedes.DSM.15528 GCA_000374145.1_ASM37414v1
Chelativorans.oligotrophicus GCA_011317505.1_ASM1131750v1
Ahrensia.kielensis.DSM.5890 GCA_000374465.1_ASM37446v1
Rhizobium.leguminosarum.bv.trifolii GCA_011604525.1_ASM1160452v1
Bartonella.quintana GCA_009936175.1_ASM993617v1
Pelagibacterium.halotolerans GCA_013004005.1_ASM1300400v1
Roseibium.hamelinense GCA_007830545.1_ASM783054v1
Polymorphum.gilvum.SL003B.26A1 GCA_000192745.1_ASM19274v1
Pseudovibrio.sp.FO.BEG1 GCA_000236645.1_ASM23664v1
Methylocystis.bryophila GCA_002117405.1_ASM211740v1
Methylocella.silvestris.BL2 GCA_000021745.1_ASM2174v1
Methylorubrum.extorquens.PA1 GCA_000018845.1_ASM1884v1
Xanthobacter.autotrophicus.Py2 GCA_000017645.1_ASM1764v1
Rhodopseudomonas.palustris GCA_013415845.1_ASM1341584v1
Rhodomicrobium.vannielii.ATCC.17100 GCA_000166055.1_ASM16605v1
Hyphomicrobium.denitrificans.ATCC.51888 GCA_000143145.1_ASM14314v1
Thermopetrobacter.sp.TC1 GCA_000746275.1_ASM74627v1
alpha.proteobacterium.IMCC14465 GCA_000293845.2_ASM29384v2
Candidatus.Micropelagos.thuwalensis GCA_000469155.1.RS24
Parvibaculum.lavamentivorans.DS.1 GCA_000017565.1_ASM1756v1
alpha.proteobacterium.HIMB5 GCA_000299095.1_ASM29909v1
Candidatus.Pelagibacter.ubique.HTCC1062 GCA_000012345.1_ASM1234v1
Candidatus.Pelagibacter.ubique.HIMB058 GCA_000419545.1_ASM41954v1
alpha.proteobacterium.HIMB114 GCA_000163555.2
Sphingopyxis.macrogoltabida GCA_001314325.1_ASM131432v1
Citromicrobium.sp.JL477 GCA_001304795.1_ASM130479v1
Sphingobium.indicum.B90A GCA_000264945.2_ASM26494v2
Zymomonas.mobilis.subsp.mobilis.ATCC.31821 GCA_000007105.1_ASM710v1
Sphingomonas.wittichii.RW1 GCA_000016765.1_ASM1676v1
alpha.proteobacterium.AAP81b GCA_001295935.1_ASM129593v1
Kordiimonas.gwangyangensis.DSM.19435 GCA_000375545.1_ASM37554v1
Sneathiella.glossodoripedis.JCM.23214 GCA_000616095.1_ASM61609v1
Gluconobacter.oxydans.DSM.3504 GCA_000583855.1_ASM58385v1
Acetobacter.aceti.NBRC.14818 GCA_000193495.2_ASM19349v2
Gluconacetobacter.diazotrophicus.PA1.5 GCA_000067045.1_ASM6704v1
Commensalibacter.intestini.A911 GCA_000231445.2_ASM23144v1
Granulibacter.bethesdensis.CGDNIH4 GCA_000576145.2_ASM57614v2
Acidisphaera.rubrifaciens.HS.AP3 GCA_000964365.1_ASM96436v1
Acidiphilium.multivorum.AIU301 GCA_000202835.1_ASM20283v1
Rubritepida.flocculans.DSM.14296 GCA_000425365.1_ASM42536v1
Belnapia.moabensis.DSM.16746 GCA_000745835.1_ASM74583v1
Roseomonas.gilardii GCA_001941945.1_ASM194194v1
Elioraea.tepidiphila.DSM.17972 GCA_000378465.1_ASM37846v1
Rhodospirillum.rubrum.F11 GCA_000225955.1_ASM22595v1
Pararhodospirillum.photometricum.DSM.122 GCA_000284415.2_ASM28441v2
Roseospirillum.parvum GCA_900100455.1
Caenispirillum.salinarum.AK4 GCA_000315795.1
Magnetospirillum.magneticum.AMB.1 GCA_000009985.1_ASM998v1
Thalassospira.profundimaris.WP0211 GCA_000300275.1_ASM30027v1
Terasakiella.pusilla.DSM.6293 GCA_000688235.1_ASM68823v1
GCA_002724635.1_ASM272463v1*
GCA_002722515.1_ASM272251v1*
GCA_002727315.1_ASM272731v1*
GCA_002937505.1_ASM293750v1*
GCA_002713365.1_ASM271336v1*
GCA_003230015.1_ASM323001v1*
GCA_005240015.1_ASM524001v1*
GCA_002238725.1_ASM223872v1*
Rhodovibrio.salinarum.DSM.9154 GCA_000515255.1_ASM51525v1
Kiloniella.laminariae.DSM.19542 GCA_000374005.1_ASM37400v1
alpha.proteobacterium.BAL199 GCA_000171835.1_ASM17183v1
Thalassobaculum.salexigens.DSM.19539 GCA_000423805.1_ASM42380v1
Candidatus.Puniceispirillum.marinum.IMCC1322 GCA_000024465.1_ASM2446v1
Oceanibaculum.indicum GCA_003633955.1_ASM363395v1
Rhodospirillum.centenum GCA_014197805.1_ASM1419780v1
Inquilinus.limosus.DSM.16000 GCA_000423185.1_ASM42318v1
Micavibrio.aeruginosavorus.ARL.13 GCA_000226315.1_ASM22631v1
Tistrella.mobilis.KA081020.065 GCA_000264455.2_ASM26445v2
Geminicoccus.roseus.DSM.18922 GCA_000427665.1_ASM42766v1
Candidatus.Paracaedibacter.acanthamoebae GCA_000742835.1_ASM74283v1
Candidatus.Odyssella.thessalonicensis.L13 GCA_000190415.2_ASM19041v1
Candidatus.Finniella.inopinata GCA_004210305.1_ASM421030v1
Candidatus.Caedibacter.acanthamoebae GCA_000743035.1_ASM74303v1
Caedimonas.varicaedens GCA_001192655.1_ASM119265v1
Candidatus.Nucleicultrix.amoebiphila.FS5 GCA_002117145.1_ASM211714v1
Holospora.undulata.HU1 GCA_000388175.3_ASM38817v3
Candidatus.Hepatobacter.penaei GCA_000742475.1_ASM74247v1
Candidatus.Neoehrlichia.lotoris.str.RAC413 GCA_000964795.1_ASM96479v1
Anaplasma.phagocytophilum.str.HZ GCA_000013125.1_ASM1312v1
Ehrlichia.ruminantium.str.Welgevonden GCA_000026005.1_ASM2600v1
Wolbachia.endosymbiont.of.Drosophila.melanogaster GCA_000008025.1_ASM802v1
Candidatus.Deianiraea.vastatrix GCA_007993655.1_ASM799365v1
Rickettsiales.endosymbiont.of.Trichoplax.sp.H2 GCA_009690945.1
Candidatus.Midichloria.mitochondrii.IricVA GCA_000219355.1_ASM21935v1
Rickettsiales.endosymbiont.of.Peranema.trichophorum GCA_004210275.1_ASM421027v1
Candidatus.Jidaibacter.acanthamoeba GCA_000815465.1.UWC36
Rickettsia.bellii.RML369.C GCA_000012385.1_ASM1238v1
Candidatus.Megaira.polyxenophila
Rickettsiales.endosymbiont.of.Stachyamoeba.lipophora GCA_003932735.1_ASM393273v1
Rickettsiales.bacterium.Ac37b GCA_000746585.2_ASM74658v2
Magnetofaba.australis.IT.1 GCA_002109495.1_ASM210949v1
Magnetococcus.marinus.MC.1 GCA_000014865.1_ASM1486v1
Nitrosospira.briensis.C.128 GCA_000619905.2_ASM61990v2
Methylotenera.versatilis.301 GCA_000093025.1_ASM9302v1
Ralstonia.pickettii GCA_902374465.1.MGYG.HGUT.01384
Burkholderia.cepacia GCA_009586235.1_ASM958623v1
Dasania.marina.DSM.21967 GCA_000373485.1_ASM37348v1
Congregibacter.litoralis.KT71 GCA_000153125.2_ASM15312v2
Enterobacter.cloacae GCA_000770155.1_ASM77015v1
Alteromonas.macleodii.ATCC.27126 GCA_000172635.2_ASM17263v2
0.721651

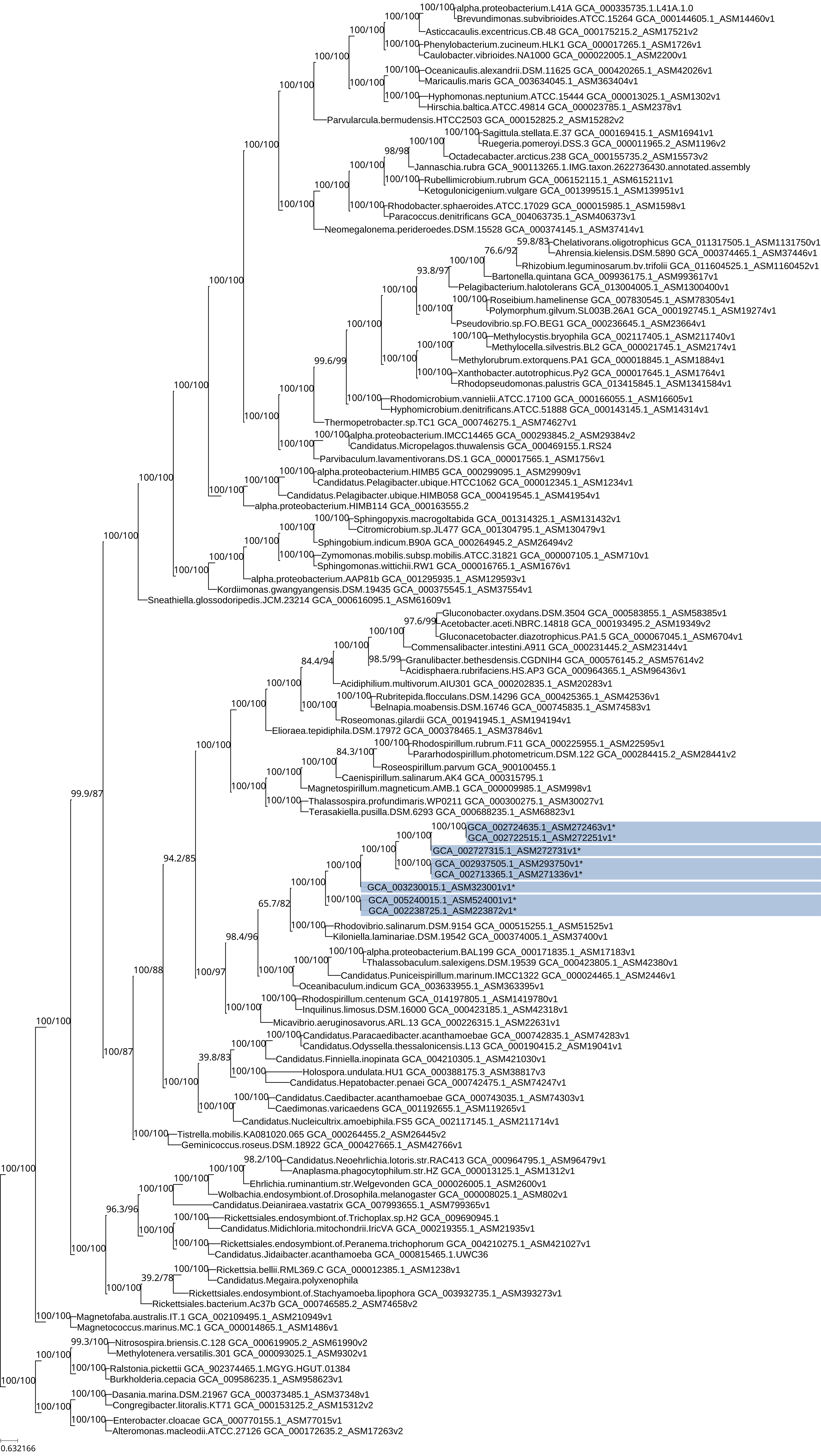
alpha.proteobacterium.L41A GCA_000335735.1_L41A.1.0
Brevundimonas.subvibrioides.ATCC.15264 GCA_000144605.1_ASM14460v1
Asticcacaulis.excentricus.CB.48 GCA_000175215.2_ASM17521v2
Phenylobacterium.zucineum.HLK1 GCA_000017265.1_ASM1726v1
Caulobacter.vibrioides.NA1000 GCA_000022005.1_ASM2200v1
Oceanicaulis.alexandrii.DSM.11625 GCA_000420265.1_ASM42026v1
Maricaulis.maris GCA_003634045.1_ASM363404v1
Hyphomonas.neptunium.ATCC.15444 GCA_000013025.1_ASM1302v1
Hirschia.baltica.ATCC.49814 GCA_000023785.1_ASM2378v1
Parvularcula.bermudensis.HTCC2503 GCA_000152825.2_ASM15282v2
Sagittula.stellata.E.37 GCA_000169415.1_ASM16941v1
Ruegeria.pomeroyi.DSS.3 GCA_000011965.2_ASM1196v2
Octadecabacter.arcticus.238 GCA_000155735.2_ASM15573v2
Jannaschia.rubra GCA_900113265.1.IMG.taxon.2622736430.annotated.assembly
Rubellimicrobium.rubrum GCA_006152115.1_ASM615211v1
Ketogulonicigenium.vulgare GCA_001399515.1_ASM139951v1
Rhodobacter.sphaeroides.ATCC.17029 GCA_000015985.1_ASM1598v1
Paracoccus.denitrificans GCA_004063735.1_ASM406373v1
Neomegalonema.perideroedes.DSM.15528 GCA_000374145.1_ASM37414v1
Chelativorans.oligotrophicus GCA_011317505.1_ASM1131750v1
Ahrensia.kielensis.DSM.5890 GCA_000374465.1_ASM37446v1
Rhizobium.leguminosarum.bv.trifolii GCA_011604525.1_ASM1160452v1
Bartonella.quintana GCA_009936175.1_ASM993617v1
Pelagibacterium.halotolerans GCA_013004005.1_ASM1300400v1
Roseibium.hamelinense GCA_007830545.1_ASM783054v1
Polymorphum.gilvum.SL003B.26A1 GCA_000192745.1_ASM19274v1
Pseudovibrio.sp.FO.BEG1 GCA_000236645.1_ASM23664v1
Methylocystis.bryophila GCA_002117405.1_ASM211740v1
Methylocella.silvestris.BL2 GCA_000021745.1_ASM2174v1
Methylorubrum.extorquens.PA1 GCA_000018845.1_ASM1884v1
Xanthobacter.autotrophicus.Py2 GCA_000017645.1_ASM1764v1
Rhodopseudomonas.palustris GCA_013415845.1_ASM1341584v1
Rhodomicrobium.vannielii.ATCC.17100 GCA_000166055.1_ASM16605v1
Hyphomicrobium.denitrificans.ATCC.51888 GCA_000143145.1_ASM14314v1
Thermopetrobacter.sp.TC1 GCA_000746275.1_ASM74627v1
alpha.proteobacterium.IMCC14465 GCA_000293845.2_ASM29384v2
Candidatus.Micropelagos.thuwalensis GCA_000469155.1.RS24
Parvibaculum.lavamentivorans.DS.1 GCA_000017565.1_ASM1756v1
alpha.proteobacterium.HIMB5 GCA_000299095.1_ASM29909v1
Candidatus.Pelagibacter.ubique.HTCC1062 GCA_000012345.1_ASM1234v1
Candidatus.Pelagibacter.ubique.HIMB058 GCA_000419545.1_ASM41954v1
alpha.proteobacterium.HIMB114 GCA_000163555.2
Sphingopyxis.macrogoltabida GCA_001314325.1_ASM131432v1
Citromicrobium.sp.JL477 GCA_001304795.1_ASM130479v1
Sphingobium.indicum.B90A GCA_000264945.2_ASM26494v2
Zymomonas.mobilis.subsp.mobilis.ATCC.31821 GCA_000007105.1_ASM710v1
Sphingomonas.wittichii.RW1 GCA_000016765.1_ASM1676v1
alpha.proteobacterium.AAP81b GCA_001295935.1_ASM129593v1
Kordiimonas.gwangyangensis.DSM.19435 GCA_000375545.1_ASM37554v1
Sneathiella.glossodoripedis.JCM.23214 GCA_000616095.1_ASM61609v1
Gluconobacter.oxydans.DSM.3504 GCA_000583855.1_ASM58385v1
Acetobacter.aceti.NBRC.14818 GCA_000193495.2_ASM19349v2
Gluconacetobacter.diazotrophicus.PA1.5 GCA_000067045.1_ASM6704v1
Commensalibacter.intestini.A911 GCA_000231445.2_ASM23144v1
Granulibacter.bethesdensis.CGDNIH4 GCA_000576145.2_ASM57614v2
Acidisphaera.rubrifaciens.HS.AP3 GCA_000964365.1_ASM96436v1
Acidiphilium.multivorum.AIU301 GCA_000202835.1_ASM20283v1
Rubritepida.flocculans.DSM.14296 GCA_000425365.1_ASM42536v1
Belnapia.moabensis.DSM.16746 GCA_000745835.1_ASM74583v1
Roseomonas.gilardii GCA_001941945.1_ASM194194v1
Elioraea.tepidiphila.DSM.17972 GCA_000378465.1_ASM37846v1
Rhodospirillum.rubrum.F11 GCA_000225955.1_ASM22595v1
Pararhodospirillum.photometricum.DSM.122 GCA_000284415.2_ASM28441v2
Roseospirillum.parvum GCA_900100455.1
Caenispirillum.salinarum.AK4 GCA_000315795.1
Magnetospirillum.magneticum.AMB.1 GCA_000009985.1_ASM998v1
Thalassospira.profundimaris.WP0211 GCA_000300275.1_ASM30027v1
Terasakiella.pusilla.DSM.6293 GCA_000688235.1_ASM68823v1
GCA_002724635.1_ASM272463v1*
GCA_002722515.1_ASM272251v1*
GCA_002727315.1_ASM272731v1*
GCA_002937505.1_ASM293750v1*
GCA_002713365.1_ASM271336v1*
GCA_003230015.1_ASM323001v1*
GCA_005240015.1_ASM524001v1*
GCA_002238725.1_ASM223872v1*
Rhodovibrio.salinarum.DSM.9154 GCA_000515255.1_ASM51525v1
Kiloniella.laminariae.DSM.19542 GCA_000374005.1_ASM37400v1
alpha.proteobacterium.BAL199 GCA_000171835.1_ASM17183v1
Thalassobaculum.salexigens.DSM.19539 GCA_000423805.1_ASM42380v1
Candidatus.Puniceispirillum.marinum.IMCC1322 GCA_000024465.1_ASM2446v1
Oceanibaculum.indicum GCA_003633955.1_ASM363395v1
Rhodospirillum.centenum GCA_014197805.1_ASM1419780v1
Inquilinus.limosus.DSM.16000 GCA_000423185.1_ASM42318v1
Micavibrio.aeruginosavorus.ARL.13 GCA_000226315.1_ASM22631v1
Candidatus.Paracaedibacter.acanthamoebae GCA_000742835.1_ASM74283v1
Candidatus.Odyssella.thessalonicensis.L13 GCA_000190415.2_ASM19041v1
Candidatus.Finniella.inopinata GCA_004210305.1_ASM421030v1
Holospora.undulata.HU1 GCA_000388175.3_ASM38817v3
Candidatus.Hepatobacter.penaei GCA_000742475.1_ASM74247v1
Candidatus.Caedibacter.acanthamoebae GCA_000743035.1_ASM74303v1
Caedimonas.varicaedens GCA_001192655.1_ASM119265v1
Candidatus.Nucleicultrix.amoebiphila.FS5 GCA_002117145.1_ASM211714v1
Tistrella.mobilis.KA081020.065 GCA_000264455.2_ASM26445v2
Geminicoccus.roseus.DSM.18922 GCA_000427665.1_ASM42766v1
Candidatus.Neoehrlichia.lotoris.str.RAC413 GCA_000964795.1_ASM96479v1
Anaplasma.phagocytophilum.str.HZ GCA_000013125.1_ASM1312v1
Ehrlichia.ruminantium.str.Welgevonden GCA_000026005.1_ASM2600v1
Wolbachia.endosymbiont.of.Drosophila.melanogaster GCA_000008025.1_ASM802v1
Candidatus.Deianiraea.vastatrix GCA_007993655.1_ASM799365v1
Rickettsiales.endosymbiont.of.Trichoplax.sp.H2 GCA_009690945.1
Candidatus.Midichloria.mitochondrii.IricVA GCA_000219355.1_ASM21935v1
Rickettsiales.endosymbiont.of.Peranema.trichophorum GCA_004210275.1_ASM421027v1
Candidatus.Jidaibacter.acanthamoeba GCA_000815465.1.UWC36
Rickettsia.bellii.RML369.C GCA_000012385.1_ASM1238v1
Candidatus.Megaira.polyxenophila
Rickettsiales.endosymbiont.of.Stachyamoeba.lipophora GCA_003932735.1_ASM393273v1
Rickettsiales.bacterium.Ac37b GCA_000746585.2_ASM74658v2
Magnetofaba.australis.IT.1 GCA_002109495.1_ASM210949v1
Magnetococcus.marinus.MC.1 GCA_000014865.1_ASM1486v1
Nitrosospira.briensis.C.128 GCA_000619905.2_ASM61990v2
Methylotenera.versatilis.301 GCA_000093025.1_ASM9302v1
Ralstonia.pickettii GCA_902374465.1.MGYG.HGUT.01384
Burkholderia.cepacia GCA_009586235.1_ASM958623v1
Dasania.marina.DSM.21967 GCA_000373485.1_ASM37348v1
Congregibacter.litoralis.KT71 GCA_000153125.2_ASM15312v2
Enterobacter.cloacae GCA_000770155.1_ASM77015v1
Alteromonas.macleodii.ATCC.27126 GCA_000172635.2_ASM17263v2
0.632166

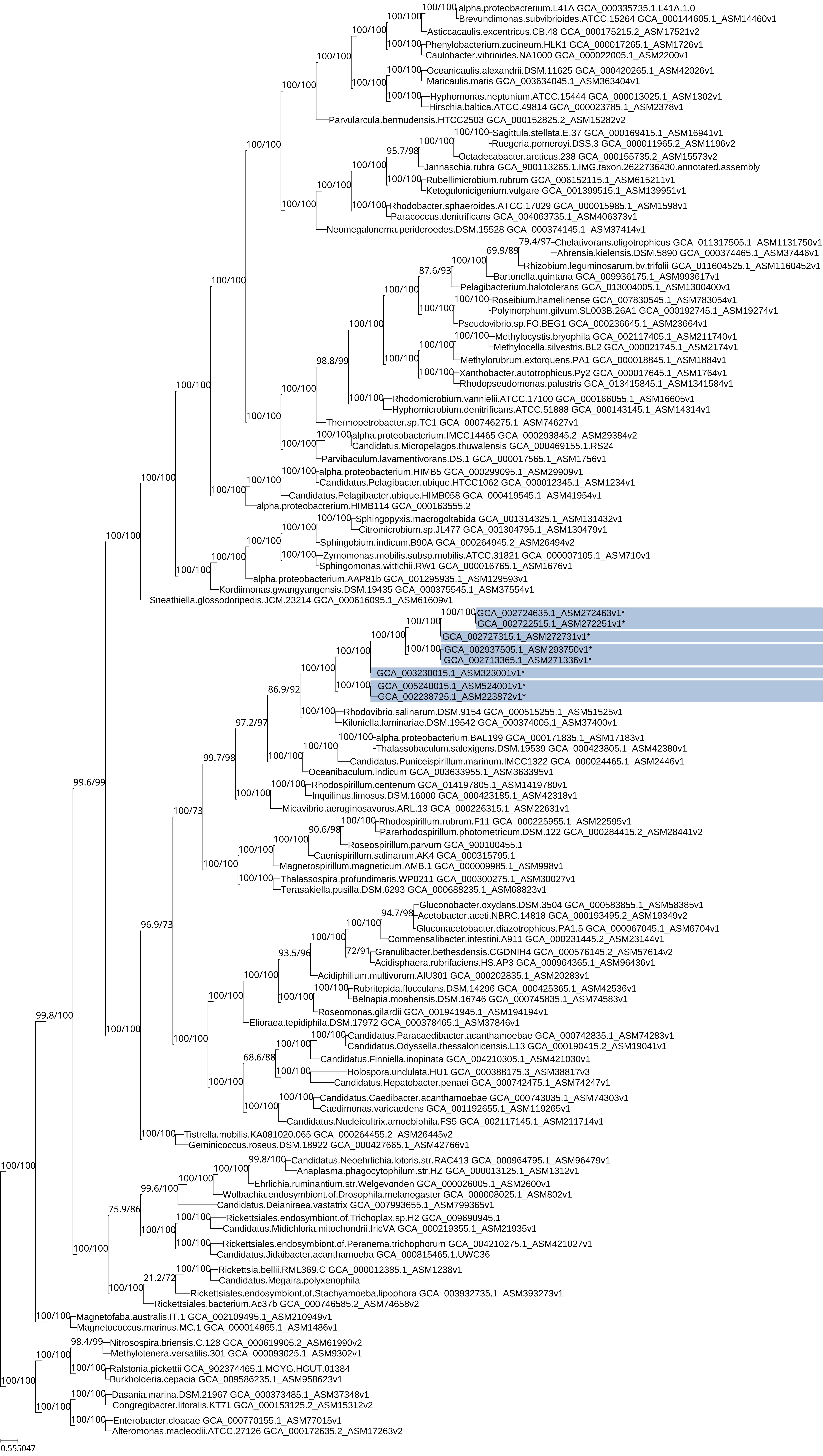
alpha.proteobacterium.L41A GCA_000335735.1_L41A.1.0
Brevundimonas.subvibrioides.ATCC.15264 GCA_000144605.1_ASM14460v1
Asticcacaulis.excentricus.CB.48 GCA_000175215.2_ASM17521v2
Phenylobacterium.zucineum.HLK1 GCA_000017265.1_ASM1726v1
Caulobacter.vibrioides.NA1000 GCA_000022005.1_ASM2200v1
Oceanicaulis.alexandrii.DSM.11625 GCA_000420265.1_ASM42026v1
Maricaulis.maris GCA_003634045.1_ASM363404v1
Hyphomonas.neptunium.ATCC.15444 GCA_000013025.1_ASM1302v1
Hirschia.baltica.ATCC.49814 GCA_000023785.1_ASM2378v1
Parvularcula.bermudensis.HTCC2503 GCA_000152825.2_ASM15282v2
Sagittula.stellata.E.37 GCA_000169415.1_ASM16941v1
Ruegeria.pomeroyi.DSS.3 GCA_000011965.2_ASM1196v2
Octadecabacter.arcticus.238 GCA_000155735.2_ASM15573v2
Jannaschia.rubra GCA_900113265.1.IMG.taxon.2622736430.annotated.assembly
Rubellimicrobium.rubrum GCA_006152115.1_ASM615211v1
Ketogulonicigenium.vulgare GCA_001399515.1_ASM139951v1
Rhodobacter.sphaeroides.ATCC.17029 GCA_000015985.1_ASM1598v1
Paracoccus.denitrificans GCA_004063735.1_ASM406373v1
Neomegalonema.perideroedes.DSM.15528 GCA_000374145.1_ASM37414v1
Chelativorans.oligotrophicus GCA_011317505.1_ASM1131750v1
Ahrensia.kielensis.DSM.5890 GCA_000374465.1_ASM37446v1
Rhizobium.leguminosarum.bv.trifolii GCA_011604525.1_ASM1160452v1
Bartonella.quintana GCA_009936175.1_ASM993617v1
Pelagibacterium.halotolerans GCA_013004005.1_ASM1300400v1
Roseibium.hamelinense GCA_007830545.1_ASM783054v1
Polymorphum.gilvum.SL003B.26A1 GCA_000192745.1_ASM19274v1
Pseudovibrio.sp.FO.BEG1 GCA_000236645.1_ASM23664v1
Methylocystis.bryophila GCA_002117405.1_ASM211740v1
Methylocella.silvestris.BL2 GCA_000021745.1_ASM2174v1
Methylorubrum.extorquens.PA1 GCA_000018845.1_ASM1884v1
Xanthobacter.autotrophicus.Py2 GCA_000017645.1_ASM1764v1
Rhodopseudomonas.palustris GCA_013415845.1_ASM1341584v1
Rhodomicrobium.vannielii.ATCC.17100 GCA_000166055.1_ASM16605v1
Hyphomicrobium.denitrificans.ATCC.51888 GCA_000143145.1_ASM14314v1
Thermopetrobacter.sp.TC1 GCA_000746275.1_ASM74627v1
alpha.proteobacterium.IMCC14465 GCA_000293845.2_ASM29384v2
Candidatus.Micropelagos.thuwalensis GCA_000469155.1.RS24
Parvibaculum.lavamentivorans.DS.1 GCA_000017565.1_ASM1756v1
alpha.proteobacterium.HIMB5 GCA_000299095.1_ASM29909v1
Candidatus.Pelagibacter.ubique.HTCC1062 GCA_000012345.1_ASM1234v1
Candidatus.Pelagibacter.ubique.HIMB058 GCA_000419545.1_ASM41954v1
alpha.proteobacterium.HIMB114 GCA_000163555.2
Sphingopyxis.macrogoltabida GCA_001314325.1_ASM131432v1
Citromicrobium.sp.JL477 GCA_001304795.1_ASM130479v1
Sphingobium.indicum.B90A GCA_000264945.2_ASM26494v2
Zymomonas.mobilis.subsp.mobilis.ATCC.31821 GCA_000007105.1_ASM710v1
Sphingomonas.wittichii.RW1 GCA_000016765.1_ASM1676v1
alpha.proteobacterium.AAP81b GCA_001295935.1_ASM129593v1
Kordiimonas.gwangyangensis.DSM.19435 GCA_000375545.1_ASM37554v1
Sneathiella.glossodoripedis.JCM.23214 GCA_000616095.1_ASM61609v1
GCA_002724635.1_ASM272463v1*
GCA_002722515.1_ASM272251v1*
GCA_002727315.1_ASM272731v1*
GCA_002937505.1_ASM293750v1*
GCA_002713365.1_ASM271336v1*
GCA_003230015.1_ASM323001v1*
GCA_005240015.1_ASM524001v1*
GCA_002238725.1_ASM223872v1*
Rhodovibrio.salinarum.DSM.9154 GCA_000515255.1_ASM51525v1
Kiloniella.laminariae.DSM.19542 GCA_000374005.1_ASM37400v1
alpha.proteobacterium.BAL199 GCA_000171835.1_ASM17183v1
Thalassobaculum.salexigens.DSM.19539 GCA_000423805.1_ASM42380v1
Candidatus.Puniceispirillum.marinum.IMCC1322 GCA_000024465.1_ASM2446v1
Oceanibaculum.indicum GCA_003633955.1_ASM363395v1
Rhodospirillum.centenum GCA_014197805.1_ASM1419780v1
Inquilinus.limosus.DSM.16000 GCA_000423185.1_ASM42318v1
Micavibrio.aeruginosavorus.ARL.13 GCA_000226315.1_ASM22631v1
Rhodospirillum.rubrum.F11 GCA_000225955.1_ASM22595v1
Pararhodospirillum.photometricum.DSM.122 GCA_000284415.2_ASM28441v2
Roseospirillum.parvum GCA_900100455.1
Caenispirillum.salinarum.AK4 GCA_000315795.1
Magnetospirillum.magneticum.AMB.1 GCA_000009985.1_ASM998v1
Thalassospira.profundimaris.WP0211 GCA_000300275.1_ASM30027v1
Terasakiella.pusilla.DSM.6293 GCA_000688235.1_ASM68823v1
Gluconobacter.oxydans.DSM.3504 GCA_000583855.1_ASM58385v1
Acetobacter.aceti.NBRC.14818 GCA_000193495.2_ASM19349v2
Gluconacetobacter.diazotrophicus.PA1.5 GCA_000067045.1_ASM6704v1
Commensalibacter.intestini.A911 GCA_000231445.2_ASM23144v1
Granulibacter.bethesdensis.CGDNIH4 GCA_000576145.2_ASM57614v2
Acidisphaera.rubrifaciens.HS.AP3 GCA_000964365.1_ASM96436v1
Acidiphilium.multivorum.AIU301 GCA_000202835.1_ASM20283v1
Rubritepida.flocculans.DSM.14296 GCA_000425365.1_ASM42536v1
Belnapia.moabensis.DSM.16746 GCA_000745835.1_ASM74583v1
Roseomonas.gilardii GCA_001941945.1_ASM194194v1
Elioraea.tepidiphila.DSM.17972 GCA_000378465.1_ASM37846v1
Candidatus.Paracaedibacter.acanthamoebae GCA_000742835.1_ASM74283v1
Candidatus.Odyssella.thessalonicensis.L13 GCA_000190415.2_ASM19041v1
Candidatus.Finniella.inopinata GCA_004210305.1_ASM421030v1
Holospora.undulata.HU1 GCA_000388175.3_ASM38817v3
Candidatus.Hepatobacter.penaei GCA_000742475.1_ASM74247v1
Candidatus.Caedibacter.acanthamoebae GCA_000743035.1_ASM74303v1
Caedimonas.varicaedens GCA_001192655.1_ASM119265v1
Candidatus.Nucleicultrix.amoebiphila.FS5 GCA_002117145.1_ASM211714v1
Tistrella.mobilis.KA081020.065 GCA_000264455.2_ASM26445v2
Geminicoccus.roseus.DSM.18922 GCA_000427665.1_ASM42766v1
Candidatus.Neoehrlichia.lotoris.str.RAC413 GCA_000964795.1_ASM96479v1
Anaplasma.phagocytophilum.str.HZ GCA_000013125.1_ASM1312v1
Ehrlichia.ruminantium.str.Welgevonden GCA_000026005.1_ASM2600v1
Wolbachia.endosymbiont.of.Drosophila.melanogaster GCA_000008025.1_ASM802v1
Candidatus.Deianiraea.vastatrix GCA_007993655.1_ASM799365v1
Rickettsiales.endosymbiont.of.Trichoplax.sp.H2 GCA_009690945.1
Candidatus.Midichloria.mitochondrii.IricVA GCA_000219355.1_ASM21935v1
Rickettsiales.endosymbiont.of.Peranema.trichophorum GCA_004210275.1_ASM421027v1
Candidatus.Jidaibacter.acanthamoeba GCA_000815465.1.UWC36
Rickettsia.bellii.RML369.C GCA_000012385.1_ASM1238v1
Candidatus.Megaira.polyxenophila
Rickettsiales.endosymbiont.of.Stachyamoeba.lipophora GCA_003932735.1_ASM393273v1
Rickettsiales.bacterium.Ac37b GCA_000746585.2_ASM74658v2
Magnetofaba.australis.IT.1 GCA_002109495.1_ASM210949v1
Magnetococcus.marinus.MC.1 GCA_000014865.1_ASM1486v1
Nitrosospira.briensis.C.128 GCA_000619905.2_ASM61990v2
Methylotenera.versatilis.301 GCA_000093025.1_ASM9302v1
Ralstonia.pickettii GCA_902374465.1.MGYG.HGUT.01384
Burkholderia.cepacia GCA_009586235.1_ASM958623v1
Dasania.marina.DSM.21967 GCA_000373485.1_ASM37348v1
Congregibacter.litoralis.KT71 GCA_000153125.2_ASM15312v2
Enterobacter.cloacae GCA_000770155.1_ASM77015v1
Alteromonas.macleodii.ATCC.27126 GCA_000172635.2_ASM17263v2
0.555047

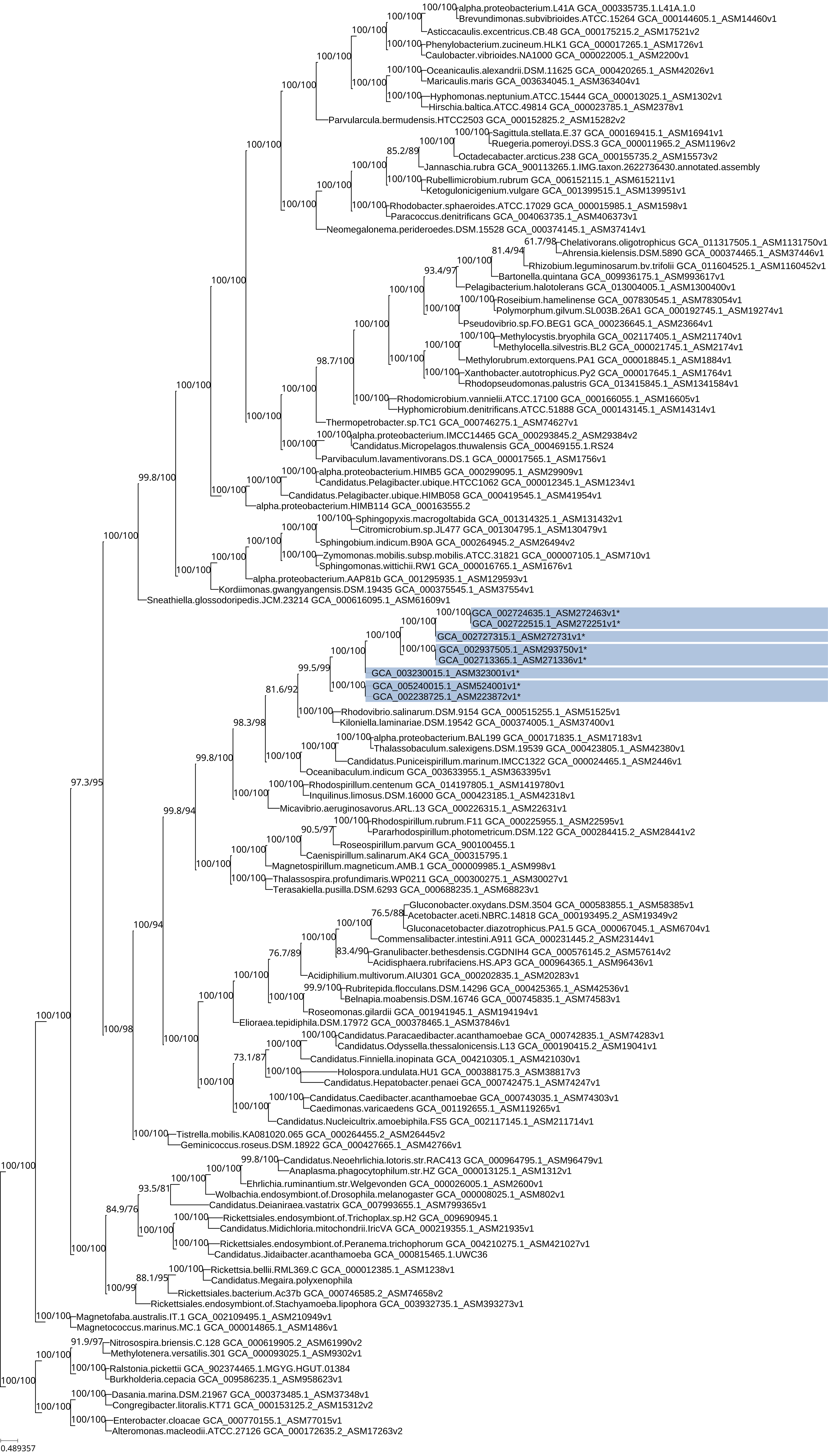

alpha.proteobacterium.L41A GCA_000335735.1.L41A.1.0
Brevundimonas.subvibrioides.ATCC.15264 GCA_000144605.1_ASM14460v1
Asticcacaulis.excentricus.CB.48 GCA_000175215.2_ASM17521v2
Phenylobacterium.zucineum.HLK1 GCA_000017265.1_ASM1726v1
Caulobacter.vibrioides.NA1000 GCA_000022005.1_ASM2200v1
Oceanicaulis.alexandrii.DSM.11625 GCA_000420265.1_ASM42026v1
Maricaulis.maris GCA_003634045.1_ASM363404v1
Hyphomonas.neptunium.ATCC.15444 GCA_000013025.1_ASM1302v1
Hirschia.baltica.ATCC.49814 GCA_000023785.1_ASM2378v1
Parvularcula.bermudensis.HTCC2503 GCA_000152825.2_ASM15282v2
Sagittula.stellata.E.37 GCA_000169415.1_ASM16941v1
Ruegeria.pomeroyi.DSS.3 GCA_000011965.2_ASM1196v2
Octadecabacter.arcticus.238 GCA_000155735.2_ASM15573v2
Jannaschia.rubra GCA_900113265.1.IMG.taxon.2622736430.annotated.assembly
Rubellimicrobium.rubrum GCA_006152115.1_ASM615211v1
Ketogulonicigenium.vulgare GCA_001399515.1_ASM139951v1
Rhodobacter.sphaeroides.ATCC.17029 GCA_000015985.1_ASM1598v1
Paracoccus.denitrificans GCA_004063735.1_ASM406373v1
Neomegalonema.perideroedes.DSM.15528 GCA_000374145.1_ASM37414v1
Chelativorans.oligotrophicus GCA_011317505.1_ASM1131750v1
Ahrensia.kielensis.DSM.5890 GCA_000374465.1_ASM37446v1
Rhizobium.leguminosarum.bv.trifolii GCA_011604525.1_ASM1160452v1
Bartonella.quintana GCA_009936175.1_ASM993617v1
Pelagibacterium.halotolerans GCA_013004005.1_ASM1300400v1
Roseibium.hamelinense GCA_007830545.1_ASM783054v1
Polymorphum.gilvum.SL003B.26A1 GCA_000192745.1_ASM19274v1
Pseudovibrio.sp.FO.BEG1 GCA_000236645.1_ASM23664v1
Methylocystis.bryophila GCA_002117405.1_ASM211740v1
Methylocella.silvestris.BL2 GCA_000021745.1_ASM2174v1
Methylorubrum.extorquens.PA1 GCA_000018845.1_ASM1884v1
Xanthobacter.autotrophicus.Py2 GCA_000017645.1_ASM1764v1
Rhodopseudomonas.palustris GCA_013415845.1_ASM1341584v1
Rhodomicrobium.vannielii.ATCC.17100 GCA_000166055.1_ASM16605v1
Hyphomicrobium.denitrificans.ATCC.51888 GCA_000143145.1_ASM14314v1
Thermopetrobacter.sp.TC1 GCA_000746275.1_ASM74627v1
alpha.proteobacterium.IMCC14465 GCA_000293845.2_ASM29384v2
Candidatus.Micropelagos.thuwalensis GCA_000469155.1.RS24
Parvibaculum.lavamentivorans.DS.1 GCA_000017565.1_ASM1756v1
alpha.proteobacterium.HIMB5 GCA_000299095.1_ASM29909v1
Candidatus.Pelagibacter.ubique.HTCC1062 GCA_000012345.1_ASM1234v1
Candidatus.Pelagibacter.ubique.HIMB058 GCA_000419545.1_ASM41954v1
alpha.proteobacterium.HIMB114 GCA_000163555.2
Sphingopyxis.macrogoltabida GCA_001314325.1_ASM131432v1
Citromicrobium.sp.JL477 GCA_001304795.1_ASM130479v1
Sphingobium.indicum.B90A GCA_000264945.2_ASM26494v2
Zymomonas.mobilis.subsp.mobilis.ATCC.31821 GCA_000007105.1_ASM710v1
Sphingomonas.wittichii.RW1 GCA_000016765.1_ASM1676v1
alpha.proteobacterium.AAP81b GCA_001295935.1_ASM129593v1
Kordiimonas.gwangyangensis.DSM.19435 GCA_000375545.1_ASM37554v1
Sneathiella.glossodoripedis.JCM.23214 GCA_000616095.1_ASM61609v1
GCA_002724635.1_ASM272463v1*
GCA_002722515.1_ASM272251v1*
GCA_002727315.1_ASM272731v1*
GCA_002937505.1_ASM293750v1*
GCA_002713365.1_ASM271336v1*
GCA_003230015.1_ASM323001v1*
GCA_005240015.1_ASM524001v1*
GCA_002238725.1_ASM223872v1*
Rhodovibrio.salinarum.DSM.9154 GCA_000515255.1_ASM51525v1
Kiloniella.laminariae.DSM.19542 GCA_000374005.1_ASM37400v1
alpha.proteobacterium.BAL199 GCA_000171835.1_ASM17183v1
Thalassobaculum.salexigens.DSM.19539 GCA_000423805.1_ASM42380v1
Candidatus.Puniceispirillum.marinum.IMCC1322 GCA_000024465.1_ASM2446v1
Oceanibaculum.indicum GCA_003633955.1_ASM363395v1
Rhodospirillum.centenum GCA_014197805.1_ASM1419780v1
Inquilinus.limosus.DSM.16000 GCA_000423185.1_ASM42318v1
Micavibrio.aeruginosavorus.ARL.13 GCA_000226315.1_ASM22631v1
Rhodospirillum.rubrum.F11 GCA_000225955.1_ASM22595v1
Pararhodospirillum.photometricum.DSM.122 GCA_000284415.2_ASM28441v2
Roseospirillum.parvum GCA_900100455.1
Caenispirillum.salinarum.AK4 GCA_000315795.1
Magnetospirillum.magneticum.AMB.1 GCA_000009985.1_ASM998v1
Thalassospira.profundimaris.WP0211 GCA_000300275.1_ASM30027v1
Terasakiella.pusilla.DSM.6293 GCA_000688235.1_ASM68823v1
Gluconobacter.oxydans.DSM.3504 GCA_000583855.1_ASM58385v1
Acetobacter.aceti.NBRC.14818 GCA_000193495.2_ASM19349v2
Gluconacetobacter.diazotrophicus.PA1.5 GCA_000067045.1_ASM6704v1
Commensalibacter.intestini.A911 GCA_000231445.2_ASM23144v1
Granulibacter.bethesdensis.CGDNIH4 GCA_000576145.2_ASM57614v2
Acidisphaera.rubrifaciens.HS.AP3 GCA_000964365.1_ASM96436v1
Acidiphilium.multivorum.AIU301 GCA_000202835.1_ASM20283v1
Rubritepida.flocculans.DSM.14296 GCA_000425365.1_ASM42536v1
Belnapia.moabensis.DSM.16746 GCA_000745835.1_ASM74583v1
Roseomonas.gilardii GCA_001941945.1_ASM194194v1
Elioraea.tepidiphila.DSM.17972 GCA_000378465.1_ASM37846v1
Candidatus.Paracaedibacter.acanthamoebae GCA_000742835.1_ASM74283v1
Candidatus.Odyssella.thessalonicensis.L13 GCA_000190415.2_ASM19041v1
Candidatus.Finniella.inopinata GCA_004210305.1_ASM421030v1
Holospora.undulata.HU1 GCA_000388175.3_ASM38817v3
Candidatus.Hepatobacter.penaei GCA_000742475.1_ASM74247v1
Candidatus.Caedibacter.acanthamoebae GCA_000743035.1_ASM74303v1
Caedimonas.varicaedens GCA_001192655.1_ASM119265v1
Candidatus.Nucleicultrix.amoebiphila.FS5 GCA_002117145.1_ASM211714v1
Tistrella.mobilis.KA081020.065 GCA_000264455.2_ASM26445v2
Geminicoccus.roseus.DSM.18922 GCA_000427665.1_ASM42766v1
Candidatus.Neoehrlichia.lotoris.str.RAC413 GCA_000964795.1_ASM96479v1
Anaplasma.phagocytophilum.str.HZ GCA_000013125.1_ASM1312v1
Ehrlichia.ruminantium.str.Welgevonden GCA_000026005.1_ASM2600v1
Wolbachia.endosymbiont.of.Drosophila.melanogaster GCA_000008025.1_ASM802v1
Candidatus.Deianiraea.vastatrix GCA_007993655.1_ASM799365v1
Rickettsiales.endosymbiont.of.Trichoplax.sp.H2 GCA_009690945.1
Candidatus.Midichloria.mitochondrii.IricVA GCA_000219355.1_ASM21935v1
Rickettsiales.endosymbiont.of.Peranema.trichophorum GCA_004210275.1_ASM421027v1
Candidatus.Jidaibacter.acanthamoeba GCA_000815465.1.UWC36
Rickettsia.bellii.RML369.C GCA_000012385.1_ASM1238v1
Candidatus.Megaira.polyxenophila
Rickettsiales.bacterium.Ac37b GCA_000746585.2_ASM74658v2
Rickettsiales.endosymbiont.of.Stachyamoeba.lipophora GCA_003932735.1_ASM393273v1
Magnetofaba.australis.IT.1 GCA_002109495.1_ASM210949v1
Magnetococcus.marinus.MC.1 GCA_000014865.1_ASM1486v1
Nitrosospira.briensis.C.128 GCA_000619905.2_ASM61990v2
Methylotenera.versatilis.301 GCA_000093025.1_ASM9302v1
Ralstonia.pickettii GCA_902374465.1.MGYG.HGUT.01384
Burkholderia.cepacia GCA_009586235.1_ASM958623v1
Dasania.marina.DSM.21967 GCA_000373485.1_ASM37348v1
Congregibacter.litoralis.KT71 GCA_000153125.2_ASM15312v2
Enterobacter.cloacae GCA_000770155.1_ASM77015v1
Alteromonas.macleodii.ATCC.27126 GCA_000172635.2_ASM17263v2
0.489357

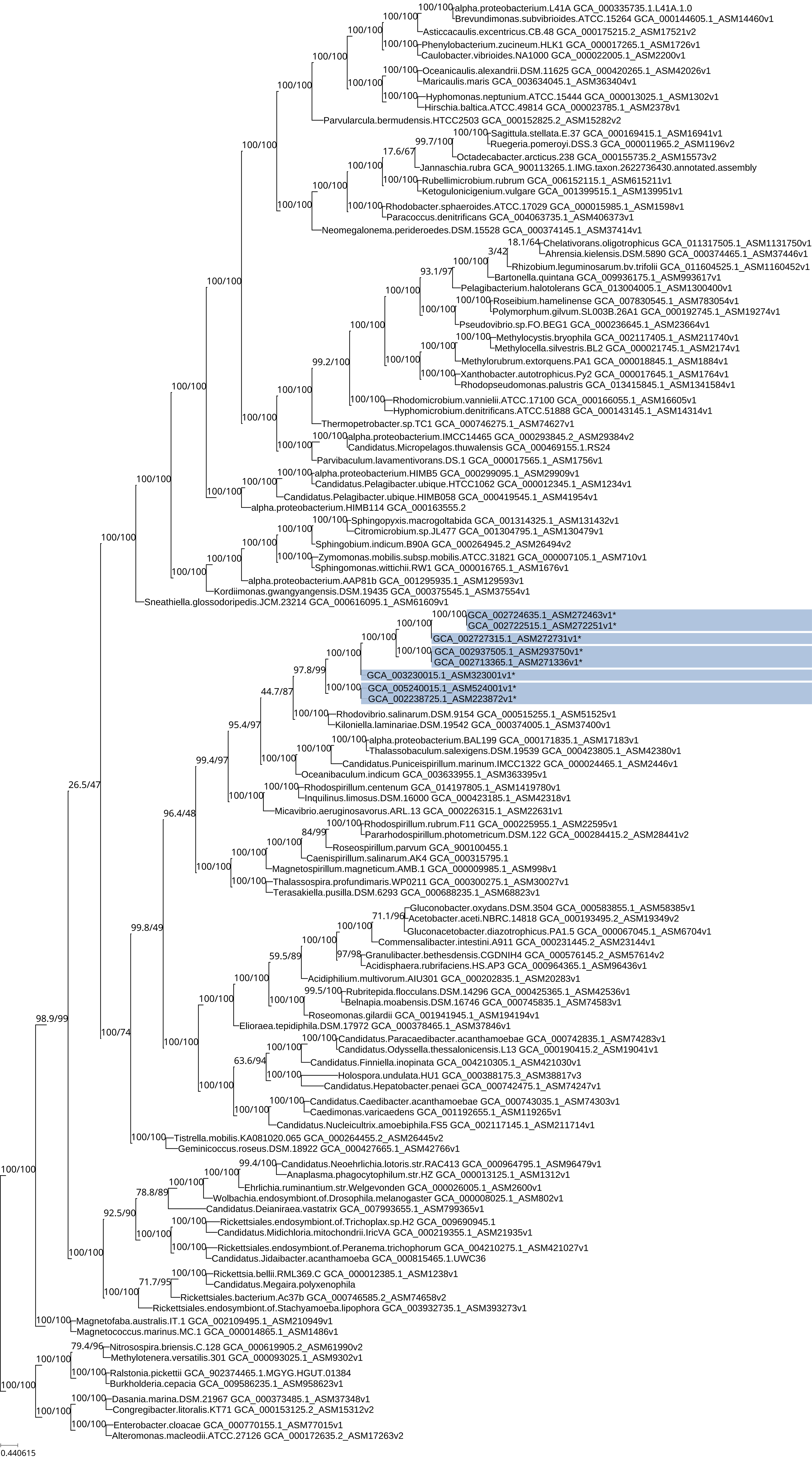
100/100
alpha.proteobacterium.L41A GCA_000335735.1.L41A.1.0
100/100
Brevundimonas.subvibrioides.ATCC.15264 GCA_000144605.1_ASM14460v1
100/100
Asticcacaulis.excentricus.CB.48 GCA_000175215.2_ASM17521v2
100/100
Phenylobacterium.zucineum.HLK1 GCA_000017265.1_ASM1726v1
100/100
Caulobacter.vibrioides.NA1000 GCA_000022005.1_ASM2200v1
100/100
Oceanicaulis.alexandrii.DSM.11625 GCA_000420265.1_ASM42026v1
Maricaulis.maris GCA_003634045.1_ASM363404v1
100/100
100/100
100/100
Hyphomonas.neptunium.ATCC.15444 GCA_000013025.1_ASM1302v1
Hirschia.baltica.ATCC.49814 GCA_000023785.1_ASM2378v1
Parvularcula.bermudensis.HTCC2503 GCA_000152825.2_ASM15282v2
100/100
Sagittula.stellata.E.37 GCA_000169415.1_ASM16941v1
99.7/100
Ruegeria.pomeroyi.DSS.3 GCA_000011965.2_ASM1196v2
100/100
17.6/67
Octadecabacter.arcticus.238 GCA_000155735.2_ASM15573v2
100/100
Jannaschia.rubra GCA_900113265.1.IMG.taxon.2622736430.annotated.assembly
100/100
Rubellimicrobium.rubrum GCA_006152115.1_ASM615211v1
100/100
Ketogulonicigenium.vulgare GCA_001399515.1_ASM139951v1
100/100
100/100
Rhodobacter.sphaeroides.ATCC.17029 GCA_000015985.1_ASM1598v1
Paracoccus.denitrificans GCA_004063735.1_ASM406373v1
Neomegalonema.perideroedes.DSM.15528 GCA_000374145.1_ASM37414v1
18.1/64
Chelativorans.oligotrophicus GCA_011317505.1_ASM1131750v1
3/42
Ahrensia.kielensis.DSM.5890 GCA_000374465.1_ASM37446v1
100/100
Rhizobium.leguminosarum.bv.trifolii GCA_011604525.1_ASM1160452v1
93.1/97
Bartonella.quintana GCA_009936175.1_ASM993617v1
100/100
Pelagibacterium.halotolerans GCA_013004005.1_ASM1300400v1
100/100
100/100
Roseibium.hamelinense GCA_007830545.1_ASM783054v1
100/100
Polymorphum.gilvum.SL003B.26A1 GCA_000192745.1_ASM19274v1
100/100
Pseudovibrio.sp.FO.BEG1 GCA_000236645.1_ASM23664v1
100/100
Methylocystis.bryophila GCA_002117405.1_ASM211740v1
100/100
Methylocella.silvestris.BL2 GCA_000021745.1_ASM2174v1
100/100
Methylorubrum.extorquens.PA1 GCA_000018845.1_ASM1884v1
99.2/100
100/100
Xanthobacter.autotrophicus.Py2 GCA_000017645.1_ASM1764v1
Rhodopseudomonas.palustris GCA_013415845.1_ASM1341584v1
100/100
100/100
100/100
Rhodomicrobium.vannielii.ATCC.17100 GCA_000166055.1_ASM16605v1
Hyphomicrobium.denitrificans.ATCC.51888 GCA_000143145.1_ASM14314v1
100/100
Thermopetrobacter.sp.TC1 GCA_000746275.1_ASM74627v1
100/100
alpha.proteobacterium.IMCC14465 GCA_000293845.2_ASM29384v2
100/100
Candidatus.Micropelagos.thuwalensis GCA_000469155.1.RS24
Parvibaculum.lavamentivorans.DS.1 GCA_000017565.1_ASM1756v1
100/100
alpha.proteobacterium.HIMB5 GCA_000299095.1_ASM29909v1
100/100
100/100
Candidatus.Pelagibacter.ubique.HTCC1062 GCA_000012345.1_ASM1234v1
100/100
Candidatus.Pelagibacter.ubique.HIMB058 GCA_000419545.1_ASM41954v1
alpha.proteobacterium.HIMB114 GCA_000163555.2
100/100
Sphingopyxis.macrogoltabida GCA_001314325.1_ASM131432v1
100/100
Citromicrobium.sp.JL477 GCA_001304795.1_ASM130479v1
100/100
100/100
Sphingobium.indicum.B90A GCA_000264945.2_ASM26494v2
100/100
Zymomonas.mobilis.subsp.mobilis.ATCC.31821 GCA_000007105.1_ASM710v1
100/100
Sphingomonas.wittichii.RW1 GCA_000016765.1_ASM1676v1
100/100
alpha.proteobacterium.AAP81b GCA_001295935.1_ASM129593v1
Kordiimonas.gwangyangensis.DSM.19435 GCA_000375545.1_ASM37554v1
Sneathiella.glossodoripedis.JCM.23214 GCA_000616095.1_ASM61609v1
100/100
GCA_002724635.1_ASM272463v1*
100/100
GCA_002722515.1_ASM272251v1*
100/100
GCA_002727315.1_ASM272731v1*
100/100
GCA_002937505.1_ASM293750v1*
100/100
GCA_002713365.1_ASM271336v1*
97.8/99
GCA_003230015.1_ASM323001v1*
100/100
GCA_005240015.1_ASM524001v1*
44.7/87
GCA_002238725.1_ASM223872v1*
100/100
Rhodovibrio.salinarum.DSM.9154 GCA_000515255.1_ASM51525v1
95.4/97
Kiloniella.laminariae.DSM.19542 GCA_000374005.1_ASM37400v1
100/100
alpha.proteobacterium.BAL199 GCA_000171835.1_ASM17183v1
100/100
Thalassobaculum.salexigens.DSM.19539 GCA_000423805.1_ASM42380v1
100/100
99.4/97
Candidatus.Puniceispirillum.marinum.IMCC1322 GCA_000024465.1_ASM2446v1
Oceanibaculum.indicum GCA_003633955.1_ASM363395v1
26.5/47
100/100
Rhodospirillum.centenum GCA_014197805.1_ASM1419780v1
100/100
Inquilinus.limosus.DSM.16000 GCA_000423185.1_ASM42318v1
Micavibrio.aeruginosavorus.ARL.13 GCA_000226315.1_ASM22631v1
96.4/48
100/100
Rhodospirillum.rubrum.F11 GCA_000225955.1_ASM22595v1
84/99
Pararhodospirillum.photometricum.DSM.122 GCA_000284415.2_ASM28441v2
100/100
Roseospirillum.parvum GCA_900100455.1
100/100
Caenispirillum.salinarum.AK4 GCA_000315795.1
100/100
Magnetospirillum.magneticum.AMB.1 GCA_000009985.1_ASM998v1
100/100
Thalassospira.profundimaris.WP0211 GCA_000300275.1_ASM30027v1
Terasakiella.pusilla.DSM.6293 GCA_000688235.1_ASM68823v1
Gluconobacter.oxydans.DSM.3504 GCA_000583855.1_ASM58385v1
71.1/96
Acetobacter.aceti.NBRC.14818 GCA_000193495.2_ASM19349v2
100/100
99.8/49
Gluconacetobacter.diazotrophicus.PA1.5 GCA_000067045.1_ASM6704v1
100/100
Commensalibacter.intestini.A911 GCA_000231445.2_ASM23144v1
97/98
Granulibacter.bethesdensis.CGDNIH4 GCA_000576145.2_ASM57614v2
59.5/89
Acidisphaera.rubrifaciens.HS.AP3 GCA_000964365.1_ASM96436v1
100/100
Acidiphilium.multivorum.AIU301 GCA_000202835.1_ASM20283v1
99.5/100
Rubritepida.flocculans.DSM.14296 GCA_000425365.1_ASM42536v1
100/100
Belnapia.moabensis.DSM.16746 GCA_000745835.1_ASM74583v1
100/100
Roseomonas.gilardii GCA_001941945.1_ASM194194v1
98.9/99
Elioraea.tepidiphila.DSM.17972 GCA_000378465.1_ASM37846v1
100/74
100/100
Candidatus.Paracaedibacter.acanthamoebae GCA_000742835.1_ASM74283v1
100/100
100/100
Candidatus.Odyssella.thessalonicensis.L13 GCA_000190415.2_ASM19041v1
63.6/94
Candidatus.Finniella.inopinata GCA_004210305.1_ASM421030v1
100/100
Holospora.undulata.HU1 GCA_000388175.3_ASM38817v3
100/100
Candidatus.Hepatobacter.penaei GCA_000742475.1_ASM74247v1
100/100
Candidatus.Caedibacter.acanthamoebae GCA_000743035.1_ASM74303v1
100/100
Caedimonas.varicaedens GCA_001192655.1_ASM119265v1
Candidatus.Nucleicultrix.amoebiphila.FS5 GCA_002117145.1_ASM211714v1
100/100
Tistrella.mobilis.KA081020.065 GCA_000264455.2_ASM26445v2
Geminicoccus.roseus.DSM.18922 GCA_000427665.1_ASM42766v1
99.4/100
Candidatus.Neoehrlichia.lotoris.str.RAC413 GCA_000964795.1_ASM96479v1
100/100
100/100
Anaplasma.phagocytophilum.str.HZ GCA_000013125.1_ASM1312v1
100/100
Ehrlichia.ruminantium.str.Welgevonden GCA_000026005.1_ASM2600v1
78.8/89
Wolbachia.endosymbiont.of.Drosophila.melanogaster GCA_000008025.1_ASM802v1
Candidatus.Deianiraea.vastatrix GCA_007993655.1_ASM799365v1
92.5/90
100/100
Rickettsiales.endosymbiont.of.Trichoplax.sp.H2 GCA_009690945.1
100/100
Candidatus.Midichloria.mitochondrii.IricVA GCA_000219355.1_ASM21935v1
100/100
Rickettsiales.endosymbiont.of.Peranema.trichophorum GCA_004210275.1_ASM421027v1
100/100
Candidatus.Jidaibacter.acanthamoeba GCA_000815465.1.UWC36
100/100
Rickettsia.bellii.RML369.C GCA_000012385.1_ASM1238v1
71.7/95
Candidatus.Megaira.polyxenophila
100/100
Rickettsiales.bacterium.Ac37b GCA_000746585.2_ASM74658v2
Rickettsiales.endosymbiont.of.Stachyamoeba.lipophora GCA_003932735.1_ASM393273v1
100/100
Magnetofaba.australis.IT.1 GCA_002109495.1_ASM210949v1
Magnetococcus.marinus.MC.1 GCA_000014865.1_ASM1486v1
79.4/96
Nitrosospira.briensis.C.128 GCA_000619905.2_ASM61990v2
100/100
Methylotenera.versatilis.301 GCA_000093025.1_ASM9302v1
100/100
Ralstonia.pickettii GCA_902374465.1.MGYG.HGUT.01384
Burkholderia.cepacia GCA_009566235.1_ASM958623v1
100/100
100/100
Dasania.marina.DSM.21967 GCA_000373485.1_ASM37348v1
100/100
Congregibacter.litoralis.KT71 GCA_000153125.2_ASM15312v2
100/100
Enterobacter.cloacae GCA_000770155.1_ASM77015v1
Alteromonas.macleodii.ATCC.27126 GCA_000172635.2_ASM17263v2
0.440615

Group B

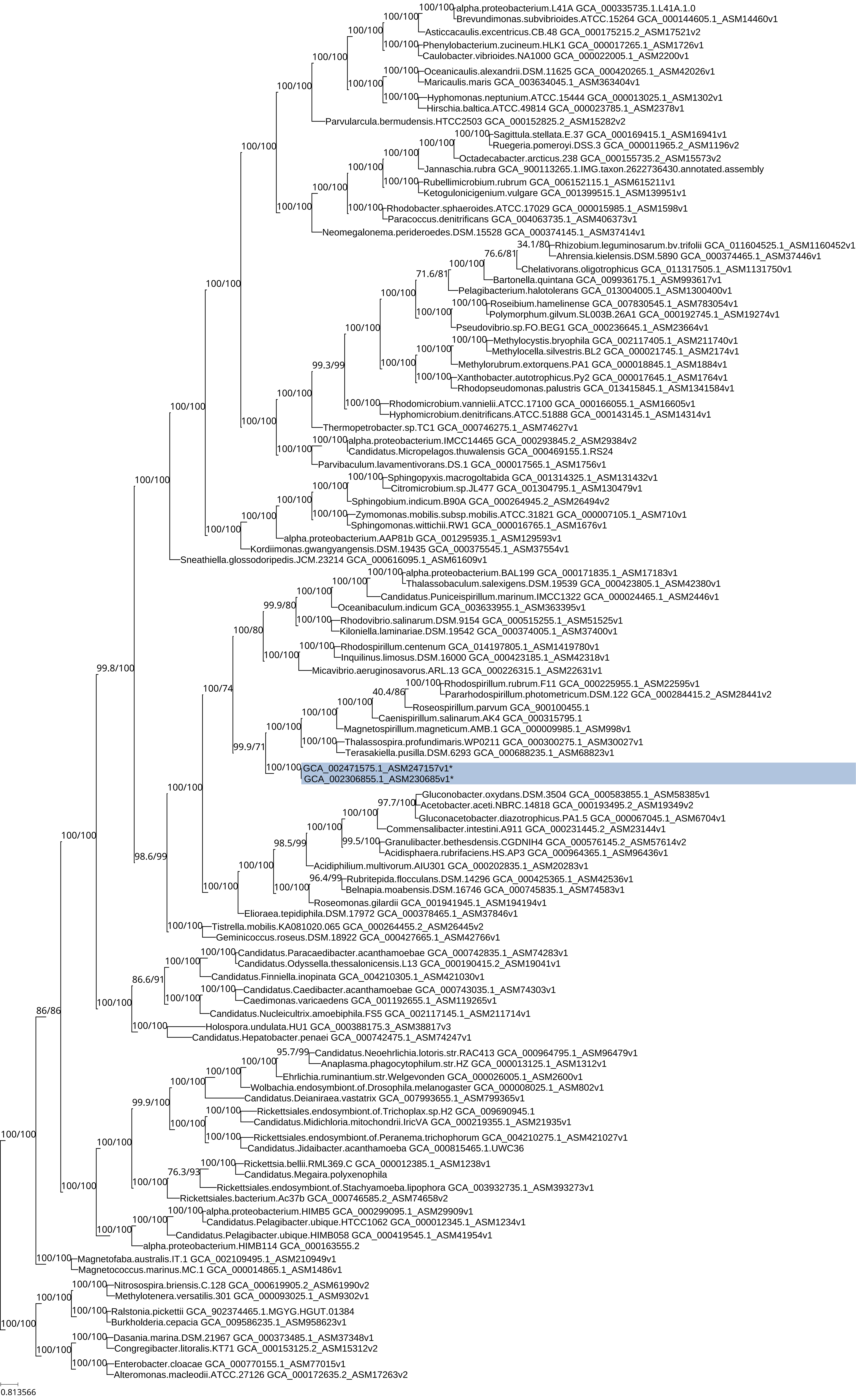
alpha.proteobacterium.L41A GCA_000335735.1.L41A.1.0
Brevundimonas.subvibrioides.ATCC.15264 GCA_000144605.1_ASM14460v1
Asticcacaulis.excentricus.CB.48 GCA_000175215.2_ASM17521v2
Phenylobacterium.zucineum.HLK1 GCA_000017265.1_ASM1726v1
Caulobacter.vibrioides.NA1000 GCA_000022005.1_ASM2200v1
Oceanicaulis.alexandrii.DSM.11625 GCA_000420265.1_ASM42026v1
Maricaulis.maris GCA_003634045.1_ASM363404v1
Hyphomonas.neptunium.ATCC.15444 GCA_000013025.1_ASM1302v1
Hirschia.baltica.ATCC.49814 GCA_000023785.1_ASM2378v1
Parvularcula.bermudensis.HTCC2503 GCA_000152825.2_ASM15282v2
Sagittula.stellata.E.37 GCA_000169415.1_ASM16941v1
Ruegeria.pomeroyi.DSS.3 GCA_000011965.2_ASM1196v2
Octadecabacter.arcticus.238 GCA_000155735.2_ASM15573v2
Jannaschia.rubra GCA_900113265.1.IMG.taxon.2622736430.annotated.assembly
Rubellimicrobium.rubrum GCA_006152115.1_ASM615211v1
Ketogulonicigenium.vulgare GCA_001399515.1_ASM139951v1
Rhodobacter.sphaeroides.ATCC.17029 GCA_000015985.1_ASM1598v1
Paracoccus.denitrificans GCA_004063735.1_ASM406373v1
Neomegalonema.perideroedes.DSM.15528 GCA_000374145.1_ASM37414v1
Rhizobium.leguminosarum.bv.trifolii GCA_011604525.1_ASM1160452v1
Ahrensia.kielensis.DSM.5890 GCA_000374465.1_ASM37446v1
Chelativorans.oligotrophicus GCA_011317505.1_ASM1131750v1
Bartonella.quintana GCA_009936175.1_ASM993617v1
Pelagibacterium.halotolerans GCA_013004005.1_ASM1300400v1
Roseibium.hamelinense GCA_007830545.1_ASM783054v1
Polymorphum.gilvum.SL003B.26A1 GCA_000192745.1_ASM19274v1
Pseudovibrio.sp.FO.BEG1 GCA_000236645.1_ASM23664v1
Methylocystis.bryophila GCA_002117405.1_ASM211740v1
Methylocella.silvestris.BL2 GCA_000021745.1_ASM2174v1
Methylorubrum.extorquens.PA1 GCA_000018845.1_ASM1884v1
Xanthobacter.autotrophicus.Py2 GCA_000017645.1_ASM1764v1
Rhodopseudomonas.palustris GCA_013415845.1_ASM1341584v1
Rhodomicrobium.vannielii.ATCC.17100 GCA_000166055.1_ASM16605v1
Hyphomicrobium.denitrificans.ATCC.51888 GCA_000143145.1_ASM14314v1
Thermopetrobacter.sp.TC1 GCA_000746275.1_ASM74627v1
alpha.proteobacterium.IMCC14465 GCA_000293845.2_ASM29384v2
Candidatus.Micropelagos.thuwalensis GCA_000469155.1.RS24
Parvibaculum.lavamentivorans.DS.1 GCA_000017565.1_ASM1756v1
Sphingopyxis.macrogoltabida GCA_001314325.1_ASM131432v1
Citromicrobium.sp.JL477 GCA_001304795.1_ASM130479v1
Sphingobium.indicum.B90A GCA_000264945.2_ASM26494v2
Zymomonas.mobilis.subsp.mobilis.ATCC.31821 GCA_000007105.1_ASM710v1
Sphingomonas.wittichii.RW1 GCA_000016765.1_ASM1676v1
alpha.proteobacterium.AAP81b GCA_001295935.1_ASM129593v1
Kordiimonas.gwangyangensis.DSM.19435 GCA_000375545.1_ASM37554v1
Sneathiella.glossodoripedis.JCM.23214 GCA_000616095.1_ASM61609v1
alpha.proteobacterium.BAL199 GCA_000171835.1_ASM17183v1
Thalassobaculum.salexigens.DSM.19539 GCA_000423805.1_ASM42380v1
Candidatus.Puniceispirillum.marinum.IMCC1322 GCA_000024465.1_ASM2446v1
Oceanibaculum.indicum GCA_003633955.1_ASM363395v1
Rhodovibrio.salinarum.DSM.9154 GCA_000515255.1_ASM51525v1
Kiloniella.laminariae.DSM.19542 GCA_000374005.1_ASM37400v1
Rhodospirillum.centenum GCA_014197805.1_ASM1419780v1
Inquilinus.limosus.DSM.16000 GCA_000423185.1_ASM42318v1
Micavibrio.aeruginosavorus.ARL.13 GCA_000226315.1_ASM22631v1
Rhodospirillum.rubrum.F11 GCA_000225955.1_ASM22595v1
Pararhodospirillum.photometricum.DSM.122 GCA_000284415.2_ASM28441v2
Roseospirillum.parvum GCA_900100455.1
Caenispirillum.salinarum.AK4 GCA_000315795.1
Magnetospirillum.magneticum.AMB.1 GCA_000009985.1_ASM998v1
Thalassospira.profundimaris.WP0211 GCA_000300275.1_ASM30027v1
Terasakiella.pusilla.DSM.6293 GCA_000688235.1_ASM68823v1
GCA_002471575.1_ASM247157v1*
GCA_002306855.1_ASM230685v1*
Gluconobacter.oxydans.DSM.3504 GCA_000583855.1_ASM58385v1
Acetobacter.aceti.NBRC.14818 GCA_000193495.2_ASM19349v2
Gluconacetobacter.diazotrophicus.PA1.5 GCA_000067045.1_ASM6704v1
Commensalibacter.intestini.A911 GCA_000231445.2_ASM23144v1
Granulibacter.bethesdensis.CGDNIH4 GCA_000576145.2_ASM57614v2
Acidisphaera.rubrifaciens.HS.AP3 GCA_000964365.1_ASM96436v1
Acidiphilium.multivorum.AIU301 GCA_000202835.1_ASM20283v1
Rubritepida.flocculans.DSM.14296 GCA_000425365.1_ASM42536v1
Belnapia.moabensis.DSM.16746 GCA_000745835.1_ASM74583v1
Roseomonas.gilardii GCA_001941945.1_ASM194194v1
Elioraea.tepidiphila.DSM.17972 GCA_000378465.1_ASM37846v1
Tistrella.mobilis.KA081020.065 GCA_000264455.2_ASM26445v2
Geminicoccus.roseus.DSM.18922 GCA_000427665.1_ASM42766v1
Candidatus.Paracaedibacter.acanthamoebae GCA_000742835.1_ASM74283v1
Candidatus.Odyssella.thessalonicensis.L13 GCA_000190415.2_ASM19041v1
Candidatus.Finniella.inopinata GCA_004210305.1_ASM421030v1
Candidatus.Caedibacter.acanthamoebae GCA_000743035.1_ASM74303v1
Caedimonas.varicaedens GCA_001192655.1_ASM119265v1
Candidatus.Nucleicultrix.amoebiphila.FS5 GCA_002117145.1_ASM211714v1
Holospora.undulata.HU1 GCA_000388175.3_ASM38817v3
Candidatus.Hepatobacter.penaei GCA_000742475.1_ASM74247v1
Candidatus.Neoehrlichia.lotoris.str.RAC413 GCA_000964795.1_ASM96479v1
Anaplasma.phagocytophilum.str.HZ GCA_000013125.1_ASM1312v1
Ehrlichia.ruminantium.str.Welgevonden GCA_000026005.1_ASM2600v1
Wolbachia.endosymbiont.of.Drosophila.melanogaster GCA_000008025.1_ASM802v1
Candidatus.Deianiraea.vastatrix GCA_007993655.1_ASM799365v1
Rickettsiales.endosymbiont.of.Trichoplax.sp.H2 GCA_009690945.1
Candidatus.Midichloria.mitochondrii.IricVA GCA_000219355.1_ASM21935v1
Rickettsiales.endosymbiont.of.Peranema.trichophorum GCA_004210275.1_ASM421027v1
Candidatus.Jidaibacter.acanthamoeba GCA_000815465.1.UWC36
Rickettsia.bellii.RML369.C GCA_000012385.1_ASM1238v1
Candidatus.Megaira.polyxenophila
Rickettsiales.endosymbiont.of.Stachyamoeba.lipophora GCA_003932735.1_ASM393273v1
Rickettsiales.bacterium.Ac37b GCA_000746585.2_ASM74658v2
alpha.proteobacterium.HIMB5 GCA_000299095.1_ASM29909v1
Candidatus.Pelagibacter.ubique.HTCC1062 GCA_000012345.1_ASM1234v1
Candidatus.Pelagibacter.ubique.HIMB058 GCA_000419545.1_ASM41954v1
alpha.proteobacterium.HIMB114 GCA_000163555.2
Magnetofaba.australis.IT.1 GCA_002109495.1_ASM210949v1
Magnetococcus.marinus.MC.1 GCA_000014865.1_ASM1486v1
Nitrosospira.briensis.C.128 GCA_000619905.2_ASM61990v2
Methylotenera.versatilis.301 GCA_000093025.1_ASM9302v1
Ralstonia.pickettii GCA_902374465.1.MGYG.HGUT.01384
Burkholderia.cepacia GCA_009586235.1_ASM958623v1
Dasania.marina.DSM.21967 GCA_000373485.1_ASM37348v1
Congregibacter.litoralis.KT71 GCA_000153125.2_ASM15312v2
Enterobacter.cloacae GCA_000770155.1_ASM77015v1
Alteromonas.macleodii.ATCC.27126 GCA_000172635.2_ASM17263v2
0.813566

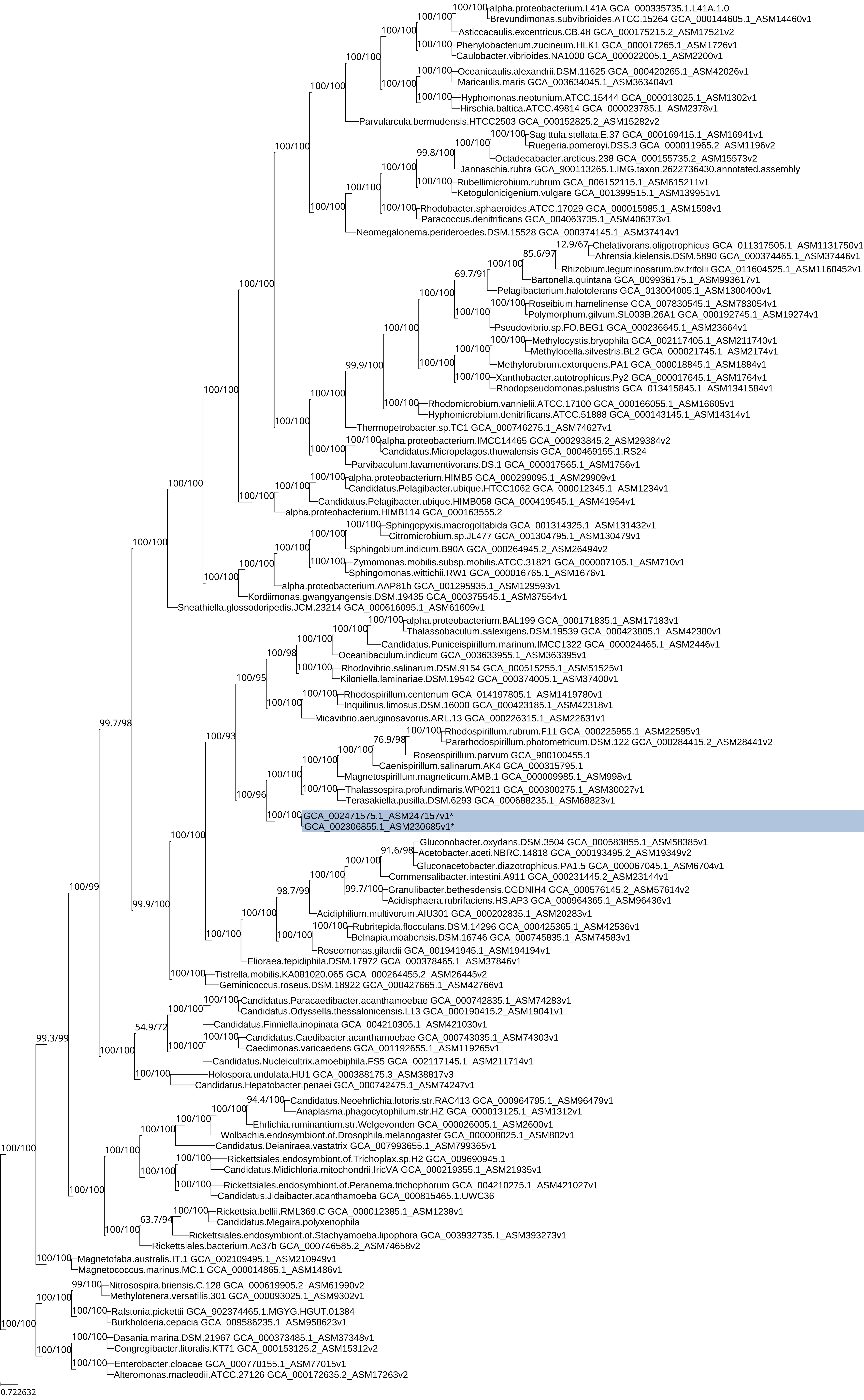
alpha.proteobacterium.L41A GCA_000335735.1_L41A.1.0
Brevundimonas.subvibrioides.ATCC.15264 GCA_000144605.1_ASM14460v1
Asticcacaulis.excentricus.CB.48 GCA_000175215.2_ASM17521v2
Phenylobacterium.zucineum.HLK1 GCA_000017265.1_ASM1726v1
Caulobacter.vibrioides.NA1000 GCA_000022005.1_ASM2200v1
Oceanicaulis.alexandrii.DSM.11625 GCA_000420265.1_ASM42026v1
Maricaulis.maris GCA_003634045.1_ASM363404v1
Hyphomonas.neptunium.ATCC.15444 GCA_000013025.1_ASM1302v1
Hirschia.baltica.ATCC.49814 GCA_000023785.1_ASM2378v1
Parvularcula.bermudensis.HTCC2503 GCA_000152825.2_ASM15282v2
Sagittula.stellata.E.37 GCA_000169415.1_ASM16941v1
Ruegeria.pomeroyi.DSS.3 GCA_000011965.2_ASM1196v2
Octadecabacter.arcticus.238 GCA_000155735.2_ASM15573v2
Jannaschia.rubra GCA_900113265.1.IMG.taxon.2622736430.annotated.assembly
Rubellimicrobium.rubrum GCA_006152115.1_ASM615211v1
Ketogulonicigenium.vulgare GCA_001399515.1_ASM139951v1
Rhodobacter.sphaeroides.ATCC.17029 GCA_000015985.1_ASM1598v1
Paracoccus.denitrificans GCA_004063735.1_ASM406373v1
Neomegalonema.perideroedes.DSM.15528 GCA_000374145.1_ASM37414v1
Chelativorans.oligotrophicus GCA_011317505.1_ASM1131750v1
Ahrensia.kielensis.DSM.5890 GCA_000374465.1_ASM37446v1
Rhizobium.leguminosarum.bv.trifolii GCA_011604525.1_ASM1160452v1
Bartonella.quintana GCA_009936175.1_ASM993617v1
Pelagibacterium.halotolerans GCA_013004005.1_ASM1300400v1
Roseibium.hamelinense GCA_007830545.1_ASM783054v1
Polymorphum.gilvum.SL003B.26A1 GCA_000192745.1_ASM19274v1
Pseudovibrio.sp.FO.BEG1 GCA_000236645.1_ASM23664v1
Methylocystis.bryophila GCA_002117405.1_ASM211740v1
Methylocella.silvestris.BL2 GCA_000021745.1_ASM2174v1
Methylorubrum.extorquens.PA1 GCA_000018845.1_ASM1884v1
Xanthobacter.autotrophicus.Py2 GCA_000017645.1_ASM1764v1
Rhodopseudomonas.palustris GCA_013415845.1_ASM1341584v1
Rhodomicrobium.vannielii.ATCC.17100 GCA_000166055.1_ASM16605v1
Hyphomicrobium.denitrificans.ATCC.51888 GCA_000143145.1_ASM14314v1
Thermopetrobacter.sp.TC1 GCA_000746275.1_ASM74627v1
alpha.proteobacterium.IMCC14465 GCA_000293845.2_ASM29384v2
Candidatus.Micropelagos.thuwalensis GCA_000469155.1.RS24
Parvibaculum.lavamentivorans.DS.1 GCA_000017565.1_ASM1756v1
alpha.proteobacterium.HIMB5 GCA_000299095.1_ASM29909v1
Candidatus.Pelagibacter.ubique.HTCC1062 GCA_000012345.1_ASM1234v1
Candidatus.Pelagibacter.ubique.HIMB058 GCA_000419545.1_ASM41954v1
alpha.proteobacterium.HIMB114 GCA_000163555.2
Sphingopyxis.macrogoltabida GCA_001314325.1_ASM131432v1
Citromicrobium.sp.JL477 GCA_001304795.1_ASM130479v1
Sphingobium.indicum.B90A GCA_000264945.2_ASM26494v2
Zymomonas.mobilis.subsp.mobilis.ATCC.31821 GCA_000007105.1_ASM710v1
Sphingomonas.wittichii.RW1 GCA_000016765.1_ASM1676v1
alpha.proteobacterium.AAP81b GCA_001295935.1_ASM129593v1
Kordiimonas.gwangyangensis.DSM.19435 GCA_000375545.1_ASM37554v1
Sneathiella.glossodoripedis.JCM.23214 GCA_000616095.1_ASM61609v1
alpha.proteobacterium.BAL199 GCA_000171835.1_ASM17183v1
Thalassobaculum.salexigens.DSM.19539 GCA_000423805.1_ASM42380v1
Candidatus.Puniceispirillum.marinum.IMCC1322 GCA_000024465.1_ASM2446v1
Oceanibaculum.indicum GCA_003633955.1_ASM363395v1
Rhodovibrio.salinarum.DSM.9154 GCA_000515255.1_ASM51525v1
Kiloniella.laminariae.DSM.19542 GCA_000374005.1_ASM37400v1
Rhodospirillum.centenum GCA_014197805.1_ASM1419780v1
Inquilinus.limosus.DSM.16000 GCA_000423185.1_ASM42318v1
Micavibrio.aeruginosavorus.ARL.13 GCA_000226315.1_ASM22631v1
Rhodospirillum.rubrum.F11 GCA_000225955.1_ASM22595v1
Pararhodospirillum.photometricum.DSM.122 GCA_000284415.2_ASM28441v2
Roseospirillum.parvum GCA_900100455.1
Caenispirillum.salinarum.AK4 GCA_000315795.1
Magnetospirillum.magneticum.AMB.1 GCA_000009985.1_ASM998v1
Thalassospira.profundimaris.WP0211 GCA_000300275.1_ASM30027v1
Terasakiella.pusilla.DSM.6293 GCA_000688235.1_ASM68823v1
GCA_002471575.1_ASM247157v1*
GCA_002306855.1_ASM230685v1*
Gluconobacter.oxydans.DSM.3504 GCA_000583855.1_ASM58385v1
Acetobacter.aceti.NBRC.14818 GCA_000193495.2_ASM19349v2
Gluconacetobacter.diazotrophicus.PA1.5 GCA_000067045.1_ASM6704v1
Commensalibacter.intestini.A911 GCA_000231445.2_ASM23144v1
Granulibacter.bethesdensis.CGDNIH4 GCA_000576145.2_ASM57614v2
Acidisphaera.rubrifaciens.HS.AP3 GCA_000964365.1_ASM96436v1
Acidiphilium.multivorum.AIU301 GCA_000202835.1_ASM20283v1
Rubritepida.flocculans.DSM.14296 GCA_000425365.1_ASM42536v1
Belnapia.moabensis.DSM.16746 GCA_000745835.1_ASM74583v1
Roseomonas.gilardii GCA_001941945.1_ASM194194v1
Elioraea.tepidiphila.DSM.17972 GCA_000378465.1_ASM37846v1
Tistrella.mobilis.KA081020.065 GCA_000264455.2_ASM26445v2
Geminicoccus.roseus.DSM.18922 GCA_000427665.1_ASM42766v1
Candidatus.Paracaedibacter.acanthamoebae GCA_000742835.1_ASM74283v1
Candidatus.Odyssella.thessalonicensis.L13 GCA_000190415.2_ASM19041v1
Candidatus.Finniella.inopinata GCA_004210305.1_ASM421030v1
Candidatus.Caedibacter.acanthamoebae GCA_000743035.1_ASM74303v1
Caedimonas.varicaedens GCA_001192655.1_ASM119265v1
Candidatus.Nucleicultrix.amoebiphila.FS5 GCA_002117145.1_ASM211714v1
Holospora.undulata.HU1 GCA_000388175.3_ASM38817v3
Candidatus.Hepatobacter.penaei GCA_000742475.1_ASM74247v1
Candidatus.Neoehrlichia.lotoris.str.RAC413 GCA_000964795.1_ASM96479v1
Anaplasma.phagocytophilum.str.HZ GCA_000013125.1_ASM1312v1
Ehrlichia.ruminantium.str.Welgevonden GCA_000026005.1_ASM2600v1
Wolbachia.endosymbiont.of.Drosophila.melanogaster GCA_000008025.1_ASM802v1
Candidatus.Deianiraea.vastatrix GCA_007993655.1_ASM799365v1
Rickettsiales.endosymbiont.of.Trichoplax.sp.H2 GCA_009690945.1
Candidatus.Midichloria.mitochondrii.IricVA GCA_000219355.1_ASM21935v1
Rickettsiales.endosymbiont.of.Peranema.trichophorum GCA_004210275.1_ASM421027v1
Candidatus.Jidaibacter.acanthamoeba GCA_000815465.1.UWC36
Rickettsia.bellii.RML369.C GCA_000012385.1_ASM1238v1
Candidatus.Megaira.polyxenophila
Rickettsiales.endosymbiont.of.Stachyamoeba.lipophora GCA_003932735.1_ASM393273v1
Rickettsiales.bacterium.Ac37b GCA_000746585.2_ASM74658v2
Magnetofaba.australis.IT.1 GCA_002109495.1_ASM210949v1
Magnetococcus.marinus.MC.1 GCA_000014865.1_ASM1486v1
Nitrosospira.briensis.C.128 GCA_000619905.2_ASM61990v2
Methylotenera.versatilis.301 GCA_000093025.1_ASM9302v1
Ralstonia.pickettii GCA_902374465.1.MGYG.HGUT.01384
Burkholderia.cepacia GCA_009586235.1_ASM958623v1
Dasania.marina.DSM.21967 GCA_000373485.1_ASM37348v1
Congregibacter.litoralis.KT71 GCA_000153125.2_ASM15312v2
Enterobacter.cloacae GCA_000770155.1_ASM77015v1
Alteromonas.macleodii.ATCC.27126 GCA_000172635.2_ASM17263v2
0.722632

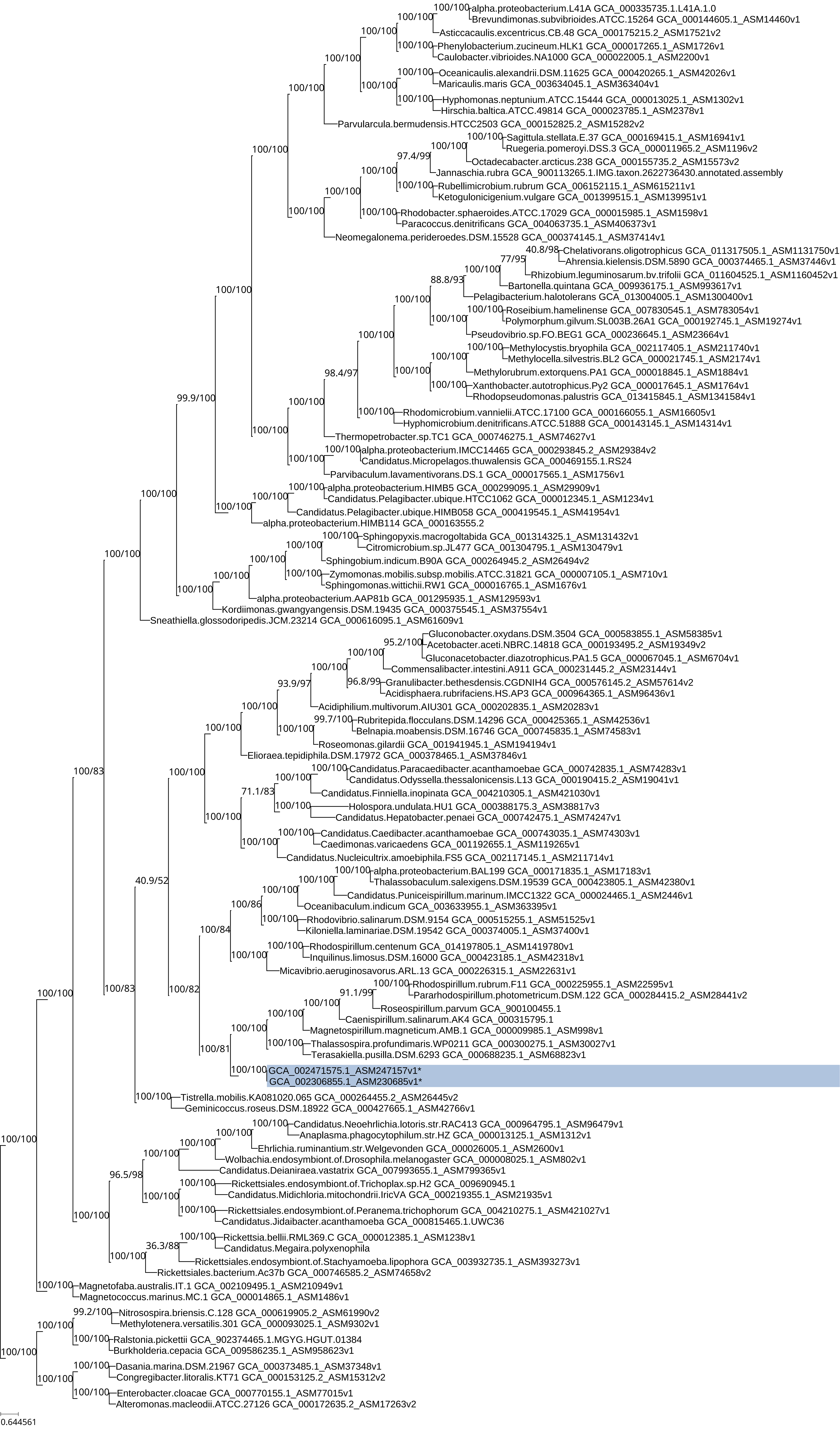

alpha.proteobacterium.L41A GCA_000335735.1.L41A.1.0
Brevundimonas.subvibrioides.ATCC.15264 GCA_000144605.1_ASM14460v1
Asticcacaulis.excentricus.CB.48 GCA_000175215.2_ASM17521v2
Phenylobacterium.zucineum.HLK1 GCA_000017265.1_ASM1726v1
Caulobacter.vibrioides.NA1000 GCA_000022005.1_ASM2200v1
Oceanicaulis.alexandrii.DSM.11625 GCA_000420265.1_ASM42026v1
Maricaulis.maris GCA_003634045.1_ASM363404v1
Hyphomonas.neptunium.ATCC.15444 GCA_000013025.1_ASM1302v1
Hirschia.baltica.ATCC.49814 GCA_000023785.1_ASM2378v1
Parvularcula.bermudensis.HTCC2503 GCA_000152825.2_ASM15282v2
Sagittula.stellata.E.37 GCA_000169415.1_ASM16941v1
Ruegeria.pomeroyi.DSS.3 GCA_000011965.2_ASM1196v2
Octadecabacter.arcticus.238 GCA_000155735.2_ASM15573v2
Jannaschia.rubra GCA_900113265.1.IMG.taxon.2622736430.annotated.assembly
Rubellimicrobium.rubrum GCA_006152115.1_ASM615211v1
Ketogulonicigenium.vulgare GCA_001399515.1_ASM139951v1
Rhodobacter.sphaeroides.ATCC.17029 GCA_000015985.1_ASM1598v1
Paracoccus.denitrificans GCA_004063735.1_ASM406373v1
Neomegalonema.perideroedes.DSM.15528 GCA_000374145.1_ASM37414v1
Chelativorans.oligotrophicus GCA_011317505.1_ASM1131750v1
Ahrensia.kielensis.DSM.5890 GCA_000374465.1_ASM37446v1
Rhizobium.leguminosarum.bv.trifolii GCA_011604525.1_ASM1160452v1
Bartonella.quintana GCA_009936175.1_ASM993617v1
Pelagibacterium.halotolerans GCA_013004005.1_ASM1300400v1
Roseibium.hamelinense GCA_007830545.1_ASM783054v1
Polymorphum.gilvum.SL003B.26A1 GCA_000192745.1_ASM19274v1
Pseudovibrio.sp.FO.BEG1 GCA_000236645.1_ASM23664v1
Methylocystis.bryophila GCA_002117405.1_ASM211740v1
Methylocella.silvestris.BL2 GCA_000021745.1_ASM2174v1
Methylorubrum.extorquens.PA1 GCA_000018845.1_ASM1884v1
Xanthobacter.autotrophicus.Py2 GCA_000017645.1_ASM1764v1
Rhodopseudomonas.palustris GCA_013415845.1_ASM1341584v1
Rhodomicrobium.vannielii.ATCC.17100 GCA_000166055.1_ASM16605v1
Hyphomicrobium.denitrificans.ATCC.51888 GCA_000143145.1_ASM14314v1
Thermopetrobacter.sp.TC1 GCA_000746275.1_ASM74627v1
alpha.proteobacterium.IMCC14465 GCA_000293845.2_ASM29384v2
Candidatus.Micropelagos.thuwalensis GCA_000469155.1.RS24
Parvibaculum.lavamentivorans.DS.1 GCA_000017565.1_ASM1756v1
alpha.proteobacterium.HIMB5 GCA_000299095.1_ASM29909v1
Candidatus.Pelagibacter.ubique.HTCC1062 GCA_000012345.1_ASM1234v1
Candidatus.Pelagibacter.ubique.HIMB058 GCA_000419545.1_ASM41954v1
alpha.proteobacterium.HIMB114 GCA_000163555.2
Sphingopyxis.macrogoltabida GCA_001314325.1_ASM131432v1
Citromicrobium.sp.JL477 GCA_001304795.1_ASM130479v1
Sphingobium.indicum.B90A GCA_000264945.2_ASM26494v2
Zymomonas.mobilis.subsp.mobilis.ATCC.31821 GCA_000007105.1_ASM710v1
Sphingomonas.wittichii.RW1 GCA_000016765.1_ASM1676v1
alpha.proteobacterium.AAP81b GCA_001295935.1_ASM129593v1
Kordiimonas.gwangyangensis.DSM.19435 GCA_000375545.1_ASM37554v1
Sneathiella.glossodoripedis.JCM.23214 GCA_000616095.1_ASM61609v1
Gluconobacter.oxydans.DSM.3504 GCA_000583855.1_ASM58385v1
Acetobacter.aceti.NBRC.14818 GCA_000193495.2_ASM19349v2
Gluconacetobacter.diazotrophicus.PA1.5 GCA_000067045.1_ASM6704v1
Commensalibacter.intestini.A911 GCA_000231445.2_ASM23144v1
Granulibacter.bethesdensis.CGDNIH4 GCA_000576145.2_ASM57614v2
Acidisphaera.rubrifaciens.HS.AP3 GCA_000964365.1_ASM96436v1
Acidiphilium.multivorum.AIU301 GCA_000202835.1_ASM20283v1
Rubritepida.flocculans.DSM.14296 GCA_000425365.1_ASM42536v1
Belnapia.moabensis.DSM.16746 GCA_000745835.1_ASM74583v1
Roseomonas.gilardii GCA_001941945.1_ASM194194v1
Elioraea.tepidiphila.DSM.17972 GCA_000378465.1_ASM37846v1
Candidatus.Paracaedibacter.acanthamoebae GCA_000742835.1_ASM74283v1
Candidatus.Odyssella.thessalonicensis.L13 GCA_000190415.2_ASM19041v1
Candidatus.Finniella.inopinata GCA_004210305.1_ASM421030v1
Holospora.undulata.HU1 GCA_000388175.3_ASM38817v3
Candidatus.Hepatobacter.penaei GCA_000742475.1_ASM74247v1
Candidatus.Caedibacter.acanthamoebae GCA_000743035.1_ASM74303v1
Caedimonas.varicaedens GCA_001192655.1_ASM119265v1
Candidatus.Nucleicultrix.amoebiphila.FS5 GCA_002117145.1_ASM211714v1
alpha.proteobacterium.BAL199 GCA_000171835.1_ASM17183v1
Thalassobaculum.salexigens.DSM.19539 GCA_000423805.1_ASM42380v1
Candidatus.Puniceispirillum.marinum.IMCC1322 GCA_000024465.1_ASM2446v1
Oceanibaculum.indicum GCA_003633955.1_ASM363395v1
Rhodovibrio.salinarum.DSM.9154 GCA_000515255.1_ASM51525v1
Kiloniella.laminariae.DSM.19542 GCA_000374005.1_ASM37400v1
Rhodospirillum.centenum GCA_014197805.1_ASM1419780v1
Inquilinus.limosus.DSM.16000 GCA_000423185.1_ASM42318v1
Micavibrio.aeruginosavorus.ARL.13 GCA_000226315.1_ASM22631v1
Rhodospirillum.rubrum.F11 GCA_000225955.1_ASM22595v1
Pararhodospirillum.photometricum.DSM.122 GCA_000284415.2_ASM28441v2
Roseospirillum.parvum GCA_900100455.1
Caenispirillum.salinarum.AK4 GCA_000315795.1
Magnetospirillum.magneticum.AMB.1 GCA_000009985.1_ASM998v1
Thalassospira.profundimaris.WP0211 GCA_000300275.1_ASM30027v1
Terasakiella.pusilla.DSM.6293 GCA_000688235.1_ASM68823v1
GCA_002471575.1_ASM247157v1*
GCA_002306855.1_ASM230685v1*
Tistrella.mobilis.KA081020.065 GCA_000264455.2_ASM26445v2
Geminicoccus.roseus.DSM.18922 GCA_000427665.1_ASM42766v1
Candidatus.Neoehrlichia.lotoris.str.RAC413 GCA_000964795.1_ASM96479v1
Anaplasma.phagocytophilum.str.HZ GCA_000013125.1_ASM1312v1
Ehrlichia.ruminantium.str.Welgevonden GCA_000026005.1_ASM2600v1
Wolbachia.endosymbiont.of.Drosophila.melanogaster GCA_000008025.1_ASM802v1
Candidatus.Deianiraea.vastatrix GCA_007993655.1_ASM799365v1
Rickettsiales.endosymbiont.of.Trichoplax.sp.H2 GCA_009690945.1
Candidatus.Midichloria.mitochondrii.IricVA GCA_000219355.1_ASM21935v1
Rickettsiales.endosymbiont.of.Peranema.trichophorum GCA_004210275.1_ASM421027v1
Candidatus.Jidaibacter.acanthamoeba GCA_000815465.1.UWC36
Rickettsia.bellii.RML369.C GCA_000012385.1_ASM1238v1
Candidatus.Megaira.polyxenophila
Rickettsiales.endosymbiont.of.Stachyamoeba.lipophora GCA_003932735.1_ASM393273v1
Rickettsiales.bacterium.Ac37b GCA_000746585.2_ASM74658v2
Magnetofaba.australis.IT.1 GCA_002109495.1_ASM210949v1
Magnetococcus.marinus.MC.1 GCA_000014865.1_ASM1486v1
Nitrosospira.briensis.C.128 GCA_000619905.2_ASM61990v2
Methylotenera.versatilis.301 GCA_000093025.1_ASM9302v1
Ralstonia.pickettii GCA_902374465.1.MGYG.HGUT.01384
Burkholderia.cepacia GCA_009586235.1_ASM958623v1
Dasania.marina.DSM.21967 GCA_000373485.1_ASM37348v1
Congregibacter.litoralis.KT71 GCA_000153125.2_ASM15312v2
Enterobacter.cloacae GCA_000770155.1_ASM77015v1
Alteromonas.macleodii.ATCC.27126 GCA_000172635.2_ASM17263v2
0.644561

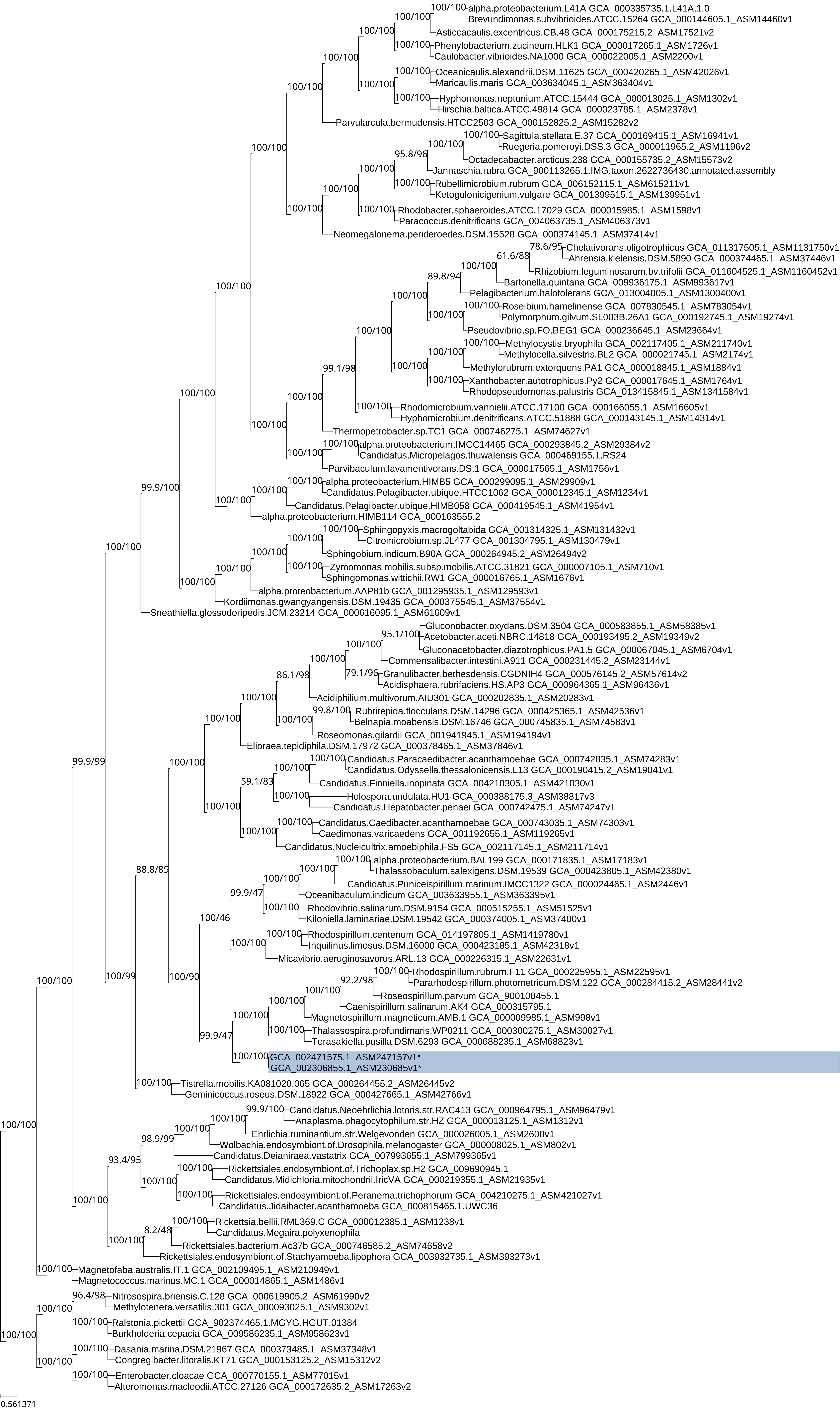
alpha.proteobacterium.L41A GCA_000335735.1.L41A.1.0
Brevundimonas.subvibrioides.ATCC.15264 GCA_000144605.1_ASM14460v1
Asticcacaulis.excentricus.CB.48 GCA_000175215.2_ASM17521v2
Phenylobacterium.zucineum.HLK1 GCA_000017265.1_ASM1726v1
Caulobacter.vibrioides.NA1000 GCA_000022005.1_ASM2200v1
Oceanicaulis.alexandrii.DSM.11625 GCA_000420265.1_ASM42026v1
Maricaulis.maris GCA_003634045.1_ASM363404v1
Hyphomonas.neptunium.ATCC.15444 GCA_000013025.1_ASM1302v1
Hirschia.baltica.ATCC.49814 GCA_000023785.1_ASM2378v1
Parvularcula.bermudensis.HTCC2503 GCA_000152825.2_ASM15282v2
Sagittula.stellata.E.37 GCA_000169415.1_ASM16941v1
Ruegeria.pomeroyi.DSS.3 GCA_000011965.2_ASM1196v2
Octadecabacter.arcticus.238 GCA_000155735.2_ASM15573v2
Jannaschia.rubra GCA_900113265.1.IMG.taxon.2622736430.annotated.assembly
Rubellimicrobium.rubrum GCA_006152115.1_ASM615211v1
Ketogulonicigenium.vulgare GCA_001399515.1_ASM139951v1
Rhodobacter.sphaeroides.ATCC.17029 GCA_000015985.1_ASM1598v1
Paracoccus.denitrificans GCA_004063735.1_ASM406373v1
Neomegalonema.perideroedes.DSM.15528 GCA_000374145.1_ASM37414v1
Chelativorans.oligotrophicus GCA_011317505.1_ASM1131750v1
Ahrensia.kielensis.DSM.5890 GCA_000374465.1_ASM37446v1
Rhizobium.leguminosarum.bv.trifolii GCA_011604525.1_ASM1160452v1
Bartonella.quintana GCA_009936175.1_ASM993617v1
Pelagibacterium.halotolerans GCA_013004005.1_ASM1300400v1
Roseibium.hamelinense GCA_007830545.1_ASM783054v1
Polymorphum.gilvum.SL003B.26A1 GCA_000192745.1_ASM19274v1
Pseudovibrio.sp.FO.BEG1 GCA_000236645.1_ASM23664v1
Methylocystis.bryophila GCA_002117405.1_ASM211740v1
Methylocella.silvestris.BL2 GCA_000021745.1_ASM2174v1
Methylorubrum.extorquens.PA1 GCA_000018845.1_ASM1884v1
Xanthobacter.autotrophicus.Py2 GCA_000017645.1_ASM1764v1
Rhodopseudomonas.palustris GCA_013415845.1_ASM1341584v1
Rhodomicrobium.vannielii.ATCC.17100 GCA_000166055.1_ASM16605v1
Hyphomicrobium.denitrificans.ATCC.51888 GCA_000143145.1_ASM14314v1
Thermopetrobacter.sp.TC1 GCA_000746275.1_ASM74627v1
alpha.proteobacterium.IMCC14465 GCA_000293845.2_ASM29384v2
Candidatus.Micropelagos.thuwalensis GCA_000469155.1.RS24
Parvibaculum.lavamentivorans.DS.1 GCA_000017565.1_ASM1756v1
alpha.proteobacterium.HIMB5 GCA_000299095.1_ASM29909v1
Candidatus.Pelagibacter.ubique.HTCC1062 GCA_000012345.1_ASM1234v1
Candidatus.Pelagibacter.ubique.HIMB058 GCA_000419545.1_ASM41954v1
alpha.proteobacterium.HIMB114 GCA_000163555.2
Sphingopyxis.macrogoltabida GCA_001314325.1_ASM131432v1
Citromicrobium.sp.JL477 GCA_001304795.1_ASM130479v1
Sphingobium.indicum.B90A GCA_000264945.2_ASM26494v2
Zymomonas.mobilis.subsp.mobilis.ATCC.31821 GCA_000007105.1_ASM710v1
Sphingomonas.wittichii.RW1 GCA_000016765.1_ASM1676v1
alpha.proteobacterium.AAP81b GCA_001295935.1_ASM129593v1
Kordiimonas.gwangyangensis.DSM.19435 GCA_000375545.1_ASM37554v1
Sneathiella.glossodoripedis.JCM.23214 GCA_000616095.1_ASM61609v1
Gluconobacter.oxydans.DSM.3504 GCA_000583855.1_ASM58385v1
Acetobacter.aceti.NBRC.14818 GCA_000193495.2_ASM19349v2
Gluconacetobacter.diazotrophicus.PA1.5 GCA_000067045.1_ASM6704v1
Commensalibacter.intestini.A911 GCA_000231445.2_ASM23144v1
Granulibacter.bethesdensis.CGDNIH4 GCA_000576145.2_ASM57614v2
Acidisphaera.rubrifaciens.HS.AP3 GCA_000964365.1_ASM96436v1
Acidiphilium.multivorum.AIU301 GCA_000202835.1_ASM20283v1
Rubritepida.flocculans.DSM.14296 GCA_000425365.1_ASM42536v1
Belnapia.moabensis.DSM.16746 GCA_000745835.1_ASM74583v1
Roseomonas.gilardii GCA_001941945.1_ASM194194v1
Elioraea.tepidiphila.DSM.17972 GCA_000378465.1_ASM37846v1
Candidatus.Paracaedibacter.acanthamoebae GCA_000742835.1_ASM74283v1
Candidatus.Odyssella.thessalonicensis.L13 GCA_000190415.2_ASM19041v1
Candidatus.Finniella.inopinata GCA_004210305.1_ASM421030v1
Holospora.undulata.HU1 GCA_000388175.3_ASM38817v3
Candidatus.Hepatobacter.penaei GCA_000742475.1_ASM74247v1
Candidatus.Caedibacter.acanthamoebae GCA_000743035.1_ASM74303v1
Caedimonas.varicaedens GCA_001192655.1_ASM119265v1
Candidatus.Nucleicultrix.amoebiphila.FS5 GCA_002117145.1_ASM211714v1
alpha.proteobacterium.BAL199 GCA_000171835.1_ASM17183v1
Thalassobaculum.salexigens.DSM.19539 GCA_000423805.1_ASM42380v1
Candidatus.Puniceispirillum.marinum.IMCC1322 GCA_000024465.1_ASM2446v1
Oceanibaculum.indicum GCA_003633955.1_ASM363395v1
Rhodovibrio.salinarum.DSM.9154 GCA_000515255.1_ASM51525v1
Kiloniella.laminariae.DSM.19542 GCA_000374005.1_ASM37400v1
Rhodospirillum.centenum GCA_014197805.1_ASM1419780v1
Inquilinus.limosus.DSM.16000 GCA_000423185.1_ASM42318v1
Micavibrio.aeruginosavorus.ARL.13 GCA_000226315.1_ASM22631v1
Rhodospirillum.rubrum.F11 GCA_000225955.1_ASM22595v1
Pararhodospirillum.photometricum.DSM.122 GCA_000284415.2_ASM28441v2
Roseospirillum.parvum GCA_900100455.1
Caenispirillum.salinarum.AK4 GCA_000315795.1
Magnetospirillum.magneticum.AMB.1 GCA_000009985.1_ASM998v1
Thalassospira.profundimaris.WP0211 GCA_000300275.1_ASM30027v1
Terasakiella.pusilla.DSM.6293 GCA_000688235.1_ASM68823v1
GCA_002471575.1_ASM247157v1*
GCA_002306855.1_ASM230685v1*
Tistrella.mobilis.KA081020.065 GCA_000264455.2_ASM26445v2
Geminicoccus.roseus.DSM.18922 GCA_000427665.1_ASM42766v1
Candidatus.Neoehrlichia.lotoris.str.RAC413 GCA_000964795.1_ASM96479v1
Anaplasma.phagocytophilum.str.HZ GCA_000013125.1_ASM1312v1
Ehrlichia.ruminantium.str.Welgevonden GCA_000026005.1_ASM2600v1
Wolbachia.endosymbiont.of.Drosophila.melanogaster GCA_000008025.1_ASM802v1
Candidatus.Deianiraea.vastatrix GCA_007993655.1_ASM799365v1
Rickettsiales.endosymbiont.of.Trichoplax.sp.H2 GCA_009690945.1
Candidatus.Midichloria.mitochondrii.IricVA GCA_000219355.1_ASM21935v1
Rickettsiales.endosymbiont.of.Peranema.trichophorum GCA_004210275.1_ASM421027v1
Candidatus.Jidaibacter.acanthamoeba GCA_000815465.1.UWC36
Rickettsia.bellii.RML369.C GCA_000012385.1_ASM1238v1
Candidatus.Megaira.polyxenophila
Rickettsiales.bacterium.Ac37b GCA_000746585.2_ASM74658v2
Rickettsiales.endosymbiont.of.Stachyamoeba.lipophora GCA_003932735.1_ASM393273v1
Magnetofaba.australis.IT.1 GCA_002109495.1_ASM210949v1
Magnetococcus.marinus.MC.1 GCA_000014865.1_ASM1486v1
Nitrosospira.briensis.C.128 GCA_000619905.2_ASM61990v2
Methylotenera.versatilis.301 GCA_000093025.1_ASM9302v1
Ralstonia.pickettii GCA_902374465.1.MGYG.HGUT.01384
Burkholderia.cepacia GCA_009586235.1_ASM958623v1
Dasania.marina.DSM.21967 GCA_000373485.1_ASM37348v1
Congregibacter.litoralis.KT71 GCA_000153125.2_ASM15312v2
Enterobacter.cloacae GCA_000770155.1_ASM77015v1
Alteromonas.macleodii.ATCC.27126 GCA_000172635.2_ASM17263v2
0.561371

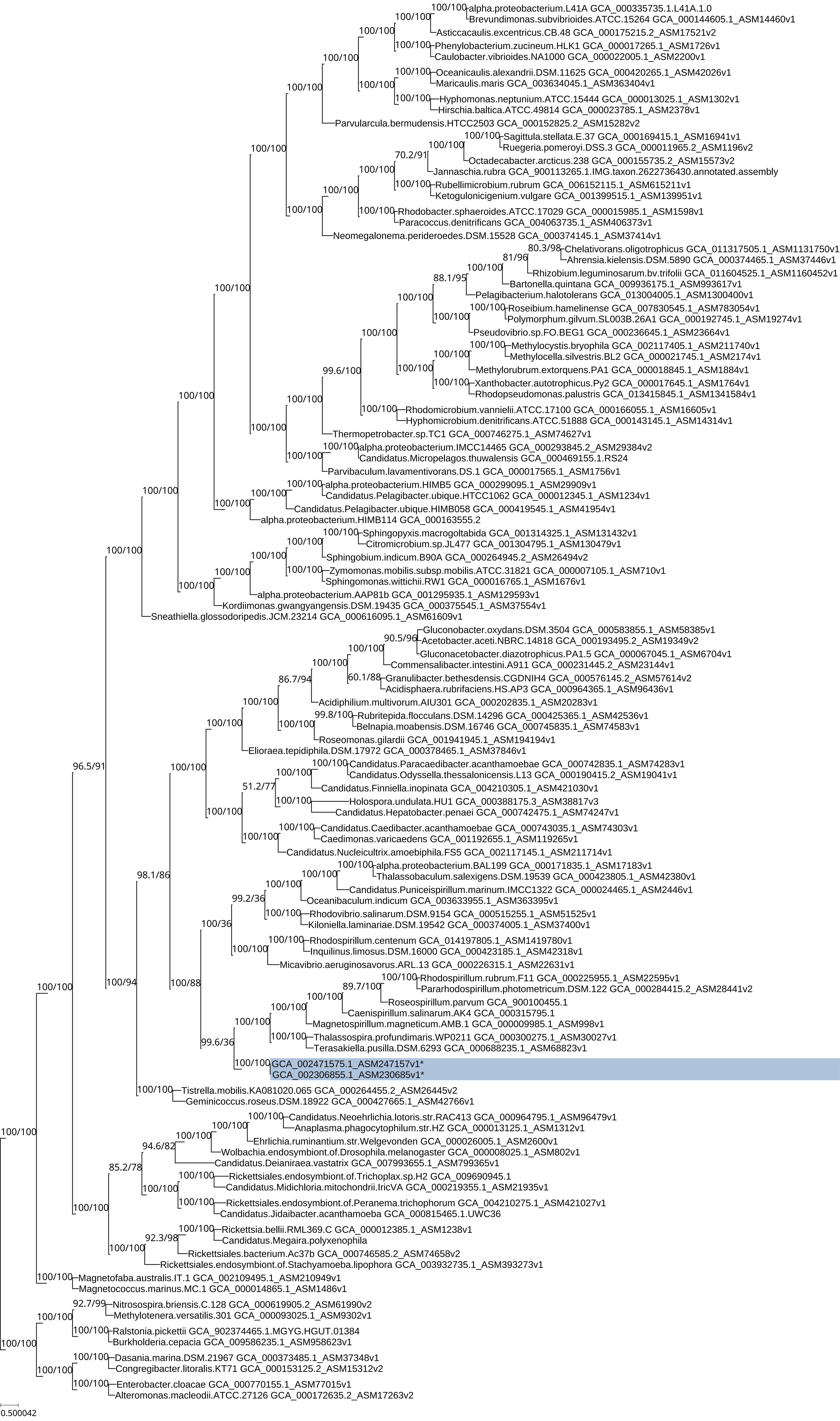
100/100
alpha.proteobacterium.L41A GCA_000335735.1.L41A.1.0
100/100
Brevundimonas.subvibrioides.ATCC.15264 GCA_000144605.1_ASM14460v1
100/100
Asticcacaulis.excentricus.CB.48 GCA_000175215.2_ASM17521v2
100/100
Phenylobacterium.zucineum.HLK1 GCA_000017265.1_ASM1726v1
100/100
Caulobacter.vibrioides.NA1000 GCA_000022005.1_ASM2200v1
100/100
Oceanicaulis.alexandrii.DSM.11625 GCA_000420265.1_ASM42026v1
100/100
Maricaulis.maris GCA_003634045.1_ASM363404v1
100/100
Hyphomonas.neptunium.ATCC.15444 GCA_000013025.1_ASM1302v1
100/100
Hirschia.baltica.ATCC.49814 GCA_000023785.1_ASM2378v1
Parvularcula.bermudensis.HTCC2503 GCA_000152825.2_ASM15282v2
100/100
Sagittula.stellata.E.37 GCA_000169415.1_ASM16941v1
100/100
Ruegeria.pomeroyi.DSS.3 GCA_000011965.2_ASM1196v2
70.2/91
Octadecabacter.arcticus.238 GCA_000155735.2_ASM15573v2
100/100
Jannaschia.rubra GCA_900113265.1.IMG.taxon.2622736430.annotated.assembly
100/100
Rubellimicrobium.rubrum GCA_006152115.1_ASM615211v1
100/100
Ketogulonicigenium.vulgare GCA_001399515.1_ASM139951v1
100/100
Rhodobacter.sphaeroides.ATCC.17029 GCA_000015985.1_ASM1598v1
100/100
Paracoccus.denitrificans GCA_004063735.1_ASM406373v1
Neomegalonema.perideroedes.DSM.15528 GCA_000374145.1_ASM37414v1
80.3/98
Chelativorans.oligotrophicus GCA_011317505.1_ASM1131750v1
81/96
Ahrensia.kielensis.DSM.5890 GCA_000374465.1_ASM37446v1
100/100
Rhizobium.leguminosarum.bv.trifolii GCA_011604525.1_ASM1160452v1
100/100
Bartonella.quintana GCA_009936175.1_ASM993617v1
88.1/95
Pelagibacterium.halotolerans GCA_013004005.1_ASM1300400v1
100/100
Roseibium.hamelinense GCA_007830545.1_ASM783054v1
100/100
Polymorphum.gilvum.SL003B.26A1 GCA_000192745.1_ASM19274v1
Pseudovibrio.sp.FO.BEG1 GCA_000236645.1_ASM23664v1
100/100
100/100
Methylocystis.bryophila GCA_002117405.1_ASM211740v1
100/100
Methylocella.silvestris.BL2 GCA_000021745.1_ASM2174v1
100/100
Methylorubrum.extorquens.PA1 GCA_000018845.1_ASM1884v1
100/100
Xanthobacter.autotrophicus.Py2 GCA_000017645.1_ASM1764v1
Rhodopseudomonas.palustris GCA_013415845.1_ASM1341584v1
99.6/100
100/100
Rhodomicrobium.vannielii.ATCC.17100 GCA_000166055.1_ASM16605v1
100/100
Hyphomicrobium.denitrificans.ATCC.51888 GCA_000143145.1_ASM14314v1
100/100
Thermopetrobacter.sp.TC1 GCA_000746275.1_ASM74627v1
100/100
alpha.proteobacterium.IMCC14465 GCA_000293845.2_ASM29384v2
100/100
Candidatus.Micropelagos.thuwalensis GCA_000469155.1.RS24
Parvibaculum.lavamentivorans.DS.1 GCA_000017565.1_ASM1756v1
100/100
100/100
alpha.proteobacterium.HIMB5 GCA_000299095.1_ASM29909v1
100/100
Candidatus.Pelagibacter.ubique.HTCC1062 GCA_000012345.1_ASM1234v1
100/100
Candidatus.Pelagibacter.ubique.HIMB058 GCA_000419545.1_ASM41954v1
alpha.proteobacterium.HIMB114 GCA_000163555.2
100/100
Sphingopyxis.macrogoltabida GCA_001314325.1_ASM131432v1
100/100
Citromicrobium.sp.JL477 GCA_001304795.1_ASM130479v1
100/100
Sphingobium.indicum.B90A GCA_000264945.2_ASM26494v2
100/100
Zymomonas.mobilis.subsp.mobilis.ATCC.31821 GCA_000007105.1_ASM710v1
100/100
Sphingomonas.wittichii.RW1 GCA_000016765.1_ASM1676v1
100/100
alpha.proteobacterium.AAP81b GCA_001295935.1_ASM129593v1
Kordiimonas.gwangyangensis.DSM.19435 GCA_000375545.1_ASM37554v1
Sneathiella.glossodoripedis.JCM.23214 GCA_000616095.1_ASM61609v1
Gluconobacter.oxydans.DSM.3504 GCA_000583855.1_ASM58385v1
90.5/96
Acetobacter.aceti.NBRC.14818 GCA_000193495.2_ASM19349v2
100/100
Gluconacetobacter.diazotrophicus.PA1.5 GCA_000067045.1_ASM6704v1
100/100
Commensalibacter.intestini.A911 GCA_000231445.2_ASM23144v1
60.1/88
Granulibacter.bethesdensis.CGDNIH4 GCA_000576145.2_ASM57614v2
86.7/94
Acidisphaera.rubrifaciens.HS.AP3 GCA_000964365.1_ASM96436v1
100/100
Acidiphilium.multivorum.AIU301 GCA_000202835.1_ASM20283v1
99.8/100
Rubritepida.flocculans.DSM.14296 GCA_000425365.1_ASM42536v1
100/100
100/100
Belnapia.moabensis.DSM.16746 GCA_000745835.1_ASM74583v1
Roseomonas.gilardii GCA_001941945.1_ASM194194v1
Elioraea.tepidiphila.DSM.17972 GCA_000378465.1_ASM37846v1
100/100
100/100
Candidatus.Paracaedibacter.acanthamoebae GCA_000742835.1_ASM74283v1
100/100
Candidatus.Odyssella.thessalonicensis.L13 GCA_000190415.2_ASM19041v1
96.5/91
51.2/77
Candidatus.Finniella.inopinata GCA_004210305.1_ASM421030v1
100/100
Holospora.undulata.HU1 GCA_000388175.3_ASM38817v3
100/100
Candidatus.Hepatobacter.penaei GCA_000742475.1_ASM74247v1
100/100
Candidatus.Caedibacter.acanthamoebae GCA_000743035.1_ASM74303v1
100/100
Caedimonas.varicaedens GCA_001192655.1_ASM119265v1
Candidatus.Nucleicultrix.amoebiphila.FS5 GCA_002117145.1_ASM211714v1
100/100
alpha.proteobacterium.BAL199 GCA_000171835.1_ASM17183v1
100/100
Thalassobaculum.salexigens.DSM.19539 GCA_000423805.1_ASM42380v1
98.1/86
100/100
Candidatus.Puniceispirillum.marinum.IMCC1322 GCA_000024465.1_ASM2446v1
99.2/36
Oceanibaculum.indicum GCA_003633955.1_ASM363395v1
100/100
Rhodovibrio.salinarum.DSM.9154 GCA_000515255.1_ASM51525v1
100/36
Kiloniella.laminariae.DSM.19542 GCA_000374005.1_ASM37400v1
100/100
Rhodospirillum.centenum GCA_014197805.1_ASM1419780v1
100/100
Inquilinus.limosus.DSM.16000 GCA_000423185.1_ASM42318v1
Micavibrio.aeruginosavorus.ARL.13 GCA_000226315.1_ASM22631v1
100/100
100/100
Rhodospirillum.rubrum.F11 GCA_000225955.1_ASM22595v1
100/94
100/88
89.7/100
Pararhodospirillum.photometricum.DSM.122 GCA_000284415.2_ASM28441v2
100/100
Roseospirillum.parvum GCA_900100455.1
100/100
Caenispirillum.salinarum.AK4 GCA_000315795.1
100/100
Magnetospirillum.magneticum.AMB.1 GCA_000009985.1_ASM998v1
100/100
Thalassospira.profundimaris.WP0211 GCA_000300275.1_ASM30027v1
99.6/36
Terasakiella.pusilla.DSM.6293 GCA_000688235.1_ASM68823v1
100/100
GCA_002471575.1_ASM247157v1*
GCA_002306855.1_ASM230685v1*
100/100
Tistrella.mobilis.KA081020.065 GCA_000264455.2_ASM26445v2
Geminicoccus.roseus.DSM.18922 GCA_000427665.1_ASM42766v1
100/100
Candidatus.Neoehrlichia.lotoris.str.RAC413 GCA_000964795.1_ASM96479v1
100/100
Anaplasma.phagocytophilum.str.HZ GCA_000013125.1_ASM1312v1
100/100
100/100
Ehrlichia.ruminantium.str.Welgevonden GCA_000026005.1_ASM2600v1
94.6/82
Wolbachia.endosymbiont.of.Drosophila.melanogaster GCA_000008025.1_ASM802v1
Candidatus.Deianiraea.vastatrix GCA_007993655.1_ASM799365v1
85.2/78
100/100
Rickettsiales.endosymbiont.of.Trichoplax.sp.H2 GCA_009690945.1
100/100
Candidatus.Midichloria.mitochondrii.IricVA GCA_000219355.1_ASM21935v1
100/100
Rickettsiales.endosymbiont.of.Peranema.trichophorum GCA_004210275.1_ASM421027v1
100/100
Candidatus.Jidaibacter.acanthamoeba GCA_000815465.1.UWC36
100/100
Rickettsia.bellii.RML369.C GCA_000012385.1_ASM1238v1
92.3/98
Candidatus.Megaira.polyxenophila
100/100
Rickettsiales.bacterium.Ac37b GCA_000746585.2_ASM74658v2
Rickettsiales.endosymbiont.of.Stachyamoeba.lipophora GCA_003932735.1_ASM393273v1
100/100
Magnetofaba.australis.IT.1 GCA_002109495.1_ASM210949v1
Magnetococcus.marinus.MC.1 GCA_000014865.1_ASM1486v1
92.7/99
Nitrosospira.briensis.C.128 GCA_000619905.2_ASM61990v2
100/100
Methylotenera.versatilis.301 GCA_000093025.1_ASM9302v1
100/100
Ralstonia.pickettii GCA_902374465.1.MGYG.HGUT.01384
100/100
Burkholderia.cepacia GCA_009586235.1_ASM958623v1
100/100
Dasania.marina.DSM.21967 GCA_000373485.1_ASM37348v1
100/100
Congregibacter.litoralis.KT71 GCA_000153125.2_ASM15312v2
100/100
Enterobacter.cloacae GCA_000770155.1_ASM77015v1
Alteromonas.macleodii.ATCC.27126 GCA_000172635.2_ASM17263v2
0.500042

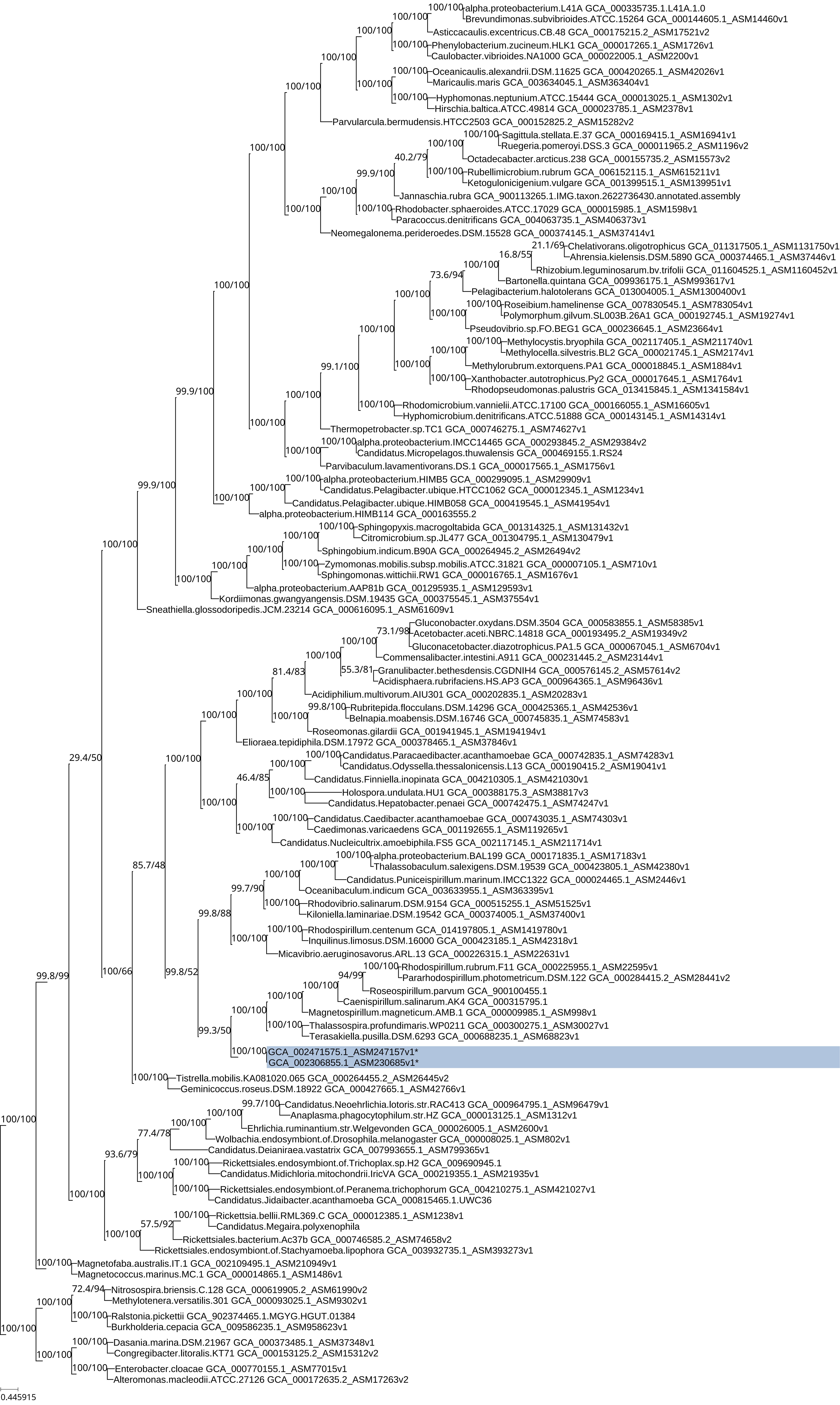
100/100
alpha.proteobacterium.L41A GCA_000335735.1_L41A.1.0
100/100
Brevundimonas.subvibrioides.ATCC.15264 GCA_000144605.1_ASM14460v1
100/100
Asticcacaulis.excentricus.CB.48 GCA_000175215.2_ASM17521v2
100/100
Phenylobacterium.zucineum.HLK1 GCA_000017265.1_ASM1726v1
Caulobacter.vibrioides.NA1000 GCA_000022005.1_ASM2200v1
100/100
100/100
Oceanicaulis.alexandrii.DSM.11625 GCA_000420265.1_ASM42026v1
100/100
Maricaulis.maris GCA_003634045.1_ASM363404v1
100/100
Hyphomonas.neptunium.ATCC.15444 GCA_000013025.1_ASM1302v1
Hirschia.baltica.ATCC.49814 GCA_000023785.1_ASM2378v1
Parvularcula.bermudensis.HTCC2503 GCA_000152825.2_ASM15282v2
100/100
100/100
Sagittula.stellata.E.37 GCA_000169415.1_ASM16941v1
100/100
Ruegeria.pomeroyi.DSS.3 GCA_000011965.2_ASM1196v2
40.2/79
Octadecabacter.arcticus.238 GCA_000155735.2_ASM15573v2
99.9/100
100/100
Rubellimicrobium.rubrum GCA_006152115.1_ASM615211v1
Ketogulonicigenium.vulgare GCA_001399515.1_ASM139951v1
100/100
Jannaschia.rubra GCA_900113265.1.IMG.taxon.2622736430.annotated.assembly
100/100
100/100
Rhodobacter.sphaeroides.ATCC.17029 GCA_000015985.1_ASM1598v1
Paracoccus.denitrificans GCA_004063735.1_ASM406373v1
Neomegalonema.perideroedes.DSM.15528 GCA_000374145.1_ASM37414v1
21.1/69
Chelativorans.oligotrophicus GCA_011317505.1_ASM1131750v1
16.8/55
Ahrensia.kielensis.DSM.5890 GCA_000374465.1_ASM37446v1
100/100
Rhizobium.leguminosarum.bv.trifolii GCA_011604525.1_ASM1160452v1
73.6/94
Bartonella.quintana GCA_009936175.1_ASM993617v1
100/100
Pelagibacterium.halotolerans GCA_013004005.1_ASM1300400v1
100/100
Roseibium.hamelinense GCA_007830545.1_ASM783054v1
100/100
Polymorphum.gilvum.SL003B.26A1 GCA_000192745.1_ASM19274v1
Pseudovibrio.sp.FO.BEG1 GCA_000236645.1_ASM23664v1
100/100
100/100
Methylocystis.bryophila GCA_002117405.1_ASM211740v1
100/100
Methylocella.silvestris.BL2 GCA_000021745.1_ASM2174v1
100/100
Methylorubrum.extorquens.PA1 GCA_000018845.1_ASM1884v1
99.1/100
100/100
Xanthobacter.autotrophicus.Py2 GCA_000017645.1_ASM1764v1
Rhodopseudomonas.palustris GCA_013415845.1_ASM1341584v1
99.9/100
100/100
100/100
Rhodomicrobium.vannielii.ATCC.17100 GCA_000166055.1_ASM16605v1
Hyphomicrobium.denitrificans.ATCC.51888 GCA_000143145.1_ASM14314v1
100/100
Thermopetrobacter.sp.TC1 GCA_000746275.1_ASM74627v1
100/100
alpha.proteobacterium.IMCC14465 GCA_000293845.2_ASM29384v2
100/100
Candidatus.Micropelagos.thuwalensis GCA_000469155.1.RS24
Parvibaculum.lavamentivorans.DS.1 GCA_000017565.1_ASM1756v1
100/100
alpha.proteobacterium.HIMB5 GCA_000299095.1_ASM29909v1
100/100
Candidatus.Pelagibacter.ubique.HTCC1062 GCA_000012345.1_ASM1234v1
100/100
Candidatus.Pelagibacter.ubique.HIMB058 GCA_000419545.1_ASM41954v1
alpha.proteobacterium.HIMB114 GCA_000163555.2
99.9/100
100/100
Sphingopyxis.macrogoltabida GCA_001314325.1_ASM131432v1
100/100
Citromicrobium.sp.JL477 GCA_001304795.1_ASM130479v1
100/100
Sphingobium.indicum.B90A GCA_000264945.2_ASM26494v2
100/100
100/100
Zymomonas.mobilis.subsp.mobilis.ATCC.31821 GCA_000007105.1_ASM710v1
100/100
Sphingomonas.wittichii.RW1 GCA_000016765.1_ASM1676v1
alpha.proteobacterium.AAP81b GCA_001295935.1_ASM129593v1
Kordiimonas.gwangyangensis.DSM.19435 GCA_000375545.1_ASM37554v1
Sneathiella.glossodoripedis.JCM.23214 GCA_000616095.1_ASM61609v1
Gluconobacter.oxydans.DSM.3504 GCA_000583855.1_ASM58385v1
73.1/98
Acetobacter.aceti.NBRC.14818 GCA_000193495.2_ASM19349v2
100/100
Gluconacetobacter.diazotrophicus.PA1.5 GCA_000067045.1_ASM6704v1
100/100
Commensalibacter.intestini.A911 GCA_000231445.2_ASM23144v1
81.4/83
55.3/81
Granulibacter.bethesdensis.CGDNIH4 GCA_000576145.2_ASM57614v2
Acidisphaera.rubrifaciens.HS.AP3 GCA_000964365.1_ASM96436v1
100/100
Acidiphilium.multivorum.AIU301 GCA_000202835.1_ASM20283v1
99.8/100
Rubritepida.flocculans.DSM.14296 GCA_000425365.1_ASM42536v1
100/100
100/100
Belnapia.moabensis.DSM.16746 GCA_000745835.1_ASM74583v1
Roseomonas.gilardii GCA_001941945.1_ASM194194v1
Elioraea.tepidiphila.DSM.17972 GCA_000378465.1_ASM37846v1
100/100
100/100
Candidatus.Paracaedibacter.acanthamoebae GCA_000742835.1_ASM74283v1
100/100
Candidatus.Odyssella.thessalonicensis.L13 GCA_000190415.2_ASM19041v1
46.4/85
Candidatus.Finniella.inopinata GCA_004210305.1_ASM421030v1
100/100
Holospora.undulata.HU1 GCA_000388175.3_ASM38817v3
100/100
Candidatus.Hepatobacter.penaei GCA_000742475.1_ASM74247v1
100/100
Candidatus.Caedibacter.acanthamoebae GCA_000743035.1_ASM74303v1
100/100
Caedimonas.varicaedens GCA_001192655.1_ASM119265v1
Candidatus.Nucleicultrix.amoebiphila.FS5 GCA_002117145.1_ASM211714v1
100/100
alpha.proteobacterium.BAL199 GCA_000171835.1_ASM17183v1
100/100
Thalassobaculum.salexigens.DSM.19539 GCA_000423805.1_ASM42380v1
100/100
Candidatus.Puniceispirillum.marinum.IMCC1322 GCA_000024465.1_ASM2446v1
99.7/90
Oceanibaculum.indicum GCA_003633955.1_ASM363395v1
100/100
Rhodovibrio.salinarum.DSM.9154 GCA_000515255.1_ASM51525v1
99.8/88
Kiloniella.laminariae.DSM.19542 GCA_000374005.1_ASM37400v1
100/100
Rhodospirillum.centenum GCA_014197805.1_ASM1419780v1
100/100
Inquilinus.limosus.DSM.16000 GCA_000423185.1_ASM42318v1
Micavibrio.aeruginosavorus.ARL.13 GCA_000226315.1_ASM22631v1
100/100
Rhodospirillum.rubrum.F11 GCA_000225955.1_ASM22595v1
94/99
Pararhodospirillum.photometricum.DSM.122 GCA_000284415.2_ASM28441v2
85.7/48
100/100
Roseospirillum.parvum GCA_900100455.1
100/100
Caenispirillum.salinarum.AK4 GCA_000315795.1
29.4/50
99.8/52
Magnetospirillum.magneticum.AMB.1 GCA_000009985.1_ASM998v1
100/66
100/100
Thalassospira.profundimaris.WP0211 GCA_000300275.1_ASM30027v1
99.3/50
Terasakiella.pusilla.DSM.6293 GCA_000688235.1_ASM68823v1
100/100
GCA_002471575.1_ASM247157v1*
GCA_002306855.1_ASM230685v1*
100/100
Tistrella.mobilis.KA081020.065 GCA_000264455.2_ASM26445v2
Geminicoccus.roseus.DSM.18922 GCA_000427665.1_ASM42766v1
99.7/100
Candidatus.Neoehrlichia.lotoris.str.RAC413 GCA_000964795.1_ASM96479v1
100/100
Anaplasma.phagocytophilum.str.HZ GCA_000013125.1_ASM1312v1
100/100
Ehrlichia.ruminantium.str.Welgevonden GCA_000026005.1_ASM2600v1
77.4/78
Wolbachia.endosymbiont.of.Drosophila.melanogaster GCA_000008025.1_ASM802v1
Candidatus.Deianiraea.vastatrix GCA_007993655.1_ASM799365v1
93.6/79
100/100
Rickettsiales.endosymbiont.of.Trichoplax.sp.H2 GCA_009690945.1
100/100
Candidatus.Midichloria.mitochondrii.IricVA GCA_000219355.1_ASM21935v1
100/100
Rickettsiales.endosymbiont.of.Peranema.trichophorum GCA_004210275.1_ASM421027v1
100/100
Candidatus.Jidaibacter.acanthamoeba GCA_000815465.1.UWC36
100/100
Rickettsia.bellii.RML369.C GCA_000012385.1_ASM1238v1
57.5/92
Candidatus.Megaira.polyxenophila
100/100
Rickettsiales.bacterium.Ac37b GCA_000746585.2_ASM74658v2
Rickettsiales.endosymbiont.of.Stachyamoeba.lipophora GCA_003932735.1_ASM393273v1
100/100
100/100
Magnetofaba.australis.IT.1 GCA_002109495.1_ASM210949v1
99.8/99
Magnetococcus.marinus.MC.1 GCA_000014865.1_ASM1486v1
72.4/94
Nitrosospira.briensis.C.128 GCA_000619905.2_ASM61990v2
100/100
Methylotenera.versatilis.301 GCA_000093025.1_ASM9302v1
100/100
Ralstonia.pickettii GCA_902374465.1.MGYG.HGUT.01384
Burkholderia.cepacia GCA_009586235.1_ASM958623v1
100/100
100/100
Dasania.marina.DSM.21967 GCA_000373485.1_ASM37348v1
100/100
Congregibacter.litoralis.KT71 GCA_000153125.2_ASM15312v2
100/100
Enterobacter.cloacae GCA_000770155.1_ASM77015v1
Alteromonas.macleodii.ATCC.27126 GCA_000172635.2_ASM17263v2
0.445915

Group C

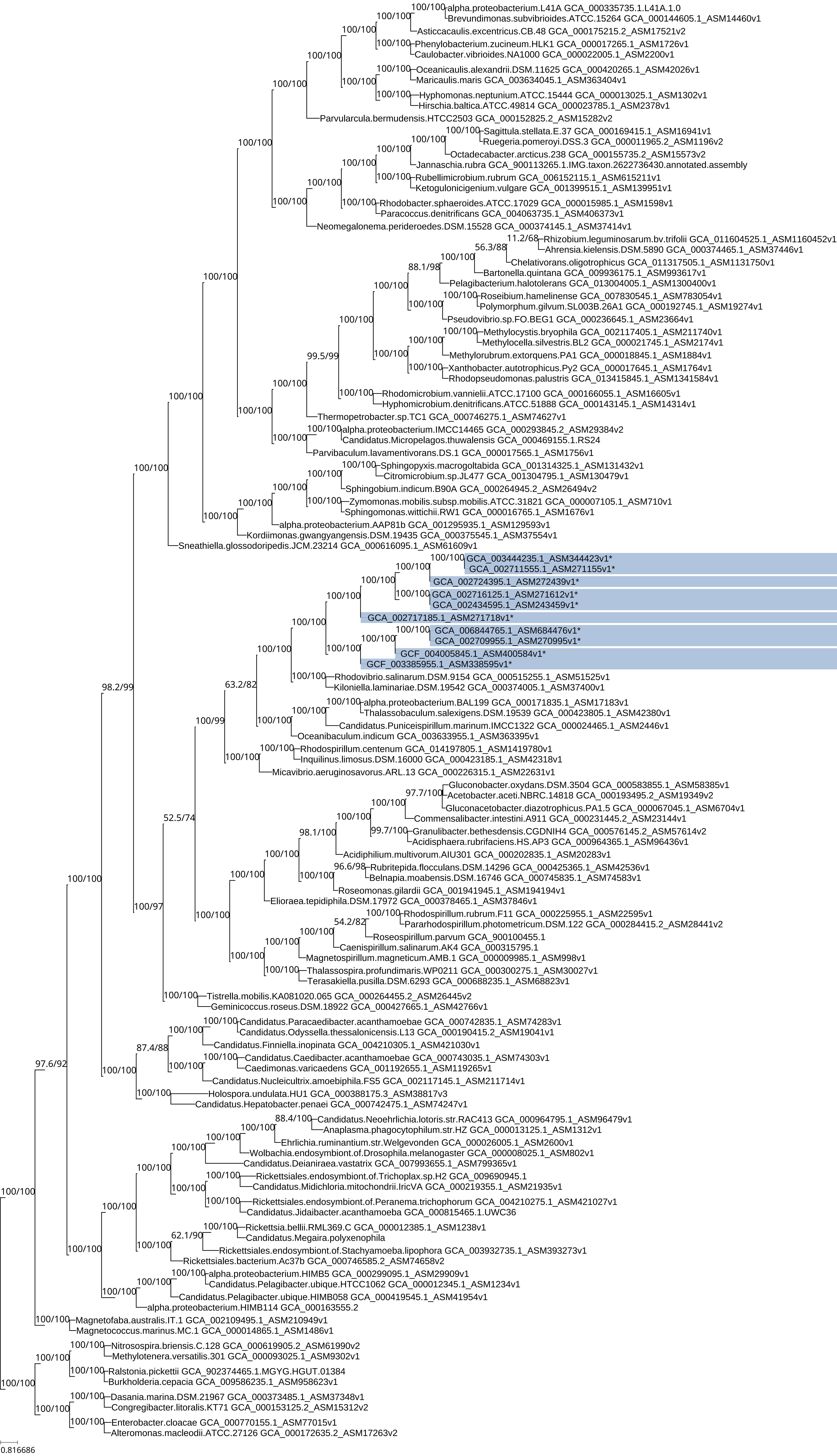

100/100
alpha.proteobacterium.L41A GCA_000335735.1_L41A.1.0
100/100
Brevundimonas.subvibrioides.ATCC.15264 GCA_000144605.1_ASM14460v1
100/100
Asticcacaulis.excentricus.CB.48 GCA_000175215.2_ASM17521v2
100/100
Phenylobacterium.zucineum.HLK1 GCA_000017265.1_ASM1726v1
100/100
Caulobacter.vibrioides.NA1000 GCA_000022005.1_ASM2200v1
100/100
Oceanicaulis.alexandrii.DSM.11625 GCA_000420265.1_ASM42026v1
100/100
Maricaulis.maris GCA_003634045.1_ASM363404v1
100/100
Hyphomonas.neptunium.ATCC.15444 GCA_000013025.1_ASM1302v1
100/100
Hirschia.baltica.ATCC.49814 GCA_000023785.1_ASM2378v1
Parvularcula.bermudensis.HTCC2503 GCA_000152825.2_ASM15282v2
100/100
Sagittula.stellata.E.37 GCA_000169415.1_ASM16941v1
100/100
Ruegeria.pomeroyi.DSS.3 GCA_000011965.2_ASM1196v2
100/100
Octadecabacter.arcticus.238 GCA_000155735.2_ASM15573v2
100/100
Jannaschia.rubra GCA_900113265.1.IMG.taxon.2622736430.annotated.assembly
100/100
Rubellimicrobium.rubrum GCA_006152115.1_ASM615211v1
100/100
Ketogulonicigenium.vulgare GCA_001399515.1_ASM139951v1
100/100
Rhodobacter.sphaeroides.ATCC.17029 GCA_000015985.1_ASM1598v1
100/100
Paracoccus.denitrificans GCA_004063735.1_ASM406373v1
Neomegalonema.perideroedes.DSM.15528 GCA_000374145.1_ASM37414v1
11.2/68
Rhizobium.leguminosarum.bv.trifolii GCA_011604525.1_ASM1160452v1
56.3/88
Ahrensia.kielensis.DSM.5890 GCA_000374465.1_ASM37446v1
100/100
Chelativorans.oligotrophicus GCA_011317505.1_ASM1131750v1
100/100
Bartonella.quintana GCA_009936175.1_ASM993617v1
88.1/98
Pelagibacterium.halotolerans GCA_013004005.1_ASM1300400v1
100/100
Roseibium.hamelinense GCA_007830545.1_ASM783054v1
100/100
Polymorphum.gilvum.SL003B.26A1 GCA_000192745.1_ASM19274v1
100/100
Pseudovibrio.sp.FO.BEG1 GCA_000236645.1_ASM23664v1
100/100
Methylocystis.bryophila GCA_002117405.1_ASM211740v1
100/100
Methylocella.silvestris.BL2 GCA_000021745.1_ASM2174v1
99.5/99
Methylorubrum.extorquens.PA1 GCA_000018845.1_ASM1884v1
100/100
Xanthobacter.autotrophicus.Py2 GCA_000017645.1_ASM1764v1
100/100
Rhodopseudomonas.palustris GCA_013415845.1_ASM1341584v1
100/100
Rhodomicrobium.vannielii.ATCC.17100 GCA_000166055.1_ASM16605v1
100/100
Hyphomicrobium.denitrificans.ATCC.51888 GCA_000143145.1_ASM14314v1
100/100
Thermopetrobacter.sp.TC1 GCA_000746275.1_ASM74627v1
100/100
alpha.proteobacterium.IMCC14465 GCA_000293845.2_ASM29384v2
100/100
Candidatus.Micropelagos.thuwalensis GCA_000469155.1.RS24
Parvibaculum.lavamentivorans.DS.1 GCA_000017565.1_ASM1756v1
100/100
Sphingopyxis.macrogoltabida GCA_001314325.1_ASM131432v1
100/100
Citromicrobium.sp.JL477 GCA_001304795.1_ASM130479v1
100/100
Sphingobium.indicum.B90A GCA_000264945.2_ASM26494v2
100/100
Zymomonas.mobilis.subsp.mobilis.ATCC.31821 GCA_000007105.1_ASM710v1
100/100
Sphingomonas.wittichii.RW1 GCA_000016765.1_ASM1676v1
100/100
alpha.proteobacterium.AAP81b GCA_001295935.1_ASM129593v1
Kordiimonas.gwangyangensis.DSM.19435 GCA_000375545.1_ASM37554v1
100/100
Sneathiella.glossodoripedis.JCM.23214 GCA_000616095.1_ASM61609v1
100/100
GCA_003444235.1_ASM344423v1*
100/100
GCA_002711555.1_ASM271155v1*
100/100
GCA_002724395.1_ASM272439v1*
100/100
GCA_002716125.1_ASM271612v1*
GCA_002434595.1_ASM243459v1*
100/100
GCA_002717185.1_ASM271718v1*
100/100
GCA_006844765.1_ASM684476v1*
100/100
GCA_002709955.1_ASM270995v1*
100/100
GCF_004005845.1_ASM400584v1*
GCF_003385955.1_ASM338595v1*
100/100
Rhodovibrio.salinarum.DSM.9154 GCA_000515255.1_ASM51525v1
Kiloniella.laminariae.DSM.19542 GCA_000374005.1_ASM37400v1
98.2/99
100/100
alpha.proteobacterium.BAL199 GCA_000171835.1_ASM17183v1
63.2/82
100/100
Thalassobaculum.salexigens.DSM.19539 GCA_000423805.1_ASM42380v1
100/99
100/100
Candidatus.Puniceispirillum.marinum.IMCC1322 GCA_000024465.1_ASM2446v1
Oceanibaculum.indicum GCA_003633955.1_ASM363395v1
100/100
Rhodospirillum.centenum GCA_014197805.1_ASM1419780v1
100/100
Inquilinus.limosus.DSM.16000 GCA_000423185.1_ASM42318v1
Micavibrio.aeruginosavorus.ARL.13 GCA_000226315.1_ASM22631v1
52.5/74
Gluconobacter.oxydans.DSM.3504 GCA_000583855.1_ASM58385v1
97.7/100
Acetobacter.aceti.NBRC.14818 GCA_000193495.2_ASM19349v2
100/100
Gluconacetobacter.diazotrophicus.PA1.5 GCA_000067045.1_ASM6704v1
100/100
Commensalibacter.intestini.A911 GCA_000231445.2_ASM23144v1
98.1/100
99.7/100
Granulibacter.bethesdensis.CGDNIH4 GCA_000576145.2_ASM57614v2
Acidisphaera.rubrifaciens.HS.AP3 GCA_000964365.1_ASM96436v1
100/100
100/100
Acidiphilium.multivorum.AIU301 GCA_000202835.1_ASM20283v1
96.6/98
Rubritepida.flocculans.DSM.14296 GCA_000425365.1_ASM42536v1
100/100
Belnapia.moabensis.DSM.16746 GCA_000745835.1_ASM74583v1
100/100
Roseomonas.gilardii GCA_001941945.1_ASM194194v1
Elioraea.tepidiphila.DSM.17972 GCA_000378465.1_ASM37846v1
100/97
100/100
Rhodospirillum.rubrum.F11 GCA_000225955.1_ASM22595v1
100/100
54.2/82
Pararhodospirillum.photometricum.DSM.122 GCA_000284415.2_ASM28441v2
100/100
Roseospirillum.parvum GCA_900100455.1
100/100
Caenispirillum.salinarum.AK4 GCA_000315795.1
100/100
Magnetospirillum.magneticum.AMB.1 GCA_000009985.1_ASM998v1
100/100
Thalassospira.profundimaris.WP0211 GCA_000300275.1_ASM30027v1
Terasakiella.pusilla.DSM.6293 GCA_000688235.1_ASM68823v1
100/100
Tistrella.mobilis.KA081020.065 GCA_000264455.2_ASM26445v2
Geminicoccus.roseus.DSM.18922 GCA_000427665.1_ASM42766v1
100/100
Candidatus.Paracaedibacter.acanthamoebae GCA_000742835.1_ASM74283v1
100/100
Candidatus.Odyssella.thessalonicensis.L13 GCA_000190415.2_ASM19041v1
87.4/88
Candidatus.Finniella.inopinata GCA_004210305.1_ASM421030v1
100/100
Candidatus.Caedibacter.acanthamoebae GCA_000743035.1_ASM74303v1
100/100
Caedimonas.varicaedens GCA_001192655.1_ASM119265v1
97.6/92
Candidatus.Nucleicultrix.amoebiphila.FS5 GCA_002117145.1_ASM211714v1
100/100
100/100
Holospora.undulata.HU1 GCA_000388175.3_ASM38817v3
Candidatus.Hepatobacter.penaei GCA_000742475.1_ASM74247v1
88.4/100
Candidatus.Neoehrlichia.lotoris.str.RAC413 GCA_000964795.1_ASM96479v1
100/100
Anaplasma.phagocytophilum.str.HZ GCA_000013125.1_ASM1312v1
100/100
Ehrlichia.ruminantium.str.Welgevonden GCA_000026005.1_ASM2600v1
100/100
Wolbachia.endosymbiont.of.Drosophila.melanogaster GCA_000008025.1_ASM802v1
100/100
Candidatus.Deianiraea.vastatrix GCA_007993655.1_ASM799365v1
100/100
100/100
Rickettsiales.endosymbiont.of.Trichoplax.sp.H2 GCA_009690945.1
100/100
Candidatus.Midichloria.mitochondrii.IricVA GCA_000219355.1_ASM21935v1
100/100
100/100
Rickettsiales.endosymbiont.of.Peranema.trichophorum GCA_004210275.1_ASM421027v1
Candidatus.Jidaibacter.acanthamoeba GCA_000815465.1.UWC36
100/100
Rickettsia.bellii.RML369.C GCA_000012385.1_ASM1238v1
62.1/90
Candidatus.Megaira.polyxenophila
100/100
100/100
Rickettsiales.endosymbiont.of.Stachyamoeba.lipophora GCA_003932735.1_ASM393273v1
Rickettsiales.bacterium.Ac37b GCA_000746585.2_ASM74658v2
100/100
alpha.proteobacterium.HIMB5 GCA_000299095.1_ASM29909v1
100/100
Candidatus.Pelagibacter.ubique.HTCC1062 GCA_000012345.1_ASM1234v1
100/100
Candidatus.Pelagibacter.ubique.HIMB058 GCA_000419545.1_ASM41954v1
alpha.proteobacterium.HIMB114 GCA_000163555.2
100/100
Magnetofaba.australis.IT.1 GCA_002109495.1_ASM210949v1
Magnetococcus.marinus.MC.1 GCA_000014865.1_ASM1486v1
100/100
Nitrosospira.briensis.C.128 GCA_000619905.2_ASM61990v2
100/100
Methylotenera.versatilis.301 GCA_000093025.1_ASM9302v1
100/100
Ralstonia.pickettii GCA_902374465.1.MGYG.HGUT.01384
Burkholderia.cepacia GCA_009586235.1_ASM958623v1
100/100
100/100
Dasania.marina.DSM.21967 GCA_000373485.1_ASM37348v1
Congregibacter.litoralis.KT71 GCA_000153125.2_ASM15312v2
100/100
100/100
Enterobacter.cloacae GCA_000770155.1_ASM77015v1
Alteromonas.macleodii.ATCC.27126 GCA_000172635.2_ASM17263v2
0.816686

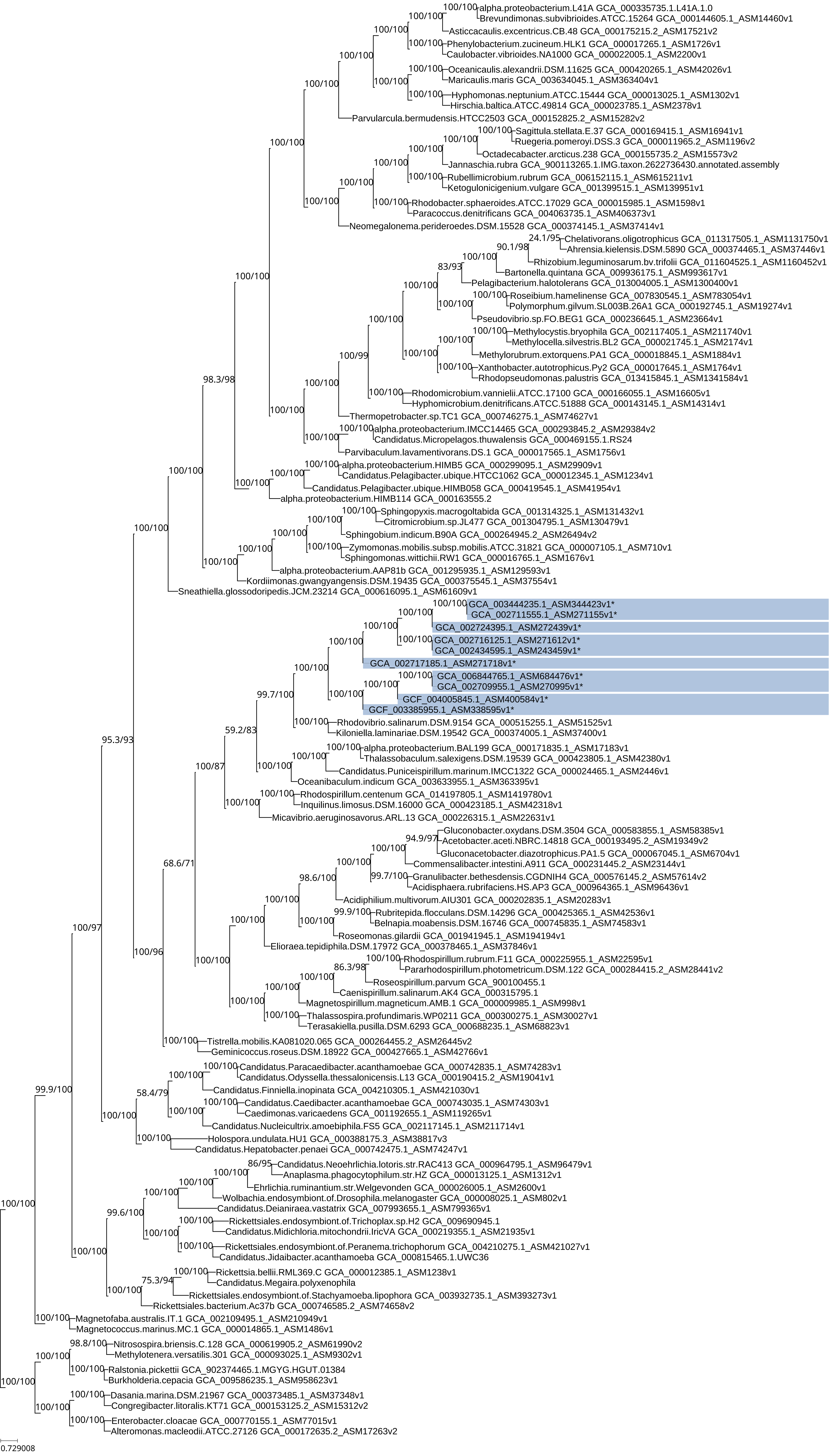

100/100 alpha.proteobacterium.L41A GCA_000335735.1.L41A.1.0
Brevundimonas.subvibrioides.ATCC.15264 GCA_000144605.1_ASM14460v1
100/100
Asticcacaulis.excentricus.CB.48 GCA_000175215.2_ASM17521v2
100/100
100/100 Phenylobacterium.zucineum.HLK1 GCA_000017265.1_ASM1726v1
Caulobacter.vibrioides.NA1000 GCA_000022005.1_ASM2200v1
100/100
100/100 Oceanicaulis.alexandrii.DSM.11625 GCA_000420265.1_ASM42026v1
100/100
Maricaulis.maris GCA_003634045.1_ASM363404v1
100/100 Hyphomonas.neptunium.ATCC.15444 GCA_000013025.1_ASM1302v1
Hirschia.baltica.ATCC.49814 GCA_000023785.1_ASM2378v1
Parvularcula.bermudensis.HTCC2503 GCA_000152825.2_ASM15282v2
100/100 Sagittula.stellata.E.37 GCA_000169415.1_ASM16941v1
100/100
Ruegeria.pomeroyi.DSS.3 GCA_000011965.2_ASM1196v2
100/100
Octadecabacter.arcticus.238 GCA_000155735.2_ASM15573v2
100/100
Jannaschia.rubra GCA_900113265.1.IMG.taxon.2622736430.annotated.assembly
100/100
100/100 Rubellimicrobium.rubrum GCA_006152115.1_ASM615211v1
100/100
Ketogulonicigenium.vulgare GCA_001399515.1_ASM139951v1
100/100
100/100 Rhodobacter.sphaeroides.ATCC.17029 GCA_000015985.1_ASM1598v1
Paracoccus.denitrificans GCA_004063735.1_ASM406373v1
Neomegalonema.perideroedes.DSM.15528 GCA_000374145.1_ASM37414v1
24.1/95 Chelativorans.oligotrophicus GCA_011317505.1_ASM1131750v1
90.1/98
Ahrensia.kielensis.DSM.5890 GCA_000374465.1_ASM37446v1
100/100
Rhizobium.leguminosarum.bv.trifolii GCA_011604525.1_ASM1160452v1
83/93
Bartonella.quintana GCA_009936175.1_ASM993617v1
100/100
Pelagibacterium.halotolerans GCA_013004005.1_ASM1300400v1
100/100 Roseibium.hamelinense GCA_007830545.1_ASM783054v1
100/100
Polymorphum.gilvum.SL003B.26A1 GCA_000192745.1_ASM19274v1
98.3/98
Pseudovibrio.sp.FO.BEG1 GCA_000236645.1_ASM23664v1
100/100
100/100 Methylocystis.bryophila GCA_002117405.1_ASM211740v1
100/100
Methylocella.silvestris.BL2 GCA_000021745.1_ASM2174v1
100/99
100/100
Methylorubrum.extorquens.PA1 GCA_000018845.1_ASM1884v1
100/100 Xanthobacter.autotrophicus.Py2 GCA_000017645.1_ASM1764v1
100/100
Rhodopseudomonas.palustris GCA_013415845.1_ASM1341584v1
100/100 Rhodomicrobium.vannielii.ATCC.17100 GCA_000166055.1_ASM16605v1
Hyphomicrobium.denitrificans.ATCC.51888 GCA_000143145.1_ASM14314v1
100/100
Thermopetrobacter.sp.TC1 GCA_000746275.1_ASM74627v1
100/100 alpha.proteobacterium.IMCC14465 GCA_000293845.2_ASM29384v2
100/100
Candidatus.Micropelagos.thuwalensis GCA_000469155.1.RS24
Parvibaculum.lavamentivorans.DS.1 GCA_000017565.1_ASM1756v1
100/100
100/100 alpha.proteobacterium.HIMB5 GCA_000299095.1_ASM29909v1
100/100
Candidatus.Pelagibacter.ubique.HTCC1062 GCA_000012345.1_ASM1234v1
100/100
Candidatus.Pelagibacter.ubique.HIMB058 GCA_000419545.1_ASM41954v1
alpha.proteobacterium.HIMB114 GCA_000163555.2
100/100
100/100 Sphingopyxis.macrogoltabida GCA_001314325.1_ASM131432v1
100/100
Citromicrobium.sp.JL477 GCA_001304795.1_ASM130479v1
100/100
Sphingobium.indicum.B90A GCA_000264945.2_ASM26494v2
100/100 Zymomonas.mobilis.subsp.mobilis.ATCC.31821 GCA_000007105.1_ASM710v1
100/100
Sphingomonas.wittichii.RW1 GCA_000016765.1_ASM1676v1
100/100
alpha.proteobacterium.AAP81b GCA_001295935.1_ASM129593v1
Kordiimonas.gwangyangensis.DSM.19435 GCA_000375545.1_ASM37554v1
Sneathiella.glossodoripedis.JCM.23214 GCA_000616095.1_ASM61609v1
100/100 GCA_003444235.1_ASM344423v1*
100/100
GCA_002711555.1_ASM271155v1*
100/100
GCA_002724395.1_ASM272439v1*
100/100 GCA_002716125.1_ASM271612v1*
100/100
GCA_002434595.1_ASM243459v1*
100/100
GCA_002717185.1_ASM271718v1*
100/100 GCA_006844765.1_ASM684476v1*
95.3/93
99.7/100
100/100
GCA_002709955.1_ASM270995v1*
100/100
GCF_004005845.1_ASM400584v1*
GCF_003385955.1_ASM338595v1*
100/100 Rhodovibrio.salinarum.DSM.9154 GCA_000515255.1_ASM51525v1
59.2/83
Kiloniella.laminariae.DSM.19542 GCA_000374005.1_ASM37400v1
100/100 alpha.proteobacterium.BAL199 GCA_000171835.1_ASM17183v1
100/100
Thalassobaculum.salexigens.DSM.19539 GCA_000423805.1_ASM42380v1
100/87
100/100
Candidatus.Puniceispirillum.marinum.IMCC1322 GCA_000024465.1_ASM2446v1
Oceanibaculum.indicum GCA_003633955.1_ASM363395v1
100/100 Rhodospirillum.centenum GCA_014197805.1_ASM1419780v1
100/100
Inquilinus.limosus.DSM.16000 GCA_000423185.1_ASM42318v1
Micavibrio.aeruginosavorus.ARL.13 GCA_000226315.1_ASM22631v1
Gluconobacter.oxydans.DSM.3504 GCA_000583855.1_ASM58385v1
94.9/97
Acetobacter.aceti.NBRC.14818 GCA_000193495.2_ASM19349v2
100/100
Gluconacetobacter.diazotrophicus.PA1.5 GCA_000067045.1_ASM6704v1
68.6/71
100/100
Commensalibacter.intestini.A911 GCA_000231445.2_ASM23144v1
99.7/100 Granulibacter.bethesdensis.CGDNIH4 GCA_000576145.2_ASM57614v2
98.6/100
Acidisphaera.rubrifaciens.HS.AP3 GCA_000964365.1_ASM96436v1
100/100
Acidiphilium.multivorum.AIU301 GCA_000202835.1_ASM20283v1
99.9/100 Rubritepida.flocculans.DSM.14296 GCA_000425365.1_ASM42536v1
100/100
100/100
Belnapia.moabensis.DSM.16746 GCA_000745835.1_ASM74583v1
100/97
Roseomonas.gilardii GCA_001941945.1_ASM194194v1
100/96
Elioraea.tepidiphila.DSM.17972 GCA_000378465.1_ASM37846v1
100/100 Rhodospirillum.rubrum.F11 GCA_000225955.1_ASM22595v1
86.3/98
Pararhodospirillum.photometricum.DSM.122 GCA_000284415.2_ASM28441v2
100/100
100/100
Roseospirillum.parvum GCA_900100455.1
Caenispirillum.salinarum.AK4 GCA_000315795.1
100/100
Magnetospirillum.magneticum.AMB.1 GCA_000009985.1_ASM998v1
100/100
100/100 Thalassospira.profundimaris.WP0211 GCA_000300275.1_ASM30027v1
Terasakiella.pusilla.DSM.6293 GCA_000688235.1_ASM68823v1
100/100 Tistrella.mobilis.KA081020.065 GCA_000264455.2_ASM26445v2
Geminicoccus.roseus.DSM.18922 GCA_000427665.1_ASM42766v1
100/100 Candidatus.Paracaedibacter.acanthamoebae GCA_000742835.1_ASM74283v1
100/100
Candidatus.Odyssella.thessalonicensis.L13 GCA_000190415.2_ASM19041v1
58.4/79
Candidatus.Finniella.inopinata GCA_004210305.1_ASM421030v1
99.9/100
100/100 Candidatus.Caedibacter.acanthamoebae GCA_000743035.1_ASM74303v1
100/100
Caedimonas.varicaedens GCA_001192655.1_ASM119265v1
100/100
Candidatus.Nucleicultrix.amoebiphila.FS5 GCA_002117145.1_ASM211714v1
100/100
Holospora.undulata.HU1 GCA_000388175.3_ASM38817v3
Candidatus.Hepatobacter.penaei GCA_000742475.1_ASM74247v1
86/95 Candidatus.Neoehrlichia.lotoris.str.RAC413 GCA_000964795.1_ASM96479v1
100/100
Anaplasma.phagocytophilum.str.HZ GCA_000013125.1_ASM1312v1
100/100
Ehrlichia.ruminantium.str.Welgevonden GCA_000026005.1_ASM2600v1
100/100
Wolbachia.endosymbiont.of.Drosophila.melanogaster GCA_000008025.1_ASM802v1
100/100
Candidatus.Deianiraea.vastatrix GCA_007993655.1_ASM799365v1
99.6/100
100/100 Rickettsiales.endosymbiont.of.Trichoplax.sp.H2 GCA_009690945.1
100/100
Candidatus.Midichloria.mitochondrii.IricVA GCA_000219355.1_ASM21935v1
100/100 Rickettsiales.endosymbiont.of.Peranema.trichophorum GCA_004210275.1_ASM421027v1
100/100
Candidatus.Jidaibacter.acanthamoeba GCA_000815465.1.UWC36
100/100 Rickettsia.bellii.RML369.C GCA_000012385.1_ASM1238v1
75.3/94
Candidatus.Megaira.polyxenophila
100/100
Rickettsiales.endosymbiont.of.Stachyamoeba.lipophora GCA_003932735.1_ASM393273v1
Rickettsiales.bacterium.Ac37b GCA_000746585.2_ASM74658v2
100/100 Magnetofaba.australis.IT.1 GCA_002109495.1_ASM210949v1
Magnetococcus.marinus.MC.1 GCA_000014865.1_ASM1486v1
98.8/100 Nitrosospira.briensis.C.128 GCA_000619905.2_ASM61990v2
100/100
Methylotenera.versatilis.301 GCA_000093025.1_ASM9302v1
100/100 Ralstonia.pickettii GCA_902374465.1.MGYG.HGUT.01384
100/100
Burkholderia.cepacia GCA_009586235.1_ASM958623v1
100/100 Dasania.marina.DSM.21967 GCA_000373485.1_ASM37348v1
100/100
Congregibacter.litoralis.KT71 GCA_000153125.2_ASM15312v2
100/100 Enterobacter.cloacae GCA_000770155.1_ASM77015v1
Alteromonas.macleodii.ATCC.27126 GCA_000172635.2_ASM17263v2
0.729008

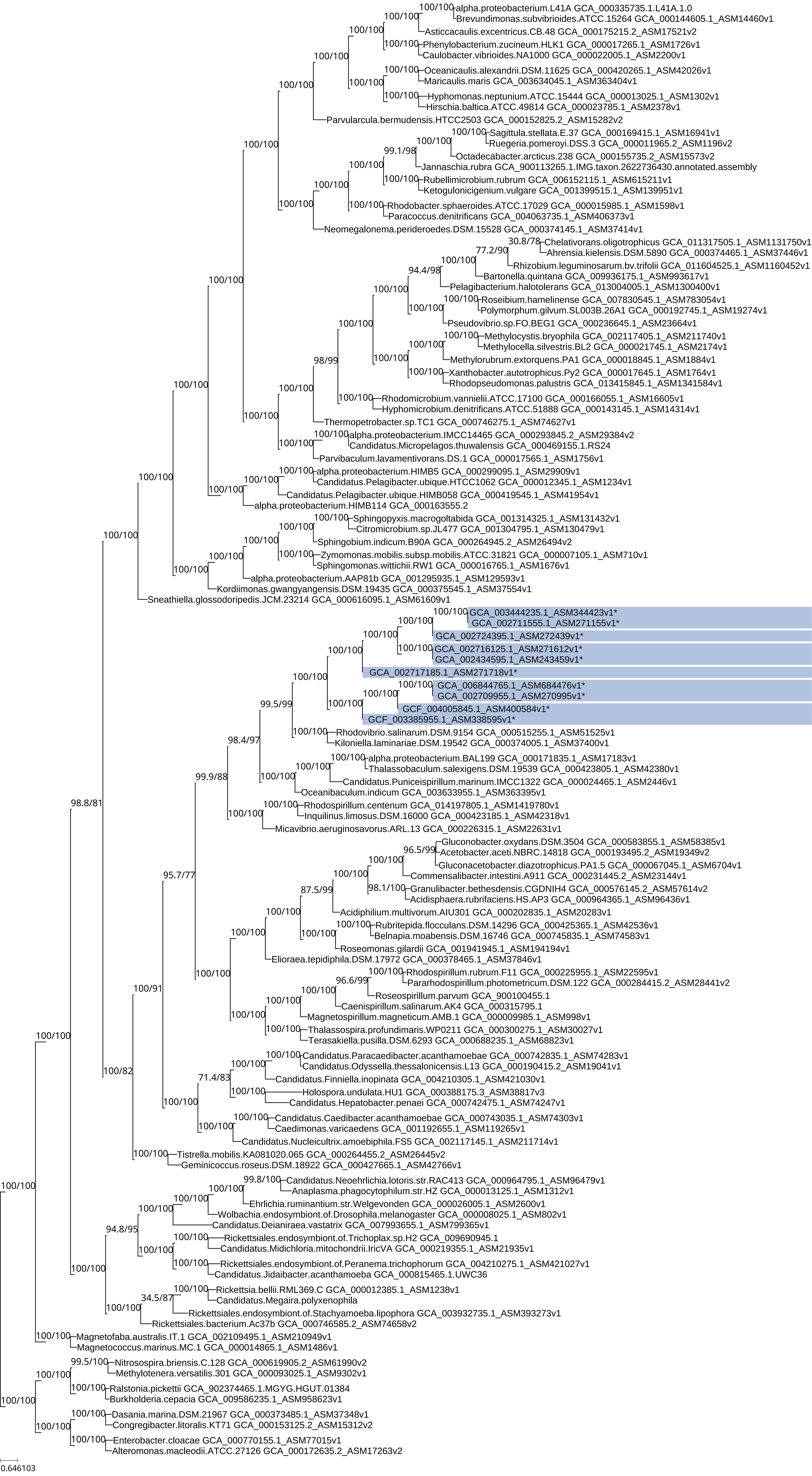
100/100
alpha.proteobacterium.L41A GCA_000335735.1.L41A.1.0
Brevundimonas.subvibrioides.ATCC.15264 GCA_000144605.1_ASM14460v1
100/100
Asticcacaulis.excentricus.CB.48 GCA_000175215.2_ASM17521v2
100/100
100/100
Phenylobacterium.zucineum.HLK1 GCA_000017265.1_ASM1726v1
Caulobacter.vibrioides.NA1000 GCA_000022005.1_ASM2200v1
100/100
100/100
Oceanicaulis.alexandrii.DSM.11625 GCA_000420265.1_ASM42026v1
Maricaulis.maris GCA_003634045.1_ASM363404v1
100/100
100/100
100/100
Hyphomonas.neptunium.ATCC.15444 GCA_000013025.1_ASM1302v1
Hirschia.baltica.ATCC.49814 GCA_000023785.1_ASM2378v1
Parvularcula.bermudensis.HTCC2503 GCA_000152825.2_ASM15282v2
100/100
Sagittula.stellata.E.37 GCA_000169415.1_ASM16941v1
100/100
Ruegeria.pomeroyi.DSS.3 GCA_000011965.2_ASM1196v2
100/100
99.1/98
Octadecabacter.arcticus.238 GCA_000155735.2_ASM15573v2
Jannaschia.rubra GCA_900113265.1.IMG.taxon.2622736430.annotated.assembly
100/100
100/100
Rubellimicrobium.rubrum GCA_006152115.1_ASM615211v1
100/100
Ketogulonicigenium.vulgare GCA_001399515.1_ASM139951v1
100/100
100/100
Rhodobacter.sphaeroides.ATCC.17029 GCA_000015985.1_ASM1598v1
Paracoccus.denitrificans GCA_004063735.1_ASM406373v1
Neomegalonema.perideroedes.DSM.15528 GCA_000374145.1_ASM37414v1
30.8/78
Chelativorans.oligotrophicus GCA_011317505.1_ASM1131750v1
77.2/90
Ahrensia.kielensis.DSM.5890 GCA_000374465.1_ASM37446v1
100/100
Rhizobium.leguminosarum.bv.trifolii GCA_011604525.1_ASM1160452v1
100/100
94.4/98
Bartonella.quintana GCA_009936175.1_ASM993617v1
Pelagibacterium.halotolerans GCA_013004005.1_ASM1300400v1
100/100
100/100
Roseibium.hamelinense GCA_007830545.1_ASM783054v1
100/100
Polymorphum.gilvum.SL003B.26A1 GCA_000192745.1_ASM19274v1
Pseudovibrio.sp.FO.BEG1 GCA_000236645.1_ASM23664v1
100/100
100/100
Methylocystis.bryophila GCA_002117405.1_ASM211740v1
100/100
Methylocella.silvestris.BL2 GCA_000021745.1_ASM2174v1
98/99
100/100
Methylorubrum.extorquens.PA1 GCA_000018845.1_ASM1884v1
100/100
Xanthobacter.autotrophicus.Py2 GCA_000017645.1_ASM1764v1
Rhodopseudomonas.palustris GCA_013415845.1_ASM1341584v1
100/100
100/100
Rhodomicrobium.vannielii.ATCC.17100 GCA_000166055.1_ASM16605v1
100/100
Hyphomicrobium.denitrificans.ATCC.51888 GCA_000143145.1_ASM14314v1
Thermopetrobacter.sp.TC1 GCA_000746275.1_ASM74627v1
100/100
alpha.proteobacterium.IMCC14465 GCA_000293845.2_ASM29384v2
100/100
Candidatus.Micropelagos.thuwalensis GCA_000469155.1.RS24
Parvibaculum.lavamentivorans.DS.1 GCA_000017565.1_ASM1756v1
100/100
100/100
alpha.proteobacterium.HIMB5 GCA_000299095.1_ASM29909v1
100/100
Candidatus.Pelagibacter.ubique.HTCC1062 GCA_000012345.1_ASM1234v1
100/100
Candidatus.Pelagibacter.ubique.HIMB058 GCA_000419545.1_ASM41954v1
alpha.proteobacterium.HIMB114 GCA_000163555.2
100/100
100/100
Sphingopyxis.macrogoltabida GCA_001314325.1_ASM131432v1
100/100
Citromicrobium.sp.JL477 GCA_001304795.1_ASM130479v1
100/100
Sphingobium.indicum.B90A GCA_000264945.2_ASM26494v2
100/100
100/100
Zymomonas.mobilis.subsp.mobilis.ATCC.31821 GCA_000007105.1_ASM710v1
Sphingomonas.wittichii.RW1 GCA_000016765.1_ASM1676v1
100/100
alpha.proteobacterium.AAP81b GCA_001295935.1_ASM129593v1
Kordiimonas.gwangyangensis.DSM.19435 GCA_000375545.1_ASM37554v1
Sneathiella.glossodoripedis.JCM.23214 GCA_000616095.1_ASM61609v1
100/100
GCA_003444235.1_ASM344423v1*
100/100
GCA_002711555.1_ASM271155v1*
100/100
GCA_002724395.1_ASM272439v1*
100/100
100/100
GCA_002716125.1_ASM271612v1*
GCA_002434595.1_ASM243459v1*
GCA_002717185.1_ASM271718v1*
100/100
100/100
GCA_006844765.1_ASM684476v1*
100/100
GCA_002709955.1_ASM270995v1*
100/100
GCF_004005845.1_ASM400584v1*
99.5/99
GCF_003385955.1_ASM338595v1*
100/100
Rhodovibrio.salinarum.DSM.9154 GCA_000515255.1_ASM51525v1
Kiloniella.laminariae.DSM.19542 GCA_000374005.1_ASM37400v1
98.4/97
100/100
alpha.proteobacterium.BAL199 GCA_000171835.1_ASM17183v1
100/100
Thalassobaculum.salexigens.DSM.19539 GCA_000423805.1_ASM42380v1
99.9/88
100/100
Candidatus.Puniceispirillum.marinum.IMCC1322 GCA_000024465.1_ASM2446v1
Oceanibaculum.indicum GCA_003633955.1_ASM363395v1
98.8/81
100/100
Rhodospirillum.centenum GCA_014197805.1_ASM1419780v1
100/100
Inquilinus.limosus.DSM.16000 GCA_000423185.1_ASM42318v1
Micavibrio.aeruginosavorus.ARL.13 GCA_000226315.1_ASM22631v1
96.5/99
Gluconobacter.oxydans.DSM.3504 GCA_000583855.1_ASM58385v1
100/100
Acetobacter.aceti.NBRC.14818 GCA_000193495.2_ASM19349v2
Gluconacetobacter.diazotrophicus.PA1.5 GCA_000067045.1_ASM6704v1
100/100
Commensalibacter.intestini.A911 GCA_000231445.2_ASM23144v1
95.7/77
87.5/99
98.1/100
Granulibacter.bethesdensis.CGDNIH4 GCA_000576145.2_ASM57614v2
Acidisphaera.rubrifaciens.HS.AP3 GCA_000964365.1_ASM96436v1
100/100
Acidiphilium.multivorum.AIU301 GCA_000202835.1_ASM20283v1
100/100
Rubritepida.flocculans.DSM.14296 GCA_000425365.1_ASM42536v1
100/100
100/100
Belnapia.moabensis.DSM.16746 GCA_000745835.1_ASM74583v1
Roseomonas.gilardii GCA_001941945.1_ASM194194v1
100/100
Elioraea.tepidiphila.DSM.17972 GCA_000378465.1_ASM37846v1
100/100
Rhodospirillum.rubrum.F11 GCA_000225955.1_ASM22595v1
96.6/99
Pararhodospirillum.photometricum.DSM.122 GCA_000284415.2_ASM28441v2
100/91
100/100
Roseospirillum.parvum GCA_900100455.1
100/100
Caenispirillum.salinarum.AK4 GCA_000315795.1
100/100
Magnetospirillum.magneticum.AMB.1 GCA_000009985.1_ASM998v1
100/100
Thalassospira.profundimaris.WP0211 GCA_000300275.1_ASM30027v1
Terasakiella.pusilla.DSM.6293 GCA_000688235.1_ASM68823v1
100/100
100/100
Candidatus.Paracaedibacter.acanthamoebae GCA_000742835.1_ASM74283v1
100/100
Candidatus.Odyssella.thessalonicensis.L13 GCA_000190415.2_ASM19041v1
100/82
71.4/83
Candidatus.Finniella.inopinata GCA_004210305.1_ASM421030v1
100/100
Holospora.undulata.HU1 GCA_000388175.3_ASM38817v3
100/100
Candidatus.Hepatobacter.penaei GCA_000742475.1_ASM74247v1
100/100
Candidatus.Caedibacter.acanthamoebae GCA_000743035.1_ASM74303v1
100/100
Caedimonas.varicaedens GCA_001192655.1_ASM119265v1
Candidatus.Nucleicultrix.amoebiphila.FS5 GCA_002117145.1_ASM211714v1
100/100
Tistrella.mobilis.KA081020.065 GCA_000264455.2_ASM26445v2
Geminicoccus.roseus.DSM.18922 GCA_000427665.1_ASM42766v1
100/100
99.8/100
Candidatus.Neoehrlichia.lotoris.str.RAC413 GCA_000964795.1_ASM96479v1
100/100
Anaplasma.phagocytophilum.str.HZ GCA_000013125.1_ASM1312v1
100/100
Ehrlichia.ruminantium.str.Welgevonden GCA_000026005.1_ASM2600v1
100/100
Wolbachia.endosymbiont.of.Drosophila.melanogaster GCA_000008025.1_ASM802v1
Candidatus.Deianiraea.vastatrix GCA_007993655.1_ASM799365v1
94.8/95
100/100
Rickettsiales.endosymbiont.of.Trichoplax.sp.H2 GCA_009690945.1
100/100
Candidatus.Midichloria.mitochondrii.IricVA GCA_000219355.1_ASM21935v1
100/100
Rickettsiales.endosymbiont.of.Peranema.trichophorum GCA_004210275.1_ASM421027v1
100/100
Candidatus.Jidaibacter.acanthamoeba GCA_000815465.1.UWC36
100/100
Rickettsia.bellii.RML369.C GCA_000012385.1_ASM1238v1
34.5/87
Candidatus.Megaira.polyxenophila
100/100
Rickettsiales.endosymbiont.of.Stachyamoeba.lipophora GCA_003932735.1_ASM393273v1
Rickettsiales.bacterium.Ac37b GCA_000746585.2_ASM74658v2
100/100
Magnetofaba.australis.IT.1 GCA_002109495.1_ASM210949v1
Magnetococcus.marinus.MC.1 GCA_000014865.1_ASM1486v1
99.5/100
Nitrosospira.briensis.C.128 GCA_000619905.2_ASM61990v2
100/100
Methylotenera.versatilis.301 GCA_000093025.1_ASM9302v1
100/100
Ralstonia.pickettii GCA_902374465.1.MGYG.HGUT.01384
Burkholderia.cepacia GCA_009586235.1_ASM958623v1
100/100
100/100
Dasania.marina.DSM.21967 GCA_000373485.1_ASM37348v1
Congregibacter.litoralis.KT71 GCA_000153125.2_ASM15312v2
100/100
100/100
Enterobacter.cloacae GCA_000770155.1_ASM77015v1
Alteromonas.macleodii.ATCC.27126 GCA_000172635.2_ASM17263v2
0.646103

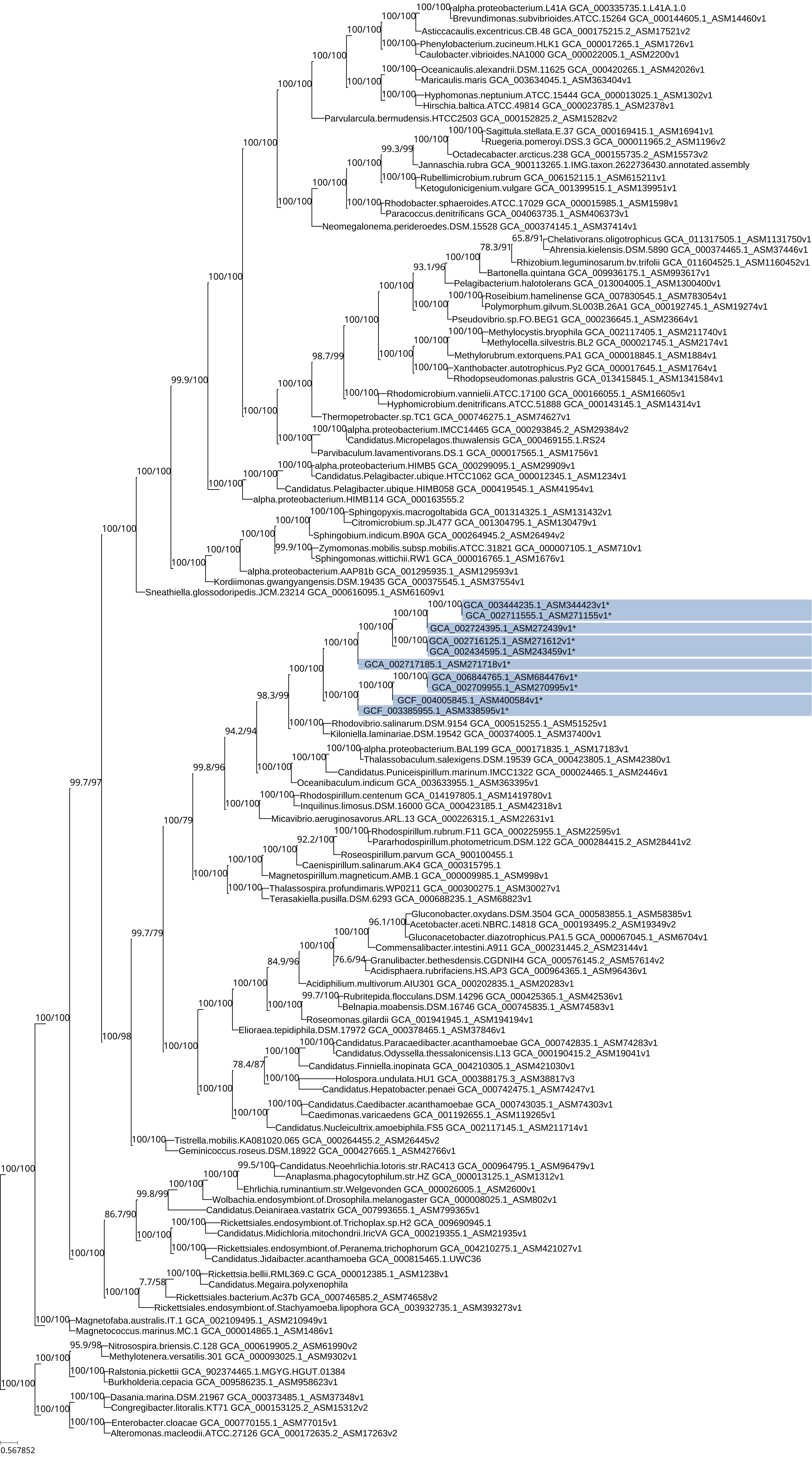

alpha.proteobacterium.L41A GCA_000335735.1.L41A.1.0
Brevundimonas.subvibrioides.ATCC.15264 GCA_000144605.1_ASM14460v1
Asticcacaulis.excentricus.CB.48 GCA_000175215.2_ASM17521v2
Phenylobacterium.zucineum.HLK1 GCA_000017265.1_ASM1726v1
Caulobacter.vibrioides.NA1000 GCA_000022005.1_ASM2200v1
Oceanicaulis.alexandrii.DSM.11625 GCA_000420265.1_ASM42026v1
Maricaulis.maris GCA_003634045.1_ASM363404v1
Hyphomonas.neptunium.ATCC.15444 GCA_000013025.1_ASM1302v1
Hirschia.baltica.ATCC.49814 GCA_000023785.1_ASM2378v1
Parvularcula.bermudensis.HTCC2503 GCA_000152825.2_ASM15282v2
Sagittula.stellata.E.37 GCA_000169415.1_ASM16941v1
Ruegeria.pomeroyi.DSS.3 GCA_000011965.2_ASM1196v2
Octadecabacter.arcticus.238 GCA_000155735.2_ASM15573v2
Jannaschia.rubra GCA_900113265.1.IMG.taxon.2622736430.annotated.assembly
Rubellimicrobium.rubrum GCA_006152115.1_ASM615211v1
Ketogulonicigenium.vulgare GCA_001399515.1_ASM139951v1
Rhodobacter.sphaeroides.ATCC.17029 GCA_000015985.1_ASM1598v1
Paracoccus.denitrificans GCA_004063735.1_ASM406373v1
Neomegalonema.perideroedes.DSM.15528 GCA_000374145.1_ASM37414v1
Chelativorans.oligotrophicus GCA_011317505.1_ASM1131750v1
Ahrensia.kielensis.DSM.5890 GCA_000374465.1_ASM37446v1
Rhizobium.leguminosarum.bv.trifolii GCA_011604525.1_ASM1160452v1
Bartonella.quintana GCA_009936175.1_ASM993617v1
Pelagibacterium.halotolerans GCA_013004005.1_ASM1300400v1
Roseibium.hamelinense GCA_007830545.1_ASM783054v1
Polymorphum.gilvum.SL003B.26A1 GCA_000192745.1_ASM19274v1
Pseudovibrio.sp.FO.BEG1 GCA_000236645.1_ASM23664v1
Methylocystis.bryophila GCA_002117405.1_ASM211740v1
Methylocella.silvestris.BL2 GCA_000021745.1_ASM2174v1
Methylorubrum.extorquens.PA1 GCA_000018845.1_ASM1884v1
Xanthobacter.autotrophicus.Py2 GCA_000017645.1_ASM1764v1
Rhodopseudomonas.palustris GCA_013415845.1_ASM1341584v1
Rhodomicrobium.vannielii.ATCC.17100 GCA_000166055.1_ASM16605v1
Hyphomicrobium.denitrificans.ATCC.51888 GCA_000143145.1_ASM14314v1
Thermopetrobacter.sp.TC1 GCA_000746275.1_ASM74627v1
alpha.proteobacterium.IMCC14465 GCA_000293845.2_ASM29384v2
Candidatus.Micropelagos.thuwalensis GCA_000469155.1.RS24
Parvibaculum.lavamentivorans.DS.1 GCA_000017565.1_ASM1756v1
alpha.proteobacterium.HIMB5 GCA_000299095.1_ASM29909v1
Candidatus.Pelagibacter.ubique.HTCC1062 GCA_000012345.1_ASM1234v1
Candidatus.Pelagibacter.ubique.HIMB058 GCA_000419545.1_ASM41954v1
alpha.proteobacterium.HIMB114 GCA_000163555.2
Sphingopyxis.macrogoltabida GCA_001314325.1_ASM131432v1
Citromicrobium.sp.JL477 GCA_001304795.1_ASM130479v1
Sphingobium.indicum.B90A GCA_000264945.2_ASM26494v2
Zymomonas.mobilis.subsp.mobilis.ATCC.31821 GCA_000007105.1_ASM710v1
Sphingomonas.wittichii.RW1 GCA_000016765.1_ASM1676v1
alpha.proteobacterium.AAP81b GCA_001295935.1_ASM129593v1
Kordiimonas.gwangyangensis.DSM.19435 GCA_000375545.1_ASM37554v1
Sneathiella.glossodoripedis.JCM.23214 GCA_000616095.1_ASM61609v1
GCA_003444235.1_ASM344423v1*
GCA_002711555.1_ASM271155v1*
GCA_002724395.1_ASM272439v1*
GCA_002716125.1_ASM271612v1*
GCA_002434595.1_ASM243459v1*
GCA_002717185.1_ASM271718v1*
GCA_006844765.1_ASM684476v1*
GCA_002709955.1_ASM270995v1*
GCF_004005845.1_ASM400584v1*
GCF_003385955.1_ASM338595v1*
Rhodovibrio.salinarum.DSM.9154 GCA_000515255.1_ASM51525v1
Kiloniella.laminariae.DSM.19542 GCA_000374005.1_ASM37400v1
alpha.proteobacterium.BAL199 GCA_000171835.1_ASM17183v1
Thalassobaculum.salexigens.DSM.19539 GCA_000423805.1_ASM42380v1
Candidatus.Puniceispirillum.marinum.IMCC1322 GCA_000024465.1_ASM2446v1
Oceanibaculum.indicum GCA_003633955.1_ASM363395v1
Rhodospirillum.centenum GCA_014197805.1_ASM1419780v1
Inquilinus.limosus.DSM.16000 GCA_000423185.1_ASM42318v1
Micavibrio.aeruginosavorus.ARL.13 GCA_000226315.1_ASM22631v1
Rhodospirillum.rubrum.F11 GCA_000225955.1_ASM22595v1
Pararhodospirillum.photometricum.DSM.122 GCA_000284415.2_ASM28441v2
Roseospirillum.parvum GCA_900100455.1
Caenispirillum.salinarum.AK4 GCA_000315795.1
Magnetospirillum.magneticum.AMB.1 GCA_000009985.1_ASM998v1
Thalassospira.profundimaris.WP0211 GCA_000300275.1_ASM30027v1
Terasakiella.pusilla.DSM.6293 GCA_000688235.1_ASM68823v1
Gluconobacter.oxydans.DSM.3504 GCA_000583855.1_ASM58385v1
Acetobacter.aceti.NBRC.14818 GCA_000193495.2_ASM19349v2
Gluconacetobacter.diazotrophicus.PA1.5 GCA_000067045.1_ASM6704v1
Commensalibacter.intestini.A911 GCA_000231445.2_ASM23144v1
Granulibacter.bethesdensis.CGDNIH4 GCA_000576145.2_ASM57614v2
Acidisphaera.rubrifaciens.HS.AP3 GCA_000964365.1_ASM96436v1
Acidiphilium.multivorum.AIU301 GCA_000202835.1_ASM20283v1
Rubritepida.flocculans.DSM.14296 GCA_000425365.1_ASM42536v1
Belnapia.moabensis.DSM.16746 GCA_000745835.1_ASM74583v1
Roseomonas.gilardii GCA_001941945.1_ASM194194v1
Elioraea.tepidiphila.DSM.17972 GCA_000378465.1_ASM37846v1
Candidatus.Paracaedibacter.acanthamoebae GCA_000742835.1_ASM74283v1
Candidatus.Odyssella.thessalonicensis.L13 GCA_000190415.2_ASM19041v1
Candidatus.Finniella.inopinata GCA_004210305.1_ASM421030v1
Holospora.undulata.HU1 GCA_000388175.3_ASM38817v3
Candidatus.Hepatobacter.penaei GCA_000742475.1_ASM74247v1
Candidatus.Caedibacter.acanthamoebae GCA_000743035.1_ASM74303v1
Caedimonas.varicaedens GCA_001192655.1_ASM119265v1
Candidatus.Nucleicultrix.amoebiphila.FS5 GCA_002117145.1_ASM211714v1
Tistrella.mobilis.KA081020.065 GCA_000264455.2_ASM26445v2
Geminicoccus.roseus.DSM.18922 GCA_000427665.1_ASM42766v1
Candidatus.Neoehrlichia.lotoris.str.RAC413 GCA_000964795.1_ASM96479v1
Anaplasma.phagocytophilum.str.HZ GCA_000013125.1_ASM1312v1
Ehrlichia.ruminantium.str.Welgevonden GCA_000026005.1_ASM2600v1
Wolbachia.endosymbiont.of.Drosophila.melanogaster GCA_000008025.1_ASM802v1
Candidatus.Deianiraea.vastatrix GCA_007993655.1_ASM799365v1
Rickettsiales.endosymbiont.of.Trichoplax.sp.H2 GCA_009690945.1
Candidatus.Midichloria.mitochondrii.IricVA GCA_000219355.1_ASM21935v1
Rickettsiales.endosymbiont.of.Peranema.trichophorum GCA_004210275.1_ASM421027v1
Candidatus.Jidaibacter.acanthamoeba GCA_000815465.1.UWC36
Rickettsia.bellii.RML369.C GCA_000012385.1_ASM1238v1
Candidatus.Megaira.polyxenophila
Rickettsiales.bacterium.Ac37b GCA_000746585.2_ASM74658v2
Rickettsiales.endosymbiont.of.Stachyamoeba.lipophora GCA_003932735.1_ASM393273v1
Magnetofaba.australis.IT.1 GCA_002109495.1_ASM210949v1
Magnetococcus.marinus.MC.1 GCA_000014865.1_ASM1486v1
Nitrosospira.briensis.C.128 GCA_000619905.2_ASM61990v2
Methylotenera.versatilis.301 GCA_000093025.1_ASM9302v1
Ralstonia.pickettii GCA_902374465.1.MGYG.HGUT.01384
Burkholderia.cepacia GCA_009586235.1_ASM958623v1
Dasania.marina.DSM.21967 GCA_000373485.1_ASM37348v1
Congregibacter.litoralis.KT71 GCA_000153125.2_ASM15312v2
Enterobacter.cloacae GCA_000770155.1_ASM77015v1
Alteromonas.macleodii.ATCC.27126 GCA_000172635.2_ASM17263v2
0.567852

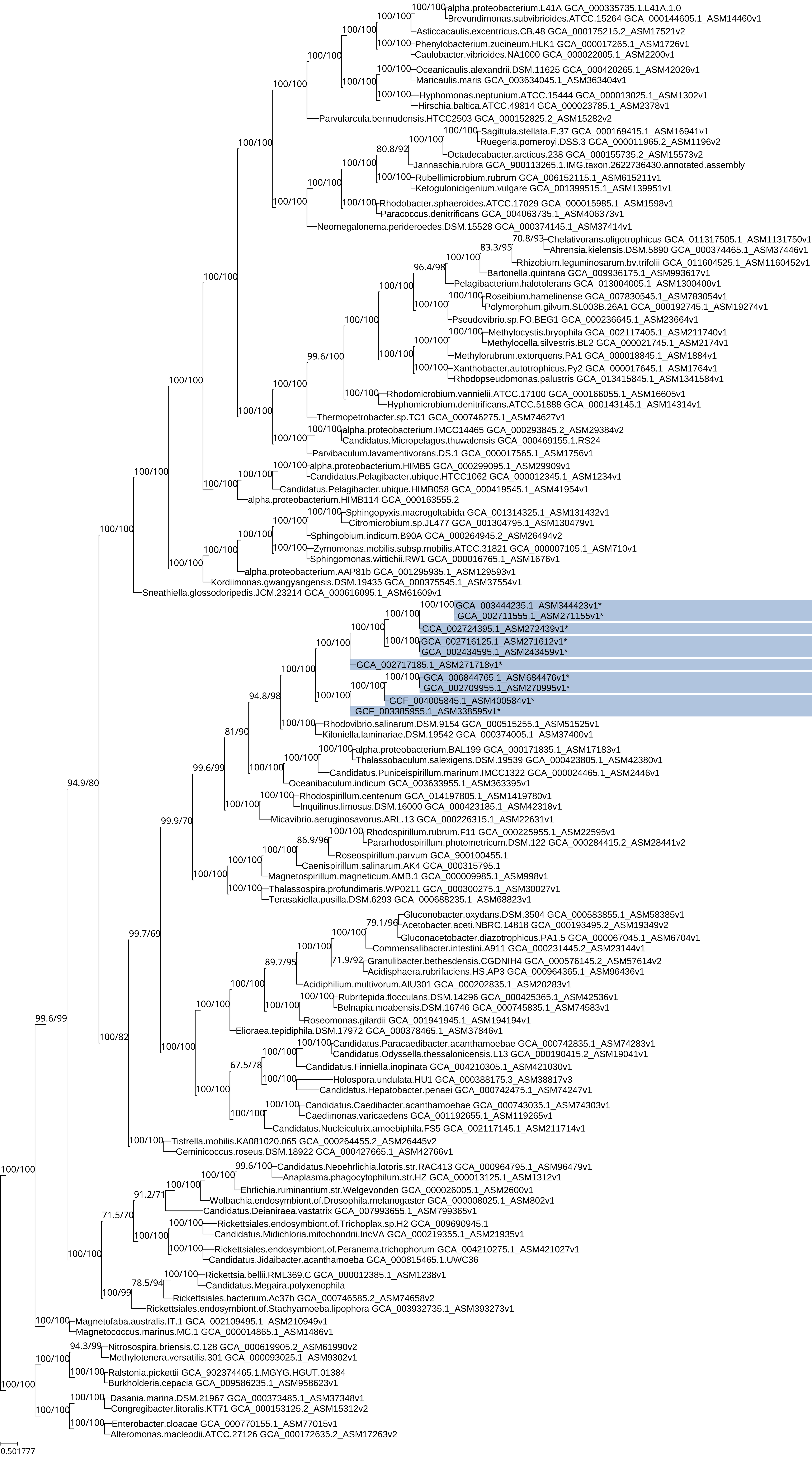
alpha.proteobacterium.L41A GCA_000335735.1.L41A.1.0
Brevundimonas.subvibrioides.ATCC.15264 GCA_000144605.1_ASM14460v1
Asticcacaulis.excentricus.CB.48 GCA_000175215.2_ASM17521v2
Phenylobacterium.zucineum.HLK1 GCA_000017265.1_ASM1726v1
Caulobacter.vibrioides.NA1000 GCA_000022005.1_ASM2200v1
Oceanicaulis.alexandrii.DSM.11625 GCA_000420265.1_ASM42026v1
Maricaulis.maris GCA_003634045.1_ASM363404v1
Hyphomonas.neptunium.ATCC.15444 GCA_000013025.1_ASM1302v1
Hirschia.baltica.ATCC.49814 GCA_000023785.1_ASM2378v1
Parvularcula.bermudensis.HTCC2503 GCA_000152825.2_ASM15282v2
Sagittula.stellata.E.37 GCA_000169415.1_ASM16941v1
Ruegeria.pomeroyi.DSS.3 GCA_000011965.2_ASM1196v2
Octadecabacter.arcticus.238 GCA_000155735.2_ASM15573v2
Jannaschia.rubra GCA_900113265.1.IMG.taxon.2622736430.annotated.assembly
Rubellimicrobium.rubrum GCA_006152115.1_ASM615211v1
Ketogulonicigenium.vulgare GCA_001399515.1_ASM139951v1
Rhodobacter.sphaeroides.ATCC.17029 GCA_000015985.1_ASM1598v1
Paracoccus.denitrificans GCA_004063735.1_ASM406373v1
Neomegalonema.perideroedes.DSM.15528 GCA_000374145.1_ASM37414v1
Chelativorans.oligotrophicus GCA_011317505.1_ASM1131750v1
Ahrensia.kielensis.DSM.5890 GCA_000374465.1_ASM37446v1
Rhizobium.leguminosarum.bv.trifolii GCA_011604525.1_ASM1160452v1
Bartonella.quintana GCA_009936175.1_ASM993617v1
Pelagibacterium.halotolerans GCA_013004005.1_ASM1300400v1
Roseibium.hamelinense GCA_007830545.1_ASM783054v1
Polymorphum.gilvum.SL003B.26A1 GCA_000192745.1_ASM19274v1
Pseudovibrio.sp.FO.BEG1 GCA_000236645.1_ASM23664v1
Methylocystis.bryophila GCA_002117405.1_ASM211740v1
Methylocella.silvestris.BL2 GCA_000021745.1_ASM2174v1
Methylorubrum.extorquens.PA1 GCA_000018845.1_ASM1884v1
Xanthobacter.autotrophicus.Py2 GCA_000017645.1_ASM1764v1
Rhodopseudomonas.palustris GCA_013415845.1_ASM1341584v1
Rhodomicrobium.vannielii.ATCC.17100 GCA_000166055.1_ASM16605v1
Hyphomicrobium.denitrificans.ATCC.51888 GCA_000143145.1_ASM14314v1
Thermopetrobacter.sp.TC1 GCA_000746275.1_ASM74627v1
alpha.proteobacterium.IMCC14465 GCA_000293845.2_ASM29384v2
Candidatus.Micropelagos.thuwalensis GCA_000469155.1.RS24
Parvibaculum.lavamentivorans.DS.1 GCA_000017565.1_ASM1756v1
alpha.proteobacterium.HIMB5 GCA_000299095.1_ASM29909v1
Candidatus.Pelagibacter.ubique.HTCC1062 GCA_000012345.1_ASM1234v1
Candidatus.Pelagibacter.ubique.HIMB058 GCA_000419545.1_ASM41954v1
alpha.proteobacterium.HIMB114 GCA_000163555.2
Sphingopyxis.macrogoltabida GCA_001314325.1_ASM131432v1
Citromicrobium.sp.JL477 GCA_001304795.1_ASM130479v1
Sphingobium.indicum.B90A GCA_000264945.2_ASM26494v2
Zymomonas.mobilis.subsp.mobilis.ATCC.31821 GCA_000007105.1_ASM710v1
Sphingomonas.wittichii.RW1 GCA_000016765.1_ASM1676v1
alpha.proteobacterium.AAP81b GCA_001295935.1_ASM129593v1
Kordiimonas.gwangyangensis.DSM.19435 GCA_000375545.1_ASM37554v1
Sneathiella.glossodoripedis.JCM.23214 GCA_000616095.1_ASM61609v1
GCA_003444235.1_ASM344423v1*
GCA_002711555.1_ASM271155v1*
GCA_002724395.1_ASM272439v1*
GCA_002716125.1_ASM271612v1*
GCA_002434595.1_ASM243459v1*
GCA_002717185.1_ASM271718v1*
GCA_006844765.1_ASM684476v1*
GCA_002709955.1_ASM270995v1*
GCF_004005845.1_ASM400584v1*
GCF_003385955.1_ASM338595v1*
Rhodovibrio.salinarum.DSM.9154 GCA_000515255.1_ASM51525v1
Kiloniella.laminariae.DSM.19542 GCA_000374005.1_ASM37400v1
alpha.proteobacterium.BAL199 GCA_000171835.1_ASM17183v1
Thalassobaculum.salexigens.DSM.19539 GCA_000423805.1_ASM42380v1
Candidatus.Puniceispirillum.marinum.IMCC1322 GCA_000024465.1_ASM2446v1
Oceanibaculum.indicum GCA_003633955.1_ASM363395v1
Rhodospirillum.centenum GCA_014197805.1_ASM1419780v1
Inquilinus.limosus.DSM.16000 GCA_000423185.1_ASM42318v1
Micavibrio.aeruginosavorus.ARL.13 GCA_000226315.1_ASM22631v1
Rhodospirillum.rubrum.F11 GCA_000225955.1_ASM22595v1
Pararhodospirillum.photometricum.DSM.122 GCA_000284415.2_ASM28441v2
Roseospirillum.parvum GCA_900100455.1
Caenispirillum.salinarum.AK4 GCA_000315795.1
Magnetospirillum.magneticum.AMB.1 GCA_000009985.1_ASM998v1
Thalassospira.profundimaris.WP0211 GCA_000300275.1_ASM30027v1
Terasakiella.pusilla.DSM.6293 GCA_000688235.1_ASM68823v1
Gluconobacter.oxydans.DSM.3504 GCA_000583855.1_ASM58385v1
Acetobacter.aceti.NBRC.14818 GCA_000193495.2_ASM19349v2
Gluconacetobacter.diazotrophicus.PA1.5 GCA_000067045.1_ASM6704v1
Commensalibacter.intestini.A911 GCA_000231445.2_ASM23144v1
Granulibacter.bethesdensis.CGDNIH4 GCA_000576145.2_ASM57614v2
Acidisphaera.rubrifaciens.HS.AP3 GCA_000964365.1_ASM96436v1
Acidiphilium.multivorum.AIU301 GCA_000202835.1_ASM20283v1
Rubritepida.flocculans.DSM.14296 GCA_000425365.1_ASM42536v1
Belnapia.moabensis.DSM.16746 GCA_000745835.1_ASM74583v1
Roseomonas.gilardii GCA_001941945.1_ASM194194v1
Elioraea.tepidiphila.DSM.17972 GCA_000378465.1_ASM37846v1
Candidatus.Paracaedibacter.acanthamoebae GCA_000742835.1_ASM74283v1
Candidatus.Odyssella.thessalonicensis.L13 GCA_000190415.2_ASM19041v1
Candidatus.Finniella.inopinata GCA_004210305.1_ASM421030v1
Holospora.undulata.HU1 GCA_000388175.3_ASM38817v3
Candidatus.Hepatobacter.penaei GCA_000742475.1_ASM74247v1
Candidatus.Caedibacter.acanthamoebae GCA_000743035.1_ASM74303v1
Caedimonas.varicaedens GCA_001192655.1_ASM119265v1
Candidatus.Nucleicultrix.amoebiphila.FS5 GCA_002117145.1_ASM211714v1
Tistrella.mobilis.KA081020.065 GCA_000264455.2_ASM26445v2
Geminicoccus.roseus.DSM.18922 GCA_000427665.1_ASM42766v1
Candidatus.Neoehrlichia.lotoris.str.RAC413 GCA_000964795.1_ASM96479v1
Anaplasma.phagocytophilum.str.HZ GCA_000013125.1_ASM1312v1
Ehrlichia.ruminantium.str.Welgevonden GCA_000026005.1_ASM2600v1
Wolbachia.endosymbiont.of.Drosophila.melanogaster GCA_000008025.1_ASM802v1
Candidatus.Deianiraea.vastatrix GCA_007993655.1_ASM799365v1
Rickettsiales.endosymbiont.of.Trichoplax.sp.H2 GCA_009690945.1
Candidatus.Midichloria.mitochondrii.IricVA GCA_000219355.1_ASM21935v1
Rickettsiales.endosymbiont.of.Peranema.trichophorum GCA_004210275.1_ASM421027v1
Candidatus.Jidaibacter.acanthamoeba GCA_000815465.1.UWC36
Rickettsia.bellii.RML369.C GCA_000012385.1_ASM1238v1
Candidatus.Megaira.polyxenophila
Rickettsiales.bacterium.Ac37b GCA_000746585.2_ASM74658v2
Rickettsiales.endosymbiont.of.Stachyamoeba.lipophora GCA_003932735.1_ASM393273v1
Magnetofaba.australis.IT.1 GCA_002109495.1_ASM210949v1
Magnetococcus.marinus.MC.1 GCA_000014865.1_ASM1486v1
Nitrosospira.briensis.C.128 GCA_000619905.2_ASM61990v2
Methylotenera.versatilis.301 GCA_000093025.1_ASM9302v1
Ralstonia.pickettii GCA_902374465.1.MGYG.HGUT.01384
Burkholderia.cepacia GCA_009586235.1_ASM958623v1
Dasania.marina.DSM.21967 GCA_000373485.1_ASM37348v1
Congregibacter.litoralis.KT71 GCA_000153125.2_ASM15312v2
Enterobacter.cloacae GCA_000770155.1_ASM77015v1
Alteromonas.macleodii.ATCC.27126 GCA_000172635.2_ASM17263v2
0.501777

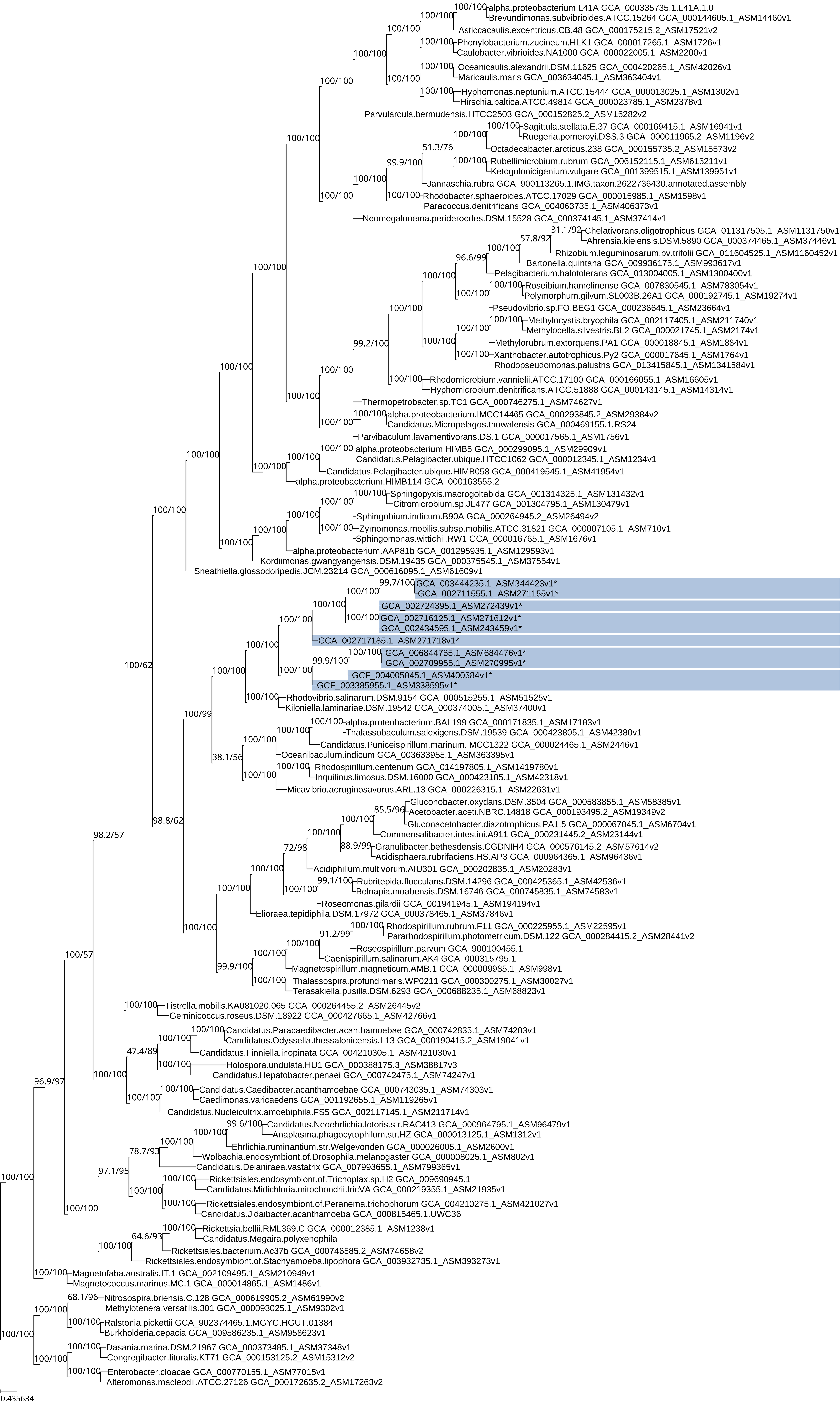
alpha.proteobacterium.L41A GCA_000335735.1_L41A.1.0
Brevundimonas.subvibrioides.ATCC.15264 GCA_000144605.1_ASM14460v1
Asticcacaulis.excentricus.CB.48 GCA_000175215.2_ASM17521v2
Phenylobacterium.zucineum.HLK1 GCA_000017265.1_ASM1726v1
Caulobacter.vibrioides.NA1000 GCA_000022005.1_ASM2200v1
Oceanicaulis.alexandrii.DSM.11625 GCA_000420265.1_ASM42026v1
Maricaulis.maris GCA_003634045.1_ASM363404v1
Hyphomonas.neptunium.ATCC.15444 GCA_000013025.1_ASM1302v1
Hirschia.baltica.ATCC.49814 GCA_000023785.1_ASM2378v1
Parvularcula.bermudensis.HTCC2503 GCA_000152825.2_ASM15282v2
Sagittula.stellata.E.37 GCA_000169415.1_ASM16941v1
Ruegeria.pomeroyi.DSS.3 GCA_000011965.2_ASM1196v2
Octadecabacter.arcticus.238 GCA_000155735.2_ASM15573v2
Rubellimicrobium.rubrum GCA_006152115.1_ASM615211v1
Ketogulonicigenium.vulgare GCA_001399515.1_ASM139951v1
Jannaschia.rubra GCA_900113265.1.IMG.taxon.2622736430.annotated.assembly
Rhodobacter.sphaeroides.ATCC.17029 GCA_000015985.1_ASM1598v1
Paracoccus.denitrificans GCA_004063735.1_ASM406373v1
Neomegalonema.perideroedes.DSM.15528 GCA_000374145.1_ASM37414v1
Chelativorans.oligotrophicus GCA_011317505.1_ASM1131750v1
Ahrensia.kielensis.DSM.5890 GCA_000374465.1_ASM37446v1
Rhizobium.leguminosarum.bv.trifolii GCA_011604525.1_ASM1160452v1
Bartonella.quintana GCA_009936175.1_ASM993617v1
Pelagibacterium.halotolerans GCA_013004005.1_ASM1300400v1
Roseibium.hamelinense GCA_007830545.1_ASM783054v1
Polymorphum.gilvum.SL003B.26A1 GCA_000192745.1_ASM19274v1
Pseudovibrio.sp.FO.BEG1 GCA_000236645.1_ASM23664v1
Methylocystis.bryophila GCA_002117405.1_ASM211740v1
Methylocella.silvestris.BL2 GCA_000021745.1_ASM2174v1
Methylorubrum.extorquens.PA1 GCA_000018845.1_ASM1884v1
Xanthobacter.autotrophicus.Py2 GCA_000017645.1_ASM1764v1
Rhodopseudomonas.palustris GCA_013415845.1_ASM1341584v1
Rhodomicrobium.vannielii.ATCC.17100 GCA_000166055.1_ASM16605v1
Hyphomicrobium.denitrificans.ATCC.51888 GCA_000143145.1_ASM14314v1
Thermopetrobacter.sp.TC1 GCA_000746275.1_ASM74627v1
alpha.proteobacterium.IMCC14465 GCA_000293845.2_ASM29384v2
Candidatus.Micropelagos.thuwalensis GCA_000469155.1.RS24
Parvibaculum.lavamentivorans.DS.1 GCA_000017565.1_ASM1756v1
alpha.proteobacterium.HIMB5 GCA_000299095.1_ASM29909v1
Candidatus.Pelagibacter.ubique.HTCC1062 GCA_000012345.1_ASM1234v1
Candidatus.Pelagibacter.ubique.HIMB058 GCA_000419545.1_ASM41954v1
alpha.proteobacterium.HIMB114 GCA_000163555.2
Sphingopyxis.macrogoltabida GCA_001314325.1_ASM131432v1
Citromicrobium.sp.JL477 GCA_001304795.1_ASM130479v1
Sphingobium.indicum.B90A GCA_000264945.2_ASM26494v2
Zymomonas.mobilis.subsp.mobilis.ATCC.31821 GCA_000007105.1_ASM710v1
Sphingomonas.wittichii.RW1 GCA_000016765.1_ASM1676v1
alpha.proteobacterium.AAP81b GCA_001295935.1_ASM129593v1
Kordiimonas.gwangyangensis.DSM.19435 GCA_000375545.1_ASM37554v1
Sneathiella.glossodoripedis.JCM.23214 GCA_000616095.1_ASM61609v1
GCA_003444235.1_ASM344423v1*
GCA_002711555.1_ASM271155v1*
GCA_002724395.1_ASM272439v1*
GCA_002716125.1_ASM271612v1*
GCA_002434595.1_ASM243459v1*
GCA_002717185.1_ASM271718v1*
GCA_006844765.1_ASM684476v1*
GCA_002709955.1_ASM270995v1*
GCF_004005845.1_ASM400584v1*
GCF_003385955.1_ASM338595v1*
Rhodovibrio.salinarum.DSM.9154 GCA_000515255.1_ASM51525v1
Kiloniella.laminariae.DSM.19542 GCA_000374005.1_ASM37400v1
alpha.proteobacterium.BAL199 GCA_000171835.1_ASM17183v1
Thalassobaculum.salexigens.DSM.19539 GCA_000423805.1_ASM42380v1
Candidatus.Puniceispirillum.marinum.IMCC1322 GCA_000024465.1_ASM2446v1
Oceanibaculum.indicum GCA_003633955.1_ASM363395v1
Rhodospirillum.centenum GCA_014197805.1_ASM1419780v1
Inquilinus.limosus.DSM.16000 GCA_000423185.1_ASM42318v1
Micavibrio.aeruginosavorus.ARL.13 GCA_000226315.1_ASM22631v1
Gluconobacter.oxydans.DSM.3504 GCA_000583855.1_ASM58385v1
Acetobacter.aceti.NBRC.14818 GCA_000193495.2_ASM19349v2
Gluconacetobacter.diazotrophicus.PA1.5 GCA_000067045.1_ASM6704v1
Commensalibacter.intestini.A911 GCA_000231445.2_ASM23144v1
Granulibacter.bethesdensis.CGDNIH4 GCA_000576145.2_ASM57614v2
Acidisphaera.rubrifaciens.HS.AP3 GCA_000964365.1_ASM96436v1
Acidiphilium.multivorum.AIU301 GCA_000202835.1_ASM20283v1
Rubritepida.flocculans.DSM.14296 GCA_000425365.1_ASM42536v1
Belnapia.moabensis.DSM.16746 GCA_000745835.1_ASM74583v1
Roseomonas.gilardii GCA_001941945.1_ASM194194v1
Elioraea.tepidiphila.DSM.17972 GCA_000378465.1_ASM37846v1
Rhodospirillum.rubrum.F11 GCA_000225955.1_ASM22595v1
Pararhodospirillum.photometricum.DSM.122 GCA_000284415.2_ASM28441v2
Roseospirillum.parvum GCA_900100455.1
Caenispirillum.salinarum.AK4 GCA_000315795.1
Magnetospirillum.magneticum.AMB.1 GCA_000009985.1_ASM998v1
Thalassospira.profundimaris.WP0211 GCA_000300275.1_ASM30027v1
Terasakiella.pusilla.DSM.6293 GCA_000688235.1_ASM68823v1
Tistrella.mobilis.KA081020.065 GCA_000264455.2_ASM26445v2
Geminicoccus.roseus.DSM.18922 GCA_000427665.1_ASM42766v1
Candidatus.Paracaedibacter.acanthamoebae GCA_000742835.1_ASM74283v1
Candidatus.Odyssella.thessalonicensis.L13 GCA_000190415.2_ASM19041v1
Candidatus.Finniella.inopinata GCA_004210305.1_ASM421030v1
Holospora.undulata.HU1 GCA_000388175.3_ASM38817v3
Candidatus.Hepatobacter.penaei GCA_000742475.1_ASM74247v1
Candidatus.Caedibacter.acanthamoebae GCA_000743035.1_ASM74303v1
Caedimonas.varicaedens GCA_001192655.1_ASM119265v1
Candidatus.Nucleicultrix.amoebiphila.FS5 GCA_002117145.1_ASM211714v1
Candidatus.Neoehrlichia.lotoris.str.RAC413 GCA_000964795.1_ASM96479v1
Anaplasma.phagocytophilum.str.HZ GCA_000013125.1_ASM1312v1
Ehrlichia.ruminantium.str.Welgevonden GCA_000026005.1_ASM2600v1
Wolbachia.endosymbiont.of.Drosophila.melanogaster GCA_000008025.1_ASM802v1
Candidatus.Deianiraea.vastatrix GCA_007993655.1_ASM799365v1
Rickettsiales.endosymbiont.of.Trichoplax.sp.H2 GCA_009690945.1
Candidatus.Midichloria.mitochondrii.IricVA GCA_000219355.1_ASM21935v1
Rickettsiales.endosymbiont.of.Peranema.trichophorum GCA_004210275.1_ASM421027v1
Candidatus.Jidaibacter.acanthamoeba GCA_000815465.1.UWC36
Rickettsia.bellii.RML369.C GCA_000012385.1_ASM1238v1
Candidatus.Megaira.polyxenophila
Rickettsiales.bacterium.Ac37b GCA_000746585.2_ASM74658v2
Rickettsiales.endosymbiont.of.Stachyamoeba.lipophora GCA_003932735.1_ASM393273v1
Magnetofaba.australis.IT.1 GCA_002109495.1_ASM210949v1
Magnetococcus.marinus.MC.1 GCA_000014865.1_ASM1486v1
Nitrosospira.briensis.C.128 GCA_000619905.2_ASM61990v2
Methylotenera.versatilis.301 GCA_000093025.1_ASM9302v1
Ralstonia.pickettii GCA_902374465.1.MGYG.HGUT.01384
Burkholderia.cepacia GCA_009586235.1_ASM958623v1
Dasania.marina.DSM.21967 GCA_000373485.1_ASM37348v1
Congregibacter.litoralis.KT71 GCA_000153125.2_ASM15312v2
Enterobacter.cloacae GCA_000770155.1_ASM77015v1
Alteromonas.macleodii.ATCC.27126 GCA_000172635.2_ASM17263v2
0.435634

Group D

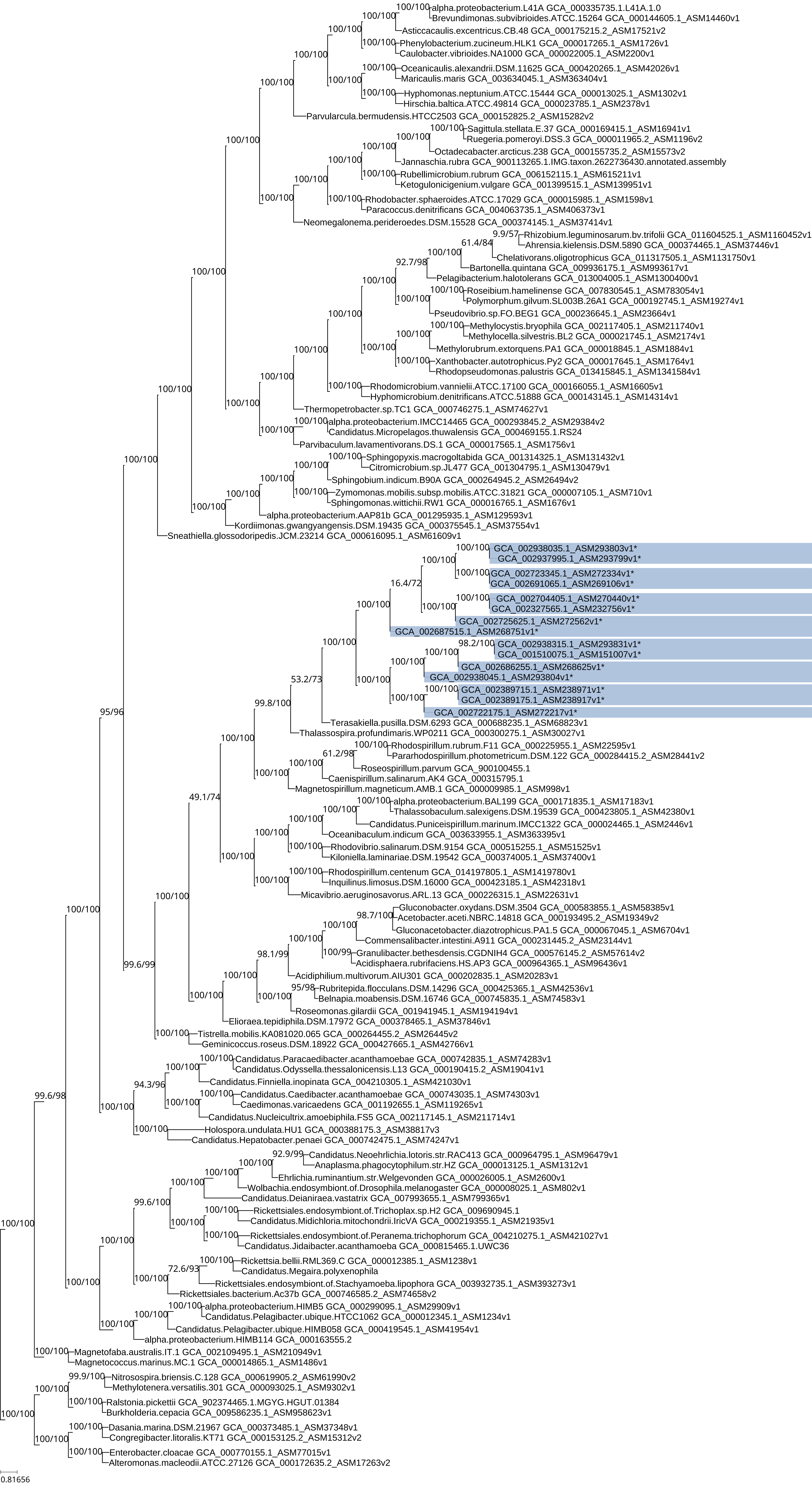
alpha.proteobacterium.L41A GCA_000335735.1_L41A.1.0
Brevundimonas.subvibrioides.ATCC.15264 GCA_000144605.1_ASM14460v1
Asticcacaulis.excentricus.CB.48 GCA_000175215.2_ASM17521v2
Phenylobacterium.zucineum.HLK1 GCA_000017265.1_ASM1726v1
Caulobacter.vibrioides.NA1000 GCA_000022005.1_ASM2200v1
Oceanicaulis.alexandrii.DSM.11625 GCA_000420265.1_ASM42026v1
Maricaulis.maris GCA_003634045.1_ASM363404v1
Hyphomonas.neptunium.ATCC.15444 GCA_000013025.1_ASM1302v1
Hirschia.baltica.ATCC.49814 GCA_000023785.1_ASM2378v1
Parvularcula.bermudensis.HTCC2503 GCA_000152825.2_ASM15282v2
Sagittula.stellata.E.37 GCA_000169415.1_ASM16941v1
Ruegeria.pomeroyi.DSS.3 GCA_000011965.2_ASM1196v2
Octadecabacter.arcticus.238 GCA_000155735.2_ASM15573v2
Jannaschia.rubra GCA_900113265.1.IMG.taxon.2622736430.annotated.assembly
Rubellimicrobium.rubrum GCA_006152115.1_ASM615211v1
Ketogulonicigenium.vulgare GCA_001399515.1_ASM139951v1
Rhodobacter.sphaeroides.ATCC.17029 GCA_000015985.1_ASM1598v1
Paracoccus.denitrificans GCA_004063735.1_ASM406373v1
Neomegalonema.perideroedes.DSM.15528 GCA_000374145.1_ASM37414v1
Rhizobium.leguminosarum.bv.trifolii GCA_011604525.1_ASM1160452v1
Ahrensia.kielensis.DSM.5890 GCA_000374465.1_ASM37446v1
Chelativorans.oligotrophicus GCA_011317505.1_ASM1131750v1
Bartonella.quintana GCA_009936175.1_ASM993617v1
Pelagibacterium.halotolerans GCA_013004005.1_ASM1300400v1
Roseibium.hamelinense GCA_007830545.1_ASM783054v1
Polymorphum.gilvum.SL003B.26A1 GCA_000192745.1_ASM19274v1
Pseudovibrio.sp.FO.BEG1 GCA_000236645.1_ASM23664v1
Methylocystis.bryophila GCA_002117405.1_ASM211740v1
Methylocella.silvestris.BL2 GCA_000021745.1_ASM2174v1
Methylorubrum.extorquens.PA1 GCA_000018845.1_ASM1884v1
Xanthobacter.autotrophicus.Py2 GCA_000017645.1_ASM1764v1
Rhodopseudomonas.palustris GCA_013415845.1_ASM1341584v1
Rhodomicrobium.vannielii.ATCC.17100 GCA_000166055.1_ASM16605v1
Hyphomicrobium.denitrificans.ATCC.51888 GCA_000143145.1_ASM14314v1
Thermopetrobacter.sp.TC1 GCA_000746275.1_ASM74627v1
alpha.proteobacterium.IMCC14465 GCA_000293845.2_ASM29384v2
Candidatus.Micropelagos.thuwalensis GCA_000469155.1.RS24
Parvibaculum.lavamentivorans.DS.1 GCA_000017565.1_ASM1756v1
Sphingopyxis.macrogoltabida GCA_001314325.1_ASM131432v1
Citromicrobium.sp.JL477 GCA_001304795.1_ASM130479v1
Sphingobium.indicum.B90A GCA_000264945.2_ASM26494v2
Zymomonas.mobilis.subsp.mobilis.ATCC.31821 GCA_000007105.1_ASM710v1
Sphingomonas.wittichii.RW1 GCA_000016765.1_ASM1676v1
alpha.proteobacterium.AAP81b GCA_001295935.1_ASM129593v1
Kordiimonas.gwangyangensis.DSM.19435 GCA_000375545.1_ASM37554v1
Sneathiella.glossodoripedis.JCM.23214 GCA_000616095.1_ASM61609v1
GCA_002938035.1_ASM293803v1*
GCA_002937995.1_ASM293799v1*
GCA_002723345.1_ASM272334v1*
GCA_002691065.1_ASM269106v1*
GCA_002704405.1_ASM270440v1*
GCA_002327565.1_ASM232756v1*
GCA_002725625.1_ASM272562v1*
GCA_002687515.1_ASM268751v1*
GCA_002938315.1_ASM293831v1*
GCA_001510075.1_ASM151007v1*
GCA_002686255.1_ASM268625v1*
GCA_002938045.1_ASM293804v1*
GCA_002389715.1_ASM238971v1*
GCA_002389175.1_ASM238917v1*
GCA_002722175.1_ASM272217v1*
Terasakiella.pusilla.DSM.6293 GCA_000688235.1_ASM68823v1
Thalassospira.profundimaris.WP0211 GCA_000300275.1_ASM30027v1
Rhodospirillum.rubrum.F11 GCA_000225955.1_ASM22595v1
Pararhodospirillum.photometricum.DSM.122 GCA_000284415.2_ASM28441v2
Roseospirillum.parvum GCA_900100455.1
Caenispirillum.salinarum.AK4 GCA_000315795.1
Magnetospirillum.magneticum.AMB.1 GCA_000009985.1_ASM998v1
alpha.proteobacterium.BAL199 GCA_000171835.1_ASM17183v1
Thalassobaculum.salexigens.DSM.19539 GCA_000423805.1_ASM42380v1
Candidatus.Puniceispirillum.marinum.IMCC1322 GCA_000024465.1_ASM2446v1
Oceanibaculum.indicum GCA_003633955.1_ASM363395v1
Rhodovibrio.salinarum.DSM.9154 GCA_000515255.1_ASM51525v1
Kiloniella.laminariae.DSM.19542 GCA_000374005.1_ASM37400v1
Rhodospirillum.centenum GCA_014197805.1_ASM1419780v1
Inquilinus.limosus.DSM.16000 GCA_000423185.1_ASM42318v1
Micavibrio.aeruginosavorus.ARL.13 GCA_000226315.1_ASM22631v1
Gluconobacter.oxydans.DSM.3504 GCA_000583855.1_ASM58385v1
Acetobacter.aceti.NBRC.14818 GCA_000193495.2_ASM19349v2
Gluconacetobacter.diazotrophicus.PA1.5 GCA_000067045.1_ASM6704v1
Commensalibacter.intestini.A911 GCA_000231445.2_ASM23144v1
Granulibacter.bethesdensis.CGDNIH4 GCA_000576145.2_ASM57614v2
Acidisphaera.rubrifaciens.HS.AP3 GCA_000964365.1_ASM96436v1
Acidiphilium.multivorum.AIU301 GCA_000202835.1_ASM20283v1
Rubritepida.flocculans.DSM.14296 GCA_000425365.1_ASM42536v1
Belnapia.moabensis.DSM.16746 GCA_000745835.1_ASM74583v1
Roseomonas.gilardii GCA_001941945.1_ASM194194v1
Elioraea.tepidiphila.DSM.17972 GCA_000378465.1_ASM37846v1
Tistrella.mobilis.KA081020.065 GCA_000264455.2_ASM26445v2
Geminicoccus.roseus.DSM.18922 GCA_000427665.1_ASM42766v1
Candidatus.Paracaedibacter.acanthamoebae GCA_000742835.1_ASM74283v1
Candidatus.Odyssella.thessalonicensis.L13 GCA_000190415.2_ASM19041v1
Candidatus.Finniella.inopinata GCA_004210305.1_ASM421030v1
Candidatus.Caedibacter.acanthamoebae GCA_000743035.1_ASM74303v1
Caedimonas.varicaedens GCA_001192655.1_ASM119265v1
Candidatus.Nucleicultrix.amoebiphila.FS5 GCA_002117145.1_ASM211714v1
Holospora.undulata.HU1 GCA_000388175.3_ASM38817v3
Candidatus.Hepatobacter.penaei GCA_000742475.1_ASM74247v1
Candidatus.Neoehrlichia.lotoris.str.RAC413 GCA_000964795.1_ASM96479v1
Anaplasma.phagocytophilum.str.HZ GCA_000013125.1_ASM1312v1
Ehrlichia.ruminantium.str.Welgevonden GCA_000026005.1_ASM2600v1
Wolbachia.endosymbiont.of.Drosophila.melanogaster GCA_000008025.1_ASM802v1
Candidatus.Deianiraea.vastatrix GCA_007993655.1_ASM799365v1
Rickettsiales.endosymbiont.of.Trichoplax.sp.H2 GCA_009690945.1
Candidatus.Midichloria.mitochondrii.IricVA GCA_000219355.1_ASM21935v1
Rickettsiales.endosymbiont.of.Peranema.trichophorum GCA_004210275.1_ASM421027v1
Candidatus.Jidaibacter.acanthamoeba GCA_000815465.1.UWC36
Rickettsia.bellii.RML369.C GCA_000012385.1_ASM1238v1
Candidatus.Megaira.polyxenophila
Rickettsiales.endosymbiont.of.Stachyamoeba.lipophora GCA_003932735.1_ASM393273v1
Rickettsiales.bacterium.Ac37b GCA_000746585.2_ASM74658v2
alpha.proteobacterium.HIMB5 GCA_000299095.1_ASM29909v1
Candidatus.Pelagibacter.ubique.HTCC1062 GCA_000012345.1_ASM1234v1
Candidatus.Pelagibacter.ubique.HIMB058 GCA_000419545.1_ASM41954v1
alpha.proteobacterium.HIMB114 GCA_000163555.2
Magnetofaba.australis.IT.1 GCA_002109495.1_ASM210949v1
Magnetococcus.marinus.MC.1 GCA_000014865.1_ASM1486v1
Nitrosospira.briensis.C.128 GCA_000619905.2_ASM61990v2
Methylotenera.versatilis.301 GCA_000093025.1_ASM9302v1
Ralstonia.pickettii GCA_902374465.1.MGYG.HGUT.01384
Burkholderia.cepacia GCA_009586235.1_ASM958623v1
Dasania.marina.DSM.21967 GCA_000373485.1_ASM37348v1
Congregibacter.litoralis.KT71 GCA_000153125.2_ASM15312v2
Enterobacter.cloacae GCA_000770155.1_ASM77015v1
Alteromonas.macleodii.ATCC.27126 GCA_000172635.2_ASM17263v2
0.81656

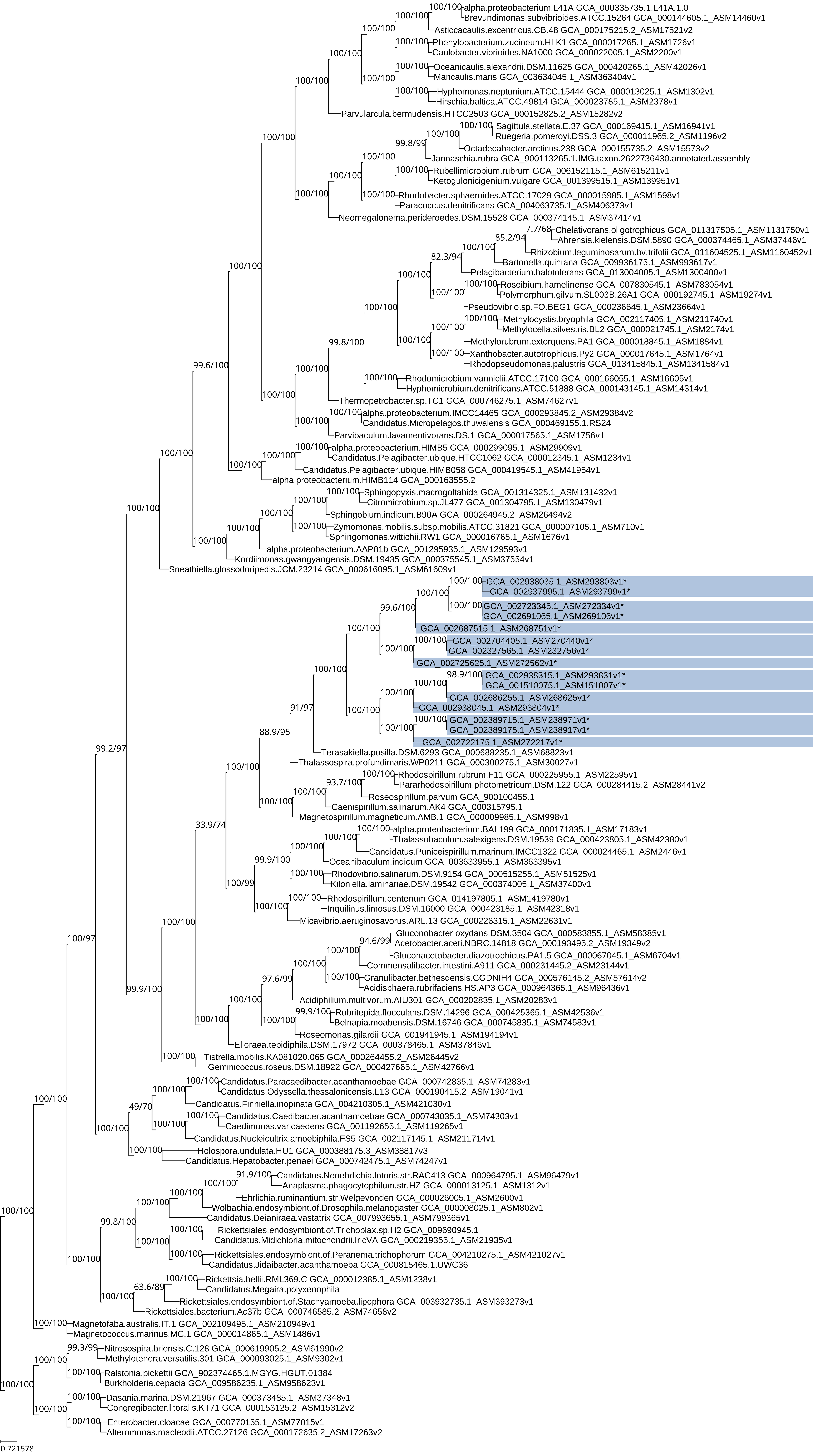

100/100
alpha.proteobacterium.L41A GCA_000335735.1.L41A.1.0
Brevundimonas.subvibrioides.ATCC.15264 GCA_000144605.1_ASM14460v1
100/100
Asticcacaulis.excentricus.CB.48 GCA_000175215.2_ASM17521v2
100/100
100/100
Phenylobacterium.zucineum.HLK1 GCA_000017265.1_ASM1726v1
Caulobacter.vibrioides.NA1000 GCA_000022005.1_ASM2200v1
100/100
100/100
Oceanicaulis.alexandrii.DSM.11625 GCA_000420265.1_ASM42026v1
Maricaulis.maris GCA_003634045.1_ASM363404v1
100/100
100/100
100/100
Hyphomonas.neptunium.ATCC.15444 GCA_000013025.1_ASM1302v1
Hirschia.baltica.ATCC.49814 GCA_000023785.1_ASM2378v1
Parvularcula.bermudensis.HTCC2503 GCA_000152825.2_ASM15282v2
100/100
Sagittula.stellata.E.37 GCA_000169415.1_ASM16941v1
100/100
Ruegeria.pomeroyi.DSS.3 GCA_000011965.2_ASM1196v2
100/100
99.8/99
Octadecabacter.arcticus.238 GCA_000155735.2_ASM15573v2
Jannaschia.rubra GCA_900113265.1.IMG.taxon.2622736430.annotated.assembly
100/100
100/100
Rubellimicrobium.rubrum GCA_006152115.1_ASM615211v1
100/100
Ketogulonicigenium.vulgare GCA_001399515.1_ASM139951v1
100/100
100/100
Rhodobacter.sphaeroides.ATCC.17029 GCA_000015985.1_ASM1598v1
Paracoccus.denitrificans GCA_004063735.1_ASM406373v1
Neomegalonema.perideroedes.DSM.15528 GCA_000374145.1_ASM37414v1
7.7/68
Chelativorans.oligotrophicus GCA_011317505.1_ASM1131750v1
85.2/94
Ahrensia.kielensis.DSM.5890 GCA_000374465.1_ASM37446v1
100/100
Rhizobium.leguminosarum.bv.trifolii GCA_011604525.1_ASM1160452v1
100/100
82.3/94
Bartonella.quintana GCA_009936175.1_ASM993617v1
Pelagibacterium.halotolerans GCA_013004005.1_ASM1300400v1
100/100
100/100
Roseibium.hamelinense GCA_007830545.1_ASM783054v1
100/100
Polymorphum.gilvum.SL003B.26A1 GCA_000192745.1_ASM19274v1
100/100
Pseudovibrio.sp.FO.BEG1 GCA_000236645.1_ASM23664v1
100/100
Methylocystis.bryophila GCA_002117405.1_ASM211740v1
100/100
Methylocella.silvestris.BL2 GCA_000021745.1_ASM2174v1
100/100
Methylorubrum.extorquens.PA1 GCA_000018845.1_ASM1884v1
99.8/100
100/100
Xanthobacter.autotrophicus.Py2 GCA_000017645.1_ASM1764v1
Rhodopseudomonas.palustris GCA_013415845.1_ASM1341584v1
99.6/100
100/100
100/100
Rhodomicrobium.vannielii.ATCC.17100 GCA_000166055.1_ASM16605v1
Hyphomicrobium.denitrificans.ATCC.51888 GCA_000143145.1_ASM14314v1
100/100
Thermopetrobacter.sp.TC1 GCA_000746275.1_ASM74627v1
100/100
alpha.proteobacterium.IMCC14465 GCA_000293845.2_ASM29384v2
100/100
Candidatus.Micropelagos.thuwalensis GCA_000469155.1.RS24
Parvibaculum.lavamentivorans.DS.1 GCA_000017565.1_ASM1756v1
100/100
100/100
alpha.proteobacterium.HIMB5 GCA_000299095.1_ASM29909v1
100/100
Candidatus.Pelagibacter.ubique.HTCC1062 GCA_000012345.1_ASM1234v1
100/100
Candidatus.Pelagibacter.ubique.HIMB058 GCA_000419545.1_ASM41954v1
alpha.proteobacterium.HIMB114 GCA_000163555.2
100/100
Sphingopyxis.macrogoltabida GCA_001314325.1_ASM131432v1
100/100
Citromicrobium.sp.JL477 GCA_001304795.1_ASM130479v1
100/100
100/100
Sphingobium.indicum.B90A GCA_000264945.2_ASM26494v2
100/100
Zymomonas.mobilis.subsp.mobilis.ATCC.31821 GCA_000007105.1_ASM710v1
100/100
Sphingomonas.wittichii.RW1 GCA_000016765.1_ASM1676v1
100/100
alpha.proteobacterium.AAP81b GCA_001295935.1_ASM129593v1
Kordiimonas.gwangyangensis.DSM.19435 GCA_000375545.1_ASM37554v1
Sneathiella.glossodoripedis.JCM.23214 GCA_000616095.1_ASM61609v1
100/100
GCA_002938035.1_ASM293803v1*
100/100
GCA_002937995.1_ASM293799v1*
99.6/100
100/100
GCA_002723345.1_ASM272334v1*
GCA_002691065.1_ASM269106v1*
100/100
GCA_002687515.1_ASM268751v1*
100/100
GCA_002704405.1_ASM270440v1*
100/100
GCA_002327565.1_ASM232756v1*
GCA_002725625.1_ASM272562v1*
100/100
98.9/100
GCA_002938315.1_ASM293831v1*
100/100
GCA_001510075.1_ASM151007v1*
100/100
GCA_002686255.1_ASM268625v1*
91/97
100/100
GCA_002938045.1_ASM293804v1*
100/100
GCA_002389715.1_ASM238971v1*
99.2/97
88.9/95
100/100
GCA_002389175.1_ASM238917v1*
GCA_002722175.1_ASM272217v1*
Terasakiella.pusilla.DSM.6293 GCA_000688235.1_ASM68823v1
Thalassospira.profundimaris.WP0211 GCA_000300275.1_ASM30027v1
100/100
100/100
Rhodospirillum.rubrum.F11 GCA_000225955.1_ASM22595v1
93.7/100
Pararhodospirillum.photometricum.DSM.122 GCA_000284415.2_ASM28441v2
100/100
Roseospirillum.parvum GCA_900100455.1
100/100
Caenispirillum.salinarum.AK4 GCA_000315795.1
Magnetospirillum.magneticum.AMB.1 GCA_000009985.1_ASM998v1
33.9/74
100/100
alpha.proteobacterium.BAL199 GCA_000171835.1_ASM17183v1
100/100
Thalassobaculum.salexigens.DSM.19539 GCA_000423805.1_ASM42380v1
100/100
Candidatus.Puniceispirillum.marinum.IMCC1322 GCA_000024465.1_ASM2446v1
99.9/100
Oceanibaculum.indicum GCA_003633955.1_ASM363395v1
100/100
Rhodovibrio.salinarum.DSM.9154 GCA_000515255.1_ASM51525v1
100/99
Kiloniella.laminariae.DSM.19542 GCA_000374005.1_ASM37400v1
100/100
Rhodospirillum.centenum GCA_014197805.1_ASM1419780v1
100/100
Inquilinus.limosus.DSM.16000 GCA_000423185.1_ASM42318v1
100/100
Micavibrio.aeruginosavorus.ARL.13 GCA_000226315.1_ASM22631v1
Gluconobacter.oxydans.DSM.3504 GCA_000583855.1_ASM58385v1
94.6/99
Acetobacter.aceti.NBRC.14818 GCA_000193495.2_ASM19349v2
100/97
100/100
Gluconacetobacter.diazotrophicus.PA1.5 GCA_000067045.1_ASM6704v1
100/100
Commensalibacter.intestini.A911 GCA_000231445.2_ASM23144v1
100/100
Granulibacter.bethesdensis.CGDNIH4 GCA_000576145.2_ASM57614v2
97.6/99
Acidisphaera.rubrifaciens.HS.AP3 GCA_000964365.1_ASM96436v1
99.9/100
Acidiphilium.multivorum.AIU301 GCA_000202835.1_ASM20283v1
100/100
99.9/100
Rubritepida.flocculans.DSM.14296 GCA_000425365.1_ASM42536v1
100/100
100/100
Belnapia.moabensis.DSM.16746 GCA_000745835.1_ASM74583v1
Roseomonas.gilardii GCA_001941945.1_ASM194194v1
Elioraea.tepidiphila.DSM.17972 GCA_000378465.1_ASM37846v1
100/100
Tistrella.mobilis.KA081020.065 GCA_000264455.2_ASM26445v2
Geminicoccus.roseus.DSM.18922 GCA_000427665.1_ASM42766v1
100/100
Candidatus.Paracaedibacter.acanthamoebae GCA_000742835.1_ASM74283v1
100/100
Candidatus.Odyssella.thessalonicensis.L13 GCA_000190415.2_ASM19041v1
100/100
49/70
Candidatus.Finniella.inopinata GCA_004210305.1_ASM421030v1
100/100
Candidatus.Caedibacter.acanthamoebae GCA_000743035.1_ASM74303v1
100/100
100/100
Caedimonas.varicaedens GCA_001192655.1_ASM119265v1
Candidatus.Nucleicultrix.amoebiphila.FS5 GCA_002117145.1_ASM211714v1
100/100
Holospora.undulata.HU1 GCA_000388175.3_ASM38817v3
Candidatus.Hepatobacter.penaei GCA_000742475.1_ASM74247v1
91.9/100
Candidatus.Neoehrlichia.lotoris.str.RAC413 GCA_000964795.1_ASM96479v1
100/100
Anaplasma.phagocytophilum.str.HZ GCA_000013125.1_ASM1312v1
100/100
Ehrlichia.ruminantium.str.Welgevonden GCA_000026005.1_ASM2600v1
100/100
100/100
Wolbachia.endosymbiont.of.Drosophila.melanogaster GCA_000008025.1_ASM802v1
Candidatus.Deianiraea.vastatrix GCA_007993655.1_ASM799365v1
99.8/100
100/100
Rickettsiales.endosymbiont.of.Trichoplax.sp.H2 GCA_009690945.1
100/100
Candidatus.Midichloria.mitochondrii.IricVA GCA_000219355.1_ASM21935v1
100/100
Rickettsiales.endosymbiont.of.Peranema.trichophorum GCA_004210275.1_ASM421027v1
100/100
Candidatus.Jidaibacter.acanthamoeba GCA_000815465.1.UWC36
100/100
Rickettsia.bellii.RML369.C GCA_000012385.1_ASM1238v1
63.6/89
Candidatus.Megaira.polyxenophila
100/100
Rickettsiales.endosymbiont.of.Stachyamoeba.lipophora GCA_003932735.1_ASM393273v1
Rickettsiales.bacterium.Ac37b GCA_000746585.2_ASM74658v2
100/100
Magnetofaba.australis.IT.1 GCA_002109495.1_ASM210949v1
Magnetococcus.marinus.MC.1 GCA_000014865.1_ASM1486v1
99.3/99
Nitrosospira.briensis.C.128 GCA_000619905.2_ASM61990v2
100/100
Methylotenera.versatilis.301 GCA_000093025.1_ASM9302v1
100/100
Ralstonia.pickettii GCA_902374465.1.MGYG.HGUT.01384
Burkholderia.cepacia GCA_009586235.1_ASM958623v1
100/100
100/100
Dasania.marina.DSM.21967 GCA_000373485.1_ASM37348v1
100/100
Congregibacter.litoralis.KT71 GCA_000153125.2_ASM15312v2
100/100
Enterobacter.cloacae GCA_000770155.1_ASM77015v1
Alteromonas.macleodii.ATCC.27126 GCA_000172635.2_ASM17263v2
0.721578

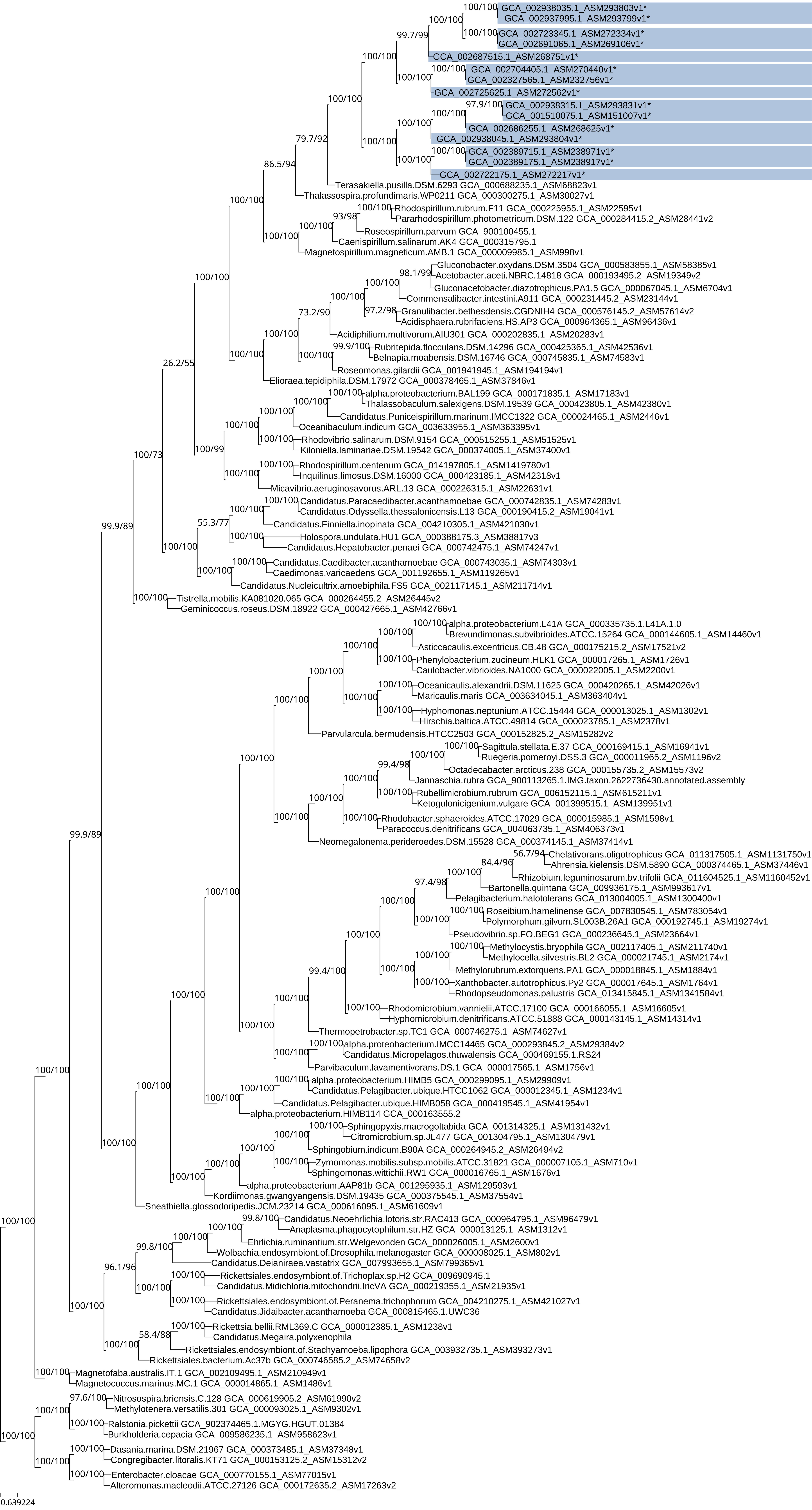
100/100
GCA_002938035.1_ASM293803v1*
GCA_002937995.1_ASM293799v1*
100/100
100/100
GCA_002723345.1_ASM272334v1*
GCA_002691065.1_ASM269106v1*
99.7/99
GCA_002687515.1_ASM268751v1*
100/100
100/100
GCA_002704405.1_ASM270440v1*
GCA_002327565.1_ASM232756v1*
100/100
GCA_002725625.1_ASM272562v1*
100/100
97.9/100
GCA_002938315.1_ASM293831v1*
GCA_001510075.1_ASM151007v1*
100/100
GCA_002686255.1_ASM268625v1*
100/100
GCA_002938045.1_ASM293804v1*
100/100
100/100
GCA_002389715.1_ASM238971v1*
GCA_002389175.1_ASM238917v1*
100/100
GCA_002722175.1_ASM272217v1*
79.7/92
Terasakiella.pusilla.DSM.6293 GCA_000688235.1_ASM68823v1
86.5/94
Thalassospira.profundimaris.WP0211 GCA_000300275.1_ASM30027v1
100/100
100/100
Rhodospirillum.rubrum.F11 GCA_000225955.1_ASM22595v1
Pararhodospirillum.photometricum.DSM.122 GCA_000284415.2_ASM28441v2
93/98
Roseospirillum.parvum GCA_900100455.1
100/100
Caenispirillum.salinarum.AK4 GCA_000315795.1
100/100
Magnetospirillum.magneticum.AMB.1 GCA_000009985.1_ASM998v1
100/100
Gluconobacter.oxydans.DSM.3504 GCA_000583855.1_ASM58385v1
98.1/99
Acetobacter.aceti.NBRC.14818 GCA_000193495.2_ASM19349v2
100/100
Gluconacetobacter.diazotrophicus.PA1.5 GCA_000067045.1_ASM6704v1
Commensalibacter.intestini.A911 GCA_000231445.2_ASM23144v1
100/100
97.2/98
Granulibacter.bethesdensis.CGDNIH4 GCA_000576145.2_ASM57614v2
Acidisphaera.rubrifaciens.HS.AP3 GCA_000964365.1_ASM96436v1
73.2/90
Acidiphilium.multivorum.AIU301 GCA_000202835.1_ASM20283v1
100/100
99.9/100
Rubritepida.flocculans.DSM.14296 GCA_000425365.1_ASM42536v1
Belnapia.moabensis.DSM.16746 GCA_000745835.1_ASM74583v1
100/100
Roseomonas.gilardii GCA_001941945.1_ASM194194v1
100/100
Elioraea.tepidiphila.DSM.17972 GCA_000378465.1_ASM37846v1
26.2/55
100/100
alpha.proteobacterium.BAL199 GCA_000171835.1_ASM17183v1
Thalassobaculum.salexigens.DSM.19539 GCA_000423805.1_ASM42380v1
100/100
Candidatus.Puniceispirillum.marinum.IMCC1322 GCA_000024465.1_ASM2446v1
100/100
Oceanibaculum.indicum GCA_003633955.1_ASM363395v1
100/100
100/100
Rhodovibrio.salinarum.DSM.9154 GCA_000515255.1_ASM51525v1
Kiloniella.laminariae.DSM.19542 GCA_000374005.1_ASM37400v1
100/99
100/100
Rhodospirillum.centenum GCA_014197805.1_ASM1419780v1
Inquilinus.limosus.DSM.16000 GCA_000423185.1_ASM42318v1
100/100
Micavibrio.aeruginosavorus.ARL.13 GCA_000226315.1_ASM22631v1
100/73
100/100
Candidatus.Paracaedibacter.acanthamoebae GCA_000742835.1_ASM74283v1
Candidatus.Odyssella.thessalonicensis.L13 GCA_000190415.2_ASM19041v1
100/100
Candidatus.Finniella.inopinata GCA_004210305.1_ASM421030v1
55.3/77
99.9/89
100/100
Holospora.undulata.HU1 GCA_000388175.3_ASM38817v3
Candidatus.Hepatobacter.penaei GCA_000742475.1_ASM74247v1
100/100
100/100
Candidatus.Caedibacter.acanthamoebae GCA_000743035.1_ASM74303v1
Caedimonas.varicaedens GCA_001192655.1_ASM119265v1
100/100
Candidatus.Nucleicultrix.amoebiphila.FS5 GCA_002117145.1_ASM211714v1
100/100
Tistrella.mobilis.KA081020.065 GCA_000264455.2_ASM26445v2
Geminicoccus.roseus.DSM.18922 GCA_000427665.1_ASM42766v1
100/100
alpha.proteobacterium.L41A GCA_000335735.1.L41A.1.0
100/100
Brevundimonas.subvibrioides.ATCC.15264 GCA_000144605.1_ASM14460v1
100/100
Asticcacaulis.excentricus.CB.48 GCA_000175215.2_ASM17521v2
100/100
Phenylobacterium.zucineum.HLK1 GCA_000017265.1_ASM1726v1
Caulobacter.vibrioides.NA1000 GCA_000022005.1_ASM2200v1
100/100
100/100
Oceanicaulis.alexandrii.DSM.11625 GCA_000420265.1_ASM42026v1
100/100
Maricaulis.maris GCA_003634045.1_ASM363404v1
100/100
100/100
Hyphomonas.neptunium.ATCC.15444 GCA_000013025.1_ASM1302v1
Hirschia.baltica.ATCC.49814 GCA_000023785.1_ASM2378v1
Parvularcula.bermudensis.HTCC2503 GCA_000152825.2_ASM15282v2
100/100
100/100
Sagittula.stellata.E.37 GCA_000169415.1_ASM16941v1
100/100
Ruegeria.pomeroyi.DSS.3 GCA_000011965.2_ASM1196v2
99.4/98
Octadecabacter.arcticus.238 GCA_000155735.2_ASM15573v2
100/100
Jannaschia.rubra GCA_900113265.1.IMG.taxon.2622736430.annotated.assembly
100/100
Rubellimicrobium.rubrum GCA_006152115.1_ASM615211v1
100/100
Ketogulonicigenium.vulgare GCA_001399515.1_ASM139951v1
100/100
100/100
Rhodobacter.sphaeroides.ATCC.17029 GCA_000015985.1_ASM1598v1
Paracoccus.denitrificans GCA_004063735.1_ASM406373v1
Neomegalonema.perideroedes.DSM.15528 GCA_000374145.1_ASM37414v1
99.9/89
56.7/94
Chelativorans.oligotrophicus GCA_011317505.1_ASM1131750v1
84.4/96
Ahrensia.kielensis.DSM.5890 GCA_000374465.1_ASM37446v1
100/100
Rhizobium.leguminosarum.bv.trifolii GCA_011604525.1_ASM1160452v1
97.4/98
Bartonella.quintana GCA_009936175.1_ASM993617v1
100/100
Pelagibacterium.halotolerans GCA_013004005.1_ASM1300400v1
100/100
100/100
Roseibium.hamelinense GCA_007830545.1_ASM783054v1
100/100
Polymorphum.gilvum.SL003B.26A1 GCA_000192745.1_ASM19274v1
Pseudovibrio.sp.FO.BEG1 GCA_000236645.1_ASM23664v1
100/100
100/100
Methylocystis.bryophila GCA_002117405.1_ASM211740v1
100/100
Methylocella.silvestris.BL2 GCA_000021745.1_ASM2174v1
100/100
Methylorubrum.extorquens.PA1 GCA_000018845.1_ASM1884v1
99.4/100
100/100
Xanthobacter.autotrophicus.Py2 GCA_000017645.1_ASM1764v1
Rhodopseudomonas.palustris GCA_013415845.1_ASM1341584v1
100/100
100/100
Rhodomicrobium.vannielii.ATCC.17100 GCA_000166055.1_ASM16605v1
Hyphomicrobium.denitrificans.ATCC.51888 GCA_000143145.1_ASM14314v1
100/100
Thermopetrobacter.sp.TC1 GCA_000746275.1_ASM74627v1
100/100
alpha.proteobacterium.IMCC14465 GCA_000293845.2_ASM29384v2
100/100
Candidatus.Micropelagos.thuwalensis GCA_000469155.1.RS24
Parvibaculum.lavamentivorans.DS.1 GCA_000017565.1_ASM1756v1
100/100
100/100
alpha.proteobacterium.HIMB5 GCA_000299095.1_ASM29909v1
100/100
Candidatus.Pelagibacter.ubique.HTCC1062 GCA_000012345.1_ASM1234v1
100/100
Candidatus.Pelagibacter.ubique.HIMB058 GCA_000419545.1_ASM41954v1
alpha.proteobacterium.HIMB114 GCA_000163555.2
100/100
100/100
Sphingopyxis.macrogoltabida GCA_001314325.1_ASM131432v1
100/100
Citromicrobium.sp.JL477 GCA_001304795.1_ASM130479v1
100/100
Sphingobium.indicum.B90A GCA_000264945.2_ASM26494v2
100/100
100/100
Zymomonas.mobilis.subsp.mobilis.ATCC.31821 GCA_000007105.1_ASM710v1
Sphingomonas.wittichii.RW1 GCA_000016765.1_ASM1676v1
100/100
alpha.proteobacterium.AAP81b GCA_001295935.1_ASM129593v1
Kordiimonas.gwangyangensis.DSM.19435 GCA_000375545.1_ASM37554v1
Sneathiella.glossodoripedis.JCM.23214 GCA_000616095.1_ASM61609v1
100/100
99.8/100
Candidatus.Neoehrlichia.lotoris.str.RAC413 GCA_000964795.1_ASM96479v1
100/100
Anaplasma.phagocytophilum.str.HZ GCA_000013125.1_ASM1312v1
100/100
Ehrlichia.ruminantium.str.Welgevonden GCA_000026005.1_ASM2600v1
99.8/100
Wolbachia.endosymbiont.of.Drosophila.melanogaster GCA_000008025.1_ASM802v1
Candidatus.Deianiraea.vastatrix GCA_007993655.1_ASM799365v1
96.1/96
100/100
Rickettsiales.endosymbiont.of.Trichoplax.sp.H2 GCA_009690945.1
100/100
Candidatus.Midichloria.mitochondrii.IricVA GCA_000219355.1_ASM21935v1
100/100
Rickettsiales.endosymbiont.of.Peranema.trichophorum GCA_004210275.1_ASM421027v1
100/100
Candidatus.Jidaibacter.acanthamoeba GCA_000815465.1.UWC36
100/100
Rickettsia.bellii.RML369.C GCA_000012385.1_ASM1238v1
58.4/88
Candidatus.Megaira.polyxenophila
100/100
Rickettsiales.endosymbiont.of.Stachyamoeba.lipophora GCA_003932735.1_ASM393273v1
Rickettsiales.bacterium.Ac37b GCA_000746585.2_ASM74658v2
100/100
Magnetofaba.australis.IT.1 GCA_002109495.1_ASM210949v1
Magnetococcus.marinus.MC.1 GCA_000014865.1_ASM1486v1
97.6/100
Nitrosospira.briensis.C.128 GCA_000619905.2_ASM61990v2
100/100
Methylotenera.versatilis.301 GCA_000093025.1_ASM9302v1
100/100
Ralstonia.pickettii GCA_902374465.1.MGYG.HGUT.01384
100/100
Burkholderia.cepacia GCA_009586235.1_ASM958623v1
100/100
Dasania.marina.DSM.21967 GCA_000373485.1_ASM37348v1
100/100
Congregibacter.litoralis.KT71 GCA_000153125.2_ASM15312v2
100/100
Enterobacter.cloacae GCA_000770155.1_ASM77015v1
Alteromonas.macleodii.ATCC.27126 GCA_000172635.2_ASM17263v2
0.639224

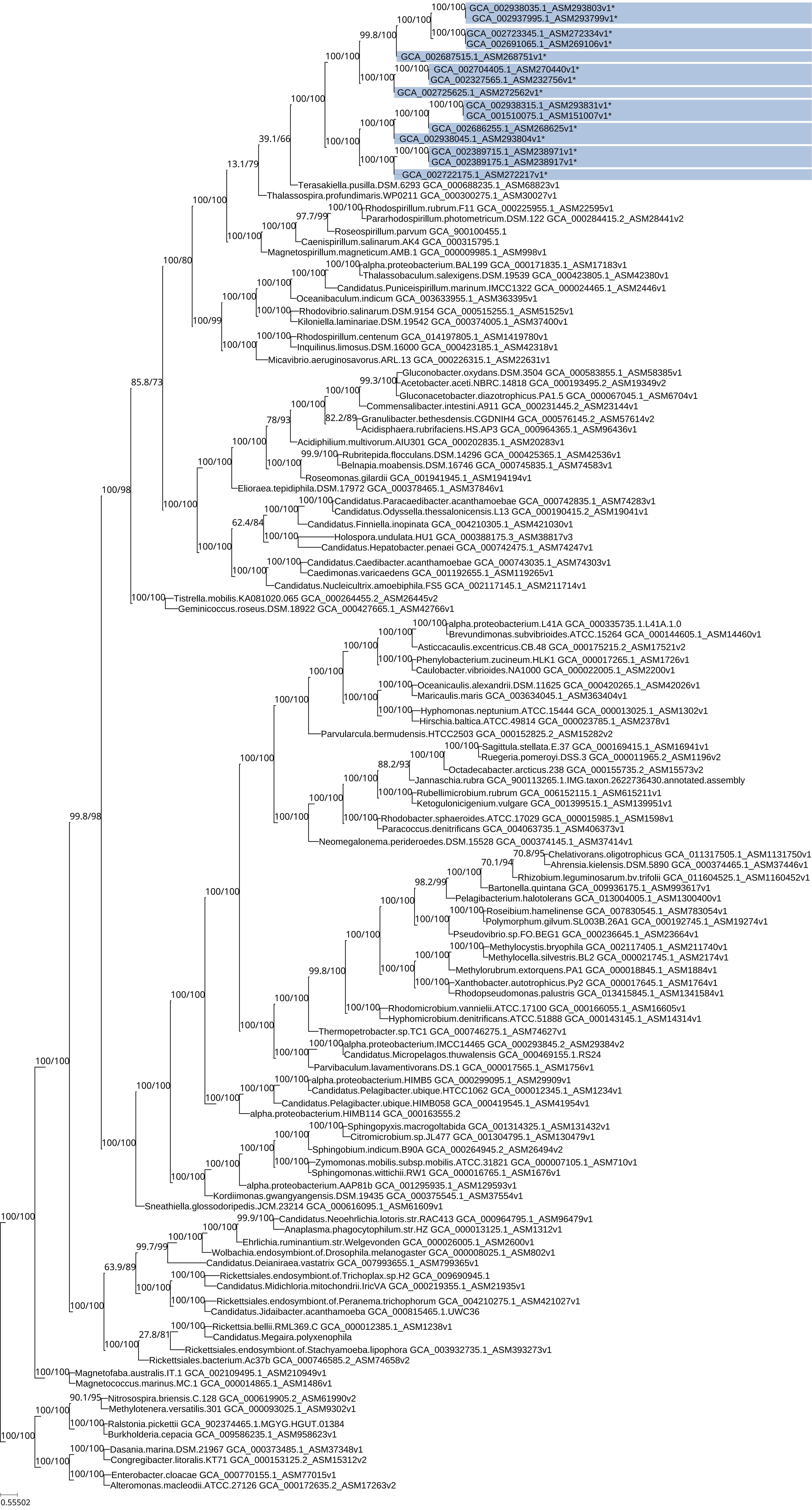
GCA_002938035.1_ASM293803v1*
GCA_002937995.1_ASM293799v1*
GCA_002723345.1_ASM272334v1*
GCA_002691065.1_ASM269106v1*
GCA_002687515.1_ASM268751v1*
GCA_002704405.1_ASM270440v1*
GCA_002327565.1_ASM232756v1*
GCA_002725625.1_ASM272562v1*
GCA_002938315.1_ASM293831v1*
GCA_001510075.1_ASM151007v1*
GCA_002686255.1_ASM268625v1*
GCA_002938045.1_ASM293804v1*
GCA_002389715.1_ASM238971v1*
GCA_002389175.1_ASM238917v1*
GCA_002722175.1_ASM272217v1*
Terasakiella.pusilla.DSM.6293 GCA_000688235.1_ASM68823v1
Thalassospira.profundimaris.WP0211 GCA_000300275.1_ASM30027v1
Rhodospirillum.rubrum.F11 GCA_000225955.1_ASM22595v1
Pararhodospirillum.photometricum.DSM.122 GCA_000284415.2_ASM28441v2
Roseospirillum.parvum GCA_900100455.1
Caenispirillum.salinarum.AK4 GCA_000315795.1
Magnetospirillum.magneticum.AMB.1 GCA_000009985.1_ASM998v1
alpha.proteobacterium.BAL199 GCA_000171835.1_ASM17183v1
Thalassobaculum.salexigens.DSM.19539 GCA_000423805.1_ASM42380v1
Candidatus.Puniceispirillum.marinum.IMCC1322 GCA_000024465.1_ASM2446v1
Oceanibaculum.indicum GCA_003633955.1_ASM363395v1
Rhodovibrio.salinarum.DSM.9154 GCA_000515255.1_ASM51525v1
Kiloniella.laminariae.DSM.19542 GCA_000374005.1_ASM37400v1
Rhodospirillum.centenum GCA_014197805.1_ASM1419780v1
Inquilinus.limosus.DSM.16000 GCA_000423185.1_ASM42318v1
Micavibrio.aeruginosavorus.ARL.13 GCA_000226315.1_ASM22631v1
Gluconobacter.oxydans.DSM.3504 GCA_000583855.1_ASM58385v1
Acetobacter.aceti.NBRC.14818 GCA_000193495.2_ASM19349v2
Gluconacetobacter.diazotrophicus.PA1.5 GCA_000067045.1_ASM6704v1
Commensalibacter.intestini.A911 GCA_000231445.2_ASM23144v1
Granulibacter.bethesdensis.CGDNIH4 GCA_000576145.2_ASM57614v2
Acidisphaera.rubrifaciens.HS.AP3 GCA_000964365.1_ASM96436v1
Acidiphilium.multivorum.AIU301 GCA_000202835.1_ASM20283v1
Rubritepida.flocculans.DSM.14296 GCA_000425365.1_ASM42536v1
Belnapia.moabensis.DSM.16746 GCA_000745835.1_ASM74583v1
Roseomonas.gilardii GCA_001941945.1_ASM194194v1
Elioraea.tepidiphila.DSM.17972 GCA_000378465.1_ASM37846v1
Candidatus.Paracaedibacter.acanthamoebae GCA_000742835.1_ASM74283v1
Candidatus.Odyssella.thessalonicensis.L13 GCA_000190415.2_ASM19041v1
Candidatus.Finniella.inopinata GCA_004210305.1_ASM421030v1
Holospora.undulata.HU1 GCA_000388175.3_ASM38817v3
Candidatus.Hepatobacter.penaei GCA_000742475.1_ASM74247v1
Candidatus.Caedibacter.acanthamoebae GCA_000743035.1_ASM74303v1
Caedimonas.varicaedens GCA_001192655.1_ASM119265v1
Candidatus.Nucleicultrix.amoebiphila.FS5 GCA_002117145.1_ASM211714v1
Tistrella.mobilis.KA081020.065 GCA_000264455.2_ASM26445v2
Geminicoccus.roseus.DSM.18922 GCA_000427665.1_ASM42766v1
alpha.proteobacterium.L41A GCA_000335735.1.L41A.1.0
Brevundimonas.subvibrioides.ATCC.15264 GCA_000144605.1_ASM14460v1
Asticcacaulis.excentricus.CB.48 GCA_000175215.2_ASM17521v2
Phenylobacterium.zucineum.HLK1 GCA_000017265.1_ASM1726v1
Caulobacter.vibrioides.NA1000 GCA_000022005.1_ASM2200v1
Oceanicaulis.alexandrii.DSM.11625 GCA_000420265.1_ASM42026v1
Maricaulis.maris GCA_003634045.1_ASM363404v1
Hyphomonas.neptunium.ATCC.15444 GCA_000013025.1_ASM1302v1
Hirschia.baltica.ATCC.49814 GCA_000023785.1_ASM2378v1
Parvularcula.bermudensis.HTCC2503 GCA_000152825.2_ASM15282v2
Sagittula.stellata.E.37 GCA_000169415.1_ASM16941v1
Ruegeria.pomeroyi.DSS.3 GCA_000011965.2_ASM1196v2
Octadecabacter.arcticus.238 GCA_000155735.2_ASM15573v2
Jannaschia.rubra GCA_900113265.1.IMG.taxon.2622736430.annotated.assembly
Rubellimicrobium.rubrum GCA_006152115.1_ASM615211v1
Ketogulonicigenium.vulgare GCA_001399515.1_ASM139951v1
Rhodobacter.sphaeroides.ATCC.17029 GCA_000015985.1_ASM1598v1
Paracoccus.denitrificans GCA_004063735.1_ASM406373v1
Neomegalonema.perideroedes.DSM.15528 GCA_000374145.1_ASM37414v1
Chelativorans.oligotrophicus GCA_011317505.1_ASM1131750v1
Ahrensia.kielensis.DSM.5890 GCA_000374465.1_ASM37446v1
Rhizobium.leguminosarum.bv.trifolii GCA_011604525.1_ASM1160452v1
Bartonella.quintana GCA_009936175.1_ASM993617v1
Pelagibacterium.halotolerans GCA_013004005.1_ASM1300400v1
Roseibium.hamelinense GCA_007830545.1_ASM783054v1
Polymorphum.gilvum.SL003B.26A1 GCA_000192745.1_ASM19274v1
Pseudovibrio.sp.FO.BEG1 GCA_000236645.1_ASM23664v1
Methylocystis.bryophila GCA_002117405.1_ASM211740v1
Methylocella.silvestris.BL2 GCA_000021745.1_ASM2174v1
Methylorubrum.extorquens.PA1 GCA_000018845.1_ASM1884v1
Xanthobacter.autotrophicus.Py2 GCA_000017645.1_ASM1764v1
Rhodopseudomonas.palustris GCA_013415845.1_ASM1341584v1
Rhodomicrobium.vannielii.ATCC.17100 GCA_000166055.1_ASM16605v1
Hyphomicrobium.denitrificans.ATCC.51888 GCA_000143145.1_ASM14314v1
Thermopetrobacter.sp.TC1 GCA_000746275.1_ASM74627v1
alpha.proteobacterium.IMCC14465 GCA_000293845.2_ASM29384v2
Candidatus.Micropelagos.thuwalensis GCA_000469155.1.RS24
Parvibaculum.lavamentivorans.DS.1 GCA_000017565.1_ASM1756v1
alpha.proteobacterium.HIMB5 GCA_000299095.1_ASM29909v1
Candidatus.Pelagibacter.ubique.HTCC1062 GCA_000012345.1_ASM1234v1
Candidatus.Pelagibacter.ubique.HIMB058 GCA_000419545.1_ASM41954v1
alpha.proteobacterium.HIMB114 GCA_000163555.2
Sphingopyxis.macrogoltabida GCA_001314325.1_ASM131432v1
Citromicrobium.sp.JL477 GCA_001304795.1_ASM130479v1
Sphingobium.indicum.B90A GCA_000264945.2_ASM26494v2
Zymomonas.mobilis.subsp.mobilis.ATCC.31821 GCA_000007105.1_ASM710v1
Sphingomonas.wittichii.RW1 GCA_000016765.1_ASM1676v1
alpha.proteobacterium.AAP81b GCA_001295935.1_ASM129593v1
Kordiimonas.gwangyangensis.DSM.19435 GCA_000375545.1_ASM37554v1
Sneathiella.glossodoripedis.JCM.23214 GCA_000616095.1_ASM61609v1
Candidatus.Neoehrlichia.lotoris.str.RAC413 GCA_000964795.1_ASM96479v1
Anaplasma.phagocytophilum.str.HZ GCA_000013125.1_ASM1312v1
Ehrlichia.ruminantium.str.Welgevonden GCA_000026005.1_ASM2600v1
Wolbachia.endosymbiont.of.Drosophila.melanogaster GCA_000008025.1_ASM802v1
Candidatus.Deianiraea.vastatrix GCA_007993655.1_ASM799365v1
Rickettsiales.endosymbiont.of.Trichoplax.sp.H2 GCA_009690945.1
Candidatus.Midichloria.mitochondrii.IricVA GCA_000219355.1_ASM21935v1
Rickettsiales.endosymbiont.of.Peranema.trichophorum GCA_004210275.1_ASM421027v1
Candidatus.Jidaibacter.acanthamoeba GCA_000815465.1.UWC36
Rickettsia.bellii.RML369.C GCA_000012385.1_ASM1238v1
Candidatus.Megaira.polyxenophila
Rickettsiales.endosymbiont.of.Stachyamoeba.lipophora GCA_003932735.1_ASM393273v1
Rickettsiales.bacterium.Ac37b GCA_000746585.2_ASM74658v2
Magnetofaba.australis.IT.1 GCA_002109495.1_ASM210949v1
Magnetococcus.marinus.MC.1 GCA_000014865.1_ASM1486v1
Nitrosospira.briensis.C.128 GCA_000619905.2_ASM61990v2
Methylotenera.versatilis.301 GCA_000093025.1_ASM9302v1
Ralstonia.pickettii GCA_902374465.1.MGYG.HGUT.01384
Burkholderia.cepacia GCA_009586235.1_ASM958623v1
Dasania.marina.DSM.21967 GCA_000373485.1_ASM37348v1
Congregibacter.litoralis.KT71 GCA_000153125.2_ASM15312v2
Enterobacter.cloacae GCA_000770155.1_ASM77015v1
Alteromonas.macleodii.ATCC.27126 GCA_000172635.2_ASM17263v2
0.55502

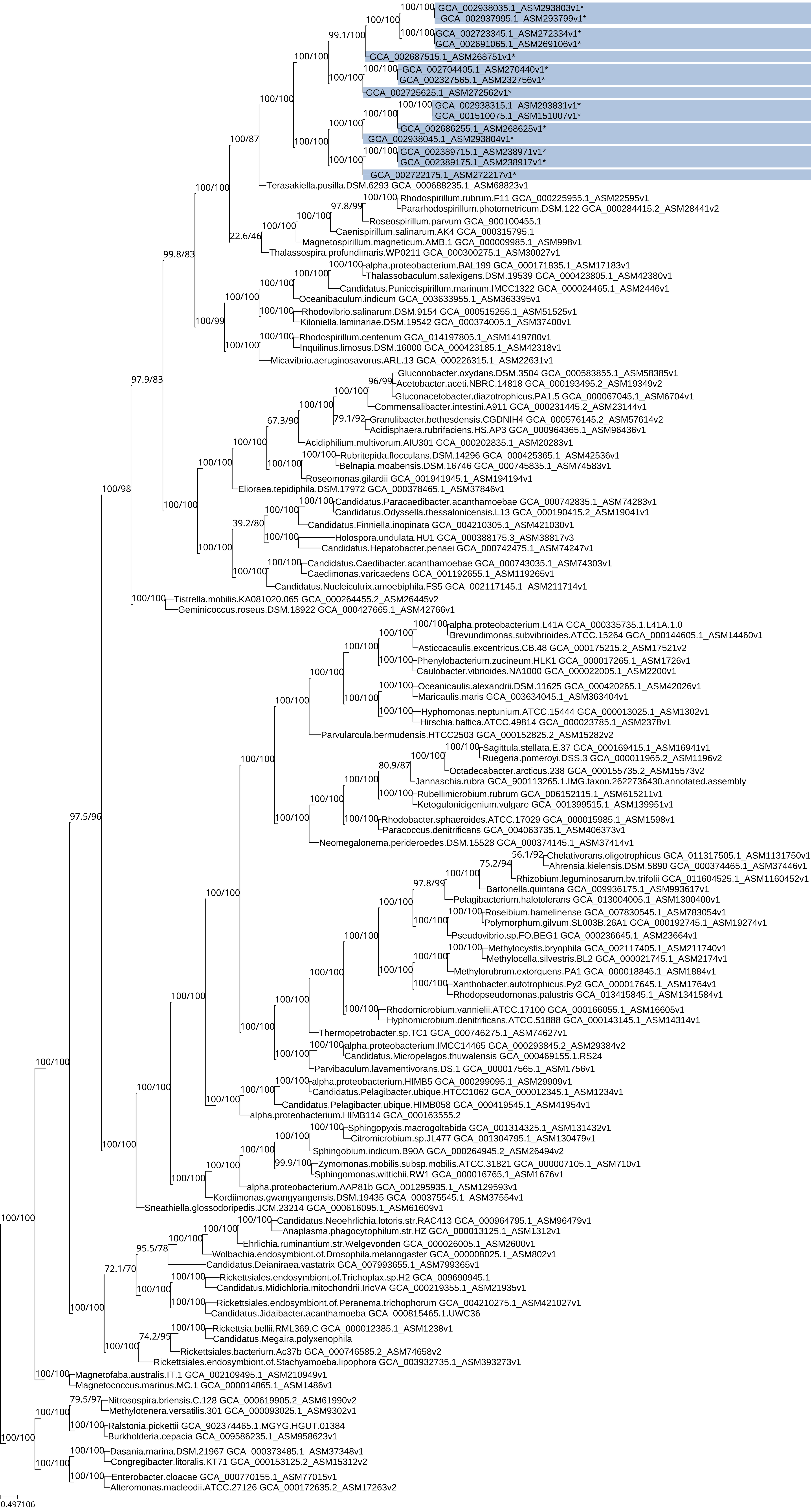
GCA_002938035.1_ASM293803v1*
GCA_002937995.1_ASM293799v1*
GCA_002723345.1_ASM272334v1*
GCA_002691065.1_ASM269106v1*
GCA_002687515.1_ASM268751v1*
GCA_002704405.1_ASM270440v1*
GCA_002327565.1_ASM232756v1*
GCA_002725625.1_ASM272562v1*
GCA_002938315.1_ASM293831v1*
GCA_001510075.1_ASM151007v1*
GCA_002686255.1_ASM268625v1*
GCA_002938045.1_ASM293804v1*
GCA_002389715.1_ASM238971v1*
GCA_002389175.1_ASM238917v1*
GCA_002722175.1_ASM272217v1*
Terasakiella.pusilla.DSM.6293 GCA_000688235.1_ASM68823v1
Rhodospirillum.rubrum.F11 GCA_000225955.1_ASM22595v1
Pararhodospirillum.photometricum.DSM.122 GCA_000284415.2_ASM28441v2
Roseospirillum.parvum GCA_900100455.1
Caenispirillum.salinarum.AK4 GCA_000315795.1
Magnetospirillum.magneticum.AMB.1 GCA_000009985.1_ASM998v1
Thalassospira.profundimaris.WP0211 GCA_000300275.1_ASM30027v1
alpha.proteobacterium.BAL199 GCA_000171835.1_ASM17183v1
Thalassobaculum.salexigens.DSM.19539 GCA_000423805.1_ASM42380v1
Candidatus.Puniceispirillum.marinum.IMCC1322 GCA_000024465.1_ASM2446v1
Oceanibaculum.indicum GCA_003633955.1_ASM363395v1
Rhodovibrio.salinarum.DSM.9154 GCA_000515255.1_ASM51525v1
Kiloniella.laminariae.DSM.19542 GCA_000374005.1_ASM37400v1
Rhodospirillum.centenum GCA_014197805.1_ASM1419780v1
Inquilinus.limosus.DSM.16000 GCA_000423185.1_ASM42318v1
Micavibrio.aeruginosavorus.ARL.13 GCA_000226315.1_ASM22631v1
Gluconobacter.oxydans.DSM.3504 GCA_000583855.1_ASM58385v1
Acetobacter.aceti.NBRC.14818 GCA_000193495.2_ASM19349v2
Gluconacetobacter.diazotrophicus.PA1.5 GCA_000067045.1_ASM6704v1
Commensalibacter.intestini.A911 GCA_000231445.2_ASM23144v1
Granulibacter.bethesdensis.CGDNIH4 GCA_000576145.2_ASM57614v2
Acidisphaera.rubrifaciens.HS.AP3 GCA_000964365.1_ASM96436v1
Acidiphilium.multivorum.AIU301 GCA_000202835.1_ASM20283v1
Rubritepida.flocculans.DSM.14296 GCA_000425365.1_ASM42536v1
Belnapia.moabensis.DSM.16746 GCA_000745835.1_ASM74583v1
Roseomonas.gilardii GCA_001941945.1_ASM194194v1
Elioraea.tepidiphila.DSM.17972 GCA_000378465.1_ASM37846v1
Candidatus.Paracaedibacter.acanthamoebae GCA_000742835.1_ASM74283v1
Candidatus.Odyssella.thessalonicensis.L13 GCA_000190415.2_ASM19041v1
Candidatus.Finniella.inopinata GCA_004210305.1_ASM421030v1
Holospora.undulata.HU1 GCA_000388175.3_ASM38817v3
Candidatus.Hepatobacter.penaei GCA_000742475.1_ASM74247v1
Candidatus.Caedibacter.acanthamoebae GCA_000743035.1_ASM74303v1
Caedimonas.varicaedens GCA_001192655.1_ASM119265v1
Candidatus.Nucleicultrix.amoebiphila.FS5 GCA_002117145.1_ASM211714v1
Tistrella.mobilis.KA081020.065 GCA_000264455.2_ASM26445v2
Geminicoccus.roseus.DSM.18922 GCA_000427665.1_ASM42766v1
alpha.proteobacterium.L41A GCA_000335735.1.L41A.1.0
Brevundimonas.subvibrioides.ATCC.15264 GCA_000144605.1_ASM14460v1
Asticcacaulis.excentricus.CB.48 GCA_000175215.2_ASM17521v2
Phenylobacterium.zucineum.HLK1 GCA_000017265.1_ASM1726v1
Caulobacter.vibrioides.NA1000 GCA_000022005.1_ASM2200v1
Oceanicaulis.alexandrii.DSM.11625 GCA_000420265.1_ASM42026v1
Maricaulis.maris GCA_003634045.1_ASM363404v1
Hyphomonas.neptunium.ATCC.15444 GCA_000013025.1_ASM1302v1
Hirschia.baltica.ATCC.49814 GCA_000023785.1_ASM2378v1
Parvularcula.bermudensis.HTCC2503 GCA_000152825.2_ASM15282v2
Sagittula.stellata.E.37 GCA_000169415.1_ASM16941v1
Ruegeria.pomeroyi.DSS.3 GCA_000011965.2_ASM1196v2
Octadecabacter.arcticus.238 GCA_000155735.2_ASM15573v2
Jannaschia.rubra GCA_900113265.1.IMG.taxon.2622736430.annotated.assembly
Rubellimicrobium.rubrum GCA_006152115.1_ASM615211v1
Ketogulonicigenium.vulgare GCA_001399515.1_ASM139951v1
Rhodobacter.sphaeroides.ATCC.17029 GCA_000015985.1_ASM1598v1
Paracoccus.denitrificans GCA_004063735.1_ASM406373v1
Neomegalonema.perideroedes.DSM.15528 GCA_000374145.1_ASM37414v1
Chelativorans.oligotrophicus GCA_011317505.1_ASM1131750v1
Ahrensia.kielensis.DSM.5890 GCA_000374465.1_ASM37446v1
Rhizobium.leguminosarum.bv.trifolii GCA_011604525.1_ASM1160452v1
Bartonella.quintana GCA_009936175.1_ASM993617v1
Pelagibacterium.halotolerans GCA_013004005.1_ASM1300400v1
Roseibium.hamelinense GCA_007830545.1_ASM783054v1
Polymorphum.gilvum.SL003B.26A1 GCA_000192745.1_ASM19274v1
Pseudovibrio.sp.FO.BEG1 GCA_000236645.1_ASM23664v1
Methylocystis.bryophila GCA_002117405.1_ASM211740v1
Methylocella.silvestris.BL2 GCA_000021745.1_ASM2174v1
Methylorubrum.extorquens.PA1 GCA_000018845.1_ASM1884v1
Xanthobacter.autotrophicus.Py2 GCA_000017645.1_ASM1764v1
Rhodopseudomonas.palustris GCA_013415845.1_ASM1341584v1
Rhodomicrobium.vannielii.ATCC.17100 GCA_000166055.1_ASM16605v1
Hyphomicrobium.denitrificans.ATCC.51888 GCA_000143145.1_ASM14314v1
Thermopetrobacter.sp.TC1 GCA_000746275.1_ASM74627v1
alpha.proteobacterium.IMCC14465 GCA_000293845.2_ASM29384v2
Candidatus.Micropelagos.thuwalensis GCA_000469155.1.RS24
Parvibaculum.lavamentivorans.DS.1 GCA_000017565.1_ASM1756v1
alpha.proteobacterium.HIMB5 GCA_000299095.1_ASM29909v1
Candidatus.Pelagibacter.ubique.HTCC1062 GCA_000012345.1_ASM1234v1
Candidatus.Pelagibacter.ubique.HIMB058 GCA_000419545.1_ASM41954v1
alpha.proteobacterium.HIMB114 GCA_000163555.2
Sphingopyxis.macrogoltabida GCA_001314325.1_ASM131432v1
Citromicrobium.sp.JL477 GCA_001304795.1_ASM130479v1
Sphingobium.indicum.B90A GCA_000264945.2_ASM26494v2
Zymomonas.mobilis.subsp.mobilis.ATCC.31821 GCA_000007105.1_ASM710v1
Sphingomonas.wittichii.RW1 GCA_000016765.1_ASM1676v1
alpha.proteobacterium.AAP81b GCA_001295935.1_ASM129593v1
Kordiimonas.gwangyangensis.DSM.19435 GCA_000375545.1_ASM37554v1
Sneathiella.glossodoripedis.JCM.23214 GCA_000616095.1_ASM61609v1
Candidatus.Neoehrlichia.lotoris.str.RAC413 GCA_000964795.1_ASM96479v1
Anaplasma.phagocytophilum.str.HZ GCA_000013125.1_ASM1312v1
Ehrlichia.ruminantium.str.Welgevonden GCA_000026005.1_ASM2600v1
Wolbachia.endosymbiont.of.Drosophila.melanogaster GCA_000008025.1_ASM802v1
Candidatus.Deianiraea.vastatrix GCA_007993655.1_ASM799365v1
Rickettsiales.endosymbiont.of.Trichoplax.sp.H2 GCA_009690945.1
Candidatus.Midichloria.mitochondrii.IricVA GCA_000219355.1_ASM21935v1
Rickettsiales.endosymbiont.of.Peranema.trichophorum GCA_004210275.1_ASM421027v1
Candidatus.Jidaibacter.acanthamoeba GCA_000815465.1.UWC36
Rickettsia.bellii.RML369.C GCA_000012385.1_ASM1238v1
Candidatus.Megaira.polyxenophila
Rickettsiales.bacterium.Ac37b GCA_000746585.2_ASM74658v2
Rickettsiales.endosymbiont.of.Stachyamoeba.lipophora GCA_003932735.1_ASM393273v1
Magnetofaba.australis.IT.1 GCA_002109495.1_ASM210949v1
Magnetococcus.marinus.MC.1 GCA_000014865.1_ASM1486v1
Nitrosospira.briensis.C.128 GCA_000619905.2_ASM61990v2
Methylotenera.versatilis.301 GCA_000093025.1_ASM9302v1
Ralstonia.pickettii GCA_902374465.1.MGYG.HGUT.01384
Burkholderia.cepacia GCA_009586235.1_ASM958623v1
Dasania.marina.DSM.21967 GCA_000373485.1_ASM37348v1
Congregibacter.litoralis.KT71 GCA_000153125.2_ASM15312v2
Enterobacter.cloacae GCA_000770155.1_ASM77015v1
Alteromonas.macleodii.ATCC.27126 GCA_000172635.2_ASM17263v2
0.497106

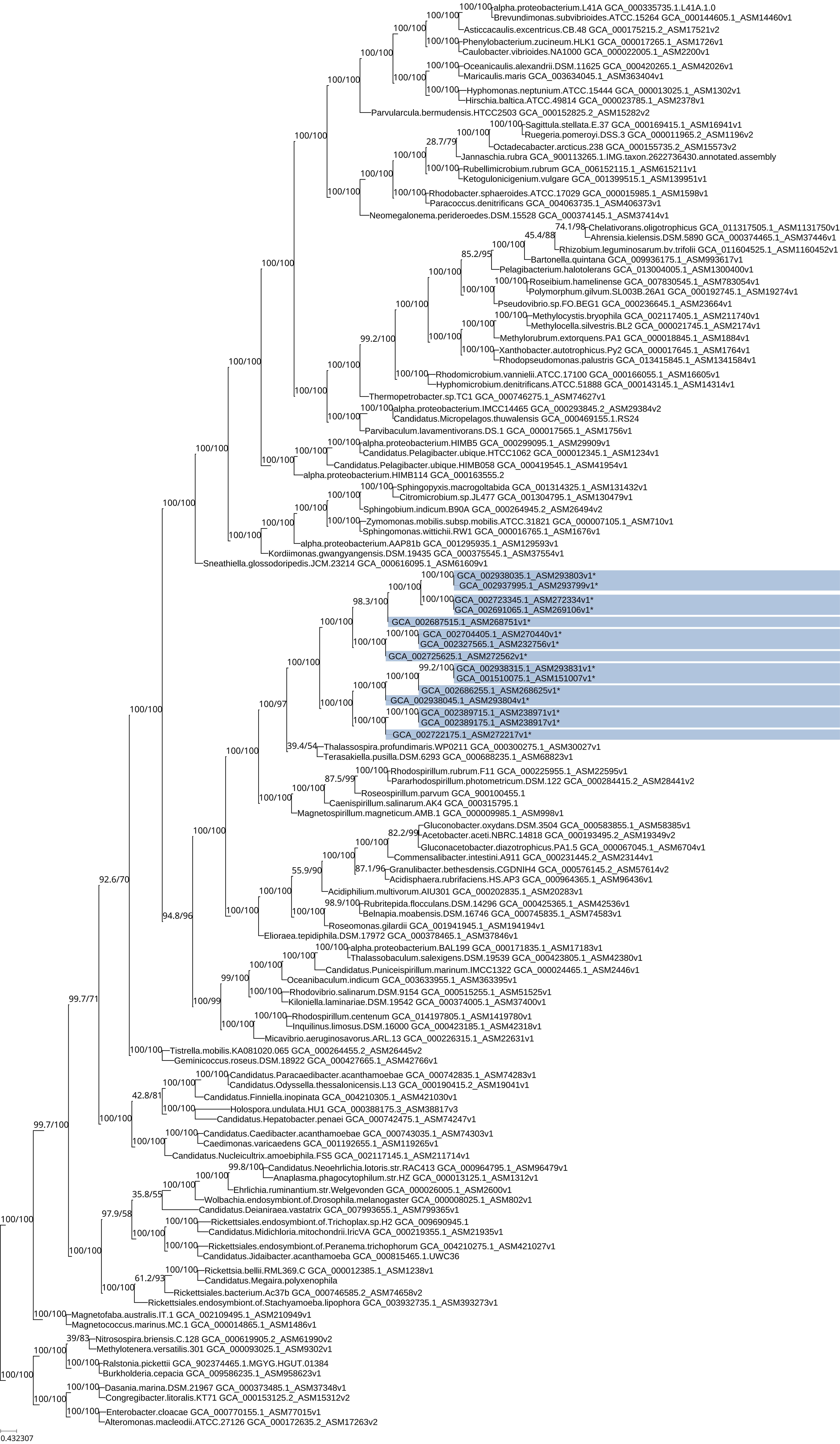
alpha.proteobacterium.L41A GCA_000335735.1.L41A.1.0
Brevundimonas.subvibrioides.ATCC.15264 GCA_000144605.1_ASM14460v1
Asticcacaulis.excentricus.CB.48 GCA_000175215.2_ASM17521v2
Phenylobacterium.zucineum.HLK1 GCA_000017265.1_ASM1726v1
Caulobacter.vibrioides.NA1000 GCA_000022005.1_ASM2200v1
Oceanicaulis.alexandrii.DSM.11625 GCA_000420265.1_ASM42026v1
Maricaulis.maris GCA_003634045.1_ASM363404v1
Hyphomonas.neptunium.ATCC.15444 GCA_000013025.1_ASM1302v1
Hirschia.baltica.ATCC.49814 GCA_000023785.1_ASM2378v1
Parvularcula.bermudensis.HTCC2503 GCA_000152825.2_ASM15282v2
Sagittula.stellata.E.37 GCA_000169415.1_ASM16941v1
Ruegeria.pomeroyi.DSS.3 GCA_000011965.2_ASM1196v2
Octadecabacter.arcticus.238 GCA_000155735.2_ASM15573v2
Jannaschia.rubra GCA_900113265.1.IMG.taxon.2622736430.annotated.assembly
Rubellimicrobium.rubrum GCA_006152115.1_ASM615211v1
Ketogulonicigenium.vulgare GCA_001399515.1_ASM139951v1
Rhodobacter.sphaeroides.ATCC.17029 GCA_000015985.1_ASM1598v1
Paracoccus.denitrificans GCA_004063735.1_ASM406373v1
Neomegalonema.perideroedes.DSM.15528 GCA_000374145.1_ASM37414v1
Chelativorans.oligotrophicus GCA_011317505.1_ASM1131750v1
Ahrensia.kielensis.DSM.5890 GCA_000374465.1_ASM37446v1
Rhizobium.leguminosarum.bv.trifolii GCA_011604525.1_ASM1160452v1
Bartonella.quintana GCA_009936175.1_ASM993617v1
Pelagibacterium.halotolerans GCA_013004005.1_ASM1300400v1
Roseibium.hamelinense GCA_007830545.1_ASM783054v1
Polymorphum.gilvum.SL003B.26A1 GCA_000192745.1_ASM19274v1
Pseudovibrio.sp.FO.BEG1 GCA_000236645.1_ASM23664v1
Methylocystis.bryophila GCA_002117405.1_ASM211740v1
Methylocella.silvestris.BL2 GCA_000021745.1_ASM2174v1
Methylorubrum.extorquens.PA1 GCA_000018845.1_ASM1884v1
Xanthobacter.autotrophicus.Py2 GCA_000017645.1_ASM1764v1
Rhodopseudomonas.palustris GCA_013415845.1_ASM1341584v1
Rhodomicrobium.vannielii.ATCC.17100 GCA_000166055.1_ASM16605v1
Hyphomicrobium.denitrificans.ATCC.51888 GCA_000143145.1_ASM14314v1
Thermopetrobacter.sp.TC1 GCA_000746275.1_ASM74627v1
alpha.proteobacterium.IMCC14465 GCA_000293845.2_ASM29384v2
Candidatus.Micropelagos.thuwalensis GCA_000469155.1.RS24
Parvibaculum.lavamentivorans.DS.1 GCA_000017565.1_ASM1756v1
alpha.proteobacterium.HIMB5 GCA_000299095.1_ASM29909v1
Candidatus.Pelagibacter.ubique.HTCC1062 GCA_000012345.1_ASM1234v1
Candidatus.Pelagibacter.ubique.HIMB058 GCA_000419545.1_ASM41954v1
alpha.proteobacterium.HIMB114 GCA_000163555.2
Sphingopyxis.macrogoltabida GCA_001314325.1_ASM131432v1
Citromicrobium.sp.JL477 GCA_001304795.1_ASM130479v1
Sphingobium.indicum.B90A GCA_000264945.2_ASM26494v2
Zymomonas.mobilis.subsp.mobilis.ATCC.31821 GCA_000007105.1_ASM710v1
Sphingomonas.wittichii.RW1 GCA_000016765.1_ASM1676v1
alpha.proteobacterium.AAP81b GCA_001295935.1_ASM129593v1
Kordiimonas.gwangyangensis.DSM.19435 GCA_000375545.1_ASM37554v1
Sneathiella.glossodoripedis.JCM.23214 GCA_000616095.1_ASM61609v1
GCA_002938035.1_ASM293803v1*
GCA_002937995.1_ASM293799v1*
GCA_002723345.1_ASM272334v1*
GCA_002691065.1_ASM269106v1*
GCA_002687515.1_ASM268751v1*
GCA_002704405.1_ASM270440v1*
GCA_002327565.1_ASM232756v1*
GCA_002725625.1_ASM272562v1*
GCA_002938315.1_ASM293831v1*
GCA_001510075.1_ASM151007v1*
GCA_002686255.1_ASM268625v1*
GCA_002938045.1_ASM293804v1*
GCA_002389715.1_ASM238971v1*
GCA_002389175.1_ASM238917v1*
GCA_002722175.1_ASM272217v1*
Thalassospira.profundimaris.WP0211 GCA_000300275.1_ASM30027v1
Terasakiella.pusilla.DSM.6293 GCA_000688235.1_ASM68823v1
Rhodospirillum.rubrum.F11 GCA_000225955.1_ASM22595v1
Pararhodospirillum.photometricum.DSM.122 GCA_000284415.2_ASM28441v2
Roseospirillum.parvum GCA_900100455.1
Caenispirillum.salinarum.AK4 GCA_000315795.1
Magnetospirillum.magneticum.AMB.1 GCA_000009985.1_ASM998v1
Gluconobacter.oxydans.DSM.3504 GCA_000583855.1_ASM58385v1
Acetobacter.aceti.NBRC.14818 GCA_000193495.2_ASM19349v2
Gluconacetobacter.diazotrophicus.PA1.5 GCA_000067045.1_ASM6704v1
Commensalibacter.intestini.A911 GCA_000231445.2_ASM23144v1
Granulibacter.bethesdensis.CGDNIH4 GCA_000576145.2_ASM57614v2
Acidisphaera.rubrifaciens.HS.AP3 GCA_000964365.1_ASM96436v1
Acidiphilium.multivorum.AIU301 GCA_000202835.1_ASM20283v1
Rubritepida.flocculans.DSM.14296 GCA_000425365.1_ASM42536v1
Belnapia.moabensis.DSM.16746 GCA_000745835.1_ASM74583v1
Roseomonas.gilardii GCA_001941945.1_ASM194194v1
Elioraea.tepidiphila.DSM.17972 GCA_000378465.1_ASM37846v1
alpha.proteobacterium.BAL199 GCA_000171835.1_ASM17183v1
Thalassobaculum.salexigens.DSM.19539 GCA_000423805.1_ASM42380v1
Candidatus.Puniceispirillum.marinum.IMCC1322 GCA_000024465.1_ASM2446v1
Oceanibaculum.indicum GCA_003633955.1_ASM363395v1
Rhodovibrio.salinarum.DSM.9154 GCA_000515255.1_ASM51525v1
Kiloniella.laminariae.DSM.19542 GCA_000374005.1_ASM37400v1
Rhodospirillum.centenum GCA_014197805.1_ASM1419780v1
Inquilinus.limosus.DSM.16000 GCA_000423185.1_ASM42318v1
Micavibrio.aeruginosavorus.ARL.13 GCA_000226315.1_ASM22631v1
Tistrella.mobilis.KA081020.065 GCA_000264455.2_ASM26445v2
Geminicoccus.roseus.DSM.18922 GCA_000427665.1_ASM42766v1
Candidatus.Paracaedibacter.acanthamoebae GCA_000742835.1_ASM74283v1
Candidatus.Odyssella.thessalonicensis.L13 GCA_000190415.2_ASM19041v1
Candidatus.Finniella.inopinata GCA_004210305.1_ASM421030v1
Holospora.undulata.HU1 GCA_000388175.3_ASM38817v3
Candidatus.Hepatobacter.penaei GCA_000742475.1_ASM74247v1
Candidatus.Caedibacter.acanthamoebae GCA_000743035.1_ASM74303v1
Caedimonas.varicaedens GCA_001192655.1_ASM119265v1
Candidatus.Nucleicultrix.amoebiphila.FS5 GCA_002117145.1_ASM211714v1
Candidatus.Neoehrlichia.lotoris.str.RAC413 GCA_000964795.1_ASM96479v1
Anaplasma.phagocytophilum.str.HZ GCA_000013125.1_ASM1312v1
Ehrlichia.ruminantium.str.Welgevonden GCA_000026005.1_ASM2600v1
Wolbachia.endosymbiont.of.Drosophila.melanogaster GCA_000008025.1_ASM802v1
Candidatus.Deianiraea.vastatrix GCA_007993655.1_ASM799365v1
Rickettsiales.endosymbiont.of.Trichoplax.sp.H2 GCA_009690945.1
Candidatus.Midichloria.mitochondrii.IricVA GCA_000219355.1_ASM21935v1
Rickettsiales.endosymbiont.of.Peranema.trichophorum GCA_004210275.1_ASM421027v1
Candidatus.Jidaibacter.acanthamoeba GCA_000815465.1.UWC36
Rickettsia.bellii.RML369.C GCA_000012385.1_ASM1238v1
Candidatus.Megaira.polyxenophila
Rickettsiales.bacterium.Ac37b GCA_000746585.2_ASM74658v2
Rickettsiales.endosymbiont.of.Stachyamoeba.lipophora GCA_003932735.1_ASM393273v1
Magnetofaba.australis.IT.1 GCA_002109495.1_ASM210949v1
Magnetococcus.marinus.MC.1 GCA_000014865.1_ASM1486v1
Nitrosospira.briensis.C.128 GCA_000619905.2_ASM61990v2
Methylotenera.versatilis.301 GCA_000093025.1_ASM9302v1
Ralstonia.pickettii GCA_902374465.1.MGYG.HGUT.01384
Burkholderia.cepacia GCA_009586235.1_ASM958623v1
Dasania.marina.DSM.21967 GCA_000373485.1_ASM37348v1
Congregibacter.litoralis.KT71 GCA_000153125.2_ASM15312v2
Enterobacter.cloacae GCA_000770155.1_ASM77015v1
Alteromonas.macleodii.ATCC.27126 GCA_000172635.2_ASM17263v2
0.432307

Group E

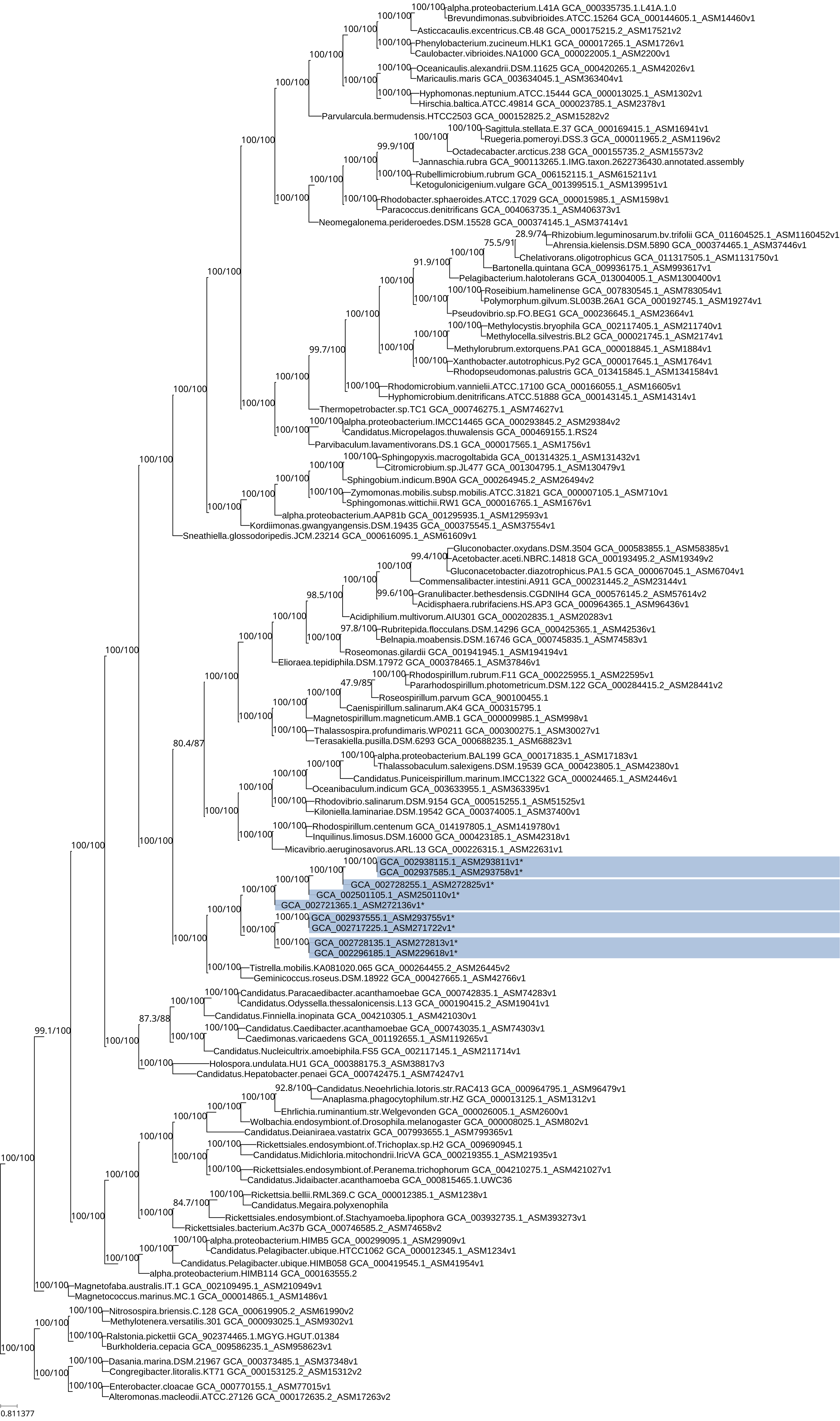

alpha.proteobacterium.L41A GCA_000335735.1.L41A.1.0
Brevundimonas.subvibrioides.ATCC.15264 GCA_000144605.1_ASM14460v1
Asticcacaulis.excentricus.CB.48 GCA_000175215.2_ASM17521v2
Phenylobacterium.zucineum.HLK1 GCA_000017265.1_ASM1726v1
Caulobacter.vibrioides.NA1000 GCA_000022005.1_ASM2200v1
Oceanicaulis.alexandrii.DSM.11625 GCA_000420265.1_ASM42026v1
Maricaulis.maris GCA_003634045.1_ASM363404v1
Hyphomonas.neptunium.ATCC.15444 GCA_000013025.1_ASM1302v1
Hirschia.baltica.ATCC.49814 GCA_000023785.1_ASM2378v1
Parvularcula.bermudensis.HTCC2503 GCA_000152825.2_ASM15282v2
Sagittula.stellata.E.37 GCA_000169415.1_ASM16941v1
Ruegeria.pomeroyi.DSS.3 GCA_000011965.2_ASM1196v2
Octadecabacter.arcticus.238 GCA_000155735.2_ASM15573v2
Jannaschia.rubra GCA_900113265.1.IMG.taxon.2622736430.annotated.assembly
Rubellimicrobium.rubrum GCA_006152115.1_ASM615211v1
Ketogulonicigenium.vulgare GCA_001399515.1_ASM139951v1
Rhodobacter.sphaeroides.ATCC.17029 GCA_000015985.1_ASM1598v1
Paracoccus.denitrificans GCA_004063735.1_ASM406373v1
Neomegalonema.perideroedes.DSM.15528 GCA_000374145.1_ASM37414v1
Rhizobium.leguminosarum.bv.trifolii GCA_011604525.1_ASM1160452v1
Ahrensia.kielensis.DSM.5890 GCA_000374465.1_ASM37446v1
Chelativorans.oligotrophicus GCA_011317505.1_ASM1131750v1
Bartonella.quintana GCA_009936175.1_ASM993617v1
Pelagibacterium.halotolerans GCA_013004005.1_ASM1300400v1
Roseibium.hamelinense GCA_007830545.1_ASM783054v1
Polymorphum.gilvum.SL003B.26A1 GCA_000192745.1_ASM19274v1
Pseudovibrio.sp.FO.BEG1 GCA_000236645.1_ASM23664v1
Methylocystis.bryophila GCA_002117405.1_ASM211740v1
Methylocella.silvestris.BL2 GCA_000021745.1_ASM2174v1
Methylorubrum.extorquens.PA1 GCA_000018845.1_ASM1884v1
Xanthobacter.autotrophicus.Py2 GCA_000017645.1_ASM1764v1
Rhodopseudomonas.palustris GCA_013415845.1_ASM1341584v1
Rhodomicrobium.vannielii.ATCC.17100 GCA_000166055.1_ASM16605v1
Hyphomicrobium.denitrificans.ATCC.51888 GCA_000143145.1_ASM14314v1
Thermopetrobacter.sp.TC1 GCA_000746275.1_ASM74627v1
alpha.proteobacterium.IMCC14465 GCA_000293845.2_ASM29384v2
Candidatus.Micropelagos.thuwalensis GCA_000469155.1.RS24
Parvibaculum.lavamentivorans.DS.1 GCA_000017565.1_ASM1756v1
Sphingopyxis.macrogoltabida GCA_001314325.1_ASM131432v1
Citromicrobium.sp.JL477 GCA_001304795.1_ASM130479v1
Sphingobium.indicum.B90A GCA_000264945.2_ASM26494v2
Zymomonas.mobilis.subsp.mobilis.ATCC.31821 GCA_000007105.1_ASM710v1
Sphingomonas.wittichii.RW1 GCA_000016765.1_ASM1676v1
alpha.proteobacterium.AAP81b GCA_001295935.1_ASM129593v1
Kordiimonas.gwangyangensis.DSM.19435 GCA_000375545.1_ASM37554v1
Sneathiella.glossodoripedis.JCM.23214 GCA_000616095.1_ASM61609v1
Gluconobacter.oxydans.DSM.3504 GCA_000583855.1_ASM58385v1
Acetobacter.aceti.NBRC.14818 GCA_000193495.2_ASM19349v2
Gluconacetobacter.diazotrophicus.PA1.5 GCA_000067045.1_ASM6704v1
Commensalibacter.intestini.A911 GCA_000231445.2_ASM23144v1
Granulibacter.bethesdensis.CGDNIH4 GCA_000576145.2_ASM57614v2
Acidisphaera.rubrifaciens.HS.AP3 GCA_000964365.1_ASM96436v1
Acidiphilium.multivorum.AIU301 GCA_000202835.1_ASM20283v1
Rubritepida.flocculans.DSM.14296 GCA_000425365.1_ASM42536v1
Belnapia.moabensis.DSM.16746 GCA_000745835.1_ASM74583v1
Roseomonas.gilardii GCA_001941945.1_ASM194194v1
Elioraea.tepidiphila.DSM.17972 GCA_000378465.1_ASM37846v1
Rhodospirillum.rubrum.F11 GCA_000225955.1_ASM22595v1
Pararhodospirillum.photometricum.DSM.122 GCA_000284415.2_ASM28441v2
Roseospirillum.parvum GCA_900100455.1
Caenispirillum.salinarum.AK4 GCA_000315795.1
Magnetospirillum.magneticum.AMB.1 GCA_000009985.1_ASM998v1
Thalassospira.profundimaris.WP0211 GCA_000300275.1_ASM30027v1
Terasakiella.pusilla.DSM.6293 GCA_000688235.1_ASM68823v1
alpha.proteobacterium.BAL199 GCA_000171835.1_ASM17183v1
Thalassobaculum.salexigens.DSM.19539 GCA_000423805.1_ASM42380v1
Candidatus.Puniceispirillum.marinum.IMCC1322 GCA_000024465.1_ASM2446v1
Oceanibaculum.indicum GCA_003633955.1_ASM363395v1
Rhodovibrio.salinarum.DSM.9154 GCA_000515255.1_ASM51525v1
Kiloniella.laminariae.DSM.19542 GCA_000374005.1_ASM37400v1
Rhodospirillum.centenum GCA_014197805.1_ASM1419780v1
Inquilinus.limosus.DSM.16000 GCA_000423185.1_ASM42318v1
Micavibrio.aeruginosavorus.ARL.13 GCA_000226315.1_ASM22631v1
GCA_002938115.1_ASM293811v1*
GCA_002937585.1_ASM293758v1*
GCA_002728255.1_ASM272825v1*
GCA_002501105.1_ASM250110v1*
GCA_002721365.1_ASM272136v1*
GCA_002937555.1_ASM293755v1*
GCA_002717225.1_ASM271722v1*
GCA_002728135.1_ASM272813v1*
GCA_002296185.1_ASM229618v1*
Tistrella.mobilis.KA081020.065 GCA_000264455.2_ASM26445v2
Geminicoccus.roseus.DSM.18922 GCA_000427665.1_ASM42766v1
Candidatus.Paracaedibacter.acanthamoebae GCA_000742835.1_ASM74283v1
Candidatus.Odyssella.thessalonicensis.L13 GCA_000190415.2_ASM19041v1
Candidatus.Finniella.inopinata GCA_004210305.1_ASM421030v1
Candidatus.Caedibacter.acanthamoebae GCA_000743035.1_ASM74303v1
Caedimonas.varicaedens GCA_001192655.1_ASM119265v1
Candidatus.Nucleicultrix.amoebiphila.FS5 GCA_002117145.1_ASM211714v1
Holospora.undulata.HU1 GCA_000388175.3_ASM38817v3
Candidatus.Hepatobacter.penaei GCA_000742475.1_ASM74247v1
Candidatus.Neoehrlichia.lotoris.str.RAC413 GCA_000964795.1_ASM96479v1
Anaplasma.phagocytophilum.str.HZ GCA_000013125.1_ASM1312v1
Ehrlichia.ruminantium.str.Welgevonden GCA_000026005.1_ASM2600v1
Wolbachia.endosymbiont.of.Drosophila.melanogaster GCA_000008025.1_ASM802v1
Candidatus.Deianiraea.vastatrix GCA_007993655.1_ASM799365v1
Rickettsiales.endosymbiont.of.Trichoplax.sp.H2 GCA_009690945.1
Candidatus.Midichloria.mitochondrii.IricVA GCA_000219355.1_ASM21935v1
Rickettsiales.endosymbiont.of.Peranema.trichophorum GCA_004210275.1_ASM421027v1
Candidatus.Jidaibacter.acanthamoeba GCA_000815465.1.UWC36
Rickettsia.bellii.RML369.C GCA_000012385.1_ASM1238v1
Candidatus.Megaira.polyxenophila
Rickettsiales.endosymbiont.of.Stachyamoeba.lipophora GCA_003932735.1_ASM393273v1
Rickettsiales.bacterium.Ac37b GCA_000746585.2_ASM74658v2
alpha.proteobacterium.HIMB5 GCA_000299095.1_ASM29909v1
Candidatus.Pelagibacter.ubique.HTCC1062 GCA_000012345.1_ASM1234v1
Candidatus.Pelagibacter.ubique.HIMB058 GCA_000419545.1_ASM41954v1
alpha.proteobacterium.HIMB114 GCA_000163555.2
Magnetofaba.australis.IT.1 GCA_002109495.1_ASM210949v1
Magnetococcus.marinus.MC.1 GCA_000014865.1_ASM1486v1
Nitrosospira.briensis.C.128 GCA_000619905.2_ASM61990v2
Methylotenera.versatilis.301 GCA_000093025.1_ASM9302v1
Ralstonia.pickettii GCA_902374465.1.MGYG.HGUT.01384
Burkholderia.cepacia GCA_009586235.1_ASM958623v1
Dasania.marina.DSM.21967 GCA_000373485.1_ASM37348v1
Congregibacter.litoralis.KT71 GCA_000153125.2_ASM15312v2
Enterobacter.cloacae GCA_000770155.1_ASM77015v1
Alteromonas.macleodii.ATCC.27126 GCA_000172635.2_ASM17263v2
0.811377

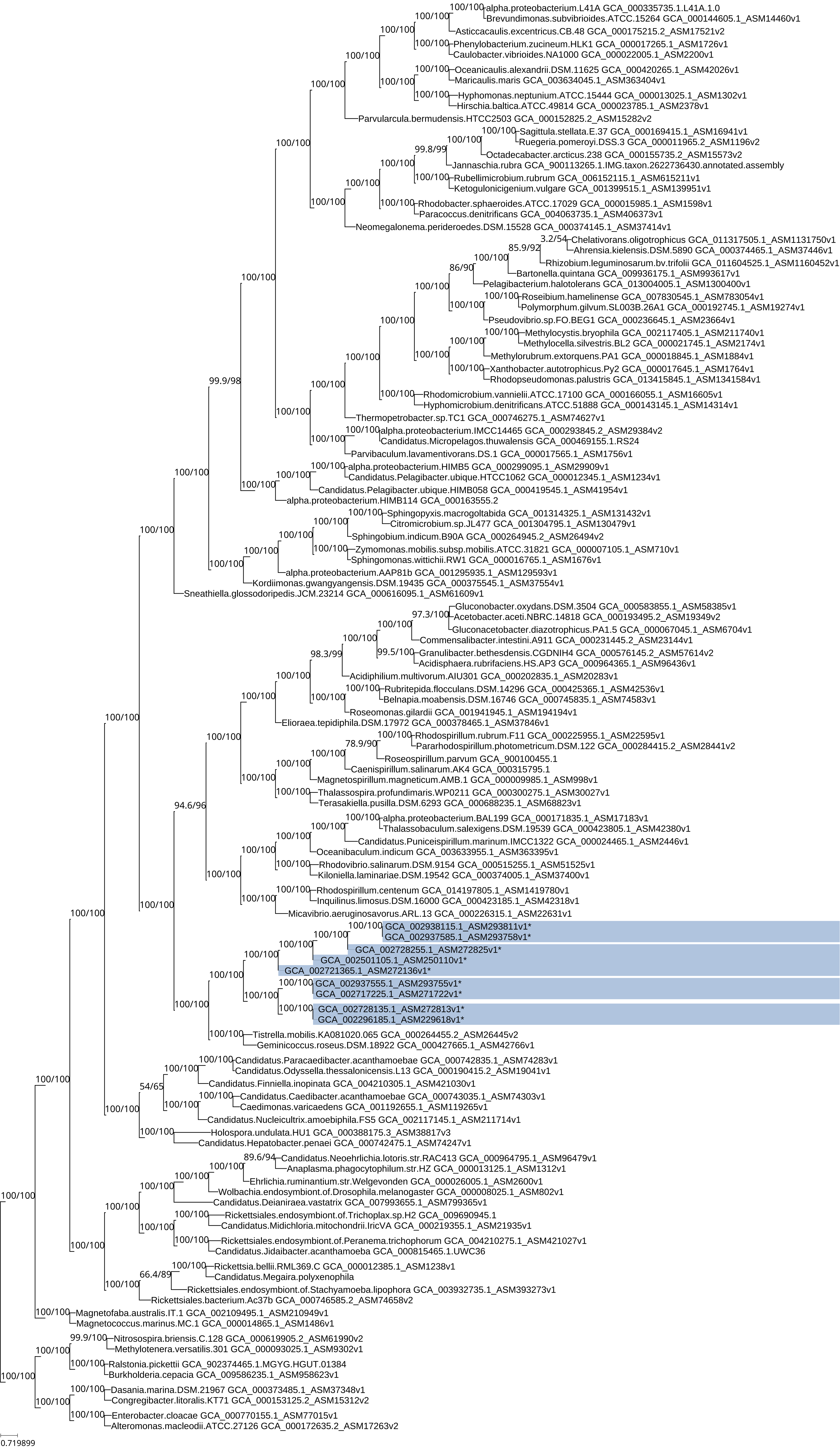

alpha.proteobacterium.L41A GCA_000335735.1_L41A.1.0
Brevundimonas.subvibrioides.ATCC.15264 GCA_000144605.1_ASM14460v1
Asticcacaulis.excentricus.CB.48 GCA_000175215.2_ASM17521v2
Phenylobacterium.zucineum.HLK1 GCA_000017265.1_ASM1726v1
Caulobacter.vibrioides.NA1000 GCA_000022005.1_ASM2200v1
Oceanicaulis.alexandrii.DSM.11625 GCA_000420265.1_ASM42026v1
Maricaulis.maris GCA_003634045.1_ASM363404v1
Hyphomonas.neptunium.ATCC.15444 GCA_000013025.1_ASM1302v1
Hirschia.baltica.ATCC.49814 GCA_000023785.1_ASM2378v1
Parvularcula.bermudensis.HTCC2503 GCA_000152825.2_ASM15282v2
Sagittula.stellata.E.37 GCA_000169415.1_ASM16941v1
Ruegeria.pomeroyi.DSS.3 GCA_000011965.2_ASM1196v2
Octadecabacter.arcticus.238 GCA_000155735.2_ASM15573v2
Jannaschia.rubra GCA_900113265.1.IMG.taxon.2622736430.annotated.assembly
Rubellimicrobium.rubrum GCA_006152115.1_ASM615211v1
Ketogulonicigenium.vulgare GCA_001399515.1_ASM139951v1
Rhodobacter.sphaeroides.ATCC.17029 GCA_000015985.1_ASM1598v1
Paracoccus.denitrificans GCA_004063735.1_ASM406373v1
Neomegalonema.perideroedes.DSM.15528 GCA_000374145.1_ASM37414v1
Chelativorans.oligotrophicus GCA_011317505.1_ASM1131750v1
Ahrensia.kielensis.DSM.5890 GCA_000374465.1_ASM37446v1
Rhizobium.leguminosarum.bv.trifolii GCA_011604525.1_ASM1160452v1
Bartonella.quintana GCA_009936175.1_ASM993617v1
Pelagibacterium.halotolerans GCA_013004005.1_ASM1300400v1
Roseibium.hamelinense GCA_007830545.1_ASM783054v1
Polymorphum.gilvum.SL003B.26A1 GCA_000192745.1_ASM19274v1
Pseudovibrio.sp.FO.BEG1 GCA_000236645.1_ASM23664v1
Methylocystis.bryophila GCA_002117405.1_ASM211740v1
Methylocella.silvestris.BL2 GCA_000021745.1_ASM2174v1
Methylorubrum.extorquens.PA1 GCA_000018845.1_ASM1884v1
Xanthobacter.autotrophicus.Py2 GCA_000017645.1_ASM1764v1
Rhodopseudomonas.palustris GCA_013415845.1_ASM1341584v1
Rhodomicrobium.vannielii.ATCC.17100 GCA_000166055.1_ASM16605v1
Hyphomicrobium.denitrificans.ATCC.51888 GCA_000143145.1_ASM14314v1
Thermopetrobacter.sp.TC1 GCA_000746275.1_ASM74627v1
alpha.proteobacterium.IMCC14465 GCA_000293845.2_ASM29384v2
Candidatus.Micropelagos.thuwalensis GCA_000469155.1.RS24
Parvibaculum.lavamentivorans.DS.1 GCA_000017565.1_ASM1756v1
alpha.proteobacterium.HIMB5 GCA_000299095.1_ASM29909v1
Candidatus.Pelagibacter.ubique.HTCC1062 GCA_000012345.1_ASM1234v1
Candidatus.Pelagibacter.ubique.HIMB058 GCA_000419545.1_ASM41954v1
alpha.proteobacterium.HIMB114 GCA_000163555.2
Sphingopyxis.macrogoltabida GCA_001314325.1_ASM131432v1
Citromicrobium.sp.JL477 GCA_001304795.1_ASM130479v1
Sphingobium.indicum.B90A GCA_000264945.2_ASM26494v2
Zymomonas.mobilis.subsp.mobilis.ATCC.31821 GCA_000007105.1_ASM710v1
Sphingomonas.wittichii.RW1 GCA_000016765.1_ASM1676v1
alpha.proteobacterium.AAP81b GCA_001295935.1_ASM129593v1
Kordiimonas.gwangyangensis.DSM.19435 GCA_000375545.1_ASM37554v1
Sneathiella.glossodoripedis.JCM.23214 GCA_000616095.1_ASM61609v1
Gluconobacter.oxydans.DSM.3504 GCA_000583855.1_ASM58385v1
Acetobacter.aceti.NBRC.14818 GCA_000193495.2_ASM19349v2
Gluconacetobacter.diazotrophicus.PA1.5 GCA_000067045.1_ASM6704v1
Commensalibacter.intestini.A911 GCA_000231445.2_ASM23144v1
Granulibacter.bethesdensis.CGDNIH4 GCA_000576145.2_ASM57614v2
Acidisphaera.rubrifaciens.HS.AP3 GCA_000964365.1_ASM96436v1
Acidiphilium.multivorum.AIU301 GCA_000202835.1_ASM20283v1
Rubritepida.flocculans.DSM.14296 GCA_000425365.1_ASM42536v1
Belnapia.moabensis.DSM.16746 GCA_000745835.1_ASM74583v1
Roseomonas.gilardii GCA_001941945.1_ASM194194v1
Elioraea.tepidiphila.DSM.17972 GCA_000378465.1_ASM37846v1
Rhodospirillum.rubrum.F11 GCA_000225955.1_ASM22595v1
Pararhodospirillum.photometricum.DSM.122 GCA_000284415.2_ASM28441v2
Roseospirillum.parvum GCA_900100455.1
Caenispirillum.salinarum.AK4 GCA_000315795.1
Magnetospirillum.magneticum.AMB.1 GCA_000009985.1_ASM998v1
Thalassospira.profundimaris.WP0211 GCA_000300275.1_ASM30027v1
Terasakiella.pusilla.DSM.6293 GCA_000688235.1_ASM68823v1
alpha.proteobacterium.BAL199 GCA_000171835.1_ASM17183v1
Thalassobaculum.salexigens.DSM.19539 GCA_000423805.1_ASM42380v1
Candidatus.Puniceispirillum.marinum.IMCC1322 GCA_000024465.1_ASM2446v1
Oceanibaculum.indicum GCA_003633955.1_ASM363395v1
Rhodovibrio.salinarum.DSM.9154 GCA_000515255.1_ASM51525v1
Kiloniella.laminariae.DSM.19542 GCA_000374005.1_ASM37400v1
Rhodospirillum.centenum GCA_014197805.1_ASM1419780v1
Inquilinus.limosus.DSM.16000 GCA_000423185.1_ASM42318v1
Micavibrio.aeruginosavorus.ARL.13 GCA_000226315.1_ASM22631v1
GCA_002938115.1_ASM293811v1*
GCA_002937585.1_ASM293758v1*
GCA_002728255.1_ASM272825v1*
GCA_002501105.1_ASM250110v1*
GCA_002721365.1_ASM272136v1*
GCA_002937555.1_ASM293755v1*
GCA_002717225.1_ASM271722v1*
GCA_002728135.1_ASM272813v1*
GCA_002296185.1_ASM229618v1*
Tistrella.mobilis.KA081020.065 GCA_000264455.2_ASM26445v2
Geminicoccus.roseus.DSM.18922 GCA_000427665.1_ASM42766v1
Candidatus.Paracaedibacter.acanthamoebae GCA_000742835.1_ASM74283v1
Candidatus.Odyssella.thessalonicensis.L13 GCA_000190415.2_ASM19041v1
Candidatus.Finniella.inopinata GCA_004210305.1_ASM421030v1
Candidatus.Caedibacter.acanthamoebae GCA_000743035.1_ASM74303v1
Caedimonas.varicaedens GCA_001192655.1_ASM119265v1
Candidatus.Nucleicultrix.amoebiphila.FS5 GCA_002117145.1_ASM211714v1
Holospora.undulata.HU1 GCA_000388175.3_ASM38817v3
Candidatus.Hepatobacter.penaei GCA_000742475.1_ASM74247v1
Candidatus.Neoehrlichia.lotoris.str.RAC413 GCA_000964795.1_ASM96479v1
Anaplasma.phagocytophilum.str.HZ GCA_000013125.1_ASM1312v1
Ehrlichia.ruminantium.str.Welgevonden GCA_000026005.1_ASM2600v1
Wolbachia.endosymbiont.of.Drosophila.melanogaster GCA_000008025.1_ASM802v1
Candidatus.Deianiraea.vastatrix GCA_007993655.1_ASM799365v1
Rickettsiales.endosymbiont.of.Trichoplax.sp.H2 GCA_009690945.1
Candidatus.Midichloria.mitochondrii.IricVA GCA_000219355.1_ASM21935v1
Rickettsiales.endosymbiont.of.Peranema.trichophorum GCA_004210275.1_ASM421027v1
Candidatus.Jidaibacter.acanthamoeba GCA_000815465.1.UWC36
Rickettsia.bellii.RML369.C GCA_000012385.1_ASM1238v1
Candidatus.Megaira.polyxenophila
Rickettsiales.endosymbiont.of.Stachyamoeba.lipophora GCA_003932735.1_ASM393273v1
Rickettsiales.bacterium.Ac37b GCA_000746585.2_ASM74658v2
Magnetofaba.australis.IT.1 GCA_002109495.1_ASM210949v1
Magnetococcus.marinus.MC.1 GCA_000014865.1_ASM1486v1
Nitrosospira.briensis.C.128 GCA_000619905.2_ASM61990v2
Methylotenera.versatilis.301 GCA_000093025.1_ASM9302v1
Ralstonia.pickettii GCA_902374465.1.MGYG.HGUT.01384
Burkholderia.cepacia GCA_009586235.1_ASM958623v1
Dasania.marina.DSM.21967 GCA_000373485.1_ASM37348v1
Congregibacter.litoralis.KT71 GCA_000153125.2_ASM15312v2
Enterobacter.cloacae GCA_000770155.1_ASM77015v1
Alteromonas.macleodii.ATCC.27126 GCA_000172635.2_ASM17263v2
0.719899

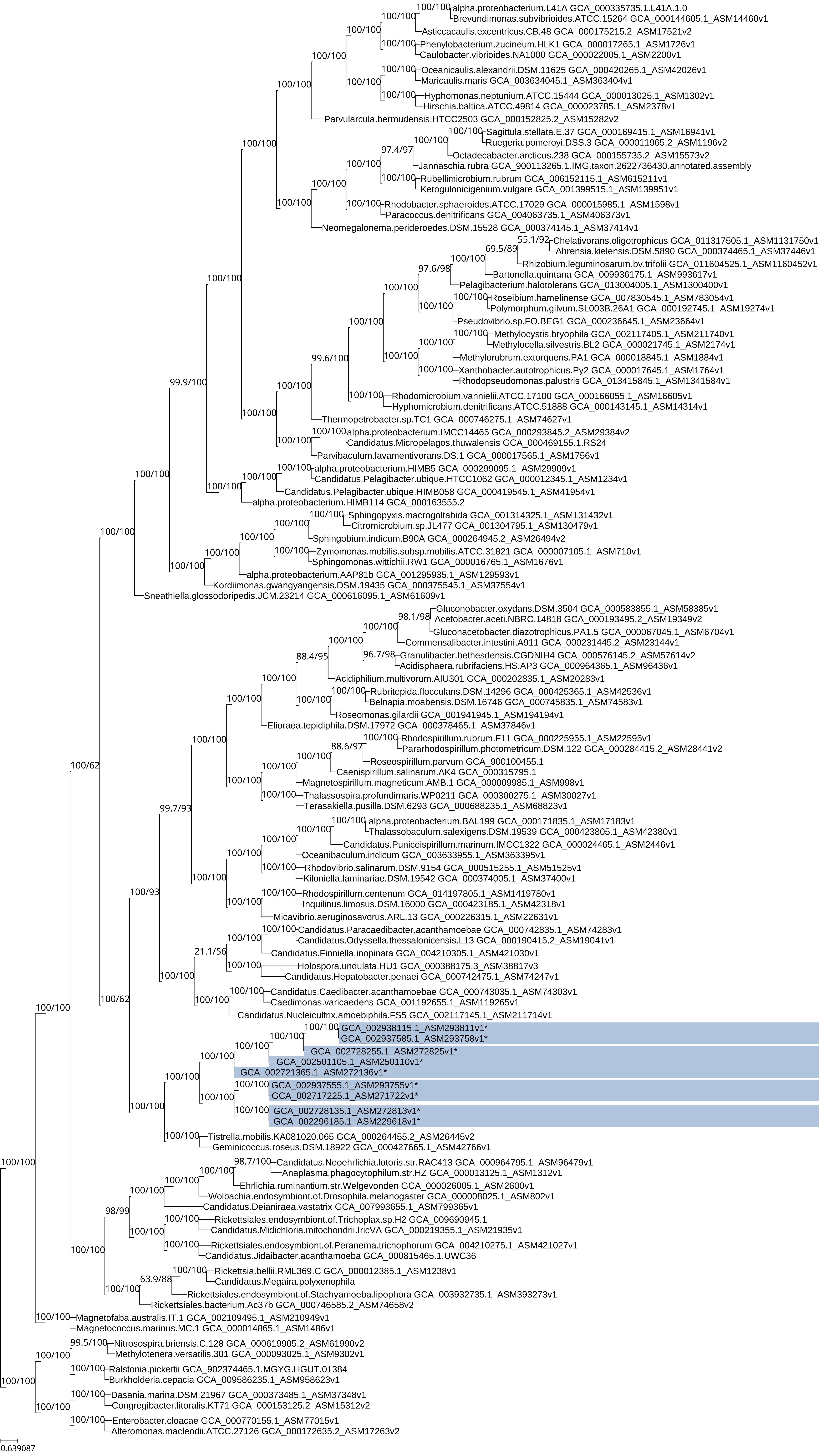

100/100
alpha.proteobacterium.L41A GCA_000335735.1.L41A.1.0
100/100
Brevundimonas.subvibrioides.ATCC.15264 GCA_000144605.1_ASM14460v1
100/100
Asticcacaulis.excentricus.CB.48 GCA_000175215.2_ASM17521v2
100/100
Phenylobacterium.zucineum.HLK1 GCA_000017265.1_ASM1726v1
100/100
Caulobacter.vibrioides.NA1000 GCA_000022005.1_ASM2200v1
100/100
Oceanicaulis.alexandrii.DSM.11625 GCA_000420265.1_ASM42026v1
100/100
Maricaulis.maris GCA_003634045.1_ASM363404v1
100/100
Hyphomonas.neptunium.ATCC.15444 GCA_000013025.1_ASM1302v1
100/100
Hirschia.baltica.ATCC.49814 GCA_000023785.1_ASM2378v1
Parvularcula.bermudensis.HTCC2503 GCA_000152825.2_ASM15282v2
100/100
Sagittula.stellata.E.37 GCA_000169415.1_ASM16941v1
100/100
Ruegeria.pomeroyi.DSS.3 GCA_000011965.2_ASM1196v2
97.4/97
Octadecabacter.arcticus.238 GCA_000155735.2_ASM15573v2
100/100
Jannaschia.rubra GCA_900113265.1.IMG.taxon.2622736430.annotated.assembly
100/100
Rubellimicrobium.rubrum GCA_006152115.1_ASM615211v1
100/100
Ketogulonicigenium.vulgare GCA_001399515.1_ASM139951v1
100/100
Rhodobacter.sphaeroides.ATCC.17029 GCA_000015985.1_ASM1598v1
100/100
Paracoccus.denitrificans GCA_004063735.1_ASM406373v1
Neomegalonema.perideroedes.DSM.15528 GCA_000374145.1_ASM37414v1
55.1/92
Chelativorans.oligotrophicus GCA_011317505.1_ASM1131750v1
69.5/89
Ahrensia.kielensis.DSM.5890 GCA_000374465.1_ASM37446v1
100/100
Rhizobium.leguminosarum.bv.trifolii GCA_011604525.1_ASM1160452v1
97.6/98
Bartonella.quintana GCA_009936175.1_ASM993617v1
Pelagibacterium.halotolerans GCA_013004005.1_ASM1300400v1
100/100
100/100
Roseibium.hamelinense GCA_007830545.1_ASM783054v1
100/100
Polymorphum.gilvum.SL003B.26A1 GCA_000192745.1_ASM19274v1
100/100
Pseudovibrio.sp.FO.BEG1 GCA_000236645.1_ASM23664v1
100/100
Methylocystis.bryophila GCA_002117405.1_ASM211740v1
100/100
Methylocella.silvestris.BL2 GCA_000021745.1_ASM2174v1
99.6/100
100/100
Methylorubrum.extorquens.PA1 GCA_000018845.1_ASM1884v1
100/100
Xanthobacter.autotrophicus.Py2 GCA_000017645.1_ASM1764v1
99.9/100
Rhodopseudomonas.palustris GCA_013415845.1_ASM1341584v1
100/100
100/100
Rhodomicrobium.vannielii.ATCC.17100 GCA_000166055.1_ASM16605v1
Hyphomicrobium.denitrificans.ATCC.51888 GCA_000143145.1_ASM14314v1
100/100
Thermopetrobacter.sp.TC1 GCA_000746275.1_ASM74627v1
100/100
alpha.proteobacterium.IMCC14465 GCA_000293845.2_ASM29384v2
100/100
Candidatus.Micropelagos.thuwalensis GCA_000469155.1.RS24
Parvibaculum.lavamentivorans.DS.1 GCA_000017565.1_ASM1756v1
100/100
100/100
alpha.proteobacterium.HIMB5 GCA_000299095.1_ASM29909v1
100/100
Candidatus.Pelagibacter.ubique.HTCC1062 GCA_000012345.1_ASM1234v1
100/100
Candidatus.Pelagibacter.ubique.HIMB058 GCA_000419545.1_ASM41954v1
alpha.proteobacterium.HIMB114 GCA_000163555.2
100/100
Sphingopyxis.macrogoltabida GCA_001314325.1_ASM131432v1
100/100
100/100
Citromicrobium.sp.JL477 GCA_001304795.1_ASM130479v1
100/100
Sphingobium.indicum.B90A GCA_000264945.2_ASM26494v2
100/100
100/100
Zymomonas.mobilis.subsp.mobilis.ATCC.31821 GCA_000007105.1_ASM710v1
100/100
Sphingomonas.wittichii.RW1 GCA_000016765.1_ASM1676v1
alpha.proteobacterium.AAP81b GCA_001295935.1_ASM129593v1
Kordiimonas.gwangyangensis.DSM.19435 GCA_000375545.1_ASM37554v1
Sneathiella.glossodoripedis.JCM.23214 GCA_000616095.1_ASM61609v1
98.1/98
Gluconobacter.oxydans.DSM.3504 GCA_000583855.1_ASM58385v1
Acetobacter.aceti.NBRC.14818 GCA_000193495.2_ASM19349v2
100/100
Gluconacetobacter.diazotrophicus.PA1.5 GCA_000067045.1_ASM6704v1
100/100
Commensalibacter.intestini.A911 GCA_000231445.2_ASM23144v1
96.7/98
88.4/95
Granulibacter.bethesdensis.CGDNIH4 GCA_000576145.2_ASM57614v2
Acidisphaera.rubrifaciens.HS.AP3 GCA_000964365.1_ASM96436v1
100/100
Acidiphilium.multivorum.AIU301 GCA_000202835.1_ASM20283v1
100/100
Rubritepida.flocculans.DSM.14296 GCA_000425365.1_ASM42536v1
100/100
100/100
Belnapia.moabensis.DSM.16746 GCA_000745835.1_ASM74583v1
Roseomonas.gilardii GCA_001941945.1_ASM194194v1
100/100
Elioraea.tepidiphila.DSM.17972 GCA_000378465.1_ASM37846v1
100/100
Rhodospirillum.rubrum.F11 GCA_000225955.1_ASM22595v1
88.6/97
Pararhodospirillum.photometricum.DSM.122 GCA_000284415.2_ASM28441v2
100/100
Roseospirillum.parvum GCA_900100455.1
100/100
Caenispirillum.salinarum.AK4 GCA_000315795.1
100/100
Magnetospirillum.magneticum.AMB.1 GCA_000009985.1_ASM998v1
100/100
Thalassospira.profundimaris.WP0211 GCA_000300275.1_ASM30027v1
99.7/93
Terasakiella.pusilla.DSM.6293 GCA_000688235.1_ASM68823v1
100/100
alpha.proteobacterium.BAL199 GCA_000171835.1_ASM17183v1
100/100
Thalassobaculum.salexigens.DSM.19539 GCA_000423805.1_ASM42380v1
100/100
Candidatus.Puniceispirillum.marinum.IMCC1322 GCA_000024465.1_ASM2446v1
100/100
Oceanibaculum.indicum GCA_003633955.1_ASM363395v1
100/100
Rhodovibrio.salinarum.DSM.9154 GCA_000515255.1_ASM51525v1
100/100
Kiloniella.laminariae.DSM.19542 GCA_000374005.1_ASM37400v1
100/100
Rhodospirillum.centenum GCA_014197805.1_ASM1419780v1
100/93
100/100
Inquilinus.limosus.DSM.16000 GCA_000423185.1_ASM42318v1
Micavibrio.aeruginosavorus.ARL.13 GCA_000226315.1_ASM22631v1
100/100
Candidatus.Paracaedibacter.acanthamoebae GCA_000742835.1_ASM74283v1
21.1/56
100/100
Candidatus.Odyssella.thessalonicensis.L13 GCA_000190415.2_ASM19041v1
Candidatus.Finniella.inopinata GCA_004210305.1_ASM421030v1
100/100
Holospora.undulata.HU1 GCA_000388175.3_ASM38817v3
100/100
Candidatus.Hepatobacter.penaei GCA_000742475.1_ASM74247v1
100/100
Candidatus.Caedibacter.acanthamoebae GCA_000743035.1_ASM74303v1
100/100
Caedimonas.varicaedens GCA_001192655.1_ASM119265v1
100/62
Candidatus.Nucleicultrix.amoebiphila.FS5 GCA_002117145.1_ASM211714v1
100/100
GCA_002938115.1_ASM293811v1*
100/100
GCA_002937585.1_ASM293758v1*
100/100
GCA_002728255.1_ASM272825v1*
100/100
GCA_002501105.1_ASM250110v1*
GCA_002721365.1_ASM272136v1*
100/100
100/100
GCA_002937555.1_ASM293755v1*
100/100
GCA_002717225.1_ASM271722v1*
100/100
GCA_002728135.1_ASM272813v1*
GCA_002296185.1_ASM229618v1*
100/100
Tistrella.mobilis.KA081020.065 GCA_000264455.2_ASM26445v2
Geminicoccus.roseus.DSM.18922 GCA_000427665.1_ASM42766v1
100/100
98.7/100
Candidatus.Neoehrlichia.lotoris.str.RAC413 GCA_000964795.1_ASM96479v1
100/100
Anaplasma.phagocytophilum.str.HZ GCA_000013125.1_ASM1312v1
100/100
Ehrlichia.ruminantium.str.Welgevonden GCA_000026005.1_ASM2600v1
100/100
Wolbachia.endosymbiont.of.Drosophila.melanogaster GCA_000008025.1_ASM802v1
98/99
Candidatus.Deianiraea.vastatrix GCA_007993655.1_ASM799365v1
100/100
Rickettsiales.endosymbiont.of.Trichoplax.sp.H2 GCA_009690945.1
100/100
Candidatus.Midichloria.mitochondrii.IricVA GCA_000219355.1_ASM21935v1
100/100
Rickettsiales.endosymbiont.of.Peranema.trichophorum GCA_004210275.1_ASM421027v1
100/100
Candidatus.Jidaibacter.acanthamoeba GCA_000815465.1.UWC36
100/100
Rickettsia.bellii.RML369.C GCA_000012385.1_ASM1238v1
63.9/88
Candidatus.Megaira.polyxenophila
100/100
Rickettsiales.endosymbiont.of.Stachyamoeba.lipophora GCA_003932735.1_ASM393273v1
Rickettsiales.bacterium.Ac37b GCA_000746585.2_ASM74658v2
100/100
Magnetofaba.australis.IT.1 GCA_002109495.1_ASM210949v1
Magnetococcus.marinus.MC.1 GCA_000014865.1_ASM1486v1
99.5/100
Nitrosospira.briensis.C.128 GCA_000619905.2_ASM61990v2
100/100
Methylotenera.versatilis.301 GCA_000093025.1_ASM9302v1
100/100
Ralstonia.pickettii GCA_902374465.1.MGYG.HGUT.01384
Burkholderia.cepacia GCA_009586235.1_ASM958623v1
100/100
100/100
Dasania.marina.DSM.21967 GCA_000373485.1_ASM37348v1
Congregibacter.litoralis.KT71 GCA_000153125.2_ASM15312v2
100/100
100/100
Enterobacter.cloacae GCA_000770155.1_ASM77015v1
Alteromonas.macleodii.ATCC.27126 GCA_000172635.2_ASM17263v2
0.639087

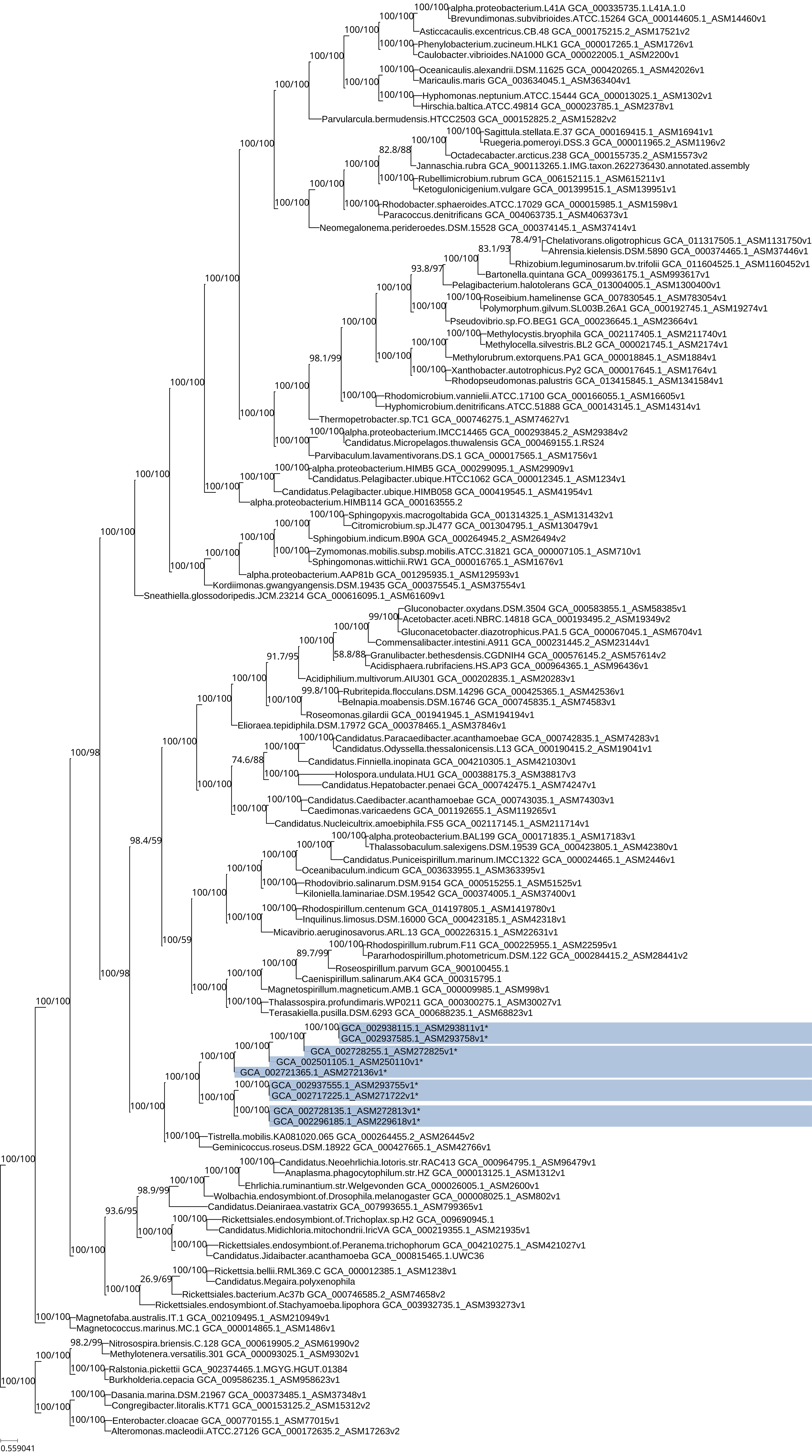
100/100
alpha.proteobacterium.L41A GCA_000335735.1.L41A.1.0
100/100
Brevundimonas.subvibrioides.ATCC.15264 GCA_000144605.1_ASM14460v1
100/100
Asticcacaulis.excentricus.CB.48 GCA_000175215.2_ASM17521v2
100/100
Phenylobacterium.zucineum.HLK1 GCA_000017265.1_ASM1726v1
100/100
Caulobacter.vibrioides.NA1000 GCA_000022005.1_ASM2200v1
100/100
Oceanicaulis.alexandrii.DSM.11625 GCA_000420265.1_ASM42026v1
100/100
Maricaulis.maris GCA_003634045.1_ASM363404v1
100/100
100/100
Hyphomonas.neptunium.ATCC.15444 GCA_000013025.1_ASM1302v1
Hirschia.baltica.ATCC.49814 GCA_000023785.1_ASM2378v1
Parvularcula.bermudensis.HTCC2503 GCA_000152825.2_ASM15282v2
100/100
100/100
Sagittula.stellata.E.37 GCA_000169415.1_ASM16941v1
100/100
Ruegeria.pomeroyi.DSS.3 GCA_000011965.2_ASM1196v2
82.8/88
Octadecabacter.arcticus.238 GCA_000155735.2_ASM15573v2
100/100
Jannaschia.rubra GCA_900113265.1.IMG.taxon.2622736430.annotated.assembly
100/100
Rubellimicrobium.rubrum GCA_006152115.1_ASM615211v1
100/100
Ketogulonicigenium.vulgare GCA_001399515.1_ASM139951v1
100/100
100/100
Rhodobacter.sphaeroides.ATCC.17029 GCA_000015985.1_ASM1598v1
Paracoccus.denitrificans GCA_004063735.1_ASM406373v1
Neomegalonema.perideroedes.DSM.15528 GCA_000374145.1_ASM37414v1
78.4/91
Chelativorans.oligotrophicus GCA_011317505.1_ASM1131750v1
83.1/93
Ahrensia.kielensis.DSM.5890 GCA_000374465.1_ASM37446v1
100/100
Rhizobium.leguminosarum.bv.trifolii GCA_011604525.1_ASM1160452v1
100/100
Bartonella.quintana GCA_009936175.1_ASM993617v1
93.8/97
Pelagibacterium.halotolerans GCA_013004005.1_ASM1300400v1
100/100
100/100
Roseibium.hamelinense GCA_007830545.1_ASM783054v1
100/100
Polymorphum.gilvum.SL003B.26A1 GCA_000192745.1_ASM19274v1
Pseudovibrio.sp.FO.BEG1 GCA_000236645.1_ASM23664v1
100/100
100/100
Methylocystis.bryophila GCA_002117405.1_ASM211740v1
100/100
Methylocella.silvestris.BL2 GCA_000021745.1_ASM2174v1
98.1/99
100/100
Methylorubrum.extorquens.PA1 GCA_000018845.1_ASM1884v1
100/100
Xanthobacter.autotrophicus.Py2 GCA_000017645.1_ASM1764v1
100/100
Rhodopseudomonas.palustris GCA_013415845.1_ASM1341584v1
100/100
100/100
Rhodomicrobium.vannielii.ATCC.17100 GCA_000166055.1_ASM16605v1
Hyphomicrobium.denitrificans.ATCC.51888 GCA_000143145.1_ASM14314v1
100/100
Thermopetrobacter.sp.TC1 GCA_000746275.1_ASM74627v1
100/100
alpha.proteobacterium.IMCC14465 GCA_000293845.2_ASM29384v2
100/100
Candidatus.Micropelagos.thuwalensis GCA_000469155.1.RS24
Parvibaculum.lavamentivorans.DS.1 GCA_000017565.1_ASM1756v1
100/100
alpha.proteobacterium.HIMB5 GCA_000299095.1_ASM29909v1
100/100
Candidatus.Pelagibacter.ubique.HTCC1062 GCA_000012345.1_ASM1234v1
100/100
Candidatus.Pelagibacter.ubique.HIMB058 GCA_000419545.1_ASM41954v1
100/100
alpha.proteobacterium.HIMB114 GCA_000163555.2
100/100
Sphingopyxis.macrogoltabida GCA_001314325.1_ASM131432v1
100/100
Citromicrobium.sp.JL477 GCA_001304795.1_ASM130479v1
100/100
Sphingobium.indicum.B90A GCA_000264945.2_ASM26494v2
100/100
100/100
Zymomonas.mobilis.subsp.mobilis.ATCC.31821 GCA_000007105.1_ASM710v1
Sphingomonas.wittichii.RW1 GCA_000016765.1_ASM1676v1
100/100
alpha.proteobacterium.AAP81b GCA_001295935.1_ASM129593v1
Kordiimonas.gwangyangensis.DSM.19435 GCA_000375545.1_ASM37554v1
Sneathiella.glossodoripedis.JCM.23214 GCA_000616095.1_ASM61609v1
Gluconobacter.oxydans.DSM.3504 GCA_000583855.1_ASM58385v1
99/100
Acetobacter.aceti.NBRC.14818 GCA_000193495.2_ASM19349v2
100/100
Gluconacetobacter.diazotrophicus.PA1.5 GCA_000067045.1_ASM6704v1
100/100
Commensalibacter.intestini.A911 GCA_000231445.2_ASM23144v1
91.7/95
58.8/88
Granulibacter.bethesdensis.CGDNIH4 GCA_000576145.2_ASM57614v2
Acidisphaera.rubrifaciens.HS.AP3 GCA_000964365.1_ASM96436v1
100/100
Acidiphilium.multivorum.AIU301 GCA_000202835.1_ASM20283v1
99.8/100
Rubritepida.flocculans.DSM.14296 GCA_000425365.1_ASM42536v1
100/100
100/100
Belnapia.moabensis.DSM.16746 GCA_000745835.1_ASM74583v1
Roseomonas.gilardii GCA_001941945.1_ASM194194v1
100/100
Elioraea.tepidiphila.DSM.17972 GCA_000378465.1_ASM37846v1
100/100
Candidatus.Paracaedibacter.acanthamoebae GCA_000742835.1_ASM74283v1
100/100
Candidatus.Odyssella.thessalonicensis.L13 GCA_000190415.2_ASM19041v1
74.6/88
Candidatus.Finniella.inopinata GCA_004210305.1_ASM421030v1
100/100
Holospora.undulata.HU1 GCA_000388175.3_ASM38817v3
100/98
100/100
Candidatus.Hepatobacter.penaei GCA_000742475.1_ASM74247v1
100/100
Candidatus.Caedibacter.acanthamoebae GCA_000743035.1_ASM74303v1
100/100
Caedimonas.varicaedens GCA_001192655.1_ASM119265v1
Candidatus.Nucleicultrix.amoebiphila.FS5 GCA_002117145.1_ASM211714v1
100/100
alpha.proteobacterium.BAL199 GCA_000171835.1_ASM17183v1
98.4/59
100/100
Thalassobaculum.salexigens.DSM.19539 GCA_000423805.1_ASM42380v1
100/100
Candidatus.Puniceispirillum.marinum.IMCC1322 GCA_000024465.1_ASM2446v1
100/100
Oceanibaculum.indicum GCA_003633955.1_ASM363395v1
100/100
Rhodovibrio.salinarum.DSM.9154 GCA_000515255.1_ASM51525v1
100/100
Kiloniella.laminariae.DSM.19542 GCA_000374005.1_ASM37400v1
100/100
Rhodospirillum.centenum GCA_014197805.1_ASM1419780v1
100/100
Inquilinus.limosus.DSM.16000 GCA_000423185.1_ASM42318v1
Micavibrio.aeruginosavorus.ARL.13 GCA_000226315.1_ASM22631v1
100/59
100/100
Rhodospirillum.rubrum.F11 GCA_000225955.1_ASM22595v1
89.7/99
Pararhodospirillum.photometricum.DSM.122 GCA_000284415.2_ASM28441v2
100/100
Roseospirillum.parvum GCA_900100455.1
100/100
Caenispirillum.salinarum.AK4 GCA_000315795.1
100/98
100/100
Magnetospirillum.magneticum.AMB.1 GCA_000009985.1_ASM998v1
100/100
Thalassospira.profundimaris.WP0211 GCA_000300275.1_ASM30027v1
Terasakiella.pusilla.DSM.6293 GCA_000688235.1_ASM68823v1
100/100
100/100
GCA_002938115.1_ASM293811v1*
GCA_002937585.1_ASM293758v1*
100/100
100/100
GCA_002728255.1_ASM272825v1*
GCA_002501105.1_ASM250110v1*
GCA_002721365.1_ASM272136v1*
100/100
100/100
GCA_002937555.1_ASM293755v1*
100/100
GCA_002717225.1_ASM271722v1*
100/100
100/100
GCA_002728135.1_ASM272813v1*
GCA_002296185.1_ASM229618v1*
100/100
Tistrella.mobilis.KA081020.065 GCA_000264455.2_ASM26445v2
Geminicoccus.roseus.DSM.18922 GCA_000427665.1_ASM42766v1
100/100
100/100
Candidatus.Neoehrlichia.lotoris.str.RAC413 GCA_000964795.1_ASM96479v1
100/100
Anaplasma.phagocytophilum.str.HZ GCA_000013125.1_ASM1312v1
100/100
Ehrlichia.ruminantium.str.Welgevonden GCA_000026005.1_ASM2600v1
98.9/99
Wolbachia.endosymbiont.of.Drosophila.melanogaster GCA_000008025.1_ASM802v1
Candidatus.Deianiraea.vastatrix GCA_007993655.1_ASM799365v1
93.6/95
100/100
Rickettsiales.endosymbiont.of.Trichoplax.sp.H2 GCA_009690945.1
100/100
Candidatus.Midichloria.mitochondrii.IricVA GCA_000219355.1_ASM21935v1
100/100
Rickettsiales.endosymbiont.of.Peranema.trichophorum GCA_004210275.1_ASM421027v1
100/100
Candidatus.Jidaibacter.acanthamoeba GCA_000815465.1.UWC36
100/100
Rickettsia.bellii.RML369.C GCA_000012385.1_ASM1238v1
26.9/69
Candidatus.Megaira.polyxenophila
100/100
Rickettsiales.bacterium.Ac37b GCA_000746585.2_ASM74658v2
Rickettsiales.endosymbiont.of.Stachyamoeba.lipophora GCA_003932735.1_ASM393273v1
100/100
Magnetofaba.australis.IT.1 GCA_002109495.1_ASM210949v1
Magnetococcus.marinus.MC.1 GCA_000014865.1_ASM1486v1
98.2/99
Nitrosospira.briensis.C.128 GCA_000619905.2_ASM61990v2
100/100
Methylotenera.versatilis.301 GCA_000093025.1_ASM9302v1
100/100
Ralstonia.pickettii GCA_902374465.1.MGYG.HGUT.01384
Burkholderia.cepacia GCA_009586235.1_ASM958623v1
100/100
100/100
Dasania.marina.DSM.21967 GCA_000373485.1_ASM37348v1
100/100
Congregibacter.litoralis.KT71 GCA_000153125.2_ASM15312v2
100/100
Enterobacter.cloacae GCA_000770155.1_ASM77015v1
Alteromonas.macleodii.ATCC.27126 GCA_000172635.2_ASM17263v2
0.559041

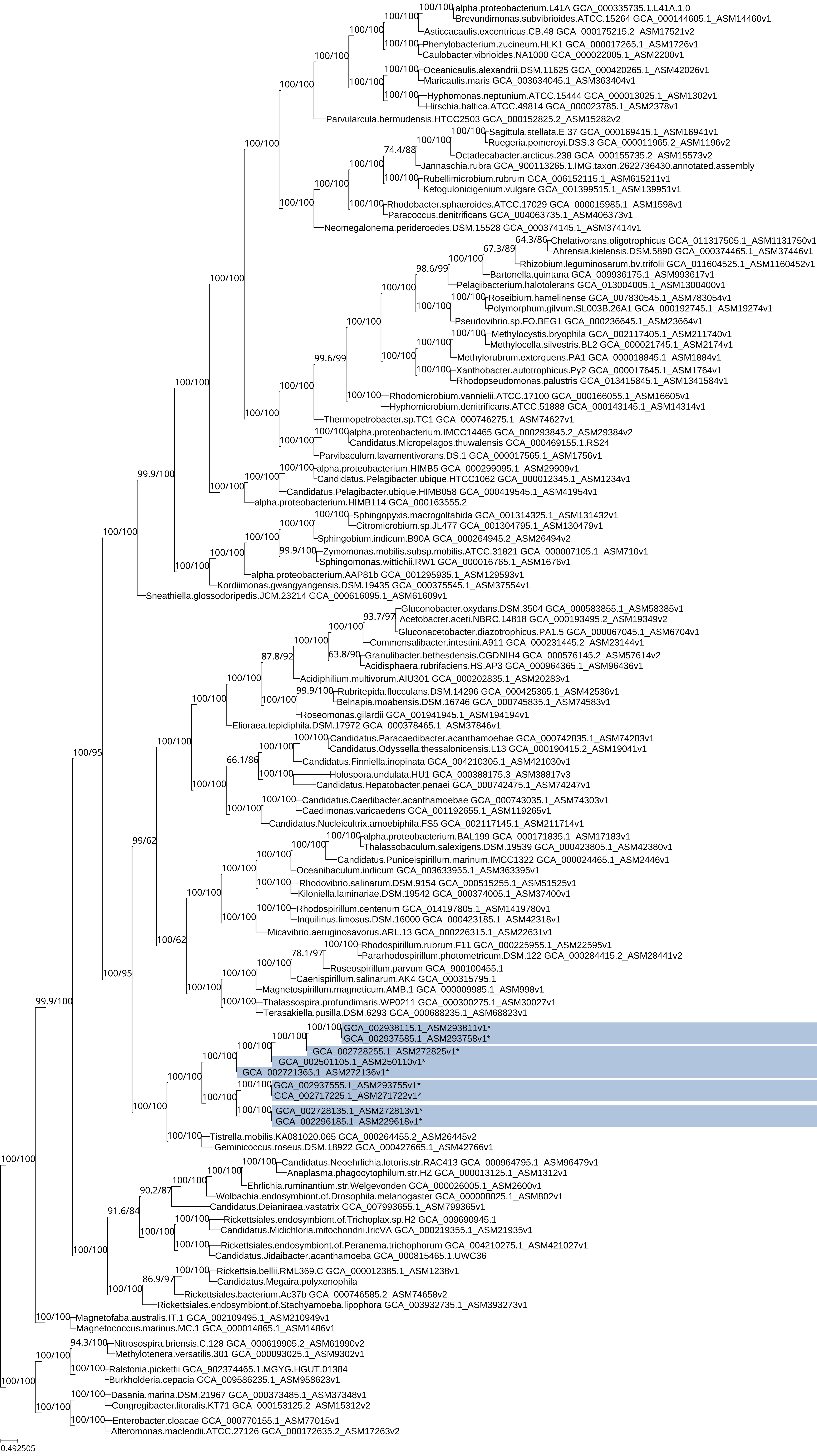

alpha.proteobacterium.L41A GCA_000335735.1.L41A.1.0
Brevundimonas.subvibrioides.ATCC.15264 GCA_000144605.1_ASM14460v1
Asticcacaulis.excentricus.CB.48 GCA_000175215.2_ASM17521v2
Phenylobacterium.zucineum.HLK1 GCA_000017265.1_ASM1726v1
Caulobacter.vibrioides.NA1000 GCA_000022005.1_ASM2200v1
Oceanicaulis.alexandrii.DSM.11625 GCA_000420265.1_ASM42026v1
Maricaulis.maris GCA_003634045.1_ASM363404v1
Hyphomonas.neptunium.ATCC.15444 GCA_000013025.1_ASM1302v1
Hirschia.baltica.ATCC.49814 GCA_000023785.1_ASM2378v1
Parvularcula.bermudensis.HTCC2503 GCA_000152825.2_ASM15282v2
Sagittula.stellata.E.37 GCA_000169415.1_ASM16941v1
Ruegeria.pomeroyi.DSS.3 GCA_000011965.2_ASM1196v2
Octadecabacter.arcticus.238 GCA_000155735.2_ASM15573v2
Jannaschia.rubra GCA_900113265.1.IMG.taxon.2622736430.annotated.assembly
Rubellimicrobium.rubrum GCA_006152115.1_ASM615211v1
Ketogulonicigenium.vulgare GCA_001399515.1_ASM139951v1
Rhodobacter.sphaeroides.ATCC.17029 GCA_000015985.1_ASM1598v1
Paracoccus.denitrificans GCA_004063735.1_ASM406373v1
Neomegalonema.perideroedes.DSM.15528 GCA_000374145.1_ASM37414v1
Chelativorans.oligotrophicus GCA_011317505.1_ASM1131750v1
Ahrensia.kielensis.DSM.5890 GCA_000374465.1_ASM37446v1
Rhizobium.leguminosarum.bv.trifolii GCA_011604525.1_ASM1160452v1
Bartonella.quintana GCA_009936175.1_ASM993617v1
Pelagibacterium.halotolerans GCA_013004005.1_ASM1300400v1
Roseibium.hamelinense GCA_007830545.1_ASM783054v1
Polymorphum.gilvum.SL003B.26A1 GCA_000192745.1_ASM19274v1
Pseudovibrio.sp.FO.BEG1 GCA_000236645.1_ASM23664v1
Methylocystis.bryophila GCA_002117405.1_ASM211740v1
Methylocella.silvestris.BL2 GCA_000021745.1_ASM2174v1
Methylorubrum.extorquens.PA1 GCA_000018845.1_ASM1884v1
Xanthobacter.autotrophicus.Py2 GCA_000017645.1_ASM1764v1
Rhodopseudomonas.palustris GCA_013415845.1_ASM1341584v1
Rhodomicrobium.vannielii.ATCC.17100 GCA_000166055.1_ASM16605v1
Hyphomicrobium.denitrificans.ATCC.51888 GCA_000143145.1_ASM14314v1
Thermopetrobacter.sp.TC1 GCA_000746275.1_ASM74627v1
alpha.proteobacterium.IMCC14465 GCA_000293845.2_ASM29384v2
Candidatus.Micropelagos.thuwalensis GCA_000469155.1.RS24
Parvibaculum.lavamentivorans.DS.1 GCA_000017565.1_ASM1756v1
alpha.proteobacterium.HIMB5 GCA_000299095.1_ASM29909v1
Candidatus.Pelagibacter.ubique.HTCC1062 GCA_000012345.1_ASM1234v1
Candidatus.Pelagibacter.ubique.HIMB058 GCA_000419545.1_ASM41954v1
alpha.proteobacterium.HIMB114 GCA_000163555.2
Sphingopyxis.macrogoltabida GCA_001314325.1_ASM131432v1
Citromicrobium.sp.JL477 GCA_001304795.1_ASM130479v1
Sphingobium.indicum.B90A GCA_000264945.2_ASM26494v2
Zymomonas.mobilis.subsp.mobilis.ATCC.31821 GCA_000007105.1_ASM710v1
Sphingomonas.wittichii.RW1 GCA_000016765.1_ASM1676v1
alpha.proteobacterium.AAP81b GCA_001295935.1_ASM129593v1
Kordiimonas.gwangyangensis.DSM.19435 GCA_000375545.1_ASM37554v1
Sneathiella.glossodoripedis.JCM.23214 GCA_000616095.1_ASM61609v1
Gluconobacter.oxydans.DSM.3504 GCA_000583855.1_ASM58385v1
Acetobacter.aceti.NBRC.14818 GCA_000193495.2_ASM19349v2
Gluconacetobacter.diazotrophicus.PA1.5 GCA_000067045.1_ASM6704v1
Commensalibacter.intestini.A911 GCA_000231445.2_ASM23144v1
Granulibacter.bethesdensis.CGDNIH4 GCA_000576145.2_ASM57614v2
Acidisphaera.rubrifaciens.HS.AP3 GCA_000964365.1_ASM96436v1
Acidiphilium.multivorum.AIU301 GCA_000202835.1_ASM20283v1
Rubritepida.flocculans.DSM.14296 GCA_000425365.1_ASM42536v1
Belnapia.moabensis.DSM.16746 GCA_000745835.1_ASM74583v1
Roseomonas.gilardii GCA_001941945.1_ASM194194v1
Elioraea.tepidiphila.DSM.17972 GCA_000378465.1_ASM37846v1
Candidatus.Paracaedibacter.acanthamoebae GCA_000742835.1_ASM74283v1
Candidatus.Odyssella.thessalonicensis.L13 GCA_000190415.2_ASM19041v1
Candidatus.Finniella.inopinata GCA_004210305.1_ASM421030v1
Holospora.undulata.HU1 GCA_000388175.3_ASM38817v3
Candidatus.Hepatobacter.penaei GCA_000742475.1_ASM74247v1
Candidatus.Caedibacter.acanthamoebae GCA_000743035.1_ASM74303v1
Caedimonas.varicaedens GCA_001192655.1_ASM119265v1
Candidatus.Nucleicultrix.amoebiphila.FS5 GCA_002117145.1_ASM211714v1
alpha.proteobacterium.BAL199 GCA_000171835.1_ASM17183v1
Thalassobaculum.salexigens.DSM.19539 GCA_000423805.1_ASM42380v1
Candidatus.Puniceispirillum.marinum.IMCC1322 GCA_000024465.1_ASM2446v1
Oceanibaculum.indicum GCA_003633955.1_ASM363395v1
Rhodovibrio.salinarum.DSM.9154 GCA_000515255.1_ASM51525v1
Kiloniella.laminariae.DSM.19542 GCA_000374005.1_ASM37400v1
Rhodospirillum.centenum GCA_014197805.1_ASM1419780v1
Inquilinus.limosus.DSM.16000 GCA_000423185.1_ASM42318v1
Micavibrio.aeruginosavorus.ARL.13 GCA_000226315.1_ASM22631v1
Rhodospirillum.rubrum.F11 GCA_000225955.1_ASM22595v1
Pararhodospirillum.photometricum.DSM.122 GCA_000284415.2_ASM28441v2
Roseospirillum.parvum GCA_900100455.1
Caenispirillum.salinarum.AK4 GCA_000315795.1
Magnetospirillum.magneticum.AMB.1 GCA_000009985.1_ASM998v1
Thalassospira.profundimaris.WP0211 GCA_000300275.1_ASM30027v1
Terasakiella.pusilla.DSM.6293 GCA_000688235.1_ASM68823v1
GCA_002938115.1_ASM293811v1*
GCA_002937585.1_ASM293758v1*
GCA_002728255.1_ASM272825v1*
GCA_002501105.1_ASM250110v1*
GCA_002721365.1_ASM272136v1*
GCA_002937555.1_ASM293755v1*
GCA_002717225.1_ASM271722v1*
GCA_002728135.1_ASM272813v1*
GCA_002296185.1_ASM229618v1*
Tistrella.mobilis.KA081020.065 GCA_000264455.2_ASM26445v2
Geminicoccus.roseus.DSM.18922 GCA_000427665.1_ASM42766v1
Candidatus.Neoehrlichia.lotoris.str.RAC413 GCA_000964795.1_ASM96479v1
Anaplasma.phagocytophilum.str.HZ GCA_000013125.1_ASM1312v1
Ehrlichia.ruminantium.str.Welgevonden GCA_000026005.1_ASM2600v1
Wolbachia.endosymbiont.of.Drosophila.melanogaster GCA_000008025.1_ASM802v1
Candidatus.Deianiraea.vastatrix GCA_007993655.1_ASM799365v1
Rickettsiales.endosymbiont.of.Trichoplax.sp.H2 GCA_009690945.1
Candidatus.Midichloria.mitochondrii.IricVA GCA_000219355.1_ASM21935v1
Rickettsiales.endosymbiont.of.Peranema.trichophorum GCA_004210275.1_ASM421027v1
Candidatus.Jidaibacter.acanthamoeba GCA_000815465.1.UWC36
Rickettsia.bellii.RML369.C GCA_000012385.1_ASM1238v1
Candidatus.Megaira.polyxenophila
Rickettsiales.bacterium.Ac37b GCA_000746585.2_ASM74658v2
Rickettsiales.endosymbiont.of.Stachyamoeba.lipophora GCA_003932735.1_ASM393273v1
Magnetofaba.australis.IT.1 GCA_002109495.1_ASM210949v1
Magnetococcus.marinus.MC.1 GCA_000014865.1_ASM1486v1
Nitrosospira.briensis.C.128 GCA_000619905.2_ASM61990v2
Methylotenera.versatilis.301 GCA_000093025.1_ASM9302v1
Ralstonia.pickettii GCA_902374465.1.MGYG.HGUT.01384
Burkholderia.cepacia GCA_009586235.1_ASM958623v1
Dasania.marina.DSM.21967 GCA_000373485.1_ASM37348v1
Congregibacter.litoralis.KT71 GCA_000153125.2_ASM15312v2
Enterobacter.cloacae GCA_000770155.1_ASM77015v1
Alteromonas.macleodii.ATCC.27126 GCA_000172635.2_ASM17263v2
0.492505

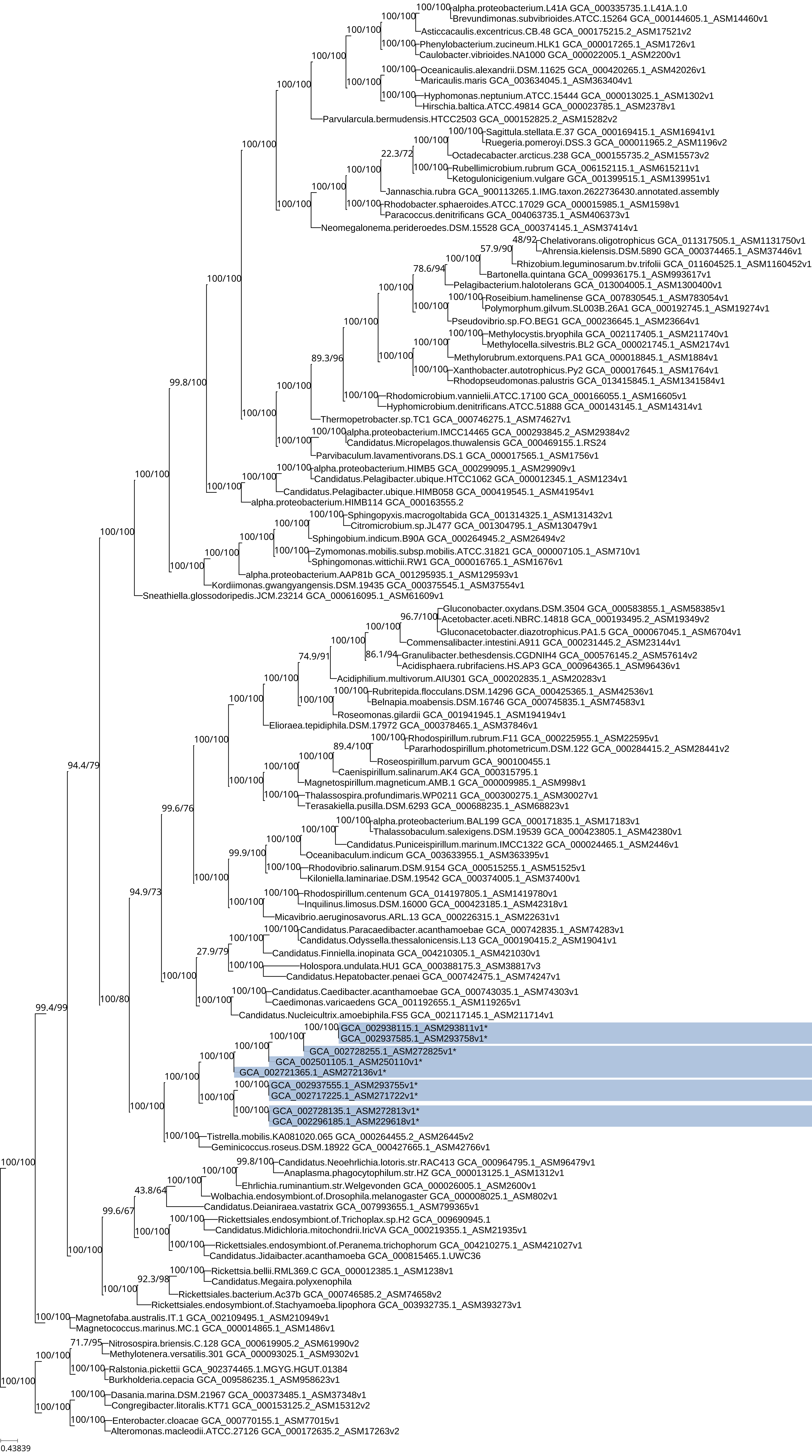

100/100 alpha.proteobacterium.L41A GCA_000335735.1.L41A.1.0
Brevundimonas.subvibrioides.ATCC.15264 GCA_000144605.1_ASM14460v1
100/100 Asticcacaulis.excentricus.CB.48 GCA_000175215.2_ASM17521v2
100/100
100/100 Phenylobacterium.zucineum.HLK1 GCA_000017265.1_ASM1726v1
Caulobacter.vibrioides.NA1000 GCA_000022005.1_ASM2200v1
100/100
100/100 Oceanicaulis.alexandrii.DSM.11625 GCA_000420265.1_ASM42026v1
Maricaulis.maris GCA_003634045.1_ASM363404v1
100/100
100/100 Hyphomonas.neptunium.ATCC.15444 GCA_000013025.1_ASM1302v1
Hirschia.baltica.ATCC.49814 GCA_000023785.1_ASM2378v1
100/100
Parvularcula.bermudensis.HTCC2503 GCA_000152825.2_ASM15282v2
100/100 Sagittula.stellata.E.37 GCA_000169415.1_ASM16941v1
Ruegeria.pomeroyi.DSS.3 GCA_000011965.2_ASM1196v2
100/100
100/100
Octadecabacter.arcticus.238 GCA_000155735.2_ASM15573v2
22.3/72
100/100
100/100 Rubellimicrobium.rubrum GCA_006152115.1_ASM615211v1
Ketogulonicigenium.vulgare GCA_001399515.1_ASM139951v1
100/100
Jannaschia.rubra GCA_900113265.1.IMG.taxon.2622736430.annotated.assembly
100/100
100/100 Rhodobacter.sphaeroides.ATCC.17029 GCA_000015985.1_ASM1598v1
Paracoccus.denitrificans GCA_004063735.1_ASM406373v1
Neomegalonema.perideroedes.DSM.15528 GCA_000374145.1_ASM37414v1
48/92 Chelativorans.oligotrophicus GCA_011317505.1_ASM1131750v1
57.9/90
Ahrensia.kielensis.DSM.5890 GCA_000374465.1_ASM37446v1
Rhizobium.leguminosarum.bv.trifolii GCA_011604525.1_ASM1160452v1
100/100
Bartonella.quintana GCA_009936175.1_ASM993617v1
78.6/94
Pelagibacterium.halotolerans GCA_013004005.1_ASM1300400v1
100/100
100/100 Roseibium.hamelinense GCA_007830545.1_ASM783054v1
Polymorphum.gilvum.SL003B.26A1 GCA_000192745.1_ASM19274v1
100/100
Pseudovibrio.sp.FO.BEG1 GCA_000236645.1_ASM23664v1
100/100
100/100 Methylocystis.bryophila GCA_002117405.1_ASM211740v1
Methylocella.silvestris.BL2 GCA_000021745.1_ASM2174v1
100/100
89.3/96
Methylorubrum.extorquens.PA1 GCA_000018845.1_ASM1884v1
100/100
100/100 Xanthobacter.autotrophicus.Py2 GCA_000017645.1_ASM1764v1
Rhodopseudomonas.palustris GCA_013415845.1_ASM1341584v1
100/100
100/100 Rhodomicrobium.vannielii.ATCC.17100 GCA_000166055.1_ASM16605v1
Hyphomicrobium.denitrificans.ATCC.51888 GCA_000143145.1_ASM14314v1
100/100
Thermopetrobacter.sp.TC1 GCA_000746275.1_ASM74627v1
100/100 alpha.proteobacterium.IMCC14465 GCA_000293845.2_ASM29384v2
100/100
Candidatus.Micropelagos.thuwalensis GCA_000469155.1.RS24
Parvibaculum.lavamentivorans.DS.1 GCA_000017565.1_ASM1756v1
100/100
99.8/100
100/100 alpha.proteobacterium.HIMB5 GCA_000299095.1_ASM29909v1
Candidatus.Pelagibacter.ubique.HTCC1062 GCA_000012345.1_ASM1234v1
100/100
Candidatus.Pelagibacter.ubique.HIMB058 GCA_000419545.1_ASM41954v1
100/100
alpha.proteobacterium.HIMB114 GCA_000163555.2
100/100
100/100 Sphingopyxis.macrogoltabida GCA_001314325.1_ASM131432v1
100/100
Citromicrobium.sp.JL477 GCA_001304795.1_ASM130479v1
100/100
Sphingobium.indicum.B90A GCA_000264945.2_ASM26494v2
100/100
100/100 Zymomonas.mobilis.subsp.mobilis.ATCC.31821 GCA_000007105.1_ASM710v1
Sphingomonas.wittichii.RW1 GCA_000016765.1_ASM1676v1
100/100
alpha.proteobacterium.AAP81b GCA_001295935.1_ASM129593v1
Kordiimonas.gwangyangensis.DSM.19435 GCA_000375545.1_ASM37554v1
Sneathiella.glossodoripedis.JCM.23214 GCA_000616095.1_ASM61609v1
Gluconobacter.oxydans.DSM.3504 GCA_000583855.1_ASM58385v1
96.7/100
Acetobacter.aceti.NBRC.14818 GCA_000193495.2_ASM19349v2
100/100
Gluconacetobacter.diazotrophicus.PA1.5 GCA_000067045.1_ASM6704v1
100/100
Commensalibacter.intestini.A911 GCA_000231445.2_ASM23144v1
74.9/91
86.1/94 Granulibacter.bethesdensis.CGDNIH4 GCA_000576145.2_ASM57614v2
Acidisphaera.rubrifaciens.HS.AP3 GCA_000964365.1_ASM96436v1
100/100
Acidiphilium.multivorum.AIU301 GCA_000202835.1_ASM20283v1
100/100 Rubritepida.flocculans.DSM.14296 GCA_000425365.1_ASM42536v1
100/100
Belnapia.moabensis.DSM.16746 GCA_000745835.1_ASM74583v1
100/100
Roseomonas.gilardii GCA_001941945.1_ASM194194v1
Elioraea.tepidiphila.DSM.17972 GCA_000378465.1_ASM37846v1
100/100
100/100 Rhodospirillum.rubrum.F11 GCA_000225955.1_ASM22595v1
89.4/100
Pararhodospirillum.photometricum.DSM.122 GCA_000284415.2_ASM28441v2
100/100
Roseospirillum.parvum GCA_900100455.1
100/100
Caenispirillum.salinarum.AK4 GCA_000315795.1
100/100
Magnetospirillum.magneticum.AMB.1 GCA_000009985.1_ASM998v1
100/100 Thalassospira.profundimaris.WP0211 GCA_000300275.1_ASM30027v1
Terasakiella.pusilla.DSM.6293 GCA_000688235.1_ASM68823v1
99.6/76
100/100 alpha.proteobacterium.BAL199 GCA_000171835.1_ASM17183v1
100/100
Thalassobaculum.salexigens.DSM.19539 GCA_000423805.1_ASM42380v1
Candidatus.Puniceispirillum.marinum.IMCC1322 GCA_000024465.1_ASM2446v1
100/100
94.4/79
Oceanibaculum.indicum GCA_003633955.1_ASM363395v1
99.9/100
100/100 Rhodovibrio.salinarum.DSM.9154 GCA_000515255.1_ASM51525v1
Kiloniella.laminariae.DSM.19542 GCA_000374005.1_ASM37400v1
100/100
94.9/73
100/100 Rhodospirillum.centenum GCA_014197805.1_ASM1419780v1
Inquilinus.limosus.DSM.16000 GCA_000423185.1_ASM42318v1
100/100
Micavibrio.aeruginosavorus.ARL.13 GCA_000226315.1_ASM22631v1
100/100 Candidatus.Paracaedibacter.acanthamoebae GCA_000742835.1_ASM74283v1
Candidatus.Odyssella.thessalonicensis.L13 GCA_000190415.2_ASM19041v1
100/100
27.9/79
Candidatus.Finniella.inopinata GCA_004210305.1_ASM421030v1
100/100 Holospora.undulata.HU1 GCA_000388175.3_ASM38817v3
Candidatus.Hepatobacter.penaei GCA_000742475.1_ASM74247v1
100/100
100/100 Candidatus.Caedibacter.acanthamoebae GCA_000743035.1_ASM74303v1
Caedimonas.varicaedens GCA_001192655.1_ASM119265v1
100/100
100/80
Candidatus.Nucleicultrix.amoebiphila.FS5 GCA_002117145.1_ASM211714v1
99.4/99
100/100 GCA_002938115.1_ASM293811v1*
100/100
GCA_002937585.1_ASM293758v1*
100/100
GCA_002728255.1_ASM272825v1*
100/100
GCA_002501105.1_ASM250110v1*
GCA_002721365.1_ASM272136v1*
100/100
100/100 GCA_002937555.1_ASM293755v1*
100/100
GCA_002717225.1_ASM271722v1*
100/100
100/100 GCA_002728135.1_ASM272813v1*
GCA_002296185.1_ASM229618v1*
100/100 Tistrella.mobilis.KA081020.065 GCA_000264455.2_ASM26445v2
Geminicoccus.roseus.DSM.18922 GCA_000427665.1_ASM42766v1
100/100
99.8/100 Candidatus.Neoehrlichia.lotoris.str.RAC413 GCA_000964795.1_ASM96479v1
100/100
Anaplasma.phagocytophilum.str.HZ GCA_000013125.1_ASM1312v1
100/100
43.8/64
Ehrlichia.ruminantium.str.Welgevonden GCA_000026005.1_ASM2600v1
Wolbachia.endosymbiont.of.Drosophila.melanogaster GCA_000008025.1_ASM802v1
Candidatus.Deianiraea.vastatrix GCA_007993655.1_ASM799365v1
99.6/67
100/100 Rickettsiales.endosymbiont.of.Trichoplax.sp.H2 GCA_009690945.1
100/100
Candidatus.Midichloria.mitochondrii.IricVA GCA_000219355.1_ASM21935v1
100/100 Rickettsiales.endosymbiont.of.Peranema.trichophorum GCA_004210275.1_ASM421027v1
100/100
Candidatus.Jidaibacter.acanthamoeba GCA_000815465.1.UWC36
100/100 Rickettsia.bellii.RML369.C GCA_000012385.1_ASM1238v1
92.3/98
Candidatus.Megaira.polyxenophila
100/100
Rickettsiales.bacterium.Ac37b GCA_000746585.2_ASM74658v2
Rickettsiales.endosymbiont.of.Stachyamoeba.lipophora GCA_003932735.1_ASM393273v1
100/100 Magnetofaba.australis.IT.1 GCA_002109495.1_ASM210949v1
Magnetococcus.marinus.MC.1 GCA_000014865.1_ASM1486v1
71.7/95 Nitrosospira.briensis.C.128 GCA_000619905.2_ASM61990v2
100/100
Methylotenera.versatilis.301 GCA_000093025.1_ASM9302v1
100/100 Ralstonia.pickettii GCA_902374465.1.MGYG.HGUT.01384
Burkholderia.cepacia GCA_009586235.1_ASM958623v1
100/100
100/100 Dasania.marina.DSM.21967 GCA_000373485.1_ASM37348v1
Congregibacter.litoralis.KT71 GCA_000153125.2_ASM15312v2
100/100
100/100 Enterobacter.cloacae GCA_000770155.1_ASM77015v1
Alteromonas.macleodii.ATCC.27126 GCA_000172635.2_ASM17263v2
0.43839

Group F

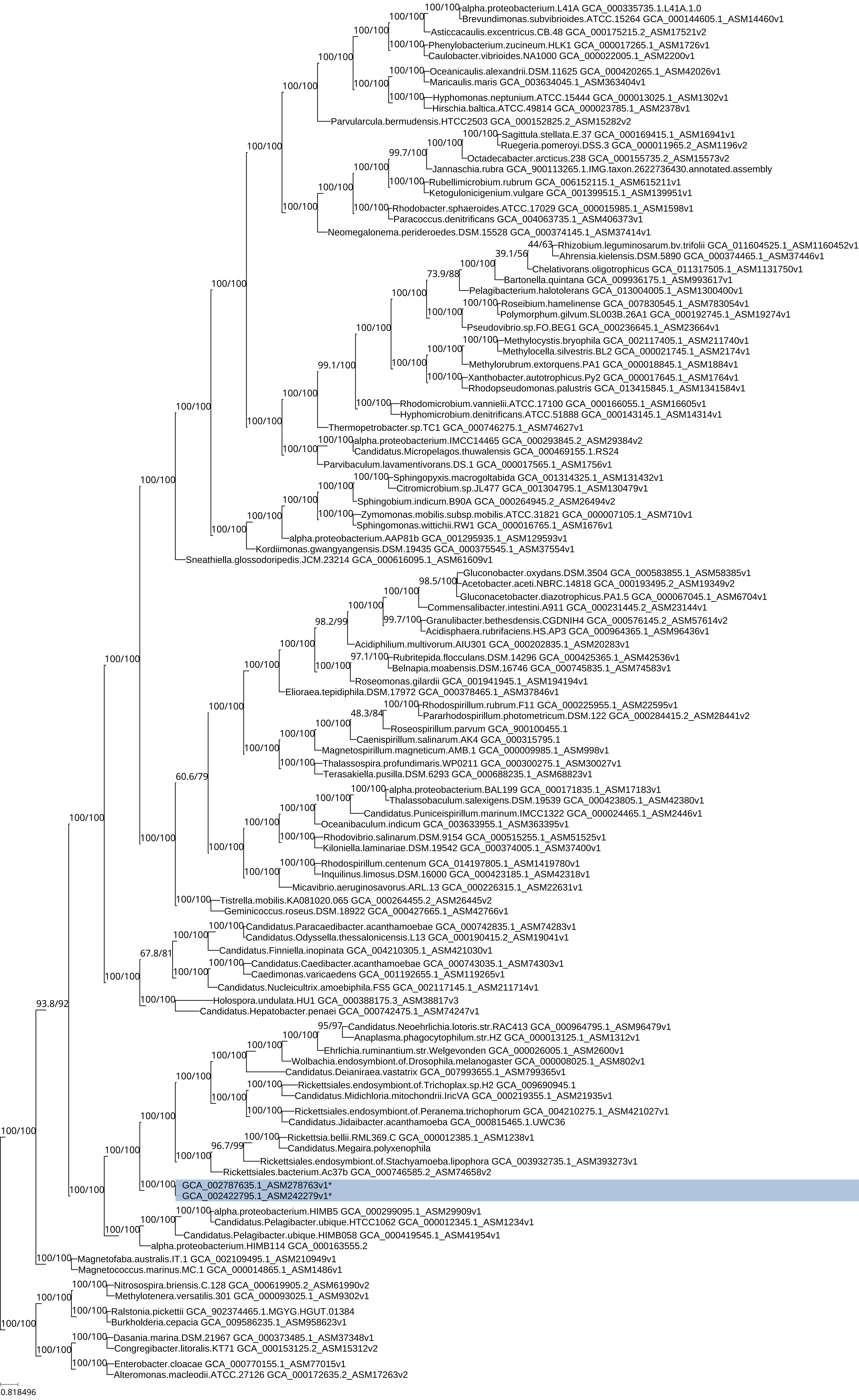
alpha.proteobacterium.L41A GCA_000335735.1.L41A.1.0
Brevundimonas.subvibrioides.ATCC.15264 GCA_000144605.1_ASM14460v1
Asticcacaulis.excentricus.CB.48 GCA_000175215.2_ASM17521v2
Phenylobacterium.zucineum.HLK1 GCA_000017265.1_ASM1726v1
Caulobacter.vibrioides.NA1000 GCA_000022005.1_ASM2200v1
Oceanicaulis.alexandrii.DSM.11625 GCA_000420265.1_ASM42026v1
Maricaulis.maris GCA_003634045.1_ASM363404v1
Hyphomonas.neptunium.ATCC.15444 GCA_000013025.1_ASM1302v1
Hirschia.baltica.ATCC.49814 GCA_000023785.1_ASM2378v1
Parvularcula.bermudensis.HTCC2503 GCA_000152825.2_ASM15282v2
Sagittula.stellata.E.37 GCA_000169415.1_ASM16941v1
Ruegeria.pomeroyi.DSS.3 GCA_000011965.2_ASM1196v2
Octadecabacter.arcticus.238 GCA_000155735.2_ASM15573v2
Jannaschia.rubra GCA_900113265.1.IMG.taxon.2622736430.annotated.assembly
Rubellimicrobium.rubrum GCA_006152115.1_ASM615211v1
Ketogulonicigenium.vulgare GCA_001399515.1_ASM139951v1
Rhodobacter.sphaeroides.ATCC.17029 GCA_000015985.1_ASM1598v1
Paracoccus.denitrificans GCA_004063735.1_ASM406373v1
Neomegalonema.perideroedes.DSM.15528 GCA_000374145.1_ASM37414v1
Rhizobium.leguminosarum.bv.trifolii GCA_011604525.1_ASM1160452v1
Ahrensia.kielensis.DSM.5890 GCA_000374465.1_ASM37446v1
Chelativorans.oligotrophicus GCA_011317505.1_ASM1131750v1
Bartonella.quintana GCA_009936175.1_ASM993617v1
Pelagibacterium.halotolerans GCA_013004005.1_ASM1300400v1
Roseibium.hamelinense GCA_007830545.1_ASM783054v1
Polymorphum.gilvum.SL003B.26A1 GCA_000192745.1_ASM19274v1
Pseudovibrio.sp.FO.BEG1 GCA_000236645.1_ASM23664v1
Methylocystis.bryophila GCA_002117405.1_ASM211740v1
Methylocella.silvestris.BL2 GCA_000021745.1_ASM2174v1
Methylorubrum.extorquens.PA1 GCA_000018845.1_ASM1884v1
Xanthobacter.autotrophicus.Py2 GCA_000017645.1_ASM1764v1
Rhodopseudomonas.palustris GCA_013415845.1_ASM1341584v1
Rhodomicrobium.vannielii.ATCC.17100 GCA_000166055.1_ASM16605v1
Hyphomicrobium.denitrificans.ATCC.51888 GCA_000143145.1_ASM14314v1
Thermopetrobacter.sp.TC1 GCA_000746275.1_ASM74627v1
alpha.proteobacterium.IMCC14465 GCA_000293845.2_ASM29384v2
Candidatus.Micropelagos.thuwalensis GCA_000469155.1.RS24
Parvibaculum.lavamentivorans.DS.1 GCA_000017565.1_ASM1756v1
Sphingopyxis.macrogoltabida GCA_001314325.1_ASM131432v1
Citromicrobium.sp.JL477 GCA_001304795.1_ASM130479v1
Sphingobium.indicum.B90A GCA_000264945.2_ASM26494v2
Zymomonas.mobilis.subsp.mobilis.ATCC.31821 GCA_000007105.1_ASM710v1
Sphingomonas.wittichii.RW1 GCA_000016765.1_ASM1676v1
alpha.proteobacterium.AAP81b GCA_001295935.1_ASM129593v1
Kordiimonas.gwangyangensis.DSM.19435 GCA_000375545.1_ASM37554v1
Sneathiella.glossodoripedis.JCM.23214 GCA_000616095.1_ASM61609v1
Gluconobacter.oxydans.DSM.3504 GCA_000583855.1_ASM58385v1
Acetobacter.aceti.NBRC.14818 GCA_000193495.2_ASM19349v2
Gluconacetobacter.diazotrophicus.PA1.5 GCA_000067045.1_ASM6704v1
Commensalibacter.intestini.A911 GCA_000231445.2_ASM23144v1
Granulibacter.bethesdensis.CGDNIH4 GCA_000576145.2_ASM57614v2
Acidisphaera.rubrifaciens.HS.AP3 GCA_000964365.1_ASM96436v1
Acidiphilium.multivorum.AIU301 GCA_000202835.1_ASM20283v1
Rubritepida.flocculans.DSM.14296 GCA_000425365.1_ASM42536v1
Belnapia.moabensis.DSM.16746 GCA_000745835.1_ASM74583v1
Roseomonas.gilardii GCA_001941945.1_ASM194194v1
Elioraea.tepidiphila.DSM.17972 GCA_000378465.1_ASM37846v1
Rhodospirillum.rubrum.F11 GCA_000225955.1_ASM22595v1
Pararhodospirillum.photometricum.DSM.122 GCA_000284415.2_ASM28441v2
Roseospirillum.parvum GCA_900100455.1
Caenispirillum.salinarum.AK4 GCA_000315795.1
Magnetospirillum.magneticum.AMB.1 GCA_000009985.1_ASM998v1
Thalassospira.profundimaris.WP0211 GCA_000300275.1_ASM30027v1
Terasakiella.pusilla.DSM.6293 GCA_000688235.1_ASM68823v1
alpha.proteobacterium.BAL199 GCA_000171835.1_ASM17183v1
Thalassobaculum.salexigens.DSM.19539 GCA_000423805.1_ASM42380v1
Candidatus.Puniceispirillum.marinum.IMCC1322 GCA_000024465.1_ASM2446v1
Oceanibaculum.indicum GCA_003633955.1_ASM363395v1
Rhodovibrio.salinarum.DSM.9154 GCA_000515255.1_ASM51525v1
Kiloniella.laminariae.DSM.19542 GCA_000374005.1_ASM37400v1
Rhodospirillum.centenum GCA_014197805.1_ASM1419780v1
Inquilinus.limosus.DSM.16000 GCA_000423185.1_ASM42318v1
Micavibrio.aeruginosavorus.ARL.13 GCA_000226315.1_ASM22631v1
Tistrella.mobilis.KA081020.065 GCA_000264455.2_ASM26445v2
Geminicoccus.roseus.DSM.18922 GCA_000427665.1_ASM42766v1
Candidatus.Paracaedibacter.acanthamoebae GCA_000742835.1_ASM74283v1
Candidatus.Odyssella.thessalonicensis.L13 GCA_000190415.2_ASM19041v1
Candidatus.Finniella.inopinata GCA_004210305.1_ASM421030v1
Candidatus.Caedibacter.acanthamoebae GCA_000743035.1_ASM74303v1
Caedimonas.varicaedens GCA_001192655.1_ASM119265v1
Candidatus.Nucleicultrix.amoebiphila.FS5 GCA_002117145.1_ASM211714v1
Holospora.undulata.HU1 GCA_000388175.3_ASM38817v3
Candidatus.Hepatobacter.penaei GCA_000742475.1_ASM74247v1
Candidatus.Neoehrlichia.lotoris.str.RAC413 GCA_000964795.1_ASM96479v1
Anaplasma.phagocytophilum.str.HZ GCA_000013125.1_ASM1312v1
Ehrlichia.ruminantium.str.Welgevonden GCA_000026005.1_ASM2600v1
Wolbachia.endosymbiont.of.Drosophila.melanogaster GCA_000008025.1_ASM802v1
Candidatus.Deianiraea.vastatrix GCA_007993655.1_ASM799365v1
Rickettsiales.endosymbiont.of.Trichoplax.sp.H2 GCA_009690945.1
Candidatus.Midichloria.mitochondrii.IricVA GCA_000219355.1_ASM21935v1
Rickettsiales.endosymbiont.of.Peranema.trichophorum GCA_004210275.1_ASM421027v1
Candidatus.Jidaibacter.acanthamoeba GCA_000815465.1.UWC36
Rickettsia.bellii.RML369.C GCA_000012385.1_ASM1238v1
Candidatus.Megaira.polyxenophila
Rickettsiales.endosymbiont.of.Stachyamoeba.lipophora GCA_003932735.1_ASM393273v1
Rickettsiales.bacterium.Ac37b GCA_000746585.2_ASM74658v2
GCA_002787635.1_ASM278763v1*
GCA_002422795.1_ASM242279v1*
alpha.proteobacterium.HIMB5 GCA_000299095.1_ASM29909v1
Candidatus.Pelagibacter.ubique.HTCC1062 GCA_000012345.1_ASM1234v1
Candidatus.Pelagibacter.ubique.HIMB058 GCA_000419545.1_ASM41954v1
alpha.proteobacterium.HIMB114 GCA_000163555.2
Magnetofaba.australis.IT.1 GCA_002109495.1_ASM210949v1
Magnetococcus.marinus.MC.1 GCA_000014865.1_ASM1486v1
Nitrosospira.briensis.C.128 GCA_000619905.2_ASM61990v2
Methylotenera.versatilis.301 GCA_000093025.1_ASM9302v1
Ralstonia.pickettii GCA_902374465.1.MGYG.HGUT.01384
Burkholderia.cepacia GCA_009586235.1_ASM958623v1
Dasania.marina.DSM.21967 GCA_000373485.1_ASM37348v1
Congregibacter.litoralis.KT71 GCA_000153125.2_ASM15312v2
Enterobacter.cloacae GCA_000770155.1_ASM77015v1
Alteromonas.macleodii.ATCC.27126 GCA_000172635.2_ASM17263v2
0.818496

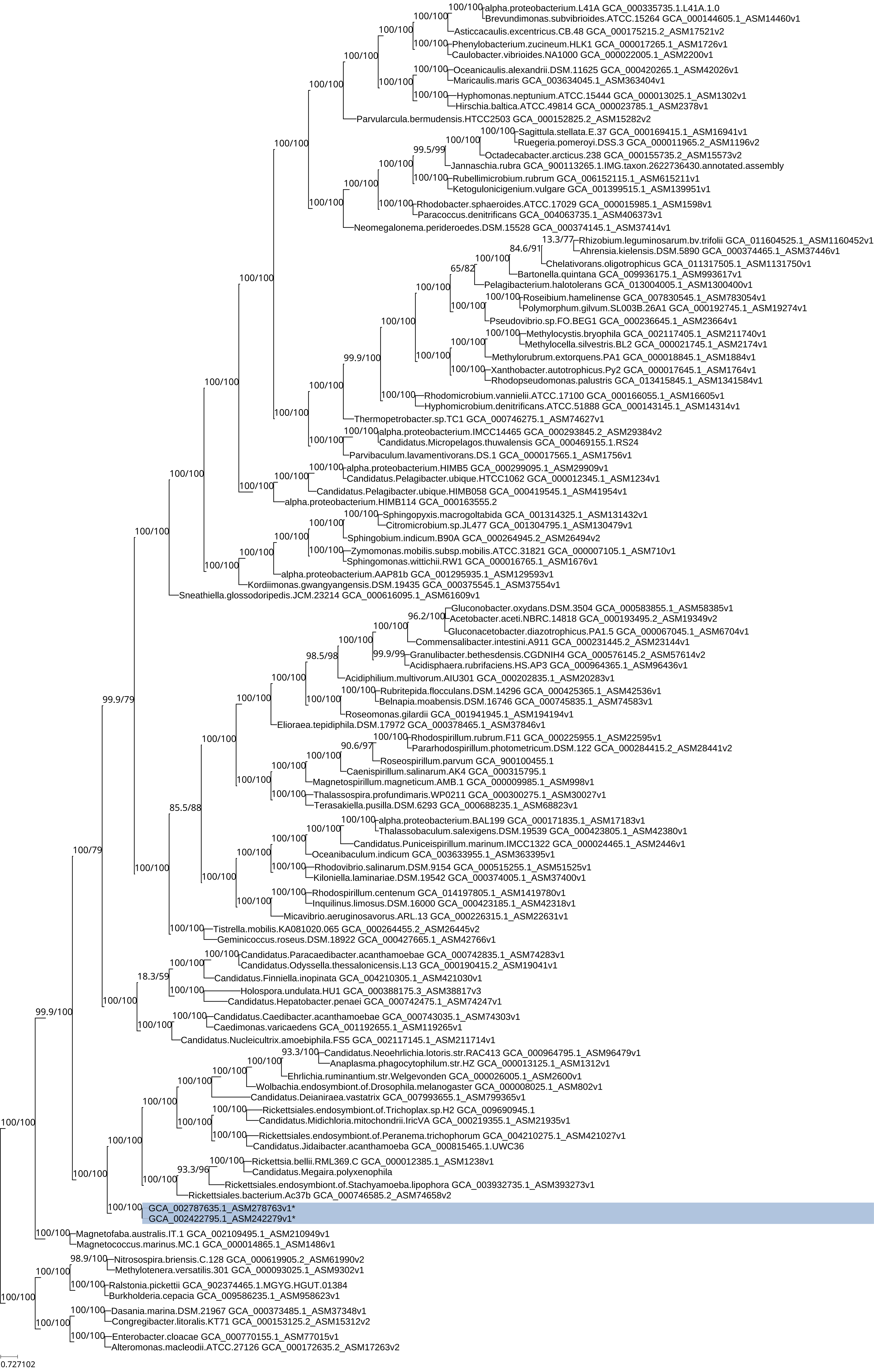
alpha.proteobacterium.L41A GCA_000335735.1.L41A.1.0
Brevundimonas.subvibrioides.ATCC.15264 GCA_000144605.1_ASM14460v1
Asticcacaulis.excentricus.CB.48 GCA_000175215.2_ASM17521v2
Phenylobacterium.zucineum.HLK1 GCA_000017265.1_ASM1726v1
Caulobacter.vibrioides.NA1000 GCA_000022005.1_ASM2200v1
Oceanicaulis.alexandrii.DSM.11625 GCA_000420265.1_ASM42026v1
Maricaulis.maris GCA_003634045.1_ASM363404v1
Hyphomonas.neptunium.ATCC.15444 GCA_000013025.1_ASM1302v1
Hirschia.baltica.ATCC.49814 GCA_000023785.1_ASM2378v1
Parvularcula.bermudensis.HTCC2503 GCA_000152825.2_ASM15282v2
Sagittula.stellata.E.37 GCA_000169415.1_ASM16941v1
Ruegeria.pomeroyi.DSS.3 GCA_000011965.2_ASM1196v2
Octadecabacter.arcticus.238 GCA_000155735.2_ASM15573v2
Jannaschia.rubra GCA_900113265.1.IMG.taxon.2622736430.annotated.assembly
Rubellimicrobium.rubrum GCA_006152115.1_ASM615211v1
Ketogulonicigenium.vulgare GCA_001399515.1_ASM139951v1
Rhodobacter.sphaeroides.ATCC.17029 GCA_000015985.1_ASM1598v1
Paracoccus.denitrificans GCA_004063735.1_ASM406373v1
Neomegalonema.perideroedes.DSM.15528 GCA_000374145.1_ASM37414v1
Rhizobium.leguminosarum.bv.trifolii GCA_011604525.1_ASM1160452v1
Ahrensia.kielensis.DSM.5890 GCA_000374465.1_ASM37446v1
Chelativorans.oligotrophicus GCA_011317505.1_ASM1131750v1
Bartonella.quintana GCA_009936175.1_ASM993617v1
Pelagibacterium.halotolerans GCA_013004005.1_ASM1300400v1
Roseibium.hamelinense GCA_007830545.1_ASM783054v1
Polymorphum.gilvum.SL003B.26A1 GCA_000192745.1_ASM19274v1
Pseudovibrio.sp.FO.BEG1 GCA_000236645.1_ASM23664v1
Methylocystis.bryophila GCA_002117405.1_ASM211740v1
Methylocella.silvestris.BL2 GCA_000021745.1_ASM2174v1
Methylorubrum.extorquens.PA1 GCA_000018845.1_ASM1884v1
Xanthobacter.autotrophicus.Py2 GCA_000017645.1_ASM1764v1
Rhodopseudomonas.palustris GCA_013415845.1_ASM1341584v1
Rhodomicrobium.vannielii.ATCC.17100 GCA_000166055.1_ASM16605v1
Hyphomicrobium.denitrificans.ATCC.51888 GCA_000143145.1_ASM14314v1
Thermopetrobacter.sp.TC1 GCA_000746275.1_ASM74627v1
alpha.proteobacterium.IMCC14465 GCA_000293845.2_ASM29384v2
Candidatus.Micropelagos.thuwalensis GCA_000469155.1.RS24
Parvibaculum.lavamentivorans.DS.1 GCA_000017565.1_ASM1756v1
alpha.proteobacterium.HIMB5 GCA_000299095.1_ASM29909v1
Candidatus.Pelagibacter.ubique.HTCC1062 GCA_000012345.1_ASM1234v1
Candidatus.Pelagibacter.ubique.HIMB058 GCA_000419545.1_ASM41954v1
alpha.proteobacterium.HIMB114 GCA_000163555.2
Sphingopyxis.macrogoltabida GCA_001314325.1_ASM131432v1
Citromicrobium.sp.JL477 GCA_001304795.1_ASM130479v1
Sphingobium.indicum.B90A GCA_000264945.2_ASM26494v2
Zymomonas.mobilis.subsp.mobilis.ATCC.31821 GCA_000007105.1_ASM710v1
Sphingomonas.wittichii.RW1 GCA_000016765.1_ASM1676v1
alpha.proteobacterium.AAP81b GCA_001295935.1_ASM129593v1
Kordiimonas.gwangyangensis.DSM.19435 GCA_000375545.1_ASM37554v1
Sneathiella.glossodoripedis.JCM.23214 GCA_000616095.1_ASM61609v1
Gluconobacter.oxydans.DSM.3504 GCA_000583855.1_ASM58385v1
Acetobacter.aceti.NBRC.14818 GCA_000193495.2_ASM19349v2
Gluconacetobacter.diazotrophicus.PA1.5 GCA_000067045.1_ASM6704v1
Commensalibacter.intestini.A911 GCA_000231445.2_ASM23144v1
Granulibacter.bethesdensis.CGDNIH4 GCA_000576145.2_ASM57614v2
Acidisphaera.rubrifaciens.HS.AP3 GCA_000964365.1_ASM96436v1
Acidiphilium.multivorum.AIU301 GCA_000202835.1_ASM20283v1
Rubritepida.flocculans.DSM.14296 GCA_000425365.1_ASM42536v1
Belnapia.moabensis.DSM.16746 GCA_000745835.1_ASM74583v1
Roseomonas.gilardii GCA_001941945.1_ASM194194v1
Elioraea.tepidiphila.DSM.17972 GCA_000378465.1_ASM37846v1
Rhodospirillum.rubrum.F11 GCA_000225955.1_ASM22595v1
Pararhodospirillum.photometricum.DSM.122 GCA_000284415.2_ASM28441v2
Roseospirillum.parvum GCA_900100455.1
Caenispirillum.salinarum.AK4 GCA_000315795.1
Magnetospirillum.magneticum.AMB.1 GCA_000009985.1_ASM998v1
Thalassospira.profundimaris.WP0211 GCA_000300275.1_ASM30027v1
Terasakiella.pusilla.DSM.6293 GCA_000688235.1_ASM68823v1
alpha.proteobacterium.BAL199 GCA_000171835.1_ASM17183v1
Thalassobaculum.salexigens.DSM.19539 GCA_000423805.1_ASM42380v1
Candidatus.Puniceispirillum.marinum.IMCC1322 GCA_000024465.1_ASM2446v1
Oceanibaculum.indicum GCA_003633955.1_ASM363395v1
Rhodovibrio.salinarum.DSM.9154 GCA_000515255.1_ASM51525v1
Kiloniella.laminariae.DSM.19542 GCA_000374005.1_ASM37400v1
Rhodospirillum.centenum GCA_014197805.1_ASM1419780v1
Inquilinus.limosus.DSM.16000 GCA_000423185.1_ASM42318v1
Micavibrio.aeruginosavorus.ARL.13 GCA_000226315.1_ASM22631v1
Tistrella.mobilis.KA081020.065 GCA_000264455.2_ASM26445v2
Geminicoccus.roseus.DSM.18922 GCA_000427665.1_ASM42766v1
Candidatus.Paracaedibacter.acanthamoebae GCA_000742835.1_ASM74283v1
Candidatus.Odyssella.thessalonicensis.L13 GCA_000190415.2_ASM19041v1
Candidatus.Finniella.inopinata GCA_004210305.1_ASM421030v1
Holospora.undulata.HU1 GCA_000388175.3_ASM38817v3
Candidatus.Hepatobacter.penaei GCA_000742475.1_ASM74247v1
Candidatus.Caedibacter.acanthamoebae GCA_000743035.1_ASM74303v1
Caedimonas.varicaedens GCA_001192655.1_ASM119265v1
Candidatus.Nucleicultrix.amoebiphila.FS5 GCA_002117145.1_ASM211714v1
Candidatus.Neoehrlichia.lotoris.str.RAC413 GCA_000964795.1_ASM96479v1
Anaplasma.phagocytophilum.str.HZ GCA_000013125.1_ASM1312v1
Ehrlichia.ruminantium.str.Welgevonden GCA_000026005.1_ASM2600v1
Wolbachia.endosymbiont.of.Drosophila.melanogaster GCA_000008025.1_ASM802v1
Candidatus.Deianiraea.vastatrix GCA_007993655.1_ASM799365v1
Rickettsiales.endosymbiont.of.Trichoplax.sp.H2 GCA_009690945.1
Candidatus.Midichloria.mitochondrii.IricVA GCA_000219355.1_ASM21935v1
Rickettsiales.endosymbiont.of.Peranema.trichophorum GCA_004210275.1_ASM421027v1
Candidatus.Jidaibacter.acanthamoeba GCA_000815465.1.UWC36
Rickettsia.bellii.RML369.C GCA_000012385.1_ASM1238v1
Candidatus.Megaira.polyxenophila
Rickettsiales.endosymbiont.of.Stachyamoeba.lipophora GCA_003932735.1_ASM393273v1
Rickettsiales.bacterium.Ac37b GCA_000746585.2_ASM74658v2
GCA_002787635.1_ASM278763v1*
GCA_002422795.1_ASM242279v1*
Magnetofaba.australis.IT.1 GCA_002109495.1_ASM210949v1
Magnetococcus.marinus.MC.1 GCA_000014865.1_ASM1486v1
Nitrosospira.briensis.C.128 GCA_000619905.2_ASM61990v2
Methylotenera.versatilis.301 GCA_000093025.1_ASM9302v1
Ralstonia.pickettii GCA_902374465.1.MGYG.HGUT.01384
Burkholderia.cepacia GCA_009586235.1_ASM958623v1
Dasania.marina.DSM.21967 GCA_000373485.1_ASM37348v1
Congregibacter.litoralis.KT71 GCA_000153125.2_ASM15312v2
Enterobacter.cloacae GCA_000770155.1_ASM77015v1
Alteromonas.macleodii.ATCC.27126 GCA_000172635.2_ASM17263v2
0.727102

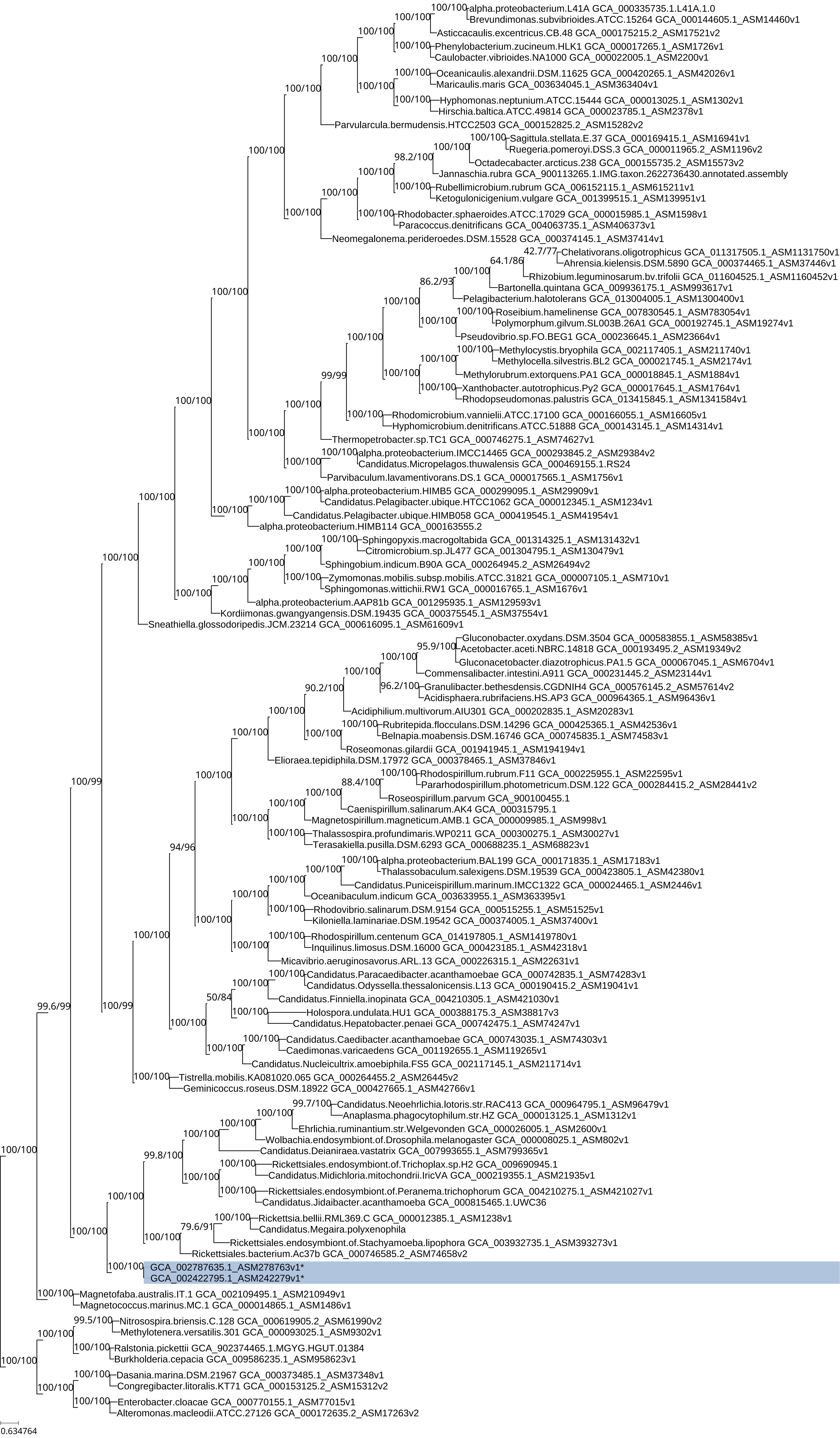
alpha.proteobacterium.L41A GCA_000335735.1.L41A.1.0
Brevundimonas.subvibrioides.ATCC.15264 GCA_000144605.1_ASM14460v1
Asticcacaulis.excentricus.CB.48 GCA_000175215.2_ASM17521v2
Phenylobacterium.zucineum.HLK1 GCA_000017265.1_ASM1726v1
Caulobacter.vibrioides.NA1000 GCA_000022005.1_ASM2200v1
Oceanicaulis.alexandrii.DSM.11625 GCA_000420265.1_ASM42026v1
Maricaulis.maris GCA_003634045.1_ASM363404v1
Hyphomonas.neptunium.ATCC.15444 GCA_000013025.1_ASM1302v1
Hirschia.baltica.ATCC.49814 GCA_000023785.1_ASM2378v1
Parvularcula.bermudensis.HTCC2503 GCA_000152825.2_ASM15282v2
Sagittula.stellata.E.37 GCA_000169415.1_ASM16941v1
Ruegeria.pomeroyi.DSS.3 GCA_000011965.2_ASM1196v2
Octadecabacter.arcticus.238 GCA_000155735.2_ASM15573v2
Jannaschia.rubra GCA_900113265.1.IMG.taxon.2622736430.annotated.assembly
Rubellimicrobium.rubrum GCA_006152115.1_ASM615211v1
Ketogulonicigenium.vulgare GCA_001399515.1_ASM139951v1
Rhodobacter.sphaeroides.ATCC.17029 GCA_000015985.1_ASM1598v1
Paracoccus.denitrificans GCA_004063735.1_ASM406373v1
Neomegalonema.perideroedes.DSM.15528 GCA_000374145.1_ASM37414v1
Chelativorans.oligotrophicus GCA_011317505.1_ASM1131750v1
Ahrensia.kielensis.DSM.5890 GCA_000374465.1_ASM37446v1
Rhizobium.leguminosarum.bv.trifolii GCA_011604525.1_ASM1160452v1
Bartonella.quintana GCA_009936175.1_ASM993617v1
Pelagibacterium.halotolerans GCA_013004005.1_ASM1300400v1
Roseibium.hamelinense GCA_007830545.1_ASM783054v1
Polymorphum.gilvum.SL003B.26A1 GCA_000192745.1_ASM19274v1
Pseudovibrio.sp.FO.BEG1 GCA_000236645.1_ASM23664v1
Methylocystis.bryophila GCA_002117405.1_ASM211740v1
Methylocella.silvestris.BL2 GCA_000021745.1_ASM2174v1
Methylorubrum.extorquens.PA1 GCA_000018845.1_ASM1884v1
Xanthobacter.autotrophicus.Py2 GCA_000017645.1_ASM1764v1
Rhodopseudomonas.palustris GCA_013415845.1_ASM1341584v1
Rhodomicrobium.vannielii.ATCC.17100 GCA_000166055.1_ASM16605v1
Hyphomicrobium.denitrificans.ATCC.51888 GCA_000143145.1_ASM14314v1
Thermopetrobacter.sp.TC1 GCA_000746275.1_ASM74627v1
alpha.proteobacterium.IMCC14465 GCA_000293845.2_ASM29384v2
Candidatus.Micropelagos.thuwalensis GCA_000469155.1.RS24
Parvibaculum.lavamentivorans.DS.1 GCA_000017565.1_ASM1756v1
alpha.proteobacterium.HIMB5 GCA_000299095.1_ASM29909v1
Candidatus.Pelagibacter.ubique.HTCC1062 GCA_000012345.1_ASM1234v1
Candidatus.Pelagibacter.ubique.HIMB058 GCA_000419545.1_ASM41954v1
alpha.proteobacterium.HIMB114 GCA_000163555.2
Sphingopyxis.macrogoltabida GCA_001314325.1_ASM131432v1
Citromicrobium.sp.JL477 GCA_001304795.1_ASM130479v1
Sphingobium.indicum.B90A GCA_000264945.2_ASM26494v2
Zymomonas.mobilis.subsp.mobilis.ATCC.31821 GCA_000007105.1_ASM710v1
Sphingomonas.wittichii.RW1 GCA_000016765.1_ASM1676v1
alpha.proteobacterium.AAP81b GCA_001295935.1_ASM129593v1
Kordiimonas.gwangyangensis.DSM.19435 GCA_000375545.1_ASM37554v1
Sneathiella.glossodoripedis.JCM.23214 GCA_000616095.1_ASM61609v1
Gluconobacter.oxydans.DSM.3504 GCA_000583855.1_ASM58385v1
Acetobacter.aceti.NBRC.14818 GCA_000193495.2_ASM19349v2
Gluconacetobacter.diazotrophicus.PA1.5 GCA_000067045.1_ASM6704v1
Commensalibacter.intestini.A911 GCA_000231445.2_ASM23144v1
Granulibacter.bethesdensis.CGDNIH4 GCA_000576145.2_ASM57614v2
Acidisphaera.rubrifaciens.HS.AP3 GCA_000964365.1_ASM96436v1
Acidiphilium.multivorum.AIU301 GCA_000202835.1_ASM20283v1
Rubritepida.flocculans.DSM.14296 GCA_000425365.1_ASM42536v1
Belnapia.moabensis.DSM.16746 GCA_000745835.1_ASM74583v1
Roseomonas.gilardii GCA_001941945.1_ASM194194v1
Elioraea.tepidiphila.DSM.17972 GCA_000378465.1_ASM37846v1
Rhodospirillum.rubrum.F11 GCA_000225955.1_ASM22595v1
Pararhodospirillum.photometricum.DSM.122 GCA_000284415.2_ASM28441v2
Roseospirillum.parvum GCA_900100455.1
Caenispirillum.salinarum.AK4 GCA_000315795.1
Magnetospirillum.magneticum.AMB.1 GCA_000009985.1_ASM998v1
Thalassospira.profundimaris.WP0211 GCA_000300275.1_ASM30027v1
Terasakiella.pusilla.DSM.6293 GCA_000688235.1_ASM68823v1
alpha.proteobacterium.BAL199 GCA_000171835.1_ASM17183v1
Thalassobaculum.salexigens.DSM.19539 GCA_000423805.1_ASM42380v1
Candidatus.Puniceispirillum.marinum.IMCC1322 GCA_000024465.1_ASM2446v1
Oceanibaculum.indicum GCA_003633955.1_ASM363395v1
Rhodovibrio.salinarum.DSM.9154 GCA_000515255.1_ASM51525v1
Kiloniella.laminariae.DSM.19542 GCA_000374005.1_ASM37400v1
Rhodospirillum.centenum GCA_014197805.1_ASM1419780v1
Inquilinus.limosus.DSM.16000 GCA_000423185.1_ASM42318v1
Micavibrio.aeruginosavorus.ARL.13 GCA_000226315.1_ASM22631v1
Candidatus.Paracaedibacter.acanthamoebae GCA_000742835.1_ASM74283v1
Candidatus.Odyssella.thessalonicensis.L13 GCA_000190415.2_ASM19041v1
Candidatus.Finniella.inopinata GCA_004210305.1_ASM421030v1
Holospora.undulata.HU1 GCA_000388175.3_ASM38817v3
Candidatus.Hepatobacter.penaei GCA_000742475.1_ASM74247v1
Candidatus.Caedibacter.acanthamoebae GCA_000743035.1_ASM74303v1
Caedimonas.varicaedens GCA_001192655.1_ASM119265v1
Candidatus.Nucleicultrix.amoebiphila.FS5 GCA_002117145.1_ASM211714v1
Tistrella.mobilis.KA081020.065 GCA_000264455.2_ASM26445v2
Geminicoccus.roseus.DSM.18922 GCA_000427665.1_ASM42766v1
Candidatus.Neoehrlichia.lotoris.str.RAC413 GCA_000964795.1_ASM96479v1
Anaplasma.phagocytophilum.str.HZ GCA_000013125.1_ASM1312v1
Ehrlichia.ruminantium.str.Welgevonden GCA_000026005.1_ASM2600v1
Wolbachia.endosymbiont.of.Drosophila.melanogaster GCA_000008025.1_ASM802v1
Candidatus.Deianiraea.vastatrix GCA_007993655.1_ASM799365v1
Rickettsiales.endosymbiont.of.Trichoplax.sp.H2 GCA_009690945.1
Candidatus.Midichloria.mitochondrii.IricVA GCA_000219355.1_ASM21935v1
Rickettsiales.endosymbiont.of.Peranema.trichophorum GCA_004210275.1_ASM421027v1
Candidatus.Jidaibacter.acanthamoeba GCA_000815465.1.UWC36
Rickettsia.bellii.RML369.C GCA_000012385.1_ASM1238v1
Candidatus.Megaira.polyxenophila
Rickettsiales.endosymbiont.of.Stachyamoeba.lipophora GCA_003932735.1_ASM393273v1
Rickettsiales.bacterium.Ac37b GCA_000746585.2_ASM74658v2
GCA_002787635.1_ASM278763v1*
GCA_002422795.1_ASM242279v1*
Magnetofaba.australis.IT.1 GCA_002109495.1_ASM210949v1
Magnetococcus.marinus.MC.1 GCA_000014865.1_ASM1486v1
Nitrosospira.briensis.C.128 GCA_000619905.2_ASM61990v2
Methylotenera.versatilis.301 GCA_000093025.1_ASM9302v1
Ralstonia.pickettii GCA_902374465.1.MGYG.HGUT.01384
Burkholderia.cepacia GCA_009586235.1_ASM958623v1
Dasania.marina.DSM.21967 GCA_000373485.1_ASM37348v1
Congregibacter.litoralis.KT71 GCA_000153125.2_ASM15312v2
Enterobacter.cloacae GCA_000770155.1_ASM77015v1
Alteromonas.macleodii.ATCC.27126 GCA_000172635.2_ASM17263v2
0.634764

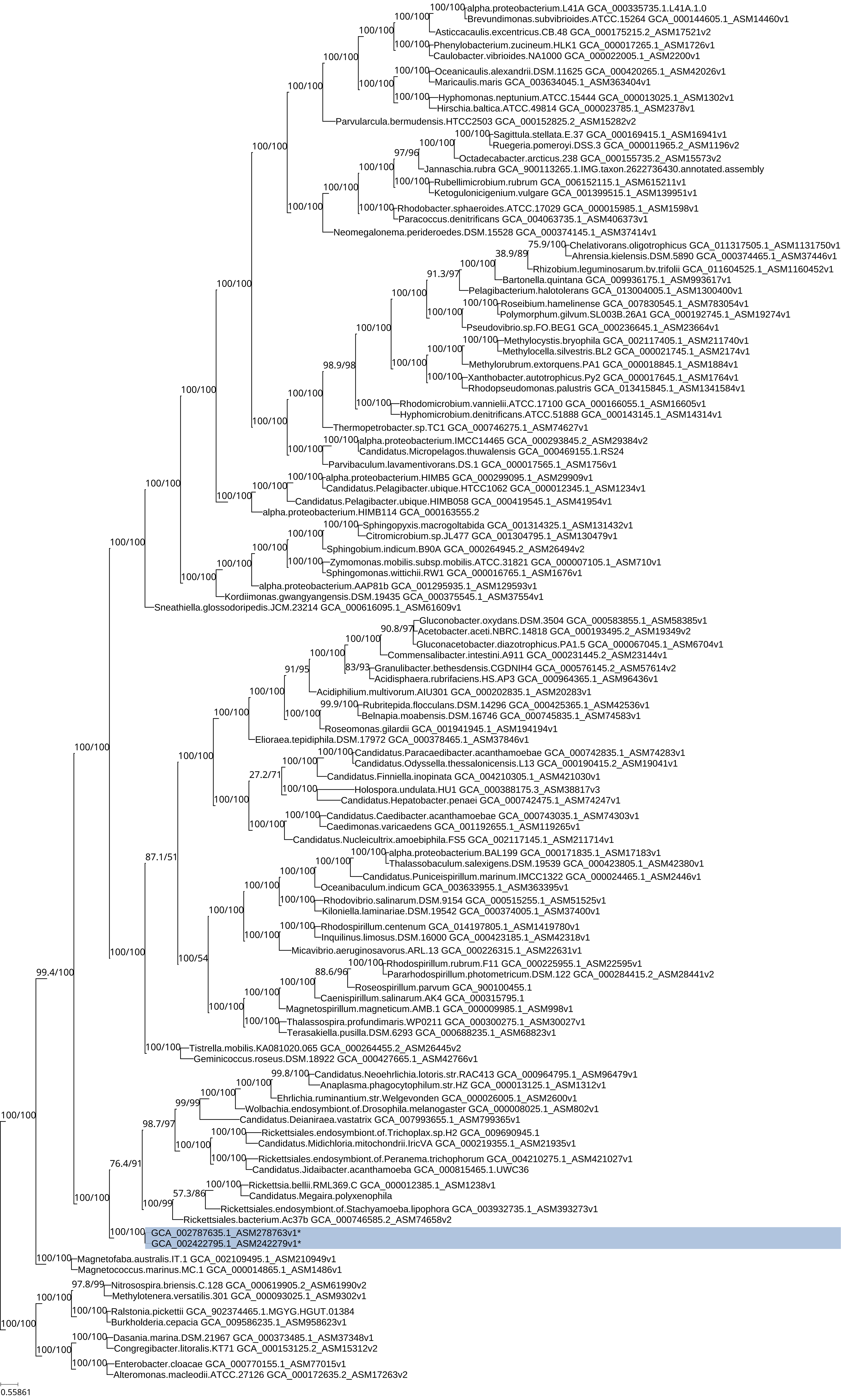

100/100
alpha.proteobacterium.L41A GCA_000335735.1_L41A.1.0
Brevundimonas.subvibrioides.ATCC.15264 GCA_000144605.1_ASM14460v1
100/100
Asticcacaulis.excentricus.CB.48 GCA_000175215.2_ASM17521v2
100/100
100/100
Phenylobacterium.zucineum.HLK1 GCA_000017265.1_ASM1726v1
Caulobacter.vibrioides.NA1000 GCA_000022005.1_ASM2200v1
100/100
100/100
Oceanicaulis.alexandrii.DSM.11625 GCA_000420265.1_ASM42026v1
Maricaulis.maris GCA_003634045.1_ASM363404v1
100/100
100/100
Hyphomonas.neptunium.ATCC.15444 GCA_000013025.1_ASM1302v1
Hirschia.baltica.ATCC.49814 GCA_000023785.1_ASM2378v1
Parvularcula.bermudensis.HTCC2503 GCA_000152825.2_ASM15282v2
100/100
Sagittula.stellata.E.37 GCA_000169415.1_ASM16941v1
100/100
Ruegeria.pomeroyi.DSS.3 GCA_000011965.2_ASM1196v2
100/100
Octadecabacter.arcticus.238 GCA_000155735.2_ASM15573v2
97/96
Jannaschia.rubra GCA_900113265.1.IMG.taxon.2622736430.annotated.assembly
100/100
100/100
Rubellimicrobium.rubrum GCA_006152115.1_ASM615211v1
100/100
Ketogulonicigenium.vulgare GCA_001399515.1_ASM139951v1
100/100
100/100
Rhodobacter.sphaeroides.ATCC.17029 GCA_000015985.1_ASM1598v1
Paracoccus.denitrificans GCA_004063735.1_ASM406373v1
Neomegalonema.perideroedes.DSM.15528 GCA_000374145.1_ASM37414v1
75.9/100
Chelativorans.oligotrophicus GCA_011317505.1_ASM1131750v1
38.9/89
Ahrensia.kielensis.DSM.5890 GCA_000374465.1_ASM37446v1
100/100
Rhizobium.leguminosarum.bv.trifolii GCA_011604525.1_ASM1160452v1
91.3/97
Bartonella.quintana GCA_009936175.1_ASM993617v1
100/100
Pelagibacterium.halotolerans GCA_013004005.1_ASM1300400v1
100/100
100/100
Roseibium.hamelinense GCA_007830545.1_ASM783054v1
100/100
Polymorphum.gilvum.SL003B.26A1 GCA_000192745.1_ASM19274v1
100/100
Pseudovibrio.sp.FO.BEG1 GCA_000236645.1_ASM23664v1
100/100
Methylocystis.bryophila GCA_002117405.1_ASM211740v1
100/100
Methylocella.silvestris.BL2 GCA_000021745.1_ASM2174v1
98.9/98
100/100
Methylorubrum.extorquens.PA1 GCA_000018845.1_ASM1884v1
100/100
Xanthobacter.autotrophicus.Py2 GCA_000017645.1_ASM1764v1
100/100
Rhodopseudomonas.palustris GCA_013415845.1_ASM1341584v1
100/100
100/100
Rhodomicrobium.vannielii.ATCC.17100 GCA_000166055.1_ASM16605v1
Hyphomicrobium.denitrificans.ATCC.51888 GCA_000143145.1_ASM14314v1
100/100
Thermopetrobacter.sp.TC1 GCA_000746275.1_ASM74627v1
100/100
alpha.proteobacterium.IMCC14465 GCA_000293845.2_ASM29384v2
100/100
Candidatus.Micropelagos.thuwalensis GCA_000469155.1.RS24
Parvibaculum.lavamentivorans.DS.1 GCA_000017565.1_ASM1756v1
100/100
alpha.proteobacterium.HIMB5 GCA_000299095.1_ASM29909v1
100/100
100/100
Candidatus.Pelagibacter.ubique.HTCC1062 GCA_000012345.1_ASM1234v1
100/100
Candidatus.Pelagibacter.ubique.HIMB058 GCA_000419545.1_ASM41954v1
alpha.proteobacterium.HIMB114 GCA_000163555.2
100/100
Sphingopyxis.macrogoltabida GCA_001314325.1_ASM131432v1
100/100
100/100
Citromicrobium.sp.JL477 GCA_001304795.1_ASM130479v1
100/100
Sphingobium.indicum.B90A GCA_000264945.2_ASM26494v2
100/100
Zymomonas.mobilis.subsp.mobilis.ATCC.31821 GCA_000007105.1_ASM710v1
100/100
Sphingomonas.wittichii.RW1 GCA_000016765.1_ASM1676v1
100/100
alpha.proteobacterium.AAP81b GCA_001295935.1_ASM129593v1
Kordiimonas.gwangyangensis.DSM.19435 GCA_000375545.1_ASM37554v1
Sneathiella.glossodoripedis.JCM.23214 GCA_000616095.1_ASM61609v1
Gluconobacter.oxydans.DSM.3504 GCA_000583855.1_ASM58385v1
90.8/97
Acetobacter.aceti.NBRC.14818 GCA_000193495.2_ASM19349v2
100/100
Gluconacetobacter.diazotrophicus.PA1.5 GCA_000067045.1_ASM6704v1
100/100
Commensalibacter.intestini.A911 GCA_000231445.2_ASM23144v1
91/95
83/93
Granulibacter.bethesdensis.CGDNIH4 GCA_000576145.2_ASM57614v2
Acidisphaera.rubrifaciens.HS.AP3 GCA_000964365.1_ASM96436v1
100/100
Acidiphilium.multivorum.AIU301 GCA_000202835.1_ASM20283v1
100/100
99.9/100
Rubritepida.flocculans.DSM.14296 GCA_000425365.1_ASM42536v1
100/100
Belnapia.moabensis.DSM.16746 GCA_000745835.1_ASM74583v1
Roseomonas.gilardii GCA_001941945.1_ASM194194v1
Elioraea.tepidiphila.DSM.17972 GCA_000378465.1_ASM37846v1
100/100
100/100
Candidatus.Paracaedibacter.acanthamoebae GCA_000742835.1_ASM74283v1
100/100
Candidatus.Odyssella.thessalonicensis.L13 GCA_000190415.2_ASM19041v1
100/100
27.2/71
Candidatus.Finniella.inopinata GCA_004210305.1_ASM421030v1
100/100
Holospora.undulata.HU1 GCA_000388175.3_ASM38817v3
100/100
Candidatus.Hepatobacter.penaei GCA_000742475.1_ASM74247v1
100/100
Candidatus.Caedibacter.acanthamoebae GCA_000743035.1_ASM74303v1
100/100
Caedimonas.varicaedens GCA_001192655.1_ASM119265v1
Candidatus.Nucleicultrix.amoebiphila.FS5 GCA_002117145.1_ASM211714v1
87.1/51
100/100
alpha.proteobacterium.BAL199 GCA_000171835.1_ASM17183v1
100/100
Thalassobaculum.salexigens.DSM.19539 GCA_000423805.1_ASM42380v1
100/100
Candidatus.Puniceispirillum.marinum.IMCC1322 GCA_000024465.1_ASM2446v1
100/100
Oceanibaculum.indicum GCA_003633955.1_ASM363395v1
100/100
Rhodovibrio.salinarum.DSM.9154 GCA_000515255.1_ASM51525v1
100/100
Kiloniella.laminariae.DSM.19542 GCA_000374005.1_ASM37400v1
100/100
Rhodospirillum.centenum GCA_014197805.1_ASM1419780v1
100/100
Inquilinus.limosus.DSM.16000 GCA_000423185.1_ASM42318v1
100/100
Micavibrio.aeruginosavorus.ARL.13 GCA_000226315.1_ASM22631v1
100/54
100/100
Rhodospirillum.rubrum.F11 GCA_000225955.1_ASM22595v1
99.4/100
88.6/96
Pararhodospirillum.photometricum.DSM.122 GCA_000284415.2_ASM28441v2
100/100
Roseospirillum.parvum GCA_900100455.1
100/100
Caenispirillum.salinarum.AK4 GCA_000315795.1
100/100
Magnetospirillum.magneticum.AMB.1 GCA_000009985.1_ASM998v1
100/100
Thalassospira.profundimaris.WP0211 GCA_000300275.1_ASM30027v1
Terasakiella.pusilla.DSM.6293 GCA_000688235.1_ASM68823v1
100/100
Tistrella.mobilis.KA081020.065 GCA_000264455.2_ASM26445v2
Geminicoccus.roseus.DSM.18922 GCA_000427665.1_ASM42766v1
99.8/100
Candidatus.Neoehrlichia.lotoris.str.RAC413 GCA_000964795.1_ASM96479v1
100/100
Anaplasma.phagocytophilum.str.HZ GCA_000013125.1_ASM1312v1
100/100
Ehrlichia.ruminantium.str.Welgevonden GCA_000026005.1_ASM2600v1
99/99
Wolbachia.endosymbiont.of.Drosophila.melanogaster GCA_000008025.1_ASM802v1
100/100
Candidatus.Deianiraea.vastatrix GCA_007993655.1_ASM799365v1
98.7/97
100/100
Rickettsiales.endosymbiont.of.Trichoplax.sp.H2 GCA_009690945.1
100/100
Candidatus.Midichloria.mitochondrii.IricVA GCA_000219355.1_ASM21935v1
76.4/91
100/100
Rickettsiales.endosymbiont.of.Peranema.trichophorum GCA_004210275.1_ASM421027v1
Candidatus.Jidaibacter.acanthamoeba GCA_000815465.1.UWC36
100/100
Rickettsia.bellii.RML369.C GCA_000012385.1_ASM1238v1
57.3/86
Candidatus.Megaira.polyxenophila
100/100
100/99
Rickettsiales.endosymbiont.of.Stachyamoeba.lipophora GCA_003932735.1_ASM393273v1
Rickettsiales.bacterium.Ac37b GCA_000746585.2_ASM74658v2
100/100
GCA_002787635.1_ASM278763v1*
GCA_002422795.1_ASM242279v1*
100/100
Magnetofaba.australis.IT.1 GCA_002109495.1_ASM210949v1
Magnetococcus.marinus.MC.1 GCA_000014865.1_ASM1486v1
97.8/99
Nitrosospira.briensis.C.128 GCA_000619905.2_ASM61990v2
100/100
Methylotenera.versatilis.301 GCA_000093025.1_ASM9302v1
100/100
100/100
Ralstonia.pickettii GCA_902374465.1.MGYG.HGUT.01384
Burkholderia.cepacia GCA_009586235.1_ASM958623v1
100/100
Dasania.marina.DSM.21967 GCA_000373485.1_ASM37348v1
100/100
Congregibacter.litoralis.KT71 GCA_000153125.2_ASM15312v2
100/100
Enterobacter.cloacae GCA_000770155.1_ASM77015v1
Alteromonas.macleodii.ATCC.27126 GCA_000172635.2_ASM17263v2
0.55861

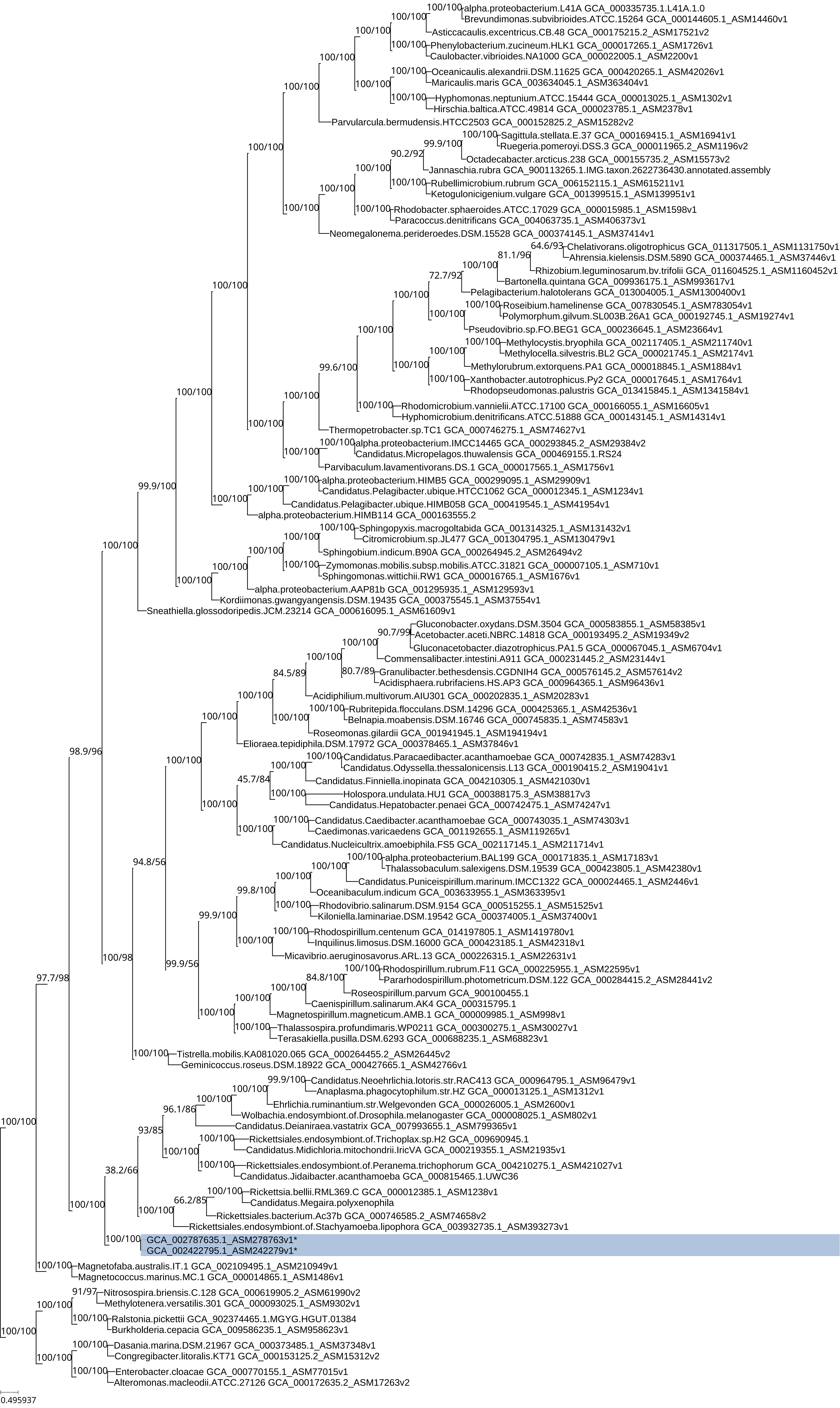
alpha.proteobacterium.L41A GCA_000335735.1.L41A.1.0
Brevundimonas.subvibrioides.ATCC.15264 GCA_000144605.1_ASM14460v1
Asticcacaulis.excentricus.CB.48 GCA_000175215.2_ASM17521v2
Phenylobacterium.zucineum.HLK1 GCA_000017265.1_ASM1726v1
Caulobacter.vibrioides.NA1000 GCA_000022005.1_ASM2200v1
Oceanicaulis.alexandrii.DSM.11625 GCA_000420265.1_ASM42026v1
Maricaulis.maris GCA_003634045.1_ASM363404v1
Hyphomonas.neptunium.ATCC.15444 GCA_000013025.1_ASM1302v1
Hirschia.baltica.ATCC.49814 GCA_000023785.1_ASM2378v1
Parvularcula.bermudensis.HTCC2503 GCA_000152825.2_ASM15282v2
Sagittula.stellata.E.37 GCA_000169415.1_ASM16941v1
Ruegeria.pomeroyi.DSS.3 GCA_000011965.2_ASM1196v2
Octadecabacter.arcticus.238 GCA_000155735.2_ASM15573v2
Jannaschia.rubra GCA_900113265.1.IMG.taxon.2622736430.annotated.assembly
Rubellimicrobium.rubrum GCA_006152115.1_ASM615211v1
Ketogulonicigenium.vulgare GCA_001399515.1_ASM139951v1
Rhodobacter.sphaeroides.ATCC.17029 GCA_000015985.1_ASM1598v1
Paracoccus.denitrificans GCA_004063735.1_ASM406373v1
Neomegalonema.perideroedes.DSM.15528 GCA_000374145.1_ASM37414v1
Chelativorans.oligotrophicus GCA_011317505.1_ASM1131750v1
Ahrensia.kielensis.DSM.5890 GCA_000374465.1_ASM37446v1
Rhizobium.leguminosarum.bv.trifolii GCA_011604525.1_ASM1160452v1
Bartonella.quintana GCA_009936175.1_ASM993617v1
Pelagibacterium.halotolerans GCA_013004005.1_ASM1300400v1
Roseibium.hamelinense GCA_007830545.1_ASM783054v1
Polymorphum.gilvum.SL003B.26A1 GCA_000192745.1_ASM19274v1
Pseudovibrio.sp.FO.BEG1 GCA_000236645.1_ASM23664v1
Methylocystis.bryophila GCA_002117405.1_ASM211740v1
Methylocella.silvestris.BL2 GCA_000021745.1_ASM2174v1
Methylorubrum.extorquens.PA1 GCA_000018845.1_ASM1884v1
Xanthobacter.autotrophicus.Py2 GCA_000017645.1_ASM1764v1
Rhodopseudomonas.palustris GCA_013415845.1_ASM1341584v1
Rhodomicrobium.vannielii.ATCC.17100 GCA_000166055.1_ASM16605v1
Hyphomicrobium.denitrificans.ATCC.51888 GCA_000143145.1_ASM14314v1
Thermopetrobacter.sp.TC1 GCA_000746275.1_ASM74627v1
alpha.proteobacterium.IMCC14465 GCA_000293845.2_ASM29384v2
Candidatus.Micropelagos.thuwalensis GCA_000469155.1.RS24
Parvibaculum.lavamentivorans.DS.1 GCA_000017565.1_ASM1756v1
alpha.proteobacterium.HIMB5 GCA_000299095.1_ASM29909v1
Candidatus.Pelagibacter.ubique.HTCC1062 GCA_000012345.1_ASM1234v1
Candidatus.Pelagibacter.ubique.HIMB058 GCA_000419545.1_ASM41954v1
alpha.proteobacterium.HIMB114 GCA_000163555.2
Sphingopyxis.macrogoltabida GCA_001314325.1_ASM131432v1
Citromicrobium.sp.JL477 GCA_001304795.1_ASM130479v1
Sphingobium.indicum.B90A GCA_000264945.2_ASM26494v2
Zymomonas.mobilis.subsp.mobilis.ATCC.31821 GCA_000007105.1_ASM710v1
Sphingomonas.wittichii.RW1 GCA_000016765.1_ASM1676v1
alpha.proteobacterium.AAP81b GCA_001295935.1_ASM129593v1
Kordiimonas.gwangyangensis.DSM.19435 GCA_000375545.1_ASM37554v1
Sneathiella.glossodoripedis.JCM.23214 GCA_000616095.1_ASM61609v1
Gluconobacter.oxydans.DSM.3504 GCA_000583855.1_ASM58385v1
Acetobacter.aceti.NBRC.14818 GCA_000193495.2_ASM19349v2
Gluconacetobacter.diazotrophicus.PA1.5 GCA_000067045.1_ASM6704v1
Commensalibacter.intestini.A911 GCA_000231445.2_ASM23144v1
Granulibacter.bethesdensis.CGDNIH4 GCA_000576145.2_ASM57614v2
Acidisphaera.rubrifaciens.HS.AP3 GCA_000964365.1_ASM96436v1
Acidiphilium.multivorum.AIU301 GCA_000202835.1_ASM20283v1
Rubritepida.flocculans.DSM.14296 GCA_000425365.1_ASM42536v1
Belnapia.moabensis.DSM.16746 GCA_000745835.1_ASM74583v1
Roseomonas.gilardii GCA_001941945.1_ASM194194v1
Elioraea.tepidiphila.DSM.17972 GCA_000378465.1_ASM37846v1
Candidatus.Paracaedibacter.acanthamoebae GCA_000742835.1_ASM74283v1
Candidatus.Odyssella.thessalonicensis.L13 GCA_000190415.2_ASM19041v1
Candidatus.Finniella.inopinata GCA_004210305.1_ASM421030v1
Holospora.undulata.HU1 GCA_000388175.3_ASM38817v3
Candidatus.Hepatobacter.penaei GCA_000742475.1_ASM74247v1
Candidatus.Caedibacter.acanthamoebae GCA_000743035.1_ASM74303v1
Caedimonas.varicaedens GCA_001192655.1_ASM119265v1
Candidatus.Nucleicultrix.amoebiphila.FS5 GCA_002117145.1_ASM211714v1
alpha.proteobacterium.BAL199 GCA_000171835.1_ASM17183v1
Thalassobaculum.salexigens.DSM.19539 GCA_000423805.1_ASM42380v1
Candidatus.Puniceispirillum.marinum.IMCC1322 GCA_000024465.1_ASM2446v1
Oceanibaculum.indicum GCA_003633955.1_ASM363395v1
Rhodovibrio.salinarum.DSM.9154 GCA_000515255.1_ASM51525v1
Kiloniella.laminariae.DSM.19542 GCA_000374005.1_ASM37400v1
Rhodospirillum.centenum GCA_014197805.1_ASM1419780v1
Inquilinus.limosus.DSM.16000 GCA_000423185.1_ASM42318v1
Micavibrio.aeruginosavorus.ARL.13 GCA_000226315.1_ASM22631v1
Rhodospirillum.rubrum.F11 GCA_000225955.1_ASM22595v1
Pararhodospirillum.photometricum.DSM.122 GCA_000284415.2_ASM28441v2
Roseospirillum.parvum GCA_900100455.1
Caenispirillum.salinarum.AK4 GCA_000315795.1
Magnetospirillum.magneticum.AMB.1 GCA_000009985.1_ASM998v1
Thalassospira.profundimaris.WP0211 GCA_000300275.1_ASM30027v1
Terasakiella.pusilla.DSM.6293 GCA_000688235.1_ASM68823v1
Tistrella.mobilis.KA081020.065 GCA_000264455.2_ASM26445v2
Geminicoccus.roseus.DSM.18922 GCA_000427665.1_ASM42766v1
Candidatus.Neoehrlichia.lotoris.str.RAC413 GCA_000964795.1_ASM96479v1
Anaplasma.phagocytophilum.str.HZ GCA_000013125.1_ASM1312v1
Ehrlichia.ruminantium.str.Welgevonden GCA_000026005.1_ASM2600v1
Wolbachia.endosymbiont.of.Drosophila.melanogaster GCA_000008025.1_ASM802v1
Candidatus.Deianiraea.vastatrix GCA_007993655.1_ASM799365v1
Rickettsiales.endosymbiont.of.Trichoplax.sp.H2 GCA_009690945.1
Candidatus.Midichloria.mitochondrii.IricVA GCA_000219355.1_ASM21935v1
Rickettsiales.endosymbiont.of.Peranema.trichophorum GCA_004210275.1_ASM421027v1
Candidatus.Jidaibacter.acanthamoeba GCA_000815465.1.UWC36
Rickettsia.bellii.RML369.C GCA_000012385.1_ASM1238v1
Candidatus.Megaira.polyxenophila
Rickettsiales.bacterium.Ac37b GCA_000746585.2_ASM74658v2
Rickettsiales.endosymbiont.of.Stachyamoeba.lipophora GCA_003932735.1_ASM393273v1
GCA_002787635.1_ASM278763v1*
GCA_002422795.1_ASM242279v1*
Magnetofaba.australis.IT.1 GCA_002109495.1_ASM210949v1
Magnetococcus.marinus.MC.1 GCA_000014865.1_ASM1486v1
Nitrosospira.briensis.C.128 GCA_000619905.2_ASM61990v2
Methylotenera.versatilis.301 GCA_000093025.1_ASM9302v1
Ralstonia.pickettii GCA_902374465.1.MGYG.HGUT.01384
Burkholderia.cepacia GCA_009586235.1_ASM958623v1
Dasania.marina.DSM.21967 GCA_000373485.1_ASM37348v1
Congregibacter.litoralis.KT71 GCA_000153125.2_ASM15312v2
Enterobacter.cloacae GCA_000770155.1_ASM77015v1
Alteromonas.macleodii.ATCC.27126 GCA_000172635.2_ASM17263v2
0.495937

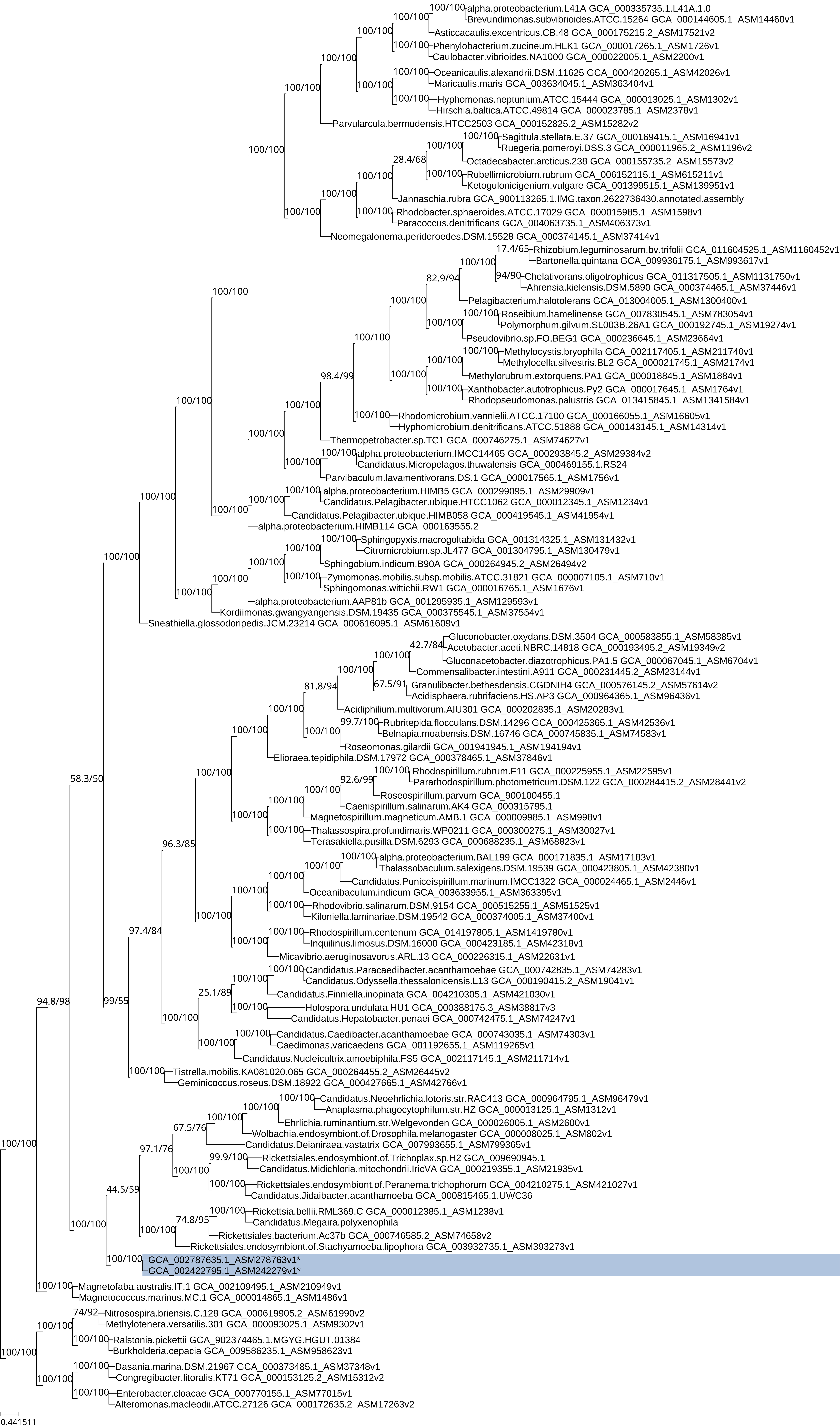
alpha.proteobacterium.L41A GCA_000335735.1.L41A.1.0
Brevundimonas.subvibrioides.ATCC.15264 GCA_000144605.1_ASM14460v1
Asticcacaulis.excentricus.CB.48 GCA_000175215.2_ASM17521v2
Phenylobacterium.zucineum.HLK1 GCA_000017265.1_ASM1726v1
Caulobacter.vibrioides.NA1000 GCA_000022005.1_ASM2200v1
Oceanicaulis.alexandrii.DSM.11625 GCA_000420265.1_ASM42026v1
Maricaulis.maris GCA_003634045.1_ASM363404v1
Hyphomonas.neptunium.ATCC.15444 GCA_000013025.1_ASM1302v1
Hirschia.baltica.ATCC.49814 GCA_000023785.1_ASM2378v1
Parvularcula.bermudensis.HTCC2503 GCA_000152825.2_ASM15282v2
Sagittula.stellata.E.37 GCA_000169415.1_ASM16941v1
Ruegeria.pomeroyi.DSS.3 GCA_000011965.2_ASM1196v2
Octadecabacter.arcticus.238 GCA_000155735.2_ASM15573v2
Rubellimicrobium.rubrum GCA_006152115.1_ASM615211v1
Ketogulonicigenium.vulgare GCA_001399515.1_ASM139951v1
Jannaschia.rubra GCA_900113265.1.IMG.taxon.2622736430.annotated.assembly
Rhodobacter.sphaeroides.ATCC.17029 GCA_000015985.1_ASM1598v1
Paracoccus.denitrificans GCA_004063735.1_ASM406373v1
Neomegalonema.perideroedes.DSM.15528 GCA_000374145.1_ASM37414v1
Rhizobium.leguminosarum.bv.trifolii GCA_011604525.1_ASM1160452v1
Bartonella.quintana GCA_009936175.1_ASM993617v1
Chelativorans.oligotrophicus GCA_011317505.1_ASM1131750v1
Ahrensia.kielensis.DSM.5890 GCA_000374465.1_ASM37446v1
Pelagibacterium.halotolerans GCA_013004005.1_ASM1300400v1
Roseibium.hamelinense GCA_007830545.1_ASM783054v1
Polymorphum.gilvum.SL003B.26A1 GCA_000192745.1_ASM19274v1
Pseudovibrio.sp.FO.BEG1 GCA_000236645.1_ASM23664v1
Methylocystis.bryophila GCA_002117405.1_ASM211740v1
Methylocella.silvestris.BL2 GCA_000021745.1_ASM2174v1
Methylorubrum.extorquens.PA1 GCA_000018845.1_ASM1884v1
Xanthobacter.autotrophicus.Py2 GCA_000017645.1_ASM1764v1
Rhodopseudomonas.palustris GCA_013415845.1_ASM1341584v1
Rhodomicrobium.vannielii.ATCC.17100 GCA_000166055.1_ASM16605v1
Hyphomicrobium.denitrificans.ATCC.51888 GCA_000143145.1_ASM14314v1
Thermopetrobacter.sp.TC1 GCA_000746275.1_ASM74627v1
alpha.proteobacterium.IMCC14465 GCA_000293845.2_ASM29384v2
Candidatus.Micropelagos.thuwalensis GCA_000469155.1.RS24
Parvibaculum.lavamentivorans.DS.1 GCA_000017565.1_ASM1756v1
alpha.proteobacterium.HIMB5 GCA_000299095.1_ASM29909v1
Candidatus.Pelagibacter.ubique.HTCC1062 GCA_000012345.1_ASM1234v1
Candidatus.Pelagibacter.ubique.HIMB058 GCA_000419545.1_ASM41954v1
alpha.proteobacterium.HIMB114 GCA_000163555.2
Sphingopyxis.macrogoltabida GCA_001314325.1_ASM131432v1
Citromicrobium.sp.JL477 GCA_001304795.1_ASM130479v1
Sphingobium.indicum.B90A GCA_000264945.2_ASM26494v2
Zymomonas.mobilis.subsp.mobilis.ATCC.31821 GCA_000007105.1_ASM710v1
Sphingomonas.wittichii.RW1 GCA_000016765.1_ASM1676v1
alpha.proteobacterium.AAP81b GCA_001295935.1_ASM129593v1
Kordiimonas.gwangyangensis.DSM.19435 GCA_000375545.1_ASM37554v1
Sneathiella.glossodoripedis.JCM.23214 GCA_000616095.1_ASM61609v1
Gluconobacter.oxydans.DSM.3504 GCA_000583855.1_ASM58385v1
Acetobacter.aceti.NBRC.14818 GCA_000193495.2_ASM19349v2
Gluconacetobacter.diazotrophicus.PA1.5 GCA_000067045.1_ASM6704v1
Commensalibacter.intestini.A911 GCA_000231445.2_ASM23144v1
Granulibacter.bethesdensis.CGDNIH4 GCA_000576145.2_ASM57614v2
Acidisphaera.rubrifaciens.HS.AP3 GCA_000964365.1_ASM96436v1
Acidiphilium.multivorum.AIU301 GCA_000202835.1_ASM20283v1
Rubritepida.flocculans.DSM.14296 GCA_000425365.1_ASM42536v1
Belnapia.moabensis.DSM.16746 GCA_000745835.1_ASM74583v1
Roseomonas.gilardii GCA_001941945.1_ASM194194v1
Elioraea.tepidiphila.DSM.17972 GCA_000378465.1_ASM37846v1
Rhodospirillum.rubrum.F11 GCA_000225955.1_ASM22595v1
Pararhodospirillum.photometricum.DSM.122 GCA_000284415.2_ASM28441v2
Roseospirillum.parvum GCA_900100455.1
Caenispirillum.salinarum.AK4 GCA_000315795.1
Magnetospirillum.magneticum.AMB.1 GCA_000009985.1_ASM998v1
Thalassospira.profundimaris.WP0211 GCA_000300275.1_ASM30027v1
Terasakiella.pusilla.DSM.6293 GCA_000688235.1_ASM68823v1
alpha.proteobacterium.BAL199 GCA_000171835.1_ASM17183v1
Thalassobaculum.salexigens.DSM.19539 GCA_000423805.1_ASM42380v1
Candidatus.Puniceispirillum.marinum.IMCC1322 GCA_000024465.1_ASM2446v1
Oceanibaculum.indicum GCA_003633955.1_ASM363395v1
Rhodovibrio.salinarum.DSM.9154 GCA_000515255.1_ASM51525v1
Kiloniella.laminariae.DSM.19542 GCA_000374005.1_ASM37400v1
Rhodospirillum.centenum GCA_014197805.1_ASM1419780v1
Inquilinus.limosus.DSM.16000 GCA_000423185.1_ASM42318v1
Micavibrio.aeruginosavorus.ARL.13 GCA_000226315.1_ASM22631v1
Candidatus.Paracaedibacter.acanthamoebae GCA_000742835.1_ASM74283v1
Candidatus.Odyssella.thessalonicensis.L13 GCA_000190415.2_ASM19041v1
Candidatus.Finniella.inopinata GCA_004210305.1_ASM421030v1
Holospora.undulata.HU1 GCA_000388175.3_ASM38817v3
Candidatus.Hepatobacter.penaei GCA_000742475.1_ASM74247v1
Candidatus.Caedibacter.acanthamoebae GCA_000743035.1_ASM74303v1
Caedimonas.varicaedens GCA_001192655.1_ASM119265v1
Candidatus.Nucleicultrix.amoebiphila.FS5 GCA_002117145.1_ASM211714v1
Tistrella.mobilis.KA081020.065 GCA_000264455.2_ASM26445v2
Geminicoccus.roseus.DSM.18922 GCA_000427665.1_ASM42766v1
Candidatus.Neoehrlichia.lotoris.str.RAC413 GCA_000964795.1_ASM96479v1
Anaplasma.phagocytophilum.str.HZ GCA_000013125.1_ASM1312v1
Ehrlichia.ruminantium.str.Welgevonden GCA_000026005.1_ASM2600v1
Wolbachia.endosymbiont.of.Drosophila.melanogaster GCA_000008025.1_ASM802v1
Candidatus.Deianiraea.vastatrix GCA_007993655.1_ASM799365v1
Rickettsiales.endosymbiont.of.Trichoplax.sp.H2 GCA_009690945.1
Candidatus.Midichloria.mitochondrii.IricVA GCA_000219355.1_ASM21935v1
Rickettsiales.endosymbiont.of.Peranema.trichophorum GCA_004210275.1_ASM421027v1
Candidatus.Jidaibacter.acanthamoeba GCA_000815465.1.UWC36
Rickettsia.bellii.RML369.C GCA_000012385.1_ASM1238v1
Candidatus.Megaira.polyxenophila
Rickettsiales.bacterium.Ac37b GCA_000746585.2_ASM74658v2
Rickettsiales.endosymbiont.of.Stachyamoeba.lipophora GCA_003932735.1_ASM393273v1
GCA_002787635.1_ASM278763v1*
GCA_002422795.1_ASM242279v1*
Magnetofaba.australis.IT.1 GCA_002109495.1_ASM210949v1
Magnetococcus.marinus.MC.1 GCA_000014865.1_ASM1486v1
Nitrosospira.briensis.C.128 GCA_000619905.2_ASM61990v2
Methylotenera.versatilis.301 GCA_000093025.1_ASM9302v1
Ralstonia.pickettii GCA_902374465.1.MGYG.HGUT.01384
Burkholderia.cepacia GCA_009586235.1_ASM958623v1
Dasania.marina.DSM.21967 GCA_000373485.1_ASM37348v1
Congregibacter.litoralis.KT71 GCA_000153125.2_ASM15312v2
Enterobacter.cloacae GCA_000770155.1_ASM77015v1
Alteromonas.macleodii.ATCC.27126 GCA_000172635.2_ASM17263v2
0.441511

Group G

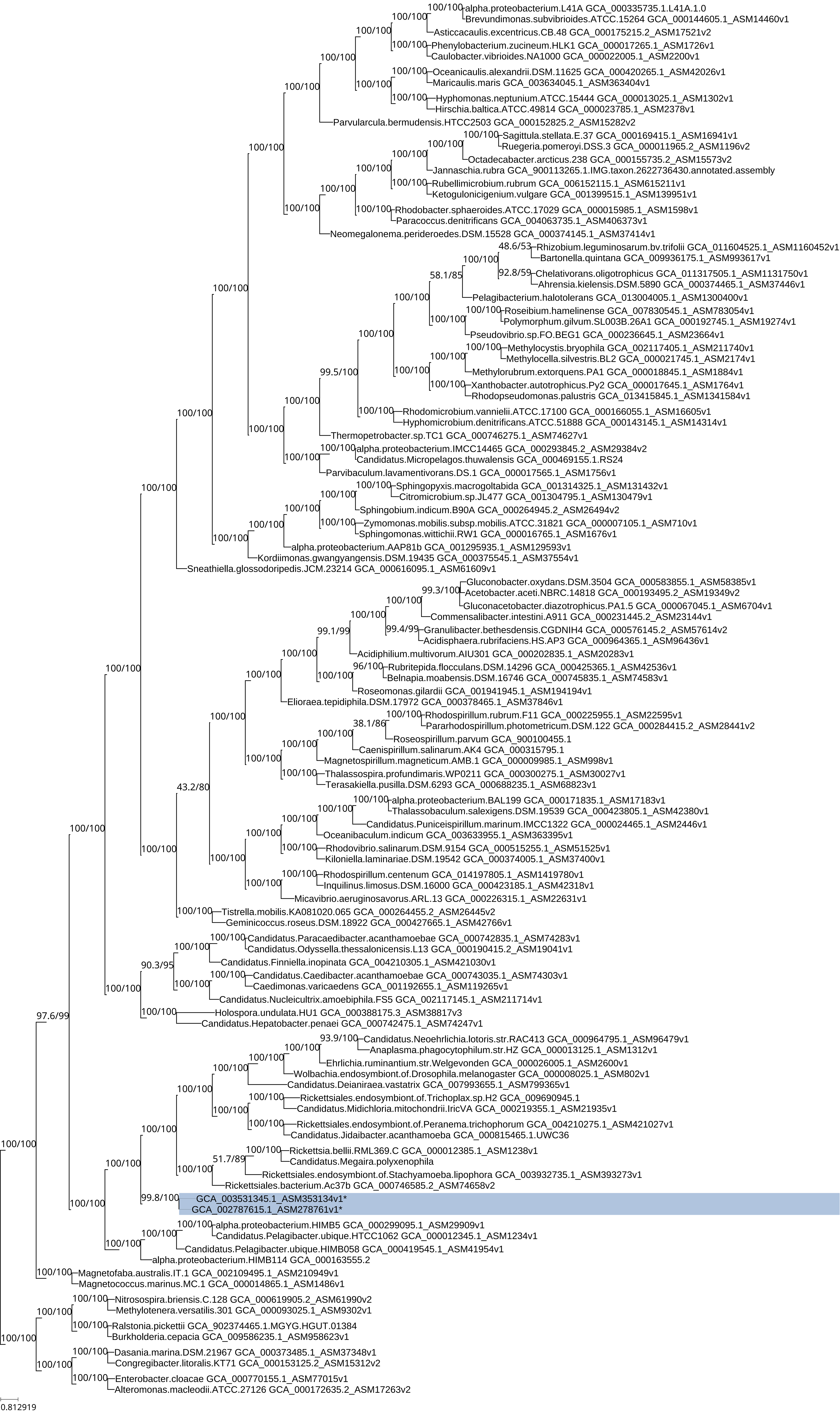
alpha.proteobacterium.L41A GCA_000335735.1_L41A.1.0
Brevundimonas.subvibrioides.ATCC.15264 GCA_000144605.1_ASM14460v1
Asticcacaulis.excentricus.CB.48 GCA_000175215.2_ASM17521v2
Phenylobacterium.zucineum.HLK1 GCA_000017265.1_ASM1726v1
Caulobacter.vibrioides.NA1000 GCA_000022005.1_ASM2200v1
Oceanicaulis.alexandrii.DSM.11625 GCA_000420265.1_ASM42026v1
Maricaulis.maris GCA_003634045.1_ASM363404v1
Hyphomonas.neptunium.ATCC.15444 GCA_000013025.1_ASM1302v1
Hirschia.baltica.ATCC.49814 GCA_000023785.1_ASM2378v1
Parvularcula.bermudensis.HTCC2503 GCA_000152825.2_ASM15282v2
Sagittula.stellata.E.37 GCA_000169415.1_ASM16941v1
Ruegeria.pomeroyi.DSS.3 GCA_000011965.2_ASM1196v2
Octadecabacter.arcticus.238 GCA_000155735.2_ASM15573v2
Jannaschia.rubra GCA_900113265.1.IMG.taxon.2622736430.annotated.assembly
Rubellimicrobium.rubrum GCA_006152115.1_ASM615211v1
Ketogulonicigenium.vulgare GCA_001399515.1_ASM139951v1
Rhodobacter.sphaeroides.ATCC.17029 GCA_000015985.1_ASM1598v1
Paracoccus.denitrificans GCA_004063735.1_ASM406373v1
Neomegalonema.perideroedes.DSM.15528 GCA_000374145.1_ASM37414v1
Rhizobium.leguminosarum.bv.trifolii GCA_011604525.1_ASM1160452v1
Bartonella.quintana GCA_009936175.1_ASM993617v1
Chelativorans.oligotrophicus GCA_011317505.1_ASM1131750v1
Ahrensia.kielensis.DSM.5890 GCA_000374465.1_ASM37446v1
Pelagibacterium.halotolerans GCA_013004005.1_ASM1300400v1
Roseibium.hamelinense GCA_007830545.1_ASM783054v1
Polymorphum.gilvum.SL003B.26A1 GCA_000192745.1_ASM19274v1
Pseudovibrio.sp.FO.BEG1 GCA_000236645.1_ASM23664v1
Methylocystis.bryophila GCA_002117405.1_ASM211740v1
Methylocella.silvestris.BL2 GCA_000021745.1_ASM2174v1
Methylorubrum.extorquens.PA1 GCA_000018845.1_ASM1884v1
Xanthobacter.autotrophicus.Py2 GCA_000017645.1_ASM1764v1
Rhodopseudomonas.palustris GCA_013415845.1_ASM1341584v1
Rhodomicrobium.vannielii.ATCC.17100 GCA_000166055.1_ASM16605v1
Hyphomicrobium.denitrificans.ATCC.51888 GCA_000143145.1_ASM14314v1
Thermopetrobacter.sp.TC1 GCA_000746275.1_ASM74627v1
alpha.proteobacterium.IMCC14465 GCA_000293845.2_ASM29384v2
Candidatus.Micropelagos.thuwalensis GCA_000469155.1.RS24
Parvibaculum.lavamentivorans.DS.1 GCA_000017565.1_ASM1756v1
Sphingopyxis.macrogoltabida GCA_001314325.1_ASM131432v1
Citromicrobium.sp.JL477 GCA_001304795.1_ASM130479v1
Sphingobium.indicum.B90A GCA_000264945.2_ASM26494v2
Zymomonas.mobilis.subsp.mobilis.ATCC.31821 GCA_000007105.1_ASM710v1
Sphingomonas.wittichii.RW1 GCA_000016765.1_ASM1676v1
alpha.proteobacterium.AAP81b GCA_001295935.1_ASM129593v1
Kordiimonas.gwangyangensis.DSM.19435 GCA_000375545.1_ASM37554v1
Sneathiella.glossodoripedis.JCM.23214 GCA_000616095.1_ASM61609v1
Gluconobacter.oxydans.DSM.3504 GCA_000583855.1_ASM58385v1
Acetobacter.aceti.NBRC.14818 GCA_000193495.2_ASM19349v2
Gluconacetobacter.diazotrophicus.PA1.5 GCA_000067045.1_ASM6704v1
Commensalibacter.intestini.A911 GCA_000231445.2_ASM23144v1
Granulibacter.bethesdensis.CGDNIH4 GCA_000576145.2_ASM57614v2
Acidisphaera.rubrifaciens.HS.AP3 GCA_000964365.1_ASM96436v1
Acidiphilium.multivorum.AIU301 GCA_000202835.1_ASM20283v1
Rubritepida.flocculans.DSM.14296 GCA_000425365.1_ASM42536v1
Belnapia.moabensis.DSM.16746 GCA_000745835.1_ASM74583v1
Roseomonas.gilardii GCA_001941945.1_ASM194194v1
Elioraea.tepidiphila.DSM.17972 GCA_000378465.1_ASM37846v1
Rhodospirillum.rubrum.F11 GCA_000225955.1_ASM22595v1
Pararhodospirillum.photometricum.DSM.122 GCA_000284415.2_ASM28441v2
Roseospirillum.parvum GCA_900100455.1
Caenispirillum.salinarum.AK4 GCA_000315795.1
Magnetospirillum.magneticum.AMB.1 GCA_000009985.1_ASM998v1
Thalassospira.profundimaris.WP0211 GCA_000300275.1_ASM30027v1
Terasakiella.pusilla.DSM.6293 GCA_000688235.1_ASM68823v1
alpha.proteobacterium.BAL199 GCA_000171835.1_ASM17183v1
Thalassobaculum.salexigens.DSM.19539 GCA_000423805.1_ASM42380v1
Candidatus.Puniceispirillum.marinum.IMCC1322 GCA_000024465.1_ASM2446v1
Oceanibaculum.indicum GCA_003633955.1_ASM363395v1
Rhodovibrio.salinarum.DSM.9154 GCA_000515255.1_ASM51525v1
Kiloniella.laminariae.DSM.19542 GCA_000374005.1_ASM37400v1
Rhodospirillum.centenum GCA_014197805.1_ASM1419780v1
Inquilinus.limosus.DSM.16000 GCA_000423185.1_ASM42318v1
Micavibrio.aeruginosavorus.ARL.13 GCA_000226315.1_ASM22631v1
Tistrella.mobilis.KA081020.065 GCA_000264455.2_ASM26445v2
Geminicoccus.roseus.DSM.18922 GCA_000427665.1_ASM42766v1
Candidatus.Paracaedibacter.acanthamoebae GCA_000742835.1_ASM74283v1
Candidatus.Odyssella.thessalonicensis.L13 GCA_000190415.2_ASM19041v1
Candidatus.Finniella.inopinata GCA_004210305.1_ASM421030v1
Candidatus.Caedibacter.acanthamoebae GCA_000743035.1_ASM74303v1
Caedimonas.varicaedens GCA_001192655.1_ASM119265v1
Candidatus.Nucleicultrix.amoebiphila.FS5 GCA_002117145.1_ASM211714v1
Holospora.undulata.HU1 GCA_000388175.3_ASM38817v3
Candidatus.Hepatobacter.penaei GCA_000742475.1_ASM74247v1
Candidatus.Neoehrlichia.lotoris.str.RAC413 GCA_000964795.1_ASM96479v1
Anaplasma.phagocytophilum.str.HZ GCA_000013125.1_ASM1312v1
Ehrlichia.ruminantium.str.Welgevonden GCA_000026005.1_ASM2600v1
Wolbachia.endosymbiont.of.Drosophila.melanogaster GCA_000008025.1_ASM802v1
Candidatus.Deianiraea.vastatrix GCA_007993655.1_ASM799365v1
Rickettsiales.endosymbiont.of.Trichoplax.sp.H2 GCA_009690945.1
Candidatus.Midichloria.mitochondrii.IricVA GCA_000219355.1_ASM21935v1
Rickettsiales.endosymbiont.of.Peranema.trichophorum GCA_004210275.1_ASM421027v1
Candidatus.Jidaibacter.acanthamoeba GCA_000815465.1.UWC36
Rickettsia.bellii.RML369.C GCA_000012385.1_ASM1238v1
Candidatus.Megaira.polyxenophila
Rickettsiales.endosymbiont.of.Stachyamoeba.lipophora GCA_003932735.1_ASM393273v1
Rickettsiales.bacterium.Ac37b GCA_000746585.2_ASM74658v2
GCA_003531345.1_ASM353134v1*
GCA_002787615.1_ASM278761v1*
alpha.proteobacterium.HIMB5 GCA_000299095.1_ASM29909v1
Candidatus.Pelagibacter.ubique.HTCC1062 GCA_000012345.1_ASM1234v1
Candidatus.Pelagibacter.ubique.HIMB058 GCA_000419545.1_ASM41954v1
alpha.proteobacterium.HIMB114 GCA_000163555.2
Magnetofaba.australis.IT.1 GCA_002109495.1_ASM210949v1
Magnetococcus.marinus.MC.1 GCA_000014865.1_ASM1486v1
Nitrosospira.briensis.C.128 GCA_000619905.2_ASM61990v2
Methylotenera.versatilis.301 GCA_000093025.1_ASM9302v1
Ralstonia.pickettii GCA_902374465.1.MGYG.HGUT.01384
Burkholderia.cepacia GCA_009586235.1_ASM958623v1
Dasania.marina.DSM.21967 GCA_000373485.1_ASM37348v1
Congregibacter.litoralis.KT71 GCA_000153125.2_ASM15312v2
Enterobacter.cloacae GCA_000770155.1_ASM77015v1
Alteromonas.macleodii.ATCC.27126 GCA_000172635.2_ASM17263v2
0.812919

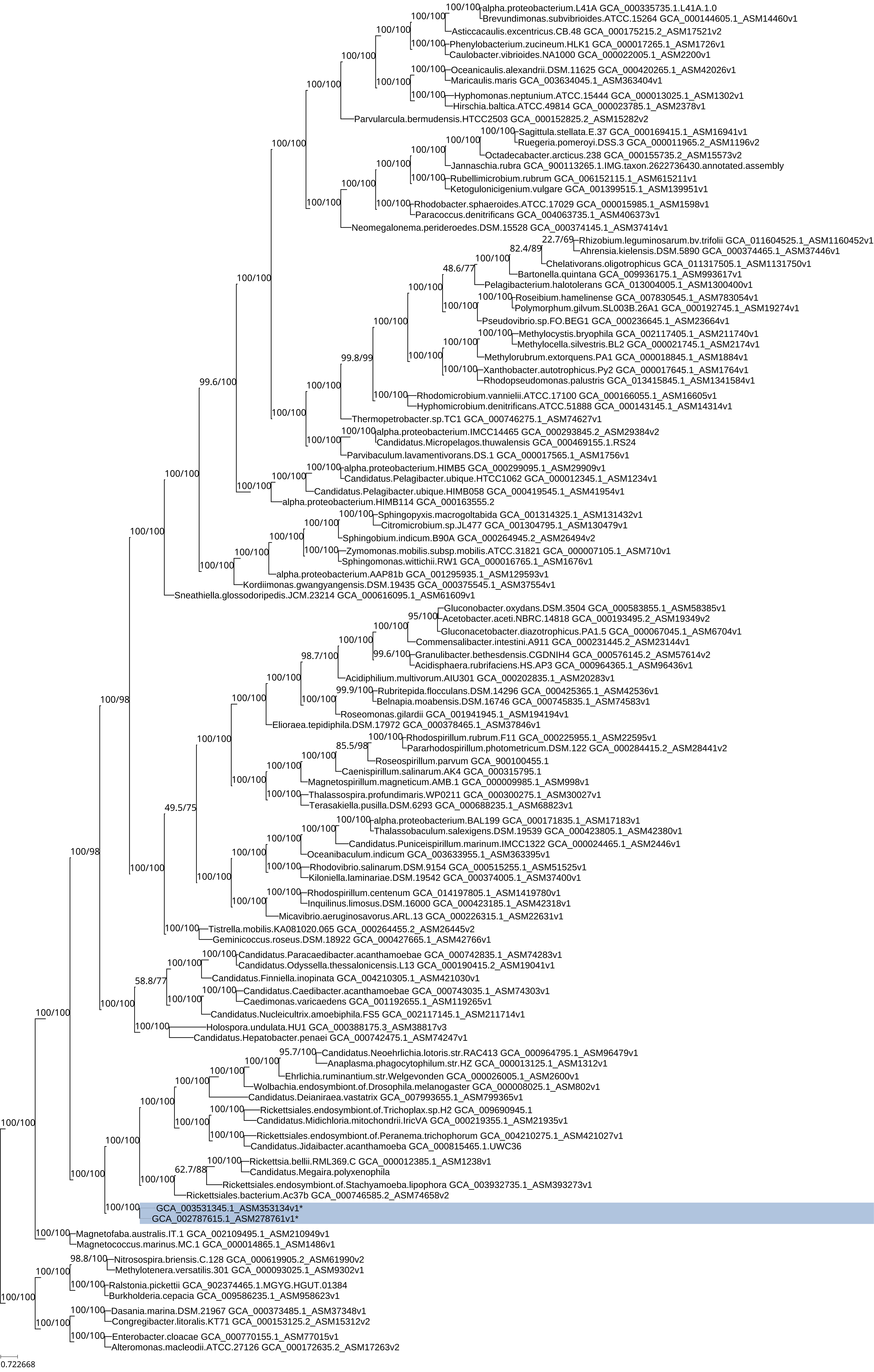
100/100
alpha.proteobacterium.L41A GCA_000335735.1_L41A.1.0
Brevundimonas.subvibrioides.ATCC.15264 GCA_000144605.1_ASM14460v1
100/100
100/100
Asticcacaulis.excentricus.CB.48 GCA_000175215.2_ASM17521v2
100/100
Phenylobacterium.zucineum.HLK1 GCA_000017265.1_ASM1726v1
100/100
Caulobacter.vibrioides.NA1000 GCA_000022005.1_ASM2200v1
100/100
Oceanicaulis.alexandrii.DSM.11625 GCA_000420265.1_ASM42026v1
100/100
Maricaulis.maris GCA_003634045.1_ASM363404v1
100/100
100/100
Hyphomonas.neptunium.ATCC.15444 GCA_000013025.1_ASM1302v1
Hirschia.baltica.ATCC.49814 GCA_000023785.1_ASM2378v1
Parvularcula.bermudensis.HTCC2503 GCA_000152825.2_ASM15282v2
100/100
Sagittula.stellata.E.37 GCA_000169415.1_ASM16941v1
100/100
100/100
Ruegeria.pomeroyi.DSS.3 GCA_000011965.2_ASM1196v2
100/100
Octadecabacter.arcticus.238 GCA_000155735.2_ASM15573v2
100/100
Jannaschia.rubra GCA_900113265.1.IMG.taxon.2622736430.annotated.assembly
100/100
Rubellimicrobium.rubrum GCA_006152115.1_ASM615211v1
100/100
Ketogulonicigenium.vulgare GCA_001399515.1_ASM139951v1
100/100
100/100
Rhodobacter.sphaeroides.ATCC.17029 GCA_000015985.1_ASM1598v1
Paracoccus.denitrificans GCA_004063735.1_ASM406373v1
Neomegalonema.perideroedes.DSM.15528 GCA_000374145.1_ASM37414v1
22.7/69
Rhizobium.leguminosarum.bv.trifolii GCA_011604525.1_ASM1160452v1
82.4/89
Ahrensia.kielensis.DSM.5890 GCA_000374465.1_ASM37446v1
100/100
100/100
Chelativorans.oligotrophicus GCA_011317505.1_ASM1131750v1
48.6/77
Bartonella.quintana GCA_009936175.1_ASM993617v1
Pelagibacterium.halotolerans GCA_013004005.1_ASM1300400v1
100/100
100/100
Roseibium.hamelinense GCA_007830545.1_ASM783054v1
100/100
Polymorphum.gilvum.SL003B.26A1 GCA_000192745.1_ASM19274v1
Pseudovibrio.sp.FO.BEG1 GCA_000236645.1_ASM23664v1
100/100
100/100
Methylocystis.bryophila GCA_002117405.1_ASM211740v1
100/100
Methylocella.silvestris.BL2 GCA_000021745.1_ASM2174v1
100/100
Methylorubrum.extorquens.PA1 GCA_000018845.1_ASM1884v1
99.8/99
100/100
Xanthobacter.autotrophicus.Py2 GCA_000017645.1_ASM1764v1
Rhodopseudomonas.palustris GCA_013415845.1_ASM1341584v1
99.6/100
100/100
100/100
Rhodomicrobium.vannielii.ATCC.17100 GCA_000166055.1_ASM16605v1
Hyphomicrobium.denitrificans.ATCC.51888 GCA_000143145.1_ASM14314v1
100/100
Thermopetrobacter.sp.TC1 GCA_000746275.1_ASM74627v1
100/100
alpha.proteobacterium.IMCC14465 GCA_000293845.2_ASM29384v2
100/100
Candidatus.Micropelagos.thuwalensis GCA_000469155.1.RS24
Parvibaculum.lavamentivorans.DS.1 GCA_000017565.1_ASM1756v1
100/100
100/100
alpha.proteobacterium.HIMB5 GCA_000299095.1_ASM29909v1
100/100
Candidatus.Pelagibacter.ubique.HTCC1062 GCA_000012345.1_ASM1234v1
100/100
Candidatus.Pelagibacter.ubique.HIMB058 GCA_000419545.1_ASM41954v1
alpha.proteobacterium.HIMB114 GCA_000163555.2
100/100
Sphingopyxis.macrogoltabida GCA_001314325.1_ASM131432v1
100/100
Citromicrobium.sp.JL477 GCA_001304795.1_ASM130479v1
100/100
100/100
Sphingobium.indicum.B90A GCA_000264945.2_ASM26494v2
100/100
Zymomonas.mobilis.subsp.mobilis.ATCC.31821 GCA_000007105.1_ASM710v1
Sphingomonas.wittichii.RW1 GCA_000016765.1_ASM1676v1
100/100
alpha.proteobacterium.AAP81b GCA_001295935.1_ASM129593v1
Kordiimonas.gwangyangensis.DSM.19435 GCA_000375545.1_ASM37554v1
Sneathiella.glossodoripedis.JCM.23214 GCA_000616095.1_ASM61609v1
Gluconobacter.oxydans.DSM.3504 GCA_000583855.1_ASM58385v1
95/100
Acetobacter.aceti.NBRC.14818 GCA_000193495.2_ASM19349v2
100/100
Gluconacetobacter.diazotrophicus.PA1.5 GCA_000067045.1_ASM6704v1
100/100
Commensalibacter.intestini.A911 GCA_000231445.2_ASM23144v1
99.6/100
Granulibacter.bethesdensis.CGDNIH4 GCA_000576145.2_ASM57614v2
98.7/100
Acidisphaera.rubrifaciens.HS.AP3 GCA_000964365.1_ASM96436v1
100/98
100/100
Acidiphilium.multivorum.AIU301 GCA_000202835.1_ASM20283v1
99.9/100
Rubritepida.flocculans.DSM.14296 GCA_000425365.1_ASM42536v1
100/100
100/100
Belnapia.moabensis.DSM.16746 GCA_000745835.1_ASM74583v1
Roseomonas.gilardii GCA_001941945.1_ASM194194v1
Elioraea.tepidiphila.DSM.17972 GCA_000378465.1_ASM37846v1
100/100
Rhodospirillum.rubrum.F11 GCA_000225955.1_ASM22595v1
85.5/98
100/100
Pararhodospirillum.photometricum.DSM.122 GCA_000284415.2_ASM28441v2
100/100
Roseospirillum.parvum GCA_900100455.1
100/100
Caenispirillum.salinarum.AK4 GCA_000315795.1
100/100
Magnetospirillum.magneticum.AMB.1 GCA_000009985.1_ASM998v1
100/100
Thalassospira.profundimaris.WP0211 GCA_000300275.1_ASM30027v1
Terasakiella.pusilla.DSM.6293 GCA_000688235.1_ASM68823v1
100/100
alpha.proteobacterium.BAL199 GCA_000171835.1_ASM17183v1
100/100
Thalassobaculum.salexigens.DSM.19539 GCA_000423805.1_ASM42380v1
49.5/75
100/100
Candidatus.Puniceispirillum.marinum.IMCC1322 GCA_000024465.1_ASM2446v1
100/98
100/100
Oceanibaculum.indicum GCA_003633955.1_ASM363395v1
100/100
Rhodovibrio.salinarum.DSM.9154 GCA_000515255.1_ASM51525v1
100/100
100/100
Kiloniella.laminariae.DSM.19542 GCA_000374005.1_ASM37400v1
100/100
Rhodospirillum.centenum GCA_014197805.1_ASM1419780v1
100/100
Inquilinus.limosus.DSM.16000 GCA_000423185.1_ASM42318v1
Micavibrio.aeruginosavorus.ARL.13 GCA_000226315.1_ASM22631v1
100/100
Tistrella.mobilis.KA081020.065 GCA_000264455.2_ASM26445v2
Geminicoccus.roseus.DSM.18922 GCA_000427665.1_ASM42766v1
100/100
Candidatus.Paracaedibacter.acanthamoebae GCA_000742835.1_ASM74283v1
100/100
Candidatus.Odyssella.thessalonicensis.L13 GCA_000190415.2_ASM19041v1
58.8/77
Candidatus.Finniella.inopinata GCA_004210305.1_ASM421030v1
100/100
Candidatus.Caedibacter.acanthamoebae GCA_000743035.1_ASM74303v1
100/100
100/100
Caedimonas.varicaedens GCA_001192655.1_ASM119265v1
100/100
Candidatus.Nucleicultrix.amoebiphila.FS5 GCA_002117145.1_ASM211714v1
100/100
Holospora.undulata.HU1 GCA_000388175.3_ASM38817v3
Candidatus.Hepatobacter.penaei GCA_000742475.1_ASM74247v1
95.7/100
Candidatus.Neoehrlichia.lotoris.str.RAC413 GCA_000964795.1_ASM96479v1
100/100
Anaplasma.phagocytophilum.str.HZ GCA_000013125.1_ASM1312v1
100/100
Ehrlichia.ruminantium.str.Welgevonden GCA_000026005.1_ASM2600v1
100/100
Wolbachia.endosymbiont.of.Drosophila.melanogaster GCA_000008025.1_ASM802v1
Candidatus.Deianiraea.vastatrix GCA_007993655.1_ASM799365v1
100/100
100/100
Rickettsiales.endosymbiont.of.Trichoplax.sp.H2 GCA_009690945.1
100/100
100/100
Candidatus.Midichloria.mitochondrii.IricVA GCA_000219355.1_ASM21935v1
100/100
Rickettsiales.endosymbiont.of.Peranema.trichophorum GCA_004210275.1_ASM421027v1
100/100
Candidatus.Jidaibacter.acanthamoeba GCA_000815465.1.UWC36
100/100
Rickettsia.bellii.RML369.C GCA_000012385.1_ASM1238v1
62.7/88
Candidatus.Megaira.polyxenophila
100/100
100/100
Rickettsiales.endosymbiont.of.Stachyamoeba.lipophora GCA_003932735.1_ASM393273v1
Rickettsiales.bacterium.Ac37b GCA_000746585.2_ASM74658v2
100/100
GCA_003531345.1_ASM353134v1*
GCA_002787615.1_ASM278761v1*
100/100
Magnetofaba.australis.IT.1 GCA_002109495.1_ASM210949v1
Magnetococcus.marinus.MC.1 GCA_000014865.1_ASM1486v1
98.8/100
Nitrosospira.briensis.C.128 GCA_000619905.2_ASM61990v2
100/100
Methylotenera.versatilis.301 GCA_000093025.1_ASM9302v1
100/100
Ralstonia.pickettii GCA_902374465.1.MGYG.HGUT.01384
100/100
Burkholderia.cepacia GCA_009586235.1_ASM958623v1
100/100
Dasania.marina.DSM.21967 GCA_000373485.1_ASM37348v1
100/100
Congregibacter.litoralis.KT71 GCA_000153125.2_ASM15312v2
100/100
Enterobacter.cloacae GCA_000770155.1_ASM77015v1
Alteromonas.macleodii.ATCC.27126 GCA_000172635.2_ASM17263v2
0.722668

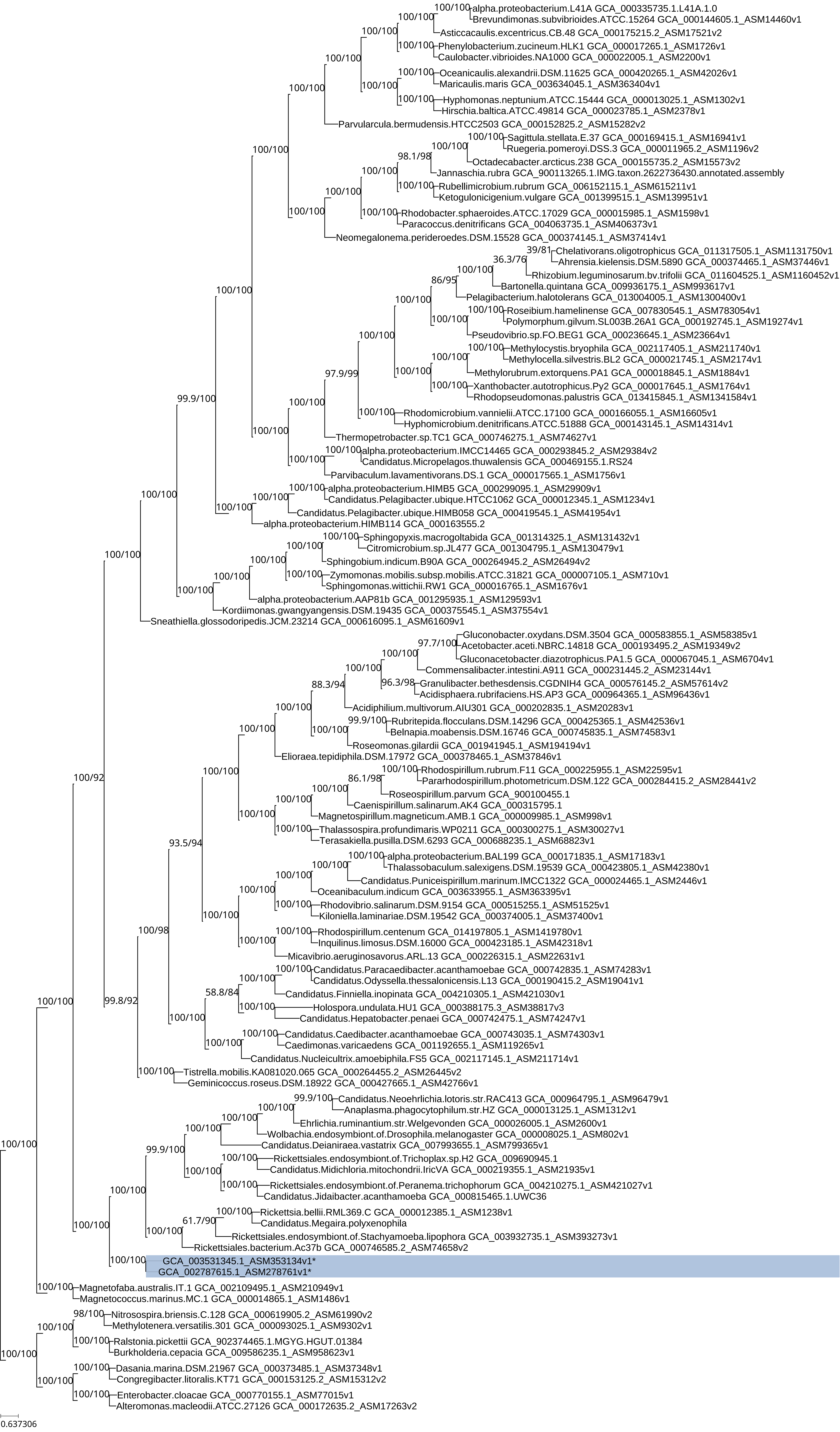

100/100
alpha.proteobacterium.L41A GCA_000335735.1.L41A.1.0
100/100
Brevundimonas.subvibrioides.ATCC.15264 GCA_000144605.1_ASM14460v1
Asticcacaulis.excentricus.CB.48 GCA_000175215.2_ASM17521v2
100/100
Phenylobacterium.zucineum.HLK1 GCA_000017265.1_ASM1726v1
100/100
Caulobacter.vibrioides.NA1000 GCA_000022005.1_ASM2200v1
100/100
Oceanicaulis.alexandrii.DSM.11625 GCA_000420265.1_ASM42026v1
100/100
Maricaulis.maris GCA_003634045.1_ASM363404v1
100/100
Hyphomonas.neptunium.ATCC.15444 GCA_000013025.1_ASM1302v1
Hirschia.baltica.ATCC.49814 GCA_000023785.1_ASM2378v1
Parvularcula.bermudensis.HTCC2503 GCA_000152825.2_ASM15282v2
100/100
Sagittula.stellata.E.37 GCA_000169415.1_ASM16941v1
100/100
Ruegeria.pomeroyi.DSS.3 GCA_000011965.2_ASM1196v2
100/100
Octadecabacter.arcticus.238 GCA_000155735.2_ASM15573v2
98.1/98
Jannaschia.rubra GCA_900113265.1.IMG.taxon.2622736430.annotated.assembly
100/100
100/100
Rubellimicrobium.rubrum GCA_006152115.1_ASM615211v1
Ketogulonicigenium.vulgare GCA_001399515.1_ASM139951v1
100/100
100/100
Rhodobacter.sphaeroides.ATCC.17029 GCA_000015985.1_ASM1598v1
Paracoccus.denitrificans GCA_004063735.1_ASM406373v1
Neomegalonema.perideroedes.DSM.15528 GCA_000374145.1_ASM37414v1
39/81
Chelativorans.oligotrophicus GCA_011317505.1_ASM1131750v1
Ahrensia.kielensis.DSM.5890 GCA_000374465.1_ASM37446v1
36.3/76
100/100
Rhizobium.leguminosarum.bv.trifolii GCA_011604525.1_ASM1160452v1
86/95
Bartonella.quintana GCA_009936175.1_ASM993617v1
100/100
Pelagibacterium.halotolerans GCA_013004005.1_ASM1300400v1
100/100
Roseibium.hamelinense GCA_007830545.1_ASM783054v1
100/100
Polymorphum.gilvum.SL003B.26A1 GCA_000192745.1_ASM19274v1
Pseudovibrio.sp.FO.BEG1 GCA_000236645.1_ASM23664v1
100/100
100/100
Methylocystis.bryophila GCA_002117405.1_ASM211740v1
100/100
Methylocella.silvestris.BL2 GCA_000021745.1_ASM2174v1
100/100
Methylorubrum.extorquens.PA1 GCA_000018845.1_ASM1884v1
100/100
Xanthobacter.autotrophicus.Py2 GCA_000017645.1_ASM1764v1
Rhodopseudomonas.palustris GCA_013415845.1_ASM1341584v1
99.9/100
97.9/99
100/100
Rhodomicrobium.vannielii.ATCC.17100 GCA_000166055.1_ASM16605v1
100/100
Hyphomicrobium.denitrificans.ATCC.51888 GCA_000143145.1_ASM14314v1
100/100
Thermopetrobacter.sp.TC1 GCA_000746275.1_ASM74627v1
100/100
alpha.proteobacterium.IMCC14465 GCA_000293845.2_ASM29384v2
100/100
Candidatus.Micropelagos.thuwalensis GCA_000469155.1.RS24
Parvibaculum.lavamentivorans.DS.1 GCA_000017565.1_ASM1756v1
100/100
alpha.proteobacterium.HIMB5 GCA_000299095.1_ASM29909v1
100/100
100/100
Candidatus.Pelagibacter.ubique.HTCC1062 GCA_000012345.1_ASM1234v1
100/100
Candidatus.Pelagibacter.ubique.HIMB058 GCA_000419545.1_ASM41954v1
alpha.proteobacterium.HIMB114 GCA_000163555.2
100/100
Sphingopyxis.macrogoltabida GCA_001314325.1_ASM131432v1
100/100
Citromicrobium.sp.JL477 GCA_001304795.1_ASM130479v1
100/100
Sphingobium.indicum.B90A GCA_000264945.2_ASM26494v2
100/100
Zymomonas.mobilis.subsp.mobilis.ATCC.31821 GCA_000007105.1_ASM710v1
100/100
100/100
Sphingomonas.wittichii.RW1 GCA_000016765.1_ASM1676v1
100/100
alpha.proteobacterium.AAP81b GCA_001295935.1_ASM129593v1
Kordiimonas.gwangyangensis.DSM.19435 GCA_000375545.1_ASM37554v1
Sneathiella.glossodoripedis.JCM.23214 GCA_000616095.1_ASM61609v1
Gluconobacter.oxydans.DSM.3504 GCA_000583855.1_ASM58385v1
97.7/100
Acetobacter.aceti.NBRC.14818 GCA_000193495.2_ASM19349v2
100/100
Gluconacetobacter.diazotrophicus.PA1.5 GCA_000067045.1_ASM6704v1
100/100
Commensalibacter.intestini.A911 GCA_000231445.2_ASM23144v1
96.3/98
Granulibacter.bethesdensis.CGDNIH4 GCA_000576145.2_ASM57614v2
88.3/94
Acidisphaera.rubrifaciens.HS.AP3 GCA_000964365.1_ASM96436v1
100/100
Acidiphilium.multivorum.AIU301 GCA_000202835.1_ASM20283v1
99.9/100
Rubritepida.flocculans.DSM.14296 GCA_000425365.1_ASM42536v1
100/100
Belnapia.moabensis.DSM.16746 GCA_000745835.1_ASM74583v1
100/100
Roseomonas.gilardii GCA_001941945.1_ASM194194v1
Elioraea.tepidiphila.DSM.17972 GCA_000378465.1_ASM37846v1
100/100
100/100
Rhodospirillum.rubrum.F11 GCA_000225955.1_ASM22595v1
86.1/98
Pararhodospirillum.photometricum.DSM.122 GCA_000284415.2_ASM28441v2
100/92
100/100
Roseospirillum.parvum GCA_900100455.1
100/100
Caenispirillum.salinarum.AK4 GCA_000315795.1
100/100
Magnetospirillum.magneticum.AMB.1 GCA_000009985.1_ASM998v1
100/100
Thalassospira.profundimaris.WP0211 GCA_000300275.1_ASM30027v1
Terasakiella.pusilla.DSM.6293 GCA_000688235.1_ASM68823v1
93.5/94
100/100
alpha.proteobacterium.BAL199 GCA_000171835.1_ASM17183v1
100/100
Thalassobaculum.salexigens.DSM.19539 GCA_000423805.1_ASM42380v1
100/100
Candidatus.Puniceispirillum.marinum.IMCC1322 GCA_000024465.1_ASM2446v1
100/100
Oceanibaculum.indicum GCA_003633955.1_ASM363395v1
100/100
Rhodovibrio.salinarum.DSM.9154 GCA_000515255.1_ASM51525v1
100/100
Kiloniella.laminariae.DSM.19542 GCA_000374005.1_ASM37400v1
100/100
Rhodospirillum.centenum GCA_014197805.1_ASM1419780v1
100/98
100/100
Inquilinus.limosus.DSM.16000 GCA_000423185.1_ASM42318v1
Micavibrio.aeruginosavorus.ARL.13 GCA_000226315.1_ASM22631v1
100/100
Candidatus.Paracaedibacter.acanthamoebae GCA_000742835.1_ASM74283v1
100/100
Candidatus.Odyssella.thessalonicensis.L13 GCA_000190415.2_ASM19041v1
100/100
58.8/84
Candidatus.Finniella.inopinata GCA_004210305.1_ASM421030v1
99.8/92
100/100
Holospora.undulata.HU1 GCA_000388175.3_ASM38817v3
100/100
Candidatus.Hepatobacter.penaei GCA_000742475.1_ASM74247v1
100/100
Candidatus.Caedibacter.acanthamoebae GCA_000743035.1_ASM74303v1
100/100
Caedimonas.varicaedens GCA_001192655.1_ASM119265v1
Candidatus.Nucleicultrix.amoebiphila.FS5 GCA_002117145.1_ASM211714v1
100/100
Tistrella.mobilis.KA081020.065 GCA_000264455.2_ASM26445v2
Geminicoccus.roseus.DSM.18922 GCA_000427665.1_ASM42766v1
99.9/100
Candidatus.Neoehrlichia.lotoris.str.RAC413 GCA_000964795.1_ASM96479v1
100/100
Anaplasma.phagocytophilum.str.HZ GCA_000013125.1_ASM1312v1
100/100
Ehrlichia.ruminantium.str.Welgevonden GCA_000026005.1_ASM2600v1
100/100
Wolbachia.endosymbiont.of.Drosophila.melanogaster GCA_000008025.1_ASM802v1
Candidatus.Deianiraea.vastatrix GCA_007993655.1_ASM799365v1
100/100
99.9/100
Rickettsiales.endosymbiont.of.Trichoplax.sp.H2 GCA_009690945.1
100/100
Candidatus.Midichloria.mitochondrii.IricVA GCA_000219355.1_ASM21935v1
100/100
Rickettsiales.endosymbiont.of.Peranema.trichophorum GCA_004210275.1_ASM421027v1
100/100
Candidatus.Jidaibacter.acanthamoeba GCA_000815465.1.UWC36
100/100
Rickettsia.bellii.RML369.C GCA_000012385.1_ASM1238v1
100/100
61.7/90
Candidatus.Megaira.polyxenophila
100/100
Rickettsiales.endosymbiont.of.Stachyamoeba.lipophora GCA_003932735.1_ASM393273v1
Rickettsiales.bacterium.Ac37b GCA_000746585.2_ASM74658v2
100/100
GCA_003531345.1_ASM353134v1*
GCA_002787615.1_ASM278761v1*
100/100
Magnetofaba.australis.IT.1 GCA_002109495.1_ASM210949v1
Magnetococcus.marinus.MC.1 GCA_000014865.1_ASM1486v1
98/100
Nitrosospira.briensis.C.128 GCA_000619905.2_ASM61990v2
100/100
Methylotenera.versatilis.301 GCA_000093025.1_ASM9302v1
100/100
Ralstonia.pickettii GCA_902374465.1.MGYG.HGUT.01384
100/100
Burkholderia.cepacia GCA_009586235.1_ASM958623v1
100/100
Dasania.marina.DSM.21967 GCA_000373485.1_ASM37348v1
100/100
Congregibacter.litoralis.KT71 GCA_000153125.2_ASM15312v2
100/100
Enterobacter.cloacae GCA_000770155.1_ASM77015v1
Alteromonas.macleodii.ATCC.27126 GCA_000172635.2_ASM17263v2
0.637306

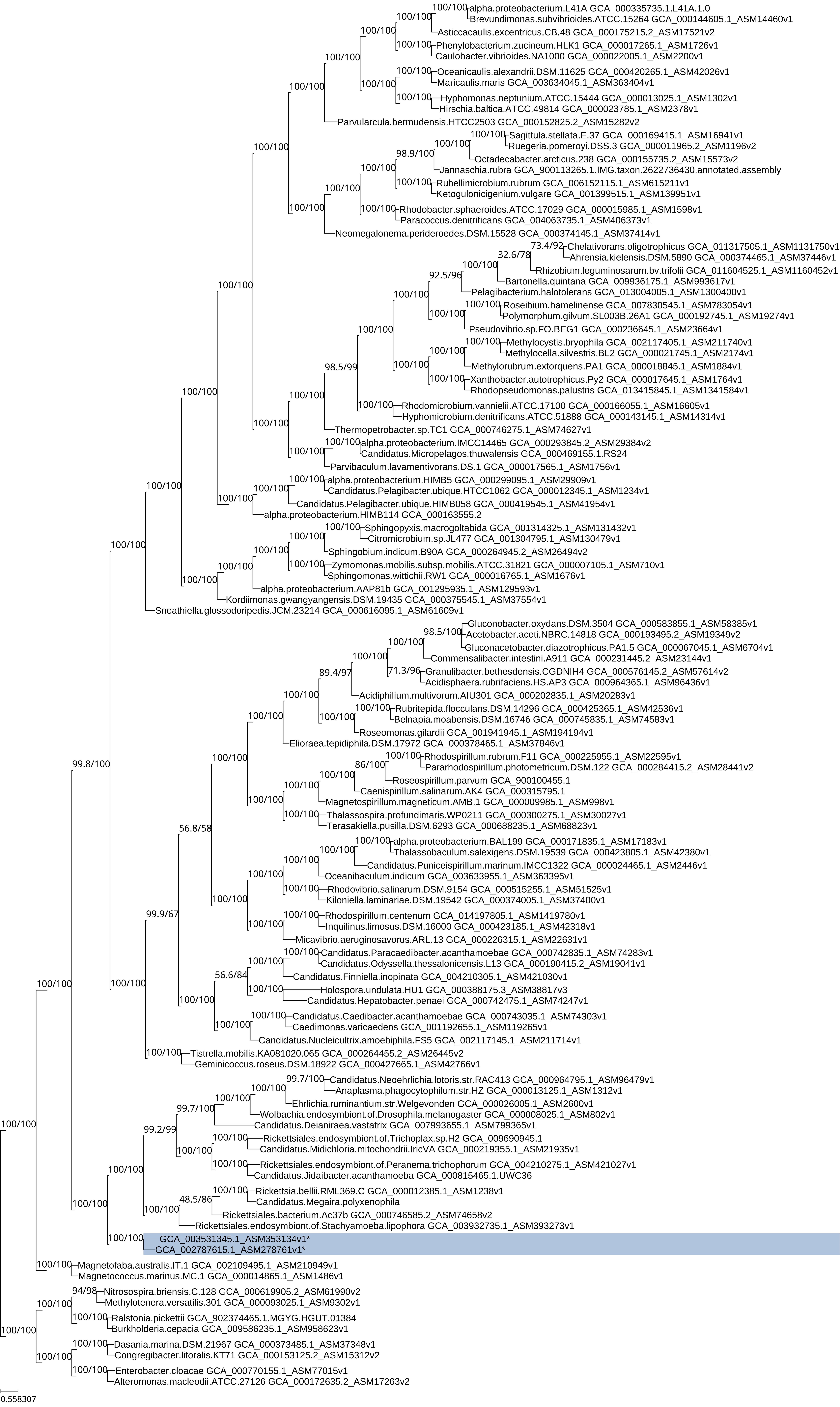
alpha.proteobacterium.L41A GCA_000335735.1.L41A.1.0
Brevundimonas.subvibrioides.ATCC.15264 GCA_000144605.1_ASM14460v1
Asticcacaulis.excentricus.CB.48 GCA_000175215.2_ASM17521v2
Phenylobacterium.zucineum.HLK1 GCA_000017265.1_ASM1726v1
Caulobacter.vibrioides.NA1000 GCA_000022005.1_ASM2200v1
Oceanicaulis.alexandrii.DSM.11625 GCA_000420265.1_ASM42026v1
Maricaulis.maris GCA_003634045.1_ASM363404v1
Hyphomonas.neptunium.ATCC.15444 GCA_000013025.1_ASM1302v1
Hirschia.baltica.ATCC.49814 GCA_000023785.1_ASM2378v1
Parvularcula.bermudensis.HTCC2503 GCA_000152825.2_ASM15282v2
Sagittula.stellata.E.37 GCA_000169415.1_ASM16941v1
Ruegeria.pomeroyi.DSS.3 GCA_000011965.2_ASM1196v2
Octadecabacter.arcticus.238 GCA_000155735.2_ASM15573v2
Jannaschia.rubra GCA_900113265.1.IMG.taxon.2622736430.annotated.assembly
Rubellimicrobium.rubrum GCA_006152115.1_ASM615211v1
Ketogulonicigenium.vulgare GCA_001399515.1_ASM139951v1
Rhodobacter.sphaeroides.ATCC.17029 GCA_000015985.1_ASM1598v1
Paracoccus.denitrificans GCA_004063735.1_ASM406373v1
Neomegalonema.perideroedes.DSM.15528 GCA_000374145.1_ASM37414v1
Chelativorans.oligotrophicus GCA_011317505.1_ASM1131750v1
Ahrensia.kielensis.DSM.5890 GCA_000374465.1_ASM37446v1
Rhizobium.leguminosarum.bv.trifolii GCA_011604525.1_ASM1160452v1
Bartonella.quintana GCA_009936175.1_ASM993617v1
Pelagibacterium.halotolerans GCA_013004005.1_ASM1300400v1
Roseibium.hamelinense GCA_007830545.1_ASM783054v1
Polymorphum.gilvum.SL003B.26A1 GCA_000192745.1_ASM19274v1
Pseudovibrio.sp.FO.BEG1 GCA_000236645.1_ASM23664v1
Methylocystis.bryophila GCA_002117405.1_ASM211740v1
Methylocella.silvestris.BL2 GCA_000021745.1_ASM2174v1
Methylorubrum.extorquens.PA1 GCA_000018845.1_ASM1884v1
Xanthobacter.autotrophicus.Py2 GCA_000017645.1_ASM1764v1
Rhodopseudomonas.palustris GCA_013415845.1_ASM1341584v1
Rhodomicrobium.vannielii.ATCC.17100 GCA_000166055.1_ASM16605v1
Hyphomicrobium.denitrificans.ATCC.51888 GCA_000143145.1_ASM14314v1
Thermopetrobacter.sp.TC1 GCA_000746275.1_ASM74627v1
alpha.proteobacterium.IMCC14465 GCA_000293845.2_ASM29384v2
Candidatus.Micropelagos.thuwalensis GCA_000469155.1.RS24
Parvibaculum.lavamentivorans.DS.1 GCA_000017565.1_ASM1756v1
alpha.proteobacterium.HIMB5 GCA_000299095.1_ASM29909v1
Candidatus.Pelagibacter.ubique.HTCC1062 GCA_000012345.1_ASM1234v1
Candidatus.Pelagibacter.ubique.HIMB058 GCA_000419545.1_ASM41954v1
alpha.proteobacterium.HIMB114 GCA_000163555.2
Sphingopyxis.macrogoltabida GCA_001314325.1_ASM131432v1
Citromicrobium.sp.JL477 GCA_001304795.1_ASM130479v1
Sphingobium.indicum.B90A GCA_000264945.2_ASM26494v2
Zymomonas.mobilis.subsp.mobilis.ATCC.31821 GCA_000007105.1_ASM710v1
Sphingomonas.wittichii.RW1 GCA_000016765.1_ASM1676v1
alpha.proteobacterium.AAP81b GCA_001295935.1_ASM129593v1
Kordiimonas.gwangyangensis.DSM.19435 GCA_000375545.1_ASM37554v1
Sneathiella.glossodoripedis.JCM.23214 GCA_000616095.1_ASM61609v1
Gluconobacter.oxydans.DSM.3504 GCA_000583855.1_ASM58385v1
Acetobacter.aceti.NBRC.14818 GCA_000193495.2_ASM19349v2
Gluconacetobacter.diazotrophicus.PA1.5 GCA_000067045.1_ASM6704v1
Commensalibacter.intestini.A911 GCA_000231445.2_ASM23144v1
Granulibacter.bethesdensis.CGDNIH4 GCA_000576145.2_ASM57614v2
Acidisphaera.rubrifaciens.HS.AP3 GCA_000964365.1_ASM96436v1
Acidiphilium.multivorum.AIU301 GCA_000202835.1_ASM20283v1
Rubritepida.flocculans.DSM.14296 GCA_000425365.1_ASM42536v1
Belnapia.moabensis.DSM.16746 GCA_000745835.1_ASM74583v1
Roseomonas.gilardii GCA_001941945.1_ASM194194v1
Elioraea.tepidiphila.DSM.17972 GCA_000378465.1_ASM37846v1
Rhodospirillum.rubrum.F11 GCA_000225955.1_ASM22595v1
Pararhodospirillum.photometricum.DSM.122 GCA_000284415.2_ASM28441v2
Roseospirillum.parvum GCA_900100455.1
Caenispirillum.salinarum.AK4 GCA_000315795.1
Magnetospirillum.magneticum.AMB.1 GCA_000009985.1_ASM998v1
Thalassospira.profundimaris.WP0211 GCA_000300275.1_ASM30027v1
Terasakiella.pusilla.DSM.6293 GCA_000688235.1_ASM68823v1
alpha.proteobacterium.BAL199 GCA_000171835.1_ASM17183v1
Thalassobaculum.salexigens.DSM.19539 GCA_000423805.1_ASM42380v1
Candidatus.Puniceispirillum.marinum.IMCC1322 GCA_000024465.1_ASM2446v1
Oceanibaculum.indicum GCA_003633955.1_ASM363395v1
Rhodovibrio.salinarum.DSM.9154 GCA_000515255.1_ASM51525v1
Kiloniella.laminariae.DSM.19542 GCA_000374005.1_ASM37400v1
Rhodospirillum.centenum GCA_014197805.1_ASM1419780v1
Inquilinus.limosus.DSM.16000 GCA_000423185.1_ASM42318v1
Micavibrio.aeruginosavorus.ARL.13 GCA_000226315.1_ASM22631v1
Candidatus.Paracaedibacter.acanthamoebae GCA_000742835.1_ASM74283v1
Candidatus.Odyssella.thessalonicensis.L13 GCA_000190415.2_ASM19041v1
Candidatus.Finniella.inopinata GCA_004210305.1_ASM421030v1
Holospora.undulata.HU1 GCA_000388175.3_ASM38817v3
Candidatus.Hepatobacter.penaei GCA_000742475.1_ASM74247v1
Candidatus.Caedibacter.acanthamoebae GCA_000743035.1_ASM74303v1
Caedimonas.varicaedens GCA_001192655.1_ASM119265v1
Candidatus.Nucleicultrix.amoebiphila.FS5 GCA_002117145.1_ASM211714v1
Tistrella.mobilis.KA081020.065 GCA_000264455.2_ASM26445v2
Geminicoccus.roseus.DSM.18922 GCA_000427665.1_ASM42766v1
Candidatus.Neoehrlichia.lotoris.str.RAC413 GCA_000964795.1_ASM96479v1
Anaplasma.phagocytophilum.str.HZ GCA_000013125.1_ASM1312v1
Ehrlichia.ruminantium.str.Welgevonden GCA_000026005.1_ASM2600v1
Wolbachia.endosymbiont.of.Drosophila.melanogaster GCA_000008025.1_ASM802v1
Candidatus.Deianiraea.vastatrix GCA_007993655.1_ASM799365v1
Rickettsiales.endosymbiont.of.Trichoplax.sp.H2 GCA_009690945.1
Candidatus.Midichloria.mitochondrii.IricVA GCA_000219355.1_ASM21935v1
Rickettsiales.endosymbiont.of.Peranema.trichophorum GCA_004210275.1_ASM421027v1
Candidatus.Jidaibacter.acanthamoeba GCA_000815465.1.UWC36
Rickettsia.bellii.RML369.C GCA_000012385.1_ASM1238v1
Candidatus.Megaira.polyxenophila
Rickettsiales.bacterium.Ac37b GCA_000746585.2_ASM74658v2
Rickettsiales.endosymbiont.of.Stachyamoeba.lipophora GCA_003932735.1_ASM393273v1
GCA_003531345.1_ASM353134v1*
GCA_002787615.1_ASM278761v1*
Magnetofaba.australis.IT.1 GCA_002109495.1_ASM210949v1
Magnetococcus.marinus.MC.1 GCA_000014865.1_ASM1486v1
Nitrosospira.briensis.C.128 GCA_000619905.2_ASM61990v2
Methylotenera.versatilis.301 GCA_000093025.1_ASM9302v1
Ralstonia.pickettii GCA_902374465.1.MGYG.HGUT.01384
Burkholderia.cepacia GCA_009586235.1_ASM958623v1
Dasania.marina.DSM.21967 GCA_000373485.1_ASM37348v1
Congregibacter.litoralis.KT71 GCA_000153125.2_ASM15312v2
Enterobacter.cloacae GCA_000770155.1_ASM77015v1
Alteromonas.macleodii.ATCC.27126 GCA_000172635.2_ASM17263v2
0.558307

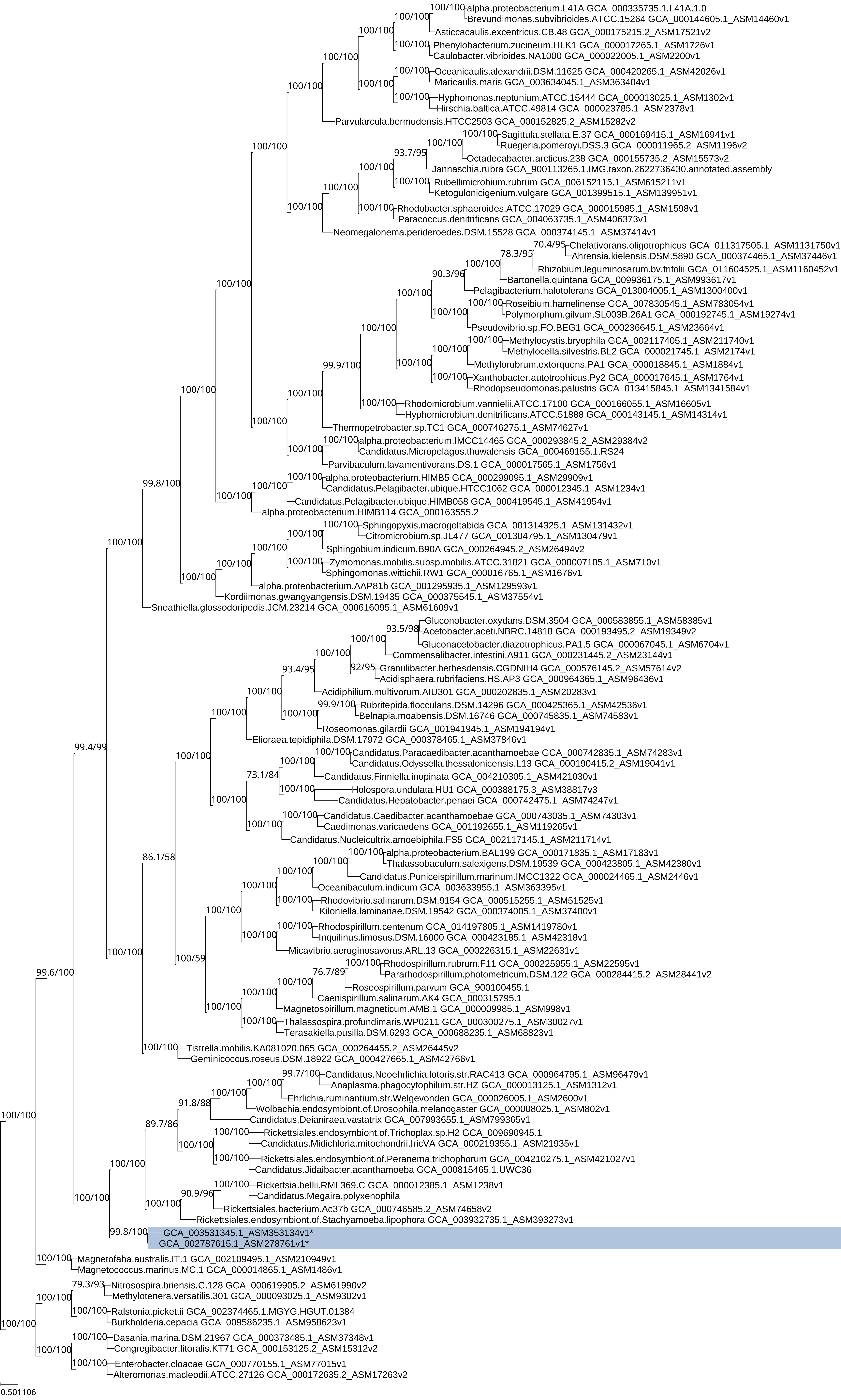
100/100
alpha.proteobacterium.L41A GCA_000335735.1.L41A.1.0
Brevundimonas.subvibrioides.ATCC.15264 GCA_000144605.1_ASM14460v1
100/100
Asticcacaulis.excentricus.CB.48 GCA_000175215.2_ASM17521v2
100/100
100/100
Phenylobacterium.zucineum.HLK1 GCA_000017265.1_ASM1726v1
Caulobacter.vibrioides.NA1000 GCA_000022005.1_ASM2200v1
100/100
100/100
Oceanicaulis.alexandrii.DSM.11625 GCA_000420265.1_ASM42026v1
Maricaulis.maris GCA_003634045.1_ASM363404v1
100/100
100/100
Hyphomonas.neptunium.ATCC.15444 GCA_000013025.1_ASM1302v1
Hirschia.baltica.ATCC.49814 GCA_000023785.1_ASM2378v1
100/100
Parvularcula.bermudensis.HTCC2503 GCA_000152825.2_ASM15282v2
100/100
Sagittula.stellata.E.37 GCA_000169415.1_ASM16941v1
Ruegeria.pomeroyi.DSS.3 GCA_000011965.2_ASM1196v2
100/100
Octadecabacter.arcticus.238 GCA_000155735.2_ASM15573v2
93.7/95
100/100
Jannaschia.rubra GCA_900113265.1.IMG.taxon.2622736430.annotated.assembly
100/100
Rubellimicrobium.rubrum GCA_006152115.1_ASM615211v1
100/100
Ketogulonicigenium.vulgare GCA_001399515.1_ASM139951v1
100/100
Rhodobacter.sphaeroides.ATCC.17029 GCA_000015985.1_ASM1598v1
100/100
Paracoccus.denitrificans GCA_004063735.1_ASM406373v1
Neomegalonema.perideroedes.DSM.15528 GCA_000374145.1_ASM37414v1
70.4/95
Chelativorans.oligotrophicus GCA_011317505.1_ASM1131750v1
78.3/95
Ahrensia.kielensis.DSM.5890 GCA_000374465.1_ASM37446v1
100/100
Rhizobium.leguminosarum.bv.trifolii GCA_011604525.1_ASM1160452v1
90.3/96
Bartonella.quintana GCA_009936175.1_ASM993617v1
100/100
Pelagibacterium.halotolerans GCA_013004005.1_ASM1300400v1
100/100
Roseibium.hamelinense GCA_007830545.1_ASM783054v1
100/100
100/100
Polymorphum.gilvum.SL003B.26A1 GCA_000192745.1_ASM19274v1
Pseudovibrio.sp.FO.BEG1 GCA_000236645.1_ASM23664v1
100/100
Methylocystis.bryophila GCA_002117405.1_ASM211740v1
100/100
Methylocella.silvestris.BL2 GCA_000021745.1_ASM2174v1
100/100
99.9/100
Methylorubrum.extorquens.PA1 GCA_000018845.1_ASM1884v1
100/100
Xanthobacter.autotrophicus.Py2 GCA_000017645.1_ASM1764v1
Rhodopseudomonas.palustris GCA_013415845.1_ASM1341584v1
100/100
100/100
Rhodomicrobium.vannielii.ATCC.17100 GCA_000166055.1_ASM16605v1
Hyphomicrobium.denitrificans.ATCC.51888 GCA_000143145.1_ASM14314v1
100/100
Thermopetrobacter.sp.TC1 GCA_000746275.1_ASM74627v1
100/100
alpha.proteobacterium.IMCC14465 GCA_000293845.2_ASM29384v2
100/100
Candidatus.Micropelagos.thuwalensis GCA_000469155.1.RS24
Parvibaculum.lavamentivorans.DS.1 GCA_000017565.1_ASM1756v1
100/100
alpha.proteobacterium.HIMB5 GCA_000299095.1_ASM29909v1
100/100
Candidatus.Pelagibacter.ubique.HTCC1062 GCA_000012345.1_ASM1234v1
99.8/100
100/100
Candidatus.Pelagibacter.ubique.HIMB058 GCA_000419545.1_ASM41954v1
alpha.proteobacterium.HIMB114 GCA_000163555.2
100/100
Sphingopyxis.macrogoltabida GCA_001314325.1_ASM131432v1
100/100
Citromicrobium.sp.JL477 GCA_001304795.1_ASM130479v1
100/100
100/100
Sphingobium.indicum.B90A GCA_000264945.2_ASM26494v2
100/100
Zymomonas.mobilis.subsp.mobilis.ATCC.31821 GCA_000007105.1_ASM710v1
100/100
Sphingomonas.wittichii.RW1 GCA_000016765.1_ASM1676v1
alpha.proteobacterium.AAP81b GCA_001295935.1_ASM129593v1
Kordiimonas.gwangyangensis.DSM.19435 GCA_000375545.1_ASM37554v1
Sneathiella.glossodoripedis.JCM.23214 GCA_000616095.1_ASM61609v1
Gluconobacter.oxydans.DSM.3504 GCA_000583855.1_ASM58385v1
93.5/98
Acetobacter.aceti.NBRC.14818 GCA_000193495.2_ASM19349v2
100/100
Gluconacetobacter.diazotrophicus.PA1.5 GCA_000067045.1_ASM6704v1
100/100
Commensalibacter.intestini.A911 GCA_000231445.2_ASM23144v1
93.4/95
92/95
Granulibacter.bethesdensis.CGDNIH4 GCA_000576145.2_ASM57614v2
Acidisphaera.rubrifaciens.HS.AP3 GCA_000964365.1_ASM96436v1
100/100
Acidiphilium.multivorum.AIU301 GCA_000202835.1_ASM20283v1
99.4/99
100/100
99.9/100
Rubritepida.flocculans.DSM.14296 GCA_000425365.1_ASM42536v1
100/100
Belnapia.moabensis.DSM.16746 GCA_000745835.1_ASM74583v1
Roseomonas.gilardii GCA_001941945.1_ASM194194v1
100/100
Elioraea.tepidiphila.DSM.17972 GCA_000378465.1_ASM37846v1
100/100
Candidatus.Paracaedibacter.acanthamoebae GCA_000742835.1_ASM74283v1
100/100
Candidatus.Odyssella.thessalonicensis.L13 GCA_000190415.2_ASM19041v1
73.1/84
Candidatus.Finniella.inopinata GCA_004210305.1_ASM421030v1
100/100
Holospora.undulata.HU1 GCA_000388175.3_ASM38817v3
100/100
Candidatus.Hepatobacter.penaei GCA_000742475.1_ASM74247v1
100/100
Candidatus.Caedibacter.acanthamoebae GCA_000743035.1_ASM74303v1
100/100
Caedimonas.varicaedens GCA_001192655.1_ASM119265v1
Candidatus.Nucleicultrix.amoebiphila.FS5 GCA_002117145.1_ASM211714v1
86.1/58
100/100
alpha.proteobacterium.BAL199 GCA_000171835.1_ASM17183v1
100/100
Thalassobaculum.salexigens.DSM.19539 GCA_000423805.1_ASM42380v1
100/100
Candidatus.Puniceispirillum.marinum.IMCC1322 GCA_000024465.1_ASM2446v1
100/100
Oceanibaculum.indicum GCA_003633955.1_ASM363395v1
100/100
100/100
Rhodovibrio.salinarum.DSM.9154 GCA_000515255.1_ASM51525v1
Kiloniella.laminariae.DSM.19542 GCA_000374005.1_ASM37400v1
100/100
Rhodospirillum.centenum GCA_014197805.1_ASM1419780v1
100/100
Inquilinus.limosus.DSM.16000 GCA_000423185.1_ASM42318v1
Micavibrio.aeruginosavorus.ARL.13 GCA_000226315.1_ASM22631v1
100/100
100/59
100/100
Rhodospirillum.rubrum.F11 GCA_000225955.1_ASM22595v1
76.7/89
Pararhodospirillum.photometricum.DSM.122 GCA_000284415.2_ASM28441v2
99.6/100
100/100
Roseospirillum.parvum GCA_900100455.1
100/100
Caenispirillum.salinarum.AK4 GCA_000315795.1
Magnetospirillum.magneticum.AMB.1 GCA_000009985.1_ASM998v1
100/100
100/100
Thalassospira.profundimaris.WP0211 GCA_000300275.1_ASM30027v1
Terasakiella.pusilla.DSM.6293 GCA_000688235.1_ASM68823v1
100/100
Tistrella.mobilis.KA081020.065 GCA_000264455.2_ASM26445v2
Geminicoccus.roseus.DSM.18922 GCA_000427665.1_ASM42766v1
99.7/100
Candidatus.Neoehrlichia.lotoris.str.RAC413 GCA_000964795.1_ASM96479v1
100/100
Anaplasma.phagocytophilum.str.HZ GCA_000013125.1_ASM1312v1
100/100
Ehrlichia.ruminantium.str.Welgevonden GCA_000026005.1_ASM2600v1
91.8/88
Wolbachia.endosymbiont.of.Drosophila.melanogaster GCA_000008025.1_ASM802v1
100/100
Candidatus.Deianiraea.vastatrix GCA_007993655.1_ASM799365v1
89.7/86
Rickettsiales.endosymbiont.of.Trichoplax.sp.H2 GCA_009690945.1
100/100
Candidatus.Midichloria.mitochondrii.IricVA GCA_000219355.1_ASM21935v1
100/100
100/100
Rickettsiales.endosymbiont.of.Peranema.trichophorum GCA_004210275.1_ASM421027v1
Candidatus.Jidaibacter.acanthamoeba GCA_000815465.1.UWC36
100/100
Rickettsia.bellii.RML369.C GCA_000012385.1_ASM1238v1
90.9/96
Candidatus.Megaira.polyxenophila
100/100
100/100
Rickettsiales.bacterium.Ac37b GCA_000746585.2_ASM74658v2
Rickettsiales.endosymbiont.of.Stachyamoeba.lipophora GCA_003932735.1_ASM393273v1
99.8/100
GCA_003531345.1_ASM353134v1*
GCA_002787615.1_ASM278761v1*
100/100
Magnetofaba.australis.IT.1 GCA_002109495.1_ASM210949v1
Magnetococcus.marinus.MC.1 GCA_000014865.1_ASM1486v1
79.3/93
Nitrosospira.briensis.C.128 GCA_000619905.2_ASM61990v2
100/100
Methylotenera.versatilis.301 GCA_000093025.1_ASM9302v1
100/100
Ralstonia.pickettii GCA_902374465.1.MGYG.HGUT.01384
Burkholderia.cepacia GCA_009586235.1_ASM958623v1
100/100
100/100
Dasania.marina.DSM.21967 GCA_000373485.1_ASM37348v1
Congregibacter.litoralis.KT71 GCA_000153125.2_ASM15312v2
100/100
100/100
Enterobacter.cloacae GCA_000770155.1_ASM77015v1
Alteromonas.macleodii.ATCC.27126 GCA_000172635.2_ASM17263v2
0.501106

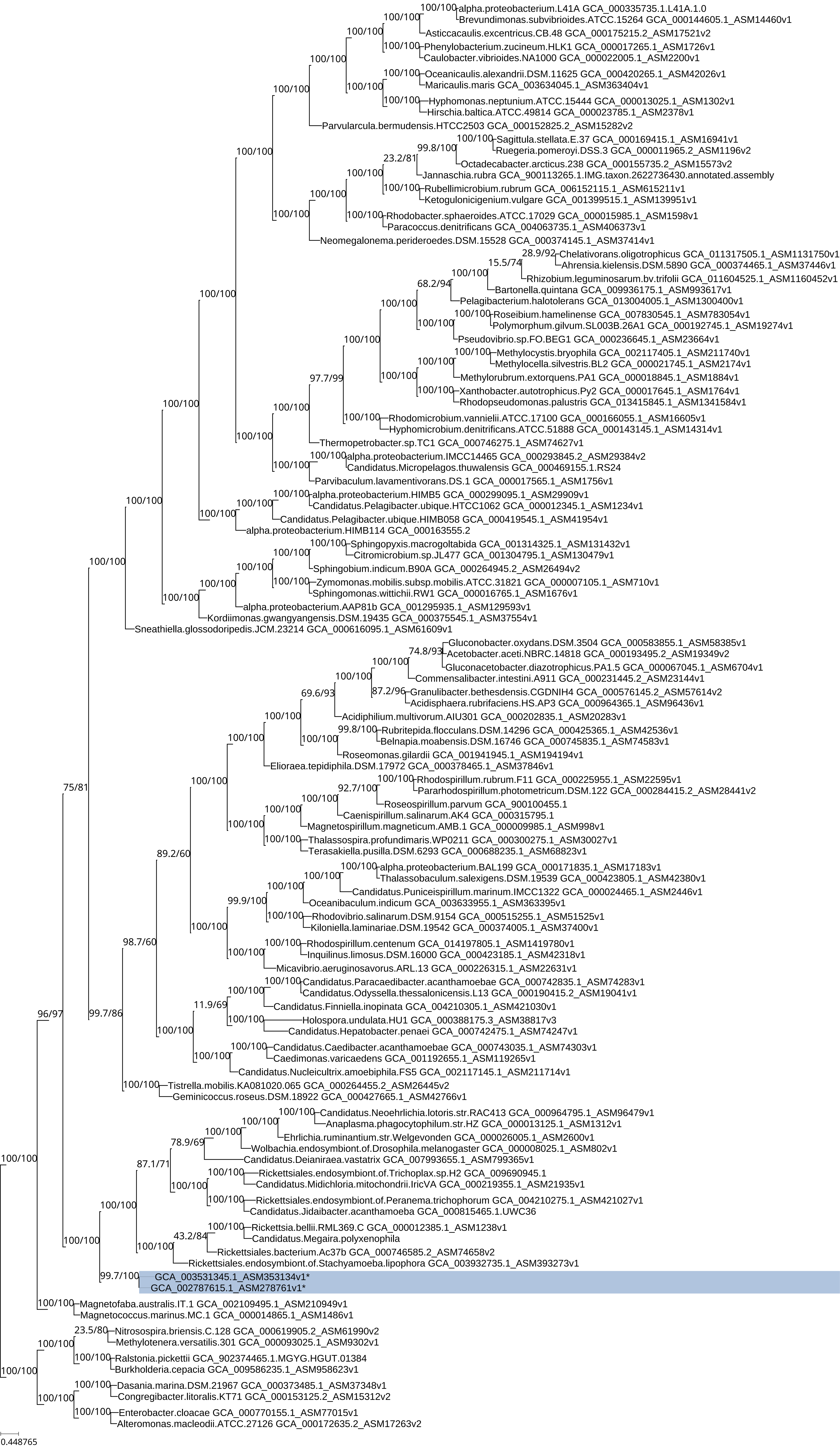
alpha.proteobacterium.L41A GCA_000335735.1_L41A.1.0
Brevundimonas.subvibrioides.ATCC.15264 GCA_000144605.1_ASM14460v1
Asticcacaulis.excentricus.CB.48 GCA_000175215.2_ASM17521v2
Phenylobacterium.zucineum.HLK1 GCA_000017265.1_ASM1726v1
Caulobacter.vibrioides.NA1000 GCA_000022005.1_ASM2200v1
Oceanicaulis.alexandrii.DSM.11625 GCA_000420265.1_ASM42026v1
Maricaulis.maris GCA_003634045.1_ASM363404v1
Hyphomonas.neptunium.ATCC.15444 GCA_000013025.1_ASM1302v1
Hirschia.baltica.ATCC.49814 GCA_000023785.1_ASM2378v1
Parvularcula.bermudensis.HTCC2503 GCA_000152825.2_ASM15282v2
Sagittula.stellata.E.37 GCA_000169415.1_ASM16941v1
Ruegeria.pomeroyi.DSS.3 GCA_000011965.2_ASM1196v2
Octadecabacter.arcticus.238 GCA_000155735.2_ASM15573v2
Jannaschia.rubra GCA_900113265.1.IMG.taxon.2622736430.annotated.assembly
Rubellimicrobium.rubrum GCA_006152115.1_ASM615211v1
Ketogulonicigenium.vulgare GCA_001399515.1_ASM139951v1
Rhodobacter.sphaeroides.ATCC.17029 GCA_000015985.1_ASM1598v1
Paracoccus.denitrificans GCA_004063735.1_ASM406373v1
Neomegalonema.perideroedes.DSM.15528 GCA_000374145.1_ASM37414v1
Chelativorans.oligotrophicus GCA_011317505.1_ASM1131750v1
Ahrensia.kielensis.DSM.5890 GCA_000374465.1_ASM37446v1
Rhizobium.leguminosarum.bv.trifolii GCA_011604525.1_ASM1160452v1
Bartonella.quintana GCA_009936175.1_ASM993617v1
Pelagibacterium.halotolerans GCA_013004005.1_ASM1300400v1
Roseibium.hamelinense GCA_007830545.1_ASM783054v1
Polymorphum.gilvum.SL003B.26A1 GCA_000192745.1_ASM19274v1
Pseudovibrio.sp.FO.BEG1 GCA_000236645.1_ASM23664v1
Methylocystis.bryophila GCA_002117405.1_ASM211740v1
Methylocella.silvestris.BL2 GCA_000021745.1_ASM2174v1
Methylorubrum.extorquens.PA1 GCA_000018845.1_ASM1884v1
Xanthobacter.autotrophicus.Py2 GCA_000017645.1_ASM1764v1
Rhodopseudomonas.palustris GCA_013415845.1_ASM1341584v1
Rhodomicrobium.vannielii.ATCC.17100 GCA_000166055.1_ASM16605v1
Hyphomicrobium.denitrificans.ATCC.51888 GCA_000143145.1_ASM14314v1
Thermopetrobacter.sp.TC1 GCA_000746275.1_ASM74627v1
alpha.proteobacterium.IMCC14465 GCA_000293845.2_ASM29384v2
Candidatus.Micropelagos.thuwalensis GCA_000469155.1.RS24
Parvibaculum.lavamentivorans.DS.1 GCA_000017565.1_ASM1756v1
alpha.proteobacterium.HIMB5 GCA_000299095.1_ASM29909v1
Candidatus.Pelagibacter.ubique.HTCC1062 GCA_000012345.1_ASM1234v1
Candidatus.Pelagibacter.ubique.HIMB058 GCA_000419545.1_ASM41954v1
alpha.proteobacterium.HIMB114 GCA_000163555.2
Sphingopyxis.macrogoltabida GCA_001314325.1_ASM131432v1
Citromicrobium.sp.JL477 GCA_001304795.1_ASM130479v1
Sphingobium.indicum.B90A GCA_000264945.2_ASM26494v2
Zymomonas.mobilis.subsp.mobilis.ATCC.31821 GCA_000007105.1_ASM710v1
Sphingomonas.wittichii.RW1 GCA_000016765.1_ASM1676v1
alpha.proteobacterium.AAP81b GCA_001295935.1_ASM129593v1
Kordiimonas.gwangyangensis.DSM.19435 GCA_000375545.1_ASM37554v1
Sneathiella.glossodoripedis.JCM.23214 GCA_000616095.1_ASM61609v1
Gluconobacter.oxydans.DSM.3504 GCA_000583855.1_ASM58385v1
Acetobacter.aceti.NBRC.14818 GCA_001193495.2_ASM19349v2
Gluconacetobacter.diazotrophicus.PA1.5 GCA_000067045.1_ASM6704v1
Commensalibacter.intestini.A911 GCA_000231445.2_ASM23144v1
Granulibacter.bethesdensis.CGDNIH4 GCA_000576145.2_ASM57614v2
Acidisphaera.rubrifaciens.HS.AP3 GCA_000964365.1_ASM96436v1
Acidiphilium.multivorum.AIU301 GCA_000202835.1_ASM20283v1
Rubritepida.flocculans.DSM.14296 GCA_000425365.1_ASM42536v1
Belnapia.moabensis.DSM.16746 GCA_000745835.1_ASM74583v1
Roseomonas.gilardii GCA_001941945.1_ASM194194v1
Elioraea.tepidiphila.DSM.17972 GCA_000378465.1_ASM37846v1
Rhodospirillum.rubrum.F11 GCA_000225955.1_ASM22595v1
Pararhodospirillum.photometricum.DSM.122 GCA_000284415.2_ASM28441v2
Roseospirillum.parvum GCA_900100455.1
Caenispirillum.salinarum.AK4 GCA_000315795.1
Magnetospirillum.magneticum.AMB.1 GCA_000009985.1_ASM998v1
Thalassospira.profundimaris.WP0211 GCA_000300275.1_ASM30027v1
Terasakiella.pusilla.DSM.6293 GCA_000688235.1_ASM68823v1
alpha.proteobacterium.BAL199 GCA_000171835.1_ASM17183v1
Thalassobaculum.salexigens.DSM.19539 GCA_000423805.1_ASM42380v1
Candidatus.Puniceispirillum.marinum.IMCC1322 GCA_000024465.1_ASM2446v1
Oceanibaculum.indicum GCA_003633955.1_ASM363395v1
Rhodovibrio.salinarum.DSM.9154 GCA_000515255.1_ASM51525v1
Kiloniella.laminariae.DSM.19542 GCA_000374005.1_ASM37400v1
Rhodospirillum.centenum GCA_014197805.1_ASM1419780v1
Inquilinus.limosus.DSM.16000 GCA_000423185.1_ASM42318v1
Micavibrio.aeruginosavorus.ARL.13 GCA_000226315.1_ASM22631v1
Candidatus.Paracaedibacter.acanthamoebae GCA_000742835.1_ASM74283v1
Candidatus.Odyssella.thessalonicensis.L13 GCA_000190415.2_ASM19041v1
Candidatus.Finniella.inopinata GCA_004210305.1_ASM421030v1
Holospora.undulata.HU1 GCA_000388175.3_ASM38817v3
Candidatus.Hepatobacter.penaei GCA_000742475.1_ASM74247v1
Candidatus.Caedibacter.acanthamoebae GCA_000743035.1_ASM74303v1
Caedimonas.varicaedens GCA_001192655.1_ASM119265v1
Candidatus.Nucleicultrix.amoebiphila.FS5 GCA_002117145.1_ASM211714v1
Tistrella.mobilis.KA081020.065 GCA_000264455.2_ASM26445v2
Geminicoccus.roseus.DSM.18922 GCA_000427665.1_ASM42766v1
Candidatus.Neoehrlichia.lotoris.str.RAC413 GCA_000964795.1_ASM96479v1
Anaplasma.phagocytophilum.str.HZ GCA_000013125.1_ASM1312v1
Ehrlichia.ruminantium.str.Welgevonden GCA_000026005.1_ASM2600v1
Wolbachia.endosymbiont.of.Drosophila.melanogaster GCA_000008025.1_ASM802v1
Candidatus.Deianiraea.vastatrix GCA_007993655.1_ASM799365v1
Rickettsiales.endosymbiont.of.Trichoplax.sp.H2 GCA_009690945.1
Candidatus.Midichloria.mitochondrii.IricVA GCA_000219355.1_ASM21935v1
Rickettsiales.endosymbiont.of.Peranema.trichophorum GCA_004210275.1_ASM421027v1
Candidatus.Jidaibacter.acanthamoeba GCA_000815465.1.UWC36
Rickettsia.bellii.RML369.C GCA_000012385.1_ASM1238v1
Candidatus.Megaira.polyxenophila
Rickettsiales.bacterium.Ac37b GCA_000746585.2_ASM74658v2
Rickettsiales.endosymbiont.of.Stachyamoeba.lipophora GCA_003932735.1_ASM393273v1
GCA_003531345.1_ASM353134v1*
GCA_002787615.1_ASM278761v1*
Magnetofaba.australis.IT.1 GCA_002109495.1_ASM210949v1
Magnetococcus.marinus.MC.1 GCA_000014865.1_ASM1486v1
Nitrosospira.briensis.C.128 GCA_000619905.2_ASM61990v2
Methylotenera.versatilis.301 GCA_000093025.1_ASM9302v1
Ralstonia.pickettii GCA_902374465.1.MGYG.HGUT.01384
Burkholderia.cepacia GCA_009586235.1_ASM958623v1
Dasania.marina.DSM.21967 GCA_000373485.1_ASM37348v1
Congregibacter.litoralis.KT71 GCA_000153125.2_ASM15312v2
Enterobacter.cloacae GCA_000770155.1_ASM77015v1
Alteromonas.macleodii.ATCC.27126 GCA_000172635.2_ASM17263v2
0.448765

Group H

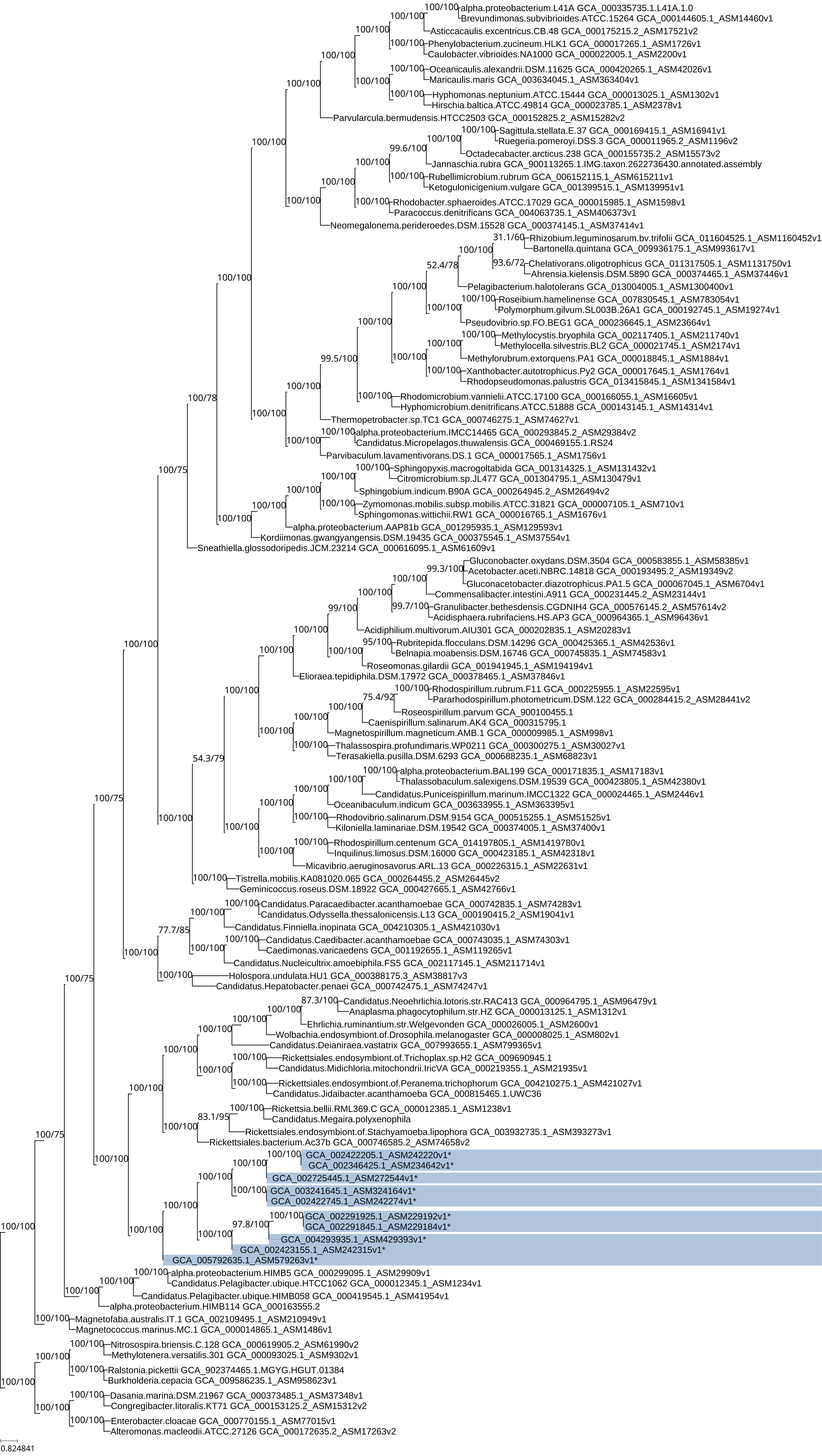
100/100
alpha.proteobacterium.L41A GCA_000335735.1_L41A.1.0
Brevundimonas.subvibrioides.ATCC.15264 GCA_000144605.1_ASM14460v1
100/100
Asticcacaulis.excentricus.CB.48 GCA_000175215.2_ASM17521v2
100/100
Phenylobacterium.zucineum.HLK1 GCA_000017265.1_ASM1726v1
Caulobacter.vibrioides.NA1000 GCA_000022005.1_ASM2200v1
100/100
100/100
Oceanicaulis.alexandrii.DSM.11625 GCA_000420265.1_ASM42026v1
Maricaulis.maris GCA_003634045.1_ASM363404v1
100/100
100/100
Hyphomonas.neptunium.ATCC.15444 GCA_000013025.1_ASM1302v1
Hirschia.baltica.ATCC.49814 GCA_000023785.1_ASM2378v1
100/100
Parvularcula.bermudensis.HTCC2503 GCA_000152825.2_ASM15282v2
100/100
100/100
Sagittula.stellata.E.37 GCA_000169415.1_ASM16941v1
Ruegeria.pomeroyi.DSS.3 GCA_000011965.2_ASM1196v2
100/100
99.6/100
Octadecabacter.arcticus.238 GCA_000155735.2_ASM15573v2
Jannaschia.rubra GCA_900113265.1.IMG.taxon.2622736430.annotated.assembly
100/100
100/100
Rubellimicrobium.rubrum GCA_006152115.1_ASM615211v1
Ketogulonicigenium.vulgare GCA_001399515.1_ASM139951v1
100/100
100/100
Rhodobacter.sphaeroides.ATCC.17029 GCA_000015985.1_ASM1598v1
100/100
Paracoccus.denitrificans GCA_004063735.1_ASM406373v1
Neomegalonema.perideroedes.DSM.15528 GCA_000374145.1_ASM37414v1
31.1/60
Rhizobium.leguminosarum.bv.trifolii GCA_011604525.1_ASM1160452v1
100/100
Bartonella.quintana GCA_009936175.1_ASM993617v1
93.6/72
Chelativorans.oligotrophicus GCA_011317505.1_ASM1131750v1
52.4/78
Ahrensia.kielensis.DSM.5890 GCA_000374465.1_ASM37446v1
100/100
Pelagibacterium.halotolerans GCA_013004005.1_ASM1300400v1
100/100
Roseibium.hamelinense GCA_007830545.1_ASM783054v1
100/100
Polymorphum.gilvum.SL003B.26A1 GCA_000192745.1_ASM19274v1
100/100
Pseudovibrio.sp.FO.BEG1 GCA_000236645.1_ASM23664v1
100/100
Methylocystis.bryophila GCA_002117405.1_ASM211740v1
100/100
Methylocella.silvestris.BL2 GCA_000021745.1_ASM2174v1
99.5/100
100/100
Methylorubrum.extorquens.PA1 GCA_000018845.1_ASM1884v1
100/100
Xanthobacter.autotrophicus.Py2 GCA_000017645.1_ASM1764v1
100/78
Rhodopseudomonas.palustris GCA_013415845.1_ASM1341584v1
100/100
100/100
Rhodomicrobium.vannielii.ATCC.17100 GCA_000166055.1_ASM16605v1
Hyphomicrobium.denitrificans.ATCC.51888 GCA_000143145.1_ASM14314v1
100/100
Thermopetrobacter.sp.TC1 GCA_000746275.1_ASM74627v1
100/100
alpha.proteobacterium.IMCC14465 GCA_000293845.2_ASM29384v2
100/100
Candidatus.Micropelagos.thuwalensis GCA_000469155.1.RS24
Parvibaculum.lavamentivorans.DS.1 GCA_000017565.1_ASM1756v1
100/75
100/100
Sphingopyxis.macrogoltabida GCA_001314325.1_ASM131432v1
100/100
Citromicrobium.sp.JL477 GCA_001304795.1_ASM130479v1
100/100
Sphingobium.indicum.B90A GCA_000264945.2_ASM26494v2
100/100
100/100
Zymomonas.mobilis.subsp.mobilis.ATCC.31821 GCA_000007105.1_ASM710v1
100/100
Sphingomonas.wittichii.RW1 GCA_000016765.1_ASM1676v1
alpha.proteobacterium.AAP81b GCA_001295935.1_ASM129593v1
Kordiimonas.gwangyangensis.DSM.19435 GCA_000375545.1_ASM37554v1
Sneathiella.glossodoripedis.JCM.23214 GCA_000616095.1_ASM61609v1
Gluconobacter.oxydans.DSM.3504 GCA_000583855.1_ASM58385v1
99.3/100
Acetobacter.aceti.NBRC.14818 GCA_000193495.2_ASM19349v2
100/100
Gluconacetobacter.diazotrophicus.PA1.5 GCA_000067045.1_ASM6704v1
100/100
Commensalibacter.intestini.A911 GCA_000231445.2_ASM23144v1
99/100
99.7/100
Granulibacter.bethesdensis.CGDNIH4 GCA_000576145.2_ASM57614v2
Acidisphaera.rubrifaciens.HS.AP3 GCA_000964365.1_ASM96436v1
100/100
Acidiphilium.multivorum.AIU301 GCA_000202835.1_ASM20283v1
100/100
95/100
Rubritepida.flocculans.DSM.14296 GCA_000425365.1_ASM42536v1
100/100
100/100
Belnapia.moabensis.DSM.16746 GCA_000745835.1_ASM74583v1
Roseomonas.gilardii GCA_001941945.1_ASM194194v1
Elioraea.tepidiphila.DSM.17972 GCA_000378465.1_ASM37846v1
100/100
100/100
Rhodospirillum.rubrum.F11 GCA_000225955.1_ASM22595v1
75.4/92
Pararhodospirillum.photometricum.DSM.122 GCA_000284415.2_ASM28441v2
100/100
Roseospirillum.parvum GCA_900100455.1
100/100
Caenispirillum.salinarum.AK4 GCA_000315795.1
100/100
Magnetospirillum.magneticum.AMB.1 GCA_000009985.1_ASM998v1
100/100
Thalassospira.profundimaris.WP0211 GCA_000300275.1_ASM30027v1
Terasakiella.pusilla.DSM.6293 GCA_000688235.1_ASM68823v1
54.3/79
100/100
alpha.proteobacterium.BAL199 GCA_000171835.1_ASM17183v1
100/100
Thalassobaculum.salexigens.DSM.19539 GCA_000423805.1_ASM42380v1
100/100
Candidatus.Puniceispirillum.marinum.IMCC1322 GCA_000024465.1_ASM2446v1
100/75
100/100
Oceanibaculum.indicum GCA_003633955.1_ASM363395v1
100/100
Rhodovibrio.salinarum.DSM.9154 GCA_000515255.1_ASM51525v1
100/100
100/100
Kiloniella.laminariae.DSM.19542 GCA_000374005.1_ASM37400v1
100/100
Rhodospirillum.centenum GCA_014197805.1_ASM1419780v1
100/100
Inquilinus.limosus.DSM.16000 GCA_000423185.1_ASM42318v1
Micavibrio.aeruginosavorus.ARL.13 GCA_000226315.1_ASM22631v1
100/100
Tistrella.mobilis.KA081020.065 GCA_000264455.2_ASM26445v2
Geminicoccus.roseus.DSM.18922 GCA_000427665.1_ASM42766v1
100/100
Candidatus.Paracaedibacter.acanthamoebae GCA_000742835.1_ASM74283v1
100/100
Candidatus.Odyssella.thessalonicensis.L13 GCA_000190415.2_ASM19041v1
77.7/85
Candidatus.Finniella.inopinata GCA_004210305.1_ASM421030v1
100/100
Candidatus.Caedibacter.acanthamoebae GCA_000743035.1_ASM74303v1
100/100
Caedimonas.varicaedens GCA_001192655.1_ASM119265v1
100/100
Candidatus.Nucleicultrix.amoebiphila.FS5 GCA_002117145.1_ASM211714v1
100/100
Holospora.undulata.HU1 GCA_000388175.3_ASM38817v3
100/75
Candidatus.Hepatobacter.penaei GCA_000742475.1_ASM74247v1
87.3/100
Candidatus.Neoehrlichia.lotoris.str.RAC413 GCA_000964795.1_ASM96479v1
100/100
Anaplasma.phagocytophilum.str.HZ GCA_000013125.1_ASM1312v1
100/100
Ehrlichia.ruminantium.str.Welgevonden GCA_000026005.1_ASM2600v1
100/100
Wolbachia.endosymbiont.of.Drosophila.melanogaster GCA_000008025.1_ASM802v1
Candidatus.Deianiraea.vastatrix GCA_007993655.1_ASM799365v1
100/100
100/100
Rickettsiales.endosymbiont.of.Trichoplax.sp.H2 GCA_009690945.1
100/100
Candidatus.Midichloria.mitochondrii.IricVA GCA_000219355.1_ASM21935v1
100/100
100/100
Rickettsiales.endosymbiont.of.Peranema.trichophorum GCA_004210275.1_ASM421027v1
Candidatus.Jidaibacter.acanthamoeba GCA_000815465.1.UWC36
100/100
Rickettsia.bellii.RML369.C GCA_000012385.1_ASM1238v1
83.1/95
Candidatus.Megaira.polyxenophila
100/100
Rickettsiales.endosymbiont.of.Stachyamoeba.lipophora GCA_003932735.1_ASM393273v1
100/75
Rickettsiales.bacterium.Ac37b GCA_000746585.2_ASM74658v2
100/100
100/100
GCA_002422205.1_ASM242220v1*
100/100
GCA_002346425.1_ASM234642v1*
100/100
GCA_002725445.1_ASM272544v1*
100/100
GCA_003241645.1_ASM324164v1*
GCA_002422745.1_ASM242274v1*
100/100
100/100
GCA_002291925.1_ASM229192v1*
100/100
97.8/100
GCA_002291845.1_ASM229184v1*
100/100
GCA_004293935.1_ASM429393v1*
GCA_002423155.1_ASM242315v1*
GCA_005792635.1_ASM579263v1*
100/100
100/100
alpha.proteobacterium.HIMB5 GCA_000299095.1_ASM29909v1
100/100
Candidatus.Pelagibacter.ubique.HTCC1062 GCA_000012345.1_ASM1234v1
100/100
Candidatus.Pelagibacter.ubique.HIMB058 GCA_000419545.1_ASM41954v1
alpha.proteobacterium.HIMB114 GCA_000163555.2
100/100
Magnetofaba.australis.IT.1 GCA_002109495.1_ASM210949v1
Magnetococcus.marinus.MC.1 GCA_000014865.1_ASM1486v1
100/100
Nitrosospira.briensis.C.128 GCA_000619905.2_ASM61990v2
100/100
Methylotenera.versatilis.301 GCA_000093025.1_ASM9302v1
100/100
Ralstonia.pickettii GCA_902374465.1.MGYG.HGUT.01384
Burkholderia.cepacia GCA_009586235.1_ASM958623v1
100/100
100/100
Dasania.marina.DSM.21967 GCA_000373485.1_ASM37348v1
100/100
Congregibacter.litoralis.KT71 GCA_000153125.2_ASM15312v2
100/100
Enterobacter.cloacae GCA_000770155.1_ASM77015v1
Alteromonas.macleodii.ATCC.27126 GCA_000172635.2_ASM17263v2
0.824841

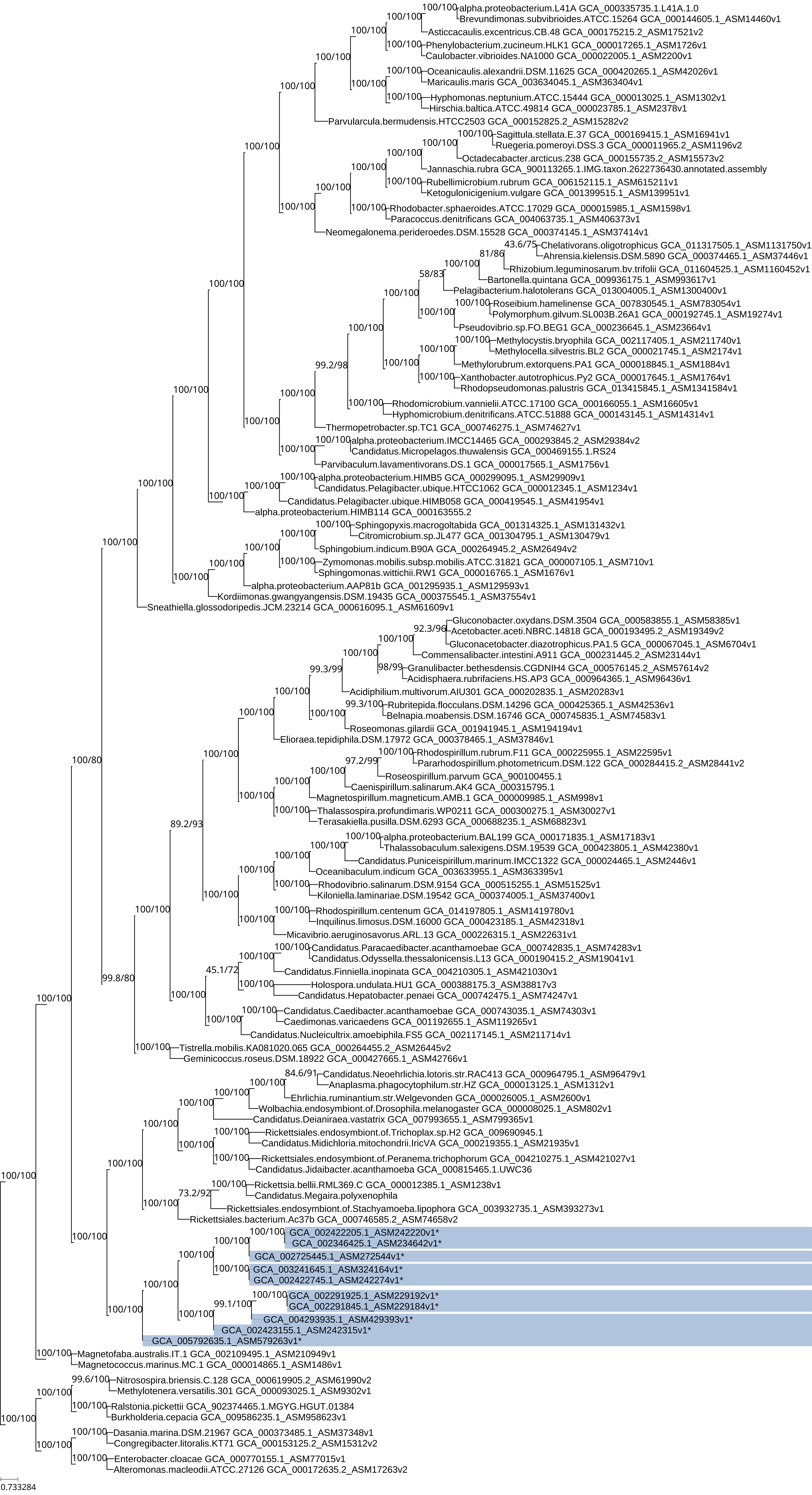
100/100
alpha.proteobacterium.L41A GCA_000335735.1_L41A.1.0
Brevundimonas.subvibrioides.ATCC.15264 GCA_000144605.1_ASM14460v1
100/100
Asticcacaulis.excentricus.CB.48 GCA_000175215.2_ASM17521v2
100/100
Phenylobacterium.zucineum.HLK1 GCA_000017265.1_ASM1726v1
100/100
Caulobacter.vibrioides.NA1000 GCA_000022005.1_ASM2200v1
100/100
Oceanicaulis.alexandrii.DSM.11625 GCA_000420265.1_ASM42026v1
Maricaulis.maris GCA_003634045.1_ASM363404v1
100/100
100/100
Hyphomonas.neptunium.ATCC.15444 GCA_000013025.1_ASM1302v1
Hirschia.baltica.ATCC.49814 GCA_000023785.1_ASM2378v1
Parvularcula.bermudensis.HTCC2503 GCA_000152825.2_ASM15282v2
100/100
Sagittula.stellata.E.37 GCA_000169415.1_ASM16941v1
100/100
Ruegeria.pomeroyi.DSS.3 GCA_000011965.2_ASM1196v2
100/100
Octadecabacter.arcticus.238 GCA_000155735.2_ASM15573v2
100/100
Jannaschia.rubra GCA_900113265.1.IMG.taxon.2622736430.annotated.assembly
100/100
Rubellimicrobium.rubrum GCA_006152115.1_ASM615211v1
100/100
Ketogulonicigenium.vulgare GCA_001399515.1_ASM139951v1
100/100
100/100
Rhodobacter.sphaeroides.ATCC.17029 GCA_000015985.1_ASM1598v1
100/100
Paracoccus.denitrificans GCA_004063735.1_ASM406373v1
Neomegalonema.perideroedes.DSM.15528 GCA_000374145.1_ASM37414v1
43.6/75
Chelativorans.oligotrophicus GCA_011317505.1_ASM1131750v1
81/86
Ahrensia.kielensis.DSM.5890 GCA_000374465.1_ASM37446v1
100/100
Rhizobium.leguminosarum.bv.trifolii GCA_011604525.1_ASM1160452v1
Bartonella.quintana GCA_009936175.1_ASM993617v1
58/83
100/100
Pelagibacterium.halotolerans GCA_013004005.1_ASM1300400v1
100/100
Roseibium.hamelinense GCA_007830545.1_ASM783054v1
100/100
Polymorphum.gilvum.SL003B.26A1 GCA_000192745.1_ASM19274v1
Pseudovibrio.sp.FO.BEG1 GCA_000236645.1_ASM23664v1
100/100
100/100
Methylocystis.bryophila GCA_002117405.1_ASM211740v1
100/100
Methylocella.silvestris.BL2 GCA_000021745.1_ASM2174v1
100/100
Methylorubrum.extorquens.PA1 GCA_000018845.1_ASM1884v1
99.2/98
100/100
Xanthobacter.autotrophicus.Py2 GCA_000017645.1_ASM1764v1
Rhodopseudomonas.palustris GCA_013415845.1_ASM1341584v1
100/100
100/100
Rhodomicrobium.vannielii.ATCC.17100 GCA_000166055.1_ASM16605v1
Hyphomicrobium.denitrificans.ATCC.51888 GCA_000143145.1_ASM14314v1
100/100
Thermopetrobacter.sp.TC1 GCA_000746275.1_ASM74627v1
100/100
alpha.proteobacterium.IMCC14465 GCA_000293845.2_ASM29384v2
100/100
Candidatus.Micropelagos.thuwalensis GCA_000469155.1.RS24
Parvibaculum.lavamentivorans.DS.1 GCA_000017565.1_ASM1756v1
100/100
100/100
alpha.proteobacterium.HIMB5 GCA_000299095.1_ASM29909v1
100/100
Candidatus.Pelagibacter.ubique.HTCC1062 GCA_000012345.1_ASM1234v1
100/100
Candidatus.Pelagibacter.ubique.HIMB058 GCA_000419545.1_ASM41954v1
alpha.proteobacterium.HIMB114 GCA_000163555.2
100/100
100/100
Sphingopyxis.macrogoltabida GCA_001314325.1_ASM131432v1
100/100
Citromicrobium.sp.JL477 GCA_001304795.1_ASM130479v1
100/100
Sphingobium.indicum.B90A GCA_000264945.2_ASM26494v2
100/100
Zymomonas.mobilis.subsp.mobilis.ATCC.31821 GCA_000007105.1_ASM710v1
100/100
Sphingomonas.wittichii.RW1 GCA_000016765.1_ASM1676v1
100/100
alpha.proteobacterium.AAP81b GCA_001295935.1_ASM129593v1
Kordiimonas.gwangyangensis.DSM.19435 GCA_000375545.1_ASM37554v1
Sneathiella.glossodoripedis.JCM.23214 GCA_000616095.1_ASM61609v1
Gluconobacter.oxydans.DSM.3504 GCA_000583855.1_ASM58385v1
92.3/96
Acetobacter.aceti.NBRC.14818 GCA_000193495.2_ASM19349v2
100/100
Gluconacetobacter.diazotrophicus.PA1.5 GCA_000067045.1_ASM6704v1
100/100
Commensalibacter.intestini.A911 GCA_000231445.2_ASM23144v1
98/99
Granulibacter.bethesdensis.CGDNIH4 GCA_000576145.2_ASM57614v2
99.3/99
Acidisphaera.rubrifaciens.HS.AP3 GCA_000964365.1_ASM96436v1
100/100
Acidiphilium.multivorum.AIU301 GCA_000202835.1_ASM20283v1
99.3/100
Rubritepida.flocculans.DSM.14296 GCA_000425365.1_ASM42536v1
100/100
100/100
Belnapia.moabensis.DSM.16746 GCA_000745835.1_ASM74583v1
Roseomonas.gilardii GCA_001941945.1_ASM194194v1
Elioraea.tepidiphila.DSM.17972 GCA_000378465.1_ASM37846v1
100/100
100/100
Rhodospirillum.rubrum.F11 GCA_000225955.1_ASM22595v1
97.2/99
Pararhodospirillum.photometricum.DSM.122 GCA_000284415.2_ASM28441v2
100/100
Roseospirillum.parvum GCA_900100455.1
100/100
Caenispirillum.salinarum.AK4 GCA_000315795.1
100/100
Magnetospirillum.magneticum.AMB.1 GCA_000009985.1_ASM998v1
100/100
Thalassospira.profundimaris.WP0211 GCA_000300275.1_ASM30027v1
Terasakiella.pusilla.DSM.6293 GCA_000688235.1_ASM68823v1
89.2/93
100/100
alpha.proteobacterium.BAL199 GCA_000171835.1_ASM17183v1
100/100
Thalassobaculum.salexigens.DSM.19539 GCA_000423805.1_ASM42380v1
100/100
Candidatus.Puniceispirillum.marinum.IMCC1322 GCA_000024465.1_ASM2446v1
100/100
Oceanibaculum.indicum GCA_003633955.1_ASM363395v1
100/100
Rhodovibrio.salinarum.DSM.9154 GCA_000515255.1_ASM51525v1
100/100
Kiloniella.laminariae.DSM.19542 GCA_000374005.1_ASM37400v1
100/100
Rhodospirillum.centenum GCA_014197805.1_ASM1419780v1
100/100
Inquilinus.limosus.DSM.16000 GCA_000423185.1_ASM42318v1
100/100
Micavibrio.aeruginosavorus.ARL.13 GCA_000226315.1_ASM22631v1
100/100
Candidatus.Paracaedibacter.acanthamoebae GCA_000742835.1_ASM74283v1
100/100
Candidatus.Odyssella.thessalonicensis.L13 GCA_000190415.2_ASM19041v1
45.1/72
Candidatus.Finniella.inopinata GCA_004210305.1_ASM421030v1
100/100
Holospora.undulata.HU1 GCA_000388175.3_ASM38817v3
100/100
Candidatus.Hepatobacter.penaei GCA_000742475.1_ASM74247v1
100/100
Candidatus.Caedibacter.acanthamoebae GCA_000743035.1_ASM74303v1
100/100
Caedimonas.varicaedens GCA_001192655.1_ASM119265v1
Candidatus.Nucleicultrix.amoebiphila.FS5 GCA_002117145.1_ASM211714v1
100/100
Tistrella.mobilis.KA081020.065 GCA_000264455.2_ASM26445v2
Geminicoccus.roseus.DSM.18922 GCA_000427665.1_ASM42766v1
100/80
99.8/80
100/100
84.6/91
Candidatus.Neoehrlichia.lotoris.str.RAC413 GCA_000964795.1_ASM96479v1
100/100
Anaplasma.phagocytophilum.str.HZ GCA_000013125.1_ASM1312v1
100/100
Ehrlichia.ruminantium.str.Welgevonden GCA_000026005.1_ASM2600v1
100/100
Wolbachia.endosymbiont.of.Drosophila.melanogaster GCA_000008025.1_ASM802v1
Candidatus.Deianiraea.vastatrix GCA_007993655.1_ASM799365v1
100/100
100/100
Rickettsiales.endosymbiont.of.Trichoplax.sp.H2 GCA_009690945.1
100/100
Candidatus.Midichloria.mitochondrii.IricVA GCA_000219355.1_ASM21935v1
100/100
Rickettsiales.endosymbiont.of.Peranema.trichophorum GCA_004210275.1_ASM421027v1
Candidatus.Jidaibacter.acanthamoeba GCA_000815465.1.UWC36
100/100
100/100
Rickettsia.bellii.RML369.C GCA_000012385.1_ASM1238v1
73.2/92
Candidatus.Megaira.polyxenophila
100/100
Rickettsiales.endosymbiont.of.Stachyamoeba.lipophora GCA_003932735.1_ASM393273v1
Rickettsiales.bacterium.Ac37b GCA_000746585.2_ASM74658v2
100/100
100/100
GCA_002422205.1_ASM242220v1*
100/100
GCA_002346425.1_ASM234642v1*
100/100
GCA_002725445.1_ASM272544v1*
100/100
GCA_003241645.1_ASM324164v1*
GCA_002422745.1_ASM242274v1*
100/100
100/100
GCA_002291925.1_ASM229192v1*
99.1/100
GCA_002291845.1_ASM229184v1*
100/100
GCA_004293935.1_ASM429393v1*
GCA_002423155.1_ASM242315v1*
GCA_005792635.1_ASM579263v1*
100/100
Magnetofaba.australis.IT.1 GCA_002109495.1_ASM210949v1
Magnetococcus.marinus.MC.1 GCA_000014865.1_ASM1486v1
100/100
99.6/100
Nitrosospira.briensis.C.128 GCA_000619905.2_ASM61990v2
100/100
Methylotenera.versatilis.301 GCA_000093025.1_ASM9302v1
100/100
Ralstonia.pickettii GCA_902374465.1.MGYG.HGUT.01384
Burkholderia.cepacia GCA_009586235.1_ASM958623v1
100/100
100/100
Dasania.marina.DSM.21967 GCA_000373485.1_ASM37348v1
100/100
Congregibacter.litoralis.KT71 GCA_000153125.2_ASM15312v2
100/100
Enterobacter.cloacae GCA_000770155.1_ASM77015v1
Alteromonas.macleodii.ATCC.27126 GCA_000172635.2_ASM17263v2
0.733284

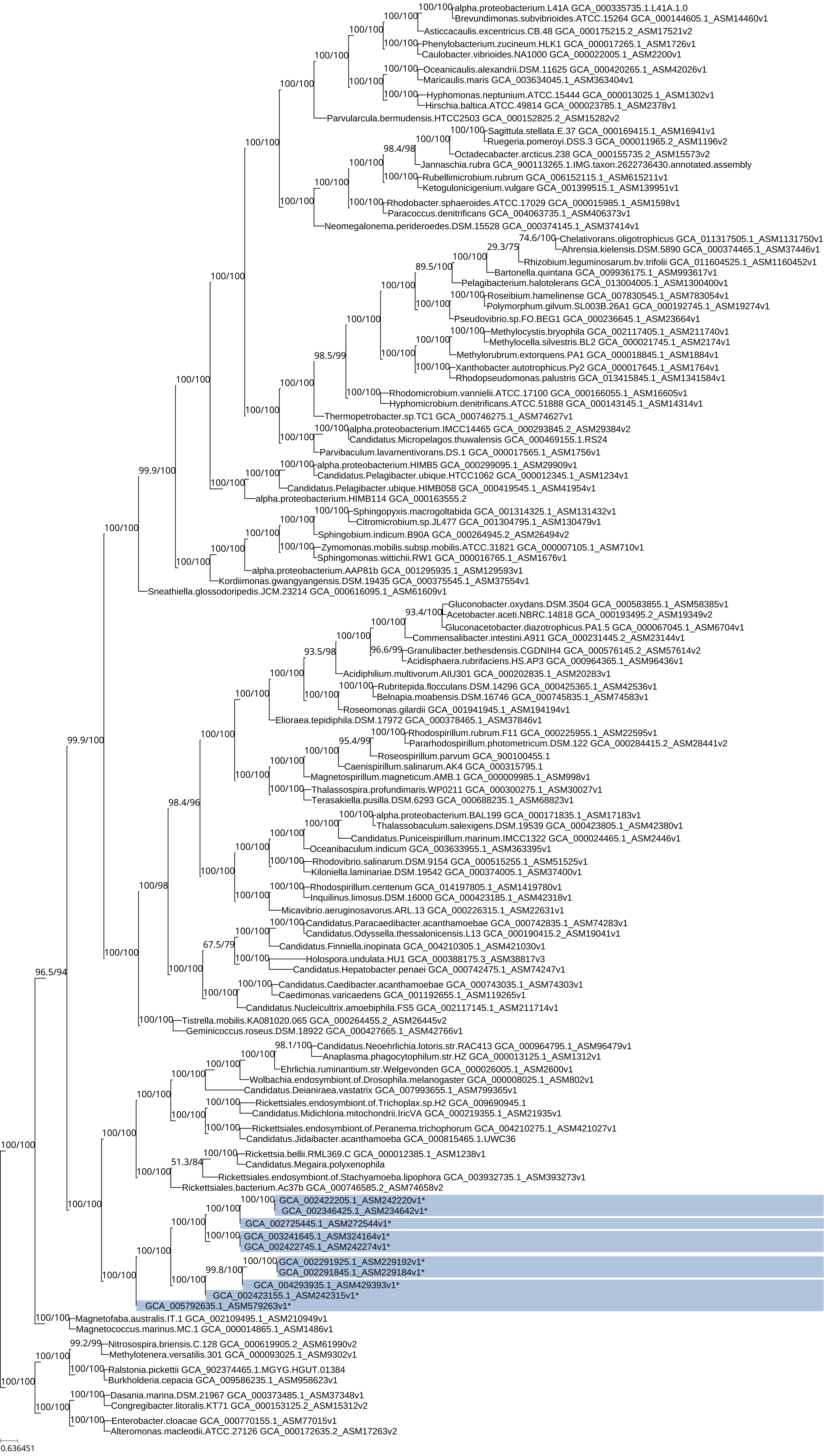
alpha.proteobacterium.L41A GCA_000335735.1_L41A.1.0
Brevundimonas.subvibrioides.ATCC.15264 GCA_000144605.1_ASM14460v1
Asticcacaulis.excentricus.CB.48 GCA_000175215.2_ASM17521v2
Phenylobacterium.zucineum.HLK1 GCA_000017265.1_ASM1726v1
Caulobacter.vibrioides.NA1000 GCA_000022005.1_ASM2200v1
Oceanicaulis.alexandrii.DSM.11625 GCA_000420265.1_ASM42026v1
Maricaulis.maris GCA_003634045.1_ASM363404v1
Hyphomonas.neptunium.ATCC.15444 GCA_000013025.1_ASM1302v1
Hirschia.baltica.ATCC.49814 GCA_000023785.1_ASM2378v1
Parvularcula.bermudensis.HTCC2503 GCA_000152825.2_ASM15282v2
Sagittula.stellata.E.37 GCA_000169415.1_ASM16941v1
Ruegeria.pomeroyi.DSS.3 GCA_000011965.2_ASM1196v2
Octadecabacter.arcticus.238 GCA_000155735.2_ASM15573v2
Jannaschia.rubra GCA_900113265.1.IMG.taxon.2622736430.annotated.assembly
Rubellimicrobium.rubrum GCA_006152115.1_ASM615211v1
Ketogulonicigenium.vulgare GCA_001399515.1_ASM139951v1
Rhodobacter.sphaeroides.ATCC.17029 GCA_000015985.1_ASM1598v1
Paracoccus.denitrificans GCA_004063735.1_ASM406373v1
Neomegalonema.perideroedes.DSM.15528 GCA_000374145.1_ASM37414v1
Chelativorans.oligotrophicus GCA_011317505.1_ASM1131750v1
Ahrensia.kielensis.DSM.5890 GCA_000374465.1_ASM37446v1
Rhizobium.leguminosarum.bv.trifolii GCA_011604525.1_ASM1160452v1
Bartonella.quintana GCA_009936175.1_ASM993617v1
Pelagibacterium.halotolerans GCA_013004005.1_ASM1300400v1
Roseibium.hamelinense GCA_007830545.1_ASM783054v1
Polymorphum.gilvum.SL003B.26A1 GCA_000192745.1_ASM19274v1
Pseudovibrio.sp.FO.BEG1 GCA_000236645.1_ASM23664v1
Methylocystis.bryophila GCA_002117405.1_ASM211740v1
Methylocella.silvestris.BL2 GCA_000021745.1_ASM2174v1
Methylorubrum.extorquens.PA1 GCA_000018845.1_ASM1884v1
Xanthobacter.autotrophicus.Py2 GCA_000017645.1_ASM1764v1
Rhodopseudomonas.palustris GCA_013415845.1_ASM1341584v1
Rhodomicrobium.vannielii.ATCC.17100 GCA_000166055.1_ASM16605v1
Hyphomicrobium.denitrificans.ATCC.51888 GCA_000143145.1_ASM14314v1
Thermopetrobacter.sp.TC1 GCA_000746275.1_ASM74627v1
alpha.proteobacterium.IMCC14465 GCA_000293845.2_ASM29384v2
Candidatus.Micropelagos.thuwalensis GCA_000469155.1.RS24
Parvibaculum.lavamentivorans.DS.1 GCA_000017565.1_ASM1756v1
alpha.proteobacterium.HIMB5 GCA_000299095.1_ASM29909v1
Candidatus.Pelagibacter.ubique.HTCC1062 GCA_000012345.1_ASM1234v1
Candidatus.Pelagibacter.ubique.HIMB058 GCA_000419545.1_ASM41954v1
alpha.proteobacterium.HIMB114 GCA_000163555.2
Sphingopyxis.macrogoltabida GCA_001314325.1_ASM131432v1
Citromicrobium.sp.JL477 GCA_001304795.1_ASM130479v1
Sphingobium.indicum.B90A GCA_000264945.2_ASM26494v2
Zymomonas.mobilis.subsp.mobilis.ATCC.31821 GCA_000007105.1_ASM710v1
Sphingomonas.wittichii.RW1 GCA_000016765.1_ASM1676v1
alpha.proteobacterium.AAP81b GCA_001295935.1_ASM129593v1
Kordiimonas.gwangyangensis.DSM.19435 GCA_000375545.1_ASM37554v1
Sneathiella.glossodoripedis.JCM.23214 GCA_000616095.1_ASM61609v1
Gluconobacter.oxydans.DSM.3504 GCA_000583855.1_ASM58385v1
Acetobacter.aceti.NBRC.14818 GCA_000193495.2_ASM19349v2
Gluconacetobacter.diazotrophicus.PA1.5 GCA_000067045.1_ASM6704v1
Commensalibacter.intestini.A911 GCA_000231445.2_ASM23144v1
Granulibacter.bethesdensis.CGDNIH4 GCA_000576145.2_ASM57614v2
Acidisphaera.rubrifaciens.HS.AP3 GCA_000964365.1_ASM96436v1
Acidiphilium.multivorum.AIU301 GCA_000202835.1_ASM20283v1
Rubritepida.flocculans.DSM.14296 GCA_000425365.1_ASM42536v1
Belnapia.moabensis.DSM.16746 GCA_000745835.1_ASM74583v1
Roseomonas.gilardii GCA_001941945.1_ASM194194v1
Elioraea.tepidiphila.DSM.17972 GCA_000378465.1_ASM37846v1
Rhodospirillum.rubrum.F11 GCA_000225955.1_ASM22595v1
Pararhodospirillum.photometricum.DSM.122 GCA_000284415.2_ASM28441v2
Roseospirillum.parvum GCA_900100455.1
Caenispirillum.salinarum.AK4 GCA_000315795.1
Magnetospirillum.magneticum.AMB.1 GCA_000009985.1_ASM998v1
Thalassospira.profundimaris.WP0211 GCA_000300275.1_ASM30027v1
Terasakiella.pusilla.DSM.6293 GCA_000688235.1_ASM68823v1
alpha.proteobacterium.BAL199 GCA_000171835.1_ASM17183v1
Thalassobaculum.salexigens.DSM.19539 GCA_000423805.1_ASM42380v1
Candidatus.Puniceispirillum.marinum.IMCC1322 GCA_000024465.1_ASM2446v1
Oceanibaculum.indicum GCA_003633955.1_ASM363395v1
Rhodovibrio.salinarum.DSM.9154 GCA_000515255.1_ASM51525v1
Kiloniella.laminariae.DSM.19542 GCA_000374005.1_ASM37400v1
Rhodospirillum.centenum GCA_014197805.1_ASM1419780v1
Inquilinus.limosus.DSM.16000 GCA_000423185.1_ASM42318v1
Micavibrio.aeruginosavorus.ARL.13 GCA_000226315.1_ASM22631v1
Candidatus.Paracaedibacter.acanthamoebae GCA_000742835.1_ASM74283v1
Candidatus.Odyssella.thessalonicensis.L13 GCA_000190415.2_ASM19041v1
Candidatus.Finniella.inopinata GCA_004210305.1_ASM421030v1
Holospora.undulata.HU1 GCA_000388175.3_ASM38817v3
Candidatus.Hepatobacter.penaei GCA_000742475.1_ASM74247v1
Candidatus.Caedibacter.acanthamoebae GCA_000743035.1_ASM74303v1
Caedimonas.varicaedens GCA_001192655.1_ASM119265v1
Candidatus.Nucleicultrix.amoebiphila.FS5 GCA_002117145.1_ASM211714v1
Tistrella.mobilis.KA081020.065 GCA_000264455.2_ASM26445v2
Geminicoccus.roseus.DSM.18922 GCA_000427665.1_ASM42766v1
Candidatus.Neoehrlichia.lotoris.str.RAC413 GCA_000964795.1_ASM96479v1
Anaplasma.phagocytophilum.str.HZ GCA_000013125.1_ASM1312v1
Ehrlichia.ruminantium.str.Welgevonden GCA_000026005.1_ASM2600v1
Wolbachia.endosymbiont.of.Drosophila.melanogaster GCA_000008025.1_ASM802v1
Candidatus.Deianiraea.vastatrix GCA_007993655.1_ASM799365v1
Rickettsiales.endosymbiont.of.Trichoplax.sp.H2 GCA_009690945.1
Candidatus.Midichloria.mitochondrii.IricVA GCA_000219355.1_ASM21935v1
Rickettsiales.endosymbiont.of.Peranema.trichophorum GCA_004210275.1_ASM421027v1
Candidatus.Jidaibacter.acanthamoeba GCA_000815465.1.UWC36
Rickettsia.bellii.RML369.C GCA_000012385.1_ASM1238v1
Candidatus.Megaira.polyxenophila
Rickettsiales.endosymbiont.of.Stachyamoeba.lipophora GCA_003932735.1_ASM393273v1
Rickettsiales.bacterium.Ac37b GCA_000746585.2_ASM74658v2
GCA_002422205.1_ASM242220v1*
GCA_002346425.1_ASM234642v1*
GCA_002725445.1_ASM272544v1*
GCA_003241645.1_ASM324164v1*
GCA_002422745.1_ASM242274v1*
GCA_002291925.1_ASM229192v1*
GCA_002291845.1_ASM229184v1*
GCA_004293935.1_ASM429393v1*
GCA_002423155.1_ASM242315v1*
GCA_005792635.1_ASM579263v1*
Magnetofaba.australis.IT.1 GCA_002109495.1_ASM210949v1
Magnetococcus.marinus.MC.1 GCA_000014865.1_ASM1486v1
Nitrosospira.briensis.C.128 GCA_000619905.2_ASM61990v2
Methylotenera.versatilis.301 GCA_000093025.1_ASM9302v1
Ralstonia.pickettii GCA_902374465.1.MGYG.HGUT.01384
Burkholderia.cepacia GCA_009586235.1_ASM958623v1
Dasania.marina.DSM.21967 GCA_000373485.1_ASM37348v1
Congregibacter.litoralis.KT71 GCA_000153125.2_ASM15312v2
Enterobacter.cloacae GCA_000770155.1_ASM77015v1
Alteromonas.macleodii.ATCC.27126 GCA_000172635.2_ASM17263v2
0.636451

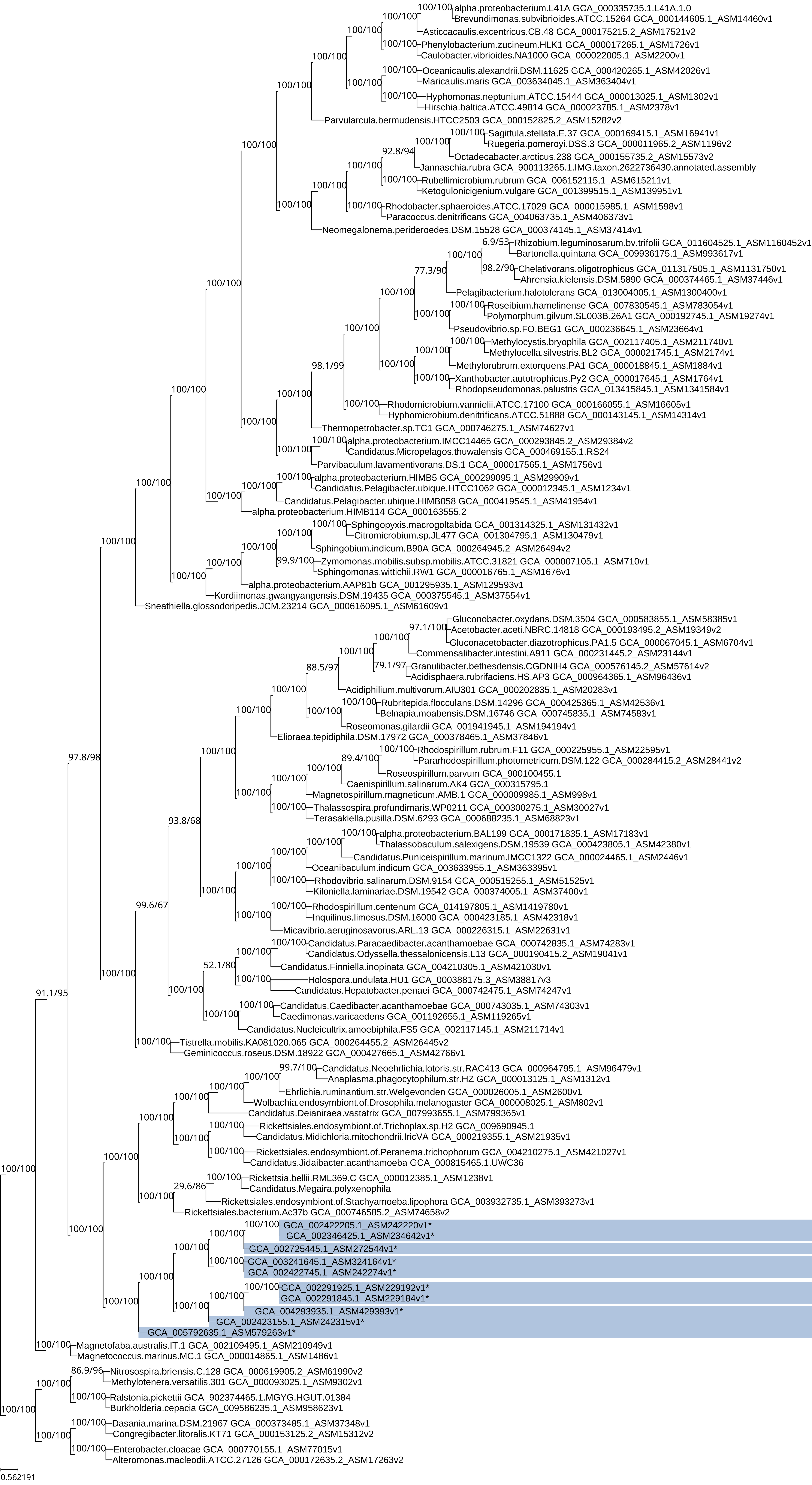
100/100
alpha.proteobacterium.L41A GCA_000335735.1_L41A.1.0
Brevundimonas.subvibrioides.ATCC.15264 GCA_000144605.1_ASM14460v1
100/100
Asticcacaulis.excentricus.CB.48 GCA_000175215.2_ASM17521v2
100/100
100/100
Phenylobacterium.zucineum.HLK1 GCA_000017265.1_ASM1726v1
Caulobacter.vibrioides.NA1000 GCA_000022005.1_ASM2200v1
100/100
100/100
Oceanicaulis.alexandrii.DSM.11625 GCA_000420265.1_ASM42026v1
Maricaulis.maris GCA_003634045.1_ASM363404v1
100/100
100/100
100/100
Hyphomonas.neptunium.ATCC.15444 GCA_000013025.1_ASM1302v1
Hirschia.baltica.ATCC.49814 GCA_000023785.1_ASM2378v1
Parvularcula.bermudensis.HTCC2503 GCA_000152825.2_ASM15282v2
100/100
Sagittula.stellata.E.37 GCA_000169415.1_ASM16941v1
100/100
Ruegeria.pomeroyi.DSS.3 GCA_000011965.2_ASM1196v2
100/100
92.8/94
Octadecabacter.arcticus.238 GCA_000155735.2_ASM15573v2
Jannaschia.rubra GCA_900113265.1.IMG.taxon.2622736430.annotated.assembly
100/100
100/100
Rubellimicrobium.rubrum GCA_006152115.1_ASM615211v1
Ketogulonicigenium.vulgare GCA_001399515.1_ASM139951v1
100/100
100/100
100/100
Rhodobacter.sphaeroides.ATCC.17029 GCA_000015985.1_ASM1598v1
Paracoccus.denitrificans GCA_004063735.1_ASM406373v1
Neomegalonema.perideroedes.DSM.15528 GCA_000374145.1_ASM37414v1
6.9/53
Rhizobium.leguminosarum.bv.trifolii GCA_011604525.1_ASM1160452v1
Bartonella.quintana GCA_009936175.1_ASM993617v1
100/100
98.2/90
Chelativorans.oligotrophicus GCA_011317505.1_ASM1131750v1
Ahrensia.kielensis.DSM.5890 GCA_000374465.1_ASM37446v1
77.3/90
100/100
Pelagibacterium.halotolerans GCA_013004005.1_ASM1300400v1
100/100
Roseibium.hamelinense GCA_007830545.1_ASM783054v1
Polymorphum.gilvum.SL003B.26A1 GCA_000192745.1_ASM19274v1
100/100
Pseudovibrio.sp.FO.BEG1 GCA_000236645.1_ASM23664v1
100/100
100/100
Methylocystis.bryophila GCA_002117405.1_ASM211740v1
Methylocella.silvestris.BL2 GCA_000021745.1_ASM2174v1
100/100
Methylorubrum.extorquens.PA1 GCA_000018845.1_ASM1884v1
98.1/99
100/100
100/100
Xanthobacter.autotrophicus.Py2 GCA_000017645.1_ASM1764v1
Rhodopseudomonas.palustris GCA_013415845.1_ASM1341584v1
100/100
100/100
Rhodomicrobium.vannielii.ATCC.17100 GCA_000166055.1_ASM16605v1
Hyphomicrobium.denitrificans.ATCC.51888 GCA_000143145.1_ASM14314v1
100/100
Thermopetrobacter.sp.TC1 GCA_000746275.1_ASM74627v1
100/100
alpha.proteobacterium.IMCC14465 GCA_000293845.2_ASM29384v2
Candidatus.Micropelagos.thuwalensis GCA_000469155.1.RS24
100/100
Parvibaculum.lavamentivorans.DS.1 GCA_000017565.1_ASM1756v1
100/100
alpha.proteobacterium.HIMB5 GCA_000299095.1_ASM29909v1
Candidatus.Pelagibacter.ubique.HTCC1062 GCA_000012345.1_ASM1234v1
100/100
100/100
Candidatus.Pelagibacter.ubique.HIMB058 GCA_000419545.1_ASM41954v1
alpha.proteobacterium.HIMB114 GCA_000163555.2
100/100
Sphingopyxis.macrogoltabida GCA_001314325.1_ASM131432v1
Citromicrobium.sp.JL477 GCA_001304795.1_ASM130479v1
100/100
Sphingobium.indicum.B90A GCA_000264945.2_ASM26494v2
100/100
99.9/100
Zymomonas.mobilis.subsp.mobilis.ATCC.31821 GCA_000007105.1_ASM710v1
Sphingomonas.wittichii.RW1 GCA_000016765.1_ASM1676v1
100/100
alpha.proteobacterium.AAP81b GCA_001295935.1_ASM129593v1
100/100
Kordiimonas.gwangyangensis.DSM.19435 GCA_000375545.1_ASM37554v1
100/100
Sneathiella.glossodoripedis.JCM.23214 GCA_000616095.1_ASM61609v1
100/100
Gluconobacter.oxydans.DSM.3504 GCA_000583855.1_ASM58385v1
97.1/100
Acetobacter.aceti.NBRC.14818 GCA_000193495.2_ASM19349v2
Gluconacetobacter.diazotrophicus.PA1.5 GCA_000067045.1_ASM6704v1
100/100
Commensalibacter.intestini.A911 GCA_000231445.2_ASM23144v1
100/100
79.1/97
Granulibacter.bethesdensis.CGDNIH4 GCA_000576145.2_ASM57614v2
Acidisphaera.rubrifaciens.HS.AP3 GCA_000964365.1_ASM96436v1
88.5/97
Acidiphilium.multivorum.AIU301 GCA_000202835.1_ASM20283v1
100/100
100/100
Rubritepida.flocculans.DSM.14296 GCA_000425365.1_ASM42536v1
Belnapia.moabensis.DSM.16746 GCA_000745835.1_ASM74583v1
100/100
Roseomonas.gilardii GCA_001941945.1_ASM194194v1
100/100
Elioraea.tepidiphila.DSM.17972 GCA_000378465.1_ASM37846v1
89.4/100
100/100
Rhodospirillum.rubrum.F11 GCA_000225955.1_ASM22595v1
Pararhodospirillum.photometricum.DSM.122 GCA_000284415.2_ASM28441v2
100/100
Roseospirillum.parvum GCA_900100455.1
100/100
Caenispirillum.salinarum.AK4 GCA_000315795.1
100/100
Magnetospirillum.magneticum.AMB.1 GCA_000009985.1_ASM998v1
100/100
Thalassospira.profundimaris.WP0211 GCA_000300275.1_ASM30027v1
Terasakiella.pusilla.DSM.6293 GCA_000688235.1_ASM68823v1
93.8/68
100/100
alpha.proteobacterium.BAL199 GCA_000171835.1_ASM17183v1
100/100
Thalassobaculum.salexigens.DSM.19539 GCA_000423805.1_ASM42380v1
100/100
Candidatus.Puniceispirillum.marinum.IMCC1322 GCA_000024465.1_ASM2446v1
100/100
Oceanibaculum.indicum GCA_003633955.1_ASM363395v1
100/100
Rhodovibrio.salinarum.DSM.9154 GCA_000515255.1_ASM51525v1
Kiloniella.laminariae.DSM.19542 GCA_000374005.1_ASM37400v1
100/100
100/100
Rhodospirillum.centenum GCA_014197805.1_ASM1419780v1
100/100
Inquilinus.limosus.DSM.16000 GCA_000423185.1_ASM42318v1
99.6/67
Micavibrio.aeruginosavorus.ARL.13 GCA_000226315.1_ASM22631v1
100/100
Candidatus.Paracaedibacter.acanthamoebae GCA_000742835.1_ASM74283v1
100/100
Candidatus.Odyssella.thessalonicensis.L13 GCA_000190415.2_ASM19041v1
Candidatus.Finniella.inopinata GCA_004210305.1_ASM421030v1
52.1/80
100/100
Holospora.undulata.HU1 GCA_000388175.3_ASM38817v3
100/100
Candidatus.Hepatobacter.penaei GCA_000742475.1_ASM74247v1
100/100
100/100
Candidatus.Caedibacter.acanthamoebae GCA_000743035.1_ASM74303v1
100/100
Caedimonas.varicaedens GCA_001192655.1_ASM119265v1
Candidatus.Nucleicultrix.amoebiphila.FS5 GCA_002117145.1_ASM211714v1
100/100
Tistrella.mobilis.KA081020.065 GCA_000264455.2_ASM26445v2
Geminicoccus.roseus.DSM.18922 GCA_000427665.1_ASM42766v1
97.8/98
99.7/100
Candidatus.Neoehrlichia.lotoris.str.RAC413 GCA_000964795.1_ASM96479v1
100/100
Anaplasma.phagocytophilum.str.HZ GCA_000013125.1_ASM1312v1
100/100
Ehrlichia.ruminantium.str.Welgevonden GCA_000026005.1_ASM2600v1
100/100
Wolbachia.endosymbiont.of.Drosophila.melanogaster GCA_000008025.1_ASM802v1
Candidatus.Deianiraea.vastatrix GCA_007993655.1_ASM799365v1
100/100
100/100
Rickettsiales.endosymbiont.of.Trichoplax.sp.H2 GCA_009690945.1
Candidatus.Midichloria.mitochondrii.IricVA GCA_000219355.1_ASM21935v1
100/100
100/100
Rickettsiales.endosymbiont.of.Peranema.trichophorum GCA_004210275.1_ASM421027v1
Candidatus.Jidaibacter.acanthamoeba GCA_000815465.1.UWC36
100/100
100/100
Rickettsia.bellii.RML369.C GCA_000012385.1_ASM1238v1
29.6/86
Candidatus.Megaira.polyxenophila
100/100
Rickettsiales.endosymbiont.of.Stachyamoeba.lipophora GCA_003932735.1_ASM393273v1
Rickettsiales.bacterium.Ac37b GCA_000746585.2_ASM74658v2
100/100
GCA_002422205.1_ASM242220v1*
100/100
GCA_002346425.1_ASM234642v1*
100/100
GCA_002725445.1_ASM272544v1*
100/100
GCA_003241645.1_ASM324164v1*
GCA_002422745.1_ASM242274v1*
100/100
100/100
GCA_002291925.1_ASM229192v1*
100/100
GCA_002291845.1_ASM229184v1*
100/100
100/100
GCA_004293935.1_ASM429393v1*
GCA_002423155.1_ASM242315v1*
GCA_005792635.1_ASM579263v1*
91.1/95
100/100
Magnetofaba.australis.IT.1 GCA_002109495.1_ASM210949v1
Magnetococcus.marinus.MC.1 GCA_000014865.1_ASM1486v1
100/100
86.9/96
Nitrosospira.briensis.C.128 GCA_000619905.2_ASM61990v2
Methylotenera.versatilis.301 GCA_000093025.1_ASM9302v1
100/100
100/100
Ralstonia.pickettii GCA_902374465.1.MGYG.HGUT.01384
Burkholderia.cepacia GCA_009586235.1_ASM958623v1
100/100
100/100
Dasania.marina.DSM.21967 GCA_000373485.1_ASM37348v1
Congregibacter.litoralis.KT71 GCA_000153125.2_ASM15312v2
100/100
100/100
Enterobacter.cloacae GCA_000770155.1_ASM77015v1
Alteromonas.macleodii.ATCC.27126 GCA_000172635.2_ASM17263v2
0.562191

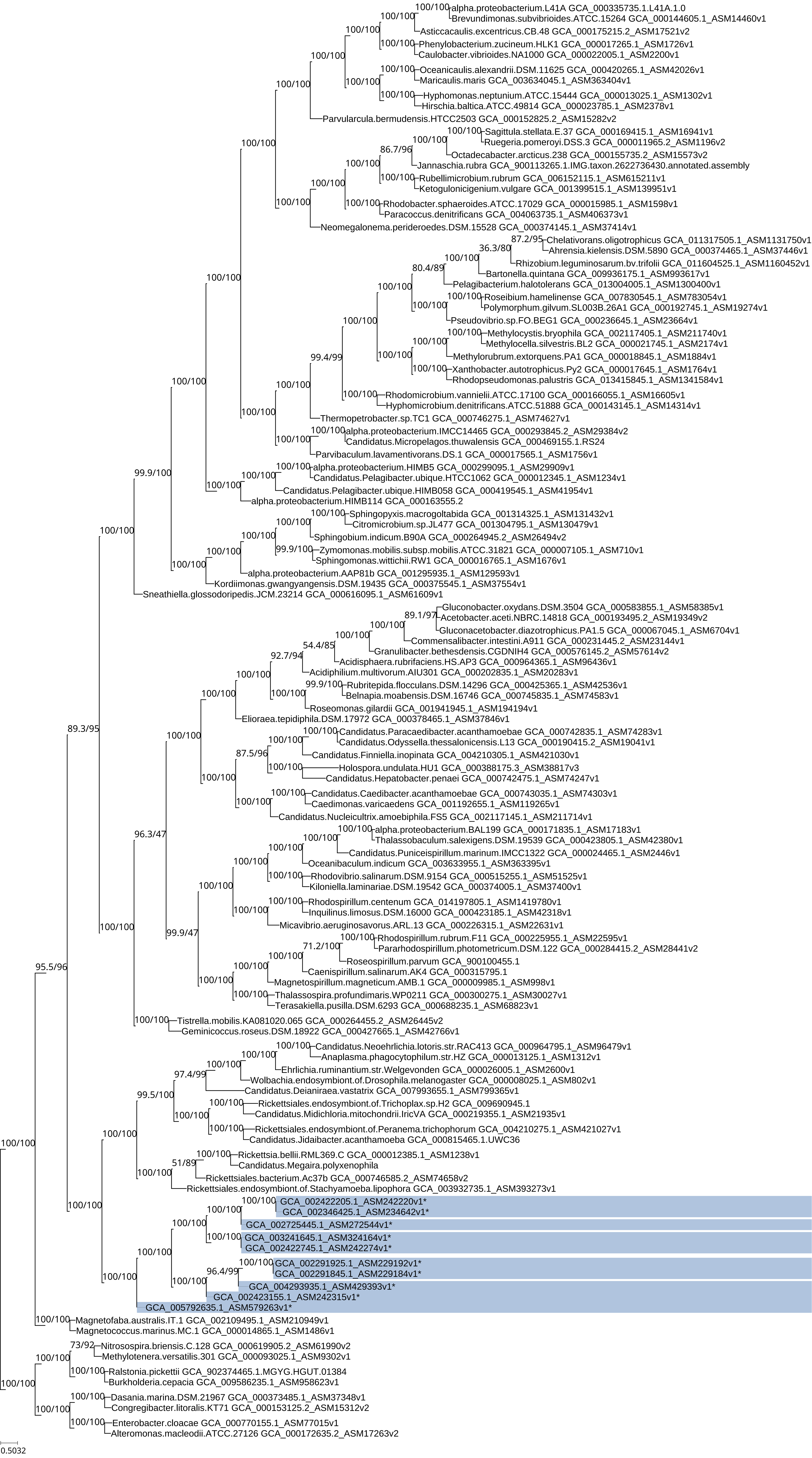
100/100 alpha.proteobacterium.L41A GCA_000335735.1.L41A.1.0
Brevundimonas.subvibrioides.ATCC.15264 GCA_000144605.1_ASM14460v1
100/100
Asticcacaulis.excentricus.CB.48 GCA_000175215.2_ASM17521v2
100/100
100/100 Phenylobacterium.zucineum.HLK1 GCA_000017265.1_ASM1726v1
Caulobacter.vibrioides.NA1000 GCA_000022005.1_ASM2200v1
100/100
100/100 Oceanicaulis.alexandrii.DSM.11625 GCA_000420265.1_ASM42026v1
Maricaulis.maris GCA_003634045.1_ASM363404v1
100/100
100/100
100/100 Hyphomonas.neptunium.ATCC.15444 GCA_000013025.1_ASM1302v1
Hirschia.baltica.ATCC.49814 GCA_000023785.1_ASM2378v1
Parvularcula.bermudensis.HTCC2503 GCA_000152825.2_ASM15282v2
100/100 Sagittula.stellata.E.37 GCA_000169415.1_ASM16941v1
Ruegeria.pomeroyi.DSS.3 GCA_000011965.2_ASM1196v2
100/100
100/100
86.7/96
Octadecabacter.arcticus.238 GCA_000155735.2_ASM15573v2
Jannaschia.rubra GCA_900113265.1.IMG.taxon.2622736430.annotated.assembly
100/100
100/100 Rubellimicrobium.rubrum GCA_006152115.1_ASM615211v1
Ketogulonicigenium.vulgare GCA_001399515.1_ASM139951v1
100/100
100/100
100/100 Rhodobacter.sphaeroides.ATCC.17029 GCA_000015985.1_ASM1598v1
Paracoccus.denitrificans GCA_004063735.1_ASM406373v1
Neomegalonema.perideroedes.DSM.15528 GCA_000374145.1_ASM37414v1
87.2/95 Chelativorans.oligotrophicus GCA_011317505.1_ASM1131750v1
Ahrensia.kielensis.DSM.5890 GCA_000374465.1_ASM37446v1
36.3/80
Rhizobium.leguminosarum.bv.trifolii GCA_011604525.1_ASM1160452v1
100/100
Bartonella.quintana GCA_009936175.1_ASM993617v1
80.4/89
Pelagibacterium.halotolerans GCA_013004005.1_ASM1300400v1
100/100
100/100 Roseibium.hamelinense GCA_007830545.1_ASM783054v1
Polymorphum.gilvum.SL003B.26A1 GCA_000192745.1_ASM19274v1
100/100
Pseudovibrio.sp.FO.BEG1 GCA_000236645.1_ASM23664v1
100/100
100/100 Methylocystis.bryophila GCA_002117405.1_ASM211740v1
Methylocella.silvestris.BL2 GCA_000021745.1_ASM2174v1
100/100
100/100
Methylorubrum.extorquens.PA1 GCA_000018845.1_ASM1884v1
99.4/99
100/100 Xanthobacter.autotrophicus.Py2 GCA_000017645.1_ASM1764v1
Rhodopseudomonas.palustris GCA_013415845.1_ASM1341584v1
100/100
100/100 Rhodomicrobium.vannielii.ATCC.17100 GCA_000166055.1_ASM16605v1
Hyphomicrobium.denitrificans.ATCC.51888 GCA_000143145.1_ASM14314v1
100/100
100/100
Thermopetrobacter.sp.TC1 GCA_000746275.1_ASM74627v1
100/100 alpha.proteobacterium.IMCC14465 GCA_000293845.2_ASM29384v2
Candidatus.Micropelagos.thuwalensis GCA_000469155.1.RS24
100/100
Parvibaculum.lavamentivorans.DS.1 GCA_000017565.1_ASM1756v1
100/100 alpha.proteobacterium.HIMB5 GCA_000299095.1_ASM29909v1
Candidatus.Pelagibacter.ubique.HTCC1062 GCA_000012345.1_ASM1234v1
100/100
99.9/100
Candidatus.Pelagibacter.ubique.HIMB058 GCA_000419545.1_ASM41954v1
100/100
alpha.proteobacterium.HIMB114 GCA_000163555.2
100/100 Sphingopyxis.macrogoltabida GCA_001314325.1_ASM131432v1
Citromicrobium.sp.JL477 GCA_001304795.1_ASM130479v1
100/100
100/100
Sphingobium.indicum.B90A GCA_000264945.2_ASM26494v2
100/100
99.9/100 Zymomonas.mobilis.subsp.mobilis.ATCC.31821 GCA_000007105.1_ASM710v1
Sphingomonas.wittichii.RW1 GCA_000016765.1_ASM1676v1
100/100
alpha.proteobacterium.AAP81b GCA_001295935.1_ASM129593v1
Kordiimonas.gwangyangensis.DSM.19435 GCA_000375545.1_ASM37554v1
Sneathiella.glossodoripedis.JCM.23214 GCA_000616095.1_ASM61609v1
Gluconobacter.oxydans.DSM.3504 GCA_000583855.1_ASM58385v1
89.1/97
Acetobacter.aceti.NBRC.14818 GCA_000193495.2_ASM19349v2
100/100
Gluconacetobacter.diazotrophicus.PA1.5 GCA_000067045.1_ASM6704v1
100/100
Commensalibacter.intestini.A911 GCA_000231445.2_ASM23144v1
54.4/85
Granulibacter.bethesdensis.CGDNIH4 GCA_000576145.2_ASM57614v2
92.7/94
Acidisphaera.rubrifaciens.HS.AP3 GCA_000964365.1_ASM96436v1
100/100
Acidiphilium.multivorum.AIU301 GCA_000202835.1_ASM20283v1
99.9/100 Rubritepida.flocculans.DSM.14296 GCA_000425365.1_ASM42536v1
100/100
100/100
Belnapia.moabensis.DSM.16746 GCA_000745835.1_ASM74583v1
Roseomonas.gilardii GCA_001941945.1_ASM194194v1
89.3/95
Elioraea.tepidiphila.DSM.17972 GCA_000378465.1_ASM37846v1
100/100 Candidatus.Paracaedibacter.acanthamoebae GCA_000742835.1_ASM74283v1
100/100
Candidatus.Odyssella.thessalonicensis.L13 GCA_000190415.2_ASM19041v1
100/100
Candidatus.Finniella.inopinata GCA_004210305.1_ASM421030v1
87.5/96
100/100
Holospora.undulata.HU1 GCA_000388175.3_ASM38817v3
100/100
Candidatus.Hepatobacter.penaei GCA_000742475.1_ASM74247v1
100/100 Candidatus.Caedibacter.acanthamoebae GCA_000743035.1_ASM74303v1
Caedimonas.varicaedens GCA_001192655.1_ASM119265v1
100/100
Candidatus.Nucleicultrix.amoebiphila.FS5 GCA_002117145.1_ASM211714v1
100/100 alpha.proteobacterium.BAL199 GCA_000171835.1_ASM17183v1
Thalassobaculum.salexigens.DSM.19539 GCA_000423805.1_ASM42380v1
100/100
96.3/47
100/100
Candidatus.Puniceispirillum.marinum.IMCC1322 GCA_000024465.1_ASM2446v1
100/100
Oceanibaculum.indicum GCA_003633955.1_ASM363395v1
100/100 Rhodovibrio.salinarum.DSM.9154 GCA_000515255.1_ASM51525v1
100/100
Kiloniella.laminariae.DSM.19542 GCA_000374005.1_ASM37400v1
100/100 Rhodospirillum.centenum GCA_014197805.1_ASM1419780v1
100/100
Inquilinus.limosus.DSM.16000 GCA_000423185.1_ASM42318v1
100/100
Micavibrio.aeruginosavorus.ARL.13 GCA_000226315.1_ASM22631v1
99.9/47
100/100 Rhodospirillum.rubrum.F11 GCA_000225955.1_ASM22595v1
71.2/100
Pararhodospirillum.photometricum.DSM.122 GCA_000284415.2_ASM28441v2
100/100
Roseospirillum.parvum GCA_900100455.1
95.5/96
Caenispirillum.salinarum.AK4 GCA_000315795.1
100/100
Magnetospirillum.magneticum.AMB.1 GCA_000009985.1_ASM998v1
100/100 Thalassospira.profundimaris.WP0211 GCA_000300275.1_ASM30027v1
Terasakiella.pusilla.DSM.6293 GCA_000688235.1_ASM68823v1
100/100 Tistrella.mobilis.KA081020.065 GCA_000264455.2_ASM26445v2
Geminicoccus.roseus.DSM.18922 GCA_000427665.1_ASM42766v1
100/100 Candidatus.Neoehrlichia.lotoris.str.RAC413 GCA_000964795.1_ASM96479v1
Anaplasma.phagocytophilum.str.HZ GCA_000013125.1_ASM1312v1
100/100
Ehrlichia.ruminantium.str.Welgevonden GCA_000026005.1_ASM2600v1
100/100
97.4/99
Wolbachia.endosymbiont.of.Drosophila.melanogaster GCA_000008025.1_ASM802v1
Candidatus.Deianiraea.vastatrix GCA_007993655.1_ASM799365v1
99.5/100
100/100 Rickettsiales.endosymbiont.of.Trichoplax.sp.H2 GCA_009690945.1
100/100
Candidatus.Midichloria.mitochondrii.IricVA GCA_000219355.1_ASM21935v1
100/100
100/100 Rickettsiales.endosymbiont.of.Peranema.trichophorum GCA_004210275.1_ASM421027v1
Candidatus.Jidaibacter.acanthamoeba GCA_000815465.1.UWC36
100/100
100/100 Rickettsia.bellii.RML369.C GCA_000012385.1_ASM1238v1
Candidatus.Megaira.polyxenophila
51/89
Rickettsiales.bacterium.Ac37b GCA_000746585.2_ASM74658v2
100/100
Rickettsiales.endosymbiont.of.Stachyamoeba.lipophora GCA_003932735.1_ASM393273v1
100/100 GCA_002422205.1_ASM242220v1*
100/100
GCA_002346425.1_ASM234642v1*
100/100
GCA_002725445.1_ASM272544v1*
100/100
GCA_003241645.1_ASM324164v1*
GCA_002422745.1_ASM242274v1*
100/100
100/100 GCA_002291925.1_ASM229192v1*
100/100
GCA_002291845.1_ASM229184v1*
100/100
96.4/99
GCA_004293935.1_ASM429393v1*
GCA_002423155.1_ASM242315v1*
GCA_005792635.1_ASM579263v1*
100/100 Magnetofaba.australis.IT.1 GCA_002109495.1_ASM210949v1
Magnetococcus.marinus.MC.1 GCA_000014865.1_ASM1486v1
73/92 Nitrosospira.briensis.C.128 GCA_000619905.2_ASM61990v2
100/100
Methylotenera.versatilis.301 GCA_000093025.1_ASM9302v1
100/100 Ralstonia.pickettii GCA_902374465.1.MGYG.HGUT.01384
Burkholderia.cepacia GCA_009586235.1_ASM958623v1
100/100
100/100 Dasania.marina.DSM.21967 GCA_000373485.1_ASM37348v1
Congregibacter.litoralis.KT71 GCA_000153125.2_ASM15312v2
100/100
100/100 Enterobacter.cloacae GCA_000770155.1_ASM77015v1
Alteromonas.macleodii.ATCC.27126 GCA_000172635.2_ASM17263v2
0.5032

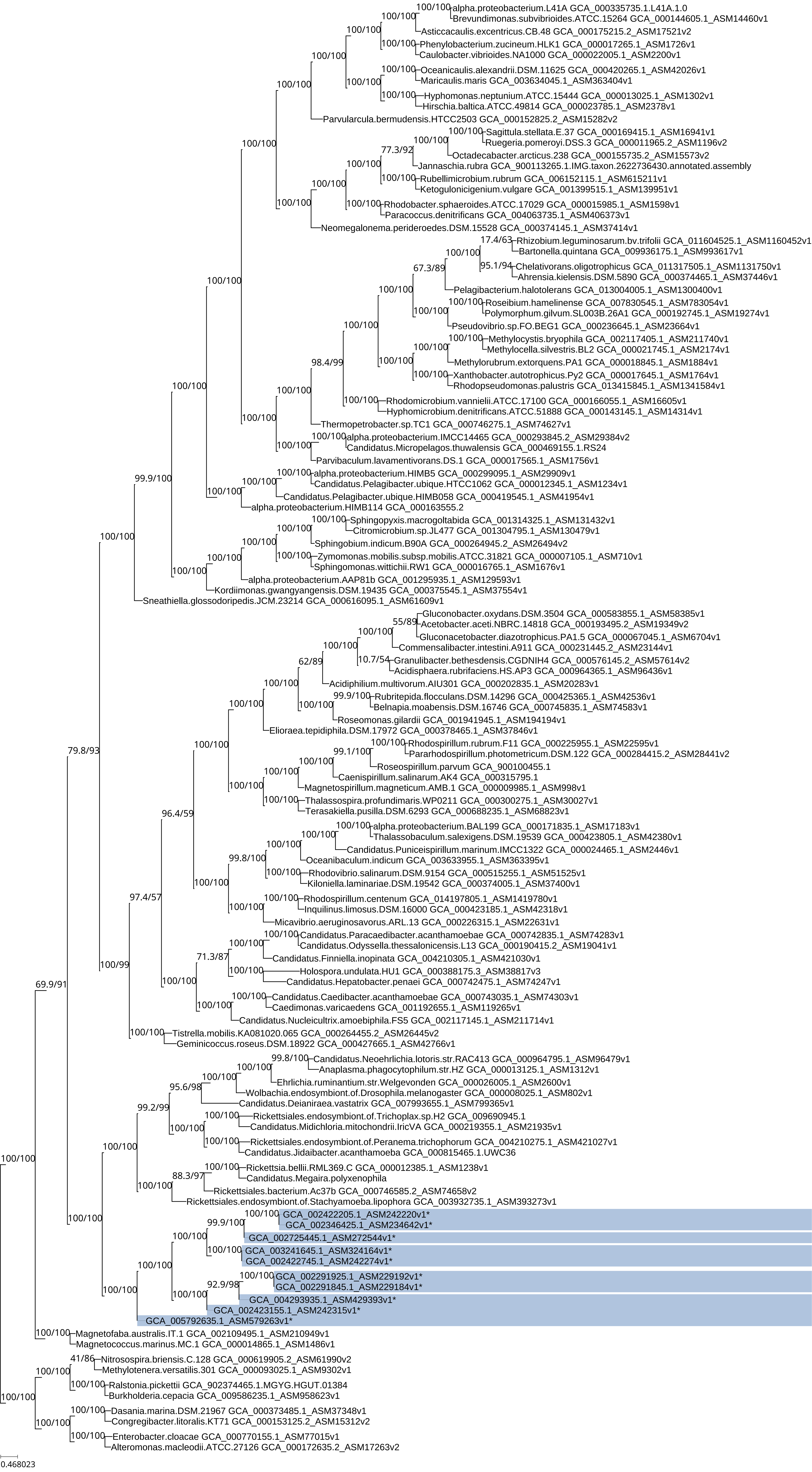

alpha.proteobacterium.L41A GCA_000335735.1.L41A.1.0
Brevundimonas.subvibrioides.ATCC.15264 GCA_000144605.1_ASM14460v1
Asticcacaulis.excentricus.CB.48 GCA_000175215.2_ASM17521v2
Phenylobacterium.zucineum.HLK1 GCA_000017265.1_ASM1726v1
Caulobacter.vibrioides.NA1000 GCA_000022005.1_ASM2200v1
Oceanicaulis.alexandrii.DSM.11625 GCA_000420265.1_ASM42026v1
Maricaulis.maris GCA_003634045.1_ASM363404v1
Hyphomonas.neptunium.ATCC.15444 GCA_000013025.1_ASM1302v1
Hirschia.baltica.ATCC.49814 GCA_000023785.1_ASM2378v1
Parvularcula.bermudensis.HTCC2503 GCA_000152825.2_ASM15282v2
Sagittula.stellata.E.37 GCA_000169415.1_ASM16941v1
Ruegeria.pomeroyi.DSS.3 GCA_000011965.2_ASM1196v2
Octadecabacter.arcticus.238 GCA_000155735.2_ASM15573v2
Jannaschia.rubra GCA_900113265.1.IMG.taxon.2622736430.annotated.assembly
Rubellimicrobium.rubrum GCA_006152115.1_ASM615211v1
Ketogulonicigenium.vulgare GCA_001399515.1_ASM139951v1
Rhodobacter.sphaeroides.ATCC.17029 GCA_000015985.1_ASM1598v1
Paracoccus.denitrificans GCA_004063735.1_ASM406373v1
Neomegalonema.perideroedes.DSM.15528 GCA_000374145.1_ASM37414v1
Rhizobium.leguminosarum.bv.trifolii GCA_011604525.1_ASM1160452v1
Bartonella.quintana GCA_009936175.1_ASM993617v1
Chelativorans.oligotrophicus GCA_011317505.1_ASM1131750v1
Ahrensia.kielensis.DSM.5890 GCA_000374465.1_ASM37446v1
Pelagibacterium.halotolerans GCA_013004005.1_ASM1300400v1
Roseibium.hamelinense GCA_007830545.1_ASM783054v1
Polymorphum.gilvum.SL003B.26A1 GCA_000192745.1_ASM19274v1
Pseudovibrio.sp.FO.BEG1 GCA_000236645.1_ASM23664v1
Methylocystis.bryophila GCA_002117405.1_ASM211740v1
Methylocella.silvestris.BL2 GCA_000021745.1_ASM2174v1
Methylorubrum.extorquens.PA1 GCA_000018845.1_ASM1884v1
Xanthobacter.autotrophicus.Py2 GCA_000017645.1_ASM1764v1
Rhodopseudomonas.palustris GCA_013415845.1_ASM1341584v1
Rhodomicrobium.vannielii.ATCC.17100 GCA_000166055.1_ASM16605v1
Hyphomicrobium.denitrificans.ATCC.51888 GCA_000143145.1_ASM14314v1
Thermopetrobacter.sp.TC1 GCA_000746275.1_ASM74627v1
alpha.proteobacterium.IMCC14465 GCA_000293845.2_ASM29384v2
Candidatus.Micropelagos.thuwalensis GCA_000469155.1.RS24
Parvibaculum.lavamentivorans.DS.1 GCA_000017565.1_ASM1756v1
alpha.proteobacterium.HIMB5 GCA_000299095.1_ASM29909v1
Candidatus.Pelagibacter.ubique.HTCC1062 GCA_000012345.1_ASM1234v1
Candidatus.Pelagibacter.ubique.HIMB058 GCA_000419545.1_ASM41954v1
alpha.proteobacterium.HIMB114 GCA_000163555.2
Sphingopyxis.macrogoltabida GCA_001314325.1_ASM131432v1
Citromicrobium.sp.JL477 GCA_001304795.1_ASM130479v1
Sphingobium.indicum.B90A GCA_000264945.2_ASM26494v2
Zymomonas.mobilis.subsp.mobilis.ATCC.31821 GCA_000007105.1_ASM710v1
Sphingomonas.wittichii.RW1 GCA_000016765.1_ASM1676v1
alpha.proteobacterium.AAP81b GCA_001295935.1_ASM129593v1
Kordiimonas.gwangyangensis.DSM.19435 GCA_000375545.1_ASM37554v1
Sneathiella.glossodoripedis.JCM.23214 GCA_000616095.1_ASM61609v1
Gluconobacter.oxydans.DSM.3504 GCA_000583855.1_ASM58385v1
Acetobacter.aceti.NBRC.14818 GCA_000193495.2_ASM19349v2
Gluconacetobacter.diazotrophicus.PA1.5 GCA_000067045.1_ASM6704v1
Commensalibacter.intestini.A911 GCA_000231445.2_ASM23144v1
Granulibacter.bethesdensis.CGDNIH4 GCA_000576145.2_ASM57614v2
Acidisphaera.rubrifaciens.HS.AP3 GCA_000964365.1_ASM96436v1
Acidiphilium.multivorum.AIU301 GCA_000202835.1_ASM20283v1
Rubritepida.flocculans.DSM.14296 GCA_000425365.1_ASM42536v1
Belnapia.moabensis.DSM.16746 GCA_000745835.1_ASM74583v1
Roseomonas.gilardii GCA_001941945.1_ASM194194v1
Elioraea.tepidiphila.DSM.17972 GCA_000378465.1_ASM37846v1
Rhodospirillum.rubrum.F11 GCA_000225955.1_ASM22595v1
Pararhodospirillum.photometricum.DSM.122 GCA_000284415.2_ASM28441v2
Roseospirillum.parvum GCA_900100455.1
Caenispirillum.salinarum.AK4 GCA_000315795.1
Magnetospirillum.magneticum.AMB.1 GCA_000009985.1_ASM998v1
Thalassospira.profundimaris.WP0211 GCA_000300275.1_ASM30027v1
Terasakiella.pusilla.DSM.6293 GCA_000688235.1_ASM68823v1
alpha.proteobacterium.BAL199 GCA_000171835.1_ASM17183v1
Thalassobaculum.salexigens.DSM.19539 GCA_000423805.1_ASM42380v1
Candidatus.Puniceispirillum.marinum.IMCC1322 GCA_000024465.1_ASM2446v1
Oceanibaculum.indicum GCA_003633955.1_ASM363395v1
Rhodovibrio.salinarum.DSM.9154 GCA_000515255.1_ASM51525v1
Kiloniella.laminariae.DSM.19542 GCA_000374005.1_ASM37400v1
Rhodospirillum.centenum GCA_014197805.1_ASM1419780v1
Inquilinus.limosus.DSM.16000 GCA_000423185.1_ASM42318v1
Micavibrio.aeruginosavorus.ARL.13 GCA_000226315.1_ASM22631v1
Candidatus.Paracaedibacter.acanthamoebae GCA_000742835.1_ASM74283v1
Candidatus.Odyssella.thessalonicensis.L13 GCA_000190415.2_ASM19041v1
Candidatus.Finniella.inopinata GCA_004210305.1_ASM421030v1
Holospora.undulata.HU1 GCA_000388175.3_ASM38817v3
Candidatus.Hepatobacter.penaei GCA_000742475.1_ASM74247v1
Candidatus.Caedibacter.acanthamoebae GCA_000743035.1_ASM74303v1
Caedimonas.varicaedens GCA_001192655.1_ASM119265v1
Candidatus.Nucleicultrix.amoebiphila.FS5 GCA_002117145.1_ASM211714v1
Tistrella.mobilis.KA081020.065 GCA_000264455.2_ASM26445v2
Geminicoccus.roseus.DSM.18922 GCA_000427665.1_ASM42766v1
Candidatus.Neoehrlichia.lotoris.str.RAC413 GCA_000964795.1_ASM96479v1
Anaplasma.phagocytophilum.str.HZ GCA_000013125.1_ASM1312v1
Ehrlichia.ruminantium.str.Welgevonden GCA_000026005.1_ASM2600v1
Wolbachia.endosymbiont.of.Drosophila.melanogaster GCA_000008025.1_ASM802v1
Candidatus.Deianiraea.vastatrix GCA_007993655.1_ASM799365v1
Rickettsiales.endosymbiont.of.Trichoplax.sp.H2 GCA_009690945.1
Candidatus.Midichloria.mitochondrii.IricVA GCA_000219355.1_ASM21935v1
Rickettsiales.endosymbiont.of.Peranema.trichophorum GCA_004210275.1_ASM421027v1
Candidatus.Jidaibacter.acanthamoeba GCA_000815465.1.UWC36
Rickettsia.bellii.RML369.C GCA_000012385.1_ASM1238v1
Candidatus.Megaira.polyxenophila
Rickettsiales.bacterium.Ac37b GCA_000746585.2_ASM74658v2
Rickettsiales.endosymbiont.of.Stachyamoeba.lipophora GCA_003932735.1_ASM393273v1
GCA_002422205.1_ASM242220v1*
GCA_002346425.1_ASM234642v1*
GCA_002725445.1_ASM272544v1*
GCA_003241645.1_ASM324164v1*
GCA_002422745.1_ASM242274v1*
GCA_002291925.1_ASM229192v1*
GCA_002291845.1_ASM229184v1*
GCA_004293935.1_ASM429393v1*
GCA_002423155.1_ASM242315v1*
GCA_005792635.1_ASM579263v1*
Magnetofaba.australis.IT.1 GCA_002109495.1_ASM210949v1
Magnetococcus.marinus.MC.1 GCA_000014865.1_ASM1486v1
Nitrosospira.briensis.C.128 GCA_000619905.2_ASM61990v2
Methylotenera.versatilis.301 GCA_000093025.1_ASM9302v1
Ralstonia.pickettii GCA_902374465.1.MGYG.HGUT.01384
Burkholderia.cepacia GCA_009586235.1_ASM958623v1
Dasania.marina.DSM.21967 GCA_000373485.1_ASM37348v1
Congregibacter.litoralis.KT71 GCA_000153125.2_ASM15312v2
Enterobacter.cloacae GCA_000770155.1_ASM77015v1
Alteromonas.macleodii.ATCC.27126 GCA_000172635.2_ASM17263v2
0.468023

Group I

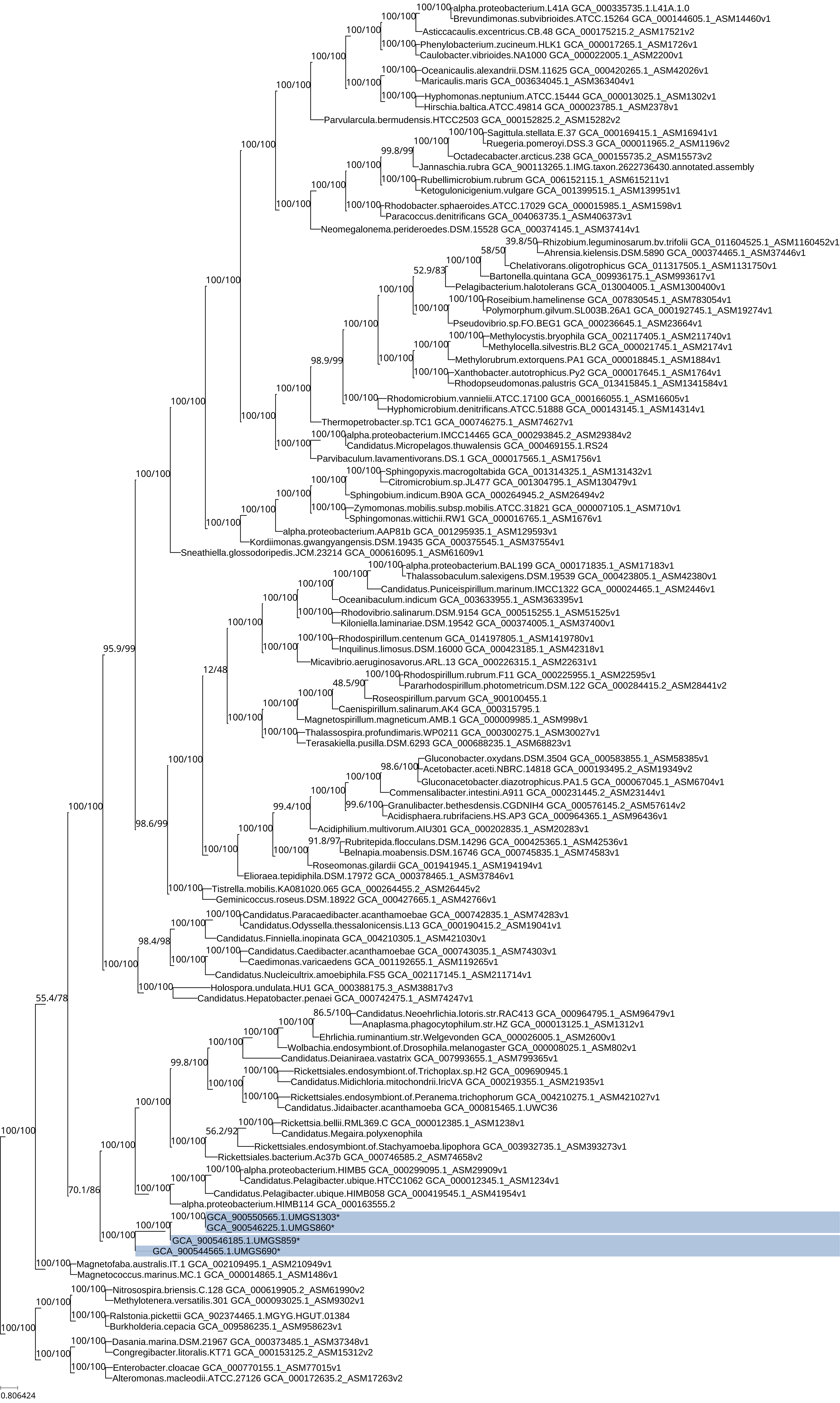
alpha.proteobacterium.L41A GCA_000335735.1.L41A.1.0
Brevundimonas.subvibrioides.ATCC.15264 GCA_000144605.1_ASM14460v1
Asticcacaulis.excentricus.CB.48 GCA_000175215.2_ASM17521v2
Phenylobacterium.zucineum.HLK1 GCA_000017265.1_ASM1726v1
Caulobacter.vibrioides.NA1000 GCA_000022005.1_ASM2200v1
Oceanicaulis.alexandrii.DSM.11625 GCA_000420265.1_ASM42026v1
Maricaulis.maris GCA_003634045.1_ASM363404v1
Hyphomonas.neptunium.ATCC.15444 GCA_000013025.1_ASM1302v1
Hirschia.baltica.ATCC.49814 GCA_000023785.1_ASM2378v1
Parvularcula.bermudensis.HTCC2503 GCA_000152825.2_ASM15282v2
Sagittula.stellata.E.37 GCA_000169415.1_ASM16941v1
Ruegeria.pomeroyi.DSS.3 GCA_000011965.2_ASM1196v2
Octadecabacter.arcticus.238 GCA_000155735.2_ASM15573v2
Jannaschia.rubra GCA_900113265.1.IMG.taxon.2622736430.annotated.assembly
Rubellimicrobium.rubrum GCA_006152115.1_ASM615211v1
Ketogulonicigenium.vulgare GCA_001399515.1_ASM139951v1
Rhodobacter.sphaeroides.ATCC.17029 GCA_000015985.1_ASM1598v1
Paracoccus.denitrificans GCA_004063735.1_ASM406373v1
Neomegalonema.perideroedes.DSM.15528 GCA_000374145.1_ASM37414v1
Rhizobium.leguminosarum.bv.trifolii GCA_011604525.1_ASM1160452v1
Ahrensia.kielensis.DSM.5890 GCA_000374465.1_ASM37446v1
Chelativorans.oligotrophicus GCA_011317505.1_ASM1131750v1
Bartonella.quintana GCA_009936175.1_ASM993617v1
Pelagibacterium.halotolerans GCA_013004005.1_ASM1300400v1
Roseibium.hamelinense GCA_007830545.1_ASM783054v1
Polymorphum.gilvum.SL003B.26A1 GCA_000192745.1_ASM19274v1
Pseudovibrio.sp.FO.BEG1 GCA_000236645.1_ASM23664v1
Methylocystis.bryophila GCA_002117405.1_ASM211740v1
Methylocella.silvestris.BL2 GCA_000021745.1_ASM2174v1
Methylorubrum.extorquens.PA1 GCA_000018845.1_ASM1884v1
Xanthobacter.autotrophicus.Py2 GCA_000017645.1_ASM1764v1
Rhodopseudomonas.palustris GCA_013415845.1_ASM1341584v1
Rhodomicrobium.vannielii.ATCC.17100 GCA_000166055.1_ASM16605v1
Hyphomicrobium.denitrificans.ATCC.51888 GCA_000143145.1_ASM14314v1
Thermopetrobacter.sp.TC1 GCA_000746275.1_ASM74627v1
alpha.proteobacterium.IMCC14465 GCA_000293845.2_ASM29384v2
Candidatus.Micropelagos.thuwalensis GCA_000469155.1.RS24
Parvibaculum.lavamentivorans.DS.1 GCA_000017565.1_ASM1756v1
Sphingopyxis.macrogoltabida GCA_001314325.1_ASM131432v1
Citromicrobium.sp.JL477 GCA_001304795.1_ASM130479v1
Sphingobium.indicum.B90A GCA_000264945.2_ASM26494v2
Zymomonas.mobilis.subsp.mobilis.ATCC.31821 GCA_000007105.1_ASM710v1
Sphingomonas.wittichii.RW1 GCA_000016765.1_ASM1676v1
alpha.proteobacterium.AAP81b GCA_001295935.1_ASM129593v1
Kordiimonas.gwangyangensis.DSM.19435 GCA_000375545.1_ASM37554v1
Sneathiella.glossodoripedis.JCM.23214 GCA_000616095.1_ASM61609v1
alpha.proteobacterium.BAL199 GCA_000171835.1_ASM17183v1
Thalassobaculum.salexigens.DSM.19539 GCA_000423805.1_ASM42380v1
Candidatus.Puniceispirillum.marinum.IMCC1322 GCA_000024465.1_ASM2446v1
Oceanibaculum.indicum GCA_003633955.1_ASM363395v1
Rhodovibrio.salinarum.DSM.9154 GCA_000515255.1_ASM51525v1
Kiloniella.laminariae.DSM.19542 GCA_000374005.1_ASM37400v1
Rhodospirillum.centenum GCA_014197805.1_ASM1419780v1
Inquilinus.limosus.DSM.16000 GCA_000423185.1_ASM42318v1
Micavibrio.aeruginosavorus.ARL.13 GCA_000226315.1_ASM22631v1
Rhodospirillum.rubrum.F11 GCA_000225955.1_ASM22595v1
Pararhodospirillum.photometricum.DSM.122 GCA_000284415.2_ASM28441v2
Roseospirillum.parvum GCA_900100455.1
Caenispirillum.salinarum.AK4 GCA_000315795.1
Magnetospirillum.magneticum.AMB.1 GCA_000009985.1_ASM998v1
Thalassospira.profundimaris.WP0211 GCA_000300275.1_ASM30027v1
Terasakiella.pusilla.DSM.6293 GCA_000688235.1_ASM68823v1
Gluconobacter.oxydans.DSM.3504 GCA_000583855.1_ASM58385v1
Acetobacter.aceti.NBRC.14818 GCA_000193495.2_ASM19349v2
Gluconacetobacter.diazotrophicus.PA1.5 GCA_000067045.1_ASM6704v1
Commensalibacter.intestini.A911 GCA_000231445.2_ASM23144v1
Granulibacter.bethesdensis.CGDNIH4 GCA_000576145.2_ASM57614v2
Acidisphaera.rubrifaciens.HS.AP3 GCA_000964365.1_ASM96436v1
Acidiphilium.multivorum.AIU301 GCA_000202835.1_ASM20283v1
Rubritepida.flocculans.DSM.14296 GCA_000425365.1_ASM42536v1
Belnapia.moabensis.DSM.16746 GCA_000745835.1_ASM74583v1
Roseomonas.gilardii GCA_001941945.1_ASM194194v1
Elioraea.tepidiphila.DSM.17972 GCA_000378465.1_ASM37846v1
Tistrella.mobilis.KA081020.065 GCA_000264455.2_ASM26445v2
Geminicoccus.roseus.DSM.18922 GCA_000427665.1_ASM42766v1
Candidatus.Paracaedibacter.acanthamoebae GCA_000742835.1_ASM74283v1
Candidatus.Odyssella.thessalonicensis.L13 GCA_000190415.2_ASM19041v1
Candidatus.Finniella.inopinata GCA_004210305.1_ASM421030v1
Candidatus.Caedibacter.acanthamoebae GCA_000743035.1_ASM74303v1
Caedimonas.varicaedens GCA_001192655.1_ASM119265v1
Candidatus.Nucleicultrix.amoebiphila.FS5 GCA_002117145.1_ASM211714v1
Holospora.undulata.HU1 GCA_000388175.3_ASM38817v3
Candidatus.Hepatobacter.penaei GCA_000742475.1_ASM74247v1
Candidatus.Neoehrlichia.lotoris.str.RAC413 GCA_000964795.1_ASM96479v1
Anaplasma.phagocytophilum.str.HZ GCA_000013125.1_ASM1312v1
Ehrlichia.ruminantium.str.Welgevonden GCA_000026005.1_ASM2600v1
Wolbachia.endosymbiont.of.Drosophila.melanogaster GCA_000008025.1_ASM802v1
Candidatus.Deianiraea.vastatrix GCA_007993655.1_ASM799365v1
Rickettsiales.endosymbiont.of.Trichoplax.sp.H2 GCA_009690945.1
Candidatus.Midichloria.mitochondrii.IricVA GCA_000219355.1_ASM21935v1
Rickettsiales.endosymbiont.of.Peranema.trichophorum GCA_004210275.1_ASM421027v1
Candidatus.Jidaibacter.acanthamoeba GCA_000815465.1.UWC36
Rickettsia.bellii.RML369.C GCA_000012385.1_ASM1238v1
Candidatus.Megaira.polyxenophila
Rickettsiales.endosymbiont.of.Stachyamoeba.lipophora GCA_003932735.1_ASM393273v1
Rickettsiales.bacterium.Ac37b GCA_000746585.2_ASM74658v2
alpha.proteobacterium.HIMB5 GCA_000299095.1_ASM29909v1
Candidatus.Pelagibacter.ubique.HTCC1062 GCA_000012345.1_ASM1234v1
Candidatus.Pelagibacter.ubique.HIMB058 GCA_000419545.1_ASM41954v1
alpha.proteobacterium.HIMB114 GCA_000163555.2
GCA_900550565.1.UMGS1303*
GCA_900546225.1.UMGS860*
GCA_900546185.1.UMGS859*
GCA_900544565.1.UMGS690*
Magnetofaba.australis.IT.1 GCA_002109495.1_ASM210949v1
Magnetococcus.marinus.MC.1 GCA_000014865.1_ASM1486v1
Nitrosospira.briensis.C.128 GCA_000619905.2_ASM61990v2
Methylotenera.versatilis.301 GCA_000093025.1_ASM9302v1
Ralstonia.pickettii GCA_902374465.1.MGYG.HGUT.01384
Burkholderia.cepacia GCA_009586235.1_ASM958623v1
Dasania.marina.DSM.21967 GCA_000373485.1_ASM37348v1
Congregibacter.litoralis.KT71 GCA_000153125.2_ASM15312v2
Enterobacter.cloacae GCA_000770155.1_ASM77015v1
Alteromonas.macleodii.ATCC.27126 GCA_000172635.2_ASM17263v2
0.806424

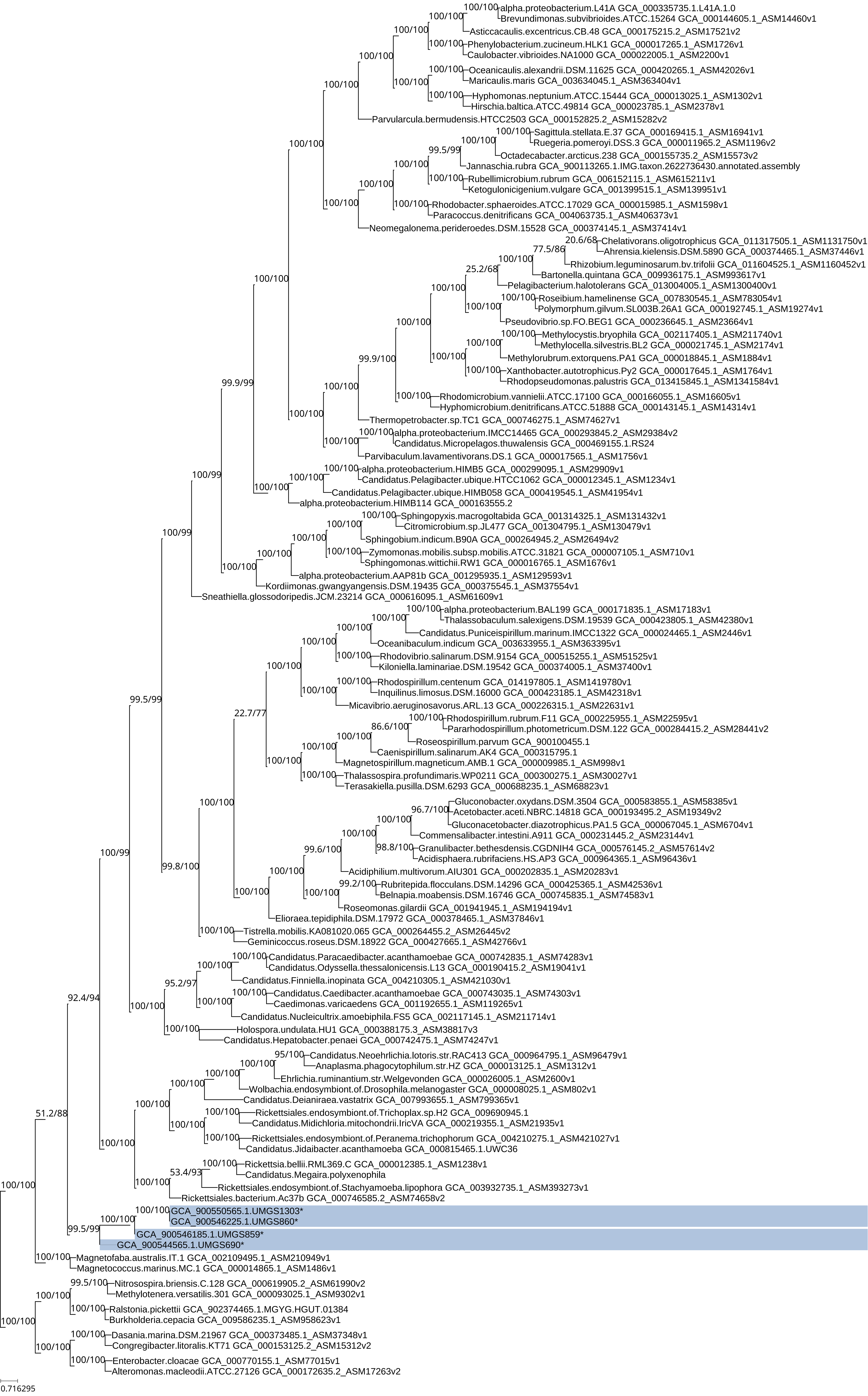
100/100
alpha.proteobacterium.L41A GCA_000335735.1.L41A.1.0
100/100
Brevundimonas.subvibrioides.ATCC.15264 GCA_000144605.1_ASM14460v1
100/100
Asticcacaulis.excentricus.CB.48 GCA_000175215.2_ASM17521v2
100/100
Phenylobacterium.zucineum.HLK1 GCA_000017265.1_ASM1726v1
100/100
Caulobacter.vibrioides.NA1000 GCA_000022005.1_ASM2200v1
100/100
Oceanicaulis.alexandrii.DSM.11625 GCA_000420265.1_ASM42026v1
100/100
Maricaulis.maris GCA_003634045.1_ASM363404v1
100/100
100/100
Hyphomonas.neptunium.ATCC.15444 GCA_000013025.1_ASM1302v1
Hirschia.baltica.ATCC.49814 GCA_000023785.1_ASM2378v1
Parvularcula.bermudensis.HTCC2503 GCA_000152825.2_ASM15282v2
100/100
100/100
Sagittula.stellata.E.37 GCA_000169415.1_ASM16941v1
100/100
Ruegeria.pomeroyi.DSS.3 GCA_000011965.2_ASM1196v2
99.5/99
Octadecabacter.arcticus.238 GCA_000155735.2_ASM15573v2
100/100
Jannaschia.rubra GCA_900113265.1.IMG.taxon.2622736430.annotated.assembly
100/100
Rubellimicrobium.rubrum GCA_006152115.1_ASM615211v1
100/100
Ketogulonicigenium.vulgare GCA_001399515.1_ASM139951v1
100/100
100/100
Rhodobacter.sphaeroides.ATCC.17029 GCA_000015985.1_ASM1598v1
Paracoccus.denitrificans GCA_004063735.1_ASM406373v1
Neomegalonema.perideroedes.DSM.15528 GCA_000374145.1_ASM37414v1
20.6/68
Chelativorans.oligotrophicus GCA_011317505.1_ASM1131750v1
77.5/86
Ahrensia.kielensis.DSM.5890 GCA_000374465.1_ASM37446v1
100/100
Rhizobium.leguminosarum.bv.trifolii GCA_011604525.1_ASM1160452v1
100/100
25.2/68
Bartonella.quintana GCA_009936175.1_ASM993617v1
Pelagibacterium.halotolerans GCA_013004005.1_ASM1300400v1
100/100
100/100
Roseibium.hamelinense GCA_007830545.1_ASM783054v1
100/100
Polymorphum.gilvum.SL003B.26A1 GCA_000192745.1_ASM19274v1
Pseudovibrio.sp.FO.BEG1 GCA_000236645.1_ASM23664v1
100/100
100/100
Methylocystis.bryophila GCA_002117405.1_ASM211740v1
100/100
Methylocella.silvestris.BL2 GCA_000021745.1_ASM2174v1
99.9/100
Methylorubrum.extorquens.PA1 GCA_000018845.1_ASM1884v1
100/100
100/100
Xanthobacter.autotrophicus.Py2 GCA_000017645.1_ASM1764v1
99.9/99
100/100
Rhodopseudomonas.palustris GCA_013415845.1_ASM1341584v1
100/100
Rhodomicrobium.vannielii.ATCC.17100 GCA_000166055.1_ASM16605v1
Hyphomicrobium.denitrificans.ATCC.51888 GCA_000143145.1_ASM14314v1
100/100
Thermopetrobacter.sp.TC1 GCA_000746275.1_ASM74627v1
100/100
alpha.proteobacterium.IMCC14465 GCA_000293845.2_ASM29384v2
100/100
Candidatus.Micropelagos.thuwalensis GCA_000469155.1.RS24
Parvibaculum.lavamentivorans.DS.1 GCA_000017565.1_ASM1756v1
100/99
100/100
alpha.proteobacterium.HIMB5 GCA_000299095.1_ASM29909v1
100/100
Candidatus.Pelagibacter.ubique.HTCC1062 GCA_000012345.1_ASM1234v1
100/100
Candidatus.Pelagibacter.ubique.HIMB058 GCA_000419545.1_ASM41954v1
alpha.proteobacterium.HIMB114 GCA_000163555.2
100/99
100/100
Sphingopyxis.macrogoltabida GCA_001314325.1_ASM131432v1
100/100
Citromicrobium.sp.JL477 GCA_001304795.1_ASM130479v1
100/100
Sphingobium.indicum.B90A GCA_000264945.2_ASM26494v2
100/100
100/100
Zymomonas.mobilis.subsp.mobilis.ATCC.31821 GCA_000007105.1_ASM710v1
Sphingomonas.wittichii.RW1 GCA_000016765.1_ASM1676v1
100/100
alpha.proteobacterium.AAP81b GCA_001295935.1_ASM129593v1
Kordiimonas.gwangyangensis.DSM.19435 GCA_000375545.1_ASM37554v1
Sneathiella.glossodoripedis.JCM.23214 GCA_000616095.1_ASM61609v1
100/100
alpha.proteobacterium.BAL199 GCA_000171835.1_ASM17183v1
100/100
Thalassobaculum.salexigens.DSM.19539 GCA_000423805.1_ASM42380v1
100/100
Candidatus.Puniceispirillum.marinum.IMCC1322 GCA_000024465.1_ASM2446v1
100/100
Oceanibaculum.indicum GCA_003633955.1_ASM363395v1
100/100
Rhodovibrio.salinarum.DSM.9154 GCA_000515255.1_ASM51525v1
100/100
Kiloniella.laminariae.DSM.19542 GCA_000374005.1_ASM37400v1
100/100
Rhodospirillum.centenum GCA_014197805.1_ASM1419780v1
100/100
Inquilinus.limosus.DSM.16000 GCA_000423185.1_ASM42318v1
99.5/99
Micavibrio.aeruginosavorus.ARL.13 GCA_000226315.1_ASM22631v1
22.7/77
100/100
Rhodospirillum.rubrum.F11 GCA_000225955.1_ASM22595v1
86.6/100
Pararhodospirillum.photometricum.DSM.122 GCA_000284415.2_ASM28441v2
100/100
Roseospirillum.parvum GCA_900100455.1
100/100
Caenispirillum.salinarum.AK4 GCA_000315795.1
100/100
Magnetospirillum.magneticum.AMB.1 GCA_000009985.1_ASM998v1
100/100
Thalassospira.profundimaris.WP0211 GCA_000300275.1_ASM30027v1
Terasakiella.pusilla.DSM.6293 GCA_000688235.1_ASM68823v1
100/100
Gluconobacter.oxydans.DSM.3504 GCA_000583855.1_ASM58385v1
96.7/100
Acetobacter.aceti.NBRC.14818 GCA_000193495.2_ASM19349v2
100/100
Gluconacetobacter.diazotrophicus.PA1.5 GCA_000067045.1_ASM6704v1
100/100
Commensalibacter.intestini.A911 GCA_000231445.2_ASM23144v1
100/99
98.8/100
Granulibacter.bethesdensis.CGDNIH4 GCA_000576145.2_ASM57614v2
99.6/100
Acidisphaera.rubrifaciens.HS.AP3 GCA_000964365.1_ASM96436v1
Acidiphilium.multivorum.AIU301 GCA_000202835.1_ASM20283v1
99.8/100
100/100
99.2/100
Rubritepida.flocculans.DSM.14296 GCA_000425365.1_ASM42536v1
100/100
100/100
Belnapia.moabensis.DSM.16746 GCA_000745835.1_ASM74583v1
Roseomonas.gilardii GCA_001941945.1_ASM194194v1
Elioraea.tepidiphila.DSM.17972 GCA_000378465.1_ASM37846v1
100/100
Tistrella.mobilis.KA081020.065 GCA_000264455.2_ASM26445v2
Geminicoccus.roseus.DSM.18922 GCA_000427665.1_ASM42766v1
100/100
Candidatus.Paracaedibacter.acanthamoebae GCA_000742835.1_ASM74283v1
100/100
Candidatus.Odyssella.thessalonicensis.L13 GCA_000190415.2_ASM19041v1
95.2/97
Candidatus.Finniella.inopinata GCA_004210305.1_ASM421030v1
100/100
Candidatus.Caedibacter.acanthamoebae GCA_000743035.1_ASM74303v1
92.4/94
100/100
100/100
Caedimonas.varicaedens GCA_001192655.1_ASM119265v1
Candidatus.Nucleicultrix.amoebiphila.FS5 GCA_002117145.1_ASM211714v1
100/100
Holospora.undulata.HU1 GCA_000388175.3_ASM38817v3
Candidatus.Hepatobacter.penaei GCA_000742475.1_ASM74247v1
95/100
Candidatus.Neoehrlichia.lotoris.str.RAC413 GCA_000964795.1_ASM96479v1
100/100
Anaplasma.phagocytophilum.str.HZ GCA_000013125.1_ASM1312v1
100/100
Ehrlichia.ruminantium.str.Welgevonden GCA_000026005.1_ASM2600v1
100/100
Wolbachia.endosymbiont.of.Drosophila.melanogaster GCA_000008025.1_ASM802v1
100/100
Candidatus.Deianiraea.vastatrix GCA_007993655.1_ASM799365v1
100/100
Rickettsiales.endosymbiont.of.Trichoplax.sp.H2 GCA_009690945.1
51.2/88
100/100
Candidatus.Midichloria.mitochondrii.IricVA GCA_000219355.1_ASM21935v1
100/100
Rickettsiales.endosymbiont.of.Peranema.trichophorum GCA_004210275.1_ASM421027v1
100/100
Candidatus.Jidaibacter.acanthamoeba GCA_000815465.1.UWC36
100/100
Rickettsia.bellii.RML369.C GCA_000012385.1_ASM1238v1
53.4/93
Candidatus.Megaira.polyxenophila
100/100
Rickettsiales.endosymbiont.of.Stachyamoeba.lipophora GCA_003932735.1_ASM393273v1
100/100
Rickettsiales.bacterium.Ac37b GCA_000746585.2_ASM74658v2
100/100
GCA_900550565.1.UMGS1303*
100/100
GCA_900546225.1.UMGS860*
99.5/99
GCA_900546185.1.UMGS859*
GCA_900544565.1.UMGS690*
100/100
Magnetofaba.australis.IT.1 GCA_002109495.1_ASM210949v1
Magnetococcus.marinus.MC.1 GCA_000014865.1_ASM1486v1
99.5/100
Nitrosospira.briensis.C.128 GCA_000619905.2_ASM61990v2
100/100
Methylotenera.versatilis.301 GCA_000093025.1_ASM9302v1
100/100
Ralstonia.pickettii GCA_902374465.1.MGYG.HGUT.01384
100/100
Burkholderia.cepacia GCA_009586235.1_ASM958623v1
100/100
Dasania.marina.DSM.21967 GCA_000373485.1_ASM37348v1
100/100
Congregibacter.litoralis.KT71 GCA_000153125.2_ASM15312v2
100/100
Enterobacter.cloacae GCA_000770155.1_ASM77015v1
Alteromonas.macleodii.ATCC.27126 GCA_000172635.2_ASM17263v2
0.716295

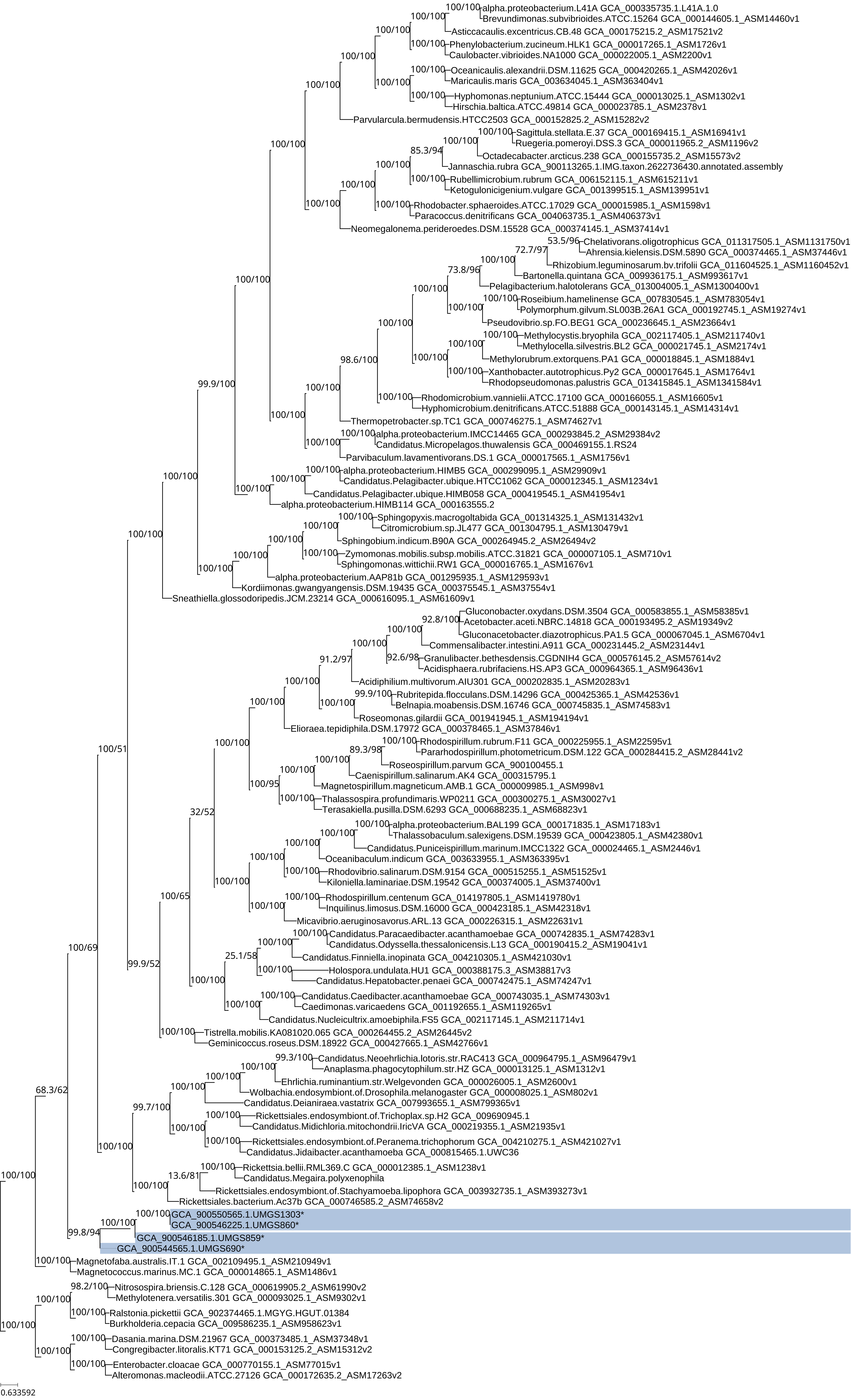

alpha.proteobacterium.L41A GCA_000335735.1.L41A.1.0
Brevundimonas.subvibrioides.ATCC.15264 GCA_000144605.1_ASM14460v1
Asticcacaulis.excentricus.CB.48 GCA_000175215.2_ASM17521v2
Phenylobacterium.zucineum.HLK1 GCA_000017265.1_ASM1726v1
Caulobacter.vibrioides.NA1000 GCA_000022005.1_ASM2200v1
Oceanicaulis.alexandrii.DSM.11625 GCA_000420265.1_ASM42026v1
Maricaulis.maris GCA_003634045.1_ASM363404v1
Hyphomonas.neptunium.ATCC.15444 GCA_000013025.1_ASM1302v1
Hirschia.baltica.ATCC.49814 GCA_000023785.1_ASM2378v1
Parvularcula.bermudensis.HTCC2503 GCA_000152825.2_ASM15282v2
Sagittula.stellata.E.37 GCA_000169415.1_ASM16941v1
Ruegeria.pomeroyi.DSS.3 GCA_000011965.2_ASM1196v2
Octadecabacter.arcticus.238 GCA_000155735.2_ASM15573v2
Jannaschia.rubra GCA_900113265.1.IMG.taxon.2622736430.annotated.assembly
Rubellimicrobium.rubrum GCA_006152115.1_ASM615211v1
Ketogulonicigenium.vulgare GCA_001399515.1_ASM139951v1
Rhodobacter.sphaeroides.ATCC.17029 GCA_000015985.1_ASM1598v1
Paracoccus.denitrificans GCA_004063735.1_ASM406373v1
Neomegalonema.perideroedes.DSM.15528 GCA_000374145.1_ASM37414v1
Chelativorans.oligotrophicus GCA_011317505.1_ASM1131750v1
Ahrensia.kielensis.DSM.5890 GCA_000374465.1_ASM37446v1
Rhizobium.leguminosarum.bv.trifolii GCA_011604525.1_ASM1160452v1
Bartonella.quintana GCA_009936175.1_ASM993617v1
Pelagibacterium.halotolerans GCA_013004005.1_ASM1300400v1
Roseibium.hamelinense GCA_007830545.1_ASM783054v1
Polymorphum.gilvum.SL003B.26A1 GCA_000192745.1_ASM19274v1
Pseudovibrio.sp.FO.BEG1 GCA_000236645.1_ASM23664v1
Methylocystis.bryophila GCA_002117405.1_ASM211740v1
Methylocella.silvestris.BL2 GCA_000021745.1_ASM2174v1
Methylorubrum.extorquens.PA1 GCA_000018845.1_ASM1884v1
Xanthobacter.autotrophicus.Py2 GCA_000017645.1_ASM1764v1
Rhodopseudomonas.palustris GCA_013415845.1_ASM1341584v1
Rhodomicrobium.vannielii.ATCC.17100 GCA_000166055.1_ASM16605v1
Hyphomicrobium.denitrificans.ATCC.51888 GCA_000143145.1_ASM14314v1
Thermopetrobacter.sp.TC1 GCA_000746275.1_ASM74627v1
alpha.proteobacterium.IMCC14465 GCA_000293845.2_ASM29384v2
Candidatus.Micropelagos.thuwalensis GCA_000469155.1.RS24
Parvibaculum.lavamentivorans.DS.1 GCA_000017565.1_ASM1756v1
alpha.proteobacterium.HIMB5 GCA_000299095.1_ASM29909v1
Candidatus.Pelagibacter.ubique.HTCC1062 GCA_000012345.1_ASM1234v1
Candidatus.Pelagibacter.ubique.HIMB058 GCA_000419545.1_ASM41954v1
alpha.proteobacterium.HIMB114 GCA_000163555.2
Sphingopyxis.macrogoltabida GCA_001314325.1_ASM131432v1
Citromicrobium.sp.JL477 GCA_001304795.1_ASM130479v1
Sphingobium.indicum.B90A GCA_000264945.2_ASM26494v2
Zymomonas.mobilis.subsp.mobilis.ATCC.31821 GCA_000007105.1_ASM710v1
Sphingomonas.wittichii.RW1 GCA_000016765.1_ASM1676v1
alpha.proteobacterium.AAP81b GCA_001295935.1_ASM129593v1
Kordiimonas.gwangyangensis.DSM.19435 GCA_000375545.1_ASM37554v1
Sneathiella.glossodoripedis.JCM.23214 GCA_000616095.1_ASM61609v1
Gluconobacter.oxydans.DSM.3504 GCA_000583855.1_ASM58385v1
Acetobacter.aceti.NBRC.14818 GCA_000193495.2_ASM19349v2
Gluconacetobacter.diazotrophicus.PA1.5 GCA_000067045.1_ASM6704v1
Commensalibacter.intestini.A911 GCA_000231445.2_ASM23144v1
Granulibacter.bethesdensis.CGDNIH4 GCA_000576145.2_ASM57614v2
Acidisphaera.rubrifaciens.HS.AP3 GCA_000964365.1_ASM96436v1
Acidiphilium.multivorum.AIU301 GCA_000202835.1_ASM20283v1
Rubritepida.flocculans.DSM.14296 GCA_000425365.1_ASM42536v1
Belnapia.moabensis.DSM.16746 GCA_000745835.1_ASM74583v1
Roseomonas.gilardii GCA_001941945.1_ASM194194v1
Elioraea.tepidiphila.DSM.17972 GCA_000378465.1_ASM37846v1
Rhodospirillum.rubrum.F11 GCA_000225955.1_ASM22595v1
Pararhodospirillum.photometricum.DSM.122 GCA_000284415.2_ASM28441v2
Roseospirillum.parvum GCA_900100455.1
Caenispirillum.salinarum.AK4 GCA_000315795.1
Magnetospirillum.magneticum.AMB.1 GCA_000009985.1_ASM998v1
Thalassospira.profundimaris.WP0211 GCA_000300275.1_ASM30027v1
Terasakiella.pusilla.DSM.6293 GCA_000688235.1_ASM68823v1
alpha.proteobacterium.BAL199 GCA_000171835.1_ASM17183v1
Thalassobaculum.salexigens.DSM.19539 GCA_000423805.1_ASM42380v1
Candidatus.Puniceispirillum.marinum.IMCC1322 GCA_000024465.1_ASM2446v1
Oceanibaculum.indicum GCA_003633955.1_ASM363395v1
Rhodovibrio.salinarum.DSM.9154 GCA_000515255.1_ASM51525v1
Kiloniella.laminariae.DSM.19542 GCA_000374005.1_ASM37400v1
Rhodospirillum.centenum GCA_014197805.1_ASM1419780v1
Inquilinus.limosus.DSM.16000 GCA_000423185.1_ASM42318v1
Micavibrio.aeruginosavorus.ARL.13 GCA_000226315.1_ASM22631v1
Candidatus.Paracaedibacter.acanthamoebae GCA_000742835.1_ASM74283v1
Candidatus.Odyssella.thessalonicensis.L13 GCA_000190415.2_ASM19041v1
Candidatus.Finniella.inopinata GCA_004210305.1_ASM421030v1
Holospora.undulata.HU1 GCA_000388175.3_ASM38817v3
Candidatus.Hepatobacter.penaei GCA_000742475.1_ASM74247v1
Candidatus.Caedibacter.acanthamoebae GCA_000743035.1_ASM74303v1
Caedimonas.varicaedens GCA_001192655.1_ASM119265v1
Candidatus.Nucleicultrix.amoebiphila.FS5 GCA_002117145.1_ASM211714v1
Tistrella.mobilis.KA081020.065 GCA_000264455.2_ASM26445v2
Geminicoccus.roseus.DSM.18922 GCA_000427665.1_ASM42766v1
Candidatus.Neoehrlichia.lotoris.str.RAC413 GCA_000964795.1_ASM96479v1
Anaplasma.phagocytophilum.str.HZ GCA_000013125.1_ASM1312v1
Ehrlichia.ruminantium.str.Welgevonden GCA_000026005.1_ASM2600v1
Wolbachia.endosymbiont.of.Drosophila.melanogaster GCA_000008025.1_ASM802v1
Candidatus.Deianiraea.vastatrix GCA_007993665.1_ASM799365v1
Rickettsiales.endosymbiont.of.Trichoplax.sp.H2 GCA_009690945.1
Candidatus.Midichloria.mitochondrii.IricVA GCA_000219355.1_ASM21935v1
Rickettsiales.endosymbiont.of.Peranema.trichophorum GCA_004210275.1_ASM421027v1
Candidatus.Jidaibacter.acanthamoeba GCA_000815465.1.UWC36
Rickettsia.bellii.RML369.C GCA_000012385.1_ASM1238v1
Candidatus.Megaira.polyxenophila
Rickettsiales.endosymbiont.of.Stachyamoeba.lipophora GCA_003932735.1_ASM393273v1
Rickettsiales.bacterium.Ac37b GCA_000746585.2_ASM74658v2
GCA_900550565.1.UMGS1303*
GCA_900546225.1.UMGS860*
GCA_900546185.1.UMGS859*
GCA_900544565.1.UMGS690*
Magnetofaba.australis.IT.1 GCA_002109495.1_ASM210949v1
Magnetococcus.marinus.MC.1 GCA_000014865.1_ASM1486v1
Nitrosospira.briensis.C.128 GCA_000619905.2_ASM61990v2
Methylotenera.versatilis.301 GCA_000093025.1_ASM9302v1
Ralstonia.pickettii GCA_902374465.1.MGYG.HGUT.01384
Burkholderia.cepacia GCA_009586235.1_ASM958623v1
Dasania.marina.DSM.21967 GCA_000373485.1_ASM37348v1
Congregibacter.litoralis.KT71 GCA_000153125.2_ASM15312v2
Enterobacter.cloacae GCA_000770155.1_ASM77015v1
Alteromonas.macleodii.ATCC.27126 GCA_000172635.2_ASM17263v2
0.633592

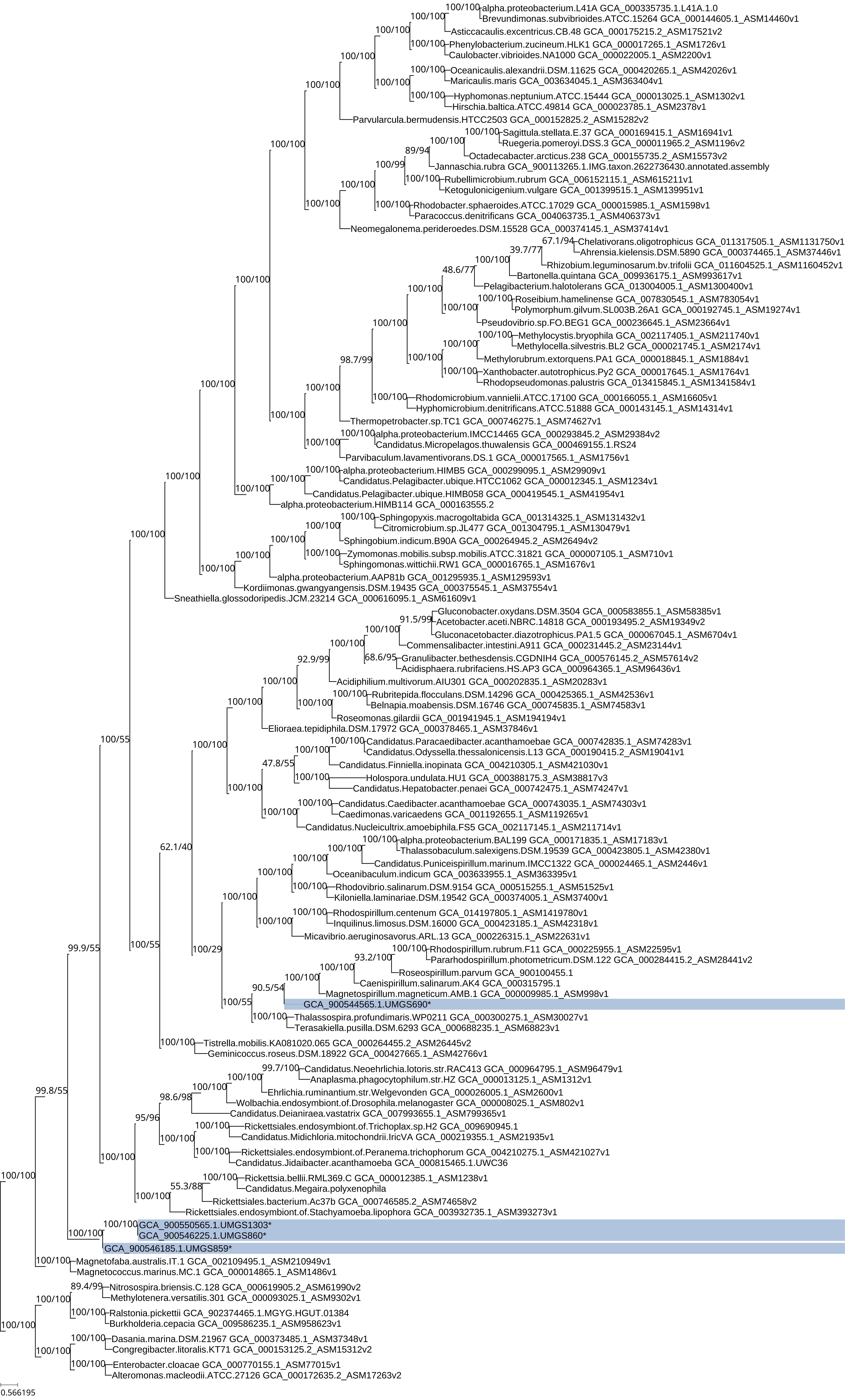

100/100 alpha.proteobacterium.L41A GCA_000335735.1.L41A.1.0
Brevundimonas.subvibrioides.ATCC.15264 GCA_000144605.1_ASM14460v1
100/100
Asticcacaulis.excentricus.CB.48 GCA_000175215.2_ASM17521v2
100/100
100/100 Phenylobacterium.zucineum.HLK1 GCA_000017265.1_ASM1726v1
Caulobacter.vibrioides.NA1000 GCA_000022005.1_ASM2200v1
100/100 Oceanicaulis.alexandrii.DSM.11625 GCA_000420265.1_ASM42026v1
100/100
Maricaulis.maris GCA_003634045.1_ASM363404v1
100/100
100/100 Hyphomonas.neptunium.ATCC.15444 GCA_000013025.1_ASM1302v1
Hirschia.baltica.ATCC.49814 GCA_000023785.1_ASM2378v1
Parvularcula.bermudensis.HTCC2503 GCA_000152825.2_ASM15282v2
100/100 Sagittula.stellata.E.37 GCA_000169415.1_ASM16941v1
100/100
Ruegeria.pomeroyi.DSS.3 GCA_000011965.2_ASM1196v2
100/100
89/94
Octadecabacter.arcticus.238 GCA_000155735.2_ASM15573v2
100/99
Jannaschia.rubra GCA_900113265.1.IMG.taxon.2622736430.annotated.assembly
100/100 Rubellimicrobium.rubrum GCA_006152115.1_ASM615211v1
100/100
Ketogulonicigenium.vulgare GCA_001399515.1_ASM139951v1
100/100
100/100 Rhodobacter.sphaeroides.ATCC.17029 GCA_000015985.1_ASM1598v1
Paracoccus.denitrificans GCA_004063735.1_ASM406373v1
Neomegalonema.perideroedes.DSM.15528 GCA_000374145.1_ASM37414v1
67.1/94 Chelativorans.oligotrophicus GCA_011317505.1_ASM1131750v1
39.7/77
Ahrensia.kielensis.DSM.5890 GCA_000374465.1_ASM37446v1
100/100
Rhizobium.leguminosarum.bv.trifolii GCA_011604525.1_ASM1160452v1
100/100
48.6/77
Bartonella.quintana GCA_009936175.1_ASM993617v1
Pelagibacterium.halotolerans GCA_013004005.1_ASM1300400v1
100/100
100/100 Roseibium.hamelinense GCA_007830545.1_ASM783054v1
100/100
Polymorphum.gilvum.SL003B.26A1 GCA_000192745.1_ASM19274v1
Pseudovibrio.sp.FO.BEG1 GCA_000236645.1_ASM23664v1
100/100
100/100 Methylocystis.bryophila GCA_002117405.1_ASM211740v1
100/100
Methylocella.silvestris.BL2 GCA_000021745.1_ASM2174v1
100/100
Methylorubrum.extorquens.PA1 GCA_000018845.1_ASM1884v1
98.7/99
100/100 Xanthobacter.autotrophicus.Py2 GCA_000017645.1_ASM1764v1
Rhodopseudomonas.palustris GCA_013415845.1_ASM1341584v1
100/100
100/100
100/100 Rhodomicrobium.vannielii.ATCC.17100 GCA_000166055.1_ASM16605v1
Hyphomicrobium.denitrificans.ATCC.51888 GCA_000143145.1_ASM14314v1
100/100
Thermopetrobacter.sp.TC1 GCA_000746275.1_ASM74627v1
100/100 alpha.proteobacterium.IMCC14465 GCA_000293845.2_ASM29384v2
100/100
Candidatus.Micropelagos.thuwalensis GCA_000469155.1.RS24
Parvibaculum.lavamentivorans.DS.1 GCA_000017565.1_ASM1756v1
100/100
100/100 alpha.proteobacterium.HIMB5 GCA_000299095.1_ASM29909v1
100/100
Candidatus.Pelagibacter.ubique.HTCC1062 GCA_000012345.1_ASM1234v1
100/100
Candidatus.Pelagibacter.ubique.HIMB058 GCA_000419545.1_ASM41954v1
alpha.proteobacterium.HIMB114 GCA_000163555.2
100/100
100/100 Sphingopyxis.macrogoltabida GCA_001314325.1_ASM131432v1
100/100
Citromicrobium.sp.JL477 GCA_001304795.1_ASM130479v1
100/100
Sphingobium.indicum.B90A GCA_000264945.2_ASM26494v2
100/100
100/100 Zymomonas.mobilis.subsp.mobilis.ATCC.31821 GCA_000007105.1_ASM710v1
100/100
Sphingomonas.wittichii.RW1 GCA_000016765.1_ASM1676v1
alpha.proteobacterium.AAP81b GCA_001295935.1_ASM129593v1
Kordiimonas.gwangyangensis.DSM.19435 GCA_000375545.1_ASM37554v1
Sneathiella.glossodoripedis.JCM.23214 GCA_000616095.1_ASM61609v1
Gluconobacter.oxydans.DSM.3504 GCA_000583855.1_ASM58385v1
91.5/99
Acetobacter.aceti.NBRC.14818 GCA_000193495.2_ASM19349v2
100/100
Gluconacetobacter.diazotrophicus.PA1.5 GCA_000067045.1_ASM6704v1
100/100
Commensalibacter.intestini.A911 GCA_000231445.2_ASM23144v1
92.9/99
68.6/95 Granulibacter.bethesdensis.CGDNIH4 GCA_000576145.2_ASM57614v2
Acidisphaera.rubrifaciens.HS.AP3 GCA_000964365.1_ASM96436v1
100/100
Acidiphilium.multivorum.AIU301 GCA_000202835.1_ASM20283v1
100/100 Rubritepida.flocculans.DSM.14296 GCA_000425365.1_ASM42536v1
100/100
100/100
Belnapia.moabensis.DSM.16746 GCA_000745835.1_ASM74583v1
Roseomonas.gilardii GCA_001941945.1_ASM194194v1
100/100
Elioraea.tepidiphila.DSM.17972 GCA_000378465.1_ASM37846v1
100/100 Candidatus.Paracaedibacter.acanthamoebae GCA_000742835.1_ASM74283v1
100/100
Candidatus.Odyssella.thessalonicensis.L13 GCA_000190415.2_ASM19041v1
47.8/55
Candidatus.Finniella.inopinata GCA_004210305.1_ASM421030v1
100/100 Holospora.undulata.HU1 GCA_000388175.3_ASM38817v3
100/100
Candidatus.Hepatobacter.penaei GCA_000742475.1_ASM74247v1
100/100 Candidatus.Caedibacter.acanthamoebae GCA_000743035.1_ASM74303v1
100/100
Caedimonas.varicaedens GCA_001192655.1_ASM119265v1
Candidatus.Nucleicultrix.amoebiphila.FS5 GCA_002117145.1_ASM211714v1
62.1/40
100/100 alpha.proteobacterium.BAL199 GCA_000171835.1_ASM17183v1
100/100
Thalassobaculum.salexigens.DSM.19539 GCA_000423805.1_ASM42380v1
100/100
Candidatus.Puniceispirillum.marinum.IMCC1322 GCA_000024465.1_ASM2446v1
100/100
Oceanibaculum.indicum GCA_003633955.1_ASM363395v1
100/100 Rhodovibrio.salinarum.DSM.9154 GCA_000515255.1_ASM51525v1
100/100
Kiloniella.laminariae.DSM.19542 GCA_000374005.1_ASM37400v1
100/100 Rhodospirillum.centenum GCA_014197805.1_ASM1419780v1
100/100
Inquilinus.limosus.DSM.16000 GCA_000423185.1_ASM42318v1
Micavibrio.aeruginosavorus.ARL.13 GCA_000226315.1_ASM22631v1
100/100 Rhodospirillum.rubrum.F11 GCA_000225955.1_ASM22595v1
100/55
100/29
93.2/100
Pararhodospirillum.photometricum.DSM.122 GCA_000284415.2_ASM28441v2
100/100
Roseospirillum.parvum GCA_900100455.1
100/100
Caenispirillum.salinarum.AK4 GCA_000315795.1
90.5/54
Magnetospirillum.magneticum.AMB.1 GCA_000009985.1_ASM998v1
GCA_900544565.1.UMGS690*
100/55
100/100 Thalassospira.profundimaris.WP0211 GCA_000300275.1_ASM30027v1
Terasakiella.pusilla.DSM.6293 GCA_000688235.1_ASM68823v1
100/100 Tistrella.mobilis.KA081020.065 GCA_000264455.2_ASM26445v2
Geminicoccus.roseus.DSM.18922 GCA_000427665.1_ASM42766v1
99.7/100 Candidatus.Neoehrlichia.lotoris.str.RAC413 GCA_000964795.1_ASM96479v1
99.9/55
100/100
Anaplasma.phagocytophilum.str.HZ GCA_000013125.1_ASM1312v1
100/100
Ehrlichia.ruminantium.str.Welgevonden GCA_000026005.1_ASM2600v1
98.6/98
Wolbachia.endosymbiont.of.Drosophila.melanogaster GCA_000008025.1_ASM802v1
99.8/55
Candidatus.Deianiraea.vastatrix GCA_007993655.1_ASM799365v1
95/96
100/100 Rickettsiales.endosymbiont.of.Trichoplax.sp.H2 GCA_009690945.1
100/100
Candidatus.Midichloria.mitochondrii.IricVA GCA_000219355.1_ASM21935v1
100/100 Rickettsiales.endosymbiont.of.Peranema.trichophorum GCA_004210275.1_ASM421027v1
Candidatus.Jidaibacter.acanthamoeba GCA_000815465.1.UWC36
100/100
100/100 Rickettsia.bellii.RML369.C GCA_000012385.1_ASM1238v1
55.3/88
Candidatus.Megaira.polyxenophila
100/100
Rickettsiales.bacterium.Ac37b GCA_000746585.2_ASM74658v2
Rickettsiales.endosymbiont.of.Stachyamoeba.lipophora GCA_003932735.1_ASM393273v1
100/100 GCA_900550565.1.UMGS1303*
100/100
GCA_900546225.1.UMGS860*
GCA_900546185.1.UMGS859*
100/100 Magnetofaba.australis.IT.1 GCA_002109495.1_ASM210949v1
Magnetococcus.marinus.MC.1 GCA_000014865.1_ASM1486v1
89.4/99 Nitrosospira.briensis.C.128 GCA_000619905.2_ASM61990v2
100/100
Methylotenera.versatilis.301 GCA_000093025.1_ASM9302v1
100/100 Ralstonia.pickettii GCA_902374465.1.MGYG.HGUT.01384
100/100
Burkholderia.cepacia GCA_009586235.1_ASM958623v1
100/100 Dasania.marina.DSM.21967 GCA_000373485.1_ASM37348v1
100/100
Congregibacter.litoralis.KT71 GCA_000153125.2_ASM15312v2
100/100 Enterobacter.cloacae GCA_000770155.1_ASM77015v1
Alteromonas.macleodii.ATCC.27126 GCA_000172635.2_ASM17263v2
0.566195

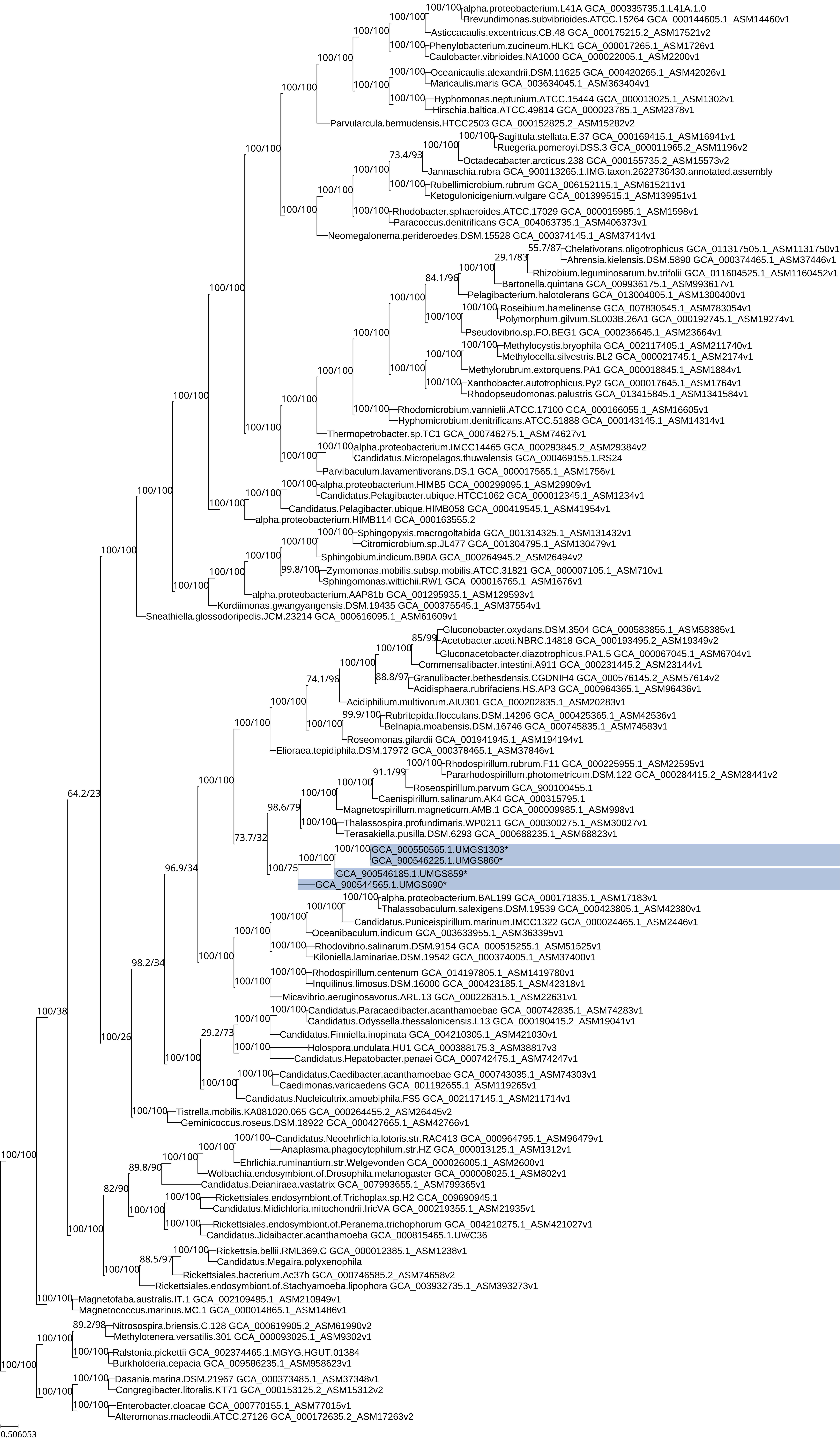
alpha.proteobacterium.L41A GCA_000335735.1.L41A.1.0
Brevundimonas.subvibrioides.ATCC.15264 GCA_000144605.1_ASM14460v1
Asticcacaulis.excentricus.CB.48 GCA_000175215.2_ASM17521v2
Phenylobacterium.zucineum.HLK1 GCA_000017265.1_ASM1726v1
Caulobacter.vibrioides.NA1000 GCA_000022005.1_ASM2200v1
Oceanicaulis.alexandrii.DSM.11625 GCA_000420265.1_ASM42026v1
Maricaulis.maris GCA_003634045.1_ASM363404v1
Hyphomonas.neptunium.ATCC.15444 GCA_000013025.1_ASM1302v1
Hirschia.baltica.ATCC.49814 GCA_000023785.1_ASM2378v1
Parvularcula.bermudensis.HTCC2503 GCA_000152825.2_ASM15282v2
Sagittula.stellata.E.37 GCA_000169415.1_ASM16941v1
Ruegeria.pomeroyi.DSS.3 GCA_000011965.2_ASM1196v2
Octadecabacter.arcticus.238 GCA_000155735.2_ASM15573v2
Jannaschia.rubra GCA_900113265.1.IMG.taxon.2622736430.annotated.assembly
Rubellimicrobium.rubrum GCA_006152115.1_ASM615211v1
Ketogulonicigenium.vulgare GCA_001399515.1_ASM139951v1
Rhodobacter.sphaeroides.ATCC.17029 GCA_000015985.1_ASM1598v1
Paracoccus.denitrificans GCA_004063735.1_ASM406373v1
Neomegalonema.perideroedes.DSM.15528 GCA_000374145.1_ASM37414v1
Chelativorans.oligotrophicus GCA_011317505.1_ASM1131750v1
Ahrensia.kielensis.DSM.5890 GCA_000374465.1_ASM37446v1
Rhizobium.leguminosarum.bv.trifolii GCA_011604525.1_ASM1160452v1
Bartonella.quintana GCA_009936175.1_ASM993617v1
Pelagibacterium.halotolerans GCA_013004005.1_ASM1300400v1
Roseibium.hamelinense GCA_007830545.1_ASM783054v1
Polymorphum.gilvum.SL003B.26A1 GCA_000192745.1_ASM19274v1
Pseudovibrio.sp.FO.BEG1 GCA_000236645.1_ASM23664v1
Methylocystis.bryophila GCA_002117405.1_ASM211740v1
Methylocella.silvestris.BL2 GCA_000021745.1_ASM2174v1
Methylorubrum.extorquens.PA1 GCA_000018845.1_ASM1884v1
Xanthobacter.autotrophicus.Py2 GCA_000017645.1_ASM1764v1
Rhodopseudomonas.palustris GCA_013415845.1_ASM1341584v1
Rhodomicrobium.vannielii.ATCC.17100 GCA_000166055.1_ASM16605v1
Hyphomicrobium.denitrificans.ATCC.51888 GCA_000143145.1_ASM14314v1
Thermopetrobacter.sp.TC1 GCA_000746275.1_ASM74627v1
alpha.proteobacterium.IMCC14465 GCA_000293845.2_ASM29384v2
Candidatus.Micropelagos.thuwalensis GCA_000469155.1.RS24
Parvibaculum.lavamentivorans.DS.1 GCA_000017565.1_ASM1756v1
alpha.proteobacterium.HIMB5 GCA_000299095.1_ASM29909v1
Candidatus.Pelagibacter.ubique.HTCC1062 GCA_000012345.1_ASM1234v1
Candidatus.Pelagibacter.ubique.HIMB058 GCA_000419545.1_ASM41954v1
alpha.proteobacterium.HIMB114 GCA_000163555.2
Sphingopyxis.macrogoltabida GCA_001314325.1_ASM131432v1
Citromicrobium.sp.JL477 GCA_001304795.1_ASM130479v1
Sphingobium.indicum.B90A GCA_000264945.2_ASM26494v2
Zymomonas.mobilis.subsp.mobilis.ATCC.31821 GCA_000007105.1_ASM710v1
Sphingomonas.wittichii.RW1 GCA_000016765.1_ASM1676v1
alpha.proteobacterium.AAP81b GCA_001295935.1_ASM129593v1
Kordiimonas.gwangyangensis.DSM.19435 GCA_000375545.1_ASM37554v1
Sneathiella.glossodoripedis.JCM.23214 GCA_000616095.1_ASM61609v1
Gluconobacter.oxydans.DSM.3504 GCA_000583855.1_ASM58385v1
Acetobacter.aceti.NBRC.14818 GCA_000193495.2_ASM19349v2
Gluconacetobacter.diazotrophicus.PA1.5 GCA_000067045.1_ASM6704v1
Commensalibacter.intestini.A911 GCA_000231445.2_ASM23144v1
Granulibacter.bethesdensis.CGDNIH4 GCA_000576145.2_ASM57614v2
Acidisphaera.rubrifaciens.HS.AP3 GCA_000964365.1_ASM96436v1
Acidiphilium.multivorum.AIU301 GCA_000202835.1_ASM20283v1
Rubritepida.flocculans.DSM.14296 GCA_000425365.1_ASM42536v1
Belnapia.moabensis.DSM.16746 GCA_000745835.1_ASM74583v1
Roseomonas.gilardii GCA_001941945.1_ASM194194v1
Elioraea.tepidiphila.DSM.17972 GCA_000378465.1_ASM37846v1
Rhodospirillum.rubrum.F11 GCA_000225955.1_ASM22595v1
Pararhodospirillum.photometricum.DSM.122 GCA_000284415.2_ASM28441v2
Roseospirillum.parvum GCA_900100455.1
Caenispirillum.salinarum.AK4 GCA_000315795.1
Magnetospirillum.magneticum.AMB.1 GCA_000009985.1_ASM998v1
Thalassospira.profundimaris.WP0211 GCA_000300275.1_ASM30027v1
Terasakiella.pusilla.DSM.6293 GCA_000688235.1_ASM68823v1
GCA_900550565.1.UMGS1303*
GCA_900546225.1.UMGS860*
GCA_900546185.1.UMGS859*
GCA_900544565.1.UMGS690*
alpha.proteobacterium.BAL199 GCA_000171835.1_ASM17183v1
Thalassobaculum.salexigens.DSM.19539 GCA_000423805.1_ASM42380v1
Candidatus.Puniceispirillum.marinum.IMCC1322 GCA_000024465.1_ASM2446v1
Oceanibaculum.indicum GCA_003633955.1_ASM363395v1
Rhodovibrio.salinarum.DSM.9154 GCA_000515255.1_ASM51525v1
Kiloniella.laminariae.DSM.19542 GCA_000374005.1_ASM37400v1
Rhodospirillum.centenum GCA_014197805.1_ASM1419780v1
Inquilinus.limosus.DSM.16000 GCA_000423185.1_ASM42318v1
Micavibrio.aeruginosavorus.ARL.13 GCA_000226315.1_ASM22631v1
Candidatus.Paracaedibacter.acanthamoebae GCA_000742835.1_ASM74283v1
Candidatus.Odyssella.thessalonicensis.L13 GCA_000190415.2_ASM19041v1
Candidatus.Finniella.inopinata GCA_004210305.1_ASM421030v1
Holospora.undulata.HU1 GCA_000388175.3_ASM38817v3
Candidatus.Hepatobacter.penaei GCA_000742475.1_ASM74247v1
Candidatus.Caedibacter.acanthamoebae GCA_000743035.1_ASM74303v1
Caedimonas.varicaedens GCA_001192655.1_ASM119265v1
Candidatus.Nucleicultrix.amoebiphila.FS5 GCA_002117145.1_ASM211714v1
Tistrella.mobilis.KA081020.065 GCA_000264455.2_ASM26445v2
Geminicoccus.roseus.DSM.18922 GCA_000427665.1_ASM42766v1
Candidatus.Neoehrlichia.lotoris.str.RAC413 GCA_000964795.1_ASM96479v1
Anaplasma.phagocytophilum.str.HZ GCA_000013125.1_ASM1312v1
Ehrlichia.ruminantium.str.Welgevonden GCA_000026005.1_ASM2600v1
Wolbachia.endosymbiont.of.Drosophila.melanogaster GCA_000008025.1_ASM802v1
Candidatus.Deianiraea.vastatrix GCA_007993655.1_ASM799365v1
Rickettsiales.endosymbiont.of.Trichoplax.sp.H2 GCA_009690945.1
Candidatus.Midichloria.mitochondrii.IricVA GCA_000219355.1_ASM21935v1
Rickettsiales.endosymbiont.of.Peranema.trichophorum GCA_004210275.1_ASM421027v1
Candidatus.Jidaibacter.acanthamoeba GCA_000815465.1.UWC36
Rickettsia.bellii.RML369.C GCA_000012385.1_ASM1238v1
Candidatus.Megaira.polyxenophila
Rickettsiales.bacterium.Ac37b GCA_000746585.2_ASM74658v2
Rickettsiales.endosymbiont.of.Stachyamoeba.lipophora GCA_003932735.1_ASM393273v1
Magnetofaba.australis.IT.1 GCA_002109495.1_ASM210949v1
Magnetococcus.marinus.MC.1 GCA_000014865.1_ASM1486v1
Nitrosospira.briensis.C.128 GCA_000619905.2_ASM61990v2
Methylotenera.versatilis.301 GCA_000093025.1_ASM9302v1
Ralstonia.pickettii GCA_902374465.1.MGYG.HGUT.01384
Burkholderia.cepacia GCA_009586235.1_ASM958623v1
Dasania.marina.DSM.21967 GCA_000373485.1_ASM37348v1
Congregibacter.litoralis.KT71 GCA_000153125.2_ASM15312v2
Enterobacter.cloacae GCA_000770155.1_ASM77015v1
Alteromonas.macleodii.ATCC.27126 GCA_000172635.2_ASM17263v2
0.506053

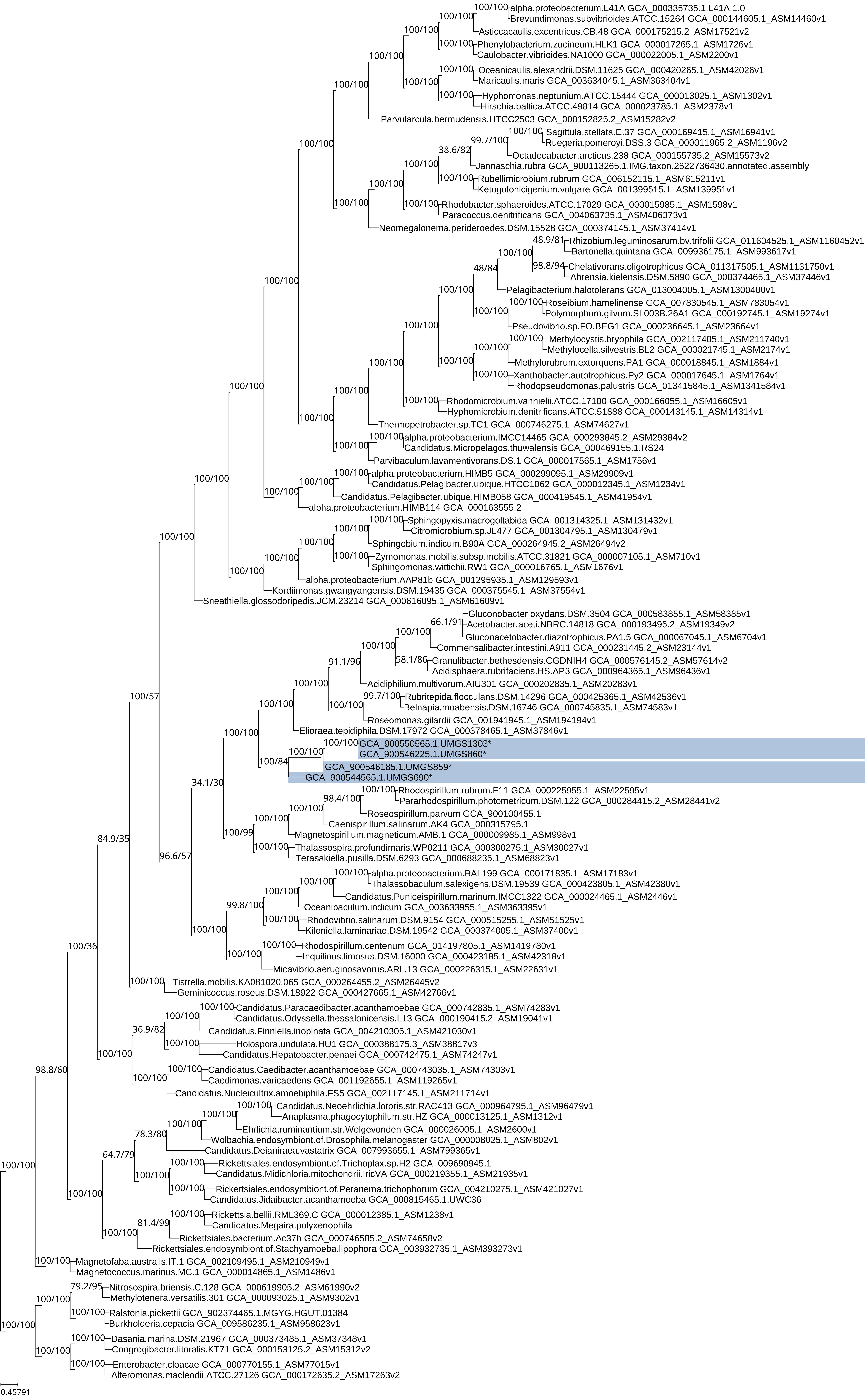
alpha.proteobacterium.L41A GCA_000335735.1.L41A.1.0
Brevundimonas.subvibrioides.ATCC.15264 GCA_000144605.1_ASM14460v1
Asticcacaulis.excentricus.CB.48 GCA_000175215.2_ASM17521v2
Phenylobacterium.zucineum.HLK1 GCA_000017265.1_ASM1726v1
Caulobacter.vibrioides.NA1000 GCA_000022005.1_ASM2200v1
Oceanicaulis.alexandrii.DSM.11625 GCA_000420265.1_ASM42026v1
Maricaulis.maris GCA_003634045.1_ASM363404v1
Hyphomonas.neptunium.ATCC.15444 GCA_000013025.1_ASM1302v1
Hirschia.baltica.ATCC.49814 GCA_000023785.1_ASM2378v1
Parvularcula.bermudensis.HTCC2503 GCA_000152825.2_ASM15282v2
Sagittula.stellata.E.37 GCA_000169415.1_ASM16941v1
Ruegeria.pomeroyi.DSS.3 GCA_000011965.2_ASM1196v2
Octadecabacter.arcticus.238 GCA_000155735.2_ASM15573v2
Jannaschia.rubra GCA_900113265.1.IMG.taxon.2622736430.annotated.assembly
Rubellimicrobium.rubrum GCA_006152115.1_ASM615211v1
Ketogulonicigenium.vulgare GCA_001399515.1_ASM139951v1
Rhodobacter.sphaeroides.ATCC.17029 GCA_000015985.1_ASM1598v1
Paracoccus.denitrificans GCA_004063735.1_ASM406373v1
Neomegalonema.perideroedes.DSM.15528 GCA_000374145.1_ASM37414v1
Rhizobium.leguminosarum.bv.trifolii GCA_011604525.1_ASM1160452v1
Bartonella.quintana GCA_009936175.1_ASM993617v1
Chelativorans.oligotrophicus GCA_011317505.1_ASM1131750v1
Ahrensia.kielensis.DSM.5890 GCA_000374465.1_ASM37446v1
Pelagibacterium.halotolerans GCA_013004005.1_ASM1300400v1
Roseibium.hamelinense GCA_007830545.1_ASM783054v1
Polymorphum.gilvum.SL003B.26A1 GCA_000192745.1_ASM19274v1
Pseudovibrio.sp.FO.BEG1 GCA_000236645.1_ASM23664v1
Methylocystis.bryophila GCA_002117405.1_ASM211740v1
Methylocella.silvestris.BL2 GCA_000021745.1_ASM2174v1
Methylorubrum.extorquens.PA1 GCA_000018845.1_ASM1884v1
Xanthobacter.autotrophicus.Py2 GCA_000017645.1_ASM1764v1
Rhodopseudomonas.palustris GCA_013415845.1_ASM1341584v1
Rhodomicrobium.vannielii.ATCC.17100 GCA_000166055.1_ASM16605v1
Hyphomicrobium.denitrificans.ATCC.51888 GCA_000143145.1_ASM14314v1
Thermopetrobacter.sp.TC1 GCA_000746275.1_ASM74627v1
alpha.proteobacterium.IMCC14465 GCA_000293845.2_ASM29384v2
Candidatus.Micropelagos.thuwalensis GCA_000469155.1.RS24
Parvibaculum.lavamentivorans.DS.1 GCA_000017565.1_ASM1756v1
alpha.proteobacterium.HIMB5 GCA_000299095.1_ASM29909v1
Candidatus.Pelagibacter.ubique.HTCC1062 GCA_000012345.1_ASM1234v1
Candidatus.Pelagibacter.ubique.HIMB058 GCA_000419545.1_ASM41954v1
alpha.proteobacterium.HIMB114 GCA_000163555.2
Sphingopyxis.macrogoltabida GCA_001314325.1_ASM131432v1
Citromicrobium.sp.JL477 GCA_001304795.1_ASM130479v1
Sphingobium.indicum.B90A GCA_000264945.2_ASM26494v2
Zymomonas.mobilis.subsp.mobilis.ATCC.31821 GCA_000007105.1_ASM710v1
Sphingomonas.wittichii.RW1 GCA_000016765.1_ASM1676v1
alpha.proteobacterium.AAP81b GCA_001295935.1_ASM129593v1
Kordiimonas.gwangyangensis.DSM.19435 GCA_000375545.1_ASM37554v1
Sneathiella.glossodoripedis.JCM.23214 GCA_000616095.1_ASM61609v1
Gluconobacter.oxydans.DSM.3504 GCA_000583855.1_ASM58385v1
Acetobacter.aceti.NBRC.14818 GCA_000193495.2_ASM19349v2
Gluconacetobacter.diazotrophicus.PA1.5 GCA_000067045.1_ASM6704v1
Commensalibacter.intestini.A911 GCA_000231445.2_ASM23144v1
Granulibacter.bethesdensis.CGDNIH4 GCA_000576145.2_ASM57614v2
Acidisphaera.rubrifaciens.HS.AP3 GCA_000964365.1_ASM96436v1
Acidiphilium.multivorum.AIU301 GCA_000202835.1_ASM20283v1
Rubritepida.flocculans.DSM.14296 GCA_000425365.1_ASM42536v1
Belnapia.moabensis.DSM.16746 GCA_000745835.1_ASM74583v1
Roseomonas.gilardii GCA_001941945.1_ASM194194v1
Elioraea.tepidiphila.DSM.17972 GCA_000378465.1_ASM37846v1
GCA_900550565.1.UMGS1303*
GCA_900546225.1.UMGS860*
GCA_900546185.1.UMGS859*
GCA_900544565.1.UMGS690*
Rhodospirillum.rubrum.F11 GCA_000225955.1_ASM22595v1
Pararhodospirillum.photometricum.DSM.122 GCA_000284415.2_ASM28441v2
Roseospirillum.parvum GCA_900100455.1
Caenispirillum.salinarum.AK4 GCA_000315795.1
Magnetospirillum.magneticum.AMB.1 GCA_000009985.1_ASM998v1
Thalassospira.profundimaris.WP0211 GCA_000300275.1_ASM30027v1
Terasakiella.pusilla.DSM.6293 GCA_000688235.1_ASM68823v1
alpha.proteobacterium.BAL199 GCA_000171835.1_ASM17183v1
Thalassobaculum.salexigens.DSM.19539 GCA_000423805.1_ASM42380v1
Candidatus.Puniceispirillum.marinum.IMCC1322 GCA_000024465.1_ASM2446v1
Oceanibaculum.indicum GCA_003633955.1_ASM363395v1
Rhodovibrio.salinarum.DSM.9154 GCA_000515255.1_ASM51525v1
Kiloniella.laminariae.DSM.19542 GCA_000374005.1_ASM37400v1
Rhodospirillum.centenum GCA_014197805.1_ASM1419780v1
Inquilinus.limosus.DSM.16000 GCA_000423185.1_ASM42318v1
Micavibrio.aeruginosavorus.ARL.13 GCA_000226315.1_ASM22631v1
Tistrella.mobilis.KA081020.065 GCA_000264455.2_ASM26445v2
Geminicoccus.roseus.DSM.18922 GCA_000427665.1_ASM42766v1
Candidatus.Paracaedibacter.acanthamoebae GCA_000742835.1_ASM74283v1
Candidatus.Odyssella.thessalonicensis.L13 GCA_000190415.2_ASM19041v1
Candidatus.Finniella.inopinata GCA_004210305.1_ASM421030v1
Holospora.undulata.HU1 GCA_000388175.3_ASM38817v3
Candidatus.Hepatobacter.penaei GCA_000742475.1_ASM74247v1
Candidatus.Caedibacter.acanthamoebae GCA_000743035.1_ASM74303v1
Caedimonas.varicaedens GCA_001192655.1_ASM119265v1
Candidatus.Nucleicultrix.amoebiphila.FS5 GCA_002117145.1_ASM211714v1
Candidatus.Neoehrlichia.lotoris.str.RAC413 GCA_000964795.1_ASM96479v1
Anaplasma.phagocytophilum.str.HZ GCA_000013125.1_ASM1312v1
Ehrlichia.ruminantium.str.Welgevonden GCA_000026005.1_ASM2600v1
Wolbachia.endosymbiont.of.Drosophila.melanogaster GCA_000008025.1_ASM802v1
Candidatus.Deianiraea.vastatrix GCA_007993655.1_ASM799365v1
Rickettsiales.endosymbiont.of.Trichoplax.sp.H2 GCA_009690945.1
Candidatus.Midichloria.mitochondrii.IricVA GCA_000219355.1_ASM21935v1
Rickettsiales.endosymbiont.of.Peranema.trichophorum GCA_004210275.1_ASM421027v1
Candidatus.Jidaibacter.acanthamoeba GCA_000815465.1.UWC36
Rickettsia.bellii.RML369.C GCA_000012385.1_ASM1238v1
Candidatus.Megaira.polyxenophila
Rickettsiales.bacterium.Ac37b GCA_000746585.2_ASM74658v2
Rickettsiales.endosymbiont.of.Stachyamoeba.lipophora GCA_003932735.1_ASM393273v1
Magnetofaba.australis.IT.1 GCA_002109495.1_ASM210949v1
Magnetococcus.marinus.MC.1 GCA_000014865.1_ASM1486v1
Nitrosospira.briensis.C.128 GCA_000619905.2_ASM61990v2
Methylotenera.versatilis.301 GCA_000093025.1_ASM9302v1
Ralstonia.pickettii GCA_902374465.1.MGYG.HGUT.01384
Burkholderia.cepacia GCA_009586235.1_ASM958623v1
Dasania.marina.DSM.21967 GCA_000373485.1_ASM37348v1
Congregibacter.litoralis.KT71 GCA_000153125.2_ASM15312v2
Enterobacter.cloacae GCA_000770155.1_ASM77015v1
Alteromonas.macleodii.ATCC.27126 GCA_000172635.2_ASM17263v2
0.45791

Group J

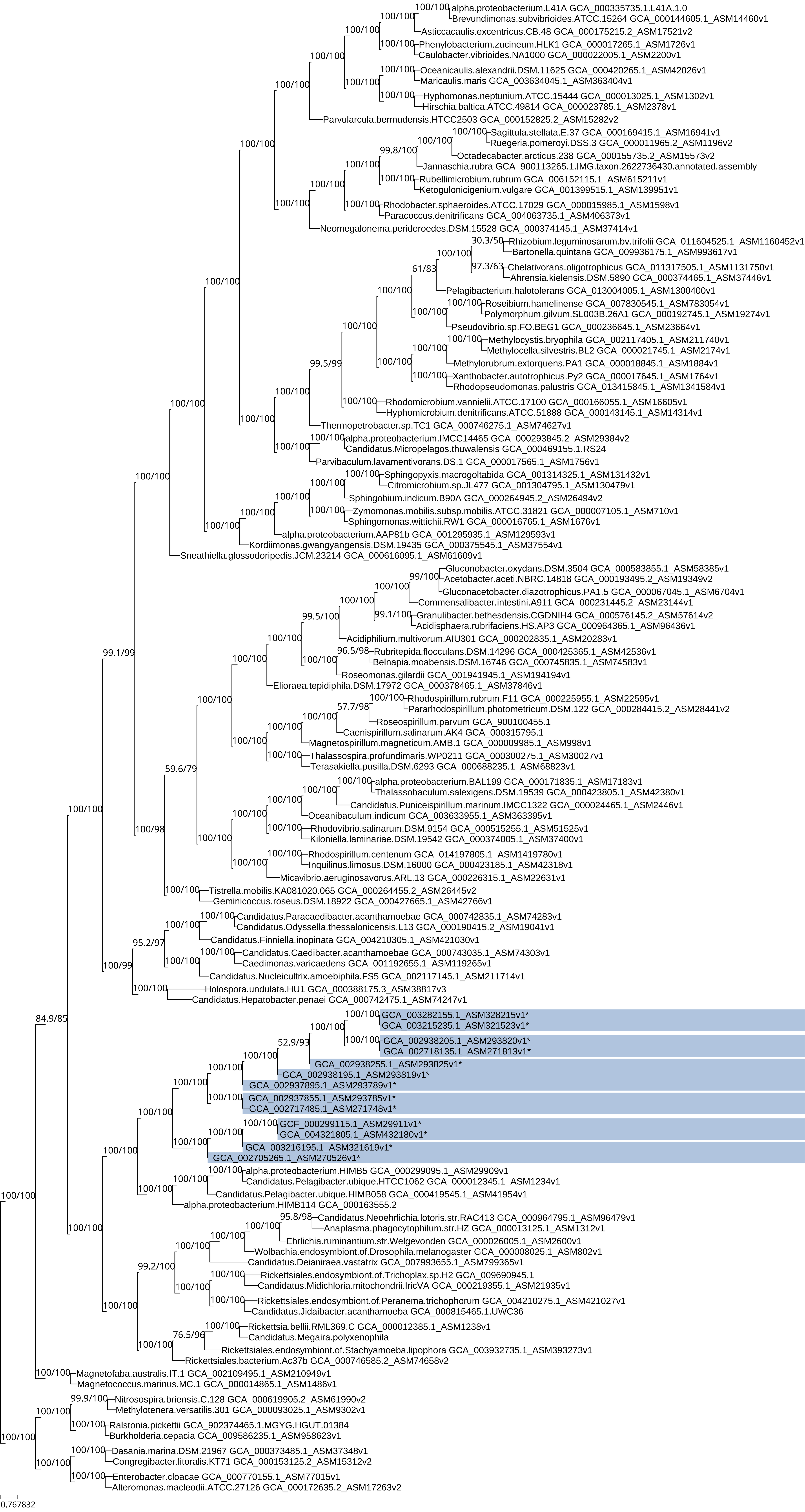
alpha.proteobacterium.L41A GCA_000335735.1.L41A.1.0
Brevundimonas.subvibrioides.ATCC.15264 GCA_000144605.1_ASM14460v1
Asticcacaulis.excentricus.CB.48 GCA_000175215.2_ASM17521v2
Phenylobacterium.zucineum.HLK1 GCA_000017265.1_ASM1726v1
Caulobacter.vibrioides.NA1000 GCA_000022005.1_ASM2200v1
Oceanicaulis.alexandrii.DSM.11625 GCA_000420265.1_ASM42026v1
Maricaulis.maris GCA_003634045.1_ASM363404v1
Hyphomonas.neptunium.ATCC.15444 GCA_000013025.1_ASM1302v1
Hirschia.baltica.ATCC.49814 GCA_000023785.1_ASM2378v1
Parvularcula.bermudensis.HTCC2503 GCA_000152825.2_ASM15282v2
Sagittula.stellata.E.37 GCA_000169415.1_ASM16941v1
Ruegeria.pomeroyi.DSS.3 GCA_000011965.2_ASM1196v2
Octadecabacter.arcticus.238 GCA_000155735.2_ASM15573v2
Jannaschia.rubra GCA_900113265.1.IMG.taxon.2622736430.annotated.assembly
Rubellimicrobium.rubrum GCA_006152115.1_ASM615211v1
Ketogulonicigenium.vulgare GCA_001399515.1_ASM139951v1
Rhodobacter.sphaeroides.ATCC.17029 GCA_000015985.1_ASM1598v1
Paracoccus.denitrificans GCA_004063735.1_ASM406373v1
Neomegalonema.perideroedes.DSM.15528 GCA_000374145.1_ASM37414v1
Rhizobium.leguminosarum.bv.trifolii GCA_011604525.1_ASM1160452v1
Bartonella.quintana GCA_009936175.1_ASM993617v1
Chelativorans.oligotrophicus GCA_011317505.1_ASM1131750v1
Ahrensia.kielensis.DSM.5890 GCA_000374465.1_ASM37446v1
Pelagibacterium.halotolerans GCA_013004005.1_ASM1300400v1
Roseibium.hamelinense GCA_007830545.1_ASM783054v1
Polymorphum.gilvum.SL003B.26A1 GCA_000192745.1_ASM19274v1
Pseudovibrio.sp.FO.BEG1 GCA_000236645.1_ASM23664v1
Methylocystis.bryophila GCA_002117405.1_ASM211740v1
Methylocella.silvestris.BL2 GCA_000021745.1_ASM2174v1
Methylorubrum.extorquens.PA1 GCA_000018845.1_ASM1884v1
Xanthobacter.autotrophicus.Py2 GCA_000017645.1_ASM1764v1
Rhodopseudomonas.palustris GCA_013415845.1_ASM1341584v1
Rhodomicrobium.vannielii.ATCC.17100 GCA_000166055.1_ASM16605v1
Hyphomicrobium.denitrificans.ATCC.51888 GCA_000143145.1_ASM14314v1
Thermopetrobacter.sp.TC1 GCA_000746275.1_ASM74627v1
alpha.proteobacterium.IMCC14465 GCA_000293845.2_ASM29384v2
Candidatus.Micropelagos.thuwalensis GCA_000469155.1.RS24
Parvibaculum.lavamentivorans.DS.1 GCA_000017565.1_ASM1756v1
Sphingopyxis.macrogoltabida GCA_001314325.1_ASM131432v1
Citromicrobium.sp.JL477 GCA_001304795.1_ASM130479v1
Sphingobium.indicum.B90A GCA_000264945.2_ASM26494v2
Zymomonas.mobilis.subsp.mobilis.ATCC.31821 GCA_000007105.1_ASM710v1
Sphingomonas.wittichii.RW1 GCA_000016765.1_ASM1676v1
alpha.proteobacterium.AAP81b GCA_001295935.1_ASM129593v1
Kordiimonas.gwangyangensis.DSM.19435 GCA_000375545.1_ASM37554v1
Sneathiella.glossodoripedis.JCM.23214 GCA_000616095.1_ASM61609v1
Gluconobacter.oxydans.DSM.3504 GCA_000583855.1_ASM58385v1
Acetobacter.aceti.NBRC.14818 GCA_000193495.2_ASM19349v2
Gluconacetobacter.diazotrophicus.PA1.5 GCA_000067045.1_ASM6704v1
Commensalibacter.intestini.A911 GCA_000231445.2_ASM23144v1
Granulibacter.bethesdensis.CGDNIH4 GCA_000576145.2_ASM57614v2
Acidisphaera.rubrifaciens.HS.AP3 GCA_000964365.1_ASM96436v1
Acidiphilium.multivorum.AIU301 GCA_000202835.1_ASM20283v1
Rubritepida.flocculans.DSM.14296 GCA_000425365.1_ASM42536v1
Belnapia.moabensis.DSM.16746 GCA_000745835.1_ASM74583v1
Roseomonas.gilardii GCA_001941945.1_ASM194194v1
Elioraea.tepidiphila.DSM.17972 GCA_000378465.1_ASM37846v1
Rhodospirillum.rubrum.F11 GCA_000225955.1_ASM22595v1
Pararhodospirillum.photometricum.DSM.122 GCA_000284415.2_ASM28441v2
Roseospirillum.parvum GCA_900100455.1
Caenispirillum.salinarum.AK4 GCA_000315795.1
Magnetospirillum.magneticum.AMB.1 GCA_000009985.1_ASM998v1
Thalassospira.profundimaris.WP0211 GCA_000300275.1_ASM30027v1
Terasakiella.pusilla.DSM.6293 GCA_000688235.1_ASM68823v1
alpha.proteobacterium.BAL199 GCA_000171835.1_ASM17183v1
Thalassobaculum.salexigens.DSM.19539 GCA_000423805.1_ASM42380v1
Candidatus.Puniceispirillum.marinum.IMCC1322 GCA_000024465.1_ASM2446v1
Oceanibaculum.indicum GCA_003633955.1_ASM363395v1
Rhodovibrio.salinarum.DSM.9154 GCA_000515255.1_ASM51525v1
Kiloniella.laminariae.DSM.19542 GCA_000374005.1_ASM37400v1
Rhodospirillum.centenum GCA_014197805.1_ASM1419780v1
Inquilinus.limosus.DSM.16000 GCA_000423185.1_ASM42318v1
Micavibrio.aeruginosavorus.ARL.13 GCA_000226315.1_ASM22631v1
Tistrella.mobilis.KA081020.065 GCA_000264455.2_ASM26445v2
Geminicoccus.roseus.DSM.18922 GCA_000427665.1_ASM42766v1
Candidatus.Paracaedibacter.acanthamoebae GCA_000742835.1_ASM74283v1
Candidatus.Odyssella.thessalonicensis.L13 GCA_000190415.2_ASM19041v1
Candidatus.Finniella.inopinata GCA_004210305.1_ASM421030v1
Candidatus.Caedibacter.acanthamoebae GCA_000743035.1_ASM74303v1
Caedimonas.varicaedens GCA_001192655.1_ASM119265v1
Candidatus.Nucleicultrix.amoebiphila.FS5 GCA_002117145.1_ASM211714v1
Holospora.undulata.HU1 GCA_000388175.3_ASM38817v3
Candidatus.Hepatobacter.penaei GCA_000742475.1_ASM74247v1
GCA_003282155.1_ASM328215v1*
GCA_003215235.1_ASM321523v1*
GCA_002938205.1_ASM293820v1*
GCA_002718135.1_ASM271813v1*
GCA_002938255.1_ASM293825v1*
GCA_002938195.1_ASM293819v1*
GCA_002937895.1_ASM293789v1*
GCA_002937855.1_ASM293785v1*
GCA_002717485.1_ASM271748v1*
GCF_000299115.1_ASM29911v1*
GCA_004321805.1_ASM432180v1*
GCA_003216195.1_ASM321619v1*
GCA_002705265.1_ASM270526v1*
alpha.proteobacterium.HIMB5 GCA_000299095.1_ASM29909v1
Candidatus.Pelagibacter.ubique.HTCC1062 GCA_000012345.1_ASM1234v1
Candidatus.Pelagibacter.ubique.HIMB058 GCA_000419545.1_ASM41954v1
alpha.proteobacterium.HIMB114 GCA_000163555.2
Candidatus.Neoehrlichia.lotoris.str.RAC413 GCA_000964795.1_ASM96479v1
Anaplasma.phagocytophilum.str.HZ GCA_000013125.1_ASM1312v1
Ehrlichia.ruminantium.str.Welgevonden GCA_000026005.1_ASM2600v1
Wolbachia.endosymbiont.of.Drosophila.melanogaster GCA_000008025.1_ASM802v1
Candidatus.Deianiraea.vastatrix GCA_007993655.1_ASM799365v1
Rickettsiales.endosymbiont.of.Trichoplax.sp.H2 GCA_009690945.1
Candidatus.Midichloria.mitochondrii.IricVA GCA_000219355.1_ASM21935v1
Rickettsiales.endosymbiont.of.Peranema.trichophorum GCA_004210275.1_ASM421027v1
Candidatus.Jidaibacter.acanthamoeba GCA_000815465.1.UWC36
Rickettsia.bellii.RML369.C GCA_000012385.1_ASM1238v1
Candidatus.Megaira.polyxenophila
Rickettsiales.endosymbiont.of.Stachyamoeba.lipophora GCA_003932735.1_ASM393273v1
Rickettsiales.bacterium.Ac37b GCA_000746585.2_ASM74658v2
Magnetofaba.australis.IT.1 GCA_002109495.1_ASM210949v1
Magnetococcus.marinus.MC.1 GCA_000014865.1_ASM1486v1
Nitrosospira.briensis.C.128 GCA_000619905.2_ASM61990v2
Methylotenera.versatilis.301 GCA_000093025.1_ASM9302v1
Ralstonia.pickettii GCA_902374465.1.MGYG.HGUT.01384
Burkholderia.cepacia GCA_009586235.1_ASM958623v1
Dasania.marina.DSM.21967 GCA_000373485.1_ASM37348v1
Congregibacter.litoralis.KT71 GCA_000153125.2_ASM15312v2
Enterobacter.cloacae GCA_000770155.1_ASM77015v1
Alteromonas.macleodii.ATCC.27126 GCA_000172635.2_ASM17263v2
0.767832

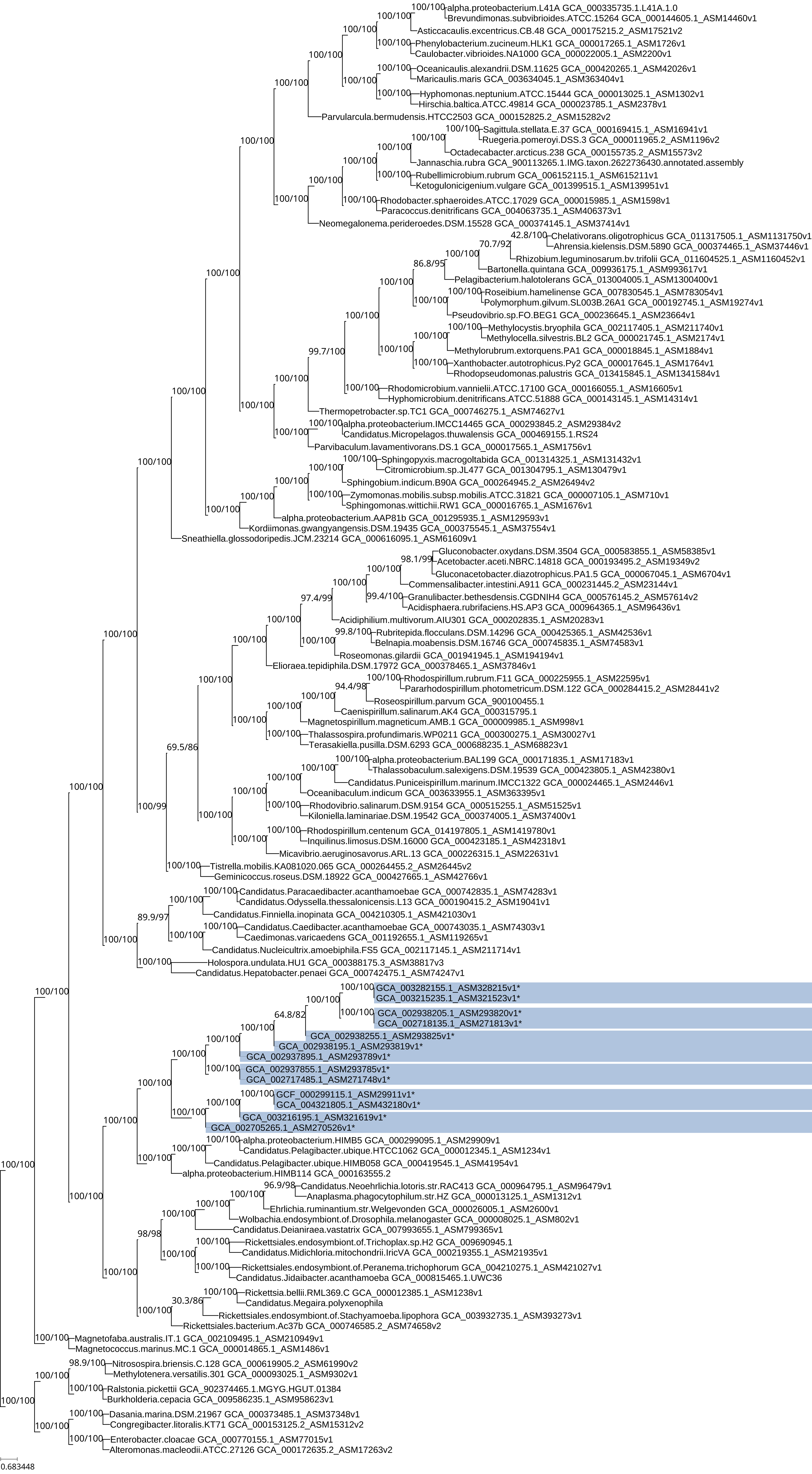

alpha.proteobacterium.L41A GCA_000335735.1.L41A.1.0
Brevundimonas.subvibrioides.ATCC.15264 GCA_000144605.1_ASM14460v1
Asticcacaulis.excentricus.CB.48 GCA_000175215.2_ASM17521v2
Phenylobacterium.zucineum.HLK1 GCA_000017265.1_ASM1726v1
Caulobacter.vibrioides.NA1000 GCA_000022005.1_ASM2200v1
Oceanicaulis.alexandrii.DSM.11625 GCA_000420265.1_ASM42026v1
Maricaulis.maris GCA_003634045.1_ASM363404v1
Hyphomonas.neptunium.ATCC.15444 GCA_000013025.1_ASM1302v1
Hirschia.baltica.ATCC.49814 GCA_000023785.1_ASM2378v1
Parvularcula.bermudensis.HTCC2503 GCA_000152825.2_ASM15282v2
Sagittula.stellata.E.37 GCA_000169415.1_ASM16941v1
Ruegeria.pomeroyi.DSS.3 GCA_000011965.2_ASM1196v2
Octadecabacter.arcticus.238 GCA_000155735.2_ASM15573v2
Jannaschia.rubra GCA_900113265.1.IMG.taxon.2622736430.annotated.assembly
Rubellimicrobium.rubrum GCA_006152115.1_ASM615211v1
Ketogulonicigenium.vulgare GCA_001399515.1_ASM139951v1
Rhodobacter.sphaeroides.ATCC.17029 GCA_000015985.1_ASM1598v1
Paracoccus.denitrificans GCA_004063735.1_ASM406373v1
Neomegalonema.perideroedes.DSM.15528 GCA_000374145.1_ASM37414v1
Chelativorans.oligotrophicus GCA_011317505.1_ASM1131750v1
Ahrensia.kielensis.DSM.5890 GCA_000374465.1_ASM37446v1
Rhizobium.leguminosarum.bv.trifolii GCA_011604525.1_ASM1160452v1
Bartonella.quintana GCA_009936175.1_ASM993617v1
Pelagibacterium.halotolerans GCA_013004005.1_ASM1300400v1
Roseibium.hamelinense GCA_007830545.1_ASM783054v1
Polymorphum.gilvum.SL003B.26A1 GCA_000192745.1_ASM19274v1
Pseudovibrio.sp.FO.BEG1 GCA_000236645.1_ASM23664v1
Methylocystis.bryophila GCA_002117405.1_ASM211740v1
Methylocella.silvestris.BL2 GCA_000021745.1_ASM2174v1
Methylorubrum.extorquens.PA1 GCA_000018845.1_ASM1884v1
Xanthobacter.autotrophicus.Py2 GCA_000017645.1_ASM1764v1
Rhodopseudomonas.palustris GCA_013415845.1_ASM1341584v1
Rhodomicrobium.vannielii.ATCC.17100 GCA_000166055.1_ASM16605v1
Hyphomicrobium.denitrificans.ATCC.51888 GCA_000143145.1_ASM14314v1
Thermopetrobacter.sp.TC1 GCA_000746275.1_ASM74627v1
alpha.proteobacterium.IMCC14465 GCA_000293845.2_ASM29384v2
Candidatus.Micropelagos.thuwalensis GCA_000469155.1.RS24
Parvibaculum.lavamentivorans.DS.1 GCA_000017565.1_ASM1756v1
Sphingopyxis.macrogoltabida GCA_001314325.1_ASM131432v1
Citromicrobium.sp.JL477 GCA_001304795.1_ASM130479v1
Sphingobium.indicum.B90A GCA_000264945.2_ASM26494v2
Zymomonas.mobilis.subsp.mobilis.ATCC.31821 GCA_000007105.1_ASM710v1
Sphingomonas.wittichii.RW1 GCA_000016765.1_ASM1676v1
alpha.proteobacterium.AAP81b GCA_001295935.1_ASM129593v1
Kordiimonas.gwangyangensis.DSM.19435 GCA_000375545.1_ASM37554v1
Sneathiella.glossodoripedis.JCM.23214 GCA_000616095.1_ASM61609v1
Gluconobacter.oxydans.DSM.3504 GCA_000583855.1_ASM58385v1
Acetobacter.aceti.NBRC.14818 GCA_000193495.2_ASM19349v2
Gluconacetobacter.diazotrophicus.PA1.5 GCA_000067045.1_ASM6704v1
Commensalibacter.intestini.A911 GCA_000231445.2_ASM23144v1
Granulibacter.bethesdensis.CGDNIH4 GCA_000576145.2_ASM57614v2
Acidisphaera.rubrifaciens.HS.AP3 GCA_000964365.1_ASM96436v1
Acidiphilium.multivorum.AIU301 GCA_000202835.1_ASM20283v1
Rubritepida.flocculans.DSM.14296 GCA_000425365.1_ASM42536v1
Belnapia.moabensis.DSM.16746 GCA_000745835.1_ASM74583v1
Roseomonas.gilardii GCA_001941945.1_ASM194194v1
Elioraea.tepidiphila.DSM.17972 GCA_000378465.1_ASM37846v1
Rhodospirillum.rubrum.F11 GCA_000225955.1_ASM22595v1
Pararhodospirillum.photometricum.DSM.122 GCA_000284415.2_ASM28441v2
Roseospirillum.parvum GCA_900100455.1
Caenispirillum.salinarum.AK4 GCA_000315795.1
Magnetospirillum.magneticum.AMB.1 GCA_000009985.1_ASM998v1
Thalassospira.profundimaris.WP0211 GCA_000300275.1_ASM30027v1
Terasakiella.pusilla.DSM.6293 GCA_000688235.1_ASM68823v1
alpha.proteobacterium.BAL199 GCA_000171835.1_ASM17183v1
Thalassobaculum.salexigens.DSM.19539 GCA_000423805.1_ASM42380v1
Candidatus.Puniceispirillum.marinum.IMCC1322 GCA_000024465.1_ASM2446v1
Oceanibaculum.indicum GCA_003633955.1_ASM363395v1
Rhodovibrio.salinarum.DSM.9154 GCA_000515255.1_ASM51525v1
Kiloniella.laminariae.DSM.19542 GCA_000374005.1_ASM37400v1
Rhodospirillum.centenum GCA_014197805.1_ASM1419780v1
Inquilinus.limosus.DSM.16000 GCA_000423185.1_ASM42318v1
Micavibrio.aeruginosavorus.ARL.13 GCA_000226315.1_ASM22631v1
Tistrella.mobilis.KA081020.065 GCA_000264455.2_ASM26445v2
Geminicoccus.roseus.DSM.18922 GCA_000427665.1_ASM42766v1
Candidatus.Paracaedibacter.acanthamoebae GCA_000742835.1_ASM74283v1
Candidatus.Odyssella.thessalonicensis.L13 GCA_000190415.2_ASM19041v1
Candidatus.Finniella.inopinata GCA_004210305.1_ASM421030v1
Candidatus.Caedibacter.acanthamoebae GCA_000743035.1_ASM74303v1
Caedimonas.varicaedens GCA_001192655.1_ASM119265v1
Candidatus.Nucleicultrix.amoebiphila.FS5 GCA_002117145.1_ASM211714v1
Holospora.undulata.HU1 GCA_000388175.3_ASM38817v3
Candidatus.Hepatobacter.penaei GCA_000742475.1_ASM74247v1
GCA_003282155.1_ASM328215v1*
GCA_003215235.1_ASM321523v1*
GCA_002938205.1_ASM293820v1*
GCA_002718135.1_ASM271813v1*
GCA_002938255.1_ASM293825v1*
GCA_002938195.1_ASM293819v1*
GCA_002937895.1_ASM293789v1*
GCA_002937855.1_ASM293785v1*
GCA_002717485.1_ASM271748v1*
GCF_000299115.1_ASM29911v1*
GCA_004321805.1_ASM432180v1*
GCA_003216195.1_ASM321619v1*
GCA_002705265.1_ASM270526v1*
alpha.proteobacterium.HIMB5 GCA_000299095.1_ASM29909v1
Candidatus.Pelagibacter.ubique.HTCC1062 GCA_000012345.1_ASM1234v1
Candidatus.Pelagibacter.ubique.HIMB058 GCA_000419545.1_ASM41954v1
alpha.proteobacterium.HIMB114 GCA_000163555.2
Candidatus.Neoehrlichia.lotoris.str.RAC413 GCA_000964795.1_ASM96479v1
Anaplasma.phagocytophilum.str.HZ GCA_000013125.1_ASM1312v1
Ehrlichia.ruminantium.str.Welgevonden GCA_000026005.1_ASM2600v1
Wolbachia.endosymbiont.of.Drosophila.melanogaster GCA_000008025.1_ASM802v1
Candidatus.Deianiraea.vastatrix GCA_007993655.1_ASM799365v1
Rickettsiales.endosymbiont.of.Trichoplax.sp.H2 GCA_009690945.1
Candidatus.Midichloria.mitochondrii.IricVA GCA_000219355.1_ASM21935v1
Rickettsiales.endosymbiont.of.Peranema.trichophorum GCA_004210275.1_ASM421027v1
Candidatus.Jidaibacter.acanthamoeba GCA_000815465.1_UWC36
Rickettsia.bellii.RML369.C GCA_000012385.1_ASM1238v1
Candidatus.Megaira.polyxenophila
Rickettsiales.endosymbiont.of.Stachyamoeba.lipophora GCA_003932735.1_ASM393273v1
Rickettsiales.bacterium.Ac37b GCA_000746585.2_ASM74658v2
Magnetofaba.australis.IT.1 GCA_002109495.1_ASM210949v1
Magnetococcus.marinus.MC.1 GCA_000014865.1_ASM1486v1
Nitrosospira.briensis.C.128 GCA_000619905.2_ASM61990v2
Methylotenera.versatilis.301 GCA_000093025.1_ASM9302v1
Ralstonia.pickettii GCA_902374465.1.MGYG.HGUT.01384
Burkholderia.cepacia GCA_009586235.1_ASM958623v1
Dasania.marina.DSM.21967 GCA_000373485.1_ASM37348v1
Congregibacter.litoralis.KT71 GCA_000153125.2_ASM15312v2
Enterobacter.cloacae GCA_000770155.1_ASM77015v1
Alteromonas.macleodii.ATCC.27126 GCA_000172635.2_ASM17263v2
0.683448

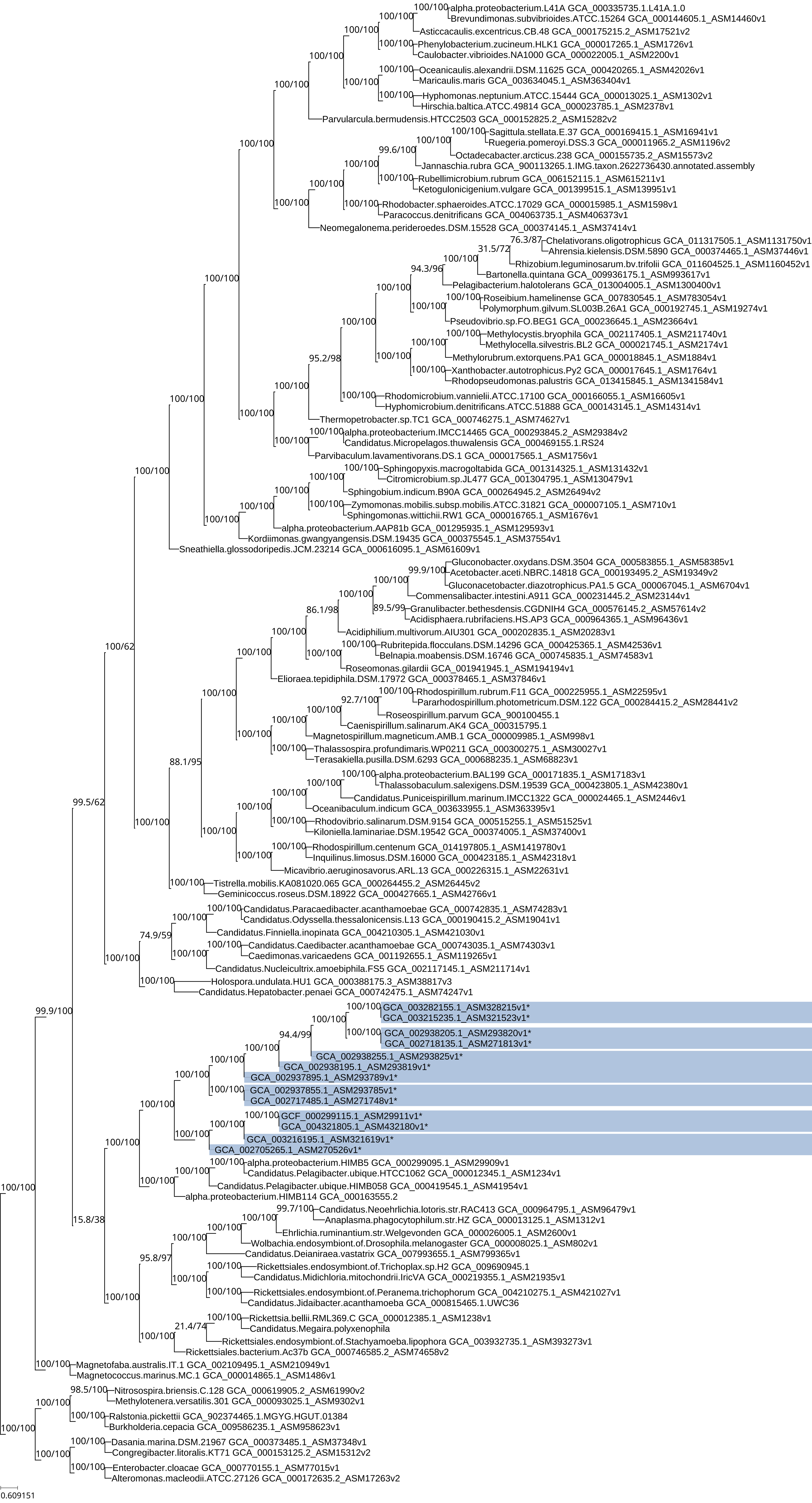
100/100 alpha.proteobacterium.L41A GCA_000335735.1.L41A.1.0
Brevundimonas.subvibrioides.ATCC.15264 GCA_000144605.1_ASM14460v1
100/100
Asticcacaulis.excentricus.CB.48 GCA_000175215.2_ASM17521v2
100/100
100/100 Phenylobacterium.zucineum.HLK1 GCA_000017265.1_ASM1726v1
Caulobacter.vibrioides.NA1000 GCA_000022005.1_ASM2200v1
100/100
100/100 Oceanicaulis.alexandrii.DSM.11625 GCA_000420265.1_ASM42026v1
100/100
Maricaulis.maris GCA_003634045.1_ASM363404v1
100/100
100/100 Hyphomonas.neptunium.ATCC.15444 GCA_000013025.1_ASM1302v1
Hirschia.baltica.ATCC.49814 GCA_000023785.1_ASM2378v1
Parvularcula.bermudensis.HTCC2503 GCA_000152825.2_ASM15282v2
100/100 Sagittula.stellata.E.37 GCA_000169415.1_ASM16941v1
100/100
100/100 Ruegeria.pomeroyi.DSS.3 GCA_000011965.2_ASM1196v2
99.6/100
Octadecabacter.arcticus.238 GCA_000155735.2_ASM15573v2
Jannaschia.rubra GCA_900113265.1.IMG.taxon.2622736430.annotated.assembly
100/100
100/100 Rubellimicrobium.rubrum GCA_006152115.1_ASM615211v1
100/100
Ketogulonicigenium.vulgare GCA_001399515.1_ASM139951v1
100/100
100/100 Rhodobacter.sphaeroides.ATCC.17029 GCA_000015985.1_ASM1598v1
Paracoccus.denitrificans GCA_004063735.1_ASM406373v1
Neomegalonema.perideroedes.DSM.15528 GCA_000374145.1_ASM37414v1
76.3/87 Chelativorans.oligotrophicus GCA_011317505.1_ASM1131750v1
31.5/72
Ahrensia.kielensis.DSM.5890 GCA_000374465.1_ASM37446v1
100/100
Rhizobium.leguminosarum.bv.trifolii GCA_011604525.1_ASM1160452v1
100/100
94.3/96
Bartonella.quintana GCA_009936175.1_ASM993617v1
Pelagibacterium.halotolerans GCA_013004005.1_ASM1300400v1
100/100
100/100 Roseibium.hamelinense GCA_007830545.1_ASM783054v1
100/100
Polymorphum.gilvum.SL003B.26A1 GCA_000192745.1_ASM19274v1
100/100
Pseudovibrio.sp.FO.BEG1 GCA_000236645.1_ASM23664v1
100/100 Methylocystis.bryophila GCA_002117405.1_ASM211740v1
100/100
Methylocella.silvestris.BL2 GCA_000021745.1_ASM2174v1
95.2/98
100/100
Methylorubrum.extorquens.PA1 GCA_000018845.1_ASM1884v1
100/100
100/100 Xanthobacter.autotrophicus.Py2 GCA_000017645.1_ASM1764v1
Rhodopseudomonas.palustris GCA_013415845.1_ASM1341584v1
100/100
100/100 Rhodomicrobium.vannielii.ATCC.17100 GCA_000166055.1_ASM16605v1
Hyphomicrobium.denitrificans.ATCC.51888 GCA_000143145.1_ASM14314v1
100/100
Thermopetrobacter.sp.TC1 GCA_000746275.1_ASM74627v1
100/100 alpha.proteobacterium.IMCC14465 GCA_000293845.2_ASM29384v2
100/100
Candidatus.Micropelagos.thuwalensis GCA_000469155.1.RS24
Parvibaculum.lavamentivorans.DS.1 GCA_000017565.1_ASM1756v1
100/100
100/100 Sphingopyxis.macrogoltabida GCA_001314325.1_ASM131432v1
100/100
Citromicrobium.sp.JL477 GCA_001304795.1_ASM130479v1
100/100
Sphingobium.indicum.B90A GCA_000264945.2_ASM26494v2
100/100
100/100 Zymomonas.mobilis.subsp.mobilis.ATCC.31821 GCA_000007105.1_ASM710v1
100/100
Sphingomonas.wittichii.RW1 GCA_000016765.1_ASM1676v1
alpha.proteobacterium.AAP81b GCA_001295935.1_ASM129593v1
Kordiimonas.gwangyangensis.DSM.19435 GCA_000375545.1_ASM37554v1
Sneathiella.glossodoripedis.JCM.23214 GCA_000616095.1_ASM61609v1
Gluconobacter.oxydans.DSM.3504 GCA_000583855.1_ASM58385v1
99.9/100
Acetobacter.aceti.NBRC.14818 GCA_000193495.2_ASM19349v2
100/100
Gluconacetobacter.diazotrophicus.PA1.5 GCA_000067045.1_ASM6704v1
100/100
Commensalibacter.intestini.A911 GCA_000231445.2_ASM23144v1
86.1/98
89.5/99 Granulibacter.bethesdensis.CGDNIH4 GCA_000576145.2_ASM57614v2
Acidisphaera.rubrifaciens.HS.AP3 GCA_000964365.1_ASM96436v1
100/100
Acidiphilium.multivorum.AIU301 GCA_000202835.1_ASM20283v1
100/100
100/100 Rubritepida.flocculans.DSM.14296 GCA_000425365.1_ASM42536v1
100/100
Belnapia.moabensis.DSM.16746 GCA_000745835.1_ASM74583v1
Roseomonas.gilardii GCA_001941945.1_ASM194194v1
100/62
Elioraea.tepidiphila.DSM.17972 GCA_000378465.1_ASM37846v1
100/100 Rhodospirillum.rubrum.F11 GCA_000225955.1_ASM22595v1
100/100
92.7/100
Pararhodospirillum.photometricum.DSM.122 GCA_000284415.2_ASM28441v2
100/100
Roseospirillum.parvum GCA_900100455.1
100/100
Caenispirillum.salinarum.AK4 GCA_000315795.1
Magnetospirillum.magneticum.AMB.1 GCA_000009985.1_ASM998v1
100/100
100/100 Thalassospira.profundimaris.WP0211 GCA_000300275.1_ASM30027v1
Terasakiella.pusilla.DSM.6293 GCA_000688235.1_ASM68823v1
88.1/95
100/100 alpha.proteobacterium.BAL199 GCA_000171835.1_ASM17183v1
100/100
Thalassobaculum.salexigens.DSM.19539 GCA_000423805.1_ASM42380v1
100/100
Candidatus.Puniceispirillum.marinum.IMCC1322 GCA_000024465.1_ASM2446v1
99.5/62
100/100
Oceanibaculum.indicum GCA_003633955.1_ASM363395v1
100/100
100/100 Rhodovibrio.salinarum.DSM.9154 GCA_000515255.1_ASM51525v1
100/100
Kiloniella.laminariae.DSM.19542 GCA_000374005.1_ASM37400v1
100/100
100/100 Rhodospirillum.centenum GCA_014197805.1_ASM1419780v1
100/100
Inquilinus.limosus.DSM.16000 GCA_000423185.1_ASM42318v1
Micavibrio.aeruginosavorus.ARL.13 GCA_000226315.1_ASM22631v1
100/100 Tistrella.mobilis.KA081020.065 GCA_000264455.2_ASM26445v2
Geminicoccus.roseus.DSM.18922 GCA_000427665.1_ASM42766v1
100/100 Candidatus.Paracaedibacter.acanthamoebae GCA_000742835.1_ASM74283v1
100/100
Candidatus.Odyssella.thessalonicensis.L13 GCA_000190415.2_ASM19041v1
74.9/59
Candidatus.Finniella.inopinata GCA_004210305.1_ASM421030v1
100/100 Candidatus.Caedibacter.acanthamoebae GCA_000743035.1_ASM74303v1
100/100
100/100
Caedimonas.varicaedens GCA_001192655.1_ASM119265v1
Candidatus.Nucleicultrix.amoebiphila.FS5 GCA_002117145.1_ASM211714v1
100/100
Holospora.undulata.HU1 GCA_000388175.3_ASM38817v3
Candidatus.Hepatobacter.penaei GCA_000742475.1_ASM74247v1
100/100 GCA_003282155.1_ASM328215v1*
99.9/100
100/100
GCA_003215235.1_ASM321523v1*
100/100 GCA_002938205.1_ASM293820v1*
94.4/99
GCA_002718135.1_ASM271813v1*
100/100
GCA_002938255.1_ASM293825v1*
100/100
GCA_002938195.1_ASM293819v1*
100/100
GCA_002937895.1_ASM293789v1*
100/100 GCA_002937855.1_ASM293785v1*
100/100
GCA_002717485.1_ASM271748v1*
100/100 GCF_000299115.1_ASM29911v1*
100/100
GCA_004321805.1_ASM432180v1*
100/100
100/100 GCA_003216195.1_ASM321619v1*
GCA_002705265.1_ASM270526v1*
100/100 alpha.proteobacterium.HIMB5 GCA_000299095.1_ASM29909v1
100/100
Candidatus.Pelagibacter.ubique.HTCC1062 GCA_000012345.1_ASM1234v1
100/100
Candidatus.Pelagibacter.ubique.HIMB058 GCA_000419545.1_ASM41954v1
100/100
alpha.proteobacterium.HIMB114 GCA_000163555.2
99.7/100 Candidatus.Neoehrlichia.lotoris.str.RAC413 GCA_000964795.1_ASM96479v1
15.8/38
100/100
Anaplasma.phagocytophilum.str.HZ GCA_000013125.1_ASM1312v1
100/100
Ehrlichia.ruminantium.str.Welgevonden GCA_000026005.1_ASM2600v1
100/100
Wolbachia.endosymbiont.of.Drosophila.melanogaster GCA_000008025.1_ASM802v1
Candidatus.Deianiraea.vastatrix GCA_007993655.1_ASM799365v1
95.8/97
100/100 Rickettsiales.endosymbiont.of.Trichoplax.sp.H2 GCA_009690945.1
100/100
Candidatus.Midichloria.mitochondrii.IricVA GCA_000219355.1_ASM21935v1
100/100 Rickettsiales.endosymbiont.of.Peranema.trichophorum GCA_004210275.1_ASM421027v1
100/100
Candidatus.Jidaibacter.acanthamoeba GCA_000815465.1.UWC36
100/100 Rickettsia.bellii.RML369.C GCA_000012385.1_ASM1238v1
21.4/74
Candidatus.Megaira.polyxenophila
100/100
Rickettsiales.endosymbiont.of.Stachyamoeba.lipophora GCA_003932735.1_ASM393273v1
Rickettsiales.bacterium.Ac37b GCA_000746585.2_ASM74658v2
100/100 Magnetofaba.australis.IT.1 GCA_002109495.1_ASM210949v1
100/100
Magnetococcus.marinus.MC.1 GCA_000014865.1_ASM1486v1
98.5/100 Nitrosospira.briensis.C.128 GCA_000619905.2_ASM61990v2
100/100
Methylotenera.versatilis.301 GCA_000093025.1_ASM9302v1
100/100 Ralstonia.pickettii GCA_902374465.1.MGYG.HGUT.01384
Burkholderia.cepacia GCA_009586235.1_ASM958623v1
100/100
100/100 Dasania.marina.DSM.21967 GCA_000373485.1_ASM37348v1
Congregibacter.litoralis.KT71 GCA_000153125.2_ASM15312v2
100/100
100/100 Enterobacter.cloacae GCA_000770155.1_ASM77015v1
Alteromonas.macleodii.ATCC.27126 GCA_000172635.2_ASM17263v2
0.609151

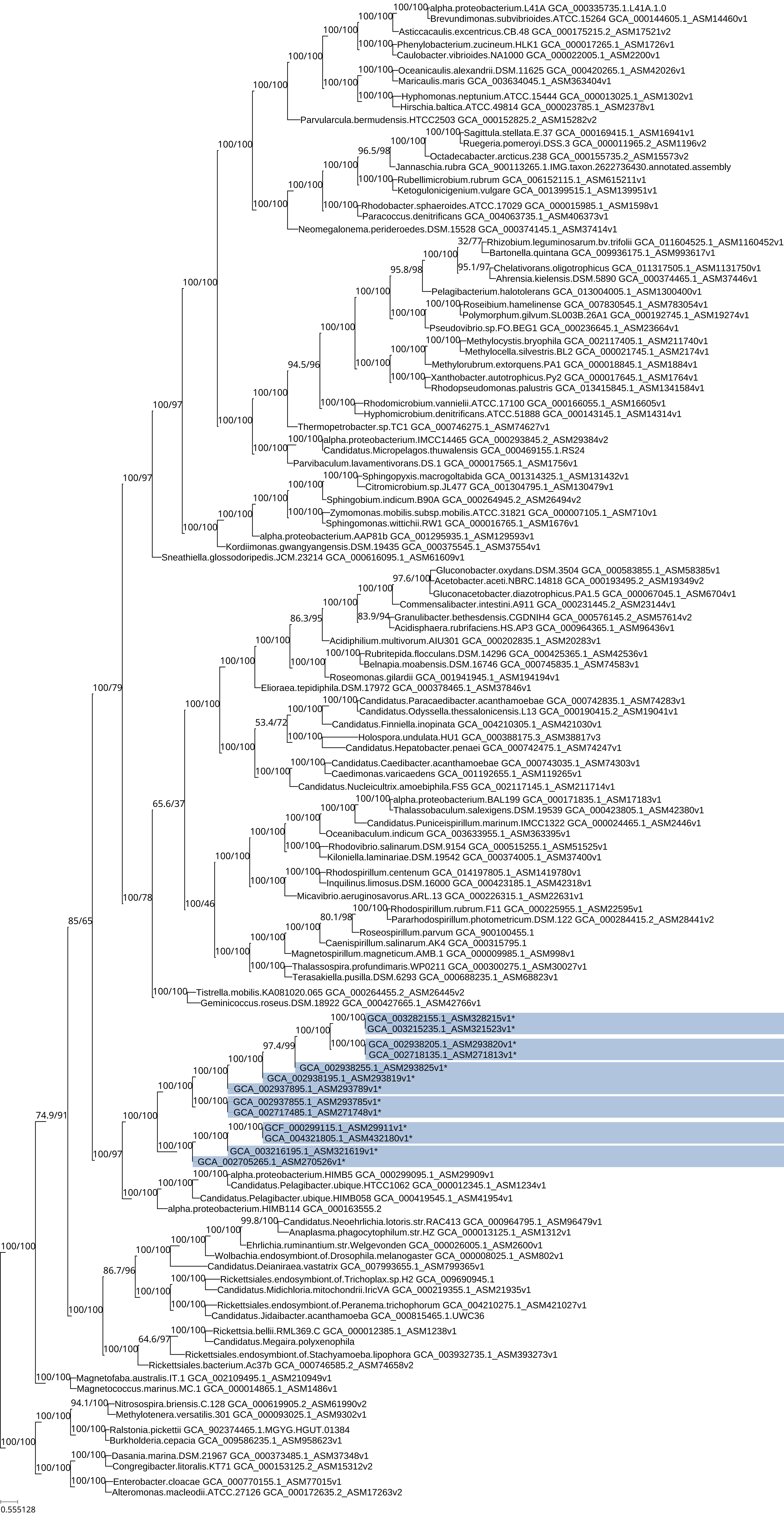
alpha.proteobacterium.L41A GCA_000335735.1.L41A.1.0
Brevundimonas.subvibrioides.ATCC.15264 GCA_000144605.1_ASM14460v1
Asticcacaulis.excentricus.CB.48 GCA_000175215.2_ASM17521v2
Phenylobacterium.zucineum.HLK1 GCA_000017265.1_ASM1726v1
Caulobacter.vibrioides.NA1000 GCA_000022005.1_ASM2200v1
Oceanicaulis.alexandrii.DSM.11625 GCA_000420265.1_ASM42026v1
Maricaulis.maris GCA_003634045.1_ASM363404v1
Hyphomonas.neptunium.ATCC.15444 GCA_000013025.1_ASM1302v1
Hirschia.baltica.ATCC.49814 GCA_000023785.1_ASM2378v1
Parvularcula.bermudensis.HTCC2503 GCA_000152825.2_ASM15282v2
Sagittula.stellata.E.37 GCA_000169415.1_ASM16941v1
Ruegeria.pomeroyi.DSS.3 GCA_000011965.2_ASM1196v2
Octadecabacter.arcticus.238 GCA_000155735.2_ASM15573v2
Jannaschia.rubra GCA_900113265.1.IMG.taxon.2622736430.annotated.assembly
Rubellimicrobium.rubrum GCA_006152115.1_ASM615211v1
Ketogulonicigenium.vulgare GCA_001399515.1_ASM139951v1
Rhodobacter.sphaeroides.ATCC.17029 GCA_000015985.1_ASM1598v1
Paracoccus.denitrificans GCA_004063735.1_ASM406373v1
Neomegalonema.perideroedes.DSM.15528 GCA_000374145.1_ASM37414v1
Rhizobium.leguminosarum.bv.trifolii GCA_011604525.1_ASM1160452v1
Bartonella.quintana GCA_009936175.1_ASM993617v1
Chelativorans.oligotrophicus GCA_011317505.1_ASM1131750v1
Ahrensia.kielensis.DSM.5890 GCA_000374465.1_ASM37446v1
Pelagibacterium.halotolerans GCA_013004005.1_ASM1300400v1
Roseibium.hamelinense GCA_007830545.1_ASM783054v1
Polymorphum.gilvum.SL003B.26A1 GCA_000192745.1_ASM19274v1
Pseudovibrio.sp.FO.BEG1 GCA_000236645.1_ASM23664v1
Methylocystis.bryophila GCA_002117405.1_ASM211740v1
Methylocella.silvestris.BL2 GCA_000021745.1_ASM2174v1
Methylorubrum.extorquens.PA1 GCA_000018845.1_ASM1884v1
Xanthobacter.autotrophicus.Py2 GCA_000017645.1_ASM1764v1
Rhodopseudomonas.palustris GCA_013415845.1_ASM1341584v1
Rhodomicrobium.vannielii.ATCC.17100 GCA_000166055.1_ASM16605v1
Hyphomicrobium.denitrificans.ATCC.51888 GCA_000143145.1_ASM14314v1
Thermopetrobacter.sp.TC1 GCA_000746275.1_ASM74627v1
alpha.proteobacterium.IMCC14465 GCA_000293845.2_ASM29384v2
Candidatus.Micropelagos.thuwalensis GCA_000469155.1.RS24
Parvibaculum.lavamentivorans.DS.1 GCA_000017565.1_ASM1756v1
Sphingopyxis.macrogoltabida GCA_001314325.1_ASM131432v1
Citromicrobium.sp.JL477 GCA_001304795.1_ASM130479v1
Sphingobium.indicum.B90A GCA_000264945.2_ASM26494v2
Zymomonas.mobilis.subsp.mobilis.ATCC.31821 GCA_000007105.1_ASM710v1
Sphingomonas.wittichii.RW1 GCA_000016765.1_ASM1676v1
alpha.proteobacterium.AAP81b GCA_001295935.1_ASM129593v1
Kordiimonas.gwangyangensis.DSM.19435 GCA_000375545.1_ASM37554v1
Sneathiella.glossodoripedis.JCM.23214 GCA_000616095.1_ASM61609v1
Gluconobacter.oxydans.DSM.3504 GCA_000583855.1_ASM58385v1
Acetobacter.aceti.NBRC.14818 GCA_000193495.2_ASM19349v2
Gluconacetobacter.diazotrophicus.PA1.5 GCA_000067045.1_ASM6704v1
Commensalibacter.intestini.A911 GCA_000231445.2_ASM23144v1
Granulibacter.bethesdensis.CGDNIH4 GCA_000576145.2_ASM57614v2
Acidisphaera.rubrifaciens.HS.AP3 GCA_000964365.1_ASM96436v1
Acidiphilium.multivorum.AIU301 GCA_000202835.1_ASM20283v1
Rubritepida.flocculans.DSM.14296 GCA_000425365.1_ASM42536v1
Belnapia.moabensis.DSM.16746 GCA_000745835.1_ASM74583v1
Roseomonas.gilardii GCA_001941945.1_ASM194194v1
Elioraea.tepidiphila.DSM.17972 GCA_000378465.1_ASM37846v1
Candidatus.Paracaedibacter.acanthamoebae GCA_000742835.1_ASM74283v1
Candidatus.Odyssella.thessalonicensis.L13 GCA_000190415.2_ASM19041v1
Candidatus.Finniella.inopinata GCA_004210305.1_ASM421030v1
Holospora.undulata.HU1 GCA_000388175.3_ASM38817v3
Candidatus.Hepatobacter.penaei GCA_000742475.1_ASM74247v1
Candidatus.Caedibacter.acanthamoebae GCA_000743035.1_ASM74303v1
Caedimonas.varicaedens GCA_001192655.1_ASM119265v1
Candidatus.Nucleicultrix.amoebiphila.FS5 GCA_002117145.1_ASM211714v1
alpha.proteobacterium.BAL199 GCA_000171835.1_ASM17183v1
Thalassobaculum.salexigens.DSM.19539 GCA_000423805.1_ASM42380v1
Candidatus.Puniceispirillum.marinum.IMCC1322 GCA_000024465.1_ASM2446v1
Oceanibaculum.indicum GCA_003633955.1_ASM363395v1
Rhodovibrio.salinarum.DSM.9154 GCA_000515255.1_ASM51525v1
Kiloniella.laminariae.DSM.19542 GCA_000374005.1_ASM37400v1
Rhodospirillum.centenum GCA_014197805.1_ASM1419780v1
Inquilinus.limosus.DSM.16000 GCA_000423185.1_ASM42318v1
Micavibrio.aeruginosavorus.ARL.13 GCA_000226315.1_ASM22631v1
Rhodospirillum.rubrum.F11 GCA_000225955.1_ASM22595v1
Pararhodospirillum.photometricum.DSM.122 GCA_000284415.2_ASM28441v2
Roseospirillum.parvum GCA_900100455.1
Caenispirillum.salinarum.AK4 GCA_000315795.1
Magnetospirillum.magneticum.AMB.1 GCA_000009985.1_ASM998v1
Thalassospira.profundimaris.WP0211 GCA_000300275.1_ASM30027v1
Terasakiella.pusilla.DSM.6293 GCA_000688235.1_ASM68823v1
Tistrella.mobilis.KA081020.065 GCA_000264455.2_ASM26445v2
Geminicoccus.roseus.DSM.18922 GCA_000427665.1_ASM42766v1
GCA_003282155.1_ASM328215v1*
GCA_003215235.1_ASM321523v1*
GCA_002938205.1_ASM293820v1*
GCA_002718135.1_ASM271813v1*
GCA_002938255.1_ASM293825v1*
GCA_002938195.1_ASM293819v1*
GCA_002937895.1_ASM293789v1*
GCA_002937855.1_ASM293785v1*
GCA_002717485.1_ASM271748v1*
GCF_000299115.1_ASM29911v1*
GCA_004321805.1_ASM432180v1*
GCA_003216195.1_ASM321619v1*
GCA_002705265.1_ASM270526v1*
alpha.proteobacterium.HIMB5 GCA_000299095.1_ASM29909v1
Candidatus.Pelagibacter.ubique.HTCC1062 GCA_000012345.1_ASM1234v1
Candidatus.Pelagibacter.ubique.HIMB058 GCA_000419545.1_ASM41954v1
alpha.proteobacterium.HIMB114 GCA_000163555.2
Candidatus.Neoehrlichia.lotoris.str.RAC413 GCA_000964795.1_ASM96479v1
Anaplasma.phagocytophilum.str.HZ GCA_000013125.1_ASM1312v1
Ehrlichia.ruminantium.str.Welgevonden GCA_000026005.1_ASM2600v1
Wolbachia.endosymbiont.of.Drosophila.melanogaster GCA_000008025.1_ASM802v1
Candidatus.Deianiraea.vastatrix GCA_007993655.1_ASM799365v1
Rickettsiales.endosymbiont.of.Trichoplax.sp.H2 GCA_009690945.1
Candidatus.Midichloria.mitochondrii.IricVA GCA_000219355.1_ASM21935v1
Rickettsiales.endosymbiont.of.Peranema.trichophorum GCA_004210275.1_ASM421027v1
Candidatus.Jidaibacter.acanthamoeba GCA_000815465.1.UWC36
Rickettsia.bellii.RML369.C GCA_000012385.1_ASM1238v1
Candidatus.Megaira.polyxenophila
Rickettsiales.endosymbiont.of.Stachyamoeba.lipophora GCA_003932735.1_ASM393273v1
Rickettsiales.bacterium.Ac37b GCA_000746585.2_ASM74658v2
Magnetofaba.australis.IT.1 GCA_002109495.1_ASM210949v1
Magnetococcus.marinus.MC.1 GCA_000014865.1_ASM1486v1
Nitrosospira.briensis.C.128 GCA_000619905.2_ASM61990v2
Methylotenera.versatilis.301 GCA_000093025.1_ASM9302v1
Ralstonia.pickettii GCA_902374465.1.MGYG.HGUT.01384
Burkholderia.cepacia GCA_009586235.1_ASM958623v1
Dasania.marina.DSM.21967 GCA_000373485.1_ASM37348v1
Congregibacter.litoralis.KT71 GCA_000153125.2_ASM15312v2
Enterobacter.cloacae GCA_000770155.1_ASM77015v1
Alteromonas.macleodii.ATCC.27126 GCA_000172635.2_ASM17263v2
0.555128

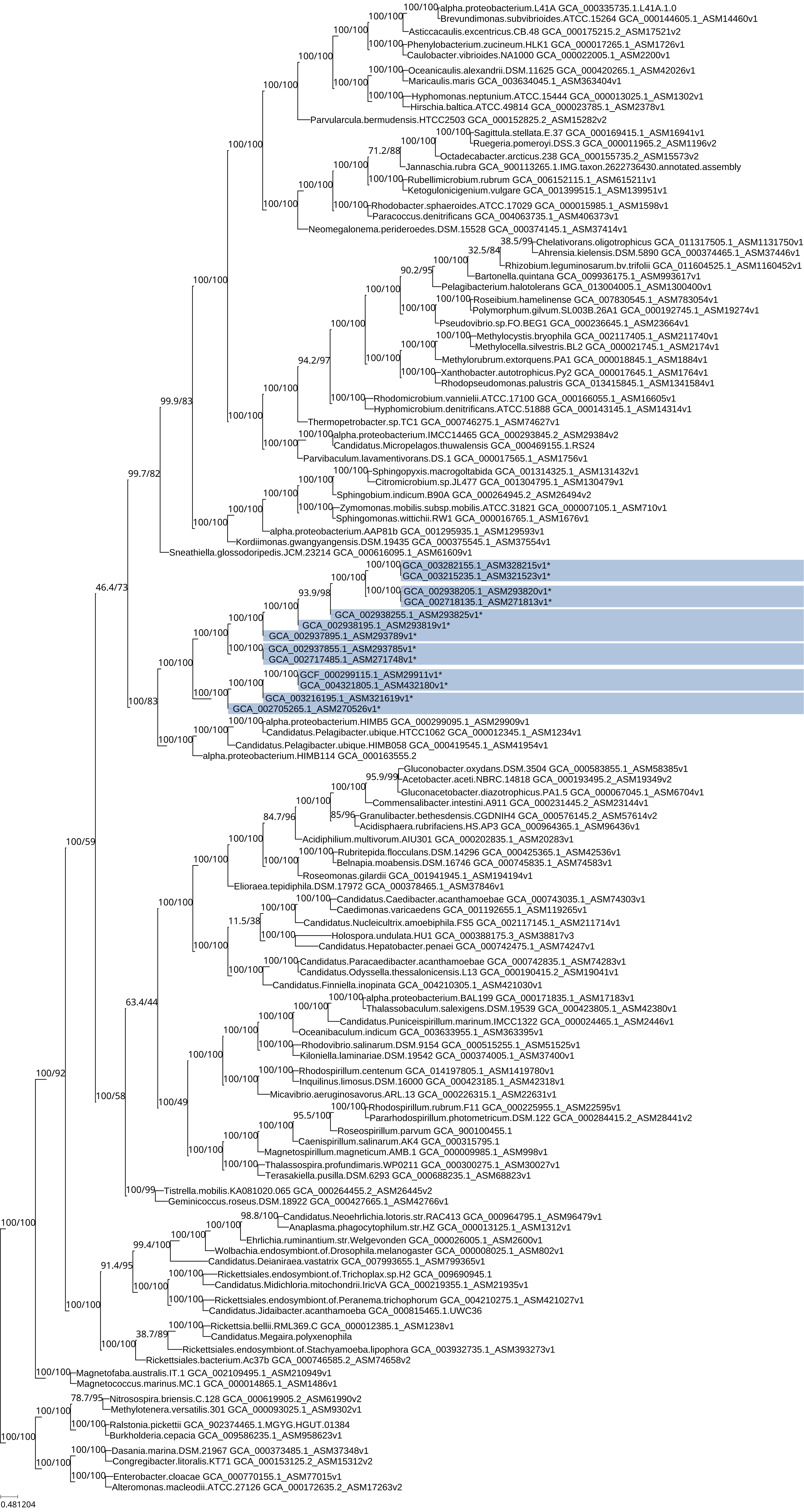
100/100
alpha.proteobacterium.L41A GCA_000335735.1_L41A.1.0
100/100
Brevundimonas.subvibrioides.ATCC.15264 GCA_000144605.1_ASM14460v1
100/100
Asticcacaulis.excentricus.CB.48 GCA_000175215.2_ASM17521v2
100/100
Phenylobacterium.zucineum.HLK1 GCA_000017265.1_ASM1726v1
100/100
Caulobacter.vibrioides.NA1000 GCA_000022005.1_ASM2200v1
100/100
Oceanicaulis.alexandrii.DSM.11625 GCA_000420265.1_ASM42026v1
100/100
Maricaulis.maris GCA_003634045.1_ASM363404v1
100/100
Hyphomonas.neptunium.ATCC.15444 GCA_000013025.1_ASM1302v1
100/100
Hirschia.baltica.ATCC.49814 GCA_000023785.1_ASM2378v1
100/100
Parvularcula.bermudensis.HTCC2503 GCA_000152825.2_ASM15282v2
100/100
Sagittula.stellata.E.37 GCA_000169415.1_ASM16941v1
100/100
Ruegeria.pomeroyi.DSS.3 GCA_000011965.2_ASM1196v2
100/100
Octadecabacter.arcticus.238 GCA_000155735.2_ASM15573v2
71.2/88
Jannaschia.rubra GCA_900113265.1.IMG.taxon.2622736430.annotated.assembly
100/100
Rubellimicrobium.rubrum GCA_006152115.1_ASM615211v1
100/100
Ketogulonicigenium.vulgare GCA_001399515.1_ASM139951v1
100/100
Rhodobacter.sphaeroides.ATCC.17029 GCA_000015985.1_ASM1598v1
100/100
Paracoccus.denitrificans GCA_004063735.1_ASM406373v1
100/100
Neomegalonema.perideroedes.DSM.15528 GCA_000374145.1_ASM37414v1
38.5/99
Chelativorans.oligotrophicus GCA_011317505.1_ASM1131750v1
32.5/84
Ahrensia.kielensis.DSM.5890 GCA_000374465.1_ASM37446v1
100/100
Rhizobium.leguminosarum.bv.trifolii GCA_011604525.1_ASM1160452v1
90.2/95
Bartonella.quintana GCA_009936175.1_ASM993617v1
100/100
Pelagibacterium.halotolerans GCA_013004005.1_ASM1300400v1
100/100
Roseibium.hamelinense GCA_007830545.1_ASM783054v1
100/100
Polymorphum.gilvum.SL003B.26A1 GCA_000192745.1_ASM19274v1
100/100
Pseudovibrio.sp.FO.BEG1 GCA_000236645.1_ASM23664v1
100/100
Methylocystis.bryophila GCA_002117405.1_ASM211740v1
100/100
Methylocella.silvestris.BL2 GCA_000021745.1_ASM2174v1
100/100
Methylorubrum.extorquens.PA1 GCA_000018845.1_ASM1884v1
94.2/97
Xanthobacter.autotrophicus.Py2 GCA_000017645.1_ASM1764v1
100/100
Rhodopseudomonas.palustris GCA_013415845.1_ASM1341584v1
100/100
Rhodomicrobium.vannielii.ATCC.17100 GCA_000166055.1_ASM16605v1
100/100
Hyphomicrobium.denitrificans.ATCC.51888 GCA_000143155.1_ASM14314v1
99.9/83
Thermopetrobacter.sp.TC1 GCA_000746275.1_ASM74627v1
100/100
alpha.proteobacterium.IMCC14465 GCA_000293845.2_ASM29384v2
100/100
Candidatus.Micropelagos.thuwalensis GCA_000469155.1.RS24
Parvibaculum.lavamentivorans.DS.1 GCA_000017565.1_ASM1756v1
99.7/82
100/100
Sphingopyxis.macrogoltabida GCA_001314325.1_ASM131432v1
100/100
Citromicrobium.sp.JL477 GCA_001304795.1_ASM130479v1
100/100
Sphingobium.indicum.B90A GCA_000264945.2_ASM26494v2
100/100
Zymomonas.mobilis.subsp.mobilis.ATCC.31821 GCA_000007105.1_ASM710v1
100/100
Sphingomonas.wittichii.RW1 GCA_000016765.1_ASM1676v1
100/100
alpha.proteobacterium.AAP81b GCA_001295935.1_ASM129593v1
Kordiimonas.gwangyangensis.DSM.19435 GCA_000375545.1_ASM37554v1
Sneathiella.glossodoripedis.JCM.23214 GCA_000616095.1_ASM61609v1
100/100
GCA_003282155.1_ASM328215v1*
GCA_003215235.1_ASM321523v1*
100/100
100/100
GCA_002938205.1_ASM293820v1*
93.9/98
GCA_002718135.1_ASM271813v1*
46.4/73
100/100
GCA_002938255.1_ASM293825v1*
100/100
GCA_002938195.1_ASM293819v1*
100/100
GCA_002937895.1_ASM293789v1*
100/100
GCA_002937855.1_ASM293785v1*
100/100
GCA_002717485.1_ASM271748v1*
100/100
GCF_000299115.1_ASM29911v1*
100/100
GCA_004321805.1_ASM432180v1*
100/83
100/100
GCA_003216195.1_ASM321619v1*
GCA_002705265.1_ASM270526v1*
100/100
alpha.proteobacterium.HIMB5 GCA_000299095.1_ASM29909v1
100/100
Candidatus.Pelagibacter.ubique.HTCC1062 GCA_000012345.1_ASM1234v1
100/100
Candidatus.Pelagibacter.ubique.HIMB058 GCA_000419545.1_ASM41954v1
alpha.proteobacterium.HIMB114 GCA_000163555.2
Gluconobacter.oxydans.DSM.3504 GCA_000583855.1_ASM58385v1
95.9/99
Acetobacter.aceti.NBRC.14818 GCA_000193495.2_ASM19349v2
100/100
Gluconacetobacter.diazotrophicus.PA1.5 GCA_000067045.1_ASM6704v1
100/100
Commensalibacter.intestini.A911 GCA_000231445.2_ASM23144v1
85/96
Granulibacter.bethesdensis.CGDNIH4 GCA_000576145.2_ASM57614v2
84.7/96
Acidisphaera.rubrifaciens.HS.AP3 GCA_000964365.1_ASM96436v1
100/59
Acidiphilium.multivorum.AIU301 GCA_000202835.1_ASM20283v1
100/100
100/100
Rubritepida.flocculans.DSM.14296 GCA_000425365.1_ASM42536v1
100/100
Belnapia.moabensis.DSM.16746 GCA_000745835.1_ASM74583v1
100/100
Roseomonas.gilardii GCA_001941945.1_ASM194194v1
Elioraea.tepidiphila.DSM.17972 GCA_000378465.1_ASM37846v1
100/100
100/100
Candidatus.Caedibacter.acanthamoebae GCA_000743035.1_ASM74303v1
100/100
Caedimonas.varicaedens GCA_001192655.1_ASM119265v1
11.5/38
Candidatus.Nucleicultrix.amoebiphila.FS5 GCA_002117145.1_ASM211714v1
100/100
Holospora.undulata.HU1 GCA_000388175.3_ASM38817v3
100/100
Candidatus.Hepatobacter.penaei GCA_000742475.1_ASM74247v1
100/100
Candidatus.Paracaedibacter.acanthamoebae GCA_000742835.1_ASM74283v1
100/100
Candidatus.Odyssella.thessalonicensis.L13 GCA_000190415.2_ASM19041v1
Candidatus.Finniella.inopinata GCA_004210305.1_ASM421030v1
63.4/44
100/100
alpha.proteobacterium.BAL199 GCA_000171835.1_ASM17183v1
100/100
Thalassobaculum.salexigens.DSM.19539 GCA_000423805.1_ASM42380v1
100/100
Candidatus.Puniceispirillum.marinum.IMCC1322 GCA_000024465.1_ASM2446v1
100/100
Oceanibaculum.indicum GCA_003633955.1_ASM363395v1
100/100
Rhodovibrio.salinarum.DSM.9154 GCA_000515255.1_ASM51525v1
100/100
Kiloniella.laminariae.DSM.19542 GCA_000374005.1_ASM37400v1
100/100
Rhodospirillum.centenum GCA_014197805.1_ASM1419780v1
100/92
100/100
Inquilinus.limosus.DSM.16000 GCA_000423185.1_ASM42318v1
Micavibrio.aeruginosavorus.ARL.13 GCA_000226315.1_ASM22631v1
100/58
100/49
100/100
Rhodospirillum.rubrum.F11 GCA_000225955.1_ASM22595v1
95.5/100
Pararhodospirillum.photometricum.DSM.122 GCA_000284415.2_ASM28441v2
100/100
Roseospirillum.parvum GCA_900100455.1
100/100
Caenispirillum.salinarum.AK4 GCA_000315795.1
100/100
Magnetospirillum.magneticum.AMB.1 GCA_000009985.1_ASM998v1
100/100
Thalassospira.profundimaris.WP0211 GCA_000300275.1_ASM30027v1
Terasakiella.pusilla.DSM.6293 GCA_000688235.1_ASM68823v1
100/99
Tistrella.mobilis.KA081020.065 GCA_000264455.2_ASM26445v2
Geminicoccus.roseus.DSM.18922 GCA_000427665.1_ASM42766v1
98.8/100
Candidatus.Neoehrlichia.lotoris.str.RAC413 GCA_000964795.1_ASM96479v1
100/100
100/100
Anaplasma.phagocytophilum.str.HZ GCA_000013125.1_ASM1312v1
100/100
Ehrlichia.ruminantium.str.Welgevonden GCA_000026005.1_ASM2600v1
99.4/100
Wolbachia.endosymbiont.of.Drosophila.melanogaster GCA_000008025.1_ASM802v1
Candidatus.Deianiraea.vastatrix GCA_007993655.1_ASM799365v1
91.4/95
100/100
Rickettsiales.endosymbiont.of.Trichoplax.sp.H2 GCA_009690945.1
100/100
Candidatus.Midichloria.mitochondrii.IricVA GCA_000219355.1_ASM21935v1
100/100
Rickettsiales.endosymbiont.of.Peranema.trichophorum GCA_004210275.1_ASM421027v1
100/100
Candidatus.Jidaibacter.acanthamoeba GCA_000815465.1.UWC36
100/100
Rickettsia.bellii.RML369.C GCA_000012385.1_ASM1238v1
38.7/89
Candidatus.Megaira.polyxenophila
100/100
Rickettsiales.endosymbiont.of.Stachyamoeba.lipophora GCA_003932735.1_ASM393273v1
Rickettsiales.bacterium.Ac37b GCA_000746585.2_ASM74658v2
100/100
Magnetofaba.australis.IT.1 GCA_002109495.1_ASM210949v1
Magnetococcus.marinus.MC.1 GCA_000014865.1_ASM1486v1
78.7/95
Nitrosospira.briensis.C.128 GCA_000619905.2_ASM61990v2
100/100
Methylotenera.versatilis.301 GCA_000093025.1_ASM9302v1
100/100
Ralstonia.pickettii GCA_902374465.1.MGYG.HGUT.01384
Burkholderia.cepacia GCA_009586235.1_ASM958623v1
100/100
100/100
Dasania.marina.DSM.21967 GCA_000373485.1_ASM37348v1
100/100
Congregibacter.litoralis.KT71 GCA_000153125.2_ASM15312v2
100/100
Enterobacter.cloacae GCA_000770155.1_ASM77015v1
Alteromonas.macleodii.ATCC.27126 GCA_000172635.2_ASM17263v2
0.481204

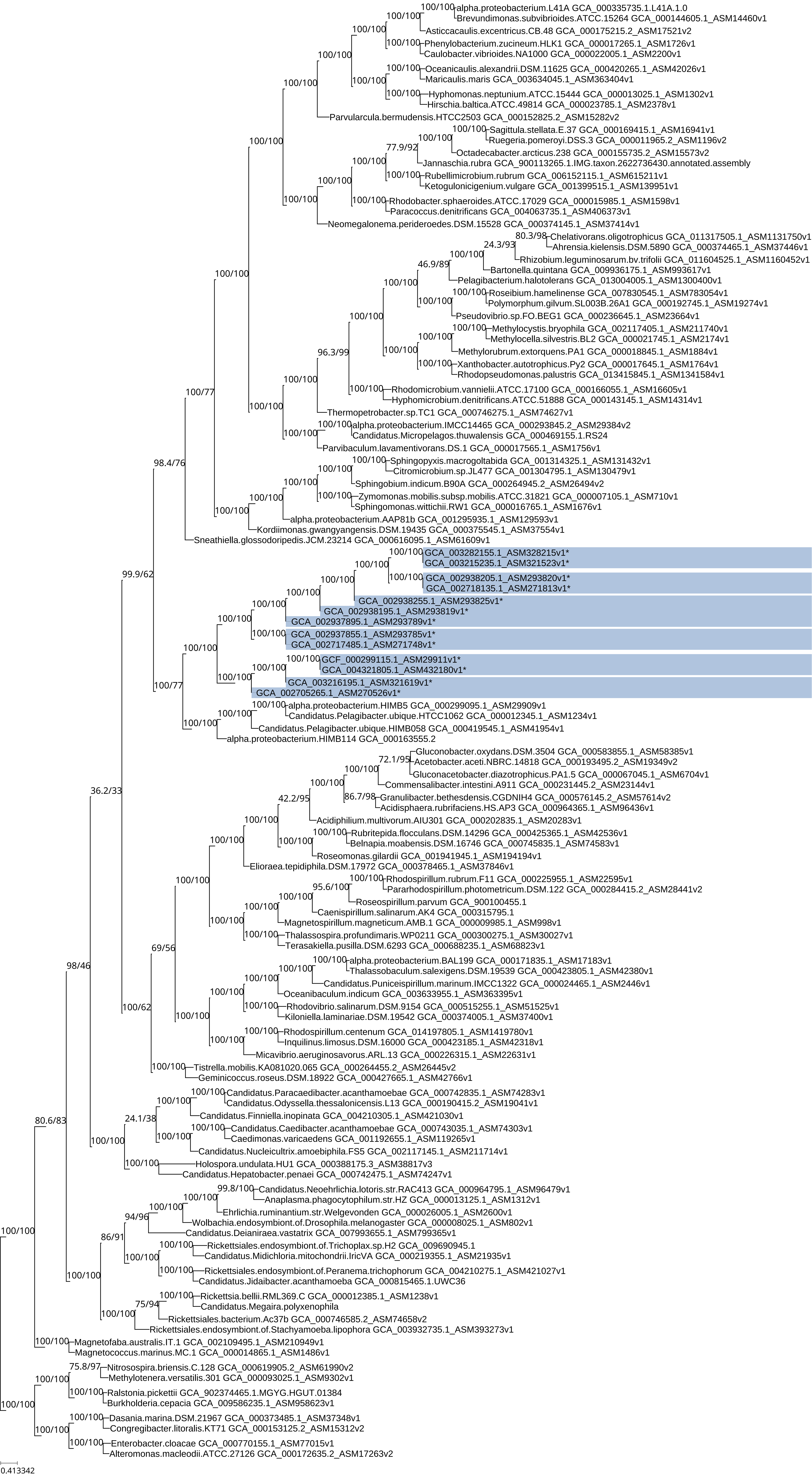
alpha.proteobacterium.L41A GCA_000335735.1.L41A.1.0
Brevundimonas.subvibrioides.ATCC.15264 GCA_000144605.1_ASM14460v1
Asticcacaulis.excentricus.CB.48 GCA_000175215.2_ASM17521v2
Phenylobacterium.zucineum.HLK1 GCA_000017265.1_ASM1726v1
Caulobacter.vibrioides.NA1000 GCA_000022005.1_ASM2200v1
Oceanicaulis.alexandrii.DSM.11625 GCA_000420265.1_ASM42026v1
Maricaulis.maris GCA_003634045.1_ASM363404v1
Hyphomonas.neptunium.ATCC.15444 GCA_000013025.1_ASM1302v1
Hirschia.baltica.ATCC.49814 GCA_000023785.1_ASM2378v1
Parvularcula.bermudensis.HTCC2503 GCA_000152825.2_ASM15282v2
Sagittula.stellata.E.37 GCA_000169415.1_ASM16941v1
Ruegeria.pomeroyi.DSS.3 GCA_000011965.2_ASM1196v2
Octadecabacter.arcticus.238 GCA_000155735.2_ASM15573v2
Jannaschia.rubra GCA_900113265.1.IMG.taxon.2622736430.annotated.assembly
Rubellimicrobium.rubrum GCA_006152115.1_ASM615211v1
Ketogulonicigenium.vulgare GCA_001399515.1_ASM139951v1
Rhodobacter.sphaeroides.ATCC.17029 GCA_000015985.1_ASM1598v1
Paracoccus.denitrificans GCA_004063735.1_ASM406373v1
Neomegalonema.perideroedes.DSM.15528 GCA_000374145.1_ASM37414v1
Chelativorans.oligotrophicus GCA_011317505.1_ASM1131750v1
Ahrensia.kielensis.DSM.5890 GCA_000374465.1_ASM37446v1
Rhizobium.leguminosarum.bv.trifolii GCA_011604525.1_ASM1160452v1
Bartonella.quintana GCA_009936175.1_ASM993617v1
Pelagibacterium.halotolerans GCA_013004005.1_ASM1300400v1
Roseibium.hamelinense GCA_007830545.1_ASM783054v1
Polymorphum.gilvum.SL003B.26A1 GCA_000192745.1_ASM19274v1
Pseudovibrio.sp.FO.BEG1 GCA_000236645.1_ASM23664v1
Methylocystis.bryophila GCA_002117405.1_ASM211740v1
Methylocella.silvestris.BL2 GCA_000021745.1_ASM2174v1
Methylorubrum.extorquens.PA1 GCA_000018845.1_ASM1884v1
Xanthobacter.autotrophicus.Py2 GCA_000017645.1_ASM1764v1
Rhodopseudomonas.palustris GCA_013415845.1_ASM1341584v1
Rhodomicrobium.vannielii.ATCC.17100 GCA_000166055.1_ASM16605v1
Hyphomicrobium.denitrificans.ATCC.51888 GCA_000143145.1_ASM14314v1
Thermopetrobacter.sp.TC1 GCA_000746275.1_ASM74627v1
alpha.proteobacterium.IMCC14465 GCA_000293845.2_ASM29384v2
Candidatus.Micropelagos.thuwalensis GCA_000469155.1.RS24
Parvibaculum.lavamentivorans.DS.1 GCA_000017565.1_ASM1756v1
Sphingopyxis.macrogoltabida GCA_001314325.1_ASM131432v1
Citromicrobium.sp.JL477 GCA_001304795.1_ASM130479v1
Sphingobium.indicum.B90A GCA_000264945.2_ASM26494v2
Zymomonas.mobilis.subsp.mobilis.ATCC.31821 GCA_000007105.1_ASM710v1
Sphingomonas.wittichii.RW1 GCA_000016765.1_ASM1676v1
alpha.proteobacterium.AAP81b GCA_001295935.1_ASM129593v1
Kordiimonas.gwangyangensis.DSM.19435 GCA_000375545.1_ASM37554v1
Sneathiella.glossodoripedis.JCM.23214 GCA_000616095.1_ASM61609v1
GCA_003282155.1_ASM328215v1*
GCA_003215235.1_ASM321523v1*
GCA_002938205.1_ASM293820v1*
GCA_002718135.1_ASM271813v1*
GCA_002938255.1_ASM293825v1*
GCA_002938195.1_ASM293819v1*
GCA_002937895.1_ASM293789v1*
GCA_002937855.1_ASM293785v1*
GCA_002717485.1_ASM271748v1*
GCF_000299115.1_ASM29911v1*
GCA_004321805.1_ASM432180v1*
GCA_003216195.1_ASM321619v1*
GCA_002705265.1_ASM270526v1*
alpha.proteobacterium.HIMB5 GCA_000299095.1_ASM29909v1
Candidatus.Pelagibacter.ubique.HTCC1062 GCA_000012345.1_ASM1234v1
Candidatus.Pelagibacter.ubique.HIMB058 GCA_000419545.1_ASM41954v1
alpha.proteobacterium.HIMB114 GCA_000163555.2
Gluconobacter.oxydans.DSM.3504 GCA_000583855.1_ASM58385v1
Acetobacter.aceti.NBRC.14818 GCA_000193495.2_ASM19349v2
Gluconacetobacter.diazotrophicus.PA1.5 GCA_000067045.1_ASM6704v1
Commensalibacter.intestini.A911 GCA_000231445.2_ASM23144v1
Granulibacter.bethesdensis.CGDNIH4 GCA_000576145.2_ASM57614v2
Acidisphaera.rubrifaciens.HS.AP3 GCA_000964365.1_ASM96436v1
Acidiphilium.multivorum.AIU301 GCA_000202835.1_ASM20283v1
Rubritepida.flocculans.DSM.14296 GCA_000425365.1_ASM42536v1
Belnapia.moabensis.DSM.16746 GCA_000745835.1_ASM74583v1
Roseomonas.gilardii GCA_001941945.1_ASM194194v1
Elioraea.tepidiphila.DSM.17972 GCA_000378465.1_ASM37846v1
Rhodospirillum.rubrum.F11 GCA_000225955.1_ASM22595v1
Pararhodospirillum.photometricum.DSM.122 GCA_000284415.2_ASM28441v2
Roseospirillum.parvum GCA_900100455.1
Caenispirillum.salinarum.AK4 GCA_000315795.1
Magnetospirillum.magneticum.AMB.1 GCA_000009985.1_ASM998v1
Thalassospira.profundimaris.WP0211 GCA_000300275.1_ASM30027v1
Terasakiella.pusilla.DSM.6293 GCA_000688235.1_ASM68823v1
alpha.proteobacterium.BAL199 GCA_000171835.1_ASM17183v1
Thalassobaculum.salexigens.DSM.19539 GCA_000423805.1_ASM42380v1
Candidatus.Puniceispirillum.marinum.IMCC1322 GCA_000024465.1_ASM2446v1
Oceanibaculum.indicum GCA_003633955.1_ASM363395v1
Rhodovibrio.salinarum.DSM.9154 GCA_000515255.1_ASM51525v1
Kiloniella.laminariae.DSM.19542 GCA_000374005.1_ASM37400v1
Rhodospirillum.centenum GCA_014197805.1_ASM1419780v1
Inquilinus.limosus.DSM.16000 GCA_000423185.1_ASM42318v1
Micavibrio.aeruginosavorus.ARL.13 GCA_000226315.1_ASM22631v1
Tistrella.mobilis.KA081020.065 GCA_000264455.2_ASM26445v2
Geminicoccus.roseus.DSM.18922 GCA_000427665.1_ASM42766v1
Candidatus.Paracaedibacter.acanthamoebae GCA_000742835.1_ASM74283v1
Candidatus.Odyssella.thessalonicensis.L13 GCA_000190415.2_ASM19041v1
Candidatus.Finniella.inopinata GCA_004210305.1_ASM421030v1
Candidatus.Caedibacter.acanthamoebae GCA_000743035.1_ASM74303v1
Caedimonas.varicaedens GCA_001192655.1_ASM119265v1
Candidatus.Nucleicultrix.amoebiphila.FS5 GCA_002117145.1_ASM211714v1
Holospora.undulata.HU1 GCA_000388175.3_ASM38817v3
Candidatus.Hepatobacter.penaei GCA_000742475.1_ASM74247v1
Candidatus.Neoehrlichia.lotoris.str.RAC413 GCA_000964795.1_ASM96479v1
Anaplasma.phagocytophilum.str.HZ GCA_000013125.1_ASM1312v1
Ehrlichia.ruminantium.str.Welgevonden GCA_000026005.1_ASM2600v1
Wolbachia.endosymbiont.of.Drosophila.melanogaster GCA_000008025.1_ASM802v1
Candidatus.Deianiraea.vastatrix GCA_007993655.1_ASM799365v1
Rickettsiales.endosymbiont.of.Trichoplax.sp.H2 GCA_009690945.1
Candidatus.Midichloria.mitochondrii.IricVA GCA_000219355.1_ASM21935v1
Rickettsiales.endosymbiont.of.Peranema.trichophorum GCA_004210275.1_ASM421027v1
Candidatus.Jidaibacter.acanthamoeba GCA_000815465.1.UWC36
Rickettsia.bellii.RML369.C GCA_000012385.1_ASM1238v1
Candidatus.Megaira.polyxenophila
Rickettsiales.bacterium.Ac37b GCA_000746585.2_ASM74658v2
Rickettsiales.endosymbiont.of.Stachyamoeba.lipophora GCA_003932735.1_ASM393273v1
Magnetofaba.australis.IT.1 GCA_002109495.1_ASM210949v1
Magnetococcus.marinus.MC.1 GCA_000014865.1_ASM1486v1
Nitrosospira.briensis.C.128 GCA_000619905.2_ASM61990v2
Methylotenera.versatilis.301 GCA_000093025.1_ASM9302v1
Ralstonia.pickettii GCA_902374465.1.MGYG.HGUT.01384
Burkholderia.cepacia GCA_009586235.1_ASM958623v1
Dasania.marina.DSM.21967 GCA_000373485.1_ASM37348v1
Congregibacter.litoralis.KT71 GCA_000153125.2_ASM15312v2
Enterobacter.cloacae GCA_000770155.1_ASM77015v1
Alteromonas.macleodii.ATCC.27126 GCA_000172635.2_ASM17263v2
0.413342

Group K

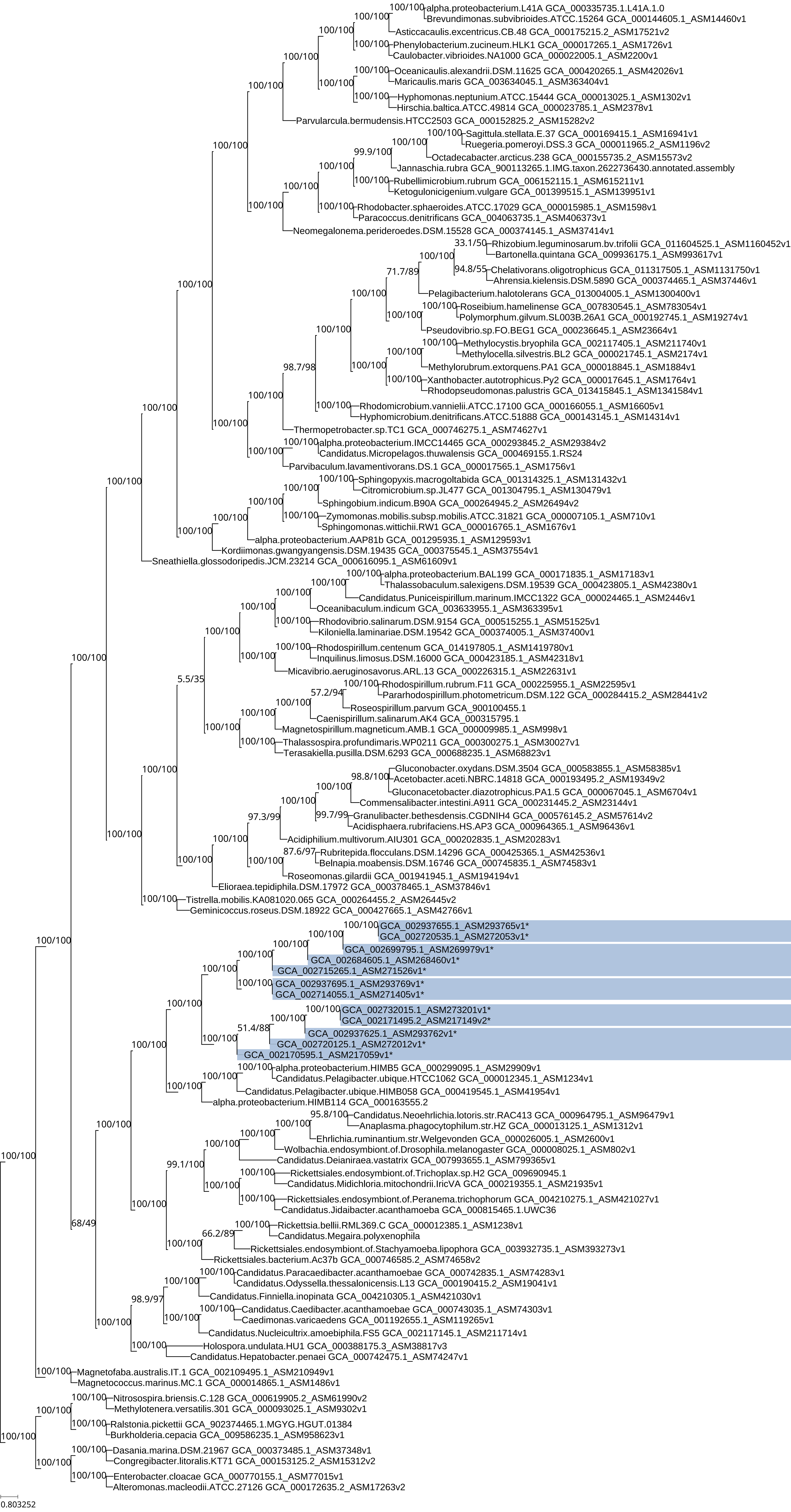

alpha.proteobacterium.L41A GCA_000335735.1_L41A.1.0
Brevundimonas.subvibrioides.ATCC.15264 GCA_000144605.1_ASM14460v1
Asticcacaulis.excentricus.CB.48 GCA_000175215.2_ASM17521v2
Phenylobacterium.zucineum.HLK1 GCA_000017265.1_ASM1726v1
Caulobacter.vibrioides.NA1000 GCA_000022005.1_ASM2200v1
Oceanicaulis.alexandrii.DSM.11625 GCA_000420265.1_ASM42026v1
Maricaulis.maris GCA_003634045.1_ASM363404v1
Hyphomonas.neptunium.ATCC.15444 GCA_000013025.1_ASM1302v1
Hirschia.baltica.ATCC.49814 GCA_000023785.1_ASM2378v1
Parvularcula.bermudensis.HTCC2503 GCA_000152825.2_ASM15282v2
Sagittula.stellata.E.37 GCA_000169415.1_ASM16941v1
Ruegeria.pomeroyi.DSS.3 GCA_000011965.2_ASM1196v2
Octadecabacter.arcticus.238 GCA_000155735.2_ASM15573v2
Jannaschia.rubra GCA_900113265.1.IMG.taxon.2622736430.annotated.assembly
Rubellimicrobium.rubrum GCA_006152115.1_ASM615211v1
Ketogulonicigenium.vulgare GCA_001399515.1_ASM139951v1
Rhodobacter.sphaeroides.ATCC.17029 GCA_000015985.1_ASM1598v1
Paracoccus.denitrificans GCA_004063735.1_ASM406373v1
Neomegalonema.perideroedes.DSM.15528 GCA_000374145.1_ASM37414v1
Rhizobium.leguminosarum.bv.trifolii GCA_011604525.1_ASM1160452v1
Bartonella.quintana GCA_009936175.1_ASM993617v1
Chelativorans.oligotrophicus GCA_011317505.1_ASM1131750v1
Ahrensia.kielensis.DSM.5890 GCA_000374465.1_ASM37446v1
Pelagibacterium.halotolerans GCA_013004005.1_ASM1300400v1
Roseibium.hamelinense GCA_007830545.1_ASM783054v1
Polymorphum.gilvum.SL003B.26A1 GCA_000192745.1_ASM19274v1
Pseudovibrio.sp.FO.BEG1 GCA_000236645.1_ASM23664v1
Methylocystis.bryophila GCA_002117405.1_ASM211740v1
Methylocella.silvestris.BL2 GCA_000021745.1_ASM2174v1
Methylorubrum.extorquens.PA1 GCA_000018845.1_ASM1884v1
Xanthobacter.autotrophicus.Py2 GCA_000017645.1_ASM1764v1
Rhodopseudomonas.palustris GCA_013415845.1_ASM1341584v1
Rhodomicrobium.vannielii.ATCC.17100 GCA_000166055.1_ASM16605v1
Hyphomicrobium.denitrificans.ATCC.51888 GCA_000143145.1_ASM14314v1
Thermopetrobacter.sp.TC1 GCA_000746275.1_ASM74627v1
alpha.proteobacterium.IMCC14465 GCA_000293845.2_ASM29384v2
Candidatus.Micropelagos.thuwalensis GCA_000469155.1.RS24
Parvibaculum.lavamentivorans.DS.1 GCA_000017565.1_ASM1756v1
Sphingopyxis.macrogoltabida GCA_001314325.1_ASM131432v1
Citromicrobium.sp.JL477 GCA_001304795.1_ASM130479v1
Sphingobium.indicum.B90A GCA_000264945.2_ASM26494v2
Zymomonas.mobilis.subsp.mobilis.ATCC.31821 GCA_000007105.1_ASM710v1
Sphingomonas.wittichii.RW1 GCA_000016765.1_ASM1676v1
alpha.proteobacterium.AAP81b GCA_001295935.1_ASM129593v1
Kordiimonas.gwangyangensis.DSM.19435 GCA_000375545.1_ASM37554v1
Sneathiella.glossodoripedis.JCM.23214 GCA_000616095.1_ASM61609v1
alpha.proteobacterium.BAL199 GCA_000171835.1_ASM17183v1
Thalassobaculum.salexigens.DSM.19539 GCA_000423805.1_ASM42380v1
Candidatus.Puniceispirillum.marinum.IMCC1322 GCA_000024465.1_ASM2446v1
Oceanibaculum.indicum GCA_003633955.1_ASM363395v1
Rhodovibrio.salinarum.DSM.9154 GCA_000515255.1_ASM51525v1
Kiloniella.laminariae.DSM.19542 GCA_000374005.1_ASM37400v1
Rhodospirillum.centenum GCA_014197805.1_ASM1419780v1
Inquilinus.limosus.DSM.16000 GCA_000423185.1_ASM42318v1
Micavibrio.aeruginosavorus.ARL.13 GCA_000226315.1_ASM22631v1
Rhodospirillum.rubrum.F11 GCA_000225955.1_ASM22595v1
Pararhodospirillum.photometricum.DSM.122 GCA_000284415.2_ASM28441v2
Roseospirillum.parvum GCA_900100455.1
Caenispirillum.salinarum.AK4 GCA_000315795.1
Magnetospirillum.magneticum.AMB.1 GCA_000009985.1_ASM998v1
Thalassospira.profundimaris.WP0211 GCA_000300275.1_ASM30027v1
Terasakiella.pusilla.DSM.6293 GCA_000688235.1_ASM68823v1
Gluconobacter.oxydans.DSM.3504 GCA_000583855.1_ASM58385v1
Acetobacter.aceti.NBRC.14818 GCA_000193495.2_ASM19349v2
Gluconacetobacter.diazotrophicus.PA1.5 GCA_000067045.1_ASM6704v1
Commensalibacter.intestini.A911 GCA_000231445.2_ASM23144v1
Granulibacter.bethesdensis.CGDNIH4 GCA_000576145.2_ASM57614v2
Acidisphaera.rubrifaciens.HS.AP3 GCA_000964365.1_ASM96436v1
Acidiphilium.multivorum.AIU301 GCA_000202835.1_ASM20283v1
Rubritepida.flocculans.DSM.14296 GCA_000425365.1_ASM42536v1
Belnapia.moabensis.DSM.16746 GCA_000745835.1_ASM74583v1
Roseomonas.gilardii GCA_001941945.1_ASM194194v1
Elioraea.tepidiphila.DSM.17972 GCA_000378465.1_ASM37846v1
Tistrella.mobilis.KA081020.065 GCA_000264455.2_ASM26445v2
Geminicoccus.roseus.DSM.18922 GCA_000427665.1_ASM42766v1
GCA_002937655.1_ASM293765v1*
GCA_002720535.1_ASM272053v1*
GCA_002699795.1_ASM269979v1*
GCA_002684605.1_ASM268460v1*
GCA_002715265.1_ASM271526v1*
GCA_002937695.1_ASM293769v1*
GCA_002714055.1_ASM271405v1*
GCA_002732015.1_ASM273201v1*
GCA_002171495.2_ASM217149v2*
GCA_002937625.1_ASM293762v1*
GCA_002720125.1_ASM272012v1*
GCA_002170595.1_ASM217059v1*
alpha.proteobacterium.HIMB5 GCA_000299095.1_ASM29909v1
Candidatus.Pelagibacter.ubique.HTCC1062 GCA_000012345.1_ASM1234v1
Candidatus.Pelagibacter.ubique.HIMB058 GCA_000419545.1_ASM41954v1
alpha.proteobacterium.HIMB114 GCA_000163555.2
Candidatus.Neoehrlichia.lotoris.str.RAC413 GCA_000964795.1_ASM96479v1
Anaplasma.phagocytophilum.str.HZ GCA_000013125.1_ASM1312v1
Ehrlichia.ruminantium.str.Welgevonden GCA_000026005.1_ASM2600v1
Wolbachia.endosymbiont.of.Drosophila.melanogaster GCA_000008025.1_ASM802v1
Candidatus.Deianiraea.vastatrix GCA_007993655.1_ASM799365v1
Rickettsiales.endosymbiont.of.Trichoplax.sp.H2 GCA_009690945.1
Candidatus.Midichloria.mitochondrii.IricVA GCA_000219355.1_ASM21935v1
Rickettsiales.endosymbiont.of.Peranema.trichophorum GCA_004210275.1_ASM421027v1
Candidatus.Jidaibacter.acanthamoeba GCA_000815465.1.UWC36
Rickettsia.bellii.RML369.C GCA_000012385.1_ASM1238v1
Candidatus.Megaira.polyxenophila
Rickettsiales.endosymbiont.of.Stachyamoeba.lipophora GCA_003932735.1_ASM393273v1
Rickettsiales.bacterium.Ac37b GCA_000746585.2_ASM74658v2
Candidatus.Paracaedibacter.acanthamoebae GCA_000742835.1_ASM74283v1
Candidatus.Odyssella.thessalonicensis.L13 GCA_000190415.2_ASM19041v1
Candidatus.Finniella.inopinata GCA_004210305.1_ASM421030v1
Candidatus.Caedibacter.acanthamoebae GCA_000743035.1_ASM74303v1
Caedimonas.varicaedens GCA_001192655.1_ASM119265v1
Candidatus.Nucleicultrix.amoebiphila.FS5 GCA_002117145.1_ASM211714v1
Holospora.undulata.HU1 GCA_000388175.3_ASM38817v3
Candidatus.Hepatobacter.penaei GCA_000742475.1_ASM74247v1
Magnetofaba.australis.IT.1 GCA_002109495.1_ASM210949v1
Magnetococcus.marinus.MC.1 GCA_000014865.1_ASM1486v1
Nitrosospira.briensis.C.128 GCA_000619905.2_ASM61990v2
Methylotenera.versatilis.301 GCA_000093025.1_ASM9302v1
Ralstonia.pickettii GCA_902374465.1.MGYG.HGUT.01384
Burkholderia.cepacia GCA_009586235.1_ASM958623v1
Dasania.marina.DSM.21967 GCA_000373485.1_ASM37348v1
Congregibacter.litoralis.KT71 GCA_000153125.2_ASM15312v2
Enterobacter.cloacae GCA_000770155.1_ASM77015v1
Alteromonas.macleodii.ATCC.27126 GCA_000172635.2_ASM17263v2
0.803252

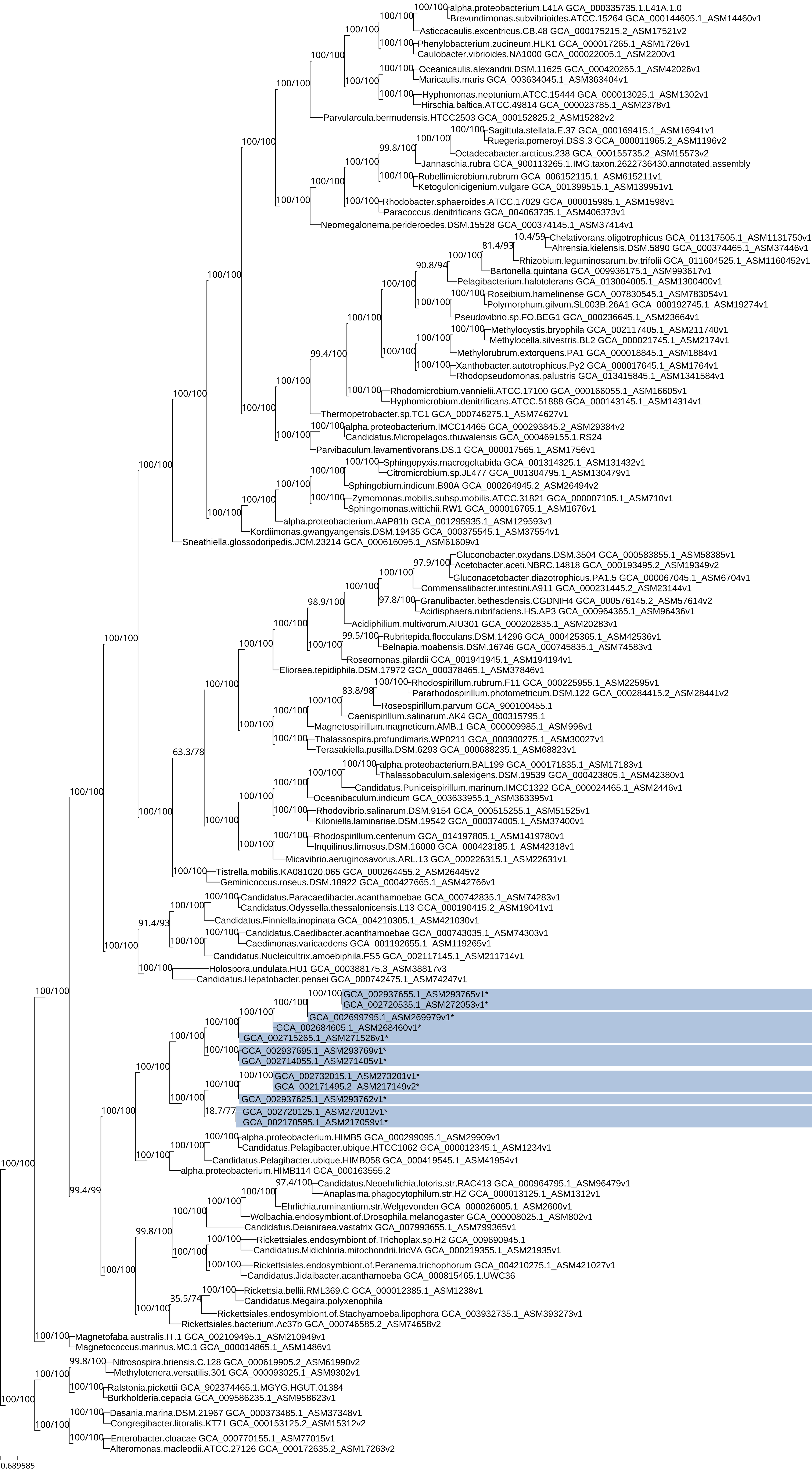
alpha.proteobacterium.L41A GCA_000335735.1.L41A.1.0
Brevundimonas.subvibrioides.ATCC.15264 GCA_000144605.1_ASM14460v1
Asticcacaulis.excentricus.CB.48 GCA_000175215.2_ASM17521v2
Phenylobacterium.zucineum.HLK1 GCA_000017265.1_ASM1726v1
Caulobacter.vibrioides.NA1000 GCA_000022005.1_ASM2200v1
Oceanicaulis.alexandrii.DSM.11625 GCA_000420265.1_ASM42026v1
Maricaulis.maris GCA_003634045.1_ASM363404v1
Hyphomonas.neptunium.ATCC.15444 GCA_000013025.1_ASM1302v1
Hirschia.baltica.ATCC.49814 GCA_000023785.1_ASM2378v1
Parvularcula.bermudensis.HTCC2503 GCA_000152825.2_ASM15282v2
Sagittula.stellata.E.37 GCA_000169415.1_ASM16941v1
Ruegeria.pomeroyi.DSS.3 GCA_000011965.2_ASM1196v2
Octadecabacter.arcticus.238 GCA_000155735.2_ASM15573v2
Jannaschia.rubra GCA_900113265.1.IMG.taxon.2622736430.annotated.assembly
Rubellimicrobium.rubrum GCA_006152115.1_ASM615211v1
Ketogulonicigenium.vulgare GCA_001399515.1_ASM139951v1
Rhodobacter.sphaeroides.ATCC.17029 GCA_000015985.1_ASM1598v1
Paracoccus.denitrificans GCA_004063735.1_ASM406373v1
Neomegalonema.perideroedes.DSM.15528 GCA_000374145.1_ASM37414v1
Chelativorans.oligotrophicus GCA_011317505.1_ASM1131750v1
Ahrensia.kielensis.DSM.5890 GCA_000374465.1_ASM37446v1
Rhizobium.leguminosarum.bv.trifolii GCA_011604525.1_ASM1160452v1
Bartonella.quintana GCA_009936175.1_ASM993617v1
Pelagibacterium.halotolerans GCA_013004005.1_ASM1300400v1
Roseibium.hamelinense GCA_007830545.1_ASM783054v1
Polymorphum.gilvum.SL003B.26A1 GCA_000192745.1_ASM19274v1
Pseudovibrio.sp.FO.BEG1 GCA_000236645.1_ASM23664v1
Methylocystis.bryophila GCA_002117405.1_ASM211740v1
Methylocella.silvestris.BL2 GCA_000021745.1_ASM2174v1
Methylorubrum.extorquens.PA1 GCA_000018845.1_ASM1884v1
Xanthobacter.autotrophicus.Py2 GCA_000017645.1_ASM1764v1
Rhodopseudomonas.palustris GCA_013415845.1_ASM1341584v1
Rhodomicrobium.vannielii.ATCC.17100 GCA_000166055.1_ASM16605v1
Hyphomicrobium.denitrificans.ATCC.51888 GCA_000143145.1_ASM14314v1
Thermopetrobacter.sp.TC1 GCA_000746275.1_ASM74627v1
alpha.proteobacterium.IMCC14465 GCA_000293845.2_ASM29384v2
Candidatus.Micropelagos.thuwalensis GCA_000469155.1.RS24
Parvibaculum.lavamentivorans.DS.1 GCA_000017565.1_ASM1756v1
Sphingopyxis.macrogoltabida GCA_001314325.1_ASM131432v1
Citromicrobium.sp.JL477 GCA_001304795.1_ASM130479v1
Sphingobium.indicum.B90A GCA_000264945.2_ASM26494v2
Zymomonas.mobilis.subsp.mobilis.ATCC.31821 GCA_000007105.1_ASM710v1
Sphingomonas.wittichii.RW1 GCA_000016765.1_ASM1676v1
alpha.proteobacterium.AAP81b GCA_001295935.1_ASM129593v1
Kordiimonas.gwangyangensis.DSM.19435 GCA_000375545.1_ASM37554v1
Sneathiella.glossodoripedis.JCM.23214 GCA_000616095.1_ASM61609v1
Gluconobacter.oxydans.DSM.3504 GCA_000583855.1_ASM58385v1
Acetobacter.aceti.NBRC.14818 GCA_000193495.2_ASM19349v2
Gluconacetobacter.diazotrophicus.PA1.5 GCA_000067045.1_ASM6704v1
Commensalibacter.intestini.A911 GCA_000231445.2_ASM23144v1
Granulibacter.bethesdensis.CGDNIH4 GCA_000576145.2_ASM57614v2
Acidisphaera.rubrifaciens.HS.AP3 GCA_000964365.1_ASM96436v1
Acidiphilium.multivorum.AIU301 GCA_000202835.1_ASM20283v1
Rubritepida.flocculans.DSM.14296 GCA_000425365.1_ASM42536v1
Belnapia.moabensis.DSM.16746 GCA_000745835.1_ASM74583v1
Roseomonas.gilardii GCA_001941945.1_ASM194194v1
Elioraea.tepidiphila.DSM.17972 GCA_000378465.1_ASM37846v1
Rhodospirillum.rubrum.F11 GCA_000225955.1_ASM22595v1
Pararhodospirillum.photometricum.DSM.122 GCA_000284415.2_ASM28441v2
Roseospirillum.parvum GCA_900100455.1
Caenispirillum.salinarum.AK4 GCA_000315795.1
Magnetospirillum.magneticum.AMB.1 GCA_000009985.1_ASM998v1
Thalassospira.profundimaris.WP0211 GCA_000300275.1_ASM30027v1
Terasakiella.pusilla.DSM.6293 GCA_000688235.1_ASM68823v1
alpha.proteobacterium.BAL199 GCA_000171835.1_ASM17183v1
Thalassobaculum.salexigens.DSM.19539 GCA_000423805.1_ASM42380v1
Candidatus.Puniceispirillum.marinum.IMCC1322 GCA_000024465.1_ASM2446v1
Oceanibaculum.indicum GCA_003633955.1_ASM363395v1
Rhodovibrio.salinarum.DSM.9154 GCA_000515255.1_ASM51525v1
Kiloniella.laminariae.DSM.19542 GCA_000374005.1_ASM37400v1
Rhodospirillum.centenum GCA_014197805.1_ASM1419780v1
Inquilinus.limosus.DSM.16000 GCA_000423185.1_ASM42318v1
Micavibrio.aeruginosavorus.ARL.13 GCA_000226315.1_ASM22631v1
Tistrella.mobilis.KA081020.065 GCA_000264455.2_ASM26445v2
Geminicoccus.roseus.DSM.18922 GCA_000427665.1_ASM42766v1
Candidatus.Paracaedibacter.acanthamoebae GCA_000742835.1_ASM74283v1
Candidatus.Odyssella.thessalonicensis.L13 GCA_000190415.2_ASM19041v1
Candidatus.Finniella.inopinata GCA_004210305.1_ASM421030v1
Candidatus.Caedibacter.acanthamoebae GCA_000743035.1_ASM74303v1
Caedimonas.varicaedens GCA_001192655.1_ASM119265v1
Candidatus.Nucleicultrix.amoebiphila.FS5 GCA_002117145.1_ASM211714v1
Holospora.undulata.HU1 GCA_000388175.3_ASM38817v3
Candidatus.Hepatobacter.penaei GCA_000742475.1_ASM74247v1
GCA_002937655.1_ASM293765v1*
GCA_002720535.1_ASM272053v1*
GCA_002699795.1_ASM269979v1*
GCA_002684605.1_ASM268460v1*
GCA_002715265.1_ASM271526v1*
GCA_002937695.1_ASM293769v1*
GCA_002714055.1_ASM271405v1*
GCA_002732015.1_ASM273201v1*
GCA_002171495.2_ASM217149v2*
GCA_002937625.1_ASM293762v1*
GCA_002720125.1_ASM272012v1*
GCA_002170595.1_ASM217059v1*
alpha.proteobacterium.HIMB5 GCA_000299095.1_ASM29909v1
Candidatus.Pelagibacter.ubique.HTCC1062 GCA_000012345.1_ASM1234v1
Candidatus.Pelagibacter.ubique.HIMB058 GCA_000419545.1_ASM41954v1
alpha.proteobacterium.HIMB114 GCA_000163555.2
Candidatus.Neoehrlichia.lotoris.str.RAC413 GCA_000964795.1_ASM96479v1
Anaplasma.phagocytophilum.str.HZ GCA_000013125.1_ASM1312v1
Ehrlichia.ruminantium.str.Welgevonden GCA_000026005.1_ASM2600v1
Wolbachia.endosymbiont.of.Drosophila.melanogaster GCA_000008025.1_ASM802v1
Candidatus.Deianiraea.vastatrix GCA_007993655.1_ASM799365v1
Rickettsiales.endosymbiont.of.Trichoplax.sp.H2 GCA_009690945.1
Candidatus.Midichloria.mitochondrii.IricVA GCA_000219355.1_ASM21935v1
Rickettsiales.endosymbiont.of.Peranema.trichophorum GCA_004210275.1_ASM421027v1
Candidatus.Jidaibacter.acanthamoeba GCA_000815465.1.UWC36
Rickettsia.bellii.RML369.C GCA_000012385.1_ASM1238v1
Candidatus.Megaira.polyxenophila
Rickettsiales.endosymbiont.of.Stachyamoeba.lipophora GCA_003932735.1_ASM393273v1
Rickettsiales.bacterium.Ac37b GCA_000746585.2_ASM74658v2
Magnetofaba.australis.IT.1 GCA_002109495.1_ASM210949v1
Magnetococcus.marinus.MC.1 GCA_000014865.1_ASM1486v1
Nitrosospira.briensis.C.128 GCA_000619905.2_ASM61990v2
Methylotenera.versatilis.301 GCA_000093025.1_ASM9302v1
Ralstonia.pickettii GCA_902374465.1.MGYG.HGUT.01384
Burkholderia.cepacia GCA_009586235.1_ASM958623v1
Dasania.marina.DSM.21967 GCA_000373485.1_ASM37348v1
Congregibacter.litoralis.KT71 GCA_000153125.2_ASM15312v2
Enterobacter.cloacae GCA_000770155.1_ASM77015v1
Alteromonas.macleodii.ATCC.27126 GCA_000172635.2_ASM17263v2
0.689585

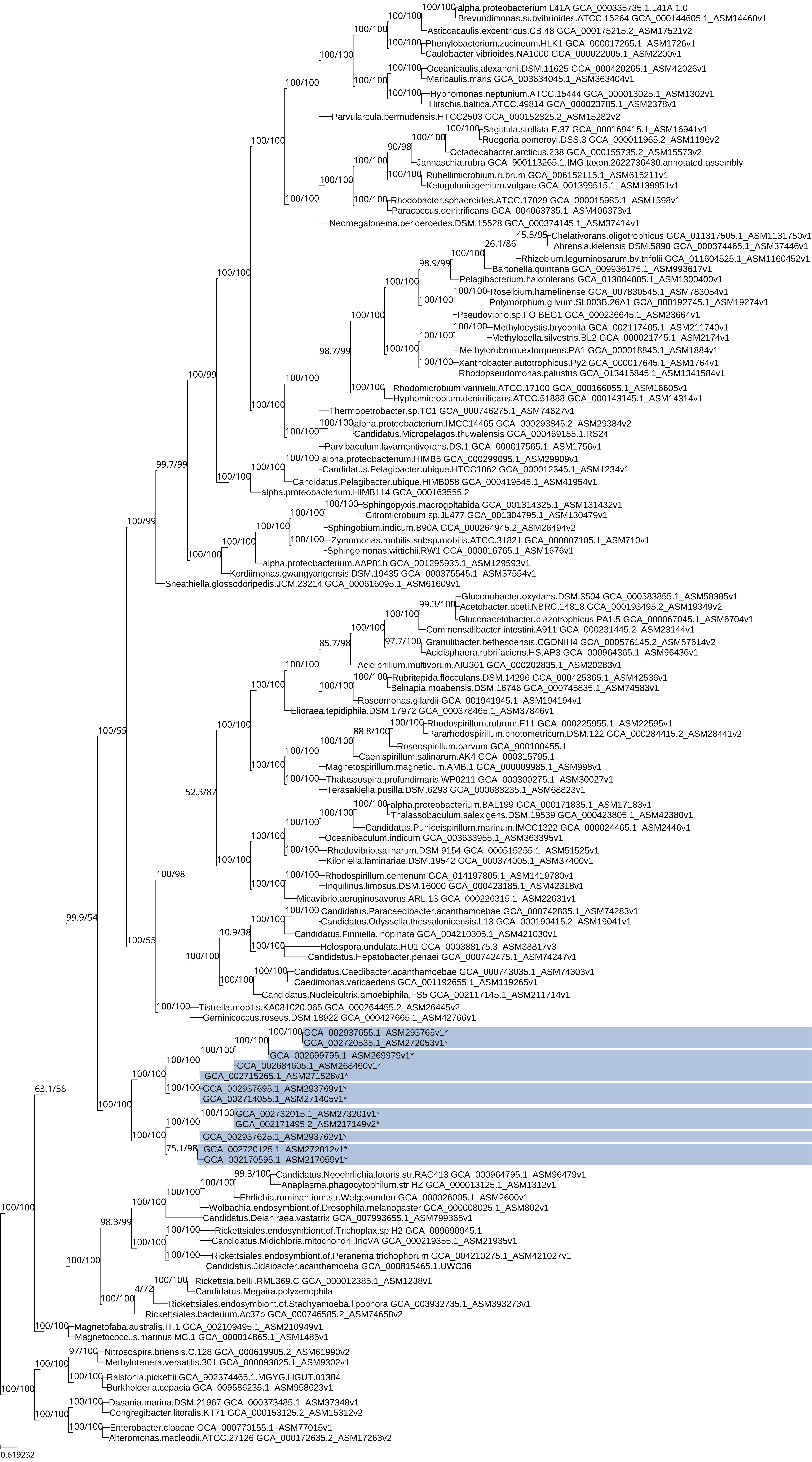
alpha.proteobacterium.L41A GCA_000335735.1.L41A.1.0
Brevundimonas.subvibrioides.ATCC.15264 GCA_000144605.1_ASM14460v1
Asticcacaulis.excentricus.CB.48 GCA_000175215.2_ASM17521v2
Phenylobacterium.zucineum.HLK1 GCA_000017265.1_ASM1726v1
Caulobacter.vibrioides.NA1000 GCA_000022005.1_ASM2200v1
Oceanicaulis.alexandrii.DSM.11625 GCA_000420265.1_ASM42026v1
Maricaulis.maris GCA_003634045.1_ASM363404v1
Hyphomonas.neptunium.ATCC.15444 GCA_000013025.1_ASM1302v1
Hirschia.baltica.ATCC.49814 GCA_000023785.1_ASM2378v1
Parvularcula.bermudensis.HTCC2503 GCA_000152825.2_ASM15282v2
Sagittula.stellata.E.37 GCA_000169415.1_ASM16941v1
Ruegeria.pomeroyi.DSS.3 GCA_000011965.2_ASM1196v2
Octadecabacter.arcticus.238 GCA_000155735.2_ASM15573v2
Jannaschia.rubra GCA_900113265.1.IMG.taxon.2622736430.annotated.assembly
Rubellimicrobium.rubrum GCA_006152115.1_ASM615211v1
Ketogulonicigenium.vulgare GCA_001399515.1_ASM139951v1
Rhodobacter.sphaeroides.ATCC.17029 GCA_000015985.1_ASM1598v1
Paracoccus.denitrificans GCA_004063735.1_ASM406373v1
Neomegalonema.perideroedes.DSM.15528 GCA_000374145.1_ASM37414v1
Chelativorans.oligotrophicus GCA_011317505.1_ASM1131750v1
Ahrensia.kielensis.DSM.5890 GCA_000374465.1_ASM37446v1
Rhizobium.leguminosarum.bv.trifolii GCA_011604525.1_ASM1160452v1
Bartonella.quintana GCA_009936175.1_ASM993617v1
Pelagibacterium.halotolerans GCA_013004005.1_ASM1300400v1
Roseibium.hamelinense GCA_007830545.1_ASM783054v1
Polymorphum.gilvum.SL003B.26A1 GCA_000192745.1_ASM19274v1
Pseudovibrio.sp.FO.BEG1 GCA_000236645.1_ASM23664v1
Methylocystis.bryophila GCA_002117405.1_ASM211740v1
Methylocella.silvestris.BL2 GCA_000021745.1_ASM2174v1
Methylorubrum.extorquens.PA1 GCA_000018845.1_ASM1884v1
Xanthobacter.autotrophicus.Py2 GCA_000017645.1_ASM1764v1
Rhodopseudomonas.palustris GCA_013415845.1_ASM1341584v1
Rhodomicrobium.vannielii.ATCC.17100 GCA_000166055.1_ASM16605v1
Hyphomicrobium.denitrificans.ATCC.51888 GCA_000143145.1_ASM14314v1
Thermopetrobacter.sp.TC1 GCA_000746275.1_ASM74627v1
alpha.proteobacterium.IMCC14465 GCA_000293845.2_ASM29384v2
Candidatus.Micropelagos.thuwalensis GCA_000469155.1.RS24
Parvibaculum.lavamentivorans.DS.1 GCA_000017565.1_ASM1756v1
alpha.proteobacterium.HIMB5 GCA_000299095.1_ASM29909v1
Candidatus.Pelagibacter.ubique.HTCC1062 GCA_000012345.1_ASM1234v1
Candidatus.Pelagibacter.ubique.HIMB058 GCA_000419545.1_ASM41954v1
alpha.proteobacterium.HIMB114 GCA_000163555.2
Sphingopyxis.macrogoltabida GCA_001314325.1_ASM131432v1
Citromicrobium.sp.JL477 GCA_001304795.1_ASM130479v1
Sphingobium.indicum.B90A GCA_000264945.2_ASM26494v2
Zymomonas.mobilis.subsp.mobilis.ATCC.31821 GCA_000007105.1_ASM710v1
Sphingomonas.wittichii.RW1 GCA_000016765.1_ASM1676v1
alpha.proteobacterium.AAP81b GCA_001295935.1_ASM129593v1
Kordiimonas.gwangyangensis.DSM.19435 GCA_000375545.1_ASM37554v1
Sneathiella.glossodoripedis.JCM.23214 GCA_000616095.1_ASM61609v1
Gluconobacter.oxydans.DSM.3504 GCA_000583855.1_ASM58385v1
Acetobacter.aceti.NBRC.14818 GCA_000193495.2_ASM19349v2
Gluconacetobacter.diazotrophicus.PA1.5 GCA_000067045.1_ASM6704v1
Commensalibacter.intestini.A911 GCA_000231445.2_ASM23144v1
Granulibacter.bethesdensis.CGDNIH4 GCA_000576145.2_ASM57614v2
Acidisphaera.rubrifaciens.HS.AP3 GCA_000964365.1_ASM96436v1
Acidiphilium.multivorum.AIU301 GCA_000202835.1_ASM20283v1
Rubritepida.flocculans.DSM.14296 GCA_000425365.1_ASM42536v1
Belnapia.moabensis.DSM.16746 GCA_000745835.1_ASM74583v1
Roseomonas.gilardii GCA_001941945.1_ASM194194v1
Elioraea.tepidiphila.DSM.17972 GCA_000378465.1_ASM37846v1
Rhodospirillum.rubrum.F11 GCA_000225955.1_ASM22595v1
Pararhodospirillum.photometricum.DSM.122 GCA_000284415.2_ASM28441v2
Roseospirillum.parvum GCA_900100455.1
Caenispirillum.salinarum.AK4 GCA_000315795.1
Magnetospirillum.magneticum.AMB.1 GCA_000009985.1_ASM998v1
Thalassospira.profundimaris.WP0211 GCA_000300275.1_ASM30027v1
Terasakiella.pusilla.DSM.6293 GCA_000688235.1_ASM68823v1
alpha.proteobacterium.BAL199 GCA_000171835.1_ASM17183v1
Thalassobaculum.salexigens.DSM.19539 GCA_000423805.1_ASM42380v1
Candidatus.Puniceispirillum.marinum.IMCC1322 GCA_000024465.1_ASM2446v1
Oceanibaculum.indicum GCA_003633955.1_ASM363395v1
Rhodovibrio.salinarum.DSM.9154 GCA_000515255.1_ASM51525v1
Kiloniella.laminariae.DSM.19542 GCA_000374005.1_ASM37400v1
Rhodospirillum.centenum GCA_014197805.1_ASM1419780v1
Inquilinus.limosus.DSM.16000 GCA_000423185.1_ASM42318v1
Micavibrio.aeruginosavorus.ARL.13 GCA_000226315.1_ASM22631v1
Candidatus.Paracaedibacter.acanthamoebae GCA_000742835.1_ASM74283v1
Candidatus.Odyssella.thessalonicensis.L13 GCA_000190415.2_ASM19041v1
Candidatus.Finniella.inopinata GCA_004210305.1_ASM421030v1
Holospora.undulata.HU1 GCA_000388175.3_ASM38817v3
Candidatus.Hepatobacter.penaei GCA_000742475.1_ASM74247v1
Candidatus.Caedibacter.acanthamoebae GCA_000743035.1_ASM74303v1
Caedimonas.varicaedens GCA_001192655.1_ASM119265v1
Candidatus.Nucleicultrix.amoebiphila.FS5 GCA_002117145.1_ASM211714v1
Tistrella.mobilis.KA081020.065 GCA_000264455.2_ASM26445v2
Geminicoccus.roseus.DSM.18922 GCA_000427665.1_ASM42766v1
GCA_002937655.1_ASM293765v1*
GCA_002720535.1_ASM272053v1*
GCA_002699795.1_ASM269979v1*
GCA_002684605.1_ASM268460v1*
GCA_002715265.1_ASM271526v1*
GCA_002937695.1_ASM293769v1*
GCA_002714055.1_ASM271405v1*
GCA_002732015.1_ASM273201v1*
GCA_002171495.2_ASM217149v2*
GCA_002937625.1_ASM293762v1*
GCA_002720125.1_ASM272012v1*
GCA_002170595.1_ASM217059v1*
Candidatus.Neoehrlichia.lotoris.str.RAC413 GCA_000964795.1_ASM96479v1
Anaplasma.phagocytophilum.str.HZ GCA_000013125.1_ASM1312v1
Ehrlichia.ruminantium.str.Welgevonden GCA_000026005.1_ASM2600v1
Wolbachia.endosymbiont.of.Drosophila.melanogaster GCA_000008025.1_ASM802v1
Candidatus.Deianiraea.vastatrix GCA_007993655.1_ASM799365v1
Rickettsiales.endosymbiont.of.Trichoplax.sp.H2 GCA_009690945.1
Candidatus.Midichloria.mitochondrii.IricVA GCA_000219355.1_ASM21935v1
Rickettsiales.endosymbiont.of.Peranema.trichophorum GCA_004210275.1_ASM421027v1
Candidatus.Jidaibacter.acanthamoeba GCA_000815465.1.UWC36
Rickettsia.bellii.RML369.C GCA_000012385.1_ASM1238v1
Candidatus.Megaira.polyxenophila
Rickettsiales.endosymbiont.of.Stachyamoeba.lipophora GCA_003932735.1_ASM393273v1
Rickettsiales.bacterium.Ac37b GCA_000746585.2_ASM74658v2
Magnetofaba.australis.IT.1 GCA_002109495.1_ASM210949v1
Magnetococcus.marinus.MC.1 GCA_000014865.1_ASM1486v1
Nitrosospira.briensis.C.128 GCA_000619905.2_ASM61990v2
Methylotenera.versatilis.301 GCA_000093025.1_ASM9302v1
Ralstonia.pickettii GCA_902374465.1.MGYG.HGUT.01384
Burkholderia.cepacia GCA_009586235.1_ASM958623v1
Dasania.marina.DSM.21967 GCA_000373485.1_ASM37348v1
Congregibacter.litoralis.KT71 GCA_000153125.2_ASM15312v2
Enterobacter.cloacae GCA_000770155.1_ASM77015v1
Alteromonas.macleodii.ATCC.27126 GCA_000172635.2_ASM17263v2
0.619232

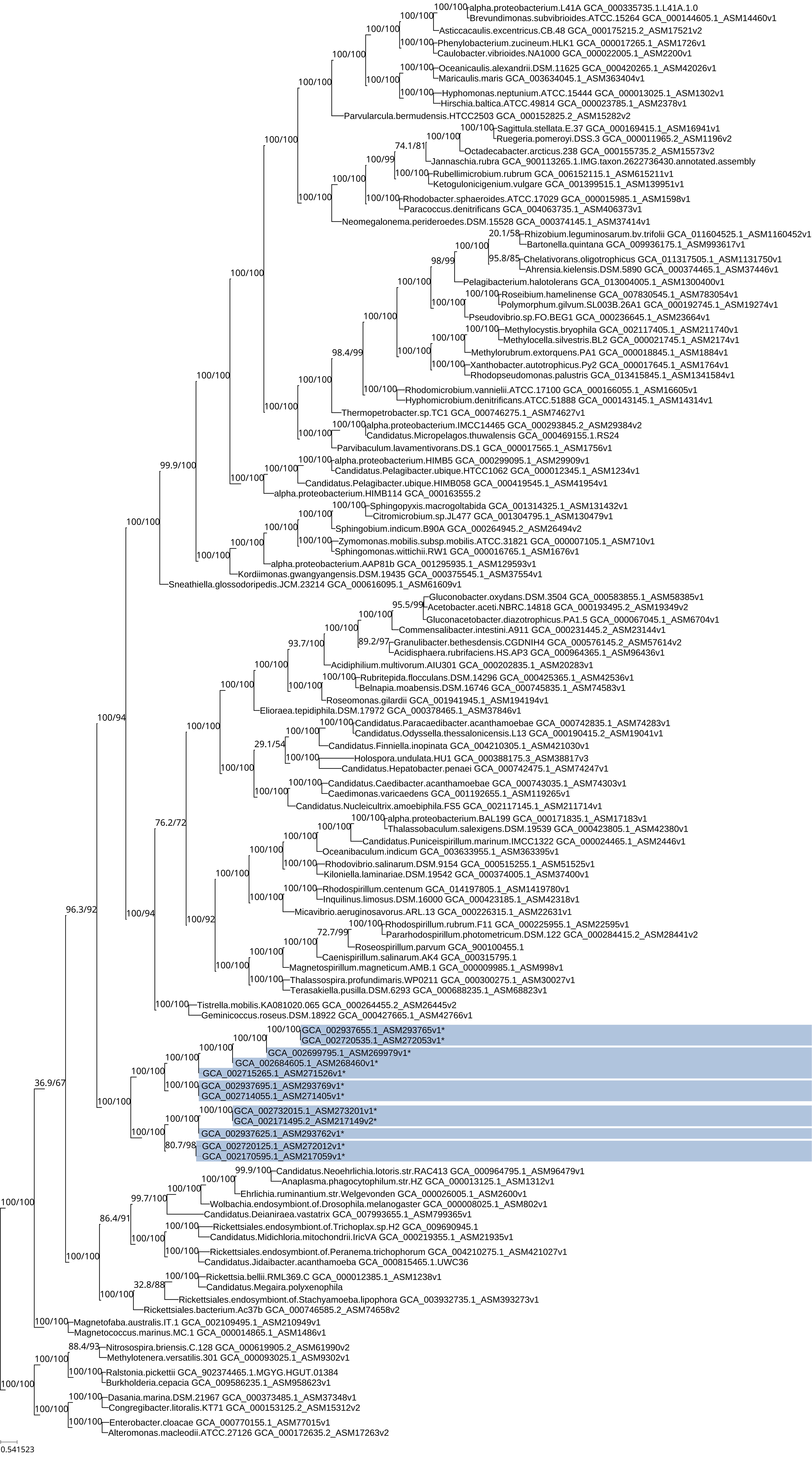
100/100 alpha.proteobacterium.L41A GCA_000335735.1.L41A.1.0
100/100 Brevundimonas.subvibrioides.ATCC.15264 GCA_000144605.1_ASM14460v1
100/100 Asticcacaulis.excentricus.CB.48 GCA_000175215.2_ASM17521v2
100/100 Phenylobacterium.zucineum.HLK1 GCA_000017265.1_ASM1726v1
Caulobacter.vibrioides.NA1000 GCA_000022005.1_ASM2200v1
100/100
100/100 Oceanicaulis.alexandrii.DSM.11625 GCA_000420265.1_ASM42026v1
100/100 Maricaulis.maris GCA_003634045.1_ASM363404v1
100/100
100/100 Hyphomonas.neptunium.ATCC.15444 GCA_000013025.1_ASM1302v1
Hirschia.baltica.ATCC.49814 GCA_000023785.1_ASM2378v1
Parvularcula.bermudensis.HTCC2503 GCA_000152825.2_ASM15282v2
100/100 Sagittula.stellata.E.37 GCA_000169415.1_ASM16941v1
100/100
Ruegeria.pomeroyi.DSS.3 GCA_000011965.2_ASM1196v2
100/100
74.1/81 Octadecabacter.arcticus.238 GCA_000155735.2_ASM15573v2
Jannaschia.rubra GCA_900113265.1.IMG.taxon.2622736430.annotated.assembly
100/99
100/100 Rubellimicrobium.rubrum GCA_006152115.1_ASM615211v1
100/100 Ketogulonicigenium.vulgare GCA_001399515.1_ASM139951v1
100/100 Rhodobacter.sphaeroides.ATCC.17029 GCA_000015985.1_ASM1598v1
100/100 Paracoccus.denitrificans GCA_004063735.1_ASM406373v1
Neomegalonema.perideroedes.DSM.15528 GCA_000374145.1_ASM37414v1
20.1/58 Rhizobium.leguminosarum.bv.trifolii GCA_011604525.1_ASM1160452v1
100/100 Bartonella.quintana GCA_009936175.1_ASM993617v1
95.8/85 Chelativorans.oligotrophicus GCA_011317505.1_ASM1131750v1
98/99 Ahrensia.kielensis.DSM.5890 GCA_000374465.1_ASM37446v1
100/100 Pelagibacterium.halotolerans GCA_013004005.1_ASM1300400v1
100/100 Roseibium.hamelinense GCA_007830545.1_ASM783054v1
100/100 Polymorphum.gilvum.SL003B.26A1 GCA_000192745.1_ASM19274v1
100/100 Pseudovibrio.sp.FO.BEG1 GCA_000236645.1_ASM23664v1
100/100 Methylocystis.bryophila GCA_002117405.1_ASM211740v1
100/100 Methylocella.silvestris.BL2 GCA_000021745.1_ASM2174v1
98.4/99 100/100 Methylorubrum.extorquens.PA1 GCA_000018845.1_ASM1884v1
100/100 Xanthobacter.autotrophicus.Py2 GCA_000017645.1_ASM1764v1
Rhodopseudomonas.palustris GCA_013415845.1_ASM1341584v1
100/100
100/100 Rhodomicrobium.vannielii.ATCC.17100 GCA_000166055.1_ASM16605v1
Hyphomicrobium.denitrificans.ATCC.51888 GCA_000143145.1_ASM14314v1
100/100 Thermopetrobacter.sp.TC1 GCA_000746275.1_ASM74627v1
100/100 alpha.proteobacterium.IMCC14465 GCA_000293845.2_ASM29384v2
100/100 Candidatus.Micropelagos.thuwalensis GCA_000469155.1.RS24
Parvibaculum.lavamentivorans.DS.1 GCA_000017565.1_ASM1756v1
99.9/100 100/100 alpha.proteobacterium.HIMB5 GCA_000299095.1_ASM29909v1
100/100 Candidatus.Pelagibacter.ubique.HTCC1062 GCA_000012345.1_ASM1234v1
100/100 Candidatus.Pelagibacter.ubique.HIMB058 GCA_000419545.1_ASM41954v1
alpha.proteobacterium.HIMB114 GCA_000163555.2
100/100 100/100 Sphingopyxis.macrogoltabida GCA_001314325.1_ASM131432v1
100/100 Citromicrobium.sp.JL477 GCA_001304795.1_ASM130479v1
100/100 Sphingobium.indicum.B90A GCA_000264945.2_ASM26494v2
100/100 100/100 Zymomonas.mobilis.subsp.mobilis.ATCC.31821 GCA_000007105.1_ASM710v1
Sphingomonas.wittichii.RW1 GCA_000016765.1_ASM1676v1
100/100 alpha.proteobacterium.AAP81b GCA_001295935.1_ASM129593v1
Kordiimonas.gwangyangensis.DSM.19435 GCA_000375545.1_ASM37554v1
Sneathiella.glossodoripedis.JCM.23214 GCA_000616095.1_ASM61609v1
95.5/99 Gluconobacter.oxydans.DSM.3504 GCA_000583855.1_ASM58385v1
Acetobacter.aceti.NBRC.14818 GCA_000193495.2_ASM19349v2
100/100 Gluconacetobacter.diazotrophicus.PA1.5 GCA_000067045.1_ASM6704v1
100/100 Commensalibacter.intestini.A911 GCA_000231445.2_ASM23144v1
93.7/100 89.2/97 Granulibacter.bethesdensis.CGDNIH4 GCA_000576145.2_ASM57614v2
Acidisphaera.rubrifaciens.HS.AP3 GCA_000964365.1_ASM96436v1
100/100 Acidiphilium.multivorum.AIU301 GCA_000202835.1_ASM20283v1
100/100 Rubritepida.flocculans.DSM.14296 GCA_000425365.1_ASM42536v1
100/100 100/100 Belnapia.moabensis.DSM.16746 GCA_000745835.1_ASM74583v1
100/94 Roseomonas.gilardii GCA_001941945.1_ASM194194v1
Elioraea.tepidiphila.DSM.17972 GCA_000378465.1_ASM37846v1
100/100 Candidatus.Paracaedibacter.acanthamoebae GCA_000742835.1_ASM74283v1
100/100 100/100 Candidatus.Odyssella.thessalonicensis.L13 GCA_000190415.2_ASM19041v1
29.1/54 Candidatus.Finniella.inopinata GCA_004210305.1_ASM421030v1
100/100 Holospora.undulata.HU1 GCA_000388175.3_ASM38817v3
100/100 Candidatus.Hepatobacter.penaei GCA_000742475.1_ASM74247v1
100/100 Candidatus.Caedibacter.acanthamoebae GCA_000743035.1_ASM74303v1
100/100 Caedimonas.varicaedens GCA_001192655.1_ASM119265v1
Candidatus.Nucleicultrix.amoebiphila.FS5 GCA_002117145.1_ASM211714v1
76.2/72 100/100 alpha.proteobacterium.BAL199 GCA_000171835.1_ASM17183v1
100/100 Thalassobaculum.salexigens.DSM.19539 GCA_000423805.1_ASM42380v1
100/100 Candidatus.Puniceispirillum.marinum.IMCC1322 GCA_000024465.1_ASM2446v1
100/100 Oceanibaculum.indicum GCA_003633955.1_ASM363395v1
100/100 Rhodovibrio.salinarum.DSM.9154 GCA_000515255.1_ASM51525v1
100/100 Kiloniella.laminariae.DSM.19542 GCA_000374005.1_ASM37400v1
100/100 Rhodospirillum.centenum GCA_014197805.1_ASM1419780v1
96.3/92 100/100 Inquilinus.limosus.DSM.16000 GCA_000423185.1_ASM42318v1
Micavibrio.aeruginosavorus.ARL.13 GCA_000226315.1_ASM22631v1
100/94 100/92 100/100 Rhodospirillum.rubrum.F11 GCA_000225955.1_ASM22595v1
72.7/99 Pararhodospirillum.photometricum.DSM.122 GCA_000284415.2_ASM28441v2
100/100 Roseospirillum.parvum GCA_900100455.1
100/100 Caenispirillum.salinarum.AK4 GCA_000315795.1
100/100 Magnetospirillum.magneticum.AMB.1 GCA_000009985.1_ASM998v1
100/100 Thalassospira.profundimaris.WP0211 GCA_000300275.1_ASM30027v1
Terasakiella.pusilla.DSM.6293 GCA_000688235.1_ASM68823v1
100/100 Tistrella.mobilis.KA081020.065 GCA_000264455.2_ASM26445v2
Geminicoccus.roseus.DSM.18922 GCA_000427665.1_ASM42766v1
100/100 GCA_002937655.1_ASM293765v1*
100/100 GCA_002720535.1_ASM272053v1*
100/100 GCA_002699795.1_ASM269979v1*
100/100 GCA_002684605.1_ASM268460v1*
100/100 GCA_002715265.1_ASM271526v1*
100/100 GCA_002937695.1_ASM293769v1*
36.9/67 GCA_002714055.1_ASM271405v1*
100/100 100/100 GCA_002732015.1_ASM273201v1*
100/100 GCA_002171495.2_ASM217149v2*
100/100 GCA_002937625.1_ASM293762v1*
80.7/98 GCA_002720125.1_ASM272012v1*
GCA_002170595.1_ASM217059v1*
99.9/100 Candidatus.Neoehrlichia.lotoris.str.RAC413 GCA_000964795.1_ASM96479v1
100/100 Anaplasma.phagocytophilum.str.HZ GCA_000013125.1_ASM1312v1
100/100 Ehrlichia.ruminantium.str.Welgevonden GCA_000026005.1_ASM2600v1
100/100 99.7/100 Wolbachia.endosymbiont.of.Drosophila.melanogaster GCA_000008025.1_ASM802v1
Candidatus.Deianiraea.vastatrix GCA_007993655.1_ASM799365v1
86.4/91 100/100 Rickettsiales.endosymbiont.of.Trichoplax.sp.H2 GCA_009690945.1
100/100 Candidatus.Midichloria.mitochondrii.IricVA GCA_000219355.1_ASM21935v1
100/100 Rickettsiales.endosymbiont.of.Peranema.trichophorum GCA_004210275.1_ASM421027v1
100/100 Candidatus.Jidaibacter.acanthamoeba GCA_000815465.1.UWC36
100/100 Rickettsia.bellii.RML369.C GCA_000012385.1_ASM1238v1
32.8/88 Candidatus.Megaira.polyxenophila
100/100 Rickettsiales.endosymbiont.of.Stachyamoeba.lipophora GCA_003932735.1_ASM393273v1
Rickettsiales.bacterium.Ac37b GCA_000746585.2_ASM74658v2
100/100 Magnetofaba.australis.IT.1 GCA_002109495.1_ASM210949v1
Magnetococcus.marinus.MC.1 GCA_000014865.1_ASM1486v1
88.4/93 Nitrosospira.briensis.C.128 GCA_000619905.2_ASM61990v2
100/100 Methylotenera.versatilis.301 GCA_000093025.1_ASM9302v1
100/100 Ralstonia.pickettii GCA_902374465.1.MGYG.HGUT.01384
100/100 Burkholderia.cepacia GCA_009586235.1_ASM958623v1
100/100 Dasania.marina.DSM.21967 GCA_000373485.1_ASM37348v1
100/100 Congregibacter.litoralis.KT71 GCA_000153125.2_ASM15312v2
100/100 Enterobacter.cloacae GCA_000770155.1_ASM77015v1
Alteromonas.macleodii.ATCC.27126 GCA_000172635.2_ASM17263v2
0.541523

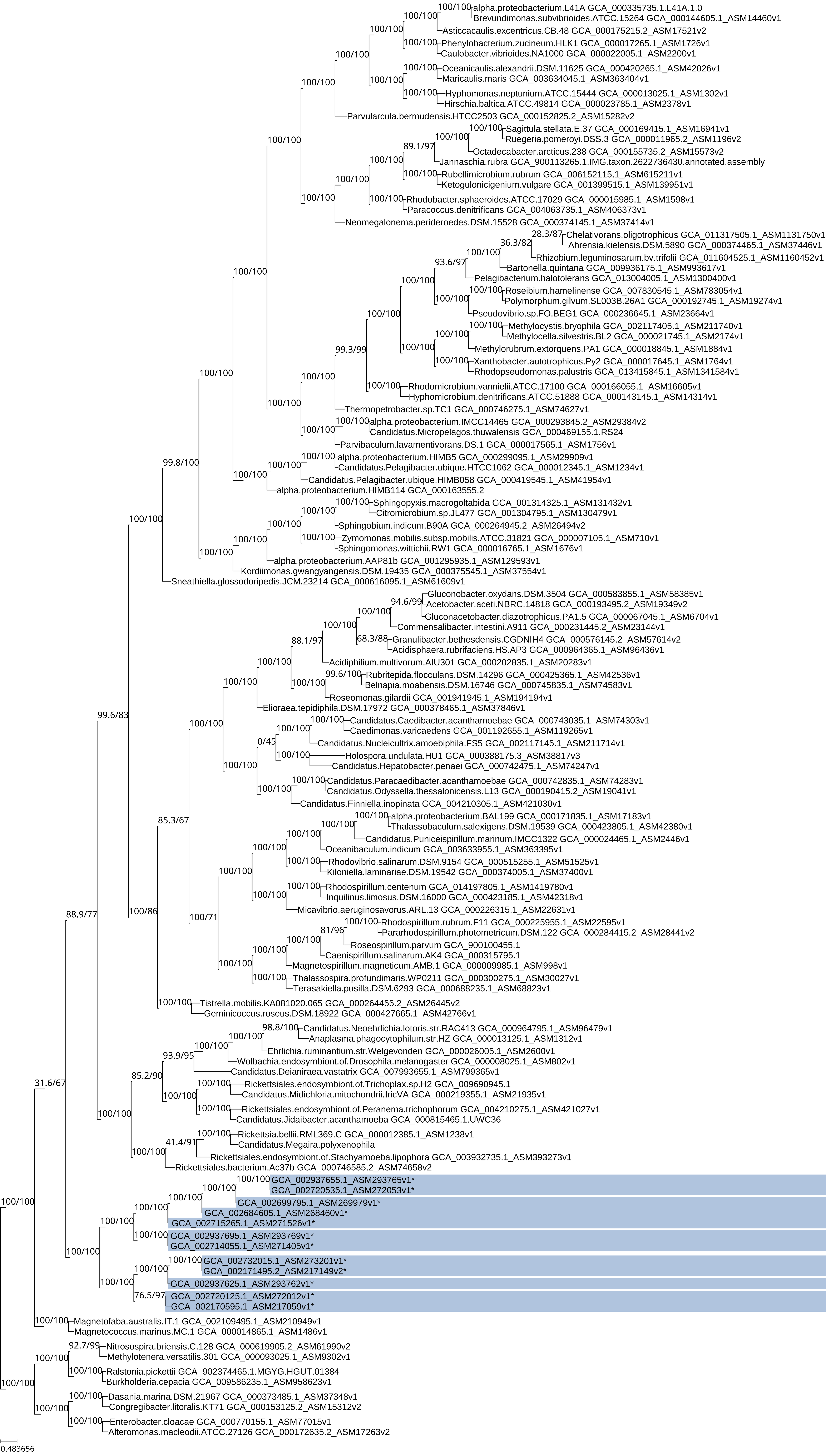
100/100
alpha.proteobacterium.L41A GCA_000335735.1.L41A.1.0
100/100
Brevundimonas.subvibrioides.ATCC.15264 GCA_000144605.1_ASM14460v1
100/100
Asticcacaulis.excentricus.CB.48 GCA_000175215.2_ASM17521v2
100/100
Phenylobacterium.zucineum.HLK1 GCA_000017265.1_ASM1726v1
Caulobacter.vibrioides.NA1000 GCA_000022005.1_ASM2200v1
100/100
100/100
Oceanicaulis.alexandrii.DSM.11625 GCA_000420265.1_ASM42026v1
100/100
Maricaulis.maris GCA_003634045.1_ASM363404v1
100/100
100/100
Hyphomonas.neptunium.ATCC.15444 GCA_000013025.1_ASM1302v1
Hirschia.baltica.ATCC.49814 GCA_000023785.1_ASM2378v1
Parvularcula.bermudensis.HTCC2503 GCA_000152825.2_ASM15282v2
100/100
Sagittula.stellata.E.37 GCA_000169415.1_ASM16941v1
100/100
Ruegeria.pomeroyi.DSS.3 GCA_000011965.2_ASM1196v2
100/100
Octadecabacter.arcticus.238 GCA_000155735.2_ASM15573v2
89.1/97
Jannaschia.rubra GCA_900113265.1_IMG.taxon.2622736430.annotated.assembly
100/100
100/100
Rubellimicrobium.rubrum GCA_006152115.1_ASM615211v1
100/100
Ketogulonicigenium.vulgare GCA_001399515.1_ASM139951v1
100/100
100/100
Rhodobacter.sphaeroides.ATCC.17029 GCA_000015985.1_ASM1598v1
Paracoccus.denitrificans GCA_004063735.1_ASM406373v1
Neomegalonema.perideroedes.DSM.15528 GCA_000374145.1_ASM37414v1
28.3/87
Chelativorans.oligotrophicus GCA_011317505.1_ASM1131750v1
36.3/82
Ahrensia.kielensis.DSM.5890 GCA_000374465.1_ASM37446v1
100/100
Rhizobium.leguminosarum.bv.trifolii GCA_011604525.1_ASM1160452v1
100/100
Bartonella.quintana GCA_009936175.1_ASM993617v1
93.6/97
Pelagibacterium.halotolerans GCA_013004005.1_ASM1300400v1
100/100
Roseibium.hamelinense GCA_007830545.1_ASM783054v1
100/100
100/100
Polymorphum.gilvum.SL003B.26A1 GCA_000192745.1_ASM19274v1
100/100
Pseudovibrio.sp.FO.BEG1 GCA_000236645.1_ASM23664v1
100/100
Methylocystis.bryophila GCA_002117405.1_ASM211740v1
100/100
Methylocella.silvestris.BL2 GCA_000021745.1_ASM2174v1
99.3/99
100/100
Methylorubrum.extorquens.PA1 GCA_000018845.1_ASM1884v1
100/100
Xanthobacter.autotrophicus.Py2 GCA_000017645.1_ASM1764v1
100/100
Rhodopseudomonas.palustris GCA_013415845.1_ASM1341584v1
100/100
100/100
Rhodomicrobium.vannielii.ATCC.17100 GCA_000166055.1_ASM16605v1
Hyphomicrobium.denitrificans.ATCC.51888 GCA_000143145.1_ASM14314v1
100/100
Thermopetrobacter.sp.TC1 GCA_000746275.1_ASM74627v1
100/100
100/100
alpha.proteobacterium.IMCC14465 GCA_000293845.2_ASM29384v2
Candidatus.Micropelagos.thuwalensis GCA_000469155.1.RS24
100/100
Parvibaculum.lavamentivorans.DS.1 GCA_000017565.1_ASM1756v1
100/100
alpha.proteobacterium.HIMB5 GCA_000299095.1_ASM29909v1
99.8/100
100/100
Candidatus.Pelagibacter.ubique.HTCC1062 GCA_000012345.1_ASM1234v1
100/100
Candidatus.Pelagibacter.ubique.HIMB058 GCA_000419545.1_ASM41954v1
alpha.proteobacterium.HIMB114 GCA_000163555.2
100/100
Sphingopyxis.macrogoltabida GCA_001314325.1_ASM131432v1
100/100
Citromicrobium.sp.JL477 GCA_001304795.1_ASM130479v1
100/100
Sphingobium.indicum.B90A GCA_000264945.2_ASM26494v2
100/100
100/100
Zymomonas.mobilis.subsp.mobilis.ATCC.31821 GCA_000007105.1_ASM710v1
100/100
Sphingomonas.wittichii.RW1 GCA_000016765.1_ASM1676v1
100/100
alpha.proteobacterium.AAP81b GCA_001295935.1_ASM129593v1
Kordiimonas.gwangyangensis.DSM.19435 GCA_000375545.1_ASM37554v1
Sneathiella.glossodoripedis.JCM.23214 GCA_000616095.1_ASM61609v1
Gluconobacter.oxydans.DSM.3504 GCA_000583855.1_ASM58385v1
94.6/99
Acetobacter.aceti.NBRC.14818 GCA_000193495.2_ASM19349v2
100/100
Gluconacetobacter.diazotrophicus.PA1.5 GCA_000067045.1_ASM6704v1
100/100
Commensalibacter.intestini.A911 GCA_000231445.2_ASM23144v1
88.1/97
68.3/88
Granulibacter.bethesdensis.CGDNIH4 GCA_000576145.2_ASM57614v2
Acidisphaera.rubrifaciens.HS.AP3 GCA_000964365.1_ASM96436v1
100/100
Acidiphilium.multivorum.AIU301 GCA_000202835.1_ASM20283v1
99.6/100
Rubritepida.flocculans.DSM.14296 GCA_000425365.1_ASM42536v1
100/100
100/100
Belnapia.moabensis.DSM.16746 GCA_000745835.1_ASM74583v1
99.6/83
Roseomonas.gilardii GCA_001941945.1_ASM194194v1
Elioraea.tepidiphila.DSM.17972 GCA_000378465.1_ASM37846v1
100/100
100/100
Candidatus.Caedibacter.acanthamoebae GCA_000743035.1_ASM74303v1
100/100
Caedimonas.varicaedens GCA_001192655.1_ASM119265v1
0/45
Candidatus.Nucleicultrix.amoebiphila.FS5 GCA_002117145.1_ASM211714v1
100/100
Holospora.undulata.HU1 GCA_000388175.3_ASM38817v3
100/100
Candidatus.Hepatobacter.penaei GCA_000742475.1_ASM74247v1
100/100
Candidatus.Paracaedibacter.acanthamoebae GCA_000742835.1_ASM74283v1
100/100
Candidatus.Odyssella.thessalonicensis.L13 GCA_000190415.2_ASM19041v1
Candidatus.Finniella.inopinata GCA_004210305.1_ASM421030v1
85.3/67
100/100
alpha.proteobacterium.BAL199 GCA_000171835.1_ASM17183v1
100/100
Thalassobaculum.salexigens.DSM.19539 GCA_000423805.1_ASM42380v1
100/100
Candidatus.Puniceispirillum.marinum.IMCC1322 GCA_000024465.1_ASM2446v1
100/100
Oceanibaculum.indicum GCA_003633955.1_ASM363395v1
100/100
100/100
Rhodovibrio.salinarum.DSM.9154 GCA_000515255.1_ASM51525v1
Kiloniella.laminariae.DSM.19542 GCA_000374005.1_ASM37400v1
100/100
Rhodospirillum.centenum GCA_014197805.1_ASM1419780v1
100/100
Inquilinus.limosus.DSM.16000 GCA_000423185.1_ASM42318v1
100/86
Micavibrio.aeruginosavorus.ARL.13 GCA_000226315.1_ASM22631v1
100/71
100/100
Rhodospirillum.rubrum.F11 GCA_000225955.1_ASM22595v1
88.9/77
81/96
Pararhodospirillum.photometricum.DSM.122 GCA_000284415.2_ASM28441v2
100/100
Roseospirillum.parvum GCA_900100455.1
100/100
Caenispirillum.salinarum.AK4 GCA_000315795.1
100/100
Magnetospirillum.magneticum.AMB.1 GCA_000009985.1_ASM998v1
100/100
Thalassospira.profundimaris.WP0211 GCA_000300275.1_ASM30027v1
Terasakiella.pusilla.DSM.6293 GCA_000688235.1_ASM68823v1
100/100
Tistrella.mobilis.KA081020.065 GCA_000264455.2_ASM26445v2
Geminicoccus.roseus.DSM.18922 GCA_000427665.1_ASM42766v1
98.8/100
Candidatus.Neoehrlichia.lotoris.str.RAC413 GCA_000964795.1_ASM96479v1
100/100
Anaplasma.phagocytophilum.str.HZ GCA_000013125.1_ASM1312v1
100/100
Ehrlichia.ruminantium.str.Welgevonden GCA_000026005.1_ASM2600v1
93.9/95
Wolbachia.endosymbiont.of.Drosophila.melanogaster GCA_000008025.1_ASM802v1
Candidatus.Deianiraea.vastatrix GCA_007993655.1_ASM799365v1
31.6/67
85.2/90
100/100
Rickettsiales.endosymbiont.of.Trichoplax.sp.H2 GCA_009690945.1
100/100
Candidatus.Midichloria.mitochondrii.IricVA GCA_000219355.1_ASM21935v1
100/100
Rickettsiales.endosymbiont.of.Peranema.trichophorum GCA_004210275.1_ASM421027v1
100/100
Candidatus.Jidaibacter.acanthamoeba GCA_000815465.1.UWC36
100/100
Rickettsia.bellii.RML369.C GCA_000012385.1_ASM1238v1
41.4/91
Candidatus.Megaira.polyxenophila
100/100
Rickettsiales.endosymbiont.of.Stachyamoeba.lipophora GCA_003932735.1_ASM393273v1
Rickettsiales.bacterium.Ac37b GCA_000746585.2_ASM74658v2
100/100
GCA_002937655.1_ASM293765v1*
100/100
GCA_002720535.1_ASM272053v1*
100/100
GCA_002699795.1_ASM269979v1*
100/100
GCA_002684605.1_ASM268460v1*
100/100
100/100
GCA_002715265.1_ASM271526v1*
100/100
GCA_002937695.1_ASM293769v1*
100/100
GCA_002714055.1_ASM271405v1*
100/100
GCA_002732015.1_ASM273201v1*
100/100
GCA_002171495.2_ASM217149v2*
100/100
GCA_002937625.1_ASM293762v1*
76.5/97
GCA_002720125.1_ASM272012v1*
GCA_002170595.1_ASM217059v1*
100/100
Magnetofaba.australis.IT.1 GCA_002109495.1_ASM210949v1
Magnetococcus.marinus.MC.1 GCA_000014865.1_ASM1486v1
92.7/99
Nitrosospira.briensis.C.128 GCA_000619905.2_ASM61990v2
100/100
Methylotenera.versatilis.301 GCA_000093025.1_ASM9302v1
100/100
Ralstonia.pickettii GCA_902374465.1.MGYG.HGUT.01384
Burkholderia.cepacia GCA_009586235.1_ASM958623v1
100/100
100/100
Dasania.marina.DSM.21967 GCA_000373485.1_ASM37348v1
Congregibacter.litoralis.KT71 GCA_000153125.2_ASM15312v2
100/100
100/100
Enterobacter.cloacae GCA_000770155.1_ASM77015v1
Alteromonas.macleodii.ATCC.27126 GCA_000172635.2_ASM17263v2
0.483656

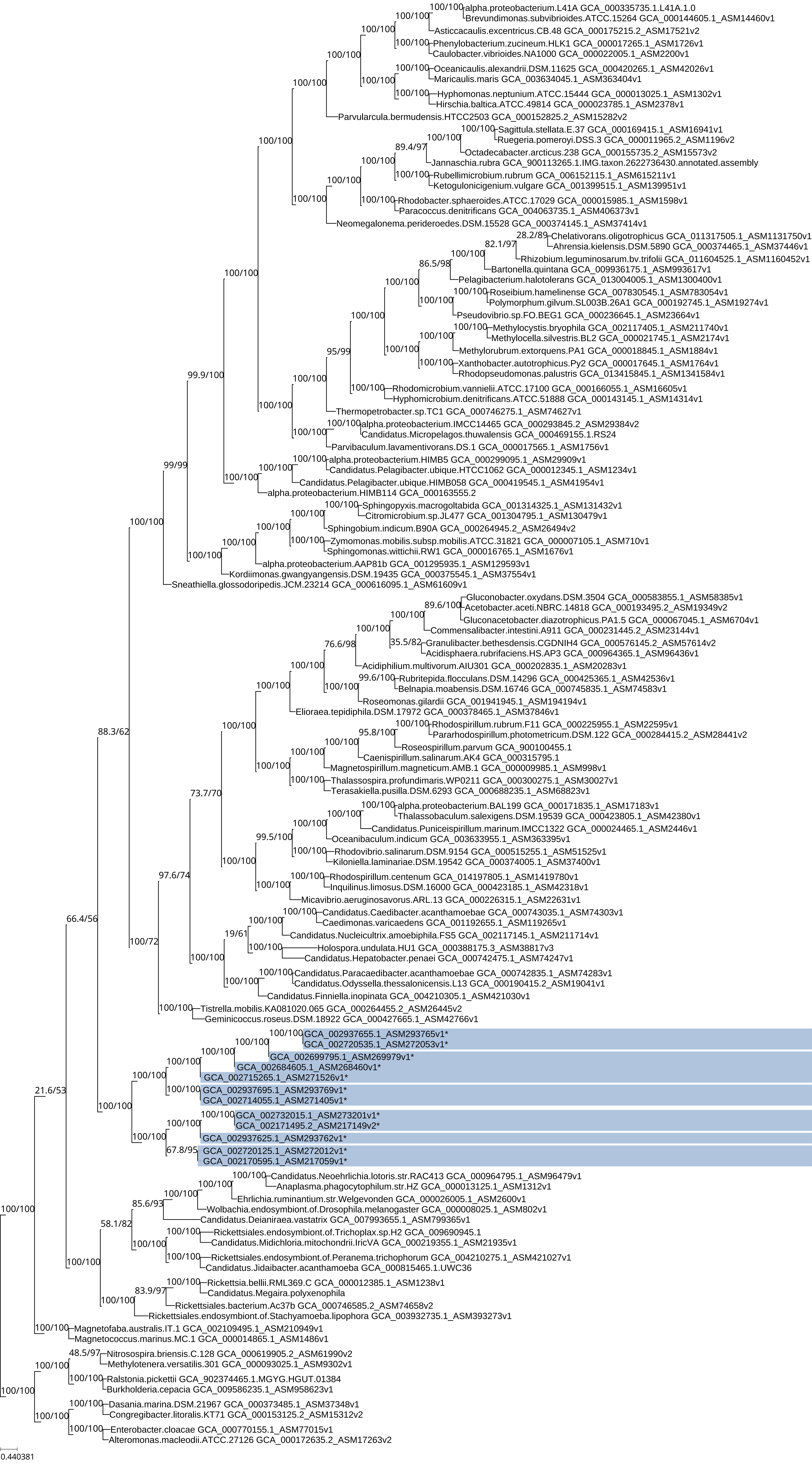

100/100
alpha.proteobacterium.L41A GCA_000335735.1.L41A.1.0
Brevundimonas.subvibrioides.ATCC.15264 GCA_000144605.1_ASM14460v1
100/100
Asticcacaulis.excentricus.CB.48 GCA_000175215.2_ASM17521v2
100/100
Phenylobacterium.zucineum.HLK1 GCA_000017265.1_ASM1726v1
Caulobacter.vibrioides.NA1000 GCA_000022005.1_ASM2200v1
100/100
100/100
Oceanicaulis.alexandrii.DSM.11625 GCA_000420265.1_ASM42026v1
Maricaulis.maris GCA_003634045.1_ASM363404v1
100/100
100/100
Hyphomonas.neptunium.ATCC.15444 GCA_000013025.1_ASM1302v1
Hirschia.baltica.ATCC.49814 GCA_000023785.1_ASM2378v1
100/100
Parvularcula.bermudensis.HTCC2503 GCA_000152825.2_ASM15282v2
100/100
Sagittula.stellata.E.37 GCA_000169415.1_ASM16941v1
Ruegeria.pomeroyi.DSS.3 GCA_000011965.2_ASM1196v2
100/100
89.4/97
Octadecabacter.arcticus.238 GCA_000155735.2_ASM15573v2
Jannaschia.rubra GCA_900113265.1.IMG.taxon.2622736430.annotated.assembly
100/100
100/100
Rubellimicrobium.rubrum GCA_006152115.1_ASM615211v1
Ketogulonicigenium.vulgare GCA_001399515.1_ASM139951v1
100/100
100/100
Rhodobacter.sphaeroides.ATCC.17029 GCA_000015985.1_ASM1598v1
Paracoccus.denitrificans GCA_004063735.1_ASM406373v1
100/100
Neomegalonema.perideroedes.DSM.15528 GCA_000374145.1_ASM37414v1
28.2/89
Chelativorans.oligotrophicus GCA_011317505.1_ASM1131750v1
Ahrensia.kielensis.DSM.5890 GCA_000374465.1_ASM37446v1
82.1/97
Rhizobium.leguminosarum.bv.trifolii GCA_011604525.1_ASM1160452v1
100/100
Bartonella.quintana GCA_009936175.1_ASM993617v1
86.5/98
Pelagibacterium.halotolerans GCA_013004005.1_ASM1300400v1
100/100
100/100
Roseibium.hamelinense GCA_007830545.1_ASM783054v1
Polymorphum.gilvum.SL003B.26A1 GCA_000192745.1_ASM19274v1
100/100
Pseudovibrio.sp.FO.BEG1 GCA_000236645.1_ASM23664v1
100/100
100/100
Methylocystis.bryophila GCA_002117405.1_ASM211740v1
100/100
Methylocella.silvestris.BL2 GCA_000021745.1_ASM2174v1
Methylorubrum.extorquens.PA1 GCA_000018845.1_ASM1884v1
100/100
100/100
Xanthobacter.autotrophicus.Py2 GCA_000017645.1_ASM1764v1
Rhodopseudomonas.palustris GCA_013415845.1_ASM1341584v1
95/99
100/100
100/100
Rhodomicrobium.vannielii.ATCC.17100 GCA_000166055.1_ASM16605v1
Hyphomicrobium.denitrificans.ATCC.51888 GCA_000143145.1_ASM14314v1
100/100
Thermopetrobacter.sp.TC1 GCA_000746275.1_ASM74627v1
100/100
alpha.proteobacterium.IMCC14465 GCA_000293845.2_ASM29384v2
Candidatus.Micropelagos.thuwalensis GCA_000469155.1.RS24
100/100
Parvibaculum.lavamentivorans.DS.1 GCA_000017565.1_ASM1756v1
100/100
99.9/100
100/100
alpha.proteobacterium.HIMB5 GCA_000299095.1_ASM29909v1
100/100
Candidatus.Pelagibacter.ubique.HTCC1062 GCA_000012345.1_ASM1234v1
Candidatus.Pelagibacter.ubique.HIMB058 GCA_000419545.1_ASM41954v1
100/100
alpha.proteobacterium.HIMB114 GCA_000163555.2
99/99
100/100
Sphingopyxis.macrogoltabida GCA_001314325.1_ASM131432v1
100/100
Citromicrobium.sp.JL477 GCA_001304795.1_ASM130479v1
100/100
Sphingobium.indicum.B90A GCA_000264945.2_ASM26494v2
100/100
Zymomonas.mobilis.subsp.mobilis.ATCC.31821 GCA_000007105.1_ASM710v1
100/100
Sphingomonas.wittichii.RW1 GCA_000016765.1_ASM1676v1
100/100
alpha.proteobacterium.AAP81b GCA_001295935.1_ASM129593v1
100/100
Kordiimonas.gwangyangensis.DSM.19435 GCA_000375545.1_ASM37554v1
Sneathiella.glossodoripedis.JCM.23214 GCA_000616095.1_ASM61609v1
89.6/100
Gluconobacter.oxydans.DSM.3504 GCA_000583855.1_ASM58385v1
Acetobacter.aceti.NBRC.14818 GCA_000193495.2_ASM19349v2
100/100
Gluconacetobacter.diazotrophicus.PA1.5 GCA_000067045.1_ASM6704v1
100/100
Commensalibacter.intestini.A911 GCA_000231445.2_ASM23144v1
35.5/82
Granulibacter.bethesdensis.CGDNIH4 GCA_000576145.2_ASM57614v2
76.6/98
Acidisphaera.rubrifaciens.HS.AP3 GCA_000964365.1_ASM96436v1
Acidiphilium.multivorum.AIU301 GCA_000202835.1_ASM20283v1
100/100
99.6/100
Rubritepida.flocculans.DSM.14296 GCA_000425365.1_ASM42536v1
100/100
Belnapia.moabensis.DSM.16746 GCA_000745835.1_ASM74583v1
100/100
Roseomonas.gilardii GCA_001941945.1_ASM194194v1
Elioraea.tepidiphila.DSM.17972 GCA_000378465.1_ASM37846v1
88.3/62
100/100
100/100
Rhodospirillum.rubrum.F11 GCA_000225955.1_ASM22595v1
95.8/100
Pararhodospirillum.photometricum.DSM.122 GCA_000284415.2_ASM28441v2
100/100
Roseospirillum.parvum GCA_900100455.1
100/100
Caenispirillum.salinarum.AK4 GCA_003315795.1
100/100
Magnetospirillum.magneticum.AMB.1 GCA_000009985.1_ASM998v1
100/100
Thalassospira.profundimaris.WP0211 GCA_000300275.1_ASM30027v1
Terasakiella.pusilla.DSM.6293 GCA_000688235.1_ASM68823v1
73.7/70
100/100
alpha.proteobacterium.BAL199 GCA_000171835.1_ASM17183v1
100/100
Thalassobaculum.salexigens.DSM.19539 GCA_000423805.1_ASM42380v1
Candidatus.Puniceispirillum.marinum.IMCC1322 GCA_000024465.1_ASM2446v1
100/100
99.5/100
Oceanibaculum.indicum GCA_003633955.1_ASM363395v1
100/100
Rhodovibrio.salinarum.DSM.9154 GCA_000515255.1_ASM51525v1
100/100
Kiloniella.laminariae.DSM.19542 GCA_000374005.1_ASM37400v1
100/100
Rhodospirillum.centenum GCA_014197805.1_ASM1419780v1
97.6/74
100/100
Inquilinus.limosus.DSM.16000 GCA_000423185.1_ASM42318v1
Micavibrio.aeruginosavorus.ARL.13 GCA_000226315.1_ASM22631v1
100/100
Candidatus.Caedibacter.acanthamoebae GCA_000743035.1_ASM74303v1
100/100
Caedimonas.varicaedens GCA_001192655.1_ASM119265v1
66.4/56
19/61
Candidatus.Nucleicultrix.amoebiphila.FS5 GCA_002117145.1_ASM211714v1
100/100
Holospora.undulata.HU1 GCA_000388175.3_ASM38817v3
100/72
Candidatus.Hepatobacter.penaei GCA_000742475.1_ASM74247v1
100/100
100/100
Candidatus.Paracaedibacter.acanthamoebae GCA_000742835.1_ASM74283v1
100/100
Candidatus.Odyssella.thessalonicensis.L13 GCA_000190415.2_ASM19041v1
Candidatus.Finniella.inopinata GCA_004210305.1_ASM421030v1
100/100
Tistrella.mobilis.KA081020.065 GCA_000264455.2_ASM26445v2
Geminicoccus.roseus.DSM.18922 GCA_000427665.1_ASM42766v1
100/100
GCA_002937655.1_ASM293765v1*
100/100
GCA_002720535.1_ASM272053v1*
100/100
GCA_002699795.1_ASM269979v1*
100/100
GCA_002684605.1_ASM268460v1*
100/100
GCA_002715265.1_ASM271526v1*
21.6/53
100/100
GCA_002937695.1_ASM293769v1*
GCA_002714055.1_ASM271405v1*
100/100
100/100
GCA_002732015.1_ASM273201v1*
100/100
GCA_002171495.2_ASM217149v2*
100/100
GCA_002937625.1_ASM293762v1*
67.8/95
GCA_002720125.1_ASM272012v1*
GCA_002170595.1_ASM217059v1*
100/100
Candidatus.Neoehrlichia.lotoris.str.RAC413 GCA_000964795.1_ASM96479v1
100/100
Anaplasma.phagocytophilum.str.HZ GCA_000013125.1_ASM1312v1
100/100
Ehrlichia.ruminantium.str.Welgevonden GCA_000026005.1_ASM2600v1
85.6/93
Wolbachia.endosymbiont.of.Drosophila.melanogaster GCA_000008025.1_ASM802v1
Candidatus.Deianiraea.vastatrix GCA_007993655.1_ASM799365v1
100/100
58.1/82
100/100
Rickettsiales.endosymbiont.of.Trichoplax.sp.H2 GCA_009690945.1
100/100
Candidatus.Midichloria.mitochondrii.IricVA GCA_000219355.1_ASM21935v1
100/100
Rickettsiales.endosymbiont.of.Peranema.trichophorum GCA_004210275.1_ASM421027v1
100/100
Candidatus.Jidaibacter.acanthamoeba GCA_000815465.1.UWC36
100/100
Rickettsia.bellii.RML369.C GCA_000012385.1_ASM1238v1
83.9/97
Candidatus.Megaira.polyxenophila
100/100
Rickettsiales.bacterium.Ac37b GCA_000746585.2_ASM74658v2
Rickettsiales.endosymbiont.of.Stachyamoeba.lipophora GCA_003932735.1_ASM393273v1
100/100
Magnetofaba.australis.IT.1 GCA_002109495.1_ASM210949v1
Magnetococcus.marinus.MC.1 GCA_000014865.1_ASM1486v1
48.5/97
Nitrosospira.briensis.C.128 GCA_000619905.2_ASM61990v2
100/100
Methylotenera.versatilis.301 GCA_000093025.1_ASM9302v1
100/100
Ralstonia.pickettii GCA_902374465.1.MGYG.HGUT.01384
Burkholderia.cepacia GCA_009586235.1_ASM958623v1
100/100
100/100
Dasania.marina.DSM.21967 GCA_000373485.1_ASM37348v1
Congregibacter.litoralis.KT71 GCA_000153125.2_ASM15312v2
100/100
100/100
Enterobacter.cloacae GCA_000770155.1_ASM77015v1
Alteromonas.macleodii.ATCC.27126 GCA_000172635.2_ASM17263v2
0.440381

Group L

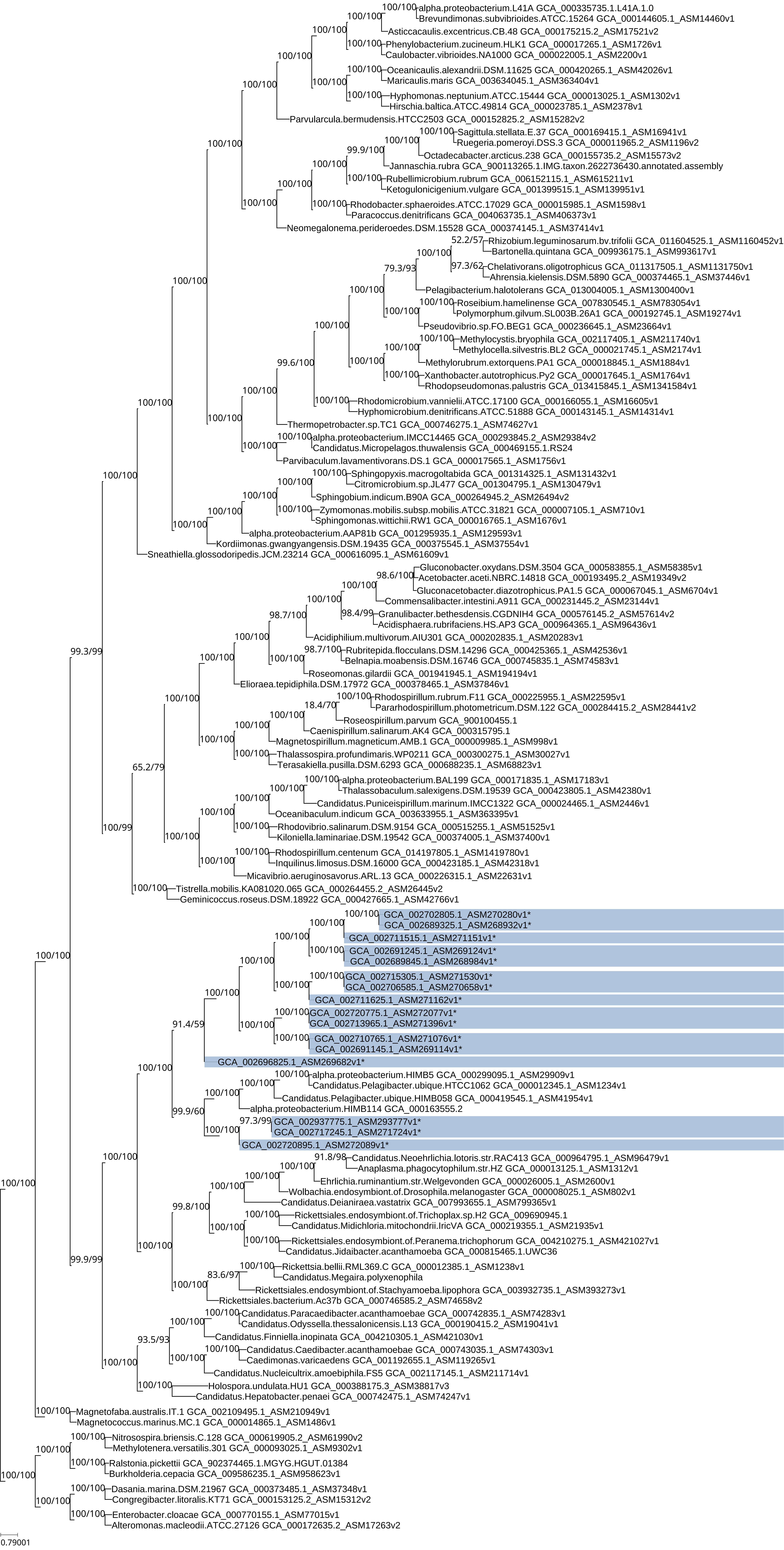
100/100 alpha.proteobacterium.L41A GCA_000335735.1.L41A.1.0
100/100 Brevundimonas.subvibrioides.ATCC.15264 GCA_000144605.1_ASM14460v1
100/100 Asticcacaulis.excentricus.CB.48 GCA_000175215.2_ASM17521v2
100/100 Phenylobacterium.zucineum.HLK1 GCA_000017265.1_ASM1726v1
Caulobacter.vibrioides.NA1000 GCA_000022005.1_ASM2200v1
100/100
100/100 Oceanicaulis.alexandrii.DSM.11625 GCA_000420265.1_ASM42026v1
Maricaulis.maris GCA_003634045.1_ASM363404v1
100/100
100/100 Hyphomonas.neptunium.ATCC.15444 GCA_000013025.1_ASM1302v1
Hirschia.baltica.ATCC.49814 GCA_000023785.1_ASM2378v1
Parvularcula.bermudensis.HTCC2503 GCA_000152825.2_ASM15282v2
100/100 Sagittula.stellata.E.37 GCA_000169415.1_ASM16941v1
100/100 Ruegeria.pomeroyi.DSS.3 GCA_000011965.2_ASM1196v2
99.9/100 Octadecabacter.arcticus.238 GCA_000155735.2_ASM15573v2
100/100 Jannaschia.rubra GCA_900113265.1.IMG.taxon.2622736430.annotated.assembly
100/100 Rubellimicrobium.rubrum GCA_006152115.1_ASM615211v1
100/100 Ketogulonicigenium.vulgare GCA_001399515.1_ASM139951v1
100/100 Rhodobacter.sphaeroides.ATCC.17029 GCA_000015985.1_ASM1598v1
100/100 Paracoccus.denitrificans GCA_004063735.1_ASM406373v1
Neomegalonema.perideroedes.DSM.15528 GCA_000374145.1_ASM37414v1
52.2/57 Rhizobium.leguminosarum.bv.trifolii GCA_011604525.1_ASM1160452v1
100/100 Bartonella.quintana GCA_009936175.1_ASM993617v1
97.3/62 Chelativorans.oligotrophicus GCA_011317505.1_ASM1131750v1
79.3/93 Ahrensia.kielensis.DSM.5890 GCA_000374465.1_ASM37446v1
100/100 Pelagibacterium.halotolerans GCA_013004005.1_ASM1300400v1
100/100 Roseibium.hamelinense GCA_007830545.1_ASM783054v1
100/100 Polymorphum.gilvum.SL003B.26A1 GCA_000192745.1_ASM19274v1
100/100 Pseudovibrio.sp.FO.BEG1 GCA_000236645.1_ASM23664v1
100/100 Methylocystis.bryophila GCA_002117405.1_ASM211740v1
100/100 Methylocella.silvestris.BL2 GCA_000021745.1_ASM2174v1
99.6/100 Methylorubrum.extorquens.PA1 GCA_000018845.1_ASM1884v1
100/100 Xanthobacter.autotrophicus.Py2 GCA_000017645.1_ASM1764v1
100/100 Rhodopseudomonas.palustris GCA_013415845.1_ASM1341584v1
100/100 Rhodomicrobium.vannielii.ATCC.17100 GCA_000166055.1_ASM16605v1
100/100 Hyphomicrobium.denitrificans.ATCC.51888 GCA_000143145.1_ASM14314v1
100/100 Thermopetrobacter.sp.TC1 GCA_000746275.1_ASM74627v1
100/100 alpha.proteobacterium.IMCC14465 GCA_000293845.2_ASM29384v2
100/100 Candidatus.Micropelagos.thuwalensis GCA_000469155.1.RS24
100/100 Parvibaculum.lavamentivorans.DS.1 GCA_000017565.1_ASM1756v1
100/100 Sphingopyxis.macrogoltabida GCA_001314325.1_ASM131432v1
100/100 Citromicrobium.sp.JL477 GCA_001304795.1_ASM130479v1
100/100 Sphingobium.indicum.B90A GCA_000264945.2_ASM26494v2
100/100 Zymomonas.mobilis.subsp.mobilis.ATCC.31821 GCA_000007105.1_ASM710v1
100/100 Sphingomonas.wittichii.RW1 GCA_000016765.1_ASM1676v1
100/100 alpha.proteobacterium.AAP81b GCA_001295935.1_ASM129593v1
100/100 Kordiimonas.gwangyangensis.DSM.19435 GCA_000375545.1_ASM37554v1
100/100 Sneathiella.glossodoripedis.JCM.23214 GCA_000616095.1_ASM61609v1
98.6/100 Gluconobacter.oxydans.DSM.3504 GCA_000583855.1_ASM58385v1
Acetobacter.aceti.NBRC.14818 GCA_000193495.2_ASM19349v2
100/100 Gluconacetobacter.diazotrophicus.PA1.5 GCA_000067045.1_ASM6704v1
100/100 Commensalibacter.intestini.A911 GCA_000231445.2_ASM23144v1
98.4/99 Granulibacter.bethesdensis.CGDNIH4 GCA_000576145.2_ASM57614v2
98.7/100 Acidisphaera.rubrifaciens.HS.AP3 GCA_000964365.1_ASM96436v1
100/100 Acidiphilium.multivorum.AIU301 GCA_000202835.1_ASM20283v1
98.7/100 Rubritepida.flocculans.DSM.14296 GCA_000425365.1_ASM42536v1
100/100 Belnapia.moabensis.DSM.16746 GCA_000745835.1_ASM74583v1
100/100 Roseomonas.gilardii GCA_001941945.1_ASM194194v1
Elioraea.tepidiphila.DSM.17972 GCA_000378465.1_ASM37846v1
100/100 Rhodospirillum.rubrum.F11 GCA_000225955.1_ASM22595v1
18.4/70 Pararhodospirillum.photometricum.DSM.122 GCA_000284415.2_ASM28441v2
100/100 Roseospirillum.parvum GCA_900100455.1
100/100 Caenispirillum.salinarum.AK4 GCA_000315795.1
100/100 Magnetospirillum.magneticum.AMB.1 GCA_000009985.1_ASM998v1
100/100 Thalassospira.profundimaris.WP0211 GCA_000300275.1_ASM30027v1
Terasakiella.pusilla.DSM.6293 GCA_000688235.1_ASM68823v1
100/100 alpha.proteobacterium.BAL199 GCA_000171835.1_ASM17183v1
100/100 Thalassobaculum.salexigens.DSM.19539 GCA_000423805.1_ASM42380v1
100/100 Candidatus.Puniceispirillum.marinum.IMCC1322 GCA_000024465.1_ASM2446v1
100/100 Oceanibaculum.indicum GCA_003633955.1_ASM363395v1
100/100 Rhodovibrio.salinarum.DSM.9154 GCA_000515255.1_ASM51525v1
65.2/79 Kiloniella.laminariae.DSM.19542 GCA_000374005.1_ASM37400v1
100/100 Rhodospirillum.centenum GCA_014197805.1_ASM1419780v1
100/100 Inquilinus.limosus.DSM.16000 GCA_000423185.1_ASM42318v1
100/99 Micavibrio.aeruginosavorus.ARL.13 GCA_000226315.1_ASM22631v1
100/100 Tistrella.mobilis.KA081020.065 GCA_000264455.2_ASM26445v2
Geminicoccus.roseus.DSM.18922 GCA_000427665.1_ASM42766v1
99.3/99
100/100 GCA_002702805.1_ASM270280v1*
GCA_002689325.1_ASM268932v1*
100/100 GCA_002711515.1_ASM271151v1*
100/100 GCA_002691245.1_ASM269124v1*
GCA_002689845.1_ASM268984v1*
100/100 GCA_002715305.1_ASM271530v1*
GCA_002706585.1_ASM270658v1*
100/100 GCA_002711625.1_ASM271162v1*
100/100 GCA_002720775.1_ASM272077v1*
GCA_002713965.1_ASM271396v1*
100/100 GCA_002710765.1_ASM271076v1*
GCA_002691145.1_ASM269114v1*
91.4/59 GCA_002696825.1_ASM269682v1*
100/100 alpha.proteobacterium.HIMB5 GCA_000299095.1_ASM29909v1
100/100 Candidatus.Pelagibacter.ubique.HTCC1062 GCA_000012345.1_ASM1234v1
100/100 Candidatus.Pelagibacter.ubique.HIMB058 GCA_000419545.1_ASM41954v1
alpha.proteobacterium.HIMB114 GCA_000163555.2
97.3/99 GCA_002937775.1_ASM293777v1*
99.9/60 GCA_002717245.1_ASM271724v1*
100/100 GCA_002720895.1_ASM272089v1*
91.8/98 Candidatus.Neoehrlichia.lotoris.str.RAC413 GCA_000964795.1_ASM96479v1
100/100 Anaplasma.phagocytophilum.str.HZ GCA_000013125.1_ASM1312v1
100/100 Ehrlichia.ruminantium.str.Welgevonden GCA_000026005.1_ASM2600v1
100/100 Wolbachia.endosymbiont.of.Drosophila.melanogaster GCA_000008025.1_ASM802v1
Candidatus.Deianiraea.vastatrix GCA_007993655.1_ASM799365v1
99.8/100 Rickettsiales.endosymbiont.of.Trichoplax.sp.H2 GCA_009690945.1
100/100 Candidatus.Midichloria.mitochondrii.IricVA GCA_000219355.1_ASM21935v1
100/100 Rickettsiales.endosymbiont.of.Peranema.trichophorum GCA_004210275.1_ASM421027v1
Candidatus.Jidaibacter.acanthamoeba GCA_000815465.1.UWC36
99.9/99 Rickettsia.bellii.RML369.C GCA_000012385.1_ASM1238v1
83.6/97 Candidatus.Megaira.polyxenophila
100/100 Rickettsiales.endosymbiont.of.Stachyamoeba.lipophora GCA_003932735.1_ASM393273v1
Rickettsiales.bacterium.Ac37b GCA_000746585.2_ASM74658v2
100/100 Candidatus.Paracaedibacter.acanthamoebae GCA_000742835.1_ASM74283v1
100/100 Candidatus.Odyssella.thessalonicensis.L13 GCA_000190415.2_ASM19041v1
Candidatus.Finniella.inopinata GCA_004210305.1_ASM421030v1
93.5/93 Candidatus.Caedibacter.acanthamoebae GCA_000743035.1_ASM74303v1
100/100 Caedimonas.varicaedens GCA_001192655.1_ASM119265v1
100/100 Candidatus.Nucleicultrix.amoebiphila.FS5 GCA_002117145.1_ASM211714v1
100/100 Holospora.undulata.HU1 GCA_000388175.3_ASM38817v3
Candidatus.Hepatobacter.penaei GCA_000742475.1_ASM74247v1
100/100 Magnetofaba.australis.IT.1 GCA_002109495.1_ASM210949v1
Magnetococcus.marinus.MC.1 GCA_000014865.1_ASM1486v1
100/100 Nitrosospira.briensis.C.128 GCA_000619905.2_ASM61990v2
100/100 Methylotenera.versatilis.301 GCA_000093025.1_ASM9302v1
100/100 Ralstonia.pickettii GCA_902374465.1.MGYG.HGUT.01384
Burkholderia.cepacia GCA_009586235.1_ASM958623v1
100/100 Dasania.marina.DSM.21967 GCA_000373485.1_ASM37348v1
100/100 Congregibacter.litoralis.KT71 GCA_000153125.2_ASM15312v2
100/100 Enterobacter.cloacae GCA_000770155.1_ASM77015v1
Alteromonas.macleodii.ATCC.27126 GCA_000172635.2_ASM17263v2
0.79001

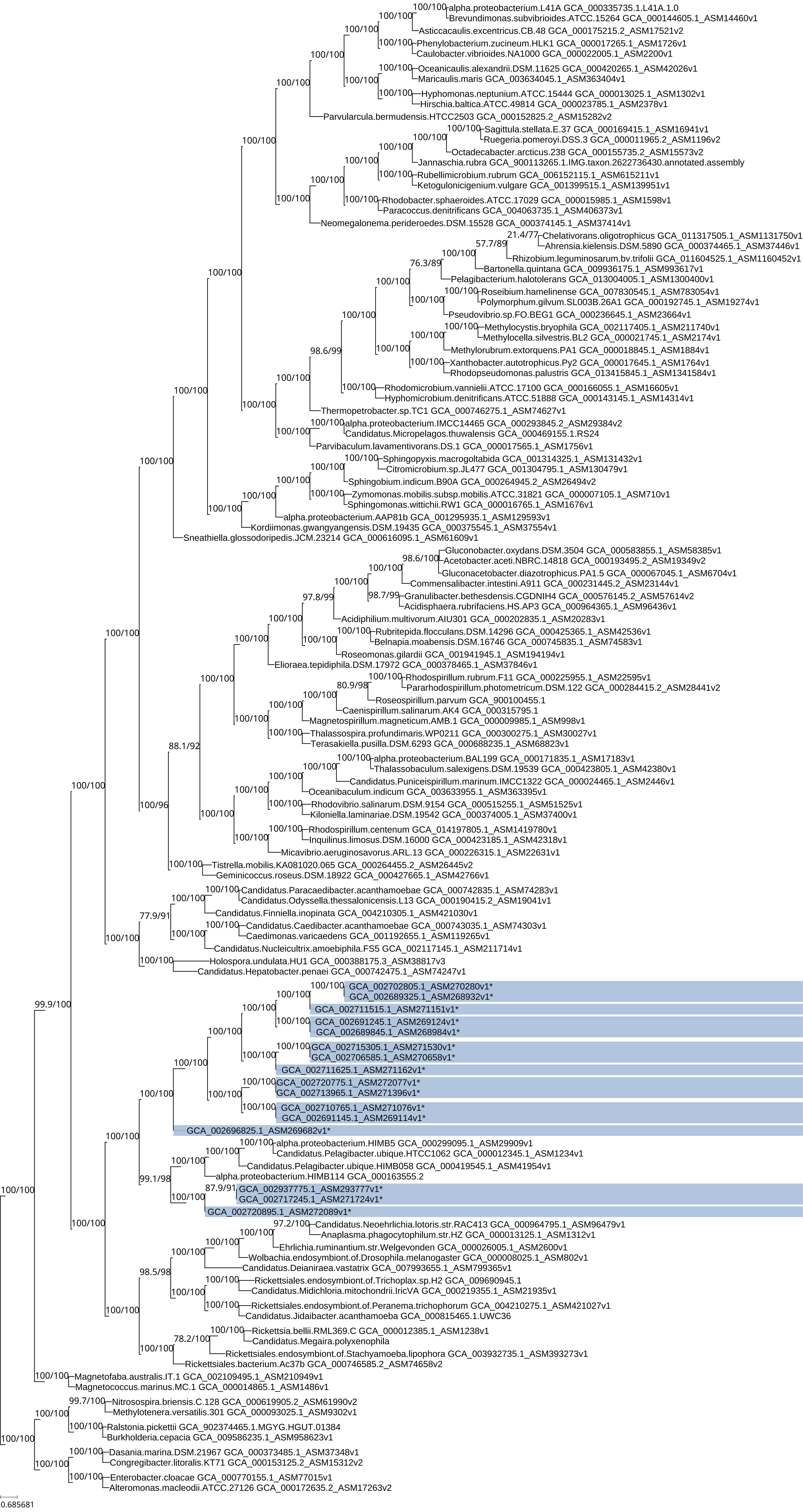

alpha.proteobacterium.L41A GCA_000335735.1.L41A.1.0
Brevundimonas.subvibrioides.ATCC.15264 GCA_000144605.1_ASM14460v1
Asticcacaulis.excentricus.CB.48 GCA_000175215.2_ASM17521v2
Phenylobacterium.zucineum.HLK1 GCA_000017265.1_ASM1726v1
Caulobacter.vibrioides.NA1000 GCA_000022005.1_ASM2200v1
Oceanicaulis.alexandrii.DSM.11625 GCA_000420265.1_ASM42026v1
Maricaulis.maris GCA_003634045.1_ASM363404v1
Hyphomonas.neptunium.ATCC.15444 GCA_000013025.1_ASM1302v1
Hirschia.baltica.ATCC.49814 GCA_000023785.1_ASM2378v1
Parvularcula.bermudensis.HTCC2503 GCA_000152825.2_ASM15282v2
Sagittula.stellata.E.37 GCA_000169415.1_ASM16941v1
Ruegeria.pomeroyi.DSS.3 GCA_000011965.2_ASM1196v2
Octadecabacter.arcticus.238 GCA_000155735.2_ASM15573v2
Jannaschia.rubra GCA_900113265.1.IMG.taxon.2622736430.annotated.assembly
Rubellimicrobium.rubrum GCA_006152115.1_ASM615211v1
Ketogulonicigenium.vulgare GCA_001399515.1_ASM139951v1
Rhodobacter.sphaeroides.ATCC.17029 GCA_000015985.1_ASM1598v1
Paracoccus.denitrificans GCA_004063735.1_ASM406373v1
Neomegalonema.perideroedes.DSM.15528 GCA_000374145.1_ASM37414v1
Chelativorans.oligotrophicus GCA_011317505.1_ASM1131750v1
Ahrensia.kielensis.DSM.5890 GCA_000374465.1_ASM37446v1
Rhizobium.leguminosarum.bv.trifolii GCA_011604525.1_ASM1160452v1
Bartonella.quintana GCA_009936175.1_ASM993617v1
Pelagibacterium.halotolerans GCA_013004005.1_ASM1300400v1
Roseibium.hamelinense GCA_007830545.1_ASM783054v1
Polymorphum.gilvum.SL003B.26A1 GCA_000192745.1_ASM19274v1
Pseudovibrio.sp.FO.BEG1 GCA_000236645.1_ASM23664v1
Methylocystis.bryophila GCA_002117405.1_ASM211740v1
Methylocella.silvestris.BL2 GCA_000021745.1_ASM2174v1
Methylorubrum.extorquens.PA1 GCA_000018845.1_ASM1884v1
Xanthobacter.autotrophicus.Py2 GCA_000017645.1_ASM1764v1
Rhodopseudomonas.palustris GCA_013415845.1_ASM1341584v1
Rhodomicrobium.vannielii.ATCC.17100 GCA_000166055.1_ASM16605v1
Hyphomicrobium.denitrificans.ATCC.51888 GCA_000143145.1_ASM14314v1
Thermopetrobacter.sp.TC1 GCA_000746275.1_ASM74627v1
alpha.proteobacterium.IMCC14465 GCA_000293845.2_ASM29384v2
Candidatus.Micropelagos.thuwalensis GCA_000469155.1.RS24
Parvibaculum.lavamentivorans.DS.1 GCA_000017565.1_ASM1756v1
Sphingopyxis.macrogoltabida GCA_001314325.1_ASM131432v1
Citromicrobium.sp.JL477 GCA_001304795.1_ASM130479v1
Sphingobium.indicum.B90A GCA_000264945.2_ASM26494v2
Zymomonas.mobilis.subsp.mobilis.ATCC.31821 GCA_000007105.1_ASM710v1
Sphingomonas.wittichii.RW1 GCA_000016765.1_ASM1676v1
alpha.proteobacterium.AAP81b GCA_001295935.1_ASM129593v1
Kordiimonas.gwangyangensis.DSM.19435 GCA_000375545.1_ASM37554v1
Sneathiella.glossodoripedis.JCM.23214 GCA_000616095.1_ASM61609v1
Gluconobacter.oxydans.DSM.3504 GCA_000583855.1_ASM58385v1
Acetobacter.aceti.NBRC.14818 GCA_000193495.2_ASM19349v2
Gluconacetobacter.diazotrophicus.PA1.5 GCA_000067045.1_ASM6704v1
Commensalibacter.intestini.A911 GCA_000231445.2_ASM23144v1
Granulibacter.bethesdensis.CGDNIH4 GCA_000576145.2_ASM57614v2
Acidisphaera.rubrifaciens.HS.AP3 GCA_000964365.1_ASM96436v1
Acidiphilium.multivorum.AIU301 GCA_000202835.1_ASM20283v1
Rubritepida.flocculans.DSM.14296 GCA_000425365.1_ASM42536v1
Belnapia.moabensis.DSM.16746 GCA_000745835.1_ASM74583v1
Roseomonas.gilardii GCA_001941945.1_ASM194194v1
Elioraea.tepidiphila.DSM.17972 GCA_000378465.1_ASM37846v1
Rhodospirillum.rubrum.F11 GCA_000225955.1_ASM22595v1
Pararhodospirillum.photometricum.DSM.122 GCA_000284415.2_ASM28441v2
Roseospirillum.parvum GCA_900100455.1
Caenispirillum.salinarum.AK4 GCA_000315795.1
Magnetospirillum.magneticum.AMB.1 GCA_000009985.1_ASM998v1
Thalassospira.profundimaris.WP0211 GCA_000300275.1_ASM30027v1
Terasakiella.pusilla.DSM.6293 GCA_000688235.1_ASM68823v1
alpha.proteobacterium.BAL199 GCA_000171835.1_ASM17183v1
Thalassobaculum.salexigens.DSM.19539 GCA_000423805.1_ASM42380v1
Candidatus.Puniceispirillum.marinum.IMCC1322 GCA_000024465.1_ASM2446v1
Oceanibaculum.indicum GCA_003633955.1_ASM363395v1
Rhodovibrio.salinarum.DSM.9154 GCA_000515255.1_ASM51525v1
Kiloniella.laminariae.DSM.19542 GCA_000374005.1_ASM37400v1
Rhodospirillum.centenum GCA_014197805.1_ASM1419780v1
Inquilinus.limosus.DSM.16000 GCA_000423185.1_ASM42318v1
Micavibrio.aeruginosavorus.ARL.13 GCA_000226315.1_ASM22631v1
Tistrella.mobilis.KA081020.065 GCA_000264455.2_ASM26445v2
Geminicoccus.roseus.DSM.18922 GCA_000427665.1_ASM42766v1
Candidatus.Paracaedibacter.acanthamoebae GCA_000742835.1_ASM74283v1
Candidatus.Odyssella.thessalonicensis.L13 GCA_000190415.2_ASM19041v1
Candidatus.Finniella.inopinata GCA_004210305.1_ASM421030v1
Candidatus.Caedibacter.acanthamoebae GCA_000743035.1_ASM74303v1
Caedimonas.varicaedens GCA_001192655.1_ASM119265v1
Candidatus.Nucleicultrix.amoebiphila.FS5 GCA_002117145.1_ASM211714v1
Holospora.undulata.HU1 GCA_000388175.3_ASM38817v3
Candidatus.Hepatobacter.penaei GCA_000742475.1_ASM74247v1
GCA_002702805.1_ASM270280v1*
GCA_002689325.1_ASM268932v1*
GCA_002711515.1_ASM271151v1*
GCA_002691245.1_ASM269124v1*
GCA_002689845.1_ASM268984v1*
GCA_002715305.1_ASM271530v1*
GCA_002706585.1_ASM270658v1*
GCA_002711625.1_ASM271162v1*
GCA_002720775.1_ASM272077v1*
GCA_002713965.1_ASM271396v1*
GCA_002710765.1_ASM271076v1*
GCA_002691145.1_ASM269114v1*
GCA_002696825.1_ASM269682v1*
alpha.proteobacterium.HIMB5 GCA_000299095.1_ASM29909v1
Candidatus.Pelagibacter.ubique.HTCC1062 GCA_000012345.1_ASM1234v1
Candidatus.Pelagibacter.ubique.HIMB058 GCA_000419545.1_ASM41954v1
alpha.proteobacterium.HIMB114 GCA_000163555.2
GCA_002937775.1_ASM293777v1*
GCA_002717245.1_ASM271724v1*
GCA_002720895.1_ASM272089v1*
Candidatus.Neoehrlichia.lotoris.str.RAC413 GCA_000964795.1_ASM96479v1
Anaplasma.phagocytophilum.str.HZ GCA_000013125.1_ASM1312v1
Ehrlichia.ruminantium.str.Welgevonden GCA_000026005.1_ASM2600v1
Wolbachia.endosymbiont.of.Drosophila.melanogaster GCA_000008025.1_ASM802v1
Candidatus.Deianiraea.vastatrix GCA_007993655.1_ASM799365v1
Rickettsiales.endosymbiont.of.Trichoplax.sp.H2 GCA_009690945.1
Candidatus.Midichloria.mitochondrii.IricVA GCA_000219355.1_ASM21935v1
Rickettsiales.endosymbiont.of.Peranema.trichophorum GCA_004210275.1_ASM421027v1
Candidatus.Jidaibacter.acanthamoeba GCA_000815465.1.UWC36
Rickettsia.bellii.RML369.C GCA_000012385.1_ASM1238v1
Candidatus.Megaira.polyxenophila
Rickettsiales.endosymbiont.of.Stachyamoeba.lipophora GCA_003932735.1_ASM393273v1
Rickettsiales.bacterium.Ac37b GCA_000746585.2_ASM74658v2
Magnetofaba.australis.IT.1 GCA_002109495.1_ASM210949v1
Magnetococcus.marinus.MC.1 GCA_000014865.1_ASM1486v1
Nitrosospira.briensis.C.128 GCA_000619905.2_ASM61990v2
Methylotenera.versatilis.301 GCA_000093025.1_ASM9302v1
Ralstonia.pickettii GCA_902374465.1.MGYG.HGUT.01384
Burkholderia.cepacia GCA_009586235.1_ASM958623v1
Dasania.marina.DSM.21967 GCA_000373485.1_ASM37348v1
Congregibacter.litoralis.KT71 GCA_000153125.2_ASM15312v2
Enterobacter.cloacae GCA_000770155.1_ASM77015v1
Alteromonas.macleodii.ATCC.27126 GCA_000172635.2_ASM17263v2
0.685681

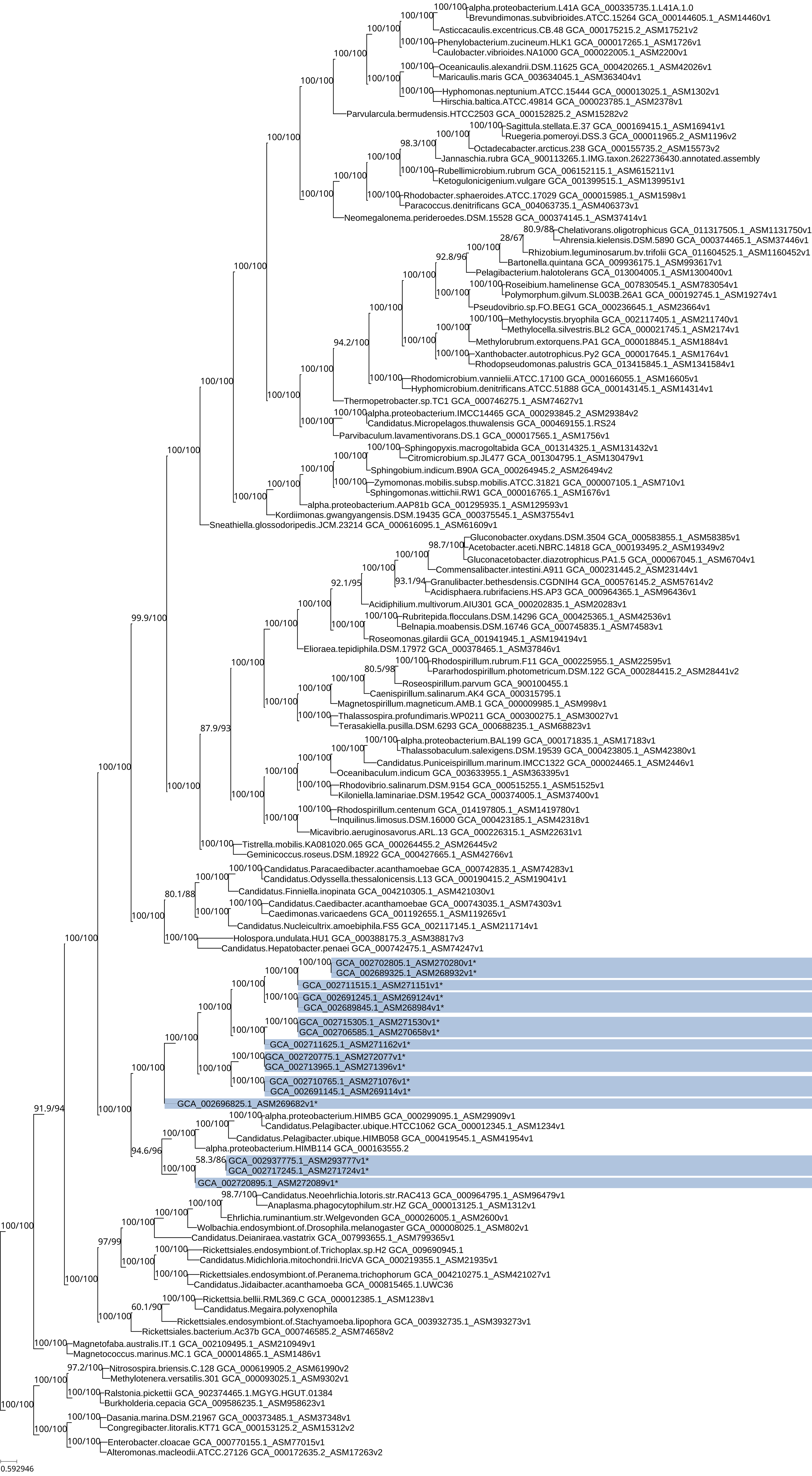
100/100
alpha.proteobacterium.L41A GCA_000335735.1.L41A.1.0
100/100
Brevundimonas.subvibrioides.ATCC.15264 GCA_000144605.1_ASM14460v1
100/100
Asticcacaulis.excentricus.CB.48 GCA_000175215.2_ASM17521v2
100/100
Phenylobacterium.zucineum.HLK1 GCA_000017265.1_ASM1726v1
100/100
Caulobacter.vibrioides.NA1000 GCA_000022005.1_ASM2200v1
100/100
Oceanicaulis.alexandrii.DSM.11625 GCA_000420265.1_ASM42026v1
100/100
Maricaulis.maris GCA_003634045.1_ASM363404v1
100/100
Hyphomonas.neptunium.ATCC.15444 GCA_000013025.1_ASM1302v1
100/100
Hirschia.baltica.ATCC.49814 GCA_000023785.1_ASM2378v1
Parvularcula.bermudensis.HTCC2503 GCA_000152825.2_ASM15282v2
100/100
100/100
Sagittula.stellata.E.37 GCA_000169415.1_ASM16941v1
100/100
Ruegeria.pomeroyi.DSS.3 GCA_000011965.2_ASM1196v2
98.3/100
Octadecabacter.arcticus.238 GCA_000155735.2_ASM15573v2
100/100
Jannaschia.rubra GCA_900113265.1.IMG.taxon.2622736430.annotated.assembly
100/100
Rubellimicrobium.rubrum GCA_006152115.1_ASM615211v1
100/100
Ketogulonicigenium.vulgare GCA_001399515.1_ASM139951v1
100/100
Rhodobacter.sphaeroides.ATCC.17029 GCA_000015985.1_ASM1598v1
Paracoccus.denitrificans GCA_004063735.1_ASM406373v1
Neomegalonema.perideroedes.DSM.15528 GCA_000374145.1_ASM37414v1
80.9/88
Chelativorans.oligotrophicus GCA_011317505.1_ASM1131750v1
28/67
Ahrensia.kielensis.DSM.5890 GCA_000374465.1_ASM37446v1
100/100
Rhizobium.leguminosarum.bv.trifolii GCA_011604525.1_ASM1160452v1
100/100
Bartonella.quintana GCA_009936175.1_ASM993617v1
92.8/96
Pelagibacterium.halotolerans GCA_013004005.1_ASM1300400v1
100/100
Roseibium.hamelinense GCA_007830545.1_ASM783054v1
100/100
Polymorphum.gilvum.SL003B.26A1 GCA_000192745.1_ASM19274v1
100/100
Pseudovibrio.sp.FO.BEG1 GCA_000236645.1_ASM23664v1
100/100
Methylocystis.bryophila GCA_002117405.1_ASM211740v1
100/100
Methylocella.silvestris.BL2 GCA_000021745.1_ASM2174v1
94.2/100
Methylorubrum.extorquens.PA1 GCA_000018845.1_ASM1884v1
100/100
Xanthobacter.autotrophicus.Py2 GCA_000017645.1_ASM1764v1
100/100
Rhodopseudomonas.palustris GCA_013415845.1_ASM1341584v1
100/100
Rhodomicrobium.vannielii.ATCC.17100 GCA_000166055.1_ASM16605v1
Hyphomicrobium.denitrificans.ATCC.51888 GCA_000143145.1_ASM14314v1
100/100
Thermopetrobacter.sp.TC1 GCA_000746275.1_ASM74627v1
100/100
alpha.proteobacterium.IMCC14465 GCA_000293845.2_ASM29384v2
100/100
Candidatus.Micropelagos.thuwalensis GCA_000469155.1.RS24
Parvibaculum.lavamentivorans.DS.1 GCA_000017565.1_ASM1756v1
100/100
100/100
Sphingopyxis.macrogoltabida GCA_001314325.1_ASM131432v1
100/100
Citromicrobium.sp.JL477 GCA_001304795.1_ASM130479v1
100/100
Sphingobium.indicum.B90A GCA_000264945.2_ASM26494v2
100/100
Zymomonas.mobilis.subsp.mobilis.ATCC.31821 GCA_000007105.1_ASM710v1
100/100
Sphingomonas.wittichii.RW1 GCA_000016765.1_ASM1676v1
alpha.proteobacterium.AAP81b GCA_001295935.1_ASM129593v1
Kordiimonas.gwangyangensis.DSM.19435 GCA_000375545.1_ASM37554v1
Sneathiella.glossodoripedis.JCM.23214 GCA_000616095.1_ASM61609v1
98.7/100
Gluconobacter.oxydans.DSM.3504 GCA_000583855.1_ASM58385v1
Acetobacter.aceti.NBRC.14818 GCA_000193495.2_ASM19349v2
100/100
Gluconacetobacter.diazotrophicus.PA1.5 GCA_000067045.1_ASM6704v1
100/100
Commensalibacter.intestini.A911 GCA_000231445.2_ASM23144v1
92.1/95
93.1/94
Granulibacter.bethesdensis.CGDNIH4 GCA_000576145.2_ASM57614v2
Acidisphaera.rubrifaciens.HS.AP3 GCA_000964365.1_ASM96436v1
100/100
Acidiphilium.multivorum.AIU301 GCA_000202835.1_ASM20283v1
100/100
100/100
Rubritepida.flocculans.DSM.14296 GCA_000425365.1_ASM42536v1
100/100
Belnapia.moabensis.DSM.16746 GCA_000745835.1_ASM74583v1
99.9/100
Roseomonas.gilardii GCA_001941945.1_ASM194194v1
Elioraea.tepidiphila.DSM.17972 GCA_000378465.1_ASM37846v1
100/100
100/100
Rhodospirillum.rubrum.F11 GCA_000225955.1_ASM22595v1
80.5/98
Pararhodospirillum.photometricum.DSM.122 GCA_000284415.2_ASM28441v2
100/100
Roseospirillum.parvum GCA_900100455.1
100/100
Caenispirillum.salinarum.AK4 GCA_000315795.1
100/100
Magnetospirillum.magneticum.AMB.1 GCA_000009985.1_ASM998v1
100/100
Thalassospira.profundimaris.WP0211 GCA_000300275.1_ASM30027v1
87.9/93
Terasakiella.pusilla.DSM.6293 GCA_000688235.1_ASM68823v1
100/100
alpha.proteobacterium.BAL199 GCA_000171835.1_ASM17183v1
100/100
Thalassobaculum.salexigens.DSM.19539 GCA_000423805.1_ASM42380v1
100/100
Candidatus.Puniceispirillum.marinum.IMCC1322 GCA_000024465.1_ASM2446v1
100/100
Oceanibaculum.indicum GCA_003633955.1_ASM363395v1
100/100
Rhodovibrio.salinarum.DSM.9154 GCA_000515255.1_ASM51525v1
100/100
Kiloniella.laminariae.DSM.19542 GCA_000374005.1_ASM37400v1
100/100
Rhodospirillum.centenum GCA_014197805.1_ASM1419780v1
100/100
Inquilinus.limosus.DSM.16000 GCA_000423185.1_ASM42318v1
Micavibrio.aeruginosavorus.ARL.13 GCA_000226315.1_ASM22631v1
100/100
Tistrella.mobilis.KA081020.065 GCA_000264455.2_ASM26445v2
Geminicoccus.roseus.DSM.18922 GCA_000427665.1_ASM42766v1
100/100
Candidatus.Paracaedibacter.acanthamoebae GCA_000742835.1_ASM74283v1
100/100
Candidatus.Odyssella.thessalonicensis.L13 GCA_000190415.2_ASM19041v1
80.1/88
Candidatus.Finniella.inopinata GCA_004210305.1_ASM421030v1
100/100
Candidatus.Caedibacter.acanthamoebae GCA_000743035.1_ASM74303v1
100/100
Caedimonas.varicaedens GCA_001192655.1_ASM119265v1
100/100
Candidatus.Nucleicultrix.amoebiphila.FS5 GCA_002117145.1_ASM211714v1
100/100
Holospora.undulata.HU1 GCA_000388175.3_ASM38817v3
Candidatus.Hepatobacter.penaei GCA_000742475.1_ASM74247v1
100/100
GCA_002702805.1_ASM270280v1*
100/100
GCA_002689325.1_ASM268932v1*
100/100
GCA_002711515.1_ASM271151v1*
100/100
GCA_002691245.1_ASM269124v1*
100/100
GCA_002689845.1_ASM268984v1*
100/100
GCA_002715305.1_ASM271530v1*
100/100
GCA_002706585.1_ASM270658v1*
100/100
GCA_002711625.1_ASM271162v1*
100/100
GCA_002720775.1_ASM272077v1*
100/100
GCA_002713965.1_ASM271396v1*
100/100
GCA_002710765.1_ASM271076v1*
GCA_002691145.1_ASM269114v1*
100/100
GCA_002696825.1_ASM269682v1*
91.9/94
100/100
alpha.proteobacterium.HIMB5 GCA_000299095.1_ASM29909v1
100/100
Candidatus.Pelagibacter.ubique.HTCC1062 GCA_000012345.1_ASM1234v1
100/100
Candidatus.Pelagibacter.ubique.HIMB058 GCA_000419545.1_ASM41954v1
94.6/96
alpha.proteobacterium.HIMB114 GCA_000163555.2
58.3/86
GCA_002937775.1_ASM293777v1*
100/100
GCA_002717245.1_ASM271724v1*
GCA_002720895.1_ASM272089v1*
98.7/100
Candidatus.Neoehrlichia.lotoris.str.RAC413 GCA_000964795.1_ASM96479v1
100/100
Anaplasma.phagocytophilum.str.HZ GCA_000013125.1_ASM1312v1
100/100
Ehrlichia.ruminantium.str.Welgevonden GCA_000026005.1_ASM2600v1
100/100
Wolbachia.endosymbiont.of.Drosophila.melanogaster GCA_000008025.1_ASM802v1
100/100
Candidatus.Deianiraea.vastatrix GCA_007993655.1_ASM799365v1
97/99
100/100
Rickettsiales.endosymbiont.of.Trichoplax.sp.H2 GCA_009690945.1
100/100
Candidatus.Midichloria.mitochondrii.IricVA GCA_000219355.1_ASM21935v1
100/100
Rickettsiales.endosymbiont.of.Peranema.trichophorum GCA_004210275.1_ASM421027v1
Candidatus.Jidaibacter.acanthamoeba GCA_000815465.1.UWC36
100/100
Rickettsia.bellii.RML369.C GCA_000012385.1_ASM1238v1
60.1/90
Candidatus.Megaira.polyxenophila
100/100
Rickettsiales.endosymbiont.of.Stachyamoeba.lipophora GCA_003932735.1_ASM393273v1
Rickettsiales.bacterium.Ac37b GCA_000746585.2_ASM74658v2
100/100
Magnetofaba.australis.IT.1 GCA_002109495.1_ASM210949v1
Magnetococcus.marinus.MC.1 GCA_000014865.1_ASM1486v1
97.2/100
Nitrosospira.briensis.C.128 GCA_000619905.2_ASM61990v2
100/100
Methylotenera.versatilis.301 GCA_000093025.1_ASM9302v1
100/100
Ralstonia.pickettii GCA_902374465.1.MGYG.HGUT.01384
100/100
Burkholderia.cepacia GCA_009586235.1_ASM958623v1
100/100
Dasania.marina.DSM.21967 GCA_000373485.1_ASM37348v1
100/100
Congregibacter.litoralis.KT71 GCA_000153125.2_ASM15312v2
100/100
Enterobacter.cloacae GCA_000770155.1_ASM77015v1
Alteromonas.macleodii.ATCC.27126 GCA_000172635.2_ASM17263v2
0.592946

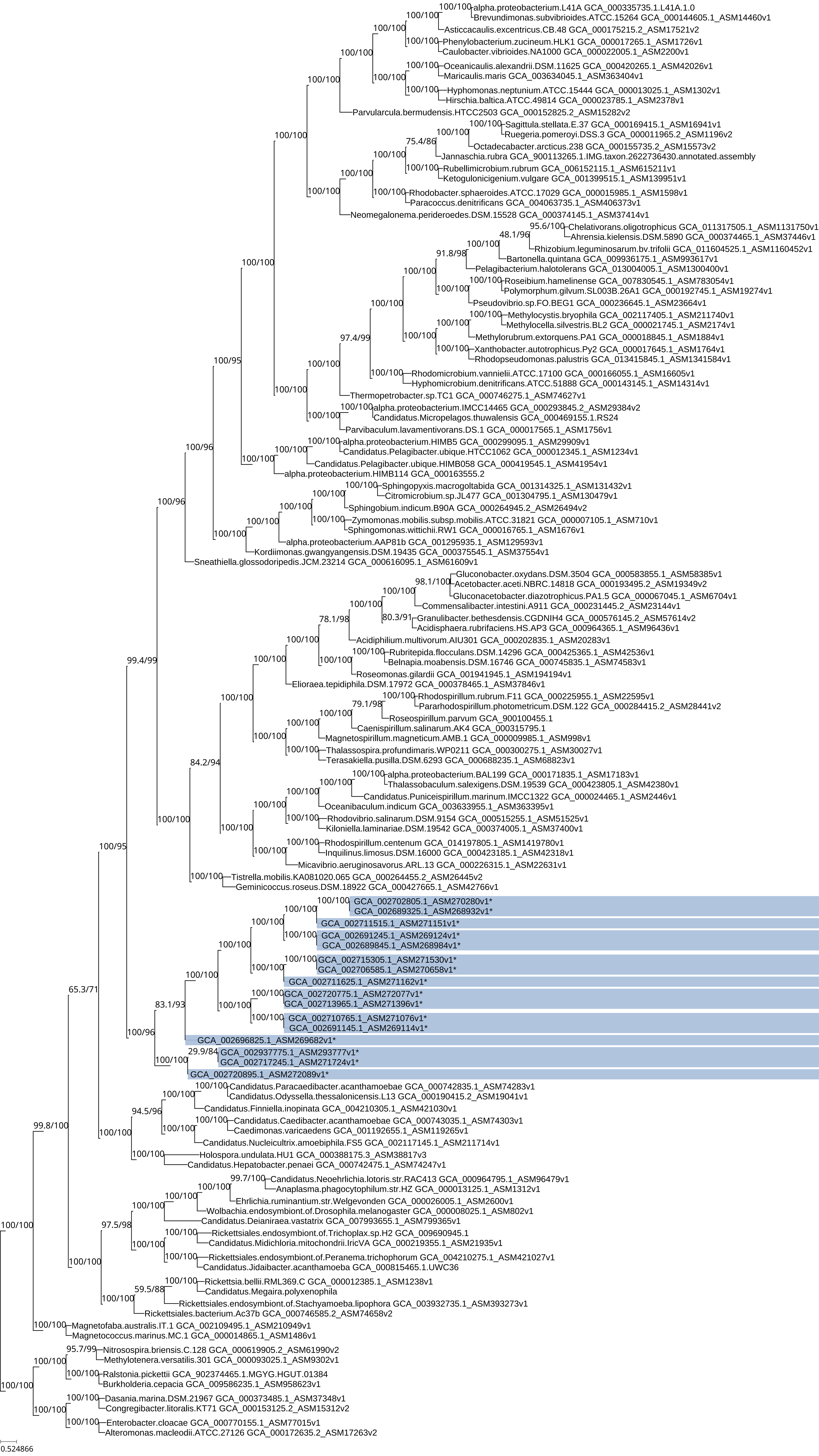
100/100
alpha.proteobacterium.L41A GCA_000335735.1.L41A.1.0
100/100
Brevundimonas.subvibrioides.ATCC.15264 GCA_000144605.1_ASM14460v1
100/100
Asticcacaulis.excentricus.CB.48 GCA_000175215.2_ASM17521v2
100/100
Phenylobacterium.zucineum.HLK1 GCA_000017265.1_ASM1726v1
100/100
Caulobacter.vibrioides.NA1000 GCA_000022005.1_ASM2200v1
100/100
Oceanicaulis.alexandrii.DSM.11625 GCA_000420265.1_ASM42026v1
100/100
Maricaulis.maris GCA_003634045.1_ASM363404v1
100/100
Hyphomonas.neptunium.ATCC.15444 GCA_000013025.1_ASM1302v1
100/100
Hirschia.baltica.ATCC.49814 GCA_000023785.1_ASM2378v1
100/100
Parvularcula.bermudensis.HTCC2503 GCA_000152825.2_ASM15282v2
100/100
100/100
Sagittula.stellata.E.37 GCA_000169415.1_ASM16941v1
Ruegeria.pomeroyi.DSS.3 GCA_000011965.2_ASM1196v2
100/100
Octadecabacter.arcticus.238 GCA_000155735.2_ASM15573v2
75.4/86
100/100
Jannaschia.rubra GCA_900113265.1.IMG.taxon.2622736430.annotated.assembly
100/100
Rubellimicrobium.rubrum GCA_006152115.1_ASM615211v1
100/100
Ketogulonicigenium.vulgare GCA_001399515.1_ASM139951v1
100/100
Rhodobacter.sphaeroides.ATCC.17029 GCA_000015985.1_ASM1598v1
100/100
Paracoccus.denitrificans GCA_004063735.1_ASM406373v1
Neomegalonema.perideroedes.DSM.15528 GCA_000374145.1_ASM37414v1
95.6/100
Chelativorans.oligotrophicus GCA_011317505.1_ASM1131750v1
48.1/96
Ahrensia.kielensis.DSM.5890 GCA_000374465.1_ASM37446v1
100/100
Rhizobium.leguminosarum.bv.trifolii GCA_011604525.1_ASM1160452v1
Bartonella.quintana GCA_009936175.1_ASM993617v1
91.8/98
Pelagibacterium.halotolerans GCA_013004005.1_ASM1300400v1
100/100
100/100
Roseibium.hamelinense GCA_007830545.1_ASM783054v1
100/100
Polymorphum.gilvum.SL003B.26A1 GCA_000192745.1_ASM19274v1
100/100
Pseudovibrio.sp.FO.BEG1 GCA_000236645.1_ASM23664v1
100/100
Methylocystis.bryophila GCA_002117405.1_ASM211740v1
100/100
Methylocella.silvestris.BL2 GCA_000021745.1_ASM2174v1
100/100
Methylorubrum.extorquens.PA1 GCA_000018845.1_ASM1884v1
100/100
Xanthobacter.autotrophicus.Py2 GCA_000017645.1_ASM1764v1
97.4/99
Rhodopseudomonas.palustris GCA_013415845.1_ASM1341584v1
100/100
100/100
Rhodomicrobium.vannielii.ATCC.17100 GCA_000166055.1_ASM16605v1
Hyphomicrobium.denitrificans.ATCC.51888 GCA_000143145.1_ASM14314v1
100/100
Thermopetrobacter.sp.TC1 GCA_000746275.1_ASM74627v1
100/95
100/100
alpha.proteobacterium.IMCC14465 GCA_000293845.2_ASM29384v2
100/100
Candidatus.Micropelagos.thuwalensis GCA_000469155.1.RS24
Parvibaculum.lavamentivorans.DS.1 GCA_000017565.1_ASM1756v1
100/100
alpha.proteobacterium.HIMB5 GCA_000299095.1_ASM29909v1
100/96
100/100
Candidatus.Pelagibacter.ubique.HTCC1062 GCA_000012345.1_ASM1234v1
100/100
Candidatus.Pelagibacter.ubique.HIMB058 GCA_000419545.1_ASM41954v1
alpha.proteobacterium.HIMB114 GCA_000163555.2
100/100
Sphingopyxis.macrogoltabida GCA_001314325.1_ASM131432v1
100/100
Citromicrobium.sp.JL477 GCA_001304795.1_ASM130479v1
100/96
100/100
Sphingobium.indicum.B90A GCA_000264945.2_ASM26494v2
100/100
Zymomonas.mobilis.subsp.mobilis.ATCC.31821 GCA_000007105.1_ASM710v1
100/100
Sphingomonas.wittichii.RW1 GCA_000016765.1_ASM1676v1
100/100
alpha.proteobacterium.AAP81b GCA_001295935.1_ASM129593v1
Kordiimonas.gwangyangensis.DSM.19435 GCA_000375545.1_ASM37554v1
Sneathiella.glossodoripedis.JCM.23214 GCA_000616095.1_ASM61609v1
Gluconobacter.oxydans.DSM.3504 GCA_000583855.1_ASM58385v1
98.1/100
Acetobacter.aceti.NBRC.14818 GCA_000193495.2_ASM19349v2
100/100
Gluconacetobacter.diazotrophicus.PA1.5 GCA_000067045.1_ASM6704v1
Commensalibacter.intestini.A911 GCA_000231445.2_ASM23144v1
100/100
80.3/91
Granulibacter.bethesdensis.CGDNIH4 GCA_000576145.2_ASM57614v2
78.1/98
Acidisphaera.rubrifaciens.HS.AP3 GCA_000964365.1_ASM96436v1
Acidiphilium.multivorum.AIU301 GCA_000202835.1_ASM20283v1
100/100
100/100
Rubritepida.flocculans.DSM.14296 GCA_000425365.1_ASM42536v1
100/100
Belnapia.moabensis.DSM.16746 GCA_000745835.1_ASM74583v1
99.4/99
100/100
Roseomonas.gilardii GCA_001941945.1_ASM194194v1
Elioraea.tepidiphila.DSM.17972 GCA_000378465.1_ASM37846v1
100/100
Rhodospirillum.rubrum.F11 GCA_000225955.1_ASM22595v1
100/100
79.1/98
Pararhodospirillum.photometricum.DSM.122 GCA_000284415.2_ASM28441v2
100/100
Roseospirillum.parvum GCA_900100455.1
100/100
Caenispirillum.salinarum.AK4 GCA_000315795.1
100/100
Magnetospirillum.magneticum.AMB.1 GCA_000009985.1_ASM998v1
100/100
Thalassospira.profundimaris.WP0211 GCA_000300275.1_ASM30027v1
Terasakiella.pusilla.DSM.6293 GCA_000688235.1_ASM68823v1
84.2/94
100/100
alpha.proteobacterium.BAL199 GCA_000171835.1_ASM17183v1
100/100
Thalassobaculum.salexigens.DSM.19539 GCA_000423805.1_ASM42380v1
100/100
Candidatus.Puniceispirillum.marinum.IMCC1322 GCA_000024465.1_ASM2446v1
100/100
Oceanibaculum.indicum GCA_003633955.1_ASM363395v1
100/100
100/100
Rhodovibrio.salinarum.DSM.9154 GCA_000515255.1_ASM51525v1
100/100
Kiloniella.laminariae.DSM.19542 GCA_000374005.1_ASM37400v1
100/100
Rhodospirillum.centenum GCA_014197805.1_ASM1419780v1
100/100
Inquilinus.limosus.DSM.16000 GCA_000423185.1_ASM42318v1
Micavibrio.aeruginosavorus.ARL.13 GCA_000226315.1_ASM22631v1
100/95
100/100
Tistrella.mobilis.KA081020.065 GCA_000264455.2_ASM26445v2
Geminicoccus.roseus.DSM.18922 GCA_000427665.1_ASM42766v1
100/100
GCA_002702805.1_ASM270280v1*
100/100
GCA_002689325.1_ASM268932v1*
100/100
GCA_002711515.1_ASM271151v1*
100/100
GCA_002691245.1_ASM269124v1*
100/100
GCA_002689845.1_ASM268984v1*
100/100
GCA_002715305.1_ASM271530v1*
100/100
100/100
GCA_002706585.1_ASM270658v1*
GCA_002711625.1_ASM271162v1*
65.3/71
100/100
GCA_002720775.1_ASM272077v1*
83.1/93
100/100
GCA_002713965.1_ASM271396v1*
100/100
GCA_002710765.1_ASM271076v1*
GCA_002691145.1_ASM269114v1*
100/96
GCA_002696825.1_ASM269682v1*
29.9/84
GCA_002937775.1_ASM293777v1*
100/100
GCA_002717245.1_ASM271724v1*
GCA_002720895.1_ASM272089v1*
100/100
Candidatus.Paracaedibacter.acanthamoebae GCA_000742835.1_ASM74283v1
100/100
Candidatus.Odyssella.thessalonicensis.L13 GCA_000190415.2_ASM19041v1
94.5/96
Candidatus.Finniella.inopinata GCA_004210305.1_ASM421030v1
100/100
Candidatus.Caedibacter.acanthamoebae GCA_000743035.1_ASM74303v1
99.8/100
100/100
100/100
Caedimonas.varicaedens GCA_001192655.1_ASM119265v1
Candidatus.Nucleicultrix.amoebiphila.FS5 GCA_002117145.1_ASM211714v1
100/100
Holospora.undulata.HU1 GCA_000388175.3_ASM38817v3
Candidatus.Hepatobacter.penaei GCA_000742475.1_ASM74247v1
99.7/100
Candidatus.Neoehrlichia.lotoris.str.RAC413 GCA_000964795.1_ASM96479v1
100/100
Anaplasma.phagocytophilum.str.HZ GCA_000013125.1_ASM1312v1
100/100
Ehrlichia.ruminantium.str.Welgevonden GCA_000026005.1_ASM2600v1
100/100
Wolbachia.endosymbiont.of.Drosophila.melanogaster GCA_000008025.1_ASM802v1
97.5/98
Candidatus.Deianiraea.vastatrix GCA_007993655.1_ASM799365v1
100/100
Rickettsiales.endosymbiont.of.Trichoplax.sp.H2 GCA_009690945.1
100/100
100/100
Candidatus.Midichloria.mitochondrii.IricVA GCA_000219355.1_ASM21935v1
100/100
Rickettsiales.endosymbiont.of.Peranema.trichophorum GCA_004210275.1_ASM421027v1
100/100
Candidatus.Jidaibacter.acanthamoeba GCA_000815465.1.UWC36
100/100
Rickettsia.bellii.RML369.C GCA_000012385.1_ASM1238v1
59.5/88
Candidatus.Megaira.polyxenophila
100/100
Rickettsiales.endosymbiont.of.Stachyamoeba.lipophora GCA_003932735.1_ASM393273v1
Rickettsiales.bacterium.Ac37b GCA_000746585.2_ASM74658v2
100/100
Magnetofaba.australis.IT.1 GCA_002109495.1_ASM210949v1
Magnetococcus.marinus.MC.1 GCA_000014865.1_ASM1486v1
95.7/99
Nitrosospira.briensis.C.128 GCA_000619905.2_ASM61990v2
100/100
Methylotenera.versatilis.301 GCA_000093025.1_ASM9302v1
100/100
Ralstonia.pickettii GCA_902374465.1.MGYG.HGUT.01384
100/100
Burkholderia.cepacia GCA_009586235.1_ASM958623v1
100/100
Dasania.marina.DSM.21967 GCA_000373485.1_ASM37348v1
100/100
Congregibacter.litoralis.KT71 GCA_000153125.2_ASM15312v2
100/100
Enterobacter.cloacae GCA_000770155.1_ASM77015v1
Alteromonas.macleodii.ATCC.27126 GCA_000172635.2_ASM17263v2
0.524866

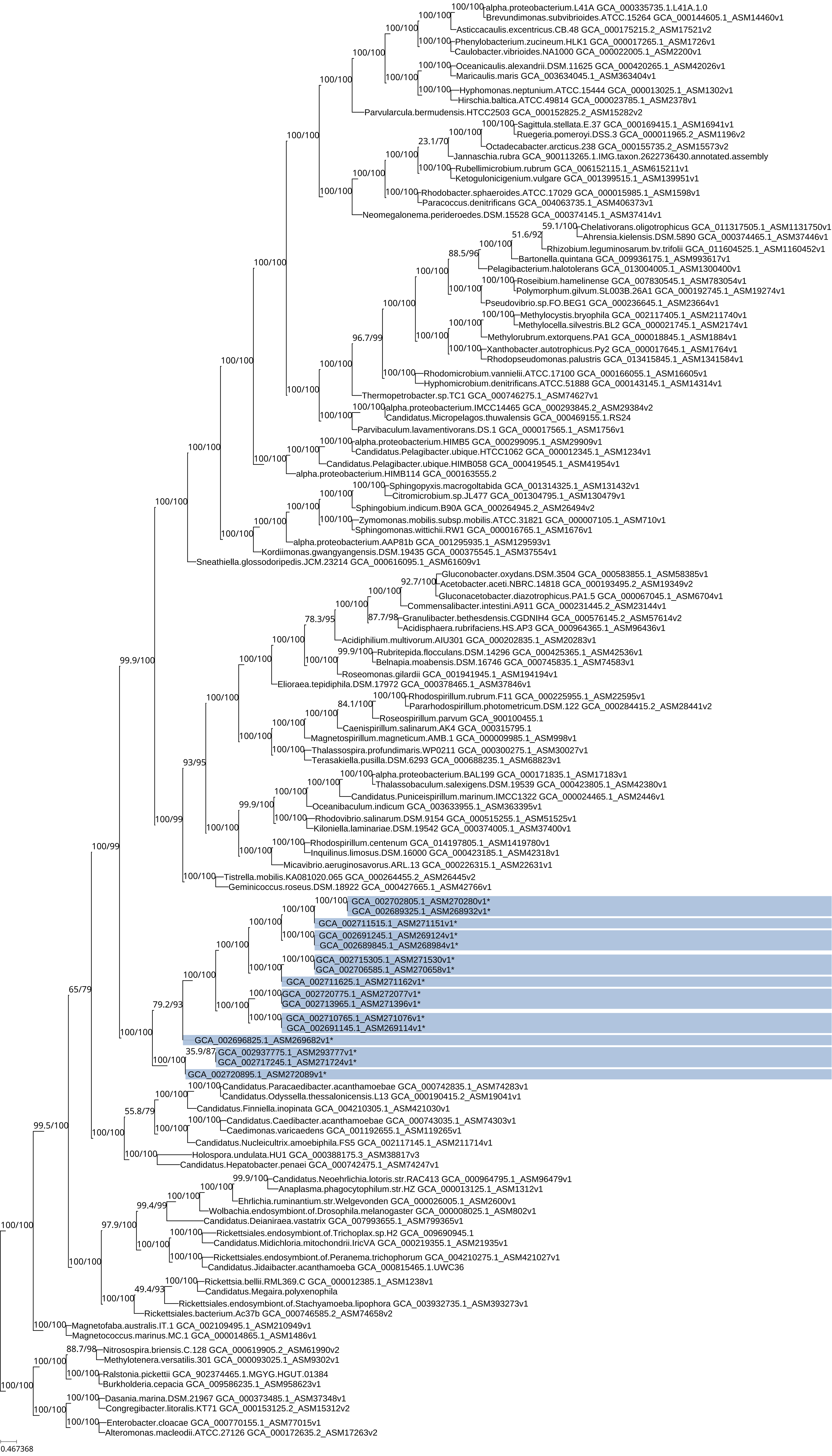
alpha.proteobacterium.L41A GCA_000335735.1.L41A.1.0
Brevundimonas.subvibrioides.ATCC.15264 GCA_000144605.1_ASM14460v1
Asticcacaulis.excentricus.CB.48 GCA_000175215.2_ASM17521v2
Phenylobacterium.zucineum.HLK1 GCA_000017265.1_ASM1726v1
Caulobacter.vibrioides.NA1000 GCA_000022005.1_ASM2200v1
Oceanicaulis.alexandrii.DSM.11625 GCA_000420265.1_ASM42026v1
Maricaulis.maris GCA_003634045.1_ASM363404v1
Hyphomonas.neptunium.ATCC.15444 GCA_000013025.1_ASM1302v1
Hirschia.baltica.ATCC.49814 GCA_000023785.1_ASM2378v1
Parvularcula.bermudensis.HTCC2503 GCA_000152825.2_ASM15282v2
Sagittula.stellata.E.37 GCA_000169415.1_ASM16941v1
Ruegeria.pomeroyi.DSS.3 GCA_000011965.2_ASM1196v2
Octadecabacter.arcticus.238 GCA_000155735.2_ASM15573v2
Jannaschia.rubra GCA_900113265.1.IMG.taxon.2622736430.annotated.assembly
Rubellimicrobium.rubrum GCA_006152115.1_ASM615211v1
Ketogulonicigenium.vulgare GCA_001399515.1_ASM139951v1
Rhodobacter.sphaeroides.ATCC.17029 GCA_000015985.1_ASM1598v1
Paracoccus.denitrificans GCA_004063735.1_ASM406373v1
Neomegalonema.perideroedes.DSM.15528 GCA_000374145.1_ASM37414v1
Chelativorans.oligotrophicus GCA_011317505.1_ASM1131750v1
Ahrensia.kielensis.DSM.5890 GCA_000374465.1_ASM37446v1
Rhizobium.leguminosarum.bv.trifolii GCA_011604525.1_ASM1160452v1
Bartonella.quintana GCA_009936175.1_ASM993617v1
Pelagibacterium.halotolerans GCA_013004005.1_ASM1300400v1
Roseibium.hamelinense GCA_007830545.1_ASM783054v1
Polymorphum.gilvum.SL003B.26A1 GCA_000192745.1_ASM19274v1
Pseudovibrio.sp.FO.BEG1 GCA_000236645.1_ASM23664v1
Methylocystis.bryophila GCA_002117405.1_ASM211740v1
Methylocella.silvestris.BL2 GCA_000021745.1_ASM2174v1
Methylorubrum.extorquens.PA1 GCA_000018845.1_ASM1884v1
Xanthobacter.autotrophicus.Py2 GCA_000017645.1_ASM1764v1
Rhodopseudomonas.palustris GCA_013415845.1_ASM1341584v1
Rhodomicrobium.vannielii.ATCC.17100 GCA_000166055.1_ASM16605v1
Hyphomicrobium.denitrificans.ATCC.51888 GCA_000143145.1_ASM14314v1
Thermopetrobacter.sp.TC1 GCA_000746275.1_ASM74627v1
alpha.proteobacterium.IMCC14465 GCA_000293845.2_ASM29384v2
Candidatus.Micropelagos.thuwalensis GCA_000469155.1.RS24
Parvibaculum.lavamentivorans.DS.1 GCA_000017565.1_ASM1756v1
alpha.proteobacterium.HIMB5 GCA_000299095.1_ASM29909v1
Candidatus.Pelagibacter.ubique.HTCC1062 GCA_000012345.1_ASM1234v1
Candidatus.Pelagibacter.ubique.HIMB058 GCA_000419545.1_ASM41954v1
alpha.proteobacterium.HIMB114 GCA_000163555.2
Sphingopyxis.macrogoltabida GCA_001314325.1_ASM131432v1
Citromicrobium.sp.JL477 GCA_001304795.1_ASM130479v1
Sphingobium.indicum.B90A GCA_000264945.2_ASM26494v2
Zymomonas.mobilis.subsp.mobilis.ATCC.31821 GCA_000007105.1_ASM710v1
Sphingomonas.wittichii.RW1 GCA_000016765.1_ASM1676v1
alpha.proteobacterium.AAP81b GCA_001295935.1_ASM129593v1
Kordiimonas.gwangyangensis.DSM.19435 GCA_000375545.1_ASM37554v1
Sneathiella.glossodoripedis.JCM.23214 GCA_000616095.1_ASM61609v1
Gluconobacter.oxydans.DSM.3504 GCA_000583855.1_ASM58385v1
Acetobacter.aceti.NBRC.14818 GCA_000193495.2_ASM19349v2
Gluconacetobacter.diazotrophicus.PA1.5 GCA_000067045.1_ASM6704v1
Commensalibacter.intestini.A911 GCA_000231445.2_ASM23144v1
Granulibacter.bethesdensis.CGDNIH4 GCA_000576145.2_ASM57614v2
Acidisphaera.rubrifaciens.HS.AP3 GCA_000964365.1_ASM96436v1
Acidiphilium.multivorum.AIU301 GCA_000202835.1_ASM20283v1
Rubritepida.flocculans.DSM.14296 GCA_000425365.1_ASM42536v1
Belnapia.moabensis.DSM.16746 GCA_000745835.1_ASM74583v1
Roseomonas.gilardii GCA_001941945.1_ASM194194v1
Elioraea.tepidiphila.DSM.17972 GCA_000378465.1_ASM37846v1
Rhodospirillum.rubrum.F11 GCA_000225955.1_ASM22595v1
Pararhodospirillum.photometricum.DSM.122 GCA_000284415.2_ASM28441v2
Roseospirillum.parvum GCA_900100455.1
Caenispirillum.salinarum.AK4 GCA_000315795.1
Magnetospirillum.magneticum.AMB.1 GCA_000009985.1_ASM998v1
Thalassospira.profundimaris.WP0211 GCA_000300275.1_ASM30027v1
Terasakiella.pusilla.DSM.6293 GCA_000688235.1_ASM68823v1
alpha.proteobacterium.BAL199 GCA_000171835.1_ASM17183v1
Thalassobaculum.salexigens.DSM.19539 GCA_000423805.1_ASM42380v1
Candidatus.Puniceispirillum.marinum.IMCC1322 GCA_000024465.1_ASM2446v1
Oceanibaculum.indicum GCA_003633955.1_ASM363395v1
Rhodovibrio.salinarum.DSM.9154 GCA_000515255.1_ASM51525v1
Kiloniella.laminariae.DSM.19542 GCA_000374005.1_ASM37400v1
Rhodospirillum.centenum GCA_014197805.1_ASM1419780v1
Inquilinus.limosus.DSM.16000 GCA_000423185.1_ASM42318v1
Micavibrio.aeruginosavorus.ARL.13 GCA_000226315.1_ASM22631v1
Tistrella.mobilis.KA081020.065 GCA_000264455.2_ASM26445v2
Geminicoccus.roseus.DSM.18922 GCA_000427665.1_ASM42766v1
GCA_002702805.1_ASM270280v1*
GCA_002689325.1_ASM268932v1*
GCA_002711515.1_ASM271151v1*
GCA_002691245.1_ASM269124v1*
GCA_002689845.1_ASM268984v1*
GCA_002715305.1_ASM271530v1*
GCA_002706585.1_ASM270658v1*
GCA_002711625.1_ASM271162v1*
GCA_002720775.1_ASM272077v1*
GCA_002713965.1_ASM271396v1*
GCA_002710765.1_ASM271076v1*
GCA_002691145.1_ASM269114v1*
GCA_002696825.1_ASM269682v1*
GCA_002937775.1_ASM293777v1*
GCA_002717245.1_ASM271724v1*
GCA_002720895.1_ASM272089v1*
Candidatus.Paracaedibacter.acanthamoebae GCA_000742835.1_ASM74283v1
Candidatus.Odyssella.thessalonicensis.L13 GCA_000190415.2_ASM19041v1
Candidatus.Finniella.inopinata GCA_004210305.1_ASM421030v1
Candidatus.Caedibacter.acanthamoebae GCA_000743035.1_ASM74303v1
Caedimonas.varicaedens GCA_001192655.1_ASM119265v1
Candidatus.Nucleicultrix.amoebiphila.FS5 GCA_002117145.1_ASM211714v1
Holospora.undulata.HU1 GCA_000388175.3_ASM38817v3
Candidatus.Hepatobacter.penaei GCA_000742475.1_ASM74247v1
Candidatus.Neoehrlichia.lotoris.str.RAC413 GCA_000964795.1_ASM96479v1
Anaplasma.phagocytophilum.str.HZ GCA_000013125.1_ASM1312v1
Ehrlichia.ruminantium.str.Welgevonden GCA_000026005.1_ASM2600v1
Wolbachia.endosymbiont.of.Drosophila.melanogaster GCA_000008025.1_ASM802v1
Candidatus.Deianiraea.vastatrix GCA_007993655.1_ASM799365v1
Rickettsiales.endosymbiont.of.Trichoplax.sp.H2 GCA_009690945.1
Candidatus.Midichloria.mitochondrii.IricVA GCA_000219355.1_ASM21935v1
Rickettsiales.endosymbiont.of.Peranema.trichophorum GCA_004210275.1_ASM421027v1
Candidatus.Jidaibacter.acanthamoeba GCA_000815465.1.UWC36
Rickettsia.bellii.RML369.C GCA_000012385.1_ASM1238v1
Candidatus.Megaira.polyxenophila
Rickettsiales.endosymbiont.of.Stachyamoeba.lipophora GCA_003932735.1_ASM393273v1
Rickettsiales.bacterium.Ac37b GCA_000746585.2_ASM74658v2
Magnetofaba.australis.IT.1 GCA_002109495.1_ASM210949v1
Magnetococcus.marinus.MC.1 GCA_000014865.1_ASM1486v1
Nitrosospira.briensis.C.128 GCA_000619905.2_ASM61990v2
Methylotenera.versatilis.301 GCA_000093025.1_ASM9302v1
Ralstonia.pickettii GCA_902374465.1.MGYG.HGUT.01384
Burkholderia.cepacia GCA_009586235.1_ASM958623v1
Dasania.marina.DSM.21967 GCA_000373485.1_ASM37348v1
Congregibacter.litoralis.KT71 GCA_000153125.2_ASM15312v2
Enterobacter.cloacae GCA_000770155.1_ASM77015v1
Alteromonas.macleodii.ATCC.27126 GCA_000172635.2_ASM17263v2
0.467368

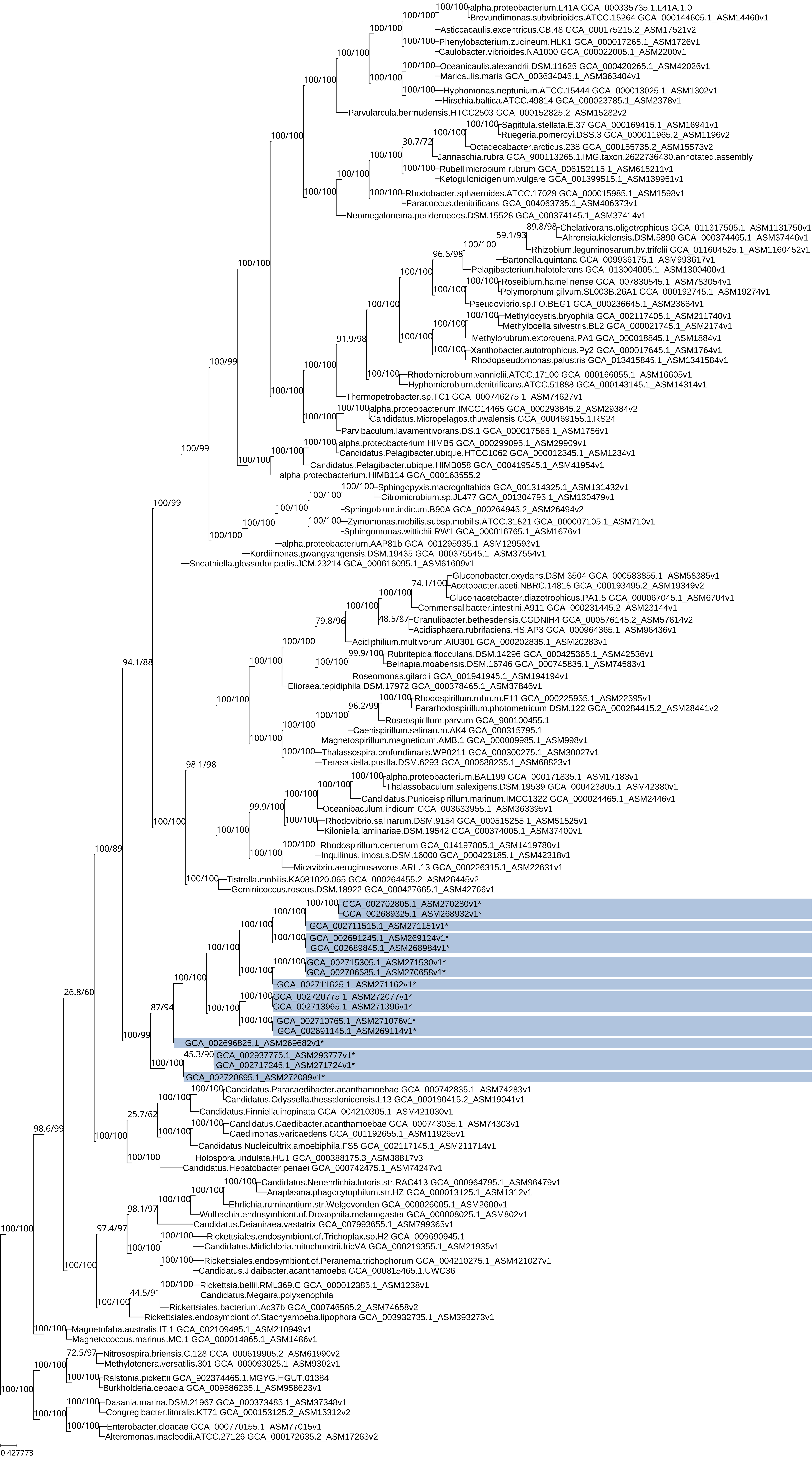
alpha.proteobacterium.L41A GCA_000335735.1.L41A.1.0
Brevundimonas.subvibrioides.ATCC.15264 GCA_000144605.1_ASM14460v1
Asticcacaulis.excentricus.CB.48 GCA_000175215.2_ASM17521v2
Phenylobacterium.zucineum.HLK1 GCA_000017265.1_ASM1726v1
Caulobacter.vibrioides.NA1000 GCA_000022005.1_ASM2200v1
Oceanicaulis.alexandrii.DSM.11625 GCA_000420265.1_ASM42026v1
Maricaulis.maris GCA_003634045.1_ASM363404v1
Hyphomonas.neptunium.ATCC.15444 GCA_000013025.1_ASM1302v1
Hirschia.baltica.ATCC.49814 GCA_000023785.1_ASM2378v1
Parvularcula.bermudensis.HTCC2503 GCA_000152825.2_ASM15282v2
Sagittula.stellata.E.37 GCA_000169415.1_ASM16941v1
Ruegeria.pomeroyi.DSS.3 GCA_000011965.2_ASM1196v2
Octadecabacter.arcticus.238 GCA_000155735.2_ASM15573v2
Jannaschia.rubra GCA_900113265.1.IMG.taxon.2622736430.annotated.assembly
Rubellimicrobium.rubrum GCA_006152115.1_ASM615211v1
Ketogulonicigenium.vulgare GCA_001399515.1_ASM139951v1
Rhodobacter.sphaeroides.ATCC.17029 GCA_000015985.1_ASM1598v1
Paracoccus.denitrificans GCA_004063735.1_ASM406373v1
Neomegalonema.perideroedes.DSM.15528 GCA_000374145.1_ASM37414v1
Chelativorans.oligotrophicus GCA_011317505.1_ASM1131750v1
Ahrensia.kielensis.DSM.5890 GCA_000374465.1_ASM37446v1
Rhizobium.leguminosarum.bv.trifolii GCA_011604525.1_ASM1160452v1
Bartonella.quintana GCA_009936175.1_ASM993617v1
Pelagibacterium.halotolerans GCA_013004005.1_ASM1300400v1
Roseibium.hamelinense GCA_007830545.1_ASM783054v1
Polymorphum.gilvum.SL003B.26A1 GCA_000192745.1_ASM19274v1
Pseudovibrio.sp.FO.BEG1 GCA_000236645.1_ASM23664v1
Methylocystis.bryophila GCA_002117405.1_ASM211740v1
Methylocella.silvestris.BL2 GCA_000021745.1_ASM2174v1
Methylorubrum.extorquens.PA1 GCA_000018845.1_ASM1884v1
Xanthobacter.autotrophicus.Py2 GCA_000017645.1_ASM1764v1
Rhodopseudomonas.palustris GCA_013415845.1_ASM1341584v1
Rhodomicrobium.vannielii.ATCC.17100 GCA_000166055.1_ASM16605v1
Hyphomicrobium.denitrificans.ATCC.51888 GCA_000143145.1_ASM14314v1
Thermopetrobacter.sp.TC1 GCA_000746275.1_ASM74627v1
alpha.proteobacterium.IMCC14465 GCA_000293845.2_ASM29384v2
Candidatus.Micropelagos.thuwalensis GCA_000469155.1.RS24
Parvibaculum.lavamentivorans.DS.1 GCA_000017565.1_ASM1756v1
alpha.proteobacterium.HIMB5 GCA_000299095.1_ASM29909v1
Candidatus.Pelagibacter.ubique.HTCC1062 GCA_000012345.1_ASM1234v1
Candidatus.Pelagibacter.ubique.HIMB058 GCA_000419545.1_ASM41954v1
alpha.proteobacterium.HIMB114 GCA_000163555.2
Sphingopyxis.macrogoltabida GCA_001314325.1_ASM131432v1
Citromicrobium.sp.JL477 GCA_001304795.1_ASM130479v1
Sphingobium.indicum.B90A GCA_000264945.2_ASM26494v2
Zymomonas.mobilis.subsp.mobilis.ATCC.31821 GCA_000007105.1_ASM710v1
Sphingomonas.wittichii.RW1 GCA_000016765.1_ASM1676v1
alpha.proteobacterium.AAP81b GCA_001295935.1_ASM129593v1
Kordiimonas.gwangyangensis.DSM.19435 GCA_000375545.1_ASM37554v1
Sneathiella.glossodoripedis.JCM.23214 GCA_000616095.1_ASM61609v1
Gluconobacter.oxydans.DSM.3504 GCA_000583855.1_ASM58385v1
Acetobacter.aceti.NBRC.14818 GCA_000193495.2_ASM19349v2
Gluconacetobacter.diazotrophicus.PA1.5 GCA_000067045.1_ASM6704v1
Commensalibacter.intestini.A911 GCA_000231445.2_ASM23144v1
Granulibacter.bethesdensis.CGDNIH4 GCA_000576145.2_ASM57614v2
Acidisphaera.rubrifaciens.HS.AP3 GCA_000964365.1_ASM96436v1
Acidiphilium.multivorum.AIU301 GCA_000202835.1_ASM20283v1
Rubritepida.flocculans.DSM.14296 GCA_000425365.1_ASM42536v1
Belnapia.moabensis.DSM.16746 GCA_000745835.1_ASM74583v1
Roseomonas.gilardii GCA_001941945.1_ASM194194v1
Elioraea.tepidiphila.DSM.17972 GCA_000378465.1_ASM37846v1
Rhodospirillum.rubrum.F11 GCA_000225955.1_ASM22595v1
Pararhodospirillum.photometricum.DSM.122 GCA_000284415.2_ASM28441v2
Roseospirillum.parvum GCA_900100455.1
Caenispirillum.salinarum.AK4 GCA_000315795.1
Magnetospirillum.magneticum.AMB.1 GCA_000009985.1_ASM998v1
Thalassospira.profundimaris.WP0211 GCA_000300275.1_ASM30027v1
Terasakiella.pusilla.DSM.6293 GCA_000688235.1_ASM68823v1
alpha.proteobacterium.BAL199 GCA_000171835.1_ASM17183v1
Thalassobaculum.salexigens.DSM.19539 GCA_000423805.1_ASM42380v1
Candidatus.Puniceispirillum.marinum.IMCC1322 GCA_000024465.1_ASM2446v1
Oceanibaculum.indicum GCA_003633955.1_ASM363395v1
Rhodovibrio.salinarum.DSM.9154 GCA_000515255.1_ASM51525v1
Kiloniella.laminariae.DSM.19542 GCA_000374005.1_ASM37400v1
Rhodospirillum.centenum GCA_014197805.1_ASM1419780v1
Inquilinus.limosus.DSM.16000 GCA_000423185.1_ASM42318v1
Micavibrio.aeruginosavorus.ARL.13 GCA_000226315.1_ASM22631v1
Tistrella.mobilis.KA081020.065 GCA_000264455.2_ASM26445v2
Geminicoccus.roseus.DSM.18922 GCA_000427665.1_ASM42766v1
GCA_002702805.1_ASM270280v1*
GCA_002689325.1_ASM268932v1*
GCA_002711515.1_ASM271151v1*
GCA_002691245.1_ASM269124v1*
GCA_002689845.1_ASM268984v1*
GCA_002715305.1_ASM271530v1*
GCA_002706585.1_ASM270658v1*
GCA_002711625.1_ASM271162v1*
GCA_002720775.1_ASM272077v1*
GCA_002713965.1_ASM271396v1*
GCA_002710765.1_ASM271076v1*
GCA_002691145.1_ASM269114v1*
GCA_002696825.1_ASM269682v1*
GCA_002937775.1_ASM293777v1*
GCA_002717245.1_ASM271724v1*
GCA_002720895.1_ASM272089v1*
Candidatus.Paracaedibacter.acanthamoebae GCA_000742835.1_ASM74283v1
Candidatus.Odyssella.thessalonicensis.L13 GCA_000190415.2_ASM19041v1
Candidatus.Finniella.inopinata GCA_004210305.1_ASM421030v1
Candidatus.Caedibacter.acanthamoebae GCA_000743035.1_ASM74303v1
Caedimonas.varicaedens GCA_001192655.1_ASM119265v1
Candidatus.Nucleicultrix.amoebiphila.FS5 GCA_002117145.1_ASM211714v1
Holospora.undulata.HU1 GCA_000388175.3_ASM38817v3
Candidatus.Hepatobacter.penaei GCA_000742475.1_ASM74247v1
Candidatus.Neoehrlichia.lotoris.str.RAC413 GCA_000964795.1_ASM96479v1
Anaplasma.phagocytophilum.str.HZ GCA_000013125.1_ASM1312v1
Ehrlichia.ruminantium.str.Welgevonden GCA_000026005.1_ASM2600v1
Wolbachia.endosymbiont.of.Drosophila.melanogaster GCA_000008025.1_ASM802v1
Candidatus.Deianiraea.vastatrix GCA_007993655.1_ASM799365v1
Rickettsiales.endosymbiont.of.Trichoplax.sp.H2 GCA_009690945.1
Candidatus.Midichloria.mitochondrii.IricVA GCA_000219355.1_ASM21935v1
Rickettsiales.endosymbiont.of.Peranema.trichophorum GCA_004210275.1_ASM421027v1
Candidatus.Jidaibacter.acanthamoeba GCA_000815465.1.UWC36
Rickettsia.bellii.RML369.C GCA_000012385.1_ASM1238v1
Candidatus.Megaira.polyxenophila
Rickettsiales.bacterium.Ac37b GCA_000746585.2_ASM74658v2
Rickettsiales.endosymbiont.of.Stachyamoeba.lipophora GCA_003932735.1_ASM393273v1
Magnetofaba.australis.IT.1 GCA_002109495.1_ASM210949v1
Magnetococcus.marinus.MC.1 GCA_000014865.1_ASM1486v1
Nitrosospira.briensis.C.128 GCA_000619905.2_ASM61990v2
Methylotenera.versatilis.301 GCA_000093025.1_ASM9302v1
Ralstonia.pickettii GCA_902374465.1.MGYG.HGUT.01384
Burkholderia.cepacia GCA_009586235.1_ASM958623v1
Dasania.marina.DSM.21967 GCA_000373485.1_ASM37348v1
Congregibacter.litoralis.KT71 GCA_000153125.2_ASM15312v2
Enterobacter.cloacae GCA_000770155.1_ASM77015v1
Alteromonas.macleodii.ATCC.27126 GCA_000172635.2_ASM17263v2
0.427773

Group M

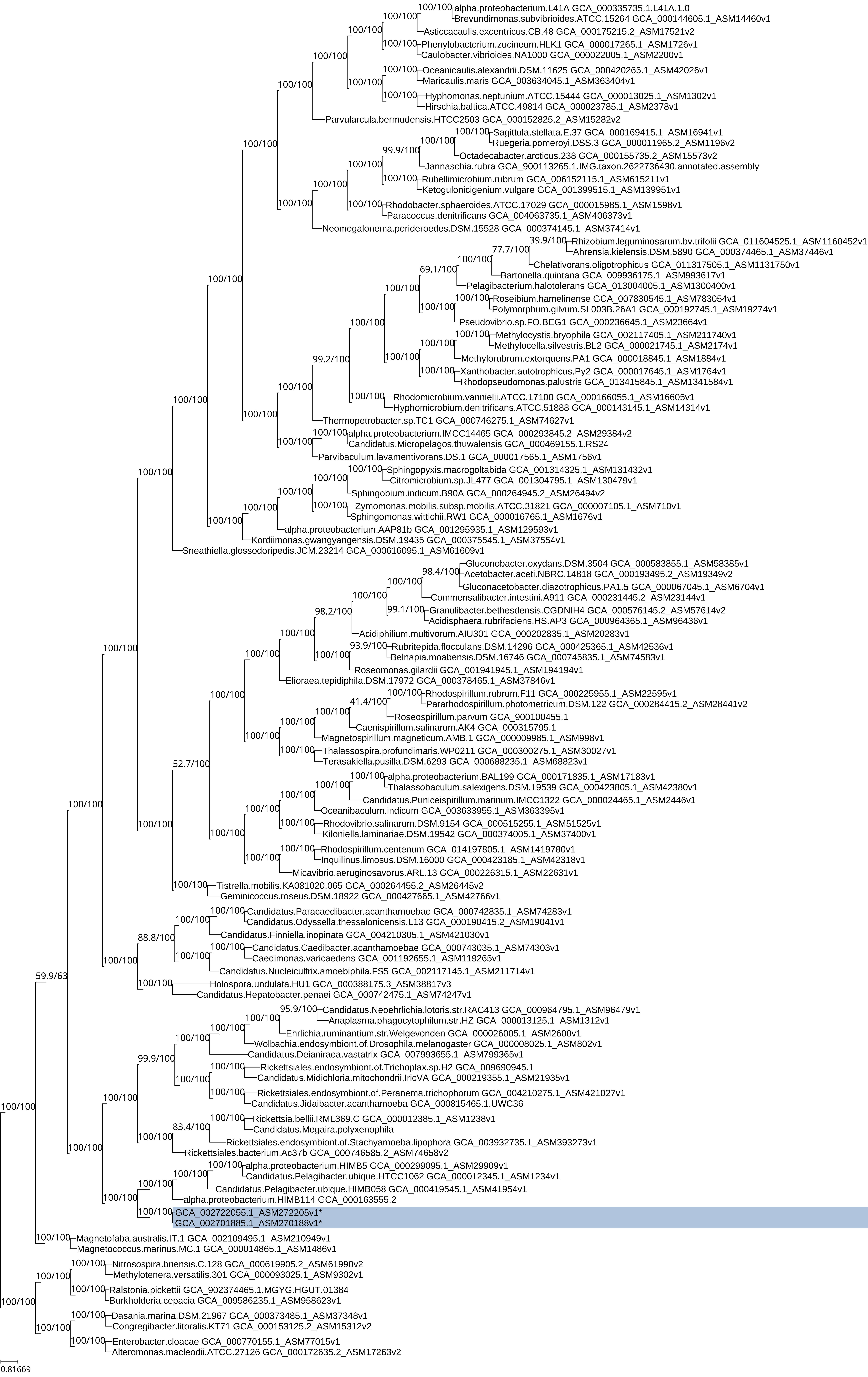
alpha.proteobacterium.L41A GCA_000335735.1.L41A.1.0
Brevundimonas.subvibrioides.ATCC.15264 GCA_000144605.1_ASM14460v1
Asticcacaulis.excentricus.CB.48 GCA_000175215.2_ASM17521v2
Phenylobacterium.zucineum.HLK1 GCA_000017265.1_ASM1726v1
Caulobacter.vibrioides.NA1000 GCA_000022005.1_ASM2200v1
Oceanicaulis.alexandrii.DSM.11625 GCA_000420265.1_ASM42026v1
Maricaulis.maris GCA_003634045.1_ASM363404v1
Hyphomonas.neptunium.ATCC.15444 GCA_000013025.1_ASM1302v1
Hirschia.baltica.ATCC.49814 GCA_000023785.1_ASM2378v1
Parvularcula.bermudensis.HTCC2503 GCA_000152825.2_ASM15282v2
Sagittula.stellata.E.37 GCA_000169415.1_ASM16941v1
Ruegeria.pomeroyi.DSS.3 GCA_000011965.2_ASM1196v2
Octadecabacter.arcticus.238 GCA_000155735.2_ASM15573v2
Jannaschia.rubra GCA_900113265.1.IMG.taxon.2622736430.annotated.assembly
Rubellimicrobium.rubrum GCA_006152115.1_ASM615211v1
Ketogulonicigenium.vulgare GCA_001399515.1_ASM139951v1
Rhodobacter.sphaeroides.ATCC.17029 GCA_000015985.1_ASM1598v1
Paracoccus.denitrificans GCA_004063735.1_ASM406373v1
Neomegalonema.perideroedes.DSM.15528 GCA_000374145.1_ASM37414v1
Rhizobium.leguminosarum.bv.trifolii GCA_011604525.1_ASM1160452v1
Ahrensia.kielensis.DSM.5890 GCA_000374465.1_ASM37446v1
Chelativorans.oligotrophicus GCA_011317505.1_ASM1131750v1
Bartonella.quintana GCA_009936175.1_ASM993617v1
Pelagibacterium.halotolerans GCA_013004005.1_ASM1300400v1
Roseibium.hamelinense GCA_007830545.1_ASM783054v1
Polymorphum.gilvum.SL003B.26A1 GCA_000192745.1_ASM19274v1
Pseudovibrio.sp.FO.BEG1 GCA_000236645.1_ASM23664v1
Methylocystis.bryophila GCA_002117405.1_ASM211740v1
Methylocella.silvestris.BL2 GCA_000021745.1_ASM2174v1
Methylorubrum.extorquens.PA1 GCA_000018845.1_ASM1884v1
Xanthobacter.autotrophicus.Py2 GCA_000017645.1_ASM1764v1
Rhodopseudomonas.palustris GCA_013415845.1_ASM1341584v1
Rhodomicrobium.vannielii.ATCC.17100 GCA_000166055.1_ASM16605v1
Hyphomicrobium.denitrificans.ATCC.51888 GCA_000143145.1_ASM14314v1
Thermopetrobacter.sp.TC1 GCA_000746275.1_ASM74627v1
alpha.proteobacterium.IMCC14465 GCA_000293845.2_ASM29384v2
Candidatus.Micropelagos.thuwalensis GCA_000469155.1.RS24
Parvibaculum.lavamentivorans.DS.1 GCA_000017565.1_ASM1756v1
Sphingopyxis.macrogoltabida GCA_001314325.1_ASM131432v1
Citromicrobium.sp.JL477 GCA_001304795.1_ASM130479v1
Sphingobium.indicum.B90A GCA_000264945.2_ASM26494v2
Zymomonas.mobilis.subsp.mobilis.ATCC.31821 GCA_000007105.1_ASM710v1
Sphingomonas.wittichii.RW1 GCA_000016765.1_ASM1676v1
alpha.proteobacterium.AAP81b GCA_001295935.1_ASM129593v1
Kordiimonas.gwangyangensis.DSM.19435 GCA_000375545.1_ASM37554v1
Sneathiella.glossodoripedis.JCM.23214 GCA_000616095.1_ASM61609v1
Gluconobacter.oxydans.DSM.3504 GCA_000583855.1_ASM58385v1
Acetobacter.aceti.NBRC.14818 GCA_000193495.2_ASM19349v2
Gluconacetobacter.diazotrophicus.PA1.5 GCA_000067045.1_ASM6704v1
Commensalibacter.intestini.A911 GCA_000231445.2_ASM23144v1
Granulibacter.bethesdensis.CGDNIH4 GCA_000576145.2_ASM57614v2
Acidisphaera.rubrifaciens.HS.AP3 GCA_000964365.1_ASM96436v1
Acidiphilium.multivorum.AIU301 GCA_000202835.1_ASM20283v1
Rubritepida.flocculans.DSM.14296 GCA_000425365.1_ASM42536v1
Belnapia.moabensis.DSM.16746 GCA_000745835.1_ASM74583v1
Roseomonas.gilardii GCA_001941945.1_ASM194194v1
Elioraea.tepidiphila.DSM.17972 GCA_000378465.1_ASM37846v1
Rhodospirillum.rubrum.F11 GCA_000225955.1_ASM22595v1
Pararhodospirillum.photometricum.DSM.122 GCA_000284415.2_ASM28441v2
Roseospirillum.parvum GCA_900100455.1
Caenispirillum.salinarum.AK4 GCA_000315795.1
Magnetospirillum.magneticum.AMB.1 GCA_000009985.1_ASM998v1
Thalassospira.profundimaris.WP0211 GCA_000300275.1_ASM30027v1
Terasakiella.pusilla.DSM.6293 GCA_000688235.1_ASM68823v1
alpha.proteobacterium.BAL199 GCA_000171835.1_ASM17183v1
Thalassobaculum.salexigens.DSM.19539 GCA_000423805.1_ASM42380v1
Candidatus.Puniceispirillum.marinum.IMCC1322 GCA_000024465.1_ASM2446v1
Oceanibaculum.indicum GCA_003633955.1_ASM363395v1
Rhodovibrio.salinarum.DSM.9154 GCA_000515255.1_ASM51525v1
Kiloniella.laminariae.DSM.19542 GCA_000374005.1_ASM37400v1
Rhodospirillum.centenum GCA_014197805.1_ASM1419780v1
Inquilinus.limosus.DSM.16000 GCA_000423185.1_ASM42318v1
Micavibrio.aeruginosavorus.ARL.13 GCA_000226315.1_ASM22631v1
Tistrella.mobilis.KA081020.065 GCA_000264455.2_ASM26445v2
Geminicoccus.roseus.DSM.18922 GCA_000427665.1_ASM42766v1
Candidatus.Paracaedibacter.acanthamoebae GCA_000742835.1_ASM74283v1
Candidatus.Odyssella.thessalonicensis.L13 GCA_000190415.2_ASM19041v1
Candidatus.Finniella.inopinata GCA_004210305.1_ASM421030v1
Candidatus.Caedibacter.acanthamoebae GCA_000743035.1_ASM74303v1
Caedimonas.varicaedens GCA_001192655.1_ASM119265v1
Candidatus.Nucleicultrix.amoebiphila.FS5 GCA_002117145.1_ASM211714v1
Holospora.undulata.HU1 GCA_000388175.3_ASM38817v3
Candidatus.Hepatobacter.penaei GCA_000742475.1_ASM74247v1
Candidatus.Neoehrlichia.lotoris.str.RAC413 GCA_000964795.1_ASM96479v1
Anaplasma.phagocytophilum.str.HZ GCA_000013125.1_ASM1312v1
Ehrlichia.ruminantium.str.Welgevonden GCA_000026005.1_ASM2600v1
Wolbachia.endosymbiont.of.Drosophila.melanogaster GCA_000008025.1_ASM802v1
Candidatus.Deianiraea.vastatrix GCA_007993655.1_ASM799365v1
Rickettsiales.endosymbiont.of.Trichoplax.sp.H2 GCA_009690945.1
Candidatus.Midichloria.mitochondrii.IricVA GCA_000219355.1_ASM21935v1
Rickettsiales.endosymbiont.of.Peranema.trichophorum GCA_004210275.1_ASM421027v1
Candidatus.Jidaibacter.acanthamoeba GCA_000815465.1.UWC36
Rickettsia.bellii.RML369.C GCA_000012385.1_ASM1238v1
Candidatus.Megaira.polyxenophila
Rickettsiales.endosymbiont.of.Stachyamoeba.lipophora GCA_003932735.1_ASM393273v1
Rickettsiales.bacterium.Ac37b GCA_000746585.2_ASM74658v2
alpha.proteobacterium.HIMB5 GCA_000299095.1_ASM29909v1
Candidatus.Pelagibacter.ubique.HTCC1062 GCA_000012345.1_ASM1234v1
Candidatus.Pelagibacter.ubique.HIMB058 GCA_000419545.1_ASM41954v1
alpha.proteobacterium.HIMB114 GCA_000163555.2
GCA_002722055.1_ASM272205v1*
GCA_002701885.1_ASM270188v1*
Magnetofaba.australis.IT.1 GCA_002109495.1_ASM210949v1
Magnetococcus.marinus.MC.1 GCA_000014865.1_ASM1486v1
Nitrosospira.briensis.C.128 GCA_000619905.2_ASM61990v2
Methylotenera.versatilis.301 GCA_000093025.1_ASM9302v1
Ralstonia.pickettii GCA_902374465.1.MGYG.HGUT.01384
Burkholderia.cepacia GCA_009586235.1_ASM958623v1
Dasania.marina.DSM.21967 GCA_000373485.1_ASM37348v1
Congregibacter.litoralis.KT71 GCA_000153125.2_ASM15312v2
Enterobacter.cloacae GCA_000770155.1_ASM77015v1
Alteromonas.macleodii.ATCC.27126 GCA_000172635.2_ASM17263v2
0.81669

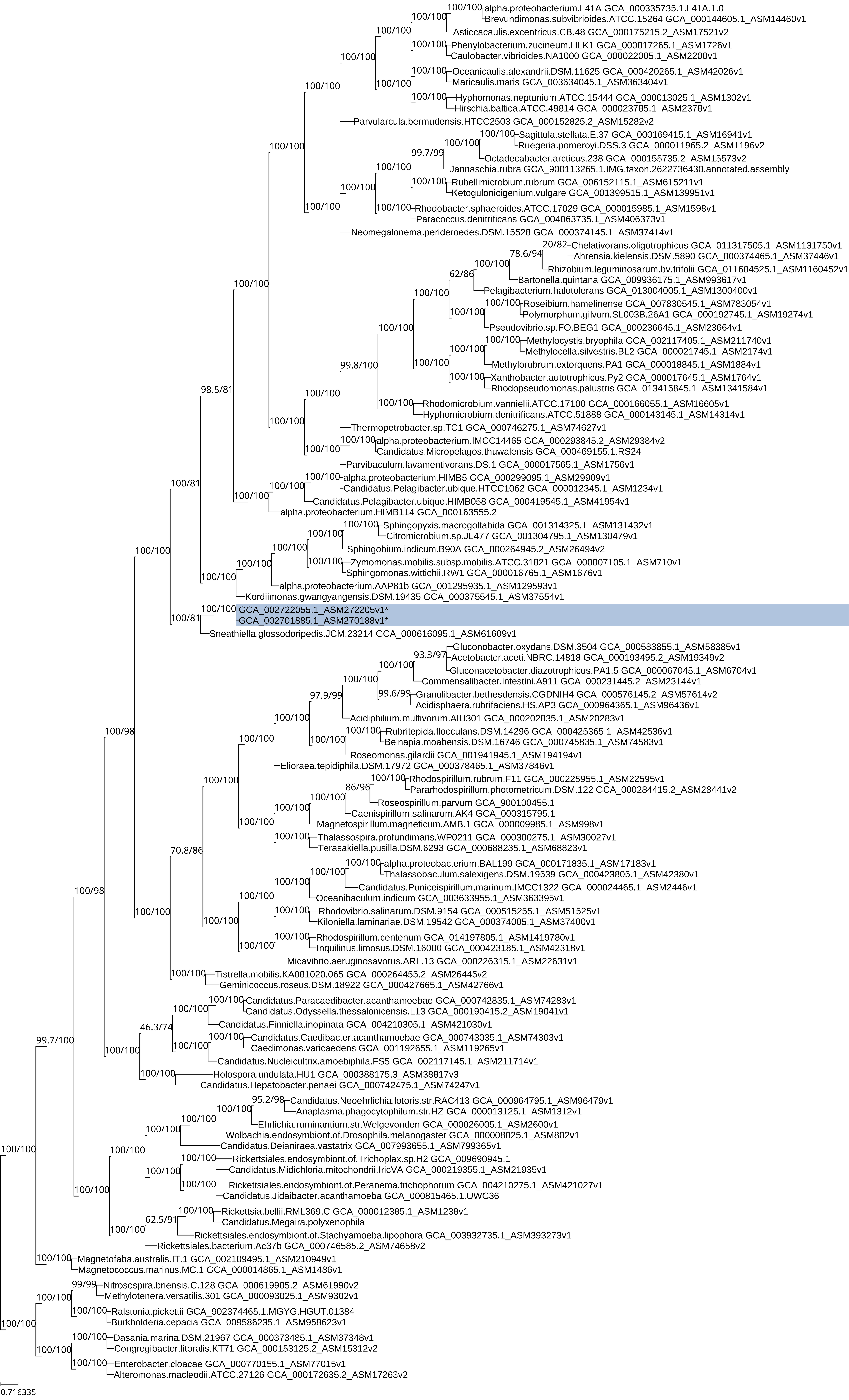
100/100
alpha.proteobacterium.L41A GCA_000335735.1.L41A.1.0
Brevundimonas.subvibrioides.ATCC.15264 GCA_000144605.1_ASM14460v1
100/100
Asticcacaulis.excentricus.CB.48 GCA_000175215.2_ASM17521v2
100/100
100/100
Phenylobacterium.zucineum.HLK1 GCA_000017265.1_ASM1726v1
Caulobacter.vibrioides.NA1000 GCA_000022005.1_ASM2200v1
100/100
100/100
Oceanicaulis.alexandrii.DSM.11625 GCA_000420265.1_ASM42026v1
100/100
Maricaulis.maris GCA_003634045.1_ASM363404v1
100/100
Hyphomonas.neptunium.ATCC.15444 GCA_000013025.1_ASM1302v1
Hirschia.baltica.ATCC.49814 GCA_000023785.1_ASM2378v1
Parvularcula.bermudensis.HTCC2503 GCA_000152825.2_ASM15282v2
100/100
Sagittula.stellata.E.37 GCA_000169415.1_ASM16941v1
100/100
Ruegeria.pomeroyi.DSS.3 GCA_000011965.2_ASM1196v2
100/100
99.7/99
Octadecabacter.arcticus.238 GCA_000155735.2_ASM15573v2
100/100
Jannaschia.rubra GCA_900113265.1.IMG.taxon.2622736430.annotated.assembly
100/100
Rubellimicrobium.rubrum GCA_006152115.1_ASM615211v1
100/100
Ketogulonicigenium.vulgare GCA_001399515.1_ASM139951v1
100/100
100/100
Rhodobacter.sphaeroides.ATCC.17029 GCA_000015985.1_ASM1598v1
Paracoccus.denitrificans GCA_004063735.1_ASM406373v1
Neomegalonema.perideroedes.DSM.15528 GCA_000374145.1_ASM37414v1
20/82
Chelativorans.oligotrophicus GCA_011317505.1_ASM1131750v1
78.6/94
Ahrensia.kielensis.DSM.5890 GCA_000374465.1_ASM37446v1
100/100
Rhizobium.leguminosarum.bv.trifolii GCA_011604525.1_ASM1160452v1
62/86
Bartonella.quintana GCA_009936175.1_ASM993617v1
100/100
Pelagibacterium.halotolerans GCA_013004005.1_ASM1300400v1
100/100
100/100
Roseibium.hamelinense GCA_007830545.1_ASM783054v1
Polymorphum.gilvum.SL003B.26A1 GCA_000192745.1_ASM19274v1
100/100
Pseudovibrio.sp.FO.BEG1 GCA_000236645.1_ASM23664v1
100/100
Methylocystis.bryophila GCA_002117405.1_ASM211740v1
100/100
Methylocella.silvestris.BL2 GCA_000021745.1_ASM2174v1
100/100
Methylorubrum.extorquens.PA1 GCA_000018845.1_ASM1884v1
99.8/100
100/100
Xanthobacter.autotrophicus.Py2 GCA_000017645.1_ASM1764v1
Rhodopseudomonas.palustris GCA_013415845.1_ASM1341584v1
98.5/81
100/100
100/100
Rhodomicrobium.vannielii.ATCC.17100 GCA_000166055.1_ASM16605v1
Hyphomicrobium.denitrificans.ATCC.51888 GCA_000143145.1_ASM14314v1
100/100
Thermopetrobacter.sp.TC1 GCA_000746275.1_ASM74627v1
100/100
alpha.proteobacterium.IMCC14465 GCA_000293845.2_ASM29384v2
100/100
Candidatus.Micropelagos.thuwalensis GCA_000469155.1.RS24
Parvibaculum.lavamentivorans.DS.1 GCA_000017565.1_ASM1756v1
100/100
100/81
alpha.proteobacterium.HIMB5 GCA_000299095.1_ASM29909v1
100/100
Candidatus.Pelagibacter.ubique.HTCC1062 GCA_000012345.1_ASM1234v1
100/100
Candidatus.Pelagibacter.ubique.HIMB058 GCA_000419545.1_ASM41954v1
alpha.proteobacterium.HIMB114 GCA_000163555.2
100/100
Sphingopyxis.macrogoltabida GCA_001314325.1_ASM131432v1
100/100
Citromicrobium.sp.JL477 GCA_001304795.1_ASM130479v1
100/100
100/100
Sphingobium.indicum.B90A GCA_000264945.2_ASM26494v2
100/100
Zymomonas.mobilis.subsp.mobilis.ATCC.31821 GCA_000007105.1_ASM710v1
100/100
Sphingomonas.wittichii.RW1 GCA_000016765.1_ASM1676v1
100/100
alpha.proteobacterium.AAP81b GCA_001295935.1_ASM129593v1
Kordiimonas.gwangyangensis.DSM.19435 GCA_000375545.1_ASM37554v1
100/100
GCA_002722055.1_ASM272205v1*
100/81
GCA_002701885.1_ASM270188v1*
Sneathiella.glossodoripedis.JCM.23214 GCA_000616095.1_ASM61609v1
Gluconobacter.oxydans.DSM.3504 GCA_000583855.1_ASM58385v1
93.3/97
Acetobacter.aceti.NBRC.14818 GCA_000193495.2_ASM19349v2
100/100
Gluconacetobacter.diazotrophicus.PA1.5 GCA_000067045.1_ASM6704v1
100/100
Commensalibacter.intestini.A911 GCA_000231445.2_ASM23144v1
97.9/99
99.6/99
Granulibacter.bethesdensis.CGDNIH4 GCA_000576145.2_ASM57614v2
Acidisphaera.rubrifaciens.HS.AP3 GCA_000964365.1_ASM96436v1
100/98
100/100
Acidiphilium.multivorum.AIU301 GCA_000202835.1_ASM20283v1
100/100
Rubritepida.flocculans.DSM.14296 GCA_000425365.1_ASM42536v1
100/100
100/100
Belnapia.moabensis.DSM.16746 GCA_000745835.1_ASM74583v1
Roseomonas.gilardii GCA_001941945.1_ASM194194v1
Elioraea.tepidiphila.DSM.17972 GCA_000378465.1_ASM37846v1
100/100
100/100
Rhodospirillum.rubrum.F11 GCA_000225955.1_ASM22595v1
86/96
Pararhodospirillum.photometricum.DSM.122 GCA_000284415.2_ASM28441v2
100/100
Roseospirillum.parvum GCA_900100455.1
100/100
Caenispirillum.salinarum.AK4 GCA_000315795.1
100/100
Magnetospirillum.magneticum.AMB.1 GCA_000009985.1_ASM998v1
100/100
Thalassospira.profundimaris.WP0211 GCA_000300275.1_ASM30027v1
Terasakiella.pusilla.DSM.6293 GCA_000688235.1_ASM68823v1
70.8/86
100/100
alpha.proteobacterium.BAL199 GCA_000171835.1_ASM17183v1
100/100
Thalassobaculum.salexigens.DSM.19539 GCA_000423805.1_ASM42380v1
100/100
Candidatus.Puniceispirillum.marinum.IMCC1322 GCA_000024465.1_ASM2446v1
100/98
100/100
Oceanibaculum.indicum GCA_003633955.1_ASM363395v1
100/100
100/100
Rhodovibrio.salinarum.DSM.9154 GCA_000515255.1_ASM51525v1
100/100
Kiloniella.laminariae.DSM.19542 GCA_000374005.1_ASM37400v1
100/100
Rhodospirillum.centenum GCA_014197805.1_ASM1419780v1
100/100
Inquilinus.limosus.DSM.16000 GCA_000423185.1_ASM42318v1
Micavibrio.aeruginosavorus.ARL.13 GCA_000226315.1_ASM22631v1
100/100
Tistrella.mobilis.KA081020.065 GCA_000264455.2_ASM26445v2
Geminicoccus.roseus.DSM.18922 GCA_000427665.1_ASM42766v1
100/100
Candidatus.Paracaedibacter.acanthamoebae GCA_000742835.1_ASM74283v1
100/100
Candidatus.Odyssella.thessalonicensis.L13 GCA_000190415.2_ASM19041v1
46.3/74
Candidatus.Finniella.inopinata GCA_004210305.1_ASM421030v1
99.7/100
100/100
Candidatus.Caedibacter.acanthamoebae GCA_000743035.1_ASM74303v1
100/100
Caedimonas.varicaedens GCA_001192655.1_ASM119265v1
100/100
Candidatus.Nucleicultrix.amoebiphila.FS5 GCA_002117145.1_ASM211714v1
100/100
Holospora.undulata.HU1 GCA_000388175.3_ASM38817v3
Candidatus.Hepatobacter.penaei GCA_000742475.1_ASM74247v1
95.2/98
Candidatus.Neoehrlichia.lotoris.str.RAC413 GCA_000964795.1_ASM96479v1
100/100
Anaplasma.phagocytophilum.str.HZ GCA_000013125.1_ASM1312v1
100/100
Ehrlichia.ruminantium.str.Welgevonden GCA_000026005.1_ASM2600v1
100/100
Wolbachia.endosymbiont.of.Drosophila.melanogaster GCA_000008025.1_ASM802v1
100/100
Candidatus.Deianiraea.vastatrix GCA_007993655.1_ASM799365v1
100/100
100/100
Rickettsiales.endosymbiont.of.Trichoplax.sp.H2 GCA_009690945.1
100/100
Candidatus.Midichloria.mitochondrii.IricVA GCA_000219355.1_ASM21935v1
100/100
100/100
Rickettsiales.endosymbiont.of.Peranema.trichophorum GCA_004210275.1_ASM421027v1
Candidatus.Jidaibacter.acanthamoeba GCA_000815465.1.UWC36
100/100
Rickettsia.bellii.RML369.C GCA_000012385.1_ASM1238v1
62.5/91
Candidatus.Megaira.polyxenophila
100/100
Rickettsiales.endosymbiont.of.Stachyamoeba.lipophora GCA_003932735.1_ASM393273v1
Rickettsiales.bacterium.Ac37b GCA_000746585.2_ASM74658v2
100/100
Magnetofaba.australis.IT.1 GCA_002109495.1_ASM210949v1
Magnetococcus.marinus.MC.1 GCA_000014865.1_ASM1486v1
99/99
Nitrosospira.briensis.C.128 GCA_000619905.2_ASM61990v2
100/100
Methylotenera.versatilis.301 GCA_000093025.1_ASM9302v1
100/100
Ralstonia.pickettii GCA_902374465.1.MGYG.HGUT.01384
100/100
Burkholderia.cepacia GCA_009586235.1_ASM958623v1
100/100
Dasania.marina.DSM.21967 GCA_000373485.1_ASM37348v1
100/100
Congregibacter.litoralis.KT71 GCA_000153125.2_ASM15312v2
100/100
Enterobacter.cloacae GCA_000770155.1_ASM77015v1
Alteromonas.macleodii.ATCC.27126 GCA_000172635.2_ASM17263v2
0.716335

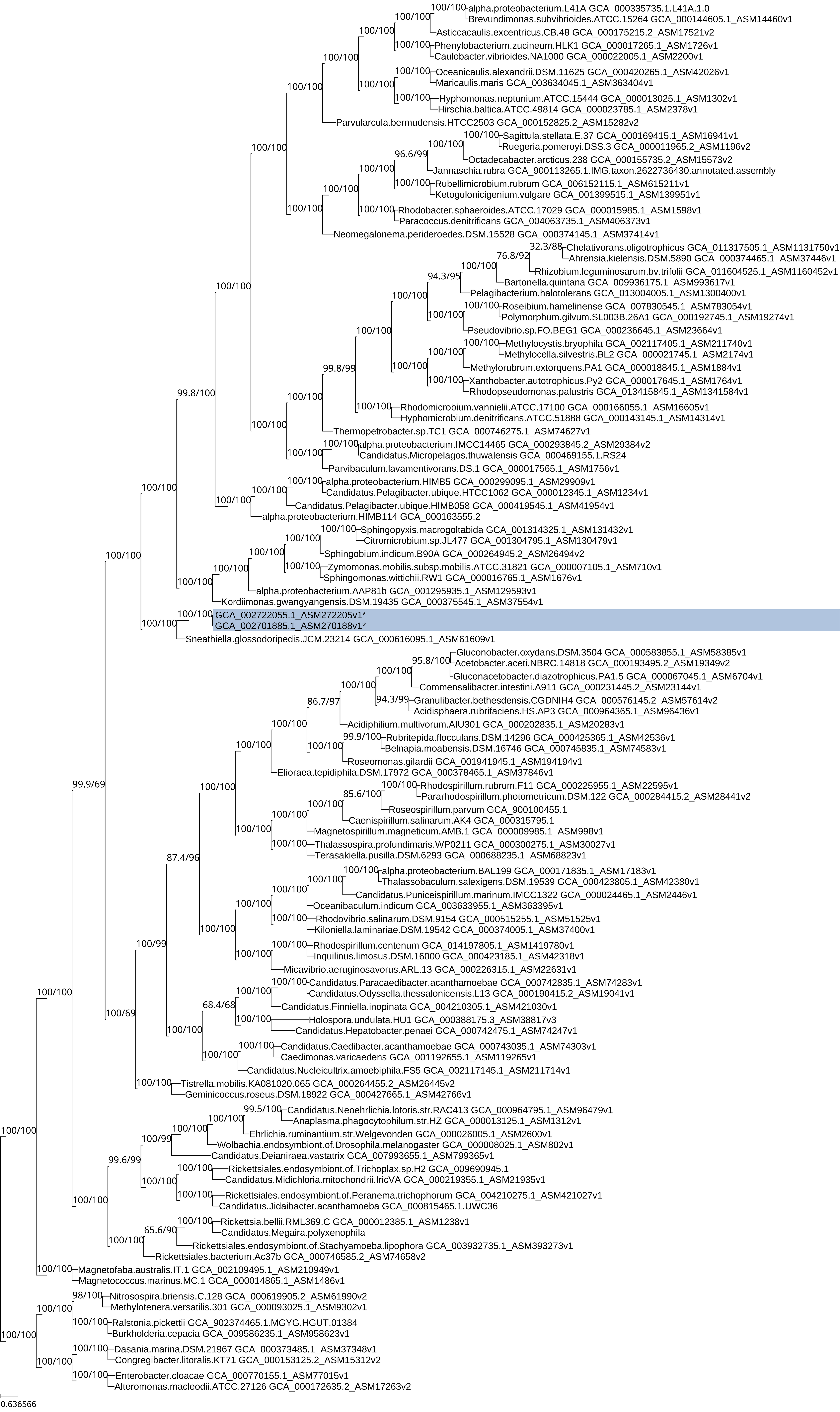
100/100
alpha.proteobacterium.L41A GCA_000335735.1.L41A.1.0
Brevundimonas.subvibrioides.ATCC.15264 GCA_000144605.1_ASM14460v1
100/100
Asticcacaulis.excentricus.CB.48 GCA_000175215.2_ASM17521v2
100/100
Phenylobacterium.zucineum.HLK1 GCA_000017265.1_ASM1726v1
Caulobacter.vibrioides.NA1000 GCA_000022005.1_ASM2200v1
100/100
100/100
Oceanicaulis.alexandrii.DSM.11625 GCA_000420265.1_ASM42026v1
Maricaulis.maris GCA_003634045.1_ASM363404v1
100/100
100/100
Hyphomonas.neptunium.ATCC.15444 GCA_000013025.1_ASM1302v1
Hirschia.baltica.ATCC.49814 GCA_000023785.1_ASM2378v1
100/100
Parvularcula.bermudensis.HTCC2503 GCA_000152825.2_ASM15282v2
100/100
Sagittula.stellata.E.37 GCA_000169415.1_ASM16941v1
Ruegeria.pomeroyi.DSS.3 GCA_000011965.2_ASM1196v2
100/100
96.6/99
Octadecabacter.arcticus.238 GCA_000155735.2_ASM15573v2
100/100
Jannaschia.rubra GCA_900113265.1.IMG.taxon.2622736430.annotated.assembly
100/100
100/100
Rubellimicrobium.rubrum GCA_006152115.1_ASM615211v1
Ketogulonicigenium.vulgare GCA_001399515.1_ASM139951v1
100/100
100/100
Rhodobacter.sphaeroides.ATCC.17029 GCA_000015985.1_ASM1598v1
Paracoccus.denitrificans GCA_004063735.1_ASM406373v1
Neomegalonema.perideroedes.DSM.15528 GCA_000374145.1_ASM37414v1
32.3/88
Chelativorans.oligotrophicus GCA_011317505.1_ASM1131750v1
76.8/92
Ahrensia.kielensis.DSM.5890 GCA_000374465.1_ASM37446v1
100/100
Rhizobium.leguminosarum.bv.trifolii GCA_011604525.1_ASM1160452v1
94.3/95
Bartonella.quintana GCA_009936175.1_ASM993617v1
100/100
Pelagibacterium.halotolerans GCA_013004005.1_ASM1300400v1
100/100
Roseibium.hamelinense GCA_007830545.1_ASM783054v1
100/100
Polymorphum.gilvum.SL003B.26A1 GCA_000192745.1_ASM19274v1
100/100
Pseudovibrio.sp.FO.BEG1 GCA_000236645.1_ASM23664v1
100/100
Methylocystis.bryophila GCA_002117405.1_ASM211740v1
100/100
Methylocella.silvestris.BL2 GCA_000021745.1_ASM2174v1
99.8/99
100/100
Methylorubrum.extorquens.PA1 GCA_000018845.1_ASM1884v1
100/100
Xanthobacter.autotrophicus.Py2 GCA_000017645.1_ASM1764v1
Rhodopseudomonas.palustris GCA_013415845.1_ASM1341584v1
99.8/100
100/100
100/100
Rhodomicrobium.vannielii.ATCC.17100 GCA_000166055.1_ASM16605v1
Hyphomicrobium.denitrificans.ATCC.51888 GCA_000143145.1_ASM14314v1
100/100
Thermopetrobacter.sp.TC1 GCA_000746275.1_ASM74627v1
100/100
alpha.proteobacterium.IMCC14465 GCA_000293845.2_ASM29384v2
100/100
Candidatus.Micropelagos.thuwalensis GCA_000469155.1.RS24
Parvibaculum.lavamentivorans.DS.1 GCA_000017565.1_ASM1756v1
100/100
alpha.proteobacterium.HIMB5 GCA_000299095.1_ASM29909v1
100/100
Candidatus.Pelagibacter.ubique.HTCC1062 GCA_000012345.1_ASM1234v1
100/100
Candidatus.Pelagibacter.ubique.HIMB058 GCA_000419545.1_ASM41954v1
100/100
alpha.proteobacterium.HIMB114 GCA_000163555.2
100/100
Sphingopyxis.macrogoltabida GCA_001314325.1_ASM131432v1
100/100
Citromicrobium.sp.JL477 GCA_001304795.1_ASM130479v1
100/100
100/100
Sphingobium.indicum.B90A GCA_000264945.2_ASM26494v2
100/100
Zymomonas.mobilis.subsp.mobilis.ATCC.31821 GCA_000007105.1_ASM710v1
100/100
Sphingomonas.wittichii.RW1 GCA_000016765.1_ASM1676v1
100/100
alpha.proteobacterium.AAP81b GCA_001295935.1_ASM129593v1
Kordiimonas.gwangyangensis.DSM.19435 GCA_000375545.1_ASM37554v1
100/100
GCA_002722055.1_ASM272205v1*
100/100
GCA_002701885.1_ASM270188v1*
Sneathiella.glossodoripedis.JCM.23214 GCA_000616095.1_ASM61609v1
Gluconobacter.oxydans.DSM.3504 GCA_000583855.1_ASM58385v1
95.8/100
Acetobacter.aceti.NBRC.14818 GCA_000193495.2_ASM19349v2
100/100
Gluconacetobacter.diazotrophicus.PA1.5 GCA_000067045.1_ASM6704v1
100/100
Commensalibacter.intestini.A911 GCA_000231445.2_ASM23144v1
86.7/97
94.3/99
Granulibacter.bethesdensis.CGDNIH4 GCA_000576145.2_ASM57614v2
Acidisphaera.rubrifaciens.HS.AP3 GCA_000964365.1_ASM96436v1
100/100
Acidiphilium.multivorum.AIU301 GCA_000202835.1_ASM20283v1
99.9/100
Rubritepida.flocculans.DSM.14296 GCA_000425365.1_ASM42536v1
100/100
100/100
Belnapia.moabensis.DSM.16746 GCA_000745835.1_ASM74583v1
Roseomonas.gilardii GCA_001941945.1_ASM194194v1
99.9/69
100/100
Elioraea.tepidiphila.DSM.17972 GCA_000378465.1_ASM37846v1
100/100
Rhodospirillum.rubrum.F11 GCA_000225955.1_ASM22595v1
85.6/100
Pararhodospirillum.photometricum.DSM.122 GCA_000284415.2_ASM28441v2
100/100
Roseospirillum.parvum GCA_900100455.1
100/100
Caenispirillum.salinarum.AK4 GCA_000315795.1
100/100
Magnetospirillum.magneticum.AMB.1 GCA_000009985.1_ASM998v1
100/100
Thalassospira.profundimaris.WP0211 GCA_000300275.1_ASM30027v1
87.4/96
Terasakiella.pusilla.DSM.6293 GCA_000688235.1_ASM68823v1
100/100
alpha.proteobacterium.BAL199 GCA_000171835.1_ASM17183v1
100/100
Thalassobaculum.salexigens.DSM.19539 GCA_000423805.1_ASM42380v1
100/100
Candidatus.Puniceispirillum.marinum.IMCC1322 GCA_000024465.1_ASM2446v1
100/100
Oceanibaculum.indicum GCA_003633955.1_ASM363395v1
100/100
Rhodovibrio.salinarum.DSM.9154 GCA_000515255.1_ASM51525v1
100/100
Kiloniella.laminariae.DSM.19542 GCA_000374005.1_ASM37400v1
100/100
Rhodospirillum.centenum GCA_014197805.1_ASM1419780v1
100/99
100/100
Inquilinus.limosus.DSM.16000 GCA_000423185.1_ASM42318v1
Micavibrio.aeruginosavorus.ARL.13 GCA_000226315.1_ASM22631v1
100/100
100/100
Candidatus.Paracaedibacter.acanthamoebae GCA_000742835.1_ASM74283v1
Candidatus.Odyssella.thessalonicensis.L13 GCA_000190415.2_ASM19041v1
100/100
Candidatus.Finniella.inopinata GCA_004210305.1_ASM421030v1
68.4/68
100/100
Holospora.undulata.HU1 GCA_000388175.3_ASM38817v3
100/69
Candidatus.Hepatobacter.penaei GCA_000742475.1_ASM74247v1
100/100
100/100
Candidatus.Caedibacter.acanthamoebae GCA_000743035.1_ASM74303v1
100/100
Caedimonas.varicaedens GCA_001192655.1_ASM119265v1
Candidatus.Nucleicultrix.amoebiphila.FS5 GCA_002117145.1_ASM211714v1
100/100
Tistrella.mobilis.KA081020.065 GCA_000264455.2_ASM26445v2
Geminicoccus.roseus.DSM.18922 GCA_000427665.1_ASM42766v1
99.5/100
Candidatus.Neoehrlichia.lotoris.str.RAC413 GCA_000964795.1_ASM96479v1
100/100
Anaplasma.phagocytophilum.str.HZ GCA_000013125.1_ASM1312v1
100/100
Ehrlichia.ruminantium.str.Welgevonden GCA_000026005.1_ASM2600v1
100/100
100/99
Wolbachia.endosymbiont.of.Drosophila.melanogaster GCA_000008025.1_ASM802v1
99.6/99
Candidatus.Deianiraea.vastatrix GCA_007993655.1_ASM799365v1
100/100
Rickettsiales.endosymbiont.of.Trichoplax.sp.H2 GCA_009690945.1
100/100
Candidatus.Midichloria.mitochondrii.IricVA GCA_000219355.1_ASM21935v1
100/100
Rickettsiales.endosymbiont.of.Peranema.trichophorum GCA_004210275.1_ASM421027v1
100/100
Candidatus.Jidaibacter.acanthamoeba GCA_000815465.1.UWC36
100/100
Rickettsia.bellii.RML369.C GCA_000012385.1_ASM1238v1
65.6/90
Candidatus.Megaira.polyxenophila
100/100
Rickettsiales.endosymbiont.of.Stachyamoeba.lipophora GCA_003932735.1_ASM393273v1
Rickettsiales.bacterium.Ac37b GCA_000746585.2_ASM74658v2
100/100
Magnetofaba.australis.IT.1 GCA_002109495.1_ASM210949v1
Magnetococcus.marinus.MC.1 GCA_000014865.1_ASM1486v1
98/100
Nitrosospira.briensis.C.128 GCA_000619905.2_ASM61990v2
100/100
Methylotenera.versatilis.301 GCA_000093025.1_ASM9302v1
100/100
Ralstonia.pickettii GCA_902374465.1.MGYG.HGUT.01384
Burkholderia.cepacia GCA_009586235.1_ASM958623v1
100/100
100/100
Dasania.marina.DSM.21967 GCA_000373485.1_ASM37348v1
Congregibacter.litoralis.KT71 GCA_000153125.2_ASM15312v2
100/100
100/100
Enterobacter.cloacae GCA_000770155.1_ASM77015v1
Alteromonas.macleodii.ATCC.27126 GCA_000172635.2_ASM17263v2
0.636566

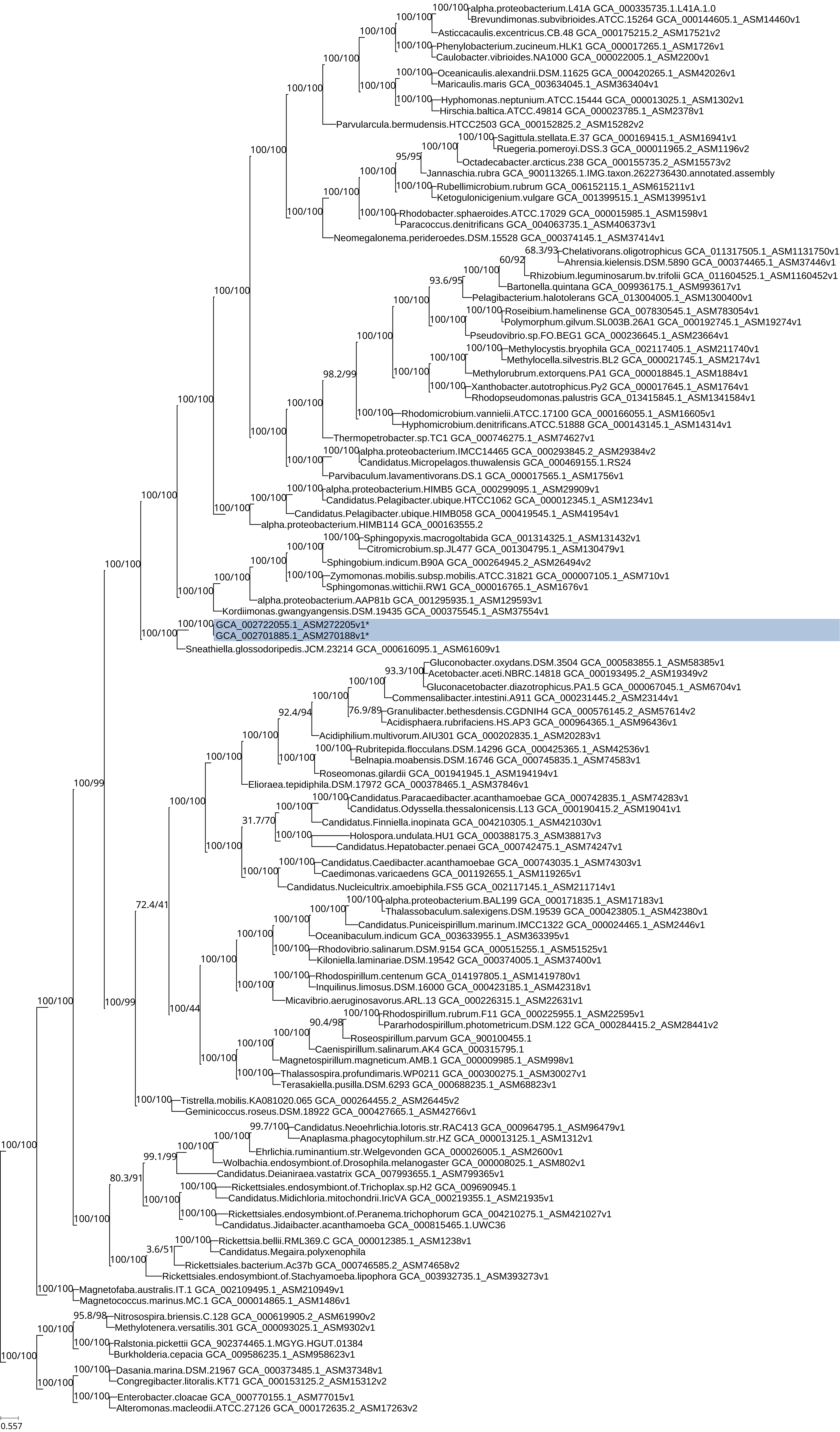

alpha.proteobacterium.L41A GCA_000335735.1.L41A.1.0
Brevundimonas.subvibrioides.ATCC.15264 GCA_000144605.1_ASM14460v1
Asticcacaulis.excentricus.CB.48 GCA_000175215.2_ASM17521v2
Phenylobacterium.zucineum.HLK1 GCA_000017265.1_ASM1726v1
Caulobacter.vibrioides.NA1000 GCA_000022005.1_ASM2200v1
Oceanicaulis.alexandrii.DSM.11625 GCA_000420265.1_ASM42026v1
Maricaulis.maris GCA_003634045.1_ASM363404v1
Hyphomonas.neptunium.ATCC.15444 GCA_000013025.1_ASM1302v1
Hirschia.baltica.ATCC.49814 GCA_000023785.1_ASM2378v1
Parvularcula.bermudensis.HTCC2503 GCA_000152825.2_ASM15282v2
Sagittula.stellata.E.37 GCA_000169415.1_ASM16941v1
Ruegeria.pomeroyi.DSS.3 GCA_000011965.2_ASM1196v2
Octadecabacter.arcticus.238 GCA_000155735.2_ASM15573v2
Jannaschia.rubra GCA_900113265.1.IMG.taxon.2622736430.annotated.assembly
Rubellimicrobium.rubrum GCA_006152115.1_ASM615211v1
Ketogulonicigenium.vulgare GCA_001399515.1_ASM139951v1
Rhodobacter.sphaeroides.ATCC.17029 GCA_000015985.1_ASM1598v1
Paracoccus.denitrificans GCA_004063735.1_ASM406373v1
Neomegalonema.perideroedes.DSM.15528 GCA_000374145.1_ASM37414v1
Chelativorans.oligotrophicus GCA_011317505.1_ASM1131750v1
Ahrensia.kielensis.DSM.5890 GCA_000374465.1_ASM37446v1
Rhizobium.leguminosarum.bv.trifolii GCA_011604525.1_ASM1160452v1
Bartonella.quintana GCA_009936175.1_ASM993617v1
Pelagibacterium.halotolerans GCA_013004005.1_ASM1300400v1
Roseibium.hamelinense GCA_007830545.1_ASM783054v1
Polymorphum.gilvum.SL003B.26A1 GCA_000192745.1_ASM19274v1
Pseudovibrio.sp.FO.BEG1 GCA_000236645.1_ASM23664v1
Methylocystis.bryophila GCA_002117405.1_ASM211740v1
Methylocella.silvestris.BL2 GCA_000021745.1_ASM2174v1
Methylorubrum.extorquens.PA1 GCA_000018845.1_ASM1884v1
Xanthobacter.autotrophicus.Py2 GCA_000017645.1_ASM1764v1
Rhodopseudomonas.palustris GCA_013415845.1_ASM1341584v1
Rhodomicrobium.vannielii.ATCC.17100 GCA_000166055.1_ASM16605v1
Hyphomicrobium.denitrificans.ATCC.51888 GCA_000143145.1_ASM14314v1
Thermopetrobacter.sp.TC1 GCA_000746275.1_ASM74627v1
alpha.proteobacterium.IMCC14465 GCA_000293845.2_ASM29384v2
Candidatus.Micropelagos.thuwalensis GCA_000469155.1.RS24
Parvibaculum.lavamentivorans.DS.1 GCA_000017565.1_ASM1756v1
alpha.proteobacterium.HIMB5 GCA_000299095.1_ASM29909v1
Candidatus.Pelagibacter.ubique.HTCC1062 GCA_000012345.1_ASM1234v1
Candidatus.Pelagibacter.ubique.HIMB058 GCA_000419545.1_ASM41954v1
alpha.proteobacterium.HIMB114 GCA_000163555.2
Sphingopyxis.macrogoltabida GCA_001314325.1_ASM131432v1
Citromicrobium.sp.JL477 GCA_001304795.1_ASM130479v1
Sphingobium.indicum.B90A GCA_000264945.2_ASM26494v2
Zymomonas.mobilis.subsp.mobilis.ATCC.31821 GCA_000007105.1_ASM710v1
Sphingomonas.wittichii.RW1 GCA_000016765.1_ASM1676v1
alpha.proteobacterium.AAP81b GCA_001295935.1_ASM129593v1
Kordiimonas.gwangyangensis.DSM.19435 GCA_000375545.1_ASM37554v1
GCA_002722055.1_ASM272205v1*
GCA_002701885.1_ASM270188v1*
Sneathiella.glossodoripedis.JCM.23214 GCA_000616095.1_ASM61609v1
Gluconobacter.oxydans.DSM.3504 GCA_000583855.1_ASM58385v1
Acetobacter.aceti.NBRC.14818 GCA_000193495.2_ASM19349v2
Gluconacetobacter.diazotrophicus.PA1.5 GCA_000067045.1_ASM6704v1
Commensalibacter.intestini.A911 GCA_000231445.2_ASM23144v1
Granulibacter.bethesdensis.CGDNIH4 GCA_000576145.2_ASM57614v2
Acidisphaera.rubrifaciens.HS.AP3 GCA_000964365.1_ASM96436v1
Acidiphilium.multivorum.AIU301 GCA_000202835.1_ASM20283v1
Rubritepida.flocculans.DSM.14296 GCA_000425365.1_ASM42536v1
Belnapia.moabensis.DSM.16746 GCA_000745835.1_ASM74583v1
Roseomonas.gilardii GCA_001941945.1_ASM194194v1
Elioraea.tepidiphila.DSM.17972 GCA_000378465.1_ASM37846v1
Candidatus.Paracaedibacter.acanthamoebae GCA_000742835.1_ASM74283v1
Candidatus.Odyssella.thessalonicensis.L13 GCA_000190415.2_ASM19041v1
Candidatus.Finniella.inopinata GCA_004210305.1_ASM421030v1
Holospora.undulata.HU1 GCA_000388175.3_ASM38817v3
Candidatus.Hepatobacter.penaei GCA_000742475.1_ASM74247v1
Candidatus.Caedibacter.acanthamoebae GCA_000743035.1_ASM74303v1
Caedimonas.varicaedens GCA_001192655.1_ASM119265v1
Candidatus.Nucleicultrix.amoebiphila.FS5 GCA_002117145.1_ASM211714v1
alpha.proteobacterium.BAL199 GCA_000171835.1_ASM17183v1
Thalassobaculum.salexigens.DSM.19539 GCA_000423805.1_ASM42380v1
Candidatus.Puniceispirillum.marinum.IMCC1322 GCA_000024465.1_ASM2446v1
Oceanibaculum.indicum GCA_003633955.1_ASM363395v1
Rhodovibrio.salinarum.DSM.9154 GCA_000515255.1_ASM51525v1
Kiloniella.laminariae.DSM.19542 GCA_000374005.1_ASM37400v1
Rhodospirillum.centenum GCA_014197805.1_ASM1419780v1
Inquilinus.limosus.DSM.16000 GCA_000423185.1_ASM42318v1
Micavibrio.aeruginosavorus.ARL.13 GCA_000226315.1_ASM22631v1
Rhodospirillum.rubrum.F11 GCA_000225955.1_ASM22595v1
Pararhodospirillum.photometricum.DSM.122 GCA_000284415.2_ASM28441v2
Roseospirillum.parvum GCA_900100455.1
Caenispirillum.salinarum.AK4 GCA_000315795.1
Magnetospirillum.magneticum.AMB.1 GCA_000009985.1_ASM998v1
Thalassospira.profundimaris.WP0211 GCA_000300275.1_ASM30027v1
Terasakiella.pusilla.DSM.6293 GCA_000688235.1_ASM68823v1
Tistrella.mobilis.KA081020.065 GCA_000264455.2_ASM26445v2
Geminicoccus.roseus.DSM.18922 GCA_000427665.1_ASM42766v1
Candidatus.Neoehrlichia.lotoris.str.RAC413 GCA_000964795.1_ASM96479v1
Anaplasma.phagocytophilum.str.HZ GCA_000013125.1_ASM1312v1
Ehrlichia.ruminantium.str.Welgevonden GCA_000026005.1_ASM2600v1
Wolbachia.endosymbiont.of.Drosophila.melanogaster GCA_000008025.1_ASM802v1
Candidatus.Deianiraea.vastatrix GCA_007993655.1_ASM799365v1
Rickettsiales.endosymbiont.of.Trichoplax.sp.H2 GCA_009690945.1
Candidatus.Midichloria.mitochondrii.IricVA GCA_000219355.1_ASM21935v1
Rickettsiales.endosymbiont.of.Peranema.trichophorum GCA_004210275.1_ASM421027v1
Candidatus.Jidaibacter.acanthamoeba GCA_000815465.1.UWC36
Rickettsia.bellii.RML369.C GCA_000012385.1_ASM1238v1
Candidatus.Megaira.polyxenophila
Rickettsiales.bacterium.Ac37b GCA_000746585.2_ASM74658v2
Rickettsiales.endosymbiont.of.Stachyamoeba.lipophora GCA_003932735.1_ASM393273v1
Magnetofaba.australis.IT.1 GCA_002109495.1_ASM210949v1
Magnetococcus.marinus.MC.1 GCA_000014865.1_ASM1486v1
Nitrosospira.briensis.C.128 GCA_000619905.2_ASM61990v2
Methylotenera.versatilis.301 GCA_000093025.1_ASM9302v1
Ralstonia.pickettii GCA_902374465.1.MGYG.HGUT.01384
Burkholderia.cepacia GCA_009586235.1_ASM958623v1
Dasania.marina.DSM.21967 GCA_000373485.1_ASM37348v1
Congregibacter.litoralis.KT71 GCA_000153125.2_ASM15312v2
Enterobacter.cloacae GCA_000770155.1_ASM77015v1
Alteromonas.macleodii.ATCC.27126 GCA_000172635.2_ASM17263v2
0.557

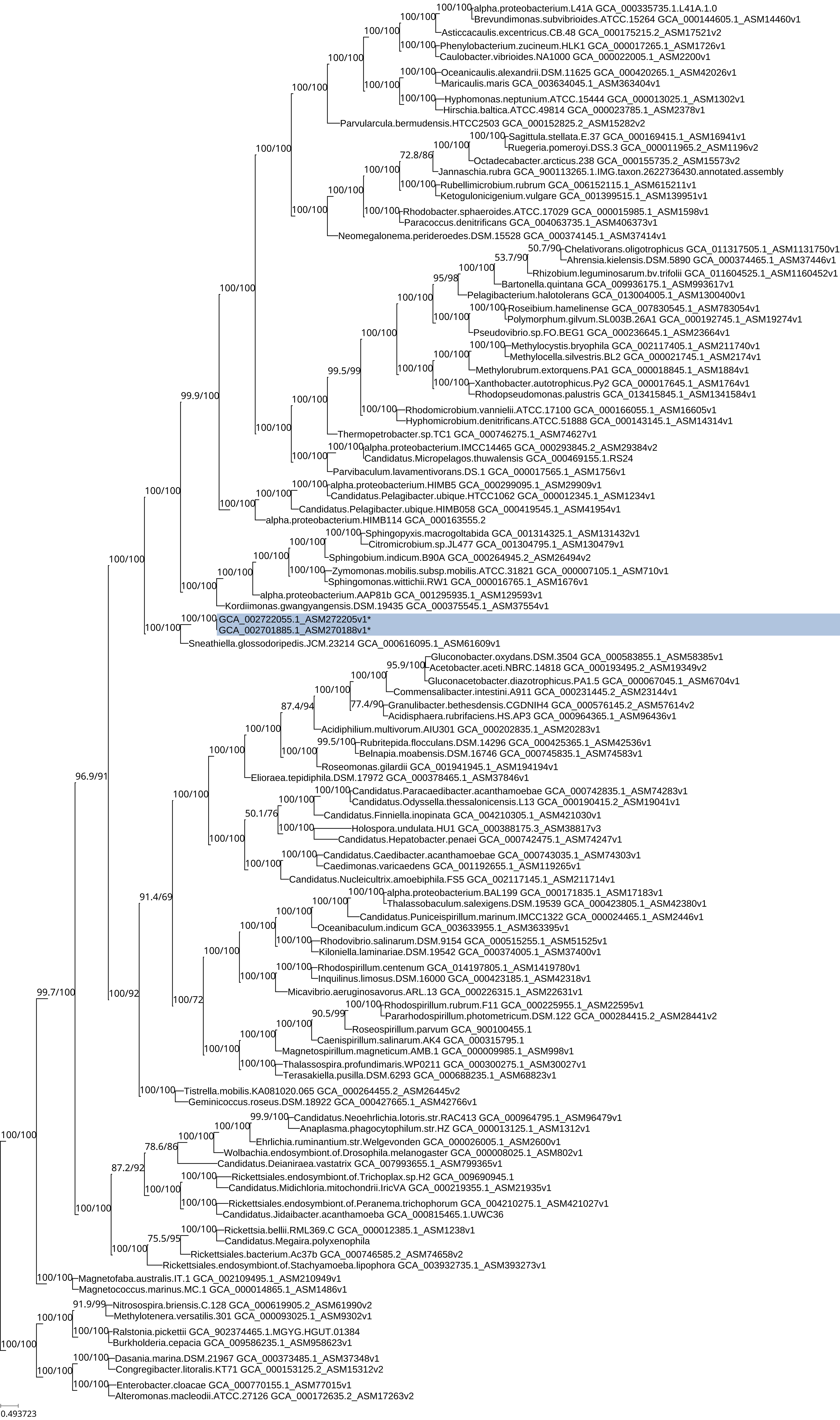

100/100
alpha.proteobacterium.L41A GCA_000335735.1.L41A.1.0
100/100
Brevundimonas.subvibrioides.ATCC.15264 GCA_000144605.1_ASM14460v1
100/100
Asticcacaulis.excentricus.CB.48 GCA_000175215.2_ASM17521v2
100/100
Phenylobacterium.zucineum.HLK1 GCA_000017265.1_ASM1726v1
100/100
Caulobacter.vibrioides.NA1000 GCA_000022005.1_ASM2200v1
100/100
Oceanicaulis.alexandrii.DSM.11625 GCA_000420265.1_ASM42026v1
100/100
Maricaulis.maris GCA_003634045.1_ASM363404v1
100/100
Hyphomonas.neptunium.ATCC.15444 GCA_000013025.1_ASM1302v1
100/100
Hirschia.baltica.ATCC.49814 GCA_000023785.1_ASM2378v1
Parvularcula.bermudensis.HTCC2503 GCA_000152825.2_ASM15282v2
100/100
100/100
Sagittula.stellata.E.37 GCA_000169415.1_ASM16941v1
100/100
Ruegeria.pomeroyi.DSS.3 GCA_000011965.2_ASM1196v2
72.8/86
Octadecabacter.arcticus.238 GCA_000155735.2_ASM15573v2
100/100
Jannaschia.rubra GCA_900113265.1.IMG.taxon.2622736430.annotated.assembly
100/100
Rubellimicrobium.rubrum GCA_006152115.1_ASM615211v1
100/100
Ketogulonicigenium.vulgare GCA_001399515.1_ASM139951v1
100/100
Rhodobacter.sphaeroides.ATCC.17029 GCA_000015985.1_ASM1598v1
100/100
Paracoccus.denitrificans GCA_004063735.1_ASM406373v1
Neomegalonema.perideroedes.DSM.15528 GCA_000374145.1_ASM37414v1
50.7/90
Chelativorans.oligotrophicus GCA_011317505.1_ASM1131750v1
53.7/90
Ahrensia.kielensis.DSM.5890 GCA_000374465.1_ASM37446v1
100/100
Rhizobium.leguminosarum.bv.trifolii GCA_011604525.1_ASM1160452v1
95/98
Bartonella.quintana GCA_009936175.1_ASM993617v1
100/100
Pelagibacterium.halotolerans GCA_013004005.1_ASM1300400v1
100/100
Roseibium.hamelinense GCA_007830545.1_ASM783054v1
100/100
Polymorphum.gilvum.SL003B.26A1 GCA_000192745.1_ASM19274v1
100/100
Pseudovibrio.sp.FO.BEG1 GCA_000236645.1_ASM23664v1
100/100
Methylocystis.bryophila GCA_002117405.1_ASM211740v1
100/100
Methylocella.silvestris.BL2 GCA_000021745.1_ASM2174v1
100/100
Methylorubrum.extorquens.PA1 GCA_000018845.1_ASM1884v1
99.5/99
Xanthobacter.autotrophicus.Py2 GCA_000017645.1_ASM1764v1
100/100
Rhodopseudomonas.palustris GCA_013415845.1_ASM1341584v1
99.9/100
100/100
Rhodomicrobium.vannielii.ATCC.17100 GCA_000166055.1_ASM16605v1
100/100
Hyphomicrobium.denitrificans.ATCC.51888 GCA_000143145.1_ASM14314v1
Thermopetrobacter.sp.TC1 GCA_000746275.1_ASM74627v1
100/100
alpha.proteobacterium.IMCC14465 GCA_000293845.2_ASM29384v2
100/100
Candidatus.Micropelagos.thuwalensis GCA_000469155.1.RS24
Parvibaculum.lavamentivorans.DS.1 GCA_000017565.1_ASM1756v1
100/100
100/100
alpha.proteobacterium.HIMB5 GCA_000299095.1_ASM29909v1
100/100
Candidatus.Pelagibacter.ubique.HTCC1062 GCA_000012345.1_ASM1234v1
100/100
Candidatus.Pelagibacter.ubique.HIMB058 GCA_000419545.1_ASM41954v1
alpha.proteobacterium.HIMB114 GCA_000163555.2
100/100
100/100
Sphingopyxis.macrogoltabida GCA_001314325.1_ASM131432v1
100/100
Citromicrobium.sp.JL477 GCA_001304795.1_ASM130479v1
100/100
Sphingobium.indicum.B90A GCA_000264945.2_ASM26494v2
100/100
Zymomonas.mobilis.subsp.mobilis.ATCC.31821 GCA_000007105.1_ASM710v1
100/100
Sphingomonas.wittichii.RW1 GCA_000016765.1_ASM1676v1
100/100
alpha.proteobacterium.AAP81b GCA_001295935.1_ASM129593v1
Kordiimonas.gwangyangensis.DSM.19435 GCA_000375545.1_ASM37554v1
100/100
GCA_002722055.1_ASM272205v1*
100/100
GCA_002701885.1_ASM270188v1*
Sneathiella.glossodoripedis.JCM.23214 GCA_000616095.1_ASM61609v1
95.9/100
Gluconobacter.oxydans.DSM.3504 GCA_000583855.1_ASM58385v1
Acetobacter.aceti.NBRC.14818 GCA_000193495.2_ASM19349v2
100/100
Gluconacetobacter.diazotrophicus.PA1.5 GCA_000067045.1_ASM6704v1
100/100
Commensalibacter.intestini.A911 GCA_000231445.2_ASM23144v1
87.4/94
77.4/90
Granulibacter.bethesdensis.CGDNIH4 GCA_000576145.2_ASM57614v2
Acidisphaera.rubrifaciens.HS.AP3 GCA_000964365.1_ASM96436v1
100/100
Acidiphilium.multivorum.AIU301 GCA_000202835.1_ASM20283v1
99.5/100
Rubritepida.flocculans.DSM.14296 GCA_000425365.1_ASM42536v1
100/100
100/100
Belnapia.moabensis.DSM.16746 GCA_000745835.1_ASM74583v1
Roseomonas.gilardii GCA_001941945.1_ASM194194v1
96.9/91
Elioraea.tepidiphila.DSM.17972 GCA_000378465.1_ASM37846v1
100/100
100/100
Candidatus.Paracaedibacter.acanthamoebae GCA_000742835.1_ASM74283v1
Candidatus.Odyssella.thessalonicensis.L13 GCA_000190415.2_ASM19041v1
100/100
Candidatus.Finniella.inopinata GCA_004210305.1_ASM421030v1
50.1/76
100/100
Holospora.undulata.HU1 GCA_000388175.3_ASM38817v3
100/100
Candidatus.Hepatobacter.penaei GCA_000742475.1_ASM74247v1
100/100
Candidatus.Caedibacter.acanthamoebae GCA_000743035.1_ASM74303v1
100/100
Caedimonas.varicaedens GCA_001192655.1_ASM119265v1
Candidatus.Nucleicultrix.amoebiphila.FS5 GCA_002117145.1_ASM211714v1
100/100
alpha.proteobacterium.BAL199 GCA_000171835.1_ASM17183v1
91.4/69
100/100
Thalassobaculum.salexigens.DSM.19539 GCA_000423805.1_ASM42380v1
100/100
Candidatus.Puniceispirillum.marinum.IMCC1322 GCA_000024465.1_ASM2446v1
100/100
Oceanibaculum.indicum GCA_003633955.1_ASM363395v1
100/100
Rhodovibrio.salinarum.DSM.9154 GCA_000515255.1_ASM51525v1
100/100
Kiloniella.laminariae.DSM.19542 GCA_000374005.1_ASM37400v1
100/100
Rhodospirillum.centenum GCA_014197805.1_ASM1419780v1
100/100
Inquilinus.limosus.DSM.16000 GCA_000423185.1_ASM42318v1
99.7/100
100/92
Micavibrio.aeruginosavorus.ARL.13 GCA_000226315.1_ASM22631v1
100/72
100/100
Rhodospirillum.rubrum.F11 GCA_000225955.1_ASM22595v1
90.5/99
Pararhodospirillum.photometricum.DSM.122 GCA_000284415.2_ASM28441v2
100/100
Roseospirillum.parvum GCA_900100455.1
100/100
Caenispirillum.salinarum.AK4 GCA_000315795.1
100/100
Magnetospirillum.magneticum.AMB.1 GCA_000009985.1_ASM998v1
100/100
Thalassospira.profundimaris.WP0211 GCA_000300275.1_ASM30027v1
Terasakiella.pusilla.DSM.6293 GCA_000688235.1_ASM68823v1
100/100
Tistrella.mobilis.KA081020.065 GCA_000264455.2_ASM26445v2
Geminicoccus.roseus.DSM.18922 GCA_000427665.1_ASM42766v1
99.9/100
Candidatus.Neoehrlichia.lotoris.str.RAC413 GCA_000964795.1_ASM96479v1
100/100
Anaplasma.phagocytophilum.str.HZ GCA_000013125.1_ASM1312v1
100/100
Ehrlichia.ruminantium.str.Welgevonden GCA_000026005.1_ASM2600v1
100/100
78.6/86
Wolbachia.endosymbiont.of.Drosophila.melanogaster GCA_000008025.1_ASM802v1
Candidatus.Deianiraea.vastatrix GCA_007993655.1_ASM799365v1
87.2/92
100/100
Rickettsiales.endosymbiont.of.Trichoplax.sp.H2 GCA_009690945.1
100/100
Candidatus.Midichloria.mitochondrii.IricVA GCA_000219355.1_ASM21935v1
100/100
Rickettsiales.endosymbiont.of.Peranema.trichophorum GCA_004210275.1_ASM421027v1
100/100
Candidatus.Jidaibacter.acanthamoeba GCA_000815465.1.UWC36
100/100
Rickettsia.bellii.RML369.C GCA_000012385.1_ASM1238v1
75.5/95
Candidatus.Megaira.polyxenophila
100/100
Rickettsiales.bacterium.Ac37b GCA_000746585.2_ASM74658v2
Rickettsiales.endosymbiont.of.Stachyamoeba.lipophora GCA_003932735.1_ASM393273v1
100/100
Magnetofaba.australis.IT.1 GCA_002109495.1_ASM210949v1
Magnetococcus.marinus.MC.1 GCA_000014865.1_ASM1486v1
91.9/99
Nitrosospira.briensis.C.128 GCA_000619905.2_ASM61990v2
100/100
Methylotenera.versatilis.301 GCA_000093025.1_ASM9302v1
100/100
Ralstonia.pickettii GCA_902374465.1.MGYG.HGUT.01384
Burkholderia.cepacia GCA_009586235.1_ASM958623v1
100/100
100/100
Dasania.marina.DSM.21967 GCA_000373485.1_ASM37348v1
100/100
Congregibacter.litoralis.KT71 GCA_000153125.2_ASM15312v2
100/100
Enterobacter.cloacae GCA_000770155.1_ASM77015v1
Alteromonas.macleodii.ATCC.27126 GCA_000172635.2_ASM17263v2
0.493723

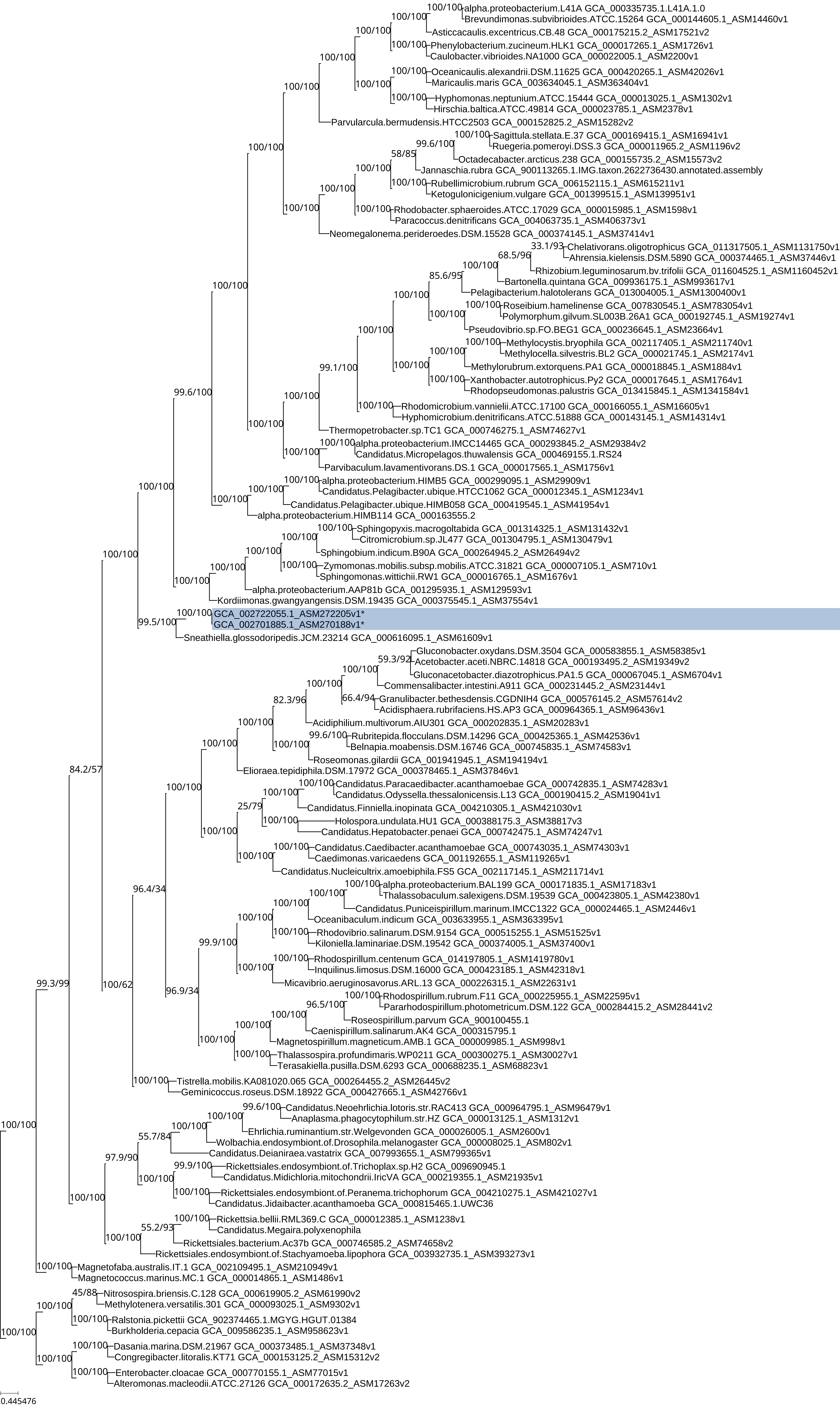

100/100 alpha.proteobacterium.L41A GCA_000335735.1_L41A.1.0
Brevundimonas.subvibrioides.ATCC.15264 GCA_000144605.1_ASM14460v1
100/100
Asticcacaulis.excentricus.CB.48 GCA_000175215.2_ASM17521v2
100/100
100/100 Phenylobacterium.zucineum.HLK1 GCA_000017265.1_ASM1726v1
Caulobacter.vibrioides.NA1000 GCA_000022005.1_ASM2200v1
100/100 Oceanicaulis.alexandrii.DSM.11625 GCA_000420265.1_ASM42026v1
100/100
Maricaulis.maris GCA_003634045.1_ASM363404v1
100/100 Hyphomonas.neptunium.ATCC.15444 GCA_000013025.1_ASM1302v1
Hirschia.baltica.ATCC.49814 GCA_000023785.1_ASM2378v1
Parvularcula.bermudensis.HTCC2503 GCA_000152825.2_ASM15282v2
100/100 Sagittula.stellata.E.37 GCA_000169415.1_ASM16941v1
99.6/100
Ruegeria.pomeroyi.DSS.3 GCA_000011965.2_ASM1196v2
100/100
58/85
Octadecabacter.arcticus.238 GCA_000155735.2_ASM15573v2
100/100
Jannaschia.rubra GCA_900113265.1.IMG.taxon.2622736430.annotated.assembly
100/100 Rubellimicrobium.rubrum GCA_006152115.1_ASM615211v1
Ketogulonicigenium.vulgare GCA_001399515.1_ASM139951v1
100/100 Rhodobacter.sphaeroides.ATCC.17029 GCA_000015985.1_ASM1598v1
100/100
Paracoccus.denitrificans GCA_004063735.1_ASM406373v1
Neomegalonema.perideroedes.DSM.15528 GCA_000374145.1_ASM37414v1
33.1/93 Chelativorans.oligotrophicus GCA_011317505.1_ASM1131750v1
68.5/96
Ahrensia.kielensis.DSM.5890 GCA_000374465.1_ASM37446v1
100/100
Rhizobium.leguminosarum.bv.trifolii GCA_011604525.1_ASM1160452v1
85.6/95
Bartonella.quintana GCA_009936175.1_ASM993617v1
100/100
Pelagibacterium.halotolerans GCA_013004005.1_ASM1300400v1
100/100
100/100 Roseibium.hamelinense GCA_007830545.1_ASM783054v1
100/100
Polymorphum.gilvum.SL003B.26A1 GCA_000192745.1_ASM19274v1
Pseudovibrio.sp.FO.BEG1 GCA_000236645.1_ASM23664v1
100/100 Methylocystis.bryophila GCA_002117405.1_ASM211740v1
100/100
Methylocella.silvestris.BL2 GCA_000021745.1_ASM2174v1
99.1/100
100/100
Methylorubrum.extorquens.PA1 GCA_000018845.1_ASM1884v1
100/100 Xanthobacter.autotrophicus.Py2 GCA_000017645.1_ASM1764v1
99.6/100
Rhodopseudomonas.palustris GCA_013415845.1_ASM1341584v1
100/100
100/100 Rhodomicrobium.vannielii.ATCC.17100 GCA_000166055.1_ASM16605v1
Hyphomicrobium.denitrificans.ATCC.51888 GCA_000143145.1_ASM14314v1
100/100
Thermopetrobacter.sp.TC1 GCA_000746275.1_ASM74627v1
100/100 alpha.proteobacterium.IMCC14465 GCA_000293845.2_ASM29384v2
100/100
Candidatus.Micropelagos.thuwalensis GCA_000469155.1.RS24
Parvibaculum.lavamentivorans.DS.1 GCA_000017565.1_ASM1756v1
100/100 alpha.proteobacterium.HIMB5 GCA_000299095.1_ASM29909v1
100/100
100/100
Candidatus.Pelagibacter.ubique.HTCC1062 GCA_000012345.1_ASM1234v1
100/100
Candidatus.Pelagibacter.ubique.HIMB058 GCA_000419545.1_ASM41954v1
alpha.proteobacterium.HIMB114 GCA_000163555.2
100/100 Sphingopyxis.macrogoltabida GCA_001314325.1_ASM131432v1
100/100
Citromicrobium.sp.JL477 GCA_001304795.1_ASM130479v1
100/100
100/100
Sphingobium.indicum.B90A GCA_000264945.2_ASM26494v2
100/100
100/100 Zymomonas.mobilis.subsp.mobilis.ATCC.31821 GCA_000007105.1_ASM710v1
Sphingomonas.wittichii.RW1 GCA_000016765.1_ASM1676v1
100/100
alpha.proteobacterium.AAP81b GCA_001295935.1_ASM129593v1
Kordiimonas.gwangyangensis.DSM.19435 GCA_000375545.1_ASM37554v1
100/100 GCA_002722055.1_ASM272205v1*
99.5/100
GCA_002701885.1_ASM270188v1*
Sneathiella.glossodoripedis.JCM.23214 GCA_000616095.1_ASM61609v1
Gluconobacter.oxydans.DSM.3504 GCA_000583855.1_ASM58385v1
59.3/92
Acetobacter.aceti.NBRC.14818 GCA_000193495.2_ASM19349v2
100/100
Gluconacetobacter.diazotrophicus.PA1.5 GCA_000067045.1_ASM6704v1
100/100
Commensalibacter.intestini.A911 GCA_000231445.2_ASM23144v1
82.3/96
66.4/94 Granulibacter.bethesdensis.CGDNIH4 GCA_000576145.2_ASM57614v2
Acidisphaera.rubrifaciens.HS.AP3 GCA_000964365.1_ASM96436v1
100/100
Acidiphilium.multivorum.AIU301 GCA_000202835.1_ASM20283v1
100/100
99.6/100 Rubritepida.flocculans.DSM.14296 GCA_000425365.1_ASM42536v1
100/100
Belnapia.moabensis.DSM.16746 GCA_000745835.1_ASM74583v1
84.2/57
Roseomonas.gilardii GCA_001941945.1_ASM194194v1
Elioraea.tepidiphila.DSM.17972 GCA_000378465.1_ASM37846v1
100/100 Candidatus.Paracaedibacter.acanthamoebae GCA_000742835.1_ASM74283v1
100/100
100/100
Candidatus.Odyssella.thessalonicensis.L13 GCA_000190415.2_ASM19041v1
25/79
Candidatus.Finniella.inopinata GCA_004210305.1_ASM421030v1
100/100 Holospora.undulata.HU1 GCA_000388175.3_ASM38817v3
100/100
Candidatus.Hepatobacter.penaei GCA_000742475.1_ASM74247v1
100/100 Candidatus.Caedibacter.acanthamoebae GCA_000743035.1_ASM74303v1
100/100
Caedimonas.varicaedens GCA_001192655.1_ASM119265v1
Candidatus.Nucleicultrix.amoebiphila.FS5 GCA_002117145.1_ASM211714v1
100/100 alpha.proteobacterium.BAL199 GCA_000171835.1_ASM17183v1
96.4/34
100/100
Thalassobaculum.salexigens.DSM.19539 GCA_000423805.1_ASM42380v1
100/100
Candidatus.Puniceispirillum.marinum.IMCC1322 GCA_000024465.1_ASM2446v1
100/100
Oceanibaculum.indicum GCA_003633955.1_ASM363395v1
100/100 Rhodovibrio.salinarum.DSM.9154 GCA_000515255.1_ASM51525v1
99.9/100
Kiloniella.laminariae.DSM.19542 GCA_000374005.1_ASM37400v1
100/100 Rhodospirillum.centenum GCA_014197805.1_ASM1419780v1
100/100
Inquilinus.limosus.DSM.16000 GCA_000423185.1_ASM42318v1
99.3/99
100/62
Micavibrio.aeruginosavorus.ARL.13 GCA_000226315.1_ASM22631v1
96.9/34
100/100 Rhodospirillum.rubrum.F11 GCA_000225955.1_ASM22595v1
96.5/100
Pararhodospirillum.photometricum.DSM.122 GCA_000284415.2_ASM28441v2
100/100
Roseospirillum.parvum GCA_900100455.1
100/100
Caenispirillum.salinarum.AK4 GCA_000315795.1
100/100
Magnetospirillum.magneticum.AMB.1 GCA_000009985.1_ASM998v1
100/100 Thalassospira.profundimaris.WP0211 GCA_000300275.1_ASM30027v1
Terasakiella.pusilla.DSM.6293 GCA_000688235.1_ASM68823v1
100/100 Tistrella.mobilis.KA081020.065 GCA_000264455.2_ASM26445v2
Geminicoccus.roseus.DSM.18922 GCA_000427665.1_ASM42766v1
99.6/100 Candidatus.Neoehrlichia.lotoris.str.RAC413 GCA_000964795.1_ASM96479v1
100/100
Anaplasma.phagocytophilum.str.HZ GCA_000013125.1_ASM1312v1
100/100
100/100
Ehrlichia.ruminantium.str.Welgevonden GCA_000026005.1_ASM2600v1
55.7/84
Wolbachia.endosymbiont.of.Drosophila.melanogaster GCA_000008025.1_ASM802v1
Candidatus.Deianiraea.vastatrix GCA_007993655.1_ASM799365v1
97.9/90
99.9/100 Rickettsiales.endosymbiont.of.Trichoplax.sp.H2 GCA_009690945.1
100/100
Candidatus.Midichloria.mitochondrii.IricVA GCA_000219355.1_ASM21935v1
100/100 Rickettsiales.endosymbiont.of.Peranema.trichophorum GCA_004210275.1_ASM421027v1
100/100
Candidatus.Jidaibacter.acanthamoeba GCA_000815465.1.UWC36
100/100 Rickettsia.bellii.RML369.C GCA_000012385.1_ASM1238v1
55.2/93
Candidatus.Megaira.polyxenophila
100/100
Rickettsiales.bacterium.Ac37b GCA_000746585.2_ASM74658v2
Rickettsiales.endosymbiont.of.Stachyamoeba.lipophora GCA_003932735.1_ASM393273v1
100/100 Magnetofaba.australis.IT.1 GCA_002109495.1_ASM210949v1
Magnetococcus.marinus.MC.1 GCA_000014865.1_ASM1486v1
45/88 Nitrosospira.briensis.C.128 GCA_000619905.2_ASM61990v2
100/100
Methylotenera.versatilis.301 GCA_000093025.1_ASM9302v1
100/100 Ralstonia.pickettii GCA_902374465.1.MGYG.HGUT.01384
100/100
Burkholderia.cepacia GCA_009586235.1_ASM958623v1
100/100 Dasania.marina.DSM.21967 GCA_000373485.1_ASM37348v1
100/100
Congregibacter.litoralis.KT71 GCA_000153125.2_ASM15312v2
100/100 Enterobacter.cloacae GCA_000770155.1_ASM77015v1
Alteromonas.macleodii.ATCC.27126 GCA_000172635.2_ASM17263v2
0.445476

Supplementary Figure 32: “step 6” phylogenomic trees. Phylogenomic trees of *Rickettsiales* and other *Alphaproteobacteria*, for testing the affiliation to *Rickettsiales* of 95 BUSCO-filtered “new putative *Rickettsiales* MAGs” (Step 6 in Supplementary Note 4). The phylogenies on the untrimmed concatenated gene alignment, as well as on alignments with the 10%, 20%, 30%, 40%, or 50% most compositionally heterogeneous sites removed are reported in order. In each tree, the “new putative *Rickettsiales* MAGs” are highlighted in blue. On each branch, support values by SH-aLRT with 1000 replicates and by 1000 ultrafast bootstraps are reported.

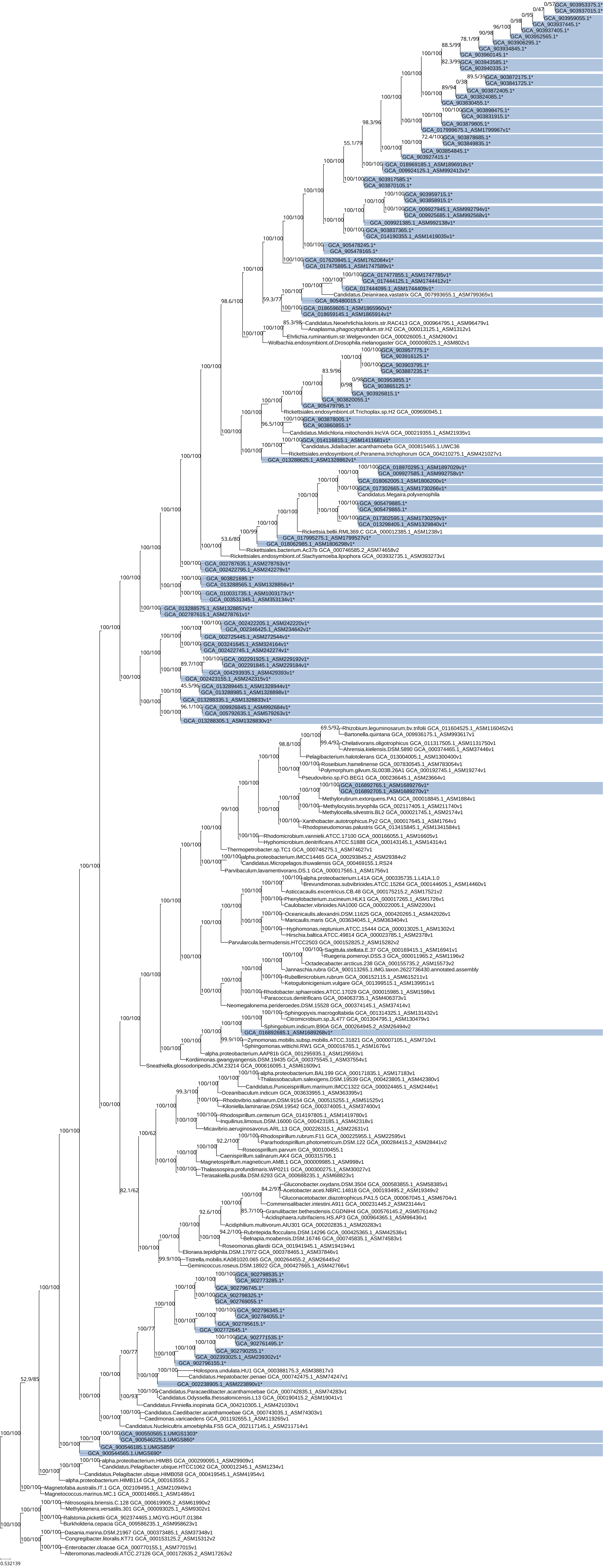

GCA_903953375.1*
GCA_903937015.1*
GCA_903959055.1*
GCA_903937445.1*
GCA_903937405.1*
GCA_903952565.1*
GCA_903906295.1*
GCA_903934845.1*
GCA_903960145.1*
GCA_903943585.1*
GCA_903940335.1*
GCA_903872175.1*
GCA_903841725.1*
GCA_903872405.1*
GCA_903824085.1*
GCA_903830455.1*
GCA_903898475.1*
GCA_903831915.1*
GCA_903879805.1*
GCA_017999675.1_ASM1799967v1*
GCA_903878685.1*
GCA_903849835.1*
GCA_903854845.1*
GCA_903927415.1*
GCA_018969185.1_ASM1896918v1*
GCA_009924125.1_ASM992412v1*
GCA_903917585.1*
GCA_903870105.1*
GCA_903959715.1*
GCA_903858915.1*
GCA_009927945.1_ASM992794v1*
GCA_009925685.1_ASM992568v1*
GCA_009921385.1_ASM992138v1*
GCA_903837365.1*
GCA_014190355.1_ASM1419035v1*
GCA_905478245.1*
GCA_905478165.1*
GCA_017620845.1_ASM1762084v1*
GCA_017475895.1_ASM1747589v1*
GCA_017477855.1_ASM1747785v1*
GCA_017444125.1_ASM1744412v1*
GCA_017444095.1_ASM1744409v1*
Candidatus.Deianiraea.vastatrix GCA_007993655.1_ASM799365v1
GCA_905480015.1*
GCA_018659605.1_ASM1865960v1*
GCA_018659145.1_ASM1865914v1*
Candidatus.Neoehrlichia.lotoris.str.RAC413 GCA_000964795.1_ASM96479v1
Anaplasma.phagocytophilum.str.HZ GCA_000013125.1_ASM1312v1
Ehrlichia.ruminantium.str.Welgevonden GCA_000026005.1_ASM2600v1
Wolbachia.endosymbiont.of.Drosophila.melanogaster GCA_000008025.1_ASM802v1
GCA_903957775.1*
GCA_903916125.1*
GCA_903903795.1*
GCA_903887235.1*
GCA_903953855.1*
GCA_903865125.1*
GCA_903926815.1*
GCA_903820055.1*
GCA_905479795.1*
Rickettsiales.endosymbiont.of.Trichoplax.sp.H2 GCA_009690945.1
GCA_903878005.1*
GCA_903860855.1*
Candidatus.Midichloria.mitochondrii.IricVA GCA_000219355.1_ASM21935v1
GCA_014116815.1_ASM1411681v1*
Candidatus.Jidaibacter.acanthamoeba GCA_000815465.1.UWC36
Rickettsiales.endosymbiont.of.Peranema.trichophorum GCA_004210275.1_ASM421027v1
GCA_013288625.1_ASM1328862v1*
GCA_018970295.1_ASM1897029v1*
GCA_009927585.1_ASM992758v1*
GCA_018062005.1_ASM1806200v1*
GCA_017302665.1_ASM1730266v1*
Candidatus.Megaira.polyxenophila
GCA_905479885.1*
GCA_905479865.1*
GCA_017302595.1_ASM1730259v1*
GCA_013298405.1_ASM1329840v1*
Rickettsia.bellii.RML369.C GCA_000012385.1_ASM1238v1
GCA_017995275.1_ASM1799527v1*
GCA_018062985.1_ASM1806298v1*
Rickettsiales.bacterium.Ac37b GCA_000746585.2_ASM74658v2
Rickettsiales.endosymbiont.of.Stachyamoeba.lipophora GCA_003932735.1_ASM393273v1
GCA_002787635.1_ASM278763v1*
GCA_002422795.1_ASM242279v1*
GCA_903821695.1*
GCA_013288565.1_ASM1328856v1*
GCA_010031735.1_ASM1003173v1*
GCA_003531345.1_ASM353134v1*
GCA_013288575.1_ASM1328857v1*
GCA_002787615.1_ASM278761v1*
GCA_002422205.1_ASM242220v1*
GCA_002346425.1_ASM234642v1*
GCA_002725445.1_ASM272544v1*
GCA_003241645.1_ASM324164v1*
GCA_002422745.1_ASM242274v1*
GCA_002291925.1_ASM229192v1*
GCA_002291845.1_ASM229184v1*
GCA_004293935.1_ASM429393v1*
GCA_002423155.1_ASM242315v1*
GCA_013289445.1_ASM1328944v1*
GCA_013288985.1_ASM1328898v1*
GCA_013288335.1_ASM1328833v1*
GCA_009926845.1_ASM992684v1*
GCA_005792635.1_ASM579263v1*
GCA_013288305.1_ASM1328830v1*
Rhizobium.leguminosarum.bv.trifolii GCA_011604525.1_ASM1160452v1
Bartonella.quintana GCA_009936175.1_ASM993617v1
Chelativorans.oligotrophicus GCA_011317505.1_ASM1131750v1
Ahrensia.kielensis.DSM.5890 GCA_000374465.1_ASM37446v1
Pelagibacterium.halotolerans GCA_013004005.1_ASM1300400v1
Roseibium.hamelinense GCA_007830545.1_ASM783054v1
Polymorphum.gilvum.SL003B.26A1 GCA_000192745.1_ASM19274v1
Pseudovibrio.sp.FO.BEG1 GCA_000236645.1_ASM23664v1
GCA_016892765.1_ASM1689276v1*
GCA_016892705.1_ASM1689270v1*
Methylorubrum.extorquens.PA1 GCA_000018845.1_ASM1884v1
Methylocystis.bryophila GCA_002117405.1_ASM211740v1
Methylocella.silvestris.BL2 GCA_000021745.1_ASM2174v1
Xanthobacter.autotrophicus.Py2 GCA_000017645.1_ASM1764v1
Rhodopseudomonas.palustris GCA_013415845.1_ASM1341584v1
Rhodomicrobium.vannielii.ATCC.17100 GCA_000166055.1_ASM16605v1
Hyphomicrobium.denitrificans.ATCC.51888 GCA_000143145.1_ASM14314v1
Thermopetrobacter.sp.TC1 GCA_000746275.1_ASM74627v1
alpha.proteobacterium.IMCC14465 GCA_000293845.2_ASM29384v2
Candidatus.Micropelagos.thuwalensis GCA_000469155.1.RS24
Parvibaculum.lavamentivorans.DS.1 GCA_000017565.1_ASM1756v1
alpha.proteobacterium.L41A GCA_000335735.1.L41A.1.0
Brevundimonas.subvibrioides.ATCC.15264 GCA_000144605.1_ASM14460v1
Asticcacaulis.excentricus.CB.48 GCA_000175215.2_ASM17521v2
Phenylobacterium.zucineum.HLK1 GCA_000017265.1_ASM1726v1
Caulobacter.vibrioides.NA1000 GCA_000022005.1_ASM2200v1
Oceanicaulis.alexandrii.DSM.11625 GCA_000420265.1_ASM42026v1
Maricaulis.maris GCA_003634045.1_ASM363404v1
Hyphomonas.neptunium.ATCC.15444 GCA_000013025.1_ASM1302v1
Hirschia.baltica.ATCC.49814 GCA_000023785.1_ASM2378v1
Parvularcula.bermudensis.HTCC2503 GCA_000152825.2_ASM15282v2
Sagittula.stellata.E.37 GCA_000169415.1_ASM16941v1
Ruegeria.pomeroyi.DSS.3 GCA_000011965.2_ASM1196v2
Octadecabacter.arcticus.238 GCA_000155735.2_ASM15573v2
Jannaschia.rubra GCA_900113265.1.IMG.taxon.2622736430.annotated.assembly
Rubellimicrobium.rubrum GCA_006152115.1_ASM615211v1
Ketogulonicigenium.vulgare GCA_001399515.1_ASM139951v1
Rhodobacter.sphaeroides.ATCC.17029 GCA_000015985.1_ASM1598v1
Paracoccus.denitrificans GCA_004063735.1_ASM406373v1
Neomegalonema.perideroedes.DSM.15528 GCA_000374145.1_ASM37414v1
Sphingopyxis.macrogoltabida GCA_001314325.1_ASM131432v1
Citromicrobium.sp.JL477 GCA_001304795.1_ASM130479v1
Sphingobium.indicum.B90A GCA_000264945.2_ASM26494v2
GCA_016892685.1_ASM1689268v1*
Zymomonas.mobilis.subsp.mobilis.ATCC.31821 GCA_000007105.1_ASM710v1
Sphingomonas.wittichii.RW1 GCA_000016765.1_ASM1676v1
alpha.proteobacterium.AAP81b GCA_001295935.1_ASM129593v1
Kordiimonas.gwangyangensis.DSM.19435 GCA_000375545.1_ASM37554v1
Sneathiella.glossodoripedis.JCM.23214 GCA_000616095.1_ASM61609v1
alpha.proteobacterium.BAL199 GCA_000171835.1_ASM17183v1
Thalassobaculum.salexigens.DSM.19539 GCA_000423805.1_ASM42380v1
Candidatus.Puniceispirillum.marinum.IMCC1322 GCA_000024465.1_ASM2446v1
Oceanibaculum.indicum GCA_003633955.1_ASM363395v1
Rhodovibrio.salinarum.DSM.9154 GCA_000515255.1_ASM51525v1
Kiloniella.laminariae.DSM.19542 GCA_000374005.1_ASM37400v1
Rhodospirillum.centenum GCA_014197805.1_ASM1419780v1
Inquilinus.limosus.DSM.16000 GCA_000423185.1_ASM42318v1
Micavibrio.aeruginosavorus.ARL.13 GCA_000226315.1_ASM22631v1
Rhodospirillum.rubrum.F11 GCA_000225955.1_ASM22595v1
Pararhodospirillum.photometricum.DSM.122 GCA_000284415.2_ASM28441v2
Roseospirillum.parvum GCA_900100455.1
Caenispirillum.salinarum.AK4 GCA_000315795.1
Magnetospirillum.magneticum.AMB.1 GCA_000009985.1_ASM998v1
Thalassospira.profundimaris.WP0211 GCA_000300275.1_ASM30027v1
Terasakiella.pusilla.DSM.6293 GCA_000688235.1_ASM68823v1
Gluconobacter.oxydans.DSM.3504 GCA_000583855.1_ASM58385v1
Acetobacter.aceti.NBRC.14818 GCA_000193495.2_ASM19349v2
Gluconacetobacter.diazotrophicus.PA1.5 GCA_000067045.1_ASM6704v1
Commensalibacter.intestini.A911 GCA_000231445.2_ASM23144v1
Granulibacter.bethesdensis.CGDNIH4 GCA_000576145.2_ASM57614v2
Acidisphaera.rubrifaciens.HS.AP3 GCA_000964365.1_ASM96436v1
Acidiphilium.multivorum.AIU301 GCA_000202835.1_ASM20283v1
Rubritepida.flocculans.DSM.14296 GCA_000425365.1_ASM42536v1
Belnapia.moabensis.DSM.16746 GCA_000745835.1_ASM74583v1
Roseomonas.gilardii GCA_001941945.1_ASM194194v1
Elioraea.tepidiphila.DSM.17972 GCA_000378465.1_ASM37846v1
Tistrella.mobilis.KA081020.065 GCA_000264455.2_ASM26445v2
Geminicoccus.roseus.DSM.18922 GCA_000427665.1_ASM42766v1
GCA_902798535.1*
GCA_902773285.1*
GCA_902796745.1*
GCA_902798325.1*
GCA_902769055.1*
GCA_902796345.1*
GCA_902784055.1*
GCA_902795615.1*
GCA_902772645.1*
GCA_902771535.1*
GCA_902761495.1*
GCA_902790255.1*
GCA_002393025.1_ASM239302v1*
GCA_902796155.1*
Holospora.undulata.HU1 GCA_000388175.3_ASM38817v3
Candidatus.Hepatobacter.penaei GCA_000742475.1_ASM74247v1
GCA_002238905.1_ASM223890v1*
Candidatus.Paracaedibacter.acanthamoebae GCA_000742835.1_ASM74283v1
Candidatus.Odyssella.thessalonicensis.L13 GCA_000190415.2_ASM19041v1
Candidatus.Finniella.inopinata GCA_004210305.1_ASM421030v1
Candidatus.Caedibacter.acanthamoebae GCA_000743035.1_ASM74303v1
Caedimonas.varicaedens GCA_001192655.1_ASM119265v1
Candidatus.Nucleicultrix.amoebiphila.FS5 GCA_002117145.1_ASM211714v1
GCA_900550565.1.UMGS1303*
GCA_900546225.1.UMGS860*
GCA_900546185.1.UMGS859*
GCA_900544565.1.UMGS690*
alpha.proteobacterium.HIMB5 GCA_000299095.1_ASM29909v1
Candidatus.Pelagibacter.ubique.HTCC1062 GCA_000012345.1_ASM1234v1
Candidatus.Pelagibacter.ubique.HIMB058 GCA_000419545.1_ASM41954v1
alpha.proteobacterium.HIMB114 GCA_000163555.2
Magnetofaba.australis.IT.1 GCA_002109495.1_ASM210949v1
Magnetococcus.marinus.MC.1 GCA_000014865.1_ASM1486v1
Nitrosospira.briensis.C.128 GCA_000619905.2_ASM61990v2
Methylotenera.versatilis.301 GCA_000093025.1_ASM9302v1
Ralstonia.pickettii GCA_902374465.1.MGYG.HGUT.01384
Burkholderia.cepacia GCA_009586235.1_ASM958623v1
Dasania.marina.DSM.21967 GCA_000373485.1_ASM37348v1
Congregibacter.litoralis.KT71 GCA_000153125.2_ASM15312v2
Enterobacter.cloacae GCA_000770155.1_ASM77015v1
Alteromonas.macleodii.ATCC.27126 GCA_000172635.2_ASM17263v2
0.532139

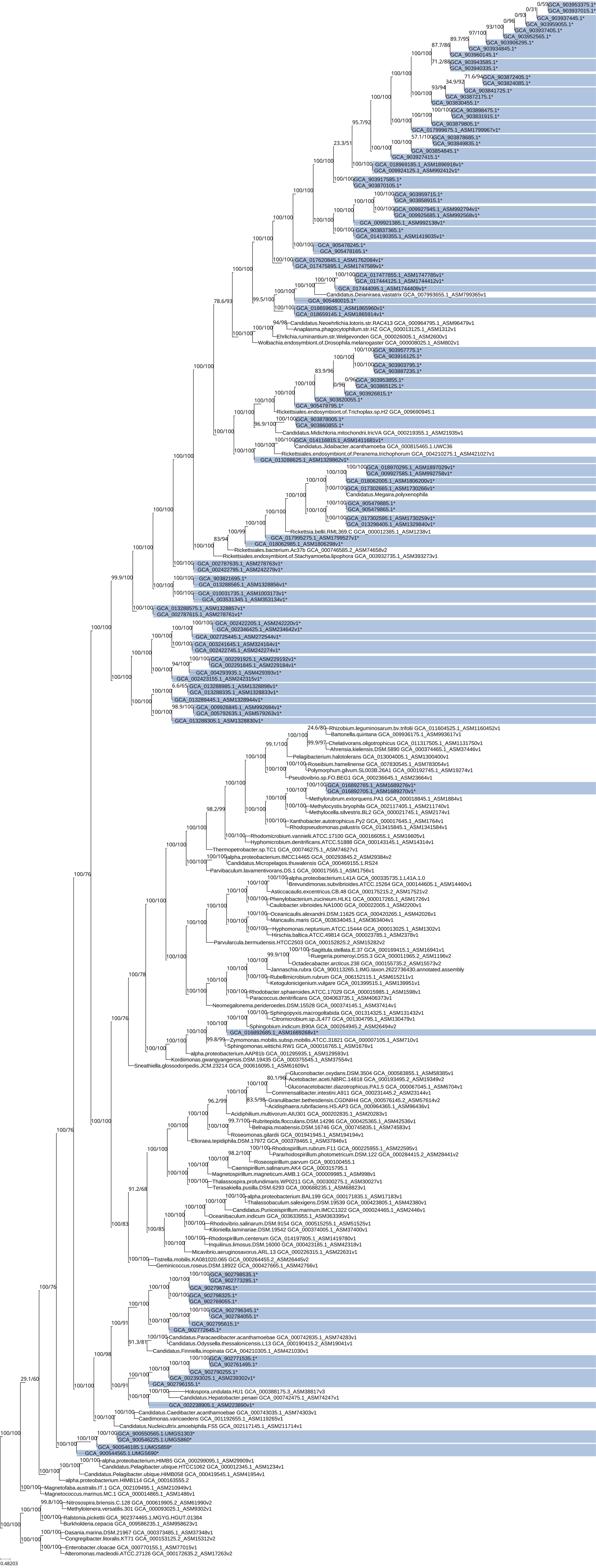

GCA_903953375.1*
GCA_903937015.1*
GCA_903937445.1*
GCA_903959055.1*
GCA_903937405.1*
GCA_903952565.1*
GCA_903906295.1*
GCA_903934845.1*
GCA_903960145.1*
GCA_903943585.1*
GCA_903940335.1*
GCA_903872405.1*
GCA_903824085.1*
GCA_903841725.1*
GCA_903872175.1*
GCA_903830455.1*
GCA_903898475.1*
GCA_903831915.1*
GCA_903879805.1*
GCA_017999675.1_ASM1799967v1*
GCA_903878685.1*
GCA_903849835.1*
GCA_903854845.1*
GCA_903927415.1*
GCA_018969185.1_ASM1896918v1*
GCA_009924125.1_ASM992412v1*
GCA_903917585.1*
GCA_903870105.1*
GCA_903959715.1*
GCA_903858915.1*
GCA_009927945.1_ASM992794v1*
GCA_009925685.1_ASM992568v1*
GCA_009921385.1_ASM992138v1*
GCA_903837365.1*
GCA_014190355.1_ASM1419035v1*
GCA_905478245.1*
GCA_905478165.1*
GCA_017620845.1_ASM1762084v1*
GCA_017475895.1_ASM1747589v1*
GCA_017477855.1_ASM1747785v1*
GCA_017444125.1_ASM1744412v1*
GCA_017444095.1_ASM1744409v1*
Candidatus.Deianiraea.vastatrix GCA_007993655.1_ASM799365v1
GCA_905480015.1*
GCA_018659605.1_ASM1865960v1*
GCA_018659145.1_ASM1865914v1*
Candidatus.Neoehrlichia.lotoris.str.RAC413 GCA_000964795.1_ASM96479v1
Anaplasma.phagocytophilum.str.HZ GCA_000013125.1_ASM1312v1
Ehrlichia.ruminantium.str.Welgevonden GCA_000026005.1_ASM2600v1
Wolbachia.endosymbiont.of.Drosophila.melanogaster GCA_000008025.1_ASM802v1
GCA_903957775.1*
GCA_903916125.1*
GCA_903903795.1*
GCA_903887235.1*
GCA_903953855.1*
GCA_903865125.1*
GCA_903926815.1*
GCA_903820055.1*
GCA_905479795.1*
Rickettsiales.endosymbiont.of.Trichoplax.sp.H2 GCA_009690945.1
GCA_903878005.1*
GCA_903860855.1*
Candidatus.Midichloria.mitochondrii.IricVA GCA_000219355.1_ASM21935v1
GCA_014116815.1_ASM1411681v1*
Candidatus.Jidaibacter.acanthamoeba GCA_000815465.1_UWC36
Rickettsiales.endosymbiont.of.Peranema.trichophorum GCA_004210275.1_ASM421027v1
GCA_013288625.1_ASM1328862v1*
GCA_018970295.1_ASM1897029v1*
GCA_009927585.1_ASM992758v1*
GCA_018062005.1_ASM1806200v1*
GCA_017302665.1_ASM1730266v1*
Candidatus.Megaira.polyxenophila
GCA_905479885.1*
GCA_905479865.1*
GCA_017302595.1_ASM1730259v1*
GCA_013298405.1_ASM1329840v1*
Rickettsia.bellii.RML369.C GCA_000012385.1_ASM1238v1
GCA_017995275.1_ASM1799527v1*
GCA_018062985.1_ASM1806298v1*
Rickettsiales.bacterium.Ac37b GCA_000746585.2_ASM74658v2
Rickettsiales.endosymbiont.of.Stachyamoeba.lipophora GCA_003932735.1_ASM393273v1
GCA_002787635.1_ASM278763v1*
GCA_002422795.1_ASM242279v1*
GCA_903821695.1*
GCA_013288565.1_ASM1328856v1*
GCA_010031735.1_ASM1003173v1*
GCA_003531345.1_ASM353134v1*
GCA_013288575.1_ASM1328857v1*
GCA_002787615.1_ASM278761v1*
GCA_002422205.1_ASM242220v1*
GCA_002346425.1_ASM234642v1*
GCA_002725445.1_ASM272544v1*
GCA_003241645.1_ASM324164v1*
GCA_002422745.1_ASM242274v1*
GCA_002291925.1_ASM229192v1*
GCA_002291845.1_ASM229184v1*
GCA_004293935.1_ASM429393v1*
GCA_002423155.1_ASM242315v1*
GCA_013288985.1_ASM1328898v1*
GCA_013288335.1_ASM1328833v1*
GCA_013289445.1_ASM1328944v1*
GCA_009926845.1_ASM992684v1*
GCA_005792635.1_ASM579263v1*
GCA_013288305.1_ASM1328830v1*
Rhizobium.leguminosarum.bv.trifolii GCA_011604525.1_ASM1160452v1
Bartonella.quintana GCA_009936175.1_ASM993617v1
Chelativorans.oligotrophicus GCA_013317505.1_ASM1131750v1
Ahrensia.kielensis.DSM.5890 GCA_000374465.1_ASM37446v1
Pelagibacterium.halotolerans GCA_013004005.1_ASM1300400v1
Roseibium.hamelinense GCA_007830545.1_ASM783054v1
Polymorphum.gilvum.SL003B.26A1 GCA_000192745.1_ASM19274v1
Pseudovibrio.sp.FO.BEG1 GCA_000236645.1_ASM23664v1
GCA_016892765.1_ASM1689276v1*
GCA_016892705.1_ASM1689270v1*
Methylorubrum.extorquens.PA1 GCA_000018845.1_ASM1884v1
Methylocystis.bryophila GCA_002117405.1_ASM211740v1
Methylocella.silvestris.BL2 GCA_000021745.1_ASM2174v1
Xanthobacter.autotrophicus.Py2 GCA_000017645.1_ASM1764v1
Rhodopseudomonas.palustris GCA_013415845.1_ASM1341584v1
Rhodomicrobium.vannielii.ATCC.17100 GCA_000166055.1_ASM16605v1
Hyphomicrobium.denitrificans.ATCC.51888 GCA_000143145.1_ASM14314v1
Thermopetrobacter.sp.TC1 GCA_000746275.1_ASM74627v1
alpha.proteobacterium.IMCC14465 GCA_000293845.2_ASM29384v2
Candidatus.Micropelagos.thuwalensis GCA_000469155.1.RS24
Parvibaculum.lavamentivorans.DS.1 GCA_000017565.1_ASM1756v1
alpha.proteobacterium.L41A GCA_000335735.1_L41A.1.0
Brevundimonas.subvibrioides.ATCC.15264 GCA_000144605.1_ASM14460v1
Asticcacaulis.excentricus.CB.48 GCA_000175215.2_ASM17521v2
Phenylobacterium.zucineum.HLK1 GCA_000017265.1_ASM1726v1
Caulobacter.vibrioides.NA1000 GCA_000022005.1_ASM2200v1
Oceanicaulis.alexandrii.DSM.11625 GCA_000420265.1_ASM42026v1
Maricaulis.maris GCA_003634045.1_ASM363404v1
Hyphomonas.neptunium.ATCC.15444 GCA_000013025.1_ASM1302v1
Hirschia.baltica.ATCC.49814 GCA_000023785.1_ASM2378v1
Parvularcula.bermudensis.HTCC2503 GCA_000152825.2_ASM15282v2
Sagittula.stellata.E.37 GCA_000169415.1_ASM16941v1
Ruegeria.pomeroyi.DSS.3 GCA_000011965.2_ASM1196v2
Octadecabacter.arcticus.238 GCA_000155735.2_ASM15573v2
Jannaschia.rubra GCA_900113265.1.IMG.taxon.2622736430.annotated.assembly
Rubellimicrobium.rubrum GCA_006152115.1_ASM615211v1
Ketogulonicigenium.vulgare GCA_001399515.1_ASM139951v1
Rhodobacter.sphaeroides.ATCC.17029 GCA_000015985.1_ASM1598v1
Paracoccus.denitrificans GCA_004063735.1_ASM406373v1
Neomegalonema.perideroedes.DSM.15528 GCA_000374145.1_ASM37414v1
Sphingopyxis.macrogoltabida GCA_001314325.1_ASM131432v1
Citromicrobium.sp.JL477 GCA_001304795.1_ASM130479v1
Sphingobium.indicum.B90A GCA_000264945.2_ASM26494v2
GCA_016892685.1_ASM1689268v1*
Zymomonas.mobilis.subsp.mobilis.ATCC.31821 GCA_000007105.1_ASM710v1
Sphingomonas.wittichii.RW1 GCA_000016765.1_ASM1676v1
alpha.proteobacterium.AAP81b GCA_001295935.1_ASM129593v1
Kordiimonas.gwangyangensis.DSM.19435 GCA_000375545.1_ASM37554v1
Sneathiella.glossodoripedis.JCM.23214 GCA_000616095.1_ASM61609v1
Gluconobacter.oxydans.DSM.3504 GCA_000583855.1_ASM58385v1
Acetobacter.aceti.NBRC.14818 GCA_000193495.2_ASM19349v2
Gluconacetobacter.diazotrophicus.PA1.5 GCA_000067045.1_ASM6704v1
Commensalibacter.intestini.A911 GCA_000231445.2_ASM23144v1
Granulibacter.bethesdensis.CGDNIH4 GCA_000576145.2_ASM57614v2
Acidisphaera.rubrifaciens.HS.AP3 GCA_000964365.1_ASM96436v1
Acidiphilium.multivorum.AIU301 GCA_000202835.1_ASM20283v1
Rubritepida.flocculans.DSM.14296 GCA_000425365.1_ASM42536v1
Belnapia.moabensis.DSM.16746 GCA_000745835.1_ASM74583v1
Roseomonas.gilardii GCA_001941945.1_ASM194194v1
Elioraea.tepidiphila.DSM.17972 GCA_000378465.1_ASM37846v1
Rhodospirillum.rubrum.F11 GCA_000225955.1_ASM22595v1
Pararhodospirillum.photometricum.DSM.122 GCA_000284415.2_ASM28441v2
Roseospirillum.parvum GCA_900100455.1
Caenispirillum.salinarum.AK4 GCA_000315795.1
Magnetospirillum.magneticum.AMB.1 GCA_000009985.1_ASM998v1
Thalassospira.profundimaris.WP0211 GCA_000300275.1_ASM30027v1
Terasakiella.pusilla.DSM.6293 GCA_000688235.1_ASM68823v1
alpha.proteobacterium.BAL199 GCA_000171835.1_ASM17183v1
Thalassobaculum.salexigens.DSM.19539 GCA_000423805.1_ASM42380v1
Candidatus.Puniceispirillum.marinum.IMCC1322 GCA_000024465.1_ASM2446v1
Oceanibaculum.indicum GCA_003633955.1_ASM363395v1
Rhodovibrio.salinarum.DSM.9154 GCA_000515255.1_ASM51525v1
Kiloniella.laminariae.DSM.19542 GCA_000374005.1_ASM37400v1
Rhodospirillum.centenum GCA_014197805.1_ASM1419780v1
Inquilinus.limosus.DSM.16000 GCA_000423185.1_ASM42318v1
Micavibrio.aeruginosavorus.ARL.13 GCA_000226315.1_ASM22631v1
Tistrella.mobilis.KA081020.065 GCA_000264455.2_ASM26445v2
Geminicoccus.roseus.DSM.18922 GCA_000427665.1_ASM42766v1
GCA_902798535.1*
GCA_902773285.1*
GCA_902796745.1*
GCA_902798325.1*
GCA_902769055.1*
GCA_902796345.1*
GCA_902784055.1*
GCA_902795615.1*
GCA_902772645.1*
Candidatus.Paracaedibacter.acanthamoebae GCA_000742835.1_ASM74283v1
Candidatus.Odyssella.thessalonicensis.L13 GCA_000190415.2_ASM19041v1
Candidatus.Finniella.inopinata GCA_004210305.1_ASM421030v1
GCA_902771535.1*
GCA_902761495.1*
GCA_902790255.1*
GCA_002393025.1_ASM239302v1*
GCA_902796155.1*
Holospora.undulata.HU1 GCA_000388175.3_ASM38817v3
Candidatus.Hepatobacter.penaei GCA_000742475.1_ASM74247v1
GCA_002238905.1_ASM223890v1*
Candidatus.Caedibacter.acanthamoebae GCA_000743035.1_ASM74303v1
Caedimonas.varicaedens GCA_001192655.1_ASM119265v1
Candidatus.Nucleicultrix.amoebiphila.FS5 GCA_002117145.1_ASM211714v1
GCA_900550565.1.UMGS1303*
GCA_900546225.1.UMGS860*
GCA_900546185.1.UMGS859*
GCA_900544565.1.UMGS690*
alpha.proteobacterium.HIMB5 GCA_000299095.1_ASM29909v1
Candidatus.Pelagibacter.ubique.HTCC1062 GCA_000012345.1_ASM1234v1
Candidatus.Pelagibacter.ubique.HIMB058 GCA_000419545.1_ASM41954v1
alpha.proteobacterium.HIMB114 GCA_000163555.2
Magnetofaba.australis.IT.1 GCA_002109495.1_ASM210949v1
Magnetococcus.marinus.MC.1 GCA_000014865.1_ASM1486v1
Nitrosospira.briensis.C.128 GCA_000619905.2_ASM61990v2
Methylotenera.versatilis.301 GCA_000093025.1_ASM9302v1
Ralstonia.pickettii GCA_902374465.1.MGYG.HGUT.01384
Burkholderia.cepacia GCA_009586235.1_ASM958623v1
Dasania.marina.DSM.21967 GCA_000373485.1_ASM37348v1
Congregibacter.litoralis.KT71 GCA_000153125.2_ASM15312v2
Enterobacter.cloacae GCA_000770155.1_ASM77015v1
Alteromonas.macleodii.ATCC.27126 GCA_000172635.2_ASM17263v2
0.48203

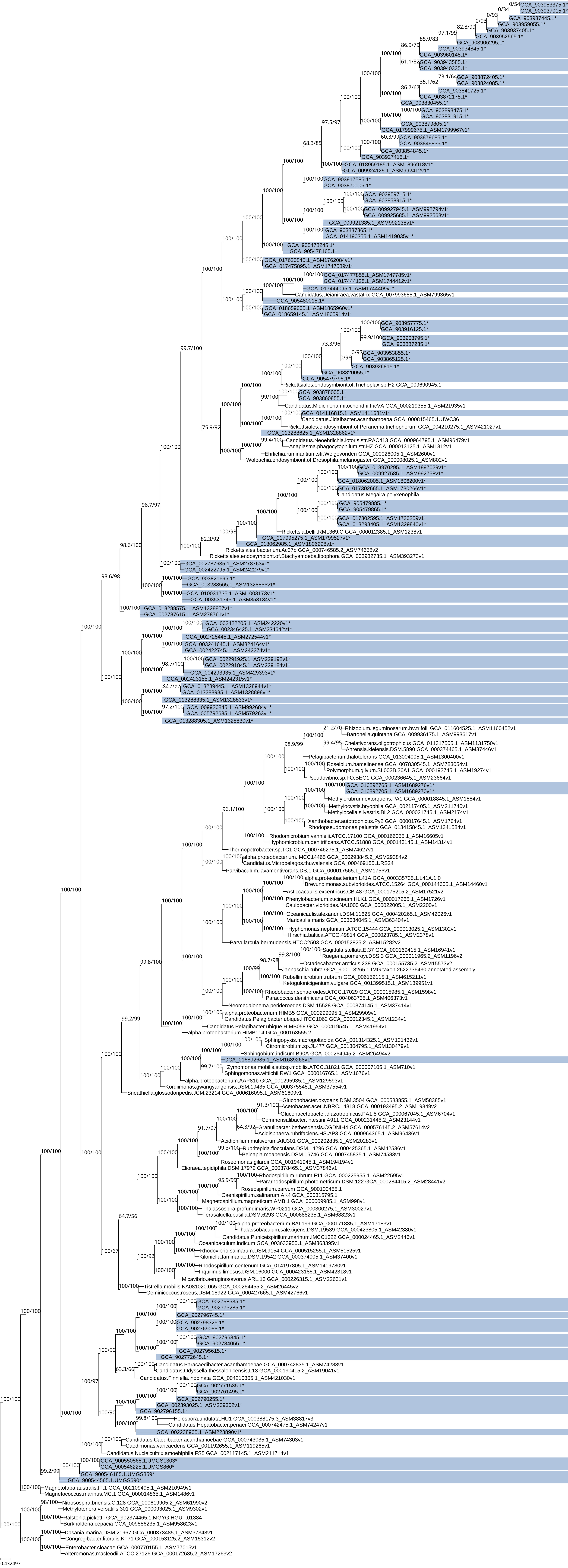

GCA_903953375.1*
GCA_903937015.1*
GCA_903937445.1*
GCA_903959055.1*
GCA_903937405.1*
GCA_903952565.1*
GCA_903906295.1*
GCA_903934845.1*
GCA_903960145.1*
GCA_903943585.1*
GCA_903940335.1*
GCA_903872405.1*
GCA_903824085.1*
GCA_903841725.1*
GCA_903872175.1*
GCA_903830455.1*
GCA_903898475.1*
GCA_903831915.1*
GCA_903879805.1*
GCA_017999675.1_ASM1799967v1*
GCA_903878685.1*
GCA_903849835.1*
GCA_903854845.1*
GCA_903927415.1*
GCA_018969185.1_ASM1896918v1*
GCA_009924125.1_ASM992412v1*
GCA_903917585.1*
GCA_903870105.1*
GCA_903959715.1*
GCA_903858915.1*
GCA_009927945.1_ASM992794v1*
GCA_009925685.1_ASM992568v1*
GCA_009921385.1_ASM992138v1*
GCA_903837365.1*
GCA_014190355.1_ASM1419035v1*
GCA_905478245.1*
GCA_905478165.1*
GCA_017620845.1_ASM1762084v1*
GCA_017475895.1_ASM1747589v1*
GCA_017477855.1_ASM1747785v1*
GCA_017444125.1_ASM1744412v1*
GCA_017444095.1_ASM1744409v1*
Candidatus.Deianiraea.vastatrix GCA_007993655.1_ASM799365v1
GCA_905480015.1*
GCA_018659605.1_ASM1865960v1*
GCA_018659145.1_ASM1865914v1*
GCA_903957775.1*
GCA_903916125.1*
GCA_903903795.1*
GCA_903887235.1*
GCA_903953855.1*
GCA_903865125.1*
GCA_903926815.1*
GCA_903820055.1*
GCA_905479795.1*
Rickettsiales.endosymbiont.of.Trichoplax.sp.H2 GCA_009690945.1
GCA_903878005.1*
GCA_903860855.1*
Candidatus.Midichloria.mitochondrii.IricVA GCA_000219355.1_ASM21935v1
GCA_014116815.1_ASM1411681v1*
Candidatus.Jidaibacter.acanthamoeba GCA_000815465.1.UWC36
Rickettsiales.endosymbiont.of.Peranema.trichophorum GCA_004210275.1_ASM421027v1
GCA_013288625.1_ASM1328862v1*
Candidatus.Neoehrlichia.lotoris.str.RAC413 GCA_000964795.1_ASM96479v1
Anaplasma.phagocytophilum.str.HZ GCA_000013125.1_ASM1312v1
Ehrlichia.ruminantium.str.Welgevonden GCA_000026005.1_ASM2600v1
Wolbachia.endosymbiont.of.Drosophila.melanogaster GCA_000008025.1_ASM802v1
GCA_018970295.1_ASM1897029v1*
GCA_009927585.1_ASM992758v1*
GCA_018062005.1_ASM1806200v1*
GCA_017302665.1_ASM1730266v1*
Candidatus.Megaira.polyxenophila
GCA_905479885.1*
GCA_905479865.1*
GCA_017302595.1_ASM1730259v1*
GCA_013298405.1_ASM1329840v1*
Rickettsia.bellii.RML369.C GCA_000012385.1_ASM1238v1
GCA_017995275.1_ASM1799527v1*
GCA_018062985.1_ASM1806298v1*
Rickettsiales.bacterium.Ac37b GCA_000746585.2_ASM74658v2
Rickettsiales.endosymbiont.of.Stachyamoeba.lipophora GCA_003932735.1_ASM393273v1
GCA_002787635.1_ASM278763v1*
GCA_002422795.1_ASM242279v1*
GCA_903821695.1*
GCA_013288565.1_ASM1328856v1*
GCA_010031735.1_ASM1003173v1*
GCA_003531345.1_ASM353134v1*
GCA_013288575.1_ASM1328857v1*
GCA_002787615.1_ASM278761v1*
GCA_002422205.1_ASM242220v1*
GCA_002346425.1_ASM234642v1*
GCA_002725445.1_ASM272544v1*
GCA_003241645.1_ASM324164v1*
GCA_002422745.1_ASM242274v1*
GCA_002291925.1_ASM229192v1*
GCA_002291845.1_ASM229184v1*
GCA_004293935.1_ASM429393v1*
GCA_002423155.1_ASM242315v1*
GCA_013289445.1_ASM1328944v1*
GCA_013288985.1_ASM1328898v1*
GCA_013288335.1_ASM1328833v1*
GCA_009926845.1_ASM992684v1*
GCA_005792635.1_ASM579263v1*
GCA_013288305.1_ASM1328830v1*
Rhizobium.leguminosarum.bv.trifolii GCA_011604525.1_ASM1160452v1
Bartonella.quintana GCA_009936175.1_ASM993617v1
Chelativorans.oligotrophicus GCA_011317505.1_ASM1131750v1
Ahrensia.kielensis.DSM.5890 GCA_000374465.1_ASM37446v1
Pelagibacterium.halotolerans GCA_013004005.1_ASM1300400v1
Roseibium.hamelinense GCA_007830545.1_ASM783054v1
Polymorphum.gilvum.SL003B.26A1 GCA_000192745.1_ASM19274v1
Pseudovibrio.sp.FO.BEG1 GCA_000236645.1_ASM23664v1
GCA_016892765.1_ASM1689276v1*
GCA_016892705.1_ASM1689270v1*
Methylorubrum.extorquens.PA1 GCA_000018845.1_ASM1884v1
Methylocystis.bryophila GCA_002117405.1_ASM211740v1
Methylocella.silvestris.BL2 GCA_000021745.1_ASM2174v1
Xanthobacter.autotrophicus.Py2 GCA_000017645.1_ASM1764v1
Rhodopseudomonas.palustris GCA_013415845.1_ASM1341584v1
Rhodomicrobium.vannielii.ATCC.17100 GCA_000166055.1_ASM16605v1
Hyphomicrobium.denitrificans.ATCC.51888 GCA_000143145.1_ASM14314v1
Thermopetrobacter.sp.TC1 GCA_000746275.1_ASM74627v1
alpha.proteobacterium.IMCC14465 GCA_000293845.2_ASM29384v2
Candidatus.Micropelagos.thuwalensis GCA_000469155.1.RS24
Parvibaculum.lavamentivorans.DS.1 GCA_000017565.1_ASM1756v1
alpha.proteobacterium.L41A GCA_000335735.1_L41A.1.0
Brevundimonas.subvibrioides.ATCC.15264 GCA_000144605.1_ASM14460v1
Asticcacaulis.excentricus.CB.48 GCA_000175215.2_ASM17521v2
Phenylobacterium.zucineum.HLK1 GCA_000017265.1_ASM1726v1
Caulobacter.vibrioides.NA1000 GCA_000022005.1_ASM2200v1
Oceanicaulis.alexandrii.DSM.11625 GCA_000420265.1_ASM42026v1
Maricaulis.maris GCA_003634045.1_ASM363404v1
Hyphomonas.neptunium.ATCC.15444 GCA_000013025.1_ASM1302v1
Hirschia.baltica.ATCC.49814 GCA_000023785.1_ASM2378v1
Parvularcula.bermudensis.HTCC2503 GCA_000152825.2_ASM15282v2
Sagittula.stellata.E.37 GCA_000169415.1_ASM16941v1
Ruegeria.pomeroyi.DSS.3 GCA_000011965.2_ASM1196v2
Octadecabacter.arcticus.238 GCA_000155735.2_ASM15573v2
Jannaschia.rubra GCA_900113265.1.IMG.taxon.2622736430.annotated.assembly
Rubellimicrobium.rubrum GCA_006152115.1_ASM615211v1
Ketogulonicigenium.vulgare GCA_001399515.1_ASM139951v1
Rhodobacter.sphaeroides.ATCC.17029 GCA_000015985.1_ASM1598v1
Paracoccus.denitrificans GCA_004063735.1_ASM406373v1
Neomegalonema.perideroedes.DSM.15528 GCA_000374145.1_ASM37414v1
alpha.proteobacterium.HIMB5 GCA_000299095.1_ASM29909v1
Candidatus.Pelagibacter.ubique.HTCC1062 GCA_000012345.1_ASM1234v1
Candidatus.Pelagibacter.ubique.HIMB058 GCA_000419545.1_ASM41954v1
alpha.proteobacterium.HIMB114 GCA_000163555.2
Sphingopyxis.macrogoltabida GCA_001314325.1_ASM131432v1
Citromicrobium.sp.JL477 GCA_001304795.1_ASM130479v1
Sphingobium.indicum.B90A GCA_000264945.2_ASM26494v2
GCA_016892685.1_ASM1689268v1*
Zymomonas.mobilis.subsp.mobilis.ATCC.31821 GCA_000007105.1_ASM710v1
Sphingomonas.wittichii.RW1 GCA_000016765.1_ASM1676v1
alpha.proteobacterium.AAP81b GCA_001295935.1_ASM129593v1
Kordiimonas.gwangyangensis.DSM.19435 GCA_000375545.1_ASM37554v1
Sneathiella.glossodoripedis.JCM.23214 GCA_000616095.1_ASM61609v1
Gluconobacter.oxydans.DSM.3504 GCA_000583855.1_ASM58385v1
Acetobacter.aceti.NBRC.14818 GCA_000193495.2_ASM19349v2
Gluconacetobacter.diazotrophicus.PA1.5 GCA_000067045.1_ASM6704v1
Commensalibacter.intestini.A911 GCA_000231445.2_ASM23144v1
Granulibacter.bethesdensis.CGDNIH4 GCA_000576145.2_ASM57614v2
Acidisphaera.rubrifaciens.HS.AP3 GCA_000964365.1_ASM96436v1
Acidiphilium.multivorum.AIU301 GCA_000202835.1_ASM20283v1
Rubritepida.flocculans.DSM.14296 GCA_000425365.1_ASM42536v1
Belnapia.moabensis.DSM.16746 GCA_000745835.1_ASM74583v1
Roseomonas.gilardii GCA_001941945.1_ASM194194v1
Elioraea.tepidiphila.DSM.17972 GCA_000378465.1_ASM37846v1
Rhodospirillum.rubrum.F11 GCA_000225955.1_ASM22595v1
Pararhodospirillum.photometricum.DSM.122 GCA_000284415.2_ASM28441v2
Roseospirillum.parvum GCA_900100455.1
Caenispirillum.salinarum.AK4 GCA_000315795.1
Magnetospirillum.magneticum.AMB.1 GCA_000009985.1_ASM998v1
Thalassospira.profundimaris.WP0211 GCA_000300275.1_ASM30027v1
Terasakiella.pusilla.DSM.6293 GCA_000688235.1_ASM68823v1
alpha.proteobacterium.BAL199 GCA_000171835.1_ASM17183v1
Thalassobaculum.salexigens.DSM.19539 GCA_000423805.1_ASM42380v1
Candidatus.Puniceispirillum.marinum.IMCC1322 GCA_000024465.1_ASM2446v1
Oceanibaculum.indicum GCA_003633955.1_ASM363395v1
Rhodovibrio.salinarum.DSM.9154 GCA_000515255.1_ASM51525v1
Kiloniella.laminariae.DSM.19542 GCA_000374005.1_ASM37400v1
Rhodospirillum.centenum GCA_014197805.1_ASM1419780v1
Inquilinus.limosus.DSM.16000 GCA_000423185.1_ASM42318v1
Micavibrio.aeruginosavorus.ARL.13 GCA_000226315.1_ASM22631v1
Tistrella.mobilis.KA081020.065 GCA_000264455.2_ASM26445v2
Geminicoccus.roseus.DSM.18922 GCA_000427665.1_ASM42766v1
GCA_902798535.1*
GCA_902773285.1*
GCA_902796745.1*
GCA_902798325.1*
GCA_902769055.1*
GCA_902796345.1*
GCA_902784055.1*
GCA_902795615.1*
GCA_902772645.1*
Candidatus.Paracaedibacter.acanthamoebae GCA_000742835.1_ASM74283v1
Candidatus.Odyssella.thessalonicensis.L13 GCA_000190415.2_ASM19041v1
Candidatus.Finniella.inopinata GCA_004210305.1_ASM421030v1
GCA_902771535.1*
GCA_902761495.1*
GCA_902790255.1*
GCA_002393025.1_ASM239302v1*
GCA_902796155.1*
Holospora.undulata.HU1 GCA_000388175.3_ASM38817v3
Candidatus.Hepatobacter.penaei GCA_000742475.1_ASM74247v1
GCA_002238905.1_ASM223890v1*
Candidatus.Caedibacter.acanthamoebae GCA_000743035.1_ASM74303v1
Caedimonas.varicaedens GCA_001192655.1_ASM119265v1
Candidatus.Nucleicultrix.amoebiphila.FS5 GCA_002117145.1_ASM211714v1
GCA_900550565.1.UMGS1303*
GCA_900546225.1.UMGS860*
GCA_900546185.1.UMGS859*
GCA_900544565.1.UMGS690*
Magnetofaba.australis.IT.1 GCA_002109495.1_ASM210949v1
Magnetococcus.marinus.MC.1 GCA_000014865.1_ASM1486v1
Nitrosospira.briensis.C.128 GCA_000619905.2_ASM61990v2
Methylotenera.versatilis.301 GCA_000093025.1_ASM9302v1
Ralstonia.pickettii GCA_902374465.1.MGYG.HGUT.01384
Burkholderia.cepacia GCA_009586235.1_ASM958623v1
Dasania.marina.DSM.21967 GCA_000373485.1_ASM37348v1
Congregibacter.litoralis.KT71 GCA_000153125.2_ASM15312v2
Enterobacter.cloacae GCA_000770155.1_ASM77015v1
Alteromonas.macleodii.ATCC.27126 GCA_000172635.2_ASM17263v2
0.432497

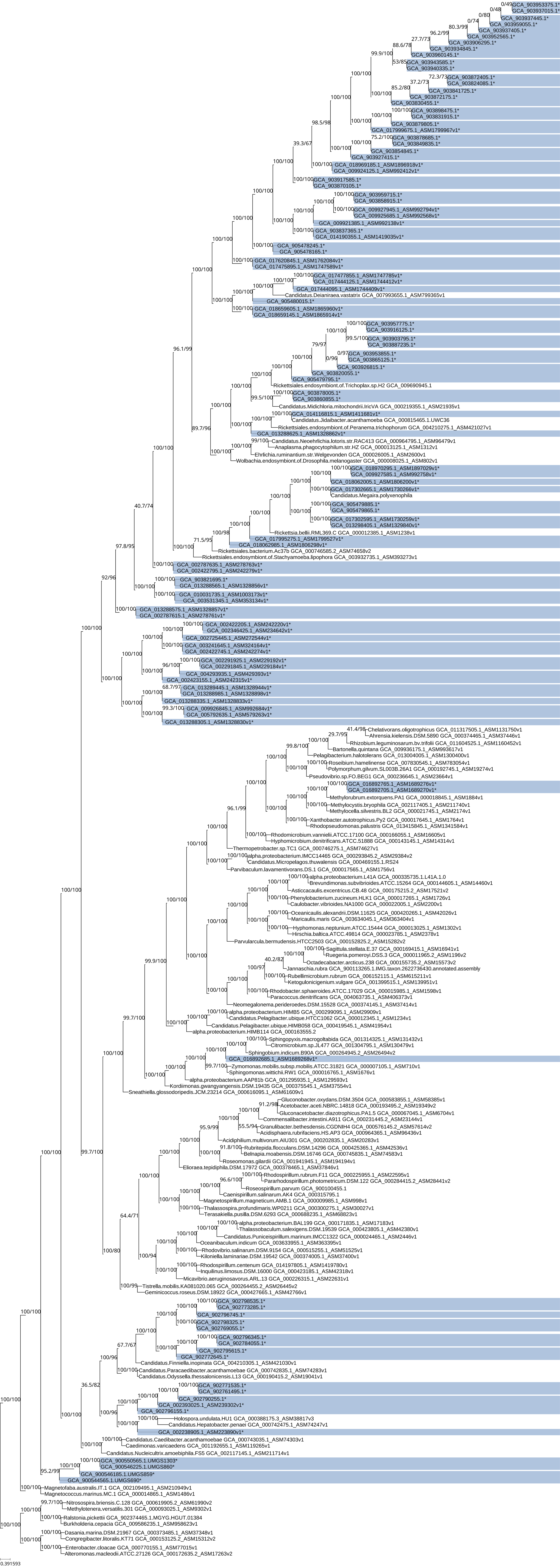

GCA_903953575.1*
GCA_903937015.1*
GCA_903937445.1*
GCA_903959055.1*
GCA_903937405.1*
GCA_903952565.1*
GCA_903906295.1*
GCA_903934845.1*
GCA_903960145.1*
GCA_903943585.1*
GCA_903940335.1*
GCA_903872405.1*
GCA_903824085.1*
GCA_903841725.1*
GCA_903872175.1*
GCA_903830455.1*
GCA_903898475.1*
GCA_903831915.1*
GCA_903879805.1*
GCA_017999675.1_ASM1799967v1*
GCA_903878685.1*
GCA_903849835.1*
GCA_903854845.1*
GCA_903927415.1*
GCA_018969185.1_ASM1896918v1*
GCA_009924125.1_ASM992412v1*
GCA_903917585.1*
GCA_903870105.1*
GCA_903959715.1*
GCA_903858915.1*
GCA_009927945.1_ASM992794v1*
GCA_009925685.1_ASM992568v1*
GCA_009921385.1_ASM992138v1*
GCA_903837365.1*
GCA_014190355.1_ASM1419035v1*
GCA_905478245.1*
GCA_905478165.1*
GCA_017620845.1_ASM1762084v1*
GCA_017475895.1_ASM1747589v1*
GCA_017477855.1_ASM1747785v1*
GCA_017444125.1_ASM1744412v1*
GCA_017444095.1_ASM1744409v1*
Candidatus.Deianiraea.vastatrix GCA_007993655.1_ASM799365v1
GCA_905480015.1*
GCA_018659605.1_ASM1865960v1*
GCA_018659145.1_ASM1865914v1*
GCA_903957775.1*
GCA_903916125.1*
GCA_903903795.1*
GCA_903887235.1*
GCA_903953855.1*
GCA_903865125.1*
GCA_903926815.1*
GCA_903820055.1*
GCA_905479795.1*
Rickettsiales.endosymbiont.of.Trichoplax.sp.H2 GCA_000690985.1
GCA_903878005.1*
GCA_903860855.1*
Candidatus.Midichloria.mitochondrii.IricVA GCA_000219355.1_ASM21935v1
GCA_014116815.1_ASM1411681v1*
Candidatus.Jidaibacter.acanthamoeba GCA_000815465.1.UWC36
Rickettsiales.endosymbiont.of.Peranema.trichophorum GCA_004210275.1_ASM421027v1
GCA_013288625.1_ASM1328862v1*
Candidatus.Neoehrlichia.lotoris.str.RAC413 GCA_000964795.1_ASM96479v1
Anaplasma.phagocytophilum.str.HZ GCA_000013125.1_ASM1312v1
Ehrlichia.ruminantium.str.Welgevonden GCA_000026005.1_ASM2600v1
Wolbachia.endosymbiont.of.Drosophila.melanogaster GCA_000008025.1_ASM802v1
GCA_018970295.1_ASM1897029v1*
GCA_009927585.1_ASM992758v1*
GCA_018062005.1_ASM1806200v1*
GCA_017302665.1_ASM1730266v1*
Candidatus.Megaira.polyxenophila
GCA_905479885.1*
GCA_905479865.1*
GCA_017302595.1_ASM1730259v1*
GCA_013298405.1_ASM1329840v1*
Rickettsia.bellii.RML369.C GCA_000012385.1_ASM1238v1
GCA_017995275.1_ASM1799527v1*
GCA_018062985.1_ASM1806298v1*
Rickettsiales.bacterium.Ac37b GCA_000746585.2_ASM74658v2
Rickettsiales.endosymbiont.of.Stachyamoeba.lipophora GCA_003932735.1_ASM393273v1
GCA_002787635.1_ASM278763v1*
GCA_002422795.1_ASM242279v1*
GCA_903821695.1*
GCA_013288565.1_ASM1328856v1*
GCA_010031735.1_ASM1003173v1*
GCA_003531345.1_ASM353134v1*
GCA_013288575.1_ASM1328857v1*
GCA_002787615.1_ASM278761v1*
GCA_002422205.1_ASM242220v1*
GCA_002346425.1_ASM234642v1*
GCA_002725445.1_ASM272544v1*
GCA_003241645.1_ASM324164v1*
GCA_002422745.1_ASM242274v1*
GCA_002291925.1_ASM229192v1*
GCA_002291845.1_ASM229184v1*
GCA_004293935.1_ASM429393v1*
GCA_002423155.1_ASM242315v1*
GCA_013289445.1_ASM1328944v1*
GCA_013288985.1_ASM1328898v1*
GCA_013288335.1_ASM1328833v1*
GCA_009926845.1_ASM992684v1*
GCA_005792635.1_ASM579263v1*
GCA_013288305.1_ASM1328830v1*
Chelativorans.oligotrophicus GCA_011317505.1_ASM1131750v1
Ahrensia.kielensis.DSM.5890 GCA_000374465.1_ASM37446v1
Rhizobium.leguminosarum.bv.trifolii GCA_011604525.1_ASM1160452v1
Bartonella.quintana GCA_009936175.1_ASM993617v1
Pelagibacterium.halotolerans GCA_013004005.1_ASM1300400v1
Roseibium.hamelinense GCA_007830545.1_ASM783054v1
Polymorphum.gilvum.SL003B.26A1 GCA_000192745.1_ASM19274v1
Pseudovibrio.sp.FO.BEG1 GCA_000236645.1_ASM23664v1
GCA_016892765.1_ASM1689276v1*
GCA_016892705.1_ASM1689270v1*
Methylorubrum.extorquens.PA1 GCA_000018845.1_ASM1884v1
Methylocystis.bryophila GCA_002117405.1_ASM211740v1
Methylocella.silvestris.BL2 GCA_000021745.1_ASM2174v1
Xanthobacter.autotrophicus.Py2 GCA_000017645.1_ASM1764v1
Rhodopseudomonas.palustris GCA_013415845.1_ASM1341584v1
Rhodomicrobium.vannielii.ATCC.17100 GCA_000166055.1_ASM16605v1
Hyphomicrobium.denitrificans.ATCC.51888 GCA_000143145.1_ASM14314v1
Thermopetrobacter.sp.TC1 GCA_000746275.1_ASM74627v1
alpha.proteobacterium.IMCC14465 GCA_000293845.2_ASM29384v2
Candidatus.Micropelagos.thuwalensis GCA_000469155.1.RS24
Parvibaculum.lavamentivorans.DS.1 GCA_000017565.1_ASM1756v1
alpha.proteobacterium.L41A GCA_000335735.1.L41A.1.0
Brevundimonas.subvibrioides.ATCC.15264 GCA_000144605.1_ASM14460v1
Asticcacaulis.excentricus.CB.48 GCA_000175215.2_ASM17521v2
Phenylobacterium.zucineum.HLK1 GCA_000017265.1_ASM1726v1
Caulobacter.vibrioides.NA1000 GCA_000022005.1_ASM2200v1
Oceanicaulis.alexandrii.DSM.11625 GCA_000420265.1_ASM42026v1
Maricaulis.maris GCA_003634045.1_ASM363404v1
Hyphomonas.neptunium.ATCC.15444 GCA_000013025.1_ASM1302v1
Hirschia.baltica.ATCC.49814 GCA_000023785.1_ASM2378v1
Parvularcula.bermudensis.HTCC2503 GCA_000152825.2_ASM15282v2
Sagittula.stellata.E.37 GCA_000169415.1_ASM16941v1
Ruegeria.pomeroyi.DSS.3 GCA_000011965.2_ASM1196v2
Octadecabacter.arcticus.238 GCA_000155735.2_ASM15573v2
Jannaschia.rubra GCA_900113265.1.IMG.taxon.2622736430.annotated.assembly
Rubellimicrobium.rubrum GCA_006152115.1_ASM615211v1
Ketogulonicigenium.vulgare GCA_001399515.1_ASM139951v1
Rhodobacter.sphaeroides.ATCC.17029 GCA_000015985.1_ASM1598v1
Paracoccus.denitrificans GCA_004063735.1_ASM406373v1
Neomegalonema.perideroedes.DSM.15528 GCA_000374145.1_ASM37414v1
alpha.proteobacterium.HIMB5 GCA_000299095.1_ASM29909v1
Candidatus.Pelagibacter.ubique.HTCC1062 GCA_000012345.1_ASM1234v1
Candidatus.Pelagibacter.ubique.HIMB058 GCA_000419545.1_ASM41954v1
alpha.proteobacterium.HIMB114 GCA_000163555.2
Sphingopyxis.macrogoltabida GCA_001314325.1_ASM131432v1
Citromicrobium.sp.JL477 GCA_001304795.1_ASM130479v1
Sphingobium.indicum.B90A GCA_000264945.2_ASM26494v2
GCA_016892685.1_ASM1689268v1*
Zymomonas.mobilis.subsp.mobilis.ATCC.31821 GCA_000007105.1_ASM710v1
Sphingomonas.wittichii.RW1 GCA_000016765.1_ASM1676v1
alpha.proteobacterium.AAP81b GCA_001295935.1_ASM129593v1
Kordiimonas.gwangyangensis.DSM.19435 GCA_000375545.1_ASM37554v1
Sneathiella.glossodoripedis.JCM.23214 GCA_000616095.1_ASM61609v1
Gluconobacter.oxydans.DSM.3504 GCA_000583855.1_ASM58385v1
Acetobacter.aceti.NBRC.14818 GCA_000193495.2_ASM19349v2
Gluconacetobacter.diazotrophicus.PA1.5 GCA_000067045.1_ASM6704v1
Commensalibacter.intestini.A911 GCA_000231445.2_ASM23144v1
Granulibacter.bethesdensis.CGDNIH4 GCA_000576145.2_ASM57614v2
Acidisphaera.rubrifaciens.HS.AP3 GCA_000964365.1_ASM96436v1
Acidiphilium.multivorum.AIU301 GCA_000202835.1_ASM20283v1
Rubritepida.flocculans.DSM.14296 GCA_000425365.1_ASM42536v1
Belnapia.moabensis.DSM.16746 GCA_000745835.1_ASM74583v1
Roseomonas.gilardii GCA_001941945.1_ASM194194v1
Elioraea.tepidiphila.DSM.17972 GCA_000378465.1_ASM37846v1
Rhodospirillum.rubrum.F11 GCA_000225955.1_ASM22595v1
Pararhodospirillum.photometricum.DSM.122 GCA_000284415.2_ASM28441v2
Roseospirillum.parvum GCA_900100455.1
Caenispirillum.salinarum.AK4 GCA_000315795.1
Magnetospirillum.magneticum.AMB.1 GCA_000009985.1_ASM998v1
Thalassospira.profundimaris.WP0211 GCA_000300275.1_ASM30027v1
Terasakiella.pusilla.DSM.6293 GCA_000688235.1_ASM68823v1
alpha.proteobacterium.BAL199 GCA_000171835.1_ASM17183v1
Thalassobaculum.salexigens.DSM.19539 GCA_000423805.1_ASM42380v1
Candidatus.Puniceispirillum.marinum.IMCC1322 GCA_000024465.1_ASM2446v1
Oceanibaculum.indicum GCA_003633955.1_ASM363395v1
Rhodovibrio.salinarum.DSM.9154 GCA_000515255.1_ASM51525v1
Kiloniella.laminariae.DSM.19542 GCA_000374005.1_ASM37400v1
Rhodospirillum.centenum GCA_014197805.1_ASM1419780v1
Inquilinus.limosus.DSM.16000 GCA_000423185.1_ASM42318v1
Micavibrio.aeruginosavorus.ARL.13 GCA_000226315.1_ASM22631v1
Tistrella.mobilis.KA081020.065 GCA_000264455.2_ASM26445v2
Geminicoccus.roseus.DSM.18922 GCA_000427665.1_ASM42766v1
GCA_902798535.1*
GCA_902773285.1*
GCA_902796745.1*
GCA_902798325.1*
GCA_902769055.1*
GCA_902796345.1*
GCA_902784055.1*
GCA_902795615.1*
GCA_902772645.1*
Candidatus.Finniella.inopinata GCA_004210305.1_ASM421030v1
Candidatus.Paracaedibacter.acanthamoebae GCA_000742835.1_ASM74283v1
Candidatus.Odyssella.thessalonicensis.L13 GCA_000190415.2_ASM19041v1
GCA_902771535.1*
GCA_902761495.1*
GCA_902790255.1*
GCA_002393025.1_ASM239302v1*
GCA_902796155.1*
Holospora.undulata.HU1 GCA_000388175.3_ASM38817v3
Candidatus.Hepatobacter.penaei GCA_000742475.1_ASM74247v1
GCA_002238905.1_ASM223890v1*
Candidatus.Caedibacter.acanthamoebae GCA_000743035.1_ASM74303v1
Caedimonas.varicaedens GCA_001192655.1_ASM119265v1
Candidatus.Nucleicultrix.amoebiphila.FS5 GCA_002117145.1_ASM211714v1
GCA_900550565.1.UMGS1303*
GCA_900546225.1.UMGS860*
GCA_900546185.1.UMGS859*
GCA_900544565.1.UMGS690*
Magnetofaba.australis.IT.1 GCA_002109495.1_ASM210949v1
Magnetococcus.marinus.MC.1 GCA_000014865.1_ASM1486v1
Nitrosospira.briensis.C.128 GCA_000619905.2_ASM61990v2
Methylotenera.versatilis.301 GCA_000093025.1_ASM9302v1
Ralstonia.pickettii GCA_902374465.1.MGYG.HGUT.01384
Burkholderia.cepacia GCA_009586235.1_ASM958623v1
Dasania.marina.DSM.21967 GCA_000373485.1_ASM37348v1
Congregibacter.litoralis.KT71 GCA_000153125.2_ASM15312v2
Enterobacter.cloacae GCA_000770155.1_ASM77015v1
Alteromonas.macleodii.ATCC.27126 GCA_000172635.2_ASM17263v2
0.391593

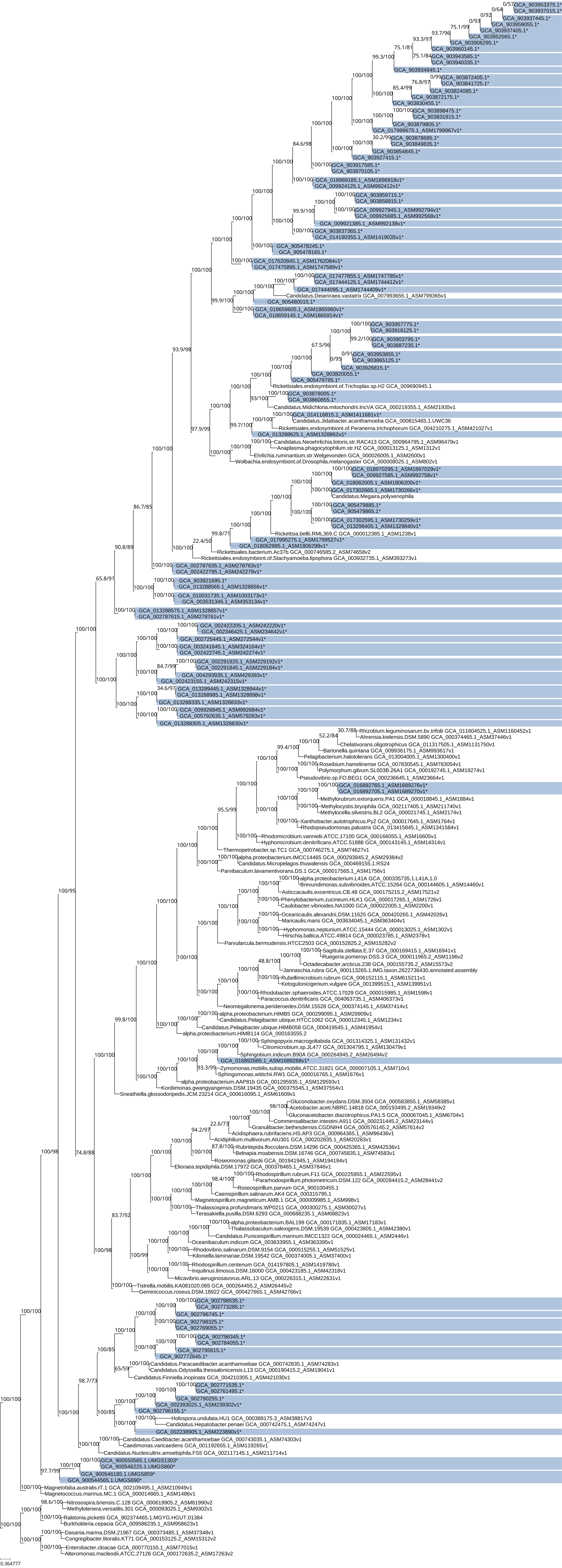

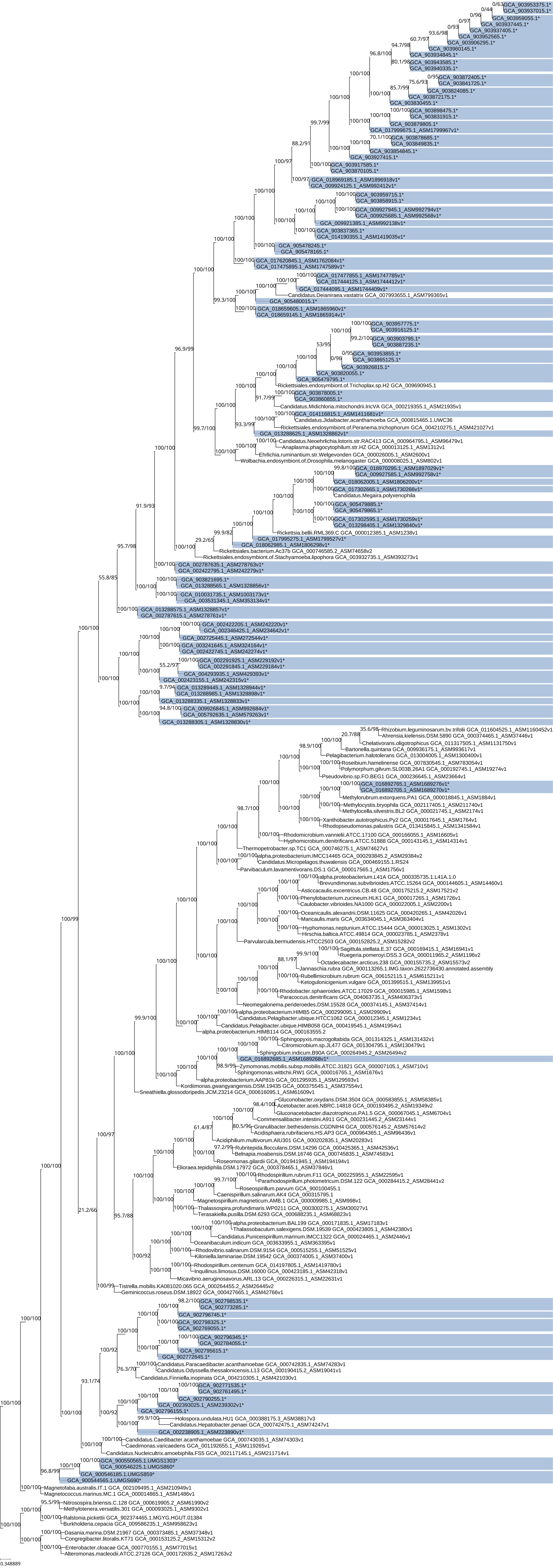

GCA_903953375.1*
GCA_903937015.1*
GCA_903959055.1*
GCA_903937445.1*
GCA_903937405.1*
GCA_903952565.1*
GCA_903906295.1*
GCA_903960145.1*
GCA_903934845.1*
GCA_903943585.1*
GCA_903940335.1*
GCA_903872405.1*
GCA_903841725.1*
GCA_903824085.1*
GCA_903872175.1*
GCA_903830455.1*
GCA_903898475.1*
GCA_903831915.1*
GCA_903879805.1*
GCA_017999675.1_ASM1799967v1*
GCA_903878685.1*
GCA_903849835.1*
GCA_903854845.1*
GCA_903927415.1*
GCA_903917585.1*
GCA_903870105.1*
GCA_018969185.1_ASM1896918v1*
GCA_009924125.1_ASM992412v1*
GCA_903959715.1*
GCA_903858915.1*
GCA_009927945.1_ASM992794v1*
GCA_009925685.1_ASM992568v1*
GCA_009921385.1_ASM992138v1*
GCA_903837365.1*
GCA_014190355.1_ASM1419035v1*
GCA_905478245.1*
GCA_905478165.1*
GCA_017620845.1_ASM1762084v1*
GCA_017475895.1_ASM1747589v1*
GCA_017477855.1_ASM1747785v1*
GCA_017444125.1_ASM1744412v1*
GCA_017444095.1_ASM1744409v1*
Candidatus.Deianiraea.vastatrix GCA_007993655.1_ASM799365v1
GCA_905480015.1*
GCA_018659605.1_ASM1865960v1*
GCA_018659145.1_ASM1865914v1*
GCA_903957775.1*
GCA_903916125.1*
GCA_903903795.1*
GCA_903887235.1*
GCA_903953855.1*
GCA_903865125.1*
GCA_903926815.1*
GCA_903820055.1*
GCA_905479795.1*
Rickettsiales.endosymbiont.of.Trichoplax.sp.H2 GCA_009690945.1
GCA_903878005.1*
GCA_903860855.1*
Candidatus.Midichloria.mitochondrii.IricVA GCA_000219355.1_ASM21935v1
GCA_014116815.1_ASM1411681v1*
Candidatus.Jidaibacter.acanthamoeba GCA_000815465.1_UWC36
Rickettsiales.endosymbiont.of.Peranema.trichophorum GCA_004210275.1_ASM421027v1
GCA_013288625.1_ASM1328862v1*
Candidatus.Neoehrlichia.lotoris.str.RAC413 GCA_000964795.1_ASM96479v1
Anaplasma.phagocytophilum.str.HZ GCA_000013125.1_ASM1312v1
Ehrlichia.ruminantium.str.Welgevonden GCA_000026005.1_ASM2600v1
Wolbachia.endosymbiont.of.Drosophila.melanogaster GCA_000008025.1_ASM802v1
GCA_018970295.1_ASM1897029v1*
GCA_009927585.1_ASM992758v1*
GCA_018062005.1_ASM1806200v1*
GCA_017302665.1_ASM1730266v1*
Candidatus.Megaira.polyxenophila
GCA_905479885.1*
GCA_905479865.1*
GCA_017302595.1_ASM1730259v1*
GCA_013298405.1_ASM1329840v1*
Rickettsia.bellii.RML369.C GCA_000012385.1_ASM1238v1
GCA_017995275.1_ASM1799527v1*
GCA_018062985.1_ASM1806298v1*
Rickettsiales.bacterium.Ac37b GCA_000746585.2_ASM74658v2
Rickettsiales.endosymbiont.of.Stachyamoeba.lipophora GCA_003932735.1_ASM393273v1
GCA_002787635.1_ASM278763v1*
GCA_002422795.1_ASM242279v1*
GCA_903821695.1*
GCA_013288565.1_ASM1328856v1*
GCA_010031735.1_ASM1003173v1*
GCA_003531345.1_ASM353134v1*
GCA_013288575.1_ASM1328857v1*
GCA_002787615.1_ASM278761v1*
GCA_002422205.1_ASM242220v1*
GCA_002346425.1_ASM234642v1*
GCA_002725445.1_ASM272544v1*
GCA_003241645.1_ASM324164v1*
GCA_002422745.1_ASM242274v1*
GCA_002291925.1_ASM229192v1*
GCA_002291845.1_ASM229184v1*
GCA_004293935.1_ASM429393v1*
GCA_002423155.1_ASM242315v1*
GCA_013289445.1_ASM1328944v1*
GCA_013288985.1_ASM1328898v1*
GCA_013288335.1_ASM1328833v1*
GCA_009926845.1_ASM992684v1*
GCA_005792635.1_ASM579263v1*
GCA_013288305.1_ASM1328830v1*
Rhizobium.leguminosarum.bv.trifolii GCA_011604525.1_ASM1160452v1
Ahrensia.kielensis.DSM.5890 GCA_000374465.1_ASM37446v1
Chelativorans.oligotrophicus GCA_011317505.1_ASM1131750v1
Bartonella.quintana GCA_009936175.1_ASM993617v1
Pelagibacterium.halotolerans GCA_013004005.1_ASM1300400v1
Roseibium.hamelinense GCA_007830545.1_ASM783054v1
Polymorphum.gilvum.SL003B.26A1 GCA_000192745.1_ASM19274v1
Pseudovibrio.sp.FO.BEG1 GCA_000236645.1_ASM23664v1
GCA_016892765.1_ASM1689276v1*
GCA_016892705.1_ASM1689270v1*
Methylorubrum.extorquens.PA1 GCA_000018845.1_ASM1884v1
Methylocystis.bryophila GCA_002117405.1_ASM211740v1
Methylocella.silvestris.BL2 GCA_000021745.1_ASM2174v1
Xanthobacter.autotrophicus.Py2 GCA_000017645.1_ASM1764v1
Rhodopseudomonas.palustris GCA_013415845.1_ASM1341584v1
Rhodomicrobium.vannielii.ATCC.17100 GCA_000166055.1_ASM16605v1
Hyphomicrobium.denitrificans.ATCC.51888 GCA_000143145.1_ASM14314v1
Thermopetrobacter.sp.TC1 GCA_000746275.1_ASM74627v1
alpha.proteobacterium.IMCC14465 GCA_000293845.2_ASM29384v2
Candidatus.Micropelagos.thuwalensis GCA_000469155.1.RS24
Parvibaculum.lavamentivorans.DS.1 GCA_000017565.1_ASM1756v1
alpha.proteobacterium.L41A GCA_000335735.1.L41A.1.0
Brevundimonas.subvibrioides.ATCC.15264 GCA_000144605.1_ASM14460v1
Asticcacaulis.excentricus.CB.48 GCA_000175215.2_ASM17521v2
Phenylobacterium.zucineum.HLK1 GCA_000017265.1_ASM1726v1
Caulobacter.vibrioides.NA1000 GCA_000022005.1_ASM2200v1
Oceanicaulis.alexandrii.DSM.11625 GCA_000420265.1_ASM42026v1
Maricaulis.maris GCA_003634045.1_ASM363404v1
Hyphomonas.neptunium.ATCC.15444 GCA_000013025.1_ASM1302v1
Hirschia.baltica.ATCC.49814 GCA_000023785.1_ASM2378v1
Parvularcula.bermudensis.HTCC2503 GCA_000152825.2_ASM15282v2
Sagittula.stellata.E.37 GCA_000169415.1_ASM16941v1
Ruegeria.pomeroyi.DSS.3 GCA_000011965.2_ASM1196v2
Octadecabacter.arcticus.238 GCA_000155735.2_ASM15573v2
Jannaschia.rubra GCA_900113265.1.IMG.taxon.2622736430.annotated.assembly
Rubellimicrobium.rubrum GCA_006152115.1_ASM615211v1
Ketogulonicigenium.vulgare GCA_001399515.1_ASM139951v1
Rhodobacter.sphaeroides.ATCC.17029 GCA_000015985.1_ASM1598v1
Paracoccus.denitrificans GCA_004063735.1_ASM406373v1
Neomegalonema.perideroedes.DSM.15528 GCA_000374145.1_ASM37414v1
alpha.proteobacterium.HIMB5 GCA_000299095.1_ASM29909v1
Candidatus.Pelagibacter.ubique.HTCC1062 GCA_000012345.1_ASM1234v1
Candidatus.Pelagibacter.ubique.HIMB058 GCA_000419545.1_ASM41954v1
alpha.proteobacterium.HIMB114 GCA_000163555.2
Sphingopyxis.macrogoltabida GCA_001314325.1_ASM131432v1
Citromicrobium.sp.JL477 GCA_001304795.1_ASM130479v1
Sphingobium.indicum.B90A GCA_000264945.2_ASM26494v2
GCA_016892685.1_ASM1689268v1*
Zymomonas.mobilis.subsp.mobilis.ATCC.31821 GCA_000007105.1_ASM710v1
Sphingomonas.wittichii.RW1 GCA_000016765.1_ASM1676v1
alpha.proteobacterium.AAP81b GCA_001295935.1_ASM129593v1
Kordiimonas.gwangyangensis.DSM.19435 GCA_000375545.1_ASM37554v1
Sneathiella.glossodoripedis.JCM.23214 GCA_000616095.1_ASM61609v1
Gluconobacter.oxydans.DSM.3504 GCA_000583855.1_ASM58385v1
Acetobacter.aceti.NBRC.14818 GCA_000193495.2_ASM19349v2
Gluconacetobacter.diazotrophicus.PA1.5 GCA_000067045.1_ASM6704v1
Commensalibacter.intestini.A911 GCA_000231445.2_ASM23144v1
Granulibacter.bethesdensis.CGDNIH4 GCA_000576145.2_ASM57614v2
Acidisphaera.rubrifaciens.HS.AP3 GCA_000964365.1_ASM96436v1
Acidiphilium.multivorum.AIU301 GCA_000202835.1_ASM20283v1
Rubritepida.flocculans.DSM.14296 GCA_000425365.1_ASM42536v1
Belnapia.moabensis.DSM.16746 GCA_000745835.1_ASM74583v1
Roseomonas.gilardii GCA_001941945.1_ASM194194v1
Elioraea.tepidiphila.DSM.17972 GCA_000378465.1_ASM37846v1
Rhodospirillum.rubrum.F11 GCA_000225955.1_ASM22595v1
Pararhodospirillum.photometricum.DSM.122 GCA_000284415.2_ASM28441v2
Roseospirillum.parvum GCA_900100455.1
Caenispirillum.salinarum.AK4 GCA_000315795.1
Magnetospirillum.magneticum.AMB.1 GCA_000009985.1_ASM998v1
Thalassospira.profundimaris.WP0211 GCA_000300275.1_ASM30027v1
Terasakiella.pusilla.DSM.6293 GCA_000688235.1_ASM68823v1
alpha.proteobacterium.BAL199 GCA_000171835.1_ASM17183v1
Thalassobaculum.salexigens.DSM.19539 GCA_000423805.1_ASM42380v1
Candidatus.Puniceispirillum.marinum.IMCC1322 GCA_000024465.1_ASM2446v1
Oceanibaculum.indicum GCA_003633955.1_ASM363395v1
Rhodovibrio.salinarum.DSM.9154 GCA_000515255.1_ASM51525v1
Kiloniella.laminariae.DSM.19542 GCA_000374005.1_ASM37400v1
Rhodospirillum.centenum GCA_014197805.1_ASM1419780v1
Inquilinus.limosus.DSM.16000 GCA_000423185.1_ASM42318v1
Micavibrio.aeruginosavorus.ARL.13 GCA_000226315.1_ASM22631v1
Tistrella.mobilis.KA081020.065 GCA_000264455.2_ASM26445v2
Geminicoccus.roseus.DSM.18922 GCA_000427665.1_ASM42766v1
GCA_902798535.1*
GCA_902773285.1*
GCA_902796745.1*
GCA_902798325.1*
GCA_902769055.1*
GCA_902796345.1*
GCA_902784055.1*
GCA_902795615.1*
GCA_902772645.1*
Candidatus.Paracaedibacter.acanthamoebae GCA_000742835.1_ASM74283v1
Candidatus.Odyssella.thessalonicensis.L13 GCA_000190415.2_ASM19041v1
Candidatus.Finniella.inopinata GCA_004210305.1_ASM421030v1
GCA_902771535.1*
GCA_902761495.1*
GCA_902790255.1*
GCA_002393025.1_ASM239302v1*
GCA_902796155.1*
Holospora.undulata.HU1 GCA_000388175.3_ASM38817v3
Candidatus.Hepatobacter.penaei GCA_000742475.1_ASM74247v1
GCA_002238905.1_ASM223890v1*
Candidatus.Caedibacter.acanthamoebae GCA_000743035.1_ASM74303v1
Caedimonas.varicaedens GCA_001192655.1_ASM119265v1
Candidatus.Nucleicultrix.amoebiphila.FS5 GCA_002117145.1_ASM211714v1
GCA_900550565.1.UMGS1303*
GCA_900546225.1.UMGS860*
GCA_900546185.1.UMGS859*
GCA_900544565.1.UMGS690*
Magnetofaba.australis.IT.1 GCA_002109495.1_ASM210949v1
Magnetococcus.marinus.MC.1 GCA_000014865.1_ASM1486v1
Nitrosospira.briensis.C.128 GCA_000619905.2_ASM61990v2
Methylotenera.versatilis.301 GCA_000093025.1_ASM9302v1
Ralstonia.pickettii GCA_902374465.1.MGYG.HGUT.01384
Burkholderia.cepacia GCA_009586235.1_ASM958623v1
Dasania.marina.DSM.21967 GCA_000373485.1_ASM37348v1
Congregibacter.litoralis.KT71 GCA_000153125.2_ASM15312v2
Enterobacter.cloacae GCA_000770155.1_ASM77015v1
Alteromonas.macleodii.ATCC.27126 GCA_000172635.2_ASM17263v2
0.348889

References

1. Floriano AM *et al.* (2018) The Genome Sequence of "*Candidatus* Fokinia solitaria": Insights on Reductive Evolution in *Rickettsiales*. Genome biology and evolution 10: 1120–1126.

2. Castelli M *et al.* (*2019) Deianiraea*, an extracellular bacterium associated with the ciliate Paramecium, suggests an alternative scenario for the evolution of *Rickettsiales.* The ISME journal 13: 2280–2294.

3. Castelli M *et al.* (2021) "*Candidatus* Sarmatiella mevalonica" endosymbiont of the ciliate *Paramecium* provides insights on evolutionary plasticity among *Rickettsiales.* Environmental microbiology 23: 1684–1701.

4. Pitcher RS, Watmough NJ (2004) The bacterial cytochrome cbb3 oxidases. Biochimica et biophysica acta 1655: 388–399.

5. George EE *et al.* (2020) Highly Reduced Genomes of Protist Endosymbionts Show Evolutionary Convergence. Current biology 30: 925–933.

6. Schön ME *et al.* (2022) The evolutionary origin of host association in the *Rickettsiales.* Nature microbiology 7: 1189–1199.

7. Moreno-Vivián C *et al.* (1999) Prokaryotic nitrate reduction: molecular properties and functional distinction among bacterial nitrate reductases. Journal of bacteriology 181: 6573–6584.

8. Driscoll TP *et al.* (2017) Wholly *Rickettsia*! Reconstructed Metabolic Profile of the Quintessential Bacterial Parasite of Eukaryotic Cells. mBio 8: doi:10.1128/mBio.00859-17.

9. Hunter ES *et al.* (2020) Metabolic Contributions of an Alphaproteobacterial Endosymbiont in the Apicomplexan *Cardiosporidium cionae.* Frontiers in microbiology 11: 580719.

10. Paight C *et al.* (2022) Codependence of individuals in the *Nephromyces* species swarm requires heterospecific bacterial endosymbionts. Current biology 32: 2948–2955.

11. Min CK *et al.* (2008) Genome-based construction of the metabolic pathways of *Orientia tsutsugamushi* and comparative analysis within the *Rickettsiales* order. Comparative and functional genomics 2008: 623145.

12. Gillespie JJ *et al.* (2012) A *Rickettsia* genome overrun by mobile genetic elements provides insight into the acquisition of genes characteristic of an obligate intracellular lifestyle. Journal of bacteriology 194: 376–394.

13. Nikoh N *et al.* (2014) Evolutionary origin of insect-*Wolbachia* nutritional mutualism. *Proceedings of the National Academy of Sciences of the United States of America* 111: 10257–10262.

14. Gerth M, Bleidorn C. (2016) Comparative genomics provides a timeframe for *Wolbachia* evolution and exposes a recent biotin synthesis operon transfer. Nature microbiology 2: 16241.

15. El Karkouri K *et al.* (2016) Origin and Evolution of Rickettsial Plasmids. PloS one 11: e0147492.

16. Mironov T, Sabaneyeva E (2020) A Robust Symbiotic Relationship Between the Ciliate *Paramecium multimicronucleatum* and the Bacterium *Ca.* Trichorickettsia Mobilis. Frontiers in

microbiology 11: 603335.

17. Glöckner G *et al.* (2014) The genome of the foraminiferan *Reticulomyxa filosa*. Curr Biol 24: 11–18 .

18. Catania F *et al.* (2009) Genetic diversity in the *Paramecium aurelia* species complex. Mol Biol Evol. 26: 421-431.

19. Boscaro V *et al.* (2013) Rediscovering the genus *Lyticum*, multiflagellated symbionts of the order *Rickettsiales*. Sci. Rep. 3:3305.

20. Lanzoni O *et al.* (2019) Diversity and environmental distribution of the cosmopolitan endosymbiont "*Candidatus* Megaira". Sci Rep 9: 1179.

21. Szokoli F *et al.* (2016) Disentangling the taxonomy of *Rickettsiales* and description of two novel symbionts ("*Candidatus* Bealeia paramacronuclearis" and "*Candidatus* Fokinia cryptica") sharing the cytoplasm of the ciliate protist *Paramecium biaurelia*. Appl Environ Microbiol 82: 7236–7247.

22. Tarcz S *et al.* (2012) Variation in ribosomal and mitochondrial DNA sequences demonstrates the existence of intraspecific groups in *Paramecium multimicronucleatum* (Ciliophora, Oligohymenophorea). Mol Phylogenet Evol 63: 500-509.

23. Vannini C *et al.* (2010) "*Candidatus* Anadelfobacter veles" and "*Candidatus* Cyrtobacter comes," two new *Rickettsiales* species hosted by the protist ciliate *Euplotes harpa (*Ciliophora, Spirotrichea). Appl Environ Microbiol. 76:4047–44054.

24. Nitla V *et al.* (2019 Critical revision of the family Plagiopylidae (Ciliophora: Plagiopylea), including the description of two novel species, *Plagiopyla ramani* and *Plagiopyla narasimhamurtii*, and redescription of *Plagiopyla nasuta* Stein, 1860 from India. Zool J Linn Soc 186:1-45.

25. Sambrook J, Russell DW (2001) Molecular Cloning: A Laboratory Manual. 3rd Edition, Vol. 1, Cold Spring Harbor Laboratory Press, New York.

26. Wick RR *et al.* (2017) Completing bacterial genome assemblies with multiplex MinION sequencing. Microb Genom. 3: e000132.

27. De Coster W *et al.* (2018) NanoPack: visualizing and processing long-read sequencing data. Bioinformatics 34: 2666-2669.

28. Andrews S (2010) FastQC: a quality control tool for high throughput sequence data. Available online at: http://www.bioinformatics.babraham.ac.uk/projects/fastqc.

29. Bankevich A *et al.* (2012) SPAdes: A new genome assembly algorithm and its applications to single-cell sequencing. J Comp Biol 19: 455-477.

30. Wick RR et al. (2017) Unicycler: Resolving bacterial genome assemblies from short and long sequencing reads. PLOS Comp Biol 13: e1005595.

31. Kumar S et al. (2013) Blobology: exploring raw genome data for contaminants, symbionts and parasites using taxon-annotated GC-coverage plots. Front. Genet. 4: 237.

32. Langmead B et al. (2012) Fast gapped-read alignment with Bowtie 2. Nat Methods 9: 357-359.

33. Wickham H (2016) ggplot2: elegant graphics for data analysis. Springer-Verlag New York.

ISBN 978-3-319-24277-4.

34. Seemann T (2013) barrnap 0.5 : rapid ribosomal RNA prediction. http://www.vicbioinformatics.com/

35. Seemann T (2014) Prokka: Rapid prokaryotic genome annotation. Bioinformatics 30, 2068-2069.

36. Wick RR *et al.* (2015) Bandage: interactive visualization of *de novo* genome assemblies. Bioinformatics 31: 3350-3352.

37. Hyatt D *et al.* (2010) Prodigal: prokaryotic gene recognition and translation initiation site identification. BMC Bioinformatics 11:119.

38. Buchfink B et al. (2015) Fast and sensitive protein alignment using DIAMOND, Nature Methods 12: 59–60.

39. Senra MVX et al. (2016) A house for two-double bacterial infection in *Euplotes woodruffi* Sq1 (Ciliophora, Euplotia) sampled in southeastern Brazil. Microb Ecol. 71: 505–517.

40. Kawafune K *et al.* (2012) Molecular identification of rickettsial endosymbionts in the non-phagotrophic volvocalean green algae. PLoS One 7: e31749.

41. Parks DH *et al.* (2018) A standardized bacterial taxonomy based on genome phylogeny substantially revises the tree of life. Nat Biotechnol 36:996-1004.

42. Muñoz-Gómez S *et al.* (2019) An updated phylogeny of the *Alphaproteobacteria* reveals that the parasitic *Rickettsiales* and *Holosporales* have independent origins. eLife 8:e42535.

43. Wang Z, Wu M (2014) Complete genome sequence of the endosymbiont of *Acanthamoeba* strain UWC8, an amoeba endosymbiont belonging to the "*Candidatus* Midichloriaceae" family in *Rickettsiales*. Genome Announc. 2:e00791–14.

44. Schulz F *et al.* (2016) A *Rickettsiales* symbiont of amoebae with ancient features. Environ Microbiol. 18:2326–2342.

45. Scholz M *et al.* (2020) Large scale genome reconstructions illuminate *Wolbachia* evolution. Nat Commun 11:5235.

46. Martijn J et al. (2018) Deep mitochondrial origin outside the sampled *Alphaproteobacteria*. Nature 557:101–105.

47. Parks DH *et al.* (2022) GTDB: an ongoing census of bacterial and archaeal diversity through a phylogenetically consistent, rank normalized and complete genome-based taxonomy. Nucl Acids Res 50: D785–D794.

48. Simão FA *et al.* (2015) BUSCO: assessing genome assembly and annotation completeness with single-copy orthologs. Bioinformatics 31: 3210–3212.

49. Huerta-Cepas J *et al.* (2019) eggNOG 5.0: a hierarchical, functionally and phylogenetically annotated orthology resource based on 5090 organisms and 2502 viruses. Nucleic Acids Res. 47:D309-D314.

50. Cantalapiedra CP *et al.* (2021) eggNOG-mapper v2: Functional annotation, orthology assignments, and domain prediction at the metagenomic scale. Mol Biol Evol: msab293.

51. Katoh K, Standley DM (2013) MAFFT multiple sequence alignment software version 7: improvements in performance and usability. Mol Biol Evol. 30:772-80.

52. Criscuolo A, Gribaldo S (2010) BMGE (Block Mapping and Gathering with Entropy): a new software for selection of phylogenetic informative regions from multiple sequence alignments. BMC Evol. Biol. 10:210.

53. Price MN *et al.* (2010) FastTree 2--approximately maximum-likelihood trees for large alignments. PLoS One 5:e9490.

54. Borowiec ML (2016) AMAS: a fast tool for alignment manipulation and computing of summary statistics. PeerJ 4:e1660.

55. Nguyen LT *et al.* (2015) IQ-TREE: a fast and effective stochastic algorithm for estimating maximum-likelihood phylogenies. Mol Biol Evol. 32:268-274.

56. Kalyaanamoorthy S *et al.* (2017) ModelFinder: fast model selection for accurate phylogenetic estimates. Nat Methods 14:587–589.

57. Minh BQ *et al.* (2013) Ultrafast approximation for phylogenetic bootstrap. Mol Biol Evol 30:1188-95.

58. Rodriguez-R LM, Konstantinidis KT (2016) The enveomics collection: a toolbox for specialized analyses of microbial genomes and metagenomes. PeerJ Preprints 4:e1900v1.

59. Otero-Bravo A *et al.* (2018) Cladogenesis and genomic streamlining in extracellular endosymbionts of tropical stink bugs. Genome Biol Evol 10: 680–693.

60. Carrier TJ *et al.* (2021) Microbiome reduction and endosymbiont gain from a switch in sea urchin life history. Proc Natl Acad Sci U S A 118:e2022023118.

61. Klinges JG *et al.* (2019) Phylogenetic, genomic, and biogeographic characterization of a novel and ubiquitous marine invertebrate-associated *Rickettsiales parasite*, Candidatus Aquarickettsia rohweri, gen. nov., sp. nov. ISME J. 13:2938-2953.

62. Karp PD *et al.* (2019) The BioCyc collection of microbial genomes and metabolic pathways. Brief Bioinform. 20: 1085-1093.

63. Marchler-Bauer A, Bryant SH (2004) CD-Search: protein domain annotations on the fly, Nucleic Acids Res. 32:327-331.

64. Saier MH *et al.* (2021) The Transporter Classification Database (TCDB): 2021 update. Nucleic Acids Res. 49:D461-D467.

65. Major P *et al.* (2017) Phylogenetic Diversity of NTT Nucleotide transport proteins in free-living and parasitic bacteria and eukaryotes. Genome Biol Evol. 9:480-487.

66. Liu B *et al.* (2022) VFDB 2022: a general classification scheme for bacterial virulence factors. Nucleic Acids Res. 50: D912-D917.

67. Emms DM, Kelly S (2019) OrthoFinder: phylogenetic orthology inference for comparative genomics. Genome Biol. 20:238.
