# Supplementary material for: Host association and intracellularity evolved multiple times independently in the Rickettsiales
Source: Nat Commun. 2024 Feb 6;15:1093. doi: 10.1038/s41467-024-45351-7 (PMC10847448; doi:10.1038/s41467-024-45351-7)
Supplement: Supplementary file 4 — Description of Additional Supplementary Files [file 41467_2024_45351_MOESM4_ESM.pdf]

File Name: Supplementary Data 1
Description: Summary of the sequencing (A) and assembly (B) procedures and results for the nine novel genomes obtained in this study.

File Name: Supplementary Data 2
Description: BUSCO scores of the selected published *Rickettsiales* genomes, the novel genomes obtained, and the two different sets of metagenome-assembled-genomes (MAGs) analysed. MAGs with <50% complete orthologs (yellow) or with ≥5% duplicated orthologs (red) were discarded.

File Name: Supplementary Data 3
Description: General features of the 113 analysed *Rickettsiales* assemblies, including family averages.

File Name: Supplementary Data 4
Description: List of suspected non-ortholog sequences in single-gene phylogenies of biosynthetic pathways for amino acids and nucleotides (Supplementary figure 8), which were removed prior to concatenation (Supplementary figure 9). Sequences are presented organised by pathway and gene.

File Name: Supplementary Data 5
Description: For each sample separately, list of the contigs of the preliminary assembly in which rRNA genes were identified with barrnap. For each contig, blobology parameters are reported, as well as the positions in which the rRNA gene was inferred, and the best blast hit of this gene sequence. Contigs are sorted by the respective sequencing coverage, and coloured according to the presumed organismal assignment (blue: host; shades of green: *Rickettsiales* symbionts; other colours: additional organisms).

File Name: Supplementary Data 6
Description: List of the 179 eggnog orthogroups employed for the phylogenomic analyses, and their presence/absence pattern in the organisms analysed in this study, including *Rickettsiales*, *Alphaproteobacteria*, outgroup, and, in a separate tab, MAGs (8 MAGs, highlighted in red, were discarded prior to phylogeny due to their identification as non-*Rickettsiales* based on the eggnog assignment).

File Name: Supplementary Data 7
Description: List of the genes analysed for the phylogeny of biosynthetic pathways of nucleotides and amino acids, subdivided by pathway.

File Name: Supplementary Data 8
Description: Proportional presence of the genes of biosynthetic pathways of nucleotides and amino acids, and number of tlc nucleotide transporters, among the recently published *Rickettsiales* genomes (updated to 18th May 2023)

File Name: Supplementary Data 9
Description: List of the genome accession numbers of the Rickettsiales genomes and MAGs analysed with this study.
