# Supplementary material for: Host association and intracellularity evolved multiple times independently in the Rickettsiales
Source: Nat Commun. 2024 Feb 6;15:1093. doi: 10.1038/s41467-024-45351-7 (PMC10847448; doi:10.1038/s41467-024-45351-7)
Supplement: Supplementary file 3 — Reporting Summary [file 41467_2024_45351_MOESM3_ESM.pdf]

nature portfolio

Corresponding author(s): Davide Sassera, Giulio Petroni

Last updated by author(s): Dec 11, 2023

Reporting Summary

Nature Portfolio wishes to improve the reproducibility of the work that we publish. This form provides structure for consistency and transparency in reporting. For further information on Nature Portfolio policies, see our Editorial Policies and the Editorial Policy Checklist.

Statistics

For all statistical analyses, confirm that the following items are present in the figure legend, table legend, main text, or Methods section.

n/a	Confirmed	
☐	☒	The exact sample size (*n*) for each experimental group/condition, given as a discrete number and unit of measurement
☒	☐	A statement on whether measurements were taken from distinct samples or whether the same sample was measured repeatedly
☒	☐	The statistical test(s) used AND whether they are one- or two-sided<br>*Only common tests should be described solely by name; describe more complex techniques in the Methods section.*
☒	☐	A description of all covariates tested
☒	☐	A description of any assumptions or corrections, such as tests of normality and adjustment for multiple comparisons
☐	☒	A full description of the statistical parameters including central tendency (e.g. means) or other basic estimates (e.g. regression coefficient) AND variation (e.g. standard deviation) or associated estimates of uncertainty (e.g. confidence intervals)
☒	☐	For null hypothesis testing, the test statistic (e.g. *F*, *t*, *r*) with confidence intervals, effect sizes, degrees of freedom and *P* value noted<br>*Give P values as exact values whenever suitable.*
☒	☐	For Bayesian analysis, information on the choice of priors and Markov chain Monte Carlo settings
☒	☐	For hierarchical and complex designs, identification of the appropriate level for tests and full reporting of outcomes
☒	☐	Estimates of effect sizes (e.g. Cohen's *d*, Pearson's *r*), indicating how they were calculated

Our web collection on statistics for biologists contains articles on many of the points above.

Software and code

Policy information about availability of computer code

Data collection	All data (genome or metagenome-assembled genome sequences and/or their annotated proteins) were individually downloaded from NCBI ftp (via unix command line). No additional software was used for data collection.
Data analysis	FastQC 0.11.7, MinKNOW 18.12.9, guppy 5.0.1, Porechop 0.2.4, NanoPlot 1.23.0, SPAdes 3.6, blobology (https://github.com/blaxterlab/blobology), bowtie2 2.4.2, Bandage 0.8.1, prokka 1.10, Unicycler 0.4.8, diamond 0.9, prodigal 2.6.3, finchtv 1.3.1, BUSCO 5.0.0, eggnog-mapper 2.0.6, MAFFT 7.475, BMGE 1.12, FastTree 2.1, AMAS (https://github.com/marekborowiec/AMAS), IQ-TREE 1.6.12, enveomics toolbox (http://enve-omics.ce.gatech.edu/aai/), OrthoFinder 2.5.4. The usage and version of each software is described in the methods section of the manuscript and/or in the Supplementary Notes.

For manuscripts utilizing custom algorithms or software that are central to the research but not yet described in published literature, software must be made available to editors and reviewers. We strongly encourage code deposition in a community repository (e.g. GitHub). See the Nature Portfolio guidelines for submitting code & software for further information.

Data

Policy information about availability of data

All manuscripts must include a data availability statement. This statement should provide the following information, where applicable:

- Accession codes, unique identifiers, or web links for publicly available datasets
- A description of any restrictions on data availability
- For clinical datasets or third party data, please ensure that the statement adheres to our policy

Sequences obtained in this project were deposited to NCBI under accession number PRJNA831616. The annotated genome sequences of the symbiont of Reticulomyxa filosa is available on Zenodo (doi: 10.5281/zenodo.10324454). Accession numbers of published Rickettsiales assemblies (including all initial MAGs) analysed in this study are provided herein (Supplementary Data 9). The eggnog (http://http://eggnog5.embl.de), BioCyc (https://biocyc.org), TCDB (https://www.tcdb.org), and VFDB (http://www.mgc.ac.cn/VFs) databases were also employed in this study.

Research involving human participants, their data, or biological material

Policy information about studies with human participants or human data. See also policy information about sex, gender (identity/presentation), and sexual orientation and race, ethnicity and racism.

Reporting on sex and gender	This study did not involve human research
Reporting on race, ethnicity, or other socially relevant groupings	This study did not involve human research
Population characteristics	This study did not involve human research
Recruitment	This study did not involve human research
Ethics oversight	This study did not involve human research

Note that full information on the approval of the study protocol must also be provided in the manuscript.

Field-specific reporting

Please select the one below that is the best fit for your research. If you are not sure, read the appropriate sections before making your selection.

☐ Life sciences ☐ Behavioural & social sciences ☒ Ecological, evolutionary & environmental sciences

For a reference copy of the document with all sections, see nature.com/documents/nr-reporting-summary-flat.pdf

Ecological, evolutionary & environmental sciences study design

All studies must disclose on these points even when the disclosure is negative.

Study description	In this study, nine novel genomes of Rickettsiales bacteria associated with unicellular eukaryotes (protists) were assembled, starting from newly obtained reads (eight genomes) or third party previously obtained ones (one genome). Together with a selection of published genomes of Rickettsiales and their relatives, as well as of putative Rickettsiales metagenome-assembled genomes, the newly obtained genome were used to derive comprehensive evolutionary history reconstructions of the whole Rickettsiales, both in terms of diversity, and of functional features, in particular interaction with eukaryotic hosts
Research sample	The starting point for this study was the de novo assembly of nine novel genomes of Rickettsiales bacteria associated with protists. All except one (Reticulomyxa filosa host, for which the reads derived from a previous third party sequencing study) were newly sequenced. The newly sequenced samples were largely derived from previous studies, except two (NGD2 and IBS-3; further details in the following sections).<br>All other data used in the subsequent analyses were derived from published assemblies, and was downloaded from NCBI repositories. No pre-determined dataset was used. Each sample was individually selected and downloaded. Selection criteria were based on phylogenetic proximity and phylogenetic representativeness, as detailed in the supplementary material (including all the accession numbers)<br>All samples were chosen based on two main sequential criteria: putative affiliation to Rickettsiales, and, secondarily, phylogenetic representativeness (in order to reduced total sample size and computationl load, closely-related samples were discarded). All criteria are detailed in the methods. Each sample is not meant to represent any specific population, rather, based on data availability, to represent the respective sublineage of Rickettsiales (approximately at the genus level).
Sampling strategy	Sampling size was not relevant for the study
Data collection	Newly obtained samples were collected from aquatic environments as described in the following sections. DNA preparation for sequencing was performed by M. Castelli, V. Serra, A. Potekhin, E. Sabaneyeva, and L. Gammuto. Sequencing was performed in

	outsourcing (Illumina reads) and by M. Castelli (Oxford Nanopore reads). Reads of the Reticulomyxa host were provide by the authors of the original study (doi:10.1016/j.cub.2013.11.027), as detailed in the acknowledgements section. Genome assembly was performed by M. Castelli, T. Nardi, L. Gammuto, and G. Bellinzona. All other employed genomes and metagenome-derived genomes were derived from published studies, and were downloaded directly from NCBI repositories. Selection criteria were based on phylogenetic proximity and representativeness, as detailed in the Supplementary information.
Timing and spatial scale	Samples were collected on:<br>-NDG2: 15th March 2013<br>-IBS-3; May 2018<br>There was no specific rationale for sampling, each sample was taken individually and then inspected for the potential presence of protists, which were then screened for the presence of Rickettsiales bacteria
Data exclusions	The newly assembled Rickettsiales bacteria are obligatorily associated with unicellular eukaryotic hosts. Thus, the raw sequencing data included host sequences (and in some cases sequences from other associated prokaryotes). An ad hoc procedure (described in the methods) was applied to remove those sequences from the assembly, and select only those belonging to the bacteria of interest.<br><br>For the phylogenetic and evolutionary reconstructions, initially selected published metagenome-assembled genomes (MAGs) below certain thresholds of BUSCO scores were discarded (below 50% proteobacterial orthologs, and/or 5% or more of duplicated ones). Furthermore, all MAGs which, based on our phylogeny, were found not to be affiliated to Rickettsiales, were discarded from the final dataset. The final dataset contained only one representative (the one with the best BUSCO scores) for each "cluster" of MAGs, namely each monophyletic lineage of MAGs all sharing average aminoacidic identity of 85% of higher.
Reproducibility	All the sources (i.e. accession numbers) of data employed in this study are reported in the Supplementary material, which also includes a detailed account of the software used and all the procedures applied, allowing reproducibility
Randomization	Randomization was not relevant for the study, because the organisms were not assigned to groups
Blinding	Blinding was not relevant for the present study

Did the study involve field work? ☒ Yes ☐ No

Field work, collection and transport

Field conditions	Climatic conditions were not recorded and are not relevant for the study
Location	-Sample NDG2: Naval Dockyard Gate in Visakhapatnam (Andhra Pradesh, India) at sea level; coordinates: 17.712083, 83.263111<br>-Plagiopyla frontata IBS-3; Baltic Sea in Inkoo (Finland) at sea level; coordinates: 60.042403, 24.006956
Access & import/export	Sampling locations were reached by car. No permissions were required for sampling in the selected sites. All other protists were derived from previous studies
Disturbance	No disturbance on the sites was caused, only small amounts of water and surface sediment were samples

Reporting for specific materials, systems and methods

We require information from authors about some types of materials, experimental systems and methods used in many studies. Here, indicate whether each material, system or method listed is relevant to your study. If you are not sure if a list item applies to your research, read the appropriate section before selecting a response.

Materials & experimental systems

n/a	Involved in the study	
☒	☐	Antibodies
☐	☒	Eukaryotic cell lines
☒	☐	Palaeontology and archaeology
☐	☒	Animals and other organisms
☒	☐	Clinical data
☒	☐	Dual use research of concern
☒	☐	Plants

Methods

n/a	Involved in the study	
☒	☐	ChIP-seq
☒	☐	Flow cytometry
☒	☐	MRI-based neuroimaging

Eukaryotic cell lines

Policy information about cell lines and Sex and Gender in Research

Cell line source(s)	Four freshwater or marine ciliate protist cell lines were obtained in previous studies (doi: 10.1038/srep03305; doi: 10.1038/

Cell line source(s)	s41598-018-37629-w; doi: :10.1128/AEM.02284-16; doi: 10.1093/molbev/msn266). Two additional ones (one marine water and another one from brackish water) were newly isolated during this study
Authentication	Microscopy and 18S rRNA gene sequencing were used for authentication
Mycoplasma contamination	This is not relevant for protist cultures
Commonly misidentified lines (See ICLAC register)	This is not relevant for protist cultures

Animals and other research organisms

Policy information about studies involving animals; ARRIVE guidelines recommended for reporting animal research, and Sex and Gender in Research

Laboratory animals	This study did not involved laboratory animals (or any other animal)
Wild animals	This study did not involved laboratory animals (or any other animal)
Reporting on sex	Not relevant for the organisms involved in this study
Field-collected samples	Sample NDG2 (and isolated protist culture) was kept at 19°C, with a light/dark period of 12h/12h. The isolated protist culture (Euplotes woodruffi) was maintained by feeding with the unicellular alga Dunaliella tertiolecta, in artificial water at 5‰ salinity, following previously established protocols (e.g., doi: 10.1128/AEM.03105-09).<br>Sample IBS-3, including protist cells, was kept at 19°C in the dark, in microoxic-anoxic conditions, following a previously established protocol (doi: 10.1093/zoolinnean/zly041)
Ethics oversight	The organisms involved in the study are not covered by ethical legislation. They are unicellular eukaryotic microbes (protists) that feed on bacteria or unicellular algae, and harbour intracellular bacteria.

Note that full information on the approval of the study protocol must also be provided in the manuscript.

Plants

Seed stocks	This study did not involve any seed stock (or any other plant material)
Novel plant genotypes	This study did not involve any plant (or plant-derived material)
Authentication	This study did not involve any plant (or plant-derived material)
